# Overview of the Nomenclature and Network of Contributors to the Development of Bioreactors for Human Gut Simulation Using Bibliometric Tools: A Fragmented Landscape

**DOI:** 10.1021/acs.jafc.2c03597

**Published:** 2022-09-12

**Authors:** Janeth Sanabria, Siobhon Egan, Reika Masuda, Alex J. Lee, Glenn R. Gibson, Jeremy K. Nicholson, Julien Wist, Elaine Holmes

**Affiliations:** † Environmental Microbiology and Biotechnology Laboratory, Engineering School of Environmental & Natural Resources, Engineering Faculty, Universidad del Valle−Sede Meléndez, Cali 76001, Colombia; ‡ Australian National Phenome Centre and Computational and Systems Medicine, Health Futures Institute, Murdoch University, Harry Perkins Building, Perth, Western Australia WA6150, Australia; § Department of Food and Nutritional Sciences, University of Reading, Reading RG6 6AH, United Kingdom; ∥ Institute of Global Health Innovation, Faculty of Medicine, Imperial College London, Level 1, Faculty Building, South Kensington Campus, London SW7 2NA, United Kingdom; ⊥ Chemistry Department, Universidad del Valle, Cali 76001, Colombia; # Department of Metabolism, Digestion, and Reproduction, Faculty of Medicine, Imperial College London, Sir Alexander Fleming Building, South Kensington, London SW7 2AZ, United Kingdom

**Keywords:** bioreactor, in vitro model, gut microbiota, gastrointestinal system

## Abstract

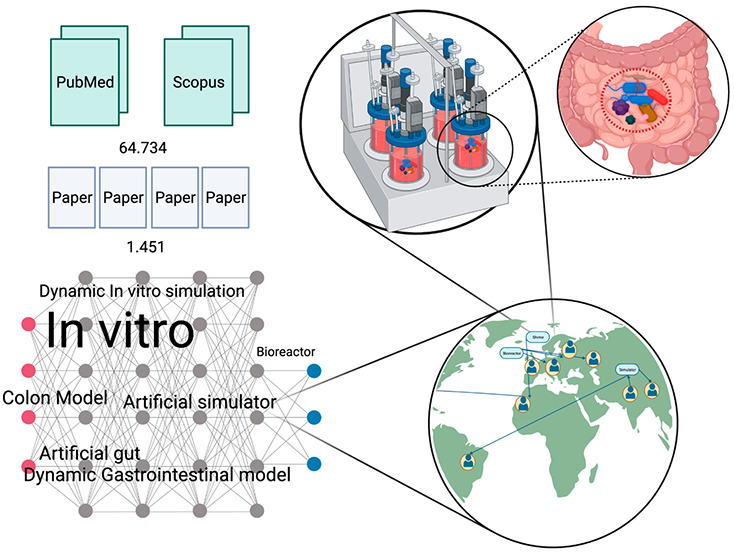

The evolution of complex in vitro models of the human gastrointestinal system to interrogate the biochemical functionality of the gut microbiome has augmented our understanding of its role in human physiology and pathology. With 5718 authors from 52 countries, gut bioreactor research reflects the growing awareness of our need to understand the contribution of the gut microbiome to human health. Although a large body of knowledge has been generated from in vitro models, it is scattered and defined by application-specific terminologies. To better grasp the capacity of bioreactors and further our knowledge of the human gastrointestinal system, we have conducted a cross-field bibliometric search and mapped the evolution of human gastrointestinal in vitro research. We present reference material with the aim of identifying key authors and bioreactor types to enable researchers to make decisions regarding the choice of method for simulating the human gut in the context of microbiome functionality.

## Introduction

The realization of the association of the microbiome with health, stemming from early work by pioneers such as Louis Pasteur and Robert Koch in the 1880s,^1^ led to an awareness of specific microorganisms being responsible for particular diseases (Koch’s postulates), such as *Vibrio cholerae* and cholera disease. Today the association between the gut microbiome and health is widely acknowledged, and etiological connections between a perturbed gut microbiome and multiple inflammatory conditions, development of an obesogenic environment, and even anxiety and cognitive performance are known to be impacted by the microbiome. In the last 100 years, more than 1000 species of gut microbiota have been isolated and chemically characterized.^2,3^ Although significant improvements in our health have been achieved by developing therapeutics targeting microbes identified as pathogens,^4,5^ the multifaceted gut–microbiome relationships are still largely unexplored. Due to the complexity of human physiology and the vast diversity of microbial species and their corresponding metabolic functionality, understanding the relationship between the microbiome and health is challenging.

Humans have a highly specific coevolved symbiotic microbiome that enables significant levels of genome reduction between the host and microbes^6^ and between gut microbes via horizontal gene transfer.^7^ Communication between the host and their microbiome is mediated via the immune system, the vagus nerve, and via direct chemical signaling.^8^ The gut microbiome is metabolically flexible and can respond to changing nutritional exposure and environmental stressors on a fairly short time scale. In turn, changes in the gut microbiome may modulate multiple pathways throughout the body and thereby strongly influence the metabolic phenotype of the host^9,10^ with a long-term impact on host fitness, resistance to disease, and interactions with drugs and toxins.^11^ Although isolation of microbes and pure culture strains are valuable tools for testing hypotheses regarding the functionality of the microbiota,^12^ only the use of mixed cultures representing the major taxa colonizing the gut that faithfully emulate complex environments will provide authentic information about the microbial ecological organization and links with the rest of the human body. However, reproducing ex vivo complex microbial communities, such as those found in the human gastrointestinal tract (GIT), is nontrivial since the GIT comprises a series of interconnected environments with different structural and physicochemical properties that respond dynamically to the consumption of food and other stimuli. Therefore, multiple in vitro models aiming to mimic the human digestive system and its bacterial communities have been proposed and implemented to explore direct relationships between the taxonomic and/or functional characterization of the microbiome and the host metabolome. These models can range from single systems aiming to replicate a specific site along the GIT to complex multicompartment/vessel models, which replicate numerous sites along the GIT with different physiological and biochemical conditions. Thorough mapping of these relationships requires assembling a highly diverse but coherent body of experimental data, recorded from as many different geographical areas, countries, and communities as possible. In addition, this is possible only if these data are collected with the highest possible degree of standardization. Although such standardization is difficult, some attempts have already been made for example, by Minekus et al.^13^

Thus, bioreactors broadly defined as “vessels where a biological transformation occurs” represent one of the most promising tools to grow and monitor organisms from different environments and study their biochemical reactions under controlled conditions.^14^ Bioreactors can be used to test and validate new culture methods for a consortium of microbes that could not be cultivated otherwise^15^ and/or to simulate biochemical–ecological interactions of the microbiome of natural environments, e.g., soil, water bodies, or mucosal interfaces in animals or humans. These devices have been developed and used in industrial and environmental processes in the last 70 years and thus exist in many different configurations.^16^ Since 1980, a wide range of human gut in vitro models have been developed ranging from a single vial to multistage multiple-vial systems with the purpose of answering specific research questions concerning food transformation,^17^ the role of specific micronutrients, toxins, antibiotics, or other components on the composition of bacterial communities.^18−20^

Various bioreactors have been developed in parallel within disparate research fields including wastewater treatment, gastroenterology, chemical engineering, etc. Consequently, the terminology used to describe the equipment and experimental setup followed a divergent evolution according to the field of origin. For instance, the results from literature searches will strongly differ if using the terms “bioreactor” and “human intestine” as opposed to “human in-vitro gut model” and “artificial gut”, despite the fact that studies in both queries may have used a similar apparatus. In addition, the terms themselves can be ambiguous in meaning. For example, the term in vitro can refer to a single test tube or a bioreactor with eight vessels, and the term bioreactor itself can be replaced by “chemostat”, “fermenter”, or “simulator of the human intestinal microbial ecosystem”. For this reason, it is challenging to aggregate a comprehensive body of information and has two major consequences for the research community. Firstly, it impacts the visibility of key research, with the work of smaller research groups often being overlooked, and second, it results in a significant challenge for researchers aiming to review developments in the field and identify state-of-the-art technologies to design their own experimental setup. If we want to improve our knowledge of the interactions of the microbiome with human health, it is critical to be able to make robust comparisons between laboratories using the same models. Again, this requires the adoption of a common, standard, terminology. Bioreactor models of the human gastrointestinal tract have multiple functions, and consideration of the architecture of such systems may be impacted by the research question. Here, we have focused only on systems that incorporate a component of microbial fermentation rather than considering the mechanical aspects of digestion.

Given the challenges described, we considered that a traditional literature review would fail to capture the entirety of the efforts required to simulate gut–microbiome environments. Instead, we applied a bibliometric approach using emerging analytical methods that enable the extraction of knowledge from numerous literature records^21^ obtained by querying comprehensive databases such as PubMed and Scopus to mention but two.^22^ We present here the results of an in-depth and systematic search to recover as many documents as possible concerned with the use of bioreactors to study the human gut microbiome, aggregating information from distinct research communities. We first identified a total of 1451 articles and then performed a text mining analysis to map the evolution of human gut models around the world and delineate the major contributors, institutions, and interlaboratory networking connections to provide a roadmap of the current research landscape.

## 2 Materials and Methods

The documents used in this manuscript were retrieved from Scopus^23^ and PubMed.^24^ Both of these tools support advanced search capabilities, offering basic query language with boolean operators to perform and refine queries.

### 2.2 Initial Query To Identify Research’s Field-Specific Terms

A preliminary wide spectrum query (see Supporting Information Tables S1–S3) was carried out in September 2021 that returned 64 734 articles. As expected for such a nonspecific query, many records were still not concerned with the topic of interest. Thus, in order to create a seeding data set for extraction of key terms, the first 460 relevant publications were selected manually and were subsequently imported into Voyant Tools^25^ and VOSviewer^26^ to search for research field-specific terms (RFST) referring to bioreactors/in vitro models of the human gut. The 21 most relevant RFST were as follows: Artificial gut, Bioreactor, Chemostat, Continuous culture, Fermentation, Fermenter, Gastrointestinal model, Git model, Gut model, Gut simulation, In vitro colon, In vitro digestion, In vitro gastrointestinal, In vitro model, Reactor, SHIME, Simulated colon(ic), Simulated gastrointestinal, Simulator of (the) human. A complete list of RFST can be found in Supporting Information Tables S4 and S5.

### 2.3 Aggregated Field-Specific Queries Using Scopus and PubMed

As an attempt to propose a simple yet robust and accurate query, two strategies were evaluated. The first strategy, more direct and probably more commonly used, consists of searching for some of the specific terms from the previous section in the title, abstract, and keyword (TAK) sections of the document and then selecting the most cited articles. From this search, the top 2000 cited articles were considered as the outcome of this first search strategy. For the second strategy, a set of refined queries was assembled to collate a more relevant set with fewer false positives. All queries shared a common block that was based on the query of the previous section, followed by a specific filter block using the terms identified in the previous section. The common block searched for terms in all of the sections of the documents, while the subsequent filters only queried the TAK sections. The resulting 21 queries were submitted to both PubMed and Scopus databases on November 25, 2021. Finally, the 42 queries (i.e., applying both strategies to each of the 21 terms) were repeated but this time adding the term “fecal” to the specific filter. The 84 output files were aggregated into a single database (5410 entries), and only documents with at least 2 occurrences were retained, based on the assumption that items mentioned in more than one specific query or present in both PubMed and Scopus are more likely to be relevant. Finally, the documents (460 unique items) that were manually selected and curated in the initial query (section 2.2) were merged to create a final list with 1451 unique entries (see Supporting Information Tables S6 and S7).

### 2.4 Data Curation and Visualization

In all output files, the title field was carefully curated and specific characters causing mismatch and punctuation were removed and set to lowercase. These curated titles were used as a hash to enumerate occurrences, as Digital Object Identifiers (DOIs) were not always present. Finally, the resulting array of DOIs was used as input to query the complete information about each document, including metadata, and to collate the final data sets for further analysis (see Supporting Information Tables S8–S13). The final data set collected in section 2.3 was imported into Biblioshiny (RShiny interface of bibliometrix package)^27^ for analysis.

## 3 Results and Discussion

The first literature search strategy described in section 2.2 detected a total of 12 409 authors. Among those authors, collection of works by Gibson GR (*n* = 42), De Vos WM (*n* = 31), Wang J (*n* = 22), Flint HI (*n* = 19), Verbeke K (*n* = 19), Li J (*n* = 18), Wang Y (*n* = 18), Li L (*n* = 17), Van De Wiele T (*n* = 16), and Asahara T (*n* = 15) dominated the search outcome. In terms of journal sources, the first search strategy collected the most articles from *Plos One* (*n* = 86), followed by *Journal of Agricultural and Food Chemistry* (*n* = 60), *Gut* (*n* = 55), *Applied and Environmental Microbiology* (*n* = 54), and the *British Journal of Nutrition* (*n* = 46). After a careful review of those 2000 citations, a large number of documents were found to be unrelated to the topic of our interest.

Therefore, the second search strategy described in section 2.3 was necessary to refine the database. A total of 5410 documents were identified by combining the results of the 84 specific queries, of which 1073 unique records remained after removing all items with only a single occurrence. This list was then combined with the 460 articles identified previously (as described in section 2.2), which gave a final list of 1451 articles (after duplicates were removed) that were used for subsequent bibliometric analysis. The most relevant differences identified using the Biblioshiny tools are highlighted in Supporting Information Figures S1–S9.

### Knowledge Regarding Simulation of the Gut Microbiome Is Scattered among Several Different Research Fields

**Figure 1:**
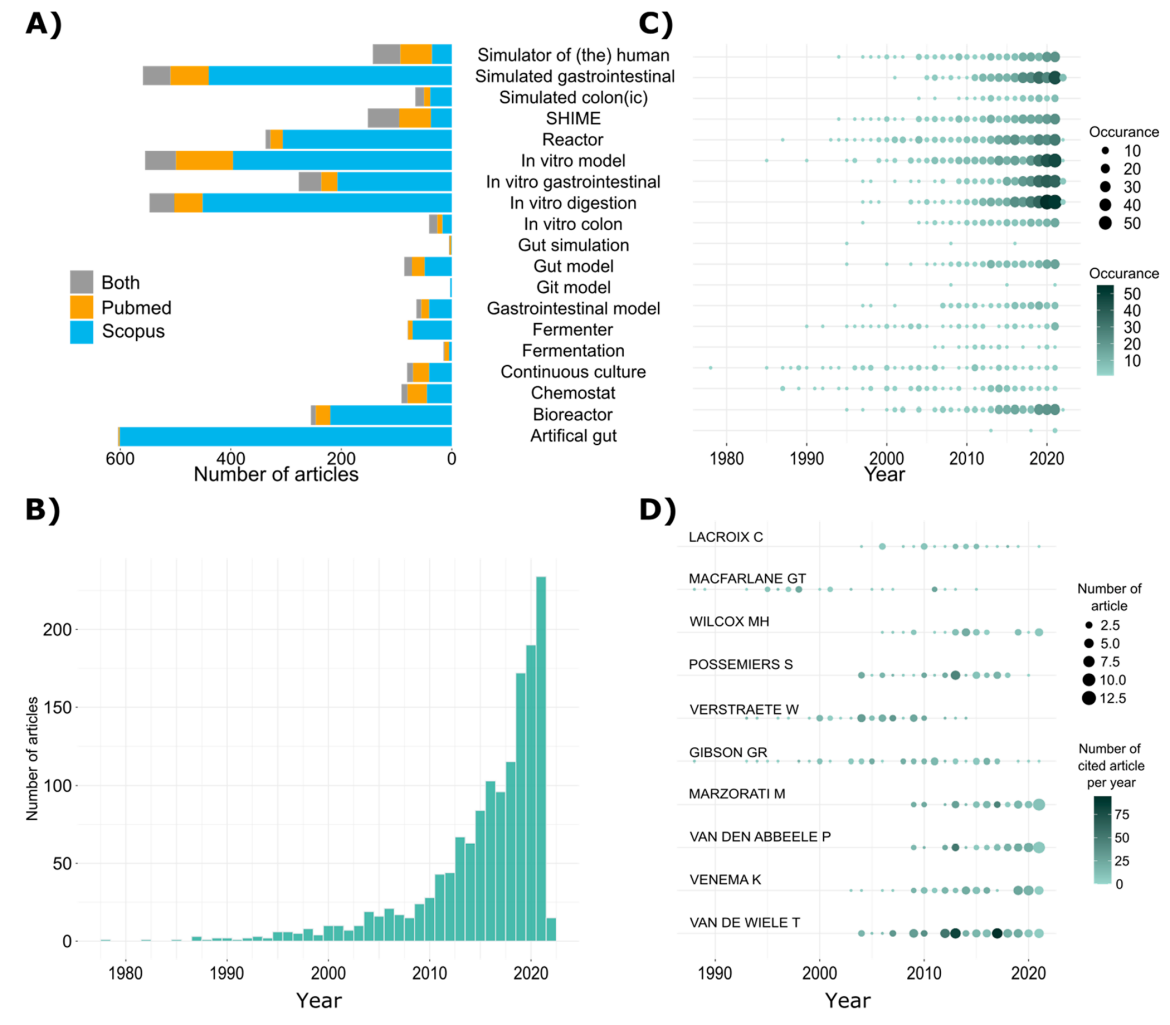
Summary of bibliographic data related to bioreactors. (A) Comparison of the number of articles retrieved from Scopus versus PubMed for the 21 search queries (synonyms for bioreactor and related research) as described in section 2.2 using the second strategy from section 2.3. (B) Histogram showing the number of publications per year from the data set containing 1451 publications. (C) Prevalence of keywords from the data set containing 1451 publications (presence of keywords searched from the title, abstract, and article keyword fields). (D) Top 10 authors with the highest number of publications from the retrieved dataset showing the number of articles over time and yearly average number of times each article has been cited.

For visualization of the data, similar RFST used in literature searches were merged for downstream analysis. These included “simulator of human” and “simulation of the human” which became “Simulator of (the) human”; “simulated colon” and “simulated colonic” were merged as “simulated colon(ic)”. We observed that the group of documents that contributed the highest number of overlaps for PubMed and Scopus were from the search terms “simulated gastrointestinal”, “in vitro digestion”, “in vitro model”, and “reactor” with 19.9%, 16.6%, 15.6%, and 10.1% respectively (Figure 1A). However, these terms also showed the highest percentage of nonduplicated articles; this is clearly demonstrated by the use of “in vitro” in multiple experimental research activities. Surprisingly, the term “bioreactor” was one of the least representative with only 3.1% of duplicates. This term is commonly used in environmental biotechnology and in the bioindustry but, in contrast, is rarely used in the area of pharmaceuticals and medicine (see Supporting Information Table S4). In general, more documents were retrieved from Scopus, but results from PubMed were found to be more reliable in terms of identifying research using true bioreactors.

Bioreactor research has undergone exponential growth since 1980. The exponential growth of article publications (Figure 1B) has occurred since the development of the first “artificial gut” in 1988.^28^ The majority of these articles have been published in food chemistry with the highest number of publications in *Food Research International Journal* (*n* = 60), followed by *Journal Food and Function* (*n* = 59), *Journal of Agricultural and Food Chemistry* (*n* = 58), *Journal of Functional Foods* (*n* = 58), and *Food Chemistry* (*n* = 50). The complete list of journals can be found in Supporting Information Table S8. Between 1978 and 2022, an average of 146.6 new authors appeared each year with a minimum of 2 and a maximum of 876 new authors in the years 1978 and 2021, respectively, again pointing to the emerging body of bioreactor research (see Supporting Information Table S9).

From this search exercise, it is clear that the language used to describe bioreactors has changed over time. Analysis of keywords over time showed that “Continuous Culture”, “Chemostat”, and “Fermenter” appear in earlier publications (Figure 1C). In contrast, “in vitro digestion”, “in vitro gastrointestinal”, “in vitro model”, and “simulated gastrointestinal” appear more frequently in publications from 2013 onward. In particular, within the last 5 years, “in vitro digestion” is one of the most frequently mentioned terms (Figure 1C) and is the most effective terminology to gather articles related to the study of human gut bioreactor models using the Scopus search engine (Figure 1A).

### Author Distribution and Networking

**Figure 2:**
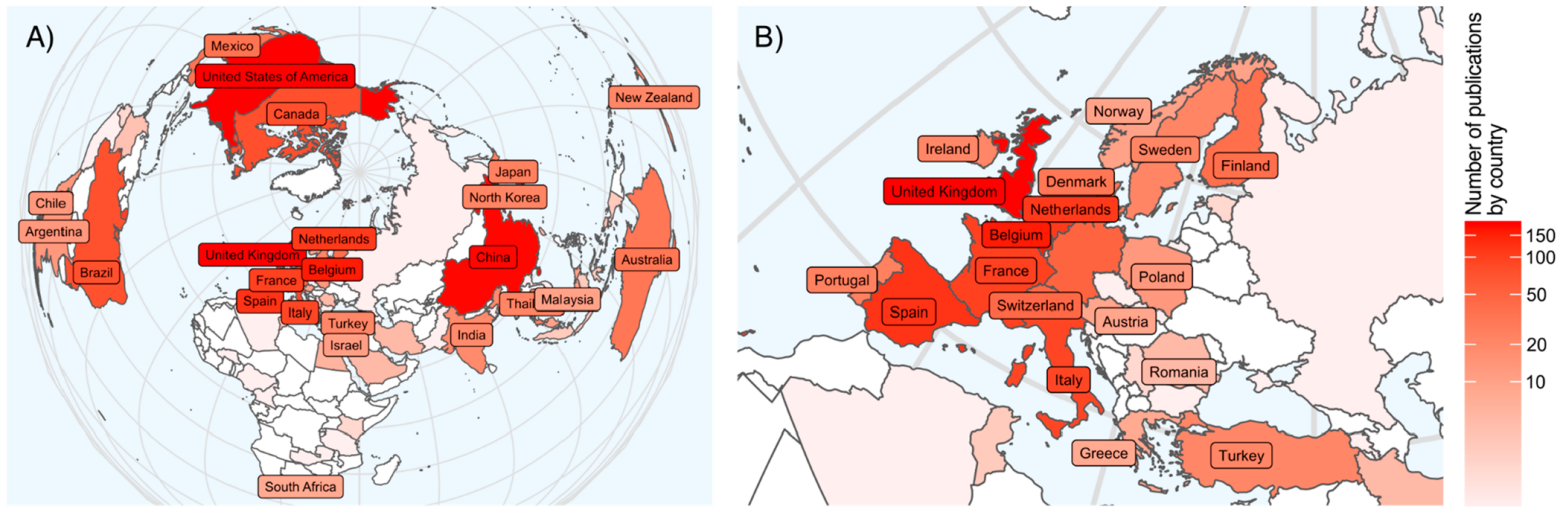
Geographical map displaying the number of publications related to research on the use of bioreactors to study human gut microbiome. (A) World map. (B) Map highlighting countries with the most intensive research output.

In order to identify key bioreactor research hotspots, the authors from selected papers were mapped according to their geographical location (Figure 2). A total of 5718 authors from 52 countries and 5 continents are represented in the list of articles selected, with 1240 authors being associated with more than 1 publication and therefore more likely to use bioreactors as a mainstay of their research. Single-authored documents were observed in less than 1% of cases (*n* = 11), and the average number of authors per article was 3.94. As shown in Figure 1D, the top 10 authors contributed to a total of 405 publications between them, the group of Van De Wiele (Ghent University, Belgium) producing the highest number (*n* = 78).

Of the 10 authors with the highest number of publications, 5 were from Belgium, 3 from the United Kingdom (UK), with 1 each from The Netherlands and Switzerland, indicating a European dominance in the field (see Supporting Information Table S13, for a complete list of countries and the number of publications). However, it can be seen that outside of this group of top-publishing researchers, there are many more groups in human gut bioreactor research that are publishing in the field. A total of 63 of the authors identified from the full list of papers are associated with at least 10 publications, and these span 12 countries and 4 continents (Figure 2A). Of these 63 authors, only 3 authors had 0 connections with other listed researchers. As expected, authors with 10 or more publications are likely to collaborate with colleagues within the same country. For example, Van de Wiele shared up to 23% of publications with fellow Belgium authors (e.g., 18/79 shared publications between Van de Wiele and Possemiers). Of the 12 authors from the United Kingdom with ≥10 publications, only two (Gibson and Rastall) displayed strong international connections. China is the country with the highest number of publications, followed by the United Kingdom, the United States, Belgium, and Spain with 195, 131, 123, 106, and 99 publications, respectively. However, the total number of citations ranked differently, led by the United Kingdom and followed by Belgium, the United States, Ireland, and Spain.

**Figure 3:**
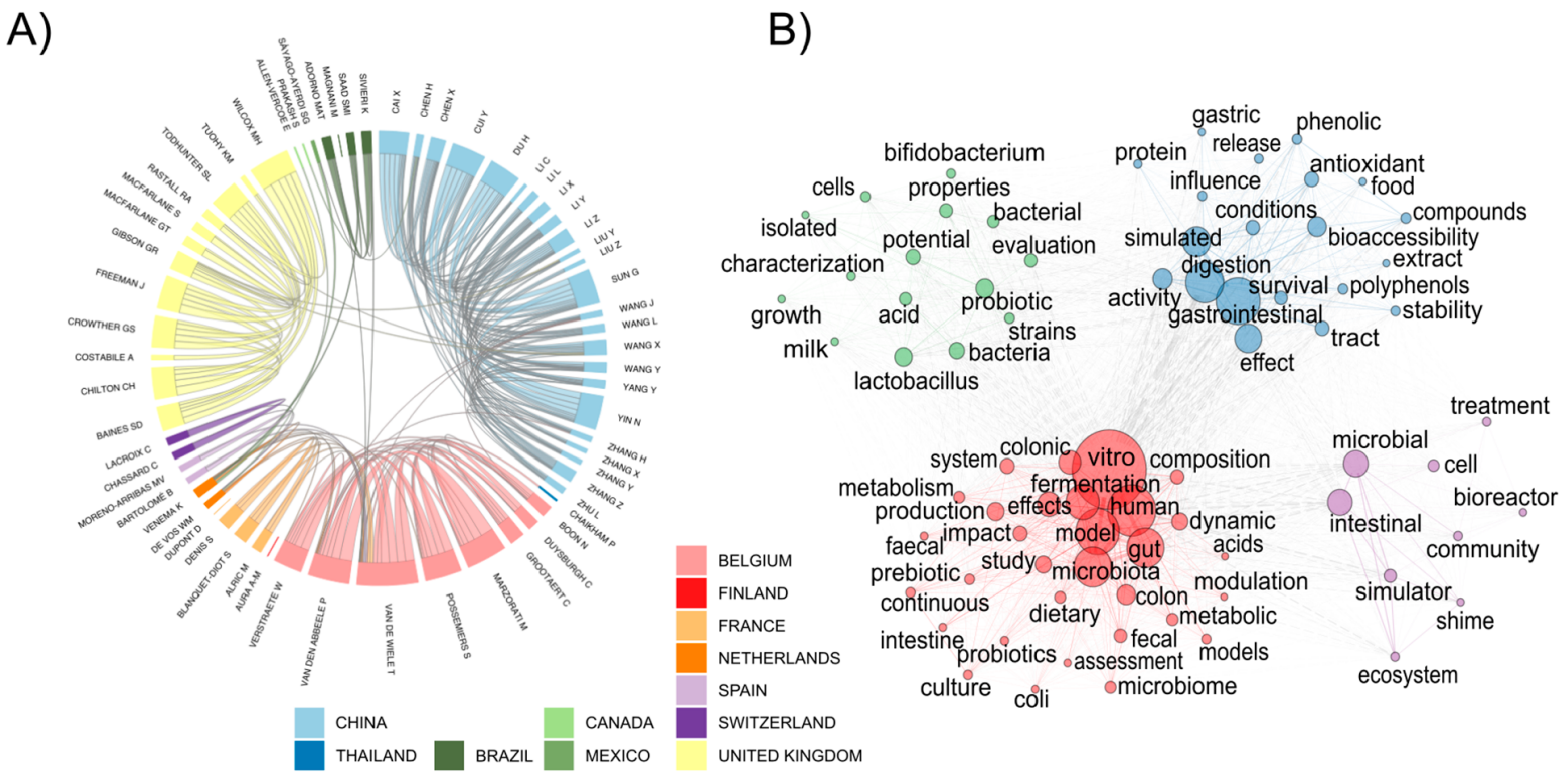
(A) Chord diagram showing collaborations between authors that had 10 or more publications identified in the bibliometric analysis (*n* = 63). Country of authorship was chosen by selecting the most frequently used affiliation (authors are grouped by country and continent). (B) Association network analysis of the top 75 frequently used words within the publication titles (network displayed using the Fruchterman–Reingold method).

The chord diagram in Figure 3A further illustrates that intracountry research networks among authors are substantially more common than intercountry networks. This is particularly true for China and the United Kingdom, whereas Belgium, France, and The Netherlands do not follow that trend. De Vos and Venema from The Netherlands both display strong international networks, and Van de Wiele (also from The Netherlands) contributed with the largest number of publications in collaboration with authors from outside their country of origin, although in the chord diagram this is somewhat obscured by the high level of intracountry coauthors.

### Keyword Network Analysis

The top 5 frequently used words within the publication titles were “vitro”, “human”, “model”, “gastrointestinal”, and “microbiota”, forming the initial network cluster. As more words were added to the network analysis, the cluster was split into subclusters. The first split occurred with 6 words between “vitro” and “human” clusters. With 75 frequently used words, four clusters were formed (Figure 3B). The core words for the biggest cluster depicted in red are “vitro”, “human”, and “model” strongly linked together by 26 connections. The second cluster denoted in blue is formed around the words “gastrointestinal”, “digestion”, and “simulated” along with 16 other words. Unlike the others, the third cluster (green) contains 16 equally spaced terms, led by “probiotic” and “lactobacillus”. The smallest cluster (purple) was formed around the word “intestinal” with 10 other words such as “bioreactor” and “simulator”.

### Overview of Bioreactor Models Described in the Literature

**Figure 4:**
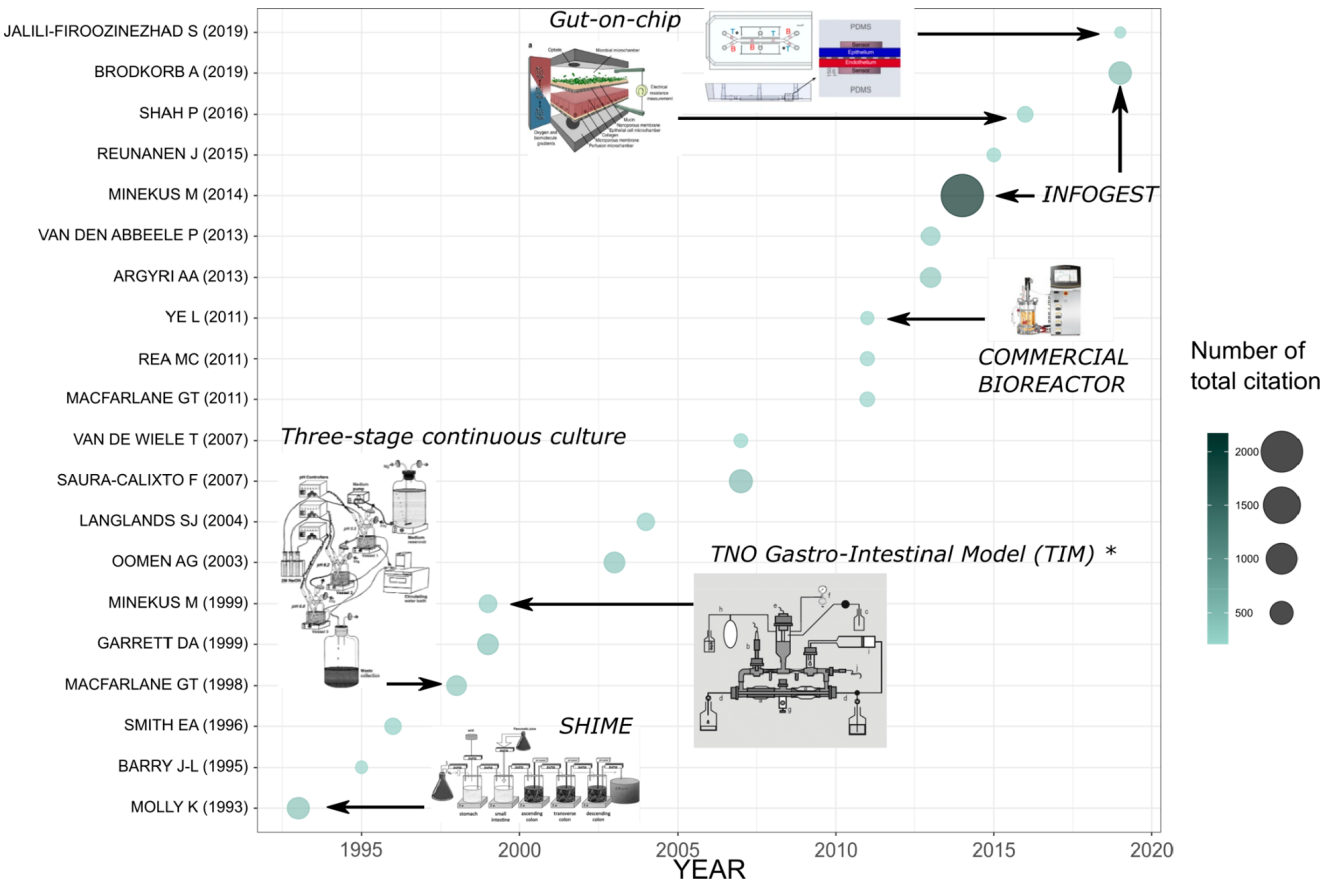
Timeline showing the year of publication for the top 20 most cited articles related to research on the use of bioreactors to study human gut microbiome. Icons are displayed for the major types of bioreactors or models used to replicate human intestinal physiological conditions in vitro. Asterisk (*) denoted the publication by Minekus et al.^30^ that refers to the software component of the system rather than the primary description;^31^ in addition, this system was later renamed as the TNO gastrointestinal model (TIM).

The persistent interest in using bioreactors to model the human gut resulted in the evolution of a variety of apparatus. Figure 4 shows the trends found in bioreactor design based on the top 20 most cited publications in our data set. The first article in Figure 4 was published by Molly et al.^29^ and highlights the recent interest in replication of the human gastrointestinal system in vitro. Earlier designs were complex research laboratory setups, while commercial bioreactors appear later, and more recent designs have had more of an emphasis on miniaturization. The most cited article, by Minekus et al.^13^ with 2172 citations, describes a standardized static in vitro system for modeling adult human digestion. While the oldest articles in this list describe pure cultures of intestinal bacteria, three earlier papers already explored the idea of mixed community microbiome cultivation in bioreactors.

The earliest article in our final search was by Edwar et al. in 1985,^32^ who experimented with human fecal bacteria in continuous flow simulating the proximal colon. In the second by Coutts et al. in 1987,^33^ human gastric samples were taken and physiological conditions were simulated in vitro using a continuous culture chemostat model. The media contained a sum of components for the species’ pure cultures. The third was by Gibson et al. in 1988,^28^ who set up for the first time a series of three continuous flow reactors using gravity as a mechanism to transfer the media. They proposed a complex synthetic medium that has been used as a reference in many later works. The first major attempt to standardize methods among researchers (referred to as the COST Infogest network) was undertaken by Minekus et al.^13^ The authors described standardized methods to replicate in vitro static digestion of food based on physiological conditions (e.g., pH, mineral types, digestion time, and enzymes). This was refined in more detail in Brodkorb et al.^34^ More recent developments, displayed in Figure 4, show a movement toward miniaturization of in vitro methods with microfluidic-based or “gut-on-a-chip” models.^34^ The most recent publication included in our data set was that of Yang et al.,^35^ who used the parameters recommended by Minekus et al.^13^ to study the effect of glutathione on the digestion of albumin.

Eight out of the top 10 authors, determined by the number of their publications and depicted in Figure 1D, worked with only two of the many bioreactor models. Five of these authors were associated with the SHIME system, which is a 5-step multichamber reactor and was developed by Molly et al.^29^ A further 3 of the top 10 authors used a “three-stage continuous culture” model which is also known as the “Triple-stage chemostat” model, which was developed by Gibson et al.^28^ In contrast, Venema’s publications focus on the TIM-1 or TIM-2 (TNO intestinal model) model used for studying the impact of food/nutrients on human health. Lacroix published articles using a range of bioreactor models including single-stage and triple-stage continuous culture models and, more recently, the PolyFermS (Polyfermentor Intestinal Model) model to address research questions centered around infant and elderly populations.^36^ Only 2 of the 10 top authors by publication from the “refined” data set were found in the “unrefined” data set’s top 10 authors by publication.

### Keywords about Microorganism

**Figure 5:**
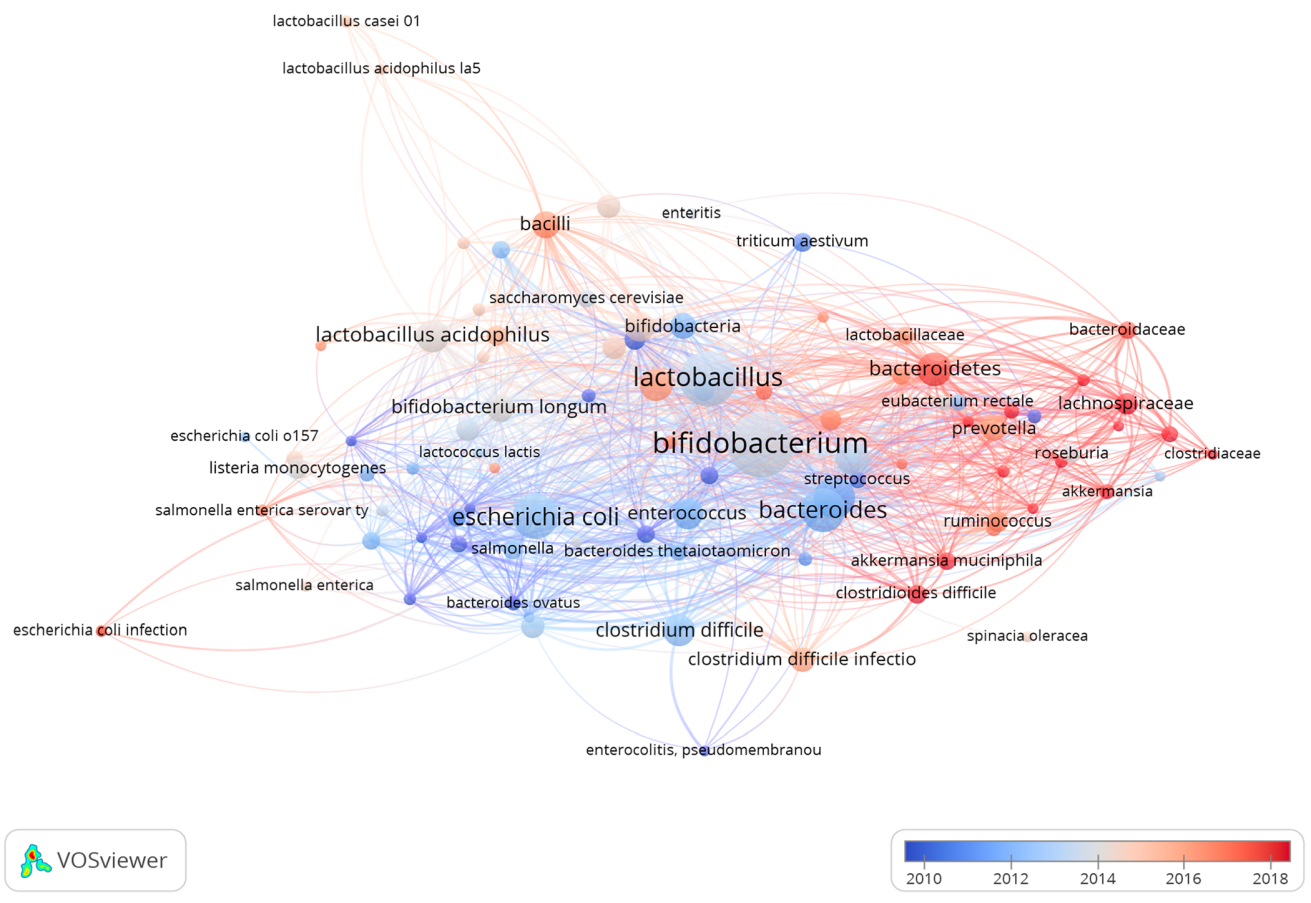
Bacterial taxa identified from the text of articles related to the use of bioreactors to study the human gut microbiome (*n* = 1451). Color of the lines and nodes represent the year of publication with the red clusters displaying the most recent terms, while the blue clusters indicate the terms used in “older literature”.

In this work, we do not delve into the microbial groups or the techniques used for cultivation or classification. Instead, we focus on the co-occurrences of terms related to microbial nomenclature found in the title, abstract, and keyword sections, which were computed and displayed as a graph of relationships (Figure 5). The color of the nodes and edges relates to the year of publication and shows an evolution over time of the microbes of interest to the research communities using bioreactors. The discipline of environmental engineering has made important advances in reducing uncertainty in the study of natural simulations of microbial communities, as reflected in the standardization of methods and parameters, regarding the design of the system and data processing.^37−39^ Similar strategies could be transposed to the topic of human gut microbial engineering taking into account (i) increased sampling efforts to capture geographic diversity in bioreactors, (ii) generation of public protocols for the construction of bioreactors, and (iii) technical support from more established groups. In order to harness the expertise and data generated by multiple research groups and to validate observations, there must be a greater agreement between researchers on the most important variables in bioreactor design and operation with the use of standardized protocols. Ideally, the information generated by researchers should be easily accessible and with unequivocal nomenclature. The discrepancy in the terminology that is currently used to describe experimental equipment and processes hinders progress toward standardization. As shown in the present analysis, the large variation in terms results in difficulty collating bibliographic data and produces a distorted reality.

Although a technical comparison of the different types of bioreactors is beyond the scope of the current review, it is clear that the physical structure, size, flow of media, and physicochemical environment (pH, light, temperature, atmospheric gas, etc.) all influence the diversity, composition, and functionality of the microbial community in the bioreactor, for example, reviews done by Sardelli et al.^40^ and Guzman-Rodriguez et al.^41^ However, prior to consideration of those parameters, it is necessary to understand the landscape of current bioreactor research drawn from the different research fields and the complex and nonstandardized nomenclature used within those disparate research communities. An understanding of how bioreactor research has evolved across different research communities is the first step in providing a platform to enable a standardized comparison of conditions.

To our knowledge, this bibliometric analysis presents the largest compilation of articles about the use of bioreactors for the study of the human gut microbiome. This was made possible by incorporating multiple search terms drawn from disparate research fields. The intention is to provide reference material to enable researchers to identify authors, countries, and bioreactor types and to place this research in the context of bioreactors for human gut simulation. The visualization of top groups, new authors, and topic trends are useful landmarks for supporting experimental decisions, particularly for newcomers to the field who are considering developing capacity for bioreactor research.

It is probable that some articles may have been omitted due to the use of different terms or combinations of terms (for example “gastrointestinal model” vs “model of the gastrointestinal”). In addition, we recognize that manual selection of approximately 30% of the articles performed as described in section 2.2 may have resulted in biased outcomes; however, we found this step necessary due to the large number of articles retrieved from searches that were still not relevant to the topic of interest. Conducting a thorough literature search is time consuming and can be hindered by the massive growth in the number of publications over recent decades.^42^ Even what is considered a “targeted search” in various databases can retrieve many thousands of results. Despite this, we believe the curated list of articles we have generated is highly representative of relevant publications in the field.

For future publications, we recommend the use of “bioreactor to simulate the gut microbiome” since the term bioreactor implies use of in vitro or ex vivo models to simulate in vivo environments. The bioreactor simulates flow conditions and retention time and uses feces or microorganisms from biofilms without prior cultivation, nutrients, and microbial environment in the intestine. If only one of these conditions is simulated, it should be clarified which one, for example, “Human gut bioreactor-simulation of the microbiome”, “Human gut bioreactor-simulation of the colon” or “Human gut bioreactor-simulation of nutritional conditions”, etc. The term fermentor is a term that was coined many years ago when the biochemical basis of ATP synthesis in the oxidative pathways and at the substrate level had not been defined: these two ATP synthesis processes occur in the cultivation of the human microbiota; therefore, they should be omitted as a generic denomination. Another recommendation is to specify the mode of operation of a reactor or series of reactors, i.e., continuous, semicontinuous, or batch with respect to the management of the media composition, since the mode of delivery of nutrients can substantially impact the microbial ecology. For example “Human gut bioreactor-Microbiome simulation-batch”, etc. The adoption of a more standardized terminology among researchers in the field will improve literature searches for related studies in the future.

## Supporting Information Available

Initial queries and results from Scopus and PubMed; results from PubMed using 21 terms; results from Scopus using 21 terms; frequency of key terms generated from searches grouped by year; list of key terms generated from analysis of initial search results (*n* = 460); list of publication titles with DOI for bibliographic data included in the final analysis (*n* = 1451); duplicated term in the searches; source journals of publications (e.g., journal names) (*n* = 401); new authors introduced per year (1978–2022); author’s affiliation country; author’s affiliation institution; new terms introduced per year; summary of countries associated with each publication; search outcome comparison of the publications and citations per year between Refined and Unrefined search queries; prevalence of RFST from the Refined data set containing 1451 publications compared to Unrefined data set containing 2000 publications where no specific keywords were used upon article search; top 10 authors by publication timeline plot; world heatmap illustration of the number of publications per country from two Scopus search query outcomes; most cited articles shown as Global Citations (TC); most cited articles shown as Local Citations (LC); visualization of the thematic evolution in titles presents the main research areas and their evolution using the Sankey diagram; visualization of the trend topics in key terms; visualization of the main items of three fields (country, authors, and word frequency in the title) and their relation through a Sankey diagram (PDF)
