# Supplementary material for: Overview of the Nomenclature and Network of Contributors to the Development of Bioreactors for Human Gut Simulation Using Bibliometric Tools: A Fragmented Landscape
Source: J Agric Food Chem. 2022 Sep 12;70(37):11458–67. doi: 10.1021/acs.jafc.2c03597 (PMC9501909; doi:10.1021/acs.jafc.2c03597)
Supplement: Supplementary file 1 — jf2c03597_si_002.pdf [file jf2c03597_si_002.pdf]

An overview of the nomenclature and network of contributors to the development of bioreactors for human gut simulation using bibliometric tools: A fragmented landscape

Supplementary Material

AUTHORS

Janeth Sanabria[1,2]*, Siobhon Egan[2], Reika Masuda[2], Alex Lee[2], Glenn R. Gibson[3], Jeremy K. Nicholson[2,4], Julien Wist[2,5], Elaine Holmes[2,6]*

1. Environmental Microbiology and Biotechnology Laboratory, Engineering School of Environmental & Natural Resources, Engineering Faculty, Universidad del Valle – Sede Meléndez, Cali, Colombia
2. Australian National Phenome Centre and Computational and Systems Medicine, Health Futures Institute, Murdoch University, Harry Perkins Building, Perth, Australia, WA6150.
3. Department of Food and Nutritional Sciences, University of Reading, Reading, UK.
4. Institute of Global Health Innovation, Faculty of Medicine, Imperial College London, Level 1, Faculty Building, South Kensington Campus, London, SW7 2NA, UK.
5. Chemistry Department, Universidad del Valle, Cali 76001, Colombia.
6. Department of Metabolism, Digestion, and Reproduction, Faculty of Medicine, Imperial College London, Sir Alexander Fleming Building, South Kensington, London SW7 2AZ, UK.

*corresponding authors, Janeth Sanabria (Tel: +572 3212100; Fax: +618 93606491; E-mail: janeth.sanabria@correounivalle.edu.co) and Elaine Holmes (Tel: +618 93601373; Fax: +618 93606491; E-mail: elaine.holmes@murdoch.edu.au).

List of Tables

S1 Initial queries and results from Scopus and Pubmed. 4
S2 Results from PubMed using 21 terms. 6
S3 Results from Scopus using 21 terms. 48
S4 Frequency of key terms generated from searches grouped by year. 62
S5 List of key terms generated from analysis of initial search results (n = 460). 132
S6 List of publication titles with DOI for bibliographic data included in the final analysis (n = 1,451). 135
S7 Duplicated term in the searches. 198
S8 Source journals of publications (e.g. journal names) (n = 401). 250
S9 New authors introduced per year (1978 - 2022). 257
S10 Authors affiliation country. List was retrieved with interllixir program using the same list of documents and manually verified. 258
S11 Authors affiliation institution. List was retrieved with interllixir program using the same list of documents and manually verified. 346
S12 New terms introduced per year. List was retrieved with interllixir program using the same list of documents. 443
S13 Summary of countries associated with each publication. 471

List of Figures

S1 Search outcome comparison of the A) publications and B) citations per year between Refined (red) and Unrefined (blue) search queries. 472
S2 Prevalence of RFST from the (A) Refined dataset containing 1,451 publications (presence of keywords searched from the title, abstract and article keyword fields) compared to (B) Unrefined dataset containing 2,000 publications where no specific keywords were used upon article search. 473
S3 Top 10 authors by publication timeline plot. The number of articles over time is illustrated by the size of the circle and the yearly averaged number of citations is presented by the colour gradient. A) Refined dataset, B) Unrefined dataset. 473
S4 World heatmap illustration of the number of publications per country from two Scopus search query outcomes. A) Refined dataset, B) Unrefined dataset. 474
S5 Most cited articles shown as Global Citations (TC) which represents the total citations that an article has received from the documents indexed. A) Refined dataset, B) Unrefined dataset. 475
S6 Most cited articles shown as Local Citations (LC) which represents the total citations that an article, included in each collection, received internally. A) Refined dataset, B) Unrefined dataset. 476
S7 Visualization of the thematic evolution in titles presents the main research areas and their evolution using the Sankey diagram. A) Refined dataset, B) Unrefined dataset. 477
S8 Visualization of the trend topics in key terms. A) Refined dataset, B) Unrefined dataset. . . . 478
S9 Visualization of the main items of three fields (Country, Authors and word frequency in the title), and their relation through a Sankey diagram. A) Refined dataset, B) Unrefined dataset. 479

Supplementary Material Section 1

Initial queries and searches

Table S1: Initial queries and results from Scopus and Pubmed.

Query	Database	No. documents	Date	Coments
TITLE-ABS-KEY (intestine OR gut OR bowels OR innards OR insides OR viscera OR digestive) AND (microbiota OR microbiome OR microorganism OR bacteria) AND (reactor OR chemostat OR fermenter OR bioreactor OR model OR digester) AND human	Scopus	91777.05	2021-09-01	
ALL (bioreactor OR chemostat OR reactor OR shime OR simulator OR simulation OR dynamic OR model OR vitro) AND ALL (gut OR intestine OR gastrointestinal) AND ALL (microbiota OR microbiome OR microbial)	Scopus	282110.85	2021-09-02	
ALL (bioreactor OR chemostat OR reactor OR shime OR simulator OR simulation OR dynamic OR model OR vitro) AND ALL (gut OR intestine OR gastrointestinal) AND ALL (microbiota OR microbiome OR microbial) AND human	Scopus	242954.13	2021-09-03	
ALL (bioreactor OR chemostat OR reactor OR shime OR simulator OR simulation OR dynamic OR model OR vitro) AND ALL (gut OR intestine OR gastrointestinal) AND ALL (microbiota OR microbiome OR microbial) AND human AND (LIMIT-TO (DOCTYPE , ”ar”)) AND (LIMIT-TO (LANGUAGE , ”English”)) AND (EXCLUDE (EXACTKEYWORD , ”Nonhuman”) OR EXCLUDE (EXACTKEYWORD , ”Animals”) OR EXCLUDE (EXACTKEYWORD , ”Animal”) OR EXCLUDE (EXACTKEYWORD , ”Animal Experiment”) OR EXCLUDE (EXACTKEYWORD , ”Animal Model”) OR EXCLUDE (EXACTKEYWORD , ”Animal Tissue”) OR EXCLUDE (EXACTKEYWORD , ”Mice, Inbred C57BL”) OR EXCLUDE (EXACTKEYWORD , ”Rat”) OR EXCLUDE (EXACTKEYWORD , ”Disease Models, Animal”) OR EXCLUDE (EXACTKEYWORD , ”Rats”) OR EXCLUDE (EXACTKEYWORD , ”In Vivo Study”) OR EXCLUDE (EXACTKEYWORD , ”Animalia”) OR EXCLUDE (EXACTKEYWORD , ”Swine”) OR EXCLUDE (EXACTKEYWORD , ”Mice, Knockout”))	Scopus	64734.00	2021-09-04	From This query the 460 articles were Manually retrieved and the refined process was done
TITLE-ABS-KEY (bioreactor OR chemostat OR reactor OR shime OR simulator OR simulation OR dynamic OR model OR vitro) AND TITLE-ABS-KEY (gut OR intestine OR gastrointestinal) AND TITLE-ABS-KEY (microbiota OR microbiome OR microbial) AND human	Scopus	25724.73	2021-09-05	Include reviews and animals
TITLE-ABS-KEY (bioreactor OR chemostat OR reactor OR shime OR simulator OR simulation OR dynamic OR model OR vitro) AND TITLE-ABS-KEY (gut OR intestine OR gastrointestinal) AND TITLE-ABS-KEY (microbiota OR microbiome OR microbial) AND human AND (LIMIT-TO (DOCTYPE , ”ar”)) AND (LIMIT-TO (LANGUAGE , ”English”)) AND (EXCLUDE (EXACTKEYWORD , ”Nonhuman”) OR EXCLUDE (EXACTKEYWORD , ”Animals”) OR EXCLUDE (EXACTKEYWORD , ”Animal”) OR EXCLUDE (EXACTKEYWORD , ”Animal Experiment”) OR EXCLUDE (EXACTKEYWORD , ”Animal Model”) OR EXCLUDE (EXACTKEYWORD , ”Animal Tissue”) OR EXCLUDE (EXACTKEYWORD , ”Mice, Inbred C57BL”) OR EXCLUDE (EXACTKEYWORD , ”Rat”) OR EXCLUDE (EXACTKEYWORD , ”Disease Models, Animal”) OR EXCLUDE (EXACTKEYWORD , ”Rats”) OR EXCLUDE (EXACTKEYWORD , ”In Vivo Study”) OR EXCLUDE (EXACTKEYWORD , ”Animalia”) OR EXCLUDE (EXACTKEYWORD , ”Swine”) OR EXCLUDE (EXACTKEYWORD , ”Mice, Knockout”))	Scopus	4231.50	2021-09-06	From This query 2000 most cited artcles
(((bioreactor) OR (chemostat) OR (reactor) OR (shime) OR (simulator) OR (simulation) OR (dynamic) OR (model) OR (vitro)) AND ((gut) OR (intestine) OR (gastrointestinal))) AND ((microbiota) OR (microbiome) OR (microbial))	PubMed	11022.00	2021-09-06	From this query the refined search was done

* TITLE-ABS, for the following keywords: Reactor, Bioreactor, Chemostat, Fermenter, Continuous culture, Continuous Fermentation, Simulator of Human, Simulator of the human, Simulated colon, Simulated colonic, Simulated gastrointestinal, In vitro colon, In vitro digestion, In vitro gastrointestinal, In vitro model, Gastrointestinal model, Gut model, Git model, Gut simulation, Artificial gut, and Shime.

Table S2: Results from PubMed using 21 terms.

Search number	Output refined search	Documents	Details
A-1	((”bioreactor s”[All Fields] OR ”bioreactors”[MeSH Terms] OR ”bioreactors”[All Fields] OR ”bioreactor”[All Fields] OR (”chemostat”[All Fields] OR ”chemostatic”[All Fields] OR ”chemostats”[All Fields]) OR (”reactor”[All Fields] OR ”reactor s”[All Fields] OR ”reactors”[All Fields]) OR ”shime”[All Fields] OR (”computer simulation”[MeSH Terms] OR (”computer”[All Fields] AND ”simulation”[All Fields]) OR ”computer simulation”[All Fields] OR ”simulation”[All Fields] OR ”simul”[All Fields] OR ”simulate”[All Fields] OR ”simulated”[All Fields] OR ”simulates”[All Fields] OR ”simulating”[All Fields] OR ”simulation s”[All Fields] OR ”simulational”[All Fields] OR ”simulations”[All Fields] OR ”simulative”[All Fields] OR ”simulator”[All Fields] OR ”simulator s”[All Fields] OR ”simulators”[All Fields]) OR (”computer simulation”[MeSH Terms] OR (”computer”[All Fields] AND ”simulation”[All Fields]) OR ”computer simulation”[All Fields] OR ”simulation”[All Fields] OR ”simul”[All Fields] OR ”simulate”[All Fields] OR ”simulated”[All Fields] OR ”simulates”[All Fields] OR ”simulating”[All Fields] OR ”simulation s”[All Fields] OR ”simulational”[All Fields] OR ”simulations”[All Fields] OR ”simulative”[All Fields] OR ”simulator”[All Fields] OR ”simulator s”[All Fields] OR ”simulators”[All Fields]) OR (”dynamer”[All Fields] OR ”dynamers”[All Fields] OR ”dynamic”[All Fields] OR ”dynamical”[All Fields] OR ”dynamically”[All Fields] OR ”dynamicity”[All Fields] OR ”dynamics”[All Fields] OR ”dynamism”[All Fields] OR ”dynamisms”[All Fields]) OR (”model”[All Fields] OR ”model s”[All Fields] OR ”modeled”[All Fields] OR ”modeler”[All Fields] OR ”modeler s”[All Fields] OR ”modelers”[All Fields] OR ”modeling”[All Fields] OR ”modelings”[All Fields] OR ”modelization”[All Fields] OR ”modelizations”[All Fields] OR ”modelize”[All Fields] OR ”modelized”[All Fields] OR ”modelled”[All Fields] OR ”modeller”[All Fields] OR ”modellers”[All Fields] OR ”modelling”[All Fields] OR ”modellings”[All Fields] OR ”models”[All Fields]) OR ”vitro”[All Fields]) AND (”gut”[Journal] OR ”gut”[All Fields] OR (”intestinalization”[All Fields] OR ”intestinalized”[All Fields] OR ”intestinally”[All Fields] OR ”intestinals”[All Fields] OR ”intestine s”[All Fields] OR ”intestines”[MeSH Terms] OR ”intestines”[All Fields] OR ”intestinal”[All Fields] OR ”intestine”[All Fields]) OR (”gastrointestinal”[All Fields] OR ”gastrointestinally”[All Fields] OR ”gastrointestine”[All Fields])) AND (”microbiota”[MeSH Terms] OR ”microbiota”[All Fields] OR ”microbiotas”[All Fields] OR ”microbiota s”[All Fields] OR ”microbiotae”[All Fields] OR (”microbiome s”[All Fields] OR ”microbiomic”[All Fields] OR ”microbiomics”[All Fields] OR ”microbiota”[MeSH Terms] OR ”microbiota”[All Fields] OR ”microbiome”[All Fields] OR ”microbiomes”[All Fields]) OR (”microbial”[All Fields] OR ”microbially”[All Fields] OR ”microbials”[All Fields])) AND ”humans”[MeSH Terms] AND ”continuous culture”[Title/Abstract]) AND (humans[Filter])	40	

A-2	((”bioreactor s”[All Fields] OR ”bioreactors”[MeSH Terms] OR ”bioreactors”[All Fields] OR ”bioreactor”[All Fields] OR (”chemostat”[All Fields] OR ”chemostatic”[All Fields] OR ”chemostats”[All Fields]) OR (”reactor”[All Fields] OR ”reactor s”[All Fields] OR ”reactors”[All Fields]) OR ”shime”[All Fields] OR (”computer simulation”[MeSH Terms] OR (”computer”[All Fields] AND ”simulation”[All Fields]) OR ”computer simulation”[All Fields] OR ”simulation”[All Fields] OR ”simul”[All Fields] OR ”simulate”[All Fields] OR ”simulated”[All Fields] OR ”simulates”[All Fields] OR ”simulating”[All Fields] OR ”simulation s”[All Fields] OR ”simulational”[All Fields] OR ”simulations”[All Fields] OR ”simulative”[All Fields] OR ”simulator”[All Fields] OR ”simulator s”[All Fields] OR ”simulators”[All Fields]) OR (”computer simulation”[MeSH Terms] OR (”computer”[All Fields] AND ”simulation”[All Fields]) OR ”computer simulation”[All Fields] OR ”simulation”[All Fields] OR ”simul”[All Fields] OR ”simulate”[All Fields] OR ”simulated”[All Fields] OR ”simulates”[All Fields] OR ”simulating”[All Fields] OR ”simulation s”[All Fields] OR ”simulational”[All Fields] OR ”simulations”[All Fields] OR ”simulative”[All Fields] OR ”simulator”[All Fields] OR ”simulator s”[All Fields] OR ”simulators”[All Fields]) OR (”dynamer”[All Fields] OR ”dynamers”[All Fields] OR ”dynamic”[All Fields] OR ”dynamical”[All Fields] OR ”dynamically”[All Fields] OR ”dynamicity”[All Fields] OR ”dynamics”[All Fields] OR ”dynamism”[All Fields] OR ”dynamisms”[All Fields]) OR (”model”[All Fields] OR ”model s”[All Fields] OR ”modeled”[All Fields] OR ”modeler”[All Fields] OR ”modeler s”[All Fields] OR ”modelers”[All Fields] OR ”modeling”[All Fields] OR ”modelings”[All Fields] OR ”modelization”[All Fields] OR ”modelizations”[All Fields] OR ”modelize”[All Fields] OR ”modelized”[All Fields] OR ”modelled”[All Fields] OR ”modeller”[All Fields] OR ”modellers”[All Fields] OR ”modelling”[All Fields] OR ”modellings”[All Fields] OR ”models”[All Fields]) OR ”vitro”[All Fields]) AND (”gut”[Journal] OR ”gut”[All Fields] OR (”intestinalization”[All Fields] OR ”intestinalized”[All Fields] OR ”intestinally”[All Fields] OR ”intestinals”[All Fields] OR ”intestine s”[All Fields] OR ”intestines”[MeSH Terms] OR ”intestines”[All Fields] OR ”intestinal”[All Fields] OR ”intestine”[All Fields]) OR (”gastrointestinal”[All Fields] OR ”gastrointestinally”[All Fields] OR ”gastrointestine”[All Fields])) AND (”microbiota”[MeSH Terms] OR ”microbiota”[All Fields] OR ”microbiotas”[All Fields] OR ”microbiota s”[All Fields] OR ”microbiotae”[All Fields] OR (”microbiome s”[All Fields] OR ”microbiomic”[All Fields] OR ”microbiomics”[All Fields] OR ”microbiota”[MeSH Terms] OR ”microbiota”[All Fields] OR ”microbiome”[All Fields] OR ”microbiomes”[All Fields]) OR (”microbial”[All Fields] OR ”microbially”[All Fields] OR ”microbials”[All Fields])) AND ”humans”[MeSH Terms] AND ”continuous fermentation”[Title/Abstract]) AND (humans[Filter])	10	1899-12-31 23:40:55

A-3	((”bioreactor s”[All Fields] OR ”bioreactors”[MeSH Terms] OR ”bioreactors”[All Fields] OR ”bioreactor”[All Fields] OR (”chemostat”[All Fields] OR ”chemostatic”[All Fields] OR ”chemostats”[All Fields]) OR (”reactor”[All Fields] OR ”reactor s”[All Fields] OR ”reactors”[All Fields]) OR ”shime”[All Fields] OR (”computer simulation”[MeSH Terms] OR (”computer”[All Fields] AND ”simulation”[All Fields]) OR ”computer simulation”[All Fields] OR ”simulation”[All Fields] OR ”simul”[All Fields] OR ”simulate”[All Fields] OR ”simulated”[All Fields] OR ”simulates”[All Fields] OR ”simulating”[All Fields] OR ”simulation s”[All Fields] OR ”simulational”[All Fields] OR ”simulations”[All Fields] OR ”simulative”[All Fields] OR ”simulator”[All Fields] OR ”simulator s”[All Fields] OR ”simulators”[All Fields]) OR (”computer simulation”[MeSH Terms] OR (”computer”[All Fields] AND ”simulation”[All Fields]) OR ”computer simulation”[All Fields] OR ”simulation”[All Fields] OR ”simul”[All Fields] OR ”simulate”[All Fields] OR ”simulated”[All Fields] OR ”simulates”[All Fields] OR ”simulating”[All Fields] OR ”simulation s”[All Fields] OR ”simulational”[All Fields] OR ”simulations”[All Fields] OR ”simulative”[All Fields] OR ”simulator”[All Fields] OR ”simulator s”[All Fields] OR ”simulators”[All Fields]) OR (”dynamer”[All Fields] OR ”dynamers”[All Fields] OR ”dynamic”[All Fields] OR ”dynamical”[All Fields] OR ”dynamically”[All Fields] OR ”dynamicity”[All Fields] OR ”dynamics”[All Fields] OR ”dynamism”[All Fields] OR ”dynamisms”[All Fields]) OR (”model”[All Fields] OR ”model s”[All Fields] OR ”modeled”[All Fields] OR ”modeler”[All Fields] OR ”modeler s”[All Fields] OR ”modelers”[All Fields] OR ”modeling”[All Fields] OR ”modelings”[All Fields] OR ”modelization”[All Fields] OR ”modelizations”[All Fields] OR ”modelize”[All Fields] OR ”modelized”[All Fields] OR ”modelled”[All Fields] OR ”modeller”[All Fields] OR ”modellers”[All Fields] OR ”modelling”[All Fields] OR ”modellings”[All Fields] OR ”models”[All Fields]) OR ”vitro”[All Fields]) AND (”gut”[Journal] OR ”gut”[All Fields] OR (”intestinalization”[All Fields] OR ”intestinalized”[All Fields] OR ”intestinally”[All Fields] OR ”intestinals”[All Fields] OR ”intestine s”[All Fields] OR ”intestines”[MeSH Terms] OR ”intestines”[All Fields] OR ”intestinal”[All Fields] OR ”intestine”[All Fields]) OR (”gastrointestinal”[All Fields] OR ”gastrointestinally”[All Fields] OR ”gastrointestine”[All Fields])) AND (”microbiota”[MeSH Terms] OR ”microbiota”[All Fields] OR ”microbiotas”[All Fields] OR ”microbiota s”[All Fields] OR ”microbiotae”[All Fields] OR (”microbiome s”[All Fields] OR ”microbiomic”[All Fields] OR ”microbiomics”[All Fields] OR ”microbiota”[MeSH Terms] OR ”microbiota”[All Fields] OR ”microbiome”[All Fields] OR ”microbiomes”[All Fields]) OR (”microbial”[All Fields] OR ”microbially”[All Fields] OR ”microbials”[All Fields])) AND ”humans”[MeSH Terms] AND ”gastrointestinal model”[Title/Abstract]) AND (humans[Filter])	23	1899-12-31 23:43:15

A-4	((”bioreactor s”[All Fields] OR ”bioreactors”[MeSH Terms] OR ”bioreactors”[All Fields] OR ”bioreactor”[All Fields] OR (”chemostat”[All Fields] OR ”chemostatic”[All Fields] OR ”chemostats”[All Fields]) OR (”reactor”[All Fields] OR ”reactor s”[All Fields] OR ”reactors”[All Fields]) OR ”shime”[All Fields] OR (”computer simulation”[MeSH Terms] OR (”computer”[All Fields] AND ”simulation”[All Fields]) OR ”computer simulation”[All Fields] OR ”simulation”[All Fields] OR ”simul”[All Fields] OR ”simulate”[All Fields] OR ”simulated”[All Fields] OR ”simulates”[All Fields] OR ”simulating”[All Fields] OR ”simulation s”[All Fields] OR ”simulational”[All Fields] OR ”simulations”[All Fields] OR ”simulative”[All Fields] OR ”simulator”[All Fields] OR ”simulator s”[All Fields] OR ”simulators”[All Fields]) OR (”computer simulation”[MeSH Terms] OR (”computer”[All Fields] AND ”simulation”[All Fields]) OR ”computer simulation”[All Fields] OR ”simulation”[All Fields] OR ”simul”[All Fields] OR ”simulate”[All Fields] OR ”simulated”[All Fields] OR ”simulates”[All Fields] OR ”simulating”[All Fields] OR ”simulation s”[All Fields] OR ”simulational”[All Fields] OR ”simulations”[All Fields] OR ”simulative”[All Fields] OR ”simulator”[All Fields] OR ”simulator s”[All Fields] OR ”simulators”[All Fields]) OR (”dynamer”[All Fields] OR ”dynamers”[All Fields] OR ”dynamic”[All Fields] OR ”dynamical”[All Fields] OR ”dynamically”[All Fields] OR ”dynamicity”[All Fields] OR ”dynamics”[All Fields] OR ”dynamism”[All Fields] OR ”dynamisms”[All Fields]) OR (”model”[All Fields] OR ”model s”[All Fields] OR ”modeled”[All Fields] OR ”modeler”[All Fields] OR ”modeler s”[All Fields] OR ”modelers”[All Fields] OR ”modeling”[All Fields] OR ”modelings”[All Fields] OR ”modelization”[All Fields] OR ”modelizations”[All Fields] OR ”modelize”[All Fields] OR ”modelized”[All Fields] OR ”modelled”[All Fields] OR ”modeller”[All Fields] OR ”modellers”[All Fields] OR ”modelling”[All Fields] OR ”modellings”[All Fields] OR ”models”[All Fields]) OR ”vitro”[All Fields]) AND (”gut”[Journal] OR ”gut”[All Fields] OR (”intestinalization”[All Fields] OR ”intestinalized”[All Fields] OR ”intestinally”[All Fields] OR ”intestinals”[All Fields] OR ”intestine s”[All Fields] OR ”intestines”[MeSH Terms] OR ”intestines”[All Fields] OR ”intestinal”[All Fields] OR ”intestine”[All Fields]) OR (”gastrointestinal”[All Fields] OR ”gastrointestinally”[All Fields] OR ”gastrointestine”[All Fields])) AND (”microbiota”[MeSH Terms] OR ”microbiota”[All Fields] OR ”microbiotas”[All Fields] OR ”microbiota s”[All Fields] OR ”microbiotae”[All Fields] OR (”microbiome s”[All Fields] OR ”microbiomic”[All Fields] OR ”microbiomics”[All Fields] OR ”microbiota”[MeSH Terms] OR ”microbiota”[All Fields] OR ”microbiome”[All Fields] OR ”microbiomes”[All Fields]) OR (”microbial”[All Fields] OR ”microbially”[All Fields] OR ”microbials”[All Fields])) AND ”humans”[MeSH Terms] AND ”git model”[Title/Abstract]) AND (humans[Filter])	0	1899-12-31 23:45:07

A-5	((”bioreactor s”[All Fields] OR ”bioreactors”[MeSH Terms] OR ”bioreactors”[All Fields] OR ”bioreactor”[All Fields] OR (”chemostat”[All Fields] OR ”chemostatic”[All Fields] OR ”chemostats”[All Fields]) OR (”reactor”[All Fields] OR ”reactor s”[All Fields] OR ”reactors”[All Fields]) OR ”shime”[All Fields] OR (”computer simulation”[MeSH Terms] OR (”computer”[All Fields] AND ”simulation”[All Fields]) OR ”computer simulation”[All Fields] OR ”simulation”[All Fields] OR ”simul”[All Fields] OR ”simulate”[All Fields] OR ”simulated”[All Fields] OR ”simulates”[All Fields] OR ”simulating”[All Fields] OR ”simulation s”[All Fields] OR ”simulational”[All Fields] OR ”simulations”[All Fields] OR ”simulative”[All Fields] OR ”simulator”[All Fields] OR ”simulator s”[All Fields] OR ”simulators”[All Fields]) OR (”computer simulation”[MeSH Terms] OR (”computer”[All Fields] AND ”simulation”[All Fields]) OR ”computer simulation”[All Fields] OR ”simulation”[All Fields] OR ”simul”[All Fields] OR ”simulate”[All Fields] OR ”simulated”[All Fields] OR ”simulates”[All Fields] OR ”simulating”[All Fields] OR ”simulation s”[All Fields] OR ”simulational”[All Fields] OR ”simulations”[All Fields] OR ”simulative”[All Fields] OR ”simulator”[All Fields] OR ”simulator s”[All Fields] OR ”simulators”[All Fields]) OR (”dynamer”[All Fields] OR ”dynamers”[All Fields] OR ”dynamic”[All Fields] OR ”dynamical”[All Fields] OR ”dynamically”[All Fields] OR ”dynamicity”[All Fields] OR ”dynamics”[All Fields] OR ”dynamism”[All Fields] OR ”dynamisms”[All Fields]) OR (”model”[All Fields] OR ”model s”[All Fields] OR ”modeled”[All Fields] OR ”modeler”[All Fields] OR ”modeler s”[All Fields] OR ”modelers”[All Fields] OR ”modeling”[All Fields] OR ”modelings”[All Fields] OR ”modelization”[All Fields] OR ”modelizations”[All Fields] OR ”modelize”[All Fields] OR ”modelized”[All Fields] OR ”modelled”[All Fields] OR ”modeller”[All Fields] OR ”modellers”[All Fields] OR ”modelling”[All Fields] OR ”modellings”[All Fields] OR ”models”[All Fields]) OR ”vitro”[All Fields]) AND (”gut”[Journal] OR ”gut”[All Fields] OR (”intestinalization”[All Fields] OR ”intestinalized”[All Fields] OR ”intestinally”[All Fields] OR ”intestinals”[All Fields] OR ”intestine s”[All Fields] OR ”intestines”[MeSH Terms] OR ”intestines”[All Fields] OR ”intestinal”[All Fields] OR ”intestine”[All Fields]) OR (”gastrointestinal”[All Fields] OR ”gastrointestinally”[All Fields] OR ”gastrointestine”[All Fields])) AND (”microbiota”[MeSH Terms] OR ”microbiota”[All Fields] OR ”microbiotas”[All Fields] OR ”microbiota s”[All Fields] OR ”microbiotae”[All Fields] OR (”microbiome s”[All Fields] OR ”microbiomic”[All Fields] OR ”microbiomics”[All Fields] OR ”microbiota”[MeSH Terms] OR ”microbiota”[All Fields] OR ”microbiome”[All Fields] OR ”microbiomes”[All Fields]) OR (”microbial”[All Fields] OR ”microbially”[All Fields] OR ”microbials”[All Fields])) AND ”humans”[MeSH Terms] AND ”gut model”[Title/Abstract]) AND (humans[Filter])	75	1899-12-31 23:45:07

A-6	((”bioreactor s”[All Fields] OR ”bioreactors”[MeSH Terms] OR ”bioreactors”[All Fields] OR ”bioreactor”[All Fields] OR (”chemostat”[All Fields] OR ”chemostatic”[All Fields] OR ”chemostats”[All Fields]) OR (”reactor”[All Fields] OR ”reactor s”[All Fields] OR ”reactors”[All Fields]) OR ”shime”[All Fields] OR (”computer simulation”[MeSH Terms] OR (”computer”[All Fields] AND ”simulation”[All Fields]) OR ”computer simulation”[All Fields] OR ”simulation”[All Fields] OR ”simul”[All Fields] OR ”simulate”[All Fields] OR ”simulated”[All Fields] OR ”simulates”[All Fields] OR ”simulating”[All Fields] OR ”simulation s”[All Fields] OR ”simulational”[All Fields] OR ”simulations”[All Fields] OR ”simulative”[All Fields] OR ”simulator”[All Fields] OR ”simulator s”[All Fields] OR ”simulators”[All Fields]) OR (”computer simulation”[MeSH Terms] OR (”computer”[All Fields] AND ”simulation”[All Fields]) OR ”computer simulation”[All Fields] OR ”simulation”[All Fields] OR ”simul”[All Fields] OR ”simulate”[All Fields] OR ”simulated”[All Fields] OR ”simulates”[All Fields] OR ”simulating”[All Fields] OR ”simulation s”[All Fields] OR ”simulational”[All Fields] OR ”simulations”[All Fields] OR ”simulative”[All Fields] OR ”simulator”[All Fields] OR ”simulator s”[All Fields] OR ”simulators”[All Fields]) OR (”dynamer”[All Fields] OR ”dynamers”[All Fields] OR ”dynamic”[All Fields] OR ”dynamical”[All Fields] OR ”dynamically”[All Fields] OR ”dynamicity”[All Fields] OR ”dynamics”[All Fields] OR ”dynamism”[All Fields] OR ”dynamisms”[All Fields]) OR (”model”[All Fields] OR ”model s”[All Fields] OR ”modeled”[All Fields] OR ”modeler”[All Fields] OR ”modeler s”[All Fields] OR ”modelers”[All Fields] OR ”modeling”[All Fields] OR ”modelings”[All Fields] OR ”modelization”[All Fields] OR ”modelizations”[All Fields] OR ”modelize”[All Fields] OR ”modelized”[All Fields] OR ”modelled”[All Fields] OR ”modeller”[All Fields] OR ”modellers”[All Fields] OR ”modelling”[All Fields] OR ”modellings”[All Fields] OR ”models”[All Fields]) OR ”vitro”[All Fields]) AND (”gut”[Journal] OR ”gut”[All Fields] OR (”intestinalization”[All Fields] OR ”intestinalized”[All Fields] OR ”intestinally”[All Fields] OR ”intestinals”[All Fields] OR ”intestine s”[All Fields] OR ”intestines”[MeSH Terms] OR ”intestines”[All Fields] OR ”intestinal”[All Fields] OR ”intestine”[All Fields]) OR (”gastrointestinal”[All Fields] OR ”gastrointestinally”[All Fields] OR ”gastrointestine”[All Fields])) AND (”microbiota”[MeSH Terms] OR ”microbiota”[All Fields] OR ”microbiotas”[All Fields] OR ”microbiota s”[All Fields] OR ”microbiotae”[All Fields] OR (”microbiome s”[All Fields] OR ”microbiomic”[All Fields] OR ”microbiomics”[All Fields] OR ”microbiota”[MeSH Terms] OR ”microbiota”[All Fields] OR ”microbiome”[All Fields] OR ”microbiomes”[All Fields]) OR (”microbial”[All Fields] OR ”microbially”[All Fields] OR ”microbials”[All Fields])) AND ”humans”[MeSH Terms] AND ”gut simulation”[Title/Abstract]) AND (humans[Filter])	4	1899-12-31 23:46:13

A-7	((”bioreactor s”[All Fields] OR ”bioreactors”[MeSH Terms] OR ”bioreactors”[All Fields] OR ”bioreactor”[All Fields] OR (”chemostat”[All Fields] OR ”chemostatic”[All Fields] OR ”chemostats”[All Fields]) OR (”reactor”[All Fields] OR ”reactor s”[All Fields] OR ”reactors”[All Fields]) OR ”shime”[All Fields] OR (”computer simulation”[MeSH Terms] OR (”computer”[All Fields] AND ”simulation”[All Fields]) OR ”computer simulation”[All Fields] OR ”simulation”[All Fields] OR ”simul”[All Fields] OR ”simulate”[All Fields] OR ”simulated”[All Fields] OR ”simulates”[All Fields] OR ”simulating”[All Fields] OR ”simulation s”[All Fields] OR ”simulational”[All Fields] OR ”simulations”[All Fields] OR ”simulative”[All Fields] OR ”simulator”[All Fields] OR ”simulator s”[All Fields] OR ”simulators”[All Fields]) OR (”computer simulation”[MeSH Terms] OR (”computer”[All Fields] AND ”simulation”[All Fields]) OR ”computer simulation”[All Fields] OR ”simulation”[All Fields] OR ”simul”[All Fields] OR ”simulate”[All Fields] OR ”simulated”[All Fields] OR ”simulates”[All Fields] OR ”simulating”[All Fields] OR ”simulation s”[All Fields] OR ”simulational”[All Fields] OR ”simulations”[All Fields] OR ”simulative”[All Fields] OR ”simulator”[All Fields] OR ”simulator s”[All Fields] OR ”simulators”[All Fields]) OR (”dynamer”[All Fields] OR ”dynamers”[All Fields] OR ”dynamic”[All Fields] OR ”dynamical”[All Fields] OR ”dynamically”[All Fields] OR ”dynamicity”[All Fields] OR ”dynamics”[All Fields] OR ”dynamism”[All Fields] OR ”dynamisms”[All Fields]) OR (”model”[All Fields] OR ”model s”[All Fields] OR ”modeled”[All Fields] OR ”modeler”[All Fields] OR ”modeler s”[All Fields] OR ”modelers”[All Fields] OR ”modeling”[All Fields] OR ”modelings”[All Fields] OR ”modelization”[All Fields] OR ”modelizations”[All Fields] OR ”modelize”[All Fields] OR ”modelized”[All Fields] OR ”modelled”[All Fields] OR ”modeller”[All Fields] OR ”modellers”[All Fields] OR ”modelling”[All Fields] OR ”modellings”[All Fields] OR ”models”[All Fields]) OR ”vitro”[All Fields]) AND (”gut”[Journal] OR ”gut”[All Fields] OR (”intestinalization”[All Fields] OR ”intestinalized”[All Fields] OR ”intestinally”[All Fields] OR ”intestinals”[All Fields] OR ”intestine s”[All Fields] OR ”intestines”[MeSH Terms] OR ”intestines”[All Fields] OR ”intestinal”[All Fields] OR ”intestine”[All Fields]) OR (”gastrointestinal”[All Fields] OR ”gastrointestinally”[All Fields] OR ”gastrointestine”[All Fields])) AND (”microbiota”[MeSH Terms] OR ”microbiota”[All Fields] OR ”microbiotas”[All Fields] OR ”microbiota s”[All Fields] OR ”microbiotae”[All Fields] OR (”microbiome s”[All Fields] OR ”microbiomic”[All Fields] OR ”microbiomics”[All Fields] OR ”microbiota”[MeSH Terms] OR ”microbiota”[All Fields] OR ”microbiome”[All Fields] OR ”microbiomes”[All Fields]) OR (”microbial”[All Fields] OR ”microbially”[All Fields] OR ”microbials”[All Fields])) AND ”humans”[MeSH Terms] AND ”in vitro colon”[Title/Abstract]) AND (humans[Filter])	24	1899-12-31 23:47:18

A-8	((”bioreactor s”[All Fields] OR ”bioreactors”[MeSH Terms] OR ”bioreactors”[All Fields] OR ”bioreactor”[All Fields] OR (”chemostat”[All Fields] OR ”chemostatic”[All Fields] OR ”chemostats”[All Fields]) OR (”reactor”[All Fields] OR ”reactor s”[All Fields] OR ”reactors”[All Fields]) OR ”shime”[All Fields] OR (”computer simulation”[MeSH Terms] OR (”computer”[All Fields] AND ”simulation”[All Fields]) OR ”computer simulation”[All Fields] OR ”simulation”[All Fields] OR ”simul”[All Fields] OR ”simulate”[All Fields] OR ”simulated”[All Fields] OR ”simulates”[All Fields] OR ”simulating”[All Fields] OR ”simulation s”[All Fields] OR ”simulational”[All Fields] OR ”simulations”[All Fields] OR ”simulative”[All Fields] OR ”simulator”[All Fields] OR ”simulator s”[All Fields] OR ”simulators”[All Fields]) OR (”computer simulation”[MeSH Terms] OR (”computer”[All Fields] AND ”simulation”[All Fields]) OR ”computer simulation”[All Fields] OR ”simulation”[All Fields] OR ”simul”[All Fields] OR ”simulate”[All Fields] OR ”simulated”[All Fields] OR ”simulates”[All Fields] OR ”simulating”[All Fields] OR ”simulation s”[All Fields] OR ”simulational”[All Fields] OR ”simulations”[All Fields] OR ”simulative”[All Fields] OR ”simulator”[All Fields] OR ”simulator s”[All Fields] OR ”simulators”[All Fields]) OR (”dynamer”[All Fields] OR ”dynamers”[All Fields] OR ”dynamic”[All Fields] OR ”dynamical”[All Fields] OR ”dynamically”[All Fields] OR ”dynamicity”[All Fields] OR ”dynamics”[All Fields] OR ”dynamism”[All Fields] OR ”dynamisms”[All Fields]) OR (”model”[All Fields] OR ”model s”[All Fields] OR ”modeled”[All Fields] OR ”modeler”[All Fields] OR ”modeler s”[All Fields] OR ”modelers”[All Fields] OR ”modeling”[All Fields] OR ”modelings”[All Fields] OR ”modelization”[All Fields] OR ”modelizations”[All Fields] OR ”modelize”[All Fields] OR ”modelized”[All Fields] OR ”modelled”[All Fields] OR ”modeller”[All Fields] OR ”modellers”[All Fields] OR ”modelling”[All Fields] OR ”modellings”[All Fields] OR ”models”[All Fields]) OR ”vitro”[All Fields]) AND (”gut”[Journal] OR ”gut”[All Fields] OR (”intestinalization”[All Fields] OR ”intestinalized”[All Fields] OR ”intestinally”[All Fields] OR ”intestinals”[All Fields] OR ”intestine s”[All Fields] OR ”intestines”[MeSH Terms] OR ”intestines”[All Fields] OR ”intestinal”[All Fields] OR ”intestine”[All Fields]) OR (”gastrointestinal”[All Fields] OR ”gastrointestinally”[All Fields] OR ”gastrointestine”[All Fields])) AND (”microbiota”[MeSH Terms] OR ”microbiota”[All Fields] OR ”microbiotas”[All Fields] OR ”microbiota s”[All Fields] OR ”microbiotae”[All Fields] OR (”microbiome s”[All Fields] OR ”microbiomic”[All Fields] OR ”microbiomics”[All Fields] OR ”microbiota”[MeSH Terms] OR ”microbiota”[All Fields] OR ”microbiome”[All Fields] OR ”microbiomes”[All Fields]) OR (”microbial”[All Fields] OR ”microbially”[All Fields] OR ”microbials”[All Fields])) AND ”humans”[MeSH Terms] AND ”in vitro digestion”[Title/Abstract]) AND (humans[Filter])	96	1899-12-31 23:48:50

A-9	((”bioreactor s”[All Fields] OR ”bioreactors”[MeSH Terms] OR ”bioreactors”[All Fields] OR ”bioreactor”[All Fields] OR (”chemostat”[All Fields] OR ”chemostatic”[All Fields] OR ”chemostats”[All Fields]) OR (”reactor”[All Fields] OR ”reactor s”[All Fields] OR ”reactors”[All Fields]) OR ”shime”[All Fields] OR (”computer simulation”[MeSH Terms] OR (”computer”[All Fields] AND ”simulation”[All Fields]) OR ”computer simulation”[All Fields] OR ”simulation”[All Fields] OR ”simul”[All Fields] OR ”simulate”[All Fields] OR ”simulated”[All Fields] OR ”simulates”[All Fields] OR ”simulating”[All Fields] OR ”simulation s”[All Fields] OR ”simulational”[All Fields] OR ”simulations”[All Fields] OR ”simulative”[All Fields] OR ”simulator”[All Fields] OR ”simulator s”[All Fields] OR ”simulators”[All Fields]) OR (”computer simulation”[MeSH Terms] OR (”computer”[All Fields] AND ”simulation”[All Fields]) OR ”computer simulation”[All Fields] OR ”simulation”[All Fields] OR ”simul”[All Fields] OR ”simulate”[All Fields] OR ”simulated”[All Fields] OR ”simulates”[All Fields] OR ”simulating”[All Fields] OR ”simulation s”[All Fields] OR ”simulational”[All Fields] OR ”simulations”[All Fields] OR ”simulative”[All Fields] OR ”simulator”[All Fields] OR ”simulator s”[All Fields] OR ”simulators”[All Fields]) OR (”dynamer”[All Fields] OR ”dynamers”[All Fields] OR ”dynamic”[All Fields] OR ”dynamical”[All Fields] OR ”dynamically”[All Fields] OR ”dynamicity”[All Fields] OR ”dynamics”[All Fields] OR ”dynamism”[All Fields] OR ”dynamisms”[All Fields]) OR (”model”[All Fields] OR ”model s”[All Fields] OR ”modeled”[All Fields] OR ”modeler”[All Fields] OR ”modeler s”[All Fields] OR ”modelers”[All Fields] OR ”modeling”[All Fields] OR ”modelings”[All Fields] OR ”modelization”[All Fields] OR ”modelizations”[All Fields] OR ”modelize”[All Fields] OR ”modelized”[All Fields] OR ”modelled”[All Fields] OR ”modeller”[All Fields] OR ”modellers”[All Fields] OR ”modelling”[All Fields] OR ”modellings”[All Fields] OR ”models”[All Fields]) OR ”vitro”[All Fields]) AND (”gut”[Journal] OR ”gut”[All Fields] OR (”intestinalization”[All Fields] OR ”intestinalized”[All Fields] OR ”intestinally”[All Fields] OR ”intestinals”[All Fields] OR ”intestine s”[All Fields] OR ”intestines”[MeSH Terms] OR ”intestines”[All Fields] OR ”intestinal”[All Fields] OR ”intestine”[All Fields]) OR (”gastrointestinal”[All Fields] OR ”gastrointestinally”[All Fields] OR ”gastrointestine”[All Fields])) AND (”microbiota”[MeSH Terms] OR ”microbiota”[All Fields] OR ”microbiotas”[All Fields] OR ”microbiota s”[All Fields] OR ”microbiotae”[All Fields] OR (”microbiome s”[All Fields] OR ”microbiomic”[All Fields] OR ”microbiomics”[All Fields] OR ”microbiota”[MeSH Terms] OR ”microbiota”[All Fields] OR ”microbiome”[All Fields] OR ”microbiomes”[All Fields]) OR (”microbial”[All Fields] OR ”microbially”[All Fields] OR ”microbials”[All Fields])) AND ”humans”[MeSH Terms] AND ”in vitro gastrointestinal”[Title/Abstract]) AND (humans[Filter])	70	1899-12-31 23:49:54

A-10	((”bioreactor s”[All Fields] OR ”bioreactors”[MeSH Terms] OR ”bioreactors”[All Fields] OR ”bioreactor”[All Fields] OR (”chemostat”[All Fields] OR ”chemostatic”[All Fields] OR ”chemostats”[All Fields]) OR (”reactor”[All Fields] OR ”reactor s”[All Fields] OR ”reactors”[All Fields]) OR ”shime”[All Fields] OR (”computer simulation”[MeSH Terms] OR (”computer”[All Fields] AND ”simulation”[All Fields]) OR ”computer simulation”[All Fields] OR ”simulation”[All Fields] OR ”simul”[All Fields] OR ”simulate”[All Fields] OR ”simulated”[All Fields] OR ”simulates”[All Fields] OR ”simulating”[All Fields] OR ”simulation s”[All Fields] OR ”simulational”[All Fields] OR ”simulations”[All Fields] OR ”simulative”[All Fields] OR ”simulator”[All Fields] OR ”simulator s”[All Fields] OR ”simulators”[All Fields]) OR (”computer simulation”[MeSH Terms] OR (”computer”[All Fields] AND ”simulation”[All Fields]) OR ”computer simulation”[All Fields] OR ”simulation”[All Fields] OR ”simul”[All Fields] OR ”simulate”[All Fields] OR ”simulated”[All Fields] OR ”simulates”[All Fields] OR ”simulating”[All Fields] OR ”simulation s”[All Fields] OR ”simulational”[All Fields] OR ”simulations”[All Fields] OR ”simulative”[All Fields] OR ”simulator”[All Fields] OR ”simulator s”[All Fields] OR ”simulators”[All Fields]) OR (”dynamer”[All Fields] OR ”dynamers”[All Fields] OR ”dynamic”[All Fields] OR ”dynamical”[All Fields] OR ”dynamically”[All Fields] OR ”dynamicity”[All Fields] OR ”dynamics”[All Fields] OR ”dynamism”[All Fields] OR ”dynamisms”[All Fields]) OR (”model”[All Fields] OR ”model s”[All Fields] OR ”modeled”[All Fields] OR ”modeler”[All Fields] OR ”modeler s”[All Fields] OR ”modelers”[All Fields] OR ”modeling”[All Fields] OR ”modelings”[All Fields] OR ”modelization”[All Fields] OR ”modelizations”[All Fields] OR ”modelize”[All Fields] OR ”modelized”[All Fields] OR ”modelled”[All Fields] OR ”modeller”[All Fields] OR ”modellers”[All Fields] OR ”modelling”[All Fields] OR ”modellings”[All Fields] OR ”models”[All Fields]) OR ”vitro”[All Fields]) AND (”gut”[Journal] OR ”gut”[All Fields] OR (”intestinalization”[All Fields] OR ”intestinalized”[All Fields] OR ”intestinally”[All Fields] OR ”intestinals”[All Fields] OR ”intestine s”[All Fields] OR ”intestines”[MeSH Terms] OR ”intestines”[All Fields] OR ”intestinal”[All Fields] OR ”intestine”[All Fields]) OR (”gastrointestinal”[All Fields] OR ”gastrointestinally”[All Fields] OR ”gastrointestine”[All Fields])) AND (”microbiota”[MeSH Terms] OR ”microbiota”[All Fields] OR ”microbiotas”[All Fields] OR ”microbiota s”[All Fields] OR ”microbiotae”[All Fields] OR (”microbiome s”[All Fields] OR ”microbiomic”[All Fields] OR ”microbiomics”[All Fields] OR ”microbiota”[MeSH Terms] OR ”microbiota”[All Fields] OR ”microbiome”[All Fields] OR ”microbiomes”[All Fields]) OR (”microbial”[All Fields] OR ”microbially”[All Fields] OR ”microbials”[All Fields])) AND ”humans”[MeSH Terms] AND ”in vitro model”[Title/Abstract]) AND (humans[Filter])	160	1899-12-31 23:51:03

A-11	((”bioreactor s”[All Fields] OR ”bioreactors”[MeSH Terms] OR ”bioreactors”[All Fields] OR ”bioreactor”[All Fields] OR (”chemostat”[All Fields] OR ”chemostatic”[All Fields] OR ”chemostats”[All Fields]) OR (”reactor”[All Fields] OR ”reactor s”[All Fields] OR ”reactors”[All Fields]) OR ”shime”[All Fields] OR (”computer simulation”[MeSH Terms] OR (”computer”[All Fields] AND ”simulation”[All Fields]) OR ”computer simulation”[All Fields] OR ”simulation”[All Fields] OR ”simul”[All Fields] OR ”simulate”[All Fields] OR ”simulated”[All Fields] OR ”simulates”[All Fields] OR ”simulating”[All Fields] OR ”simulation s”[All Fields] OR ”simulational”[All Fields] OR ”simulations”[All Fields] OR ”simulative”[All Fields] OR ”simulator”[All Fields] OR ”simulator s”[All Fields] OR ”simulators”[All Fields]) OR (”computer simulation”[MeSH Terms] OR (”computer”[All Fields] AND ”simulation”[All Fields]) OR ”computer simulation”[All Fields] OR ”simulation”[All Fields] OR ”simul”[All Fields] OR ”simulate”[All Fields] OR ”simulated”[All Fields] OR ”simulates”[All Fields] OR ”simulating”[All Fields] OR ”simulation s”[All Fields] OR ”simulational”[All Fields] OR ”simulations”[All Fields] OR ”simulative”[All Fields] OR ”simulator”[All Fields] OR ”simulator s”[All Fields] OR ”simulators”[All Fields]) OR (”dynamer”[All Fields] OR ”dynamers”[All Fields] OR ”dynamic”[All Fields] OR ”dynamical”[All Fields] OR ”dynamically”[All Fields] OR ”dynamicity”[All Fields] OR ”dynamics”[All Fields] OR ”dynamism”[All Fields] OR ”dynamisms”[All Fields]) OR (”model”[All Fields] OR ”model s”[All Fields] OR ”modeled”[All Fields] OR ”modeler”[All Fields] OR ”modeler s”[All Fields] OR ”modelers”[All Fields] OR ”modeling”[All Fields] OR ”modelings”[All Fields] OR ”modelization”[All Fields] OR ”modelizations”[All Fields] OR ”modelize”[All Fields] OR ”modelized”[All Fields] OR ”modelled”[All Fields] OR ”modeller”[All Fields] OR ”modellers”[All Fields] OR ”modelling”[All Fields] OR ”modellings”[All Fields] OR ”models”[All Fields]) OR ”vitro”[All Fields]) AND (”gut”[Journal] OR ”gut”[All Fields] OR (”intestinalization”[All Fields] OR ”intestinalized”[All Fields] OR ”intestinally”[All Fields] OR ”intestinals”[All Fields] OR ”intestine s”[All Fields] OR ”intestines”[MeSH Terms] OR ”intestines”[All Fields] OR ”intestinal”[All Fields] OR ”intestine”[All Fields]) OR (”gastrointestinal”[All Fields] OR ”gastrointestinally”[All Fields] OR ”gastrointestine”[All Fields])) AND (”microbiota”[MeSH Terms] OR ”microbiota”[All Fields] OR ”microbiotas”[All Fields] OR ”microbiota s”[All Fields] OR ”microbiotae”[All Fields] OR (”microbiome s”[All Fields] OR ”microbiomic”[All Fields] OR ”microbiomics”[All Fields] OR ”microbiota”[MeSH Terms] OR ”microbiota”[All Fields] OR ”microbiome”[All Fields] OR ”microbiomes”[All Fields]) OR (”microbial”[All Fields] OR ”microbially”[All Fields] OR ”microbials”[All Fields])) AND ”humans”[MeSH Terms] AND ”shime”[Title/Abstract]) AND (humans[Filter])	114	1899-12-31 00:00:19

A-12	((”bioreactor s”[All Fields] OR ”bioreactors”[MeSH Terms] OR ”bioreactors”[All Fields] OR ”bioreactor”[All Fields] OR (”chemostat”[All Fields] OR ”chemostatic”[All Fields] OR ”chemostats”[All Fields]) OR (”reactor”[All Fields] OR ”reactor s”[All Fields] OR ”reactors”[All Fields]) OR ”shime”[All Fields] OR (”computer simulation”[MeSH Terms] OR (”computer”[All Fields] AND ”simulation”[All Fields]) OR ”computer simulation”[All Fields] OR ”simulation”[All Fields] OR ”simul”[All Fields] OR ”simulate”[All Fields] OR ”simulated”[All Fields] OR ”simulates”[All Fields] OR ”simulating”[All Fields] OR ”simulation s”[All Fields] OR ”simulational”[All Fields] OR ”simulations”[All Fields] OR ”simulative”[All Fields] OR ”simulator”[All Fields] OR ”simulator s”[All Fields] OR ”simulators”[All Fields]) OR (”computer simulation”[MeSH Terms] OR (”computer”[All Fields] AND ”simulation”[All Fields]) OR ”computer simulation”[All Fields] OR ”simulation”[All Fields] OR ”simul”[All Fields] OR ”simulate”[All Fields] OR ”simulated”[All Fields] OR ”simulates”[All Fields] OR ”simulating”[All Fields] OR ”simulation s”[All Fields] OR ”simulational”[All Fields] OR ”simulations”[All Fields] OR ”simulative”[All Fields] OR ”simulator”[All Fields] OR ”simulator s”[All Fields] OR ”simulators”[All Fields]) OR (”dynamer”[All Fields] OR ”dynamers”[All Fields] OR ”dynamic”[All Fields] OR ”dynamical”[All Fields] OR ”dynamically”[All Fields] OR ”dynamicity”[All Fields] OR ”dynamics”[All Fields] OR ”dynamism”[All Fields] OR ”dynamisms”[All Fields]) OR (”model”[All Fields] OR ”model s”[All Fields] OR ”modeled”[All Fields] OR ”modeler”[All Fields] OR ”modeler s”[All Fields] OR ”modelers”[All Fields] OR ”modeling”[All Fields] OR ”modelings”[All Fields] OR ”modelization”[All Fields] OR ”modelizations”[All Fields] OR ”modelize”[All Fields] OR ”modelized”[All Fields] OR ”modelled”[All Fields] OR ”modeller”[All Fields] OR ”modellers”[All Fields] OR ”modelling”[All Fields] OR ”modellings”[All Fields] OR ”models”[All Fields]) OR ”vitro”[All Fields]) AND (”gut”[Journal] OR ”gut”[All Fields] OR (”intestinalization”[All Fields] OR ”intestinalized”[All Fields] OR ”intestinally”[All Fields] OR ”intestinals”[All Fields] OR ”intestine s”[All Fields] OR ”intestines”[MeSH Terms] OR ”intestines”[All Fields] OR ”intestinal”[All Fields] OR ”intestine”[All Fields]) OR (”gastrointestinal”[All Fields] OR ”gastrointestinally”[All Fields] OR ”gastrointestine”[All Fields])) AND (”microbiota”[MeSH Terms] OR ”microbiota”[All Fields] OR ”microbiotas”[All Fields] OR ”microbiota s”[All Fields] OR ”microbiotae”[All Fields] OR (”microbiome s”[All Fields] OR ”microbiomic”[All Fields] OR ”microbiomics”[All Fields] OR ”microbiota”[MeSH Terms] OR ”microbiota”[All Fields] OR ”microbiome”[All Fields] OR ”microbiomes”[All Fields]) OR (”microbial”[All Fields] OR ”microbially”[All Fields] OR ”microbials”[All Fields])) AND ”humans”[MeSH Terms] AND ”simulated colon”[Title/Abstract]) AND (humans[Filter])	13	1899-12-31 23:52:10

A-13	((”bioreactor s”[All Fields] OR ”bioreactors”[MeSH Terms] OR ”bioreactors”[All Fields] OR ”bioreactor”[All Fields] OR (”chemostat”[All Fields] OR ”chemostatic”[All Fields] OR ”chemostats”[All Fields]) OR (”reactor”[All Fields] OR ”reactor s”[All Fields] OR ”reactors”[All Fields]) OR ”shime”[All Fields] OR (”computer simulation”[MeSH Terms] OR (”computer”[All Fields] AND ”simulation”[All Fields]) OR ”computer simulation”[All Fields] OR ”simulation”[All Fields] OR ”simul”[All Fields] OR ”simulate”[All Fields] OR ”simulated”[All Fields] OR ”simulates”[All Fields] OR ”simulating”[All Fields] OR ”simulation s”[All Fields] OR ”simulational”[All Fields] OR ”simulations”[All Fields] OR ”simulative”[All Fields] OR ”simulator”[All Fields] OR ”simulator s”[All Fields] OR ”simulators”[All Fields]) OR (”computer simulation”[MeSH Terms] OR (”computer”[All Fields] AND ”simulation”[All Fields]) OR ”computer simulation”[All Fields] OR ”simulation”[All Fields] OR ”simul”[All Fields] OR ”simulate”[All Fields] OR ”simulated”[All Fields] OR ”simulates”[All Fields] OR ”simulating”[All Fields] OR ”simulation s”[All Fields] OR ”simulational”[All Fields] OR ”simulations”[All Fields] OR ”simulative”[All Fields] OR ”simulator”[All Fields] OR ”simulator s”[All Fields] OR ”simulators”[All Fields]) OR (”dynamer”[All Fields] OR ”dynamers”[All Fields] OR ”dynamic”[All Fields] OR ”dynamical”[All Fields] OR ”dynamically”[All Fields] OR ”dynamicity”[All Fields] OR ”dynamics”[All Fields] OR ”dynamism”[All Fields] OR ”dynamisms”[All Fields]) OR (”model”[All Fields] OR ”model s”[All Fields] OR ”modeled”[All Fields] OR ”modeler”[All Fields] OR ”modeler s”[All Fields] OR ”modelers”[All Fields] OR ”modeling”[All Fields] OR ”modelings”[All Fields] OR ”modelization”[All Fields] OR ”modelizations”[All Fields] OR ”modelize”[All Fields] OR ”modelized”[All Fields] OR ”modelled”[All Fields] OR ”modeller”[All Fields] OR ”modellers”[All Fields] OR ”modelling”[All Fields] OR ”modellings”[All Fields] OR ”models”[All Fields]) OR ”vitro”[All Fields]) AND (”gut”[Journal] OR ”gut”[All Fields] OR (”intestinalization”[All Fields] OR ”intestinalized”[All Fields] OR ”intestinally”[All Fields] OR ”intestinals”[All Fields] OR ”intestine s”[All Fields] OR ”intestines”[MeSH Terms] OR ”intestines”[All Fields] OR ”intestinal”[All Fields] OR ”intestine”[All Fields]) OR (”gastrointestinal”[All Fields] OR ”gastrointestinally”[All Fields] OR ”gastrointestine”[All Fields])) AND (”microbiota”[MeSH Terms] OR ”microbiota”[All Fields] OR ”microbiotas”[All Fields] OR ”microbiota s”[All Fields] OR ”microbiotae”[All Fields] OR (”microbiome s”[All Fields] OR ”microbiomic”[All Fields] OR ”microbiomics”[All Fields] OR ”microbiota”[MeSH Terms] OR ”microbiota”[All Fields] OR ”microbiome”[All Fields] OR ”microbiomes”[All Fields]) OR (”microbial”[All Fields] OR ”microbially”[All Fields] OR ”microbials”[All Fields])) AND ”humans”[MeSH Terms] AND ”simulated colonic”[Title/Abstract]) AND (humans[Filter])	14	1899-12-31 23:53:18

A-14	((”bioreactor s”[All Fields] OR ”bioreactors”[MeSH Terms] OR ”bioreactors”[All Fields] OR ”bioreactor”[All Fields] OR (”chemostat”[All Fields] OR ”chemostatic”[All Fields] OR ”chemostats”[All Fields]) OR (”reactor”[All Fields] OR ”reactor s”[All Fields] OR ”reactors”[All Fields]) OR ”shime”[All Fields] OR (”computer simulation”[MeSH Terms] OR (”computer”[All Fields] AND ”simulation”[All Fields]) OR ”computer simulation”[All Fields] OR ”simulation”[All Fields] OR ”simul”[All Fields] OR ”simulate”[All Fields] OR ”simulated”[All Fields] OR ”simulates”[All Fields] OR ”simulating”[All Fields] OR ”simulation s”[All Fields] OR ”simulational”[All Fields] OR ”simulations”[All Fields] OR ”simulative”[All Fields] OR ”simulator”[All Fields] OR ”simulator s”[All Fields] OR ”simulators”[All Fields]) OR (”computer simulation”[MeSH Terms] OR (”computer”[All Fields] AND ”simulation”[All Fields]) OR ”computer simulation”[All Fields] OR ”simulation”[All Fields] OR ”simul”[All Fields] OR ”simulate”[All Fields] OR ”simulated”[All Fields] OR ”simulates”[All Fields] OR ”simulating”[All Fields] OR ”simulation s”[All Fields] OR ”simulational”[All Fields] OR ”simulations”[All Fields] OR ”simulative”[All Fields] OR ”simulator”[All Fields] OR ”simulator s”[All Fields] OR ”simulators”[All Fields]) OR (”dynamer”[All Fields] OR ”dynamers”[All Fields] OR ”dynamic”[All Fields] OR ”dynamical”[All Fields] OR ”dynamically”[All Fields] OR ”dynamicity”[All Fields] OR ”dynamics”[All Fields] OR ”dynamism”[All Fields] OR ”dynamisms”[All Fields]) OR (”model”[All Fields] OR ”model s”[All Fields] OR ”modeled”[All Fields] OR ”modeler”[All Fields] OR ”modeler s”[All Fields] OR ”modelers”[All Fields] OR ”modeling”[All Fields] OR ”modelings”[All Fields] OR ”modelization”[All Fields] OR ”modelizations”[All Fields] OR ”modelize”[All Fields] OR ”modelized”[All Fields] OR ”modelled”[All Fields] OR ”modeller”[All Fields] OR ”modellers”[All Fields] OR ”modelling”[All Fields] OR ”modellings”[All Fields] OR ”models”[All Fields]) OR ”vitro”[All Fields]) AND (”gut”[Journal] OR ”gut”[All Fields] OR (”intestinalization”[All Fields] OR ”intestinalized”[All Fields] OR ”intestinally”[All Fields] OR ”intestinals”[All Fields] OR ”intestine s”[All Fields] OR ”intestines”[MeSH Terms] OR ”intestines”[All Fields] OR ”intestinal”[All Fields] OR ”intestine”[All Fields]) OR (”gastrointestinal”[All Fields] OR ”gastrointestinally”[All Fields] OR ”gastrointestine”[All Fields])) AND (”microbiota”[MeSH Terms] OR ”microbiota”[All Fields] OR ”microbiotas”[All Fields] OR ”microbiota s”[All Fields] OR ”microbiotae”[All Fields] OR (”microbiome s”[All Fields] OR ”microbiomic”[All Fields] OR ”microbiomics”[All Fields] OR ”microbiota”[MeSH Terms] OR ”microbiota”[All Fields] OR ”microbiome”[All Fields] OR ”microbiomes”[All Fields]) OR (”microbial”[All Fields] OR ”microbially”[All Fields] OR ”microbials”[All Fields])) AND ”humans”[MeSH Terms] AND ”simulated gastrointestinal”[Title/Abstract]) AND (humans[Filter])	119	1899-12-31 23:54:18

A-15	((”bioreactor s”[All Fields] OR ”bioreactors”[MeSH Terms] OR ”bioreactors”[All Fields] OR ”bioreactor”[All Fields] OR (”chemostat”[All Fields] OR ”chemostatic”[All Fields] OR ”chemostats”[All Fields]) OR (”reactor”[All Fields] OR ”reactor s”[All Fields] OR ”reactors”[All Fields]) OR ”shime”[All Fields] OR (”computer simulation”[MeSH Terms] OR (”computer”[All Fields] AND ”simulation”[All Fields]) OR ”computer simulation”[All Fields] OR ”simulation”[All Fields] OR ”simul”[All Fields] OR ”simulate”[All Fields] OR ”simulated”[All Fields] OR ”simulates”[All Fields] OR ”simulating”[All Fields] OR ”simulation s”[All Fields] OR ”simulational”[All Fields] OR ”simulations”[All Fields] OR ”simulative”[All Fields] OR ”simulator”[All Fields] OR ”simulator s”[All Fields] OR ”simulators”[All Fields]) OR (”computer simulation”[MeSH Terms] OR (”computer”[All Fields] AND ”simulation”[All Fields]) OR ”computer simulation”[All Fields] OR ”simulation”[All Fields] OR ”simul”[All Fields] OR ”simulate”[All Fields] OR ”simulated”[All Fields] OR ”simulates”[All Fields] OR ”simulating”[All Fields] OR ”simulation s”[All Fields] OR ”simulational”[All Fields] OR ”simulations”[All Fields] OR ”simulative”[All Fields] OR ”simulator”[All Fields] OR ”simulator s”[All Fields] OR ”simulators”[All Fields]) OR (”dynamer”[All Fields] OR ”dynamers”[All Fields] OR ”dynamic”[All Fields] OR ”dynamical”[All Fields] OR ”dynamically”[All Fields] OR ”dynamicity”[All Fields] OR ”dynamics”[All Fields] OR ”dynamism”[All Fields] OR ”dynamisms”[All Fields]) OR (”model”[All Fields] OR ”model s”[All Fields] OR ”modeled”[All Fields] OR ”modeler”[All Fields] OR ”modeler s”[All Fields] OR ”modelers”[All Fields] OR ”modeling”[All Fields] OR ”modelings”[All Fields] OR ”modelization”[All Fields] OR ”modelizations”[All Fields] OR ”modelize”[All Fields] OR ”modelized”[All Fields] OR ”modelled”[All Fields] OR ”modeller”[All Fields] OR ”modellers”[All Fields] OR ”modelling”[All Fields] OR ”modellings”[All Fields] OR ”models”[All Fields]) OR ”vitro”[All Fields]) AND (”gut”[Journal] OR ”gut”[All Fields] OR (”intestinalization”[All Fields] OR ”intestinalized”[All Fields] OR ”intestinally”[All Fields] OR ”intestinals”[All Fields] OR ”intestine s”[All Fields] OR ”intestines”[MeSH Terms] OR ”intestines”[All Fields] OR ”intestinal”[All Fields] OR ”intestine”[All Fields]) OR (”gastrointestinal”[All Fields] OR ”gastrointestinally”[All Fields] OR ”gastrointestine”[All Fields])) AND (”microbiota”[MeSH Terms] OR ”microbiota”[All Fields] OR ”microbiotas”[All Fields] OR ”microbiota s”[All Fields] OR ”microbiotae”[All Fields] OR (”microbiome s”[All Fields] OR ”microbiomic”[All Fields] OR ”microbiomics”[All Fields] OR ”microbiota”[MeSH Terms] OR ”microbiota”[All Fields] OR ”microbiome”[All Fields] OR ”microbiomes”[All Fields]) OR (”microbial”[All Fields] OR ”microbially”[All Fields] OR ”microbials”[All Fields])) AND ”humans”[MeSH Terms] AND ”simulator of human”[Title/Abstract]) AND (humans[Filter])	22	1899-12-31 00:00:26

A-16	((”bioreactor s”[All Fields] OR ”bioreactors”[MeSH Terms] OR ”bioreactors”[All Fields] OR ”bioreactor”[All Fields] OR (”chemostat”[All Fields] OR ”chemostatic”[All Fields] OR ”chemostats”[All Fields]) OR (”reactor”[All Fields] OR ”reactor s”[All Fields] OR ”reactors”[All Fields]) OR ”shime”[All Fields] OR (”computer simulation”[MeSH Terms] OR (”computer”[All Fields] AND ”simulation”[All Fields]) OR ”computer simulation”[All Fields] OR ”simulation”[All Fields] OR ”simul”[All Fields] OR ”simulate”[All Fields] OR ”simulated”[All Fields] OR ”simulates”[All Fields] OR ”simulating”[All Fields] OR ”simulation s”[All Fields] OR ”simulational”[All Fields] OR ”simulations”[All Fields] OR ”simulative”[All Fields] OR ”simulator”[All Fields] OR ”simulator s”[All Fields] OR ”simulators”[All Fields]) OR (”computer simulation”[MeSH Terms] OR (”computer”[All Fields] AND ”simulation”[All Fields]) OR ”computer simulation”[All Fields] OR ”simulation”[All Fields] OR ”simul”[All Fields] OR ”simulate”[All Fields] OR ”simulated”[All Fields] OR ”simulates”[All Fields] OR ”simulating”[All Fields] OR ”simulation s”[All Fields] OR ”simulational”[All Fields] OR ”simulations”[All Fields] OR ”simulative”[All Fields] OR ”simulator”[All Fields] OR ”simulator s”[All Fields] OR ”simulators”[All Fields]) OR (”dynamer”[All Fields] OR ”dynamers”[All Fields] OR ”dynamic”[All Fields] OR ”dynamical”[All Fields] OR ”dynamically”[All Fields] OR ”dynamicity”[All Fields] OR ”dynamics”[All Fields] OR ”dynamism”[All Fields] OR ”dynamisms”[All Fields]) OR (”model”[All Fields] OR ”model s”[All Fields] OR ”modeled”[All Fields] OR ”modeler”[All Fields] OR ”modeler s”[All Fields] OR ”modelers”[All Fields] OR ”modeling”[All Fields] OR ”modelings”[All Fields] OR ”modelization”[All Fields] OR ”modelizations”[All Fields] OR ”modelize”[All Fields] OR ”modelized”[All Fields] OR ”modelled”[All Fields] OR ”modeller”[All Fields] OR ”modellers”[All Fields] OR ”modelling”[All Fields] OR ”modellings”[All Fields] OR ”models”[All Fields]) OR ”vitro”[All Fields]) AND (”gut”[Journal] OR ”gut”[All Fields] OR (”intestinalization”[All Fields] OR ”intestinalized”[All Fields] OR ”intestinally”[All Fields] OR ”intestinals”[All Fields] OR ”intestine s”[All Fields] OR ”intestines”[MeSH Terms] OR ”intestines”[All Fields] OR ”intestinal”[All Fields] OR ”intestine”[All Fields]) OR (”gastrointestinal”[All Fields] OR ”gastrointestinally”[All Fields] OR ”gastrointestine”[All Fields])) AND (”microbiota”[MeSH Terms] OR ”microbiota”[All Fields] OR ”microbiotas”[All Fields] OR ”microbiota s”[All Fields] OR ”microbiotae”[All Fields] OR (”microbiome s”[All Fields] OR ”microbiomic”[All Fields] OR ”microbiomics”[All Fields] OR ”microbiota”[MeSH Terms] OR ”microbiota”[All Fields] OR ”microbiome”[All Fields] OR ”microbiomes”[All Fields]) OR (”microbial”[All Fields] OR ”microbially”[All Fields] OR ”microbials”[All Fields])) AND ”humans”[MeSH Terms] AND ”simulator of the human”[Title/Abstract]) AND (humans[Filter])	85	1899-12-31 23:55:10

A-17	((”bioreactor s”[All Fields] OR ”bioreactors”[MeSH Terms] OR ”bioreactors”[All Fields] OR ”bioreactor”[All Fields] OR (”chemostat”[All Fields] OR ”chemostatic”[All Fields] OR ”chemostats”[All Fields]) OR (”reactor”[All Fields] OR ”reactor s”[All Fields] OR ”reactors”[All Fields]) OR ”shime”[All Fields] OR (”computer simulation”[MeSH Terms] OR (”computer”[All Fields] AND ”simulation”[All Fields]) OR ”computer simulation”[All Fields] OR ”simulation”[All Fields] OR ”simul”[All Fields] OR ”simulate”[All Fields] OR ”simulated”[All Fields] OR ”simulates”[All Fields] OR ”simulating”[All Fields] OR ”simulation s”[All Fields] OR ”simulational”[All Fields] OR ”simulations”[All Fields] OR ”simulative”[All Fields] OR ”simulator”[All Fields] OR ”simulator s”[All Fields] OR ”simulators”[All Fields]) OR (”computer simulation”[MeSH Terms] OR (”computer”[All Fields] AND ”simulation”[All Fields]) OR ”computer simulation”[All Fields] OR ”simulation”[All Fields] OR ”simul”[All Fields] OR ”simulate”[All Fields] OR ”simulated”[All Fields] OR ”simulates”[All Fields] OR ”simulating”[All Fields] OR ”simulation s”[All Fields] OR ”simulational”[All Fields] OR ”simulations”[All Fields] OR ”simulative”[All Fields] OR ”simulator”[All Fields] OR ”simulator s”[All Fields] OR ”simulators”[All Fields]) OR (”dynamer”[All Fields] OR ”dynamers”[All Fields] OR ”dynamic”[All Fields] OR ”dynamical”[All Fields] OR ”dynamically”[All Fields] OR ”dynamicity”[All Fields] OR ”dynamics”[All Fields] OR ”dynamism”[All Fields] OR ”dynamisms”[All Fields]) OR (”model”[All Fields] OR ”model s”[All Fields] OR ”modeled”[All Fields] OR ”modeler”[All Fields] OR ”modeler s”[All Fields] OR ”modelers”[All Fields] OR ”modeling”[All Fields] OR ”modelings”[All Fields] OR ”modelization”[All Fields] OR ”modelizations”[All Fields] OR ”modelize”[All Fields] OR ”modelized”[All Fields] OR ”modelled”[All Fields] OR ”modeller”[All Fields] OR ”modellers”[All Fields] OR ”modelling”[All Fields] OR ”modellings”[All Fields] OR ”models”[All Fields]) OR ”vitro”[All Fields]) AND (”gut”[Journal] OR ”gut”[All Fields] OR (”intestinalization”[All Fields] OR ”intestinalized”[All Fields] OR ”intestinally”[All Fields] OR ”intestinals”[All Fields] OR ”intestine s”[All Fields] OR ”intestines”[MeSH Terms] OR ”intestines”[All Fields] OR ”intestinal”[All Fields] OR ”intestine”[All Fields]) OR (”gastrointestinal”[All Fields] OR ”gastrointestinally”[All Fields] OR ”gastrointestine”[All Fields])) AND (”microbiota”[MeSH Terms] OR ”microbiota”[All Fields] OR ”microbiotas”[All Fields] OR ”microbiota s”[All Fields] OR ”microbiotae”[All Fields] OR (”microbiome s”[All Fields] OR ”microbiomic”[All Fields] OR ”microbiomics”[All Fields] OR ”microbiota”[MeSH Terms] OR ”microbiota”[All Fields] OR ”microbiome”[All Fields] OR ”microbiomes”[All Fields]) OR (”microbial”[All Fields] OR ”microbially”[All Fields] OR ”microbials”[All Fields])) AND ”humans”[MeSH Terms] AND ”bioreactor”[Title/Abstract]) AND (humans[Filter])	35	1899-12-31 23:33:53

A-18	((”bioreactor s”[All Fields] OR ”bioreactors”[MeSH Terms] OR ”bioreactors”[All Fields] OR ”bioreactor”[All Fields] OR (”chemostat”[All Fields] OR ”chemostatic”[All Fields] OR ”chemostats”[All Fields]) OR (”reactor”[All Fields] OR ”reactor s”[All Fields] OR ”reactors”[All Fields]) OR ”shime”[All Fields] OR (”computer simulation”[MeSH Terms] OR (”computer”[All Fields] AND ”simulation”[All Fields]) OR ”computer simulation”[All Fields] OR ”simulation”[All Fields] OR ”simul”[All Fields] OR ”simulate”[All Fields] OR ”simulated”[All Fields] OR ”simulates”[All Fields] OR ”simulating”[All Fields] OR ”simulation s”[All Fields] OR ”simulational”[All Fields] OR ”simulations”[All Fields] OR ”simulative”[All Fields] OR ”simulator”[All Fields] OR ”simulator s”[All Fields] OR ”simulators”[All Fields]) OR (”computer simulation”[MeSH Terms] OR (”computer”[All Fields] AND ”simulation”[All Fields]) OR ”computer simulation”[All Fields] OR ”simulation”[All Fields] OR ”simul”[All Fields] OR ”simulate”[All Fields] OR ”simulated”[All Fields] OR ”simulates”[All Fields] OR ”simulating”[All Fields] OR ”simulation s”[All Fields] OR ”simulational”[All Fields] OR ”simulations”[All Fields] OR ”simulative”[All Fields] OR ”simulator”[All Fields] OR ”simulator s”[All Fields] OR ”simulators”[All Fields]) OR (”dynamer”[All Fields] OR ”dynamers”[All Fields] OR ”dynamic”[All Fields] OR ”dynamical”[All Fields] OR ”dynamically”[All Fields] OR ”dynamicity”[All Fields] OR ”dynamics”[All Fields] OR ”dynamism”[All Fields] OR ”dynamisms”[All Fields]) OR (”model”[All Fields] OR ”model s”[All Fields] OR ”modeled”[All Fields] OR ”modeler”[All Fields] OR ”modeler s”[All Fields] OR ”modelers”[All Fields] OR ”modeling”[All Fields] OR ”modelings”[All Fields] OR ”modelization”[All Fields] OR ”modelizations”[All Fields] OR ”modelize”[All Fields] OR ”modelized”[All Fields] OR ”modelled”[All Fields] OR ”modeller”[All Fields] OR ”modellers”[All Fields] OR ”modelling”[All Fields] OR ”modellings”[All Fields] OR ”models”[All Fields]) OR ”vitro”[All Fields]) AND (”gut”[Journal] OR ”gut”[All Fields] OR (”intestinalization”[All Fields] OR ”intestinalized”[All Fields] OR ”intestinally”[All Fields] OR ”intestinals”[All Fields] OR ”intestine s”[All Fields] OR ”intestines”[MeSH Terms] OR ”intestines”[All Fields] OR ”intestinal”[All Fields] OR ”intestine”[All Fields]) OR (”gastrointestinal”[All Fields] OR ”gastrointestinally”[All Fields] OR ”gastrointestine”[All Fields])) AND (”microbiota”[MeSH Terms] OR ”microbiota”[All Fields] OR ”microbiotas”[All Fields] OR ”microbiota s”[All Fields] OR ”microbiotae”[All Fields] OR (”microbiome s”[All Fields] OR ”microbiomic”[All Fields] OR ”microbiomics”[All Fields] OR ”microbiota”[MeSH Terms] OR ”microbiota”[All Fields] OR ”microbiome”[All Fields] OR ”microbiomes”[All Fields]) OR (”microbial”[All Fields] OR ”microbially”[All Fields] OR ”microbials”[All Fields])) AND ”humans”[MeSH Terms] AND ”chemostat”[Title/Abstract]) AND (humans[Filter])	46	1899-12-31 23:40:24

A-19	((”bioreactor s”[All Fields] OR ”bioreactors”[MeSH Terms] OR ”bioreactors”[All Fields] OR ”bioreactor”[All Fields] OR (”chemostat”[All Fields] OR ”chemostatic”[All Fields] OR ”chemostats”[All Fields]) OR (”reactor”[All Fields] OR ”reactor s”[All Fields] OR ”reactors”[All Fields]) OR ”shime”[All Fields] OR (”computer simulation”[MeSH Terms] OR (”computer”[All Fields] AND ”simulation”[All Fields]) OR ”computer simulation”[All Fields] OR ”simulation”[All Fields] OR ”simul”[All Fields] OR ”simulate”[All Fields] OR ”simulated”[All Fields] OR ”simulates”[All Fields] OR ”simulating”[All Fields] OR ”simulation s”[All Fields] OR ”simulational”[All Fields] OR ”simulations”[All Fields] OR ”simulative”[All Fields] OR ”simulator”[All Fields] OR ”simulator s”[All Fields] OR ”simulators”[All Fields]) OR (”computer simulation”[MeSH Terms] OR (”computer”[All Fields] AND ”simulation”[All Fields]) OR ”computer simulation”[All Fields] OR ”simulation”[All Fields] OR ”simul”[All Fields] OR ”simulate”[All Fields] OR ”simulated”[All Fields] OR ”simulates”[All Fields] OR ”simulating”[All Fields] OR ”simulation s”[All Fields] OR ”simulational”[All Fields] OR ”simulations”[All Fields] OR ”simulative”[All Fields] OR ”simulator”[All Fields] OR ”simulator s”[All Fields] OR ”simulators”[All Fields]) OR (”dynamer”[All Fields] OR ”dynamers”[All Fields] OR ”dynamic”[All Fields] OR ”dynamical”[All Fields] OR ”dynamically”[All Fields] OR ”dynamicity”[All Fields] OR ”dynamics”[All Fields] OR ”dynamism”[All Fields] OR ”dynamisms”[All Fields]) OR (”model”[All Fields] OR ”model s”[All Fields] OR ”modeled”[All Fields] OR ”modeler”[All Fields] OR ”modeler s”[All Fields] OR ”modelers”[All Fields] OR ”modeling”[All Fields] OR ”modelings”[All Fields] OR ”modelization”[All Fields] OR ”modelizations”[All Fields] OR ”modelize”[All Fields] OR ”modelized”[All Fields] OR ”modelled”[All Fields] OR ”modeller”[All Fields] OR ”modellers”[All Fields] OR ”modelling”[All Fields] OR ”modellings”[All Fields] OR ”models”[All Fields]) OR ”vitro”[All Fields]) AND (”gut”[Journal] OR ”gut”[All Fields] OR (”intestinalization”[All Fields] OR ”intestinalized”[All Fields] OR ”intestinally”[All Fields] OR ”intestinals”[All Fields] OR ”intestine s”[All Fields] OR ”intestines”[MeSH Terms] OR ”intestines”[All Fields] OR ”intestinal”[All Fields] OR ”intestine”[All Fields]) OR (”gastrointestinal”[All Fields] OR ”gastrointestinally”[All Fields] OR ”gastrointestine”[All Fields])) AND (”microbiota”[MeSH Terms] OR ”microbiota”[All Fields] OR ”microbiotas”[All Fields] OR ”microbiota s”[All Fields] OR ”microbiotae”[All Fields] OR (”microbiome s”[All Fields] OR ”microbiomic”[All Fields] OR ”microbiomics”[All Fields] OR ”microbiota”[MeSH Terms] OR ”microbiota”[All Fields] OR ”microbiome”[All Fields] OR ”microbiomes”[All Fields]) OR (”microbial”[All Fields] OR ”microbially”[All Fields] OR ”microbials”[All Fields])) AND ”humans”[MeSH Terms] AND ”fermenter”[Title/Abstract]) AND (humans[Filter])	10	1899-12-31 23:42:12

A-20	((”bioreactor s”[All Fields] OR ”bioreactors”[MeSH Terms] OR ”bioreactors”[All Fields] OR ”bioreactor”[All Fields] OR (”chemostat”[All Fields] OR ”chemostatic”[All Fields] OR ”chemostats”[All Fields]) OR (”reactor”[All Fields] OR ”reactor s”[All Fields] OR ”reactors”[All Fields]) OR ”shime”[All Fields] OR (”computer simulation”[MeSH Terms] OR (”computer”[All Fields] AND ”simulation”[All Fields]) OR ”computer simulation”[All Fields] OR ”simulation”[All Fields] OR ”simul”[All Fields] OR ”simulate”[All Fields] OR ”simulated”[All Fields] OR ”simulates”[All Fields] OR ”simulating”[All Fields] OR ”simulation s”[All Fields] OR ”simulational”[All Fields] OR ”simulations”[All Fields] OR ”simulative”[All Fields] OR ”simulator”[All Fields] OR ”simulator s”[All Fields] OR ”simulators”[All Fields]) OR (”computer simulation”[MeSH Terms] OR (”computer”[All Fields] AND ”simulation”[All Fields]) OR ”computer simulation”[All Fields] OR ”simulation”[All Fields] OR ”simul”[All Fields] OR ”simulate”[All Fields] OR ”simulated”[All Fields] OR ”simulates”[All Fields] OR ”simulating”[All Fields] OR ”simulation s”[All Fields] OR ”simulational”[All Fields] OR ”simulations”[All Fields] OR ”simulative”[All Fields] OR ”simulator”[All Fields] OR ”simulator s”[All Fields] OR ”simulators”[All Fields]) OR (”dynamer”[All Fields] OR ”dynamers”[All Fields] OR ”dynamic”[All Fields] OR ”dynamical”[All Fields] OR ”dynamically”[All Fields] OR ”dynamicity”[All Fields] OR ”dynamics”[All Fields] OR ”dynamism”[All Fields] OR ”dynamisms”[All Fields]) OR (”model”[All Fields] OR ”model s”[All Fields] OR ”modeled”[All Fields] OR ”modeler”[All Fields] OR ”modeler s”[All Fields] OR ”modelers”[All Fields] OR ”modeling”[All Fields] OR ”modelings”[All Fields] OR ”modelization”[All Fields] OR ”modelizations”[All Fields] OR ”modelize”[All Fields] OR ”modelized”[All Fields] OR ”modelled”[All Fields] OR ”modeller”[All Fields] OR ”modellers”[All Fields] OR ”modelling”[All Fields] OR ”modellings”[All Fields] OR ”models”[All Fields]) OR ”vitro”[All Fields]) AND (”gut”[Journal] OR ”gut”[All Fields] OR (”intestinalization”[All Fields] OR ”intestinalized”[All Fields] OR ”intestinally”[All Fields] OR ”intestinals”[All Fields] OR ”intestine s”[All Fields] OR ”intestines”[MeSH Terms] OR ”intestines”[All Fields] OR ”intestinal”[All Fields] OR ”intestine”[All Fields]) OR (”gastrointestinal”[All Fields] OR ”gastrointestinally”[All Fields] OR ”gastrointestine”[All Fields])) AND (”microbiota”[MeSH Terms] OR ”microbiota”[All Fields] OR ”microbiotas”[All Fields] OR ”microbiota s”[All Fields] OR ”microbiotae”[All Fields] OR (”microbiome s”[All Fields] OR ”microbiomic”[All Fields] OR ”microbiomics”[All Fields] OR ”microbiota”[MeSH Terms] OR ”microbiota”[All Fields] OR ”microbiome”[All Fields] OR ”microbiomes”[All Fields]) OR (”microbial”[All Fields] OR ”microbially”[All Fields] OR ”microbials”[All Fields])) AND ”humans”[MeSH Terms] AND ”Reactor”[Title/Abstract]) AND (humans[Filter])	3	1899-12-31 00:42:12

A-21	((”bioreactor s”[All Fields] OR ”bioreactors”[MeSH Terms] OR ”bioreactors”[All Fields] OR ”bioreactor”[All Fields] OR (”chemostat”[All Fields] OR ”chemostatic”[All Fields] OR ”chemostats”[All Fields]) OR (”reactor”[All Fields] OR ”reactor s”[All Fields] OR ”reactors”[All Fields]) OR ”shime”[All Fields] OR (”computer simulation”[MeSH Terms] OR (”computer”[All Fields] AND ”simulation”[All Fields]) OR ”computer simulation”[All Fields] OR ”simulation”[All Fields] OR ”simul”[All Fields] OR ”simulate”[All Fields] OR ”simulated”[All Fields] OR ”simulates”[All Fields] OR ”simulating”[All Fields] OR ”simulation s”[All Fields] OR ”simulational”[All Fields] OR ”simulations”[All Fields] OR ”simulative”[All Fields] OR ”simulator”[All Fields] OR ”simulator s”[All Fields] OR ”simulators”[All Fields]) OR (”computer simulation”[MeSH Terms] OR (”computer”[All Fields] AND ”simulation”[All Fields]) OR ”computer simulation”[All Fields] OR ”simulation”[All Fields] OR ”simul”[All Fields] OR ”simulate”[All Fields] OR ”simulated”[All Fields] OR ”simulates”[All Fields] OR ”simulating”[All Fields] OR ”simulation s”[All Fields] OR ”simulational”[All Fields] OR ”simulations”[All Fields] OR ”simulative”[All Fields] OR ”simulator”[All Fields] OR ”simulator s”[All Fields] OR ”simulators”[All Fields]) OR (”dynamer”[All Fields] OR ”dynamers”[All Fields] OR ”dynamic”[All Fields] OR ”dynamical”[All Fields] OR ”dynamically”[All Fields] OR ”dynamicity”[All Fields] OR ”dynamics”[All Fields] OR ”dynamism”[All Fields] OR ”dynamisms”[All Fields]) OR (”model”[All Fields] OR ”model s”[All Fields] OR ”modeled”[All Fields] OR ”modeler”[All Fields] OR ”modeler s”[All Fields] OR ”modelers”[All Fields] OR ”modeling”[All Fields] OR ”modelings”[All Fields] OR ”modelization”[All Fields] OR ”modelizations”[All Fields] OR ”modelize”[All Fields] OR ”modelized”[All Fields] OR ”modelled”[All Fields] OR ”modeller”[All Fields] OR ”modellers”[All Fields] OR ”modelling”[All Fields] OR ”modellings”[All Fields] OR ”models”[All Fields]) OR ”vitro”[All Fields]) AND (”gut”[Journal] OR ”gut”[All Fields] OR (”intestinalization”[All Fields] OR ”intestinalized”[All Fields] OR ”intestinally”[All Fields] OR ”intestinals”[All Fields] OR ”intestine s”[All Fields] OR ”intestines”[MeSH Terms] OR ”intestines”[All Fields] OR ”intestinal”[All Fields] OR ”intestine”[All Fields]) OR (”gastrointestinal”[All Fields] OR ”gastrointestinally”[All Fields] OR ”gastrointestine”[All Fields])) AND (”microbiota”[MeSH Terms] OR ”microbiota”[All Fields] OR ”microbiotas”[All Fields] OR ”microbiota s”[All Fields] OR ”microbiotae”[All Fields] OR (”microbiome s”[All Fields] OR ”microbiomic”[All Fields] OR ”microbiomics”[All Fields] OR ”microbiota”[MeSH Terms] OR ”microbiota”[All Fields] OR ”microbiome”[All Fields] OR ”microbiomes”[All Fields]) OR (”microbial”[All Fields] OR ”microbially”[All Fields] OR ”microbials”[All Fields])) AND ”humans”[MeSH Terms] AND ”Artificial gut”[Title/Abstract]) AND (humans[Filter])	31	1899-12-31 00:42:12

B-1	((”bioreactor s”[All Fields] OR ”bioreactors”[MeSH Terms] OR ”bioreactors”[All Fields] OR ”bioreactor”[All Fields] OR (”chemostat”[All Fields] OR ”chemostatic”[All Fields] OR ”chemostats”[All Fields]) OR (”reactor”[All Fields] OR ”reactor s”[All Fields] OR ”reactors”[All Fields]) OR ”shime”[All Fields] OR (”computer simulation”[MeSH Terms] OR (”computer”[All Fields] AND ”simulation”[All Fields]) OR ”computer simulation”[All Fields] OR ”simulation”[All Fields] OR ”simul”[All Fields] OR ”simulate”[All Fields] OR ”simulated”[All Fields] OR ”simulates”[All Fields] OR ”simulating”[All Fields] OR ”simulation s”[All Fields] OR ”simulational”[All Fields] OR ”simulations”[All Fields] OR ”simulative”[All Fields] OR ”simulator”[All Fields] OR ”simulator s”[All Fields] OR ”simulators”[All Fields]) OR (”computer simulation”[MeSH Terms] OR (”computer”[All Fields] AND ”simulation”[All Fields]) OR ”computer simulation”[All Fields] OR ”simulation”[All Fields] OR ”simul”[All Fields] OR ”simulate”[All Fields] OR ”simulated”[All Fields] OR ”simulates”[All Fields] OR ”simulating”[All Fields] OR ”simulation s”[All Fields] OR ”simulational”[All Fields] OR ”simulations”[All Fields] OR ”simulative”[All Fields] OR ”simulator”[All Fields] OR ”simulator s”[All Fields] OR ”simulators”[All Fields]) OR (”dynamer”[All Fields] OR ”dynamers”[All Fields] OR ”dynamic”[All Fields] OR ”dynamical”[All Fields] OR ”dynamically”[All Fields] OR ”dynamicity”[All Fields] OR ”dynamics”[All Fields] OR ”dynamism”[All Fields] OR ”dynamisms”[All Fields]) OR (”model”[All Fields] OR ”model s”[All Fields] OR ”modeled”[All Fields] OR ”modeler”[All Fields] OR ”modeler s”[All Fields] OR ”modelers”[All Fields] OR ”modeling”[All Fields] OR ”modelings”[All Fields] OR ”modelization”[All Fields] OR ”modelizations”[All Fields] OR ”modelize”[All Fields] OR ”modelized”[All Fields] OR ”modelled”[All Fields] OR ”modeller”[All Fields] OR ”modellers”[All Fields] OR ”modelling”[All Fields] OR ”modellings”[All Fields] OR ”models”[All Fields]) OR ”vitro”[All Fields]) AND (”gut”[Journal] OR ”gut”[All Fields] OR (”intestinalization”[All Fields] OR ”intestinalized”[All Fields] OR ”intestinally”[All Fields] OR ”intestinals”[All Fields] OR ”intestine s”[All Fields] OR ”intestines”[MeSH Terms] OR ”intestines”[All Fields] OR ”intestinal”[All Fields] OR ”intestine”[All Fields]) OR (”gastrointestinal”[All Fields] OR ”gastrointestinally”[All Fields] OR ”gastrointestine”[All Fields])) AND (”microbiota”[MeSH Terms] OR ”microbiota”[All Fields] OR ”microbiotas”[All Fields] OR ”microbiota s”[All Fields] OR ”microbiotae”[All Fields] OR (”microbiome s”[All Fields] OR ”microbiomic”[All Fields] OR ”microbiomics”[All Fields] OR ”microbiota”[MeSH Terms] OR ”microbiota”[All Fields] OR ”microbiome”[All Fields] OR ”microbiomes”[All Fields]) OR (”microbial”[All Fields] OR ”microbially”[All Fields] OR ”microbials”[All Fields])) AND (”faecally”[All Fields] OR ”fecally”[All Fields] OR ”fecals”[All Fields] OR ”feces”[MeSH Terms] OR ”feces”[All Fields] OR ”faecal”[All Fields] OR ”fecal”[All Fields]) AND ”humans”[MeSH Terms] AND ”shime”[Title/Abstract]) AND (humans[Filter])	2	1899-12-31 23:59:00

B-2	(((”bioreactor s”[All Fields] OR ”bioreactors”[MeSH Terms] OR ”bioreactors”[All Fields] OR ”bioreactor”[All Fields] OR (”chemostat”[All Fields] OR ”chemostatic”[All Fields] OR ”chemostats”[All Fields]) OR (”reactor”[All Fields] OR ”reactor s”[All Fields] OR ”reactors”[All Fields]) OR ”shime”[All Fields] OR (”computer simulation”[MeSH Terms] OR (”computer”[All Fields] AND ”simulation”[All Fields]) OR ”computer simulation”[All Fields] OR ”simulation”[All Fields] OR ”simul”[All Fields] OR ”simulate”[All Fields] OR ”simulated”[All Fields] OR ”simulates”[All Fields] OR ”simulating”[All Fields] OR ”simulation s”[All Fields] OR ”simulational”[All Fields] OR ”simulations”[All Fields] OR ”simulative”[All Fields] OR ”simulator”[All Fields] OR ”simulator s”[All Fields] OR ”simulators”[All Fields]) OR (”computer simulation”[MeSH Terms] OR (”computer”[All Fields] AND ”simulation”[All Fields]) OR ”computer simulation”[All Fields] OR ”simulation”[All Fields] OR ”simul”[All Fields] OR ”simulate”[All Fields] OR ”simulated”[All Fields] OR ”simulates”[All Fields] OR ”simulating”[All Fields] OR ”simulation s”[All Fields] OR ”simulational”[All Fields] OR ”simulations”[All Fields] OR ”simulative”[All Fields] OR ”simulator”[All Fields] OR ”simulator s”[All Fields] OR ”simulators”[All Fields]) OR (”dynamer”[All Fields] OR ”dynamers”[All Fields] OR ”dynamic”[All Fields] OR ”dynamical”[All Fields] OR ”dynamically”[All Fields] OR ”dynamicity”[All Fields] OR ”dynamics”[All Fields] OR ”dynamism”[All Fields] OR ”dynamisms”[All Fields]) OR (”model”[All Fields] OR ”model s”[All Fields] OR ”modeled”[All Fields] OR ”modeler”[All Fields] OR ”modeler s”[All Fields] OR ”modelers”[All Fields] OR ”modeling”[All Fields] OR ”modelings”[All Fields] OR ”modelization”[All Fields] OR ”modelizations”[All Fields] OR ”modelize”[All Fields] OR ”modelized”[All Fields] OR ”modelled”[All Fields] OR ”modeller”[All Fields] OR ”modellers”[All Fields] OR ”modelling”[All Fields] OR ”modellings”[All Fields] OR ”models”[All Fields]) OR ”vitro”[All Fields]) AND (”gut”[Journal] OR ”gut”[All Fields] OR (”intestinalization”[All Fields] OR ”intestinalized”[All Fields] OR ”intestinally”[All Fields] OR ”intestinals”[All Fields] OR ”intestine s”[All Fields] OR ”intestines”[MeSH Terms] OR ”intestines”[All Fields] OR ”intestinal”[All Fields] OR ”intestine”[All Fields]) OR (”gastrointestinal”[All Fields] OR ”gastrointestinally”[All Fields] OR ”gastrointestine”[All Fields])) AND (”microbiota”[MeSH Terms] OR ”microbiota”[All Fields] OR ”microbiotas”[All Fields] OR ”microbiota s”[All Fields] OR ”microbiotae”[All Fields] OR (”microbiome s”[All Fields] OR ”microbiomic”[All Fields] OR ”microbiomics”[All Fields] OR ”microbiota”[MeSH Terms] OR ”microbiota”[All Fields] OR ”microbiome”[All Fields] OR ”microbiomes”[All Fields]) OR (”microbial”[All Fields] OR ”microbially”[All Fields] OR ”microbials”[All Fields]))) AND (”faecally”[All Fields] OR ”fecally”[All Fields] OR ”fecals”[All Fields] OR ”feces”[MeSH Terms] OR ”feces”[All Fields] OR ”faecal”[All Fields] OR ”fecal”[All Fields]) AND ”humans”[MeSH Terms] AND ”bioreactor”[Title/Abstract]) AND (humans[Filter])	15	1899-12-31 00:30:30

B-3	(((”bioreactor s”[All Fields] OR ”bioreactors”[MeSH Terms] OR ”bioreactors”[All Fields] OR ”bioreactor”[All Fields] OR (”chemostat”[All Fields] OR ”chemostatic”[All Fields] OR ”chemostats”[All Fields]) OR (”reactor”[All Fields] OR ”reactor s”[All Fields] OR ”reactors”[All Fields]) OR ”shime”[All Fields] OR (”computer simulation”[MeSH Terms] OR (”computer”[All Fields] AND ”simulation”[All Fields]) OR ”computer simulation”[All Fields] OR ”simulation”[All Fields] OR ”simul”[All Fields] OR ”simulate”[All Fields] OR ”simulated”[All Fields] OR ”simulates”[All Fields] OR ”simulating”[All Fields] OR ”simulation s”[All Fields] OR ”simulational”[All Fields] OR ”simulations”[All Fields] OR ”simulative”[All Fields] OR ”simulator”[All Fields] OR ”simulator s”[All Fields] OR ”simulators”[All Fields]) OR (”computer simulation”[MeSH Terms] OR (”computer”[All Fields] AND ”simulation”[All Fields]) OR ”computer simulation”[All Fields] OR ”simulation”[All Fields] OR ”simul”[All Fields] OR ”simulate”[All Fields] OR ”simulated”[All Fields] OR ”simulates”[All Fields] OR ”simulating”[All Fields] OR ”simulation s”[All Fields] OR ”simulational”[All Fields] OR ”simulations”[All Fields] OR ”simulative”[All Fields] OR ”simulator”[All Fields] OR ”simulator s”[All Fields] OR ”simulators”[All Fields]) OR (”dynamer”[All Fields] OR ”dynamers”[All Fields] OR ”dynamic”[All Fields] OR ”dynamical”[All Fields] OR ”dynamically”[All Fields] OR ”dynamicity”[All Fields] OR ”dynamics”[All Fields] OR ”dynamism”[All Fields] OR ”dynamisms”[All Fields]) OR (”model”[All Fields] OR ”model s”[All Fields] OR ”modeled”[All Fields] OR ”modeler”[All Fields] OR ”modeler s”[All Fields] OR ”modelers”[All Fields] OR ”modeling”[All Fields] OR ”modelings”[All Fields] OR ”modelization”[All Fields] OR ”modelizations”[All Fields] OR ”modelize”[All Fields] OR ”modelized”[All Fields] OR ”modelled”[All Fields] OR ”modeller”[All Fields] OR ”modellers”[All Fields] OR ”modelling”[All Fields] OR ”modellings”[All Fields] OR ”models”[All Fields]) OR ”vitro”[All Fields]) AND (”gut”[Journal] OR ”gut”[All Fields] OR (”intestinalization”[All Fields] OR ”intestinalized”[All Fields] OR ”intestinally”[All Fields] OR ”intestinals”[All Fields] OR ”intestine s”[All Fields] OR ”intestines”[MeSH Terms] OR ”intestines”[All Fields] OR ”intestinal”[All Fields] OR ”intestine”[All Fields]) OR (”gastrointestinal”[All Fields] OR ”gastrointestinally”[All Fields] OR ”gastrointestine”[All Fields])) AND (”microbiota”[MeSH Terms] OR ”microbiota”[All Fields] OR ”microbiotas”[All Fields] OR ”microbiota s”[All Fields] OR ”microbiotae”[All Fields] OR (”microbiome s”[All Fields] OR ”microbiomic”[All Fields] OR ”microbiomics”[All Fields] OR ”microbiota”[MeSH Terms] OR ”microbiota”[All Fields] OR ”microbiome”[All Fields] OR ”microbiomes”[All Fields]) OR (”microbial”[All Fields] OR ”microbially”[All Fields] OR ”microbials”[All Fields]))) AND (”faecally”[All Fields] OR ”fecally”[All Fields] OR ”fecals”[All Fields] OR ”feces”[MeSH Terms] OR ”feces”[All Fields] OR ”faecal”[All Fields] OR ”fecal”[All Fields]) AND ”humans”[MeSH Terms] AND ”chemostat”[Title/Abstract]) AND (humans[Filter])	29	1899-12-31 00:28:52

B-4	(((”bioreactor s”[All Fields] OR ”bioreactors”[MeSH Terms] OR ”bioreactors”[All Fields] OR ”bioreactor”[All Fields] OR (”chemostat”[All Fields] OR ”chemostatic”[All Fields] OR ”chemostats”[All Fields]) OR (”reactor”[All Fields] OR ”reactor s”[All Fields] OR ”reactors”[All Fields]) OR ”shime”[All Fields] OR (”computer simulation”[MeSH Terms] OR (”computer”[All Fields] AND ”simulation”[All Fields]) OR ”computer simulation”[All Fields] OR ”simulation”[All Fields] OR ”simul”[All Fields] OR ”simulate”[All Fields] OR ”simulated”[All Fields] OR ”simulates”[All Fields] OR ”simulating”[All Fields] OR ”simulation s”[All Fields] OR ”simulational”[All Fields] OR ”simulations”[All Fields] OR ”simulative”[All Fields] OR ”simulator”[All Fields] OR ”simulator s”[All Fields] OR ”simulators”[All Fields]) OR (”computer simulation”[MeSH Terms] OR (”computer”[All Fields] AND ”simulation”[All Fields]) OR ”computer simulation”[All Fields] OR ”simulation”[All Fields] OR ”simul”[All Fields] OR ”simulate”[All Fields] OR ”simulated”[All Fields] OR ”simulates”[All Fields] OR ”simulating”[All Fields] OR ”simulation s”[All Fields] OR ”simulational”[All Fields] OR ”simulations”[All Fields] OR ”simulative”[All Fields] OR ”simulator”[All Fields] OR ”simulator s”[All Fields] OR ”simulators”[All Fields]) OR (”dynamer”[All Fields] OR ”dynamers”[All Fields] OR ”dynamic”[All Fields] OR ”dynamical”[All Fields] OR ”dynamically”[All Fields] OR ”dynamicity”[All Fields] OR ”dynamics”[All Fields] OR ”dynamism”[All Fields] OR ”dynamisms”[All Fields]) OR (”model”[All Fields] OR ”model s”[All Fields] OR ”modeled”[All Fields] OR ”modeler”[All Fields] OR ”modeler s”[All Fields] OR ”modelers”[All Fields] OR ”modeling”[All Fields] OR ”modelings”[All Fields] OR ”modelization”[All Fields] OR ”modelizations”[All Fields] OR ”modelize”[All Fields] OR ”modelized”[All Fields] OR ”modelled”[All Fields] OR ”modeller”[All Fields] OR ”modellers”[All Fields] OR ”modelling”[All Fields] OR ”modellings”[All Fields] OR ”models”[All Fields]) OR ”vitro”[All Fields]) AND (”gut”[Journal] OR ”gut”[All Fields] OR (”intestinalization”[All Fields] OR ”intestinalized”[All Fields] OR ”intestinally”[All Fields] OR ”intestinals”[All Fields] OR ”intestine s”[All Fields] OR ”intestines”[MeSH Terms] OR ”intestines”[All Fields] OR ”intestinal”[All Fields] OR ”intestine”[All Fields]) OR (”gastrointestinal”[All Fields] OR ”gastrointestinally”[All Fields] OR ”gastrointestine”[All Fields])) AND (”microbiota”[MeSH Terms] OR ”microbiota”[All Fields] OR ”microbiotas”[All Fields] OR ”microbiota s”[All Fields] OR ”microbiotae”[All Fields] OR (”microbiome s”[All Fields] OR ”microbiomic”[All Fields] OR ”microbiomics”[All Fields] OR ”microbiota”[MeSH Terms] OR ”microbiota”[All Fields] OR ”microbiome”[All Fields] OR ”microbiomes”[All Fields]) OR (”microbial”[All Fields] OR ”microbially”[All Fields] OR ”microbials”[All Fields]))) AND (”faecally”[All Fields] OR ”fecally”[All Fields] OR ”fecals”[All Fields] OR ”feces”[MeSH Terms] OR ”feces”[All Fields] OR ”faecal”[All Fields] OR ”fecal”[All Fields]) AND ”humans”[MeSH Terms] AND ”continuous culture”[Title/Abstract]) AND (humans[Filter])	22	1899-12-31 00:27:51

B-5	(((”bioreactor s”[All Fields] OR ”bioreactors”[MeSH Terms] OR ”bioreactors”[All Fields] OR ”bioreactor”[All Fields] OR (”chemostat”[All Fields] OR ”chemostatic”[All Fields] OR ”chemostats”[All Fields]) OR (”reactor”[All Fields] OR ”reactor s”[All Fields] OR ”reactors”[All Fields]) OR ”shime”[All Fields] OR (”computer simulation”[MeSH Terms] OR (”computer”[All Fields] AND ”simulation”[All Fields]) OR ”computer simulation”[All Fields] OR ”simulation”[All Fields] OR ”simul”[All Fields] OR ”simulate”[All Fields] OR ”simulated”[All Fields] OR ”simulates”[All Fields] OR ”simulating”[All Fields] OR ”simulation s”[All Fields] OR ”simulational”[All Fields] OR ”simulations”[All Fields] OR ”simulative”[All Fields] OR ”simulator”[All Fields] OR ”simulator s”[All Fields] OR ”simulators”[All Fields]) OR (”computer simulation”[MeSH Terms] OR (”computer”[All Fields] AND ”simulation”[All Fields]) OR ”computer simulation”[All Fields] OR ”simulation”[All Fields] OR ”simul”[All Fields] OR ”simulate”[All Fields] OR ”simulated”[All Fields] OR ”simulates”[All Fields] OR ”simulating”[All Fields] OR ”simulation s”[All Fields] OR ”simulational”[All Fields] OR ”simulations”[All Fields] OR ”simulative”[All Fields] OR ”simulator”[All Fields] OR ”simulator s”[All Fields] OR ”simulators”[All Fields]) OR (”dynamer”[All Fields] OR ”dynamers”[All Fields] OR ”dynamic”[All Fields] OR ”dynamical”[All Fields] OR ”dynamically”[All Fields] OR ”dynamicity”[All Fields] OR ”dynamics”[All Fields] OR ”dynamism”[All Fields] OR ”dynamisms”[All Fields]) OR (”model”[All Fields] OR ”model s”[All Fields] OR ”modeled”[All Fields] OR ”modeler”[All Fields] OR ”modeler s”[All Fields] OR ”modelers”[All Fields] OR ”modeling”[All Fields] OR ”modelings”[All Fields] OR ”modelization”[All Fields] OR ”modelizations”[All Fields] OR ”modelize”[All Fields] OR ”modelized”[All Fields] OR ”modelled”[All Fields] OR ”modeller”[All Fields] OR ”modellers”[All Fields] OR ”modelling”[All Fields] OR ”modellings”[All Fields] OR ”models”[All Fields]) OR ”vitro”[All Fields]) AND (”gut”[Journal] OR ”gut”[All Fields] OR (”intestinalization”[All Fields] OR ”intestinalized”[All Fields] OR ”intestinally”[All Fields] OR ”intestinals”[All Fields] OR ”intestine s”[All Fields] OR ”intestines”[MeSH Terms] OR ”intestines”[All Fields] OR ”intestinal”[All Fields] OR ”intestine”[All Fields]) OR (”gastrointestinal”[All Fields] OR ”gastrointestinally”[All Fields] OR ”gastrointestine”[All Fields])) AND (”microbiota”[MeSH Terms] OR ”microbiota”[All Fields] OR ”microbiotas”[All Fields] OR ”microbiota s”[All Fields] OR ”microbiotae”[All Fields] OR (”microbiome s”[All Fields] OR ”microbiomic”[All Fields] OR ”microbiomics”[All Fields] OR ”microbiota”[MeSH Terms] OR ”microbiota”[All Fields] OR ”microbiome”[All Fields] OR ”microbiomes”[All Fields]) OR (”microbial”[All Fields] OR ”microbially”[All Fields] OR ”microbials”[All Fields]))) AND (”faecally”[All Fields] OR ”fecally”[All Fields] OR ”fecals”[All Fields] OR ”feces”[MeSH Terms] OR ”feces”[All Fields] OR ”faecal”[All Fields] OR ”fecal”[All Fields]) AND ”humans”[MeSH Terms] AND ”continuous fermentation”[Title/Abstract]) AND (humans[Filter])	5	1899-12-31 00:26:56

B-6	(((”bioreactor s”[All Fields] OR ”bioreactors”[MeSH Terms] OR ”bioreactors”[All Fields] OR ”bioreactor”[All Fields] OR (”chemostat”[All Fields] OR ”chemostatic”[All Fields] OR ”chemostats”[All Fields]) OR (”reactor”[All Fields] OR ”reactor s”[All Fields] OR ”reactors”[All Fields]) OR ”shime”[All Fields] OR (”computer simulation”[MeSH Terms] OR (”computer”[All Fields] AND ”simulation”[All Fields]) OR ”computer simulation”[All Fields] OR ”simulation”[All Fields] OR ”simul”[All Fields] OR ”simulate”[All Fields] OR ”simulated”[All Fields] OR ”simulates”[All Fields] OR ”simulating”[All Fields] OR ”simulation s”[All Fields] OR ”simulational”[All Fields] OR ”simulations”[All Fields] OR ”simulative”[All Fields] OR ”simulator”[All Fields] OR ”simulator s”[All Fields] OR ”simulators”[All Fields]) OR (”computer simulation”[MeSH Terms] OR (”computer”[All Fields] AND ”simulation”[All Fields]) OR ”computer simulation”[All Fields] OR ”simulation”[All Fields] OR ”simul”[All Fields] OR ”simulate”[All Fields] OR ”simulated”[All Fields] OR ”simulates”[All Fields] OR ”simulating”[All Fields] OR ”simulation s”[All Fields] OR ”simulational”[All Fields] OR ”simulations”[All Fields] OR ”simulative”[All Fields] OR ”simulator”[All Fields] OR ”simulator s”[All Fields] OR ”simulators”[All Fields]) OR (”dynamer”[All Fields] OR ”dynamers”[All Fields] OR ”dynamic”[All Fields] OR ”dynamical”[All Fields] OR ”dynamically”[All Fields] OR ”dynamicity”[All Fields] OR ”dynamics”[All Fields] OR ”dynamism”[All Fields] OR ”dynamisms”[All Fields]) OR (”model”[All Fields] OR ”model s”[All Fields] OR ”modeled”[All Fields] OR ”modeler”[All Fields] OR ”modeler s”[All Fields] OR ”modelers”[All Fields] OR ”modeling”[All Fields] OR ”modelings”[All Fields] OR ”modelization”[All Fields] OR ”modelizations”[All Fields] OR ”modelize”[All Fields] OR ”modelized”[All Fields] OR ”modelled”[All Fields] OR ”modeller”[All Fields] OR ”modellers”[All Fields] OR ”modelling”[All Fields] OR ”modellings”[All Fields] OR ”models”[All Fields]) OR ”vitro”[All Fields]) AND (”gut”[Journal] OR ”gut”[All Fields] OR (”intestinalization”[All Fields] OR ”intestinalized”[All Fields] OR ”intestinally”[All Fields] OR ”intestinals”[All Fields] OR ”intestine s”[All Fields] OR ”intestines”[MeSH Terms] OR ”intestines”[All Fields] OR ”intestinal”[All Fields] OR ”intestine”[All Fields]) OR (”gastrointestinal”[All Fields] OR ”gastrointestinally”[All Fields] OR ”gastrointestine”[All Fields])) AND (”microbiota”[MeSH Terms] OR ”microbiota”[All Fields] OR ”microbiotas”[All Fields] OR ”microbiota s”[All Fields] OR ”microbiotae”[All Fields] OR (”microbiome s”[All Fields] OR ”microbiomic”[All Fields] OR ”microbiomics”[All Fields] OR ”microbiota”[MeSH Terms] OR ”microbiota”[All Fields] OR ”microbiome”[All Fields] OR ”microbiomes”[All Fields]) OR (”microbial”[All Fields] OR ”microbially”[All Fields] OR ”microbials”[All Fields]))) AND (”faecally”[All Fields] OR ”fecally”[All Fields] OR ”fecals”[All Fields] OR ”feces”[MeSH Terms] OR ”feces”[All Fields] OR ”faecal”[All Fields] OR ”fecal”[All Fields]) AND ”humans”[MeSH Terms] AND ”fermenter”[Title/Abstract]) AND (humans[Filter])	7	1899-12-31 00:25:40

B-7	(((”bioreactor s”[All Fields] OR ”bioreactors”[MeSH Terms] OR ”bioreactors”[All Fields] OR ”bioreactor”[All Fields] OR (”chemostat”[All Fields] OR ”chemostatic”[All Fields] OR ”chemostats”[All Fields]) OR (”reactor”[All Fields] OR ”reactor s”[All Fields] OR ”reactors”[All Fields]) OR ”shime”[All Fields] OR (”computer simulation”[MeSH Terms] OR (”computer”[All Fields] AND ”simulation”[All Fields]) OR ”computer simulation”[All Fields] OR ”simulation”[All Fields] OR ”simul”[All Fields] OR ”simulate”[All Fields] OR ”simulated”[All Fields] OR ”simulates”[All Fields] OR ”simulating”[All Fields] OR ”simulation s”[All Fields] OR ”simulational”[All Fields] OR ”simulations”[All Fields] OR ”simulative”[All Fields] OR ”simulator”[All Fields] OR ”simulator s”[All Fields] OR ”simulators”[All Fields]) OR (”computer simulation”[MeSH Terms] OR (”computer”[All Fields] AND ”simulation”[All Fields]) OR ”computer simulation”[All Fields] OR ”simulation”[All Fields] OR ”simul”[All Fields] OR ”simulate”[All Fields] OR ”simulated”[All Fields] OR ”simulates”[All Fields] OR ”simulating”[All Fields] OR ”simulation s”[All Fields] OR ”simulational”[All Fields] OR ”simulations”[All Fields] OR ”simulative”[All Fields] OR ”simulator”[All Fields] OR ”simulator s”[All Fields] OR ”simulators”[All Fields]) OR (”dynamer”[All Fields] OR ”dynamers”[All Fields] OR ”dynamic”[All Fields] OR ”dynamical”[All Fields] OR ”dynamically”[All Fields] OR ”dynamicity”[All Fields] OR ”dynamics”[All Fields] OR ”dynamism”[All Fields] OR ”dynamisms”[All Fields]) OR (”model”[All Fields] OR ”model s”[All Fields] OR ”modeled”[All Fields] OR ”modeler”[All Fields] OR ”modeler s”[All Fields] OR ”modelers”[All Fields] OR ”modeling”[All Fields] OR ”modelings”[All Fields] OR ”modelization”[All Fields] OR ”modelizations”[All Fields] OR ”modelize”[All Fields] OR ”modelized”[All Fields] OR ”modelled”[All Fields] OR ”modeller”[All Fields] OR ”modellers”[All Fields] OR ”modelling”[All Fields] OR ”modellings”[All Fields] OR ”models”[All Fields]) OR ”vitro”[All Fields]) AND (”gut”[Journal] OR ”gut”[All Fields] OR (”intestinalization”[All Fields] OR ”intestinalized”[All Fields] OR ”intestinally”[All Fields] OR ”intestinals”[All Fields] OR ”intestine s”[All Fields] OR ”intestines”[MeSH Terms] OR ”intestines”[All Fields] OR ”intestinal”[All Fields] OR ”intestine”[All Fields]) OR (”gastrointestinal”[All Fields] OR ”gastrointestinally”[All Fields] OR ”gastrointestine”[All Fields])) AND (”microbiota”[MeSH Terms] OR ”microbiota”[All Fields] OR ”microbiotas”[All Fields] OR ”microbiota s”[All Fields] OR ”microbiotae”[All Fields] OR (”microbiome s”[All Fields] OR ”microbiomic”[All Fields] OR ”microbiomics”[All Fields] OR ”microbiota”[MeSH Terms] OR ”microbiota”[All Fields] OR ”microbiome”[All Fields] OR ”microbiomes”[All Fields]) OR (”microbial”[All Fields] OR ”microbially”[All Fields] OR ”microbials”[All Fields]))) AND (”faecally”[All Fields] OR ”fecally”[All Fields] OR ”fecals”[All Fields] OR ”feces”[MeSH Terms] OR ”feces”[All Fields] OR ”faecal”[All Fields] OR ”fecal”[All Fields]) AND ”humans”[MeSH Terms] AND ”gastrointestinal model”[Title/Abstract]) AND (humans[Filter])	5	1899-12-31 00:23:54

B-8	(((”bioreactor s”[All Fields] OR ”bioreactors”[MeSH Terms] OR ”bioreactors”[All Fields] OR ”bioreactor”[All Fields] OR (”chemostat”[All Fields] OR ”chemostatic”[All Fields] OR ”chemostats”[All Fields]) OR (”reactor”[All Fields] OR ”reactor s”[All Fields] OR ”reactors”[All Fields]) OR ”shime”[All Fields] OR (”computer simulation”[MeSH Terms] OR (”computer”[All Fields] AND ”simulation”[All Fields]) OR ”computer simulation”[All Fields] OR ”simulation”[All Fields] OR ”simul”[All Fields] OR ”simulate”[All Fields] OR ”simulated”[All Fields] OR ”simulates”[All Fields] OR ”simulating”[All Fields] OR ”simulation s”[All Fields] OR ”simulational”[All Fields] OR ”simulations”[All Fields] OR ”simulative”[All Fields] OR ”simulator”[All Fields] OR ”simulator s”[All Fields] OR ”simulators”[All Fields]) OR (”computer simulation”[MeSH Terms] OR (”computer”[All Fields] AND ”simulation”[All Fields]) OR ”computer simulation”[All Fields] OR ”simulation”[All Fields] OR ”simul”[All Fields] OR ”simulate”[All Fields] OR ”simulated”[All Fields] OR ”simulates”[All Fields] OR ”simulating”[All Fields] OR ”simulation s”[All Fields] OR ”simulational”[All Fields] OR ”simulations”[All Fields] OR ”simulative”[All Fields] OR ”simulator”[All Fields] OR ”simulator s”[All Fields] OR ”simulators”[All Fields]) OR (”dynamer”[All Fields] OR ”dynamers”[All Fields] OR ”dynamic”[All Fields] OR ”dynamical”[All Fields] OR ”dynamically”[All Fields] OR ”dynamicity”[All Fields] OR ”dynamics”[All Fields] OR ”dynamism”[All Fields] OR ”dynamisms”[All Fields]) OR (”model”[All Fields] OR ”model s”[All Fields] OR ”modeled”[All Fields] OR ”modeler”[All Fields] OR ”modeler s”[All Fields] OR ”modelers”[All Fields] OR ”modeling”[All Fields] OR ”modelings”[All Fields] OR ”modelization”[All Fields] OR ”modelizations”[All Fields] OR ”modelize”[All Fields] OR ”modelized”[All Fields] OR ”modelled”[All Fields] OR ”modeller”[All Fields] OR ”modellers”[All Fields] OR ”modelling”[All Fields] OR ”modellings”[All Fields] OR ”models”[All Fields]) OR ”vitro”[All Fields]) AND (”gut”[Journal] OR ”gut”[All Fields] OR (”intestinalization”[All Fields] OR ”intestinalized”[All Fields] OR ”intestinally”[All Fields] OR ”intestinals”[All Fields] OR ”intestine s”[All Fields] OR ”intestines”[MeSH Terms] OR ”intestines”[All Fields] OR ”intestinal”[All Fields] OR ”intestine”[All Fields]) OR (”gastrointestinal”[All Fields] OR ”gastrointestinally”[All Fields] OR ”gastrointestine”[All Fields])) AND (”microbiota”[MeSH Terms] OR ”microbiota”[All Fields] OR ”microbiotas”[All Fields] OR ”microbiota s”[All Fields] OR ”microbiotae”[All Fields] OR (”microbiome s”[All Fields] OR ”microbiomic”[All Fields] OR ”microbiomics”[All Fields] OR ”microbiota”[MeSH Terms] OR ”microbiota”[All Fields] OR ”microbiome”[All Fields] OR ”microbiomes”[All Fields]) OR (”microbial”[All Fields] OR ”microbially”[All Fields] OR ”microbials”[All Fields]))) AND (”faecally”[All Fields] OR ”fecally”[All Fields] OR ”fecals”[All Fields] OR ”feces”[MeSH Terms] OR ”feces”[All Fields] OR ”faecal”[All Fields] OR ”fecal”[All Fields]) AND ”humans”[MeSH Terms] AND ”git model”[Title/Abstract]) AND (humans[Filter])	0	1899-12-31 00:23:29

B-9	(((”bioreactor s”[All Fields] OR ”bioreactors”[MeSH Terms] OR ”bioreactors”[All Fields] OR ”bioreactor”[All Fields] OR (”chemostat”[All Fields] OR ”chemostatic”[All Fields] OR ”chemostats”[All Fields]) OR (”reactor”[All Fields] OR ”reactor s”[All Fields] OR ”reactors”[All Fields]) OR ”shime”[All Fields] OR (”computer simulation”[MeSH Terms] OR (”computer”[All Fields] AND ”simulation”[All Fields]) OR ”computer simulation”[All Fields] OR ”simulation”[All Fields] OR ”simul”[All Fields] OR ”simulate”[All Fields] OR ”simulated”[All Fields] OR ”simulates”[All Fields] OR ”simulating”[All Fields] OR ”simulation s”[All Fields] OR ”simulational”[All Fields] OR ”simulations”[All Fields] OR ”simulative”[All Fields] OR ”simulator”[All Fields] OR ”simulator s”[All Fields] OR ”simulators”[All Fields]) OR (”computer simulation”[MeSH Terms] OR (”computer”[All Fields] AND ”simulation”[All Fields]) OR ”computer simulation”[All Fields] OR ”simulation”[All Fields] OR ”simul”[All Fields] OR ”simulate”[All Fields] OR ”simulated”[All Fields] OR ”simulates”[All Fields] OR ”simulating”[All Fields] OR ”simulation s”[All Fields] OR ”simulational”[All Fields] OR ”simulations”[All Fields] OR ”simulative”[All Fields] OR ”simulator”[All Fields] OR ”simulator s”[All Fields] OR ”simulators”[All Fields]) OR (”dynamer”[All Fields] OR ”dynamers”[All Fields] OR ”dynamic”[All Fields] OR ”dynamical”[All Fields] OR ”dynamically”[All Fields] OR ”dynamicity”[All Fields] OR ”dynamics”[All Fields] OR ”dynamism”[All Fields] OR ”dynamisms”[All Fields]) OR (”model”[All Fields] OR ”model s”[All Fields] OR ”modeled”[All Fields] OR ”modeler”[All Fields] OR ”modeler s”[All Fields] OR ”modelers”[All Fields] OR ”modeling”[All Fields] OR ”modelings”[All Fields] OR ”modelization”[All Fields] OR ”modelizations”[All Fields] OR ”modelize”[All Fields] OR ”modelized”[All Fields] OR ”modelled”[All Fields] OR ”modeller”[All Fields] OR ”modellers”[All Fields] OR ”modelling”[All Fields] OR ”modellings”[All Fields] OR ”models”[All Fields]) OR ”vitro”[All Fields]) AND (”gut”[Journal] OR ”gut”[All Fields] OR (”intestinalization”[All Fields] OR ”intestinalized”[All Fields] OR ”intestinally”[All Fields] OR ”intestinals”[All Fields] OR ”intestine s”[All Fields] OR ”intestines”[MeSH Terms] OR ”intestines”[All Fields] OR ”intestinal”[All Fields] OR ”intestine”[All Fields]) OR (”gastrointestinal”[All Fields] OR ”gastrointestinally”[All Fields] OR ”gastrointestine”[All Fields])) AND (”microbiota”[MeSH Terms] OR ”microbiota”[All Fields] OR ”microbiotas”[All Fields] OR ”microbiota s”[All Fields] OR ”microbiotae”[All Fields] OR (”microbiome s”[All Fields] OR ”microbiomic”[All Fields] OR ”microbiomics”[All Fields] OR ”microbiota”[MeSH Terms] OR ”microbiota”[All Fields] OR ”microbiome”[All Fields] OR ”microbiomes”[All Fields]) OR (”microbial”[All Fields] OR ”microbially”[All Fields] OR ”microbials”[All Fields]))) AND (”faecally”[All Fields] OR ”fecally”[All Fields] OR ”fecals”[All Fields] OR ”feces”[MeSH Terms] OR ”feces”[All Fields] OR ”faecal”[All Fields] OR ”fecal”[All Fields]) AND ”humans”[MeSH Terms] AND ”gut model”[Title/Abstract]) AND (humans[Filter])	37	1899-12-31 00:22:46

B-10	(((”bioreactor s”[All Fields] OR ”bioreactors”[MeSH Terms] OR ”bioreactors”[All Fields] OR ”bioreactor”[All Fields] OR (”chemostat”[All Fields] OR ”chemostatic”[All Fields] OR ”chemostats”[All Fields]) OR (”reactor”[All Fields] OR ”reactor s”[All Fields] OR ”reactors”[All Fields]) OR ”shime”[All Fields] OR (”computer simulation”[MeSH Terms] OR (”computer”[All Fields] AND ”simulation”[All Fields]) OR ”computer simulation”[All Fields] OR ”simulation”[All Fields] OR ”simul”[All Fields] OR ”simulate”[All Fields] OR ”simulated”[All Fields] OR ”simulates”[All Fields] OR ”simulating”[All Fields] OR ”simulation s”[All Fields] OR ”simulational”[All Fields] OR ”simulations”[All Fields] OR ”simulative”[All Fields] OR ”simulator”[All Fields] OR ”simulator s”[All Fields] OR ”simulators”[All Fields]) OR (”computer simulation”[MeSH Terms] OR (”computer”[All Fields] AND ”simulation”[All Fields]) OR ”computer simulation”[All Fields] OR ”simulation”[All Fields] OR ”simul”[All Fields] OR ”simulate”[All Fields] OR ”simulated”[All Fields] OR ”simulates”[All Fields] OR ”simulating”[All Fields] OR ”simulation s”[All Fields] OR ”simulational”[All Fields] OR ”simulations”[All Fields] OR ”simulative”[All Fields] OR ”simulator”[All Fields] OR ”simulator s”[All Fields] OR ”simulators”[All Fields]) OR (”dynamer”[All Fields] OR ”dynamers”[All Fields] OR ”dynamic”[All Fields] OR ”dynamical”[All Fields] OR ”dynamically”[All Fields] OR ”dynamicity”[All Fields] OR ”dynamics”[All Fields] OR ”dynamism”[All Fields] OR ”dynamisms”[All Fields]) OR (”model”[All Fields] OR ”model s”[All Fields] OR ”modeled”[All Fields] OR ”modeler”[All Fields] OR ”modeler s”[All Fields] OR ”modelers”[All Fields] OR ”modeling”[All Fields] OR ”modelings”[All Fields] OR ”modelization”[All Fields] OR ”modelizations”[All Fields] OR ”modelize”[All Fields] OR ”modelized”[All Fields] OR ”modelled”[All Fields] OR ”modeller”[All Fields] OR ”modellers”[All Fields] OR ”modelling”[All Fields] OR ”modellings”[All Fields] OR ”models”[All Fields]) OR ”vitro”[All Fields]) AND (”gut”[Journal] OR ”gut”[All Fields] OR (”intestinalization”[All Fields] OR ”intestinalized”[All Fields] OR ”intestinally”[All Fields] OR ”intestinals”[All Fields] OR ”intestine s”[All Fields] OR ”intestines”[MeSH Terms] OR ”intestines”[All Fields] OR ”intestinal”[All Fields] OR ”intestine”[All Fields]) OR (”gastrointestinal”[All Fields] OR ”gastrointestinally”[All Fields] OR ”gastrointestine”[All Fields])) AND (”microbiota”[MeSH Terms] OR ”microbiota”[All Fields] OR ”microbiotas”[All Fields] OR ”microbiota s”[All Fields] OR ”microbiotae”[All Fields] OR (”microbiome s”[All Fields] OR ”microbiomic”[All Fields] OR ”microbiomics”[All Fields] OR ”microbiota”[MeSH Terms] OR ”microbiota”[All Fields] OR ”microbiome”[All Fields] OR ”microbiomes”[All Fields]) OR (”microbial”[All Fields] OR ”microbially”[All Fields] OR ”microbials”[All Fields]))) AND (”faecally”[All Fields] OR ”fecally”[All Fields] OR ”fecals”[All Fields] OR ”feces”[MeSH Terms] OR ”feces”[All Fields] OR ”faecal”[All Fields] OR ”fecal”[All Fields]) AND ”humans”[MeSH Terms] AND ”gut simulation”[Title/Abstract]) AND (humans[Filter])	2	1899-12-31 00:19:25

B-11	(((”bioreactor s”[All Fields] OR ”bioreactors”[MeSH Terms] OR ”bioreactors”[All Fields] OR ”bioreactor”[All Fields] OR (”chemostat”[All Fields] OR ”chemostatic”[All Fields] OR ”chemostats”[All Fields]) OR (”reactor”[All Fields] OR ”reactor s”[All Fields] OR ”reactors”[All Fields]) OR ”shime”[All Fields] OR (”computer simulation”[MeSH Terms] OR (”computer”[All Fields] AND ”simulation”[All Fields]) OR ”computer simulation”[All Fields] OR ”simulation”[All Fields] OR ”simul”[All Fields] OR ”simulate”[All Fields] OR ”simulated”[All Fields] OR ”simulates”[All Fields] OR ”simulating”[All Fields] OR ”simulation s”[All Fields] OR ”simulational”[All Fields] OR ”simulations”[All Fields] OR ”simulative”[All Fields] OR ”simulator”[All Fields] OR ”simulator s”[All Fields] OR ”simulators”[All Fields]) OR (”computer simulation”[MeSH Terms] OR (”computer”[All Fields] AND ”simulation”[All Fields]) OR ”computer simulation”[All Fields] OR ”simulation”[All Fields] OR ”simul”[All Fields] OR ”simulate”[All Fields] OR ”simulated”[All Fields] OR ”simulates”[All Fields] OR ”simulating”[All Fields] OR ”simulation s”[All Fields] OR ”simulational”[All Fields] OR ”simulations”[All Fields] OR ”simulative”[All Fields] OR ”simulator”[All Fields] OR ”simulator s”[All Fields] OR ”simulators”[All Fields]) OR (”dynamer”[All Fields] OR ”dynamers”[All Fields] OR ”dynamic”[All Fields] OR ”dynamical”[All Fields] OR ”dynamically”[All Fields] OR ”dynamicity”[All Fields] OR ”dynamics”[All Fields] OR ”dynamism”[All Fields] OR ”dynamisms”[All Fields]) OR (”model”[All Fields] OR ”model s”[All Fields] OR ”modeled”[All Fields] OR ”modeler”[All Fields] OR ”modeler s”[All Fields] OR ”modelers”[All Fields] OR ”modeling”[All Fields] OR ”modelings”[All Fields] OR ”modelization”[All Fields] OR ”modelizations”[All Fields] OR ”modelize”[All Fields] OR ”modelized”[All Fields] OR ”modelled”[All Fields] OR ”modeller”[All Fields] OR ”modellers”[All Fields] OR ”modelling”[All Fields] OR ”modellings”[All Fields] OR ”models”[All Fields]) OR ”vitro”[All Fields]) AND (”gut”[Journal] OR ”gut”[All Fields] OR (”intestinalization”[All Fields] OR ”intestinalized”[All Fields] OR ”intestinally”[All Fields] OR ”intestinals”[All Fields] OR ”intestine s”[All Fields] OR ”intestines”[MeSH Terms] OR ”intestines”[All Fields] OR ”intestinal”[All Fields] OR ”intestine”[All Fields]) OR (”gastrointestinal”[All Fields] OR ”gastrointestinally”[All Fields] OR ”gastrointestine”[All Fields])) AND (”microbiota”[MeSH Terms] OR ”microbiota”[All Fields] OR ”microbiotas”[All Fields] OR ”microbiota s”[All Fields] OR ”microbiotae”[All Fields] OR (”microbiome s”[All Fields] OR ”microbiomic”[All Fields] OR ”microbiomics”[All Fields] OR ”microbiota”[MeSH Terms] OR ”microbiota”[All Fields] OR ”microbiome”[All Fields] OR ”microbiomes”[All Fields]) OR (”microbial”[All Fields] OR ”microbially”[All Fields] OR ”microbials”[All Fields]))) AND (”faecally”[All Fields] OR ”fecally”[All Fields] OR ”fecals”[All Fields] OR ”feces”[MeSH Terms] OR ”feces”[All Fields] OR ”faecal”[All Fields] OR ”fecal”[All Fields]) AND ”humans”[MeSH Terms] AND ”in vitro colon”[Title/Abstract]) AND (humans[Filter])	17	1899-12-31 00:18:09

B-12	(((”bioreactor s”[All Fields] OR ”bioreactors”[MeSH Terms] OR ”bioreactors”[All Fields] OR ”bioreactor”[All Fields] OR (”chemostat”[All Fields] OR ”chemostatic”[All Fields] OR ”chemostats”[All Fields]) OR (”reactor”[All Fields] OR ”reactor s”[All Fields] OR ”reactors”[All Fields]) OR ”shime”[All Fields] OR (”computer simulation”[MeSH Terms] OR (”computer”[All Fields] AND ”simulation”[All Fields]) OR ”computer simulation”[All Fields] OR ”simulation”[All Fields] OR ”simul”[All Fields] OR ”simulate”[All Fields] OR ”simulated”[All Fields] OR ”simulates”[All Fields] OR ”simulating”[All Fields] OR ”simulation s”[All Fields] OR ”simulational”[All Fields] OR ”simulations”[All Fields] OR ”simulative”[All Fields] OR ”simulator”[All Fields] OR ”simulator s”[All Fields] OR ”simulators”[All Fields]) OR (”computer simulation”[MeSH Terms] OR (”computer”[All Fields] AND ”simulation”[All Fields]) OR ”computer simulation”[All Fields] OR ”simulation”[All Fields] OR ”simul”[All Fields] OR ”simulate”[All Fields] OR ”simulated”[All Fields] OR ”simulates”[All Fields] OR ”simulating”[All Fields] OR ”simulation s”[All Fields] OR ”simulational”[All Fields] OR ”simulations”[All Fields] OR ”simulative”[All Fields] OR ”simulator”[All Fields] OR ”simulator s”[All Fields] OR ”simulators”[All Fields]) OR (”dynamer”[All Fields] OR ”dynamers”[All Fields] OR ”dynamic”[All Fields] OR ”dynamical”[All Fields] OR ”dynamically”[All Fields] OR ”dynamicity”[All Fields] OR ”dynamics”[All Fields] OR ”dynamism”[All Fields] OR ”dynamisms”[All Fields]) OR (”model”[All Fields] OR ”model s”[All Fields] OR ”modeled”[All Fields] OR ”modeler”[All Fields] OR ”modeler s”[All Fields] OR ”modelers”[All Fields] OR ”modeling”[All Fields] OR ”modelings”[All Fields] OR ”modelization”[All Fields] OR ”modelizations”[All Fields] OR ”modelize”[All Fields] OR ”modelized”[All Fields] OR ”modelled”[All Fields] OR ”modeller”[All Fields] OR ”modellers”[All Fields] OR ”modelling”[All Fields] OR ”modellings”[All Fields] OR ”models”[All Fields]) OR ”vitro”[All Fields]) AND (”gut”[Journal] OR ”gut”[All Fields] OR (”intestinalization”[All Fields] OR ”intestinalized”[All Fields] OR ”intestinally”[All Fields] OR ”intestinals”[All Fields] OR ”intestine s”[All Fields] OR ”intestines”[MeSH Terms] OR ”intestines”[All Fields] OR ”intestinal”[All Fields] OR ”intestine”[All Fields]) OR (”gastrointestinal”[All Fields] OR ”gastrointestinally”[All Fields] OR ”gastrointestine”[All Fields])) AND (”microbiota”[MeSH Terms] OR ”microbiota”[All Fields] OR ”microbiotas”[All Fields] OR ”microbiota s”[All Fields] OR ”microbiotae”[All Fields] OR (”microbiome s”[All Fields] OR ”microbiomic”[All Fields] OR ”microbiomics”[All Fields] OR ”microbiota”[MeSH Terms] OR ”microbiota”[All Fields] OR ”microbiome”[All Fields] OR ”microbiomes”[All Fields]) OR (”microbial”[All Fields] OR ”microbially”[All Fields] OR ”microbials”[All Fields]))) AND (”faecally”[All Fields] OR ”fecally”[All Fields] OR ”fecals”[All Fields] OR ”feces”[MeSH Terms] OR ”feces”[All Fields] OR ”faecal”[All Fields] OR ”fecal”[All Fields]) AND ”humans”[MeSH Terms] AND ”in vitro digestion”[Title/Abstract]) AND (humans[Filter])	37	1899-12-31 00:17:26

B-13	(((”bioreactor s”[All Fields] OR ”bioreactors”[MeSH Terms] OR ”bioreactors”[All Fields] OR ”bioreactor”[All Fields] OR (”chemostat”[All Fields] OR ”chemostatic”[All Fields] OR ”chemostats”[All Fields]) OR (”reactor”[All Fields] OR ”reactor s”[All Fields] OR ”reactors”[All Fields]) OR ”shime”[All Fields] OR (”computer simulation”[MeSH Terms] OR (”computer”[All Fields] AND ”simulation”[All Fields]) OR ”computer simulation”[All Fields] OR ”simulation”[All Fields] OR ”simul”[All Fields] OR ”simulate”[All Fields] OR ”simulated”[All Fields] OR ”simulates”[All Fields] OR ”simulating”[All Fields] OR ”simulation s”[All Fields] OR ”simulational”[All Fields] OR ”simulations”[All Fields] OR ”simulative”[All Fields] OR ”simulator”[All Fields] OR ”simulator s”[All Fields] OR ”simulators”[All Fields]) OR (”computer simulation”[MeSH Terms] OR (”computer”[All Fields] AND ”simulation”[All Fields]) OR ”computer simulation”[All Fields] OR ”simulation”[All Fields] OR ”simul”[All Fields] OR ”simulate”[All Fields] OR ”simulated”[All Fields] OR ”simulates”[All Fields] OR ”simulating”[All Fields] OR ”simulation s”[All Fields] OR ”simulational”[All Fields] OR ”simulations”[All Fields] OR ”simulative”[All Fields] OR ”simulator”[All Fields] OR ”simulator s”[All Fields] OR ”simulators”[All Fields]) OR (”dynamer”[All Fields] OR ”dynamers”[All Fields] OR ”dynamic”[All Fields] OR ”dynamical”[All Fields] OR ”dynamically”[All Fields] OR ”dynamicity”[All Fields] OR ”dynamics”[All Fields] OR ”dynamism”[All Fields] OR ”dynamisms”[All Fields]) OR (”model”[All Fields] OR ”model s”[All Fields] OR ”modeled”[All Fields] OR ”modeler”[All Fields] OR ”modeler s”[All Fields] OR ”modelers”[All Fields] OR ”modeling”[All Fields] OR ”modelings”[All Fields] OR ”modelization”[All Fields] OR ”modelizations”[All Fields] OR ”modelize”[All Fields] OR ”modelized”[All Fields] OR ”modelled”[All Fields] OR ”modeller”[All Fields] OR ”modellers”[All Fields] OR ”modelling”[All Fields] OR ”modellings”[All Fields] OR ”models”[All Fields]) OR ”vitro”[All Fields]) AND (”gut”[Journal] OR ”gut”[All Fields] OR (”intestinalization”[All Fields] OR ”intestinalized”[All Fields] OR ”intestinally”[All Fields] OR ”intestinals”[All Fields] OR ”intestine s”[All Fields] OR ”intestines”[MeSH Terms] OR ”intestines”[All Fields] OR ”intestinal”[All Fields] OR ”intestine”[All Fields]) OR (”gastrointestinal”[All Fields] OR ”gastrointestinally”[All Fields] OR ”gastrointestine”[All Fields])) AND (”microbiota”[MeSH Terms] OR ”microbiota”[All Fields] OR ”microbiotas”[All Fields] OR ”microbiota s”[All Fields] OR ”microbiotae”[All Fields] OR (”microbiome s”[All Fields] OR ”microbiomic”[All Fields] OR ”microbiomics”[All Fields] OR ”microbiota”[MeSH Terms] OR ”microbiota”[All Fields] OR ”microbiome”[All Fields] OR ”microbiomes”[All Fields]) OR (”microbial”[All Fields] OR ”microbially”[All Fields] OR ”microbials”[All Fields]))) AND (”faecally”[All Fields] OR ”fecally”[All Fields] OR ”fecals”[All Fields] OR ”feces”[MeSH Terms] OR ”feces”[All Fields] OR ”faecal”[All Fields] OR ”fecal”[All Fields]) AND ”humans”[MeSH Terms] AND ”in vitro gastrointestinal”[Title/Abstract]) AND (humans[Filter])	21	1899-12-31 00:07:59

B-14	(((”bioreactor s”[All Fields] OR ”bioreactors”[MeSH Terms] OR ”bioreactors”[All Fields] OR ”bioreactor”[All Fields] OR (”chemostat”[All Fields] OR ”chemostatic”[All Fields] OR ”chemostats”[All Fields]) OR (”reactor”[All Fields] OR ”reactor s”[All Fields] OR ”reactors”[All Fields]) OR ”shime”[All Fields] OR (”computer simulation”[MeSH Terms] OR (”computer”[All Fields] AND ”simulation”[All Fields]) OR ”computer simulation”[All Fields] OR ”simulation”[All Fields] OR ”simul”[All Fields] OR ”simulate”[All Fields] OR ”simulated”[All Fields] OR ”simulates”[All Fields] OR ”simulating”[All Fields] OR ”simulation s”[All Fields] OR ”simulational”[All Fields] OR ”simulations”[All Fields] OR ”simulative”[All Fields] OR ”simulator”[All Fields] OR ”simulator s”[All Fields] OR ”simulators”[All Fields]) OR (”computer simulation”[MeSH Terms] OR (”computer”[All Fields] AND ”simulation”[All Fields]) OR ”computer simulation”[All Fields] OR ”simulation”[All Fields] OR ”simul”[All Fields] OR ”simulate”[All Fields] OR ”simulated”[All Fields] OR ”simulates”[All Fields] OR ”simulating”[All Fields] OR ”simulation s”[All Fields] OR ”simulational”[All Fields] OR ”simulations”[All Fields] OR ”simulative”[All Fields] OR ”simulator”[All Fields] OR ”simulator s”[All Fields] OR ”simulators”[All Fields]) OR (”dynamer”[All Fields] OR ”dynamers”[All Fields] OR ”dynamic”[All Fields] OR ”dynamical”[All Fields] OR ”dynamically”[All Fields] OR ”dynamicity”[All Fields] OR ”dynamics”[All Fields] OR ”dynamism”[All Fields] OR ”dynamisms”[All Fields]) OR (”model”[All Fields] OR ”model s”[All Fields] OR ”modeled”[All Fields] OR ”modeler”[All Fields] OR ”modeler s”[All Fields] OR ”modelers”[All Fields] OR ”modeling”[All Fields] OR ”modelings”[All Fields] OR ”modelization”[All Fields] OR ”modelizations”[All Fields] OR ”modelize”[All Fields] OR ”modelized”[All Fields] OR ”modelled”[All Fields] OR ”modeller”[All Fields] OR ”modellers”[All Fields] OR ”modelling”[All Fields] OR ”modellings”[All Fields] OR ”models”[All Fields]) OR ”vitro”[All Fields]) AND (”gut”[Journal] OR ”gut”[All Fields] OR (”intestinalization”[All Fields] OR ”intestinalized”[All Fields] OR ”intestinally”[All Fields] OR ”intestinals”[All Fields] OR ”intestine s”[All Fields] OR ”intestines”[MeSH Terms] OR ”intestines”[All Fields] OR ”intestinal”[All Fields] OR ”intestine”[All Fields]) OR (”gastrointestinal”[All Fields] OR ”gastrointestinally”[All Fields] OR ”gastrointestine”[All Fields])) AND (”microbiota”[MeSH Terms] OR ”microbiota”[All Fields] OR ”microbiotas”[All Fields] OR ”microbiota s”[All Fields] OR ”microbiotae”[All Fields] OR (”microbiome s”[All Fields] OR ”microbiomic”[All Fields] OR ”microbiomics”[All Fields] OR ”microbiota”[MeSH Terms] OR ”microbiota”[All Fields] OR ”microbiome”[All Fields] OR ”microbiomes”[All Fields]) OR (”microbial”[All Fields] OR ”microbially”[All Fields] OR ”microbials”[All Fields]))) AND (”faecally”[All Fields] OR ”fecally”[All Fields] OR ”fecals”[All Fields] OR ”feces”[MeSH Terms] OR ”feces”[All Fields] OR ”faecal”[All Fields] OR ”fecal”[All Fields]) AND ”humans”[MeSH Terms] AND ”in vitro model”[Title/Abstract]) AND (humans[Filter])	54	1899-12-31 00:05:49

B-15	(((”bioreactor s”[All Fields] OR ”bioreactors”[MeSH Terms] OR ”bioreactors”[All Fields] OR ”bioreactor”[All Fields] OR (”chemostat”[All Fields] OR ”chemostatic”[All Fields] OR ”chemostats”[All Fields]) OR (”reactor”[All Fields] OR ”reactor s”[All Fields] OR ”reactors”[All Fields]) OR ”shime”[All Fields] OR (”computer simulation”[MeSH Terms] OR (”computer”[All Fields] AND ”simulation”[All Fields]) OR ”computer simulation”[All Fields] OR ”simulation”[All Fields] OR ”simul”[All Fields] OR ”simulate”[All Fields] OR ”simulated”[All Fields] OR ”simulates”[All Fields] OR ”simulating”[All Fields] OR ”simulation s”[All Fields] OR ”simulational”[All Fields] OR ”simulations”[All Fields] OR ”simulative”[All Fields] OR ”simulator”[All Fields] OR ”simulator s”[All Fields] OR ”simulators”[All Fields]) OR (”computer simulation”[MeSH Terms] OR (”computer”[All Fields] AND ”simulation”[All Fields]) OR ”computer simulation”[All Fields] OR ”simulation”[All Fields] OR ”simul”[All Fields] OR ”simulate”[All Fields] OR ”simulated”[All Fields] OR ”simulates”[All Fields] OR ”simulating”[All Fields] OR ”simulation s”[All Fields] OR ”simulational”[All Fields] OR ”simulations”[All Fields] OR ”simulative”[All Fields] OR ”simulator”[All Fields] OR ”simulator s”[All Fields] OR ”simulators”[All Fields]) OR (”dynamer”[All Fields] OR ”dynamers”[All Fields] OR ”dynamic”[All Fields] OR ”dynamical”[All Fields] OR ”dynamically”[All Fields] OR ”dynamicity”[All Fields] OR ”dynamics”[All Fields] OR ”dynamism”[All Fields] OR ”dynamisms”[All Fields]) OR (”model”[All Fields] OR ”model s”[All Fields] OR ”modeled”[All Fields] OR ”modeler”[All Fields] OR ”modeler s”[All Fields] OR ”modelers”[All Fields] OR ”modeling”[All Fields] OR ”modelings”[All Fields] OR ”modelization”[All Fields] OR ”modelizations”[All Fields] OR ”modelize”[All Fields] OR ”modelized”[All Fields] OR ”modelled”[All Fields] OR ”modeller”[All Fields] OR ”modellers”[All Fields] OR ”modelling”[All Fields] OR ”modellings”[All Fields] OR ”models”[All Fields]) OR ”vitro”[All Fields]) AND (”gut”[Journal] OR ”gut”[All Fields] OR (”intestinalization”[All Fields] OR ”intestinalized”[All Fields] OR ”intestinally”[All Fields] OR ”intestinals”[All Fields] OR ”intestine s”[All Fields] OR ”intestines”[MeSH Terms] OR ”intestines”[All Fields] OR ”intestinal”[All Fields] OR ”intestine”[All Fields]) OR (”gastrointestinal”[All Fields] OR ”gastrointestinally”[All Fields] OR ”gastrointestine”[All Fields])) AND (”microbiota”[MeSH Terms] OR ”microbiota”[All Fields] OR ”microbiotas”[All Fields] OR ”microbiota s”[All Fields] OR ”microbiotae”[All Fields] OR (”microbiome s”[All Fields] OR ”microbiomic”[All Fields] OR ”microbiomics”[All Fields] OR ”microbiota”[MeSH Terms] OR ”microbiota”[All Fields] OR ”microbiome”[All Fields] OR ”microbiomes”[All Fields]) OR (”microbial”[All Fields] OR ”microbially”[All Fields] OR ”microbials”[All Fields]))) AND (”faecally”[All Fields] OR ”fecally”[All Fields] OR ”fecals”[All Fields] OR ”feces”[MeSH Terms] OR ”feces”[All Fields] OR ”faecal”[All Fields] OR ”fecal”[All Fields]) AND ”humans”[MeSH Terms] AND ”reactor”[Title/Abstract]) AND (humans[Filter])	15	1899-12-31 00:30:30

B-16	((”bioreactor s”[All Fields] OR ”bioreactors”[MeSH Terms] OR ”bioreactors”[All Fields] OR ”bioreactor”[All Fields] OR (”chemostat”[All Fields] OR ”chemostatic”[All Fields] OR ”chemostats”[All Fields]) OR (”reactor”[All Fields] OR ”reactor s”[All Fields] OR ”reactors”[All Fields]) OR ”shime”[All Fields] OR (”computer simulation”[MeSH Terms] OR (”computer”[All Fields] AND ”simulation”[All Fields]) OR ”computer simulation”[All Fields] OR ”simulation”[All Fields] OR ”simul”[All Fields] OR ”simulate”[All Fields] OR ”simulated”[All Fields] OR ”simulates”[All Fields] OR ”simulating”[All Fields] OR ”simulation s”[All Fields] OR ”simulational”[All Fields] OR ”simulations”[All Fields] OR ”simulative”[All Fields] OR ”simulator”[All Fields] OR ”simulator s”[All Fields] OR ”simulators”[All Fields]) OR (”computer simulation”[MeSH Terms] OR (”computer”[All Fields] AND ”simulation”[All Fields]) OR ”computer simulation”[All Fields] OR ”simulation”[All Fields] OR ”simul”[All Fields] OR ”simulate”[All Fields] OR ”simulated”[All Fields] OR ”simulates”[All Fields] OR ”simulating”[All Fields] OR ”simulation s”[All Fields] OR ”simulational”[All Fields] OR ”simulations”[All Fields] OR ”simulative”[All Fields] OR ”simulator”[All Fields] OR ”simulator s”[All Fields] OR ”simulators”[All Fields]) OR (”dynamer”[All Fields] OR ”dynamers”[All Fields] OR ”dynamic”[All Fields] OR ”dynamical”[All Fields] OR ”dynamically”[All Fields] OR ”dynamicity”[All Fields] OR ”dynamics”[All Fields] OR ”dynamism”[All Fields] OR ”dynamisms”[All Fields]) OR (”model”[All Fields] OR ”model s”[All Fields] OR ”modeled”[All Fields] OR ”modeler”[All Fields] OR ”modeler s”[All Fields] OR ”modelers”[All Fields] OR ”modeling”[All Fields] OR ”modelings”[All Fields] OR ”modelization”[All Fields] OR ”modelizations”[All Fields] OR ”modelize”[All Fields] OR ”modelized”[All Fields] OR ”modelled”[All Fields] OR ”modeller”[All Fields] OR ”modellers”[All Fields] OR ”modelling”[All Fields] OR ”modellings”[All Fields] OR ”models”[All Fields]) OR ”vitro”[All Fields]) AND (”gut”[Journal] OR ”gut”[All Fields] OR (”intestinalization”[All Fields] OR ”intestinalized”[All Fields] OR ”intestinally”[All Fields] OR ”intestinals”[All Fields] OR ”intestine s”[All Fields] OR ”intestines”[MeSH Terms] OR ”intestines”[All Fields] OR ”intestinal”[All Fields] OR ”intestine”[All Fields]) OR (”gastrointestinal”[All Fields] OR ”gastrointestinally”[All Fields] OR ”gastrointestine”[All Fields])) AND (”microbiota”[MeSH Terms] OR ”microbiota”[All Fields] OR ”microbiotas”[All Fields] OR ”microbiota s”[All Fields] OR ”microbiotae”[All Fields] OR (”microbiome s”[All Fields] OR ”microbiomic”[All Fields] OR ”microbiomics”[All Fields] OR ”microbiota”[MeSH Terms] OR ”microbiota”[All Fields] OR ”microbiome”[All Fields] OR ”microbiomes”[All Fields]) OR (”microbial”[All Fields] OR ”microbially”[All Fields] OR ”microbials”[All Fields])) AND (”faecally”[All Fields] OR ”fecally”[All Fields] OR ”fecals”[All Fields] OR ”feces”[MeSH Terms] OR ”feces”[All Fields] OR ”faecal”[All Fields] OR ”fecal”[All Fields]) AND ”humans”[MeSH Terms] AND ”shime”[Title/Abstract]) AND (humans[Filter])	31	1899-12-31 23:59:00

B-17	(((”bioreactor s”[All Fields] OR ”bioreactors”[MeSH Terms] OR ”bioreactors”[All Fields] OR ”bioreactor”[All Fields] OR (”chemostat”[All Fields] OR ”chemostatic”[All Fields] OR ”chemostats”[All Fields]) OR (”reactor”[All Fields] OR ”reactor s”[All Fields] OR ”reactors”[All Fields]) OR ”shime”[All Fields] OR (”computer simulation”[MeSH Terms] OR (”computer”[All Fields] AND ”simulation”[All Fields]) OR ”computer simulation”[All Fields] OR ”simulation”[All Fields] OR ”simul”[All Fields] OR ”simulate”[All Fields] OR ”simulated”[All Fields] OR ”simulates”[All Fields] OR ”simulating”[All Fields] OR ”simulation s”[All Fields] OR ”simulational”[All Fields] OR ”simulations”[All Fields] OR ”simulative”[All Fields] OR ”simulator”[All Fields] OR ”simulator s”[All Fields] OR ”simulators”[All Fields]) OR (”computer simulation”[MeSH Terms] OR (”computer”[All Fields] AND ”simulation”[All Fields]) OR ”computer simulation”[All Fields] OR ”simulation”[All Fields] OR ”simul”[All Fields] OR ”simulate”[All Fields] OR ”simulated”[All Fields] OR ”simulates”[All Fields] OR ”simulating”[All Fields] OR ”simulation s”[All Fields] OR ”simulational”[All Fields] OR ”simulations”[All Fields] OR ”simulative”[All Fields] OR ”simulator”[All Fields] OR ”simulator s”[All Fields] OR ”simulators”[All Fields]) OR (”dynamer”[All Fields] OR ”dynamers”[All Fields] OR ”dynamic”[All Fields] OR ”dynamical”[All Fields] OR ”dynamically”[All Fields] OR ”dynamicity”[All Fields] OR ”dynamics”[All Fields] OR ”dynamism”[All Fields] OR ”dynamisms”[All Fields]) OR (”model”[All Fields] OR ”model s”[All Fields] OR ”modeled”[All Fields] OR ”modeler”[All Fields] OR ”modeler s”[All Fields] OR ”modelers”[All Fields] OR ”modeling”[All Fields] OR ”modelings”[All Fields] OR ”modelization”[All Fields] OR ”modelizations”[All Fields] OR ”modelize”[All Fields] OR ”modelized”[All Fields] OR ”modelled”[All Fields] OR ”modeller”[All Fields] OR ”modellers”[All Fields] OR ”modelling”[All Fields] OR ”modellings”[All Fields] OR ”models”[All Fields]) OR ”vitro”[All Fields]) AND (”gut”[Journal] OR ”gut”[All Fields] OR (”intestinalization”[All Fields] OR ”intestinalized”[All Fields] OR ”intestinally”[All Fields] OR ”intestinals”[All Fields] OR ”intestine s”[All Fields] OR ”intestines”[MeSH Terms] OR ”intestines”[All Fields] OR ”intestinal”[All Fields] OR ”intestine”[All Fields]) OR (”gastrointestinal”[All Fields] OR ”gastrointestinally”[All Fields] OR ”gastrointestine”[All Fields])) AND (”microbiota”[MeSH Terms] OR ”microbiota”[All Fields] OR ”microbiotas”[All Fields] OR ”microbiota s”[All Fields] OR ”microbiotae”[All Fields] OR (”microbiome s”[All Fields] OR ”microbiomic”[All Fields] OR ”microbiomics”[All Fields] OR ”microbiota”[MeSH Terms] OR ”microbiota”[All Fields] OR ”microbiome”[All Fields] OR ”microbiomes”[All Fields]) OR (”microbial”[All Fields] OR ”microbially”[All Fields] OR ”microbials”[All Fields]))) AND (”faecally”[All Fields] OR ”fecally”[All Fields] OR ”fecals”[All Fields] OR ”feces”[MeSH Terms] OR ”feces”[All Fields] OR ”faecal”[All Fields] OR ”fecal”[All Fields]) AND ”humans”[MeSH Terms] AND ”simulated colon”[Title/Abstract]) AND (humans[Filter])	6	1899-12-31 00:05:03

B-18	(((”bioreactor s”[All Fields] OR ”bioreactors”[MeSH Terms] OR ”bioreactors”[All Fields] OR ”bioreactor”[All Fields] OR (”chemostat”[All Fields] OR ”chemostatic”[All Fields] OR ”chemostats”[All Fields]) OR (”reactor”[All Fields] OR ”reactor s”[All Fields] OR ”reactors”[All Fields]) OR ”shime”[All Fields] OR (”computer simulation”[MeSH Terms] OR (”computer”[All Fields] AND ”simulation”[All Fields]) OR ”computer simulation”[All Fields] OR ”simulation”[All Fields] OR ”simul”[All Fields] OR ”simulate”[All Fields] OR ”simulated”[All Fields] OR ”simulates”[All Fields] OR ”simulating”[All Fields] OR ”simulation s”[All Fields] OR ”simulational”[All Fields] OR ”simulations”[All Fields] OR ”simulative”[All Fields] OR ”simulator”[All Fields] OR ”simulator s”[All Fields] OR ”simulators”[All Fields]) OR (”computer simulation”[MeSH Terms] OR (”computer”[All Fields] AND ”simulation”[All Fields]) OR ”computer simulation”[All Fields] OR ”simulation”[All Fields] OR ”simul”[All Fields] OR ”simulate”[All Fields] OR ”simulated”[All Fields] OR ”simulates”[All Fields] OR ”simulating”[All Fields] OR ”simulation s”[All Fields] OR ”simulational”[All Fields] OR ”simulations”[All Fields] OR ”simulative”[All Fields] OR ”simulator”[All Fields] OR ”simulator s”[All Fields] OR ”simulators”[All Fields]) OR (”dynamer”[All Fields] OR ”dynamers”[All Fields] OR ”dynamic”[All Fields] OR ”dynamical”[All Fields] OR ”dynamically”[All Fields] OR ”dynamicity”[All Fields] OR ”dynamics”[All Fields] OR ”dynamism”[All Fields] OR ”dynamisms”[All Fields]) OR (”model”[All Fields] OR ”model s”[All Fields] OR ”modeled”[All Fields] OR ”modeler”[All Fields] OR ”modeler s”[All Fields] OR ”modelers”[All Fields] OR ”modeling”[All Fields] OR ”modelings”[All Fields] OR ”modelization”[All Fields] OR ”modelizations”[All Fields] OR ”modelize”[All Fields] OR ”modelized”[All Fields] OR ”modelled”[All Fields] OR ”modeller”[All Fields] OR ”modellers”[All Fields] OR ”modelling”[All Fields] OR ”modellings”[All Fields] OR ”models”[All Fields]) OR ”vitro”[All Fields]) AND (”gut”[Journal] OR ”gut”[All Fields] OR (”intestinalization”[All Fields] OR ”intestinalized”[All Fields] OR ”intestinally”[All Fields] OR ”intestinals”[All Fields] OR ”intestine s”[All Fields] OR ”intestines”[MeSH Terms] OR ”intestines”[All Fields] OR ”intestinal”[All Fields] OR ”intestine”[All Fields]) OR (”gastrointestinal”[All Fields] OR ”gastrointestinally”[All Fields] OR ”gastrointestine”[All Fields])) AND (”microbiota”[MeSH Terms] OR ”microbiota”[All Fields] OR ”microbiotas”[All Fields] OR ”microbiota s”[All Fields] OR ”microbiotae”[All Fields] OR (”microbiome s”[All Fields] OR ”microbiomic”[All Fields] OR ”microbiomics”[All Fields] OR ”microbiota”[MeSH Terms] OR ”microbiota”[All Fields] OR ”microbiome”[All Fields] OR ”microbiomes”[All Fields]) OR (”microbial”[All Fields] OR ”microbially”[All Fields] OR ”microbials”[All Fields]))) AND (”faecally”[All Fields] OR ”fecally”[All Fields] OR ”fecals”[All Fields] OR ”feces”[MeSH Terms] OR ”feces”[All Fields] OR ”faecal”[All Fields] OR ”fecal”[All Fields]) AND ”humans”[MeSH Terms] AND ”simulated colonic”[Title/Abstract]) AND (humans[Filter])	8	1899-12-31 00:04:15

B-19	(((”bioreactor s”[All Fields] OR ”bioreactors”[MeSH Terms] OR ”bioreactors”[All Fields] OR ”bioreactor”[All Fields] OR (”chemostat”[All Fields] OR ”chemostatic”[All Fields] OR ”chemostats”[All Fields]) OR (”reactor”[All Fields] OR ”reactor s”[All Fields] OR ”reactors”[All Fields]) OR ”shime”[All Fields] OR (”computer simulation”[MeSH Terms] OR (”computer”[All Fields] AND ”simulation”[All Fields]) OR ”computer simulation”[All Fields] OR ”simulation”[All Fields] OR ”simul”[All Fields] OR ”simulate”[All Fields] OR ”simulated”[All Fields] OR ”simulates”[All Fields] OR ”simulating”[All Fields] OR ”simulation s”[All Fields] OR ”simulational”[All Fields] OR ”simulations”[All Fields] OR ”simulative”[All Fields] OR ”simulator”[All Fields] OR ”simulator s”[All Fields] OR ”simulators”[All Fields]) OR (”computer simulation”[MeSH Terms] OR (”computer”[All Fields] AND ”simulation”[All Fields]) OR ”computer simulation”[All Fields] OR ”simulation”[All Fields] OR ”simul”[All Fields] OR ”simulate”[All Fields] OR ”simulated”[All Fields] OR ”simulates”[All Fields] OR ”simulating”[All Fields] OR ”simulation s”[All Fields] OR ”simulational”[All Fields] OR ”simulations”[All Fields] OR ”simulative”[All Fields] OR ”simulator”[All Fields] OR ”simulator s”[All Fields] OR ”simulators”[All Fields]) OR (”dynamer”[All Fields] OR ”dynamers”[All Fields] OR ”dynamic”[All Fields] OR ”dynamical”[All Fields] OR ”dynamically”[All Fields] OR ”dynamicity”[All Fields] OR ”dynamics”[All Fields] OR ”dynamism”[All Fields] OR ”dynamisms”[All Fields]) OR (”model”[All Fields] OR ”model s”[All Fields] OR ”modeled”[All Fields] OR ”modeler”[All Fields] OR ”modeler s”[All Fields] OR ”modelers”[All Fields] OR ”modeling”[All Fields] OR ”modelings”[All Fields] OR ”modelization”[All Fields] OR ”modelizations”[All Fields] OR ”modelize”[All Fields] OR ”modelized”[All Fields] OR ”modelled”[All Fields] OR ”modeller”[All Fields] OR ”modellers”[All Fields] OR ”modelling”[All Fields] OR ”modellings”[All Fields] OR ”models”[All Fields]) OR ”vitro”[All Fields]) AND (”gut”[Journal] OR ”gut”[All Fields] OR (”intestinalization”[All Fields] OR ”intestinalized”[All Fields] OR ”intestinally”[All Fields] OR ”intestinals”[All Fields] OR ”intestine s”[All Fields] OR ”intestines”[MeSH Terms] OR ”intestines”[All Fields] OR ”intestinal”[All Fields] OR ”intestine”[All Fields]) OR (”gastrointestinal”[All Fields] OR ”gastrointestinally”[All Fields] OR ”gastrointestine”[All Fields])) AND (”microbiota”[MeSH Terms] OR ”microbiota”[All Fields] OR ”microbiotas”[All Fields] OR ”microbiota s”[All Fields] OR ”microbiotae”[All Fields] OR (”microbiome s”[All Fields] OR ”microbiomic”[All Fields] OR ”microbiomics”[All Fields] OR ”microbiota”[MeSH Terms] OR ”microbiota”[All Fields] OR ”microbiome”[All Fields] OR ”microbiomes”[All Fields]) OR (”microbial”[All Fields] OR ”microbially”[All Fields] OR ”microbials”[All Fields]))) AND (”faecally”[All Fields] OR ”fecally”[All Fields] OR ”fecals”[All Fields] OR ”feces”[MeSH Terms] OR ”feces”[All Fields] OR ”faecal”[All Fields] OR ”fecal”[All Fields]) AND ”humans”[MeSH Terms] AND ”simulated gastrointestinal”[Title/Abstract]) AND (humans[Filter])	26	1899-12-31 00:03:06

B-20	(((”bioreactor s”[All Fields] OR ”bioreactors”[MeSH Terms] OR ”bioreactors”[All Fields] OR ”bioreactor”[All Fields] OR (”chemostat”[All Fields] OR ”chemostatic”[All Fields] OR ”chemostats”[All Fields]) OR (”reactor”[All Fields] OR ”reactor s”[All Fields] OR ”reactors”[All Fields]) OR ”shime”[All Fields] OR (”computer simulation”[MeSH Terms] OR (”computer”[All Fields] AND ”simulation”[All Fields]) OR ”computer simulation”[All Fields] OR ”simulation”[All Fields] OR ”simul”[All Fields] OR ”simulate”[All Fields] OR ”simulated”[All Fields] OR ”simulates”[All Fields] OR ”simulating”[All Fields] OR ”simulation s”[All Fields] OR ”simulational”[All Fields] OR ”simulations”[All Fields] OR ”simulative”[All Fields] OR ”simulator”[All Fields] OR ”simulator s”[All Fields] OR ”simulators”[All Fields]) OR (”computer simulation”[MeSH Terms] OR (”computer”[All Fields] AND ”simulation”[All Fields]) OR ”computer simulation”[All Fields] OR ”simulation”[All Fields] OR ”simul”[All Fields] OR ”simulate”[All Fields] OR ”simulated”[All Fields] OR ”simulates”[All Fields] OR ”simulating”[All Fields] OR ”simulation s”[All Fields] OR ”simulational”[All Fields] OR ”simulations”[All Fields] OR ”simulative”[All Fields] OR ”simulator”[All Fields] OR ”simulator s”[All Fields] OR ”simulators”[All Fields]) OR (”dynamer”[All Fields] OR ”dynamers”[All Fields] OR ”dynamic”[All Fields] OR ”dynamical”[All Fields] OR ”dynamically”[All Fields] OR ”dynamicity”[All Fields] OR ”dynamics”[All Fields] OR ”dynamism”[All Fields] OR ”dynamisms”[All Fields]) OR (”model”[All Fields] OR ”model s”[All Fields] OR ”modeled”[All Fields] OR ”modeler”[All Fields] OR ”modeler s”[All Fields] OR ”modelers”[All Fields] OR ”modeling”[All Fields] OR ”modelings”[All Fields] OR ”modelization”[All Fields] OR ”modelizations”[All Fields] OR ”modelize”[All Fields] OR ”modelized”[All Fields] OR ”modelled”[All Fields] OR ”modeller”[All Fields] OR ”modellers”[All Fields] OR ”modelling”[All Fields] OR ”modellings”[All Fields] OR ”models”[All Fields]) OR ”vitro”[All Fields]) AND (”gut”[Journal] OR ”gut”[All Fields] OR (”intestinalization”[All Fields] OR ”intestinalized”[All Fields] OR ”intestinally”[All Fields] OR ”intestinals”[All Fields] OR ”intestine s”[All Fields] OR ”intestines”[MeSH Terms] OR ”intestines”[All Fields] OR ”intestinal”[All Fields] OR ”intestine”[All Fields]) OR (”gastrointestinal”[All Fields] OR ”gastrointestinally”[All Fields] OR ”gastrointestine”[All Fields])) AND (”microbiota”[MeSH Terms] OR ”microbiota”[All Fields] OR ”microbiotas”[All Fields] OR ”microbiota s”[All Fields] OR ”microbiotae”[All Fields] OR (”microbiome s”[All Fields] OR ”microbiomic”[All Fields] OR ”microbiomics”[All Fields] OR ”microbiota”[MeSH Terms] OR ”microbiota”[All Fields] OR ”microbiome”[All Fields] OR ”microbiomes”[All Fields]) OR (”microbial”[All Fields] OR ”microbially”[All Fields] OR ”microbials”[All Fields]))) AND (”faecally”[All Fields] OR ”fecally”[All Fields] OR ”fecals”[All Fields] OR ”feces”[MeSH Terms] OR ”feces”[All Fields] OR ”faecal”[All Fields] OR ”fecal”[All Fields]) AND ”humans”[MeSH Terms] AND ”simulator of human”[Title/Abstract]) AND (humans[Filter])	6	1899-12-31 00:00:55

B-21	(((”bioreactor s”[All Fields] OR ”bioreactors”[MeSH Terms] OR ”bioreactors”[All Fields] OR ”bioreactor”[All Fields] OR (”chemostat”[All Fields] OR ”chemostatic”[All Fields] OR ”chemostats”[All Fields]) OR (”reactor”[All Fields] OR ”reactor s”[All Fields] OR ”reactors”[All Fields]) OR ”shime”[All Fields] OR (”computer simulation”[MeSH Terms] OR (”computer”[All Fields] AND ”simulation”[All Fields]) OR ”computer simulation”[All Fields] OR ”simulation”[All Fields] OR ”simul”[All Fields] OR ”simulate”[All Fields] OR ”simulated”[All Fields] OR ”simulates”[All Fields] OR ”simulating”[All Fields] OR ”simulation s”[All Fields] OR ”simulational”[All Fields] OR ”simulations”[All Fields] OR ”simulative”[All Fields] OR ”simulator”[All Fields] OR ”simulator s”[All Fields] OR ”simulators”[All Fields]) OR (”computer simulation”[MeSH Terms] OR (”computer”[All Fields] AND ”simulation”[All Fields]) OR ”computer simulation”[All Fields] OR ”simulation”[All Fields] OR ”simul”[All Fields] OR ”simulate”[All Fields] OR ”simulated”[All Fields] OR ”simulates”[All Fields] OR ”simulating”[All Fields] OR ”simulation s”[All Fields] OR ”simulational”[All Fields] OR ”simulations”[All Fields] OR ”simulative”[All Fields] OR ”simulator”[All Fields] OR ”simulator s”[All Fields] OR ”simulators”[All Fields]) OR (”dynamer”[All Fields] OR ”dynamers”[All Fields] OR ”dynamic”[All Fields] OR ”dynamical”[All Fields] OR ”dynamically”[All Fields] OR ”dynamicity”[All Fields] OR ”dynamics”[All Fields] OR ”dynamism”[All Fields] OR ”dynamisms”[All Fields]) OR (”model”[All Fields] OR ”model s”[All Fields] OR ”modeled”[All Fields] OR ”modeler”[All Fields] OR ”modeler s”[All Fields] OR ”modelers”[All Fields] OR ”modeling”[All Fields] OR ”modelings”[All Fields] OR ”modelization”[All Fields] OR ”modelizations”[All Fields] OR ”modelize”[All Fields] OR ”modelized”[All Fields] OR ”modelled”[All Fields] OR ”modeller”[All Fields] OR ”modellers”[All Fields] OR ”modelling”[All Fields] OR ”modellings”[All Fields] OR ”models”[All Fields]) OR ”vitro”[All Fields]) AND (”gut”[Journal] OR ”gut”[All Fields] OR (”intestinalization”[All Fields] OR ”intestinalized”[All Fields] OR ”intestinally”[All Fields] OR ”intestinals”[All Fields] OR ”intestine s”[All Fields] OR ”intestines”[MeSH Terms] OR ”intestines”[All Fields] OR ”intestinal”[All Fields] OR ”intestine”[All Fields]) OR (”gastrointestinal”[All Fields] OR ”gastrointestinally”[All Fields] OR ”gastrointestine”[All Fields])) AND (”microbiota”[MeSH Terms] OR ”microbiota”[All Fields] OR ”microbiotas”[All Fields] OR ”microbiota s”[All Fields] OR ”microbiotae”[All Fields] OR (”microbiome s”[All Fields] OR ”microbiomic”[All Fields] OR ”microbiomics”[All Fields] OR ”microbiota”[MeSH Terms] OR ”microbiota”[All Fields] OR ”microbiome”[All Fields] OR ”microbiomes”[All Fields]) OR (”microbial”[All Fields] OR ”microbially”[All Fields] OR ”microbials”[All Fields]))) AND (”faecally”[All Fields] OR ”fecally”[All Fields] OR ”fecals”[All Fields] OR ”feces”[MeSH Terms] OR ”feces”[All Fields] OR ”faecal”[All Fields] OR ”fecal”[All Fields]) AND ”humans”[MeSH Terms] AND ”simulator of the human”[Title/Abstract]) AND (humans[Filter])	24	1899-12-31 00:01:57

[*] Searches conducted with and without the word ’fecal’ are noted in the ’Search number’ column where (A) represents with FECAL in title (B) represents without FECAL in title

Table S3: Results from Scopus using 21 terms.

Search number	Output refined search	Documents	Details
A-1	ALL (bioreactor OR chemostat OR reactor OR shime OR simulator OR simulation OR dynamic OR model OR vitro) AND ALL (gut OR intestine OR gastrointestinal) AND ALL (microbiota OR microbiome OR microbial) AND human AND TITLE-ABS (”artificial gut”) AND (LIMIT-TO (DOCTYPE , ”ar”)) AND (LIMIT-TO (LANGUAGE , ”English”)) AND (EXCLUDE (EXACTKEYWORD , ”Nonhuman”) OR EXCLUDE (EXACTKEYWORD , ”Animals”) OR EXCLUDE (EXACTKEYWORD , ”Animal”) OR EXCLUDE (EXACTKEYWORD , ”Animal Experiment”) OR EXCLUDE (EXACTKEYWORD , ”Animal Model”) OR EXCLUDE (EXACTKEYWORD , ”Animal Tissue”) OR EXCLUDE (EXACTKEYWORD , ”Mice, Inbred C57BL”) OR EXCLUDE (EXACTKEYWORD , ”Rat”) OR EXCLUDE (EXACTKEYWORD , ”Disease Models, Animal”) OR EXCLUDE (EXACTKEYWORD , ”Rats”) OR EXCLUDE (EXACTKEYWORD , ”In Vivo Study”) OR EXCLUDE (EXACTKEYWORD , ”Animalia”) OR EXCLUDE (EXACTKEYWORD , ”Swine”) OR EXCLUDE (EXACTKEYWORD , ”Mice, Knockout”))	27	2021-11-25
A-2	ALL (bioreactor OR chemostat OR reactor OR shime OR simulator OR simulation OR dynamic OR model OR vitro) AND ALL (gut OR intestine OR gastrointestinal) AND ALL (microbiota OR microbiome OR microbial) AND human AND TITLE-ABS (”continuous culture”) AND (LIMIT-TO (DOCTYPE , ”ar”)) AND (LIMIT-TO (LANGUAGE , ”English”)) AND (EXCLUDE (EXACTKEYWORD , ”Nonhuman”) OR EXCLUDE (EXACTKEYWORD , ”Animals”) OR EXCLUDE (EXACTKEYWORD , ”Animal”) OR EXCLUDE (EXACTKEYWORD , ”Animal Experiment”) OR EXCLUDE (EXACTKEYWORD , ”Animal Model”) OR EXCLUDE (EXACTKEYWORD , ”Animal Tissue”) OR EXCLUDE (EXACTKEYWORD , ”Mice, Inbred C57BL”) OR EXCLUDE (EXACTKEYWORD , ”Rat”) OR EXCLUDE (EXACTKEYWORD , ”Disease Models, Animal”) OR EXCLUDE (EXACTKEYWORD , ”Rats”) OR EXCLUDE (EXACTKEYWORD , ”In Vivo Study”) OR EXCLUDE (EXACTKEYWORD , ”Animalia”) OR EXCLUDE (EXACTKEYWORD , ”Swine”) OR EXCLUDE (EXACTKEYWORD , ”Mice, Knockout”)) ...View More	52	2021-11-25
A-3	ALL (bioreactor OR chemostat OR reactor OR shime OR simulator OR simulation OR dynamic OR model OR vitro) AND ALL (gut OR intestine OR gastrointestinal) AND ALL (microbiota OR microbiome OR microbial) AND human AND TITLE-ABS (”continuous fermentation”) AND (LIMIT-TO (DOCTYPE , ”ar”)) AND (LIMIT-TO (LANGUAGE , ”English”)) AND (EXCLUDE (EXACTKEYWORD , ”Nonhuman”) OR EXCLUDE (EXACTKEYWORD , ”Animals”) OR EXCLUDE (EXACTKEYWORD , ”Animal”) OR EXCLUDE (EXACTKEYWORD , ”Animal Experiment”) OR EXCLUDE (EXACTKEYWORD , ”Animal Model”) OR EXCLUDE (EXACTKEYWORD , ”Animal Tissue”) OR EXCLUDE (EXACTKEYWORD , ”Mice, Inbred C57BL”) OR EXCLUDE (EXACTKEYWORD , ”Rat”) OR EXCLUDE (EXACTKEYWORD , ”Disease Models, Animal”) OR EXCLUDE (EXACTKEYWORD , ”Rats”) OR EXCLUDE (EXACTKEYWORD , ”In Vivo Study”) OR EXCLUDE (EXACTKEYWORD , ”Animalia”) OR EXCLUDE (EXACTKEYWORD , ”Swine”) OR EXCLUDE (EXACTKEYWORD , ”Mice, Knockout”)) ...View More	7	2021-11-25

A-4	ALL (bioreactor OR chemostat OR reactor OR shime OR simulator OR simulation OR dynamic OR model OR vitro) AND ALL (gut OR intestine OR gastrointestinal) AND ALL (microbiota OR microbiome OR microbial) AND human AND TITLE-ABS (”gastrointestinal model”) AND (LIMIT-TO (DOCTYPE , ”ar”)) AND (LIMIT-TO (LANGUAGE , ”English”)) AND (EXCLUDE (EXACTKEYWORD , ”Nonhuman”) OR EXCLUDE (EXACTKEYWORD , ”Animals”) OR EXCLUDE (EXACTKEYWORD , ”Animal”) OR EXCLUDE (EXACTKEYWORD , ”Animal Experiment”) OR EXCLUDE (EXACTKEYWORD , ”Animal Model”) OR EXCLUDE (EXACTKEYWORD , ”Animal Tissue”) OR EXCLUDE (EXACTKEYWORD , ”Mice, Inbred C57BL”) OR EXCLUDE (EXACTKEYWORD , ”Rat”) OR EXCLUDE (EXACTKEYWORD , ”Disease Models, Animal”) OR EXCLUDE (EXACTKEYWORD , ”Rats”) OR EXCLUDE (EXACTKEYWORD , ”In Vivo Study”) OR EXCLUDE (EXACTKEYWORD , ”Animalia”) OR EXCLUDE (EXACTKEYWORD , ”Swine”) OR EXCLUDE (EXACTKEYWORD , ”Mice, Knockout”))	50	2021-11-25
A-5	ALL (bioreactor OR chemostat OR reactor OR shime OR simulator OR simulation OR dynamic OR model OR vitro) AND ALL (gut OR intestine OR gastrointestinal) AND ALL (microbiota OR microbiome OR microbial) AND human AND TITLE-ABS (”git model”) AND (LIMIT-TO (DOCTYPE , ”ar”)) AND (LIMIT-TO (LANGUAGE , ”English”)) AND (EXCLUDE (EXACTKEYWORD , ”Nonhuman”) OR EXCLUDE (EXACTKEYWORD , ”Animals”) OR EXCLUDE (EXACTKEYWORD , ”Animal”) OR EXCLUDE (EXACTKEYWORD , ”Animal Experiment”) OR EXCLUDE (EXACTKEYWORD , ”Animal Model”) OR EXCLUDE (EXACTKEYWORD , ”Animal Tissue”) OR EXCLUDE (EXACTKEYWORD , ”Mice, Inbred C57BL”) OR EXCLUDE (EXACTKEYWORD , ”Rat”) OR EXCLUDE (EXACTKEYWORD , ”Disease Models, Animal”) OR EXCLUDE (EXACTKEYWORD , ”Rats”) OR EXCLUDE (EXACTKEYWORD , ”In Vivo Study”) OR EXCLUDE (EXACTKEYWORD , ”Animalia”) OR EXCLUDE (EXACTKEYWORD , ”Swine”) OR EXCLUDE (EXACTKEYWORD , ”Mice, Knockout”))	3	2021-11-25
A-6	ALL (bioreactor OR chemostat OR reactor OR shime OR simulator OR simulation OR dynamic OR model OR vitro) AND ALL (gut OR intestine OR gastrointestinal) AND ALL (microbiota OR microbiome OR microbial) AND human AND TITLE-ABS (”gut model”) AND (LIMIT-TO (DOCTYPE , ”ar”)) AND (LIMIT-TO (LANGUAGE , ”English”)) AND (EXCLUDE (EXACTKEYWORD , ”Nonhuman”) OR EXCLUDE (EXACTKEYWORD , ”Animals”) OR EXCLUDE (EXACTKEYWORD , ”Animal”) OR EXCLUDE (EXACTKEYWORD , ”Animal Experiment”) OR EXCLUDE (EXACTKEYWORD , ”Animal Model”) OR EXCLUDE (EXACTKEYWORD , ”Animal Tissue”) OR EXCLUDE (EXACTKEYWORD , ”Mice, Inbred C57BL”) OR EXCLUDE (EXACTKEYWORD , ”Rat”) OR EXCLUDE (EXACTKEYWORD , ”Disease Models, Animal”) OR EXCLUDE (EXACTKEYWORD , ”Rats”) OR EXCLUDE (EXACTKEYWORD , ”In Vivo Study”) OR EXCLUDE (EXACTKEYWORD , ”Animalia”) OR EXCLUDE (EXACTKEYWORD , ”Swine”) OR EXCLUDE (EXACTKEYWORD , ”Mice, Knockout”))	63	2021-11-25

A-7	ALL (bioreactor OR chemostat OR reactor OR shime OR simulator OR simulation OR dynamic OR model OR vitro) AND ALL (gut OR intestine OR gastrointestinal) AND ALL (microbiota OR microbiome OR microbial) AND human AND TITLE-ABS (”gut simulation”) AND (LIMIT-TO (DOCTYPE , ”ar”)) AND (LIMIT-TO (LANGUAGE , ”English”)) AND (EXCLUDE (EXACTKEYWORD , ”Nonhuman”) OR EXCLUDE (EXACTKEYWORD , ”Animals”) OR EXCLUDE (EXACTKEYWORD , ”Animal”) OR EXCLUDE (EXACTKEYWORD , ”Animal Experiment”) OR EXCLUDE (EXACTKEYWORD , ”Animal Model”) OR EXCLUDE (EXACTKEYWORD , ”Animal Tissue”) OR EXCLUDE (EXACTKEYWORD , ”Mice, Inbred C57BL”) OR EXCLUDE (EXACTKEYWORD , ”Rat”) OR EXCLUDE (EXACTKEYWORD , ”Disease Models, Animal”) OR EXCLUDE (EXACTKEYWORD , ”Rats”) OR EXCLUDE (EXACTKEYWORD , ”In Vivo Study”) OR EXCLUDE (EXACTKEYWORD , ”Animalia”) OR EXCLUDE (EXACTKEYWORD , ”Swine”) OR EXCLUDE (EXACTKEYWORD , ”Mice, Knockout”))	2	2021-11-25
A-8	ALL (bioreactor OR chemostat OR reactor OR shime OR simulator OR simulation OR dynamic OR model OR vitro) AND ALL (gut OR intestine OR gastrointestinal) AND ALL (microbiota OR microbiome OR microbial) AND human AND TITLE-ABS (”in vitro colon”) AND (LIMIT-TO (DOCTYPE , ”ar”)) AND (LIMIT-TO (LANGUAGE , ”English”)) AND (EXCLUDE (EXACTKEYWORD , ”Nonhuman”) OR EXCLUDE (EXACTKEYWORD , ”Animals”) OR EXCLUDE (EXACTKEYWORD , ”Animal”) OR EXCLUDE (EXACTKEYWORD , ”Animal Experiment”) OR EXCLUDE (EXACTKEYWORD , ”Animal Model”) OR EXCLUDE (EXACTKEYWORD , ”Animal Tissue”) OR EXCLUDE (EXACTKEYWORD , ”Mice, Inbred C57BL”) OR EXCLUDE (EXACTKEYWORD , ”Rat”) OR EXCLUDE (EXACTKEYWORD , ”Disease Models, Animal”) OR EXCLUDE (EXACTKEYWORD , ”Rats”) OR EXCLUDE (EXACTKEYWORD , ”In Vivo Study”) OR EXCLUDE (EXACTKEYWORD , ”Animalia”) OR EXCLUDE (EXACTKEYWORD , ”Swine”) OR EXCLUDE (EXACTKEYWORD , ”Mice, Knockout”))	32	2021-11-25
A-9	ALL (bioreactor OR chemostat OR reactor OR shime OR simulator OR simulation OR dynamic OR model OR vitro) AND ALL (gut OR intestine OR gastrointestinal) AND ALL (microbiota OR microbiome OR microbial) AND human AND TITLE-ABS (”in vitro digestion”) AND (LIMIT-TO (DOCTYPE , ”ar”)) AND (LIMIT-TO (LANGUAGE , ”English”)) AND (EXCLUDE (EXACTKEYWORD , ”Nonhuman”) OR EXCLUDE (EXACTKEYWORD , ”Animals”) OR EXCLUDE (EXACTKEYWORD , ”Animal”) OR EXCLUDE (EXACTKEYWORD , ”Animal Experiment”) OR EXCLUDE (EXACTKEYWORD , ”Animal Model”) OR EXCLUDE (EXACTKEYWORD , ”Animal Tissue”) OR EXCLUDE (EXACTKEYWORD , ”Mice, Inbred C57BL”) OR EXCLUDE (EXACTKEYWORD , ”Rat”) OR EXCLUDE (EXACTKEYWORD , ”Disease Models, Animal”) OR EXCLUDE (EXACTKEYWORD , ”Rats”) OR EXCLUDE (EXACTKEYWORD , ”In Vivo Study”) OR EXCLUDE (EXACTKEYWORD , ”Animalia”) OR EXCLUDE (EXACTKEYWORD , ”Swine”) OR EXCLUDE (EXACTKEYWORD , ”Mice, Knockout”))	496	2021-11-25

A-10	ALL (bioreactor OR chemostat OR reactor OR shime OR simulator OR simulation OR dynamic OR model OR vitro) AND ALL (gut OR intestine OR gastrointestinal) AND ALL (microbiota OR microbiome OR microbial) AND human AND TITLE-ABS (”in vitro gastrointestinal”) AND (LIMIT-TO (DOCTYPE , ”ar”)) AND (LIMIT-TO (LANGUAGE , ”English”)) AND (EXCLUDE (EXACTKEYWORD , ”Nonhuman”) OR EXCLUDE (EXACTKEYWORD , ”Animals”) OR EXCLUDE (EXACTKEYWORD , ”Animal”) OR EXCLUDE (EXACTKEYWORD , ”Animal Experiment”) OR EXCLUDE (EXACTKEYWORD , ”Animal Model”) OR EXCLUDE (EXACTKEYWORD , ”Animal Tissue”) OR EXCLUDE (EXACTKEYWORD , ”Mice, Inbred C57BL”) OR EXCLUDE (EXACTKEYWORD , ”Rat”) OR EXCLUDE (EXACTKEYWORD , ”Disease Models, Animal”) OR EXCLUDE (EXACTKEYWORD , ”Rats”) OR EXCLUDE (EXACTKEYWORD , ”In Vivo Study”) OR EXCLUDE (EXACTKEYWORD , ”Animalia”) OR EXCLUDE (EXACTKEYWORD , ”Swine”) OR EXCLUDE (EXACTKEYWORD , ”Mice, Knockout”))	248	2021-11-25
A-11	ALL (bioreactor OR chemostat OR reactor OR shime OR simulator OR simulation OR dynamic OR model OR vitro) AND ALL (gut OR intestine OR gastrointestinal) AND ALL (microbiota OR microbiome OR microbial) AND human AND TITLE-ABS (”in vitro model”) AND (LIMIT-TO (DOCTYPE , ”ar”)) AND (LIMIT-TO (LANGUAGE , ”English”)) AND (EXCLUDE (EXACTKEYWORD , ”Nonhuman”) OR EXCLUDE (EXACTKEYWORD , ”Animals”) OR EXCLUDE (EXACTKEYWORD , ”Animal”) OR EXCLUDE (EXACTKEYWORD , ”Animal Experiment”) OR EXCLUDE (EXACTKEYWORD , ”Animal Model”) OR EXCLUDE (EXACTKEYWORD , ”Animal Tissue”) OR EXCLUDE (EXACTKEYWORD , ”Mice, Inbred C57BL”) OR EXCLUDE (EXACTKEYWORD , ”Rat”) OR EXCLUDE (EXACTKEYWORD , ”Disease Models, Animal”) OR EXCLUDE (EXACTKEYWORD , ”Rats”) OR EXCLUDE (EXACTKEYWORD , ”In Vivo Study”) OR EXCLUDE (EXACTKEYWORD , ”Animalia”) OR EXCLUDE (EXACTKEYWORD , ”Swine”) OR EXCLUDE (EXACTKEYWORD , ”Mice, Knockout”))	452	2021-11-25
A-12	ALL (bioreactor OR chemostat OR reactor OR shime OR simulator OR simulation OR dynamic OR model OR vitro) AND ALL (gut OR intestine OR gastrointestinal) AND ALL (microbiota OR microbiome OR microbial) AND human AND TITLE-ABS (”reactor”) AND (LIMIT-TO (DOCTYPE , ”ar”)) AND (LIMIT-TO (LANGUAGE , ”English”)) AND (EXCLUDE (EXACTKEYWORD , ”Nonhuman”) OR EXCLUDE (EXACTKEYWORD , ”Animals”) OR EXCLUDE (EXACTKEYWORD , ”Animal”) OR EXCLUDE (EXACTKEYWORD , ”Animal Experiment”) OR EXCLUDE (EXACTKEYWORD , ”Animal Model”) OR EXCLUDE (EXACTKEYWORD , ”Animal Tissue”) OR EXCLUDE (EXACTKEYWORD , ”Mice, Inbred C57BL”) OR EXCLUDE (EXACTKEYWORD , ”Rat”) OR EXCLUDE (EXACTKEYWORD , ”Disease Models, Animal”) OR EXCLUDE (EXACTKEYWORD , ”Rats”) OR EXCLUDE (EXACTKEYWORD , ”In Vivo Study”) OR EXCLUDE (EXACTKEYWORD , ”Animalia”) OR EXCLUDE (EXACTKEYWORD , ”Swine”) OR EXCLUDE (EXACTKEYWORD , ”Mice, Knockout”)) ...View More	316	2021-11-25

A-13	ALL (bioreactor OR chemostat OR reactor OR shime OR simulator OR simulation OR dynamic OR model OR vitro) AND ALL (gut OR intestine OR gastrointestinal) AND ALL (microbiota OR microbiome OR microbial) AND human AND TITLE-ABS (”shime”) AND (LIMIT-TO (DOCTYPE , ”ar”)) AND (LIMIT-TO (LANGUAGE , ”English”)) AND (EXCLUDE (EXACTKEYWORD , ”Nonhuman”) OR EXCLUDE (EXACTKEYWORD , ”Animals”) OR EXCLUDE (EXACTKEYWORD , ”Animal”) OR EXCLUDE (EXACTKEYWORD , ”Animal Experiment”) OR EXCLUDE (EXACTKEYWORD , ”Animal Model”) OR EXCLUDE (EXACTKEYWORD , ”Animal Tissue”) OR EXCLUDE (EXACTKEYWORD , ”Mice, Inbred C57BL”) OR EXCLUDE (EXACTKEYWORD , ”Rat”) OR EXCLUDE (EXACTKEYWORD , ”Disease Models, Animal”) OR EXCLUDE (EXACTKEYWORD , ”Rats”) OR EXCLUDE (EXACTKEYWORD , ”In Vivo Study”) OR EXCLUDE (EXACTKEYWORD , ”Animalia”) OR EXCLUDE (EXACTKEYWORD , ”Swine”) OR EXCLUDE (EXACTKEYWORD , ”Mice, Knockout”))	95	2021-11-25
A-14	ALL (bioreactor OR chemostat OR reactor OR shime OR simulator OR simulation OR dynamic OR model OR vitro) AND ALL (gut OR intestine OR gastrointestinal) AND ALL (microbiota OR microbiome OR microbial) AND human AND TITLE-ABS (”simulated colon”) AND (LIMIT-TO (DOCTYPE , ”ar”)) AND (LIMIT-TO (LANGUAGE , ”English”)) AND (EXCLUDE (EXACTKEYWORD , ”Nonhuman”) OR EXCLUDE (EXACTKEYWORD , ”Animals”) OR EXCLUDE (EXACTKEYWORD , ”Animal”) OR EXCLUDE (EXACTKEYWORD , ”Animal Experiment”) OR EXCLUDE (EXACTKEYWORD , ”Animal Model”) OR EXCLUDE (EXACTKEYWORD , ”Animal Tissue”) OR EXCLUDE (EXACTKEYWORD , ”Mice, Inbred C57BL”) OR EXCLUDE (EXACTKEYWORD , ”Rat”) OR EXCLUDE (EXACTKEYWORD , ”Disease Models, Animal”) OR EXCLUDE (EXACTKEYWORD , ”Rats”) OR EXCLUDE (EXACTKEYWORD , ”In Vivo Study”) OR EXCLUDE (EXACTKEYWORD , ”Animalia”) OR EXCLUDE (EXACTKEYWORD , ”Swine”) OR EXCLUDE (EXACTKEYWORD , ”Mice, Knockout”))	24	2021-11-25
A-15	ALL (bioreactor OR chemostat OR reactor OR shime OR simulator OR simulation OR dynamic OR model OR vitro) AND ALL (gut OR intestine OR gastrointestinal) AND ALL (microbiota OR microbiome OR microbial) AND human AND TITLE-ABS (”simulated colonic”) AND (LIMIT-TO (DOCTYPE , ”ar”)) AND (LIMIT-TO (LANGUAGE , ”English”)) AND (EXCLUDE (EXACTKEYWORD , ”Nonhuman”) OR EXCLUDE (EXACTKEYWORD , ”Animals”) OR EXCLUDE (EXACTKEYWORD , ”Animal”) OR EXCLUDE (EXACTKEYWORD , ”Animal Experiment”) OR EXCLUDE (EXACTKEYWORD , ”Animal Model”) OR EXCLUDE (EXACTKEYWORD , ”Animal Tissue”) OR EXCLUDE (EXACTKEYWORD , ”Mice, Inbred C57BL”) OR EXCLUDE (EXACTKEYWORD , ”Rat”) OR EXCLUDE (EXACTKEYWORD , ”Disease Models, Animal”) OR EXCLUDE (EXACTKEYWORD , ”Rats”) OR EXCLUDE (EXACTKEYWORD , ”In Vivo Study”) OR EXCLUDE (EXACTKEYWORD , ”Animalia”) OR EXCLUDE (EXACTKEYWORD , ”Swine”) OR EXCLUDE (EXACTKEYWORD , ”Mice, Knockout”))	31	2021-11-25

A-16	ALL (bioreactor OR chemostat OR reactor OR shime OR simulator OR simulation OR dynamic OR model OR vitro) AND ALL (gut OR intestine OR gastrointestinal) AND ALL (microbiota OR microbiome OR microbial) AND human AND TITLE-ABS (”simulated gastrointestinal”) AND (LIMIT-TO (DOCTYPE , ”ar”)) AND (LIMIT-TO (LANGUAGE , ”English”)) AND (EXCLUDE (EXACTKEYWORD , ”Nonhuman”) OR EXCLUDE (EXACTKEYWORD , ”Animals”) OR EXCLUDE (EXACTKEYWORD , ”Animal”) OR EXCLUDE (EXACTKEYWORD , ”Animal Experiment”) OR EXCLUDE (EXACTKEYWORD , ”Animal Model”) OR EXCLUDE (EXACTKEYWORD , ”Animal Tissue”) OR EXCLUDE (EXACTKEYWORD , ”Mice, Inbred C57BL”) OR EXCLUDE (EXACTKEYWORD , ”Rat”) OR EXCLUDE (EXACTKEYWORD , ”Disease Models, Animal”) OR EXCLUDE (EXACTKEYWORD , ”Rats”) OR EXCLUDE (EXACTKEYWORD , ”In Vivo Study”) OR EXCLUDE (EXACTKEYWORD , ”Animalia”) OR EXCLUDE (EXACTKEYWORD , ”Swine”) OR EXCLUDE (EXACTKEYWORD , ”Mice, Knockout”))	490	2021-11-25
A-17	ALL (bioreactor OR chemostat OR reactor OR shime OR simulator OR simulation OR dynamic OR model OR vitro) AND ALL (gut OR intestine OR gastrointestinal) AND ALL (microbiota OR microbiome OR microbial) AND human AND TITLE-ABS (”simulator of human”) AND (LIMIT-TO (DOCTYPE , ”ar”)) AND (LIMIT-TO (LANGUAGE , ”English”)) AND (EXCLUDE (EXACTKEYWORD , ”Nonhuman”) OR EXCLUDE (EXACTKEYWORD , ”Animals”) OR EXCLUDE (EXACTKEYWORD , ”Animal”) OR EXCLUDE (EXACTKEYWORD , ”Animal Experiment”) OR EXCLUDE (EXACTKEYWORD , ”Animal Model”) OR EXCLUDE (EXACTKEYWORD , ”Animal Tissue”) OR EXCLUDE (EXACTKEYWORD , ”Mice, Inbred C57BL”) OR EXCLUDE (EXACTKEYWORD , ”Rat”) OR EXCLUDE (EXACTKEYWORD , ”Disease Models, Animal”) OR EXCLUDE (EXACTKEYWORD , ”Rats”) OR EXCLUDE (EXACTKEYWORD , ”In Vivo Study”) OR EXCLUDE (EXACTKEYWORD , ”Animalia”) OR EXCLUDE (EXACTKEYWORD , ”Swine”) OR EXCLUDE (EXACTKEYWORD , ”Mice, Knockout”))	13	2021-11-25
A-18	ALL (bioreactor OR chemostat OR reactor OR shime OR simulator OR simulation OR dynamic OR model OR vitro) AND ALL (gut OR intestine OR gastrointestinal) AND ALL (microbiota OR microbiome OR microbial) AND human AND TITLE-ABS (”simulator of the human”) AND (LIMIT-TO (DOCTYPE , ”ar”)) AND (LIMIT-TO (LANGUAGE , ”English”)) AND (EXCLUDE (EXACTKEYWORD , ”Nonhuman”) OR EXCLUDE (EXACTKEYWORD , ”Animals”) OR EXCLUDE (EXACTKEYWORD , ”Animal”) OR EXCLUDE (EXACTKEYWORD , ”Animal Experiment”) OR EXCLUDE (EXACTKEYWORD , ”Animal Model”) OR EXCLUDE (EXACTKEYWORD , ”Animal Tissue”) OR EXCLUDE (EXACTKEYWORD , ”Mice, Inbred C57BL”) OR EXCLUDE (EXACTKEYWORD , ”Rat”) OR EXCLUDE (EXACTKEYWORD , ”Disease Models, Animal”) OR EXCLUDE (EXACTKEYWORD , ”Rats”) OR EXCLUDE (EXACTKEYWORD , ”In Vivo Study”) OR EXCLUDE (EXACTKEYWORD , ”Animalia”) OR EXCLUDE (EXACTKEYWORD , ”Swine”) OR EXCLUDE (EXACTKEYWORD , ”Mice, Knockout”))	73	2021-11-25

A-19	ALL (bioreactor OR chemostat OR reactor OR shime OR simulator OR simulation OR dynamic OR model OR vitro) AND ALL (gut OR intestine OR gastrointestinal) AND ALL (microbiota OR microbiome OR microbial) AND human AND TITLE-ABS (bioreactor) AND (LIMIT-TO (SRCTYPE , "j")) AND (LIMIT-TO (DOCTYPE , "ar")) AND (LIMIT-TO (LANGUAGE , "English")) AND (EXCLUDE (EXACTKEYWORD , "Nonhuman") OR EXCLUDE (EXACTKEYWORD , "Animals") OR EXCLUDE (EXACTKEYWORD , "Animal") OR EXCLUDE (EXACTKEYWORD , "Animal Experiment") OR EXCLUDE (EXACTKEYWORD , "Animal Model") OR EXCLUDE (EXACTKEYWORD , "Animal Tissue") OR EXCLUDE (EXACTKEYWORD , "Mice, Inbred C57BL") OR EXCLUDE (EXACTKEYWORD , "Rat") OR EXCLUDE (EXACTKEYWORD , "Disease Models, Animal") OR EXCLUDE (EXACTKEYWORD , "Rats") OR EXCLUDE (EXACTKEYWORD , "In Vivo Study") OR EXCLUDE (EXACTKEYWORD , "Animalia") OR EXCLUDE (EXACTKEYWORD , "Swine") OR EXCLUDE (EXACTKEYWORD , "Mice, Knockout"))	229	2021-11-25
A-20	ALL (bioreactor OR chemostat OR reactor OR shime OR simulator OR simulation OR dynamic OR model OR vitro) AND ALL (gut OR intestine OR gastrointestinal) AND ALL (microbiota OR microbiome OR microbial) AND human AND TITLE-ABS (reactor) AND (LIMIT-TO (DOCTYPE , "ar")) AND (LIMIT-TO (LANGUAGE , "English")) AND (EXCLUDE (EXACTKEYWORD , "Nonhuman") OR EXCLUDE (EXACTKEYWORD , "Animals") OR EXCLUDE (EXACTKEYWORD , "Animal") OR EXCLUDE (EXACTKEYWORD , "Animal Experiment") OR EXCLUDE (EXACTKEYWORD , "Animal Model") OR EXCLUDE (EXACTKEYWORD , "Animal Tissue") OR EXCLUDE (EXACTKEYWORD , "Mice, Inbred C57BL") OR EXCLUDE (EXACTKEYWORD , "Rat") OR EXCLUDE (EXACTKEYWORD , "Disease Models, Animal") OR EXCLUDE (EXACTKEYWORD , "Rats") OR EXCLUDE (EXACTKEYWORD , "In Vivo Study") OR EXCLUDE (EXACTKEYWORD , "Animalia") OR EXCLUDE (EXACTKEYWORD , "Swine") OR EXCLUDE (EXACTKEYWORD , "Mice, Knockout"))	316	2021-11-25
A-21	ALL (bioreactor OR chemostat OR reactor OR shime OR simulator OR simulation OR dynamic OR model OR vitro) AND ALL (gut OR intestine OR gastrointestinal) AND ALL (microbiota OR microbiome OR microbial) AND human AND TITLE-ABS-KEY (artificial gut) AND (LIMIT-TO (DOCTYPE , "ar")) AND (LIMIT-TO (LANGUAGE , "English")) AND (EXCLUDE (EXACTKEYWORD , "Nonhuman") OR EXCLUDE (EXACTKEYWORD , "Animals") OR EXCLUDE (EXACTKEYWORD , "Animal") OR EXCLUDE (EXACTKEYWORD , "Animal Experiment") OR EXCLUDE (EXACTKEYWORD , "Animal Model") OR EXCLUDE (EXACTKEYWORD , "Animal Tissue") OR EXCLUDE (EXACTKEYWORD , "Mice, Inbred C57BL") OR EXCLUDE (EXACTKEYWORD , "Rat") OR EXCLUDE (EXACTKEYWORD , "Disease Models, Animal") OR EXCLUDE (EXACTKEYWORD , "Rats") OR EXCLUDE (EXACTKEYWORD , "In Vivo Study") OR EXCLUDE (EXACTKEYWORD , "Animalia") OR EXCLUDE (EXACTKEYWORD , "Swine") OR EXCLUDE (EXACTKEYWORD , "Mice, Knockout"))	602	2021-11-25

B-1	ALL (bioreactor OR chemostat OR reactor OR shime OR simulator OR simulation OR dynamic OR model OR vitro) AND ALL (gut OR intestine OR gastrointestinal) AND ALL (microbiota OR microbiome OR microbial) AND human AND TITLE-ABS ("artificial gut") AND fecal AND (LIMIT-TO (DOCTYPE , "ar")) AND (LIMIT-TO (LANGUAGE , "English")) AND (EXCLUDE (EXACTKEYWORD , "Nonhuman") OR EXCLUDE (EXACTKEYWORD , "Animals") OR EXCLUDE (EXACTKEYWORD , "Animal") OR EXCLUDE (EXACTKEYWORD , "Animal Experiment") OR EXCLUDE (EXACTKEYWORD , "Animal Model") OR EXCLUDE (EXACTKEYWORD , "Animal Tissue") OR EXCLUDE (EXACTKEYWORD , "Mice, Inbred C57BL") OR EXCLUDE (EXACTKEYWORD , "Rat") OR EXCLUDE (EXACTKEYWORD , "Disease Models, Animal") OR EXCLUDE (EXACTKEYWORD , "Rats") OR EXCLUDE (EXACTKEYWORD , "In Vivo Study") OR EXCLUDE (EXACTKEYWORD , "Animalia") OR EXCLUDE (EXACTKEYWORD , "Swine") OR EXCLUDE (EXACTKEYWORD , "Mice, Knockout"))	27	2021-11-25
B-2	ALL (bioreactor OR chemostat OR reactor OR shime OR simulator OR simulation OR dynamic OR model OR vitro) AND ALL (gut OR intestine OR gastrointestinal) AND ALL (microbiota OR microbiome OR microbial) AND human AND TITLE-ABS ("Bioreactor") AND fecal AND (LIMIT-TO (DOCTYPE , "ar")) AND (LIMIT-TO (LANGUAGE , "English")) AND (EXCLUDE (EXACTKEYWORD , "Nonhuman") OR EXCLUDE (EXACTKEYWORD , "Animals") OR EXCLUDE (EXACTKEYWORD , "Animal") OR EXCLUDE (EXACTKEYWORD , "Animal Experiment") OR EXCLUDE (EXACTKEYWORD , "Animal Model") OR EXCLUDE (EXACTKEYWORD , "Animal Tissue") OR EXCLUDE (EXACTKEYWORD , "Mice, Inbred C57BL") OR EXCLUDE (EXACTKEYWORD , "Rat") OR EXCLUDE (EXACTKEYWORD , "Disease Models, Animal") OR EXCLUDE (EXACTKEYWORD , "Rats") OR EXCLUDE (EXACTKEYWORD , "In Vivo Study") OR EXCLUDE (EXACTKEYWORD , "Animalia") OR EXCLUDE (EXACTKEYWORD , "Swine") OR EXCLUDE (EXACTKEYWORD , "Mice, Knockout"))	37	2021-11-25
B-3	ALL (bioreactor OR chemostat OR reactor OR shime OR simulator OR simulation OR dynamic OR model OR vitro) AND ALL (gut OR intestine OR gastrointestinal) AND ALL (microbiota OR microbiome OR microbial) AND human AND TITLE-ABS ("Chemostat") AND fecal AND (LIMIT-TO (DOCTYPE , "ar")) AND (LIMIT-TO (LANGUAGE , "English")) AND (EXCLUDE (EXACTKEYWORD , "Nonhuman") OR EXCLUDE (EXACTKEYWORD , "Animals") OR EXCLUDE (EXACTKEYWORD , "Animal") OR EXCLUDE (EXACTKEYWORD , "Animal Experiment") OR EXCLUDE (EXACTKEYWORD , "Animal Model") OR EXCLUDE (EXACTKEYWORD , "Animal Tissue") OR EXCLUDE (EXACTKEYWORD , "Mice, Inbred C57BL") OR EXCLUDE (EXACTKEYWORD , "Rat") OR EXCLUDE (EXACTKEYWORD , "Disease Models, Animal") OR EXCLUDE (EXACTKEYWORD , "Rats") OR EXCLUDE (EXACTKEYWORD , "In Vivo Study") OR EXCLUDE (EXACTKEYWORD , "Animalia") OR EXCLUDE (EXACTKEYWORD , "Swine") OR EXCLUDE (EXACTKEYWORD , "Mice, Knockout"))	19	2021-11-25

B-4	ALL (bioreactor OR chemostat OR reactor OR shime OR simulator OR simulation OR dynamic OR model OR vitro) AND ALL (gut OR intestine OR gastrointestinal) AND ALL (microbiota OR microbiome OR microbial) AND human AND TITLE-ABS (”continuous culture”) AND fecal AND (LIMIT-TO (DOCTYPE , ”ar”)) AND (LIMIT-TO (LANGUAGE , ”English”)) AND (EXCLUDE (EXACTKEYWORD , ”Nonhuman”) OR EXCLUDE (EXACTKEYWORD , ”Animals”) OR EXCLUDE (EXACTKEYWORD , ”Animal”) OR EXCLUDE (EXACTKEYWORD , ”Animal Experiment”) OR EXCLUDE (EXACTKEYWORD , ”Animal Model”) OR EXCLUDE (EXACTKEYWORD , ”Animal Tissue”) OR EXCLUDE (EXACTKEYWORD , ”Mice, Inbred C57BL”) OR EXCLUDE (EXACTKEYWORD , ”Rat”) OR EXCLUDE (EXACTKEYWORD , ”Disease Models, Animal”) OR EXCLUDE (EXACTKEYWORD , ”Rats”) OR EXCLUDE (EXACTKEYWORD , ”In Vivo Study”) OR EXCLUDE (EXACTKEYWORD , ”Animalia”) OR EXCLUDE (EXACTKEYWORD , ”Swine”) OR EXCLUDE (EXACTKEYWORD , ”Mice, Knockout”))	26	2021-11-25
B-5	ALL (bioreactor OR chemostat OR reactor OR shime OR simulator OR simulation OR dynamic OR model OR vitro) AND ALL (gut OR intestine OR gastrointestinal) AND ALL (microbiota OR microbiome OR microbial) AND human AND TITLE-ABS (”continuous fermentation”) AND fecal AND (LIMIT-TO (DOCTYPE , ”ar”)) AND (LIMIT-TO (LANGUAGE , ”English”)) AND (EXCLUDE (EXACTKEYWORD , ”Nonhuman”) OR EXCLUDE (EXACTKEYWORD , ”Animals”) OR EXCLUDE (EXACTKEYWORD , ”Animal”) OR EXCLUDE (EXACTKEYWORD , ”Animal Experiment”) OR EXCLUDE (EXACTKEYWORD , ”Animal Model”) OR EXCLUDE (EXACTKEYWORD , ”Animal Tissue”) OR EXCLUDE (EXACTKEYWORD , ”Mice, Inbred C57BL”) OR EXCLUDE (EXACTKEYWORD , ”Rat”) OR EXCLUDE (EXACTKEYWORD , ”Disease Models, Animal”) OR EXCLUDE (EXACTKEYWORD , ”Rats”) OR EXCLUDE (EXACTKEYWORD , ”In Vivo Study”) OR EXCLUDE (EXACTKEYWORD , ”Animalia”) OR EXCLUDE (EXACTKEYWORD , ”Swine”) OR EXCLUDE (EXACTKEYWORD , ”Mice, Knockout”))	4	2021-11-25
B-6	ALL (bioreactor OR chemostat OR reactor OR shime OR simulator OR simulation OR dynamic OR model OR vitro) AND ALL (gut OR intestine OR gastrointestinal) AND ALL (microbiota OR microbiome OR microbial) AND human AND TITLE-ABS (”fermenter”) AND fecal AND (LIMIT-TO (DOCTYPE , ”ar”)) AND (LIMIT-TO (LANGUAGE , ”English”)) AND (EXCLUDE (EXACTKEYWORD , ”Nonhuman”) OR EXCLUDE (EXACTKEYWORD , ”Animals”) OR EXCLUDE (EXACTKEYWORD , ”Animal”) OR EXCLUDE (EXACTKEYWORD , ”Animal Experiment”) OR EXCLUDE (EXACTKEYWORD , ”Animal Model”) OR EXCLUDE (EXACTKEYWORD , ”Animal Tissue”) OR EXCLUDE (EXACTKEYWORD , ”Mice, Inbred C57BL”) OR EXCLUDE (EXACTKEYWORD , ”Rat”) OR EXCLUDE (EXACTKEYWORD , ”Disease Models, Animal”) OR EXCLUDE (EXACTKEYWORD , ”Rats”) OR EXCLUDE (EXACTKEYWORD , ”In Vivo Study”) OR EXCLUDE (EXACTKEYWORD , ”Animalia”) OR EXCLUDE (EXACTKEYWORD , ”Swine”) OR EXCLUDE (EXACTKEYWORD , ”Mice, Knockout”))	23	2021-11-25

B-7	ALL (bioreactor OR chemostat OR reactor OR shime OR simulator OR simulation OR dynamic OR model OR vitro) AND ALL (gut OR intestine OR gastrointestinal) AND ALL (microbiota OR microbiome OR microbial) AND human AND TITLE-ABS (”gastrointestinal model”) AND fecal AND (LIMIT-TO (DOCTYPE , ”ar”)) AND (LIMIT-TO (LANGUAGE , ”English”)) AND (EXCLUDE (EXACTKEYWORD , ”Nonhuman”) OR EXCLUDE (EXACTKEYWORD , ”Animals”) OR EXCLUDE (EXACTKEYWORD , ”Animal”) OR EXCLUDE (EXACTKEYWORD , ”Animal Experiment”) OR EXCLUDE (EXACTKEYWORD , ”Animal Model”) OR EXCLUDE (EXACTKEYWORD , ”Animal Tissue”) OR EXCLUDE (EXACTKEYWORD , ”Mice, Inbred C57BL”) OR EXCLUDE (EXACTKEYWORD , ”Rat”) OR EXCLUDE (EXACTKEYWORD , ”Disease Models, Animal”) OR EXCLUDE (EXACTKEYWORD , ”Rats”) OR EXCLUDE (EXACTKEYWORD , ”In Vivo Study”) OR EXCLUDE (EXACTKEYWORD , ”Animalia”) OR EXCLUDE (EXACTKEYWORD , ”Swine”) OR EXCLUDE (EXACTKEYWORD , ”Mice, Knockout”))	12	2021-11-25
B-8	ALL (bioreactor OR chemostat OR reactor OR shime OR simulator OR simulation OR dynamic OR model OR vitro) AND ALL (gut OR intestine OR gastrointestinal) AND ALL (microbiota OR microbiome OR microbial) AND human AND TITLE-ABS (”git model”) AND fecal AND (LIMIT-TO (DOCTYPE , ”ar”)) AND (LIMIT-TO (LANGUAGE , ”English”)) AND (EXCLUDE (EXACTKEYWORD , ”Nonhuman”) OR EXCLUDE (EXACTKEYWORD , ”Animals”) OR EXCLUDE (EXACTKEYWORD , ”Animal”) OR EXCLUDE (EXACTKEYWORD , ”Animal Experiment”) OR EXCLUDE (EXACTKEYWORD , ”Animal Model”) OR EXCLUDE (EXACTKEYWORD , ”Animal Tissue”) OR EXCLUDE (EXACTKEYWORD , ”Mice, Inbred C57BL”) OR EXCLUDE (EXACTKEYWORD , ”Rat”) OR EXCLUDE (EXACTKEYWORD , ”Disease Models, Animal”) OR EXCLUDE (EXACTKEYWORD , ”Rats”) OR EXCLUDE (EXACTKEYWORD , ”In Vivo Study”) OR EXCLUDE (EXACTKEYWORD , ”Animalia”) OR EXCLUDE (EXACTKEYWORD , ”Swine”) OR EXCLUDE (EXACTKEYWORD , ”Mice, Knockout”))	1	2021-11-25
B-9	ALL (bioreactor OR chemostat OR reactor OR shime OR simulator OR simulation OR dynamic OR model OR vitro) AND ALL (gut OR intestine OR gastrointestinal) AND ALL (microbiota OR microbiome OR microbial) AND human AND TITLE-ABS (”gut model”) AND fecal AND (LIMIT-TO (DOCTYPE , ”ar”)) AND (LIMIT-TO (LANGUAGE , ”English”)) AND (EXCLUDE (EXACTKEYWORD , ”Nonhuman”) OR EXCLUDE (EXACTKEYWORD , ”Animals”) OR EXCLUDE (EXACTKEYWORD , ”Animal”) OR EXCLUDE (EXACTKEYWORD , ”Animal Experiment”) OR EXCLUDE (EXACTKEYWORD , ”Animal Model”) OR EXCLUDE (EXACTKEYWORD , ”Animal Tissue”) OR EXCLUDE (EXACTKEYWORD , ”Mice, Inbred C57BL”) OR EXCLUDE (EXACTKEYWORD , ”Rat”) OR EXCLUDE (EXACTKEYWORD , ”Disease Models, Animal”) OR EXCLUDE (EXACTKEYWORD , ”Rats”) OR EXCLUDE (EXACTKEYWORD , ”In Vivo Study”) OR EXCLUDE (EXACTKEYWORD , ”Animalia”) OR EXCLUDE (EXACTKEYWORD , ”Swine”) OR EXCLUDE (EXACTKEYWORD , ”Mice, Knockout”))	39	2021-11-25

B-10	ALL (bioreactor OR chemostat OR reactor OR shime OR simulator OR simulation OR dynamic OR model OR vitro) AND ALL (gut OR intestine OR gastrointestinal) AND ALL (microbiota OR microbiome OR microbial) AND human AND TITLE-ABS (”gut simulation”) AND fecal AND (LIMIT-TO (DOCTYPE , ”ar”)) AND (LIMIT-TO (LANGUAGE , ”English”)) AND (EXCLUDE (EXACTKEYWORD , ”Nonhuman”) OR EXCLUDE (EXACTKEYWORD , ”Animals”) OR EXCLUDE (EXACTKEYWORD , ”Animal”) OR EXCLUDE (EXACTKEYWORD , ”Animal Experiment”) OR EXCLUDE (EXACTKEYWORD , ”Animal Model”) OR EXCLUDE (EXACTKEYWORD , ”Animal Tissue”) OR EXCLUDE (EXACTKEYWORD , ”Mice, Inbred C57BL”) OR EXCLUDE (EXACTKEYWORD , ”Rat”) OR EXCLUDE (EXACTKEYWORD , ”Disease Models, Animal”) OR EXCLUDE (EXACTKEYWORD , ”Rats”) OR EXCLUDE (EXACTKEYWORD , ”In Vivo Study”) OR EXCLUDE (EXACTKEYWORD , ”Animalia”) OR EXCLUDE (EXACTKEYWORD , ”Swine”) OR EXCLUDE (EXACTKEYWORD , ”Mice, Knockout”))	1	2021-11-25
B-11	ALL (bioreactor OR chemostat OR reactor OR shime OR simulator OR simulation OR dynamic OR model OR vitro) AND ALL (gut OR intestine OR gastrointestinal) AND ALL (microbiota OR microbiome OR microbial) AND human AND TITLE-ABS (”in vitro colon”) AND fecal AND (LIMIT-TO (DOCTYPE , ”ar”)) AND (LIMIT-TO (LANGUAGE , ”English”)) AND (EXCLUDE (EXACTKEYWORD , ”Nonhuman”) OR EXCLUDE (EXACTKEYWORD , ”Animals”) OR EXCLUDE (EXACTKEYWORD , ”Animal”) OR EXCLUDE (EXACTKEYWORD , ”Animal Experiment”) OR EXCLUDE (EXACTKEYWORD , ”Animal Model”) OR EXCLUDE (EXACTKEYWORD , ”Animal Tissue”) OR EXCLUDE (EXACTKEYWORD , ”Mice, Inbred C57BL”) OR EXCLUDE (EXACTKEYWORD , ”Rat”) OR EXCLUDE (EXACTKEYWORD , ”Disease Models, Animal”) OR EXCLUDE (EXACTKEYWORD , ”Rats”) OR EXCLUDE (EXACTKEYWORD , ”In Vivo Study”) OR EXCLUDE (EXACTKEYWORD , ”Animalia”) OR EXCLUDE (EXACTKEYWORD , ”Swine”) OR EXCLUDE (EXACTKEYWORD , ”Mice, Knockout”))	24	2021-11-25
B-12	ALL (bioreactor OR chemostat OR reactor OR shime OR simulator OR simulation OR dynamic OR model OR vitro) AND ALL (gut OR intestine OR gastrointestinal) AND ALL (microbiota OR microbiome OR microbial) AND human AND TITLE-ABS (”in vitro digestion”) AND fecal AND (LIMIT-TO (DOCTYPE , ”ar”)) AND (LIMIT-TO (LANGUAGE , ”English”)) AND (EXCLUDE (EXACTKEYWORD , ”Nonhuman”) OR EXCLUDE (EXACTKEYWORD , ”Animals”) OR EXCLUDE (EXACTKEYWORD , ”Animal”) OR EXCLUDE (EXACTKEYWORD , ”Animal Experiment”) OR EXCLUDE (EXACTKEYWORD , ”Animal Model”) OR EXCLUDE (EXACTKEYWORD , ”Animal Tissue”) OR EXCLUDE (EXACTKEYWORD , ”Mice, Inbred C57BL”) OR EXCLUDE (EXACTKEYWORD , ”Rat”) OR EXCLUDE (EXACTKEYWORD , ”Disease Models, Animal”) OR EXCLUDE (EXACTKEYWORD , ”Rats”) OR EXCLUDE (EXACTKEYWORD , ”In Vivo Study”) OR EXCLUDE (EXACTKEYWORD , ”Animalia”) OR EXCLUDE (EXACTKEYWORD , ”Swine”) OR EXCLUDE (EXACTKEYWORD , ”Mice, Knockout”))	103	2021-11-25

B-13	ALL (bioreactor OR chemostat OR reactor OR shime OR simulator OR simulation OR dynamic OR model OR vitro) AND ALL (gut OR intestine OR gastrointestinal) AND ALL (microbiota OR microbiome OR microbial) AND human AND TITLE-ABS (”in vitro gastrointestinal”) AND fecal AND (LIMIT-TO (DOCTYPE , ”ar”)) AND (LIMIT-TO (LANGUAGE , ”English”)) AND (EXCLUDE (EXACTKEYWORD , ”Nonhuman”) OR EXCLUDE (EXACTKEYWORD , ”Animals”) OR EXCLUDE (EXACTKEYWORD , ”Animal”) OR EXCLUDE (EXACTKEYWORD , ”Animal Experiment”) OR EXCLUDE (EXACTKEYWORD , ”Animal Model”) OR EXCLUDE (EXACTKEYWORD , ”Animal Tissue”) OR EXCLUDE (EXACTKEYWORD , ”Mice, Inbred C57BL”) OR EXCLUDE (EXACTKEYWORD , ”Rat”) OR EXCLUDE (EXACTKEYWORD , ”Disease Models, Animal”) OR EXCLUDE (EXACTKEYWORD , ”Rats”) OR EXCLUDE (EXACTKEYWORD , ”In Vivo Study”) OR EXCLUDE (EXACTKEYWORD , ”Animalia”) OR EXCLUDE (EXACTKEYWORD , ”Swine”) OR EXCLUDE (EXACTKEYWORD , ”Mice, Knockout”))	49	2021-11-25
B-14	ALL (bioreactor OR chemostat OR reactor OR shime OR simulator OR simulation OR dynamic OR model OR vitro) AND ALL (gut OR intestine OR gastrointestinal) AND ALL (microbiota OR microbiome OR microbial) AND human AND TITLE-ABS (”in vitro model”) AND fecal AND (LIMIT-TO (DOCTYPE , ”ar”)) AND (LIMIT-TO (LANGUAGE , ”English”)) AND (EXCLUDE (EXACTKEYWORD , ”Nonhuman”) OR EXCLUDE (EXACTKEYWORD , ”Animals”) OR EXCLUDE (EXACTKEYWORD , ”Animal”) OR EXCLUDE (EXACTKEYWORD , ”Animal Experiment”) OR EXCLUDE (EXACTKEYWORD , ”Animal Model”) OR EXCLUDE (EXACTKEYWORD , ”Animal Tissue”) OR EXCLUDE (EXACTKEYWORD , ”Mice, Inbred C57BL”) OR EXCLUDE (EXACTKEYWORD , ”Rat”) OR EXCLUDE (EXACTKEYWORD , ”Disease Models, Animal”) OR EXCLUDE (EXACTKEYWORD , ”Rats”) OR EXCLUDE (EXACTKEYWORD , ”In Vivo Study”) OR EXCLUDE (EXACTKEYWORD , ”Animalia”) OR EXCLUDE (EXACTKEYWORD , ”Swine”) OR EXCLUDE (EXACTKEYWORD , ”Mice, Knockout”))	110	2021-11-25
B-15	ALL (bioreactor OR chemostat OR reactor OR shime OR simulator OR simulation OR dynamic OR model OR vitro) AND ALL (gut OR intestine OR gastrointestinal) AND ALL (microbiota OR microbiome OR microbial) AND human AND TITLE-ABS (”reactor”) AND fecal AND (LIMIT-TO (DOCTYPE , ”ar”)) AND (LIMIT-TO (LANGUAGE , ”English”)) AND (EXCLUDE (EXACTKEYWORD , ”Nonhuman”) OR EXCLUDE (EXACTKEYWORD , ”Animals”) OR EXCLUDE (EXACTKEYWORD , ”Animal”) OR EXCLUDE (EXACTKEYWORD , ”Animal Experiment”) OR EXCLUDE (EXACTKEYWORD , ”Animal Model”) OR EXCLUDE (EXACTKEYWORD , ”Animal Tissue”) OR EXCLUDE (EXACTKEYWORD , ”Mice, Inbred C57BL”) OR EXCLUDE (EXACTKEYWORD , ”Rat”) OR EXCLUDE (EXACTKEYWORD , ”Disease Models, Animal”) OR EXCLUDE (EXACTKEYWORD , ”Rats”) OR EXCLUDE (EXACTKEYWORD , ”In Vivo Study”) OR EXCLUDE (EXACTKEYWORD , ”Animalia”) OR EXCLUDE (EXACTKEYWORD , ”Swine”) OR EXCLUDE (EXACTKEYWORD , ”Mice, Knockout”)) ...View More	62	2021-11-25

B-16	ALL (bioreactor OR chemostat OR reactor OR shime OR simulator OR simulation OR dynamic OR model OR vitro) AND ALL (gut OR intestine OR gastrointestinal) AND ALL (microbiota OR microbiome OR microbial) AND human AND TITLE-ABS (”shime”) AND fecal AND (LIMIT-TO (DOCTYPE , ”ar”)) AND (LIMIT-TO (LANGUAGE , ”English”)) AND (EXCLUDE (EXACTKEYWORD , ”Nonhuman”) OR EXCLUDE (EXACTKEYWORD , ”Animals”) OR EXCLUDE (EXACTKEYWORD , ”Animal”) OR EXCLUDE (EXACTKEYWORD , ”Animal Experiment”) OR EXCLUDE (EXACTKEYWORD , ”Animal Model”) OR EXCLUDE (EXACTKEYWORD , ”Animal Tissue”) OR EXCLUDE (EXACTKEYWORD , ”Mice, Inbred C57BL”) OR EXCLUDE (EXACTKEYWORD , ”Rat”) OR EXCLUDE (EXACTKEYWORD , ”Disease Models, Animal”) OR EXCLUDE (EXACTKEYWORD , ”Rats”) OR EXCLUDE (EXACTKEYWORD , ”In Vivo Study”) OR EXCLUDE (EXACTKEYWORD , ”Animalia”) OR EXCLUDE (EXACTKEYWORD , ”Swine”) OR EXCLUDE (EXACTKEYWORD , ”Mice, Knockout”))	48	2021-11-25
B-17	ALL (bioreactor OR chemostat OR reactor OR shime OR simulator OR simulation OR dynamic OR model OR vitro) AND ALL (gut OR intestine OR gastrointestinal) AND ALL (microbiota OR microbiome OR microbial) AND human AND TITLE-ABS (”simulated colon”) AND fecal AND (LIMIT-TO (DOCTYPE , ”ar”)) AND (LIMIT-TO (LANGUAGE , ”English”)) AND (EXCLUDE (EXACTKEYWORD , ”Nonhuman”) OR EXCLUDE (EXACTKEYWORD , ”Animals”) OR EXCLUDE (EXACTKEYWORD , ”Animal”) OR EXCLUDE (EXACTKEYWORD , ”Animal Experiment”) OR EXCLUDE (EXACTKEYWORD , ”Animal Model”) OR EXCLUDE (EXACTKEYWORD , ”Animal Tissue”) OR EXCLUDE (EXACTKEYWORD , ”Mice, Inbred C57BL”) OR EXCLUDE (EXACTKEYWORD , ”Rat”) OR EXCLUDE (EXACTKEYWORD , ”Disease Models, Animal”) OR EXCLUDE (EXACTKEYWORD , ”Rats”) OR EXCLUDE (EXACTKEYWORD , ”In Vivo Study”) OR EXCLUDE (EXACTKEYWORD , ”Animalia”) OR EXCLUDE (EXACTKEYWORD , ”Swine”) OR EXCLUDE (EXACTKEYWORD , ”Mice, Knockout”))	12	2021-11-25
B-18	ALL (bioreactor OR chemostat OR reactor OR shime OR simulator OR simulation OR dynamic OR model OR vitro) AND ALL (gut OR intestine OR gastrointestinal) AND ALL (microbiota OR microbiome OR microbial) AND human AND TITLE-ABS (”simulated colonic”) AND fecal AND (LIMIT-TO (DOCTYPE , ”ar”)) AND (LIMIT-TO (LANGUAGE , ”English”)) AND (EXCLUDE (EXACTKEYWORD , ”Nonhuman”) OR EXCLUDE (EXACTKEYWORD , ”Animals”) OR EXCLUDE (EXACTKEYWORD , ”Animal”) OR EXCLUDE (EXACTKEYWORD , ”Animal Experiment”) OR EXCLUDE (EXACTKEYWORD , ”Animal Model”) OR EXCLUDE (EXACTKEYWORD , ”Animal Tissue”) OR EXCLUDE (EXACTKEYWORD , ”Mice, Inbred C57BL”) OR EXCLUDE (EXACTKEYWORD , ”Rat”) OR EXCLUDE (EXACTKEYWORD , ”Disease Models, Animal”) OR EXCLUDE (EXACTKEYWORD , ”Rats”) OR EXCLUDE (EXACTKEYWORD , ”In Vivo Study”) OR EXCLUDE (EXACTKEYWORD , ”Animalia”) OR EXCLUDE (EXACTKEYWORD , ”Swine”) OR EXCLUDE (EXACTKEYWORD , ”Mice, Knockout”))	15	2021-11-25

B-19	ALL (bioreactor OR chemostat OR reactor OR shime OR simulator OR simulation OR dynamic OR model OR vitro) AND ALL (gut OR intestine OR gastrointestinal) AND ALL (microbiota OR microbiome OR microbial) AND human AND TITLE-ABS (”simulated gastrointestinal”) AND fecal AND (LIMIT-TO (DOCTYPE , ”ar”)) AND (LIMIT-TO (LANGUAGE , ”English”)) AND (EXCLUDE (EXACTKEYWORD , ”Nonhuman”) OR EXCLUDE (EXACTKEYWORD , ”Animals”) OR EXCLUDE (EXACTKEYWORD , ”Animal”) OR EXCLUDE (EXACTKEYWORD , ”Animal Experiment”) OR EXCLUDE (EXACTKEYWORD , ”Animal Model”) OR EXCLUDE (EXACTKEYWORD , ”Animal Tissue”) OR EXCLUDE (EXACTKEYWORD , ”Mice, Inbred C57BL”) OR EXCLUDE (EXACTKEYWORD , ”Rat”) OR EXCLUDE (EXACTKEYWORD , ”Disease Models, Animal”) OR EXCLUDE (EXACTKEYWORD , ”Rats”) OR EXCLUDE (EXACTKEYWORD , ”In Vivo Study”) OR EXCLUDE (EXACTKEYWORD , ”Animalia”) OR EXCLUDE (EXACTKEYWORD , ”Swine”) OR EXCLUDE (EXACTKEYWORD , ”Mice, Knockout”))	89	2021-11-25
B-20	ALL (bioreactor OR chemostat OR reactor OR shime OR simulator OR simulation OR dynamic OR model OR vitro) AND ALL (gut OR intestine OR gastrointestinal) AND ALL (microbiota OR microbiome OR microbial) AND human AND TITLE-ABS (”simulator of human”) AND fecal AND (LIMIT-TO (DOCTYPE , ”ar”)) AND (LIMIT-TO (LANGUAGE , ”English”)) AND (EXCLUDE (EXACTKEYWORD , ”Nonhuman”) OR EXCLUDE (EXACTKEYWORD , ”Animals”) OR EXCLUDE (EXACTKEYWORD , ”Animal”) OR EXCLUDE (EXACTKEYWORD , ”Animal Experiment”) OR EXCLUDE (EXACTKEYWORD , ”Animal Model”) OR EXCLUDE (EXACTKEYWORD , ”Animal Tissue”) OR EXCLUDE (EXACTKEYWORD , ”Mice, Inbred C57BL”) OR EXCLUDE (EXACTKEYWORD , ”Rat”) OR EXCLUDE (EXACTKEYWORD , ”Disease Models, Animal”) OR EXCLUDE (EXACTKEYWORD , ”Rats”) OR EXCLUDE (EXACTKEYWORD , ”In Vivo Study”) OR EXCLUDE (EXACTKEYWORD , ”Animalia”) OR EXCLUDE (EXACTKEYWORD , ”Swine”) OR EXCLUDE (EXACTKEYWORD , ”Mice, Knockout”))	7	2021-11-25
B-21	ALL (bioreactor OR chemostat OR reactor OR shime OR simulator OR simulation OR dynamic OR model OR vitro) AND ALL (gut OR intestine OR gastrointestinal) AND ALL (microbiota OR microbiome OR microbial) AND human AND TITLE-ABS (”simulator of the human”) AND fecal AND (LIMIT-TO (DOCTYPE , ”ar”)) AND (LIMIT-TO (LANGUAGE , ”English”)) AND (EXCLUDE (EXACTKEYWORD , ”Nonhuman”) OR EXCLUDE (EXACTKEYWORD , ”Animals”) OR EXCLUDE (EXACTKEYWORD , ”Animal”) OR EXCLUDE (EXACTKEYWORD , ”Animal Experiment”) OR EXCLUDE (EXACTKEYWORD , ”Animal Model”) OR EXCLUDE (EXACTKEYWORD , ”Animal Tissue”) OR EXCLUDE (EXACTKEYWORD , ”Mice, Inbred C57BL”) OR EXCLUDE (EXACTKEYWORD , ”Rat”) OR EXCLUDE (EXACTKEYWORD , ”Disease Models, Animal”) OR EXCLUDE (EXACTKEYWORD , ”Rats”) OR EXCLUDE (EXACTKEYWORD , ”In Vivo Study”) OR EXCLUDE (EXACTKEYWORD , ”Animalia”) OR EXCLUDE (EXACTKEYWORD , ”Swine”) OR EXCLUDE (EXACTKEYWORD , ”Mice, Knockout”))	38	2021-11-25

* Searches conducted with and without the word ’fecal’ are noted in the ’Search number’ column where (A) represents with FECAL in title (B) represents without FECAL in title

Supplementary Material Section 2

Group of terms from preliminary searches

Table S4: Frequency of key terms generated from searches grouped by year.

Year	id	keyword	occurrences	total link strength
**1982-2000**				
	1	acetic acid	1	28
	2	acetic acids	1	28
	3	ammonia	1	22
	4	anaerobic bacterium	1	22
	5	animal	1	25
	6	arabinogalactan	1	22
	7	article	4	97
	8	bacteria	2	50
	9	bacteria (microorganisms)	2	35
	10	bacteria, anaerobic	1	22
	11	bacterial growth	1	21
	12	bacterial proteins	1	22
	13	bacteriological techniques	1	22
	14	bacterium	2	42
	15	bacteroides fragilis	1	21
	16	bacteroides ovatus	2	35
	17	bacteroides thetaiotaomicron	1	21
	18	beta vulgaris subsp. vulgaris	1	25
	19	bifidobacterium	1	21
	20	bifidobacterium adolescentis	1	14
	21	bifidobacterium bifidum	1	21
	22	biological model	1	14
	23	biosynthesis	1	22
	24	bran	1	25
	25	butyric acid	1	28
	26	butyric acids	1	28
	27	carbohydrate	1	25
	28	carbohydrate analysis	1	25
	29	carbohydrates	1	22
	30	cellulose	1	25
	31	chemical composition	1	28
	32	clostridia	1	2
	33	clostridium	1	21
	34	colon	2	35
	35	comparative study	1	25
	36	conference paper	1	14
	37	culture media	1	22
	38	degradation	1	28
	39	dextrin	1	22
	40	dietary carbohydrates	1	28
	41	dietary fiber	2	53
	42	dietary fibre	1	28
	43	ecology	1	22
	44	ecosystem	1	22
	45	energy metabolism	1	22
	46	enterobacteriaceae	1	21
	47	equipment design	1	22

48	eubacterium	1	21
49	europe	1	25
50	euryarchaeota	1	22
51	fatty acid	1	25
52	fatty acids, volatile	4	97
53	feces	2	47
54	feces analysis	1	28
55	feces microflora	1	14
56	fermentation	5	99
57	fungi	1	21
58	fungus	1	21
59	gases	1	22
60	gastrointestinal microbiology	1	1
61	glycine max	1	25
62	growth, development and aging	1	22
63	human	6	132
64	human tissue	1	28
65	humans	1	22
66	in vitro	1	25
67	in vitro simulation	1	1
68	in vitro study	1	14
69	intestine flora	1	22
70	intestines	1	22
71	lactobacillus	1	21
72	lactobacillus bifidus	1	21
73	lactulose	1	2
74	maize	1	25
75	metabolism	1	22
76	methane	1	22
77	methodology	1	14
78	microbiology	1	22
79	microorganism	1	14
80	mucin	1	22
81	mucins	1	22
82	multiple regression	1	28
83	nonhuman	2	49
84	nuclear reactor	1	22
85	ovatus	2	35
86	oxidation reduction reaction	1	22
87	oxidation-reduction	1	22
88	pectin	1	22
89	peptococcus	1	21
90	peptostreptococcus	1	21
91	polysaccharide	1	25
92	polysaccharides	1	25
93	priority journal	2	42
94	propionibacteriaceae	1	21
95	propionibacterium	1	21
96	propionic acid	1	28
97	propionic acids	1	28
98	rats	1	25
99	regression analysis	1	28
100	short chain fatty acid	2	53
101	short-chain fatty acids	1	28
102	soybean	1	25
103	starch	2	47
104	sugars	1	28
105	sulfate	1	22
106	sulfates	1	22
107	sulfide	1	22
108	sulfides	1	22

	109	support, non-u.s. gov't	1	25
	110	uronic acid	1	28
	111	volatile fatty acid	1	22
	112	wheat bran	1	28
	113	xenobiotic metabolism	1	14
	114	xylan	1	22
	115	xylose	1	28
	116	zea mays	1	25
**2001-2005**				
	1	acetic acid	1	20
	2	adult	2	68
	3	aeromonas hydrophila	1	10
	4	alpha-glucosidases	1	24
	5	amino acid metabolism	2	29
	6	amino acids	2	62
	7	anaerobic bacterium	2	68
	8	antibiotic agent	1	20
	9	aromatic amino acid	1	23
	10	article	16	402
	11	autopsy	1	23
	12	avena sativa	1	29
	13	baby food	1	28
	14	bacteria	2	57
	15	bacteria (microorganisms)	9	223
	16	bacteria, anaerobic	2	63
	17	bacterial growth	4	104
	18	bacterial metabolism	5	137
	19	bacteriological techniques	1	29
	20	bacterium culture	2	60
	21	bacterium isolation	1	24
	22	bacteroides	3	83
	23	bacteroides ovatus	1	24
	24	bacteroides thetaiotaomicron	1	39
	25	bacteroides vulgatus	1	39
	26	beta galactosidase	1	20
	27	beta vulgaris subsp. vulgaris	1	34
	28	beta-amylase	1	24
	29	bifidobacteria	1	39
	30	bifidobacterin	1	14
	31	bifidobacterium	3	84
	32	bifidobacterium adolescentis	1	39
	33	bifidobacterium bifidum	2	78
	34	bifidobacterium longum	1	39
	35	bifidobacterium longum bv. infantis	1	39
	36	bifidobacterium pseudolongum	1	39
	37	bifidobacterium saccharolytic	1	39
	38	bifidobacterium sp.	1	39
	39	bile salt	1	20
	40	bioavailability	2	48
	41	biodiversity	1	20
	42	biofilm	1	29

43	biofilms	1	29
44	biological availability	1	28
45	bioreactor	1	12
46	bioreactors	1	20
47	butyric acid	2	48
48	caco-2 cells	1	28
49	cancer cell culture	1	28
50	candida albicans	1	21
51	carbohydrate	1	23
52	carbohydrate analysis	1	39
53	carbohydrate metabolism	4	82
54	carbomer	1	34
55	carbon dioxide	2	49
56	carotene	1	28
57	carotenes	1	28
58	carotenoid	1	28
59	carotenoid bioavailability	1	28
60	carotenoids	1	28
61	carrot	1	28
62	cell strain caco 2	1	28
63	chemistry, pharmaceutical	1	34
64	chemostat	1	21
65	chemotaxonomy	1	39
66	chromatography, gas	1	39
67	cisapride	1	34
68	clinical trial	1	34
69	clostridia	1	39
70	clostridium	3	75
71	clostridium bifermentans	1	39
72	clostridium butyricum	1	39
73	clostridium innocuum	1	39
74	clostridium perfringens	2	45
75	colon	3	96
76	colon cancer	2	59
77	colonic bacterium	1	20
78	colony count, microbial	2	62
79	comparative study	1	29
80	competition	1	21
81	competitive inhibition	1	20
82	computer system	1	16
83	conjugation	1	20
84	controlled clinical trial	1	34
85	controlled drug release	1	34
86	controlled study	5	123
87	culture medium	3	42
88	cytotoxicity	1	20
89	daucus carota	1	28
90	desulfovibrio	1	39
91	desulfovibrio desulfuricans	1	39
92	dextran	1	14
93	dextrin	1	14
94	diet	1	34

95	dietary fiber	1	34
96	digestion	1	28
97	digestive system	3	93
98	drug use	1	34
99	duodenum	1	39
100	ecosystem	4	117
101	enterobacteria	1	20
102	enterobacteriaceae	4	85
103	enterococcus	1	39
104	enterococcus faecalis	2	68
105	enterococcus faecium	1	39
106	enzyme activity	1	39
107	enzyme assay	1	39
108	enzymology	1	29
109	escherichia coli	5	104
110	ethyl cellulose	1	34
111	eubacterium	1	21
112	eubacterium saburreum	1	21
113	fat intake	1	20
114	fatty acid ester	1	39
115	fatty acid metabolism	1	6
116	fatty acid synthesis	1	34
117	fatty acids	1	29
118	fatty acids, volatile	3	102
119	feces	4	110
120	feces microflora	1	34
121	female	1	34
122	fermentation	9	219
123	food intake	1	28
124	formic acids	1	29
125	fructose oligosaccharide	1	12
126	fungus culture	1	21
127	fungus growth	1	21
128	fusobacterium nucleatum	1	21
129	gamma scintigraphy	1	34
130	gastrointestinal agents	1	34
131	gastrointestinal microbiology	1	39
132	gastrointestinal tract function	1	20
133	gastrointestinal transit	2	68
134	gel	1	34
135	glucan 1,4-alpha-glucosidase	1	24
136	glucose metabolism	1	21
137	glycosidase	1	29
138	glycoside hydrolases	2	53
139	gram-positive cocci	1	20
140	growth, development and aging	1	29
141	hemolysis	1	20
142	human	5	139
143	human cell	1	28
144	human experiment	2	68
145	human tissue	1	14
146	humans	10	299
147	hydrogels	1	34
148	hydrogen-ion concentration	1	23

149	hydrolase	1	20
150	hydrolysis	2	44
151	hydrophilic matrix tablets	1	34
152	hydroxypropylmethylcellulose	1	34
153	ileum	1	39
154	in vitro digestion	1	28
155	in vitro models	1	39
156	in vitro simulation	1	10
157	in vitro study	3	77
158	in vitro/in vivo correlation	1	34
159	indole derivative	1	23
160	indoles	1	23
161	infant	1	28
162	infant food	1	28
163	inoculation efficiency	1	10
164	intestinal microbiota	1	10
165	intestinal mucosa	1	29
166	intestine absorption	1	16
167	intestine flora	7	159
168	intestine function	1	16
169	intestine motility	1	16
170	intestine mucosa	1	29
171	intestine transit time	1	34
172	intestine, large	3	76
173	intestines	1	29
174	ionic strength	1	34
175	isoelectric focusing	1	24
176	jejunum	1	39
177	lactic acid	3	88
178	lactic acid bacteria	1	39
179	lactic acid bacterium	2	68
180	lactobacilli	1	10
181	lactobacillus	6	157
182	lactobacillus acidophilus	2	51
183	lactobacillus acidophylus	1	39
184	lactobacillus bifidus	2	78
185	lactobacillus casei	1	21
186	lactobacillus paracasei subsp. paracasei	1	39
187	lactobacillus plantarum	2	49
188	lactobacillus reuteri	1	20
189	lactobacillus rhamnosus	2	68
190	lactococcus	1	29
191	lactococcus lactis	1	29
192	lactococcus lactis subsp. lactis	1	29
193	large intestine	4	83
194	lonchocarpus glaucifolius	1	20
195	loperamide	1	34
196	lutein	1	28
197	lycopersicon esculentum	1	28
198	male	2	68
199	matrix tablet	1	34
200	mechanical stress	1	34
201	metabolism	3	77
202	methane	2	54

203	methanogenesis	1	20
204	methylcellulose	1	34
205	microbial function	1	6
206	microbiological examination	1	29
207	microbiology	1	29
208	microbiota	4	98
209	microflora	1	14
210	microscopy, electron, scanning	1	29
211	model	2	63
212	models, biological	3	88
213	mouth flora	1	21
214	nalidixic acid	1	20
215	negibacteria	3	65
216	nifedipine	1	34
217	nitrate	1	20
218	nitrate reduction	1	20
219	nitrite	1	20
220	nitrogen	1	29
221	nonhuman	10	234
222	normal human	2	68
223	oligosaccharide	1	14
224	oligosaccharides	1	24
225	oral drug administration	1	34
226	ovatus	1	24
227	oxidation-reduction	1	20
228	peptostreptococcus	1	20
229	ph	2	57
230	phenol derivative	1	23
231	phenols	1	23
232	phenotype	1	20
233	physiology	1	29
234	polysaccharide	1	29
235	polysaccharides	1	29
236	population density	2	51
237	posibacteria	6	130
238	priority journal	3	65
239	probiotic	1	39
240	probiotics	2	68
241	propionic acid	1	34
242	radionuclide imaging	1	34
243	randomization	1	34
244	receptor affinity	1	20
245	risk factor	1	20
246	ruminococcus productus	1	20
247	scanning electron microscopy	1	29
248	short chain fatty acid	2	54
249	sodium chloride	1	34
250	spinach	1	28
251	spinacia oleracea	1	28
252	starch	1	24
253	steady state	1	21
254	stomach emptying	1	34
255	strain difference	1	39
256	streptococcus	1	29
257	streptococcus mitis	1	21
258	streptococcus sanguinis	1	21
259	streptococcus sanguis	1	21
260	streptococcus sobrinus	1	21

	261	substrate specificity	1	24
	262	symbiosis	1	12
	263	tablet disintegration	1	34
	264	tablet erosion	1	34
	265	tablets	1	34
	266	technique	1	29
	267	tomato	1	28
	268	transverse colon	1	20
	269	turbidimetry	1	29
	270	veillonella	1	21
	271	volatile fatty acid	1	29
	272	volatile fatty acids	1	34
	273	water absorption	1	16
	274	xanthophyll	1	28
**2006-2010**				
	1	1 hydroxypyrene	2	80
	2	7 hydroxy-benzo[a]pyrene	1	37
	3	9 anthracenepropionic acid	1	52
	4	9 phenanthrol	1	37
	5	accuracy	1	35
	6	acetic acid	2	76
	7	acetyl coenzyme a	1	42
	8	administration, oral	1	43
	9	adult	2	68
	10	aggr gene	1	28
	11	ammonia	4	125
	12	amylase	1	35
	13	anaerobic bacterium	2	96
	14	anaerobic metabolism	2	51
	15	anaerobic reactor	1	61
	16	anaerobiosis	2	89
	17	analysis of variance	1	31
	18	analytic method	2	72
	19	animal experiment	1	41
	20	animal tissue	1	41
	21	animalia	1	31
	22	animals	1	35
	23	anion exchange chromatography	1	53
	24	antacid agent	1	42
	25	anti-bacterial agents	1	34
	26	antibacterial	1	34
	27	antibacterial activity	1	34
	28	antiviral agents	1	42
	29	arabinose	1	53
	30	article	23	826
	31	artificial milk	1	27
	32	aryl hydrocarbon receptor	1	43
	33	arylsulfatase	1	25
	34	ascending colon	2	77
	35	azo reductase	1	25
	36	bacillus subtilis	1	52
	37	bacteria	8	330
	38	bacteria (microorganisms)	10	339
	39	bacteria, anaerobic	1	61
	40	bacterial colonization	1	20
	41	bacterial count	2	64
	42	bacterial culture	1	53
	43	bacterial gene	1	28

44	bacterial growth	5	170
45	bacterial membrane	1	34
46	bacterial metabolism	3	103
47	bacterial overgrowth	2	73
48	bacterial strain	3	123
49	bacterial survival	3	112
50	bacterial virulence	1	28
51	bactericidal activity	1	34
52	bacteriological techniques	1	43
53	bacteriology	2	70
54	bacteriolysis	1	42
55	bacteriophage	1	42
56	bacteriophages	1	42
57	bacterium	2	92
58	bacterium adherence	1	41
59	bacterium culture	6	243
60	bacterium identification	2	55
61	bacterium isolation	1	42
62	bacteroides	4	165
63	bacteroides fragilis	1	35
64	benz[a]anthracene	1	52
65	benzo[a]pyrene	2	95
66	benzo[a]pyrene derivative	1	37
67	benzo[b]fluoranthene	1	52
68	benzo[ghi]perylene	1	52
69	benzo[k]fluoranthene	1	52
70	beta glucosidase	1	25
71	beta glucuronidase	1	25
72	beverages	1	53
73	bifidobacteria	1	12
74	bifidobacterium	9	289
75	bifidobacterium adolescentis	2	61
76	bifidobacterium angulatum	1	23
77	bifidobacterium animalis	1	31
78	bifidobacterium bifidum	1	41
79	bifidobacterium boum	1	12
80	bifidobacterium choerinum	1	12
81	bifidobacterium longum	2	54
82	bifidobacterium longum bv. infantis	1	31
83	bifidobacterium longum infantis	1	31
84	bifidobacterium pseudolongum	1	12
85	bifidogenic	1	35
86	bile	1	24
87	bile acids and salts	1	42
88	bile salt	1	52
89	bile salts	1	52
90	bioaccessibility	1	24
91	bioassay	1	43
92	bioavailability	2	76
93	biofilms	1	38
94	biological availability	2	67
95	biological model	2	74

96	biological organs	1	38
97	biomass	1	37
98	bioreactor	1	20
99	bioreactors	2	78
100	biosynthesis	1	42
101	biotransformation	2	80
102	bovids	1	42
103	bread	1	35
104	bucco	1	35
105	butyrate synthesis	1	42
106	butyrates	1	61
107	butyric acid	5	191
108	calibration	1	37
109	caloric intake	1	26
110	candida	1	38
111	carbohydrate	3	119
112	carbohydrate analysis	2	76
113	carbohydrate metabolism	1	25
114	carbon 13	2	79
115	carbon dioxide	1	35
116	carbon nuclear magnetic resonance	1	42
117	carboxylic acid	1	31
118	carboxylic acids	2	84
119	cell adhesion	1	17
120	cell culture	1	38
121	cell culture techniques	1	28
122	cell growth	1	38
123	cell membranes	1	17
124	cell strain ht29	1	41
125	cells, immobilized	1	31
126	centrifugation	2	76
127	chemostat	1	17
128	chicory	1	35
129	child	1	27
130	choline	1	52
131	chromatography, high pressure liquid	2	68
132	chromatography, ion exchange	1	53
133	chrysene	1	52
134	cichorium intybus	1	35
135	citrus sinensis	1	53
136	clinical article	2	64
137	clostridium	5	181
138	clostridium coccoides	1	61
139	clostridium difficile	1	26
140	clostridium histolyticum	2	94
141	clostridium perfringens	2	60
142	cluster analysis	1	43
143	colon	7	301
144	colon flora	4	132
145	colonic fermentation	1	42
146	colonic microbiota	1	26
147	colony count, microbial	3	138
148	colony forming unit	1	42
149	community stability	1	43
150	comparative study	1	31
151	compartment model	1	24

152	competitive ability	1	17
153	complex formation	1	52
154	complexation	1	52
155	computer simulation	1	17
156	concentration response	2	95
157	continuous stirred tank reactor	1	17
158	controlled study	12	473
159	correlation analysis	2	77
160	culture media	4	173
161	culture medium	3	119
162	culture system	1	27
163	cytosine	1	61
164	dahlia	1	61
165	denaturing gradient gel electrophoresis	3	105
166	descending colon	1	35
167	desorption	1	52
168	device	1	20
169	diarrhea	1	28
170	diet	1	34
171	dietary carbohydrates	2	96
172	dietary intake	1	61
173	dietary proteins	1	35
174	digestion	3	90
175	digestive physiology	1	24
176	digestive system	4	143
177	diseases	1	38
178	dissolved organic matter	1	52
179	dna fingerprinting	1	27
180	dna, bacterial	1	43
181	dna, viral	1	42
182	duodenum	2	90
183	dysphagia	1	38
184	ecological modeling	1	17
185	ecosystem	6	238
186	electricity	1	34
187	electrophoresis, gel, two-dimensional	1	35
188	electrophoresis, polyacrylamide gel	1	43
189	energy resource	1	61
190	engineering model	1	17
191	enteral nutrition	1	38
192	enteric feeding	1	38
193	enterobacter	1	42
194	enterobacter aerogenes	1	42
195	enterobacteriaceae	2	40
196	enterococcus	4	149
197	environmental exposure	1	43
198	enzyme activity	2	66
199	enzyme inhibition	1	25
200	enzyme release	1	35
201	escherichia coli	4	130
202	estrogen	1	43
203	estrogen activity	1	43
204	estrogen receptor	1	43
205	estrogens	1	43
206	ethinylestradiol	1	43
207	eubacterium	2	114

208	eubacterium cylindroides	1	61
209	eubacterium rectale	2	114
210	eubacterium sp.	1	61
211	experimental model	1	35
212	extraction	1	37
213	faecalibacterium prausnitzii	1	61
214	fatty acid	2	70
215	fatty acid analysis	1	53
216	fatty acids	2	96
217	fatty acids, volatile	1	42
218	feces	3	126
219	feces analysis	1	27
220	feces microflora	4	179
221	female	2	68
222	fermentation	11	401
223	fermentation technique	1	31
224	fermented product	1	35
225	fermenters	1	17
226	fermentors	1	61
227	flow conditions	1	17
228	flow cytometry	1	34
229	flow of fluids	1	17
230	fluoranthene	1	52
231	fluorescence	1	61
232	fluorescence in situ hybridization	3	155
233	food analysis	1	35
234	food composition	1	35
235	food contamination	1	43
236	food intake	1	31
237	food microbiology	1	34
238	food processing	1	35
239	fructo-oligosaccharides	1	35
240	fructose	1	23
241	fructose oligosaccharide	1	23
242	fucose	1	53
243	galactooligosaccharides	1	41
244	galactose	2	78
245	galactose oligosaccharide	2	64
246	galactosyltransferase	1	41
247	galacturonic acid	1	53
248	gas chromatography	1	42
249	gas chromatography-mass spectrometry	1	42
250	gastric acid	1	38
251	gastric acidity determination	2	73
252	gastric emptying	1	35
253	gastroenterology	2	75
254	gastrointestinal (gi) tract	1	38
255	gastrointestinal absorption	1	27
256	gastrointestinal tract	5	186
257	gastrointestinal tracts	1	37
258	gene control	1	28
259	gene expression	1	28

260	gene expression profiling	1	28
261	gene expression regulation	1	28
262	genes, bacterial	1	42
263	genes, reporter	1	42
264	genetic association	1	28
265	genetic strain	1	17
266	genotoxicity	1	25
267	glucose	1	53
268	gram negative bacterium	1	34
269	gram positive bacterium	1	34
270	green fluorescent protein	1	42
271	growth inhibition	1	26
272	guanine	1	61
273	gut fermentation	1	27
274	health promotion	1	34
275	hexanoic acid	1	27
276	high performance liquid chromatography	1	31
277	hospitals	1	42
278	human	9	323
279	human cell	1	41
280	human colon	1	17
281	human intestinal microflora	1	42
282	human intestine	1	17
283	humans	12	477
284	hydrogen-ion concentration	5	174
285	hydroxylation	1	37
286	ileum	1	35
287	immobilized cell	1	31
288	impurities	1	52
289	in situ hybridization, fluorescence	1	61
290	in vitro	1	31
291	in vitro model	1	26
292	in vitro study	8	309
293	incubation temperature	1	53
294	incubation time	2	80
295	indeno[1,2,3 cd]pyrene	1	52
296	infant	2	58
297	infant feeding	1	27
298	ingestion	3	119
299	interspecific competition	1	17
300	intestinal bacteria	1	43
301	intestinal microbiota	1	27
302	intestinal microflora	1	41
303	intestine	2	51
304	intestine absorption	2	78
305	intestine flora	10	350
306	intestine motility	1	26
307	intestine transit time	2	49
308	intestines	4	154
309	inulin	5	188
310	isotope labeling	1	42
311	kefir	1	31

312	klebsiella pneumoniae	1	38
313	lactate metabolism	1	42
314	lactic acid	2	83
315	lactic acid bacteria	1	35
316	lactic acid bacterium	2	77
317	lactobacillaceae	1	35
318	lactobacillus	10	318
319	lactobacillus acidophilus	1	31
320	lactobacillus johnsonii	1	31
321	lactobacillus kefiranofaciens subsp. kefirgranum	1	31
322	lactobacillus kefiri	1	31
323	lactobacillus rhamnosus	2	51
324	lactose	1	41
325	lactulose	1	20
326	large intestine	2	77
327	lead	1	24
328	leuconostoc mesenteroides	1	31
329	liquid chromatography	2	80
330	magnetic resonance spectroscopy	1	42
331	male	2	68
332	mammalia	1	17
333	mass spectrometry	3	122
334	mastication	1	35
335	mathematical analysis	1	52
336	mathematical model	1	17
337	mathematical models	2	69
338	megasphaera	1	27
339	membrane damage	1	34
340	membrane fermenters	1	17
341	metabolic activation	1	26
342	metabolic activity	1	26
343	metabolism	4	161
344	metabolite	1	43
345	metabolites	2	54
346	microbial community	3	95
347	microbial ecology	1	35
348	microbial growth	1	17
349	microbiological examination	1	35
350	microbiology	5	196
351	microbiota	7	210
352	middle aged	1	42
353	milk	1	35
354	milk proteins	1	35
355	mode of action	1	34
356	model	3	76
357	models, biological	5	166
358	models, theoretical	1	43
359	molecular weight	1	35
360	monosaccharide	1	53
361	monosaccharides	1	53
362	mouth	1	35
363	moving window correlation	1	43
364	nanofiltration	1	53

365	naphthalene	2	95
366	negibacteria	3	137
367	nitroreductase	1	25
368	nmr spectroscopy	1	42
369	nonhuman	16	586
370	normal human	1	27
371	nucleotide sequence	1	42
372	nutrient availability	1	35
373	nutrient uptake	1	17
374	nutrition	1	38
375	nutritional value	1	26
376	oligomers	1	53
377	oligonucleotide probes	1	61
378	oligosaccharide	5	177
379	oligosaccharides	2	65
380	oral exposure	1	43
381	orange juice	1	53
382	organic acids	1	38
383	organic matter	1	52
384	organic matters	1	52
385	oropharyngeal disease	1	38
386	pancreas juice	1	42
387	pancreatic juice	1	42
388	particle size	1	35
389	pcr-dgge	1	43
390	pectic oligosaccharide	1	53
391	pectin	1	53
392	pectins	1	53
393	peptostreptococcus	1	25
394	ph	7	250
395	ph effects	2	55
396	ph measurement	1	31
397	phenanthrene	2	95
398	phenanthrene derivative	1	37
399	physical chemistry	1	35
400	physiological models	1	38
401	physiology	2	48
402	piglet	1	12
403	plaque assay	1	42
404	plaque forming cell	1	42
405	plate count	1	34
406	plug flow reactor	1	17
407	pollution exposure	1	24
408	polycyclic aromatic hydrocarbon	3	132
409	polycyclic aromatic hydrocarbons	2	89
410	polycyclic compounds	1	37
411	polycyclic hydrocarbons, aromatic	1	43
412	polyethylene glycols	1	17
413	polymerase chain reaction	3	120
414	polymerization	1	26
415	polynuclear aromatic hydrocarbons	1	37
416	polysaccharide	1	61
417	polysaccharides	2	114
418	population dynamics	2	78
419	posibacteria	2	95

420	prebiotic	1	41
421	prebiotic agent	4	171
422	prebiotic effect	1	53
423	precipitation	1	52
424	prediction	1	52
425	principal component analysis	1	43
426	priority journal	11	331
427	probiotic agent	2	92
428	probiotics	2	92
429	process model	1	52
430	propionic acid	5	197
431	protein	1	35
432	protein degradation	1	35
433	proteins	1	53
434	pyrene	1	43
435	qualitative analysis	1	43
436	quantitative analysis	3	122
437	reactor	3	126
438	real time polymerase chain reaction	3	105
439	regulatory mechanism	1	35
440	reynolds number	1	17
441	rhamnose	1	53
442	ribosome rna	1	23
443	ribotyping	1	20
444	risk assessment	2	67
445	risk benefit analysis	1	26
446	rna	1	61
447	rna 16s	2	102
448	rna analysis	1	23
449	rna probe	1	61
450	rna sequence	1	61
451	rna, ribosomal, 16s	1	61
452	roseburia	1	61
453	roseburia intestinalis	1	61
454	ruminococcus	1	61
455	ruminococcus bromii	1	61
456	ruminococcus flavefaciens	1	61
457	saliva	1	35
458	salmonella enterica	1	41
459	salmonella enterica subsp. enterica serovar typhimurium	1	41
460	salmonella typhimurium	1	41
461	sensitivity and specificity	1	37
462	sewage	1	42
463	shime	3	112
464	short chain fatty acid	3	130
465	signal noise ratio	1	37
466	simulation	9	309
467	simulator	4	149
468	simulator of human intestinal microbial ecosystem	1	52
469	simulator of the human intestinal microbial ecosystem (shime)	1	43
470	small intestine	1	43
471	sodium chloride	2	62

	472	soil	1	43
	473	soil ingestion	1	52
	474	soil pollutants	2	67
	475	soil pollution	3	119
	476	soils	1	52
	477	solid phase extraction	1	37
	478	species composition	1	43
	479	species specificity	1	43
	480	specimen handling	1	35
	481	sphingolipids	1	34
	482	sphingosine	1	34
	483	standard	1	37
	484	staphylococcus	3	109
	485	staphylococcus aureus	1	38
	486	starch	1	53
	487	statistical analysis	1	43
	488	statistical significance	1	35
	489	steady state	1	17
	490	stearic acid	1	34
	491	stomach	4	164
	492	stomach emptying	1	35
	493	stomach function	1	35
	494	stomach juice	1	24
	495	stomach ph	2	73
	496	strain	1	61
	497	streptococcus	1	35
	498	sugar	1	27
	499	supernatants	1	52
	500	surfactant	1	34
	501	sus scrofa	1	41
	502	swine	1	41
	503	temperature	2	66
	504	temperature dependence	1	31
	505	toscana virus	1	12
	506	toxicity testing	1	43
	507	transcription regulation	1	28
	508	unclassified drug	5	206
	509	unidentified bacteriophage	1	42
	510	valeric acid	2	69
	511	validation process	1	52
	512	veillonella	1	28
	513	virulence factor	1	28
	514	virus inactivation	1	42
	515	washout model	1	17
	516	water absorption	1	26
	517	weaning	1	27
	518	whey	1	35
	519	xylose	1	53
	520	yeast	1	12
**2011-2015**				
	1	16s rrna gene	2	109
	2	16s rrna gene-targeted qpcr	1	56
	3	3 (3’ hydroxyphenyl)propionic acid	1	51
	4	3s-ecsim	1	53
	5	4 hydroxybenzoic acid	1	77

7	absorption	1	52
8	abundance	1	56
11	acetaminophen	1	44
13	acetate coenzyme a transferase	1	51
14	acetic acid	9	304
15	acetic acid derivative	1	75
18	actinobacteria	2	78
19	acyl coenzyme a	2	100
20	adaptation	2	85
22	adherence	1	51
23	adhesion	2	103
24	adme process	1	49
25	adme processes	1	49
26	administration, oral	2	89
27	adsorption	2	101
28	adsorption kinetics	1	49
29	adult	10	417
30	agar	6	165
31	aged	3	154
33	albumin	1	69
36	alginate polylysine alginate	1	45
37	alginates	3	120
38	alginic acid	1	45
40	alkylation	1	52
45	alpha arabinofuranosidase	1	60
46	alpha fetoprotein	1	69
48	alphafetoprotein (afp)	1	69
52	amidohydrolases	1	51
54	ammonia	3	126
55	ammonium compounds	2	57
56	ammonium derivative	2	57
59	amylase	1	51
60	amylases	2	67
62	anaerobic atmosphere	1	56
63	anaerobic digestion	2	43
64	anaerobic fermentation	1	53
65	anaerobiosis	3	101
67	anaerostipes caccae	1	48
68	analogs and derivatives	1	45
69	analysis	4	167
71	analytical techniques	1	56
72	animal	9	286
73	animal experiment	2	87
75	animalia	3	87
76	animals	11	336
77	anion exchange chromatography	2	45
79	anthocyanin	2	104
80	anthocyanins	1	75
81	anti-bacterial agents	3	125
83	anti-inflammatory activity	1	45
84	anti-inflammatory agents	3	143
87	antibiotic sensitivity	1	50

89	antihypertensive activity	1	77
90	antiinfective agent	2	103
91	antiinflammatory activity	1	77
92	antiinflammatory agent	2	66
94	antimicrobial activity	1	77
95	antioxidant	1	55
96	antioxidant activity	1	75
97	antioxidants	1	55
100	arabinoxylan	2	93
101	arabinoxylan-degrading enzymes	1	60
102	arachis hypogaea	1	45
104	arsenic	1	52
105	arsenic trioxide	1	52
106	arsenite	1	52
107	article	67	2564
108	artificial simulator	1	51
109	ascending colon	6	288
111	atmosphere	1	56
113	bacilli	6	194
118	bacteria	21	832
119	bacteria (microorganisms)	14	471
120	bacterial adhesion	5	195
121	bacterial attachment	1	52
122	bacterial capsule	1	52
123	bacterial capsules	1	52
124	bacterial cell	5	215
125	bacterial colonization	4	145
126	bacterial count	5	192
127	bacterial dna	3	134
128	bacterial enzyme	1	60
129	bacterial gene	3	165
130	bacterial growth	9	351
133	bacterial metabolism	4	158
134	bacterial phenomena and functions	1	53
135	bacterial physiological phenomena	1	53
136	bacterial protein	2	75
137	bacterial proteins	3	126
138	bacterial retentions	1	52
139	bacterial rna	1	52
140	bacterial spore	2	73
141	bacterial strain	4	193
142	bacterial survival	3	74
143	bacterial toxin	1	50
144	bacterial viability	3	122
148	bacteriology	1	46
149	bacterium	17	690
150	bacterium adherence	6	248
152	bacterium culture	10	480
153	bacterium detection	1	49
154	bacterium identification	2	62
156	bacteroidaceae	2	102
157	bacteroides	9	357
159	bacteroides fragilis	1	50
160	bacteroides ovatus	1	53
161	bacteroides sp.	1	44

162	bacteroides thetaiotaomicron	2	78
163	bacteroides uniformis	1	53
165	bacteroidetes	6	220
167	base sequence	2	53
169	batch cell culture	1	56
170	batch culture	1	56
171	batch fermentation	3	101
172	batch fermentations	2	85
174	beneficial organism	1	47
175	benzo(a)pyrene	1	49
176	benzo[a]pyrene	1	49
177	benzoic acid	2	132
178	benzoic acid derivative	1	55
181	beverages	2	38
183	bifidobacteria	3	94
184	bifidobacterium	21	869
185	bifidobacterium adolescentis	3	82
187	bifidobacterium animalis	2	85
189	bifidobacterium bifidum	1	46
190	bifidobacterium catenulatum	1	46
191	bifidobacterium longum	5	210
192	bifidobacterium longum infantis	2	71
197	bile acid	3	94
198	bile acids and salts	4	145
199	bile salt	2	76
200	bile salt hydrolase	1	51
201	binding affinity	1	49
202	binding competition	1	47
206	bioactivity	1	75
207	bioassay	3	149
208	bioavailability	3	175
209	biochemistry	3	105
210	bioconversion	1	69
211	biodegradation	3	152
212	biodiversity	2	64
213	bioequivalence	2	29
214	biofilm	5	243
215	biofilm reactor	1	53
216	biofilms	4	189
217	biological activity	2	152
218	biological availability	3	176
219	biological marker	1	69
220	biological model	27	1046
222	biomarkers	1	69
223	biomass	2	55
225	biomaterials	1	69
226	biomolecules	2	59
227	bioreactor	7	309
228	bioreactors	10	410
230	biosynthesis	1	52
231	biota	4	135
232	biotechnology	1	77
233	biotransformation	3	138
235	black tea	1	50
236	black tea extract	1	77
237	black tea polyphenol	1	77
238	blautia	2	104

240	blood pressure	1	77
241	blueberry	1	75
242	blueberry plant	1	75
244	bone marrow cell	1	69
245	bone marrow cells	1	69
247	bowel	2	81
249	brassica	1	53
250	brassica oleracea var. italica	1	53
251	bread	2	41
252	breakthrough curve	1	48
253	breast milk	1	51
256	broccoli	1	53
259	butyrate	2	55
260	butyrates	8	291
261	butyric acid	10	360
262	butyric acid derivative	7	267
263	butyryl coenzyme a	3	151
264	butyryl-coenzyme a	2	100
267	caco-2 cells	2	71
269	caffeic acid	1	75
270	calcination	1	45
271	calibration	1	49
272	caloric intake	2	53
273	camellia sinensis	3	140
274	capsular polysaccharides	1	52
275	capsules	2	89
276	carbohydrate	3	100
278	carbohydrate derivative	1	60
280	carbohydrate metabolism	4	151
281	carbon	3	99
282	carbon 13	1	46
283	carbon dioxide	1	53
284	carbon isotopes	1	46
285	carbon source	1	47
286	carboxylic acid	2	75
288	carcinogens	1	49
289	cardiovascular agents	1	77
290	cardiovascular diseases	1	77
291	cardiovascular system	1	77
292	carrageenan	2	60
294	catechin	2	107
295	catechins	1	50
296	catechol	2	132
298	cd235a antigen	1	69
299	cd309 antigen	1	69
300	cd31 antigen	1	69
301	cd34 antigen	1	69
303	cell adhesion	1	51
304	cell assay	2	72
306	cell culture	3	189
307	cell culture technique	2	98
308	cell culture techniques	1	69
309	cell density	2	95
310	cell differentiation	1	69
311	cell fate	1	49
312	cell function	1	77
313	cell line	2	68

314	cell membrane permeability	1	49
316	cell metabolism	2	118
318	cell proliferation	1	69
319	cell strain caco 2	1	49
320	cell strain hepg2	1	49
321	cell surface marker	1	69
322	cell survival	2	68
323	cell viability	3	138
325	cells	2	144
327	cells, immobilized	2	74
334	chemistry	23	908
335	chemistry, pharmaceutical	2	50
336	chemostat	6	284
337	chemostats	2	127
340	child	4	156
341	child health	1	44
342	chlorogenic acid	1	75
344	chromatography	2	131
347	chromatography, high pressure liquid	3	121
348	chromatography, ion exchange	1	56
349	chromatography, liquid	2	74
351	chymotrypsin	1	51
352	cinammic acid	1	75
353	cinnamic acid	1	75
354	cinnamic acid derivative	1	77
357	classification	6	240
358	clindamycin	2	103
359	clinical article	1	53
360	clinical trial	1	53
361	clostridia	5	146
362	clostridium	13	537
365	clostridium coccoides	1	51
366	clostridium difficile	3	106
367	clostridium difficile infection	2	83
368	clostridium infections	1	50
370	clostridium leptum	2	63
372	cluster analysis	3	129
373	coculture	1	45
374	coculture techniques	2	94
375	coenzyme a	1	51
376	coenzyme a transferase	2	103
377	coenzyme a-transferases	2	103
378	coffea	1	47
379	coffee	1	47
380	colitis, ulcerative	1	52
381	collagen	1	54
382	colon	25	1018
385	colon flora	8	317
389	colonic metabolism	1	51
390	colonic microbiota	2	85
392	colonization	3	130
393	colony count, microbial	3	91
394	colony forming unit	4	124
396	community dynamics	2	57
397	community stability	1	46

398	community structure	2	87
399	community structures	1	45
400	comparative study	4	144
403	computer model	3	62
404	computer simulation	3	112
405	concentration (composition)	1	44
406	concentration (parameters)	5	180
410	continuous culture	1	56
411	continuous feeding	1	50
412	continuous fermentation	2	82
413	continuous fermentation systems	1	56
414	continuous flow reactor	2	104
415	control parameters	1	45
418	controlled study	27	1019
419	cooking	2	69
420	coriobacteriaceae	1	53
424	cranberry	1	55
425	cranberry extract	1	55
426	crl-1790	1	75
428	cruciferous vegetables	1	53
430	culture media	4	177
431	culture media, conditioned	1	49
432	culture medium	7	312
434	culture techniques	2	89
435	cyanidin 3 glucoside	1	75
436	cyanobacterium	2	106
437	cyclopeptide	1	50
438	cytochrome p450 3a4	1	69
439	cytokeratin 19	1	69
440	cytokine	1	45
441	cytokine production	1	54
442	cytokines	1	45
443	cytology	2	92
444	cytotoxin	1	50
445	daptomycin	2	72
446	data processing	1	56
448	delivery system	1	45
449	delphinidin 6 acetyl 3 glucoside	1	75
450	denaturing gradient gel electrophoresis	9	344
452	descending colon	2	102
453	desulfovibrionaceae	1	53
454	dgge	2	81
455	diagnostic techniques, digestive system	1	45
456	diet	2	53
457	diet restriction	1	44
458	diet supplementation	2	57
461	dietary fiber	7	210
463	dietary intake	1	53
465	differential response	1	56
466	differentially expressed gene	1	52
467	diffusion	1	69
468	diffusion coefficient	1	44
470	digestion	17	549

476	digestive fluids	1	51
477	digestive system	6	202
478	digestive system disorder	1	52
479	digestive system examination	1	45
481	dihydrocaffeic acid	1	77
482	dilution	1	56
484	dilution rate	1	56
485	dimocarpus longan	2	45
488	dissolution	3	74
489	distal colons	1	50
493	dna extraction	3	127
494	dna fingerprinting	3	115
495	dna microarray	3	106
496	dna sequence	4	183
497	dna, bacterial	5	208
498	dna, ribosomal	1	60
501	down regulation	1	51
503	drug antagonism	1	45
504	drug bioavailability	2	152
505	drug blood level	1	77
506	drug capsule	2	60
508	drug delivery	1	51
509	drug delivery system	1	45
510	drug determination	1	77
512	drug effects	6	216
513	drug efficacy	2	125
514	drug formulation	3	73
515	drug metabolism	1	77
516	drug release	3	82
517	drug retention	1	46
518	drug solubility	5	123
519	drug stability	1	75
520	drug transformation	1	77
521	drug urine level	1	77
522	duodenum	3	100
524	dynamic gastric model	5	124
526	dynamic simulation	1	44
527	dynamic simulators	1	47
528	dysbiosis	2	82
530	ecology	2	52
531	ecosystem	4	122
532	ecosystems	5	88
536	electrophoresis, polyacrylamide gel	2	37
537	electrospray mass spectrometry	1	47
538	embryology	1	69
541	endo 1,4 beta xylanase	1	60
542	endothelial cells	1	69
543	endothelial differentiation	1	69
544	endothelial nitric oxide synthase	1	69
545	endothelium	2	146
546	endothelium cell	1	69
547	energy intake	2	53
549	energy resource	1	60
553	enterobacteriaceae	7	270
554	enterococcus	7	219
558	enterocytes	2	99

560	environmental change	1	56
561	environmental control system	1	56
562	environmental exposure	1	52
564	environmental monitoring	1	52
569	enzyme activity	4	142
570	enzyme inhibition	1	46
572	enzymes	2	44
573	enzymology	1	51
574	epicatechin	1	77
575	epigallocatechin	1	77
576	epigallocatechin gallate	1	77
577	epithelial cells	1	54
578	epithelium cell	2	129
579	erythrocyte membrane	1	69
580	escherichia coli	5	177
583	ethical considerations	1	51
584	eubacterium	4	193
585	eubacterium rectale	5	233
586	ex vivo study	1	53
587	excipients	1	44
589	experimental study	2	32
590	experiments	2	26
591	extraction	1	52
592	extraction solution	1	52
596	faecalibacterium prausnitzii	4	187
597	fasting	1	44
599	fatty acid	3	154
601	fatty acids	8	258
602	fatty acids, volatile	8	273
603	feasibility studies	2	96
604	feasibility study	2	96
605	feasibility tests	1	45
606	feces	17	678
607	feces analysis	7	282
608	feces microflora	7	257
609	feeding	3	145
610	female	12	456
611	fermentation	29	1070
613	fermentation gas	1	53
614	fermentation model	5	166
620	ferredoxin	1	51
621	fetus	1	69
622	fetus liver	1	69
625	firmicutes	6	220
627	flavonoid glycoside	1	77
628	flavonoids	3	131
632	flow cytometry	2	92
636	fluorescence microscopy	1	51
637	follow up	1	54
638	food	4	143
639	food additives	2	37
642	food composition	2	54
643	food contamination	1	47
644	food digestion	1	45
649	food intake	1	54
652	food particles	1	45
655	food quality	1	75

657	food-drug interactions	2	69
658	formic acid	2	64
660	fractionation	2	81
663	fructo-oligosaccharides	2	52
666	fructose oligosaccharide	3	71
667	fruit	3	160
671	functional food	1	75
672	fusobacteria	1	53
675	galactooligosaccharides	3	86
676	galactose	3	118
677	galactose oligosaccharide	4	133
678	gallic acid	1	77
679	gallic acids	1	50
680	gamma scintigraphy	1	44
681	gas	1	53
682	gas chromatography	3	108
684	gases	1	56
687	gastric emptying	2	73
688	gastric juice	4	150
691	gastrointestinal	1	51
695	gastrointestinal microbiome	5	213
696	gastrointestinal model	3	102
697	gastrointestinal motility	2	47
698	gastrointestinal resource management	3	59
700	gastrointestinal tract	25	949
702	gastrointestinal transit	3	135
705	gel permeation chromatography	2	37
706	gelatin	2	60
707	gellan	2	60
708	gene	1	56
709	gene dosage	1	49
710	gene expression	5	239
711	gene expression profiling	1	52
714	gene locus	1	52
715	gene sequence	3	134
717	general device	1	54
718	genes	1	69
719	genes, bacterial	2	105
720	genetic polymorphism	1	53
721	genetics	16	706
722	genomic dna	1	51
723	gi tract	2	33
724	glass vessel	1	45
725	glucoraphanin	1	53
726	glucose	5	130
728	glucose intake	1	69
729	glucoside	1	75
730	glucosinolate	1	53
731	glucosinolates	1	53
732	glucuronic acid	1	51
736	glycan	1	52
740	glycerol	1	56
742	glycosidase	1	60

743	glycoside hydrolases	1	60
746	gnotobiotics	1	52
747	gram positive bacterium	1	51
748	gram-positive bacteria	1	51
749	grape	2	84
750	grape seed extract	1	55
751	grape seeds	1	55
755	growth and development	1	51
756	growth rate	2	79
758	growth, development and aging	14	549
761	gut bacteria	1	53
762	gut microbiota	16	572
763	gut model	1	51
764	health	1	75
765	health impact	1	44
766	health risk	1	52
767	health risks	1	52
768	healthy volunteers	1	60
769	hep g2 cells	1	49
770	hepatic differentiation	1	69
771	hexuronic acids	1	51
772	hier-archical clustering	1	52
773	high performance liquid chromatography	4	158
778	hippuric acid	2	152
779	hitchip	1	53
781	host	1	47
782	host microbiota interaction	1	54
783	host microbiota interaction module	1	54
785	ht 29 cell line	1	75
786	ht-29	1	75
787	hugchip	1	53
788	human	62	2358
789	human cell	4	214
790	human colon	1	56
794	human experiment	2	38
796	human gut microbiota	3	138
797	human intestinal microbiota	1	47
799	human microbiota	1	56
800	human tissue	5	240
801	humans	64	2411
803	hydrocinnamic acid	1	77
804	hydrodynamic flows	1	45
805	hydrogen	2	104
806	hydrogen-ion concentration	9	256
807	hydrolase	1	51
815	hydroxypropylmethylcellulose	3	85
817	idolax	2	54
819	imidoester	1	53
820	imidoesters	1	53
822	immobilized cell	2	74
823	immune response	1	45
824	immunohistochemistry	1	69

825	immunologic factor	1	45
826	immunologic factors	1	45
827	immunology	1	45
832	in vitro digestion	3	93
833	in vitro digestive system	1	56
839	in vitro model	1	51
840	in vitro models	2	48
842	in vitro study	36	1434
844	in vitro techniques	4	128
845	in vitro-in vivo extrapolation	1	49
847	in vivo study	7	343
848	in-vitro	8	232
850	in-vitro digestions	4	136
851	in-vitro models	1	51
852	in-vitro simulation of the colon	1	53
854	in-vivo	1	56
855	infant	1	51
856	infant formula	1	51
857	infant nutrition	1	51
858	infant nutritional physiological phenomena	1	51
859	infant nutritions	1	51
860	infant, newborn	1	51
861	infant, premature	1	51
862	inflammatory bowel disease	1	52
864	inflorescence	1	53
870	inoculation	4	140
873	instrumentation	2	70
874	interleukin 8	1	54
875	intermediate metabolites	1	51
876	intermethod comparison	1	53
877	interphase	1	54
878	intestinal absorption	1	49
879	intestinal bacteria	2	78
880	intestinal microbiota	3	78
882	intestinal mucosa	5	199
885	intestine	7	305
886	intestine cell	3	120
887	intestine epithelium	2	77
888	intestine flora	27	1103
889	intestine function	1	49
890	intestine mucosa	7	273
891	intestine transit time	2	90
892	intestine, large	5	150
893	intestine, small	1	49
894	intestines	3	121
895	inulin	2	56
897	ion exchange chromatography	2	91
899	iron	2	95
900	iron deficiency	2	95
902	isolation and purification	8	325
903	isothiocyanates	1	53
904	isothiocyanic acid	1	53
905	isothiocyanic acid derivative	1	53
906	isotope labeling	1	46

908	juice extracts	1	50
909	kaempferol 3 rhamnoside	1	75
910	kinetic process	1	45
911	kinetics	2	94
913	knowledge	1	77
914	l-lactate dehydrogenase	1	51
915	lachnospiraceae	2	97
916	lactate dehydrogenase	2	120
917	lactate dehydrogenase activities	1	69
918	lactic acid	8	313
919	lactic acid bacteria	2	57
920	lactobacillaceae	1	45
922	lactobacilli	2	49
923	lactobacillus	20	712
924	lactobacillus acidophilus	7	206
925	lactobacillus acidophilus la5	2	45
926	lactobacillus amylovorus	2	85
927	lactobacillus casei	4	70
928	lactobacillus casei 01	2	45
930	lactobacillus fermentum	1	51
931	lactobacillus gasseri	1	46
933	lactobacillus plantarum	4	133
934	lactobacillus reuteri	3	120
935	lactobacillus rhamnosus	2	77
936	lactobacillus salivarius	1	46
938	lactococcus lactis	2	47
940	lactone derivative	1	77
941	large intestine	4	117
948	limit of detection	1	50
950	lipase	1	51
953	lipid	1	45
955	lipids	1	45
956	lipopeptide	1	50
957	lipopeptides	1	50
958	liquid chromatography	7	310
959	liquid chromatography tandem mass spectroscopy	1	77
960	liver	2	118
961	liver metabolism	1	49
963	long chain arabinoxylan derivative	1	60
964	long-chain arabinoxylans	1	60
970	male	10	333
971	malvidin 3 arabinoside	1	75
972	malvidin 3 galactoside	1	75
973	malvidin 3 glucoside	1	75

974	malvidin 6 acetyl 3 glucoside	1	75
975	mamc	1	52
977	mandelic acid	1	77
983	mass spectrometry	5	186
992	mean particle size	1	45
994	medical applications	1	45
997	membrane protein	1	77
999	metabolic activity	1	56
1001	metabolic fate	1	47
1002	metabolic profiling	1	50
1003	metabolic stability	1	49
1004	metabolism	40	1529
1005	metabolite	6	321
1007	metabolites	4	162
1008	metabolome	2	52
1009	metabolomics	2	127
1010	metagenome	13	463
1011	metagenomics	1	77
1013	methodology	2	47
1014	methylation	1	52
1015	methylcellulose	1	44
1017	mice	2	81
1018	microarray analysis	4	180
1019	microbial activity	4	199
1020	microbial biomass	1	46
1021	microbial colonization	1	54
1022	microbial communities	3	70
1023	microbial community	19	778
1025	microbial consortia	2	70
1026	microbial consortium	1	44
1027	microbial degradation	2	105
1028	microbial diversity	3	137
1029	microbial eco system	5	127
1030	microbial ecology	3	89
1031	microbial fermentation	2	75
1033	microbial interactions	2	54
1034	microbial metabolism	5	197
1035	microbial modulation	1	55
1036	microbial morphology	1	53
1037	microbial production	1	50
1038	microbial reduction	1	52
1039	microbial viability	7	207
1041	microbiological phenomena and functions	1	54
1042	microbiology	34	1292
1043	microbiota	22	844
1044	microbiotas	10	319
1045	microcapsule	1	45
1047	microencapsulation	1	45
1048	microflora	17	654
1049	microfluidics	1	49
1050	microsphere	2	55
1051	microspheres	2	55
1052	middle aged	2	105
1053	milk	3	74
1054	milk, human	1	51

1056	minimum inhibitory concentration	3	96
1057	model	2	69
1059	models, anatomic	2	84
1060	models, biological	39	1456
1061	models, theoretical	9	316
1062	modulating effect	1	45
1063	molecular analysis	2	79
1064	molecular docking	1	47
1065	molecular fingerprint	1	56
1068	molecular weight	1	54
1069	monocyte	1	45
1070	monocytes	1	45
1071	monte carlo method	1	48
1074	mouse	2	81
1075	mucin	6	168
1077	mucins	4	174
1078	mucosa	2	104
1079	mucus	5	190
1082	mus	1	52
1084	newborn	1	51
1085	newborn infant	1	51
1090	non-specific adsorption	1	49
1092	nonhuman	29	1180
1093	normal human	4	148
1096	nucleotide sequence	4	109
1097	numerical model	1	56
1103	nutrition	1	51
1105	nutritional status	1	44
1108	ochratoxin	1	47
1109	ochratoxin a	1	47
1110	ochratoxins	1	47
1111	oligonucleotide array sequence analysis	2	58
1112	oligosaccharide	5	157
1113	oligosaccharides	7	232
1115	oral capsule shell	1	44
1116	oral drug administration	1	45
1117	oral drug delivery	2	95
1119	organic acids	1	50
1120	organismal interaction	2	54
1121	oxidative stress	1	77
1122	oxidoreductase	1	51
1123	oxygen	1	54
1124	oxygen concentration	1	54
1125	oxygen diffusion	1	54
1130	para coumaric acid	1	77
1131	parabacteroides distasonis	1	53
1132	paracetamol	2	60
1133	particle size	3	87
1134	particle size analysis	1	45
1138	pathogen	2	76
1139	pbpk model	1	49
1140	pcr	1	46
1141	pcr-dgge	2	64
1142	peanut	1	45
1145	peonidin 3 arabinoside	1	75
1146	peonidin 3 galactose	1	75
1147	peonidin 3 galactoside	1	75

1148	pepsin a	2	96
1151	peptides, cyclic	1	50
1152	peptoclostridium difficile	4	135
1153	perfusion	2	118
1154	perfusion conditions	1	69
1155	perfusion culture	1	49
1156	peristalsis	2	58
1158	petunidin 3 galactoside	1	75
1159	ph	11	339
1163	phenol derivative	3	155
1164	phenolic acids	1	50
1166	phenolic compounds	2	52
1167	phenolic metabolism	1	55
1168	phenolic metabolisms	1	55
1170	phenolics	1	50
1171	phenols	3	128
1172	phenotype	1	77
1173	phenotypic variation	1	77
1174	phenylacetic acid	2	132
1175	phenylpropionic acid	1	51
1176	phenylpropionic acid derivative	2	132
1181	phylogeny	7	266
1183	physical chemistry	1	52
1185	physicochemical property	1	56
1188	physiological models	1	45
1189	physiological process	1	45
1191	physiologically based extraction tests	1	52
1192	physiology	18	637
1193	phytotherapy	1	77
1195	plankton	3	138
1198	plant extract	3	180
1199	plant extracts	4	257
1200	plant medicinal product	1	77
1202	plant seed	1	55
1205	plants (botany)	1	75
1206	plate count	1	46
1207	platelet derived growth factor beta receptor	1	69
1210	polyacrylamide gel electrophoresis	2	37
1211	polyferms model	1	49
1213	polylysine	1	45
1215	polymerase chain reaction	13	436
1217	polymorphism, genetic	1	53
1218	polyphenol	6	274
1219	polyphenol derivative	1	77
1220	polyphenols	9	387
1221	polysaccharide	6	148
1222	polysaccharides	3	118
1223	polysaccharides, bacterial	2	68
1224	population abundance	3	115
1226	porphyromonodaceae	1	53
1228	potential health	1	50
1230	prebiotic	1	45

1231	prebiotic agent	8	291
1233	prebiotics	12	356
1234	prematurity	1	51
1239	preterm infants	1	51
1241	prevotella	3	111
1242	prevotellaceae	2	86
1243	principal component analysis	3	135
1244	priority journal	14	494
1245	pro-inflammatory cytokines	1	45
1246	proanthocyanidin	2	59
1247	proanthocyanidins	2	59
1248	probes	1	45
1249	probiotic	8	261
1250	probiotic agent	11	412
1252	probiotics	13	430
1253	procedures	1	52
1258	propionates	2	49
1259	propionibacteriaceae	1	53
1260	propionic acid	7	231
1262	protein	3	96
1264	protein carbohydrate interaction	1	60
1265	protein degradation	2	71
1266	protein release	1	45
1267	protein secretion	1	69
1268	proteins	4	102
1269	proteobacteria	3	134
1271	protocatechuic acid	3	159
1272	public health	3	108
1275	pyrogallol	1	77
1277	pyrosequencing	3	117
1278	pyruvate synthase	1	51
1279	pyruvic acid	1	51
1283	quercetin arabinoside	1	75
1284	radionuclide imaging	1	44
1289	rats	2	41
1293	real time polymerase chain reaction	3	97
1294	real-time polymerase chain reaction	2	74
1295	recurrence	1	50
1296	red wine	3	130
1297	reduction	1	52
1298	reproducibility	4	139
1299	reproducibility of results	4	111
1302	restriction fragment length polymorphism	1	53
1303	retention time	1	46
1306	rhamnetin	1	75
1309	ribosome dna	1	60
1310	ribotype 027	1	50
1311	risk assessment	1	52
1312	risk to human health	1	52
1313	rna	1	56
1314	rna 16s	10	446
1315	rna extraction	1	51
1316	rna gene	1	53
1317	rna sequence	1	51
1318	rna, bacterial	1	52
1319	rna, ribosomal, 16s	9	423
1321	roseburia	4	179
1322	roseburia intestinalis	1	48

1323	ruminococcaceae	2	97
1325	rupture	1	44
1326	saccharomyces cerevisiae	3	73
1329	salts	1	51
1331	scanning electron microscopy	1	51
1333	screening	1	47
1334	secretion (process)	1	45
1335	seeds	1	55
1336	sequence analysis	1	33
1337	sequence analysis, dna	5	216
1338	sequence homology	1	60
1339	sequential extraction	1	52
1341	shear stress	1	54
1342	shime	6	245
1343	shime®	3	63
1344	short chain fatty acid	7	205
1345	short chain fatty acids	2	62
1349	short-chain fatty acids	5	154
1350	signal transduction	1	54
1353	simulation	4	188
1354	simulator	1	51
1361	sludge digestion	1	51
1362	small intestine	2	69
1365	soil	1	52
1366	soil analysis	1	52
1367	soil chemistry	1	52
1368	soil pollution	1	52
1369	soils	1	52
1371	solid	1	52
1373	solid phase extraction	1	75
1374	solubility	3	94
1378	solution and solubility	1	52
1379	solutions	1	52
1380	speciation (chemistry)	1	52
1381	species difference	2	83
1382	species differentiation	1	52
1383	species diversity	1	52
1384	species richness	2	66
1385	species specificity	2	101
1387	sphingomyelin phosphodiesterase	1	69
1390	stability	1	46
1391	staining and labeling	1	46
1393	staphylococcus	2	49
1394	starch	2	43
1396	static electricity	1	49
1398	steady state	3	159
1399	stomach	6	213
1401	stomach antrum	2	41
1402	stomach content	1	44
1403	stomach emptying	6	178
1405	stomach juice	5	169
1406	stomach mucosa	1	44
1408	stomach secretion	3	66
1409	storage time	1	56
1410	strain difference	1	60
1415	suidae	1	56

1417	surface microstructures	1	45
1419	surotomycin	1	50
1420	survival	3	52
1422	sus scrofa	1	56
1424	swine	3	107
1425	synbiotic agent	1	46
1426	synbiotics	2	75
1428	syringetin 3 galactoside	1	75
1429	syringic acid	2	104
1431	system simulating	1	56
1434	tablet disintegration	2	57
1437	tandem mass spectrometry	2	127
1439	tea	2	127
1440	technical difficulties	1	56
1442	temperature	2	45
1446	thearubigin	1	77
1447	theoretical model	6	208
1448	three dimensional cell culture	1	69
1450	thrombocyte function	1	77
1451	tim-2	2	72
1453	time factors	2	81
1454	tissue engineering	1	69
1457	toxicokinetic model	1	49
1458	toxicokinetics	1	49
1459	toxin analysis	1	47
1462	transcriptome	2	75
1463	transcriptomics	1	52
1464	transferase	1	51
1465	treatment outcome	1	50
1466	triacylglycerol lipase	1	51
1470	trypsin	1	51
1471	ulcerative colitis	1	52
1472	ultra performance liquid chromatography	1	55
1473	unclassified drug	7	407
1474	unindexed drug	1	77
1475	upper gastrointestinal tract	2	46
1476	upregulation	2	84
1477	urine	1	53
1479	vaccinium angustifolium	1	75
1480	vaccinium macrocarpon	1	55
1481	valeric acid	3	87
1482	valeric acid derivative	2	132
1483	valerolactone	1	77
1485	validation process	1	46
1486	validation study	2	86
1487	vancomycin	1	50
1488	variable diameter	1	45
1489	vascular endothelial cadherin	1	69
1491	vegetative growth	1	50
1494	viscosity	4	71
1496	vitaceae	3	97
1497	vitis	3	134
1499	volatile fatty acid	5	188

	1500	volatile fatty acids	4	106
	1501	volunteers	1	53
	1502	von willebrand factor	1	69
	1506	wild blueberries	1	75
	1507	wild blueberry	1	75
	1508	wine	5	167
	1510	x ray absorption	1	52
	1511	x ray absorption near edge structure spectroscopy	1	52
	1512	x ray absorption spectroscopy	1	52
	1513	x-ray absorption near-edge structure	1	52
	1514	x-ray absorption spectroscopy	1	52
	1515	xenobiotics	1	49
	1516	xylan	2	93
	1517	xylan 1,4 beta xylosidase	1	60
	1518	xylan endo 1,3 beta xylosidase	1	60
	1519	xylans	2	93
	1521	yeast	3	123
	1522	yeast fermentate	1	45
	1523	young adult	2	83
**2016-2020**				
	1	1,3 propanediol	1	36
	2	2 amino 1 methyl 6 phenylimidazo[4,5 b]pyridine	1	59
	3	8 prenylnaringenin	1	32
	4	8-prenylnaringenin	1	32
	5	abiotic factors	1	72
	6	acidaminococcaceae	2	99
	7	adhesion	1	58
	8	adhesion related prebiotic index	1	58
	9	administration, oral	1	49
	10	adsorption	1	47
	11	adult	7	271
	12	aerobe	1	59
	13	aerobic bacterium	1	49
	14	agar	1	58
	15	agar diffusion	1	54
	16	aged	1	36
	17	aged, 80 and over	1	36
	18	akkermansia	1	44
	19	alcohol	1	36
	20	aldehydes	1	36
	21	alginate	1	49
	22	alginates	1	49
	23	alginic acid	1	49
	24	alkylation	1	75
	25	all metal	1	57
	26	alpha galactosidase	1	49
	27	alpha glucosidase	1	49
	28	ambrotose	1	43
	29	ammonia	4	218
	30	amoxicillin	1	49
	31	amylase	1	47
	32	anaerobe	2	117
	33	anaerobic bacteria	1	30
	34	anaerobic bacterium	4	171
	35	anaerobic digestion	1	23

36	anaerobic fermentation	1	37
37	anaerobic growth	1	72
38	anaerobic microflora	1	72
39	anaerobiosis	2	102
40	animal	2	137
41	animal experiment	1	65
42	animal health	1	72
43	animal model	1	65
44	animalia	1	72
45	animals	4	226
46	anoxic conditions	1	55
47	anti-bacterial agents	1	49
48	anti-infective agents	1	46
49	antibacterial activity	1	54
50	antibiotic resistance	1	33
51	antibiotics	2	121
52	antibodies, monoclonal	1	25
53	antiinfective agent	1	36
54	antimicrobial activity	1	49
55	antimony	1	75
56	apppa	1	49
57	approximation algorithms	1	35
58	arabinofuranosidase	1	43
59	arabinoxylan	3	146
60	arabinoxylan oligosaccharides	1	43
61	arctic	1	47
62	arctic brownfields	1	47
63	aromatic compound	1	46
64	aromatic hydrocarbons	1	47
65	arsenic	3	181
66	arsenic concentration	1	57
67	arsenic release	1	57
68	arsine	1	75
69	article	40	1668
70	artificial gut	1	49
71	artificial milk	1	46
72	ascending colon	3	149
73	atopobium	2	104
74	avena	1	55
75	avena sativa	1	55
76	baby food	1	46
77	bacilli	1	29
78	bacilli (class)	1	29
79	bacteria	14	696
80	bacteria (microorganisms)	13	495
81	bacteria, anaerobic	1	46
82	bacterial adhesion	2	88
83	bacterial colonization	2	91
84	bacterial count	5	241
85	bacterial disease	1	49
86	bacterial growth	3	102
87	bacterial load	2	72
88	bacterial metabolism	4	186
89	bacterial physiological phenomena	2	88
90	bacterial strain	3	115
91	bacterial survival	4	199
92	bacterial viability	2	101

93	bacteriocin	1	54
94	bacteriocins	2	87
95	bacteriological techniques	3	133
96	bacteriology	3	177
97	bacteriotherapy	1	65
98	bacterium	12	590
99	bacterium adherence	1	58
100	bacterium culture	6	246
101	bacterium examination	1	43
102	bacterium isolation	1	33
103	bacteroidaceae	1	43
104	bacteroides	7	311
105	bacteroides (class)	1	44
106	bacteroides fragilis	1	72
107	bacteroides sp.	1	44
108	bacteroidetes	3	116
109	barley	1	55
110	beef	1	35
111	beer	1	32
112	beta galactosidase	1	49
113	beta glucan	1	55
114	beta glucan hydrolase	1	55
115	beta glucosidase	1	49
116	beta glucuronidase	1	49
117	beta-glucans	1	55
118	bicarbonate	1	54
119	bifidobacteria	3	159
120	bifidobacteriaceae	3	140
121	bifidobacterium	13	514
122	bifidobacterium dentium	1	33
123	bifidobacterium thermophilum	1	33
124	bile acid	1	18
125	bile acids and salts	2	101
126	bile salt	2	101
127	bile salts	1	47
128	bio reactor	1	47
129	bioaccessibility	3	141
130	bioaccessible	1	57
131	bioaccumulation	2	104
132	bioactivation	1	31
133	bioactivity	2	108
134	bioassay	2	115
135	bioavailability	7	315
136	bioavailable	1	35
137	biochemical composition	1	72
138	biochemistry	1	47
139	biodiversity	4	162
140	biofilm	1	30
141	biofilms	1	30
142	biological activity	1	18
143	biological availability	4	161
144	biological model	8	299
145	biological monitoring	1	55
146	biological organs	2	100
147	biological uptake	3	151
148	biomedical engineering	1	65
149	bioreactor	7	362
150	bioreactor equipment	1	33

151	bioreactor systems	1	47
152	bioreactors	6	296
153	biosynthesis	1	36
154	biotransformation	3	125
155	biovolatilization	1	75
156	bismuth	1	75
157	blood	1	65
158	blood urea nitrogen	1	65
159	body fluids	2	105
160	body weight	2	108
161	bovinae	2	97
162	brownfield site	1	47
163	brownfields	1	47
164	bulk fraction	1	57
165	butanediol	1	31
166	butylene glycols	1	31
167	butyrate	1	18
168	butyrates	1	43
169	butyric acid	2	79
170	canada	1	57
171	cancer	2	106
172	cancer prevention	1	59
173	cancer risk	1	47
174	canning	1	47
175	capsules	1	49
176	carbohydrate	6	273
177	carbohydrate metabolism	3	109
178	carbohydrates	2	121
179	carboxylic acid	2	85
180	carboxylic acids	1	41
181	casein	1	25
182	caseins	1	25
183	cattle	2	107
184	cell adhesion	1	49
185	cell assay	1	46
186	cell immobilization	1	41
187	cells, immobilized	4	172
188	centrifugation	1	35
189	chemistry	2	119
190	chemoprophylaxis	2	105
191	chemostat	1	41
192	chicken	1	35
193	chickens	1	35
194	chicory	2	103
195	child	2	82
196	child health	1	49
197	child, preschool	2	82
198	chromatographic analysis	1	75
199	chromatography, gas	1	37
200	chromatography, high pressure liquid	2	80
201	chronic kidney disease	1	65
202	chronic kidney failure	1	65
203	cichorium intybus	1	59
204	clostridia	3	163
205	clostridium	8	340
206	clostridium coccoides	2	94
207	clostridium histolyticum	3	127
208	clostridium perfringens	2	72
209	cluster analysis	1	29

210	coliform bacterium	1	41
211	colon	18	795
212	colon biopsy	1	30
213	colon flora	6	222
214	colon simulator	1	36
215	colon, ascending	1	49
216	colon, descending	1	49
217	colon, transverse	1	49
218	colonic fermentation	2	66
219	colonization	2	102
220	colonization process	1	44
221	colony count, microbial	10	457
222	colony forming unit	1	65
223	comet assay	1	46
224	commensal	1	58
225	community composition	1	43
226	comparative study	8	349
227	complex carbohydrates	1	72
228	computer model	1	23
229	computer simulation	4	216
230	computers	1	49
231	concentration (parameters)	4	231
232	concentration (process)	1	57
233	concentration response	1	49
234	confocal microscopy	1	30
235	contaminated sites	1	47
236	contaminated soils	1	57
237	contamination	1	57
238	continuous culture	1	46
239	controlled study	13	561
240	cooked food	1	35
241	cooking	1	35
242	coriobacteriales	1	35
243	cresol	1	45
244	crohn disease	1	46
245	culture media	3	151
246	culture medium	2	102
247	daidzein	1	18
248	dairy products	1	47
249	data interpretation, statistical	1	35
250	data processing	1	58
251	daucus carota	1	20
252	decanol	1	46
253	demethylation	1	32
254	denaturing gradient gel electrophoresis	6	268
255	descending colon	2	77
256	deuterium	1	47
257	dialysis	1	65
258	diet	2	63
259	diet restriction	1	35
260	diet supplementation	3	147
261	dietary fiber	1	44
262	dietary supplements	1	43
263	digestion	10	307
264	digestive system	5	251
265	digestive system disorder	1	49

266	disease activity	1	46
267	disease control	1	65
268	diseases	1	47
269	dissolution	2	96
270	distal regions	1	44
271	dna damage	1	46
272	dna fingerprinting	3	149
273	dna isolation	1	30
274	dna, bacterial	4	173
275	drug cytotoxicity	1	46
276	drug effect	2	108
277	drug formulation	1	65
278	duodenum	1	54
279	duodenum secretion	1	54
280	dynamic gastric model	1	16
281	dynamic systems	1	23
282	eating	1	57
283	ecosystem	7	385
284	ecosystem modeling	1	49
285	ecosystems	1	57
286	ecotoxicology	1	75
287	egg	1	25
288	electrokinesis	2	89
289	electrophoresis, gel, two-dimensional	1	46
290	empetrum nigrum	1	49
291	enterobacteriaceae	2	90
292	enterococcus	3	150
293	enterolactone	1	31
294	enumeration	1	37
295	environmental matrixes	1	75
296	enzyme activation	1	49
297	enzyme activity	1	49
298	enzyme substrate	1	37
299	enzymes	1	49
300	equol	1	18
301	ericaceae	1	49
302	erratum	1	2
303	escherichia coli	3	118
304	ester	1	55
305	estrogen	2	63
306	estrogens	1	31
307	eubacterium	3	149
308	eubacterium rectale	2	94
309	evaluation	1	65
310	evaluation studies	1	65
311	exopolysaccharide	1	41
312	exopolysaccharides	1	41
313	experimental model	1	58
314	experimental study	4	195
315	exponential phase	1	47
316	extracellular matrix	1	49
317	extraction	1	49
318	faecalibacterium prausnitzii	1	43
319	fatty acid	2	114
320	fatty acids	2	127
321	fatty acids, volatile	6	291
322	fecal coliform	3	159
323	feces	12	496
324	feces analysis	2	47
325	feces microflora	8	374
326	feed additives	1	72

327	feeding behavior	1	35
328	female	4	188
329	fermentation	19	855
330	fermentation medium	1	46
331	fermentation model	1	49
332	fermentors	1	72
333	fiber	2	54
334	fibruline	1	59
335	filtration	1	35
336	firmicutes	1	29
337	fishes	1	35
338	flax seed	1	31
339	flow cytometry	2	85
340	fluids	1	47
341	fluorescence	1	46
342	fluorescence in situ hybridization	5	242
343	fluorometry	1	55
344	food	2	84
345	food allergy	1	25
346	food analysis	1	37
347	food composition	2	80
348	food disintegration	1	20
349	food industry	1	47
350	food intake	2	67
351	food matrixes	1	47
352	food microbiology	2	84
353	food microbiotechnology	1	47
354	food preservation	1	47
355	formula-fed infants	1	46
356	fructan	2	90
357	fructans	2	90
358	fructo-oligosaccharides	1	41
359	fructose oligosaccharide	2	87
360	frying	1	35
361	fugacity	1	47
362	functional food	3	107
363	fungus	1	59
364	galactans	1	43
365	gas	1	37
366	gas chromatography	2	112
367	gases	1	37
368	gasphase	1	75
369	gastric emptying	1	20
370	gastrointestinal absorption	2	65
371	gastrointestinal contents	1	47
372	gastrointestinal model	5	275
373	gastrointestinal resource management	1	43
374	gastrointestinal tract	12	473
375	gastrointestinal tract (git)	1	47
376	gastrointestinal tract simulator	1	47
377	gastrointestinal transit	2	53
378	genetic selection	1	44
379	genotoxic	1	59
380	genotoxicity	2	105

381	geologic models	1	47
382	germanium	1	75
383	gi enzymes	1	49
384	gi model	1	49
385	gi models	1	49
386	git model	1	47
387	glucan	1	36
388	glucans	1	36
389	glucose	1	37
390	glucoside	1	31
391	glucosides	1	31
392	glyceraldehyde	1	36
393	glycerol	1	36
394	glyconutrients	1	43
395	goldenville gold mine	1	57
396	gram positive bacterium	1	46
397	gram-positive bacteria	1	46
398	growth curve	1	46
399	growth medium	1	47
400	growth performance	1	72
401	growth promoters	1	72
402	growth substances	1	41
403	growth, development and aging	5	201
404	gut	1	58
405	gut bacteria	1	18
406	gut microbiota	1	44
407	half time (t1/2)	1	20
408	hand	1	47
409	hardness	1	20
410	health food	1	36
411	health hazard	1	47
412	health impact	1	58
413	health risk	1	47
414	health risks	1	47
415	heterocyclic amines	1	59
416	high concentration	1	72
417	high performance liquid chromatography	3	138
418	high resolution	1	44
419	high resolution analysis	1	44
420	hominid	1	75
421	hops	1	32
422	hordeum	1	55
423	host	1	58
424	human	21	840
425	human cell	2	121
426	human colonic bacteria	1	59
427	human engineering	1	49
428	human experiment	1	36
429	human fecal	1	44
430	human gut	1	35
431	human gut model	1	46
432	human guts	1	75
433	human health risk assessment	1	47
434	human intestinal microbial ecosystem	1	44
435	human tissue	3	199
436	humans	33	1396

437	humulus lupulus	1	32
438	humulus lupulus extract	1	32
439	hybridization	1	46
440	hydrogen peroxide	1	46
441	hydrogen-ion concentration	3	120
442	hydrogenation	1	75
443	hydrolysis	1	65
444	hydrophobicity	1	58
445	hypothesis testing	2	96
446	ileum	1	54
447	imidazoles	1	59
448	immobilised faecal microbiota	1	46
449	immobilization	2	85
450	immobilized cell	1	41
451	immobilized cell culture	1	46
452	immobilized cell reactor	1	46
453	immobilized cells	1	49
454	immobilized fecal microbiota	1	46
455	immobilized intestinal microbiota	1	36
456	immunology	1	25
457	in situ hybridization	1	46
458	in situ hybridization, fluorescence	4	174
459	in situ test	1	46
460	in vitro colon model	1	46
461	in vitro continuous fermentation model	1	49
462	in vitro digestion	2	60
463	in vitro digestive model	1	54
464	in vitro fermentation	1	55
465	in vitro fermentation model	1	36
466	in vitro model	1	18
467	in vitro modeling	1	47
468	in vitro study	21	1021
469	in vitro techniques	1	72
470	in vivo study	1	65
471	in-vitro	6	344
472	in-vivo	2	116
473	in-vivo experiments	1	72
474	incubation time	1	75
475	inductively coupled plasma	1	75
476	inductively coupled plasma mass spectrometry	1	75
477	inductively coupled plasma method	1	75
478	industrial chemicals	1	47
479	industrial waste	1	49
480	infant	4	158
481	infant food	1	46
482	infant nutrition	1	46
483	inflammatory bowel disease	1	46
484	inflammatory bowel disorder	1	46

485	ingestion	1	47
486	ingestion (engines)	1	47
487	inoculation	8	334
488	inorganic species	1	75
489	interference competition	1	59
490	intestinal	1	31
491	intestinal bacteria	1	32
492	intestinal microbiota	2	79
493	intestinal mucosa	2	88
494	intestinal tract	2	119
495	intestine	6	278
496	intestine absorption	2	56
497	intestine flora	9	438
498	intestine fluid	1	47
499	intestine function	2	69
500	intestine mucosa	1	23
501	intestine parameters	1	65
502	intestine, large	2	47
503	intestine, small	1	57
504	intestines	5	264
505	inulin	7	338
506	isolation and purification	3	95
507	jejunum	1	54
508	kidney failure, chronic	1	65
509	kinetics	1	20
510	lachnospiraceae	1	43
511	lactates	1	72
512	lactic acid	3	103
513	lactic acid bacterium	1	41
514	lactic acid derivative	1	72
515	lactobacillaceae	1	43
516	lactobacilli	1	44
517	lactobacillus	9	415
518	lactobacillus acidophilus	1	47
519	lactobacillus casei	3	96
520	lactobacillus casei lc01	1	4
521	lactobacillus casei shirota	1	4
522	lactobacillus delbrueckii	1	5
523	lactobacillus delbrueckii subsp. bulgaricus	1	5
524	lactobacillus gasseri	1	33
525	lactobacillus johnsonii	1	47
526	lactobacillus johnsonii ncc 533	1	47
527	lactobacillus reuteri	1	36
528	lactobacillus reuteri atcc 55730	1	36
529	lactobacillus rhamnosus	4	162
530	lactobacillus sp.	1	49
531	lactoglobulin	1	25
532	lactoglobulins	1	25
533	lanthanum	1	47
534	large intestine	3	70
535	lead	2	110
536	lead poisoning	1	35

537	lifespan	1	65
538	ligand	1	49
539	lignan	1	31
540	lignans	1	31
541	linum usitatissimum	1	31
542	lipid	1	72
543	lipid sources	1	72
544	liquid chromatography	1	59
545	listeria	1	54
546	listeria ivanovii	1	54
547	macromolecular substances	1	31
548	macromolecule	1	31
549	maize	1	18
550	male	5	235
551	mass fragmentography	1	46
552	mass spectrometers	1	75
553	mass spectrometry	1	75
554	mathematical analysis	1	23
555	mathematical model	1	23
556	meat	1	35
557	mercury	1	75
558	mercury (metal)	1	75
559	metabolic activity	1	72
560	metabolism	12	506
561	metabolite	2	134
562	metabolite concentrations	1	72
563	metabolite production	1	72
564	metabolites	2	147
565	metagenome	8	272
566	metal	1	75
567	metal concentrations	1	57
568	metal recovery	1	49
569	metals	2	132
570	methodology	1	58
571	methylation	1	75
572	micro-array	1	18
573	microarray analysis	1	29
574	microbial activities	1	49
575	microbial activity	4	208
576	microbial colonization	1	44
577	microbial communities	1	44
578	microbial community	10	490
579	microbial community composition	1	44
580	microbial diversity	2	75
581	microbial dynamics	1	44
582	microbial eco system	4	225
583	microbial ecology	3	159
584	microbial groups	1	72
585	microbial immunity	1	32
586	microbial levels	1	72
587	microbial metabolism	2	63
588	microbial morphology	1	43
589	microbial population dynamics	1	45

590	microbial sensitivity tests	1	49
591	microbial transformation	1	75
592	microbial viability	3	117
593	microbiology	10	419
594	microbiota	5	206
595	microbiotas	3	173
596	microcapsule	1	49
597	microcin b17	1	33
598	microflora	4	185
599	microorganism	2	100
600	microorganisms	3	168
601	microscopy, confocal	2	76
602	microspheres	1	46
603	microstructure	1	49
604	middle aged	1	36
605	milk	2	79
606	mine tailings	2	106
607	mine waste	2	106
608	mineral oils	1	47
609	mining	2	106
610	mobility	1	47
611	model	2	93
612	modeling	2	85
613	models, anatomic	1	49
614	models, biological	21	882
615	models, theoretical	2	116
616	modulation	1	58
617	molecular biology	1	30
618	molecular weight	2	102
619	monoclonal antibody	1	25
620	montague gold mine	1	57
621	mucin	1	58
622	mucins	1	58
623	mucus	2	81
624	mutagens	1	59
625	nephrectomy	1	65
626	nonhuman	21	922
627	normal human	4	158
628	north america	1	57
629	nova scotia	1	57
630	nucleic acid denaturation	1	46
631	nucleotide sequence	2	88
632	numerical model	1	23
633	nutrient availability	1	65
634	nutrition	2	72
635	nutritional state	1	49
636	nutritional status	1	49
637	oat	1	55
638	oligonucleotide array sequence analysis	1	29
639	oligosaccharide	1	45
640	oligosaccharides	4	173
641	optimization	1	35
642	oral delivery	1	49
643	organic acid	1	41
644	organic carbon	1	47
645	organic pollutant	1	47
646	organochlorine	1	47
647	organochlorine pesticides	1	47
648	ovalbumin	1	25
649	pah	2	94

650	pah compounds	1	47
651	pancreatin	1	54
652	particle size	3	124
653	pb	1	35
654	pectin	1	49
655	pediocin	1	54
656	pediocin pa 1	1	54
657	pediococci	1	54
658	pediococcus	1	54
659	pediococcus acidilactici	1	54
660	pepsin a	1	54
661	pesticide	1	47
662	ph	5	279
663	ph measurement	1	33
664	phase separation	1	47
665	phase transition	1	75
666	phenobarbital	1	58
667	phenol	1	45
668	phosphate	1	49
669	phosphate buffered saline	1	58
670	phosphatidylcholine	1	25
671	phosphatidylcholines	1	25
672	phylogeny	2	59
673	physicochemical property	1	54
674	physiological models	1	35
675	physiological states	1	47
676	physiologically based extraction tests	1	49
677	physiology	5	155
678	phytoestrogen	1	32
679	phytoestrogens	2	63
680	pilot projects	1	65
681	pilot study	1	65
682	plant gums	1	43
683	plants	1	43
684	plate count	1	46
685	pollution exposure	1	47
686	polycyclic aromatic hydrocarbon	2	94
687	polycyclic aromatic hydrocarbons	2	94
688	polycyclic hydrocarbons, aromatic	1	47
689	polydextrose	1	36
690	polylysine	1	49
691	polymerase chain reaction	4	193
692	polymerization	3	144
693	polymers	1	44
694	polyol	1	37
695	polysaccharide	4	201
696	polysaccharides	2	101
697	polysaccharides, bacterial	1	41
698	population	1	46
699	population density	1	43
700	porphyromonas	1	43
701	prebiotic	5	177
702	prebiotic agent	9	418
703	prebiotics	5	226

704	prenylflavonoid isoxanthohumol	1	32
705	preschool child	1	33
706	prescreening	1	72
707	prevotella	4	186
708	priority journal	13	459
709	probiotic	3	67
710	probiotic agent	6	268
711	probiotic bacteria	1	47
712	probiotic products	1	47
713	probiotics	8	306
714	process model	1	49
715	process simulations	1	47
716	production rates	1	75
717	productivity	1	75
718	propane	1	36
719	propanol	1	46
720	propionate	1	55
721	propionic acid	2	99
722	propionic acids	1	43
723	protein	1	45
724	protein degradation	3	143
725	protein denaturation	1	45
726	protein intake	1	45
727	protein stability	1	54
728	proteins	1	45
729	proton nuclear magnetic resonance	1	46
730	public health	2	106
731	pyrimidines	1	59
732	quantitative analysis	3	134
733	quaternary ammonium compounds	1	43
734	rat	1	65
735	rats	1	65
736	rats, sprague-dawley	1	65
737	reactor	2	77
738	real time polymerase chain reaction	1	46
739	realtime pcr	1	44
740	reference soil	1	49
741	regional planning	1	44
742	regression analysis	1	47
743	regression model	1	47
744	reproducibilities	1	44
745	reproducibility	1	46
746	retracted article	1	2
747	reuterin	1	36
748	ribosome subunits, small, bacterial	1	29
749	rifamycins	1	46
750	rifaximin	1	46
751	risk assessment	4	188
752	risk management	1	49
753	risk perception	2	96
754	roseburia	1	43
755	ruminococcus	1	55
756	saline water	1	58
757	salmon	1	35
758	salmonella	2	82
759	salmonella infections	2	82
760	salmonella serovar typhimurium	1	49

761	salmonella typhimurium	2	82
762	salmonellosis	1	49
763	salts	1	47
764	scoring system	1	58
765	screening	1	45
766	seal harbour gold mine	1	57
767	secoisolariciresinol	1	31
768	secoisolariciresinol diglucoside	1	31
769	selection, genetic	1	44
770	selenium	1	75
771	separation	1	35
772	shime	6	241
773	short chain fatty acid	6	277
774	short-chain fatty acids	2	108
775	shrub	1	49
776	sieves	1	47
777	simulation	5	228
778	simulator	6	323
779	simulator of the human intestinal microbial ecocsystem	1	18
780	simulator of the human intestinal microbial ecosystem	1	75
781	simulator of the human intestinal microbial ecosystem (shime)	2	112
782	simulators	2	106
783	site-specific	1	49
784	size fraction	2	106
785	small intestine	3	153
786	small subunit ribosomal rna	1	29
787	soil	4	178
788	soil analysis	2	82
789	soil concentrations	1	47
790	soil ingestion	1	35
791	soil microflora	1	65
792	soil organic carbon	1	47
793	soil organic matter	1	47
794	soil pollutant	2	104
795	soil pollutants	2	92
796	soil pollution	2	104
797	soil profile	1	47
798	soil quality	1	47
799	soil size fractions	1	47
800	soils	5	235
801	solid	1	47
802	solid phase microextraction	1	46
803	solid-phase	1	47
804	sorption	1	47
805	soy germ	1	18
806	species	1	75
807	species composition	1	43
808	spike wave	1	75
809	sporosarcina pasteurii	1	65
810	sprague dawley rat	1	65
811	staphylococcus	3	154

812	staphylococcus sp.	1	49
813	steady state	1	44
814	sterile conditions	1	49
815	stomach	5	186
816	stomach absorption	1	33
817	stomach content	1	49
818	stomach emptying	1	20
819	stomach juice	2	112
820	stomach model	1	20
821	stomach ph	1	54
822	stomach secretion	1	54
823	streptococcus	1	29
824	sucrose	1	37
825	sulfur	1	75
826	surface properties	1	58
827	surface tension	1	58
828	survival	3	166
829	suspension	1	75
830	tailings	2	106
831	tailings disposal	1	49
832	tandem mass spectrometry	1	25
833	tellurium	1	75
834	tellurium compounds	1	75
835	tellurium derivative	1	75
836	temperature	1	72
837	temperature effect	1	72
838	theoretical model	2	116
839	three-stage in vitro colon model	2	87
840	tim-1	1	54
841	tin	1	75
842	toxicity	1	75
843	toxicology	1	75
844	toxin	1	54
845	trace metal	1	75
846	transverse colon	1	59
847	triacylglycerol lipase	1	54
848	trophic chain	1	23
849	trophic level	1	23
850	ultrafiltration	1	35
851	unclassified drug	5	217
852	upper gastrointestinal tract	2	70
853	urban area	1	47
854	urban growth	1	47
855	urea	1	65
856	urea nitrogen blood level	1	65
857	urease	1	65
858	uremia	1	65
859	uremic intoxication	1	65
860	valeric acid	1	35
861	validation	1	46
862	validation study	1	72
863	validity	1	46
864	veterinary medicine	1	72
865	viability	2	59
866	viscosity	1	58
867	volatile agent	1	75
868	volatile fatty acid	2	108
869	volatile species	1	75
870	volatilization	1	75
871	water	1	58
872	xanthohumol	1	32

	873	xylan 1,4 beta xylosidase	1	43
	874	xylan endo 1,3 beta xylosidase	1	43
	875	xylans	2	88
	876	xylitol	1	36
	877	zea mays	1	18
	878	beta-glucans	1	55
**2021-2022**				
	4	16s rrna gene sequencing	4	157
	5	16s rrna sequencing	4	142
	14	4 aminobutyric acid	1	73
	16	4-hydroxy-2-hexenal	2	60
	17	4-hydroxy-2-nonenal	2	60
	23	absorption	2	104
	30	acetates	2	82
	31	acetic acid	10	404
	32	acetic acid derivative	3	195
	34	acetobacter	1	78
	40	acidaminococcus	3	129
	41	acidification	2	53
	43	actin filament	1	77
	44	actinobacteria	5	293
	49	administration and dosage	3	141
	51	adult	33	1249
	58	aerobic bacteria	2	60
	59	aerobic metabolism	1	73
	60	aerobiosis	1	73
	66	aged	3	124
	70	aging	3	78
	73	akkermansia	4	93
	74	akkermansia muciniphila	6	181
	76	alcohol	2	118
	77	alcohols	2	74
	78	aldehyde	2	60
	79	aldehydes	2	60
	81	alginate	2	42
	84	algorithm	1	77
	88	alkylation	5	225
	93	alpha lactalbumin	2	60
	99	amino acid	3	114
	103	amino acids	4	137
	104	ammonia	10	405
	106	ammonium compounds	4	134
	108	ammonium derivative	4	134
	111	ampicillin	2	66
	112	amplicon	5	250
	114	amylase	4	132
	117	anaerobic bacterium	4	156
	119	anaerobic cultivation	1	78
	121	anaerobic fermentation	2	98
	122	anaerobic growth	3	189
	123	anaerobiosis	3	189
	125	analysis	10	395
	129	anatomy	1	86
	131	animal	27	792
	133	animal experiment	5	198
	136	animal model	3	115
	138	animal tissue	3	84

139	animalia	3	127
140	animals	27	792
141	anoxic conditions	1	73
143	anti-bacterial agents	2	68
148	anti-inflammatory effects	1	72
150	anti-oxidant activities	4	99
153	antibiotic resistance	5	193
155	antibiotic sensitivity	2	66
157	antigens, cd	1	86
158	antiinfective agent	2	68
159	antiinflammatory activity	3	164
160	antiinflammatory agent	2	122
165	antiobesity agent	1	72
166	antioxidant	7	197
168	antioxidant capacity	2	50
169	antioxidants	7	209
172	apple	1	74
174	apples	1	74
184	arsenates	2	102
185	arsenic	6	289
186	arsenic acid	2	102
187	arsenic acid derivative	2	102
189	arsenic speciation analysis	1	69
191	arsenic trioxide	4	207
192	arsenicals	2	102
193	arsenite	2	81
194	arsenites	3	159
197	arsenous acid derivative	3	159
200	article	85	3378
204	ascending colon	10	422
209	atopobium	1	78
211	bacilli	5	168
212	bacillus	3	197
214	bacillus coagulans	5	197
218	bacillus subtilis	2	71
219	bacteria	46	1659
220	bacteria (microorganisms)	9	331
223	bacterial colonization	3	167
225	bacterial count	7	339
227	bacterial dna	9	471
229	bacterial fermentations	2	80
230	bacterial gene	1	103
231	bacterial growth	4	188
233	bacterial load	3	114
234	bacterial metabolism	2	97
235	bacterial microbiome	1	81
236	bacterial phenomena and functions	2	62
237	bacterial physiological phenomena	2	62
238	bacterial polysaccharide	2	121
239	bacterial population	2	59
241	bacterial rna	3	196
242	bacterial spore	2	80

243	bacterial strain	6	316
246	bacterial viability	3	100
247	bacterial virulence	2	131
254	bacterium	42	1592
255	bacterium adherence	3	94
257	bacterium culture	6	310
258	bacterium identification	2	66
259	bacterium isolate	2	66
260	bacterium isolation	2	126
261	bacteroidaceae	7	312
262	bacteroides	14	619
263	bacteroides caccae	1	73
266	bacteroides sp.	2	66
267	bacteroides thetaiotaomicron	2	79
270	bacteroidetes	12	561
273	batch cell culture	4	195
274	batch cell culture techniques	3	142
276	batch fermentation	3	132
286	benzoic acid	2	90
294	beta lactoglobulin	2	60
297	betaproteobacteria	2	118
299	beverages	3	72
300	bifidobacteria	4	251
302	bifidobacteriaceae	3	181
303	bifidobacterium	24	921
305	bifidobacterium bifidum	4	153
307	bifidobacterium longum	4	163
308	bifidobacterium longum bb-46	2	100
315	bile	4	114
316	bile acid	4	149
317	bile acids and salts	3	110
318	bile salt	2	73
320	bilophila	2	67
322	bioaccessibility	18	466
323	bioaccumulation	2	77
324	bioactive compounds	3	88
325	bioactive peptides	2	65
327	bioavailability	19	633
328	biochemical analysis	2	68
329	biochemistry	4	108
332	biodegradation	2	72
334	biodiversity	5	211
335	biofilm	3	136
339	bioinformatics	5	178
340	biological activity	2	86
341	biological availability	13	379
342	biological marker	3	145
343	biological materials	3	45
344	biological model	39	1277
347	biomarkers	3	145
348	biomass	3	113
350	biomimetic material	3	96
351	biomimetic materials	3	96
353	biomolecules	5	205
356	bioreactor	9	231
358	bioreactors	8	194
363	biosurfactant	1	77
364	biosynthesis	7	260
365	biotechnology	2	109

366	biotransformation	4	159
372	blautia	2	125
374	blood	2	63
381	body fluids	6	159
384	bovine	2	56
385	brain	1	81
389	bread	4	106
391	breast milk	3	113
402	butyrate	2	73
403	butyrates	13	594
404	butyric acid	22	917
405	butyric acid derivative	13	594
407	byproducts	2	87
409	c57bl mouse	3	125
410	caco 2 cell line	3	161
411	caco-2	3	53
412	caco-2 cell line	15	625
413	caco-2 cells	15	625
415	caco-2 cell	1	69
416	cacodylic acid	2	102
417	cadherin	1	86
418	cadherins	1	86
419	cadmium	2	51
420	caffeic acid	3	132
422	caffeine	2	77
430	canada	1	74
432	cancer risk	2	71
433	candida	1	86
434	candida albicans	1	86
437	capillary ion chromatography	1	103
440	carbohydrate	2	156
443	carbohydrate metabolism	2	88
444	carbohydrate polymers	1	103
446	carbon source	1	103
450	carboxymethylcellulose	2	117
455	carotenoid	3	47
456	carotenoids	3	47
457	casein	4	126
459	caseins	3	98
461	catechin	4	145
464	cattle	2	56
466	cd4+ t lymphocyte	1	73
471	cell assay	1	77
473	cell culture	6	260
474	cell culture technique	4	155
475	cell culture techniques	2	70
478	cell differentiation	1	73
480	cell growth	1	73
485	cell membrane permeability	1	86
487	cell motion	1	86
488	cell movement	1	86
489	cell proliferation	2	79
491	cell survival	2	81
492	cell suspension	1	73
494	cell viability	5	289
496	cells	3	81
497	cells and cell components	3	184
500	cellulose	3	189

505	chains	2	83
510	chemical analysis	3	78
512	chemical contamination	2	77
516	chemical phenomena	2	48
518	chemical structure	2	56
519	chemistry	59	1917
520	chemistry, pharmaceutical	2	62
527	chemotherapy	2	53
530	chicken	2	77
531	chickens	2	77
532	child	3	120
535	china	5	124
537	chloramphenicol	2	66
538	chlorogenic acid	2	96
540	chlorpyrifos	2	63
544	christensenella	1	78
545	chromatography by mobile phase	1	103
546	chromatography, gas	1	103
547	chromatography, high pressure liquid	5	150
548	chromatography, liquid	2	46
550	chromatography, thin layer	3	185
559	ciprofloxacin	2	64
560	citric acid	1	73
561	citric acid cycle	1	73
562	citrobacter	3	152
563	citrus	5	187
564	citrus fruits	4	85
567	citrus limon	1	72
572	classification	22	764
579	clindamycin	2	74
580	clinical article	3	155
581	clinical assessment	2	89
582	clinical evaluation	4	163
584	clinical trial	2	60
585	cloacibacillus	1	78
589	clostridium	9	335
595	cluster analysis	5	238
600	cobalamin	2	67
603	coculture	3	125
604	coculture techniques	3	125
610	collagen	1	73
611	collagenase 3	1	81
612	collinsella	4	273
614	colon	44	1654
619	colon flora	5	217
622	colon mucosa	2	91
627	colonic fermentation	5	82
629	colony count, microbial	3	173
630	colony forming unit	3	122
635	commensal	1	73
639	community composition	2	57
640	community dynamics	2	87
641	community structure	5	240
643	comparative study	11	430
645	complementary mechanisms	2	57
652	computer simulation	6	177

653	concentration (composition)	2	75
655	concentration (parameters)	5	178
659	confocal microscopy	2	57
663	consensus development	2	70
671	contaminated soils	2	105
675	continuous culture	1	78
678	controlled study	48	1971
682	cooking	5	113
683	copper	3	78
685	coprococcus	3	228
690	correlation analysis	3	168
706	culture techniques	2	85
707	cultured milk products	2	115
711	cyanobacterium	1	81
714	cysteine	3	115
715	cytokine	4	181
716	cytokine production	2	100
717	cytokine release	2	106
718	cytokines	3	154
719	cytology	4	167
720	cytotoxicity	3	123
724	dairies	2	53
725	dairy products	3	90
728	data analysis	1	81
730	degradation	4	207
735	deinococcales	1	103
736	deinococci	1	103
737	deinococcus	1	103
738	deinococcus-thermus	1	103
741	denaturing gradient gel electrophoresis	2	64
742	dendritic cell	1	86
744	descending colon	14	604
746	desulfovibrio	2	88
747	detection method	2	68
752	dialister	2	145
754	diet	6	254
755	diet composition	1	77
756	diet restriction	2	62
757	diet supplementation	2	78
758	diet, high-fat	2	49
761	dietary emulsifiers	1	77
763	dietary fats	2	57
764	dietary fiber	17	615
768	dietary proteins	4	105
769	dietary supplement	6	192
771	dietary supplements	6	196
776	digestion	51	1458
780	digestive enzymes	2	63
783	digestive system	18	811
790	dimethylarsinic acids	1	69
793	disease simulation	2	59
797	dissolution	2	67
798	dissolved oxygen	1	73
800	dna	2	67
801	dna 16s	4	254
807	dna extraction	14	656
808	dna microarray	1	73
809	dna sequence	5	299
810	dna, bacterial	7	345
811	dna, ribosomal	3	210

813	dopamine	2	98
814	dose response	2	89
818	drinking water	2	83
820	drug	2	78
826	drug compounding	2	79
827	drug degradation	2	82
830	drug effect	29	1071
831	drug effects	14	492
832	drug efficacy	3	123
835	drug formulation	5	185
836	drug mechanism	3	123
837	drug metabolism	2	82
838	drug metabolite	2	82
842	drug release	3	88
847	drug solubility	2	65
848	drug stability	2	47
867	dysbiosis	7	258
868	eating	2	83
870	ecosystem	7	263
873	ecosystems	5	127
879	effluents	2	88
889	elemental diet	1	81
890	ellagic acid	2	79
894	emulsification	3	88
895	emulsifier origin	1	77
896	emulsifying agent	2	117
898	emulsion	4	113
899	emulsions	7	146
903	endocrine disruptor	2	75
910	enteritis	3	100
912	enterobacteriaceae	9	368
915	enterococcus	4	149
916	enterococcus faecalis	2	62
923	environmental exposure	3	151
926	environmental monitoring	2	65
927	environmental pollutants	2	69
932	enzyme	2	133
933	enzyme activity	2	70
937	enzyme linked immunosorbent assay	2	63
941	enzymes	8	211
946	epithelial cells	3	157
948	epithelium cell	3	157
951	equipment design	3	58
953	erysipelotrichaceae	2	130
954	erythromycin	2	66
955	escherichia	3	180
956	escherichia coli	9	258
972	eubacterium	6	316
974	evaluation study	3	102
975	ex vivo study	5	210
977	exopolymer	1	103
978	exopolysaccharide	1	103
979	exopolysaccharides	1	103
984	experimental study	4	219
992	faecalibacterium	8	463
993	faecalibacterium prausnitzii	4	175
994	fasting	2	62
995	fat intake	2	57
996	fatty acid	16	558
997	fatty acid analysis	4	147

999	fatty acid synthesis	4	179
1000	fatty acids	30	944
1003	fatty acids, volatile	26	964
1007	fecal fermentation	2	56
1008	fecal microbiota	3	57
1013	feces	27	1037
1014	feces analysis	15	682
1017	feces microflora	12	584
1018	fed batch culture	1	74
1021	feeding	2	53
1022	female	25	1007
1024	fermentation	48	1706
1025	fermentation model	3	164
1027	fermentation process	2	76
1029	fermented dairy product	2	58
1031	fermented milk	2	115
1032	fermented milk product	1	78
1033	fermented product	2	71
1037	ferulic acid	2	96
1039	fiber	1	74
1041	fibers	3	85
1044	firmicutes	11	489
1046	fish	3	95
1052	fishes	2	65
1054	flagellin	2	117
1063	flavonoids	3	99
1067	flow cytometry	6	244
1070	flow rate	2	73
1075	fluorescence in situ hybridization	4	186
1076	fluorescence in situ hybridization (fish)	1	74
1077	fluorescence microscopy	3	141
1079	fluorouracil	2	53
1080	food	2	80
1081	food additive	2	69
1082	food additives	4	136
1087	food consumption	2	70
1088	food contamination	2	66
1089	food effect	2	62
1092	food industry	2	91
1093	food ingredient	2	129
1094	food ingredients	2	129
1095	food intake	3	113
1098	food processing	3	104
1105	food, formulated	1	81
1113	freeze drying	2	66
1114	freezing	2	90
1117	fructo-oligosaccharides	2	67
1120	fructose oligosaccharide	2	95
1121	fruit	7	188
1122	fruit and vegetable juice	3	70
1123	fruit and vegetable juices	3	70
1128	fruits	5	124
1129	fucose	1	103
1131	fumaric acid	1	73
1144	fungal virulence	1	86

1149	galacto-oligosaccharides	2	62
1151	galactose	2	138
1152	galactose oligosaccharide	2	96
1162	gas chromatography	6	347
1164	gas chromatography-mass spectrometry	2	79
1166	gastric digestions	6	114
1167	gastric emptying	3	60
1168	gastric juice	4	129
1176	gastrointestinal digestion	11	293
1177	gastrointestinal disease	2	58
1179	gastrointestinal microbiome	80	2885
1180	gastrointestinal model	6	114
1181	gastrointestinal motility	2	59
1183	gastrointestinal toxicity	2	83
1184	gastrointestinal tract	31	931
1187	gastrointestinal transit	3	89
1192	gellan	2	99
1194	gene amplification	4	200
1195	gene expression	6	246
1196	gene expression profiling	1	73
1197	gene expression regulation	4	158
1198	gene expression regulation, neoplastic	1	73
1200	gene sequence	15	727
1205	genes	2	85
1206	genetic analysis	3	188
1207	genetics	33	1335
1209	genome size	2	86
1210	genomic dna	3	183
1221	glucose	4	208
1227	glutathione	2	61
1232	glycerol	2	90
1235	glycosylation	3	79
1243	golden delicious	1	74
1257	growth	1	72
1259	growth, development and aging	16	602
1263	gum arabic	2	90
1264	gut	2	62
1270	gut microbiome	7	169
1271	gut microbiota	29	916
1273	gut model	3	61
1277	gut-on-chip	1	86
1290	health	3	116
1292	health hazard	4	160
1293	health risk	6	278
1295	health risks	8	348
1299	healthy volunteers	3	111
1301	heat	3	100
1304	heavy metal	2	51
1310	hep g2 cells	2	53
1311	hep-g2 cell line	3	94
1322	hexanoic acid	2	104

1325	high performance liquid chromatography	17	611
1328	high throughput screening	3	116
1329	high throughput sequencing	5	202
1330	high-throughput nucleotide sequencing	3	67
1332	high-throughput sequencing	3	130
1341	host microbial interactions	2	79
1344	hot temperature	2	65
1349	human	131	4532
1350	human activity	1	73
1351	human cell	15	789
1354	human experiment	6	230
1357	human gut microbiota	14	612
1359	human health risk assessment	2	70
1360	human hepatoma cell lines	2	84
1362	human intestinal microbiota	2	57
1363	human milk	3	78
1368	human umbilical vein endothelial cells	1	86
1370	humans	117	4056
1378	hydrogen-ion concentration	12	363
1379	hydrolysis	12	285
1387	hydroxypropyl methylcellulose	2	57
1394	ileum	3	68
1395	illumina 16s rrna gene sequencing	1	74
1400	immune response	5	260
1401	immune system	2	114
1403	immunocompetence	1	86
1404	immunocompetent cell	1	86
1405	immunocompetent intestine on chip	1	86
1409	immunological tolerance	1	86
1410	immunology	2	139
1411	immunomodulation	3	58
1414	in situ hybridization, fluorescence	2	121
1415	in vitro	4	85
1416	in vitro batch culture fermentation	1	74
1418	in vitro colon simulation	1	78
1419	in vitro colonic fermentation	2	90
1421	in vitro digestion	12	228
1422	in vitro fermentation	6	112
1425	in vitro gastrointestinal model	3	82
1430	in vitro model	6	153
1432	in vitro study	67	2638

1433	in vitro techniques	13	393
1436	in vivo study	5	180
1438	in-vitro	10	308
1439	in-vitro digestions	19	431
1443	indigestible fraction	2	86
1446	individual variation	2	94
1450	infant	7	160
1454	infant, premature	2	77
1456	infectious agent	3	140
1458	inflammation	6	209
1460	inflammatory bowel disease	2	65
1461	inflammatory bowel diseases	2	65
1467	inhibition	2	72
1469	innate immunity	1	86
1470	inoculation	3	139
1477	insects	2	68
1480	intelligent control	1	103
1486	interindividual variablility	1	77
1487	interleukin 10	3	150
1488	interleukin 1beta	3	150
1491	interleukin 6	4	176
1492	interleukin 8	5	234
1493	interleukin-10	3	150
1494	interleukin-6	3	136
1495	interleukin-8	2	72
1497	intermethod comparison	2	85
1499	intestinal absorption	7	268
1504	intestinal digestion	2	57
1506	intestinal epithelial cells	1	69
1510	intestinal lumen	2	62
1512	intestinal microbiota	4	101
1515	intestinal mucosa	8	258
1518	intestine	20	751
1519	intestine absorption	6	267
1520	intestine epithelium	1	86
1521	intestine epithelium cell	2	142
1522	intestine flora	102	3596
1523	intestine fluid	2	71
1524	intestine function	3	84
1526	intestine mucosa	9	344
1527	intestine mucosa permeability	1	86
1528	intestine on chip	1	86
1529	intestine tissue	1	86
1530	intestine transit time	3	73
1531	intestine, large	2	71
1532	intestine, small	10	297
1533	intestines	17	686
1535	inulin	11	514
1540	ion chromatography	2	145
1545	iron	5	167
1549	iron compounds	1	69
1551	iron oxide	1	69
1554	isobutyric acid	2	118
1555	isocitric acid	1	73
1558	isolation and purification	20	661
1562	jejunum	2	57
1564	kinetics	7	182

1566	klebsiella	3	153
1568	lab on a chip	2	124
1569	lab-on-a-chip devices	2	124
1572	lachnospiraceae	10	525
1577	lactic acid	6	274
1578	lactic acid bacterium	2	57
1581	lactobacillaceae	4	151
1582	lactobacilli	2	139
1583	lactobacillus	14	601
1584	lactobacillus acidophilus	2	80
1586	lactobacillus casei	2	114
1594	lactobacillus plantarum	9	340
1596	lactobacillus rhamnosus	4	229
1607	large intestine	8	237
1612	lemon	1	72
1614	leukocyte antigen	1	86
1618	limit of detection	3	152
1622	lipid	4	120
1625	lipid diet	4	119
1629	lipid peroxidation	2	68
1630	lipids	6	161
1631	lipolysis	7	179
1635	lipopolysaccharide	2	120
1636	lipopolysaccharides	2	120
1639	liquid chromatography	5	131
1661	macrophage	2	136
1664	macrophages	2	77
1666	magnetic resonance spectroscopy	2	120
1672	male	26	1075
1673	malonaldehyde	3	98
1674	malondialdehyde	3	78
1678	malus	1	74
1682	mangifera	2	86
1688	mass fragmentography	3	119
1689	mass spectrometry	13	485
1690	mathematical model	2	95
1694	meal	2	56
1697	meats	3	67
1700	medicinal chemistry	2	62
1702	megamonas	1	72
1710	mental disease	1	81
1711	mental disorders	1	81
1714	messenger rna	1	73
1716	metabolic activity assay	2	66
1724	metabolic syndrome x	2	109
1725	metabolism	105	3622
1726	metabolite	7	378
1728	metabolites	15	581
1730	metabolome	4	173
1731	metabolomics	9	327
1732	metagenome	3	145
1733	metagenomics	7	319
1738	metals, heavy	2	51
1739	metaproteomics	3	82
1744	methanearsonic acid	1	69
1747	methylation	5	222
1750	mice	4	108

1751	mice, inbred c57bl	3	125
1760	microbial activity	7	291
1761	microbial communities	8	288
1762	microbial community	33	1399
1763	microbial composition	5	179
1765	microbial consortium	3	110
1767	microbial diversity	19	784
1768	microbial eco system	12	415
1772	microbial growth	4	123
1773	microbial interaction	1	86
1774	microbial interactions	3	114
1775	microbial metabolism	8	357
1776	microbial metabolites	1	74
1778	microbial populations	2	103
1782	microbial viability	6	159
1783	microbiological examination	3	109
1784	microbiological phenomena and functions	1	86
1786	microbiology	68	2394
1787	microbiome	8	270
1788	microbiome composition	1	77
1789	microbiome functionality	1	77
1790	microbiota	30	1006
1792	microbiota-gut-brain axis	1	81
1793	microbiotas	7	252
1795	microcrystalline cellulose	1	81
1798	microflora	15	499
1801	microfluidics	2	109
1802	microorganism	7	267
1804	microphysiological system	1	86
1805	microrna	1	73
1806	micrornas	1	73
1810	microvilli	1	86
1811	microvillus	1	86
1812	middle aged	8	355
1815	milk protein	2	64
1817	milk proteins	3	66
1818	milk, human	3	113
1826	miseq	2	120
1832	model	6	230
1837	models, biological	38	1250
1842	molar ratio	2	79
1844	molecular dynamics	2	68
1845	molecular interaction	1	73
1847	molecular stability	2	84
1849	molecular weight	4	151
1851	monocyte chemotactic protein 1	2	78
1854	monomethylarsonic acids	2	109
1856	monosaccharide	1	103
1857	monosaccharide composition	1	103
1858	monosaccharides	1	103

1859	mood disorder	1	81
1860	mood disorders	1	81
1862	mouse	7	233
1866	mucin	8	277
1869	mucins	2	62
1873	mucosal immunity	1	86
1875	mucus	4	124
1878	multivariant analysis	2	61
1886	n acetylglucosamine	1	103
1891	nanoparticle	2	48
1900	next generation sequencing	4	204
1909	nitrogen	2	67
1913	nmr-based metabolomics	1	78
1917	nonhuman	52	2000
1918	normal human	18	786
1920	nuclear magnetic resonance	2	109
1921	nuclear magnetic resonance (nmr)	1	78
1923	nuclear magnetic resonance spectroscopy	2	120
1924	nucleotide sequence	2	84
1932	nutrition	4	177
1937	obese microbiota	1	72
1938	obese patient	1	72
1939	obesity	5	161
1940	occludin	2	107
1941	oil	2	58
1944	oils and fats	2	47
1949	oligosaccharide	12	438
1950	oligosaccharides	12	397
1953	on chips	1	86
1973	organismal interaction	2	114
1974	organoarsenic derivative	2	102
1982	oxidation	2	60
1983	oxidation products	2	60
1985	oxidation reduction reaction	3	98
1986	oxidation-reduction	3	98
1987	oxidative stress	2	119
1989	oxygen concentration	1	73
1990	oxygen consumption	1	77
1992	pancreatin	3	104
1995	parabacteroides	4	236
1997	particle size	8	224
1998	particle size analysis	2	56
1999	pasteurization	3	84
2001	pathogen	2	67
2002	pathogen load	1	86
2005	pathology	3	98
2012	pectin	8	340
2016	pectins	5	188
2017	pediococcus	1	78
2023	pepsin a	3	85
2024	peptide	3	90
2026	peptide fragment	2	82
2027	peptide fragments	2	82
2029	peptides	4	97
2035	perfusion	1	86
2037	permeability	2	55

2038	permeability barrier	1	86
2041	pesticide	2	63
2044	ph	17	503
2045	ph measurement	2	68
2052	pharmacogenetics	1	72
2054	pharmacology	2	97
2055	phascolarctobacterium	3	209
2058	phenol derivative	5	183
2059	phenolic compounds	3	71
2061	phenols	6	155
2062	phenotype	2	105
2069	phosphatidylcholine	1	77
2073	phylogeny	13	578
2074	phylum	2	57
2075	physical chemistry	2	66
2079	physiological models	10	329
2083	physiologically based extraction tests	4	121
2084	physiology	55	1804
2088	pickering emulsion	2	52
2090	pig	5	136
2092	pigments	4	71
2093	pink lady	1	74
2094	plant extract	11	396
2095	plant extracts	11	396
2098	plant leaf	2	47
2100	plant medicinal product	3	90
2101	plant preparations	3	90
2106	plants (botany)	3	89
2113	pollutant	2	69
2119	polyacrylamide gel electrophoresis	2	129
2123	polymer	2	60
2124	polymerase chain reaction	16	488
2125	polymerization	2	69
2128	polyphenol	10	350
2131	polyphenols	11	344
2132	polysaccharide	6	218
2134	polysaccharides	8	291
2135	polysaccharides, bacterial	2	121
2142	population abundance	3	97
2153	potable water	2	83
2159	prebiotic	11	281
2160	prebiotic agent	22	828
2161	prebiotic effect	2	80
2163	prebiotics	23	828
2167	prediction	2	112
2168	prematurity	2	77
2169	preschool child	2	78
2170	preservation	2	90
2177	prevotella	10	441
2178	prevotellaceae	2	65
2180	principal component analysis	5	142
2181	principal coordinate analysis	1	81
2182	priority journal	20	815
2183	proanthocyanidin	2	65
2184	proanthocyanidins	3	139
2186	probiotic	9	269
2188	probiotic agent	22	819

2190	probiotics	27	908
2191	procedures	17	595
2196	prophylaxis	1	72
2197	propionate	4	92
2198	propionates	6	261
2199	propionic acid	18	756
2200	propionic acid derivative	7	335
2201	protein	3	128
2203	protein analysis	6	180
2205	protein content	2	48
2208	protein degradation	10	292
2212	protein expression	1	73
2214	protein fingerprinting	1	77
2216	protein hydrolysis	3	45
2217	protein intake	4	92
2224	protein protein interaction	2	65
2227	protein zo1	2	120
2228	proteins	8	213
2229	proteobacteria	7	348
2230	proteolysis	8	234
2232	proteomics	5	136
2235	proton nuclear magnetic resonance	3	113
2236	providencia	1	78
2238	pseudomonas	3	105
2239	psycho-biotics	1	81
2240	psychology	1	81
2241	public health	3	154
2248	pyrosequencing	2	121
2251	quantitative analysis	6	296
2252	quantitative polymerase chain reaction	2	63
2253	quercetin	2	96
2261	rat	3	114
2266	reactive oxygen metabolite	2	72
2268	real time polymerase chain reaction	9	415
2270	real-time polymerase chain reaction	2	76
2275	red meat	2	56
2276	reduction	1	69
2278	reference standards	2	77
2280	relative abundance	4	119
2283	renetta canada	1	74
2284	reproducibility	5	189
2285	reproducibility of results	4	174
2293	retention time	4	250
2296	reverse transcription polymerase chain reaction	1	73
2297	rhamnose	2	138
2303	ribose	1	103
2304	ribosome dna	3	210
2308	risk assessment	11	450
2315	rna	8	370
2316	rna 16s	31	1420
2317	rna sequence	3	142
2318	rna sequencing	2	65
2320	rna, ribosomal, 16s	14	672
2324	roseburia	2	118

2326	ruminococcaceae	3	162
2327	ruminococcus	3	59
2329	rutoside	2	96
2334	saliva	2	90
2337	salmonella enterica serovar typhimurium	2	65
2338	salmonella typhimurium	2	65
2340	salts	5	125
2341	sampling	2	96
2343	saturated fatty acids	2	59
2347	scfas	3	58
2351	screening	3	88
2354	sea food	3	85
2355	seafood	3	85
2362	sequence analysis	2	87
2363	sequence analysis, dna	5	299
2364	serotonin	1	81
2367	shigella	4	227
2368	shime	11	197
2374	shime®	11	237
2375	shime® model	3	125
2376	short chain fatty acid	27	1221
2377	short chain fatty acids	7	145
2379	short chain fatty acids (scfas)	3	122
2382	short-chain fatty acids	21	575
2384	shrub	1	72
2386	signal transduction	4	144
2398	simgi® model	3	91
2406	simulation	14	522
2407	simulator	2	100
2408	simulator of human intestinal microbial ecosystem	2	85
2411	simulator of the human intestinal microbial ecosystem (shime)	7	196
2412	simulators	3	57
2414	sludge digestion	4	129
2415	small intestine	21	696
2417	small-intestinal digestion	2	42
2423	soil	7	284
2424	soil chemistry	1	69
2425	soil microflora	2	158
2426	soil pollutant	7	284
2427	soil pollutants	7	284
2428	soil pollution	3	174
2430	soils	5	236
2433	solid	1	69
2444	speciation	3	157
2445	speciation (chemistry)	3	157
2448	species composition	5	235
2449	species differentiation	2	109
2450	species diversity	2	74
2451	species richness	1	72
2452	specimen handling	2	90
2464	standard	2	77
2468	standardization	2	65

2471	starch	7	282
2490	stomach	16	554
2491	stomach emptying	4	110
2492	stomach juice	7	236
2493	stomach mucin	1	77
2494	stomach ph	4	121
2502	streptococcus	1	78
2517	subdoligranulum	1	103
2519	substrates	3	72
2520	succinic acid	3	131
2521	succinivibrionaceae	1	72
2535	surface property	2	79
2543	swine	4	109
2545	symbiosis	2	62
2547	synbiotic agent	3	172
2548	synbiotics	1	81
2549	synergistic effect	2	91
2560	tandem mass spectrometry	5	121
2566	taxonomy	4	171
2567	tea	3	89
2572	tetracycline	2	84
2576	thermal processing (foods)	3	65
2578	thermus	1	103
2579	thin layer chromatography	4	223
2583	three dimensional imaging	1	86
2584	throughput	1	103
2601	tissue	1	86
2602	tissue injury	1	86
2612	toll like receptor 5	1	77
2617	toxicity	6	210
2619	trace element	4	139
2620	trace elements	3	91
2623	transcriptome	2	115
2628	transverse colon	5	251
2630	triacylglycerol	3	103
2631	triacylglycerol lipase	4	151
2633	triglycerides	3	103
2652	ultra performance liquid chromatography	3	116
2655	ultrastructure	1	86
2658	umbilical vein endothelial cell	1	86
2659	unclassified drug	20	759
2668	upper gastrointestinal tract	3	78
2669	upregulation	2	78
2676	valeric acid	3	109
2678	validation study	4	128
2680	vancomycin	2	78
2682	varietas	1	74
2683	vascular endothelial cadherin	1	86
2687	vegetable	3	70
2692	veillonella	1	78
2693	veillonella parvula	2	61
2694	veillonellaceae	2	153
2695	verrucomicrobia	5	205
2703	viscosity	4	72
2709	vitis	3	101
2713	volatile fatty acid	26	964

2714	volatile fatty acids	9	216
2720	water	2	78
2722	water pollutant	2	51
2729	whey	2	69
2730	whey protein	5	148
2733	whey proteins	4	123
2737	x ray absorption	1	69
2738	x ray absorption near edge spectroscopy	1	69
2739	x ray absorption spectroscopy	1	69
2740	x-ray absorption near edge spectroscopy	1	69
2741	x-ray absorption spectroscopy	1	69
2742	xanthan	2	99
2749	xylan	2	105
2752	xylo oligosaccharide	2	91
2755	xylooligosaccharides	2	65
2756	yeast	2	107
2758	young adult	7	288
2762	zinc	3	78
2763	zonula occludens-1 protein	2	120

Table S5: List of key terms generated from analysis of initial search results (n = 460).

Terms
Artificial Gut
Artificial Simulator
Biological Model
Bioreactor
Bioreactor Systems
Chemostat
Colon Simulator
Continuous Fermentation Systems
Continuous Flow Bioreactor Model
Dynamic Gastric Model
Dynamic Gastrointestinal Simulator (Simgi)
Dynamic Gastrointestinal Tract Simulator
Dynamic In Vitro Digestive System
Dynamic In Vitro Simulator
Dynamic Simulation
Dynamic Simulation Of The Digestive Tract
Dynamic Simulators
Ex Vivo Model Of The Human Colon
Ex Vivo Study
Fermentation Model
Fermenters
Fermentors
Gastrointestinal Model
Gastrointestinal Simulator
Gastrointestinal Tract Simulator
Git Model
Gut Model
Human Gastric Digestion Simulator
Human Gut Model
Human Gut Reactor
In Vitro Batch Culture Fermentation
In Vitro Colon Model
In Vitro Colon Models
In Vitro Colon Simulation
In Vitro Colonic Fermentation
In Vitro Continuous Fermentation Model
In Vitro Digestion
In Vitro Digestion-fermentation
In Vitro Digestive Model
In Vitro Digestive System
In Vitro Fermentation
In Vitro Fermentation Model
In Vitro Fermentor
In Vitro Gastrointestinal Model
In Vitro Gut Model
In Vitro Metabolization
In Vitro Model
In Vitro Modeling
In Vitro Modelling Of The Human
In Vitro Semi-continuous
In Vitro Simulation
In Vitro Simulation Of Gastrointestinal Food Digestion
In Vitro Study
In Vitro Techniques
In-vitro Digestions
In-vitro Models
In-vitro Simulation Of The Colon
Membrane Fermenters
Model Of The Human Digestion System
Mucosal Simulator Of The Human Intestinal Microbial Ecosystem

Polyferms Colonic Fermentation Models
Reactor
Shime
Shime In Vitro Gut Simulator
Shime®
Shime® Model
Simgi® Model
Simulation Of Intestinal Tract
Simulator Of Human Intestinal Microbial Ecosystem
Simulator Of Human Intestinal Microbial Ecosystem Model
Simulator Of The Human Intestinal Microbial Ecosystem
Simulator Of The Human Intestinal Microbial Ecosystem (Shime ®)
Simulator Of The Human Intestinal Microbial Ecosystem (Shime)
Single Vessel Culturing
Three-stage In Vitro Colon Model
Toddler Shime

Supplementary Material Section 3

Refine search data

Table S6: List of publication titles with DOI for bibliographic data included in the final analysis (n = 1,451).

Title	DOI
"BOWEL ON THE BENCH": PROOF OF CONCEPT OF A THREE-STAGE, IN VITRO FERMENTATION MODEL OF THE EQUINE LARGE INTESTINE	10.1128/AEM.02093-19
2-FUCOSYLLACTOSE ALTERS THE COMPOSITION AND ACTIVITY OF GUT MICROBIOTA FROM FORMULA-FED INFANTS RECEIVING COMPLEMENTARY FEEDING IN A VALIDATED INTESTINAL MODEL	10.1016/j.jff.2019.103484
2'FL AND LNNT EXERT ANTIPATHOGENIC EFFECTS AGAINST C. DIFFICILE ATCC 9689 IN VITRO, COINCIDING WITH INCREASED LEVELS OF BIFIDOBACTERIACEAE AND/OR SECONDARY BILE ACIDS	10.3390/pathogens10080927
5-FLUOROURACIL AND IRINOTECAN (SN-38) HAVE LIMITED IMPACT ON COLON MICROBIAL FUNCTIONALITY AND COMPOSITION IN VITRO	10.7717/peerj.4017
A BACTERIOPHAGE COCKTAIL ELIMINATES SALMONELLA TYPHIMURIUM FROM THE HUMAN COLONIC MICROBIOME WHILE PRESERVING CYTOKINE SIGNALING AND PREVENTING ATTACHMENT TO AND INVASION OF HUMAN CELLS BY SALMONELLA IN VITRO	10.4315/0362-028X.JFP-18-587
A BACTERIOPHAGE COCKTAIL TARGETING ESCHERICHIA COLI REDUCES E. COLI IN SIMULATED GUT CONDITIONS, WHILE PRESERVING A NON-TARGETED REPRESENTATIVE COMMENSAL NORMAL MICROBIOTA	10.1080/19490976.2018.1447291
A BIOREACTOR WITH AN ELECTRO-RESPONSIVE ELASTOMERIC MEMBRANE FOR MIMICKING INTESTINAL PERISTALSIS	10.1088/1748-3190/12/1/016001
A BY-PRODUCT FROM VIRGIN OLIVE OIL PRODUCTION (PATE) ENCAPSULATED BY FLUID BED COATING: EVALUATION OF THE PHENOLIC PROFILE AFTER SHELF-LIFE TEST AND IN VITRO GASTROINTESTINAL DIGESTION	10.1111/ijfs.15068
A CASCADE OF A DENITRIFICATION BIOREACTOR AND AN AEROBIC BIOFILM REACTOR FOR HEAVY OIL REFINERY WASTEWATER TREATMENT	10.1039/C8RA10510C
A CITRUS FRUIT EXTRACT HIGH IN POLYPHENOLS BENEFICIALLY MODULATES THE GUT MICROBIOTA OF HEALTHY HUMAN VOLUNTEERS IN A VALIDATED IN VITRO MODEL OF THE COLON	10.3390/nu13113915
A COMBINATION OF XYLOOLIGOSACCHARIDES AND A POLYPHENOL BLEND AFFECT MICROBIAL COMPOSITION AND ACTIVITY IN THE DISTAL COLON EXERTING IMMUNOMODULATING PROPERTIES ON HUMAN CELLS	10.1016/j.jff.2018.05.053
A COMPARATIVE IN VITRO INVESTIGATION INTO THE EFFECTS OF COOKED MEATS ON THE HUMAN FAECAL MICROBIOTA	10.1016/j.anaerobe.2010.09.007
A COMPARISON OF THE ANTICANCER PROPERTIES OF ISOXANTHOHUMOL AND 8-PRENYLNARINGENIN USING IN VITRO MODELS OF COLON CANCER	10.1002/biof.1084
A COMPARISON OF THE IN VITRO EFFECTS OF 2'FUCOSYLLACTOSE AND LACTOSE ON THE COMPOSITION AND ACTIVITY OF GUT MICROBIOTA FROM INFANTS AND TODDLERS	10.3390/nu13030726
A COMPLEX HUMAN GUT MICROBIOME CULTURED IN AN ANAEROBIC INTESTINE-ON-A-CHIP	10.1038/s41551-019-0397-0
A COMPREHENSIVE ANTIMICROBIAL ACTIVITY EVALUATION OF THE RECOMBINANT MICROCIN J25 AGAINST THE FOODBORNE PATHOGENS SALMONELLA AND E. COLI O157:H7 BY USING A MATRIX OF CONDITIONS	10.3389/fmicb.2019.01954
A COMPUTER-CONTROLLED SYSTEM TO SIMULATE CONDITIONS OF THE LARGE INTESTINE WITH PERISTALTIC MIXING, WATER ABSORPTION AND ABSORPTION OF FERMENTATION PRODUCTS	10.1007/s002530051622
A CRITICAL EVALUATION OF IN VITRO HESPERIDIN 2S BIOAVAILABILITY IN A MODEL COMBINING LUMINAL (MICROBIAL) DIGESTION AND CACO-2 CELL ABSORPTION IN COMPARISON TO A RANDOMIZED CONTROLLED HUMAN TRIAL	10.1002/mnfr.201700881

A DRIED YEAST FERMENTATE SELECTIVELY MODULATES BOTH THE LUMINAL AND MUCOSAL GUT MICROBIOTA AND PROTECTS AGAINST INFLAMMATION, AS STUDIED IN AN INTEGRATED IN VITRO APPROACH	10.1021/jf402137r
A DYNAMIC MODEL THAT SIMULATES THE HUMAN UPPER GASTROINTESTINAL TRACT FOR THE STUDY OF PROBIOTICS	10.1016/j.ijfoodmicro.2004.08.020
A FIRST STEP TOWARDS A CONSENSUS STATIC IN VITRO MODEL FOR SIMULATING FULL-TERM INFANT DIGESTION	10.1016/j.foodchem.2017.07.145
A FORMULATION CASE STUDY COMPARING THE DYNAMIC GASTRIC MODEL WITH CONVENTIONAL DISSOLUTION METHODS	10.14227/DT190412P14
A FOUR-STRAIN PROBIOTIC EXERTS POSITIVE IMMUNOMODULATORY EFFECTS BY ENHANCING COLONIC BUTYRATE PRODUCTION IN VITRO	10.1016/j.ijpharm.2018.11.020
A MICROBIAL ENDOCRINOLOGY-BASED SIMULATED SMALL INTESTINAL MEDIUM FOR THE EVALUATION OF NEUROCHEMICAL PRODUCTION BY GUT MICROBIOTA	10.1093/femsec/fiy096
A MICROFLUIDICS-BASED IN VITRO MODEL OF THE GASTROINTESTINAL HUMAN-MICROBE INTERFACE	10.1038/ncomms11535
A MODEL STOMACH SYSTEM TO INVESTIGATE DISINTEGRATION KINETICS OF SOLID FOODS DURING GASTRIC DIGESTION	10.1111/j.1750-3841.2008.00745.x
A NEW REAL TIME PCR (TAQMAN PCR) SYSTEM FOR DETECTION OF THE16S RDNA GENE ASSOCIATED WITH FECAL BACTERIA	10.1016/j.mimet.2004.05.007
A NOVEL 3D IN VITRO MODEL OF THE HUMAN GUT MICROBIOTA	10.1038/s41598-020-78591-w
A NOVEL DISSOLUTION MEDIA FOR TESTING DRUG RELEASE FROM A NANOSTRUCTURED POLYSACCHARIDE-BASED COLON SPECIFIC DRUG DELIVERY SYSTEM: AN APPROACH TO ALTERNATIVE COLON MEDIA	10.2147/IJN.S97177
A NOVEL DISSOLUTION METHOD FOR EVALUATION OF POLYSACCHARIDE BASED COLON SPECIFIC DELIVERY SYSTEMS: A SUITABLE ALTERNATIVE TO ANIMAL SACRIFICE	10.1016/j.ejps.2015.03.012
A NOVEL DUAL-FLOW BIOREACTOR SIMULATES INCREASED FLUORESCEIN PERMEABILITY IN EPITHELIAL TISSUE BARRIERS	10.1002/biot.201400004
A NOVEL GALACTOOLIGOSACCHARIDE MIXTURE INCREASES THE BIFIDOBACTERIAL POPULATION NUMBERS IN A CONTINUOUS IN VITRO FERMENTATION SYSTEM AND IN THE PROXIMAL COLONIC CONTENTS OF PIGS IN VIVO	10.1093/jn/135.7.1726
A NOVEL METHOD FOR SCREENING OF POTENTIAL PROBIOTICS FOR HIGH ADHESION CAPABILITY	10.3168/jds.2015-9356
A NOVEL PROCESS FOR THE PRODUCTION OF HIGH-PURITY GALACTOOLIGOSACCHARIDES (GOS) USING CONSORTIUM OF MICROBES	10.1080/10826068.2016.1207082
A NOVEL, SCALABLE, AND MODULAR BIOREACTOR DESIGN FOR DYNAMIC SIMULATION OF THE DIGESTIVE TRACT	10.1002/bit.27902
A PECTIN-RICH, BAOBAB FRUIT PULP POWDER EXERTS PREBIOTIC POTENTIAL ON THE HUMAN GUT MICROBIOME IN VITRO	10.3390/microorganisms9091981
A PREBIOTIC-ENHANCED LIPID-BASED NUTRIENT SUPPLEMENT (LNSP) INCREASES BIFIDOBACTERIUM RELATIVE ABUNDANCE AND ENHANCES SHORT-CHAIN FATTY ACID PRODUCTION IN SIMULATED COLONIC MICROBIOTA FROM UNDERNOURISHED INFANTS	10.1093/FEMSEC/FIAA105
A RANDOMISED CROSSOVER STUDY INVESTIGATING THE EFFECTS OF GALACTO-OLIGOSACCHARIDES ON THE FAECAL MICROBIOTA IN MEN AND WOMEN OVER 50 YEARS OF AGE.	10.1017/S0007114511004697
A REVIEW ON CHROMATOGRAPHY-MASS SPECTROMETRY APPLICATIONS ON ANTHOCYANIN AND ELLAGITANNIN METABOLITES OF BLACKBERRIES AND RASPBERRIES	10.3390/foods10092150
A SIMPLE THREE-DIMENSIONAL GUT MODEL CONSTRUCTED IN A RESTRICTED DUCTAL MICROSPACE INDUCES INTESTINAL EPITHELIAL CELL INTEGRITY AND FACILITATES ABSORPTION ASSAYS	10.1039/d0bm00763c

A SINGLE-BATCH FERMENTATION SYSTEM TO SIMULATE HUMAN COLONIC MICROBIOTA FOR HIGH-THROUGHPUT EVALUATION OF PREBIOTICS	10.1371/journal.pone.0160533
A SMALL IN VITRO FERMENTATION MODEL FOR SCREENING THE GUT MICROBIOTA EFFECTS OF DIFFERENT FIBER PREPARATIONS	10.3390/ijms20081925
A SOFT TUBULAR MODEL REACTOR BASED ON THE BIONICS OF A SMALL INTESTINE ‚Äì STARCH HYDROLYSIS	10.1016/j.cherd.2016.06.005
A SPATIALLY CONTINUOUS MODEL OF CARBOHYDRATE DIGESTION AND TRANSPORT PROCESSES IN THE COLON	10.1371/journal.pone.0145309
A STANDARDISED SEMI-DYNAMIC: IN VITRO DIGESTION METHOD SUITABLE FOR FOOD-AN INTERNATIONAL CONSENSUS	10.1039/c9fo01293a
A STANDARDISED STATIC IN VITRO DIGESTION METHOD SUITABLE FOR FOOD-AN INTERNATIONAL CONSENSUS	10.1039/c3fo60702j
A STUDY BY 1H NMR ON THE INFLUENCE OF SOME FACTORS AFFECTING LIPID IN VITRO DIGESTION	10.1016/j.foodchem.2016.05.021
A SURVEY OF NON-STARTER LACTIC ACID BACTERIA IN TRADITIONAL CHEESES: CULTURE DEPENDENT IDENTIFICATION AND SURVIVAL TO SIMULATED GASTROINTESTINAL TRANSIT	10.1016/j.idairyj.2014.11.006
A SYNBIOTIC CONCEPT CONTAINING SPORE-FORMING BACILLUS STRAINS AND A PREBIOTIC FIBER BLEND CONSISTENTLY ENHANCED METABOLIC ACTIVITY BY MODULATION OF THE GUT MICROBIOME IN VITRO	10.1016/j.ijpx.2019.100021
A THREE-DIMENSIONAL IMMUNOCOMPETENT INTESTINE-ON-CHIP MODEL AS IN VITRO PLATFORM FOR FUNCTIONAL AND MICROBIAL INTERACTION STUDIES	10.1016/j.biomaterials.2019.119396
A THREE-STAGE CONTINUOUS CULTURE APPROACH TO STUDY THE IMPACT OF PROBIOTICS, PREBIOTICS AND FAT INTAKE ON FAECAL MICROBIOTA RELEVANT TO AN OVER 60 S POPULATION	10.1016/j.jff.2017.02.035
A TODDLER SHIMER MODEL TO STUDY MICROBIOTA OF YOUNG CHILDREN	10.1093/femsle/fnaa135
A TWO-STAGE CONTINUOUS CULTURE SYSTEM TO STUDY THE EFFECT OF SUPPLEMENTAL A-LACTALBUMIN AND GLYCOMACROPEPTIDE ON MIXED CULTURES OF HUMAN GUT BACTERIA CHALLENGED WITH ENTEROPATHOGENIC ESCHERICHIA COLI AND SALMONELLA SEROTYPE TYPHIMURIUM	10.1046/j.1365-2672.2003.01959.x
ABIRATERONE ACETATE PREFERENTIALLY ENRICHES FOR THE GUT COMMENSAL AKKERMANSIA MUCINIPHILA IN CASTRATE-RESISTANT PROSTATE CANCER PATIENTS	10.1038/s41467-020-18649-5
ABSORPTION AND DEGRADATION OF SULFATED POLYSACCHARIDE FROM PACIFIC ABALONE IN IN VITRO AND IN VIVO MODELS	10.1016/j.jff.2017.05.022
ABSORPTION AND METABOLISM OF PHENOLICS FROM DIGESTS OF POLYPHENOL-RICH POTATO EXTRACTS USING THE CACO-2/HEPG2 CO-CULTURE SYSTEM	10.3390/foods7010008
ACACIA GUM IMPROVES THE GUT BARRIER FUNCTIONALITY IN VITRO	
ACEMANNAN AND FRUCTANS FROM ALOE VERA (ALOE BARBADENSIS MILLER) PLANTS AS NOVEL PREBIOTICS	10.1021/acs.jafc.7b04100
ACEROLA BY-PRODUCT MAY IMPROVE THE IN VITRO GASTROINTESTINAL RESISTANCE OF PROBIOTIC STRAINS IN A PLANT-BASED FERMENTED BEVERAGE	10.1016/j.lwt.2021.110858
ACHIEVING ANTRAL GRINDING FORCES IN BIORELEVANT IN VITRO MODELS: COMPARING THE USP DISSOLUTION APPARATUS II AND THE DYNAMIC GASTRIC MODEL WITH HUMAN IN VIVO DATA	10.1208/s12249-011-9616-z
ACIDIC PH ENHANCES BUTYRATE PRODUCTION FROM PECTIN BY FAECAL MICROBIOTA	10.1093/femsle/fnab042
ACTIVATED ENDOTHELIAL CELLS LIMIT INFLAMMATORY RESPONSE, BUT INCREASE CHEMOATTRACTANT POTENTIAL AND BACTERIAL CLEARANCE BY HUMAN MONOCYTES	10.1002/cbin.10440
ACTIVATION OF THE SWEET TASTE RECEPTOR T1R3 BY SUCRALOSE ATTENUATES VEGF-INDUCED VASCULOGENESIS IN A CELL MODEL OF THE RETINAL MICROVASCULAR ENDOTHELIUM	10.1007/s00417-018-4157-8
ACTIVE FILMS BASED ON COCOA EXTRACT WITH ANTIOXIDANT, ANTIMICROBIAL AND BIOLOGICAL APPLICATIONS	10.1016/j.foodchem.2013.01.097
ACTIVITIES OF FREE AND ENCAPSULATED LACTOBACILLUS ACIDOPHILUS LA5 OR LACTOBACILLUS CASEI 01 IN PROCESSED LONGAN JUICES ON EXPOSURE TO SIMULATED GASTROINTESTINAL TRACT	10.1002/jsfa.6030

ACTIVITY OF VANCOMYCIN AGAINST EPIDEMIC CLOSTRIDIUM DIFFICILE STRAINS IN A HUMAN GUT MODEL	10.1093/jac/dkn502
ACUTE EFFECTS OF SUGARS AND ARTIFICIAL SWEETENERS ON SMALL INTESTINAL SUGAR TRANSPORT: A STUDY USING CACO-2 CELLS AS AN IN VITRO MODEL OF THE HUMAN ENTEROCYTE	10.1371/journal.pone.0167785
ADDING MUCINS TO AN IN VITRO BATCH FERMENTATION MODEL OF THE LARGE INTESTINE INDUCES CHANGES IN MICROBIAL POPULATION ISOLATED FROM PORCINE FECES DEPENDING ON THE SUBSTRATE	10.1093/femsec/fiv165
ADDITION OF ACACIA GUM TO A FOS/INULIN BLEND IMPROVES ITS FERMENTATION PROFILE IN THE SIMULATOR OF THE HUMAN INTESTINAL MICROBIAL ECOSYSTEM (SHIME)	10.1016/j.jff.2015.04.039
ADDITION OF PROBIOTIC BACTERIA IN A SEMI-HARD GOAT CHEESE (COALHO): SURVIVAL TO SIMULATED GASTROINTESTINAL CONDITIONS AND INHIBITORY EFFECT AGAINST PATHOGENIC BACTERIA	10.1016/j.foodres.2014.06.032
ADHERENCE AND CYTOKINE INDUCTION IN CACO-2 CELLS BY BACTERIAL POPULATIONS FROM A THREE-STAGE CONTINUOUS-CULTURE MODEL OF THE LARGE INTESTINE	10.1128/AEM.02244-10
ADMINISTRATION OF EQUOL-PRODUCING BACTERIA ALTERS THE EQUOL PRODUCTION STATUS IN THE SIMULATOR OF THE GASTROINTESTINAL MICROBIAL ECOSYSTEM (SHIME)	10.1093/jn/136.4.946
AKKERMANSIA MUCINIPHILA ADHERES TO ENTEROCYTES AND STRENGTHENS THE INTEGRITY OF THE EPITHELIAL CELL LAYER	10.1128/AEM.04050-14
ALKALI + CELLULASE-EXTRACTED CITRUS PECTINS EXHIBIT COMPACT CONFORMATION AND GOOD FERMENTATION PROPERTIES	10.1016/j.foodhyd.2020.106079
ALTERED CHICKEN CECAL MICROBIAL COMMUNITIES AFFECT SALMONELLA COLONIZATION	10.7537/marslsj120215.03
AMOXICILLIN INCREASED FUNCTIONAL PATHWAY GENES AND BETA-LACTAM RESISTANCE GENES BY PATHOGENS BLOOMED IN INTESTINAL MICROBIOTA USING A SIMULATOR OF THE HUMAN INTESTINAL MICROBIAL ECOSYSTEM	10.3389/fmicb.2020.01213
AN ADVANCED IN VITRO TECHNOLOGY PLATFORM TO STUDY THE MECHANISM OF ACTION OF PREBIOTICS AND PROBIOTICS IN THE GASTROINTESTINAL TRACT	10.1097/MCG.0000000000000711
AN ALGORITHM FOR DESIGNING MINIMAL MICROBIAL COMMUNITIES WITH DESIRED METABOLIC CAPACITIES	10.1093/bioinformatics/btw107
AN ALTERNATIVE WAY TO ENCAPSULATE PROBIOTICS WITHIN ELECTROSPUN ALGINATE NANOFIBERS AS MONITORED UNDER SIMULATED GASTROINTESTINAL CONDITIONS AND IN KEFIR	10.1016/j.carbpol.2020.116447
AN ENGINEERING MODEL OF THE HUMAN COLON	10.1205/fbp.04396
AN EVALUATION OF THE PREBIOTIC POTENTIAL OF MICROBIAL LEVANS FROM ERWINIA SP. 10119	10.1016/j.jff.2019.103668
AN EXPLORATORY STUDY INTO THE PUTATIVE PREBIOTIC ACTIVITY OF FRUCTANS ISOLATED FROM AGAVE ANGUSTIFOLIA AND THE ASSOCIATED ANTICANCER ACTIVITY	10.1016/j.anaerobe.2013.05.006
AN EXPLORATORY STUDY ON THE INFLUENCE OF ORANGE JUICE ON GUT MICROBIOTA USING A DYNAMIC COLONIC MODEL	10.1016/j.foodres.2016.03.028
AN IN VITRO APPROACH TO STUDY EFFECTS OF PREBIOTICS AND PROBIOTICS ON THE FAECAL MICROBIOTA AND SELECTED IMMUNE PARAMETERS RELEVANT TO THE ELDERLY	10.1371/journal.pone.0162604
AN IN VITRO ENRICHMENT STRATEGY FOR FORMULATING SYNERGISTIC SYNBIOTICS	10.1128/AEM.01073-19
AN IN VITRO FERMENTATION STUDY ON THE EFFECTS OF GLUTEN FRIENDLYTM BREAD ON MICROBIOTA AND SHORT CHAIN FATTY ACIDS OF FECAL SAMPLES FROM HEALTHY AND CELIAC SUBJECTS	10.3389/fmicb.2017.01722
AN IN VITRO MODEL FOR MICROBIAL FRUCTOSELYSINE DEGRADATION SHOWS SUBSTANTIAL INTERINDIVIDUAL DIFFERENCES IN METABOLIC CAPACITIES OF HUMAN FECAL SLURRIES	10.1016/j.tiv.2021.105078
AN IN VITRO MODEL MAINTAINING TAXON-SPECIFIC FUNCTIONAL ACTIVITIES OF THE GUT MICROBIOME	10.1038/s41467-019-12087-8
AN IN VITRO MODEL OF THE HUMAN COLON: STUDIES OF INTESTINAL BIOFILMS AND CLOSTRIDIUM DIFFICILE INFECTION	10.1007/978-1-4939-6361-4_17
AN IN VITRO PILOT FERMENTATION STUDY ON THE IMPACT OF CHLORELLA PYRENOIDOSA ON GUT MICROBIOME COMPOSITION AND METABOLITES IN HEALTHY AND COELIAC SUBJECTS	10.3390/molecules26082330

AN IN-VITRO UPPER GUT SIMULATOR FOR ASSESSING CONTINUOUS GAS PRODUCTION: A PROOF-OF-CONCEPT USING MILK DIGESTION	10.1016/j.jff.2018.05.054
AN INVITRO STUDY OF THE EFFECT OF PROBIOTICS, PREBIOTICS AND SYNBIOTICS ON THE ELDERLY FAECAL MICROBIOTA	10.1016/j.anaerobe.2014.03.009
AN ORAL FMT CAPSULE AS EFFICIENT AS AN ENEMA FOR MICROBIOTA RECONSTRUCTION FOLLOWING DISRUPTION BY ANTIBIOTICS, AS ASSESSED IN AN IN VITRO HUMAN GUT MODEL	10.3390/microorganisms9020358
AN: IN VITRO DIGESTION METHOD ADAPTED FOR CAROTENOIDS AND CAROTENOID ESTERS: MOVING FORWARD TOWARDS STANDARDIZATION	10.1039/c6fo01293k
AN: IN VITRO EXPLORATORY STUDY OF DIETARY STRATEGIES BASED ON POLYPHENOL-RICH BEVERAGES, FRUIT JUICES AND OILS TO CONTROL TRIMETHYLAMINE PRODUCTION IN THE COLON	10.1039/c8fo01778f
ANAEROBIC COMETABOLISM OF FRUIT AND VEGETABLE WASTES USING MAMMALIAN FECAL INOCULUMS: FAST ASSESSMENT OF BIOMETHANE PRODUCTION	10.1016/j.jclepro.2016.09.215
ANALYSIS OF A WHOLE DIET IN TERMS OF PHENOLIC CONTENT AND ANTIOXIDANT CAPACITY: EFFECTS OF A SIMULATED GASTROINTESTINAL DIGESTION	10.1080/09637486.2016.1186156
ANALYSIS OF PROBIOTIC PROPERTIES AND SAFETY OF LACTOBACILLUS REUTERI	10.13982/j.mfst.1673-9078.2016.6.048
ANALYSIS OF THE BACTERIAL COMMUNITY IN A LABORATORY-SCALE NITRIFICATION REACTOR AND A WASTEWATER TREATMENT PLANT BY 454-PYROSEQUENCING	10.1016/j.watres.2011.05.028
ANALYSIS OF THE CHARACTERISTICS AND CYTOTOXICITY OF TITANIUM DIOXIDE NANOMATERIALS FOLLOWING SIMULATED IN VITRO DIGESTION	10.3390/nano10081516
ANTACID INCREASES SURVIVAL OF VIBRIO VULNIFICUS AND VIBRIO VULNIFICUS PHAGE IN A GASTROINTESTINAL MODEL	10.1128/AEM.67.7.2895-2902.2001
ANTAGONISTIC EFFECT OF SACCHAROMYCES CEREVISIAE KTP AND ISSATCHENKIA OCCIDENTALIS APC ON HYPHAL DEVELOPMENT AND ADHESION OF CANDIDA ALBICANS	10.1093/mmy/myx156
ANTAGONISTIC MECHANISMS OF SYNBIOSIS BETWEEN LACTOBACILLUS PLANTARUM CIF17AN2 AND GREEN BANANA STARCH IN THE PROXIMAL COLON MODEL CHALLENGED WITH SALMONELLA TYPHIMURIUM	10.1016/j.anaerobe.2014.05.002
ANTI-INFLAMMATORY EFFECT OF MICROBIAL CONSORTIA DURING THE UTILIZATION OF DIETARY POLYSACCHARIDES	10.1016/j.foodres.2018.04.008
ANTIBIOTIC DISTURBANCE AFFECTS AQUATIC MICROBIAL COMMUNITY COMPOSITION AND FOOD WEB INTERACTIONS BUT NOT COMMUNITY RESILIENCE	10.1111/mec.15033
ANTIBIOTIC RESIDUES AND R-PLASMID SELECTION: ARE IN VITRO METHODS GOOD MODELS?	10.1016/S0176-6724(87)80138-4
ANTIBIOTIC RESISTANCE CAPABILITY OF CULTURED HUMAN COLONIC MICROBIOTA GROWING IN A CHEMOSTAT MODEL	10.1007/s12010-014-0882-6
ANTIBIOTIC TREATMENTS AND MICROBES IN THE GUT	10.1111/1462-2920.12399
ANTIBIOTICS IN THE HUMAN FOOD CHAIN: ESTABLISHING NO EFFECT LEVELS OF TETRACYCLINE, NEOMYCIN, AND ERYTHROMYCIN USING A CHEMOSTAT MODEL OF THE HUMAN COLONIC MICROFLORA	10.1016/j.yrtph.2005.06.005
ANTIMICROBIAL ACTIVITY OF SELECTED SYNBIOTICS TARGETED FOR THE ELDERLY AGAINST PATHOGENIC ESCHERICHIA COLI STRAINS	10.3109/09637486.2015.1134444
ANTIOXIDANT ACTIVITY AND BIO-ACCESSIBILITY OF POLYPHENOLS IN BLACK CARROT (DAUCUS CAROTA L. SSP. SATIVUS VAR. ATRORUBENS ALEF.) AND TWO DERIVED PRODUCTS DURING SIMULATED GASTROINTESTINAL DIGESTION AND COLONIC FERMENTATION	10.3390/foods10020457
ANTIOXIDANT ACTIVITY OF PICKLED SAUCED MEAT BEFORE AND AFTER COOKING AND IN VITRO GASTROINTESTINAL DIGESTION	10.1111/jfpp.14922
ANTIOXIDANT AND ANTIDIABETIC ACTIVITY OF BLACKBERRY AFTER GASTROINTESTINAL DIGESTION AND HUMAN GUT MICROBIOTA FERMENTATION	10.1016/j.foodchem.2018.07.020

ANTIOXIDANT CAPACITY OF FLAXSEED PRODUCTS: THE EFFECT OF IN VITRO DIGESTION	10.1007/s11130-012-0329-6
ANTIOXIDANT POTENTIAL OF PHENOLIC-RICH TWO VARIETIES OF NIGERIAN LOCAL RICE AND THEIR ANTI-CHOLINESTERASE ACTIVITIES AFTER IN VITRO DIGESTION	10.1108/NFS-08-2015-0093
ANTIOXIDANT VITAMINS AND PREBIOTIC FOS AND XOS DIFFERENTIALLY SHIFT MICROBIOTA COMPOSITION AND FUNCTION AND IMPROVE INTESTINAL EPITHELIAL BARRIER IN VITRO	10.3390/nu13041125
ANTIOXIDATIVE EFFECTS OF PHENOLIC COMPOUNDS OF MUSHROOM MYCELIA IN SIMULATED REGIONS OF THE HUMAN COLON, IN VITRO STUDY	10.1515/pjfns-2017-0010
ANTIPROLIFERATIVE ACTIVITY OF GREEN, BLACK TEA AND OLIVE LEAVES POLYPHENOLS SUBJECTED TO BIOSORPTION AND IN VITRO GASTROINTESTINAL DIGESTION IN CACO-2 CELLS	10.1016/j.foodres.2020.109317
APPARENT DIET DIGESTIBILITY OF CAPTIVE COLOBINES IN RELATION TO STOMACH TYPES WITH SPECIAL REFERENCE TO FIBRE DIGESTION	10.1371/journal.pone.0256548
APPLICATION OF A NEW DYNAMIC GASTROINTESTINAL SIMULATOR (SIMGI) TO STUDY THE IMPACT OF RED WINE IN COLONIC METABOLISM	10.1016/j.foodres.2015.03.003
APPLICATION OF A NOVEL GASTROINTESTINAL TRACT SIMULATOR SYSTEM BASED ON A MEMBRANE BIOREACTOR (SIMUGIT) TO STUDY THE STOMACH TOLERANCE AND EFFECTIVE DELIVERY ENHANCEMENT OF NANOENCAPSULATED MACELIGNAN	10.1016/j.ces.2015.10.006
APPLICATION OF AN IN VITRO DIGESTION MODEL FOR 210PO BIOACCESSIBILITY ASSESSMENT IN SEAFOOD	10.1088/1361-6498/aa869b
APPLICATION OF AN IN VITRO DIGESTION MODEL TO STUDY THE METABOLIC PROFILE CHANGES OF AN HERBAL EXTRACT COMBINATION BY UHPLC-HRMS	10.1016/j.phymed.2020.153221
APPLICATION OF IN VITRO GASTROINTESTINAL DIGESTION AND COLONIC FERMENTATION MODELS TO POMEGRANATE PRODUCTS (JUICE, PULP AND PEEL EXTRACT) TO STUDY THE STABILITY AND CATABOLISM OF PHENOLIC COMPOUNDS	10.1016/j.jff.2015.02.026
APPLICATION OF THE DYNAMIC GASTRIC MODEL TO EVALUATE THE EFFECT OF FOOD ON THE DRUG RELEASE CHARACTERISTICS OF A HYDROPHILIC MATRIX FORMULATION	10.1016/j.ijpharm.2014.03.031
APPLICATION OF THE DYNAMIC GASTROINTESTINAL SIMULATOR (SIMGI®) TO ASSESS THE IMPACT OF PROBIOTIC SUPPLEMENTATION IN THE METABOLISM OF GRAPE POLYPHENOLS	10.1016/j.foodres.2019.108790
APPLYING ADVANCED IN VITRO CULTURING TECHNOLOGY TO STUDY THE HUMAN GUT MICROBIOTA	10.3791/59054
APPLYING DIFFERENTIAL NEURAL NETWORKS TO CHARACTERIZE MICROBIAL INTERACTIONS IN AN EX VIVO GASTROINTESTINAL GUT SIMULATOR	10.3390/PR8050593
APPROACHES THAT ASCERTAIN THE ROLE OF DIETARY COMPOUNDS IN COLONIC CANCER CELLS	10.4251/wjgo.v6.i1.1
ARABINOGALACTAN AND FRUCTO-OLIGOSACCHARIDES HAVE A DIFFERENT FERMENTATION PROFILE IN THE SIMULATOR OF THE HUMAN INTESTINAL MICROBIAL ECOSYSTEM (SHIME®)	10.1111/1758-2229.12056
ARABINOGALACTAN AND FRUCTOOLIGOSACCHARIDES IMPROVE THE GUT BARRIER FUNCTION IN DISTINCT AREAS OF THE COLON IN THE SIMULATOR OF THE HUMAN INTESTINAL MICROBIAL ECOSYSTEM	10.1016/j.jff.2015.11.005
ARABINOXYLAN-OLIGOSACCHARIDES (AXOS) AFFECT THE PROTEIN/CARBOHYDRATE FERMENTATION BALANCE AND MICROBIAL POPULATION DYNAMICS OF THE SIMULATOR OF HUMAN INTESTINAL MICROBIAL ECOSYSTEM	10.1111/j.1751-7915.2008.00064.x
ARABINOXYLANS, INULIN AND LACTOBACILLUS REUTERI 1063 REPRESS THE ADHERENT-INVASIVE ESCHERICHIA COLI FROM MUCUS IN A MUCOSA-COMPRISING GUT MODEL	10.1038/npjbiofilms.2016.16
ARE SILVER NANOPARTICLES BETTER THAN TRICLOSAN AS A DAILY ANTIMICROBIAL? ANSWERS FROM THE PERSPECTIVES OF GUT MICROBIOME DISRUPTION AND PATHOGENICITY	10.1016/j.scitotenv.2020.143983
ARONIA (ARONIA MELANOCARPA) PHENOLICS BIOAVAILABILITY IN A COMBINED IN VITRO DIGESTION/CACO-2 CELL MODEL IS STRUCTURE AND COLON REGION DEPENDENT	10.1016/j.jff.2017.09.008

ARONIA (ARONIA MELANOCARPA) POLYPHENOLS MODULATE THE MICROBIAL COMMUNITY IN A SIMULATOR OF THE HUMAN INTESTINAL MICROBIAL ECOSYSTEM (SHIME) AND DECREASE SECRETION OF PROINFLAMMATORY MARKERS IN A CACO-2/ENDOTHELIAL CELL COCULTURE MODEL	10.1002/mnfr.201800607
ARSENIC IN COOKED RICE: EFFECT OF CHEMICAL, ENZYMATIC AND MICROBIAL PROCESSES ON BIOACCESSIBILITY AND SPECIATION IN THE HUMAN GASTROINTESTINAL TRACT	10.1016/j.envpol.2011.11.021
ARSENIC METABOLISM AND TOXICITY INFLUENCED BY FERRIC IRON IN SIMULATED GASTROINTESTINAL TRACT AND THE ROLES OF GUT MICROBIOTA	10.1021/acs.est.6b01533
ARSENIC METABOLISM BY HUMAN GUT MICROBIOTA UPON IN VITRO DIGESTION OF CONTAMINATED SOILS	10.1289/ehp.0901794
ARSENIC SPECIATION AND BIOACCESSIBILITY IN RAW AND COOKED SEAFOOD: INFLUENCE OF SEAFOOD SPECIES AND GUT MICROBIOTA	10.1016/j.envpol.2021.116958
ARSENIC UNDERGOES SIGNIFICANT SPECIATION CHANGES UPON INCUBATION OF CONTAMINATED RICE WITH HUMAN COLON MICRO BIOTA	10.1016/j.jhazmat.2012.05.042
ARTIFICIAL INTELLIGENCE AND NEXT GENERATION SEQUENCING: NEW FINDINGS IN MYELOID NEOPLASMS [KÜNSTLICHE INTELLIGENZ UND NEXT GENERATION SEQUENCING NEUE ERKENNTNISSE BEI MYELOISCHEN NEOPLASIEN]	10.1055/a-1300-9185
ASSESSING DIGESTIBILITY OF HADZA TUBERS USING A DYNAMIC IN-VITRO MODEL	10.1002/ajpa.22805
ASSESSING THE EFFECTS OF SILVER NANOPARTICLES ON MONOLAYERS OF DIFFERENTIATED CACO-2 CELLS, AS A MODEL OF INTESTINAL BARRIER	10.1016/j.fct.2018.04.008
ASSESSING THE IN VITRO DIGESTION OF SESBANIA GUM, A GALACTOMANNAN FROM S. CANNABINA, AND SUBSEQUENT IMPACT ON THE FECAL MICROBIOTA	10.1016/j.jff.2021.104766
ASSESSING THE INFLUENCE OF REACTOR SYSTEM DESIGN CRITERIA ON THE PERFORMANCE OF MODEL COLON FERMENTATION UNITS	10.1016/j.jbiosc.2013.09.015
ASSESSING THE VIABILITY OF A SYNTHETIC BACTERIAL CONSORTIUM ON THE IN VITRO GUT HOST-MICROBE INTERFACE	10.3791/57699
ASSESSMENT OF BIOACCESSIBLE AND DIALYZABLE FRACTIONS OF NICKEL IN FOOD PRODUCTS AND THEIR IMPACT ON THE CHRONIC EXPOSURE OF BELGIAN POPULATION TO NICKEL	10.1016/j.foodchem.2020.128210
ASSESSMENT OF BIOAVAILABILITY AFTER IN VITRO DIGESTION AND FIRST PASS METABOLISM OF BIOACTIVE PEPTIDES FROM COLLAGEN HYDROLYSATES	10.3390/cimb43030113
ASSESSMENT OF DYNAMIC BIOACCESSIBILITY OF CURCUMIN ENCAPSULATED IN MILLED STARCH PARTICLE STABILIZED PICKERING EMULSIONS USING TNO'S GASTROINTESTINAL MODEL	10.1039/c8fo02495b
ASSESSMENT OF NUTRIENTS EFFECT ON THE BIOACCESSIBILITY OF CD AND CU IN CONTAMINATED SOIL	10.1016/j.ecoenv.2020.110913
ASSESSMENT OF THE BACTERIAL DIVERSITY OF AGAVE SAP CONCENTRATE, RESISTANCE TO IN VITRO GASTROINTESTINAL CONDITIONS AND SHORT-CHAIN FATTY ACIDS PRODUCTION	10.1016/j.foodres.2020.109862
ASSESSMENT OF THE PREBIOTIC EFFECT OF QUINOA AND AMARANTH IN THE HUMAN INTESTINAL ECOSYSTEM	10.1039/c6fo00924g
ASSESSMENT OF THE SOLUBILITY AND BIOACCESSIBILITY OF ARSENIC IN REALGAR WINE USING A SIMULATED GASTROINTESTINAL SYSTEM	10.1016/j.scitotenv.2011.03.003
ASSOCIATION BETWEEN ARTIFICIALLY SWEETENED BEVERAGE CONSUMPTION DURING PREGNANCY AND INFANT BODY MASS INDEX	10.1001/jamapediatrics.2016.0301
ASSOCIATIVE EFFECTS BETWEEN FORAGES AND CONCENTRATES ON IN VITRO FERMENTATION OF WORKING EQUINE DIETS	10.3390/ani11082212
AZO POLYMERS FOR COLON TARGETED DRUG DELIVERY	
BACILLUS CEREUS ADHESION TO SIMULATED INTESTINAL MUCUS IS DETERMINED BY ITS GROWTH ON MUCIN, RATHER THAN INTESTINAL ENVIRONMENTAL PARAMETERS	10.1089/fpd.2014.1926
BACILLUS COAGULANS GBI-30, 6086 INCREASES PLANT PROTEIN DIGESTION IN A DYNAMIC, COMPUTER-CONTROLLED IN VITRO MODEL OF THE SMALL INTESTINE (TIM-1)	10.3920/BM2016.0196

BACILLUS SUBTILIS HU58 AND BACILLUS COAGULANS SC208 PROBIOTICS REDUCED THE EFFECTS OF ANTIBIOTIC-INDUCED GUT MICROBIOME DYSBIOSIS IN AN M-SHIME® MODEL	10.3390/microorganisms8071028
BACTERIA REMEDIATE THE EFFECTS OF FOOD ADDITIVES ON INTESTINAL FUNCTION IN AN IN VITRO MODEL OF THE GASTROINTESTINAL TRACT	10.3389/fnut.2020.00131
BACTERIAL COMMUNITY DIVERSITY DYNAMICS HIGHLIGHT DEGREES OF NESTEDNESS AND TURNOVER PATTERNS	10.1002/cyto.a.23965
BACTERIAL COMMUNITY DYNAMICS IN LIQUID SWINE MANURE DURING STORAGE: MOLECULAR ANALYSIS USING DGGE/PCR OF 16S RDNA	10.1016/S0168-6496(01)00181-7
BACTERIAL DISPERSAL AND DRIFT DRIVE MICROBIOME DIVERSITY PATTERNS WITHIN A POPULATION OF FERAL HINDGUT FERMENTERS	10.1111/mec.15747
BACTERIAL WALL ATTACHMENT IN A FLOW REACTOR	10.1137/S0036139901390416
BACTERIAL, SCFA AND GAS PROFILES OF A RANGE OF FOOD INGREDIENTS FOLLOWING IN VITRO FERMENTATION BY HUMAN COLONIC MICROBIOTA	10.1016/j.anaerobe.2010.05.006
BACTERIOLOGICAL EFFECTS OF A LACTOBACILLUS REUTERI PROBIOTIC ON IN VITRO ORAL BIOFILMS	10.1016/j.archoralbio.2011.04.004
BACTERIOPHAGES AS MODULATOR FOR THE HUMAN GUT MICROBIOTA: RELEASE FROM DAIRY FOOD SYSTEMS AND SURVIVAL IN A DYNAMIC HUMAN GASTROINTESTINAL MODEL	10.1016/j.lwt.2018.01.033
BACTEROIDES FRAGILIS PREVENTS SALMONELLA HEIDELBERG TRANSLOCATION IN CO-CULTURE MODEL MIMICKING INTESTINAL EPITHELIUM	10.3920/BM2020.0004
BACTEROIDES THETAIOTAOMICRON FOSTERS THE GROWTH OF BUTYRATE-PRODUCING ANAEROSTIPES CACCAE IN THE PRESENCE OF LACTOSE AND TOTAL HUMAN MILK CARBOHYDRATES	10.3390/microorganisms8101513
BARCODED PYROSEQUENCING ANALYSIS OF THE MICROBIAL COMMUNITY IN A SIMULATOR OF THE HUMAN GASTROINTESTINAL TRACT SHOWED A COLON REGION-SPECIFIC MICROBIOTA MODULATION FOR TWO PLANT-DERIVED POLYSACCHARIDE BLENDS	10.1007/s10482-012-9821-0
BASOLATERAL SECRETION FROM CACO-2 CELLS PRETREATED WITH FECAL WATERS FROM BREAST CANCER PATIENTS AFFECTS MCF7 CELL VIABILITY	10.3390/nu13010031
BEHAVIOR OF BACILLUS COAGULANS UNIQUE IS2 SPORES DURING PASSAGE THROUGH THE SIMULATOR OF HUMAN INTESTINAL MICROBIAL ECOSYSTEM (SHIME) MODEL	10.1016/j.lwt.2020.109196
BEHAVIOUR OF CITRUS PECTIN DURING ITS GASTROINTESTINAL DIGESTION AND FERMENTATION IN A DYNAMIC SIMULATOR (SIMGI¬Æ)	10.1016/j.carbpol.2018.11.088
BENEFICIAL EFFECTS OF FERMENTED VEGETAL BEVERAGES ON HUMAN GASTROINTESTINAL MICROBIAL ECOSYSTEM IN A SIMULATOR	10.1016/j.foodres.2014.05.072
BENEFICIAL METABOLIC EFFECTS OF SELECTED PROBIOTICS ON DIET-INDUCED OBESITY AND INSULIN RESISTANCE IN MICE ARE ASSOCIATED WITH IMPROVEMENT OF DYSBIOTIC GUT MICROBIOTA	10.1111/1462-2920.13181
BETA-GLUCAN AND PHENOLIC COMPOUNDS: THEIR CONCENTRATION AND BEHAVIOR DURING IN VITRO GASTROINTESTINAL DIGESTION AND COLONIC FERMENTATION OF DIFFERENT BARLEY-BASED FOOD PRODUCTS	10.1021/acs.jafc.8b02240
BI-COMPARTMENTAL ELDERLY OR ADULT DYNAMIC DIGESTION MODELS APPLIED TO INTERROGATE PROTEIN DIGESTIBILITY	10.1039/c4fo00478g
BIFIDOBACTERIUM ANIMALIS SSP. LACTIS BI07 MODULATES THE TUMOR NECROSIS FACTOR ALPHA-DEPENDENT IMBALANCES OF THE ENTEROCYTE-ASSOCIATED INTESTINAL MICROBIOTA FRACTION	10.1111/1574-6968.12515
BIFIDOBACTERIUM BIFIDUM AND THE INFANT GUT MICROBIOTA: AN INTRIGUING CASE OF MICROBE-HOST CO-EVOLUTION	10.1111/1462-2920.14705
BIFIDOBACTERIUM BIFIDUM ATCC 15696 AND BIFIDOBACTERIUM BREVE 24B METABOLIC INTERACTION BASED ON 2=-O-FUCOSYL-LACTOSE STUDIED IN STEADY-STATE CULTURES IN A FRETER-STYLE CHEMOSTAT	10.1128/AEM.02783-18
BIFIDOBACTERIUM LONGUM D2 ENHANCES MICROBIAL DEGRADATION OF LONG-CHAIN ARABINOXYLANS IN AN IN VITRO MODEL OF THE PROXIMAL COLON	10.3920/BM2015.0023

BIFIDOGENIC AND BUTYROGENIC EFFECTS OF YOUNG BARELY LEAF EXTRACT IN AN IN VITRO HUMAN COLONIC MICROBIOTA MODEL	10.1186/s13568-019-0911-5
BILE AMOUNT AFFECTS BOTH THE DEGREE OF MICELLARIZATION AND THE HYDROLYSIS EXTENT OF CAROTENOID ESTERS DURING IN VITRO DIGESTION	10.1039/c9fo01453e
BILE-INDUCED PROMOTERS FOR GENE EXPRESSION IN LACTOBACILLUS STRAINS	10.1007/s00253-019-09743-w
BINDING OF ACRIDINE ORANGE BY PROBIOTIC LACTOBACILLUS RHAMNOSUS STRAINS OF HUMAN ORIGIN	10.1007/s13213-014-0975-z
BINDING OF BILE ACIDS BY PASTRY PRODUCTS CONTAINING BIOACTIVE SUBSTANCES DURING IN VITRO DIGESTION	10.1039/c4fo00946k
BIOACCESSIBILITY ANALYSIS OF ANTHOCYANINS AND ELLAGITANNINS FROM BLACKBERRY AT SIMULATED GASTROINTESTINAL AND COLONIC LEVELS	10.1016/j.jfca.2018.05.007
BIOACCESSIBILITY AND ANTIOXIDANT ACTIVITY OF FREE PHENOLIC COMPOUNDS AND OLIGOSACCHARIDES FROM CORN (ZEA MAYS L.) AND COMMON BEAN (PHASEOLUS VULGARIS L.) CHIPS DURING IN VITRO GASTROINTESTINAL DIGESTION AND SIMULATED COLONIC FERMENTATION	10.1016/j.foodres.2017.07.018
BIOACCESSIBILITY AND ANTIOXIDANT ACTIVITY OF PHENOLICS IN NATIVE AND FERMENTED PRINSEPIA UTILIS ROYLE SEED DURING A SIMULATED GASTROINTESTINAL DIGESTION IN VITRO	10.1016/j.jff.2017.08.004
BIOACCESSIBILITY AND BIOAVAILABILITY OF A MARINE-DERIVED MULTIMINERAL, AQUAMIN-MAGNESIUM	10.3390/nu10070912
BIOACCESSIBILITY AND CATABOLISM OF PHENOLIC COMPOUNDS FROM JABOTICABA (MYRCIARIA TRUNCIFLORA) FRUIT PEEL DURING IN VITRO GASTROINTESTINAL DIGESTION AND COLONIC FERMENTATION	10.1016/j.jff.2019.103714
BIOACCESSIBILITY AND TRANSFORMATION PATHWAYS OF PHENOLIC COMPOUNDS IN PROCESSED MULBERRY (MORUS ALBA L.) LEAVES AFTER IN VITRO GASTROINTESTINAL DIGESTION AND FAECAL FERMENTATION	10.1016/j.jff.2019.06.008
BIOACCESSIBILITY DURING IN VITRO DIGESTION AND ANTIPROLIFERATIVE EFFECT OF BIOACTIVE COMPOUNDS FROM ANDEAN BERRY (VACCINIUM MERIDIONALE SWARTZ) JUICE	10.1021/acs.jafc.8b01604
BIOACCESSIBILITY OF (POLY)PHENOLIC COMPOUNDS OF RAW AND COOKED CARDOON (CYNARA CARDUNCULUS L.) AFTER SIMULATED GASTROINTESTINAL DIGESTION AND FERMENTATION BY HUMAN COLONIC MICROBIOTA	10.1016/j.jff.2017.02.033
BIOACCESSIBILITY OF APIGENIN FROM MANGIFERA INDICA (WATER LILY VAR.) DURING IN VITRO GASTROINTESTINAL DIGESTION	
BIOACCESSIBILITY OF ARSENIC BOUND TO CORUNDUM USING A SIMULATED GASTROINTESTINAL SYSTEM	10.1071/EN05067
BIOACCESSIBILITY OF ARSENIC FROM GASTROPOD ALONG THE XIANGJIANG RIVER: ASSESSING HUMAN HEALTH RISKS USING AN IN VITRO DIGESTION MODEL	10.1016/j.ecoenv.2020.110334
BIOACCESSIBILITY OF ARSENIC(V) BOUND TO FERRIHYDRITE USING A SIMULATED GASTROINTESTINAL SYSTEM	10.1021/es0516413
BIOACCESSIBILITY OF CASHEW NUT KERNEL FLOUR COMPOUNDS RELEASED AFTER SIMULATED IN VITRO HUMAN GASTROINTESTINAL DIGESTION	10.1016/j.foodres.2020.109906
BIOACCESSIBILITY OF LEAD SEQUESTERED TO CORUNDUM AND FERRIHYDRITE IN A SIMULATED GASTROINTESTINAL SYSTEM	10.2134/jeq2005.0467
BIOACCESSIBILITY OF MERCURY FROM TRADITIONAL NORTHERN COUNTRY FOODS MEASURED USING AN IN VITRO GASTROINTESTINAL MODEL IS INDEPENDENT OF MERCURY CONCENTRATION	10.1016/j.scitotenv.2009.08.014
BIOACCESSIBILITY OF METAL CATIONS IN SOIL IS LINEARLY RELATED TO ITS WATER EXCHANGE RATE CONSTANT	10.1021/es103710a
BIOACCESSIBILITY OF MICRONUTRIENTS IN FRESH AND FROZEN STRAWBERRY FRUITS GROWN UNDER ELEVATED CARBON DIOXIDE AND TEMPERATURE	10.1016/j.foodchem.2019.125662

BIOACCESSIBILITY OF PHENOLIC COMPOUNDS AND ANTIOXIDANT CAPACITY OF CHIA (SALVIA HISPANICA L.) SEEDS	10.1007/s11130-017-0649-7
BIOACCESSIBILITY OF POLYBROMINATED DIPHENYL ETHERS AND THEIR METHOXYLATED METABOLITES IN COOKED SEAFOOD AFTER USING A MULTI-COMPARTMENT IN VITRO DIGESTION MODEL	10.1016/j.chemosphere.2020.126462
BIOACCESSIBILITY OF POLYPHENOLIC COMPOUNDS OF SIX QUINOA SEEDS DURING IN VITRO GASTROINTESTINAL DIGESTION	10.1016/j.jff.2017.08.042
BIOACCESSIBILITY OF POLYPHENOLS ASSOCIATED WITH DIETARY FIBER AND IN VITRO KINETICS RELEASE OF POLYPHENOLS IN MEXICAN 'ATAULFO' MANGO (MANGIFERA INDICA L.) BY-PRODUCTS	10.1039/c4fo00982g
BIOACCESSIBILITY OF PROVITAMIN A CAROTENOIDS FROM FRUITS: APPLICATION OF A STANDARDISED STATIC IN VITRO DIGESTION METHOD	10.1039/c5fo01242b
BIOACCESSIBILITY OF RUTIN, CAFFEIC ACID AND ROSMARINIC ACID: INFLUENCE OF THE IN VITRO GASTROINTESTINAL DIGESTION MODELS	10.1016/j.jff.2016.08.003
BIOACCESSIBILITY OF SELENIUM FROM COOKED RICE AS DETERMINED IN A SIMULATOR OF THE HUMAN INTESTINAL TRACT (SHIME)	10.1002/jsfa.8208
BIOACCESSIBILITY OF SOME ESSENTIAL MINERALS IN THREE SELECTED AUSTRALIAN PULSE VARIETIES USING AN IN VITRO GASTROINTESTINAL DIGESTION MODEL	10.1111/1750-3841.14377
BIOACCESSIBILITY OF TUDELA ARTICHOKE (CYNARA SCOLYMUS CV. BLANCA DE TUDELA) (POLY)PHENOLS: THE EFFECTS OF HEAT TREATMENT, SIMULATED GASTROINTESTINAL DIGESTION AND HUMAN COLONIC MICROBIOTA	10.1039/d0fo03119d
BIOACCESSIBILITY, ANTIOXIDANT ACTIVITY AND MODULATION EFFECT ON GUT MICROBIOTA OF BIOACTIVE COMPOUNDS FROM MORINGA OLEIFERA LAM. LEAVES DURING DIGESTION AND FERMENTATION IN VITRO	10.1039/c9fo00793h
BIOACCESSIBILITY, BIOAVAILABILITY, AND ANTI-INFLAMMATORY EFFECTS OF ANTHOCYANINS FROM PURPLE ROOT VEGETABLES USING MONO- AND CO-CULTURE CELL MODELS	10.1002/mnfr.201600928
BIOACCESSIBILITY, CHANGES IN THE ANTIOXIDANT POTENTIAL AND COLONIC FERMENTATION OF DATE PITS AND APPLE BAGASSE FLOURS OBTAINED FROM CO-PRODUCTS DURING SIMULATED IN VITRO GASTROINTESTINAL DIGESTION	10.1016/j.foodres.2015.10.021
BIOACTIVE PEPTIDES RELEASED FROM IN VITRO DIGESTION OF HUMAN MILK WITH OR WITHOUT PASTEURIZATION	10.1038/pr.2015.10
BIOACTIVITY AND CELL METABOLISM OF IN VITRO DIGESTED SWEET CHERRY (PRUNUS AVIUM) PHENOLIC COMPOUNDS	10.1080/09637486.2018.1513996
BIOAMINERGIC RESPONSES IN AN IN VITRO SYSTEM STUDYING HUMAN GUT MICROBIOTA–KIWIFRUIT INTERACTIONS	10.3390/microorganisms8101582
BIOAVAILABILITY AND ANTIOXIDANT POTENTIALS OF FRESH AND PASTEURIZED KIWI JUICE BEFORE AND AFTER IN VITRO GASTROINTESTINAL DIGESTION	10.1007/s13197-020-04467-6
BIOAVAILABILITY OF BLACK TEA THEAFLAVINS: ABSORPTION, METABOLISM, AND COLONIC CATABOLISM	10.1021/acs.jafc.7b01707
BIOCOMPATIBILITY OF A LAB-ON-A-PILL SENSOR IN ARTIFICIAL GASTROINTESTINAL ENVIRONMENTS	10.1109/TBME.2006.883698
BIOCONVERSION BY GUT MICROBIOTA OF PREDIGESTED MANGO (MANGIFERA INDICA L) 'ATAULFO' PEEL POLYPHENOLS ASSESSED IN A DYNAMIC (TIM-2) IN VITRO MODEL OF THE HUMAN COLON	10.1016/j.foodres.2020.109963
BIOCONVERSION OF POLYPHENOLS AND ORGANIC ACIDS BY GUT MICROBIOTA OF PREDIGESTED HIBISCUS SABDARIFFA L. CALYCES AND AGAVE (A. TEQUILANA WEBER) FRUCTANS ASSESSED IN A DYNAMIC IN VITRO MODEL (TIM-2) OF THE HUMAN COLON	10.1016/j.foodres.2021.110301
BIODIVERSITY OF HUMAN FAECAL BACTERIA ISOLATED FROM PHYTIC ACID ENRICHED CHEMOSTAT FERMENTERS	
BIOEFFICACY OF TEA CATECHINS ASSOCIATED WITH MILK CASEINS TESTED USING DIFFERENT IN VITRO DIGESTION MODELS	10.1007/s13228-014-0035-y

BIOFILM PRODUCING INDIGENOUS BACTERIA ISOLATED FROM MUNICIPAL SLUDGE AND THEIR NUTRIENT REMOVAL ABILITY IN MOVING BED BIOFILM REACTOR FROM THE WASTEWATER	10.1016/j.sjbs.2021.06.084
BIOFILMS HARBOUR CLOSTRIDIOIDES DIFFICILE, SERVING AS A RESERVOIR FOR RECURRENT INFECTION	10.1038/s41522-021-00184-w
BIOFILMS IN DRINKING WATER SYSTEMS - A POSSIBLE RESERVOIR FOR HELICOBACTER PYLORI	10.2166/wst.1998.0537
BIOGENIC AMINE PRODUCTION BY THE WINE LACTOBACILLUS BREVIS IOEB 9809 IN SYSTEMS THAT PARTIALLY MIMIC THE GASTROINTESTINAL TRACT STRESS	10.1186/1471-2180-12-247
BIOMIMETIC HUMAN DISEASE MODEL OF SARS-COV-2-INDUCED LUNG INJURY AND IMMUNE RESPONSES ON ORGAN CHIP SYSTEM	10.1002/advs.202002928
BIOPROCESSING OF WHEAT BRAN IMPROVES IN VITRO BIOACCESSIBILITY AND COLONIC METABOLISM OF PHENOLIC COMPOUNDS	10.1021/jf900492h
BIOREACTOR WITH ELECTRICALLY DEFORMABLE CURVED MEMBRANES FOR MECHANICAL STIMULATION OF CELL CULTURES	10.3389/fbioe.2020.00022
BIOSORPTION OF BIOCOMPOUNDS FROM WHITE AND GREEN TEA IN SACCHAROMYCES CEREVISIAE WASTE: STUDY OF THE SECONDARY METABOLITES BY UPLC-QTOF-MS AND SIMULATED IN VITRO GASTROINTESTINAL DIGESTION	10.1016/j.fbio.2021.101001
BIOTRANSFORMATION OF POLYPHENOLS IN A DYNAMIC MULTISTAGE GASTROINTESTINAL MODEL	10.1016/j.foodchem.2016.02.140
BIOVOLATILIZATION OF METAL(LOID)S BY INTESTINAL MICROORGANISMS IN THE SIMULATOR OF THE HUMAN INTESTINAL MICROBIAL ECOSYSTEM	10.1021/es900544c
BIRCH PULP XYLAN WORKS AS A FOOD HYDROCOLLOID IN ACID MILK GELS AND IS FERMENTED SLOWLY IN VITRO	10.1016/j.carbpol.2016.06.028
BLUEBERRY PECTIN AND INCREASED ANTHOCYANINS STABILITY UNDER IN VITRO DIGESTION	10.1016/j.foodchem.2019.125343
BREAKDOWN MECHANISMS OF WHEY PROTEIN GELS DURING DYNAMIC IN VITRO GASTRIC DIGESTION	10.1039/d0fo03325a
BREAST-MILK DERIVED POTENTIAL PROBIOTICS AS STRATEGY FOR THE MANAGEMENT OF CHILDHOOD OBESITY	10.1016/j.foodres.2020.109673
BREWER'S SPENT GRAIN ENHANCED THE RECOVERY OF POTENTIAL PROBIOTIC STRAINS IN FERMENTED MILK AFTER EXPOSURE TO IN VITRO-SIMULATED GASTROINTESTINAL CONDITIONS	10.1007/s12602-021-09839-8
BUFFALO MILK INCREASES VIABILITY AND RESISTANCE OF PROBIOTIC BACTERIA IN DAIRY BEVERAGES UNDER IN VITRO SIMULATED GASTROINTESTINAL CONDITIONS	10.3168/jds.2019-18078
BUTYRATE-PRODUCING BACTERIA SUPPLEMENTED IN VITRO TO CROHN'S DISEASE PATIENT MICROBIOTA INCREASED BUTYRATE PRODUCTION AND ENHANCED INTESTINAL EPITHELIAL BARRIER INTEGRITY	10.1038/s41598-017-11734-8
BUTYRATE-PRODUCING CLOSTRIDIUM CLUSTER XIVA SPECIES SPECIFICALLY COLONIZE MUCINS IN AN IN VITRO GUT MODEL	10.1038/ismej.2012.158
CACO-2 CELL PERMEABILITY AND STABILITY OF TWO D-GLUCOPYRANURONAMIDE CONJUGATES OF THYROTROPIN-RELEASING HORMONE	10.1016/j.bmc.2007.04.042
CAFFEOYLQUINIC ACIDS AND FLAVONOIDS OF FRINGED SAGEWORT (ARTEMISIA FRIGIDAWILLD.): HPLC-DAD-ESI-QQQ-MS PROFILE, HPLC-DAD QUANTIFICATION, IN VITRO DIGESTION STABILITY, AND ANTIOXIDANT CAPACITY	10.3390/antiox8080307
CASHEW APPLE JUICE CONTAINING GLUCO-OLIGOSACCHARIDES, DEXTRAN, AND TAGATOSE PROMOTES PROBIOTIC MICROBIAL GROWTH	10.1016/j.fbio.2021.101080
CATABOLISM OF RAW AND COOKED GREEN PEPPER (CAPSICUM ANNUUM) (POLY)PHENOLIC COMPOUNDS AFTER SIMULATED GASTROINTESTINAL DIGESTION AND FAECAL FERMENTATION	10.1016/j.jff.2016.09.006
CEREAL BARS FUNCTIONALIZED THROUGH: BIFIDOBACTERIUM ANIMALIS SUBSP. LACTIS BB-12 AND INULIN INCORPORATED IN EDIBLE COATINGS OF WHEY PROTEIN ISOLATE OR ALGINATE	10.1039/c9fo00370c
CHANGES IN AMINO ACID COMPOSITION DURING FERMENTATION AND ITS EFFECTS ON THE INHIBITORY ACTIVITY OF ANGIOTENSIN-I-CONVERTING ENZYME OF JACK BEAN TEMPE FOLLOWING IN VITRO GASTROINTESTINAL DIGESTION	

CHANGES IN BIOACCESSIBILITY, POLYPHENOL PROFILE AND ANTIOXIDANT POTENTIAL OF FLOURS OBTAINED FROM PERSIMMON FRUIT (DIOSPYROS KAKI) CO-PRODUCTS DURING IN VITRO GASTROINTESTINAL DIGESTION	10.1016/j.foodchem.2018.02.128
CHANGES IN COMPOSITION AND FUNCTION OF HUMAN INTESTINAL MICROBIOTA EXPOSED TO CHLORPYRIFOS IN OIL AS ASSESSED BY THE SHIME® MODEL	10.3390/ijerph13111088
CHANGES IN GUT MICROBIOTA IN PREDIGESTED HIBISCUS SABDARIFFA L CALYCES AND AGAVE (AGAVE TEQUILANA WEBER) FRUCTANS ASSESSED IN A DYNAMIC IN VITRO MODEL (TIM-2) OF THE HUMAN COLON	10.1016/j.foodres.2020.109036
CHANGES IN INTESTINAL MICROBIOTA AND PREDICTED METABOLIC PATHWAYS DURING COLONIC FERMENTATION OF MANGO (MANGIFERA INDICA L.)—BASED BAR INDIGESTIBLE FRACTION	10.3390/nu12030683
CHANGES IN MICROBIAL PATHOGEN DYNAMICS DURING VERMICOMPOSTING MIXTURE OF COW MANURE–ORGANIC SOLID WASTE AND COW MANURE–SEWAGE SLUDGE	10.1007/s40093-016-0152-4
CHANGES IN PHYSICOCHEMICAL AND BIOLOGICAL PROPERTIES OF POLYPHENOLIC-PROTEIN-POLYSACCHARIDE TERNARY COMPLEXES FROM HOVENIA DULCIS AFTER IN VITRO SIMULATED SALIVA-GASTROINTESTINAL DIGESTION	10.3390/foods10102322
CHANGES IN THE ANTIOXIDANT PROPERTIES OF RICE BRAN PROTEIN ISOLATE UPON SIMULATED GASTROINTESTINAL DIGESTION	10.1016/j.lwt.2020.109206
CHANGES IN THE ORGANOSULFUR AND POLYPHENOL COMPOUND PROFILES OF BLACK AND FRESH ONION DURING SIMULATED GASTROINTESTINAL DIGESTION	10.3390/foods10020337
CHANGES IN THE STABILITY AND ANTIOXIDANT ACTIVITIES OF DIFFERENT MOLECULAR WEIGHT BIOACTIVE PEPTIDE EXTRACTS OBTAINED FROM BEEF DURING IN VITRO HUMAN DIGESTION BY GUT MICROBIOTA	10.1016/j.foodres.2021.110116
CHANGES OF ANTIBIOTIC RESISTANCE PHENOTYPE IN OUTBREAK-LINKED SALMONELLA ENTERICA STRAINS AFTER EXPOSURE TO HUMAN SIMULATED GASTROINTESTINAL CONDITIONS IN CHICKEN MEAT	10.4315/0362-028X.JFP-18-213
CHARACTERISATION OF IN¬†VITRO GASTROINTESTINAL DIGESTS FROM LOW FAT CAPRINE KEFIR ENRICHED WITH INULIN	10.1016/j.idairyj.2017.07.004
CHARACTERISTIC OF POLYSACCHARIDES FROM FLAMMULINA VELUTIPES IN¬†VITRO DIGESTION UNDER SALIVARY, SIMULATED GASTRIC AND SMALL INTESTINAL CONDITIONS AND FERMENTATION BY HUMAN GUT MICROBIOTA	10.1111/ijfs.14142
CHARACTERIZATION AND ANTIMICROBIAL ACTIVITY OF LACTIC ACID BACTERIA FROM FERMENTATIVE BIOREACTORS DURING HYDROGEN PRODUCTION USING CASSAVA PROCESSING WASTEWATER	10.1016/j.cej.2015.08.088
CHARACTERIZATION AND IN VITRO PROPERTIES OF POTENTIAL PROBIOTIC BIFIDOBACTERIUM STRAINS ISOLATED FROM BREAST-FED INFANT FECES	10.1007/s13213-015-1187-x
CHARACTERIZATION OF A HUMAN IN VITRO INTESTINAL MODEL FOR THE HAZARD ASSESSMENT OF NANOMATERIALS USED IN CANCER IMMUNOTHERAPY	10.3390/app11052113
CHARACTERIZATION OF AN ENGINEERED LIVE BACTERIAL THERAPEUTIC FOR THE TREATMENT OF PHENYLKETONURIA IN A HUMAN GUT-ON-A-CHIP	10.1038/s41467-021-23072-5
CHARACTERIZATION OF BIOFILM BACTERIAL COMMUNITIES IN A VERTICAL UNSATURATED-FLOW BIOREACTOR TREATING DOMESTIC GREYWATER	10.1007/s40710-016-0162-2
CHARACTERIZATION OF EDIBLE SWIFTLET'S NEST AS A PREBIOTIC INGREDIENT USING A SIMULATED COLON MODEL	10.1007/s13213-019-01507-1
CHARACTERIZATION OF FUNCTIONAL PROPERTIES OF ENTEROCOCCUS FAECIUM STRAINS ISOLATED FROM HUMAN GUT	10.1139/cjm-2015-0446
CHARACTERIZATION OF FUNCTIONAL, SAFETY, AND PROBIOTIC PROPERTIES OF ENTEROCOCCUS FAECALIS AG5 ISOLATED FROM WISTAR RAT, DEMONSTRATING ADHERENCE TO HCT 116 CELLS AND GASTROINTESTINAL SURVIVABILITY	10.1007/s12602-018-9387-x
CHARACTERIZATION OF INDIVIDUAL PARTICLE MOVEMENT DURING IN VITRO GASTRIC DIGESTION IN THE HUMAN GASTRIC SIMULATOR (HGS)	10.1016/j.jfoodeng.2019.07.021

CHARACTERIZATION OF LACTOBACILLI STRAINS DERIVED FROM COCOA FERMENTATION IN THE SOUTH OF BAHIA FOR THE DEVELOPMENT OF PROBIOTIC CULTURES	10.1016/j.lwt.2016.06.003
CHARACTERIZATION OF MICROBIAL METABOLISM OF SYRAH GRAPE PRODUCTS IN AN IN VITRO COLON MODEL USING TARGETED AND NON-TARGETED ANALYTICAL APPROACHES	10.1007/s00394-012-0391-8
CHARACTERIZATION OF PLANKTONIC AND BIOFILM COMMUNITIES OF DAY-OF-HATCH CHICKS CECAL MICROFLORA AND THEIR RESISTANCE TO SALMONELLA COLONIZATION	10.4315/0362-028X-72.5.959
CHARACTERIZATION OF POLYSACCHARIDE FROM PLEUROTUS ERYNGII DURING SIMULATED GASTROINTESTINAL DIGESTION AND FERMENTATION	10.1016/j.foodchem.2021.131303
CHARACTERIZATION OF PREBIOTICS AND THEIR SYNERGISTIC ACTIVITIES WITH LACTOBACILLUS PROBIOTICS FOR Œí-GLUCURONIDASE REDUCTION	10.2306/scienceasia1513-1874.2019.45.538
CHARACTERIZATION OF RHODOPSEUDOMONAS PALUSTRIS STRAIN 2C AS A POTENTIAL PROBIOTIC	10.1111/j.1600-0463.2012.02902.x
CHARACTERIZATION OF THE BACTERICIDAL EFFECT OF DIETARY SPHINGOSINE AND ITS ACTIVITY UNDER INTESTINAL CONDITIONS	10.1016/j.ijfoodmicro.2005.05.007
CHARACTERIZATION OF THE PEPTIDE FRACTION FROM DIGESTED PARMIGIANO REGGIANO CHEESE AND ITS EFFECT ON GROWTH OF LACTOBACILLI AND BIFIDOBACTERIA	10.1016/j.ijfoodmicro.2017.05.015
CHEMICAL AND NUTRITIONAL PROPERTIES OF WHITE BREAD LEAVENED BY LACTIC ACID BACTERIA	10.1016/j.jff.2018.04.030
CHEMICAL COMPOSITION, IN VITRO BIOACCESSIBILITY AND ANTIOXIDANT ACTIVITY OF POLYPHENOLIC COMPOUNDS FROM NUTRACEUTICAL FENNEL WASTE EXTRACT	10.3390/molecules26071968
CHEMOPREVENTIVE EFFECTS FROM PREBIOTIC INULIN TOWARDS MICROBIAL 2-AMINO-1-METHYL-6-PHENYLIMIDAZO[4,5-B]PYRIDINE BIOACTIVATION	10.1111/j.1365-2672.2008.04015.x
CHEMOPREVENTIVE EFFECTS OF RAW AND ROASTED OAT FLAKES AFTER IN¬†VITRO FERMENTATION WITH HUMAN FAECAL MICROBIOTA	10.1080/09637486.2020.1772205
CHEMOPREVENTIVE POTENTIAL OF POWDERED RED WINE POMACE SEASONINGS AGAINST COLORECTAL CANCER IN HT-29 CELLS	10.1021/acs.jafc.6b04561
CHITIN GLUCAN SHIFTS LUMINAL AND MUCOSAL MICROBIAL COMMUNITIES, IMPROVE EPITHELIAL BARRIER AND MODULATES CYTOKINE PRODUCTION IN VITRO	10.3390/nu13093249
CHITOSAN INTERACTION WITH IRON FROM YOGHURT USING AN IN VITRO DIGESTIVE MODEL: COMPARATIVE STUDY WITH PLANT DIETARY FIBERS	10.3390/ijms12074647
CHLORELLA VULGARIS IN A HETEROTROPHIC BIOPROCESS: STUDY OF THE LIPID BIOACCESSIBILITY AND OXIDATIVE STABILITY	10.1016/j.algal.2019.101754
CHOCOLATE: AN IDEAL CARRIER FOR PROBIOTICS	
CIPROFLOXACIN AT LOW LEVELS DISRUPTS COLONIZATION RESISTANCE OF HUMAN FECAL MICROFLORA GROWING IN CHEMOSTATS	10.1016/j.yrtph.2004.08.005
CLONING OF ENVIRONMENTAL GENOMIC FRAGMENTS AS PHYSICAL MARKERS FOR MONITORING MICROBIAL POPULATIONS IN COKING WASTEWATER TREATMENT SYSTEM	10.1007/s00248-006-9157-2
CLOSTRIDIUM DIFFICILE COLONIZATION AND ANTIBIOTICS RESPONSE IN POLYFERMS CONTINUOUS MODEL MIMICKING ELDERLY INTESTINAL FERMENTATION	10.1186/s13099-016-0144-y
CLOSTRIDIUM DIFFICILE TREHALOSE METABOLISM VARIANTS ARE COMMON AND NOT ASSOCIATED WITH ADVERSE PATIENT OUTCOMES WHEN VARIABLY PRESENT IN THE SAME LINEAGE	10.1016/j.ebiom.2019.04.038
CLOSTRIDIUM PERFRINGENS SUPPRESSING ACTIVITY IN BLACK SOLDIER FLY PROTEIN PREPARATIONS	10.1016/j.lwt.2021.111806
CO-AMOXICLAV INDUCES PROLIFERATION AND CYTOTOXIN PRODUCTION OF CLOSTRIDIUM DIFFICILE RIBOTYPE 027 IN A HUMAN GUT MODEL	10.1093/jac/dkr584
CO-CULTURE OF BIFIDOBACTERIUM ADOLESCENTIS AND BACTEROIDES THETAIOTAOMICRON IN ARABINOGALACTAN-LIMITED CHEMOSTATS: EFFECTS OF DILUTION RATE AND PH	10.1006/anae.1995.1027
CO-ENCAPSULATED SYNBIOTICS AND IMMOBILIZED PROBIOTICS IN HUMAN HEALTH AND GUT MICROBIOTA MODULATION	10.3390/foods10061297

CO-ENCAPSULATION OF LACTOBACILLUS HELVETICUS CELLS AND GREEN TEA EXTRACT: INFLUENCE ON CELL SURVIVAL IN SIMULATED GASTROINTESTINAL CONDITIONS	10.1016/j.jff.2016.08.002
CO-PRODUCTS OF BEEF PROCESSING ENHANCE NON-HAEM IRON ABSORPTION IN AN IN¬†VITRO DIGESTION/CACO-2 CELL MODEL	10.1111/ijfs.14049
COEXISTENCE OF SILVER ION AND TETRACYCLINE AT ENVIRONMENTALLY RELEVANT CONCENTRATIONS GREATLY ENHANCED ANTIBIOTIC RESISTANCE GENE DEVELOPMENT IN ACTIVATED SLUDGE BIOREACTOR	10.1016/j.jhazmat.2021.127088
COLISTIN AND AMOXICILLIN COMBINATORIAL EXPOSURE ALTERS THE HUMAN INTESTINAL MICROBIOTA AND ANTIBIOTIC RESISTOME IN THE SIMULATED HUMAN INTESTINAL MICROBIOTA	10.1016/j.scitotenv.2020.141415
COLON BIOACCESSIBILITY AND ANTIOXIDANT ACTIVITY OF WHITE, GREEN AND BLACK TEA POLYPHENOLS EXTRACT AFTER IN VITRO SIMULATED GASTROINTESTINAL DIGESTION	10.3390/nu10111711
COLON BIOACCESSIBILITY UNDER IN VITRO GASTROINTESTINAL DIGESTION OF A RED CABBAGE EXTRACT CHEMICALLY PROFILED THROUGH UHPLC,ÄêQ,ÄêORBITRAP HRMS	10.3390/antiox9100955
COLON BIOACCESSIBILITY UNDER IN VITRO GASTROINTESTINAL DIGESTION OF DIFFERENT COFFEE BREWS CHEMICALLY PROFILED THROUGH UHPLC-Q-ORBITRAP HRMS	10.3390/foods10010179
COLONIC BACTERIAL METABOLISM OF CORTICOSTEROIDS	10.1016/j.ijpharm.2013.09.007
COLONIC CATABOLISM OF DIETARY PHENOLIC AND POLYPHENOLIC COMPOUNDS FROM CONCORD GRAPE JUICE	10.1039/c2fo30151b
COLONIC FERMENTATION OF POLYPHENOLS FROM CHILEAN CURRANTS (RIBES SPP.) AND ITS EFFECT ON ANTIOXIDANT CAPACITY AND METABOLIC SYNDROME-ASSOCIATED ENZYMES	10.1016/j.foodchem.2018.03.053
COLONIC IN VITRO MODEL ASSESSMENT OF THE PREBIOTIC POTENTIAL OF BREAD FORTIFIED WITH POLYPHENOLS RICH OLIVE FIBER	10.3390/nu13030787
COLONIC TRANSIT TIME IS A DRIVEN FORCE OF THE GUT MICROBIOTA COMPOSITION AND METABOLISM: IN VITRO EVIDENCE	10.5056/jnm16042
COMBINED EFFECTS OF LOW-FAT ICE CREAM SUPPLEMENTED WITH PROBIOTICS ON COLON MICROFLORAL COMMUNITIES AND THEIR METABOLITES DURING FERMENTATION IN A HUMAN GUT REACTOR	10.1016/j.fbio.2016.12.005
COMBINING IN VITRO DIGESTION MODEL WITH CELL CULTURE MODEL: ASSESSMENT OF ENCAPSULATION AND DELIVERY OF CURCUMIN IN MILLED STARCH PARTICLE STABILIZED PICKERING EMULSIONS	10.1016/j.ijbiomac.2019.08.078
COMINIGUT-A SMALL VOLUME IN VITRO COLON MODEL FOR THE SCREENING OF GUT MICROBIAL FERMENTATION PROCESSES	10.7717/peerj.4268
COMMENSAL E. COLI RAPIDLY TRANSFER ANTIBIOTIC RESISTANCE GENES TO HUMAN INTESTINAL MICROBIOTA IN THE MUCOSAL SIMULATOR OF THE HUMAN INTESTINAL MICROBIAL ECOSYSTEM (M-SHIME)	10.1016/j.ijfoodmicro.2019.108357
COMMENT ON: RIFAXIMIN MODULATES THE COLONIC MICROBIOTA OF PATIENTS WITH CROHN'S DISEASE: AN IN VITRO APPROACH USING A CONTINUOUS CULTURE COLONIC MODEL SYSTEM	10.1093/jac/dkr031
COMMERCIAL WHEY PRODUCTS PROMOTE INTESTINAL BARRIER FUNCTION WITH GLYCOMACROPEPTIDE ENHANCED ACTIVITY IN DOWNREGULATING BACTERIAL ENDOTOXIN LIPOPOLYSACCHARIDES (LPS)-INDUCED INFLAMMATION: IN VITRO	10.1039/d0fo00487a
COMPARATIVE ANALYSIS OF THE GENE EXPRESSION PROFILE OF PROBIOTIC LACTOBACILLUS CASEI ZHANG WITH AND WITHOUT FERMENTED MILK AS A VEHICLE DURING TRANSIT IN A SIMULATED GASTROINTESTINAL TRACT	10.1016/j.resmic.2012.04.002
COMPARATIVE ANALYSIS OF THE GUT MICROBIOTA CULTURED IN VITRO USING A SINGLE COLON VERSUS A 3-STAGE COLON EXPERIMENTAL DESIGN	10.1007/s00253-021-11241-x
COMPARATIVE EFFECTS OF EXOPOLYSACCHARIDES FROM LACTIC ACID BACTERIA AND FRUCTO-OLIGOSACCHARIDES ON INFANT GUT MICROBIOTA TESTED IN AN IN VITRO COLONIC MODEL WITH IMMOBILIZED CELLS	10.1111/j.1574-6941.2006.00118.x
COMPARATIVE FERMENTATION OF INSOLUBLE CARBOHYDRATES IN AN IN VITRO HUMAN FECES MODEL SPIKED WITH LACTOBACILLUS ACIDOPHILUS NCFM	10.1002/star.201200091

COMPARATIVE GENOMICS OF BIFIDOBACTERIUM SPECIES ISOLATED FROM MARMOSETS AND HUMANS	10.1002/ajp.22983
COMPARATIVE IN VITRO FERMENTATIONS OF CRANBERRY AND GRAPE SEED POLYPHENOLS WITH COLONIC MICROBIOTA	10.1016/j.foodchem.2015.03.061
COMPARATIVE METHODS FOR FECAL SAMPLE STORAGE TO PRESERVE GUT MICROBIAL STRUCTURE AND FUNCTION IN AN IN VITRO MODEL OF THE HUMAN COLON	10.1007/s00253-020-10959-4
COMPARATIVE PHENOTYPIC CHARACTERIZATION OF HYBRID SHIGA TOXIN-PRODUCING / UROPATHOGENIC ESCHERICHIA COLI, CANONICAL UROPATHOGENIC AND SHIGA TOXIN-PRODUCING ESCHERICHIA COLI	10.1016/j.ijmm.2021.151533
COMPARATIVE RESISTANCE OF FOOD PROTEINS TO ADULT AND INFANT IN VITRO DIGESTION MODELS	10.1002/mnfr.200900142
COMPARISON OF ANTIOXIDANT ACTIVITIES OF BOVINE WHEY PROTEINS BEFORE AND AFTER SIMULATED GASTROINTESTINAL DIGESTION	10.3168/jds.2018-14581
COMPARISON OF CONVENTIONAL PLATING, PMA-QPCR, AND FLOW CYTOMETRY FOR THE DETERMINATION OF VIABLE ENTEROTOXIGENIC ESCHERICHIA COLI ALONG A GASTROINTESTINAL IN VITRO MODEL	10.1007/s00253-018-9380-z
COMPARISON OF DIFFERENT SOLUBLE DIETARY FIBERS DURING THE IN VITRO FERMENTATION PROCESS	10.1021/acs.jafc.1c00237
COMPARISON OF FIVE IN VITRO DIGESTION MODELS TO IN VIVO EXPERIMENTAL RESULTS: LEAD BIOACCESSIBILITY IN THE HUMAN GASTROINTESTINAL TRACT	10.1080/10934520701434919
COMPARISON OF ORITAVANCIN VERSUS VANCOMYCIN AS TREATMENTS FOR CLINDAMYCIN-INDUCED CLOSTRIDIUM DIFFICILE PCR RIBOTYPE 027 INFECTION IN A HUMAN GUT MODEL	10.1093/jac/dkn358
COMPARISON OF PLANKTONIC AND BIOFILM-ASSOCIATED COMMUNITIES OF CLOSTRIDIUM DIFFICILE AND INDIGENOUS GUT MICROBIOTA IN A TRIPLE-STAGE CHEMOSTAT GUT MODEL	10.1093/jac/dku116
COMPARISON OF PREBIOTIC EFFECTS OF ARABINOXYLAN OLIGOSACCHARIDES AND INULIN IN A SIMULATOR OF THE HUMAN INTESTINAL MICROBIAL ECOSYSTEM	10.1111/j.1574-6941.2009.00712.x
COMPARISON OF PROTECTION AND RELEASE BEHAVIOR OF DIFFERENT CAPSULE POLYMER COMBINATIONS BASED ON L. ACIDOPHILUS SURVIVABILITY AND FUNCTION AND CAFFEINE RELEASE	10.1016/j.ijpharm.2021.120977
COMPARISON OF SYNBIOTIC BEVERAGES PRODUCED FROM RICEBERRY MALT EXTRACT USING SELECTED FREE AND ENCAPSULATED PROBIOTIC LACTIC ACID BACTERIA	10.1016/j.anres.2018.11.013
COMPARISON OF THE BIFIDOGENIC EFFECTS OF GOAT AND COW MILK-BASED INFANT FORMULAS TO HUMAN BREAST MILK IN AN IN VITRO GUT MODEL FOR 3-MONTH-OLD INFANTS	10.3389/fnut.2020.608495
COMPETITION FOR GLUCOSE BETWEEN CANDIDA ALBICANS AND ORAL BACTERIA GROWN IN MIXED CULTURE IN A CHEMOSTAT	10.1099/0022-1317-49-11-969
COMPLEMENTARY MECHANISMS FOR DEGRADATION OF INULIN-TYPE FRUCTANS AND ARABINOXYLAN OLIGOSACCHARIDES AMONG BIFIDOBACTERIAL STRAINS SUGGEST BACTERIAL COOPERATION	10.1128/AEM.02893-17
COMPOUND CHARACTERIZATION AND METABOLIC PROFILE ELUCIDATION AFTER IN VITRO GASTROINTESTINAL AND HEPATIC BIOTRANSFORMATION OF AN HERNIARIA HIRSUTA EXTRACT USING UNBIASED DYNAMIC METABOLOMIC DATA ANALYSIS	10.3390/metabo10030111
COMPUGUT: AN IN SILICO PLATFORM FOR SIMULATING INTESTINAL FERMENTATION	10.1016/j.softx.2017.06.004
CONCENTRATIONS OF ORGANOCHLORINE PESTICIDES (OCPS) IN HUMAN BLOOD PLASMA FROM HONG KONG: MARKERS OF EXPOSURE AND SOURCES FROM FISH	10.1016/j.envint.2013.01.003
CONSEQUENCES OF BIOFILM AND SESSILE GROWTH IN THE LARGE INTESTINE.	10.1177/08959374970110011801
CONSTRUCTION OF A GENETICALLY ENGINEERED MICROORGANISM WITH HIGH TOLERANCE TO ARSENITE AND STRONG ARSENITE OXIDATIVE ABILITY	10.1080/10934521003648958
CONTINUOUS CULTURE OF HUMAN FAECAL BACTERIA AS AN IN VITRO MODEL FOR THE COLONIC MICROFLORA	10.1016/0887-2333(90)90111-6
CONTINUOUS FLOW ONE CHAMBER CHEMOSTAT: EFFECT OF SELECTED ANTIMICROBIALS ON THE HUMAN INTESTINAL MICROFLORA	10.3402/mehd.v12i1.8049
CONTINUOUS PRODUCTION OF OLIGODEXTRANS VIA CONTROLLED HYDROLYSIS OF DEXTRAN IN AN ENZYME MEMBRANE REACTOR	10.1111/j.1365-2621.2002.tb08720.x

CONTINUOUS PRODUCTION OF PECTIC OLIGOSACCHARIDES IN AN ENZYME MEMBRANE REACTOR	10.1111/j.1365-2621.2001.tb08220.x
CONTINUOUS PRODUCTION OF PROTEIN HYDROLYSATES IN IMMOBILIZED ENZYME REACTORS	10.1002/abio.370070307
CONTRIBUTION OF M-CELLS AND OTHER EXPERIMENTAL VARIABLES IN THE TRANSLOCATION OF TIO2 NANOPARTICLES ACROSS IN VITRO INTESTINAL MODELS	10.1016/j.impact.2016.12.005
CONTROLLING LIPID DIGESTIBILITY: RESPONSE OF LIPID DROPLETS COATED BY Œí-LACTOGLOBULIN-DEXTRAN MAILLARD CONJUGATES TO SIMULATED GASTROINTESTINAL CONDITIONS	10.1016/j.foodhyd.2011.05.011
CORRECTION TO: DYNAMIC LINEAR MODELS GUIDE DESIGN AND ANALYSIS OF MICROBIOTA STUDIES WITHIN ARTIFICIAL HUMAN GUTS (MICROBIOME (2018) 6 (202) DOI: 10.1186/S40168-018-0584-3)	10.1186/s40168-018-0601-6
CORRECTION TO: HUMAN MICROBIOTA MODULATION VIA QSEC SENSOR KINASE MEDIATED IN THE ESCHERICHIA COLI O104:H4 OUTBREAK STRAIN INFECTION IN MICROBIOME MODEL (BMC MICROBIOLOGY, (2021), 21, 1, (163), 10.1186/S12866-021-02220-3)	10.1186/s12866-021-02266-3
COUPLING GROWTH KINETICS MODELING WITH MACHINE LEARNING REVEALS MICROBIAL IMMIGRATION IMPACTS AND IDENTIFIES KEY ENVIRONMENTAL PARAMETERS IN A BIOLOGICAL WASTEWATER TREATMENT PROCESS	10.1186/s40168-019-0682-x
CROSS-FEEDING BETWEEN BIFIDOBACTERIUM INFANTIS AND ANAEROSTIPES CACCAE ON LACTOSE AND HUMAN MILK OLIGOSACCHARIDES	10.3920/BM2020.0005
CROSS-LINKING TREATMENT OF ARABINOXYLAN IMPROVES ITS ANTIOXIDANT AND HYPOGLYCEMIC ACTIVITIES AFTER SIMULATED IN VITRO DIGESTION	10.1016/j.lwt.2021.111386
CULTIVATION OF STABLE, REPRODUCIBLE MICROBIAL COMMUNITIES FROM DIFFERENT FECAL DONORS USING MINIBIOREACTOR ARRAYS (MBRAS)	10.1186/s40168-015-0106-5
DECARBONIZATION AND DENITRIFICATION CHARACTERISTICS OF A COUPLING ABR-MFC-MEC PROCESS TREATING BLACK WATER	10.5004/dwt.2018.22797
DECHLORANE PLUS EXPOSURE ON GUT MICROBIOME EVALUATED BY USING BOTH IN VIVO AND IN VITRO ASSAYS	10.1016/j.ibiod.2021.105255
DECIPHERING THE COLONIC FERMENTATION CHARACTERISTICS OF AGAVIN AND DIGESTION-RESISTANT MALTODEXTRIN IN A SIMULATED BATCH FERMENTATION SYSTEM	10.1016/j.ijbiomac.2021.08.063
DECREASED COLONIZATION OF FECAL CLOSTRIDIUM COCCOIDES/EUBACTERIUM RECTALE SPECIES FROM ULCERATIVE COLITIS PATIENTS IN AN IN VITRO DYNAMIC GUT MODEL WITH MUCIN ENVIRONMENT	10.1111/j.1574-6941.2011.01252.x
DEGRADATION OF FIBRES FROM FRUIT BY-PRODUCTS ALLOWS SELECTIVE MODULATION OF THE GUT BACTERIA IN AN IN VITRO MODEL OF THE PROXIMAL COLON	10.1016/j.jff.2019.04.026
DEGRADATION OF PHYTATE BY HIGH-PHYTASE SACCHAROMYCES CEREVISIAE STRAINS DURING SIMULATED GASTROINTESTINAL DIGESTION	10.1021/jf0478399
DEGRADATION OF POLYSACCHARIDES FROM SARGASSUM FUSIFORME USING UV/H2O2 AND ITS EFFECTS ON STRUCTURAL CHARACTERISTICS	10.1016/j.carbpol.2019.115647
DEGRADATION OF SIALOGLYCOPROTEIN FROM CARASSIUS AURATUS EGGS BY SIMULATED GASTROINTESTINAL TRACT IN VITRO	10.7506/spkx1002-6630-20191009-053
DEGRADATIVE ACTIVITIES OF GUT ANAEROBES STUDIED IN A THREE-STAGE CONTINUOUS CULTURE MODEL OF THE COLON	10.1093/clinids/16.Supplement_4.S420
DELIVERY OF PHENOLIC COMPOUNDS, PEPTIDES AND Œí-GLUCAN TO THE GASTROINTESTINAL TRACT BY INCORPORATING DIETARY FIBRE-RICH MUSHROOMS INTO SORGHUM BISCUITS	10.3390/foods10081812
DEPHOSPHORYLATION OF MYO-INOSITOL PHOSPHATES IN THE IN VITRO INTESTINAL CACO-2 CELL MODEL	10.1080/09637486.2017.1330404
DESIGN AND INVESTIGATION OF POLYFERMS IN VITRO CONTINUOUS FERMENTATION MODELS INOCULATED WITH IMMOBILIZED FECAL MICROBIOTA MIMICKING THE ELDERLY COLON	10.1371/journal.pone.0142793
DESIGN OF A NOVEL GUT BACTERIAL ADHESION MODEL FOR PROBIOTIC APPLICATIONS	10.3109/10731199.2012.712047
DESIGNING A FUNCTIONAL RICE MUFFIN FORMULATED WITH PREBIOTIC OLIGOSACCHARIDES AND SUGAR REDUCTION	10.1016/j.fbio.2020.100858

DESIGNING AN IN-VITRO GAS PROFILING SYSTEM FOR HUMAN FAECAL SAMPLES	10.1016/j.snb.2016.07.120
DETERMINATION OF THE YIELD, SAPONIN CONTENT AND PROFILE, ANTIMICROBIAL AND ANTIOXIDANT ACTIVITIES OF THREE GYPSOPHILA SPECIES	10.1016/j.indcrop.2019.05.071
DETRIMENTAL EFFECT ON THE GUT MICROBIOTA OF 1,2-DICARBONYL COMPOUNDS AFTER IN VITRO GASTRO-INTESTINAL AND FERMENTATIVE DIGESTION	10.1016/j.foodchem.2020.128237
DEVELOPMENT AND EVALUATION OF A NOVEL NANOFIBERSOLOSOME FOR ENHANCING THE STABILITY, IN VITRO BIOACCESSIBILITY, AND COLONIC DELIVERY OF CYANIDIN-3-O-GLUCOSIDE	10.1016/j.foodres.2021.110712
DEVELOPMENT AND FUNCTIONAL CHARACTERIZATION OF NEW ANTIOXIDANT DIETARY FIBERS FROM POMEGRANATE, OLIVE AND ARTICHOKE BY-PRODUCTS	10.1016/j.foodres.2017.09.001
DEVELOPMENT AND VALIDATION OF A CHEMOSTAT GUT MODEL TO STUDY BOTH PLANKTONIC AND BIOFILM MODES OF GROWTH OF CLOSTRIDIUM DIFFICILE AND HUMAN MICROBIOTA	10.1371/journal.pone.0088396
DEVELOPMENT AND VALIDATION OF A CONTINUOUS IN VITRO SYSTEM REPRODUCING SOME BIOTIC AND ABIOTIC FACTORS OF THE VEAL CALF INTESTINE	10.1128/AEM.00524-10
DEVELOPMENT AND VALIDATION OF A NEW ARTIFICIAL GASTRIC DIGESTIVE SYSTEM	10.1016/j.foodres.2019.04.015
DEVELOPMENT AND VALIDATION OF A NEW DYNAMIC COMPUTER-CONTROLLED MODEL OF THE HUMAN STOMACH AND SMALL INTESTINE	10.1002/bit.25890
DEVELOPMENT AND VALIDATION OF THE SIMULATOR OF THE CANINE INTESTINAL MICROBIAL ECOSYSTEM (SCIME)	10.1093/jas/skz357
DEVELOPMENT OF A 5-STEP MULTI-CHAMBER REACTOR AS A SIMULATION OF THE HUMAN INTESTINAL MICROBIAL ECOSYSTEM	10.1007/BF00228615
DEVELOPMENT OF A HOST-MICROBIOME MODEL OF THE SMALL INTESTINE	10.1096/fj.201801414R
DEVELOPMENT OF A HUMAN GASTRIC DIGESTION SIMULATOR EQUIPPED WITH PERISTALSIS FUNCTION FOR THE DIRECT OBSERVATION AND ANALYSIS OF THE FOOD DIGESTION PROCESS	10.3136/fstr.20.225
DEVELOPMENT OF A SEMI-DYNAMIC IN VITRO MODEL AND ITS TESTING USING PROBIOTIC BACILLUS COAGULANS GBI-30, 6086 IN ORANGE JUICE AND YOGURT	10.1016/j.mimet.2021.106187
DEVELOPMENT OF A SIMPLE MODEL DEVICE FOR IN VITRO GASTRIC DIGESTION INVESTIGATION	10.1039/c0fo00159g
DEVELOPMENT OF A SIX-STAGE CULTURE SYSTEM FOR SIMULATING THE GASTROINTESTINAL MICROBIOTA OF WEANED INFANTS	10.1080/089106001300136183
DEVELOPMENT OF A STANDARDIZED FOOD MODEL FOR STUDYING THE IMPACT OF FOOD MATRIX EFFECTS ON THE GASTROINTESTINAL FATE AND TOXICITY OF INGESTED NANOMATERIALS	10.1016/j.impact.2018.11.002
DEVELOPMENT OF AN ANALYTICAL METHOD TO DETECT SHORT-CHAIN FATTY ACIDS BY SPME-GC,ÄìMS IN SAMPLES COMING FROM AN IN VITRO GASTROINTESTINAL MODEL	10.1016/j.jchromb.2019.06.013
DEVELOPMENT OF AN IN VITRO CO-CULTURE MODEL TO MIMIC THE HUMAN INTESTINE IN HEALTHY AND DISEASED STATE	10.1016/j.tiv.2017.08.011
DEVELOPMENT OF AN IN VITRO CONTINUOUS FLOW CULTURE MODEL OF THE MURINE INTESTINAL TRACT	10.1002/(SICI)1234-987X(199605)9:3<97::AID-MEH414>3.3.CO;2-Z
DEVELOPMENT OF AN IN VITRO DIGESTION METHOD TO ASSESS CAROTENOID BIOAVAILABILITY FROM MEALS	10.1021/jf9903298
DEVELOPMENT OF AN IN VITRO DIGESTION MODEL FOR ESTIMATING THE BIOACCESSIBILITY OF SOIL CONTAMINANTS	10.1007/s00244-002-1278-0
DEVELOPMENT OF AN IN VITRO SYSTEM SIMULATING BUCCO-GASTRIC DIGESTION TO ASSESS THE PHYSICAL AND CHEMICAL CHANGES OF FOOD	10.1080/0963748021000044732
DEVELOPMENT OF HUMAN COLONIC MICROBIOTA IN THE COMPUTER-CONTROLLED DYNAMIC SIMULATOR OF THE GASTROINTESTINAL TRACT SIMGI	10.1016/j.lwt.2014.12.014
DEVELOPMENT, VALIDATION AND IMPLEMENTATION OF AN IN VITRO MODEL FOR THE STUDY OF METABOLIC AND IMMUNE FUNCTION IN NORMAL AND INFLAMED HUMAN COLONIC EPITHELIUM	

DIET ALTERS ENTERO-MAMMARY SIGNALING TO REGULATE THE BREAST MICROBIOME AND TUMORIGENESIS	10.1158/0008-5472.CAN-20-2983
DIET DRIVES QUICK CHANGES IN THE METABOLIC ACTIVITY AND COMPOSITION OF HUMAN GUT MICROBIOTA IN A VALIDATED IN VITRO GUT MODEL	10.1016/j.resmic.2015.09.006
DIETARY EMULSIFIERS ALTER COMPOSITION AND ACTIVITY OF THE HUMAN GUT MICROBIOTA IN VITRO, IRRESPECTIVE OF CHEMICAL OR NATURAL EMULSIFIER ORIGIN	10.3389/fmicb.2020.577474
DIETARY EMULSIFIERS DIRECTLY ALTER HUMAN MICROBIOTA COMPOSITION AND GENE EXPRESSION EX VIVO POTENTIATING INTESTINAL INFLAMMATION	10.1136/gutjnl-2016-313099
DIETARY EXPOSURES TO COMMON EMULSIFIERS AND THEIR IMPACT ON THE GUT MICROBIOTA: IS THERE A CAUSE FOR CONCERN?	10.1111/1541-4337.12410
DIETARY LIPIDS INFLUENCE BIOACCESSIBILITY OF POLYPHENOLS FROM BLACK CARROTS AND AFFECT MICROBIAL DIVERSITY UNDER SIMULATED GASTROINTESTINAL DIGESTION	10.3390/antiox9080762
DIETARY SUPPLEMENT BASED ON STILBENES: A FOCUS ON GUT MICROBIAL METABOLISM BY THE: IN VITRO SIMULATOR M-SHIME®	10.1039/c6fo00784h
DIFFERENCES IN SURVIVAL AMONG 13 LISTERIA MONOCYTOGENES STRAINS IN A DYNAMIC MODEL OF THE STOMACH AND SMALL INTESTINE	10.1128/AEM.00319-08
DIFFERENCES IN THE GUT MICROBIOTA BETWEEN YOUNG AND ELDERLY PERSONS IN KOREA	10.1016/j.nutres.2020.12.013
DIFFERENT HUMAN GUT MODELS REVEAL THE DISTINCT FERMENTATION PATTERNS OF ARABINOXYLAN VERSUS INULIN	10.1021/jf4021784
DIFFERENT OAT INGREDIENTS STIMULATE SPECIFIC MICROBIAL METABOLITES IN THE GUT MICROBIOME OF THREE HUMAN INDIVIDUALS IN VITRO	10.1021/acsomega.8b01360
DIFFERENTIAL FERMENTATION OF RAW AND PROCESSED HIGH-AMYLOSE AND WAXY MAIZE STARCHES IN THE SIMULATOR OF THE HUMAN INTESTINAL MICROBIAL ECOSYSTEM (SHIME®)	10.1016/j.jff.2021.104735
DIFFERENTLY PRE-TREATED RAPESEED MEALS AFFECT IN VITRO SWINE GUT MICROBIOTA COMPOSITION	10.3389/fmicb.2020.570985
DIGESTED AND FERMENTED GREEN KIWIFRUIT INCREASES HUMAN ŒÍ-DEFENSIN 1 AND 2 PRODUCTION IN VITRO	10.1007/s11130-012-0305-1
DIGESTIBILITY AND PREBIOTIC PROPERTIES OF POTATO RHAMNOGALACTURONAN I POLYSACCHARIDE AND ITS GALACTOSE-RICH OLIGOSACCHARIDES/OLIGOMERS	10.1016/j.carbpol.2015.09.106
DIGESTIBILITY OF TRANSGLUTAMINASE CROSS-LINKED CASEINATE VERSUS NATIVE CASEINATE IN AN IN VITRO MULTICOMPARTMENTAL MODEL SIMULATING YOUNG CHILD AND ADULT GASTROINTESTINAL CONDITIONS	10.1021/jf402824u
DIGESTIBILITY, BIOACTIVITY AND PREBIOTIC POTENTIAL OF PHENOLICS RELEASED FROM WHOLE GOLD KIWIFRUIT AND POMACE BY: IN VITRO GASTROINTESTINAL DIGESTION AND COLONIC FERMENTATION	10.1039/d0fo02399j
DIGESTION AND COLONIC FERMENTATION OF RAW AND COOKED OPUNTIA FICUS-INDICA CLADODES IMPACTS BIOACCESSIBILITY AND BIOACTIVITY	10.1021/acs.jafc.8b06480
DIGESTION OF PECTIC POLYSACCHARIDE FROM BRASSICA RAPA L. IN VITRO AND ITS EFFECT ON THE INTESTINAL MICROBIOTA IN CYCLOPHOSPHAMIDE-TREATED MICE	10.1111/ijfs.15278
DIGESTION OF STARCH IN A DYNAMIC SMALL INTESTINAL MODEL	10.1007/s00394-015-1044-5
DIGESTION-ON-A-CHIP: A CONTINUOUS-FLOW MODULAR MICROSYSTEM RECREATING ENZYMATIC DIGESTION IN THE GASTROINTESTINAL TRACT	10.1039/c8lc01080c
DIGESTIVE PROPERTIES OF HALF-FIN ANCHOVY HYDROLYSATES/GLUCOSE MAILLARD REACTION PRODUCTS AND MODULATION EFFECTS ON INTESTINAL MICROBIOTA	10.1002/jsfa.11600
DIRECT MEDIUM-CHAIN CARBOXYLIC ACID OIL SEPARATION FROM A BIOREACTOR BY AN ELECTRODIALYSIS/PHASE SEPARATION CELL	10.1021/acs.est.0c04939
DISSEMINATION OF MULTIPLE CARBAPENEM RESISTANCE GENES IN AN IN VITRO GUT MODEL SIMULATING THE HUMAN COLON	10.1093/jac/dkz106

DISTURBANCE OPENS RECRUITMENT SITES FOR BACTERIAL COLONIZATION IN ACTIVATED SLUDGE	10.1111/1462-2920.12824
DIVERSE PROFILE OF FERMENTATION BYPRODUCTS FROM THIN STILLAGE	10.3389/fbioe.2021.695306
DNA ADDUCT PROFILING OF IN VITRO COLONIC MEAT DIGESTS TO MAP RED VS. WHITE MEAT GENOTOXICITY	10.1016/j.fct.2018.02.032
DO PH AND TEMPERATURE PLAY A ROLE IN GASTROSTOMY TUBE DETERIORATION?	10.1177/0148607105029005388
DOSE-DEPENDENT ALTERATIONS TO IN VITRO HUMAN MICROBIOTA COMPOSITION AND BUTYRATE INHIBITION BY A SUPERCRITICAL CARBON DIOXIDE HOPS EXTRACT	10.3390/biom9090390
DOSE-DEPENDENT PREBIOTIC EFFECT OF LACTULOSE IN A COMPUTER-CONTROLLED IN VITRO MODEL OF THE HUMAN LARGE INTESTINE	10.3390/nu9070767
DRIVERS OF HUMAN GUT MICROBIAL COMMUNITY ASSEMBLY: COADAPTATION, DETERMINISM AND STOCHASTICITY	10.1038/s41396-019-0498-5
DRUG METABOLOME OF THE SIMVASTATIN FORMED BY HUMAN INTESTINAL MICROBIOTA IN VITRO	10.1039/c0mb00023j
DYNAMIC CHANGES IN THE INTESTINAL MICROBIAL COMMUNITY OF TWO TIME-AGED SOILS UNDER COMBINED CADMIUM AND CIPROFLOXACIN CONTAMINATED CONDITIONS	10.1016/j.scitotenv.2021.150558
DYNAMIC CHANGES OF STRUCTURAL CHARACTERISTICS OF SNOW CHRYSANTHEMUM POLYSACCHARIDES DURING IN VITRO DIGESTION AND FECAL FERMENTATION AND RELATED IMPACTS ON GUT MICROBIOTA	10.1016/j.foodres.2020.109888
DYNAMIC DIGESTION OF TAMARIND SEED POLYSACCHARIDE: INDIGESTIBILITY IN GASTROINTESTINAL SIMULATIONS AND GUT MICROBIOTA CHANGES IN VITRO	10.1016/j.carbpol.2020.116194
DYNAMIC IN VITRO MODELS OF THE HUMAN GASTROINTESTINAL TRACT AS RELEVANT TOOLS TO ASSESS THE SURVIVAL OF PROBIOTIC STRAINS AND THEIR INTERACTIONS WITH GUT MICROBIOTA	10.3390/microorganisms3040725
DYNAMIC LINEAR MODELS GUIDE DESIGN AND ANALYSIS OF MICROBIOTA STUDIES WITHIN ARTIFICIAL HUMAN GUTS	10.1186/s40168-018-0584-3
DYNAMIC PROCESSES PERMITTING STABLE COEXISTENCE OF ANTIMICROBIAL RESISTANT AND NON-RESISTANT ORGANISMS IN A GASTROINTESTINAL TRACT MODEL	10.2306/scienceasia1513-1874.2007.33.197
ECO-FRIENDLY REMEDIATION AND REUSE FOR COASTAL DREDGED MATERIALS USING A BIOAUGMENTATION TECHNOLOGY	10.7845/kjm.2015.5066
ECOGENOMICS REVEALS MICROBIAL METABOLIC NETWORKS IN A PSYCHROPHILIC METHANOGENIC BIOREACTOR TREATING SOY SAUCE PRODUCTION WASTEWATER	10.1264/jsme2.ME21045
ECOLOGICAL ADAPTATION AND SUCCESSION OF HUMAN FECAL MICROBIAL COMMUNITIES IN AN AUTOMATED IN VITRO FERMENTATION SYSTEM	10.1128/mSystems.00232-21
ECOLOGICAL AND PHYSIOLOGICAL STUDIES ON LARGE INTESTINAL BACTERIA IN RELATION TO PRODUCTION OF HYDROLYTIC AND REDUCTIVE ENZYMES INVOLVED IN FORMATION OF GENOTOXIC METABOLITES	10.1099/00222615-47-5-407
ECOLOGICAL STABILITY PROPERTIES OF MICROBIAL COMMUNITIES ASSESSED BY FLOW CYTOMETRY	10.1128/mSphere.00564-17
EDIBLE LECITHIN, STEARIC ACID, AND WHEY PROTEIN BIGELS ENHANCE SURVIVAL OF PROBIOTICS DURING IN VITRO DIGESTION	10.1016/j.fbio.2020.100813
EFFECT OF A CHINESE MEDICAL NUTRITION THERAPY DIET ON GUT MICROBIOTA AND SHORT CHAIN FATTY ACIDS IN THE SIMULATOR OF THE HUMAN INTESTINAL MICROBIAL ECOSYSTEM (SHIME)	10.1016/j.jff.2019.103555
EFFECT OF A NEW PROBIOTIC SACCHAROMYCES CEREVISIAE STRAIN ON SURVIVAL OF ESCHERICHIA COLI O157:H7 IN A DYNAMIC GASTROINTESTINAL MODEL	10.1128/AEM.02130-10
EFFECT OF A SYNBIOTIC ON MICROBIAL COMMUNITY STRUCTURE IN A CONTINUOUS CULTURE MODEL OF THE GASTRIC MICROBIOTA IN ENTERAL NUTRITION PATIENTS	10.1111/j.1574-6941.2011.01279.x
EFFECT OF ACID ADAPTATION TREATMENT ON THE SURVIVAL OF VIBRIO PARAHAEMOLYTICUS IN OYSTER HOMOGENATES UNDER HEAT, COLD AND SIMULATED GASTROINTESTINAL CONDITIONS	

EFFECT OF ADDING RESISTANT MALTODEXTRIN TO PASTEURIZED ORANGE JUICE ON BIOACTIVE COMPOUNDS AND THEIR BIOACCESSIBILITY	10.3390/foods10061198
EFFECT OF ALGINATE AND CHITOSAN ON VIABILITY AND RELEASE BEHAVIOR OF BIFIDOBACTERIUM PSEUDOCATENULATUM G4 IN SIMULATED GASTROINTESTINAL FLUID	10.1016/j.carbpol.2014.05.014
EFFECT OF BEAN STRUCTURE ON MICROBIOTA UTILIZATION OF PLANT NUTRIENTS: AN IN-VITRO STUDY USING THE SIMULATOR OF THE HUMAN INTESTINAL MICROBIAL ECOSYSTEM (SHIME®)	10.1016/j.jff.2020.104087
EFFECT OF BIFIDOBACTERIUM CRUDILACTIS AND 3,Ä -SIALYLLACTOSE ON THE TODDLER MICROBIOTA USING THE SHIME® MODEL	10.1016/j.foodres.2020.109755
EFFECT OF BIOPROCESSING OF WHEAT BRAN IN WHOLEMEAL WHEAT BREADS ON THE COLONIC SCFA PRODUCTION IN VITRO AND POSTPRANDIAL PLASMA CONCENTRATIONS IN MEN	10.1016/j.foodchem.2011.03.043
EFFECT OF BIOPROCESSING ON THE IN VITRO COLONIC MICROBIAL METABOLISM OF PHENOLIC ACIDS FROM RYE BRAN FORTIFIED BREADS	10.1021/acs.jafc.6b05110
EFFECT OF BROAD- AND NARROW-SPECTRUM ANTIMICROBIALS ON CLOSTRIDIUM DIFFICILE AND MICROBIAL DIVERSITY IN A MODEL OF THE DISTAL COLON	10.1073/pnas.1001224107
EFFECT OF DIETARY FIBER ON THE BIOACCESSIBILITY OF PHENOLIC COMPOUNDS OF MANGO, PAPAYA AND PINEAPPLE FRUITS BY AN IN VITRO DIGESTION MODEL	10.1590/1678-457X.6729
EFFECT OF DIETARY NUCLEOSIDES AND YEAST EXTRACTS ON COMPOSITION AND METABOLIC ACTIVITY OF INFANT GUT MICROBIOTA IN POLYFERMS COLONIC FERMENTATION MODELS	10.1093/femsec/fix088
EFFECT OF DIFFERENT MATRICES ON PROBIOTIC RESISTANCE TO IN¬†VITRO SIMULATED GASTROINTESTINAL CONDITIONS	10.1111/1471-0307.12215
EFFECT OF DIFFERENT SOLUBLE DIETARY FIBRES ON THE PHENOLIC PROFILE OF BLACKBERRY PUREE SUBJECTED TO IN VITRO GASTROINTESTINAL DIGESTION AND LARGE INTESTINE FERMENTATION	10.1016/j.foodres.2019.108954
EFFECT OF DIFFERENT TYPES OF ENCAPSULATION ON THE SURVIVAL OF LACTOBACILLUS PLANTARUM DURING STORAGE WITH INULIN AND IN VITRO DIGESTION	10.1016/j.lwt.2015.06.049
EFFECT OF EXTRUSION COOKING AND SIMULATED IN VITRO GASTROINTESTINAL DIGESTION ON CONDENSED TANNINS AND RADICAL SCAVENGING ACTIVITY OF TYPE II AND TYPE III WHOLE GRAIN SORGHUM	10.1111/ijfs.13510
EFFECT OF FERMENTATION PH ON PROTEIN BIOACCESSIBILITY OF SOYMILK CURD WITH ADDED TEA POLYPHENOLS AS ASSESSED BY IN VITRO GASTROINTESTINAL DIGESTION	10.1021/acs.jafc.7b04456
EFFECT OF FERMENTED SAUSAGES WITH PROBIOTIC ENTEROCOCCUS FAECIUM CRL 183 ON GUT MICROBIOTA USING DYNAMIC COLONIC MODEL	10.1016/j.lwt.2020.109876
EFFECT OF FOOD THERMAL PROCESSING ON THE COMPOSITION OF THE GUT MICROBIOTA	10.1021/acs.jafc.8b04077
EFFECT OF FREEZE DRYING AND SIMULATED GASTROINTESTINAL DIGESTION ON PHENOLIC METABOLITES AND ANTIOXIDANT PROPERTY OF THE NATAL PLUM (CARISSA MACROCARPA)	10.3390/foods10061420
EFFECT OF FRUCTO-OLIGOSACCHARIDES AND TRANSGALACTO-OLIGOSACCHARIDES ON MICROBIAL POPULATIONS AND MICROBIAL ACTIVITY IN THE GASTROINTESTINAL TRACT OF PIGLETS POST-WEANING	10.1016/j.anifeedsci.2004.07.015
EFFECT OF GALACTOOLIGOSACCHARIDES AND BIFIDOBACTERIUM ANIMALIS BB-12 ON GROWTH OF LACTOBACILLUS AMYLOVORUS DSM 16698, MICROBIAL COMMUNITY STRUCTURE, AND METABOLITE PRODUCTION IN AN IN VITRO COLONIC MODEL SET UP WITH HUMAN OR PIG MICROBIOTA	10.1111/1574-6941.12041
EFFECT OF GLYCATION DEGREE ON THE STRUCTURE AND DIGESTION PROPERTIES OF OVALBUMIN: A STUDY OF AMINO ACIDS AND PEPTIDES RELEASE AFTER IN VITRO GASTROINTESTINAL SIMULATED DIGESTION	10.1016/j.foodchem.2021.131331
EFFECT OF GLYCATION OF BOVINE B-LACTOGLOBULIN WITH GALACTOOLIGOSACCHARIDES ON THE GROWTH OF HUMAN FAECAL BACTERIA	10.1016/j.idairyj.2011.06.002
EFFECT OF GUT MICROBIOTA ON IN VITRO BIOACCESSIBILITY OF HEAVY METALS AND HUMAN HEALTH RISK ASSESSMENT FROM INGESTION OF CONTAMINATED SOILS	10.1016/j.envpol.2021.116943

EFFECT OF HIGH HYDROSTATIC PRESSURE ON THE EXTRACTABILITY AND BIOACCESSIBILITY OF CAROTENOIDS AND THEIR ESTERS FROM PAPAYA (CARICA PAPAYA L.) AND ITS IMPACT ON TISSUE MICROSTRUCTURE	10.3390/foods10102435
EFFECT OF IN VITRO DIGESTION GASTROINTESTINAL OF THE EXTRACT AQUEOU OF LEAVES OF UGNI MOLINAE, ON THE VIABILITY OF COLORECTAL CANCER CELLS	10.4067/S0717-97072021000305268
EFFECT OF IN VITRO DIGESTION ON PHENOLICS AND ANTIOXIDANT ACTIVITY OF RED AND YELLOW COLORED PEA HULLS	10.1016/j.foodchem.2020.127606
EFFECT OF IN VITRO DIGESTION ON THE BIOACCESSIBILITY AND BIOACTIVITY OF PHENOLIC COMPOUNDS IN FRACTIONS OF EUGENIA PYRIFORMIS FRUIT	10.1016/j.foodres.2021.110767
EFFECT OF IN VITRO DIGESTION-FERMENTATION OF CA(II)-ALGINATE BEADS CONTAINING SUGAR AND BIOPOLYMERS OVER GLOBAL ANTIOXIDANT RESPONSE AND SHORT CHAIN FATTY ACIDS PRODUCTION	10.1016/j.foodchem.2020.127483
EFFECT OF IN VITRO DIGESTION-FERMENTATION ON GREEN AND ROASTED COFFEE BIOACTIVITY: THE ROLE OF THE GUT MICROBIOTA	10.1016/j.foodchem.2018.11.137
EFFECT OF IN VITRO GASTROINTESTINAL DIGESTION ON BIOACCESSIBILITY OF PHENOLIC COMPOUNDS AND ANTIOXIDANT CAPACITY OF CRUSTACEANS RESIDUES WITH POTENTIAL ANTIDIABETIC IMPACT	10.1016/j.lwt.2020.110004
EFFECT OF IN VITRO GASTROINTESTINAL DIGESTION ON PHENOLIC COMPOUNDS AND ANTIOXIDANT PROPERTIES OF SOLUBLE AND INSOLUBLE DIETARY FIBERS DERIVED FROM HULLESS BARLEY	10.1111/1750-3841.15592
EFFECT OF IN VITRO GASTROINTESTINAL DIGESTION ON THE BIOAVAILABILITY OF PHENOLIC COMPONENTS AND THE ANTIOXIDANT POTENTIALS OF SOME TURKISH FRUIT WINES	10.1016/j.foodres.2015.10.009
EFFECT OF IRON ADDITION ON THE PERFORMANCE OF ANOXIC-OXIC MEMBRANE PROCESS AND BIOLOGICAL PHOSPHORUS REMOVAL	10.19675/j.cnki.1006-687x.2017.07017
EFFECT OF LACTIC FERMENTATION ON SOY PROTEIN DIGESTIVE PATTERN ASSESSED BY AN IN VITRO DYNAMIC GASTROINTESTINAL DIGESTION MODEL AND THE INFLUENCE ON HUMAN FAECAL MICROBIOTA	10.1002/jsfa.10694
EFFECT OF LACTOBACILLI ON THE ECOLOGY OF THE GASTRO-INTESTINAL MICROBIOTA CULTURED IN THE SHIME REACTOR	10.3109/08910609609166446
EFFECT OF MANNOPROTEINS ON THE GROWTH, GASTROINTESTINAL VIABILITY, AND ADHERENCE TO CACO-2 CELLS OF LACTIC ACID BACTERIA	10.1111/j.1750-3841.2011.02602.x
EFFECT OF METRONIDAZOLE ON GROWTH AND TOXIN PRODUCTION BY EPIDEMIC CLOSTRIDIUM DIFFICILE PCR RIBOTYPES 001 AND 027 IN A HUMAN GUT MODEL	10.1093/jac/dkm113
EFFECT OF MICROBIAL COMMUNITY STRUCTURE ON ORGANIC REMOVAL AND BIOFOULING IN MEMBRANE ADSORPTION BIOREACTOR USED IN SEAWATER PRETREATMENT	10.1016/j.cej.2016.02.108
EFFECT OF MICROBIAL TRANSGLUTAMINASE CROSS-LINKING ON THE QUALITY CHARACTERISTICS AND POTENTIAL ALLERGENICITY OF TOFU	10.1039/c9fo01118h
EFFECT OF MICROENCAPSULATION ON SURVIVAL OF LACTOBACILLUS PLANTARUM IN SIMULATED GASTROINTESTINAL CONDITIONS, REFRIGERATION, AND YOGURT	10.1016/j.jfoodeng.2010.10.006
EFFECT OF Œí-GLUCAN AND BLACK TEA IN A FUNCTIONAL BREAD ON SHORT CHAIN FATTY ACID PRODUCTION BY THE GUT MICROBIOTA IN A GUT DIGESTION/FERMENTATION MODEL	10.3390/ijerph16020227
EFFECT OF OXYGEN CONTAMINATION ON PROPIONATE AND CAPROATE FORMATION IN ANAEROBIC FERMENTATION	10.3389/fbioe.2021.725443
EFFECT OF PECTIN ADDITION ON THE BIOACCESSIBILITY OF FOUR BAMBOO LEAF FLAVONOIDS DURING SIMULATED IN VITRO GASTROINTESTINAL DIGESTION	10.7506/spkx1002-6630-20190527-321
EFFECT OF PERESKIA ACULEATA MILL. IN VITRO AND IN OVERWEIGHT HUMANS: A RANDOMIZED CONTROLLED TRIAL	10.1111/jfbc.12903
EFFECT OF PH ON AN IN VITRO MODEL OF GASTRIC MICROBIOTA IN ENTERAL NUTRITION PATIENTS	10.1128/AEM.71.8.4777-4783.2005
EFFECT OF PROBIOTIC, PREBIOTIC, AND SYNBIOTIC ON THE GUT MICROBIOTA OF AUTISTIC CHILDREN USING AN IN VITRO GUT MICROBIOME MODEL	10.1016/j.foodres.2021.110657

EFFECT OF PROCESS ON PHYTOCHEMICALS AND THE FUNCTION OF IMPROVING FECAL MICROFLORA OF SEABUCKTHORN LEAF TEA	
EFFECT OF PROCESSING PROCEDURES ON IN VITRO DIGESTIBILITY AND COLONIC FERMENTATION OF RICEBERRY RICE	10.15414/JMBFS.2018-19.8.3.940-946
EFFECT OF PROPIONIBACTERIUM FREUDENREICHII ON RUMINAL FERMENTATION PATTERNS, METHANE PRODUCTION AND LIPID BIOHYDROGENATION OF BEEF FINISHING DIETS CONTAINING FLAXSEED OIL IN A RUMEN SIMULATION TECHNIQUE	10.4141/CJAS-2014-051
EFFECT OF PROTEIN CORONA MAGNETITE NANOPARTICLES DERIVED FROM BREAD IN VITRO DIGESTION ON CACO-2 CELLS MORPHOLOGY AND UPTAKE	10.1016/j.biocel.2015.10.019
EFFECT OF ROASTING CONDITIONS ON COCOA BIOACTIVITY AND GUT MICROBIOTA MODULATION	10.1039/d1fo01155c
EFFECT OF SHORT-CHAIN CARBOHYDRATES ON HUMAN INTESTINAL BIFIDOBACTERIA AND ESCHERICHIA COLI IN VITRO	10.1099/0022-1317-50-2-152
EFFECT OF SIMULATED GASTROINTESTINAL DIGESTION AND FERMENTATION ON POLYPHENOLIC CONTENT AND BIOACTIVITY OF BROWN SEAWEED PHLOROTANNIN-RICH EXTRACTS	10.1002/mnfr.201700223
EFFECT OF SIMULATED GASTROINTESTINAL DIGESTION IN VITRO ON THE ANTIOXIDANT ACTIVITY, MOLECULAR WEIGHT AND MICROSTRUCTURE OF POLYSACCHARIDES FROM A TROPICAL SEA CUCUMBER (HOLOTHURIA LEUCOSPILOTA)	10.1016/j.foodhyd.2018.11.040
EFFECT OF SIMULATED GASTROINTESTINAL DIGESTION ON PHENOLIC COMPOSITION AND ANTIOXIDANT CAPACITY OF COOKED COWPEA (VIGNA UNGUICULATA) VARIETIES	10.1111/ijfs.12260
EFFECT OF SOME TRADITIONAL SAUDI ARABIAN MEALS ON THE SURVIVAL OF PROBIOTIC BACTERIA IN FERMENTED MILK UNDER IN VITRO SIMULATED GASTROINTESTINAL CONDITIONS	10.3923/biotech.2015.260.266
EFFECT OF STEVIA REBAUDIANA ADDITION ON BIOACCESSIBILITY OF BIOACTIVE COMPOUNDS AND ANTIOXIDANT ACTIVITY OF BEVERAGES BASED ON EXOTIC FRUITS MIXED WITH OAT FOLLOWING SIMULATED HUMAN DIGESTION	10.1016/j.foodchem.2015.03.095
EFFECT OF THE ADDITION OF PEPTOSTREPTOCOCCUS PRODUCTUS ATCC35244 ON THE GASTRO-INTESTINAL MICROBIOTA AND ITS ACTIVITY, AS SIMULATED IN AN IN VITRO SIMULATOR OF THE HUMAN GASTRO-INTESTINAL TRACT	10.1007/s002530051022
EFFECT OF THE CARRIER MATERIAL, DRYING TECHNOLOGY AND DISSOLUTION MEDIA ON THE VIABILITY OF: LACTOBACILLUS FERMENTUM K73 DURING SIMULATED GASTROINTESTINAL TRANSIT	10.1039/c9fo01091b
EFFECT OF THE NOVEL POLYSACCHARIDE POLYGLYCOPLEX® ON SHORT-CHAIN FATTY ACID PRODUCTION IN A COMPUTER-CONTROLLED IN VITRO MODEL OF THE HUMAN LARGE INTESTINE	10.3390/nu6031115
EFFECT OF TULATHROMYCIN ON COLONIZATION RESISTANCE, ANTIMICROBIAL RESISTANCE, AND VIRULENCE OF HUMAN GUT MICROBIOTA IN CHEMOSTATS	10.3389/fmicb.2016.00477
EFFECT OF TYPE OF PROTEIN-BASED MICROCAPSULES AND STORAGE AT VARIOUS AMBIENT TEMPERATURES ON THE SURVIVAL AND HEAT TOLERANCE OF SPRAY DRIED LACTOBACILLUS ACIDOPHILUS	10.1111/1750-3841.13820
EFFECT OF ULTRASONIC FREQUENCY ON THE BACTERIAL COMMUNITY STRUCTURE DURING BIOFOULING FORMATION IN MICROFILTRATION MEMBRANE BIOREACTORS FOR WASTEWATER TREATMENT	10.1016/j.ibiod.2020.105102
EFFECTS OF ALTERNATIVE DIETARY SUBSTRATES ON COMPETITION BETWEEN HUMAN COLONIC BACTERIA IN AN ANAEROBIC FERMENTOR SYSTEM	10.1128/AEM.69.2.1136-1142.2003
EFFECTS OF ANTIBIOTIC PRETREATMENT OF AN ULCERATIVE COLITIS-DERIVED FECAL MICROBIAL COMMUNITY ON THE INTEGRATION OF THERAPEUTIC BACTERIA IN VITRO	10.1128/mSystems.00404-19
EFFECTS OF ANTIBIOTICS ON BACTERIAL SPECIES COMPOSITION AND METABOLIC ACTIVITIES IN CHEMOSTATS CONTAINING DEFINED POPULATIONS OF HUMAN GUT MICROORGANISMS	10.1128/AAC.00079-13
EFFECTS OF BIFIDOBACTERIA-PRODUCED EXOPOLYSACCHARIDES ON HUMAN GUT MICROBIOTA IN VITRO	10.1007/s00253-018-9572-6
EFFECTS OF CHICORY INULIN ON RUMINAL FERMENTATION IN VITRO	10.1501/vetfak_0000002212

EFFECTS OF COLON-TARGETED VITAMINS ON THE COMPOSITION AND METABOLIC ACTIVITY OF THE HUMAN GUT MICROBIOME- A PILOT STUDY	10.1080/19490976.2021.1875774
EFFECTS OF COMMERCIAL APPLE VARIETIES ON HUMAN GUT MICROBIOTA COMPOSITION AND METABOLIC OUTPUT USING AN IN VITRO COLONIC MODEL	10.3390/nu9060533
EFFECTS OF CRUDE SPHALLEROCARPUS GRACILIS POLYSACCHARIDES AS POTENTIAL PREBIOTICS ON ACIDIFYING ACTIVITY AND GROWTH OF PROBIOTICS IN FERMENTED MILK	10.1016/j.lwt.2021.111882
EFFECTS OF DEFINED GUT MICROBIAL ECOSYSTEM COMPONENTS ON VIRULENCE DETERMINANTS OF CLOSTRIDIOIDES DIFFICILE	10.1038/s41598-018-37547-x
EFFECTS OF DIET, HABITAT, AND PHYLOGENY ON THE FECAL MICROBIOME OF WILD AFRICAN SAVANNA (LOXODONTA AFRICANA) AND FOREST ELEPHANTS (L.CYCLOTIS)	10.1002/ece3.6305
EFFECTS OF DIFFERENT PREBIOTICS ON VIABILITY UNDER IN VITRO GASTROINTESTINAL CONDITIONS AND SENSORY PROPERTIES OF FERMENTED MILK	
EFFECTS OF DIFFERENT SATIETY LEVELS ON THE FATE OF SOYMILK PROTEIN IN GASTROINTESTINAL DIGESTION AND ANTIGENICITY ASSESSED BY AN IN VITRO DYNAMIC GASTROINTESTINAL MODEL	10.1039/c9fo01965k
EFFECTS OF DIGESTED CHEONGGUKJANG ON HUMAN MICROBIOTA ASSESSED BY IN VITRO FECAL FERMENTATION	10.1007/s12275-021-0525-x
EFFECTS OF DIGESTED FLOURS FROM FOUR DIFFERENT SWEET POTATO (IPOMOEA BATATAS L.) ROOT VARIETIES ON THE COMPOSITION AND METABOLIC ACTIVITY OF HUMAN COLONIC MICROBIOTA IN VITRO	10.1111/1750-3841.15852
EFFECTS OF DIGESTED JABUTICABA (MYRCIARIA JABOTICABA (VELL.) BERG) BY-PRODUCT ON GROWTH AND METABOLISM OF LACTOBACILLUS AND BIFIDOBACTERIUM INDICATE PREBIOTIC PROPERTIES	10.1016/j.lwt.2020.109766
EFFECTS OF DISINTEGRATION ON IN VITRO FERMENTATION AND CONVERSION PATTERNS OF WHEAT ALEURONE IN A METABOLICAL COLON MODEL	10.1021/jf4001814
EFFECTS OF DRY PLANT EXTRACTS ON FEED DEGRADATION AND THE PRODUCTION OF RUMEN MICROBIAL BIOMASS IN A DUAL OUTFLOW FERMENTER	10.1016/S0377-8401(02)00221-3
EFFECTS OF DRY PLANT EXTRACTS ON FERMENTATION AND METHANOGENESIS IN CONTINUOUS CULTURE OF RUMEN MICROBES	10.1016/S0377-8401(00)00193-0
EFFECTS OF DYNAMIC FLUID ACTIVITY FROM AN ELECTRIC TOOTHBRUSH ON IN VITRO ORAL BIOFILMS	10.1034/j.1600-051X.2003.00307.x
EFFECTS OF ENCAPSULATED LACTOBACILLUS ACIDOPHILUS ALONG WITH PASTEURIZED LONGAN JUICE ON THE COLON MICROBIOTA RESIDING IN A DYNAMIC SIMULATOR OF THE HUMAN INTESTINAL MICROBIAL ECOSYSTEM	10.1007/s00253-013-4763-7
EFFECTS OF ESCULIN AND ESCULETIN ON THE SURVIVAL OF ESCHERICHIA COLI 0157 IN HUMAN FAECAL SLURRIES, CONTINUOUS-FLOW SIMULATIONS OF THE RUMEN AND COLON AND IN CALVES	10.1079/BJN20041101
EFFECTS OF EXPOSURE OF CLOSTRIDIUM DIFFICILE PCR RIBOTYPES 027 AND 001 TO FLUOROQUINOLONES IN A HUMAN GUT MODEL	10.1128/AAC.00306-08
EFFECTS OF FERMENTATION PRODUCTS OF PRO- AND PREBIOTICS ON TRANS-EPITHELIAL ELECTRICAL RESISTANCE IN AN IN VITRO MODEL OF THE COLON	10.1207/s15327914nc5101_14
EFFECTS OF FERMENTED MILK TREATMENT ON MICROBIAL POPULATION AND METABOLOMIC OUTCOMES IN A THREE-STAGE SEMI-CONTINUOUS CULTURE SYSTEM	10.1016/j.foodchem.2018.04.095
EFFECTS OF FREEZING STORAGE ON THE DNA EXTRACTION AND MICROBIAL EVALUATION FROM ANAEROBIC DIGESTED SLUDGES	10.1186/s13104-015-1407-2
EFFECTS OF FUNCTIONAL PASTA INGREDIENTS ON DIFFERENT GUT MICROBIOTA AS REVEALED BY TIM-2 IN VITRO MODEL OF THE PROXIMAL COLON	10.3920/BM2018.0088
EFFECTS OF GASTROINTESTINAL DIGESTED POLYPHENOLIC ENRICHED EXTRACTS OF CHILEAN CURRANTS (RIBES MAGELLANICUM AND RIBES PUNCTATUM) ON IN VITRO FECAL MICROBIOTA	10.1016/j.foodres.2019.108848
EFFECTS OF HUMAN GUT MICROBIOTA ON BIOACCESSIBILITY OF SOIL CD, CR AND NI USING SHIME MODEL	10.13227/j.hjkx.2016.06.045

EFFECTS OF HUMAN MILK OLIGOSACCHARIDES ON THE ADULT GUT MICROBIOTA AND BARRIER FUNCTION	10.3390/nu12092808
EFFECTS OF IN VITRO DIGESTION AND IN VITRO COLONIC FERMENTATION ON STABILITY AND FUNCTIONAL PROPERTIES OF YERBA MATE (ILEX PARAGUARIENSIS A. ST. HIL.) BEVERAGES	10.1016/j.foodchem.2017.05.125
EFFECTS OF IN VITRO DIGESTION ON THE CONTENT AND BIOLOGICAL ACTIVITY OF POLYPHENOLS FROM ACACIA MEARNSII BARK	10.3390/molecules23071804
EFFECTS OF IN VITRO GASTROINTESTINAL DIGESTION AND COLONIC FERMENTATION ON BIOAVAILABILITY AND ANTIOXIDANT ACTIVITY OF PHENOLIC COMPOUNDS IN CHANGHEI HULLESS BARLEY	10.7506/spkx1002-6630-20191027-300
EFFECTS OF IN VITRO GASTROINTESTINAL DIGESTION ON PHENOLIC COMPOUNDS AND ANTIOXIDANT ACTIVITY OF DIFFERENT WHITE WINEMAKING BYPRODUCTS EXTRACTS	10.1016/j.foodres.2018.04.060
EFFECTS OF IN VITRO SALIVA, GASTRIC AND INTESTINAL DIGESTION ON THE CHEMICAL PROPERTIES, ANTIOXIDANT ACTIVITY OF POLYSACCHARIDE FROM ARTOCARPUS HETEROPHYLLUS LAM. (JACKFRUIT) PULP	10.1016/j.foodhyd.2018.09.014
EFFECTS OF IN VITRO SIMULATED GASTROINTESTINAL DIGESTION ON THE ANTIOXIDANT, A-GLUCOSIDASE AND A-AMYLASE INHIBITORY ACTIVITIES OF WATER-SOLUBLE POLYSACCHARIDES FROM OPILIA AMENTACEA ROXB FRUIT	10.1016/j.lwt.2019.05.079
EFFECTS OF INHOMOGENEITY ON TRIGLYCERIDE DIGESTION OF EMULSIONS USING AN: IN VITRO DIGESTION MODEL (TINY TIM)	10.1039/c4fo01045k
EFFECTS OF LACTOBACILLUS ACIDOPHILUS LA-3 ON PHYSICOCHEMICAL AND SENSORY PARAMETERS OF AÇAÍ AND MANGO BASED SMOOTHIES AND ITS SURVIVAL FOLLOWING SIMULATED GASTROINTESTINAL CONDITIONS	10.1016/j.foodres.2018.08.005
EFFECTS OF LACTOSE ON COLON MICROBIAL COMMUNITY STRUCTURE AND FUNCTION IN A FOUR-STAGE SEMI-CONTINUOUS CULTURE SYSTEM	10.1271/bbb.60022
EFFECTS OF LOW LEVELS OF CIPROFLOXACIN ON A CHEMOSTAT MODEL OF THE HUMAN COLONIC MICROFLORA	10.1006/rtph.2001.1473
EFFECTS OF OLIVE AND POMEGRANATE BY-PRODUCTS ON HUMAN MICROBIOTA: A STUDY USING THE SHIME¬Æ IN VITRO SIMULATOR	10.3390/molecules24203791
EFFECTS OF ORANGE JUICE FORMULATION ON PREBIOTIC FUNCTIONALITY USING AN IN VITRO COLONIC MODEL SYSTEM	10.1371/journal.pone.0121955
EFFECTS OF PREBIOTIC CARBOHYDRATES ON THE GROWTH PROMOTION AND CHOLESTEROL-LOWERING ABILITIES OF COMPOUND PROBIOTICS IN VITRO	10.1016/j.lwt.2019.108703
EFFECTS OF PREBIOTICS AND PROBIOTICS ON SWINE INTESTINAL MICROFLORA AND FERMENTATION PRODUCTS IN VITRO FERMENTATION	10.7845/kjm.2013.232
EFFECTS OF PROBIOTIC BACTERIA AND THEIR GENOMIC DNA ON TH1/TH2-CYTOKINE PRODUCTION BY PERIPHERAL BLOOD MONONUCLEAR CELLS (PBMCS) OF HEALTHY AND ALLERGIC SUBJECTS	10.1016/j.imbio.2008.02.001
EFFECTS OF PROBIOTICS AND ANTIBIOTICS ON THE INTESTINAL HOMEOSTASIS IN A COMPUTER CONTROLLED MODEL OF THE LARGE INTESTINE	10.1186/1471-2180-12-47
EFFECTS OF RESISTANT STARCH TYPE III POLYMORPHS ON HUMAN COLON MICROBIOTA AND SHORT CHAIN FATTY ACIDS IN HUMAN GUT MODELS	10.1021/jf800284d
EFFECTS OF SARAFLOXACIN HYDROCHLORIDE ON HUMAN ENTERIC BACTERIA UNDER SIMULATED HUMAN GUT CONDITIONS	10.1080/01652176.1995.9694519
EFFECTS OF SIMULATED GASTRIC AND INTESTINAL DIGESTION ON CHITOOLIGOSACCHARIDES IN TWO IN VITRO MODELS	10.1111/ijfs.14337
EFFECTS OF SIMULATED HUMAN GASTROINTESTINAL DIGESTION OF TWO PURPLE-FLESHED POTATO CULTIVARS ON ANTHOCYANIN COMPOSITION AND CYTOTOXICITY IN COLONIC CANCER AND NON-TUMORIGENIC CELLS	10.3390/nu9090953
EFFECTS OF SOYBEAN PROTEIN ISOLATES AND PEPTIDES ON THE GROWTH AND METABOLISM OF LACTOBACILLUS RHAMNOSUS	10.1016/j.jff.2020.104335

EFFECTS OF THE FOOD MANUFACTURING CHAIN ON THE VIABILITY AND FUNCTIONALITY OF BIFIDOBACTERIUM ANIMALIS THROUGH SIMULATED GASTROINTESTINAL CONDITIONS	10.1371/journal.pone.0157958
EFFECTS OF WINE AND ITS MICROBIAL-DERIVED METABOLITES ON INTESTINAL PERMEABILITY USING SIMULATED GASTROINTESTINAL DIGESTION/COLONIC FERMENTATION AND CACO-2 INTESTINAL CELL MODELS	10.3390/microorganisms9071378
EFFECTS OF: IN VITRO DIGESTION AND FECAL FERMENTATION ON THE STABILITY AND METABOLIC BEHAVIOR OF POLYSACCHARIDES FROM CRATERELLUS CORNUCOPIOIDES	10.1039/d0fo01430c
EFFECTS OF: IN VITRO DIGESTION-FERMENTATION OVER GLOBAL ANTIOXIDANT RESPONSE AND SHORT CHAIN FATTY ACID PRODUCTION OF BEET WASTE EXTRACTS IN CA(II)-ALGINATE BEADS	10.1039/d0fo02347g
EFFICACY OF ALTERNATIVE FIDAXOMICIN DOSING REGIMENS FOR TREATMENT OF SIMULATED CLOSTRIDIUM DIFFICILE INFECTION IN AN IN VITRO HUMAN GUT MODEL	10.1093/jac/dkv156
EFFICACY OF POLYMER COATING OF PROBIOTIC BEADS SUSPENDED IN PRESSURIZED AND PASTEURIZED LONGAN JUICES ON THE EXPOSURE TO SIMULATED GASTROINTESTINAL ENVIRONMENT	10.3109/09637486.2013.799124
EFFICACY OF SUROTOMYCIN IN AN IN VITRO GUT MODEL OF CLOSTRIDIUM DIFFICILE INFECTION	10.1093/jac/dku141
EFFICACY OF VANCOMYCIN EXTENDED-DOSING REGIMENS FOR TREATMENT OF SIMULATED CLOSTRIDIUM DIFFICILE INFECTION WITHIN AN IN VITRO HUMAN GUT MODEL	10.1093/jac/dkv453
ELUCIDATION OF INTERACTION BETWEEN WHEY PROTEINS AND PROANTHOCYANIDINS AND ITS PROTECTIVE EFFECTS ON PROANTHOCYANIDINS DURING IN-VITRO DIGESTION AND STORAGE	10.3390/molecules26185468
EMULSION AND PROTEIN DEGRADATION IN THE ELDERLY: QUALITATIVE INSIGHTS FROM A STUDY COUPLING A DYNAMIC IN VITRO DIGESTION MODEL WITH PROTEOMIC ANALYSES	10.1016/j.foodhyd.2017.02.017
EMULSION ENCAPSULATION IN CALCIUM-ALGINATE BEADS DELAYS LIPOLYSIS DURING DYNAMIC IN VITRO DIGESTION	10.1016/j.jff.2018.05.011
EMULSION STABILITY DURING GASTROINTESTINAL CONDITIONS EFFECTS LIPID DIGESTION KINETICS	10.1016/j.foodchem.2017.11.001
ENCAPSULATION OF BIFIDOBACTERIUM IN ALGINATE MICROGELS IMPROVES VIABILITY AND TARGETED GUT RELEASE	10.1016/j.foodhyd.2021.106634
ENCAPSULATION OF BIFIDOBACTERIUM PSEUDOCATENULATUM G7 IN GASTROPROTECTIVE MICROGELS: IMPROVEMENT OF THE BACTERIAL VIABILITY UNDER SIMULATED GASTROINTESTINAL CONDITIONS	10.1016/j.foodhyd.2019.01.040
ENCAPSULATION OF LACTOBACILLUS REUTERI IN W1/O/W2 DOUBLE EMULSIONS: FORMULATION, STORAGE AND IN VITRO GASTRO-INTESTINAL DIGESTION STABILITY	10.1016/j.lwt.2021.111423
ENCAPSULATION OF VITAMIN E AND SOY ISOFLAVONE USING SPIRAL DEXTRIN: COMPARATIVE STRUCTURAL CHARACTERIZATION, RELEASE KINETICS, AND ANTIOXIDANT CAPACITY DURING SIMULATED GASTROINTESTINAL TRACT	10.1021/acs.jafc.8b00644
ENHANCEMENT OF INTESTINAL EPITHELIAL BARRIER FUNCTION BY WEISSELLA CONFUSA F213 AND LACTOBACILLUS RHAMNOSUS FBB81 PROBIOTIC CANDIDATES IN AN IN VITRO MODEL OF HYDROGEN PEROXIDE-INDUCED INFLAMMATORY BOWEL DISEASE	10.1186/s13104-020-05338-1
ENRICHMENT OF BIFIDOBACTERIA FROM HUMAN GUT CONTENTS BY OLIGOFRUCTOSE USING CONTINUOUS CULTURE	10.1111/j.1574-6968.1994.tb06813.x
ENRICHMENT OF BIFIDOBACTERIUM LONGUM SUBSP. INFANTIS ATCC 15697 WITHIN THE HUMAN GUT MICROBIOTA USING ALGINATE-POLY-L-LYSINE-ALGINATE MICROENCAPSULATION ORAL DELIVERY SYSTEM: AN IN VITRO ANALYSIS USING A COMPUTER-CONTROLLED DYNAMIC HUMAN GASTROINTESTINAL MODEL	10.3109/02652048.2013.834990
ENRICHMENT OF FOOD WITH TANNIN EXTRACTS PROMOTES HEALTHY CHANGES IN THE HUMAN GUT MICROBIOTA	10.3389/fmicb.2021.625782
ENTEROAGGREGATIVE ESCHERICHIA COLI PROMOTES TRANSEPITHELIAL MIGRATION OF NEUTROPHILS THROUGH A CONSERVED 12-LIPOXYGENASE PATHWAY	10.1111/j.1462-5822.2011.01706.x
ENTEROCOCCUS FAECALIS ISOLATED FROM INFANT FECES INHIBITS TOXIGENIC CLOSTRIDIOIDES (CLOSTRIDIUM) DIFFICILE	10.3389/fped.2020.572633

ENTEROHEMORRHAGIC ESCHERICHIA COLI O157: H7 SURVIVAL IN AN IN VITRO MODEL OF THE HUMAN LARGE INTESTINE AND INTERACTIONS WITH PROBIOTIC YEASTS AND RESIDENT MICROBIOTA	10.1128/AEM.03303-12
ENUMERATION OF HUMAN COLONIE BACTERIA PRODUCING PHENOLIC AND INDOLIC COMPOUNDS : EFFECTS OF PH, CARBOHYDRATE AVAILABILITY AND RETENTION TIME ON DISSIMILATORY AROMATIC AMINO ACID METABOLISM	10.1111/j.1365-2672.1996.tb04331.x
ENZYME FUNCTIONALIZED MICROGELS ENABLE PRECISE REGULATION OF DISSOLVED OXYGEN AND ANAEROBE CULTURE	10.1016/j.mtbio.2020.100092
EPITOPES RESISTANCE TO THE SIMULATED GASTROINTESTINAL DIGESTION OF Œí-LACTOGLOBULIN SUBMITTED TO TWO-STEP ENZYMATIC MODIFICATION	10.1016/j.foodres.2015.03.044
ERAVACYCLINE, A NOVEL TETRACYCLINE DERIVATIVE, DOES NOT INDUCE CLOSTRIDIOIDES DIFFICILE INFECTION IN AN IN VITRO HUMAN GUT MODEL	10.1093/JAC/DKAA386
ERRATUM: NEW THREE-STAGE IN VITRO MODEL FOR INFANT COLONIC FERMENTATION WITH IMMOBILIZED FECAL MICROBIOTA (FEMS MICROBIOLOGY ECOLOGY (2006) 57 (226-238))	10.1111/j.1574-6941.2006.00164.x
ESTABLISHING A MUCOSAL GUT MICROBIAL COMMUNITY IN VITRO USING AN ARTIFICIAL SIMULATOR	10.1371/journal.pone.0197692
ESTABLISHMENT AND APPLICATION OF PERISTALTIC HUMAN GUT-VESSEL MICROSYSTEM FOR STUDYING HOST-MICROBIAL INTERACTION	10.3389/fbioe.2020.00272
ESTABLISHMENT OF AN IN VITRO SYSTEM OF THE HUMAN INTESTINAL MICROBIOTA: EFFECT OF CULTIVATION CONDITIONS AND INFLUENCE OF THREE DONOR STOOL SAMPLES	10.3390/microorganisms9051049
ESTIMATION OF THE BIOACCESSIBILITY AND BIOAVAILABILITY OF FE, MN, CU, AND ZN IN CHINESE VEGETABLES USING THE IN VITRO DIGESTION/CACO-2 CELL MODEL: THE INFLUENCE OF GUT MICROBIOTA	10.1039/C7FO01348E
ESTIMATION OF THE BIOAVAILABILITY OF IRON AND PHOSPHORUS IN CEREALS USING A DYNAMIC IN VITRO GASTROINTESTINAL MODEL	10.1002/(SICI)1097-0010(199705)74:1<99::AID-JSFA775>3.0.CO;2-G
ESTIMATION OF THE FERMENTABILITY OF DIETARY FIBRE IN VITRO: A EUROPEAN INTERLABORATORY STUDY	10.1079/BJN19950137
ETHYLENE VINYL ACETATE AS MATRIX FOR ORAL SUSTAINED RELEASE DOSAGE FORMS PRODUCED VIA HOT-MELT EXTRUSION	10.1016/j.ejpb.2010.12.004
EVALUATING CHANGES IN MICROBIAL POPULATION AND EARTHWORMS WEIGHT DURING VERMICOMPOSTING OF COW MANURE CONTAINING CO-TRIMOXAZOLE	10.1007/s40201-019-00404-8
EVALUATING IN VITRO CULTURE MEDIUM OF GUT MICROBIOME WITH ORTHOGONAL EXPERIMENTAL DESIGN AND A METAPROTEOMICS APPROACH	10.1021/acs.jproteome.7b00461
EVALUATING THE CLINICAL IMPORTANCE OF BACTERIAL DEGRADATION OF THERAPEUTIC AGENTS IN THE LOWER INTESTINE OF ADULTS USING ADULT FECAL MATERIAL	10.1016/j.ejps.2018.09.019
EVALUATING THE MICROBIAL DIVERSITY OF AN IN VITRO MODEL OF THE HUMAN LARGE INTESTINE BY PHYLOGENETIC MICROARRAY ANALYSIS	10.1099/mic.0.042044-0
EVALUATION OF 16S RRNA AND CELLULAR FATTY ACID PROFILES AS MARKERS OF HUMAN INTESTINAL BACTERIAL GROWTH IN THE CHEMOSTAT	10.1046/j.1365-2672.2000.01165.x
EVALUATION OF ACRYLAMIDE-REMOVING PROPERTIES OF BACTERIAL CONSORTIA UNDER SIMULATED GASTROINTESTINAL CONDITIONS	10.1002/jsfa.11149
EVALUATION OF AN OPTIMAL PREPARATION OF HUMAN STANDARDIZED FECAL INOCULA FOR IN VITRO FERMENTATION STUDIES	10.1016/j.mimet.2015.07.019
EVALUATION OF ANTIMICROBIAL ACTIVITY OF CEFTAROLINE AGAINST CLOSTRIDIUM DIFFICILE AND PROPENSITY TO INDUCE C. DIFFICILE INFECTION IN AN IN VITRO HUMAN GUT MODEL	10.1093/jac/dkt107
EVALUATION OF DAIDZEIN-LOADED CHITOSAN MICROCAPSULES FOR THE COLON CANCER DRUG DELIVERY: SYNTHESIS, CHARACTERIZATION AND RELEASE BEHAVIOUR	10.1007/s00289-021-03853-0

EVALUATION OF LACTOBACILLUS PARACASEI LP11 AND LACTOBACILLUS RHAMNOSUS 64 POTENTIAL AS CANDIDATES FOR USE AS PROBIOTICS IN FUNCTIONAL FOODS	10.15414/JMBFS.2020.9.6.1126-1133
EVALUATION OF LINEZOLID FOR THE TREATMENT OF CLOSTRIDIUM DIFFICILE INFECTION CAUSED BY EPIDEMIC STRAINS USING AN IN VITRO HUMAN GUT MODEL	10.1093/jac/dkr155
EVALUATION OF MICROBIAL COMMUNITY REPRODUCIBILITY, STABILITY AND COMPOSITION IN A HUMAN DISTAL GUT CHEMOSTAT MODEL	10.1016/j.mimet.2013.08.008
EVALUATION OF MICROENCAPSULATED SYNBIOTIC PREPARATIONS CONTAINING LACTOBIONIC ACID	10.1007/s12010-021-03622-9
EVALUATION OF NVB302 VERSUS VANCOMYCIN ACTIVITY IN AN IN VITRO HUMAN GUT MODEL OF CLOSTRIDIUM DIFFICILE INFECTION	10.1093/jac/dks359
EVALUATION OF ORAL BIOACCESSIBILITY OF AGED CITRUS PEEL EXTRACTS ENCAPSULATED IN DIFFERENT LIPID-BASED SYSTEMS: A COMPARISON STUDY USING DIFFERENT IN VITRO DIGESTION MODELS	10.1021/acs.jafc.9b05372
EVALUATION OF SOLUBLE CORN FIBER ON CHEMICAL COMPOSITION AND NITROGEN-CORRECTED TRUE METABOLIZABLE ENERGY AND ITS EFFECTS ON IN VITRO FERMENTATION AND IN VIVO RESPONSES IN DOGS	10.2527/jas.2014-8425
EVALUATION OF SOLUBLE CORN FIBER ON CHEMICAL COMPOSITION AND NITROGEN-CORRECTED TRUE METABOLIZABLE ENERGY AND ITS EFFECTS ON IN VITRO FERMENTATION AND IN VIVO RESPONSES IN DOGS	10.2527/jas.2014-8425
EVALUATION OF THE FATE OF LACTOBACILLUS CRISPATUS BC4, CARRIED IN SQUACQUERONE CHEESE, THROUGHOUT THE SIMULATOR OF THE HUMAN INTESTINAL MICROBIAL ECOSYSTEM (SHIME)	10.1016/j.foodres.2020.109580
EVALUATION OF THE PASSAGE OF LACTOBACILLUS GASSERI K7 AND BIFIDOBACTERIA FROM THE STOMACH TO INTESTINES USING A SINGLE REACTOR MODEL	10.1186/1471-2180-9-87
EVALUATION OF THE PHYSICAL CHANGES OF DIFFERENT SOLUBLE FIBRES PRODUCED DURING AN IN VITRO DIGESTION	10.1016/j.jff.2019.103518
EVALUATION OF THE PREBIOTIC POTENTIAL OF A COMMERCIAL SYNBIOTIC FOOD INGREDIENT ON GUT MICROBIOTA IN AN EX VIVO MODEL OF THE HUMAN COLON	10.3390/nu12092669
EVALUATION OF THE PREBIOTIC POTENTIAL OF FIVE KIWIFRUIT CULTIVARS AFTER SIMULATED GASTROINTESTINAL DIGESTION AND FERMENTATION WITH HUMAN FAECAL BACTERIA	10.1111/ijfs.13697
EVALUATION OF THE PROBIOTIC PROPERTIES AND THE CAPACITY TO FORM BIOFILMS OF VARIOUS LACTOBACILLUS STRAINS	10.3390/microorganisms8071053
EVALUATION OF THE ROLE OF ENVIRONMENTAL FACTORS IN THE HUMAN GASTROINTESTINAL TRACT ON THE BEHAVIOUR OF PROBIOTIC CULTURES OF LACTOBACILLUS CASEI SHIROTA AND LACTOBACILLUS CASEI LC01 BY THE USE OF A SEMI-DYNAMIC IN VITRO MODEL	10.1007/BF03175128
EVALUATION OF TWO DYNAMIC IN VITRO MODELS SIMULATING FASTED AND FED STATE CONDITIONS IN THE UPPER GASTROINTESTINAL TRACT (TIM-1 AND TINY-TIM) FOR INVESTIGATING THE BIOACCESSIBILITY OF PHARMACEUTICAL COMPOUNDS FROM ORAL DOSAGE FORMS	10.1016/j.ijpharm.2015.11.048
EVALUATION OF XYLOOLIGOSACCHARIDES AND FRUCTOOLIGOSACCHARIDES ON DIGESTIVE ENZYMES HYDROLYSIS AND AS A NUTRIENT FOR DIFFERENT PROBIOTICS AND SALMONELLA TYPHIMURIUM	10.1016/j.lwt.2019.108761
EVOLVING MICROBIAL COMMUNITIES IN CELLULOSE-FED MICROBIAL FUEL CELL	10.3390/en11010124
EXAMINING THE EFFECTS OF AN ANTI-SALMONELLA BACTERIOPHAGE PREPARATION, BAFASAL®, ON EX-VIVO HUMAN GUT MICROBIOME COMPOSITION AND FUNCTION USING A MULTI-OMICS APPROACH	10.3390/v13091734
EXOPOLYSACCHARIDES PRODUCED BY BIFIDOBACTERIUM LONGUM IPLA E44 AND BIFIDOBACTERIUM ANIMALIS SUBSP. LACTIS IPLA R1 MODIFY THE COMPOSITION AND METABOLIC ACTIVITY OF HUMAN FAECAL MICROBIOTA IN PH-CONTROLLED BATCH CULTURES	10.1016/j.ijfoodmicro.2009.08.017
EXPOSURE TO MINIMALLY PROCESSED PEAR AND MELON DURING SHELF LIFE COULD MODIFY THE PATHOGENIC POTENTIAL OF LISTERIA MONOCYTOGENES	10.1016/j.fm.2016.10.016
EXTRACTION AND CHARACTERISATION OF ARABINOXYLAN FROM BREWERS SPENT GRAIN AND INVESTIGATION OF MICROBIOME MODULATION POTENTIAL	10.1007/s00394-021-02570-8

FABRICATION AND CHARACTERIZATION OF SOYBEAN OIL BODIES ENCAPSULATED IN MALTODEXTRIN AND CHITOSAN-EGCG CONJUGATES: AN IN VITRO DIGESTIBILITY STUDY	10.1016/j.foodhyd.2019.04.001
FACTORS AFFECTING THE BIOACCESSIBILITY AND INTESTINAL TRANSPORT OF DIFENOCONAZOLE, HEXACONAZOLE, AND SPIRODICLOFEN IN HUMAN CACO-2 CELLS FOLLOWING IN VITRO DIGESTION	10.1021/acs.jafc.7b02781
FACTORS AFFECTING THE BIOACCESSIBILITY OF POLYBROMINATED DIPHENYLETHERS IN AN IN VITRO DIGESTION MODEL	10.1021/jf802659u
FACTORS AFFECTING THE CONVERSION OF APPLE POLYPHENOLS TO PHENOLIC ACIDS AND FRUIT MATRIX TO SHORT-CHAIN FATTY ACIDS BY HUMAN FAECAL MICROBIOTA IN VITRO	10.1007/s00394-008-0747-2
FAECAL MICROBIAL METABOLISM OF OLIVE OIL PHENOLIC COMPOUNDS: IN VITRO AND IN VIVO APPROACHES	10.1002/mnfr.201400124
FAECAL MICROBIOTA TRANSPLANTATION: A SUI GENERIS BIOLOGICAL DRUG, NOT A TISSUE	10.1016/j.pharma.2014.04.008
FAECAL STEROIDS AND COLORECTAL CANCER: THE EFFECT OF LACTULOSE ON FAECAL BACTERIAL METABOLISM IN A CONTINUOUS CULTURE MODEL OF THE LARGE INTESTINE	10.1097/00008469-199202000-00004
FATE AND BIOACCESSIBILITY OF IODINE IN FOOD PREPARED FROM AGRONOMICALLY BIOFORTIFIED WHEAT AND RICE AND IMPACT OF COFERTILIZATION WITH ZINC AND SELENIUM	10.1021/acs.jafc.9b05912
FATE AND EFFICACY OF LACTICIN 3147-PRODUCING LACTOCOCCUS LACTIS IN THE MAMMALIAN GASTROINTESTINAL TRACT	10.1111/j.1574-6941.2011.01069.x
FATE OF CHITIN-GLUCAN IN THE HUMAN GASTROINTESTINAL TRACT AS STUDIED IN A DYNAMIC GUT SIMULATOR (SHIME®)	10.1016/j.jff.2017.01.030
FATE OF CMY-2-ENCODING PLASMIDS INTRODUCED INTO THE HUMAN FECAL MICROBIOTA BY EXOGENOUS ESCHERICHIA COLI	10.1128/AAC.02528-18
FATE OF INGESTED CLOSTRIDIUM DIFFICILE SPORES IN MICE	10.1371/journal.pone.0072620
FATE OF SUCRALOSE DURING WASTEWATER TREATMENT	10.1089/ees.2010.0227
FECAL MICROBIOTA RESPONSES TO BRAN PARTICLES ARE SPECIFIC TO CEREAL TYPE AND IN VITRO DIGESTION METHODS THAT MIMIC UPPER GASTROINTESTINAL TRACT PASSAGE	10.1021/acs.jafc.8b03469
FEEDING BUGS TO BUGS: EDIBLE INSECTS MODIFY THE HUMAN GUT MICROBIOME IN AN IN VITRO FERMENTATION MODEL	10.3389/fmicb.2020.01763
FERMENTATION BY GUT MICROBIOTA CULTURED IN A SIMULATOR OF THE HUMAN INTESTINAL MICROBIAL ECOSYSTEM IS IMPROVED BY PROBIOTIC ENTEROCOCCUS FAECIUM CRL 183	10.31989/ffhd.v1i10.119
FERMENTATION BY GUT MICROBIOTA CULTURED IN A SIMULATOR OF THE HUMAN INTESTINAL MICROBIAL ECOSYSTEM IS IMPROVED BY SUPPLEMENTING A SOYGERM POWDER	10.1093/jn/130.10.2599
FERMENTATION IN THE HUMAN LARGE INTESTINE: ITS PHYSIOLOGIC CONSEQUENCES AND THE POTENTIAL CONTRIBUTION OF PREBIOTICS	10.1097/MCG.0b013e31822fecfe
FERMENTATION PROFILES OF WHEAT DEXTRIN, INULIN AND PARTIALLY HYDROLYZED GUAR GUM USING AN IN VITRO DIGESTION PRETREATMENT AND IN VITRO BATCH FERMENTATION SYSTEM MODEL	10.3390/nu5051500
FERMENTATION PROPERTIES OF ISOMALTOOLIGOSACCHARIDES ARE AFFECTED BY HUMAN FECAL ENTEROTYPES	10.1016/j.anaerobe.2017.08.016
FLUX ANALYSIS OF THE HUMAN PROXIMAL COLON USING ANAEROBIC DIGESTION MODEL 1	10.1016/j.anaerobe.2014.05.008
FOLLOWING THE COMMUNITY DEVELOPMENT OF SIHUMIX,ÄìA NEW INTESTINAL IN VITRO MODEL FOR BIOREACTOR USE	10.1080/19490976.2019.1702431
FOOD-GRADE TITANIUM DIOXIDE PARTICLES DECREASE THE BIOACCESSIBILITY OF IRON RELEASED FROM SPINACH LEAVES IN SIMULATED HUMAN GASTROINTESTINAL TRACT	10.1039/d1en00064k
FORMATION OF LIPID AND PROTEIN OXIDATION PRODUCTS DURING IN VITRO GASTROINTESTINAL DIGESTION OF DRY-CURED LOINS WITH DIFFERENT CONTENTS OF NITRATE/NITRITE ADDED	10.3390/foods10081748
FORMATION OF MALONDIALDEHYDE (MDA), 4-HYDROXY-2-HEXENAL (HHE) AND 4-HYDROXY-2-NONENAL (HNE) IN FISH AND FISH OIL DURING DYNAMIC GASTROINTESTINAL IN VITRO DIGESTION	10.1039/c5fo01401h

FORMATION OF PHENOLIC MICROBIAL METABOLITES AND SHORT-CHAIN FATTY ACIDS FROM RYE, WHEAT, AND OAT BRAN AND THEIR FRACTIONS IN THE METABOLICAL IN VITRO COLON MODEL	10.1021/jf3008037
FORMATION OF REACTIVE ALDEHYDES (MDA, HHE, HNE) DURING THE DIGESTION OF COD LIVER OIL: COMPARISON OF HUMAN AND PORCINE IN VITRO DIGESTION MODELS	10.1039/c5fo01332a
FORMULATION AND CHARACTERIZATION OF GELATIN-BASED HYDROGELS FOR THE ENCAPSULATION OF KLUYVEROMYCES LACTIS-APPLICATIONS IN PACKED-BED REACTORS AND PROBIOTICS DELIVERY IN HUMANS	10.3390/POLYM12061287
FRACTIONATION AND ANTIOXIDANT PROPERTIES OF RICE BRAN PROTEIN HYDROLYSATES STIMULATED BY IN VITRO GASTROINTESTINAL DIGESTION	10.1016/j.foodchem.2017.07.080
FRUCTANS WITH VARYING DEGREE OF POLYMERIZATION ENHANCE THE SELECTIVE GROWTH OF BIFIDOBACTERIUM ANIMALIS SUBSP. LACTIS BB-12 IN THE HUMAN GUT MICROBIOME IN VITRO	10.3390/app11020598
FU BRICK TEA EXTRACT SUPPLEMENTATION ENHANCED PROBIOTIC VIABILITY AND ANTIOXIDANT ACTIVITY OF TOFU UNDER SIMULATED GASTROINTESTINAL DIGESTION CONDITION	10.1039/c6ra20730h
FUNCTIONAL ANATOMY OF THE COLONIC BIOREACTOR: IMPACT OF ANTIBIOTICS AND SACCHAROMYCES BOULARDII ON BACTERIAL COMPOSITION IN HUMAN FECAL CYLINDERS	10.1016/j.syapm.2015.11.002
FUNCTIONAL EFFICACY OF PROBIOTIC LACTOBACILLUS SANFRANCISCENSIS IN APPLE, ORANGE AND TOMATO JUICES WITH SPECIAL REFERENCE TO STORAGE STABILITY AND IN VITRO GASTROINTESTINAL SURVIVAL	10.3390/beverages6010013
FUNCTIONAL PROPERTIES OF FREE AND ENCAPSULATED LACTOBACILLUS REUTERI DPC16 DURING AND AFTER PASSAGE THROUGH A SIMULATED GASTROINTESTINAL TRACT	10.1007/s11274-011-0792-5
FUNCTIONAL PROPERTIES OF LACTOBACILLUS CASEI C24 IMPROVED BY MICROENCAPSULATION USING MULTILAYER DOUBLE EMULSION	10.1016/j.foodres.2021.110136
FUSOBACTERIUM NUCLEATUM ADHERES TO CLOSTRIDIOIDES DIFFICILE VIA THE RADD ADHESIN TO ENHANCE BIOFILM FORMATION IN INTESTINAL MUCUS	10.1053/j.gastro.2020.11.034
GALACTO-OLIGOSACCHARIDES HAVE PREBIOTIC ACTIVITY IN A DYNAMIC IN VITRO COLON MODEL USING A 13C-LABELING TECHNIQUE	10.3945/jn.111.157420
GASTRIC DIGESTION IN VIVO AND IN VITRO: HOW THE STRUCTURAL ASPECTS OF FOOD INFLUENCE THE DIGESTION PROCESS	10.1146/annurev-food-030713-092346
GASTRIC EMPTYING AND MORPHOLOGY OF A 'NEAR REAL' IN VITRO HUMAN STOMACH MODEL (RD-IV-HSM)	10.1016/j.jfoodeng.2016.02.025
GASTRIC LIPASE CAN SIGNIFICANTLY INCREASE LIPOLYSIS AND CAROTENOID BIOACCESSIBILITY FROM PLANT FOOD MATRICES IN THE HARMONIZED INFOGEST STATIC IN VITRO DIGESTION MODEL	10.1039/d1fo00786f
GASTROINTESTINAL BIOACCESSIBILITY AND COLONIC FERMENTATION OF FUCOXANTHIN FROM THE EXTRACT OF THE MICROALGA NITZSCHIA LAEVIS	10.1021/acs.jafc.9b02496
GASTROINTESTINAL DIGESTION AND FERMENTATION CHARACTERISTICS IN VITRO OF BREADS INCORPORATED WITH THREE DIFFERENT POLYSACCHARIDES	10.7506/spkx1002-6630-20200110-125
GASTROINTESTINAL DIGESTION MODEL ASSESSMENT OF PEPTIDE DIVERSITY AND MICROBIAL FERMENTATION PRODUCTS OF COLLAGEN HYDROLYSATES	10.3390/nu13082720
GASTROINTESTINAL DIGESTION OF DIETARY ADVANCED GLYCATION ENDPRODUCTS USING AN: IN VITRO MODEL OF THE GASTROINTESTINAL TRACT (TIM-1)	10.1039/d0fo00450b
GASTROINTESTINAL DIGESTION OF FOOD-USE SILVER NANOPARTICLES IN THE DYNAMIC SIMULATOR OF THE GASTROINTESTINAL TRACT (SIMGI®). IMPACT ON HUMAN GUT MICROBIOTA	10.1016/j.fct.2019.110657
GASTROINTESTINAL DYSFUNCTION IN PATIENTS AND MICE EXPRESSING THE AUTISM-ASSOCIATED R451C MUTATION IN NEUROLIGIN-3	10.1002/aur.2127
GASTROINTESTINAL MICROBES INCREASE ARSENIC BIOACCESSIBILITY OF INGESTED MINE TAILINGS USING THE SIMULATOR OF THE HUMAN INTESTINAL MICROBIAL ECOSYSTEM	10.1021/es062410e

GASTROINTESTINAL OR SIMULATED IN VITRO DIGESTION CHANGES DIETARY FIBRE PROPERTIES AND THEIR FERMENTATION	10.1002/(SICI)1097-0010(199807)77:3<327::AID-JSFA41>3.0.CO;2-5
GASTROINTESTINAL SIMULATION MODEL TWIN-SHIME SHOWS DIFFERENCES BETWEEN HUMAN UROLITHIN-METABOTYPES IN GUT MICROBIOTA COMPOSITION, POMEGRANATE POLYPHENOL METABOLISM, AND TRANSPORT ALONG THE INTESTINAL TRACT	10.1021/acs.jafc.7b02049
GASTROINTESTINAL STABILITY OF UROLITHINS: AN IN VITRO APPROACH	10.1007/s00394-015-1061-4
GASTROINTESTINAL SURVIVAL AND ADAPTATION OF ANTIBIOTIC-RESISTANT ENTEROCOCCI SUBJECTED TO AN IN VITRO DIGESTION MODEL	10.1016/j.foodcont.2019.107033
GENE EXPRESSION ANALYSIS OF E. COLI STRAINS PROVIDES INSIGHTS INTO THE ROLE OF GENE REGULATION IN DIVERSIFICATION	10.1038/ismej.2014.204
GENOME-RESOLVED META-OMICS TIES MICROBIAL DYNAMICS TO PROCESS PERFORMANCE IN BIOTECHNOLOGY FOR THIOCYANATE DEGRADATION	10.1021/acs.est.6b04477
GERMINATED SOYBEAN PROTEIN HYDROLYSATE: IONIC GELATION ENCAPSULATION AND RELEASE UNDER COLONIC CONDITIONS	10.24275/rmiq/Alim2319
GLYCEROL INDUCES REUTERIN PRODUCTION AND DECREASES ESCHERICHIA COLI POPULATION IN AN IN VITRO MODEL OF COLONIC FERMENTATION WITH IMMOBILIZED HUMAN FECES	10.1111/j.1574-6941.2007.00412.x
GLYCOMACROPEPTIDE SUSTAINS MICROBIOTA DIVERSITY AND PROMOTES SPECIFIC TAXA IN AN ARTIFICIAL COLON MODEL OF ELDERLY GUT MICROBIOTA	10.1021/acs.jafc.6b05434
GLYCOSIDIC LINKAGE STRUCTURES INFLUENCE DIETARY FIBER FERMENTABILITY AND PROPIONATE PRODUCTION BY HUMAN COLONIC MICROBIOTA IN VITRO	10.1002/biot.201900523
GNOTOBIOTIC MOUSE MODEL OF PHAGE-BACTERIAL HOST DYNAMICS IN THE HUMAN GUT	10.1073/pnas.1319470110
GOLD NANOPARTICLES ENHANCE MICRORNA 31 DETECTION IN COLON CANCER CELLS AFTER INHIBITION WITH CHLOROGENIC ACID	10.3892/ol.2021.13003
GRAM-POSITIVE BACTERIA ARE HELD AT A DISTANCE IN THE COLON MUCUS BY THE LECTIN-LIKE PROTEIN ZG16	10.1073/pnas.1611400113
GROWTH OF A HUMAN INTESTINAL DESULFOVIBRIO DESULFURICANS IN CONTINUOUS CULTURES CONTAINING DEFINED POPULATIONS OF SACCHAROLYTIC AND AMINO ACID FERMENTING BACTERIA	10.1046/j.1365-2672.1998.00522.x
GROWTH STIMULATION OF BIFIDOBACTERIUM FROM HUMAN COLON USING DAIKENCHUTO IN AN IN VITRO MODEL OF HUMAN INTESTINAL MICROBIOTA	10.1038/s41598-021-84167-z
GROWTH-PROMOTING EFFECT OF ALGINATE ON FAECALIBACTERIUM PRAUSNITZII THROUGH CROSS-FEEDING WITH BACTEROIDES	10.1016/j.foodres.2021.110326
GUAVA, ORANGE AND PASSION FRUIT BY-PRODUCTS: CHARACTERIZATION AND ITS IMPACTS ON KINETICS OF ACIDIFICATION AND PROPERTIES OF PROBIOTIC FERMENTED PRODUCTS	10.1016/j.lwt.2018.08.010
GUIDELINES FOR CLINICAL CARE: ANTI-INFECTIVE AGENTS FOR INTRA-ABDOMINAL INFECTION: A SURGICAL INFECTION SOCIETY POLICY STATEMENT	10.1001/archsurg.1992.01420010097015
GUT MICROBIAL ACTIVITY AS INFLUENCED BY FIBER DIGESTION: DYNAMIC METABOLOMICS IN AN IN VITRO COLON SIMULATOR	10.1007/s11306-015-0936-y
GUT MICROBIAL METABOLISM OF POLYPHENOLS FROM BLACK TEA AND RED WINE/GRAPE JUICE IS SOURCE-SPECIFIC AND COLON-REGION DEPENDENT	10.1021/jf303165w
GUT, BUGS, AND BRAIN: ROLE OF COMMENSAL BACTERIA IN THE CONTROL OF CENTRAL NERVOUS SYSTEM DISEASE	10.1002/ana.22344
HELICOBACTER PYLORI ANTIGENS, ACETYLSALICYLIC ACID, LDL AND 7-KETOCHOLESTEROL - THEIR POTENTIAL ROLE IN DESTABILIZING THE GASTRIC EPITHELIAL CELL BARRIER. AN IN VITRO MODEL OF KATO III CELLS	10.18388/abp.2015_1122

HERRING ROE PROTEIN HAS A HIGH DIGESTIBLE INDISPENSABLE AMINO ACID SCORE (DIAAS) USING A DYNAMIC IN VITRO GASTROINTESTINAL MODEL	10.1016/j.nutres.2016.05.004
HIGH CONCENTRATION BUT LOW ACTIVITY OF HEPATOCYTE GROWTH FACTOR IN PERIODONTITIS	10.1902/jop.2013.130003
HIGH PURITY PREBIOTIC ISOMALTO-OLIGOSACCHARIDES PRODUCTION BY CELL ASSOCIATED TRANSGLUCOSIDASE OF ISOLATED STRAIN DEBARYOMYCES HANSENII SCY204 AND SELECTIVE FERMENTATION BY SACCHAROMYCES CEREVISIAE SYI065	10.1016/j.procbio.2020.07.024
HIGH-FIBER AND HIGH-PROTEIN DIETS SHAPE DIFFERENT GUT MICROBIAL COMMUNITIES, WHICH ECOLOGICALLY BEHAVE SIMILARLY UNDER STRESS CONDITIONS, AS SHOWN IN A GASTROINTESTINAL SIMULATOR	10.1002/mnfr.201600150
HIGH-LEVEL DIETARY FIBRE UP-REGULATES COLONIC FERMENTATION AND RELATIVE ABUNDANCE OF SACCHAROLYTIC BACTERIA WITHIN THE HUMAN FAECAL MICROBIOTA IN VITRO	10.1007/s00394-011-0248-6
HIGHER MOLECULAR WEIGHT POLYETHYLENE GLYCOL INCREASES CELL PROLIFERATION WHILE IMPROVING BARRIER FUNCTION IN AN IN VITRO COLON CANCER MODEL	10.1155/2011/587470
HIGHER-LEVEL PRODUCTION OF VOLATILE FATTY ACIDS IN VITRO BY CHICKEN GUT MICROBIOTAS THAN BY HUMAN GUT MICROBIOTAS AS DETERMINED BY FUNCTIONAL ANALYSES	10.1128/AEM.00327-12
HOST-MICROBE INTERACTION IN THE GASTROINTESTINAL TRACT	10.3109/10408419509113535
HOW DOES THE DEGREE OF INULIN POLYMERIZATION AFFECT THE BIOACCESSIBILITY OF BIOACTIVE COMPOUNDS FROM SOURSOP WHEY BEVERAGE DURING IN VITRO GASTROINTESTINAL DIGESTION?	10.1016/j.foodhyd.2019.105511
HPLC-ICP-MS METHOD DEVELOPMENT TO MONITOR ARSENIC SPECIATION CHANGES BY HUMAN GUT MICROBIOTA	10.1002/bmc.1700
HUMAN COLON MICROBIOTA TRANSFORM POLYCYCLIC AROMATIC HYDROCARBONS TO ESTROGENIC METABOLITES	10.1289/ehp.7259
HUMAN COLONIC BACTERIAL DEGRADABILITY OF DIETARY FIBRES FROM SEA-LETTUCE (ULVA SP)	10.1002/(SICI)1097-0010(199702)73:2<149::AID-JSFA685>3.0.CO;2-L
HUMAN HEALTH RISK ASSESSMENT IN ALUMINIUM SMELTING SITE: SOIL FLUORIDE BIOACCESSIBILITY AND RELEVANT MECHANISM IN SIMULATED GASTROINTESTINAL TRACT	10.1016/j.jhazmat.2021.125899
HUMAN MICROBIOTA MODULATION VIA QSEC SENSOR KINASE MEDIATED IN THE ESCHERICHIA COLI O104:H4 OUTBREAK STRAIN INFECTION IN MICROBIOME MODEL	10.1186/s12866-021-02220-3
HUMAN MILK OLIGOSACCHARIDE 3'-GL IMPROVES INFLUENZA-SPECIFIC VACCINATION RESPONSIVENESS AND IMMUNITY AFTER DEOXYNIVALENOL EXPOSURE IN PRECLINICAL MODELS	10.3390/nu13093190
HUMAN MILK OLIGOSACCHARIDES AND INFANT GUT MICROBIOTA: MOLECULAR STRUCTURES, UTILIZATION STRATEGIES AND IMMUNE FUNCTION	10.1016/j.carbpol.2021.118738
HUMAN MILK OLIGOSACCHARIDES MEDIATE THE CROSSTALK BETWEEN INTESTINAL EPITHELIAL CACO-2 CELLS AND LACTOBACILLUS PLANTARUM WCFS1 IN AN IN VITRO MODEL WITH INTESTINAL PERISTALTIC SHEAR FORCE	10.1093/jn/nxaa162
HYDROCOLLOIDS IN HUMAN DIGESTION: DYNAMIC IN-VITRO ASSESSMENT OF THE EFFECT OF FOOD FORMULATION ON MASS TRANSFER	10.1016/j.foodhyd.2014.06.004
IDENTIFICATION OF GLUCOSE-FERMENTING BACTERIA PRESENT IN AN IN VITRO MODEL OF THE HUMAN INTESTINE BY RNA-STABLE ISOTOPE PROBING	10.1111/j.1574-6941.2007.00281.x
IMMOBILIZATION OF INFANT FECAL MICROBIOTA AND UTILIZATION IN AN IN VITRO COLONIC FERMENTATION MODEL	10.1007/s00248-003-2022-7
IMPACT OF A FERMENTED SOY BEVERAGE SUPPLEMENTED WITH ACEROLA BY-PRODUCT ON THE GUT MICROBIOTA FROM LEAN AND OBESE SUBJECTS USING AN IN VITRO MODEL OF THE HUMAN COLON	10.1007/s00253-021-11252-8
IMPACT OF A GASTROINTESTINAL STABLE PROBIOTIC SUPPLEMENT BACILLUS COAGULANS LBSC ON HUMAN GUT MICROBIOME MODULATION	10.1080/19390211.2020.1814931

IMPACT OF A PLANT STEROL- AND GALACTOOLIGOSACCHARIDE-ENRICHED BEVERAGE ON COLONIC METABOLISM AND GUT MICROBIOTA COMPOSITION USING AN IN VITRO DYNAMIC MODEL	10.1021/acs.jafc.9b04796
IMPACT OF BIOREACTOR ENVIRONMENT AND RECOVERY METHOD ON THE PROFILE OF BACTERIAL POPULATIONS FROM WATER DISTRIBUTION SYSTEMS	10.1371/journal.pone.0133427
IMPACT OF CASHEW (ANACARDIUM OCCIDENTALE L.) BY-PRODUCT ON COMPOSITION AND METABOLIC ACTIVITY OF HUMAN COLONIC MICROBIOTA IN VITRO INDICATES PREBIOTIC PROPERTIES	10.1007/s00284-021-02502-z
IMPACT OF CHRONIC EXPOSURE TO LOW DOSES OF CHLORPYRIFOS ON THE INTESTINAL MICROBIOTA IN THE SIMULATOR OF THE HUMAN INTESTINAL MICROBIAL ECOSYSTEM (SHIME¬Æ) AND IN THE RAT	10.1007/s11356-012-1283-4
IMPACT OF CHRONIC TETRACYCLINE EXPOSURE ON HUMAN INTESTINAL MICROBIOTA IN A CONTINUOUS FLOW BIOREACTOR MODEL	10.3390/antibiotics10080886
IMPACT OF COMBINING ACEROLA BY-PRODUCT WITH A PROBIOTIC STRAIN ON A GUT MICROBIOME MODEL	10.1080/09637486.2018.1498065
IMPACT OF CYADOX ON HUMAN COLONIC MICROFLORA IN CHEMOSTAT MODELS	10.1016/j.yrtph.2013.08.011
IMPACT OF DEFINED THERMOMECHANICAL TREATMENT ON THE STRUCTURE AND CONTENT OF DIETARY FIBER AND THE STABILITY AND BIOACCESSIBILITY OF POLYPHENOLS OF CHOKEBERRY (ARONIA MELANOCARPA) POMACE	10.1016/j.foodres.2020.109232
IMPACT OF DIETARY FIBER FERMENTATION FROM CEREAL GRAINS ON METABOLITE PRODUCTION BY THE FECAL MICROBIOTA FROM NORMAL WEIGHT AND OBESE INDIVIDUALS	10.1089/jmf.2012.0292
IMPACT OF DIETARY FIBERS [METHYL CELLULOSE, CHITOSAN, AND PECTIN] ON DIGESTION OF LIPIDS UNDER SIMULATED GASTROINTESTINAL CONDITIONS	10.1039/c4fo00615a
IMPACT OF ENCAPSULATED LACTOBACILLUS CASEI 01 ALONG WITH PASTEURIZED PURPLE-RICE DRINKS ON MODULATING COLON MICROBIOME USING A DIGESTIVE MODEL	10.1515/ijfe-2016-0008
IMPACT OF ENCAPSULATING PROBIOTICS WITH COCOA POWDER ON THE VIABILITY OF PROBIOTICS DURING CHOCOLATE PROCESSING, STORAGE, AND IN VITRO GASTROINTESTINAL DIGESTION	10.1111/1750-3841.15695
IMPACT OF EXPOSURE TO COLD AND COLD-OSMOTIC STRESSES ON VIRULENCE-ASSOCIATED CHARACTERISTICS OF LISTERIA MONOCYTOGENES STRAINS	10.1016/j.fm.2019.103351
IMPACT OF EXTREME OBESITY AND DIET-INDUCED WEIGHT LOSS ON THE FECAL METABOLOME AND GUT MICROBIOTA	10.1002/mnfr.202000030
IMPACT OF FOOD GRADE AND NANO-TIO2 PARTICLES ON A HUMAN INTESTINAL COMMUNITY	10.1016/j.fct.2017.05.050
IMPACT OF FUNCTIONAL FLOURS FROM PINEAPPLE BY-PRODUCTS ON HUMAN INTESTINAL MICROBIOTA	10.1016/j.jff.2020.103830
IMPACT OF GANEDENBC30 (BACILLUS COAGULANS GBI-30, 6086) ON POPULATION DYNAMICS OF THE HUMAN GUT MICROBIOTA IN A CONTINUOUS CULTURE FERMENTATION SYSTEM	
IMPACT OF HUMAN MILK PASTEURIZATION ON THE KINETICS OF PEPTIDE RELEASE DURING IN VITRO DYNAMIC DIGESTION AT THE PRETERM NEWBORN STAGE	10.1016/j.foodchem.2018.12.086
IMPACT OF INULIN AND OKARA ON LACTOBACILLUS ACIDOPHILUS LA-5 AND BIFIDOBACTERIUM ANIMALIS BB-12 VIABILITY IN A FERMENTED SOY PRODUCT AND PROBIOTIC SURVIVAL UNDER IN VITRO SIMULATED GASTROINTESTINAL CONDITIONS	10.1016/j.fm.2013.01.012
IMPACT OF MULTI-FUNCTIONAL FERMENTED GOAT MILK BEVERAGE ON GUT MICROBIOTA IN A DYNAMIC COLON MODEL	10.1016/j.foodres.2017.05.028
IMPACT OF ORALLY ADMINISTERED MICROCAPSULES ON GASTROINTESTINAL MICROBIAL FLORA: IN-VITRO INVESTIGATION USING COMPUTER CONTROLLED DYNAMIC HUMAN GASTROINTESTINAL MODEL	10.1080/10731190701460226
IMPACT OF PASTEURIZATION OF HUMAN MILK ON PRETERM NEWBORN IN VITRO DIGESTION: GASTROINTESTINAL DISINTEGRATION, LIPOLYSIS AND PROTEOLYSIS	10.1016/j.foodchem.2016.05.028
IMPACT OF PLANT STEROLS ENRICHMENT DOSE ON GUT MICROBIOTA FROM LEAN AND OBESE SUBJECTS USING TIM-2 IN VITRO FERMENTATION MODEL	10.1016/j.jff.2019.01.005
IMPACT OF POLYPHENOLS FROM BLACK TEA AND RED WINE/GRAPE JUICE ON A GUT MODEL MICROBIOME	10.1016/j.foodres.2013.01.034
IMPACT OF PRISTINE GRAPHENE ON INTESTINAL MICROBIOTA ASSESSED USING A BIOREACTOR-ROTARY CELL CULTURE SYSTEM	10.1021/acsami.9b07635

IMPACT OF SUBSTRATUM SURFACE ON MICROBIAL COMMUNITY STRUCTURE AND TREATMENT PERFORMANCE IN BIOLOGICAL AERATED FILTERS	10.1128/AEM.03001-13
IMPACT OF THERMAL PROCESSING ON PHYSICOCHEMICAL PROPERTIES OF SILK MOTH PUPAE (BOMBYX MORI) FLOUR AND IN-VITRO GASTROINTESTINAL PROTEOLYSIS IN ADULTS AND SENIORS	10.1016/j.foodres.2019.04.042
IMPACT OF: IN VITRO GASTROINTESTINAL DIGESTION ON THE CHEMICAL COMPOSITION, BIOACTIVE PROPERTIES, AND CYTOTOXICITY OF VITIS VINIFERA L. CV. SYRAH GRAPE POMACE EXTRACT	10.1039/c8fo02534g
IMPACTS OF DIETARY SILVER NANOPARTICLES AND PROBIOTIC ADMINISTRATION ON THE MICROBIOTA OF AN IN-VITRO GUT MODEL	10.1016/j.envpol.2018.11.019
IMPACTS OF DIFFERENT OPERATIONAL TEMPERATURES AND ORGANIC LOADS IN ANAEROBIC CO-DIGESTION OF FOOD WASTE AND SEWAGE SLUDGE ON THE FATE OF SARS-COV-2	10.1016/j.psep.2020.11.035
IMPORTANCE OF LOCATION OF DIGESTION AND COLONIC FERMENTATION OF STARCH RELATED TO ITS QUALITY	10.1094/CCHEM-05-13-0095-FI
IMPORTANCE OF SPECIES SORTING AND IMMIGRATION ON THE BACTERIAL ASSEMBLY OF DIFFERENT-SIZED AGGREGATES IN A FULL-SCALE AEROBIC GRANULAR SLUDGE PLANT	10.1021/acs.est.8b07303
IMPROVED BIOACCESSIBILITY AND ANTIOXIDANT CAPACITY OF OLIVE LEAF (OLEA EUROPAEA L.) POLYPHENOLS THROUGH BIOSORPTION ON SACCHAROMYCES CEREVISIAE	10.1016/j.indcrop.2016.02.002
IMPROVED VIABILITY OF MICROENCAPSULATED PROBIOTICS IN A FREEZE-DRIED BANANA POWDER DURING STORAGE AND UNDER SIMULATED GASTROINTESTINAL TRACT	10.1007/s12602-018-9464-1
IMPROVING CAUSALITY IN MICROBIOME RESEARCH: CAN HUMAN GENETIC EPIDEMIOLOGY HELP?	10.12688/wellcomeopenres.15628.3
IN VITRO ACTIVITY OF CADAZOLID AGAINST CLINICALLY RELEVANT CLOSTRIDIUM DIFFICILE ISOLATES AND IN AN IN VITRO GUT MODEL OF C. DIFFICILE INFECTION	10.1093/jac/dkt411
IN VITRO ANAEROBIC BIOFILMS OF HUMAN COLONIC MICROBIOTA	10.1016/j.mimet.2010.09.020
IN VITRO AND IN VIVO ACTIVITY OF NEW STRAINS OF BACILLUS SUBTILIS AGAINST ESBL-PRODUCING ESCHERICHIA COLI: AN EXPERIMENTAL STUDY	10.1111/jam.15329
IN VITRO AND IN VIVO ASSESSMENT OF INTRAINTESTINAL BACTERIOTHERAPY IN CHRONIC KIDNEY DISEASE	10.1097/01.mat.0000191345.45735.00
IN VITRO AND IN VIVO EROSION OF TWO DIFFERENT HYDROPHILIC GEL MATRIX TABLETS	10.1016/S0939-6411(98)00002-2
IN VITRO AND IN VIVO METABOLISM OF CISTANCHE TUBULOSA EXTRACT IN NORMAL AND CHRONIC UNPREDICTABLE STRESS-INDUCED DEPRESSIVE RATS	10.1016/j.jchromb.2019.121728
IN VITRO AND IN VIVO SURVIVAL AND TRANSIT TOLERANCE OF POTENTIALLY PROBIOTIC STRAINS CARRIED BY ARTICHOKES IN THE GASTROINTESTINAL TRACT	10.1128/AEM.72.4.3042-3045.2006
IN VITRO APPROACHES TO ASSESS THE EFFECTS OF ACAI (EUTERPE OLERACEA) DIGESTION ON POLYPHENOL AVAILABILITY AND THE SUBSEQUENT IMPACT ON THE FAECAL MICROBIOTA	10.1016/j.foodchem.2017.04.164
IN VITRO ASSESSMENT OF ANTIMICROBIAL RESISTANCE DISSEMINATION DYNAMICS DURING MULTIDRUG-RESISTANT-BACTERIUM INVASION EVENTS BY USING A CONTINUOUS-CULTURE DEVICE	10.1128/AEM.02659-20
IN VITRO ASSESSMENT OF IRON AVAILABILITY FROM COMMERCIAL YOUNG CHILD FORMULAE SUPPLEMENTED WITH PREBIOTICS	10.1007/s00394-016-1353-3
IN VITRO ASSESSMENT OF PREBIOTIC ACTIVITY	10.1007/978-1-0716-1274-3_17
IN VITRO ASSESSMENT OF THE BIOACCESSIBILITY OF BROMINATED FLAME RETARDANTS IN INDOOR DUST USING A COLON EXTENDED MODEL OF THE HUMAN GASTROINTESTINAL TRACT	10.1039/c2em30690e
IN VITRO BENEFICIAL EFFECTS OF STREPTOCOCCUS DENTISANI AS POTENTIAL ORAL PROBIOTIC FOR PERIODONTAL DISEASES	10.1002/JPER.18-0751
IN VITRO BIOACCESSIBILITY AND GUT BIOTRANSFORMATION OF POLYPHENOLS PRESENT IN THE WATER-INSOLUBLE COCOA FRACTION	10.1002/mnfr.201000360

IN VITRO CHARACTERISATION OF THE FERMENTATION PROFILE AND PREBIOTIC CAPACITY OF GOLD FLESHED KIWIFRUIT	10.3920/BM2015.0006
IN VITRO CHARACTERIZATION OF THE IMPACT OF DIFFERENT SUBSTRATES ON METABOLITE PRODUCTION, ENERGY EXTRACTION AND COMPOSITION OF GUT MICROBIOTA FROM LEAN AND OBESE SUBJECTS	10.1371/journal.pone.0113864
IN VITRO CHEMOPREVENTIVE PROPERTIES OF PEPTIDES RELEASED FROM QUINOA (CHENOPODIUM QUINOA WILLD.) PROTEIN UNDER SIMULATED GASTROINTESTINAL DIGESTION	10.1016/j.foodres.2017.11.036
IN VITRO COLON FERMENTATION OF SOLUBLE ARABINOXYLAN IS MODIFIED THROUGH MILLING AND EXTRUSION	10.3389/fnut.2021.707763
IN VITRO COLONIC FERMENTATION OF A PLANT STEROL-ENRICHED BEVERAGE IN A DYNAMIC-COLONIC GASTROINTESTINAL DIGESTER	10.1016/j.lwt.2021.111273
IN VITRO COLONIC FERMENTATION OF MEXICAN ”TACO” FROM CORN-TORTILLA AND BLACK BEANS IN A SIMULATOR OF HUMAN MICROBIAL ECOSYSTEM (SHIME®) SYSTEM	10.1016/j.foodres.2018.05.072
IN VITRO COLONISATION OF THE DISTAL COLON BY AKKERMANSIA MUCINIPHILA IS LARGELY MUCIN AND PH DEPENDENT	10.3920/BM2016.0013
IN VITRO CONTINUOUS FERMENTATION MODEL (POLYFERMS) OF THE SWINE PROXIMAL COLON FOR SIMULTANEOUS TESTING ON THE SAME GUT MICROBIOTA	10.1371/journal.pone.0094123
IN VITRO DETERMINATION OF PREBIOTIC PROPERTIES OF OLIGOSACCHARIDES DERIVED FROM AN ORANGE JUICE MANUFACTURING BY-PRODUCT STREAM	10.1128/AEM.71.12.8383-8389.2005
IN VITRO DIGESTIBILITY AND PREBIOTIC POTENTIAL OF CURDLAN $(1 \rightarrow 3)$-B-D-GLUCAN OLIGOSACCHARIDES IN LACTOBACILLUS SPECIES	10.1016/j.carbpol.2018.01.085
IN VITRO DIGESTION AND FECAL FERMENTATION BEHAVIORS OF A PECTIC POLYSACCHARIDE FROM OKRA (ABELMOSCHUS ESCULENTUS) AND ITS IMPACTS ON HUMAN GUT MICROBIOTA	10.1016/j.foodhyd.2020.106577
IN VITRO DIGESTION AND FECAL FERMENTATION OF HIGHLY RESISTANT STARCH RICE AND ITS EFFECT ON THE GUT MICROBIOTA	10.1016/j.foodchem.2021.130095
IN VITRO DIGESTION AND FERMENTATION BY HUMAN FECAL MICROBIOTA OF POLYSACCHARIDES FROM FLAXSEED	10.3390/molecules25194354
IN VITRO DIGESTION AND FERMENTATION METHODS, INCLUDING GAS PRODUCTION TECHNIQUES, AS APPLIED TO NUTRITIVE EVALUATION OF FOODS IN THE HINDGUT OF HUMANS AND OTHER SIMPLE-STOMACHED ANIMALS	10.1016/j.anifeedsci.2005.04.021
IN VITRO DIGESTION AND FERMENTATION OF 5-FORMYL-AMINOSAILCYLATE-INULIN: A POTENTIAL PRODRUG OF 5-AMINOSALICYLIC ACID	10.1016/j.bcdf.2013.08.001
IN VITRO DIGESTION AND FERMENTATION OF MICROENCAPSULATED TRIBUTYRIN FOR THE DELIVERY OF BUTYRATE	10.1111/1750-3841.13725
IN VITRO DIGESTION AND FERMENTATION OF RELEASED EXOPOLYSACCHARIDES (R-EPS) FROM LACTOBACILLUS DELBRUECKII SSP. BULGARICUS SRFM-1	10.1016/j.carbpol.2019.115593
IN VITRO DIGESTION AND FERMENTATION OF SIALYLLACTOSES BY INFANT GUT MICROFLORA	10.1016/j.jff.2015.12.002
IN VITRO DIGESTION AND FERMENTATION OF THREE POLYSACCHARIDE FRACTIONS FROM LAMINARIA JAPONICA AND THEIR IMPACT ON LIPID METABOLISM-ASSOCIATED HUMAN GUT MICROBIOTA	10.1021/acs.jafc.9b00970
IN VITRO DIGESTION AND FERMENTATION PROPERTIES OF LINEAR SUGAR-BEET ARABINAN AND ITS OLIGOSACCHARIDES	10.1016/j.carbpol.2015.05.022
IN VITRO DIGESTION BY SALIVA, SIMULATED GASTRIC AND SMALL INTESTINAL JUICES AND FERMENTATION BY HUMAN FECAL MICROBIOTA OF SULFATED POLYSACCHARIDES FROM GRACILARIA RUBRA	10.1016/j.jff.2017.10.040
IN VITRO DIGESTION CHARACTERISTICS OF UNPROCESSED AND PROCESSED WHOLE GRAINS AND THEIR COMPONENTS	10.1021/jf801944a
IN VITRO DIGESTION MODELS FOR DIETARY PHENOLIC COMPOUNDS	
IN VITRO DIGESTION OF POLYSACCHARIDE INCLUDING WHEY PROTEIN ISOLATE HYDROGELS	10.1016/j.carbpol.2019.115469

IN VITRO DIGESTION OF RS4-TYPE RESISTANT WHEAT AND POTATO STARCHES, AND FERMENTATION OF INDIGESTIBLE FRACTIONS	10.1094/CCHEM-07-10-0098
IN VITRO DIGESTION OF SHORT-DOUGH BISCUITS ENRICHED IN PROTEINS AND/OR FIBRES, USING A MULTI-COMPARTMENTAL AND DYNAMIC SYSTEM (1): VISCOSITY MEASUREMENT AND PREDICTION	10.1016/j.foodchem.2015.02.125
IN VITRO DIGESTION OF STARCHES IN A DYNAMIC GASTROINTESTINAL MODEL: AN INNOVATIVE STUDY TO OPTIMIZE DIETARY MANAGEMENT OF PATIENTS WITH HEPATIC GLYCOGEN STORAGE DISEASES	10.1007/s10545-014-9763-y
IN VITRO DRUG RELEASE FROM ACETYLATED HIGH AMYLOSE STARCH-ZEIN FILMS FOR ORAL COLON-SPECIFIC DRUG DELIVERY	10.1016/j.ijpharm.2018.12.021
IN VITRO DYNAMIC GASTRIC DIGESTION OF SOYA PROTEIN/MILK PROTEIN BLENDED BEVERAGES: INFLUENCE OF PROTEIN COMPOSITION AND CO-PROCESSING	10.1039/d0fo02742a
IN VITRO EFFECTS OF PH, BILE SALTS AND ENZYMES ON THE RELEASE AND VIABILITY OF ENCAPSULATED LACTOBACILLUS PLANTARUM STRAINS IN A GASTROINTESTINAL TRACT MODEL	10.1016/j.idairyj.2010.09.006
IN VITRO EVALUATION OF DIFFERENT PREBIOTICS ON THE MODULATION OF GUT MICROBIOTA COMPOSITION AND FUNCTION IN MORBID OBESE AND NORMAL-WEIGHT SUBJECTS	10.3390/ijms21030906
IN VITRO EVALUATION OF GASTROINTESTINAL SURVIVAL OF LACTOBACILLUS AMYLOVORUS DSM 16698 ALONE AND COMBINED WITH GALACTOOLIGOSACCHARIDES, MILK AND/OR BIFIDOBACTERIUM ANIMALIS SUBSP. LACTIS BB-12	10.1016/j.ijfoodmicro.2011.06.010
IN VITRO EVALUATION OF PREBIOTIC PROPERTIES OF A COMMERCIAL ARTICHOKE INFLORESCENCE EXTRACT REVEALED BIFIDOGENIC EFFECTS	10.3390/nu12061552
IN VITRO EVALUATION OF THE ANTI-PATHOGENIC ACTIVITY OF OKOUBAKA AUBREVILLEI ON THE HUMAN GASTROINTESTINAL TRACT	10.1055/a-1404-3344
IN VITRO EVALUATION OF THE BIOACCESSIBILITY OF PHENOLIC ACIDS IN DIFFERENT WHOLE WHEATS AS POTENTIAL PREBIOTICS	10.1016/j.lwt.2018.10.071
IN VITRO EVALUATION OF THE CAPACITY OF ZEOLITE AND BENTONITE TO ADSORB AFLATOXIN B1 IN SIMULATED GASTROINTESTINAL FLUIDS	10.1007/BF03032338
IN VITRO EVALUATION OF THE KINETICS OF THE RELEASE OF PHENOLIC COMPOUNDS FROM GUAVA (PSIDIUM GUAJAVA L.) FRUIT	10.1016/j.jff.2018.02.011
IN VITRO FAECAL FERMENTATION OF NOVEL OLIGOSACCHARIDES ENZYMATICALLY SYNTHESIZED USING MICROBIAL TRANSGLYCOSIDASES ACTING ON SUCROSE	10.1016/j.jff.2015.11.032
IN VITRO FERMENTABILITY OF DEXTRAN, OLIGODEXTRAN AND MALTODEXTRIN BY HUMAN GUT BACTERIA	10.1017/S0007114500000325
IN VITRO FERMENTABILITY OF DIFFERENTLY DIGESTED RESISTANT STARCH PREPARATIONS	10.1002/mnfr.200600106
IN VITRO FERMENTATION OF A RETROGRADED MAIZE STARCH BY HEALTHY ADULT FECAL EXTRACT AND IMPACTS OF EXOGENOUS MICROORGANISMS ON THREE ACIDS PRODUCTION	10.1002/star.201200100
IN VITRO FERMENTATION OF B-GOS: IMPACT ON FAECAL BACTERIAL POPULATIONS AND METABOLIC ACTIVITY IN AUTISTIC AND NON-AUTISTIC CHILDREN	10.1093/femsec/fiw233
IN VITRO FERMENTATION OF CHEWED MANGO AND BANANA: PARTICLE SIZE, STARCH AND VASCULAR FIBRE EFFECTS	10.1039/c5fo00363f
IN VITRO FERMENTATION OF DIETARY FIBER BY HUMAN FECAL ORGANISMS	10.1016/0377-8401(89)90094-1
IN VITRO FERMENTATION OF DIGESTED MILK FAT GLOBULE MEMBRANE FROM RUMINANT MILK MODULATES PIGLET ILEAL AND CAECAL MICROBIOTA	10.3389/fnut.2020.00091
IN VITRO FERMENTATION OF GUM ACACIA-IMPACT ON THE FAECAL MICROBIOTA	10.1080/09637486.2017.1404970
IN VITRO FERMENTATION OF JUÇARA PULP (EUTERPE EDULIS) BY HUMAN COLONIC MICROBIOTA	10.1016/j.foodchem.2015.09.048
IN VITRO FERMENTATION OF LUPIN SEEDS (LUPINUS ALBUS) AND BROAD BEANS (VICIA FABA): DYNAMIC MODULATION OF THE INTESTINAL MICROBIOTA AND METABOLOMIC OUTPUT	10.1039/c5fo00675a
IN VITRO FERMENTATION OF NOVEL MICROWAVE-SYNTHESIZED NON-DIGESTIBLE OLIGOSACCHARIDES AND THEIR IMPACT ON THE COMPOSITION AND METABOLITES OF HUMAN GUT MICROBIOTA	10.1016/j.jff.2019.02.030

IN VITRO FERMENTATION OF NUTRIOSE¬Æ FB06, A WHEAT DEXTRIN SOLUBLE FIBRE, IN A CONTINUOUS CULTURE HUMAN COLONIC MODEL SYSTEM	10.1371/journal.pone.0077128
IN VITRO FERMENTATION OF OAT AND BARLEY DERIVED Œí-GLUCANS BY HUMAN FAECAL MICROBIOTA	10.1111/j.1574-6941.2008.00478.x
IN VITRO FERMENTATION OF OAT FLOURS FROM TYPICAL AND HIGH Œí-GLUCAN OAT LINES	10.1021/jf900788c
IN VITRO FERMENTATION OF POLYSACCHARIDES FROM ALOE VERA AND THE EVALUATION OF ANTIOXIDANT ACTIVITY AND PRODUCTION OF SHORT CHAIN FATTY ACIDS	10.3390/molecules24193605
IN VITRO FERMENTATION OF PREBIOTICS BY LACTOBACILLUS PLANTARUM CFR 2194: SELECTIVITY, VIABILITY AND EFFECT OF METABOLITES ON Œí-GLUCURONIDASE ACTIVITY	10.1007/s11274-011-0887-z
IN VITRO FERMENTATION OF RAFFINOSE TO UNRAVEL ITS POTENTIAL AS PREBIOTIC INGREDIENT	10.1016/j.lwt.2020.109322
IN VITRO FERMENTATION OF SHEEP AND COW MILK USING INFANT FECAL BACTERIA	10.3390/nu12061802
IN VITRO FERMENTATION OF SIX KINDS OF EDIBLE MUSHROOMS AND ITS EFFECTS ON FECAL MICROBIOTA COMPOSITION	10.1016/j.lwt.2018.06.012
IN VITRO FERMENTATION OF VARIOUS FIBER AND STARCH SOURCES BY PIG FECAL INOCULA	10.2527/2004.8292615x
IN VITRO FERMENTATION OF XYLOOLIGOSACCHARIDES PRODUCED FROM MISCANTHUS X GIGANTEUS BY HUMAN FECAL MICROBIOTA	10.1021/acs.jafc.5b04618
IN VITRO FERMENTED NUTS EXHIBIT CHEMOPREVENTIVE EFFECTS IN HT29 COLON CANCER CELLS	10.1017/S0007114511006647
IN VITRO GASTROINTESTINAL BIOTRANSFORMATION AND CHARACTERIZATION OF A DESMODIUM ADSCENDENS DECOCTION: THE FIRST STEP IN UNRAVELLING ITS BEHAVIOUR IN THE HUMAN BODY	10.1111/jphp.12978
IN VITRO GASTROINTESTINAL DIGESTION AND COLONIC CATABOLISM OF MANGO (MANGIFERA INDICA L.) PULP POLYPHENOLS	10.3390/foods9121836
IN VITRO GASTROINTESTINAL DIGESTION AND COLONIC FERMENTATION OF PHENOLIC COMPOUNDS IN UV-C IRRADIATED PINEAPPLE (ANANAS COMOSUS) SNACK-BARS	10.1016/j.lwt.2020.110636
IN VITRO GASTROINTESTINAL DIGESTION AND FECAL FERMENTATION REVEAL THE EFFECT OF DIFFERENT ENCAPSULATION MATERIALS ON THE RELEASE, DEGRADATION AND MODULATION OF GUT MICROBIOTA OF BLUEBERRY ANTHOCYANIN EXTRACT	10.1016/j.foodres.2020.109098
IN VITRO GASTROINTESTINAL DIGESTION AND FERMENTATION PROPERTIES OF GANODERMA LUCIDUM SPORE POWDERS AND THEIR EXTRACTS	10.1016/j.lwt.2020.110235
IN VITRO GASTROINTESTINAL DIGESTION IMPACT ON STABILITY, BIOACCESSIBILITY AND ANTIOXIDANT ACTIVITY OF POLYPHENOLS FROM WILD AND COMMERCIAL BLACKBERRIES (RUBUS SPP.)	10.1039/d1fo00986a
IN VITRO GASTROINTESTINAL DIGESTION OF A PEANUT, SOYBEAN, GUAVA AND BEET BEVERAGE SUPPLEMENTED WITH LACTOBACILLUS RHAMNOSUS GG	10.1016/j.fbio.2020.100623
IN VITRO GASTROINTESTINAL DIGESTION OF HIBISCUS SABDARIFFA L.: THE USE OF ITS NATURAL MATRIX TO IMPROVE THE CONCENTRATION OF PHENOLIC COMPOUNDS IN GUT	10.1016/j.lwt.2012.10.007
IN VITRO GASTROINTESTINAL DIGESTION OF MANGO BY-PRODUCT SNACKS: POTENTIAL ABSORPTION OF POLYPHENOLS AND ANTIOXIDANT CAPACITY	10.1111/ijfs.14224
IN VITRO GASTROINTESTINAL DIGESTION OF PALM OLEIN AND PALM STEARIN-IN-WATER EMULSIONS WITH DIFFERENT PHYSICAL STATES AND FAT CONTENTS	10.1021/acs.jafc.0c00212
IN VITRO GASTROINTESTINAL EVALUATION OF A JUCARA-BASED SMOOTHIE: EFFECT OF PROCESSING ON PHENOLIC COMPOUNDS BIOACCESSIBILITY	10.1007/s13197-019-03974-5
IN VITRO GASTROINTESTINAL RESISTANCE OF LACTOBACILLUS ACIDOPHILUS IN SOME DAIRY PRODUCTS	10.1007/s42770-021-00590-4
IN VITRO HUMAN COLONIC FERMENTATION OF INDIGESTIBLE FRACTION ISOLATED FROM LUNCH MENUS: IMPACT ON THE GUT METABOLITES AND ANTIOXIDANT CAPACITY	10.1080/09637486.2017.1416458
IN VITRO INFANT FAECAL FERMENTATION OF LOW VISCOSITY BARLEY Œí-GLUCAN AND ITS ACID HYDROLYZED DERIVATIVES: EVALUATION OF THEIR POTENTIAL AS NOVEL PREBIOTICS	10.3390/molecules24050828

IN VITRO INTERACTIONS OF DIETARY FIBRE ENRICHED FOOD INGREDIENTS WITH PRIMARY AND SECONDARY BILE ACIDS	10.3390/nu11061424
IN VITRO MAINTENANCE OF A HUMAN PROXIMAL COLON MICROBIOTA USING THE CONTINUOUS FERMENTATION SYSTEM P-ECSIM	10.1007/s00253-011-3462-5
IN VITRO METHOD FOR QUANTIFICATION OF THE FERMENTATION OF STARCH BY HUMAN FAECAL BACTERIA	10.1002/(sici)1097-0010(199606)71:2<209::aid-jsfa571>3.3.co;2-w
IN VITRO METHOD TO ASSESS SOIL ARSENIC METABOLISM BY HUMAN GUT MICROBIOTA: ARSENIC SPECIATION AND DISTRIBUTION	10.1021/acs.est.5b03046
IN VITRO MODEL TO ASSESS ARSENIC BIOACCESSIBILITY AND SPECIATION IN COOKED SHRIMP	10.1021/acs.jafc.7b06149
IN VITRO MODEL TO STUDY THE MODULATION OF THE MUCIN-ADHERED BACTERIAL COMMUNITY	10.1007/s00253-009-1947-2
IN VITRO MODULATION OF GUT MICROBIOTA BY WHEY PROTEIN TO PRESERVE INTESTINAL HEALTH	10.1039/c7fo00197e
IN VITRO MODULATION OF HUMAN GUT MICROBIOTA COMPOSITION AND METABOLITES BY BIFIDOBACTERIUM LONGUM BB-46 AND A CITRIC PECTIN	10.1016/j.foodres.2018.11.010
IN VITRO MODULATION OF THE HUMAN GASTROINTESTINAL MICROBIAL COMMUNITY BY PLANT-DERIVED POLYSACCHARIDE-RICH DIETARY SUPPLEMENTS	10.1016/j.ijfoodmicro.2010.02.030
IN VITRO ORAL BIOACCESSIBILITY INVESTIGATION AND HUMAN HEALTH RISK ASSESSMENT OF HEAVY METALS IN WHEAT GRAINS GROWN NEAR THE MINES IN NORTH CHINA	10.1016/j.chemosphere.2020.126522
IN VITRO PREBIOTIC EFFECTS OF MALTO-OLIGOSACCHARIDES CONTAINING WATER-SOLUBLE DIETARY FIBER	10.3390/molecules25215201
IN VITRO PROTEIN AND STARCH DIGESTION KINETICS OF INDIVIDUAL CHICKPEA CELLS: FROM STATIC TO MORE COMPLEX IN VITRO DIGESTION APPROACHES	10.1039/d1fo01123e
IN VITRO SIMULATED DIGESTION AND FECAL FERMENTATION OF POLYSACCHARIDES FROM LOQUAT LEAVES: DYNAMIC CHANGES IN PHYSICOCHEMICAL PROPERTIES AND IMPACTS ON HUMAN GUT MICROBIOTA	10.1016/j.ijbiomac.2020.11.130
IN VITRO STUDIES TOWARD THE USE OF CHITIN AS NUTRACEUTICAL: IMPACT ON THE INTESTINAL EPITHELIUM, MACROPHAGES, AND MICROBIOTA	10.1002/mnfr.202000324
IN VITRO STUDY FOR INVESTIGATING THE IMPACT OF DECREASING THE MOLECULAR WEIGHT OF OAT BRAN DIETARY FIBRE COMPONENTS ON THE BEHAVIOUR IN SMALL AND LARGE INTESTINE	10.1039/d0fo00367k
IN VITRO STUDY OF LACTOBACILLUS PARACASEI CNCM I-1518 IN HEALTHY AND CLOSTRIDIOIDES DIFFICILE COLONIZED ELDERLY GUT MICROBIOTA	10.3389/fnut.2019.00184
IN VITRO THREE-STAGE CONTINUOUS FERMENTATION OF GLUCO-OLIGOSACCHARIDES PRODUCED BY GLUCONOBACTER OXYDANS NCIMB 4943 BY THE HUMAN COLONIC MICROFLORA	
IN VITRO THREE-STAGE CONTINUOUS FERMENTATION OF WHEAT ARABINOXYLAN FRACTIONS AND INDUCTION OF HYDROLASE ACTIVITY BY THE GUT MICROFLORA	10.1016/j.ijbiomac.2007.07.017
IN VITRO, IN VIVO VALIDATION OF STIMULATORY EFFECT OF OAT INGREDIENTS ON LACTOBACILLI	10.3390/pathogens10020235
IN VIVO COMMENSAL CONTROL OF CLOSTRIDIOIDES DIFFICILE VIRULENCE	10.1016/j.chom.2021.09.007
IN-VITRO DIGESTION BY SIMULATED GASTROINTESTINAL JUICES OF LACTOBACILLUS RHAMNOSUS CULTURED WITH MULBERRY OLIGOSACCHARIDES AND SUBSEQUENT FERMENTATION WITH HUMAN FECAL INOCULA	10.1016/j.lwt.2018.11.029
IN-VITRO MODEL FOR STUDYING METHANOGENS IN HUMAN GUT MICROBIOTA	10.1016/j.anaerobe.2015.04.009
IN¬†VITRO STUDY OF SOIL ARSENIC RELEASE BY HUMAN GUT MICROBIOTA AND ITS INTESTINAL ABSORPTION BY CACO-2 CELLS	10.1016/j.chemosphere.2016.10.091
INACTIVATION OF BACILLUS CEREUS VEGETATIVE CELLS BY GASTRIC ACID AND BILE DURING IN VITRO GASTROINTESTINAL TRANSIT	10.1186/1757-4749-4-11
INCLUSION OF SMALL INTESTINAL ABSORPTION AND SIMULATED MUCOSAL SURFACES FURTHER IMPROVE THE MUCOSAL SIMULATOR OF THE CANINE INTESTINAL MICROBIAL ECOSYSTEM (M-SCIME™)	10.1016/j.rvsc.2021.08.011

INCORPORATING A MUCOSAL ENVIRONMENT IN A DYNAMIC GUT MODEL RESULTS IN A MORE REPRESENTATIVE COLONIZATION BY LACTOBACILLI	10.1111/j.1751-7915.2011.00308.x
INCORPORATION OF ANTHOCYANIN-RICH RICEBERRY RICE IN YOGURTS: EFFECT ON PHYSICOCHEMICAL PROPERTIES, ANTIOXIDANT ACTIVITY AND IN VITRO GASTROINTESTINAL DIGESTION	10.1016/j.lwt.2020.109571
INCREASED EHEC SURVIVAL AND VIRULENCE GENE EXPRESSION INDICATE AN ENHANCED PATHOGENICITY UPON SIMULATED PEDIATRIC GASTROINTESTINAL CONDITIONS	10.1038/pr.2016.144
INCREASED OXIDATIVE AND NITROSATIVE REACTIONS DURING DIGESTION COULD CONTRIBUTE TO THE ASSOCIATION BETWEEN WELL-DONE RED MEAT CONSUMPTION AND COLORECTAL CANCER	10.1016/j.foodchem.2015.04.029
INCREASED PROTEIN DIGESTIBILITY OF BEEF WITH AGING IN AN INFANT IN VITRO DIGESTION MODEL	10.1016/j.meatsci.2020.108210
INCREASING THE ECONOMIC VALUE OF LIGNOCELLULOSIC STILLAGE THROUGH MEDIUM-CHAIN FATTY ACID PRODUCTION	10.1186/s13068-018-1193-x
INDUCTION OF ACID RESISTANCE IN BIFIDOBACTERIUM: A MECHANISM FOR IMPROVING DESIRABLE TRAITS OF POTENTIALLY PROBIOTIC STRAINS	10.1111/j.1365-2672.2007.03342.x
INDUCTION OF HEPATIC AND ENDOTHELIAL DIFFERENTIATION BY PERFUSION IN A THREE-DIMENSIONAL CELL CULTURE MODEL OF HUMAN FETAL LIVER	10.1089/ten.tec.2014.0453
INFLUENCE OF A SYNBIOTIC MIXTURE CONSISTING OF LACTOBACILLUS ACIDOPHILUS 74-2 AND A FRUCTOOLIGOSACCHARIDE PREPARATION ON THE MICROBIAL ECOLOGY SUSTAINED IN A SIMULATION OF THE HUMAN INTESTINAL MICROBIAL ECOSYSTEM (SHIME REACTOR)	10.1007/s002530050011
INFLUENCE OF BACILLUS SUBTILIS C-3102 ON MICROBIOTA IN A DYNAMIC IN VITRO MODEL OF THE GASTROINTESTINAL TRACT SIMULATING HUMAN CONDITIONS	10.3920/BM2012.0016
INFLUENCE OF CULTIVATION PH ON COMPOSITION, DIVERSITY, AND METABOLIC PRODUCTION IN AN IN VITRO HUMAN INTESTINAL MICROBIOTA	10.3390/FERMENTATION7030156
INFLUENCE OF DICLOFENAC ON ACTIVATED SLUDGE BACTERIAL COMMUNITIES IN FED-BATCH REACTORS	10.17113/ftb.58.04.20.6424
INFLUENCE OF ENCAPSULATED PROBIOTICS COMBINED WITH PRESSURIZED LONGAN JUICE ON COLON MICROFLORA AND THEIR METABOLIC ACTIVITIES ON THE EXPOSURE TO SIMULATED DYNAMIC GASTROINTESTINAL TRACT	10.1016/j.foodres.2012.07.033
INFLUENCE OF ENZYME-RESISTANT FRACTION OF SORGHUM (SORGHUM BICOLOR L.) FLOUR ON GUT MICROFLORA COMPOSITION, SHORT CHAIN FATTY ACID PRODUCTION AND TOXIC SUBSTANCE METABOLISM	
INFLUENCE OF FERMENTATION BY LACTIC ACID BACTERIA AND IN VITRO DIGESTION ON THE BIOTRANSFORMATIONS OF BLUEBERRY JUICE PHENOLICS	10.1016/j.foodcont.2021.108603
INFLUENCE OF FERMENTATION OF PASTEURISED PAPAYA PUREE WITH DIFFERENT LACTIC ACID BACTERIAL STRAINS ON QUALITY AND BIOACCESSIBILITY OF PHENOLIC COMPOUNDS DURING IN VITRO DIGESTION	10.3390/foods10050962
INFLUENCE OF FERMENTATION WITH DIFFERENT LACTIC ACID BACTERIA AND IN VITRO DIGESTION ON THE BIOTRANSFORMATION OF PHENOLIC COMPOUNDS IN FERMENTED POMEGRANATE JUICES	10.1021/acs.jafc.6b04854
INFLUENCE OF FOOD MATRIX ON THE BIOACCESSIBILITY OF FRUIT POLYPHENOLIC COMPOUNDS	10.1021/acs.jafc.9b07680
INFLUENCE OF FUNGICIDE RESIDUES AND IN VITRO GASTROINTESTINAL DIGESTION ON TOTAL ANTIOXIDANT CAPACITY AND PHENOLIC FRACTION OF GRACIANO AND TEMPRANILLO RED WINES	10.1080/03601234.2019.1652073
INFLUENCE OF GASTROINTESTINAL TRACT ON METABOLISM OF BISPHENOL A AS DETERMINED BY IN VITRO SIMULATED SYSTEM	10.1016/j.jhazmat.2018.05.011
INFLUENCE OF IN VITRO GASTROINTESTINAL DIGESTION AND PROBIOTIC FERMENTATION ON THE BIOACCESSIBILITY OF GALLIC ACID AND ON THE ANTIOXIDANT POTENTIAL OF BRAZILIAN FRUIT RESIDUES	10.1016/j.lwt.2021.112436
INFLUENCE OF INULIN RICH CARBOHYDRATES FROM JERUSALEM ARTICHOKE (HELIANTHUS TUBEROSUS L.) TUBERS ON PROBIOTIC PROPERTIES OF LACTOBACILLUS STRAINS	10.1016/j.lwt.2018.11.074
INFLUENCE OF POLYPHENOL RICH SEABUCKTHORN BERRIES JUICE ON RELEASE OF POLYPHENOLS AND COLONIC MICROBIOTA ON EXPOSURE TO SIMULATED HUMAN DIGESTION MODEL	10.1016/j.foodres.2018.05.045

INFLUENCE OF PROBIOTIC STRAINS ADDED TO COTTAGE CHEESE ON GENERATION OF POTENTIALLY ANTIOXIDANT PEPTIDES, ANTI-LISTERIAL ACTIVITY, AND SURVIVAL OF PROBIOTIC MICROORGANISMS IN SIMULATED GASTROINTESTINAL CONDITIONS	10.1016/j.idairyj.2013.04.005
INFLUENCE OF THE CO-EXPOSURE OF MICROPLASTICS AND TETRABROMOBISPHENOL A ON HUMAN GUT: SIMULATION IN VITRO WITH HUMAN CELL CACO-2 AND GUT MICROBIOTA	10.1016/j.scitotenv.2021.146264
INFLUENCE OF THE STATIC MAGNETIC FIELD ON CELL RESPONSE IN A MINIATURIZED OPTICALLY ACCESSIBLE BIOREACTOR FOR 3D CELL CULTURE	10.1007/s10544-019-0387-8
INFLUENCE OF VISCOSITY ON THE GROWTH OF HUMAN GUT MICROBIOTA	10.1016/j.foodhyd.2017.09.031
INFLUENCES OF EXOGENOUS PROBIOTICS AND TEA POLYPHENOLS ON THE PRODUCTION OF THREE ACIDS DURING THE SIMULATED COLONIC FERMENTATION OF MAIZE RESISTANT STARCH	10.1007/s13197-014-1662-6
INFLUENCES OF STRUCTURES OF GALACTOOLIGOSACCHARIDES AND FRUCTOOLIGOSACCHARIDES ON THE FERMENTATION IN VITRO BY HUMAN INTESTINAL MICROBIOTA	10.1016/j.jff.2014.12.044
INFOGEST STATIC IN VITRO SIMULATION OF GASTROINTESTINAL FOOD DIGESTION	10.1038/s41596-018-0119-1
INHIBITING GROWTH OF CLOSTRIDIOIDES DIFFICILE BY RESTORING VALERATE, PRODUCED BY THE INTESTINAL MICROBIOTA	10.1053/j.gastro.2018.07.014
INHIBITION OF ORAL PATHOGENS ADHESION TO HUMAN GINGIVAL FIBROBLASTS BY WINE POLYPHENOLS ALONE AND IN COMBINATION WITH AN ORAL PROBIOTIC	10.1021/acs.jafc.7b05466
INHIBITORY EFFECT OF BACTERIOCIN-PRODUCING LACTOBACILLUS BREVIS DF01 AND PEDIOCOCCUS ACIDILACTICI K10 ISOLATED FROM KIMCHI ON ENTEROPATHOGENIC BACTERIAL ADHESION	10.1016/j.fbio.2019.100425
INSIGHT OF STABILITY OF PROCYANIDINS IN FREE AND LIPOSOMAL FORM UNDER AN IN VITRO DIGESTION MODEL: STUDY OF BIOACCESSIBILITY, KINETIC RELEASE PROFILE, DEGRADATION, AND ANTIOXIDANT ACTIVITY	10.1021/acs.jafc.9b00351
INSIGHT ON THE BACTERIAL ECOLOGY IN MEMBRANE BIOREACTOR: OPERATIONAL CONDITIONS EFFECT OVER DOMINANT ECOLOGICAL PLAYERS	10.1002/aic.16456
INSIGHTS INTO BREAD MELANOIDINS: FATE IN THE UPPER DIGESTIVE TRACT AND IMPACT ON THE GUT MICROBIOTA USING IN VITRO SYSTEMS	10.1039/c5fo00836k
INSOLUBLE DIETARY FIBER FROM SOY HULLS REGULATES THE GUT MICROBIOTA IN VITRO AND INCREASES THE ABUNDANCE OF BIFIDOBACTERIALES AND LACTOBACILLALES	10.1007/s13197-019-04041-9
INTAKE AND BIOACCESSIBILITY OF TOTAL POLYPHENOLS IN A WHOLE DIET	10.1016/j.foodchem.2006.02.006
INTEGRATED 'OMICS ANALYSIS FOR STUDYING THE MICROBIAL COMMUNITY RESPONSE TO A PH PERTURBATION OF A CELLULOSE-DEGRADING BIOREACTOR CULTURE	10.1111/1574-6941.12435
INTEGRATED CONTINUOUS BIOPROCESS DEVELOPMENT FOR ACE-INHIBITORY PEPTIDE PRODUCTION BY LACTOBACILLUS HELVETICUS STRAINS IN MEMBRANE BIOREACTOR	10.3389/fbioe.2020.585815
INTEGRATIVE ANALYSIS OF MICROBIOME AND METABOLOME IN RATS WITH GEST-AID PLUS ORAL LIQUID SUPPLEMENTATION REVEALS MECHANISM OF ITS HEALTHCARE FUNCTION	10.1093/fqsafe/fyab010
INTEGRATIVE AND QUANTITATIVE BIOENERGETICS: DESIGN OF A STUDY TO ASSESS THE IMPACT OF THE GUT MICROBIOME ON HOST ENERGY BALANCE	10.1016/j.conctc.2020.100646
INTERACTIONS BETWEEN BLACKCURRANT POLYPHENOLS AND FOOD MACRONUTRIENTS IN MODEL SYSTEMS: IN VITRO DIGESTION STUDIES	10.3390/foods10040847
INTERACTIONS BETWEEN FECAL BACTERIA, BILE ACIDS AND COMPONENTS OF TOMATO POMACE	10.1007/s10068-018-0527-6
INTERACTIONS BETWEEN GUT MICROBIOTA AND SOY HULL POLYSACCHARIDES REGULATE THE AIR-LIQUID INTERFACIAL ACTIVITY	10.1016/j.foodhyd.2021.106704
INTERACTIONS OF A LIGNIN-RICH FRACTION FROM BREWER'S SPENT GRAIN WITH GUT MICROBIOTA IN VITRO	10.1021/jf401738x

INTERACTIONS OF BLACK TEA POLYPHENOLS WITH HUMAN GUT MICROBIOTA: IMPLICATIONS FOR GUT AND CARDIOVASCULAR HEALTH1-4	10.3945/ajcn.113.058263
INTERACTIONS OF SALMONELLA ENTERICA SUBSPECIES ENTERICA SEROVAR TYPHIMURIUM WITH GUT BACTERIA	10.1016/j.anaerobe.2015.02.006
INTERINDIVIDUAL DIFFERENCES IN RESPONSE TO TREATMENT WITH BUTYRATE-PRODUCING BUTYRICICOCCUS PULLICAECORUM 25-3T STUDIED IN AN IN VITRO GUT MODEL	10.1093/femsec/fiv054
INTERINDIVIDUAL VARIABILITY OF SOIL ARSENIC METABOLISM BY HUMAN GUT MICROBIOTA USING SHIME MODEL	10.1016/j.chemosphere.2017.06.018
INTERPENETRATING POLYMER NETWORK HYDROGELS OF SOY PROTEIN ISOLATE AND SUGAR BEET PECTIN AS A POTENTIAL CARRIER FOR PROBIOTICS	10.1016/j.foodhyd.2020.106453
INTESTINAL BACTERIA ARE INVOLVED IN RADIX GLYCYRRHIZAE AND RADIX EUPHORBIAE PEKINENSIS INCOMPATIBILITY	10.1016/j.jep.2021.113839
INTESTINAL MICROBIOTA AS A TETRAHYDROBIOPTERIN EXOGENOUS SOURCE IN HPH-1 MICE	10.1038/srep39854
INTRACELLULAR SURVIVAL OF ENTERIC BACTERIA IN CULTURED HUMAN ENTEROCYTES	10.1097/00024382-199607000-00007
INTRODUCING INSOLUBLE WHEAT BRAN AS A GUT MICROBIOTA NICHE IN AN IN VITRO DYNAMIC GUT MODEL STIMULATES PROPIONATE AND BUTYRATE PRODUCTION AND INDUCES COLON REGION SPECIFIC SHIFTS IN THE LUMINAL AND MUCOSAL MICROBIAL COMMUNITY	10.1111/1462-2920.14381
INULIN-TYPE FRUCTAN FERMENTATION BY BIFIDOBACTERIA DEPENDS ON THE STRAIN RATHER THAN THE SPECIES AND REGION IN THE HUMAN INTESTINE	10.1007/s00253-016-7351-9
INULIN-TYPE FRUCTANS OF LONGER DEGREE OF POLYMERIZATION EXERT MORE PRONOUNCED IN VITRO PREBIOTIC EFFECTS	10.1111/j.1365-2672.2006.03084.x
INVESTIGATING THE EFFECT OF IN VITRO GASTROINTESTINAL DIGESTION ON THE STABILITY, BIOACCESSIBILITY, AND BIOLOGICAL ACTIVITIES OF BAOBAB (ADANSONIA DIGITATA) FRUIT POLYPHENOLICS	10.1016/j.lwt.2021.111348
INVESTIGATING THE USE OF BACTERIOPHAGES AS A NEW DECOLONIZATION STRATEGY FOR INTESTINAL CARRIAGE OF CTX-M-15-PRODUCING ST131 ESCHERICHIA COLI: AN IN VITRO CONTINUOUS CULTURE SYSTEM MODEL	10.1016/j.jgar.2020.05.018
INVESTIGATING UNSATURATED FAT, MONENSIN, OR BROMOETHANESULFONATE IN CONTINUOUS CULTURES RETAINING RUMINAL PROTOZOA. II. INTERACTION OF TREATMENT AND PRESENCE OF PROTOZOA ON PROKARYOTIC COMMUNITIES	10.3168/jds.2008-1437
INVESTIGATION INTO THE STABILITY AND CULTURABILITY OF CHINESE ENTEROTYPES	10.1038/s41598-017-08478-w
INVESTIGATION OF BIOACCESSIBILITY OF CU, FE, MN, AND ZN IN MARKET VEGETABLES IN THE COLON USING PBET COMBINED WITH SHIME	10.1038/s41598-017-17901-1
INVESTIGATION OF FORMATION OF WELL-KNOWN AGES PRECURSORS IN COOKIES USING AN IN VITRO SIMULATED GASTROINTESTINAL DIGESTIVE SYSTEM	10.1016/j.foodchem.2021.131451
INVESTIGATION OF GENIPIN CROSS-LINKED MICROCAPSULE FOR ORAL DELIVERY OF LIVE BACTERIAL CELLS AND OTHER BIOTHERAPEUTICS: PREPARATION AND IN VITRO ANALYSIS IN SIMULATED HUMAN GASTROINTESTINAL MODEL	10.1155/2010/985137
INVESTIGATION OF THE EFFECT OF THE ADSORBENT DAV131A ON THE PROPENSITY OF MOXIFLOXACIN TO INDUCE SIMULATED CLOSTRIDIOIDES (CLOSTRIDIUM) DIFFICILE INFECTION (CDI) IN AN IN VITRO HUMAN GUT MODEL	10.1093/JAC/DKAA062
INVITRO FERMENTATION OF POLYSACCHARIDE FROM THE SEEDS OF PLANTAGO ASIATICA L. BY HUMAN FECAL MICROBIOTA	10.1016/j.foodhyd.2013.04.006
IRON MODULATES BUTYRATE PRODUCTION BY A CHILD GUT MICROBIOTA IN VITRO	10.1128/mBio.01453-15

IRON-RELATED TRANSCRIPTOMIC VARIATIONS IN CACO-2 CELLS, AN IN VITRO MODEL OF INTESTINAL ABSORPTIVE CELLS	10.1152/physiolgenomics.00297.2005
IS THERE AN IMPACT OF THE DAIRY MATRIX ON THE SURVIVAL OF LACTOBACILLUS CASEI LC-1 DURING SHELF LIFE AND SIMULATED GASTROINTESTINAL CONDITIONS?	10.1002/jsfa.9988
ISOFLAVONE AGLYCONES ENRICHMENT IN SOYBEAN SOURDOUGH BREAD FERMENTED BY LACTIC ACID BACTERIA STRAINS ISOLATED FROM TRADITIONAL QU STARTERS: EFFECTS ON IN VITRO GASTROINTESTINAL DIGESTION, NUTRITIONAL, AND BAKING PROPERTIES	10.1002/cche.10116
ISOFLAVONE CONVERSION OF BLACK SOYBEAN BY IMMOBILIZED RHIZOPUS SPP	10.1080/08905436.2010.524459
ISOLATION AND CHARACTERISATION OF ΦCRASS002, A CRASS-LIKE PHAGE FROM THE HUMAN GUT THAT INFECTS BACTEROIDES XYLANISOLVENS	10.1186/s40168-021-01036-7
ISOLATION OF PROBIOTIC PILIATED LACTOBACILLUS RHAMNOSUS STRAINS FROM HUMAN FECAL MICROBIOTA USING SPAA ANTISERUM-BASED COLONY IMMUNOBLOTTING	10.4014/jmb.1705.05055
ISOLATION, IDENTIFICATION, AND EVALUATION OF NOVEL PROBIOTIC STRAINS ISOLATED FROM FECES OF BREAST-FED INFANTS	
KEEPING CANDIDA COMMENSAL: HOW LACTOBACILLI ANTAGONIZE PATHOGENICITY OF CANDIDA ALBICANS IN AN IN VITRO GUT MODEL	10.1242/dmm.039719
KEY MICROBES AND METABOLIC POTENTIALS CONTRIBUTING TO CYANIDE BIODEGRADATION IN STIRRED-TANK BIOREACTORS TREATING GOLD MINING EFFLUENT	10.1080/08827508.2019.1575213
KINETIC MODELING OF THE ENZYMATIC SYNTHESIS OF GALACTO-OLIGOSACCHARIDES: DESCRIBING GALACTOBIOSE FORMATION	10.1016/j.fbp.2021.02.004
KINETIC MODELLING OF IN VITRO CELL-BASED ASSAYS TO CHARACTERIZE NON-SPECIFIC BINDINGS AND ADME PROCESSES IN A STATIC AND A PERFUSED FLUIDIC SYSTEM	10.1016/j.toxlet.2011.06.021
LACTATE IS MAINLY FERMENTED TO BUTYRATE BY HUMAN INTESTINAL MICROFLORAS BUT INTER-INDIVIDUAL VARIATION IS EVIDENT	10.1111/j.1365-2672.2005.02605.x
LACTIC ACID BACTERIA ISOLATED FROM EUROPEAN BADGERS (MELES MELES) REDUCE THE VIABILITY AND SURVIVAL OF BACILLUS CALMETTE-GUERIN (BCG) VACCINE AND INFLUENCE THE IMMUNE RESPONSE TO BCG IN A HUMAN MACROPHAGE MODEL	10.1186/s12866-018-1210-z
LACTIC ACID BACTERIAL FERMENTATION MODIFIED PHENOLIC COMPOSITION IN TEA EXTRACTS AND ENHANCED THEIR ANTIOXIDANT ACTIVITY AND CELLULAR UPTAKE OF PHENOLIC COMPOUNDS FOLLOWING IN VITRO DIGESTION	10.1016/j.jff.2015.10.033
LACTICASEIBACILLUS RHAMNOSUS GG AND SACCHAROMYCES CEREVISIAE BOULARDII SUPPLEMENTATION EXERT PROTECTIVE EFFECTS ON HUMAN GUT MICROBIOME FOLLOWING ANTIBIOTIC ADMINISTRATION IN VITRO	10.3920/BM2020.0180
LACTOBACILLUS ACIDOPHILUS CRL 1014 IMPROVED " GUT HEALTH" IN THE SHIME¬Æ REACTOR	10.1186/1471-230X-13-100
LACTOBACILLUS CASEI MYL01 MODULATES THE PROINFLAMMATORY STATE INDUCED BY ETHANOL IN AN IN VITRO MODEL	10.3168/jds.2013-7514
LACTOBACILLUS GASSERI GASSER AM63T DEGRADES OXALATE IN A MULTISTAGE CONTINUOUS CULTURE SIMULATOR OF THE HUMAN COLONIC MICROBIOTA	10.1111/j.1574-6941.2007.00327.x
LACTOBACILLUS PLANTARUM IFPL935 FAVORS THE INITIAL METABOLISM OF RED WINE POLYPHENOLS WHEN ADDED TO A COLONIC MICROBIOTA	10.1021/jf402816r
LACTOBACILLUS PLANTARUM IFPL935 IMPACTS COLONIC METABOLISM IN A SIMULATOR OF THE HUMAN GUT MICROBIOTA DURING FEEDING WITH RED WINE POLYPHENOLS	10.1007/s00253-014-5744-1
LACTOBACILLUS PLANTARUM ISOLATES FROM HOMEMADE DAHI AS A POTENTIAL PROBIOTIC WITH IN VITRO A-AMYLASE INHIBITORY ACTIVITY	10.22207/JPAM.14.2.12
LACTOBACILLUS PLANTARUM-MEDIATED REGULATION OF DIETARY ALUMINUM INDUCES CHANGES IN THE HUMAN GUT MICROBIOTA: AN IN VITRO COLONIC FERMENTATION STUDY	10.1007/s12602-020-09677-0

LACTOBACILLUS SPP. IMPAIR THE ABILITY OF LISTERIA MONOCYTOGENES FBUNT TO ADHERE TO AND INVADE CACO-2 CELLS	10.1007/s10529-018-2572-x
LEAD BIOACCESSIBILITY IN FARMING AND MINING SOILS: THE INFLUENCE OF SOIL PROPERTIES, TYPES AND HUMAN GUT MICROBIOTA	10.1016/j.scitotenv.2019.135227
LEAN AND OBESE MICROBIOTA: DIFFERENCES IN IN VITRO FERMENTATION OF FOOD-BY-PRODUCTS	10.3920/BM2020.0151
LINKING PHYLOGENETIC IDENTITIES OF BACTERIA TO STARCH FERMENTATION IN AN IN VITRO MODEL OF THE LARGE INTESTINE BY RNA-BASED STABLE ISOTOPE PROBING	10.1111/j.1462-2920.2008.01815.x
LIQUID CHROMATOGRAPHY-MASS SPECTROMETRY ANALYSIS OF HYDROXYLATED POLYCYCLIC AROMATIC HYDROCARBONS, FORMED IN A SIMULATOR OF THE HUMAN GASTROINTESTINAL TRACT	10.1016/j.jchromb.2004.04.001
LISTERIA MONOCYTOGENES SURVIVAL IN RAW ATLANTIC SALMON (SALMO SALAR) FILLET UNDER IN VITRO SIMULATED GASTROINTESTINAL CONDITIONS BY CULTURE, QPCR AND PMA-QPCR DETECTION METHODS	10.1016/j.lwt.2019.03.015
LONG CHAIN ARABINOXYLANS SHIFT THE MUCOSA-ASSOCIATED MICROBIOTA IN THE PROXIMAL COLON OF THE SIMULATOR OF THE HUMAN INTESTINAL MICROBIAL ECOSYSTEM (M-SHIME)	10.1016/j.jff.2017.02.004
LOW IRON AVAILABILITY IN CONTINUOUS IN VITRO COLONIC FERMENTATIONS INDUCES STRONG DYSBIOSIS OF THE CHILD GUT MICROBIAL CONSORTIUM AND A DECREASE IN MAIN METABOLITES	10.1111/j.1574-6941.2012.01461.x
LOW PROTEIN DIGESTIBILITY OF BEEF PUREE IN INFANT IN VITRO DIGESTION MODEL	10.5851/kosfa.2019.e73
LYSOZYME-LIKE PROTEIN PRODUCED BY BIFIDOBACTERIUM LONGUM REGULATES HUMAN GUT MICROBIOTA USING IN VITRO MODELS	10.3390/molecules26216480
MAINLY DIMERS AND TRIMERS OF CHINESE BAYBERRY LEAVES PROANTHOCYANIDINS (BLPS) ARE UTILIZED BY GUT MICROBIOTA: IN VITRO DIGESTION AND FERMENTATION COUPLED WITH CACO-2 TRANSPORTATION	10.3390/molecules25010184
MAIZE BRAN PARTICLE SIZE GOVERNS THE COMMUNITY COMPOSITION AND METABOLIC OUTPUT OF HUMAN GUT MICROBIOTA IN IN VITRO FERMENTATIONS	10.3389/fmicb.2020.01009
MANIPULATION OF GUT MICROBIOTA USING ACACIA GUM POLYSACCHARIDE	10.1021/acsomega.1c00302
MASKING THE PERCEIVED ASTRINGENCY OF PROANTHOCYANIDINS IN BEVERAGES USING OXIDIZED STARCH HYDROGEL MICROENCAPSULATION	10.3390/foods9060756
MATHEMATICAL MODELLING OF CARBOHYDRATE DEGRADATION BY HUMAN COLONIC MICROBIOTA	10.1016/j.jtbi.2010.05.040
MATRIX EFFECTS ON THE STABILITY AND ANTIOXIDANT ACTIVITY OF RED CABBAGE ANTHOCYANINS UNDER SIMULATED GASTROINTESTINAL DIGESTION	10.1155/2014/365738
MEASURING NON-STEADY-STATE METABOLIC FLUXES IN STARCH-CONVERTING FAECAL MICROBIOTA IN VITRO	10.3920/BM2010.0038
MEASURING THE EFFECT OF MANKAI¬Æ (WOLFFIA GLOBOSA) ON THE GUT MICROBIOTA AND ITS METABOLIC OUTPUT USING AN IN VITRO COLON MODEL	10.1016/j.jff.2021.104597
MEMBRANE PROTEIN INSERTION INTO AND COMPATIBILITY WITH BIOMIMETIC MEMBRANES	10.1002/adbi.201700053
METABOLIC ANALYSIS OF REGIONALLY DISTINCT GUT MICROBIAL COMMUNITIES USING AN IN VITRO PLATFORM	10.1021/acs.jafc.9b05202
METABOLIC AND MICROBIAL MODULATION OF PHENOLIC COMPOUNDS FROM RASPBERRY LEAF EXTRACT UNDER IN VITRO DIGESTION AND FERMENTATION	10.1111/ijfs.15083
METABOLIC FATE OF 13C-LABELED POLYDEXTROSE AND IMPACT ON THE GUT MICROBIOME: A TRIPLE-PHASE STUDY IN A COLON SIMULATOR	10.1021/acs.jproteome.7b00683
METABOLIC FATE OF OCHRATOXIN A AS A COFFEE CONTAMINANT IN A DYNAMIC SIMULATOR OF THE HUMAN COLON	10.1016/j.foodchem.2013.05.157
METABOLIC PROFILES OF OLIGOSACCHARIDES DERIVED FROM FOUR MICROBIAL POLYSACCHARIDES BY FAECAL INOCULA FROM TYPE 2 DIABETES PATIENTS	10.1080/09637486.2021.1908964
METABOLIC TRANSFORMATIONS OF DIETARY POLYPHENOLS: COMPARISON BETWEEN IN VITRO COLONIC AND HEPATIC MODELS AND IN VIVO URINARY METABOLITES	10.1016/j.jnutbio.2016.03.007
METABOLISM OF FRUCTOOLIGOSACCHARIDES IN LACTOBACILLUS PLANTARUM ST-III VIA DIFFERENTIAL GENE TRANSCRIPTION AND ALTERATION OF CELL MEMBRANE FLUIDITY	10.1128/AEM.02426-15

METABOLISM OF MIXED HUMAN COLONIC BACTERIA IN A CONTINUOUS CULTURE MIMICKING THE HUMAN CECAL CONTENTS	10.1016/0016-5085(85)90017-4
METABOLISM OF PHENOLICS OF TETRASTIGMA HEMSLEYANUM ROOTS UNDER IN VITRO DIGESTION AND COLONIC FERMENTATION AS WELL AS THEIR IN VIVO ANTIOXIDANT ACTIVITY IN RATS	10.3390/foods10092123
METABOLISM OF THE LIGNAN MACROMOLECULE INTO ENTEROLIGNANS IN THE GASTROINTESTINAL LUMEN AS DETERMINED IN THE SIMULATOR OF THE HUMAN INTESTINAL MICROBIAL ECOSYSTEM	10.1021/jf800101s
METABOLISM OF THE SOYABEAN ISOFLAVONE GLYCOSIDE GENISTIN IN VITRO BY HUMAN GUT BACTERIA AND THE EFFECT OF PREBIOTICS	10.1079/BJN2003949
METABOLISM OF WHEAT DEXTRIN, PARTIALLY HYDROLYSED GUAR GUM AND INULIN BY BIFIDOBACTERIUM LACTIS OR LACTOBACILLUS ACIDOPHILUS IN AN IN VITRO GUT MODEL FERMENTATION SYSTEM	10.37290/ijpp2641-7197.16:22,Äì30
METABOLITE-BASED MUTUALISM BETWEEN PSEUDOMONAS AERUGINOSA PA14 AND ENTEROBACTER AEROGENES ENHANCES CURRENT GENERATION IN BIOELECTROCHEMICAL SYSTEMS	10.1039/c1ee01377g
METABOLOMIC ANALYSIS OF HUMAN FECAL MICROBIOTA: A COMPARISON OF FECES-DERIVED COMMUNITIES AND DEFINED MIXED COMMUNITIES	10.1021/pr5011247
METABOLOMIC STUDY TO EVALUATE THE TRANSFORMATIONS OF EXTRA-VIRGIN OLIVE OIL'S ANTIOXIDANT PHYTOCHEMICALS DURING IN VITRO GASTROINTESTINAL DIGESTION	10.3390/antiox9040302
METAGENOMICS SHOWS THAT LOW-ENERGY ANAEROBIC-AEROBIC TREATMENT REACTORS REDUCE ANTIBIOTIC RESISTANCE GENE LEVELS FROM DOMESTIC WASTEWATER	10.1021/es505521w
METHOD COMPARISON FOR THE DIRECT ENUMERATION OF BACTERIAL SPECIES USING A CHEMOSTAT MODEL OF THE HUMAN COLON	10.1186/s12866-019-1669-2
MICROBES INVOLVED IN DISSIMILATORY NITRATE REDUCTION IN THE HUMAN LARGE INTESTINE	10.1016/S0168-6496(99)00077-X
MICROBIAL AND COMPOSITION CHANGES DURING VERMICOMPOSTING PROCESS RESULTING FROM DECOMPOSABLE DOMESTIC WASTE, COW MANURE AND DEWATERED SLUDGE	10.4103/ijehe.ijehe_56_20
MICROBIAL BIOTRANSFORMATION OF A POLYPHENOL-RICH POTATO EXTRACT AFFECTS ANTIOXIDANT CAPACITY IN A SIMULATED GASTROINTESTINAL MODEL	10.3390/antiox7030043
MICROBIAL BIOTRANSFORMATION OF POLYPHENOLS DURING IN VITRO COLONIC FERMENTATION OF MASTICATED MANGO AND BANANA	10.1016/j.foodchem.2016.03.108
MICROBIAL COMMUNITIES IN A DYNAMIC IN VITRO MODEL FOR THE HUMAN ILEUM RESEMBLE THE HUMAN ILEAL MICROBIOTA	10.1093/femsec/fiz096
MICROBIAL COMMUNITY COMPOSITION AND DYNAMICS IN HIGH-TEMPERATURE BIOGAS REACTORS USING INDUSTRIAL BIOETHANOL WASTE AS SUBSTRATE	10.1007/s00253-014-5906-1
MICROBIAL COMMUNITY DEVELOPMENT IN A DYNAMIC GUT MODEL IS REPRODUCIBLE, COLON REGION SPECIFIC, AND SELECTIVE FOR BACTEROIDETES AND CLOSTRIDIUM CLUSTER IX	10.1128/AEM.00759-10
MICROBIAL COMPETITION IN REACTORS WITH WALL ATTACHMENT: A MATHEMATICAL COMPARISON OF CHEMOSTAT AND PLUG FLOW MODELS	10.1007/s002480000005
MICROBIAL FUEL CELL AS POWER SUPPLY FOR IMPLANTABLE MEDICAL DEVICES: A NOVEL CONFIGURATION DESIGN FOR SIMULATING COLONIC ENVIRONMENT	10.1016/j.bios.2012.10.028
MICROBIAL METABOLISM SHIFTS TOWARDS AN ADVERSE PROFILE WITH SUPPLEMENTARY IRON IN THE TIM-2 IN VITRO MODEL OF THE HUMAN COLON	10.3389/fmicb.2015.01481
MICROBIAL PRODUCTS ALTER THE EXPRESSION OF MEMBRANE-ASSOCIATED MUCIN AND ANTIMICROBIAL PEPTIDES IN A THREE-DIMENSIONAL HUMAN ENDOCERVICAL EPITHELIAL CELL MODEL	10.1095/biolreprod.112.103366
MICROBIAL TRANSGLUTAMINASE ALTERS THE IMMUNOGENIC POTENTIAL AND CROSS-REACTIVITY OF HORSE AND COW MILK PROTEINS	10.3168/jds.2019-17264
MICROBIAL VALORIZATION OF SOLID WASTES FROM A RECIRCULATING AQUACULTURE SYSTEM AND THE RELEVANT MICROBIAL FUNCTIONS	10.1016/j.aquaeng.2019.102016

MICROBIAL, PHYSICO-CHEMICAL AND SENSORY CHARACTERISTICS OF MANGO JUICE-ENRICHED PROBIOTIC DAIRY DRINKS	10.1111/1471-0307.12630
MICROBIOLOGICAL MONITORING IN THE BIODEGRADATION OF SEWAGE SLUDGE AND FOOD WASTE	10.1111/j.1365-2672.2004.02182.x
MICROBIOLOGICAL TOXICITY OF TILMICOSIN ON HUMAN COLONIC MICROFLORA IN CHEMOSTATS	10.1016/j.yrtph.2015.07.008
MICROBIOTAS FROM UC PATIENTS DISPLAY ALTERED METABOLISM AND REDUCED ABILITY OF LAB TO COLONIZE MUCUS	10.1038/srep01110
MICROENCAPSULATED BIFIDOBACTERIUM LONGUM SUBSP. INFANTIS ATCC 15697 FAVORABLY MODULATES GUT MICROBIOTA AND REDUCES CIRCULATING ENDOTOXINS IN F344 RATS	10.1155/2014/602832
MICROENCAPSULATED STARTER CULTURE DURING YOGHURT MANUFACTURING, EFFECT ON TECHNOLOGICAL FEATURES	10.1007/s11947-017-1946-8
MICROENCAPSULATION OF ANTHOCYANIN EXTRACTED FROM PURPLE FLESH CULTIVATED POTATOES BY SPRAY DRYING AND ITS EFFECTS ON IN VITRO GASTROINTESTINAL DIGESTION	10.3390/molecules25030722
MICROENCAPSULATION OF LACTOBACILLUS PLANTARUM (MTCC 5422) BY SPRAY-FREEZE-DRYING METHOD AND EVALUATION OF SURVIVAL IN SIMULATED GASTROINTESTINAL CONDITIONS	10.3109/02652048.2011.599435
MICROFLUIDIC GEL PATTERNING METHOD BY USE OF A TEMPORARY MEMBRANE FOR ORGAN-ON-CHIP APPLICATIONS	10.1002/admt.201700200
MICROSCALE BIOREACTORS FOR IN SITU CHARACTERIZATION OF GI EPITHELIAL CELL PHYSIOLOGY	10.1038/s41598-017-12984-2
MIGRATION OF BACTERIOCINS ACROSS GASTROINTESTINAL EPITHELIAL AND VASCULAR ENDOTHELIAL CELLS, AS DETERMINED USING IN VITRO SIMULATIONS	10.1038/s41598-019-47843-9
MILK FAT PROTECTS BIFIDOBACTERIUM ANIMALIS SUBSP. LACTIS BB-12 FROM IN VITRO GASTROINTESTINAL STRESS IN POTENTIALLY SYNBIOTIC TABLE SPREADS	10.1039/c8fo00506k
MIMICKING THE DYNAMIC COLONIC MICROBIOTA IN VITRO TO GAIN A BETTER UNDERSTANDING ON THE IN VIVO METABOLISM OF XENOBIOTICS: DEGRADATION OF SULFASALAZINE	10.1016/j.ijpharm.2021.120704
MIST1 PROMOTED INFLAMMATION IN COLITIS MODEL VIA K+-ATPASE NLRP3 INFLAMMASOME BY SNAI1	10.1016/j.prp.2021.153511
MIXED INFECTION BY CLOSTRIDIUM DIFFICILE IN AN IN VITRO MODEL OF THE HUMAN GUT	10.1093/jac/dks529
MOBILITY OF POLYCYCLIC AROMATIC HYDROCARBONS IN THE GASTROINTESTINAL TRACT ASSESSED USING AN IN VITRO DIGESTION MODEL WITH SORPTION RECTIFICATION	10.1021/es1010626
MOBILIZATION OF SOIL-BOUND RESIDUE OF ORGANOCHLORINE PESTICIDES AND POLYCYCLIC AROMATIC HYDROCARBONS IN AN IN VITRO GASTROINTESTINAL MODEL	10.1021/es1025849
MODELLING ORAL MALODOUR IN A LONGITUDINAL STUDY	10.1016/S0003-9969(03)00154-7
MODELLING SUGARCANE VINASSE PROCESSING IN AN ACIDOGENIC REACTOR TO PRODUCE HYDROGEN WITH AN ADM1-BASED MODEL	10.1016/j.ijhydene.2019.12.206
MODELS OF THE GUT FOR ANALYZING THE IMPACT OF FOOD AND DRUGS	10.1002/adhm.201900968
MODIFIED DIETARY FIBER FROM CASSAVA PULP AND ASSESSMENT OF MERCURY BIOACCESSIBILITY AND INTESTINAL UPTAKE USING AN IN VITRO DIGESTION/CACO-2 MODEL SYSTEM	10.1111/1750-3841.13336
MODULATION AND METABOLISM OF OBESITY-ASSOCIATED MICROBIOTA IN A DYNAMIC SIMULATOR OF THE HUMAN GUT MICROBIOTA	10.1016/j.lwt.2021.110921
MODULATION OF DENDRITIC CELL PHENOTYPE AND FUNCTION IN AN IN VITRO MODEL OF THE INTESTINAL EPITHELIUM	10.1002/eji.200535497
MODULATION OF EQUOL PRODUCTION VIA DIFFERENT DIETARY REGIMENS IN AN ARTIFICIAL MODEL OF THE HUMAN COLON	10.1016/j.jff.2020.103819
MODULATION OF GELATINIZED WHEAT STARCH DIGESTION AND FERMENTATION PROFILES BY YOUNG APPLE POLYPHENOLS IN VITRO	10.1039/d0fo02752a

MODULATION OF GENOTOXIC ENZYME ACTIVITIES BY NON-DIGESTIBLE OLIGOSACCHARIDE METABOLISM IN IN-VITRO HUMAN GUT BACTERIAL ECOSYSTEMS	10.1099/0022-1317-50-9-833
MODULATION OF GUT MICROBIOTA FROM OBESE INDIVIDUALS BY IN VITRO FERMENTATION OF CITRUS PECTIN IN COMBINATION WITH BIFIDOBACTERIUM LONGUM BB-46	10.1007/s00253-018-9234-8
MODULATION OF HUMAN GUT MICROBIOTA COMPOSITION AND METABOLITES BY ARABINOGALACTAN AND BIFIDOBACTERIUM LONGUM SUBSP. LONGUM BB536 IN THE SIMULATOR OF THE HUMAN INTESTINAL MICROBIAL ECOSYSTEM (SHIME®)	10.1016/j.jff.2021.104820
MODULATION OF OLIGOSACCHARIDES WITH DIFFERENT MONOSACCHARIDE COMPOSITION ON THE HUMAN GUT MICROBIOTA	10.16429/j.1009-7848.2020.07.006
MODULATION OF THE HUMAN GUT MICROBIOTA BY DIETARY FIBRES OCCURS AT THE SPECIES LEVEL	10.1186/s12915-015-0224-3
MOISTURE CONTENT DURING EXTRUSION OF OATS IMPACTS THE INITIAL FERMENTATION METABOLITES AND PROBIOTIC BACTERIA DURING EXTENDED FERMENTATION BY HUMAN FECAL MICROBIOTA	10.1016/j.foodres.2017.04.019
MOLECULAR ASSESSMENT OF COMPLEX MICROBIAL COMMUNITIES DEGRADING LONG CHAIN FATTY ACIDS IN METHANOGENIC BIOREACTORS	10.1111/j.1574-6941.2007.00291.x
MOLECULAR WEIGHT DISTRIBUTION AND FERMENTATION OF MECHANICALLY PRE-TREATED KONJAC ENZYMATIC HYDROLYSATES	10.1016/j.carbpol.2016.12.014
MONITORING FOOD DIGESTION WITH MAGNETIC RESONANCE TECHNIQUES	10.1017/S0029665120007867
MONITORING OF BIOTRANSFORMATION OF HOP AROMA COMPOUNDS IN AN IN VITRO DIGESTION MODEL	10.1039/c2fo30061c
MONITORING PROTEIN HYDROLYSIS BY PEPSIN USING PH-STAT: IN VITRO GASTRIC DIGESTIONS IN STATIC AND DYNAMIC PH CONDITIONS	10.1016/j.foodchem.2017.06.115
MONITORING THE DIVERSITY AND METABOLIC SHIFT OF GUT MICROBES DURING GREEN TEA FEEDING IN AN IN VITRO HUMAN COLONIC MODEL	10.3390/molecules25215101
MUCIN AS A FUNCTIONAL NICHE IS A MORE IMPORTANT DRIVER OF IN VITRO GUT MICROBIOTA COMPOSITION AND FUNCTIONALITY THAN AKKERMANSIA MUCINIPHILA SUPPLEMENTATION	10.1128/AEM.02647-20
MUCIN DEGRADATION NICHE AS A DRIVER OF MICROBIOME COMPOSITION AND AKKERMANSIA MUCINIPHILA ABUNDANCE IN A DYNAMIC GUT MODEL IS DONOR INDEPENDENT	10.1093/femsec/fiy186
MUCIN-DEGRADING MICROBES RELEASE MONOSACCHARIDES THAT CHEMOATTRACT CLOSTRIDIOIDES DIFFICILE AND FACILITATE COLONIZATION OF THE HUMAN INTESTINAL MUCUS LAYER	10.1021/acsinfecdis.0c00634
MUCOSA-ASSOCIATED BIOHYDROGENATING MICROBES PROTECT THE SIMULATED COLON MICROBIOME FROM STRESS ASSOCIATED WITH HIGH CONCENTRATIONS OF POLY-UNSATURATED FAT	10.1111/1462-2920.13622
MULTI-TARGETED PROPERTIES OF THE PROBIOTIC SACCHAROMYCES CEREVISIAE CNCM I-3856 AGAINST ENTEROTOXIGENIC ESCHERICHIA COLI (ETEC) H10407 PATHOGENESIS ACROSS HUMAN GUT MODELS	10.1080/19490976.2021.1953246
NEUTRAL MECHANISMS AND NICHE DIFFERENTIATION IN STEADY-STATE INSULAR MICROBIAL COMMUNITIES REVEALED BY SINGLE CELL ANALYSIS	10.1111/1462-2920.14437
NEW IN VITRO COLONIC FERMENTATION MODEL FOR SALMONELLA INFECTION IN THE CHILD GUT	10.1111/j.1574-6941.2008.00625.x
NEW INSIGHTS INTO THE ENTEROCOCCUS FAECIUM AND STREPTOCOCCUS GALLOLYTICUS SUBSP. GALLOLYTICUS HOST INTERACTION MECHANISMS	10.1371/journal.pone.0159159
NEW THREE-STAGE IN VITRO MODEL FOR INFANT COLONIC FERMENTATION WITH IMMOBILIZED FECAL MICROBIOTA	10.1111/j.1574-6941.2006.00117.x
NITROGEN REMOVAL THROUGH DIFFERENT PATHWAYS IN AN AGED REFUSE BIOREACTOR TREATING MATURE LANDFILL LEACHATE	10.1007/s00253-012-4623-x
NON-DIGESTIBLE GALACTOMANNAN OLIGOSACCHARIDES FROM CASSIA SEED GUM MODULATE MICROBIOTA COMPOSITION AND METABOLITES OF HUMAN FECAL INOCULUM	10.1016/j.jff.2021.104705
NONABSORBABLE DISACCHARIDES PLUS NEOMYCIN IN HEPATIC ENCEPHALOPATHY: DO THEY ENHANCE EACH OTHER?	10.1002/hep.1840120228

NONDIGESTIBLE OLIGOSACCHARIDES ENHANCE BACTERIAL COLONIZATION RESISTANCE AGAINST CLOSTRIDIUM DIFFICILE IN VITRO	10.1128/AEM.69.4.1920-1927.2003
NOVEL POLYFERMENTOR INTESTINAL MODEL (POLYFERMS) FOR CONTROLLED ECOLOGICAL STUDIES: VALIDATION AND EFFECT OF PH.	10.1371/journal.pone.0077772
NUTRIENT LOAD ACTS AS A DRIVER OF GUT MICROBIOTA LOAD, COMMUNITY COMPOSITION AND METABOLIC FUNCTIONALITY IN THE SIMULATOR OF THE HUMAN INTESTINAL MICROBIAL ECOSYSTEM	10.1093/femsec/fiab111
NUTRIENT-DRIVEN ALGAL-BACTERIAL DYNAMICS IN SEMI-CONTINUOUS, PILOT-SCALE PHOTOBIOREACTOR CULTIVATION OF NANNOCHLOROPSIS SALINA CCMP1776 WITH MUNICIPAL WASTEWATER NUTRIENTS	10.1016/j.algal.2019.101457
NUTRITIONAL QUALITY OF THE MOST CONSUMED VARIETIES OF RAW AND COOKED RICE IN SPAIN SUBMITTED TO AN IN VITRO DIGESTION MODEL	10.3390/foods10112584
NUTRITIONAL STATUS AFFECTS THE BIOACCESSIBILITY AND SPECIATION OF ARSENIC FROM SOILS IN A SIMULATOR OF THE HUMAN INTESTINAL MICROBIAL ECOSYSTEM	10.1016/j.scitotenv.2018.07.003
NUTRITIONAL STATUS AND GASTROINTESTINAL MICROBES AFFECT ARSENIC BIOACCESSIBILITY FROM SOILS AND MINE TAILINGS IN THE SIMULATOR OF THE HUMAN INTESTINAL MICROBIAL ECOSYSTEM	10.1021/es900837y
OAT BRAN FORTIFIED RASPBERRY PROBIOTIC DAIRY DRINKS: PHYSICOCHEMICAL, TEXTURAL, MICROBIOLOGIC PROPERTIES, IN VITRO BIOACCESSIBILITY OF ANTIOXIDANTS AND POLYPHENOLS	10.1016/j.fbio.2021.101223
OLIGOSACCHARIDES AS CO-ENCAPSULATING AGENTS: EFFECT ON ORAL LACTOBACILLUS FERMENTUM SURVIVAL IN A SIMULATED GASTROINTESTINAL TRACT	10.1007/s10529-018-02634-6
OMADACYCLINE GUT MICROBIOME EXPOSURE DOES NOT INDUCE CLOSTRIDIUM DIFFICILE PROLIFERATION OR TOXIN PRODUCTION IN A MODEL THAT SIMULATES THE PROXIMAL, MEDIAL, AND DISTAL HUMAN COLON	10.1128/AAC.01581-18
ON LACTOCOCCUS LACTIS UL719 COMPETITIVITY AND NISIN (NISAPLIN¬Æ) CAPACITY TO INHIBIT CLOSTRIDIUM DIFFICILE IN A MODEL OF HUMAN COLON	10.3389/fmicb.2015.01020
ON THE COLONIC BACTERIAL METABOLISM OF AZO-BONDED PRODRUGS OF 5-AMINOSALICYLIC ACID	10.1002/jps.24103
ON-SITE BLACKWATER TREATMENT FOSTERS MICROBIAL GROUPS AND FUNCTIONS TO EFFICIENTLY AND ROBUSTLY RECOVER CARBON AND NUTRIENTS	10.3390/microorganisms9010075
ONSET OF ACID-NEUTRALIZING ACTION OF A CALCIUM/MAGNESIUM CARBONATE-BASED ANTACID USING AN ARTIFICIAL STOMACH MODEL: AN IN VITRO EVALUATION	10.1186/s12876-021-01687-8
OPENING WAYS FOR SOY ISOFLAVONE BENEFITS TO A LARGER PUBLIC	
OPTIMAL CONDITIONS FOR THE ENCAPSULATION OF WEISSELLA CIBARIA JW15 USING ALGINATE AND CHICORY ROOT AND EVALUATION OF CAPSULE STABILITY IN A SIMULATED GASTROINTESTINAL SYSTEM	10.1111/1750-3841.15013
OPTIMIZATION OF AN IN VITRO GUT MICROBIOME BIOTRANSFORMATION PLATFORM WITH CHLOROGENIC ACID AS MODEL COMPOUND: FROM FECAL SAMPLE TO BIOTRANSFORMATION PRODUCT IDENTIFICATION	10.1016/j.jpba.2019.07.016
OPTIMIZATION OF ENZYMATIC SYNTHESIS OF ISOMALTO-OLIGOSACCHARIDES PRODUCTION	10.1111/j.1745-4514.2009.00222.x
OPTIMIZATION OF METABOLOMICS OF DEFINED IN VITRO GUT MICROBIAL ECOSYSTEMS	10.1016/j.ijmm.2016.03.007
OPTIMIZATION OF PROBIOTIC THERAPEUTICS USING MACHINE LEARNING IN AN ARTIFICIAL HUMAN GASTROINTESTINAL TRACT	10.1038/s41598-020-79947-y
OPTIMIZATION OF THE ANAEROBIC DENITRIFICATION PROCESS MEDIATED BY BACILLUS CEREUS IN A BATCH REACTOR	10.1016/j.eti.2019.100456
ORAL PREBIOTICS AND THE INFLUENCE OF ENVIRONMENTAL CONDITIONS IN VITRO	10.1002/JPER.17-0437
ORIGINAL BEHAVIOR OF: L. RHAMNOSUS GG ENCAPSULATED IN FREEZE-DRIED ALGINATE-SILICA MICROPARTICLES REVEALED UNDER SIMULATED GASTROINTESTINAL CONDITIONS	10.1039/c7tb02190a
OXYGEN AS A KEY PARAMETER IN IN VITRO DYNAMIC AND MULTI-COMPARTMENT MODELS TO IMPROVE MICROBIOME STUDIES OF THE SMALL INTESTINE?	10.1016/j.foodres.2020.109127
PANOSE, A NEW PREBIOTIC CANDIDATE	10.1111/j.1472-765X.2009.02698.x

PARAMETER ESTIMATION AND COLD MODEL EXPERIMENTS OF GASTROINTESTINAL SIMULATION REACTOR	10.11949/j.issn.0438-1157.20181488
PATHOGEN CHALLENGE AND DIETARY SHIFT ALTER MICROBIOTA COMPOSITION AND ACTIVITY IN A MUCIN-ASSOCIATED IN VITRO MODEL OF THE PIGLET COLON (MPIGUT-IVM) SIMULATING WEANING TRANSITION	10.3389/fmicb.2021.703421
PCR-DGGE-BASED QUANTIFICATION OF STABILITY OF THE MICROBIAL COMMUNITY IN A SIMULATOR OF THE HUMAN INTESTINAL MICROBIAL ECOSYSTEM	10.1016/j.femsec.2004.05.002
PECTIN-CHITOSAN CONJUGATED NANOLIPOSOME AS A PROMISING DELIVERY SYSTEM FOR NEOHESPERIDIN: CHARACTERIZATION, RELEASE BEHAVIOR, CELLULAR UPTAKE, AND ANTIOXIDANT PROPERTY	10.1016/j.foodhyd.2019.04.059
PENIOPHORA LYCII PHYTASE IS STABILE AND DEGRADES PHYTATE AND SOLUBILISES MINERALS IN VITRO DURING SIMULATION OF GASTROINTESTINAL DIGESTION IN THE PIG	10.1002/jsfa.3033
PEPTIDE PROFILING AND THE BIOACTIVITY CHARACTER OF YOGURT IN THE SIMULATED GASTROINTESTINAL DIGESTION	10.1016/j.jprot.2016.04.010
PERFORMANCE AND BACTERIAL COMMUNITY CHANGE DURING THE START-UP PERIOD OF A NOVEL ANAEROBIC BIOREACTOR INOCULATED WITH LONG-TIME STORAGE ANAEROBIC GRANULAR SLUDGE	10.1061/(ASCE)EE.1943-7870.0001222
PERFORMANCE AND MICROBIAL COMMUNITY STRUCTURE OF AEROBIC GRANULAR BIOREACTORS AT DIFFERENT OPERATIONAL TEMPERATURE	10.1016/j.jwpe.2019.101110
PERINATAL ENVIRONMENT SHAPES MICROBIOTA COLONIZATION AND INFANT GROWTH: IMPACT ON HOST RESPONSE AND INTESTINAL FUNCTION	10.1186/s40168-020-00940-8
PERMEABILITY OF NATIVE AND DIGESTED POLYPHENOLS FROM APPLE, BLUEBERRY AND CRANBERRY EXTRACTS USING PAMPA MEMBRANE PERMEABILITY ASSAYS	10.1016/j.jfca.2021.103945
PERSISTENCE OF ANTICANCER ACTIVITY IN BERRY EXTRACTS AFTER SIMULATED GASTROINTESTINAL DIGESTION AND COLONIC FERMENTATION	10.1371/journal.pone.0049740
PHENOLIC COMPOSITION AND INHIBITORY EFFECT AGAINST OXIDATIVE DNA DAMAGE OF COOKED COWPEAS AS AFFECTED BY SIMULATED IN VITRO GASTROINTESTINAL DIGESTION	10.1016/j.foodchem.2013.05.001
PHENOLIC COMPOUNDS FROM ”HASS” AVOCADO PEEL ARE RETAINED IN THE INDIGESTIBLE FRACTION AFTER AN IN VITRO GASTROINTESTINAL DIGESTION	10.1007/s11694-020-00794-6
PHENOLIC COMPOUNDS, MICROSTRUCTURE AND VISCOSITY OF ONION AND APPLE PRODUCTS SUBJECTED TO IN VITRO GASTROINTESTINAL DIGESTION	10.1016/j.ifset.2018.05.014
PHENOLIC PROFILE AND ANTIOXIDANT ACTIVITY OF JASONIA GLUTINOSA HERBAL TEA. INFLUENCE OF SIMULATED GASTROINTESTINAL IN VITRO DIGESTION	10.1016/j.foodchem.2019.02.101
PHENOLIC PROFILE AND FERMENTATION PATTERNS OF DIFFERENT COMMERCIAL GLUTEN-FREE PASTA DURING IN VITRO LARGE INTESTINE FERMENTATION	10.1016/j.foodres.2017.03.035
PHYSICAL EFFECTS OF DIETARY FIBRE ON SIMULATED LUMINAL FLOW, STUDIED BY: IN VITRO DYNAMIC GASTROINTESTINAL DIGESTION AND FERMENTATION	10.1039/c9fo00485h
PHYSICO-CHEMICAL PROPERTIES OF PURIFIED STARCH AFFECT THEIR IN VITRO FERMENTATION CHARACTERISTICS AND ARE LINKED TO IN VIVO FERMENTATION CHARACTERISTICS IN PIGS	10.1016/j.anifeedsci.2019.05.006
PHYSICOCHEMICAL PROPERTIES OF SOY PROTEIN HYDROLYSATE AND ITS FORMULATION AND STABILITY WITH ENCAPSULATED PROBIOTIC UNDER IN VITRO GASTROINTESTINAL ENVIRONMENT	10.1111/1750-3841.15399
PHYSICOCHEMICAL PROPERTIES, STRUCTURE AND DIGESTIBILITY IN SIMULATED GASTROINTESTINAL ENVIRONMENT OF BREAD ADDED WITH GREEN LENTIL FLOUR	10.1016/j.lwt.2021.112713
PHYTOCHEMICAL CONTENT, CELLULAR ANTIOXIDANT ACTIVITY AND ANTIPROLIFERATIVE ACTIVITY OF: ADINANDRA NITIDA TEA (SHIYACHA) INFUSION SUBJECTED TO IN VITRO GASTROINTESTINAL DIGESTION	10.1039/c7ra07429h
PHYTOCHEMICAL PROFILE, BIOACTIVITY, AND PREBIOTIC POTENTIAL OF BOUND PHENOLICS RELEASED FROM RICE BRAN DIETARY FIBER DURING IN VITRO GASTROINTESTINAL DIGESTION AND COLONIC FERMENTATION	10.1021/acs.jafc.9b06477

PHYTOCHEMICAL PROFILE, MINERAL CONTENT, AND ANTIOXIDANT ACTIVITY OF OLEA EUROPAEA L. CV. CORNEZUELO TABLE OLIVES. INFLUENCE OF IN VITRO SIMULATED GASTROINTESTINAL DIGESTION	10.1016/j.foodchem.2019.05.207
PIEZO-TOLERANT NATURAL GAS-PRODUCING MICROBES UNDER ACCUMULATING PCO2	10.1186/s13068-016-0634-7
PLANT EXTRACTS AS NATURAL MODULATORS OF GUT MICROBIOTA COMMUNITY STRUCTURE AND FUNCTIONALITY	10.1016/j.heliyon.2020.e05474
PLANT POLYPHENOLS ALTER A PATHWAY OF ENERGY METABOLISM BY INHIBITING FECAL BACTEROIDETES AND FIRMICUTES IN VITRO	10.1039/c5fo01438g
PLANT STEROLS AND HUMAN GUT MICROBIOTA RELATIONSHIP: AN IN VITRO COLONIC FERMENTATION STUDY	10.1016/j.jff.2018.03.023
POLYCYCLIC AROMATIC HYDROCARBON RELEASE FROM A SOIL MATRIX IN THE IN VITRO GASTROINTESTINAL TRACT	10.2134/jeq2004.1343
POLYDEXTROSE, LACTITOL, AND FRUCTO-OLIGOSACCHARIDE FERMENTATION BY COLONIC BACTERIA IN A THREE-STAGE CONTINUOUS CULTURE SYSTEM	10.1128/AEM.70.8.4505-4511.2004
POLYPHENOL AND NUTRIENT RELEASE FROM SKIN OF ALMONDS DURING SIMULATED HUMAN DIGESTION	10.1016/j.foodchem.2010.03.079
POLYPHENOLS AND TRYPTOPHAN METABOLITES ACTIVATE THE ARYL HYDROCARBON RECEPTOR IN AN IN VITRO MODEL OF COLONIC FERMENTATION	10.1002/mnfr.201800722
POLYPHENOLS IN CASSAVA LEAVES (MANIHOT ESCULENTA CRANTZ) AND THEIR STABILITY IN ANTIOXIDANT POTENTIAL AFTER IN VITRO GASTROINTESTINAL DIGESTION	10.1016/j.heliyon.2020.e03567
PORK LIVER PÂTÉ ENRICHED WITH PERSIMMON COPRODUCTS: EFFECT OF IN VITRO GASTROINTESTINAL DIGESTION ON ITS FATTY ACID AND POLYPHENOL PROFILE STABILITY	10.3390/nu13041332
POSTPROCESSING IN VITRO DIGESTION CHALLENGE TO EVALUATE SURVIVAL OF ESCHERICHIA COLI O157:H7 IN FERMENTED DRY SAUSAGES	10.1128/AEM.70.11.6637-6642.2004
POTENTIAL CONTRIBUTION OF OPTIONAL UREASE-POSITIVE BACTERIA TO IDIOPATHIC URINARY CALCIUM STONE FORMATION: II. MICROLITH FORMATION KINETICS IN A FERMENTER MODEL OF THE URINARY TRACT INFECTED BY OPTIONAL UREASE-POSITIVE MICROORGANISMS	10.1007/BF00431082
POTENTIAL GUT ADHERENT PROBIOTIC BACTERIA ISOLATED FROM ROHU, LABEO ROHITA (ACTINOPTERYGII: CYPRINIFORMES: CYPRINIDAE): CHARACTERISATION, EXO-ENZYME PRODUCTION, PATHOGEN INHIBITION, CELL SURFACE HYDROPHOBICITY, AND BIO-FILM FORMATION	10.3750/AIEP/02251
POTENTIAL IMPACT OF BIOPOLYMERS (E-POLYLYSINE AND/OR PECTIN) ON GASTROINTESTINAL FATE OF FOODS: IN VITRO STUDY	10.1016/j.foodres.2015.06.036
POTENTIAL OF HIGH- AND LOW-ACETYLATED GALACTOGLUCOMANNOOLIGOSACCHARIDES AS MODULATORS OF THE MICROBIOTA COMPOSITION AND THEIR ACTIVITY: A COMPARISON USING THE IN VITRO MODEL OF THE HUMAN COLON TIM-2	10.1021/acs.jafc.0c02225
POTENTIAL OF PERSIMMON DIETARY FIBER OBTAINED FROM BYPRODUCTS AS ANTIOXIDANT, PREBIOTIC AND MODULATING AGENT OF THE INTESTINAL EPITHELIAL BARRIER FUNCTION	10.3390/antiox10111668
POTENTIAL PREBIOTIC ACTIVITY OF OLIGOSACCHARIDES OBTAINED BY ENZYMATIC CONVERSION OF DURUM WHEAT INSOLUBLE DIETARY FIBRE INTO SOLUBLE DIETARY FIBRE	10.1016/j.numecd.2008.07.005
POTENTIAL PREBIOTIC ACTIVITY OF TENEBRIO MOLITOR INSECT FLOUR USING AN OPTIMIZED IN VITRO GUT MICROBIOTA MODEL	10.1039/c8fo01536h
POTENTIAL PREBIOTIC EFFECT OF FRUIT AND VEGETABLE BYPRODUCTS FLOUR USING IN VITRO GASTROINTESTINAL DIGESTION	10.1016/j.foodres.2020.109354
POTENTIAL PREBIOTIC PROPERTIES OF EXOPOLYSACCHARIDES PRODUCED BY A NOVEL: LACTOBACILLUS STRAIN, LACTOBACILLUS PENTOSUS YY-112	10.1039/d1fo01261d
POTENTIAL PREBIOTIC PROPERTIES OF FLOURS FROM DIFFERENT VARIETIES OF SWEET POTATO (IPOMOEA BATATAS L.) ROOTS CULTIVATED IN NORTHEASTERN BRAZIL	10.1016/j.fbio.2020.100614

POTENTIAL PROBIOTIC KLUYVEROMYCES MARXIANUS B0399 MODULATES THE IMMUNE RESPONSE IN CACO-2 CELLS AND PERIPHERAL BLOOD MONONUCLEAR CELLS AND IMPACTS THE HUMAN GUT MICROBIOTA IN AN IN VITRO COLONIC MODEL SYSTEM	10.1128/AEM.06385-11
PREBIOTIC CARBOHYDRATES MODIFY THE MUCOSA ASSOCIATED MICROFLORA OF THE HUMAN LARGE BOWEL	10.1136/gut.2003.037580
PREBIOTIC CHARACTERISTICS OF ARABINOGALACTANS DURING IN VITRO FERMENTATION THROUGH MULTI-OMICS ANALYSIS	10.1016/j.fct.2021.112522
PREBIOTIC EFFECT OF FRUCTOOLIGOSACCHARIDE IN THE SIMULATOR OF THE HUMAN INTESTINAL MICROBIAL ECOSYSTEM (SHIME¬Æ MODEL)	10.1089/jmf.2013.0092
PREBIOTIC EFFECT OF PREDIGESTED MANGO PEEL ON GUT MICROBIOTA ASSESSED IN A DYNAMIC IN VITRO MODEL OF THE HUMAN COLON (TIM-2)	10.1016/j.foodres.2017.12.024
PREBIOTIC EFFECTS OF CASSAVA BAGASSE IN TNO'S IN VITRO MODEL OF THE COLON IN LEAN VERSUS OBESE MICROBIOTA	10.1016/j.jff.2014.09.019
PREBIOTIC EFFECTS OF CHICORY INULIN IN THE SIMULATOR OF THE HUMAN INTESTINAL MICROBIAL ECOSYSTEM	10.1016/j.femsec.2004.07.014
PREBIOTIC EFFECTS OF OLIVE POMACE POWDERS IN THE GUT: IN VITRO EVALUATION OF THE INHIBITION OF ADHESION OF PATHOGENS, PREBIOTIC AND ANTIOXIDANT EFFECTS	10.1016/j.foodhyd.2020.106312
PREBIOTIC EFFECTS OF PECTOOLIGOSACCHARIDES OBTAINED FROM LEMON PEEL ON THE MICROBIOTA FROM ELDERLY DONORS USING AN: IN VITRO CONTINUOUS COLON MODEL (TIM-2)	10.1039/d0fo01848a
PREBIOTIC EVALUATION OF RED SEAWEED (KAPPAPHYCUS ALVAREZII) USING IN VITRO COLON MODEL	10.1080/09637486.2017.1309522
PREBIOTIC POTENTIAL OF SOME EXOPOLYSACCHARIDES PRODUCED BY LACTIC ACID BACTERIA	
PREBIOTIC PROPERTIES OF DIFFERENT POLYSACCHARIDE FRACTIONS FROM ARTEMISIA SPHAEROCEPHALA KRASCH SEEDS EVALUATED BY SIMULATED DIGESTION AND IN VITRO FERMENTATION BY HUMAN FECAL MICROBIOTA	10.1016/j.ijbiomac.2020.06.174
PREBIOTICS INHIBIT PROTEOLYSIS BY GUT BACTERIA IN A HOST DIET-DEPENDENT MANNER: A THREE-STAGE CONTINUOUS IN VITRO GUT MODEL EXPERIMENT	10.1128/AEM.02730-19
PREBIOTICS-ENCAPSULATED PROBIOTIC SPORES REGULATE GUT MICROBIOTA AND SUPPRESS COLON CANCER	10.1002/adma.202004529
PREDICTING AND TESTING BIOAVAILABILITY OF MAGNESIUM SUPPLEMENTS	10.3390/nu11071663
PREDICTING THE HUMAN IN VIVO PERFORMANCE OF DIFFERENT ORAL CAPSULE SHELL TYPES USING A NOVEL IN VITRO DYNAMIC GASTRIC MODEL	10.1016/j.ijpharm.2011.07.046
PRELIMINARY SELECTION FOR POTENTIAL PROBIOTIC BIFIDOBACTERIUM ISOLATED FROM SUBJECTS OF DIFFERENT CHINESE ETHNIC GROUPS AND EVALUATION OF THEIR FERMENTATION AND STORAGE CHARACTERISTICS IN BOVINE MILK	10.3168/jds.2013-6582
PRELIMINARY STUDIES OF THE IMPACT OF FOOD COMPONENTS ON NUTRITIONAL PROPERTIES OF NANOPARTICLES	10.1016/j.foodchem.2021.131391
PREPARATION OF A STANDARDISED FAECAL SLURRY FOR EX-VIVO MICROBIOTA STUDIES WHICH REDUCES INTER-INDIVIDUAL DONOR BIAS	10.1016/j.mimet.2016.08.002
PRESENCE OF ANAEROBIC BACTEROIDES IN AEROBICALLY GROWN MICROBIAL GRANULES	10.1007/s00248-002-2014-z
PRIMARY HUMAN COLONIC MUCOSAL BARRIER CROSSTALK WITH SUPER OXYGEN-SENSITIVE FAECALIBACTERIUM PRAUSNITZII IN CONTINUOUS CULTURE	10.1016/j.medj.2020.07.001
PROBIOTIC ASSESSMENT OF ENTEROCOCCUS FAECALIS CP58 ISOLATED FROM HUMAN GUT	10.1016/j.ijfoodmicro.2010.12.029
PROBIOTIC CHARACTERISTICS OF BACILLUS STRAINS ISOLATED FROM KOREAN TRADITIONAL SOY SAUCE	10.1016/j.lwt.2016.08.040
PROBIOTIC CHARACTERISTICS OF BACTERIOCIN-PRODUCING ENTEROCOCCUS FAECIUM STRAINS ISOLATED FROM HUMAN MILK AND COLOSTRUM	10.1007/s12223-019-00687-2

PROBIOTIC INFANT CEREAL IMPROVES CHILDREN'S GUT MICROBIOTA: INSIGHTS USING THE SIMULATOR OF HUMAN INTESTINAL MICROBIAL ECOSYSTEM (SHIME®)	10.1016/j.foodres.2021.110292
PROBIOTIC POTENTIAL AND BIOFILM INHIBITORY ACTIVITY OF LACTOBACILLUS CASEI GROUP STRAINS ISOLATED FROM INFANT FECES	10.1016/j.jff.2019.02.004
PROBIOTIC POTENTIAL OF A NOVEL VITAMIN B2-OVERPRODUCING LACTOBACILLUS PLANTARUM STRAIN, HY7715, ISOLATED FROM KIMCHI	10.3390/app11135765
PROBIOTIC POTENTIAL OF LACTOBACILLUS FERMENTUM G-4 ORIGINATING FROM THE MECONIUM OF NEWBORNS	10.2298/JSC181105015Z
PROBIOTIC PROPERTIES OF LEUCONOSTOC MESENTEROIDES ISOLATED FROM AGUAMIEL OF AGAVE SALMIANA	10.1007/s12602-015-9187-5
PROBIOTIC SUPPLEMENTATION IN A CLOSTRIDIUM DIFFICILE-INFECTED GASTROINTESTINAL MODEL IS ASSOCIATED WITH RESTORING METABOLIC FUNCTION OF MICROBIOTA	10.3390/microorganisms8010060
PROBIOTIC SUPPLEMENTATION IS ASSOCIATED WITH INCREASED ANTIOXIDANT CAPACITY AND COPPER CHELATION IN C. DIFFICILE-INFECTED FECAL WATER	10.3390/nu11092007
PROBIOTIC SURVIVAL DURING A MULTI-LAYERED TABLET DEVELOPMENT AS TESTED IN A DYNAMIC, COMPUTER-CONTROLLED IN VITRO MODEL OF THE STOMACH AND SMALL INTESTINE (TIM-1)	10.1111/lam.13211
PROCESS CONSTRUCTION AND FERMENTATION OPTIMIZATION FOR BUTYRATE PRODUCTION FROM LACTATE	10.3724/SP.J.1145.2015.11061
PROCESSING HAS DIFFERENTIAL EFFECTS ON MICROBIOTA-ACCESSIBLE CARBOHYDRATES IN WHOLE GRAINS DURING IN VITRO FERMENTATION	10.1128/AEM.01705-20
PRODUCTION AND IN VITRO FERMENTATION OF SOLUBLE, NON-DIGESTIBLE, FERULOYLATED OLIGO- AND POLYSACCHARIDES FROM MAIZE AND WHEAT BRANS	10.1021/jf404305y
PRODUCTION OF GALACTO-OLIGOSACCHARIDES FROM LACTOSE BY IMMOBILIZED Œí-GALACTOSIDASE	
PRODUCTION OF IMMUNE RESPONSE MEDIATORS BY HT-29 INTESTINAL CELL-LINES IN THE PRESENCE OF BIFIDOBACTERIUM-TREATED INFANT MICROBIOTA	10.3920/BM2014.0111
PRODUCTION OF IMMUNOGLOBULIN A IN DIFFERENT REACTOR CONFIGURATIONS	10.1007/BF00749221
PRODUCTION OF RECOMBINANT HUMAN DIPEPTIDYL PEPTIDASE IV FROM SF9 CELLS IN MICROBIAL FERMENTERS	10.3906/biy-1503-9
PRODUCTION, STORAGE STABILITY, AND SUSCEPTIBILITY TESTING OF REUTERIN AND ITS IMPACT ON THE MURINE FECAL MICROBIOME AND VOLATILE ORGANIC COMPOUND PROFILE	10.3389/fmicb.2021.699858
PROFILING HUMAN GUT BACTERIAL METABOLISM AND ITS KINETICS USING [U- 13C]GLUCOSE AND NMR	10.1002/nbm.1418
PROPIONATE-PRODUCING CONSORTIUM RESTORES ANTIBIOTIC-INDUCED DYSBIOSIS IN A DYNAMIC IN VITRO MODEL OF THE HUMAN INTESTINAL MICROBIAL ECOSYSTEM	10.3389/fmicb.2019.01206
PROTECTION FROM CHEMOTHERAPY- AND ANTIBIOTIC-MEDIATED DYSBIOSIS OF THE GUT MICROBIOTA BY A PROBIOTIC WITH DIGESTIVE ENZYMES SUPPLEMENT	10.18632/oncotarget.25778
PROTECTION OF EPIGALLOCATECHIN GALLATE AGAINST DEGRADATION DURING IN VITRO DIGESTION USING APPLE POMACE AS A CARRIER	10.1021/jf504659n
PROTECTIVE ACTION OF BACILLUS CLAUSII PROBIOTIC STRAINS IN AN IN VITRO MODEL OF ROTAVIRUS INFECTION	10.1038/s41598-020-69533-7
PROTECTIVE EFFECT OF AN AVOCADO PEEL POLYPHENOLIC EXTRACT RICH IN PROANTHOCYANIDINS ON THE ALTERATIONS OF COLONIC HOMEOSTASIS INDUCED BY A HIGH-PROTEIN DIET	10.1021/acs.jafc.9b03905
PROTECTIVE EFFECT OF PROBIOTICS ON SALMONELLA INFECTIVITY ASSESSED WITH COMBINED IN VITRO GUT FERMENTATION-CELLULAR MODELS	10.1186/1471-2180-11-264
PROTECTIVE EFFECT OF THE BILE SALT HYDROLASE-ACTIVE LACTOBACILLUS RENTERI AGAINST BILE SALT CYTOTOXICITY	10.1007/s002530000330
PROTEIN DEGRADATION AND PEPTIDE RELEASE FROM MILK PROTEINS IN HUMAN JEJUNUM. COMPARISON WITH IN VITRO GASTROINTESTINAL SIMULATION	10.1016/j.foodchem.2017.06.134

PROTEIN-OLIGOSACCHARIDE CONJUGATES AS NOVEL PREBIOTICS	10.1002/pat.4658
PULP IN SHOP-BOUGHT ORANGE JUICE HAS LITTLE EFFECT ON FLAVONOID CONTENT AND GUT BACTERIAL FLAVANONE DEGRADATION IN VITRO	10.1007/s11130-019-00739-5
PULSED ELECTRIC FIELD IMPROVED PROTEIN DIGESTION OF BEEF DURING IN-VITRO GASTROINTESTINAL SIMULATION	10.1016/j.lwt.2018.12.013
PURIFICATION AND CHARACTERIZATION OF ANGIOTENSIN-CONVERTING ENZYME-INHIBITORY PEPTIDES FROM NILE TILAPIA (OREOCHROMIS NILOTICUS) SKIN GELATINE PRODUCED BY AN ENZYMATIC MEMBRANE REACTOR	10.1016/j.jff.2017.07.011
QUANTIFICATION OF GLYPHOSATE AND AMINOMETHYLPHOSPHONIC ACID FROM MICROBIOME REACTOR FLUIDS	10.1002/rcm.8668
QUINOA FLAVONOIDS AND THEIR BIOACCESSIBILITY DURING IN VITRO GASTROINTESTINAL DIGESTION	10.1016/j.jcs.2020.103070
RAPID AND COMPLETE DEHALOGENATION OF HALONITROMETHANES IN SIMULATED GASTROINTESTINAL TRACT AND ITS INFLUENCE ON TOXICITY	10.1016/j.chemosphere.2018.08.039
RAPID SUCCESSION OF UNCULTURED MARINE BACTERIAL AND ARCHAEAL POPULATIONS IN A DENITRIFYING CONTINUOUS CULTURE	10.1111/1462-2920.12552
REAL-TIME CELLULAR IMPEDANCE MONITORING AND IMAGING OF BIOLOGICAL BARRIERS IN A DUAL-FLOW MEMBRANE BIOREACTOR	10.1016/j.bios.2019.111340
REAL-TIME MONITORING OF HT29 EPITHELIAL CELLS AS AN IN VITRO MODEL FOR ASSESSING FUNCTIONAL DIFFERENCES AMONG INTESTINAL MICROBIOTAS FROM DIFFERENT HUMAN POPULATION GROUPS	10.1016/j.mimet.2018.07.003
RECENT ADVANCEMENTS IN INTESTINAL MICROBIOTA ANALYSES: A REVIEW FOR NON-MICROBIOLOGISTS	10.1007/s11596-018-1969-z
RECENT RESEARCHES ON PREBIOTICS FOR GUT HEALTH IN THAILAND	10.31989/ffhd.v5i11.214
RECIPROCAL INTERACTIONS BETWEEN EPIGALLOCATECHIN-3-GALLATE (EGCG) AND HUMAN GUT MICROBIOTA IN VITRO	10.1021/acs.jafc.0c03587
RECOVERY OF METALS FROM WASTE LITHIUM ION BATTERY LEACHATES USING BIOGENIC HYDROGEN SULFIDE	10.3390/min9090563
RECURRENCE OF DUAL-STRAIN CLOSTRIDIUM DIFFICILE INFECTION IN AN IN VITRO HUMAN GUT MODEL	10.1093/jac/dkv108
RED WINE HIGH-MOLECULAR-WEIGHT POLYPHENOLIC COMPLEX: AN EMERGING MODULATOR OF HUMAN METABOLIC DISEASE RISK AND GUT MICROBIOTA	10.1021/acs.jafc.1c03158
REFLECTION OF CONCENTRATIONS OF POLYBROMINATED DIPHENYL ETHERS IN HEALTH RISK ASSESSMENT: A CASE STUDY IN SEDIMENTS FROM THE METROPOLITAN RIVER, NORTH CHINA	10.1016/j.envpol.2019.01.041
REGULATED EXPRESSION OF POLYSACCHARIDE UTILIZATION AND CAPSULAR BIOSYNTHESIS LOCI IN BIOFILM AND PLANKTONIC BACTEROIDES THETAIOTAOMICRON DURING GROWTH IN CHEMOSTATS	10.1002/bit.24994
RELATIONSHIPS BETWEEN TRANSIT TIME IN MAN AND IN VITRO FERMENTATION OF DIETARY FIBER BY FECAL BACTERIA	10.1038/sj.ejcn.1600687
RELEASE AND METABOLISM OF BOUND POLYPHENOLS FROM CARROT DIETARY FIBER AND THEIR POTENTIAL ACTIVITY IN: IN VITRO DIGESTION AND COLONIC FERMENTATION	10.1039/d0fo00975j
RELEASE OF MULTIFUNCTIONAL PEPTIDES FROM KIWICHA (AMARANTHUS CAUDATUS) PROTEIN UNDER IN VITRO GASTROINTESTINAL DIGESTION	10.1002/jsfa.9294
RELEASE OF SMALL PHENOLIC COMPOUNDS FROM BREWER'S SPENT GRAIN AND ITS LIGNIN FRACTIONS BY HUMAN INTESTINAL MICROBIOTA IN VITRO	10.1021/jf4024195
REMOVAL AND INACTIVATION OF WATERBORNE VIRUSES USING ZEROVALENT IRON	10.1021/es050829j
REMOVAL AND SURVIVAL OF FECAL INDICATORS IN A CONSTRUCTED WETLAND AFTER UASB PRE-TREATMENT	10.3390/su13169302
REORGANIZATION OF THE BACTERIAL AND ARCHAEAL POPULATIONS ASSOCIATED WITH ORGANIC LOADING CONDITIONS IN A THERMOPHILIC ANAEROBIC DIGESTER	10.1016/j.jbiosc.2014.09.003

REPRINT OF "BIOACCESSIBILITY OF T-2 AND HT-2 TOXINS IN MYCOTOXIN CONTAMINATED BREAD MODELS SUBMITTED TO IN VITRO HUMAN DIGESTION"	10.1016/j.ifset.2014.07.009
RESISTANCE TO SIMULATED GASTROINTESTINAL CONDITIONS AND ADHESION TO MUCUS AS PROBIOTIC CRITERIA FOR BIFIDOBACTERIUM LONGUM STRAINS	10.1007/s00284-008-9135-7
RIFAXIMIN MODULATES THE COLONIC MICROBIOTA OF PATIENTS WITH CROHN'S DISEASE: AN IN VITRO APPROACH USING A CONTINUOUS CULTURE COLONIC MODEL SYSTEM	10.1093/jac/dkq345
ROLE OF MALTODEXTRIN AND INULIN AS ENCAPSULATING AGENTS ON THE PROTECTION OF OLEUROPEIN DURING IN VITRO GASTROINTESTINAL DIGESTION	10.1016/j.foodchem.2019.125976
ROLE OF NON-THERMAL TREATMENTS AND FERMENTATION WITH PROBIOTIC LACTOBACILLUS PLANTARUM ON IN VITRO BIOACCESSIBILITY OF BIOACTIVES FROM VEGETABLE JUICE	10.1002/jsfa.11124
ROLE OF SHORT CHAIN FATTY ACID RECEPTORS IN INTESTINAL PHYSIOLOGY AND PATHOPHYSIOLOGY	10.1002/cphy.c170050
SACCHARIN AND SUCRALOSE PROTECT THE GLOMERULAR MICROVASCULATURE IN VITRO AGAINST VEGF-INDUCED PERMEABILITY	10.3390/nu13082746
SAFETY AND POTENTIAL BENEFICIAL PROPERTIES OF ENTEROCOCCUS STRAINS ISOLATED FROM KEFIR	10.1016/j.idairyj.2014.06.009
SAFETY EVALUATION AND WHOLE-GENOME ANNOTATION OF LACTOBACILLUS PLANTARUM STRAINS FROM DIFFERENT SOURCES WITH SPECIAL FOCUS ON ISOLATES FROM GREEN TEA	10.1007/s12602-019-09620-y
SALT RELEASE MONITORING WITH SPECIFIC SENSORS IN IN VITRO ORAL AND DIGESTIVE ENVIRONMENTS FROM SOFT CHEESES	10.1016/j.talanta.2012.04.013
SCREENING AND IDENTIFICATION OF LATILACTOBACILLUS CURVATUS Z12 FROM RUMEN FLUID OF AN ADULT FEMALE SIKA DEER AS A POTENTIAL PROBIOTIC FOR FEED ADDITIVES	10.3389/fvets.2021.753527
SCREENING DIETARY FIBRES FOR FERMENTATION CHARACTERISTICS AND METABOLIC PROFILES USING A RAPID IN VITRO APPROACH: IMPLICATIONS FOR IRRITABLE BOWEL SYNDROME	10.1017/S0007114520003943
SCREENING FOR PROBIOTIC PROPERTIES OF STRAINS ISOLATED FROM FECES OF VARIOUS HUMAN GROUPS	10.1007/s12275-012-2045-1
SCREENING OF INTESTINAL LACTOBACILLUS FROM BAMA CENTENARIANS AND THEIR PROBIOTIC CHARACTERISTICS	10.13982/j.mfst.1673-9078.2017.1.007
SECRETORY EXPRESSION OF Œí-MANNANASE FROM BACILLUS CIRCULANS NT 6.7 IN LACTOBACILLUS PLANTARUM	10.1016/j.pep.2017.07.005
SELECTED PROBIOTIC LACTOBACILLI HAVE THE CAPACITY TO HYDROLYZE GLUTEN PEPTIDES DURING SIMULATED GASTROINTESTINAL DIGESTION	10.1128/AEM.00376-17
SELECTION AND CHARACTERIZATION OF BROAD-SPECTRUM ANTIBACTERIAL SUBSTANCE-PRODUCING LACTOBACILLUS CURVATUS PA40 AS A POTENTIAL PROBIOTIC FOR FEED ADDITIVES	10.1111/asj.13047
SELECTION OF GUT-RESISTANT BACTERIA AND CONSTRUCTION OF MICROBIAL CONSORTIA FOR IMPROVING GLUTEN DIGESTION UNDER SIMULATED GASTROINTESTINAL CONDITIONS	10.3390/nu13030992
SELECTION OF LACTIC ACID BACTERIA AS STARTER CULTURES FOR FERMENTED MEAT PRODUCTS	10.3136/fstr.18.713
SELECTION OF POTENTIAL PROBIOTIC LACTIC ACID BACTERIA FROM FERMENTED OLIVES BY IN VITRO TESTS	10.1016/j.fm.2012.10.005
SELECTION OF POTENTIALLY PROBIOTIC KLUYVEROMYCES LACTIS FOR THE FERMENTATION OF CHEESE WHEY,ÄìBASED BEVERAGE	10.1007/s13213-019-01518-y
SET UP OF A NEW IN VITRO MODEL TO STUDY DIETARY FRUCTANS FERMENTATION IN FORMULA-FED BABIES	10.1017/S0007114509991796
SHORT-CHAIN FATTY ACIDS AS NOVEL THERAPEUTICS FOR GESTATIONAL DIABETES	10.1530/JME-20-0094
SIALYLLACTOSE AND GALACTOOLIGOSACCHARIDES PROMOTE EPITHELIAL BARRIER FUNCTIONING AND DISTINCTLY MODULATE MICROBIOTA COMPOSITION AND SHORT CHAIN FATTY ACID PRODUCTION IN VITRO	10.3389/fimmu.2019.00094
SIMULATED COLON FIBER METABOLOME REGULATES GENES INVOLVED IN CELL CYCLE, APOPTOSIS, AND ENERGY METABOLISM IN HUMAN COLON CANCER CELLS	10.1007/s11010-011-0894-2

SIMULATED DIGESTION AND FERMENTATION IN VITRO BY HUMAN GUT MICROBIOTA OF POLYSACCHARIDES FROM HELICTERES ANGUSTIFOLIA L	10.1016/j.ijbiomac.2019.09.073
SIMULATED GASTROINTESTINAL CONDITIONS INCREASE ADHESION ABILITY OF LACTOBACILLUS PARACASEI STRAINS ISOLATED FROM KEFIR TO CACO-2 CELLS AND MUCIN	10.1016/j.foodres.2017.09.093
SIMULATED GASTROINTESTINAL DIGESTION AND IN VITRO COLONIC FERMENTATION OF CAROB POLYPHENOLS: BIOACCESSIBILITY AND BIOACTIVITY	10.1016/j.lwt.2019.108623
SIMULATED GASTROINTESTINAL DIGESTION AND IN VITRO COLONIC FERMENTATION OF SPENT COFFEE (COFFEA ARABICA L.): BIOACCESSIBILITY AND INTESTINAL PERMEABILITY	10.1016/j.foodres.2015.07.024
SIMULATED GASTROINTESTINAL DIGESTION OF CRANBERRY POLYPHENOLS UNDER DYNAMIC CONDITIONS. IMPACT ON ANTIADHESIVE ACTIVITY AGAINST UROPATHOGENIC BACTERIA	10.1016/j.foodchem.2021.130871
SIMULATED GASTROINTESTINAL DIGESTION OF NISIN AND INTERACTION BETWEEN NISIN AND BILE	10.1016/j.lwt.2017.08.031
SIMULATED GASTROINTESTINAL TRACT METABOLISM AND PHARMACOLOGICAL ACTIVITIES OF WATER EXTRACT OF SCUTELLARIA BAICALENSIS ROOTS	10.1016/j.jep.2013.12.056
SIMULATING DISTAL GUT MUCOSAL AND LUMINAL COMMUNITIES USING PACKED-COLUMN BIOFILM REACTORS AND AN IN VITRO CHEMOSTAT MODEL	10.1016/j.mimet.2014.11.007
SIMULATION AND MODELING OF DIETARY CHANGES IN THE INFANT GUT MICROBIOME	10.1093/femsec/fiy140
SINGLE BIOREACTOR GASTROINTESTINAL TRACT SIMULATOR FOR STUDY OF SURVIVAL OF PROBIOTIC BACTERIA	10.1007/s00253-008-1553-8
SMT19969 AS A TREATMENT FOR CLOSTRIDIUM DIFFICILE INFECTION: AN ASSESSMENT OF ANTIMICROBIAL ACTIVITY USING CONVENTIONAL SUSCEPTIBILITY TESTING AND AN IN VITRO GUT MODEL	10.1093/jac/dku324
SOLID-LIQUID SEPARATION METHOD GOVERNS THE IN VITRO BIOACCESSIBILITY OF METALS IN CONTAMINATED SOIL-LIKE TEST MATERIALS	10.1016/j.chemosphere.2014.12.019
SOYMILK RESIDUE (OKARA) AS A NATURAL IMMOBILIZATION CARRIER FOR LACTOBACILLUS PLANTARUM CELLS ENHANCES SOYMILK FERMENTATION, GLUCOSIDIC ISOFLAVONE BIOCONVERSION, AND CELL SURVIVAL UNDER SIMULATED GASTRIC AND INTESTINAL CONDITIONS	10.7717/peerj.2701
SPATIAL AND TEMPORAL MODULATION OF ENTEROTOXIGENIC E. COLI H10407 PATHOGENESIS AND INTERPLAY WITH MICROBIOTA IN HUMAN GUT MODELS	10.1186/s12915-020-00860-x
SPECIES DELETIONS FROM MICROBIOME CONSORTIA REVEAL KEY METABOLIC INTERACTIONS BETWEEN GUT MICROBES	10.1128/mSystems.00185-19
SPECIFICITY OF INFANT DIGESTIVE CONDITIONS: SOME CLUES FOR DEVELOPING RELEVANT IN VITRO MODELS	10.1080/10408398.2011.640757
SPENT COFFEE (COFFEA ARABICA L.) GROUNDS POSITIVELY MODULATE INDICATORS OF COLONIC MICROBIAL ACTIVITY	10.1016/j.ifset.2019.102286
SPENT COFFEE GROUNDS, AN INNOVATIVE SOURCE OF COLONIC FERMENTABLE COMPOUNDS, INHIBIT INFLAMMATORY MEDIATORS IN VITRO	10.1016/j.foodchem.2016.05.175
SPORES OF BACILLUS COAGULANS GBI-30, 6086 SHOW HIGH GERMINATION, SURVIVAL AND ENZYME ACTIVITY IN A DYNAMIC, COMPUTER-CONTROLLED IN VITRO MODEL OF THE GASTROINTESTINAL TRACT	10.3920/BM2018.0037
SPRAY DRYING PROBIOTICS ALONG WITH MAOLUANG JUICE PLUS TILIACORA TRIANDRA GUM FOR EXPOSURE TO THE IN VITRO GASTROINTESTINAL ENVIRONMENTS	10.1016/j.lwt.2016.12.013
STABILITY AND ACTIVITY OF AN ENTEROBACTER AEROGENES-SPECIFIC BACTERIOPHAGE UNDER SIMULATED GASTRO-INTESTINAL CONDITIONS	10.1007/s00253-004-1585-7
STABILITY AND BIOLOGICAL ACTIVITY OF MERLOT (VITIS VINIFERA) GRAPE POMACE PHYTOCHEMICALS AFTER SIMULATED IN VITRO GASTROINTESTINAL DIGESTION AND COLONIC FERMENTATION	10.1016/j.jff.2017.07.030
STABILITY AND BIOLOGICAL ACTIVITY OF WILD BLUEBERRY (VACCINIUM ANGUSTIFOLIUM) POLYPHENOLS DURING SIMULATED IN VITRO GASTROINTESTINAL DIGESTION	10.1016/j.foodchem.2014.05.135
STABILITY AND MECHANISM OF PHENOLIC COMPOUNDS FROM RASPBERRY EXTRACT UNDER IN VITRO GASTROINTESTINAL DIGESTION	10.1016/j.lwt.2020.110552

STABILITY AND METABOLISM OF ARBUTUS UNEDO BIOACTIVE COMPOUNDS (PHENOLICS AND ANTIOXIDANTS) UNDER IN VITRO DIGESTION AND COLONIC FERMENTATION	10.1016/j.foodchem.2016.01.076
STABILITY OF ANTIOXIDANT PEPTIDES FROM DUCK MEAT AFTER POST-MORTEM AGEING	10.1111/ijfs.13536
STABILITY OF ENZYME-MODIFIED FLAVONOID C-AND O-GLYCOSIDES FROM COMMON BUCKWHEAT SPROUT EXTRACTS DURING IN VITRO DIGESTION AND COLONIC FERMENTATION	10.1021/acs.jafc.1c00542
STABILITY OF VITAMIN B12 WITH THE PROTECTION OF WHEY PROTEINS AND THEIR EFFECTS ON THE GUT MICROBIOME	10.1016/j.foodchem.2018.10.033
STAPHYLOCOCCUS AUREUS MNHF MEDIATES CHOLATE EFFLUX AND FACILITATES SURVIVAL UNDER HUMAN COLONIC CONDITIONS	10.1128/IAI.00238-15
STARCH UTILIZATION BY BACTEROIDES OVATUS ISOLATED FROM THE HUMAN LARGE INTESTINE	10.1007/s002849900184
STIMULATION OF BUTYRATE PRODUCTION THROUGH THE METABOLIC INTERACTION AMONG LACTIC ACID BACTERIA, LACTOBACILLUS ACIDOPHILUS, AND LACTIC ACID-UTILIZING BACTERIA, MEGASPHAERA ELSDENII, IN PORCINE CECAL DIGESTA	10.1111/j.1740-0929.2006.00372.x
STORAGE STABILITY AND SIMULATED GASTROINTESTINAL RELEASE OF SPRAY DRIED GRAPE MARC PHENOLICS	10.1016/j.fbp.2018.08.011
STREPTOCOCCUS THERMOPHILES DMST-H2 PROMOTES RECOVERY IN MICE WITH ANTIBIOTIC-ASSOCIATED DIARRHEA	10.3390/microorganisms8111650
STRUCTURAL CHARACTERIZATION AND IN VITRO GASTROINTESTINAL DIGESTION AND FERMENTATION OF LITCHI POLYSACCHARIDE	10.1016/j.ijbiomac.2019.08.170
STRUCTURAL REARRANGEMENT OF NATIVE AND PROCESSED PEA STARCHES FOLLOWING SIMULATED DIGESTION IN VITRO AND FERMENTATION CHARACTERISTICS OF THEIR RESISTANT STARCH RESIDUES USING HUMAN FECAL INOCULUM	10.1016/j.ijbiomac.2021.01.092
STRUCTURAL, PHYSICOCHEMICAL, AND IN-VITRO RELEASE PROPERTIES OF HYDROGEL BEADS PRODUCED BY OLIGOCHITOSAN AND DE-ESTERIFIED PECTIN FROM YUZU (CITRUS JUNOS) PEEL AS A QUERCETIN DELIVERY SYSTEM FOR COLON TARGET	10.1016/j.foodhyd.2020.106086
STRUCTURE OF BREWER'S SPENT GRAIN LIGNIN AND ITS INTERACTIONS WITH GUT MICROBIOTA IN VITRO	10.1021/acs.jafc.5b05535
STRUCTURE OF Œí-GLUCAN FROM TIBETAN HULL-LESS BARLEY AND ITS IN VITRO FERMENTATION BY HUMAN GUT MICROBIOTA	10.1186/s40538-021-00212-z
STUDIES ON MIXED POPULATIONS OF HUMAN INTESTINAL BACTERIA GROWN IN SINGLE-STAGE AND MULTISTAGE CONTINUOUS CULTURE SYSTEMS	10.1128/aem.55.3.672-678.1989
STUDIES ON THE EFFECT OF SYSTEM RETENTION TIME ON BACTERIAL POPULATIONS COLONIZING A THREE-STAGE CONTINUOUS CULTURE MODEL OF THE HUMAN LARGE GUT USING FISH TECHNIQUES	10.1111/j.1574-6941.2005.00016.x
STUDY OF ANTIOXIDANT CAPACITY AND METABOLIZATION OF QUEBRACHO AND CHESTNUT TANNINS THROUGH IN VITRO GASTROINTESTINAL DIGESTION-FERMENTATION	10.1016/j.jff.2018.07.056
STUDY OF FACTORS INFLUENCING THE BIOACCESSIBILITY OF TRIAZOLONE IN CHERRY TOMATOES USING A STATIC SHIME MODEL	10.3390/ijerph15050993
STUDY OF GROWTH, METABOLISM, AND MORPHOLOGY OF AKKERMANSIA MUCINIPHILA WITH AN IN VITRO ADVANCED BIONIC INTESTINAL REACTOR	10.1186/s12866-021-02111-7
STUDY OF IN VITRO DIGESTION OF TENEBRIO MOLITOR FLOUR FOR EVALUATION OF ITS IMPACT ON THE HUMAN GUT MICROBIOTA	10.1016/j.jff.2019.05.024
STUDY OF PROBAC PRODUCT INFLUENCE ON INFANT MICROBIOTA IN A SINGLE-CHAMBER COLONIC FERMENTATION MODEL GIS1	10.1007/s13213-012-0558-9
STUDY OF PROEXO PRODUCT INFLUENCE ON INFANT MICROBIOTA IN AN IN VITRO COLONIC FERMENTATION SYSTEM	10.1007/s13213-014-0947-3
STUDY OF THE IMPACT OF A DYNAMIC IN VITRO MODEL OF THE COLON (TIM-2) IN THE PHENOLIC COMPOSITION OF TWO MEXICAN SAUCES	10.1016/j.foodres.2020.109917

STUDY OF THE PHYSICOCHEMICAL AND BIOLOGICAL STABILITY OF PEDIOCIN PA-1 IN THE UPPER GASTROINTESTINAL TRACT CONDITIONS USING A DYNAMIC IN VITRO MODEL	10.1111/j.1365-2672.2009.04644.x
STUDY ON CHEMOPREVENTIVE EFFECTS OF RAW AND ROASTED Œí-GLUCAN-RICH WAXY WINTER BARLEY USING AN: IN VITRO HUMAN COLON DIGESTION MODEL	10.1039/c9fo03009c
STUDY ON MECHANISM OF LOW BIOAVAILABILITY OF BLACK TEA THEAFLAVINS BY USING CACO-2 CELL MONOLAYER	10.1080/10717544.2021.1949074
STUDY ON PRODUCTION OF SHORT CHAIN FATTY ACIDS FROM YAM OLIGOSACCHARIDES BY INTESTINAL PROBIOTICS FERMENTATION IN VITRO	10.3969/j.issn.2095-6002.2019.04.007
STUDY ON THE CHARACTERISTICS OF MICROBIAL COMMUNITY IN ANAEROBIC FLUIDIZED BED MEMBRANE BIOREACTOR FOR DOMESTIC WASTEWATER TREATMENT [ ]	10.13671/j.hjkxxb.2019.0041
STUDY ON THE DIGESTION OF MILK WITH PREBIOTIC CARBOHYDRATES IN A SIMULATED GASTROINTESTINAL MODEL	10.1016/j.jff.2017.03.031
STUDY ON THE EFFECT OF JUPI DECOCTION ON THE STRUCTURE OF INTESTINAL FLORA BASED ON SHIME [SHIME]	10.13982/j.mfst.1673-9078.2021.4.0782
SUBLETHAL LEVELS OF ANTIBIOTICS PROMOTE BACTERIAL PERSISTENCE IN EPITHELIAL CELLS	10.1002/advs.201900840
SUBSTRATE-SPECIFIC SELECTIVE CULTURE OF CELLULOSE SPLITTING INTESTINAL BACTERIA OF SMALL LABORATORY RODENTS	10.1007/BF02291462
SUCCESSFUL TREATMENT OF SIMULATED CLOSTRIDIUM DIFFICILE INFECTION IN A HUMAN GUT MODEL BY FIDAXOMICIN FIRST LINE AND AFTER VANCOMYCIN OR METRONIDAZOLE FAILURE	10.1093/jac/dkt347
SUGAR COMPOSITION OF DIETARY FIBRE AND SHORT-CHAIN FATTY ACID PRODUCTION DURING IN VITRO FERMENTATION BY HUMAN BACTERIA	10.1079/BJN19930116
SUPPLEMENTAL EFFECT OF XYLANASE AND MANNANASE ON NUTRIENT DIGESTIBILITY AND GUT HEALTH OF NURSERY PIGS STUDIED USING BOTH IN VIVO AND IN VITRO MODELS	10.1016/j.anifeedsci.2018.07.002
SUPPLEMENTATION OF A PROPIONATE-PRODUCING CONSORTIUM IMPROVES MARKERS OF INSULIN RESISTANCE IN AN IN VITRO MODEL OF GUT-LIVER AXIS	10.1152/ajpendo.00523.2019
SUPPLEMENTATION WITH CHLORELLA VULGARIS, CHLORELLA PROTOTHECOIDES, AND SCHIZOCHYTRIUM SP. INCREASES PROPIONATE-PRODUCING BACTERIA IN IN VITRO HUMAN GUT FERMENTATION	10.1002/jsfa.10321
SURVIVABILITY AND METABOLIC ACTIVITY OF LACTOBACILLUS CASEI 01 INCORPORATING LYCHEE JUICE PLUS INULIN UNDER SIMULATED GASTROINTESTINAL ENVIRONMENT	
SURVIVAL AND METABOLIC ACTIVITY OF PEDIOCIN PRODUCER PEDIOCOCCUS ACIDILACTICI UL5: ITS IMPACT ON INTESTINAL MICROBIOTA AND LISTERIA MONOCYTOGENES IN A MODEL OF THE HUMAN TERMINAL ILEUM	10.1007/s00248-015-0645-0
SURVIVAL AND METABOLIC ACTIVITY OF THE GANEDENBC30 STRAIN OF BACILLUS COAGULANS IN A DYNAMIC IN VITRO MODEL OF THE STOMACH AND SMALL INTESTINE	10.3920/BM2009.0009
SURVIVAL OF A PROBIOTIC-CONTAINING PRODUCT USING CAPSULE-WITHIN-CAPSULE TECHNOLOGY IN AN IN VITRO MODEL OF THE STOMACH AND SMALL INTESTINE (TIM-1)	10.3920/BM2019.0209
SURVIVAL OF CHEESE-RIPENING MICROORGANISMS IN A DYNAMIC SIMULATOR OF THE GASTROINTESTINAL TRACT	10.1016/j.fm.2015.03.002
SURVIVAL OF CLINICAL AND FOOD ISOLATES OF LISTERIA MONOCYTOGENES THROUGH SIMULATED GASTROINTESTINAL TRACT CONDITIONS	10.1089/fpd.2009.0319
SURVIVAL OF COMMERCIAL PROBIOTIC STRAINS IN DARK CHOCOLATE WITH HIGH COCOA AND PHENOLS CONTENT DURING THE STORAGE AND IN A STATIC IN VITRO DIGESTION MODEL	10.1016/j.jff.2017.05.019
SURVIVAL OF FIVE STRAINS OF SHIGA TOXIGENIC ESCHERICHIA COLI IN A SAUSAGE FERMENTATION MODEL AND SUBSEQUENT SENSITIVITY TO STRESS FROM GASTRIC ACID AND INTESTINAL FLUID	10.1155/2017/5176384
SURVIVAL OF IMMOBILIZED PROBIOTICS IN CHOCOLATE DURING STORAGE AND WITH AN IN VITRO GASTROINTESTINAL MODEL	10.1016/j.fbio.2016.09.001

SURVIVAL OF LACTIC ACID BACTERIA FROM FERMENTED MILKS IN AN IN VITRO DIGESTION MODEL EXPLOITING SEQUENTIAL INCUBATION IN HUMAN GASTRIC AND DUODENUM JUICE	10.3168/jds.2011-4705
SURVIVAL OF LACTOBACILLUS ACIDOPHILUS LA-5 AND ESCHERICHIA COLI O157:H7 IN MINAS FRESCAL CHEESE MADE WITH OREGANO AND ROSEMARY ESSENTIAL OILS	10.1016/j.fm.2019.103348
SURVIVAL OF LACTOBACILLUS PARACASEI SUBSP. PARACASEI LBC 81 IN FERMENTED BEVERAGE FROM CHICKPEAS AND COCONUT IN A STATIC IN VITRO DIGESTION MODEL	10.3390/fermentation7030135
SURVIVAL OF LACTOBACILLUS RHAMNOSUS EM1107 IN SIMULATED GASTROINTESTINAL CONDITIONS AND ITS INHIBITORY EFFECT AGAINST PATHOGENIC BACTERIA IN SEMI-HARD GOAT CHEESE	10.1016/j.lwt.2015.05.004
SURVIVAL OF LACTOBACILLUS RHAMNOSUS STRAINS IN THE UPPER GASTROINTESTINAL TRACT	10.1016/j.fm.2010.07.019
SURVIVAL OF LISTERIA MONOCYTOGENES DURING IN VITRO GASTROINTESTINAL DIGESTION AFTER EXPOSURE TO 5 AND 0.5 % SODIUM CHLORIDE	10.1016/j.fm.2018.08.010
SURVIVAL OF LISTERIA MONOCYTOGENES IN A SIMULATED DYNAMIC GASTROINTESTINAL MODEL DURING STORAGE OF INOCULATED BOLOGNA AND SALAMI SLICES IN VACUUM PACKAGES	10.4315/0362-028X-71.10.2014
SURVIVAL OF PROBIOTIC BACTERIA NANOENCAPSULATED WITHIN BIOPOLYMERS IN A SIMULATED GASTROINTESTINAL MODEL	10.1016/j.ifset.2021.102750
SURVIVAL OF PROBIOTIC LACTOBACILLI IN THE UPPER GASTROINTESTINAL TRACT USING AN IN VITRO GASTRIC MODEL OF DIGESTION	10.1016/j.fm.2011.06.007
SURVIVAL OF PROBIOTICS IN PEA PROTEIN-ALGINATE MICROCAPSULES WITH OR WITHOUT CHITOSAN COATING DURING STORAGE AND IN A SIMULATED GASTROINTESTINAL ENVIRONMENT	10.1007/s10068-017-0025-2
SURVIVAL OF PROBIOTICS IN SOYOGHURT PLUS MULBERRY (C.V. CHIANG MAI 60) LEAF EXTRACT DURING REFRIGERATED STORAGE AND THEIR ABILITY TO TOLERATE GASTROINTESTINAL TRANSIT	10.1016/j.lwt.2018.03.027
SYNBIOTIC AMAZONIAN PALM BERRY (AÇAI, EUTERPE OLERACEA MART.) ICE CREAM IMPROVED LACTOBACILLUS RHAMNOSUS GG SURVIVAL TO SIMULATED GASTROINTESTINAL STRESS	10.1039/c6fo00778c
SYNBIOTICS FOR PREVENTION AND TREATMENT OF ATOPIC DERMATITIS: A META-ANALYSIS OF RANDOMIZED CLINICAL TRIALS	10.1001/jamapediatrics.2015.3943
SYNERGISTIC DEGRADATION OF MUCIN BY STREPTOCOCCUS ORALIS AND STREPTOCOCCUS SANGUIS IN MIXED CHEMOSTAT CULTURES	10.1177/00220345910700070401
SYNERGISTIC EFFECTS OF BIFIDOBACTERIUM THERMOPHILUM RBL67 AND SELECTED PREBIOTICS ON INHIBITION OF SALMONELLA COLONIZATION IN THE SWINE PROXIMAL COLON POLYFERMS MODEL	10.1186/s13099-014-0044-y
SYNTHESIS AND IN VITRO DIGESTION AND FERMENTATION OF ACYLATED INULIN	10.1016/j.bcdf.2013.01.004
SYNTHESIS OF ISOMALTOOLIGOSACCHARIDES AND OLIGODEXTRANS IN A RECYCLE MEMBRANE BIOREACTOR BY THE COMBINED USE OF DEXTRANSUCRASE AND DEXTRANASE	10.1002/bit.20257
TECHNOLOGICAL FEATURES OF SACCHAROMYCES CEREVISIAE VAR. BOULARDII FOR POTENTIAL PROBIOTIC WHEAT BEER DEVELOPMENT	10.1016/j.lwt.2020.110233
TECHNOLOGICAL POTENTIAL OF BIFIDOBACTERIUM AESCULAPII STRAINS FOR FERMENTED SOYMILK PRODUCTION	10.1016/j.lwt.2017.11.048
TECHNOLOGICAL, REGULATORY, AND ETHICAL ASPECTS OF IN VITRO MEAT: A FUTURE SLAUGHTER-FREE HARVEST	10.1111/1541-4337.12473
TESTING EDIBLE MUSHROOMS TO INHIBIT THE PANCREATIC LIPASE ACTIVITY BY AN IN VITRO DIGESTION MODEL	10.1111/j.1365-2621.2011.02934.x
TESTING IN VITRO VIABILITY OF A THERMOPHILIC PROBIOTIC BACTERIAL STRAIN IN SIMULATED GASTROINTESTINAL CONDITIONS	10.1007/s13213-013-0734-6
THE ACTIVITY OF UROLITHIN A AND M4 VALEROLACTONE, COLONIC MICROBIOTA METABOLITES OF POLYPHENOLS, IN A PROSTATE CANCER IN VITRO MODEL	10.1055/a-0755-7715
THE ADHESION OF THE GUT MICROBIOTA TO INSOLUBLE DIETARY FIBER FROM SOY HULLS PROMOTED THE PROLIFERATION OF PROBIOTICS IN VITRO	10.1016/j.lwt.2021.112560

THE ALGA EUGLENA GRACILIS STIMULATES FAECALIBACTERIUM IN THE GUT AND CONTRIBUTES TO INCREASED DEFECATION	10.1038/s41598-020-80306-0
THE BIOAVAILABILITY OF SOYBEAN POLYSACCHARIDES AND THEIR METABOLITES ON GUT MICROBIOTA IN THE SIMULATOR OF THE HUMAN INTESTINAL MICROBIAL ECOSYSTEM (SHIME)	10.1016/j.foodchem.2021.130233
THE COLONIZATION OF A SIMULATOR OF THE HUMAN INTESTINAL MICROBIAL ECOSYSTEM BY A PROBIOTIC STRAIN FED ON A FERMENTED OAT BRAN PRODUCT: EFFECTS ON THE GASTROINTESTINAL MICROBIOTA	10.1007/s002530051284
THE COMPOSITION AND ANTIOXIDANT ACTIVITY OF BOUND PHENOLICS IN THREE LEGUMES, AND THEIR METABOLISM AND BIOACCESSIBILITY OF GASTROINTESTINAL TRACT	10.3390/foods9121816
THE COMPOSITION AND METABOLIC ACTIVITY OF CHILD GUT MICROBIOTA DEMONSTRATE DIFFERENTIAL ADAPTATION TO VARIED NUTRIENT LOADS IN AN IN VITRO MODEL OF COLONIC FERMENTATION	10.1111/j.1574-6941.2012.01330.x
THE COTYLEDON CELL WALL AND INTRACELLULAR MATRIX ARE FACTORS THAT LIMIT IRON BIOAVAILABILITY OF THE COMMON BEAN (: PHASEOLUS VULGARIS)	10.1039/c6fo00490c
THE DEGRADATION OF CURCUMINOIDS IN A HUMAN FAECAL FERMENTATION MODEL	10.3109/09637486.2015.1095865
THE DESIGN, OPERATION, AND APPLICATION OF A DYNAMIC GASTRIC MODEL	10.14227/DT190312P15
THE DIGESTIBILITY OF HIBISCUS SABDARIFFA L. POLYPHENOLS USING AN IN VITRO HUMAN DIGESTION MODEL AND EVALUATION OF THEIR ANTIMICROBIAL ACTIVITY	10.3390/nu13072360
THE EFFECT OF 2-FUCOSYLLACTOSE ON SIMULATED INFANT GUT MICROBIOME AND METABOLITES; A PILOT STUDY IN COMPARISON TO GOS AND LACTOSE	10.1038/s41598-019-49497-z
THE EFFECT OF A MODEL MELANOIDIN MIXTURE ON FAECAL BACTERIAL POPULATIONS IN VITRO	10.1017/s0007114599001749
THE EFFECT OF AGAVE FRUCTAN PRODUCTS ON THE ACTIVITY AND COMPOSITION OF THE MICROBIOTA DETERMINED IN A DYNAMIC IN VITRO MODEL OF THE HUMAN PROXIMAL LARGE INTESTINE	10.1016/j.jff.2016.01.018
THE EFFECT OF ALGINATE AND CHITOSAN CONCENTRATIONS ON SOME PROPERTIES OF CHITOSAN-COATED ALGINATE BEADS AND SURVIVABILITY OF ENCAPSULATED LACTOBACILLUS RHAMNOSUS IN SIMULATED GASTROINTESTINAL CONDITIONS AND DURING HEAT PROCESSING	10.1002/jsfa.6541
THE EFFECT OF CELL IMMOBILIZATION ON THE ANTIBACTERIAL ACTIVITY OF LACTOBACILLUS REUTERI DPC16 CELLS DURING PASSAGE THROUGH A SIMULATED GASTROINTESTINAL TRACT SYSTEM	10.1007/s11274-012-1113-3
THE EFFECT OF DIETARY ZINC (II) CHELATE AND ZINC (II) ENRICHED SOYBEAN MEAL ON SELECTED PARAMETERS OF IN VITRO CAECAL FERMENTATION OF LAYING HENS	10.3923/javaa.2012.4051.4057
THE EFFECT OF ENCAPSULATED POWDER OF GOJI BERRY (LYCIUM BARBARUM) ON GROWTH AND SURVIVAL OF PROBIOTIC BACTERIA	10.3390/microorganisms8010057
THE EFFECT OF ENCAPSULATION ON THE STABILITY OF PROBIOTIC BACTERIA IN ICE CREAM AND SIMULATED GASTROINTESTINAL CONDITIONS	10.1007/s12602-018-9485-9
THE EFFECT OF EXTRUSION COOKING OF DIFFERENT STARCH SOURCES ON THE IN VITRO AND IN VIVO DIGESTIBILITY IN GROWING PIGS	10.1016/j.anifeedsci.2006.02.009
THE EFFECT OF FORMULATION OF CURCUMINOIDS ON THEIR METABOLISM BY HUMAN COLONIC MICROBIOTA	10.3390/molecules25040940
THE EFFECT OF IN VITRO GASTROINTESTINAL SIMULATION ON BIOACTIVITIES OF KEFIR	10.1111/ijfs.14274
THE EFFECT OF INTESTINAL MICROBIOTA DYSBIOSIS ON GROWTH AND DETECTION OF CARBAPENEMASE-PRODUCING ENTEROBACTERALES WITHIN AN IN VITRO GUT MODEL	10.1016/j.jhin.2021.04.014
THE EFFECT OF LACTULOSE ON THE SURVIVAL OF LACTOBACILLUS RHAMNOSUS IN THE SIMULATOR OF THE HUMAN INTESTINAL MICROBIAL ECOSYSTEM (SHIME) AND IN VIVO	10.1080/08910600260081739
THE EFFECT OF PROBIOTIC STRAINS ON THE MICROBIOTA OF THE SIMULATOR OF THE HUMAN INTESTINAL MICROBIAL ECOSYSTEM (SHIME)	10.1016/S0168-1605(98)00182-2
THE EFFECT OF RESIDENCE TIME AND FLUID VOLUME TO SOIL MASS (LS) RATIO ON IN VITRO ARSENIC BIOACCESSIBILITY FROM POORLY CRYSTALLINE SCORODITE.	10.1080/10934521003648958

THE EFFECT OF THE UNDIGESTED FRACTION OF MAIZE PRODUCTS ON THE ACTIVITY AND COMPOSITION OF THE MICROBIOTA DETERMINED IN A DYNAMIC IN VITRO MODEL OF THE HUMAN PROXIMAL LARGE INTESTINE	10.1080/07315724.2009.10719798
THE EFFECT OF VARIOUS INULINS AND CLOSTRIDIUM DIFFICILE ON THE METABOLIC ACTIVITY OF THE HUMAN COLONIC MICROBIOTA IN VITRO	10.1080/08910600310018959
THE EFFECT OF: LACTOBACILLUS ACIDOPHILUS AND LACTOBACILLUS CASEI ON THE IN VITRO BIOACCESSIBILITY OF FLAXSEED LIGNANS (LINUM USITATISSIMUM L.)	10.1039/c8fo00390d
THE EFFECTS OF POLYDEXTROSE AND XYLITOL ON MICROBIAL COMMUNITY AND ACTIVITY IN A 4-STAGE COLON SIMULATOR	10.1111/j.1750-3841.2007.00350.x
THE EFFECTS OF PROBIOTIC BACTERIA ON GLYCAEMIC CONTROL IN OVERWEIGHT MEN AND WOMEN: A RANDOMISED CONTROLLED TRIAL	10.1038/ejcn.2013.294
THE EFFICACY AND EFFECT ON GUT MICROBIOTA OF AN AFLATOXIN BINDER AND A FUMONISIN ESTERASE USING AN IN VITRO SIMULATOR OF THE HUMAN INTESTINAL MICROBIAL ECOSYSTEM (SHIME®)	10.1016/j.foodres.2021.110395
THE FATE OF CHICORY ROOT PULP POLYSACCHARIDES DURING FERMENTATION IN THE TNO IN VITRO MODEL OF THE COLON (TIM-2)	10.1016/j.bcdf.2014.06.007
THE FERMENTATION OF LACTULOSE BY COLONIC BACTERIA	10.1099/00221287-128-2-319
THE HARMONIZED INFOGEST IN VITRO DIGESTION METHOD: FROM KNOWLEDGE TO ACTION	10.1016/j.foodres.2015.12.006
THE HMI,Ñ¢ MODULE: A NEW TOOL TO STUDY THE HOST-MICROBIOTA INTERACTION IN THE HUMAN GASTROINTESTINAL TRACT IN VITRO	10.1186/1471-2180-14-133
THE HUMAN MICROBIAL METABOLISM OF QUERCETIN IN DIFFERENT FORMULATIONS: AN IN VITRO EVALUATION	10.3390/foods9081121
THE IMPACT OF FERMENTATION AND IN VITRO DIGESTION ON THE FORMATION OF ANGIOTENSIN-I-CONVERTING ENZYME INHIBITORY ACTIVITY FROM PEA AND WHEY PROTEIN	10.3168/jds.S0022-0302(03)73621-2
THE IMPACT OF MEALS ON A PROBIOTIC DURING TRANSIT THROUGH A MODEL OF THE HUMAN UPPER GASTROINTESTINAL TRACT	10.3920/BM2011.0022
THE INFLUENCE OF BREAST MILK AND INFANT FORMULAE HYDROLYSATES ON BACTERIAL ADHESION AND CACO-2 CELLS FUNCTIONING	10.1016/j.foodres.2016.09.022
THE INFLUENCE OF STAPHYLOCOCCUS AUREUS ON GUT MICROBIAL ECOLOGY IN AN IN VITRO CONTINUOUS CULTURE HUMAN COLONIC MODEL SYSTEM	10.1371/journal.pone.0023227
THE INTESTINAL BARRIER IN IRRITABLE BOWEL SYNDROME: SUBTYPE-SPECIFIC EFFECTS OF THE SYSTEMIC COMPARTMENT IN AN IN VITRO MODEL	10.1371/journal.pone.0123498
THE LINK BETWEEN THE GUT MICROBIOTA AND PARKINSON'S DISEASE: A SYSTEMATIC MECHANISM REVIEW WITH FOCUS ON Œë-SYNUCLEIN TRANSPORT	10.1016/j.brainres.2021.147609
THE MECHANISM OF LOWERING CHOLESTEROL ABSORPTION BY CALCIUM STUDIED BY USING AN IN VITRO DIGESTION MODEL	10.1039/c5fo00856e
THE METABOLIC ACTIVITY OF FECAL MICROBIOTA FROM HEALTHY INDIVIDUALS AND PATIENTS WITH INFLAMMATORY BOWEL DISEASE	10.1023/B:DDAS.0000020508.64440.73
THE METABOLOMIC-GUT-CLINICAL AXIS OF MANKAI PLANT-DERIVED DIETARY POLYPHENOLS	10.3390/nu13061866
THE MICROBIAL COMMUNITY OF A PASSIVE BIOCHEMICAL REACTOR TREATING ARSENIC, ZINC, AND SULFATE-RICH SEEPAGE	10.3389/fbioe.2015.00027
THE MONITORING, VIA AN IN VITRO DIGESTION SYSTEM, OF THE BIOACTIVE CONTENT OF VEGETABLE JUICE FERMENTED WITH SACCHAROMYCES CEREVISIAE AND SACCHAROMYCES BOULARDII	10.1111/jfpp.12704
THE PREBIOTIC EFFECT OF A-1,2 BRANCHED, LOW MOLECULAR WEIGHT DEXTRAN IN THE BATCH AND CONTINUOUS FAECAL FERMENTATION SYSTEM	10.1016/j.jff.2013.09.015
THE PRENYLFLAVONOID ISOXANTHOHUMOL FROM HOPS (HUMULUS LUPULUS L.) IS ACTIVATED INTO THE POTENT PHYTOESTROGEN 8-PRENYLNARINGENIN IN VITRO AND IN THE HUMAN INTESTINE	10.1093/jn/136.7.1862

THE PRESENCE OF PULSES WITHIN A MEAL CAN ALTER FAT-SOLUBLE VITAMIN BIOAVAILABILITY	10.1002/mnfr.201801323
THE RECOVERY, CATABOLISM AND POTENTIAL BIOACTIVITY OF POLYPHENOLS FROM CARROT SUBJECTED TO IN VITRO SIMULATED DIGESTION AND COLONIC FERMENTATION	10.1016/j.foodres.2021.110263
THE REGULATION OF SODIUM ALGINATE ON THE STABILITY OF OVALBUMIN-PECTIN COMPLEXES FOR VD3 ENCAPSULATION AND IN VITRO SIMULATED GASTROINTESTINAL DIGESTION STUDY	10.1016/j.foodres.2020.110011
THE REGULATORY EFFECTS OF WHEY RETENTATE FROM BIFIDOBACTERIA FERMENTED MILK ON THE MICROBIOTA OF THE SIMULATOR OF THE HUMAN INTESTINAL MICROBIAL ECOSYSTEM (SHIME)	10.1046/j.1365-2672.2001.01482.x
THE RELATIONSHIP BETWEEN INSTABILITY OF H2 PRODUCTION AND COMPOSITIONS OF BACTERIAL COMMUNITIES WITHIN A DARK FERMENTATION FLUIDIZED-BED BIOREACTOR	10.1002/bit.21299
THE RELEVANCE OF A DIGESTIBILITY EVALUATION IN THE ALLERGENICITY RISK ASSESSMENT OF NOVEL PROTEINS. OPINION OF A JOINT INITIATIVE OF COST ACTION IMPARAS AND COST ACTION INFOGEST	10.1016/j.fct.2019.04.052
THE RESISTANCE OF BACILLUS, BIFIDOBACTERIUM, AND LACTOBACILLUS STRAINS WITH CLAIMED PROBIOTIC PROPERTIES IN DIFFERENT FOOD MATRICES EXPOSED TO SIMULATED GASTROINTESTINAL TRACT CONDITIONS	10.1016/j.foodres.2019.108542
THE ROLE OF METAL OXIDE NANOPARTICLES,: ESCHERICHIA COLI, AND LACTOBACILLUS RHAMNOSUS ON SMALL INTESTINAL ENZYME ACTIVITY	10.1039/d0en01001d
THE SAFETY AND TOLERABILITY OF A POTENTIAL ALGINATE-BASED IRON CHELATOR; RESULTS OF A HEALTHY PARTICIPANT STUDY	10.3390/nu11030674
THE SIMPLIFIED HUMAN INTESTINAL MICROBIOTA (SIHUMIX) SHOWS HIGH STRUCTURAL AND FUNCTIONAL RESISTANCE AGAINST CHANGING TRANSIT TIMES IN IN VITRO BIOREACTORS	10.3390/microorganisms7120641
THE SMALLEST INTESTINE (TSI) - A LOW VOLUME IN VITRO MODEL OF THE SMALL INTESTINE WITH INCREASED THROUGHPUT	10.1093/femsle/fny231
THE SURVIVAL OF AND CYTOKINE INDUCTION BY LACTIC ACID BACTERIA AFTER PASSAGE THROUGH A GASTROINTESTINAL MODEL	
THE SURVIVAL OF IRRADIATED LACTOBACILLI IN THE SIMULATED GASTROINTESTINAL CONDITIONS WITH ANTIBIOTIC CEFTAZIDIME	10.1111/lam.13080
THE USE OF A MINI-BIOREACTOR FERMENTATION SYSTEM AS A REPRODUCIBLE, HIGH-THROUGHPUT EX VIVO BATCH MODEL OF THE DISTAL COLON	10.3389/fmicb.2018.01844
THE USE OF A MODEL ILEUM TO INVESTIGATE THE EFFECTS OF NOVEL AND EXISTING ANTIMICROBIALS ON INDIGENOUS PORCINE GASTROINTESTINAL MICROFLORA: USING VANCOMYCIN AS AN EXAMPLE	10.1016/S0377-8401(02)00286-9
THE USE OF IN VITRO MODELS TO ASSESS INTERACTIONS BETWEEN HUMAN MICROBIOTA AND GENETICALLY MODIFIED MICROORGANISMS	10.3109/08910609509140161
THERMO-TOLERANT SACCHAROMYCES CEREVISIAE VAR. BOULARDII COATED CORNFLAKES AS A POTENTIAL PROBIOTIC VEHICLE	10.1016/j.fbio.2020.100668
THERMOPLASTIC POLYURETHANES FOR THE MANUFACTURING OF HIGHLY DOSED ORAL SUSTAINED RELEASE MATRICES VIA HOT MELT EXTRUSION AND INJECTION MOLDING	10.1016/j.ejpb.2014.11.003
THERMORESPONSIVE BACTERIOPHAGE NANOCARRIER AS A GENE DELIVERY VECTOR TARGETED TO THE GASTROINTESTINAL TRACT	10.1016/j.omtn.2018.04.012
THREE PROTECTIVE AGENTS FOR PECTIN-RICE BRAN CAPSULES FOR ENCAPSULATING LACTOBACILLUS PLANTARUM	10.1016/j.fbio.2016.10.001
THREE-DIMENSIONAL IN VITRO GUT MODEL ON A VILLI-SHAPED COLLAGEN SCAFFOLD	10.1007/s13206-017-1307-8
THREE-STAGE CONTINUOUS CULTURE SYSTEM WITH A SELF-GENERATED ANAEROBIA TO STUDY THE REGIONALIZED METABOLISM OF THE HUMAN GUT MICROBIOTA	10.1016/j.mimet.2013.11.015
TIGECYCLINE DOES NOT INDUCE PROLIFERATION OR CYTOTOXIN PRODUCTION BY EPIDEMIC CLOSTRIDIUM DIFFICILE STRAINS IN A HUMAN GUT MODEL	10.1093/jac/dkl364

TIO2 NANOPARTICLES AND COMMENSAL BACTERIA ALTER MUCUS LAYER THICKNESS AND COMPOSITION IN A GASTROINTESTINAL TRACT MODEL	10.1002/smll.202000601
TO POOL OR NOT TO POOL? IMPACT OF THE USE OF INDIVIDUAL AND POOLED FECAL SAMPLES FOR IN VITRO FERMENTATION STUDIES	10.1016/j.mimet.2014.08.022
TO PRE-CHALLENGE LACTIC ACID BACTERIA WITH SIMULATED GASTROINTESTINAL CONDITIONS IS A SUITABLE APPROACH TO STUDYING POTENTIAL PROBIOTIC PROPERTIES	10.1016/j.mimet.2014.09.005
TOWARD AN ACCESSIBLE AND ROBUST IN VITRO APPROACH TO EVALUATE BACTERIAL VIABILITY IN THE UPPER GASTRO-INTESTINAL TRACT: A GASTRO-INTESTINAL DIGESTIVE SIMULATOR (GIDS) COMBINED WITH ALTERNATIVE METHODS TO PLATING	10.1016/j.jff.2019.05.026
TOWARDS AN IMPROVED GLOBAL ANTIOXIDANT RESPONSE METHOD (GAR+): PHYSIOLOGICAL-RESEMBLING IN VITRO DIGESTION-FERMENTATION METHOD	10.1016/j.foodchem.2017.07.024
TOWARDS UNDERSTANDING THE MODULATION OF IN VITRO GASTROINTESTINAL LIPOLYSIS KINETICS THROUGH EMULSIONS WITH MIXED INTERFACES	10.1016/j.foodhyd.2021.107240
TOXICITY ASSESSMENT OF NANO-ZNO EXPOSURE ON THE HUMAN INTESTINAL MICROBIOME, METABOLIC FUNCTIONS, AND RESISTOME USING AN IN VITRO COLON SIMULATOR	10.1021/acs.est.1c00573
TRACKING THE FATE OF PASTA (T. DURUM SEMOLINA) IMMUNOGENIC PROTEINS BY IN VITRO SIMULATED DIGESTION	10.1021/jf505461x
TRADITIONAL AND FLAVORED KOMBUCHAS WITH PITANGA AND UMBU-CAJÁ PULPS: CHEMICAL PROPERTIES, ANTIOXIDANTS, AND BIOACTIVE COMPOUNDS	10.1016/j.fbio.2021.101380
TRADITIONAL AND NON-CONVENTIONAL PASTA-MAKING PROCESSES: EFFECT ON IN VITRO STARCH DIGESTIBILITY	10.3390/foods10050921
TRANSCRIPTIONAL ANALYSIS OF GENES ASSOCIATED WITH STRESS AND ADHESION IN LACTOBACILLUS ACIDOPHILUS NCFM DURING THE PASSAGE THROUGH AN IN VITRO GASTROINTESTINAL TRACT MODEL	10.1159/000316421
TRANSCRIPTOME ANALYSIS OF ACTIVATED SLUDGE MICROBIOMES REVEALS AN UNEXPECTED ROLE OF MINORITY NITRIFIERS IN CARBON METABOLISM	10.1038/s42003-019-0418-2
TRANSFER OF ANTIBIOTIC RESISTANCE PLASMID FROM COMMENSAL E. COLI TOWARDS HUMAN INTESTINAL MICROBIOTA IN THE M-SHIME: EFFECT OF E. COLI DOSIS, HUMAN INDIVIDUAL AND ANTIBIOTIC USE	10.3390/life11030192
TRANSFORMATION OF ARSENIC SPECIES DURING IN VITRO GASTROINTESTINAL DIGESTION OF VEGETABLES	10.1021/jf4034738
TRANSIT TIME AFFECTS THE COMMUNITY STABILITY OF LACTOBACILLUS AND BIFIDOBACTERIUM SPECIES IN AN IN VITRO MODEL OF HUMAN COLONIC MICROBIOTIA	10.3109/10731199.2011.622280
TREATMENT WITH A SPORE-BASED PROBIOTIC CONTAINING FIVE STRAINS OF BACILLUS INDUCED CHANGES IN THE METABOLIC ACTIVITY AND COMMUNITY COMPOSITION OF THE GUT MICROBIOTA IN A SHIME¬Æ MODEL OF THE HUMAN GASTROINTESTINAL SYSTEM	10.1016/j.foodres.2021.110676
TREHALOSE-INDUCED REMODELLING OF THE HUMAN MICROBIOTA AFFECTS CLOSTRIDIOIDES DIFFICILE INFECTION OUTCOME IN AN IN VITRO COLONIC MODEL: A PILOT STUDY	10.3389/fcimb.2021.670935
TYPE 1 FIMBRIAE CONTRIBUTE TO CATHETER-ASSOCIATED URINARY TRACT INFECTIONS CAUSED BY ESCHERICHIA COLI	10.1128/JB.00985-13
UHPLC-ESI-QTOF-MS PROFILE OF POLYPHENOLS IN GOJI BERRIES (LYCIUM BARBARUM L.) AND ITS DYNAMICS DURING IN VITRO GASTROINTESTINAL DIGESTION AND FERMENTATION	10.1016/j.jff.2017.11.042
ULTRA-HIGH PRESSURE TREATMENT IMPROVING PHYSICOCHEMICAL PROPERTIES AND PROBIOTIC COMMUNITY OF TARTARY BUCKWHEAT STARCH [ ]	10.11975/j.issn.1002-6819.2019.02.036
ULTRASENSITIVE DIRECT QUANTIFICATION OF NUCLEOBASE MODIFICATIONS IN DNA BY SURFACE-ENHANCED RAMAN SCATTERING: THE CASE OF CYTOSINE	10.1002/anie.201507682
UNCOVERING THE POTENTIAL OF TERMITE GUT MICROBIOME FOR LIGNOCELLULOSE BIOCONVERSION IN ANAEROBIC BATCH BIOREACTORS	10.3389/fmicb.2017.02623
UNDERSTANDING THE BIOREACTOR	10.1007/s004490100263

UNDERSTANDING THE IMPACT OF CHIA SEED MUCILAGE ON HUMAN GUT MICROBIOTA BY USING THE DYNAMIC GASTROINTESTINAL MODEL SIMGI®	10.1016/j.jff.2018.09.028
UNDERSTANDING THE PREBIOTIC POTENTIAL OF DIFFERENT DIETARY FIBERS USING AN IN VITRO CONTINUOUS ADULT FERMENTATION MODEL (POLYFERMS)	10.1038/s41598-018-22438-y
UNEXPECTED CONSEQUENCES OF ADMINISTERING BACTERIOCINOGENIC PROBIOTIC STRAINS FOR SALMONELLA POPULATIONS, REVEALED BY AN IN VITRO COLONIC MODEL OF THE CHILD GUT	10.1099/mic.0.042036-0
UNEXPECTED STABILITY OF BACTEROIDETES AND FIRMICUTES COMMUNITIES IN LABORATORY BIOGAS REACTORS FED WITH DIFFERENT DEFINED SUBSTRATES	10.1128/AEM.06394-11
UNLOCKING THE POTENTIAL OF ORGAN-ON-CHIP MODELS THROUGH PUMPLESS AND TUBELESS MICROFLUIDICS	10.1002/adhm.201901784
UNTARGETED METABOLOMIC EVALUATION OF MANGO BAGASSE AND MANGO BAGASSE BASED CONFECTION UNDER IN VITRO SIMULATED COLONIC FERMENTATION	10.1016/j.jff.2019.01.032
UNTARGETED METABOLOMICS REVEALS CHANGES IN PHENOLIC PROFILE FOLLOWING IN VITRO LARGE INTESTINE FERMENTATION OF NON-EDIBLE PARTS OF PUNICA GRANATUM L.	10.1016/j.foodres.2019.108807
UREA REMOVAL IN ROSÉ AND RED WINES BY IMMOBILISED ACID UREASE IN A PACKED BED REACTOR	10.1016/j.fbp.2020.12.008
USE OF A COMBINATION OF IN VITRO MODELS TO INVESTIGATE THE IMPACT OF CHLORPYRIFOS AND INULIN ON THE INTESTINAL MICROBIOTA AND THE PERMEABILITY OF THE INTESTINAL MUCOSA	10.1007/s11356-018-2332-4
USE OF A CONTINUOUS CULTURE FERMENTATION SYSTEM TO INVESTIGATE THE EFFECT OF GANEDENBC30 (BACILLUS COAGULANS GBI-30, 6086) SUPPLEMENTATION ON PATHOGEN SURVIVAL IN THE HUMAN GUT MICROBIOTA	10.1016/j.anaerobe.2010.12.006
USE OF A CONTINUOUS-FLOW ANAEROBIC CULTURE TO CHARACTERIZE ENTERIC VIRULENCE GENE EXPRESSION	10.1128/IAI.72.7.3793-3802.2004
USE OF A THREE-STAGE CONTINUOUS CULTURE SYSTEM TO STUDY THE EFFECT OF MUCIN ON DISSIMILATORY SULFATE REDUCTION AND METHANOGENESIS BY MIXED POPULATIONS OF HUMAN GUT BACTERIA.	10.1128/aem.54.11.2750-2755.1988
USE OF CHANGESTAT FOR GROWTH RATE STUDIES OF GUT MICROBIOTA	10.3389/fbioe.2020.00024
USE OF CONTINUOUS CULTURE TO STUDY THE GASTRIC MICROFLORA OF A HYPOCHLORHYDRIC PATIENT	10.1016/0887-2333(87)90033-6
USE OF IMAGING TECHNIQUES TO IDENTIFY EFFICIENT CONTROLLED RELEASE SYSTEMS OF: LACTOBACILLUS RHAMNOSUS GG DURING IN VITRO DIGESTION	10.1039/c6fo01737a
USE OF LACTOBACILLUS CRISPATUS TO PRODUCE A PROBIOTIC CHEESE AS POTENTIAL GENDER FOOD FOR PREVENTING GYNAECOLOGICAL INFECTIONS	10.1371/journal.pone.0208906
USE OF THE DYNAMIC GASTRIC MODEL AS A TOOL FOR INVESTIGATING FED AND FASTED SENSITIVITIES OF LOW POLYMER CONTENT HYDROPHILIC MATRIX FORMULATIONS	10.1016/j.ijpharm.2016.06.034
USE OF THE DYNAMIC GASTRO-INTESTINAL MODEL TIM TO EXPLORE THE SURVIVAL OF THE YOGURT BACTERIUM STREPTOCOCCUS THERMOPHILUS AND THE METABOLIC ACTIVITIES INDUCED IN THE SIMULATED HUMAN GUT	10.1016/j.fm.2015.05.007
VALIDATED HIGH RESOLUTION MASS SPECTROMETRY-BASED APPROACH FOR METABOLOMIC FINGERPRINTING OF THE HUMAN GUT PHENOTYPE	10.1021/acs.analchem.5b02688
VALIDATION OF A THREE-STAGE COMPOUND CONTINUOUS CULTURE SYSTEM FOR INVESTIGATING THE EFFECT OF RETENTION TIME ON THE ECOLOGY AND METABOLISM OF BACTERIA IN THE HUMAN COLON	10.1007/s002489900072
VALIDATION OF AN IN VITRO DIGESTIVE SYSTEM FOR STUDYING MACRONUTRIENT DECOMPOSITION IN HUMANS	10.3945/jn.111.148635
VALIDATION OF THE SIMULATOR OF THE HUMAN INTESTINAL MICROBIAL ECOSYSTEM (SHIME) REACTOR USING MICROORGANISM-ASSOCIATED ACTIVITIES	10.3109/08910609409141354
VALORISATION OF REJECTED UNRIPE PLANTAIN FRUITS OF MUSA AAB SIMMONDS: FROM NUTRITIONAL CHARACTERISATION TO THE CONCEPTUAL PROCESS DESIGN FOR PREBIOTIC PRODUCTION	10.1039/d0fo03379k

VALORIZATION OF PERSIMMON AND BLUEBERRY BYPRODUCTS TO OBTAIN FUNCTIONAL POWDERS: IN VITRO DIGESTION AND FERMENTATION BY GUT MICROBIOTA	10.1021/acs.jafc.0c02088
VANCOMYCIN EXPOSURE CAUSED OPPORTUNISTIC PATHOGENS BLOOM IN INTESTINAL MICROBIOME BY SIMULATOR OF THE HUMAN INTESTINAL MICROBIAL ECOSYSTEM (SHIME)	10.1016/j.envpol.2020.114399
VARIABILITY OF ARSENIC BIOACCESSIBILITY AND METABOLISM IN SOILS BY HUMAN GUT MICROBIOTA USING DIFFERENT IN VITRO METHODS COMBINED WITH SHIME	10.1016/j.scitotenv.2016.06.071
VARIABILITY OF CHROMIUM BIOACCESSIBILITY AND SPECIATION IN VEGETABLES: THE INFLUENCE OF IN VITRO METHODS, GUT MICROBIOTA AND VEGETABLE SPECIES	10.1016/j.foodchem.2018.10.120
VARIATION OF GLUCORAPHANIN METABOLISM IN VIVO AND EX VIVO BY HUMAN GUT BACTERIA	10.1017/S0007114511000274
VASQCHIP: A NOVEL MICROFLUIDIC, ARTIFICIAL BLOOD VESSEL SCAFFOLD FOR VASCULARIZED 3D TISSUES	10.1002/admt.201700246
VENTURING INTO IN VITRO PHYSIOLOGICAL UPPER GI SYSTEM FOCUSING ON THE MOTILITY EFFECT PROVIDED BY A MECHANISED RAT STOMACH MODEL	10.1007/s13228-012-0018-9
VERMICOMPOSTING SMART CLOSED REACTOR DESIGN AND PERFORMANCE ASSESSMENT BY USING SEWAGE SLUDGE	10.1007/s12649-021-01426-w
VIABILITY AND RESISTANCE OF LACTOBACILLI ISOLATED FROM COCOA FERMENTATION TO SIMULATED GASTROINTESTINAL DIGESTIVE STEPS IN SOY YOGURT	10.1111/1750-3841.12326
VIABILITY AND STABILITY EVALUATION OF LACTOBACILLUS CASEI LC03 CO-ENCAPSULATED WITH RED ONION (ALLIUM CEPA L.) PEEL EXTRACT	10.1016/j.lwt.2021.112434
VIABILITY OF LACTOBACILLUS DELBRUECKII UNDER HUMAN GASTROINTESTINAL CONDITIONS SIMULATED IN VITRO	10.3844/ajabssp.2010.37.42
VIABILITY OF MICROENCAPSULATED: AKKERMANSIA MUCINIPHILA AND LACTOBACILLUS PLANTARUM DURING FREEZE-DRYING, STORAGE AND IN VITRO SIMULATED UPPER GASTROINTESTINAL TRACT PASSAGE	10.1039/c8fo01331d
VIRAL SURROGATES IN POTABLE REUSE APPLICATIONS: EVALUATION OF A MEMBRANE BIOREACTOR AND FULL ADVANCED TREATMENT	10.1061/(ASCE)EE.1943-7870.0001617
VITAMIN B12 ENRICHED IN SPINACH AND ITS EFFECTS ON GUT MICROBIOTA	10.1021/acs.jafc.0c07597
W27 IGA SUPPRESSES GROWTH OF ESCHERICHIA IN AN IN VITRO MODEL OF THE HUMAN INTESTINAL MICROBIOTA	10.1038/s41598-021-94210-8
WEANING-ASSOCIATED FEED DEPRIVATION STRESS CAUSES MICROBIOTA DISRUPTIONS IN A NOVEL MUCIN-CONTAINING IN VITRO MODEL OF THE PIGLET COLON (MPIGUT-IVM)	10.1186/s40104-021-00584-0
WEED SEED SURVIVAL DURING ANAEROBIC DIGESTION IN BIOGAS PLANTS	10.1007/s12229-013-9118-7
WESTERNIZED DIETS LOWER ARSENIC GASTROINTESTINAL BIOACCESSIBILITY BUT INCREASE MICROBIAL ARSENIC SPECIATION CHANGES IN THE COLON	10.1016/j.chemosphere.2014.08.010
WHAT IS THE GOLD STANDARD MODEL FOR ALZHEIMER'S DISEASE DRUG DISCOVERY AND DEVELOPMENT?	10.1080/17460441.2021.1960502
WHEAT BRAN THERMAL TREATMENT IN A HOT AIR OVEN DOES NOT AFFECT THE FERMENTATION AND COLONISATION PROCESS BY HUMAN FAECAL MICROBIOTA	10.1016/j.jff.2019.103440
WHOLE BLUEBERRY AND ISOLATED POLYPHENOL-RICH FRACTIONS MODULATE SPECIFIC GUT MICROBES IN AN IN VITRO COLON MODEL AND IN A PILOT STUDY IN HUMAN CONSUMERS	10.3390/nu12092800
WHOLE TIBETAN HULL-LESS BARLEY EXHIBIT STRONGER EFFECT ON PROMOTING GROWTH OF GENUS BIFIDOBACTERIUM THAN REFINED BARLEY IN VITRO	10.1111/1750-3841.14086
WHOLEGRAIN OAT-BASED CEREALS HAVE PREBIOTIC POTENTIAL AND LOW GLYCAEMIC INDEX	10.1017/S0007114512000281
WOLFFIA GLOBOSA–MANKAI PLANT-BASED PROTEIN CONTAINS BIOACTIVE VITAMIN B12 AND IS WELL ABSORBED IN HUMANS	10.3390/nu12103067
XYLO-OLIGOSACCHARIDES ENHANCE THE GROWTH OF BIFIDOBACTERIA AND BIFIDOBACTERIUM LACTIS IN A SIMULATED COLON MODEL	10.3920/BM2009.0025
XYLO-OLIGOSACCHARIDES FROM SUGARCANE SHOW PREBIOTIC POTENTIAL IN A DYNAMIC COMPUTER-CONTROLLED IN VITRO MODEL OF THE ADULT HUMAN LARGE INTESTINE	10.3920/BM2019.0159

YIELDS OF THREE ACIDS DURING SIMULATED FERMENTATION OF INULIN AND XYLO-OLIGOSACCHARIDES ENHANCED BY SIX EXOGENOUS STRAINS	10.1007/s11694-016-9439-4
ZNO NANOPARTICLES AFFECT NUTRIENT TRANSPORT IN AN IN VITRO MODEL OF THE SMALL INTESTINE	10.1016/j.fct.2018.11.048

Table S7: Duplicated term in the searches.

N	DOI	TITLE	NAME
2	10.1002/(sici)1097-0010(199606)71:2<209::aid-jsfa571>3.3.co;2-w	in vitro method for quantification of the fermentation of starch by human faecal bacteria	In-vitro Model
2	10.1002/(SICI)1097-0010(199702)73:2<149::AID-JSFA685>3.0.CO;2-L	human colonic bacterial degradability of dietary fibres from sea-lettuce ulva sp	Fermenter
3	10.1002/(SICI)1097-0010(199705)74:1<99::AID-JSFA775>3.0.CO;2-G	estimation of the bioavailability of iron and phosphorus in cereals using a dynamic in vitro gastrointestinal model	In-vitro Digestion
2	10.1002/(SICI)1097-0010(199807)77:3<327::AID-JSFA41>3.0.CO;2-5	gastrointestinal or simulated in vitro digestion changes dietary fibre properties and their fermentation	In-vitro Digestion
2	10.1002/(SICI)1234-987X(199605)9:3<97::AID-MEH414>3.3.CO;2-Z	development of an in vitro continuous flow culture model of the murine intestinal tract	Continous Culture
2	10.1002/ajpa.22805	assessing digestibility of hadza tubers using a dynamic in-vitro model	In-vitro Digestion
2	10.1002/biof.1084	a comparison of the anticancer properties of isoxanthohumol and 8-prenylnaringenin using in vitro models of colon cancer	In-vitro Model
2	10.1002/biot.201400004	a novel dual-flow bioreactor simulates increased fluorescein permeability in epithelial tissue barriers	In-vitro Model
2	10.1002/biot.201900523	glycosidic linkage structures influence dietary fiber fermentability and propionate production by human colonic microbiota in vitro	In-vitro Model
4	10.1002/bit.20257	synthesis of isomaltooligosaccharides and oligodextrans in a recycle membrane bioreactor by the combined use of dextransucrase and dextranase	Reactor
2	10.1002/bit.21299	the relationship between instability of h2 production and compositions of bacterial communities within a dark fermentation fluidized-bed bioreactor	Reactor
6	10.1002/bit.27902	a novel scalable and modular bioreactor design for dynamic simulation of the digestive tract	Gut Model
6	10.1002/bmc.1700	hplc-icp-ms method development to monitor arsenic speciation changes by human gut microbiota	Reactor
2	10.1002/cbin.10440	activated endothelial cells limit inflammatory response but increase chemoattractant potential and bacterial clearance by human monocytes	In-vitro Model
2	10.1002/cyto.a.23965	bacterial community diversity dynamics highlight degrees of nestedness and turnover patterns	Bioreactor
2	10.1002/ece3.6305	effects of diet habitat and phylogeny on the fecal microbiome of wild african savanna loxodonta africana and forest elephants lcyclotis	Fermenter
2	10.1002/hep.1840120228	nonabsorbable disaccharides plus neomycin in hepatic encephalopathy do they enhance each other	Fermenter
2	10.1002/JPER.17-0437	oral prebiotics and the influence of environmental conditions in vitro	Chemostat
2	10.1002/JPER.18-0751	in vitro beneficial effects of streptococcus dentisani as potential oral probiotic for periodontal diseases	In-vitro Model
4	10.1002/jps.24103	on the colonic bacterial metabolism of azo-bonded prodrugs of 5-aminosalicylic acid	In-vitro Model

3	10.1002/jsfa.10321	supplementation with chlorella vulgaris chlorella protothecoides and schizochytrium sp increases propionate-producing bacteria in in vitro human gut fermentation	In-vitro Digestion
4	10.1002/jsfa.10694	effect of lactic fermentation on soy protein digestive pattern assessed by an in vitro dynamic gastrointestinal digestion model and the influence on human faecal microbiota	Gastrointestinal Model
4	10.1002/jsfa.11124	role of non-thermal treatments and fermentation with probiotic lactobacillus plantarum on in vitro bioaccessibility of bioactives from vegetable juice	Simulated Gastrointestinal
2	10.1002/jsfa.11149	evaluation of acrylamide-removing properties of bacterial consortia under simulated gastrointestinal conditions	Simulated Gastrointestinal
2	10.1002/jsfa.11600	digestive properties of half-fin anchovy hydrolysatesglucose maillard reaction products and modulation effects on intestinal microbiota	Simulated Gastrointestinal
2	10.1002/jsfa.3033	peniophora lycii phytase is stabile and degrades phytate and solubilises minerals in vitro during simulation of gastrointestinal digestion in the pig	In-vitro Digestion
3	10.1002/jsfa.6030	activities of free and encapsulated lactobacillus acidophilus la5 or lactobacillus casei 01 in processed longan juices on exposure to simulated gastrointestinal tract	Simulated Gastrointestinal
2	10.1002/jsfa.6541	the effect of alginate and chitosan concentrations on some properties of chitosan-coated alginate beads and survivability of encapsulated lactobacillus rhamnosus in simulated gastrointestinal conditions and during heat processing	Simulated Gastrointestinal
4	10.1002/jsfa.8208	bioaccessibility of selenium from cooked rice as determined in a simulator of the human intestinal tract shime	Shime
2	10.1002/jsfa.9294	release of multifunctional peptides from kiwicha amaranthus caudatus protein under in vitro gastrointestinal digestion	Simulated Gastrointestinal
2	10.1002/jsfa.9988	is there an impact of the dairy matrix on the survival of lactobacillus casei lc-1 during shelf life and simulated gastrointestinal conditions	Simulated Gastrointestinal
2	10.1002/mnfr.200600106	in vitro fermentability of differently digested resistant starch preparations	In-vitro Model
3	10.1002/mnfr.201000360	in vitro bioaccessibility and gut biotransformation of polyphenols present in the water-insoluble cocoa fraction	In-vitro Digestion
8	10.1002/mnfr.201400124	faecal microbial metabolism of olive oil phenolic compounds in vitro and in vivo approaches	In-vitro Colon
4	10.1002/mnfr.201600150	high-fiber and high-protein diets shape different gut microbial communities which ecologically behave similarly under stress conditions as shown in a gastrointestinal simulator	Shime
2	10.1002/mnfr.201600928	bioaccessibility bioavailability and anti-inflammatory effects of anthocyanins from purple root vegetables using mono- and co-culture cell models	In-vitro Gastrointestinal
6	10.1002/mnfr.201700223	effect of simulated gastrointestinal digestion and fermentation on polyphenolic content and bioactivity of brown seaweed phlorotannin-rich extracts	Simulated Gastrointestinal
6	10.1002/mnfr.201700881	a critical evaluation of in vitro hesperidin 2s bioavailability in a model combining luminal microbial digestion and caco-2 cell absorption in comparison to a randomized controlled human trial	In-vitro Model

5	10.1002/mnfr.201800607	aronia aronia melanocarpa polyphenols modulate the microbial community in a simulator of the human intestinal microbial ecosystem shime and decrease secretion of proinflammatory markers in a caco-2endothelial cell coculture model	Shime
6	10.1002/mnfr.201800722	polyphenols and tryptophan metabolites activate the aryl hydrocarbon receptor in an in vitro model of colonic fermentation	In-vitro Model
2	10.1002/mnfr.201801323	the presence of pulses within a meal can alter fat-soluble vitamin bioavailability	In-vitro Digestion
4	10.1002/mnfr.202000030	impact of extreme obesity and diet-induced weight loss on the fecal metabolome and gut microbiota	In-vitro Model
2	10.1002/nbm.1418	profiling human gut bacterial metabolism and its kinetics using u-13cglucose and nmr	In-vitro Model
2	10.1002/pat.4658	protein-oligosaccharide conjugates as novel prebiotics	Simulated Gastrointestinal
4	10.1002/smll.202000601	tio2 nanoparticles and commensal bacteria alter mucus layer thickness and composition in a gastrointestinal tract model	In-vitro Model
2	10.1002/star.201200091	comparative fermentation of insoluble carbohydrates in an in vitro human feces model spiked with lactobacillus acidophilus ncfm	Continous Fermentation
2	10.1002/star.201200100	in vitro fermentation of a retrograded maize starch by healthy adult fecal extract and impacts of exogenous microorganisms on three acids production	Simulated Colon
2	10.1006/anae.1995.1027	co-culture of bifidobacterium adolescentis and bacteroides thetaiotaomicron in arabinogalactan-limited chemostats effects of dilution rate and ph	Chemostat
4	10.1006/rtph.2001.1473	effects of low levels of ciprofloxacin on a chemostat model of the human colonic microflora	In-vitro Model
2	10.1007/978-1-0716-1274-3_17	in vitro assessment of prebiotic activity	Bioreactor
5	10.1007/978-1-4939-6361-4_17	an in vitro model of the human colon studies of intestinal biofilms and clostridium difficile infection	In-vitro Model
3	10.1007/BF00228615	development of a 5-step multi-chamber reactor as a simulation of the human intestinal microbial ecosystem	Reactor
2	10.1007/BF00431082	potential contribution of optional urease-positive bacteria to idiopathic urinary calcium stone formation ii microlith formation kinetics in a fermenter model of the urinary tract infected by optional urease-positive microorganisms	Artificial Gut
2	10.1007/BF00749221	production of immunoglobulin a in different reactor configurations	Reactor
2	10.1007/BF02291462	substrate-specific selective culture of cellulose splitting intestinal bacteria of small laboratory rodents	Continous Culture
2	10.1007/s00248-002-2014-z	presence of anaerobic bacteroides in aerobically grown microbial granules	Reactor
8	10.1007/s00248-003-2022-7	immobilization of infant fecal microbiota and utilization in an in vitro colonic fermentation model	Chemostat
2	10.1007/s00248-006-9157-2	cloning of environmental genomic fragments as physical markers for monitoring microbial populations in coking wastewater treatment system	Reactor
3	10.1007/s00248-015-0645-0	survival and metabolic activity of pediocin producer pediococcus acidilactici ul5 its impact on intestinal microbiota and listeria monocytogenes in a model of the human terminal ileum	Reactor
2	10.1007/s002480000005	microbial competition in reactors with wall attachment a mathematical comparison of chemostat and plug flow models	Reactor
2	10.1007/s002489900072	validation of a three-stage compound continuous culture system for investigating the effect of retention time on the ecology and metabolism of bacteria in the human colon	Continous Culture

4	10.1007/s00253-009-1947-2	in vitro model to study the modulation of the mucin-adhered bacterial community	In-vitro Model
4	10.1007/s00253-011-3462-5	in vitro maintenance of a human proximal colon microbiota using the continuous fermentation system p-ecsim	Continous Culture
2	10.1007/s00253-012-4623-x	nitrogen removal through different pathways in an aged refuse bioreactor treating mature landfill leachate	Reactor
2	10.1007/s00253-013-4763-7	effects of encapsulated lactobacillus acidophilus along with pasteurized longan juice on the colon microbiota residing in a dynamic simulator of the human intestinal microbial ecosystem	Simulator of Human
2	10.1007/s00253-014-5744-1	lactobacillus plantarum ifpl935 impacts colonic metabolism in a simulator of the human gut microbiota during feeding with red wine polyphenols	Shime
2	10.1007/s00253-014-5906-1	microbial community composition and dynamics in high-temperature biogas reactors using industrial bioethanol waste as substrate	Reactor
2	10.1007/s00253-016-7351-9	inulin-type fructan fermentation by bifidobacteria depends on the strain rather than the species and region in the human intestine	Shime
4	10.1007/s00253-018-9234-8	modulation of gut microbiota from obese individuals by in vitro fermentation of citrus pectin in combination with bifidobacterium longum bb-46	Shime
2	10.1007/s00253-019-09743-w	bile-induced promoters for gene expression in lactobacillus strains	Simulated Colon
9	10.1007/s00253-020-10959-4	comparative methods for fecal sample storage to preserve gut microbial structure and function in an in vitro model of the human colon	In-vitro Model
12	10.1007/s00253-021-11252-8	impact of a fermented soy beverage supplemented with acerola by-product on the gut microbiota from lean and obese subjects using an in vitro model of the human colon	Simulated Colon
2	10.1007/s002530050011	influence of a synbiotic mixture consisting of lactobacillus acidophilus 74-2 and a fructooligosaccharide preparation on the microbial ecology sustained in a simulation of the human intestinal microbial ecosystem shime reactor	Reactor
4	10.1007/s002530051022	effect of the addition of peptostreptococcus productus atcc35244 on the gastro-intestinal microbiota and its activity as simulated in an in vitro simulator of the human gastro-intestinal tract	Reactor
9	10.1007/s002530051284	the colonization of a simulator of the human intestinal microbial ecosystem by a probiotic strain fed on a fermented oat bran product effects on the gastrointestinal microbiota	Reactor
4	10.1007/s002530051622	a computer-controlled system to simulate conditions of the large intestine with peristaltic mixing water absorption and absorption of fermentation products	Reactor
2	10.1007/s00284-021-02502-z	impact of cashew anacardium occidentale l by-product on composition and metabolic activity of human colonic microbiota in vitro indicates prebiotic properties	Simulated Gastroin-testinal
2	10.1007/s00289-021-03853-0	evaluation of daidzein-loaded chitosan microcapsules for the colon cancer drug delivery synthesis characterization and release behaviour	Simulated Colon
4	10.1007/s00394-008-0747-2	factors affecting the conversion of apple polyphenols to phenolic acids and fruit matrix to short-chain fatty acids by human faecal microbiota in vitro	In-vitro Colon
8	10.1007/s00394-012-0391-8	characterization of microbial metabolism of syrah grape products in an in vitro colon model using targeted and non-targeted analytical approaches	In-vitro Colon
4	10.1007/s00394-015-1061-4	gastrointestinal stability of urolithins an in vitro approach	Simulated Gastroin-testinal
2	10.1007/s00394-016-1353-3	in vitro assessment of iron availability from commercial young child formulae supplemented with prebiotics	In-vitro Digestion

2	10.1007/s00394-021-02654-5	a randomized placebo-controlled trial investigating the acute and chronic benefits of american ginseng cereboost on mood and cognition in healthy young adults including in vitro investigation of gut microbiota changes as a possible mechanism of action	Shime
2	10.1007/s00417-018-4157-8	activation of the sweet taste receptor t1r3 by sucralose attenuates vegf-induced vasculogenesis in a cell model of the retinal microvascular endothelium	In-vitro Model
2	10.1007/s004490100263	understanding the bioreactor	Reactor
2	10.1007/s10068-017-0025-2	survival of probiotics in pea protein-alginate microcapsules with or without chitosan coating during storage and in a simulated gastrointestinal environment	Simulated Gastroin-testinal
4	10.1007/s10068-018-0527-6	interactions between fecal bacteria bile acids and components of tomato pomace	In-vitro Digestion
2	10.1007/s10482-012-9821-0	barcoded pyrosequencing analysis of the microbial community in a simulator of the human gastrointestinal tract showed a colon region-specific microbiota modulation for two plant-derived polysaccharide blends	Simulator of Human
2	10.1007/s10529-018-02634-6	oligosaccharides as co-encapsulating agents effect on oral lactobacillus fermentum survival in a simulated gastrointestinal tract	Simulated Gastroin-testinal
4	10.1007/s10529-018-2572-x	lactobacillus spp impair the ability of listeria monocytogenes fbunt to adhere to and invade caco-2 cells	Simulated Gastroin-testinal
2	10.1007/s10544-019-0387-8	influence of the static magnetic field on cell response in a miniaturized optically accessible bioreactor for 3d cell culture	In-vitro Model
4	10.1007/s10545-014-9763-y	in vitro digestion of starches in a dynamic gastrointestinal model an innovative study to optimize dietary management of patients with hepatic glycogen storage diseases	In-vitro Digestion
2	10.1007/s11010-011-0894-2	simulated colon fiber metabolome regulates genes involved in cell cycle apoptosis and energy metabolism in human colon cancer cells	In-vitro Colon
3	10.1007/s11130-012-0305-1	digested and fermented green kiwifruit increases human -defensin 1 and 2 production in vitro	In-vitro Digestion
2	10.1007/s11130-012-0329-6	antioxidant capacity of flaxseed products the effect of in vitro digestion	In-vitro Digestion
2	10.1007/s11130-017-0649-7	bioaccessibility of phenolic compounds and antioxidant capacity of chia salvia hispanica l seeds	Simulated Gastroin-testinal
2	10.1007/s11130-019-00739-5	pulp in shop-bought orange juice has little effect on flavonoid content and gut bacterial flavanone degradation in vitro	In-vitro Gastroin-testinal
2	10.1007/s11274-011-0792-5	functional properties of free and encapsulated lactobacillus reuteri dpc16 during and after passage through a simulated gastrointestinal tract	Simulated Colon
2	10.1007/s11274-012-1113-3	the effect of cell immobilization on the antibacterial activity of lactobacillus reuteri dpc16 cells during passage through a simulated gastrointestinal tract system	Simulated Gastroin-testinal
4	10.1007/s11306-015-0936-y	gut microbial activity as influenced by fiber digestion dynamic metabolomics in an in vitro colon simulator	In-vitro Colon
6	10.1007/s11356-012-1283-4	impact of chronic exposure to low doses of chlorpyrifos on the intestinal microbiota in the simulator of the human intestinal microbial ecosystem shime and in the rat	Reactor
7	10.1007/s11356-018-2332-4	use of a combination of in vitro models to investigate the impact of chlorpyrifos and inulin on the intestinal microbiota and the permeability of the intestinal mucosa	In-vitro Model

2	10.1007/s11694-016-9439-4	yields of three acids during simulated fermentation of inulin and xylo-oligosaccharides enhanced by six exogenous strains	Simulated Colon
2	10.1007/s11947-017-1946-8	microencapsulated starter culture during yoghurt manufacturing effect on technological features	Simulated Gastrointestinal
2	10.1007/s12010-021-03622-9	evaluation of microencapsulated synbiotic preparations containing lactobionic acid	In-vitro Model
2	10.1007/s12223-019-00687-2	probiotic characteristics of bacteriocin-producing enterococcus faecium strains isolated from human milk and colostrum	Simulated Gastrointestinal
2	10.1007/s12229-013-9118-7	weed seed survival during anaerobic digestion in biogas plants	Reactor
4	10.1007/s12275-012-2045-1	screening for probiotic properties of strains isolated from feces of various human groups	Simulated Gastrointestinal
4	10.1007/s12275-021-0525-x	effects of digested cheonggukjang on human microbiota assessed by in vitro fecal fermentation	In-vitro Gastrointestinal
2	10.1007/s12602-015-9187-5	probiotic properties of leuconostoc mesenteroides isolated from aguamiel of agave salmiana	In-vitro Gastrointestinal
2	10.1007/s12602-018-9387-x	characterization of functional safety and probiotic properties of enterococcus faecalis ag5 isolated from wistar rat demonstrating adherence to hct 116 cells and gastrointestinal survivability	Simulated Gastrointestinal
2	10.1007/s12602-018-9485-9	the effect of encapsulation on the stability of probiotic bacteria in ice cream and simulated gastrointestinal conditions	Simulated Gastrointestinal
2	10.1007/s12602-021-09839-8	brewers spent grain enhanced the recovery of potential probiotic strains in fermented milk after exposure to in vitro-simulated gastrointestinal conditions	Simulated Gastrointestinal
2	10.1007/s12649-021-01426-w	vermicomposting smart closed reactor design and performance assessment by using sewage sludge	Reactor
2	10.1007/s13197-014-1662-6	influences of exogenous probiotics and tea polyphenols on the production of three acids during the simulated colonic fermentation of maize resistant starch	Simulated Colon
2	10.1007/s13213-012-0558-9	study of probac product influence on infant microbiota in a single-chamber colonic fermentation model gis1	Simulated Colon
2	10.1007/s13213-014-0975-z	binding of acridine orange by probiotic lactobacillus rhamnosus strains of human origin	Simulated Gastrointestinal
2	10.1007/s13213-015-1187-x	characterization and in vitro properties of potential probiotic bifidobacterium strains isolated from breast-fed infant feces	Simulated Gastrointestinal
2	10.1007/s13213-019-01507-1	characterization of edible swiftlets nest as a prebiotic ingredient using a simulated colon model	Simulated Colon
2	10.1007/s13213-019-01518-y	selection of potentially probiotic kluyveromyces lactis for the fermentation of cheese wheybased beverage	Simulated Gastrointestinal
3	10.1007/s13228-014-0035-y	bioefficacy of tea catechins associated with milk caseins tested using different in vitro digestion models	In-vitro Digestion

2	10.1007/s40093-016-0152-4	changes in microbial pathogen dynamics during vermicomposting mixture of cow manureorganic solid waste and cow manuresewage sludge	Reactor
2	10.1007/s40201-019-00404-8	evaluating changes in microbial population and earthworms weight during vermicomposting of cow manure containing co-trimoxazole	Reactor
2	10.1007/s40710-016-0162-2	characterization of biofilm bacterial communities in a vertical unsaturated-flow bioreactor treating domestic greywater	Reactor
2	10.1007/s42770-021-00590-4	in vitro gastrointestinal resistance of lactobacillus acidophilus in some dairy products	In-vitro Gastroin-testinal
4	10.1016/0016-5085(85)90017-4	metabolism of mixed human colonic bacteria in a continuous culture mimicking the human cecal contents	In-vitro Model
2	10.1016/0377-8401(89)90094-1	in vitro fermentation of dietary fiber by human fecal organisms	Continous Culture
4	10.1016/0887-2333(87)90033-6	use of continuous culture to study the gastric microflora of a hypochlorhydric patient	Chemostat
2	10.1016/j.algal.2019.101457	nutrient-driven algal-bacterial dynamics in semi-continuous pilot-scale photobioreactor cultivation of nannochloropsis salina ccmp1776 with municipal wastewater nutrients	Bioreactor
2	10.1016/j.algal.2019.101754	chlorella vulgaris in a heterotrophic bioprocess study of the lipid bioaccessibility and oxidative stability	In-vitro Model
2	10.1016/j.anaerobe.2010.12.006	use of a continuous culture fermentation system to investigate the effect of ganedenbc30 bacillus coagulans gbi-30 6086 supplementation on pathogen survival in the human gut microbiota	Continous Culture
2	10.1016/j.anaerobe.2013.05.006	an exploratory study into the putative prebiotic activity of fructans isolated from agave angustifolia and the associated anticancer activity	Shime
4	10.1016/j.anaerobe.2014.03.009	an invitro study of the effect of probiotics prebiotics and synbiotics on the elderly faecal microbiota	Continous Culture
2	10.1016/j.anaerobe.2014.05.002	antagonistic mechanisms of synbiosis between lactobacillus plantarum cif17an2 and green banana starch in the proximal colon model challenged with salmonella typhimurium	Simulated Colon
2	10.1016/j.anaerobe.2014.05.008	flux analysis of the human proximal colon using anaerobic digestion model 1	In-vitro Model
3	10.1016/j.anaerobe.2015.02.006	interactions of salmonella enterica subspecies enterica serovar typhimurium with gut bacteria	Gut Model
2	10.1016/j.anaerobe.2015.04.009	in-vitro model for studying methanogens in human gut microbiota	In-vitro Model
2	10.1016/j.anifeedsci.2005.04.021	in vitro digestion and fermentation methods including gas production techniques as applied to nutritive evaluation of foods in the hindgut of humans and other simple-stomached animals	In-vitro Digestion
2	10.1016/j.anifeedsci.2006.02.009	the effect of extrusion cooking of different starch sources on the in vitro and in vivo digestibility in growing pigs	In-vitro Digestion
2	10.1016/j.anifeedsci.2018.07.002	supplemental effect of xylanase and mannanase on nutrient digestibility and gut health of nursery pigs studied using both in vivo and in vitro models	In-vitro Model
2	10.1016/j.anifeedsci.2019.05.006	physico-chemical properties of purified starch affect their in vitro fermentation characteristics and are linked to in vivo fermentation characteristics in pigs	In-vitro Digestion
2	10.1016/j.anres.2018.11.013	comparison of synbiotic beverages produced from riceberry malt extract using selected free and encapsulated probiotic lactic acid bacteria	Simulated Gastroin-testinal
2	10.1016/j.aquaeng.2019.102016	microbial valorization of solid wastes from a recirculating aquaculture system and the relevant microbial functions	Reactor
4	10.1016/j.archoralbio.2011.04.004	bacteriological effects of a lactobacillus reuteri probiotic on in vitro oral biofilms	Fermenter

2	10.1016/j.bcdf.2013.01.004	synthesis and in vitro digestion and fermentation of acylated inulin	In-vitro Digestion
2	10.1016/j.bcdf.2013.08.001	in vitro digestion and fermentation of 5-formyl-aminosailcylate-inulin a potential prodrug of 5-aminosalicylic acid	In-vitro Digestion
2	10.1016/j.bcdf.2014.06.007	the fate of chicory root pulp polysaccharides during fermentation in the tno in vitro model of the colon tim-2	In-vitro Model
2	10.1016/j.biomaterials.2019.119396	a three-dimensional immunocompetent intestine-on-chip model as in vitro platform for functional and microbial interaction studies	In-vitro Model
4	10.1016/j.bios.2012.10.028	microbial fuel cell as power supply for implantable medical devices a novel configuration design for simulating colonic environment	Simulated Colon
2	10.1016/j.bios.2019.111340	real-time cellular impedance monitoring and imaging of biological barriers in a dual-flow membrane bioreactor	In-vitro Model
2	10.1016/j.bmc.2007.04.042	caco-2 cell permeability and stability of two d-glucopyranuronamide conjugates of thyrotropin-releasing hormone	In-vitro Model
4	10.1016/j.bpg.2013.03.002	experimental models of the gut microbiome	Shime
2	10.1016/j.brainres.2021.147609	the link between the gut microbiota and parkinsons disease a systematic mechanism review with focus on -synuclein transport	In-vitro Model
2	10.1016/j.carbpol.2014.05.014	effect of alginate and chitosan on viability and release behavior of bifidobacterium pseudocatenulatum g4 in simulated gastrointestinal fluid	Simulated Gastroin-testinal
2	10.1016/j.carbpol.2015.05.022	in vitro digestion and fermentation properties of linear sugar-beet arabinan and its oligosaccharides	In-vitro Digestion
2	10.1016/j.carbpol.2015.09.106	digestibility and prebiotic properties of potato rhamnogalacturonan i polysaccharide and its galactose-rich oligosaccharidesoligomers	Continous Culture
2	10.1016/j.carbpol.2016.06.028	birch pulp xylan works as a food hydrocolloid in acid milk gels and is fermented slowly in vitro	In-vitro Colon
2	10.1016/j.carbpol.2016.12.014	molecular weight distribution and fermentation of mechanically pre-treated konjac enzymatic hydrolysates	Fermenter
2	10.1016/j.carbpol.2018.01.085	in vitro digestibility and prebiotic potential of curdlan 13–d-glucan oligosaccharides in lactobacillus species	Simulated Gastroin-testinal
2	10.1016/j.carbpol.2019.115469	in vitro digestion of polysaccharide including whey protein isolate hydrogels	In-vitro Digestion
2	10.1016/j.carbpol.2019.115593	in vitro digestion and fermentation of released exopolysaccharides r-eps from lactobacillus delbrueckii ssp bulgaricus srfm-1	In-vitro Digestion
2	10.1016/j.carbpol.2019.115647	degradation of polysaccharides from sargassum fusiforme using uvh2o2 and its effects on structural characteristics	In-vitro Digestion
2	10.1016/j.carbpol.2020.116447	an alternative way to encapsulate probiotics within electrospun alginate nanofibers as monitored under simulated gastrointestinal conditions and in kefir	Simulated Gastroin-testinal
2	10.1016/j.carbpol.2021.118738	human milk oligosaccharides and infant gut microbiota molecular structures utilization strategies and immune function	In-vitro Model
4	10.1016/j.cej.2015.08.088	characterization and antimicrobial activity of lactic acid bacteria from fermentative bioreactors during hydrogen production using cassava processing wastewater	Reactor
2	10.1016/j.cej.2016.02.108	effect of microbial community structure on organic removal and biofouling in membrane adsorption bioreactor used in seawater pretreatment	Reactor

3	10.1016/j.ces.2015.10.006	application of a novel gastrointestinal tract simulator system based on a membrane bioreactor simugit to study the stomach tolerance and effective delivery enhancement of nanoencapsulated macelignan	In-vitro Gastroin-testinal
5	10.1016/j.chemosphere.2010.04.061	polycyclic aromatic hydrocarbons are enriched but bioaccessibility reduced in brownfield soils adhered to human hands	Gastrointestinal Model
3	10.1016/j.chemosphere.2014.08.010	westernized diets lower arsenic gastrointestinal bioaccessibility but increase microbial arsenic speciation changes in the colon	In-vitro Gastroin-testinal
2	10.1016/j.chemosphere.2014.12.019	solid-liquid separation method governs the in vitro bioaccessibility of metals in contaminated soil-like test materials	In-vitro Gastroin-testinal
2	10.1016/j.chemosphere.2017.06.018	interindividual variability of soil arsenic metabolism by human gut microbiota using shime model	Shime
6	10.1016/j.chemosphere.2018.08.039	rapid and complete dehalogenation of halonitromethanes in simulated gastrointestinal tract and its influence on toxicity	Simulated Gastroin-testinal
7	10.1016/j.chemosphere.2020.126522	in vitro oral bioaccessibility investigation and human health risk assessment of heavy metals in wheat grains grown near the mines in north china	Shime
2	10.1016/j.chom.2021.09.007	in vivo commensal control of clostridioides difficile virulence	Fermenter
2	10.1016/j.conctc.2020.100646	integrative and quantitative bioenergetics design of a study to assess the impact of the gut microbiome on host energy balance	Bioreactor
2	10.1016/j.ebiom.2019.04.038	clostridium difficile trehalose metabolism variants are common and not associated with adverse patient outcomes when variably present in the same lineage	Gut Model
2	10.1016/j.ecoenv.2020.110913	assessment of nutrients effect on the bioaccessibility of cd and cu in contaminated soil	Shime
4	10.1016/j.ejpb.2010.12.004	ethylene vinyl acetate as matrix for oral sustained release dosage forms produced via hot-melt extrusion	Shime
2	10.1016/j.ejpb.2014.11.003	thermoplastic polyurethanes for the manufacturing of highly dosed oral sustained release matrices via hot melt extrusion and injection molding	Shime
2	10.1016/j.ejps.2015.03.012	a novel dissolution method for evaluation of polysaccharide based colon specific delivery systems a suitable alternative to animal sacrifice	Simulated Colon
4	10.1016/j.ejps.2018.09.019	evaluating the clinical importance of bacterial degradation of therapeutic agents in the lower intestine of adults using adult fecal material	Simulated Colon
3	10.1016/j.envint.2013.01.003	concentrations of organochlorine pesticides ocps in human blood plasma from hong kong markers of exposure and sources from fish	In-vitro Digestion
2	10.1016/j.envpol.2011.11.021	arsenic in cooked rice effect of chemical enzymatic and microbial processes on bioaccessibility and speciation in the human gastrointestinal tract	In-vitro Gastroin-testinal
2	10.1016/j.envpol.2019.01.041	reflection of concentrations of polybrominated diphenyl ethers in health risk assessment a case study in sediments from the metropolitan river north china	In-vitro Gastroin-testinal
4	10.1016/j.envpol.2020.114399	vancomycin exposure caused opportunistic pathogens bloom in intestinal microbiome by simulator of the human intestinal microbial ecosystem shime	Shime
2	10.1016/j.envpol.2021.116943	effect of gut microbiota on in vitro bioaccessibility of heavy metals and human health risk assessment from ingestion of contaminated soils	Simulated Gastroin-testinal
2	10.1016/j.envpol.2021.116958	arsenic speciation and bioaccessibility in raw and cooked seafood influence of seafood species and gut microbiota	In-vitro Digestion

2	10.1016/j.eti.2019.100456	optimization of the anaerobic denitrification process mediated by bacillus cereus in a batch reactor	Reactor
2	10.1016/j.fbio.2016.09.001	survival of immobilized probiotics in chocolate during storage and with an in vitro gastrointestinal model	In-vitro Gastroin-testinal
2	10.1016/j.fbio.2016.10.001	three protective agents for pectin-rice bran capsules for encapsulating lactobacillus plantarum	Simulated Gastroin-testinal
4	10.1016/j.fbio.2016.12.005	combined effects of low-fat ice cream supplemented with probiotics on colon microfloral communities and their metabolites during fermentation in a human gut reactor	Gut Model
2	10.1016/j.fbio.2019.100425	inhibitory effect of bacteriocin-producing lactobacillus brevis df01 and pediococcus acidilactici k10 isolated from kimchi on enteropathogenic bacterial adhesion	Simulated Gastroin-testinal
2	10.1016/j.fbio.2020.100614	potential prebiotic properties of flours from different varieties of sweet potato ipomoea batatas l roots cultivated in northeastern brazil	Simulated Gastroin-testinal
4	10.1016/j.fbio.2020.100668	thermo-tolerant saccharomyces cerevisiae var boulardii coated cornflakes as a potential probiotic vehicle	Simulated Gastroin-testinal
2	10.1016/j.fbio.2020.100813	edible lecithin stearic acid and whey protein bigels enhance survival of probiotics during in vitro digestion	Simulated Gastroin-testinal
2	10.1016/j.fbio.2020.100858	designing a functional rice muffin formulated with prebiotic oligosaccharides and sugar reduction	In-vitro Model
2	10.1016/j.fbio.2021.101080	cashew apple juice containing gluco-oligosaccharides dextran and tagatose promotes probiotic microbial growth	In-vitro Digestion
2	10.1016/j.fbio.2021.101223	oat bran fortified raspberry probiotic dairy drinks physicochemical textural microbiologic properties in vitro bioaccessibility of antioxidants and polyphenols	In-vitro Digestion
2	10.1016/j.fbio.2021.101380	traditional and flavored kombuchas with pitanga and umbu-caj pulps chemical properties antioxidants and bioactive compounds	Simulated Gastroin-testinal
2	10.1016/j.fbp.2018.08.011	storage stability and simulated gastrointestinal release of spray dried grape marc phenolics	Simulated Gastroin-testinal
2	10.1016/j.fbp.2020.12.008	urea removal in ros and red wines by immobilised acid urease in a packed bed reactor	Reactor
2	10.1016/j.fbp.2021.02.004	kinetic modeling of the enzymatic synthesis of galacto-oligosaccharides describing galactobiose formation	Bioreactor
2	10.1016/j.fct.2018.04.008	assessing the effects of silver nanoparticles on monolayers of differentiated caco-2 cells as a model of intestinal barrier	In-vitro Model
2	10.1016/j.fct.2018.11.048	zno nanoparticles affect nutrient transport in an in vitro model of the small intestine	In-vitro Digestion
2	10.1016/j.femsec.2004.05.002	pcr-dgge-based quantification of stability of the microbial community in a simulator of the human intestinal microbial ecosystem	Reactor
2	10.1016/j.femsec.2004.07.014	prebiotic effects of chicory inulin in the simulator of the human intestinal microbial ecosystem	Shime
3	10.1016/j.fm.2012.10.005	selection of potential probiotic lactic acid bacteria from fermented olives by in vitro tests	Simulated Gastroin-testinal

4	10.1016/j.fm.2013.01.012	impact of inulin and okara on lactobacillus acidophilus la-5 and bifidobacterium animalis bb-12 viability in a fermented soy product and probiotic survival under in vitro simulated gastrointestinal conditions	Simulated Gastrointestinal
2	10.1016/j.fm.2016.10.016	exposure to minimally processed pear and melon during shelf life could modify the pathogenic potential of listeria monocytogenes	Simulated Gastrointestinal
4	10.1016/j.fm.2018.08.010	survival of listeria monocytogenes during in vitro gastrointestinal digestion after exposure to 5 and 05 sodium chloride	In-vitro Digestion
4	10.1016/j.fm.2019.103348	survival of lactobacillus acidophilus la-5 and escherichia coli o157h7 in minas frescal cheese made with oregano and rosemary essential oils	Simulated Gastrointestinal
2	10.1016/j.fm.2019.103351	impact of exposure to cold and cold-osmotic stresses on virulence-associated characteristics of listeria monocytogenes strains	In-vitro Gastrointestinal
4	10.1016/j.foodchem.2006.02.006	intake and bioaccessibility of total polyphenols in a whole diet	In-vitro Gastrointestinal
2	10.1016/j.foodchem.2010.03.079	polyphenol and nutrient release from skin of almonds during simulated human digestion	In-vitro Digestion
2	10.1016/j.foodchem.2011.03.043	effect of bioprocessing of wheat bran in wholemeal wheat breads on the colonic scfa production in vitro and postprandial plasma concentrations in men	In-vitro Model
2	10.1016/j.foodchem.2013.01.097	active films based on cocoa extract with antioxidant antimicrobial and biological applications	In-vitro Gastrointestinal
2	10.1016/j.foodchem.2013.05.001	phenolic composition and inhibitory effect against oxidative dna damage of cooked cowpeas as affected by simulated in vitro gastrointestinal digestion	Simulated Gastrointestinal
4	10.1016/j.foodchem.2013.05.157	metabolic fate of ochratoxin a as a coffee contaminant in a dynamic simulator of the human colon	Simulator of Human
2	10.1016/j.foodchem.2015.03.095	effect of stevia rebaudiana addition on bioaccessibility of bioactive compounds and antioxidant activity of beverages based on exotic fruits mixed with oat following simulated human digestion	Simulated Gastrointestinal
2	10.1016/j.foodchem.2015.04.029	increased oxidative and nitrosative reactions during digestion could contribute to the association between well-done red meat consumption and colorectal cancer	In-vitro Digestion
2	10.1016/j.foodchem.2015.09.048	in vitro fermentation of juara pulp euterpe edulis by human colonic microbiota	In-vitro Digestion
2	10.1016/j.foodchem.2016.01.076	stability and metabolism of arbutus unedo bioactive compounds phenolics and antioxidants under in vitro digestion and colonic fermentation	In-vitro Digestion
4	10.1016/j.foodchem.2016.02.140	biotransformation of polyphenols in a dynamic multistage gastrointestinal model	Gastrointestinal Model
3	10.1016/j.foodchem.2016.03.108	microbial biotransformation of polyphenols during in vitro colonic fermentation of masticated mango and banana	In-vitro Gastrointestinal
8	10.1016/j.foodchem.2017.04.164	in vitro approaches to assess the effects of aai euterpe oleracea digestion on polyphenol availability and the subsequent impact on the faecal microbiota	In-vitro Digestion
2	10.1016/j.foodchem.2017.05.125	effects of in vitro digestion and in vitro colonic fermentation on stability and functional properties of yerba mate ilex paraguariensis a st hil beverages	In-vitro Digestion

4	10.1016/j.foodchem.2017.07.024	towards an improved global antioxidant response method gar physiological-resembling in vitro digestion-fermentation method	In-vitro Digestion
3	10.1016/j.foodchem.2017.11.001	emulsion stability during gastrointestinal conditions effects lipid digestion kinetics	Simulated Gastroin-testinal
2	10.1016/j.foodchem.2018.02.128	changes in bioaccessibility polyphenol profile and antioxidant potential of flours obtained from persimmon fruit diospyros kaki co-products during in vitro gastrointestinal digestion	In-vitro Digestion
2	10.1016/j.foodchem.2018.03.053	colonic fermentation of polyphenols from chilean currants ribes spp and its effect on antioxidant capacity and metabolic syndrome-associated enzymes	Simulated Colon
2	10.1016/j.foodchem.2018.04.095	effects of fermented milk treatment on microbial population and metabolomic outcomes in a three-stage semi-continuous culture system	In-vitro Colon
2	10.1016/j.foodchem.2018.07.020	antioxidant and antidiabetic activity of blackberry after gastrointestinal digestion and human gut microbiota fermentation	Simulated Gastroin-testinal
4	10.1016/j.foodchem.2018.10.120	variability of chromium bioaccessibility and speciation in vegetables the influence of in vitro methods gut microbiota and vegetable species	Shime
3	10.1016/j.foodchem.2018.11.137	effect of in vitro digestion-fermentation on green and roasted coffee bioactivity the role of the gut microbiota	In-vitro Digestion
2	10.1016/j.foodchem.2019.02.101	phenolic profile and antioxidant activity of jasonia glutinosa herbal tea influence of simulated gastrointestinal in vitro digestion	Simulated Gastroin-testinal
2	10.1016/j.foodchem.2019.05.207	phytochemical profile mineral content and antioxidant activity of olea europaea l cv cornezuelo table olives influence of in vitro simulated gastrointestinal digestion	Simulated Gastroin-testinal
2	10.1016/j.foodchem.2019.125343	blueberry pectin and increased anthocyanins stability under in vitro digestion	Simulated Gastroin-testinal
2	10.1016/j.foodchem.2019.125662	bioaccessibility of micronutrients in fresh and frozen strawberry fruits grown under elevated carbon dioxide and temperature	In-vitro Gastroin-testinal
2	10.1016/j.foodchem.2020.127483	effect of in vitro digestion-fermentation of caii-alginate beads containing sugar and biopolymers over global antioxidant response and short chain fatty acids production	In-vitro Digestion
2	10.1016/j.foodchem.2020.127606	effect of in vitro digestion on phenolics and antioxidant activity of red and yellow colored pea hulls	In-vitro Digestion
2	10.1016/j.foodchem.2020.128210	assessment of bioaccessible and dialyzable fractions of nickel in food products and their impact on the chronic exposure of belgian population to nickel	In-vitro Gastroin-testinal
4	10.1016/j.foodchem.2020.128237	detrimental effect on the gut microbiota of 12-dicarbonyl compounds after in vitro gastro-intestinal and fermentative digestion	Simulated Gastroin-testinal
2	10.1016/j.foodchem.2021.130095	in vitro digestion and fecal fermentation of highly resistant starch rice and its effect on the gut microbiota	In-vitro Digestion
6	10.1016/j.foodchem.2021.130233	the bioavailability of soybean polysaccharides and their metabolites on gut microbiota in the simulator of the human intestinal microbial ecosystem shime	Shime
3	10.1016/j.foodchem.2021.130871	simulated gastrointestinal digestion of cranberry polyphenols under dynamic conditions impact on antiadhesive activity against uropathogenic bacteria	Simulated Gastroin-testinal

2	10.1016/j.foodchem.2021.131303	characterization of polysaccharide from pleurotus eryngii during simulated gastrointestinal digestion and fermentation	Simulated Gastrointestinal
2	10.1016/j.foodchem.2021.131391	preliminary studies of the impact of food components on nutritional properties of nanoparticles	Simulated Gastrointestinal
2	10.1016/j.foodchem.2021.131451	investigation of formation of well-known ages precursors in cookies using an in vitro simulated gastrointestinal digestive system	Simulated Gastrointestinal
2	10.1016/j.foodcont.2021.108603	influence of fermentation by lactic acid bacteria and in vitro digestion on the biotransformations of blueberry juice phenolics	Simulated Gastrointestinal
2	10.1016/j.foodhyd.2018.09.014	effects of in vitro saliva gastric and intestinal digestion on the chemical properties antioxidant activity of polysaccharide from artocarpus heterophyllus lam jackfruit pulp	In-vitro Digestion
2	10.1016/j.foodhyd.2018.11.040	effect of simulated gastrointestinal digestion in vitro on the antioxidant activity molecular weight and microstructure of polysaccharides from a tropical sea cucumber holothuria leucospilota	Simulated Gastrointestinal
4	10.1016/j.foodhyd.2019.01.040	encapsulation of bifidobacterium pseudocatenulatum g7 in gastroprotective microgels improvement of the bacterial viability under simulated gastrointestinal conditions	Simulated Gastrointestinal
2	10.1016/j.foodhyd.2019.04.059	pectin-chitosan conjugated nanoliposome as a promising delivery system for neohesperidin characterization release behavior cellular uptake and antioxidant property	Simulated Gastrointestinal
2	10.1016/j.foodhyd.2019.105511	how does the degree of inulin polymerization affect the bioaccessibility of bioactive compounds from soursop whey beverage during in vitro gastrointestinal digestion	In-vitro Digestion
2	10.1016/j.foodhyd.2020.106086	structural physicochemical and in-vitro release properties of hydrogel beads produced by oligochitosan and de-esterified pectin from yuzu citrus junos peel as a quercetin delivery system for colon target	Simulated Colon
2	10.1016/j.foodhyd.2020.106312	prebiotic effects of olive pomace powders in the gut in vitro evaluation of the inhibition of adhesion of pathogens prebiotic and antioxidant effects	Simulated Gastrointestinal
2	10.1016/j.foodhyd.2020.106453	interpenetrating polymer network hydrogels of soy protein isolate and sugar beet pectin as a potential carrier for probiotics	Simulated Gastrointestinal
2	10.1016/j.foodhyd.2020.106577	in vitro digestion and fecal fermentation behaviors of a pectic polysaccharide from okra abelmoschus esculentus and its impacts on human gut microbiota	In-vitro Digestion
4	10.1016/j.foodhyd.2021.106634	encapsulation of bifidobacterium in alginate microgels improves viability and targeted gut release	Simulated Colon
6	10.1016/j.foodres.2012.07.033	influence of encapsulated probiotics combined with pressurized longan juice on colon microflora and their metabolic activities on the exposure to simulated dynamic gastrointestinal tract	Reactor
4	10.1016/j.foodres.2013.01.034	impact of polyphenols from black tea and red winegrape juice on a gut model microbiome	Gut Model
4	10.1016/j.foodres.2014.05.072	beneficial effects of fermented vegetal beverages on human gastrointestinal microbial ecosystem in a simulator	Shime
4	10.1016/j.foodres.2014.06.032	addition of probiotic bacteria in a semi-hard goat cheese coalho survival to simulated gastrointestinal conditions and inhibitory effect against pathogenic bacteria	Simulated Gastrointestinal

2	10.1016/j.foodres.2015.03.044	epitopes resistance to the simulated gastrointestinal digestion of -lactoglobulin submitted to two-step enzymatic modification	Simulated Gastrointestinal
2	10.1016/j.foodres.2015.06.036	potential impact of biopolymers -polylysine andor pectin on gastrointestinal fate of foods in vitro study	Simulated Gastrointestinal
2	10.1016/j.foodres.2015.07.024	simulated gastrointestinal digestion and in vitro colonic fermentation of spent coffee coffea arabica l bioaccessibility and intestinal permeability	Simulated Gastrointestinal
4	10.1016/j.foodres.2016.03.028	an exploratory study on the influence of orange juice on gut microbiota using a dynamic colonic model	Shime
2	10.1016/j.foodres.2016.09.022	the influence of breast milk and infant formulae hydrolysates on bacterial adhesion and caco-2 cells functioning	In-vitro Model
2	10.1016/j.foodres.2017.03.035	phenolic profile and fermentation patterns of different commercial gluten-free pasta during in vitro large intestine fermentation	In-vitro Gastrointestinal
4	10.1016/j.foodres.2017.04.019	moisture content during extrusion of oats impacts the initial fermentation metabolites and probiotic bacteria during extended fermentation by human fecal microbiota	In-vitro Digestion
2	10.1016/j.foodres.2017.05.028	impact of multi-functional fermented goat milk beverage on gut microbiota in a dynamic colon model	Shime
2	10.1016/j.foodres.2017.07.018	bioaccessibility and antioxidant activity of free phenolic compounds and oligosaccharides from corn zea mays l and common bean phaseolus vulgaris l chips during in vitro gastrointestinal digestion and simulated colonic fermentation	Simulated Colon
4	10.1016/j.foodres.2017.09.001	development and functional characterization of new antioxidant dietary fibers from pomegranate olive and artichoke by-products	Simulated Gastrointestinal
2	10.1016/j.foodres.2017.09.093	simulated gastrointestinal conditions increase adhesion ability of lactobacillus paracasei strains isolated from kefir to caco-2 cells and mucin	Simulated Gastrointestinal
3	10.1016/j.foodres.2017.11.036	in vitro chemopreventive properties of peptides released from quinoa chenopodium quinoa willd protein under simulated gastrointestinal digestion	Simulated Gastrointestinal
4	10.1016/j.foodres.2017.12.024	prebiotic effect of predigested mango peel on gut microbiota assessed in a dynamic in vitro model of the human colon tim-2	In-vitro Model
3	10.1016/j.foodres.2018.04.008	anti-inflammatory effect of microbial consortia during the utilization of dietary polysaccharides	Bioreactor
4	10.1016/j.foodres.2018.05.045	influence of polyphenol rich seabuckthorn berries juice on release of polyphenols and colonic microbiota on exposure to simulated human digestion model	Gut Model
11	10.1016/j.foodres.2018.05.072	in vitro colonic fermentation of mexican taco from corn-tortilla and black beans in a simulator of human microbial ecosystem shime system	Reactor
4	10.1016/j.foodres.2018.08.005	effects of lactobacillus acidophilus la-3 on physicochemical and sensory parameters of aa and mango based smoothies and its survival following simulated gastrointestinal conditions	Simulated Gastrointestinal
6	10.1016/j.foodres.2018.11.010	in vitro modulation of human gut microbiota composition and metabolites by bifidobacterium longum bb-46 and a citric pectin	Shime
3	10.1016/j.foodres.2019.108542	the resistance of bacillus bifidobacterium and lactobacillus strains with claimed probiotic properties in different food matrices exposed to simulated gastrointestinal tract conditions	Simulated Gastrointestinal

10	10.1016/j.foodres.2019.108848	effects of gastrointestinal digested polyphenolic enriched extracts of chilean currants ribes magellanicum and ribes punctatum on in vitro fecal microbiota	Simulated Gastrointestinal
4	10.1016/j.foodres.2020.109036	changes in gut microbiota in predigested hibiscus sabdariffa l calyces and agave agave tequilana weber fructans assessed in a dynamic in vitro model tim-2 of the human colon	In-vitro Model
8	10.1016/j.foodres.2020.109098	in vitro gastrointestinal digestion and fecal fermentation reveal the effect of different encapsulation materials on the release degradation and modulation of gut microbiota of blueberry anthocyanin extract	Simulated Gastrointestinal
6	10.1016/j.foodres.2020.109127	oxygen as a key parameter in in vitro dynamic and multi-compartment models to improve microbiome studies of the small intestine	In-vitro Digestion
2	10.1016/j.foodres.2020.109232	impact of defined thermomechanical treatment on the structure and content of dietary fiber and the stability and bioaccessibility of polyphenols of chokeberry aronia melanocarpa pomace	In-vitro Digestion
2	10.1016/j.foodres.2020.109317	antiproliferative activity of green black tea and olive leaves polyphenols subjected to biosorption and in vitro gastrointestinal digestion in caco-2 cells	In-vitro Gastrointestinal
6	10.1016/j.foodres.2020.109354	potential prebiotic effect of fruit and vegetable byproducts flour using in vitro gastrointestinal digestion	In-vitro Gastrointestinal
4	10.1016/j.foodres.2020.109580	evaluation of the fate of lactobacillus crispatus bc4 carried in squacquerone cheese throughout the simulator of the human intestinal microbial ecosystem shime	Shime
4	10.1016/j.foodres.2020.109673	breast-milk derived potential probiotics as strategy for the management of childhood obesity	Shime
8	10.1016/j.foodres.2020.109755	effect of bifidobacterium crudilactis and 3-sialyllactose on the toddler microbiota using the shime model	Shime
4	10.1016/j.foodres.2020.109888	dynamic changes of structural characteristics of snow chrysanthemum polysaccharides during in vitro digestion and fecal fermentation and related impacts on gut microbiota	In-vitro Digestion
2	10.1016/j.foodres.2020.109906	bioaccessibility of cashew nut kernel flour compounds released after simulated in vitro human gastrointestinal digestion	Simulated Gastrointestinal
2	10.1016/j.foodres.2020.109917	study of the impact of a dynamic in vitro model of the colon tim-2 in the phenolic composition of two mexican sauces	In-vitro Model
3	10.1016/j.foodres.2020.109963	bioconversion by gut microbiota of predigested mango mangifera indica l ataulfo peel polyphenols assessed in a dynamic tim-2 in vitro model of the human colon	In-vitro Model
2	10.1016/j.foodres.2020.110011	the regulation of sodium alginate on the stability of ovalbumin-pectin complexes for vd3 encapsulation and in vitro simulated gastrointestinal digestion study	Simulated Gastrointestinal
2	10.1016/j.foodres.2021.110136	functional properties of lactobacillus casei c24 improved by microencapsulation using multilayer double emulsion	Simulated Gastrointestinal
2	10.1016/j.foodres.2021.110263	the recovery catabolism and potential bioactivity of polyphenols from carrot subjected to in vitro simulated digestion and colonic fermentation	In-vitro Digestion
8	10.1016/j.foodres.2021.110292	probiotic infant cereal improves childrens gut microbiota insights using the simulator of human intestinal microbial ecosystem shime	Shime
3	10.1016/j.foodres.2021.110301	bioconversion of polyphenols and organic acids by gut microbiota of predigested hibiscus sabdariffa l calyces and agave a tequilana weber fructans assessed in a dynamic in vitro model tim-2 of the human colon	In-vitro Model
4	10.1016/j.foodres.2021.110326	growth-promoting effect of alginate on faecalibacterium prausnitzii through cross-feeding with bacteroides	Fermenter

2	10.1016/j.foodres.2021.110395	the efficacy and effect on gut microbiota of an aflatoxin binder and a fumonisin esterase using an in vitro simulator of the human intestinal microbial ecosystem shime	Shime
6	10.1016/j.foodres.2021.110657	effect of probiotic prebiotic and synbiotic on the gut microbiota of autistic children using an in vitro gut microbiome model	Simulated Gastroin-testinal
12	10.1016/j.foodres.2021.110676	treatment with a spore-based probiotic containing five strains of bacillus induced changes in the metabolic activity and community composition of the gut microbiota in a shime model of the human gastrointestinal system	In-vitro Model
2	10.1016/j.foodres.2021.110712	development and evaluation of a novel nanofibersolosome for enhancing the stability in vitro bioaccessibility and colonic delivery of cyanidin-3-o-glucoside	Simulated Gastroin-testinal
2	10.1016/j.foodres.2021.110767	effect of in vitro digestion on the bioaccessibility and bioactivity of phenolic compounds in fractions of eugenia pyriformis fruit	In-vitro Digestion
2	10.1016/j.heliyon.2020.e03567	polyphenols in cassava leaves manihot esculenta crantz and their stability in antioxidant potential after in vitro gastrointestinal digestion	In-vitro Digestion
2	10.1016/j.heliyon.2020.e05474	plant extracts as natural modulators of gut microbiota community structure and functionality	In-vitro Digestion
2	10.1016/j.ibiod.2020.105102	effect of ultrasonic frequency on the bacterial community structure during biofouling formation in microfiltration membrane bioreactors for wastewater treatment	Reactor
2	10.1016/j.ibiod.2021.105255	dechlorane plus exposure on gut microbiome evaluated by using both in vivo and in vitro assays	Shime
2	10.1016/j.idairyj.2010.09.006	in vitro effects of ph bile salts and enzymes on the release and viability of encapsulated lactobacillus plantarum strains in a gastrointestinal tract model	Simulated Gastroin-testinal
2	10.1016/j.idairyj.2011.06.002	effect of glycation of bovine -lactoglobulin with galactooligosaccharides on the growth of human faecal bacteria	Simulated Gastroin-testinal
2	10.1016/j.idairyj.2014.06.009	safety and potential beneficial properties of enterococcus strains isolated from kefir	Simulated Gastroin-testinal
2	10.1016/j.idairyj.2014.11.006	a survey of non-starter lactic acid bacteria in traditional cheeses culture dependent identification and survival to simulated gastrointestinal transit	Simulated Gastroin-testinal
2	10.1016/j.idairyj.2017.07.004	characterisation of invitro gastrointestinal digests from low fat caprine kefir enriched with inulin	Simulated Gastroin-testinal
2	10.1016/j.ifset.2019.102286	spent coffee coffea arabica l grounds positively modulate indicators of colonic microbial activity	In-vitro Gastroin-testinal
2	10.1016/j.ifset.2021.102750	survival of probiotic bacteria nanoencapsulated within biopolymers in a simulated gastrointestinal model	Simulated Gastroin-testinal
2	10.1016/j.ijbiomac.2019.08.170	structural characterization and in vitro gastrointestinal digestion and fermentation of litchi polysaccharide	In-vitro Digestion
2	10.1016/j.ijbiomac.2019.09.073	simulated digestion and fermentation in vitro by human gut microbiota of polysaccharides from helicteres angustifolia l	Simulated Gastroin-testinal

2	10.1016/j.ijbiomac.2020.06.174	prebiotic properties of different polysaccharide fractions from artemisia sphaerocephala krasch seeds evaluated by simulated digestion and in vitro fermentation by human fecal microbiota	Simulated Gastrointestinal
2	10.1016/j.ijbiomac.2020.11.130	in vitro simulated digestion and fecal fermentation of polysaccharides from loquat leaves dynamic changes in physicochemical properties and impacts on human gut microbiota	In-vitro Digestion
2	10.1016/j.ijbiomac.2021.01.092	structural rearrangement of native and processed pea starches following simulated digestion in vitro and fermentation characteristics of their resistant starch residues using human fecal inoculum	Simulated Gastrointestinal
4	10.1016/j.ijbiomac.2021.08.063	deciphering the colonic fermentation characteristics of agavin and digestion-resistant maltodextrin in a simulated batch fermentation system	Simulated Colon
2	10.1016/j.ijfoodmicro.2009.08.017	exopolysaccharides produced by bifidobacterium longum ipla e44 and bifidobacterium animalis subsp lactis ipla r1 modify the composition and metabolic activity of human faecal microbiota in ph-controlled batch cultures	Simulated Gastrointestinal
2	10.1016/j.ijfoodmicro.2010.12.029	probiotic assessment of enterococcus faecalis cp58 isolated from human gut	In-vitro Digestion
3	10.1016/j.ijfoodmicro.2017.05.015	characterization of the peptide fraction from digested parmigiano reggiano cheese and its effect on growth of lactobacilli and bifidobacteria	Simulated Gastrointestinal
4	10.1016/j.ijfoodmicro.2019.108357	commensal e coli rapidly transfer antibiotic resistance genes to human intestinal microbiota in the mucosal simulator of the human intestinal microbial ecosystem m-shime	In-vitro Model
2	10.1016/j.ijhydene.2019.12.206	modelling sugarcane vinasse processing in an acidogenic reactor to produce hydrogen with an adm1-based model	Reactor
3	10.1016/j.ijmm.2021.151533	comparative phenotypic characterization of hybrid shiga toxin-producing uropathogenic escherichia coli canonical uropathogenic and shiga toxin-producing escherichia coli	Simulated Colon
4	10.1016/j.ijpharm.2013.09.007	colonic bacterial metabolism of corticosteroids	Simulated Colon
2	10.1016/j.ijpharm.2018.12.021	in vitro drug release from acetylated high amylose starch-zein films for oral colon-specific drug delivery	Simulated Colon
6	10.1016/j.ijpharm.2021.120977	comparison of protection and release behavior of different capsule polymer combinations based on l acidophilus survivability and function and caffeine release	Shime
2	10.1016/j.imbio.2008.02.001	effects of probiotic bacteria and their genomic dna on th1th2-cytokine production by peripheral blood mononuclear cells pbmcs of healthy and allergic subjects	In-vitro Model
3	10.1016/j.impact.2018.11.002	development of a standardized food model for studying the impact of food matrix effects on the gastrointestinal fate and toxicity of ingested nanomaterials	Simulated Gastrointestinal
2	10.1016/j.indcrop.2016.02.002	improved bioaccessibility and antioxidant capacity of olive leaf olea europaea l polyphenols through biosorption on saccharomyces cerevisiae	Simulated Gastrointestinal
2	10.1016/j.indcrop.2019.05.071	determination of the yield saponin content and profile antimicrobial and antioxidant activities of three gypsophila species	Simulated Gastrointestinal
2	10.1016/j.jbiosc.2013.09.015	assessing the influence of reactor system design criteria on the performance of model colon fermentation units	In-vitro Colon
2	10.1016/j.jbiosc.2014.09.003	reorganization of the bacterial and archaeal populations associated with organic loading conditions in a thermophilic anaerobic digester	Reactor
4	10.1016/j.jchromb.2004.04.001	liquid chromatography-mass spectrometry analysis of hydroxylated polycyclic aromatic hydrocarbons formed in a simulator of the human gastrointestinal tract	Shime

4	10.1016/j.jchromb.2019.06.013	development of an analytical method to detect short-chain fatty acids by spme-gc-ms in samples coming from an in vitro gastrointestinal model	In-vitro Gastrointestinal
2	10.1016/j.jchromb.2019.121728	in vitro and in vivo metabolism of cistanche tubulosa extract in normal and chronic unpredictable stress-induced depressive rats	In-vitro Gastrointestinal
2	10.1016/j.jclepro.2016.09.215	anaerobic cometabolism of fruit and vegetable wastes using mammalian fecal inoculums fast assessment of biomethane production	Reactor
2	10.1016/j.jcs.2020.103070	quinoa flavonoids and their bioaccessibility during in vitro gastrointestinal digestion	In-vitro Digestion
2	10.1016/j.jep.2013.12.056	simulated gastrointestinal tract metabolism and pharmacological activities of water extract of scutellaria baicalensis roots	Simulated Gastrointestinal
2	10.1016/j.jep.2021.113839	intestinal bacteria are involved in radix glycyrrhizae and radix euphorbiae pekinensis incompatibility	In-vitro Model
3	10.1016/j.jfca.2018.05.007	bioaccessibility analysis of anthocyanins and ellagitannins from blackberry at simulated gastrointestinal and colonic levels	Simulated Gastrointestinal
2	10.1016/j.jfca.2021.103945	permeability of native and digested polyphenols from apple blueberry and cranberry extracts using pampa membrane permeability assays	In-vitro Gastrointestinal
2	10.1016/j.jff.2013.09.015	the prebiotic effect of -12 branched low molecular weight dextran in the batch and continuous faecal fermentation system	Gut Model
2	10.1016/j.jff.2014.09.019	prebiotic effects of cassava bagasse in tnos in vitro model of the colon in lean versus obese microbiota	In-vitro Model
2	10.1016/j.jff.2015.02.026	application of in vitro gastrointestinal digestion and colonic fermentation models to pomegranate products juice pulp and peel extract to study the stability and catabolism of phenolic compounds	In-vitro Gastrointestinal
2	10.1016/j.jff.2015.04.039	addition of acacia gum to a fosinulin blend improves its fermentation profile in the simulator of the human intestinal microbial ecosystem shime	Shime
2	10.1016/j.jff.2015.10.033	lactic acid bacterial fermentation modified phenolic composition in tea extracts and enhanced their antioxidant activity and cellular uptake of phenolic compounds following in vitro digestion	In-vitro Digestion
4	10.1016/j.jff.2015.11.005	arabinogalactan and fructooligosaccharides improve the gut barrier function in distinct areas of the colon in the simulator of the human intestinal microbial ecosystem	Shime
4	10.1016/j.jff.2015.12.002	in vitro digestion and fermentation of sialyllactoses by infant gut microflora	In-vitro Digestion
2	10.1016/j.jff.2016.01.018	the effect of agave fructan products on the activity and composition of the microbiota determined in a dynamic in vitro model of the human proximal large intestine	In-vitro Model
2	10.1016/j.jff.2016.08.002	co-encapsulation of lactobacillus helveticus cells and green tea extract influence on cell survival in simulated gastrointestinal conditions	Simulated Gastrointestinal
2	10.1016/j.jff.2016.09.006	catabolism of raw and cooked green pepper capsicum annuum polyphenolic compounds after simulated gastrointestinal digestion and faecal fermentation	Simulated Gastrointestinal
2	10.1016/j.jff.2017.01.030	fate of chitin-glucan in the human gastrointestinal tract as studied in a dynamic gut simulator shime	Shime

6	10.1016/j.jff.2017.02.004	long chain arabinoxylans shift the mucosa-associated microbiota in the proximal colon of the simulator of the human intestinal microbial ecosystem m-shime	In-vitro Model
2	10.1016/j.jff.2017.02.033	bioaccessibility of polyphenolic compounds of raw and cooked cardoon cynara cardunculus l after simulated gastrointestinal digestion and fermentation by human colonic microbiota	Simulated Gastroin-testinal
6	10.1016/j.jff.2017.02.035	a three-stage continuous culture approach to study the impact of probiotics prebiotics and fat intake on faecal microbiota relevant to an over 60 s population	In-vitro Model
3	10.1016/j.jff.2017.03.031	study on the digestion of milk with prebiotic carbohydrates in a simulated gastrointestinal model	Simulated Gastroin-testinal
2	10.1016/j.jff.2017.05.019	survival of commercial probiotic strains in dark chocolate with high cocoa and phenols content during the storage and in a static in vitro digestion model	Simulated Gastroin-testinal
2	10.1016/j.jff.2017.05.022	absorption and degradation of sulfated polysaccharide from pacific abalone in in vitro and in vivo models	In-vitro Model
2	10.1016/j.jff.2017.07.011	purification and characterization of angiotensin-converting enzyme-inhibitory peptides from nile tilapia oreochromis niloticus skin gelatine produced by an enzymatic membrane reactor	Simulated Gastroin-testinal
3	10.1016/j.jff.2017.07.030	stability and biological activity of merlot vitis vinifera grape pomace phytochemicals after simulated in vitro gastrointestinal digestion and colonic fermentation	Simulated Colon
2	10.1016/j.jff.2017.08.004	bioaccessibility and antioxidant activity of phenolics in native and fermented prinsepia utilis royle seed during a simulated gastrointestinal digestion in vitro	Simulated Gastroin-testinal
2	10.1016/j.jff.2017.09.008	aronia aronia melanocarpa phenolics bioavailability in a combined in vitro digestioncaco-2 cell model is structure and colon region dependent	In-vitro Digestion
2	10.1016/j.jff.2017.10.040	in vitro digestion by saliva simulated gastric and small intestinal juices and fermentation by human fecal microbiota of sulfated polysaccharides from gracilaria rubra	In-vitro Digestion
2	10.1016/j.jff.2018.02.011	in vitro evaluation of the kinetics of the release of phenolic compounds from guava psidium guajava l fruit	In-vitro Digestion
2	10.1016/j.jff.2018.03.023	plant sterols and human gut microbiota relationship an in vitro colonic fermentation study	In-vitro Digestion
2	10.1016/j.jff.2018.04.030	chemical and nutritional properties of white bread leavened by lactic acid bacteria	In-vitro Digestion
4	10.1016/j.jff.2018.05.053	a combination of xylooligosaccharides and a polyphenol blend affect microbial composition and activity in the distal colon exerting immunomodulating properties on human cells	Gut Model
4	10.1016/j.jff.2018.07.056	study of antioxidant capacity and metabolization of quebracho and chestnut tannins through in vitro gastrointestinal digestion-fermentation	In-vitro Digestion
2	10.1016/j.jff.2019.01.032	untargeted metabolomic evaluation of mango bagasse and mango bagasse based confection under in vitro simulated colonic fermentation	Simulated Colon
2	10.1016/j.jff.2019.02.004	probiotic potential and biofilm inhibitory activity of lactobacillus casei group strains isolated from infant feces	Simulated Gastroin-testinal
4	10.1016/j.jff.2019.04.026	degradation of fibres from fruit by-products allows selective modulation of the gut bacteria in an in vitro model of the proximal colon	In-vitro Colon
2	10.1016/j.jff.2019.05.024	study of in vitro digestion of tenebrio molitor flour for evaluation of its impact on the human gut microbiota	In-vitro Digestion

2	10.1016/j.jff.2019.05.026	toward an accessible and robust in vitro approach to evaluate bacterial viability in the upper gastro-intestinal tract a gastro-intestinal digestive simulator gids combined with alternative methods to plating	In-vitro Digestion
6	10.1016/j.jff.2019.06.008	bioaccessibility and transformation pathways of phenolic compounds in processed mulberry morus alba l leaves after in vitro gastrointestinal digestion and faecal fermentation	Simulated Gastrointestinal
2	10.1016/j.jff.2019.103440	wheat bran thermal treatment in a hot air oven does not affect the fermentation and colonisation process by human faecal microbiota	In-vitro Gastrointestinal
2	10.1016/j.jff.2019.103484	2-fucosyllactose alters the composition and activity of gut microbiota from formula-fed infants receiving complementary feeding in a validated intestinal model	Gut Model
2	10.1016/j.jff.2019.103555	effect of a chinese medical nutrition therapy diet on gut microbiota and short chain fatty acids in the simulator of the human intestinal microbial ecosystem shime	Shime
4	10.1016/j.jff.2019.103668	an evaluation of the prebiotic potential of microbial levans from erwinia sp 10119	Gut Model
2	10.1016/j.jff.2020.103819	modulation of equol production via different dietary regimens in an artificial model of the human colon	Artificial Gut
2	10.1016/j.jff.2020.103830	impact of functional flours from pineapple by-products on human intestinal microbiota	Simulated Gastrointestinal
4	10.1016/j.jff.2020.104087	effect of bean structure on microbiota utilization of plant nutrients an in-vitro study using the simulator of the human intestinal microbial ecosystem shime	Shime
2	10.1016/j.jff.2020.104335	effects of soybean protein isolates and peptides on the growth and metabolism of lactobacillus rhamnosus	Simulated Gastrointestinal
2	10.1016/j.jff.2021.104597	measuring the effect of mankai wolffia globosa on the gut microbiota and its metabolic output using an in vitro colon model	In-vitro Colon
2	10.1016/j.jff.2021.104705	non-digestible galactomannan oligosaccharides from cassia seed gum modulate microbiota composition and metabolites of human fecal inoculum	Simulated Gastrointestinal
4	10.1016/j.jff.2021.104735	differential fermentation of raw and processed high-amylose and waxy maize starches in the simulator of the human intestinal microbial ecosystem shime	Shime
2	10.1016/j.jff.2021.104766	assessing the in vitro digestion of sesbania gum a galactomannan from s cannabina and subsequent impact on the fecal microbiota	In-vitro Digestion
2	10.1016/j.jff.2021.104820	modulation of human gut microbiota composition and metabolites by arabinogalactan and bifidobacterium longum subsp longum bb536 in the simulator of the human intestinal microbial ecosystem shime	Shime
3	10.1016/j.jhazmat.2012.05.042	arsenic undergoes significant speciation changes upon incubation of contaminated rice with human colon micro biota	Shime
4	10.1016/j.jhazmat.2018.05.011	influence of gastrointestinal tract on metabolism of bisphenol a as determined by in vitro simulated system	Shime
2	10.1016/j.jhazmat.2021.127088	coexistence of silver ion and tetracycline at environmentally relevant concentrations greatly enhanced antibiotic resistance gene development in activated sludge bioreactor	Reactor
3	10.1016/j.jhin.2021.04.014	the effect of intestinal microbiota dysbiosis on growth and detection of carbapenemase-producing enterobacterales within an invitro gut model	In-vitro Model
2	10.1016/j.jnutbio.2016.03.007	metabolic transformations of dietary polyphenols comparison between in vitro colonic and hepatic models and in vivo urinary metabolites	In-vitro Colon

6	10.1016/j.jpba.2019.07.016	optimization of an in vitro gut microbiome biotransformation platform with chlorogenic acid as model compound from fecal sample to biotransformation product identification	In-vitro Gastroin-testinal
2	10.1016/j.jwpe.2019.101110	performance and microbial community structure of aerobic granular bioreactors at different operational temperature	Reactor
2	10.1016/j.lwt.2012.10.007	in vitro gastrointestinal digestion of hibiscus sabdariffa l the use of its natural matrix to improve the concentration of phenolic compounds in gut	In-vitro Digestion
2	10.1016/j.lwt.2014.08.040	a novel hypromellose capsule with acid resistance properties permits the targeted delivery of acid-sensitive products to the intestine	Shime
2	10.1016/j.lwt.2014.12.014	development of human colonic microbiota in the computer-controlled dynamic simulator of the gastrointestinal tract simgi	Reactor
2	10.1016/j.lwt.2015.05.004	survival of lactobacillus rhamnosus em1107 in simulated gastrointestinal conditions and its inhibitory effect against pathogenic bacteria in semi-hard goat cheese	Simulated Gastroin-testinal
2	10.1016/j.lwt.2015.06.049	effect of different types of encapsulation on the survival of lactobacillus plantarum during storage with inulin and in vitro digestion	Simulated Gastroin-testinal
2	10.1016/j.lwt.2016.06.003	characterization of lactobacilli strains derived from cocoa fermentation in the south of bahia for the development of probiotic cultures	Simulated Gastroin-testinal
2	10.1016/j.lwt.2016.08.040	probiotic characteristics of bacillus strains isolated from korean traditional soy sauce	Simulated Gastroin-testinal
8	10.1016/j.lwt.2016.12.013	spray drying probiotics along with maoluang juice plus tiliacora triandra gum for exposure to the in vitro gastrointestinal environments	Simulated Gastroin-testinal
2	10.1016/j.lwt.2017.08.031	simulated gastrointestinal digestion of nisin and interaction between nisin and bile	Simulated Gastroin-testinal
2	10.1016/j.lwt.2017.11.048	technological potential of bifidobacterium aesculapii strains for fermented soymilk production	In-vitro Model
2	10.1016/j.lwt.2018.03.027	survival of probiotics in soyoghurt plus mulberry cv chiang mai 60 leaf extract during refrigerated storage and their ability to tolerate gastrointestinal transit	In-vitro Gastroin-testinal
2	10.1016/j.lwt.2018.06.012	in vitro fermentation of six kinds of edible mushrooms and its effects on fecal microbiota composition	In-vitro Digestion
2	10.1016/j.lwt.2018.08.010	guava orange and passion fruit by-products characterization and its impacts on kinetics of acidification and properties of probiotic fermented products	Simulated Gastroin-testinal
2	10.1016/j.lwt.2018.10.071	in vitro evaluation of the bioaccessibility of phenolic acids in different whole wheats as potential prebiotics	In-vitro Digestion
5	10.1016/j.lwt.2018.11.029	in-vitro digestion by simulated gastrointestinal juices of lactobacillus rhamnosus cultured with mulberry oligosaccharides and subsequent fermentation with human fecal inocula	Simulated Gastroin-testinal
2	10.1016/j.lwt.2018.11.074	influence of inulin rich carbohydrates from jerusalem artichoke helianthus tuberosus l tubers on probiotic properties of lactobacillus strains	Simulated Gastroin-testinal

6	10.1016/j.lwt.2018.12.013	pulsed electric field improved protein digestion of beef during in-vitro gastrointestinal simulation	Simulated Gastrointestinal
2	10.1016/j.lwt.2019.03.015	listeria monocytogenes survival in raw atlantic salmon salmo salar fillet under in vitro simulated gastrointestinal conditions by culture qpcr and pma-qpcr detection methods	Simulated Gastrointestinal
2	10.1016/j.lwt.2019.05.079	effects of in vitro simulated gastrointestinal digestion on the antioxidant -glucosidase and -amylase inhibitory activities of water-soluble polysaccharides from opilia amentacea roxb fruit	Simulated Gastrointestinal
4	10.1016/j.lwt.2019.108623	simulated gastrointestinal digestion and in vitro colonic fermentation of carob polyphenols bioaccessibility and bioactivity	Simulated Gastrointestinal
2	10.1016/j.lwt.2019.108703	effects of prebiotic carbohydrates on the growth promotion and cholesterol-lowering abilities of compound probiotics in vitro	Simulated Gastrointestinal
2	10.1016/j.lwt.2019.108761	evaluation of xylooligosaccharides and fructooligosaccharides on digestive enzymes hydrolysis and as a nutrient for different probiotics and salmonella typhimurium	In-vitro Digestion
2	10.1016/j.lwt.2020.109196	behavior of bacillus coagulans unique is2 spores during passage through the simulator of human intestinal microbial ecosystem shime model	Shime
2	10.1016/j.lwt.2020.109206	changes in the antioxidant properties of rice bran protein isolate upon simulated gastrointestinal digestion	Simulated Gastrointestinal
2	10.1016/j.lwt.2020.109322	in vitro fermentation of raffinose to unravel its potential as prebiotic ingredient	In-vitro Model
2	10.1016/j.lwt.2020.109766	effects of digested jabuticaba myrciaria jaboticaba vell berg by-product on growth and metabolism of lactobacillus and bifidobacterium indicate prebiotic properties	Simulated Gastrointestinal
4	10.1016/j.lwt.2020.109876	effect of fermented sausages with probiotic enterococcus faecium crl 183 on gut microbiota using dynamic colonic model	Shime
2	10.1016/j.lwt.2020.110233	technological features of saccharomyces cerevisiae var boulardii for potential probiotic wheat beer development	In-vitro Gastrointestinal
4	10.1016/j.lwt.2020.110235	in vitro gastrointestinal digestion and fermentation properties of ganoderma lucidum spore powders and their extracts	In-vitro Digestion
4	10.1016/j.lwt.2020.110552	stability and mechanism of phenolic compounds from raspberry extract under in vitro gastrointestinal digestion	In-vitro Digestion
2	10.1016/j.lwt.2020.110636	in vitro gastrointestinal digestion and colonic fermentation of phenolic compounds in uv-c irradiated pineapple ananas comosus snack-bars	Simulated Gastrointestinal
2	10.1016/j.lwt.2021.110858	acerola by-product may improve the in vitro gastrointestinal resistance of probiotic strains in a plant-based fermented beverage	Simulated Gastrointestinal
4	10.1016/j.lwt.2021.110921	modulation and metabolism of obesity-associated microbiota in a dynamic simulator of the human gut microbiota	Gut Model
2	10.1016/j.lwt.2021.111348	investigating the effect of in vitro gastrointestinal digestion on the stability bioaccessibility and biological activities of baobab adansonia digitata fruit polyphenolics	In-vitro Digestion

2	10.1016/j.lwt.2021.111386	cross-linking treatment of arabinoxylan improves its antioxidant and hypoglycemic activities after simulated in vitro digestion	In-vitro Digestion
2	10.1016/j.lwt.2021.111423	encapsulation of lactobacillus reuteri in w1ow2 double emulsions formulation storage and in vitro gastro-intestinal digestion stability	In-vitro Digestion
2	10.1016/j.lwt.2021.111806	clostridium perfringens suppressing activity in black soldier fly protein preparations	In-vitro Model
2	10.1016/j.lwt.2021.111882	effects of crude sphallerocarpus gracilis polysaccharides as potential prebiotics on acidifying activity and growth of probiotics in fermented milk	Simulated Gastrointestinal
2	10.1016/j.lwt.2021.112434	viability and stability evaluation of lactobacillus casei lc03 co-encapsulated with red onion allium cepa l peel extract	Simulated Gastrointestinal
2	10.1016/j.lwt.2021.112713	physicochemical properties structure and digestibility in simulated gastrointestinal environment of bread added with green lentil flour	Simulated Gastrointestinal
2	10.1016/j.medj.2020.07.001	primary human colonic mucosal barrier crosstalk with super oxygen-sensitive faecalibacterium prausnitzii in continuous culture	Continous Culture
2	10.1016/j.mimet.2004.05.007	a new real time pcr taqman pcr system for detection of the16s rdna gene associated with fecal bacteria	Reactor
2	10.1016/j.mimet.2013.08.008	evaluation of microbial community reproducibility stability and composition in a human distal gut chemostat model	Chemostat
6	10.1016/j.mimet.2013.11.015	three-stage continuous culture system with a self-generated anaerobia to study the regionalized metabolism of the human gut microbiota	Reactor
2	10.1016/j.mimet.2014.08.022	to pool or not to pool impact of the use of individual and pooled fecal samples for in vitro fermentation studies	In-vitro Model
5	10.1016/j.mimet.2014.11.007	simulating distal gut mucosal and luminal communities using packed-column biofilm reactors and an in vitro chemostat model	Reactor
2	10.1016/j.mimet.2015.07.019	evaluation of an optimal preparation of human standardized fecal inocula for in vitro fermentation studies	In-vitro Model
2	10.1016/j.mimet.2018.07.003	real-time monitoring of ht29 epithelial cells as an in vitro model for assessing functional differences among intestinal microbiotas from different human population groups	In-vitro Model
2	10.1016/j.mimet.2021.106187	development of a semi-dynamic in vitro model and its testing using probiotic bacillus coagulans gbi-30 6086 in orange juice and yogurt	In-vitro Digestion
4	10.1016/j.mtbio.2020.100092	enzyme functionalized microgels enable precise regulation of dissolved oxygen and anaerobe culture	Reactor
3	10.1016/j.numecd.2008.07.005	potential prebiotic activity of oligosaccharides obtained by enzymatic conversion of durum wheat insoluble dietary fibre into soluble dietary fibre	Gut Model
2	10.1016/j.nutres.2020.12.013	differences in the gut microbiota between young and elderly persons in korea	In-vitro Model
2	10.1016/j.omtn.2018.04.012	thermoresponsive bacteriophage nanocarrier as a gene delivery vector targeted to the gastrointestinal tract	Simulated Gastrointestinal
2	10.1016/j.pep.2017.07.005	secretory expression of -mannanase from bacillus circulans nt 67 in lactobacillus plantarum	Bioreactor
2	10.1016/j.pharma.2014.04.008	faecal microbiota transplantation a sui generis biological drug not a tissue	Bioreactor
2	10.1016/j.procbio.2020.07.024	high purity prebiotic isomalto-oligosaccharides production by cell associated transglucosidase of isolated strain debaryomyces hansenii scy204 and selective fermentation by saccharomyces cerevisiae syi065	Bioreactor

2	10.1016/j.prp.2021.153511	mist1 promoted inflammation in colitis model via k-atpase nlrp3 inflammasome by snai1	In-vitro Model
2	10.1016/j.psep.2020.11.035	impacts of different operational temperatures and organic loads in anaerobic co-digestion of food waste and sewage sludge on the fate of sars-cov-2	Reactor
5	10.1016/j.resmic.2015.09.006	diet drives quick changes in the metabolic activity and composition of human gut microbiota in a validated in vitro gut model	In-vitro Model
2	10.1016/j.rvsc.2021.08.011	inclusion of small intestinal absorption and simulated mucosal surfaces further improve the mucosal simulator of the canine intestinal microbial ecosystem m-scime	In-vitro Model
4	10.1016/j.scitotenv.2009.08.014	bioaccessibility of mercury from traditional northern country foods measured using an in vitro gastrointestinal model is independent of mercury concentration	In-vitro Gastroin-testinal
4	10.1016/j.scitotenv.2018.07.003	nutritional status affects the bioaccessibility and speciation of arsenic from soils in a simulator of the human intestinal microbial ecosystem	Shime
2	10.1016/j.scitotenv.2019.135227	lead bioaccessibility in farming and mining soils the influence of soil properties types and human gut microbiota	Shime
4	10.1016/j.scitotenv.2020.141415	colistin and amoxicillin combinatorial exposure alters the human intestinal microbiota and antibiotic resistome in the simulated human intestinal microbiota	Shime
2	10.1016/j.scitotenv.2020.143983	are silver nanoparticles better than triclosan as a daily antimicrobial answers from the perspectives of gut microbiome disruption and pathogenicity	Shime
2	10.1016/j.scitotenv.2021.146264	influence of the co-exposure of microplastics and tetrabromobisphenol a on human gut simulation in vitro with human cell caco-2 and gut microbiota	Gut Simulation
2	10.1016/j.sjbs.2021.06.084	biofilm producing indigenous bacteria isolated from municipal sludge and their nutrient removal ability in moving bed biofilm reactor from the wastewater	Reactor
2	10.1016/j.snb.2016.07.120	designing an in-vitro gas profiling system for human faecal samples	Simulated Colon
6	10.1016/j.syapm.2015.11.002	functional anatomy of the colonic bioreactor impact of antibiotics and saccharomyces boulardii on bacterial composition in human fecal cylinders	Bioreactor
2	10.1016/j.talanta.2012.04.013	salt release monitoring with specific sensors in in vitro oral and digestive environments from soft cheeses	In-vitro Digestion
2	10.1016/j.tiv.2021.105078	an in vitro model for microbial fructoselysine degradation shows substantial interindividual differences in metabolic capacities of human fecal slurries	In-vitro Model
2	10.1016/j.watres.2011.05.028	analysis of the bacterial community in a laboratory-scale nitrification reactor and a wastewater treatment plant by 454-pyrosequencing	Reactor
4	10.1016/j.ymeth.2018.08.003	using bioreactors to study the effects of drugs on the human microbiota	Bioreactor
2	10.1016/j.yrtph.2004.08.005	ciprofloxacin at low levels disrupts colonization resistance of human fecal microflora growing in chemostats	Chemostat
2	10.1016/j.yrtph.2005.06.005	antibiotics in the human food chain establishing no effect levels of tetracycline neomycin and erythromycin using a chemostat model of the human colonic microflora	Chemostat
2	10.1016/j.yrtph.2013.08.011	impact of cyadox on human colonic microflora in chemostat models	Chemostat
2	10.1016/j.yrtph.2015.07.008	microbiological toxicity of tilmicosin on human colonic microflora in chemostats	Chemostat
2	10.1016/S0003-9969(03)00154-7	modelling oral malodour in a longitudinal study	In-vitro Model
3	10.1016/s0168-1605(98)00182-2	the effect of probiotic strains on the microbiota of the simulator of the human intestinal microbial ecosystem shime	In-vitro Model
2	10.1016/S0168-6496(01)00181-7	bacterial community dynamics in liquid swine manure during storage molecular analysis using dggepcr of 16s rdna	Reactor

2	10.1016/s0176-6724(87)80138-4	antibiotic residues and r-plasmid selection are in vitro methods good models	Chemostat
2	10.1016/S0377-8401(00)00193-0	effects of dry plant extracts on fermentation and methanogenesis in continuous culture of rumen microbes	Fermenter
2	10.1016/S0377-8401(02)00221-3	effects of dry plant extracts on feed degradation and the production of rumen microbial biomass in a dual outflow fermenter	Fermenter
6	10.1016/S0377-8401(02)00286-9	the use of a model ileum to investigate the effects of novel and existing antimicrobials on indigenous porcine gastrointestinal microflora using vancomycin as an example	In-vitro Model
2	10.1016/S0399-8320(10)70025-7	functional biostructure of colonic microbiota central fermenting area germinal stock area and separating mucus layer in healthy subjects and patients with diarrhea treated with saccharomyces boulardii biostructure fonctionnelle du microbiote colique zone de fermentation centrale zone de rserve germinale et couche de mucus sparatrice chez les sujets sains et chez les patients atteints de diarrhe traits par saccharomyces boulardii	Bioreactor
6	10.1017/S0007114500000325	in vitro fermentability of dextran oligodextran and maltodextrin by human gut bacteria	Gut Model
2	10.1017/S0007114509991796	set up of a new in vitro model to study dietary fructans fermentation in formula-fed babies	In-vitro Model
3	10.1017/S0007114511004697	a randomised crossover study investigating the effects of galacto-oligosaccharides on the faecal microbiota in men and women over 50 years of age	Continous Culture
2	10.1017/S0007114511006647	in vitro fermented nuts exhibit chemopreventive effects in ht29 colon cancer cells	In-vitro Digestion
2	10.1017/S0007114512000281	wholegrain oat-based cereals have prebiotic potential and low glycaemic index	In-vitro Digestion
2	10.1017/s0007114599001749	the effect of a model melanoidin mixture on faecal bacterial populations in vitro	In-vitro Model
2	10.1017/S0029665120007867	monitoring food digestion with magnetic resonance techniques	In-vitro Digestion
8	10.1021/acs.analchem.5b02688	validated high resolution mass spectrometry-based approach for metabolomic fingerprinting of the human gut phenotype	Shime
2	10.1021/acs.est.0c04939	direct medium-chain carboxylic acid oil separation from a bioreactor by an electrodialysisphase separation cell	Bioreactor
4	10.1021/acs.est.5b03046	in vitro method to assess soil arsenic metabolism by human gut microbiota arsenic speciation and distribution	Shime
7	10.1021/acs.est.6b01533	arsenic metabolism and toxicity influenced by ferric iron in simulated gastrointestinal tract and the roles of gut microbiota	Simulated Gastroin-testinal
2	10.1021/acs.est.6b04477	genome-resolved meta-omics ties microbial dynamics to process performance in biotechnology for thiocyanate degradation	Reactor
2	10.1021/acs.est.8b07303	importance of species sorting and immigration on the bacterial assembly of different-sized aggregates in a full-scale aerobic granular sludge plant	Reactor
4	10.1021/acs.jafc.0c00212	in vitro gastrointestinal digestion of palm olein and palm stearin-in-water emulsions with different physical states and fat contents	In-vitro Gastroin-testinal
6	10.1021/acs.jafc.0c02088	valorization of persimmon and blueberry byproducts to obtain functional powders in vitro digestion and fermentation by gut microbiota	In-vitro Digestion
8	10.1021/acs.jafc.0c02225	potential of high- and low-acetylated galactoglucomannooligosaccharides as modulators of the microbiota composition and their activity a comparison using the in vitro model of the human colon tim-2	In-vitro Colon

3	10.1021/acs.jafc.0c07597	vitamin b12 enriched in spinach and its effects on gut microbiota	In-vitro Gastroin-testinal
2	10.1021/acs.jafc.1c00237	comparison of different soluble dietary fibers during the in vitro fermentation process	Simulated Colon
2	10.1021/acs.jafc.1c00542	stability of enzyme-modified flavonoid c-and o-glycosides from common buckwheat sprout extracts during in vitro digestion and colonic fermentation	In-vitro Digestion
2	10.1021/acs.jafc.1c03158	red wine high-molecular-weight polyphenolic complex an emerging modulator of human metabolic disease risk and gut microbiota	Simulated Gastroin-testinal
4	10.1021/acs.jafc.5b05535	structure of brewers spent grain lignin and its interactions with gut microbiota in vitro	In-vitro Colon
2	10.1021/acs.jafc.6b04561	chemopreventive potential of powdered red wine pomace seasonings against colorectal cancer in ht-29 cells	In-vitro Gastroin-testinal
4	10.1021/acs.jafc.6b04854	influence of fermentation with different lactic acid bacteria and in vitro digestion on the biotransformation of phenolic compounds in fermented pomegranate juices	Simulated Gastroin-testinal
4	10.1021/acs.jafc.6b05110	effect of bioprocessing on the in vitro colonic microbial metabolism of phenolic acids from rye bran fortified breads	In-vitro Colon
2	10.1021/acs.jafc.7b01707	bioavailability of black tea theaflavins absorption metabolism and colonic catabolism	In-vitro Model
2	10.1021/acs.jafc.7b02781	factors affecting the bioaccessibility and intestinal transport of difenoconazole hexaconazole and spirodiclofen in human caco-2 cells following in vitro digestion	In-vitro Digestion
2	10.1021/acs.jafc.7b04100	acemannan and fructans from aloe vera aloe barbadensis miller plants as novel prebiotics	Bioreactor
2	10.1021/acs.jafc.7b05466	inhibition of oral pathogens adhesion to human gingival fibroblasts by wine polyphenols alone and in combination with an oral probiotic	In-vitro Model
3	10.1021/acs.jafc.7b06149	in vitro model to assess arsenic bioaccessibility and speciation in cooked shrimp	In-vitro Model
3	10.1021/acs.jafc.8b00644	encapsulation of vitamin e and soy isoflavone using spiral dextrin comparative structural characterization release kinetics and antioxidant capacity during simulated gastrointestinal tract	Simulated Gastroin-testinal
2	10.1021/acs.jafc.8b01604	bioaccessibility during in vitro digestion and antiproliferative effect of bioactive compounds from andean berry vaccinium meridionale swartz juice	In-vitro Digestion
2	10.1021/acs.jafc.8b02240	beta-glucan and phenolic compounds their concentration and behavior during in vitro gastrointestinal digestion and colonic fermentation of different barley-based food products	In-vitro Gastroin-testinal
4	10.1021/acs.jafc.8b03469	fecal microbiota responses to bran particles are specific to cereal type and in vitro digestion methods that mimic upper gastrointestinal tract passage	In-vitro Digestion
3	10.1021/acs.jafc.8b04077	effect of food thermal processing on the composition of the gut microbiota	In-vitro Digestion
2	10.1021/acs.jafc.8b06480	digestion and colonic fermentation of raw and cooked opuntia ficus-indica cladodes impacts bioaccessibility and bioactivity	In-vitro Digestion
2	10.1021/acs.jafc.9b00351	insight of stability of procyanidins in free and liposomal form under an in vitro digestion model study of bioaccessibility kinetic release profile degradation and antioxidant activity	In-vitro Digestion
4	10.1021/acs.jafc.9b00970	in vitro digestion and fermentation of three polysaccharide fractions from laminaria japonica and their impact on lipid metabolism-associated human gut microbiota	In-vitro Digestion

4	10.1021/acs.jafc.9b03905	protective effect of an avocado peel polyphenolic extract rich in proanthocyanidins on the alterations of colonic homeostasis induced by a high-protein diet	Bioreactor
2	10.1021/acs.jafc.9b05372	evaluation of oral bioaccessibility of aged citrus peel extracts encapsulated in different lipid-based systems a comparison study using different in vitro digestion models	In-vitro Digestion
2	10.1021/acs.jafc.9b05912	fate and bioaccessibility of iodine in food prepared from agronomically biofortified wheat and rice and impact of cofertilization with zinc and selenium	In-vitro Gastroin-testinal
4	10.1021/acs.jafc.9b06477	phytochemical profile bioactivity and prebiotic potential of bound phenolics released from rice bran dietary fiber during in vitro gastrointestinal digestion and colonic fermentation	In-vitro Gastroin-testinal
2	10.1021/acs.jafc.9b07680	influence of food matrix on the bioaccessibility of fruit polyphenolic compounds	In-vitro Digestion
8	10.1021/acs.jproteome.7b00683	metabolic fate of 13c-labeled polydextrose and impact on the gut microbiome a triple-phase study in a colon simulator	Simulated Colon
2	10.1021/acsinfecdis.0c00634	mucin-degrading microbes release monosaccharides that chemoattract clostridioides difficile and facilitate colonization of the human intestinal mucus layer	Bioreactor
2	10.1021/acsomega.1c00302	manipulation of gut microbiota using acacia gum polysaccharide	In-vitro Colon
3	10.1021/es050829j	removal and inactivation of waterborne viruses using zerovalent iron	Reactor
4	10.1021/es062410e	gastrointestinal microbes increase arsenic bioaccessibility of ingested mine tailings using the simulator of the human intestinal microbial ecosystem	In-vitro Gastroin-testinal
3	10.1021/es1010626	mobility of polycyclic aromatic hydrocarbons in the gastrointestinal tract assessed using an in vitro digestion model with sorption rectification	In-vitro Digestion
2	10.1021/es1025849	mobilization of soil-bound residue of organochlorine pesticides and polycyclic aromatic hydrocarbons in an in vitro gastrointestinal model	In-vitro Gastroin-testinal
2	10.1021/es103710a	bioaccessibility of metal cations in soil is linearly related to its water exchange rate constant	In-vitro Gastroin-testinal
4	10.1021/es505521w	metagenomics shows that low-energy anaerobic-aerobic treatment reactors reduce antibiotic resistance gene levels from domestic wastewater	Reactor
8	10.1021/es900544c	biovolatilization of metalloids by intestinal microorganisms in the simulator of the human intestinal microbial ecosystem	In-vitro Gastroin-testinal
8	10.1021/es900837y	nutritional status and gastrointestinal microbes affect arsenic bioaccessibility from soils and mine tailings in the simulator of the human intestinal microbial ecosystem	In-vitro Gastroin-testinal
4	10.1021/jf0478399	degradation of phytate by high-phytase saccharomyces cerevisiae strains during simulated gastrointestinal digestion	Simulated Gastroin-testinal
8	10.1021/jf3008037	formation of phenolic microbial metabolites and short-chain fatty acids from rye wheat and oat bran and their fractions in the metabolical in vitro colon model	In-vitro Colon
6	10.1021/jf303165w	gut microbial metabolism of polyphenols from black tea and red winegrape juice is source-specific and colon-region dependent	In-vitro Gastroin-testinal

4	10.1021/jf4001814	effects of disintegration on in vitro fermentation and conversion patterns of wheat aleurone in a metabolical colon model	In-vitro Model
4	10.1021/jf401738x	interactions of a lignin-rich fraction from brewers spent grain with gut microbiota in vitro	In-vitro Colon
2	10.1021/jf402137r	a dried yeast fermentate selectively modulates both the luminal and mucosal gut microbiota and protects against inflammation as studied in an integrated in vitro approach	Shime
3	10.1021/jf4024195	release of small phenolic compounds from brewers spent grain and its lignin fractions by human intestinal microbiota in vitro	In-vitro Colon
6	10.1021/jf402816r	lactobacillus plantarum ifpl935 favors the initial metabolism of red wine polyphenols when added to a colonic microbiota	Shime
4	10.1021/jf404305y	production and in vitro fermentation of soluble non-digestible feruloylated oligo- and polysaccharides from maize and wheat brans	In-vitro Digestion
2	10.1021/jf504659n	protection of epigallocatechin gallate against degradation during in vitro digestion using apple pomace as a carrier	Simulated Gastroin-testinal
3	10.1021/jf800101s	metabolism of the lignan macromolecule into enterolignans in the gastrointestinal lumen as determined in the simulator of the human intestinal microbial ecosystem	Artificial Gut
6	10.1021/jf800284d	effects of resistant starch type iii polymorphs on human colon microbiota and short chain fatty acids in human gut models	Gut Model
2	10.1021/jf801944a	in vitro digestion characteristics of unprocessed and processed whole grains and their components	In-vitro Digestion
2	10.1021/jf802659u	factors affecting the bioaccessibility of polybrominated diphenylethers in an in vitro digestion model	In-vitro Digestion
2	10.1021/jf900492h	bioprocessing of wheat bran improves in vitro bioaccessibility and colonic metabolism of phenolic compounds	In-vitro Model
2	10.1021/jf900788c	in vitro fermentation of oat flours from typical and high -glucan oat lines	In-vitro Digestion
2	10.1021/pr5011247	metabolomic analysis of human fecal microbiota a comparison of feces-derived communities and defined mixed communities	Reactor
2	10.1023/b:ddas.0000020508.64440.73	the metabolic activity of fecal microbiota from healthy individuals and patients with inflammatory bowel disease	In-vitro Model
2	10.1034/j.1600-051X.2003.00307.x	effects of dynamic fluid activity from an electric toothbrush on in vitro oral biofilms	In-vitro Model
2	10.1038/ejcn.2013.294	the effects of probiotic bacteria on glycaemic control in overweight men and women a randomised controlled trial	In-vitro Model
11	10.1038/ismej.2012.158	butyrate-producing clostridium cluster xiva species specifically colonize mucins in an in vitro gut model	Gut Model
2	10.1038/ismej.2014.204	gene expression analysis of e coli strains provides insights into the role of gene regulation in diversification	Chemostat
3	10.1038/ncomms11535	a microfluidics-based in vitro model of the gastrointestinal human-microbe interface	In-vitro Model
2	10.1038/npjbiofilms.2016.16	arabinoxylans inulin and lactobacillus reuteri 1063 repress the adherent-invasive escherichia coli from mucus in a mucosa-comprising gut model	Gut Model
4	10.1038/pr.2015.10	bioactive peptides released from in vitro digestion of human milk with or without pasteurization	In-vitro Digestion
3	10.1038/s41396-019-0498-5	drivers of human gut microbial community assembly coadaptation determinism and stochasticity	Artificial Gut

3	10.1038/s41522-021-00184-w	biofilms harbour clostridioides difficile serving as a reservoir for recurrent infection	Gut Model
4	10.1038/s41596-021-00537-x	an in vitro batch fermentation protocol for studying the contribution of food to gut microbiota composition and functionality	In-vitro Digestion
4	10.1038/s41598-017-08478-w	investigation into the stability and culturability of chinese enterotypes	Chemostat
3	10.1038/s41598-017-12984-2	microscale bioreactors for in situ characterization of gi epithelial cell physiology	In-vitro Model
4	10.1038/s41598-017-17901-1	investigation of bioaccessibility of cu fe mn and zn in market vegetables in the colon using pbet combined with shime	Shime
4	10.1038/s41598-018-37547-x	effects of defined gut microbial ecosystem components on virulence determinants of clostridioides difficile	In-vitro Model
2	10.1038/s41598-019-47843-9	migration of bacteriocins across gastrointestinal epithelial and vascular endothelial cells as determined using in vitro simulations	Simulated Gastroin-testinal
3	10.1038/s41598-019-49497-z	the effect of 2-fucosyllactose on simulated infant gut microbiome and metabolites a pilot study in comparison to gos and lactose	Gut Model
2	10.1038/s41598-020-69533-7	protective action of bacillus clausii probiotic strains in an in vitro model of rotavirus infection	In-vitro Model
4	10.1038/s41598-020-78591-w	a novel 3d in vitro model of the human gut microbiota	In-vitro Model
6	10.1038/s41598-020-79947-y	optimization of probiotic therapeutics using machine learning in an artificial human gastrointestinal tract	Artificial Gut
2	10.1038/s41598-020-80306-0	the alga euglena gracilis stimulates faecalibacterium in the gut and contributes to increased defecation	In-vitro Model
2	10.1038/s41598-021-84167-z	growth stimulation of bifidobacterium from human colon using daikenchuto in an in vitro model of human intestinal microbiota	In-vitro Model
4	10.1038/s41598-021-94210-8	w27 iga suppresses growth of escherichia in an in vitro model of the human intestinal microbiota	In-vitro Model
2	10.1038/s42003-019-0418-2	transcriptome analysis of activated sludge microbiomes reveals an unexpected role of minority nitrifiers in carbon metabolism	Reactor
3	10.1038/srep01110	microbiotas from uc patients display altered metabolism and reduced ability of lab to colonize mucus	Gut Model
2	10.1038/srep39854	intestinal microbiota as a tetrahydrobiopterin exogenous source in hph-1 mice	Chemostat
4	10.1039/c0mb00023j	drug metabolome of the simvastatin formed by human intestinal microbiota in vitro	In-vitro Colon
2	10.1039/c1ee01377g	metabolite-based mutualism between pseudomonas aeruginosa pa14 and enterobacter aerogenes enhances current generation in bioelectrochemical systems	Reactor
4	10.1039/c2fo30151b	colonic catabolism of dietary phenolic and polyphenolic compounds from concord grape juice	In-vitro Model
3	10.1039/c4fo00478g	bi-compartmental elderly or adult dynamic digestion models applied to interrogate protein digestibility	In-vitro Digestion
2	10.1039/c4fo00615a	impact of dietary fibers methyl cellulose chitosan and pectin on digestion of lipids under simulated gastrointestinal conditions	Simulated Gastroin-testinal
12	10.1039/c4fo00946k	binding of bile acids by pastry products containing bioactive substances during in vitro digestion	In-vitro Digestion

2	10.1039/c4fo00982g	bioaccessibility of polyphenols associated with dietary fiber and in vitro kinetics release of polyphenols in mexican ataulfo mango mangifera indica l by-products	In-vitro Model
2	10.1039/c5fo00363f	in vitro fermentation of chewed mango and banana particle size starch and vascular fibre effects	In-vitro Gastroin-testinal
4	10.1039/c5fo00675a	in vitro fermentation of lupin seeds lupinus albus and broad beans vicia faba dynamic modulation of the intestinal microbiota and metabolomic output	In-vitro Digestion
4	10.1039/c5fo01438g	plant polyphenols alter a pathway of energy metabolism by inhibiting fecal bacteroidetes and firmicutes in vitro	In-vitro Gastroin-testinal
2	10.1039/c6fo00490c	the cotyledon cell wall and intracellular matrix are factors that limit iron bioavailability of the common bean phaseolus vulgaris	In-vitro Digestion
2	10.1039/c6fo00778c	synbiotic amazonian palm berry aai euterpe oleracea mart ice cream improved lactobacillus rhamnosus gg survival to simulated gastrointestinal stress	Simulated Gastroin-testinal
2	10.1039/c6fo00784h	dietary supplement based on stilbenes a focus on gut microbial metabolism by the in vitro simulator m-shime	Shime
4	10.1039/c6fo00924g	assessment of the prebiotic effect of quinoa and amaranth in the human intestinal ecosystem	In-vitro Digestion
2	10.1039/c6fo01737a	use of imaging techniques to identify efficient controlled release systems of lactobacillus rhamnosus gg during in vitro digestion	In-vitro Digestion
4	10.1039/c6ra20730h	fu brick tea extract supplementation enhanced probiotic viability and antioxidant activity of tofu under simulated gastrointestinal digestion condition	Simulated Gastroin-testinal
2	10.1039/c7fo00197e	in vitro modulation of gut microbiota by whey protein to preserve intestinal health	In-vitro Gastroin-testinal
6	10.1039/C7FO01348E	estimation of the bioaccessibility and bioavailability of fe mn cu and zn in chinese vegetables using the in vitro digestioncaco-2 cell model the influence of gut microbiota	In-vitro Digestion
2	10.1039/c7ra07429h	phytochemical content cellular antioxidant activity and antiproliferative activity of adinandra nitida tea shiyacha infusion subjected to in vitro gastrointestinal digestion	Simulated Gastroin-testinal
3	10.1039/c7tb02190a	original behavior of l rhamnosus gg encapsulated in freeze-dried alginate-silica microparticles revealed under simulated gastrointestinal conditions	Simulated Gastroin-testinal
3	10.1039/c8fo00390d	the effect of lactobacillus acidophilus and lactobacillus casei on the in vitro bioaccessibility of flaxseed lignans linum usitatissimum l	In-vitro Digestion
2	10.1039/c8fo00506k	milk fat protects bifidobacterium animalis subsp lactis bb-12 from in vitro gastrointestinal stress in potentially synbiotic table spreads	Simulated Gastroin-testinal
2	10.1039/c8fo01536h	potential prebiotic activity of tenebrio molitor insect flour using an optimized in vitro gut microbiota model	In-vitro Model
4	10.1039/c8fo01778f	an in vitro exploratory study of dietary strategies based on polyphenol-rich beverages fruit juices and oils to control trimethylamine production in the colon	In-vitro Model
2	10.1039/c8fo02495b	assessment of dynamic bioaccessibility of curcumin encapsulated in milled starch particle stabilized pickering emulsions using tnos gastrointestinal model	In-vitro Model

2	10.1039/c8fo02534g	impact of in vitro gastrointestinal digestion on the chemical composition bioactive properties and cytotoxicity of vitis vinifera l cv syrah grape pomace extract	In-vitro Gastrointestinal
2	10.1039/c8lc01080c	digestion-on-a-chip a continuous-flow modular microsystem recreating enzymatic digestion in the gastrointestinal tract	In-vitro Digestion
2	10.1039/C8RA10510C	a cascade of a denitrification bioreactor and an aerobic biofilm reactor for heavy oil refinery wastewater treatment	Reactor
2	10.1039/c9fo00370c	cereal bars functionalized through bifidobacterium animalis subsp lactis bb-12 and inulin incorporated in edible coatings of whey protein isolate or alginate	In-vitro Gastrointestinal
6	10.1039/c9fo00485h	physical effects of dietary fibre on simulated luminal flow studied by in vitro dynamic gastrointestinal digestion and fermentation	In-vitro Digestion
3	10.1039/c9fo00793h	bioaccessibility antioxidant activity and modulation effect on gut microbiota of bioactive compounds from moringa oleifera lam leaves during digestion and fermentation in vitro	In-vitro Gastrointestinal
2	10.1039/c9fo01091b	effect of the carrier material drying technology and dissolution media on the viability of lactobacillus fermentum k73 during simulated gastrointestinal transit	Simulated Gastrointestinal
2	10.1039/c9fo01118h	effect of microbial transglutaminase cross-linking on the quality characteristics and potential allergenicity of tofu	Simulated Gastrointestinal
2	10.1039/c9fo01965k	effects of different satiety levels on the fate of soymilk protein in gastrointestinal digestion and antigenicity assessed by an in vitro dynamic gastrointestinal model	In-vitro Digestion
2	10.1039/c9fo03009c	study on chemopreventive effects of raw and roasted -glucan-rich waxy winter barley using an in vitro human colon digestion model	In-vitro Digestion
2	10.1039/d0en01001d	the role of metal oxide nanoparticles escherichia coli and lactobacillus rhamnosus on small intestinal enzyme activity	In-vitro Model
3	10.1039/d0fo00367k	in vitro study for investigating the impact of decreasing the molecular weight of oat bran dietary fibre components on the behaviour in small and large intestine	In-vitro Colon
2	10.1039/d0fo00975j	release and metabolism of bound polyphenols from carrot dietary fiber and their potential activity in in vitro digestion and colonic fermentation	In-vitro Digestion
4	10.1039/d0fo01430c	effects of in vitro digestion and fecal fermentation on the stability and metabolic behavior of polysaccharides from craterellus cornucopioides	In-vitro Digestion
4	10.1039/d0fo01848a	prebiotic effects of pectooligosaccharides obtained from lemon peel on the microbiota from elderly donors using an in vitro continuous colon model tim-2	In-vitro Colon
4	10.1039/d0fo02347g	effects of in vitro digestion-fermentation over global antioxidant response and short chain fatty acid production of beet waste extracts in caii-alginate beads	In-vitro Digestion
6	10.1039/d0fo02399j	digestibility bioactivity and prebiotic potential of phenolics released from whole gold kiwifruit and pomace by in vitro gastrointestinal digestion and colonic fermentation	In-vitro Digestion
4	10.1039/d0fo02752a	modulation of gelatinized wheat starch digestion and fermentation profiles by young apple polyphenols in vitro	In-vitro Digestion
8	10.1039/d0fo03119d	bioaccessibility of tudela artichoke cynara scolymus cv blanca de tudela polyphenols the effects of heat treatment simulated gastrointestinal digestion and human colonic microbiota	Simulated Gastrointestinal
2	10.1039/d0fo03379k	valorisation of rejected unripe plantain fruits of musa aab simmonds from nutritional characterisation to the conceptual process design for prebiotic production	In-vitro Gastrointestinal

2	10.1039/d1fo01123e	in vitro protein and starch digestion kinetics of individual chickpea cells from static to more complex in vitro digestion approaches	In-vitro Digestion
2	10.1039/d1fo01155c	effect of roasting conditions on cocoa bioactivity and gut microbiota modulation	In-vitro Digestion
3	10.1039/d1fo01261d	potential prebiotic properties of exopolysaccharides produced by a novel lactobacillus strain lactobacillus pentosus yy-112	Simulated Gastroin-testinal
2	10.1046/j.1365-2672.1998.00522.x	growth of a human intestinal desulfovibrio desulfuricans in continuous cultures containing defined populations of saccharolytic and amino acid fermenting bacteria	Chemostat
3	10.1046/j.1365-2672.2001.01482.x	the regulatory effects of whey retentate from bifidobacteria fermented milk on the microbiota of the simulator of the human intestinal microbial ecosystem shime	In-vitro Gastroin-testinal
4	10.1046/j.1365-2672.2003.01959.x	a two-stage continuous culture system to study the effect of supplemental alpha-lactalbumin and glycomacropeptide on mixed cultures of human gut bacteria challenged with enteropathogenic escherichia coli and salmonella serotype typhimurium	Fermenter
2	10.1053/j.gastro.2020.11.034	fusobacteriumnucleatum adheres to clostridioides difficile via the radd adhesin to enhance biofilm formation in intestinal mucus	Bioreactor
2	10.1055/a-0755-7715	the activity of urolithin a and m4 valerolactone colonic microbiota metabolites of polyphenols in a prostate cancer in vitro model	In-vitro Model
2	10.1055/a-1300-9185	artificial intelligence and next generation sequencing new findings in myeloid neoplasms knstliche intelligenz und next generation sequencing	Artificial Gut
8	10.1055/a-1404-3344	in vitro evaluation of the anti-pathogenic activity of okoubaka aubrevillei on the human gastrointestinal tract	Shime
2	10.1061/(ASCE)EE.1943-7870.0001222	performance and bacterial community change during the start-up period of a novel anaerobic bioreactor inoculated with long-time storage anaerobic granular sludge	Reactor
2	10.1061/(ASCE)EE.1943-7870.0001617	viral surrogates in potable reuse applications evaluation of a membrane bioreactor and full advanced treatment	Bioreactor
2	10.1073/pnas.1319470110	gnotobiotic mouse model of phage-bacterial host dynamics in the human gut	Artificial Gut
3	10.1079/bjn2003949	metabolism of the soybean isoflavone glycoside genistin in vitro by human gut bacteria and the effect of prebiotics	Gut Model
2	10.1079/BJN20041101	effects of esculin and esculetin on the survival of escherichia coli o157 in human faecal slurries continuous-flow simulations of the rumen and colon and in calves	Fermenter
3	10.1080/07315724.2009.10719798	the effect of the undigested fraction of maize products on the activity and composition of the microbiota determined in a dynamic in vitro model of the human proximal large intestine	In-vitro Model
2	10.1080/08827508.2019.1575213	key microbes and metabolic potentials contributing to cyanide biodegradation in stirred-tank bioreactors treating gold mining effluent	Reactor
2	10.1080/08905436.2010.524459	isoflavone conversion of black soybean by immobilized rhizopus spp	Reactor
2	10.1080/089106001300136183	development of a six-stage culture system for simulating the gastrointestinal microbiota of weaned infants	In-vitro Model
2	10.1080/09637486.2016.1186156	analysis of a whole diet in terms of phenolic content and antioxidant capacity effects of a simulated gastrointestinal digestion	Simulated Gastroin-testinal
3	10.1080/09637486.2017.1309522	prebiotic evaluation of red seaweed kappaphycus alvarezii using in vitro colon model	In-vitro Colon
2	10.1080/09637486.2017.1330404	dephosphorylation of myo-inositol phosphates in the in vitro intestinal caco-2 cell model	In-vitro Model

2	10.1080/09637486.2017.1416458	in vitro human colonic fermentation of indigestible fraction isolated from lunch menus impact on the gut metabolites and antioxidant capacity	In-vitro Gastroin-testinal
4	10.1080/09637486.2018.1498065	impact of combining acerola by-product with a probiotic strain on a gut microbiome model	Simulated Gastroin-testinal
2	10.1080/09637486.2018.1513996	bioactivity and cell metabolism of in vitro digested sweet cherry prunus avium phenolic compounds	In-vitro Digestion
3	10.1080/09637486.2020.1772205	chemopreventive effects of raw and roasted oat flakes after invitro fermentation with human faecal microbiota	In-vitro Digestion
2	10.1080/09637486.2021.1908964	metabolic profiles of oligosaccharides derived from four microbial polysaccharides by faecal inocula from type 2 diabetes patients	In-vitro Digestion
2	10.1080/1040841X.2021.1876631	inflammatory bowel disease tri-directional relationship between microbiota immune system and intestinal epithelium	In-vitro Gastroin-testinal
2	10.1080/10717544.2021.1949074	study on mechanism of low bioavailability of black tea theaflavins by using caco-2 cell monolayer	In-vitro Model
3	10.1080/10731190701460226	impact of orally administered microcapsules on gastrointestinal microbial flora in-vitro investigation using computer controlled dynamic human gastrointestinal model	Gastrointestinal Model
2	10.1080/10826068.2016.1207082	a novel process for the production of high-purity galactooligosaccharides gos using consortium of microbes	Reactor
2	10.1080/10934520701434919	comparison of five in vitro digestion models to in vivo experimental results lead bioaccessibility in the human gastrointestinal tract	In-vitro Digestion
4	10.1080/10934521003648958	the effect of residence time and fluid volume to soil mass ls ratio on in vitro arsenic bioaccessibility from poorly crystalline scorodite	Shime
2	10.1080/17460441.2021.1960502	what is the gold standard model for alzheimers disease drug discovery and development	In-vitro Model
3	10.1080/19390211.2020.1814931	impact of a gastrointestinal stable probiotic supplement bacillus coagulans lbsc on human gut microbiome modulation	Gut Model
4	10.1080/19490976.2019.1702431	following the community development of sihumix - a new intestinal in vitro model for bioreactor use	In-vitro Model
2	10.1088/1748-3190/12/1/016001	a bioreactor with an electro-responsive elastomeric membrane for mimicking intestinal peristalsis	Artificial Gut
2	10.1089/ees.2010.0227	fate of sucralose during wastewater treatment	Reactor
4	10.1089/jmf.2012.0292	impact of dietary fiber fermentation from cereal grains on metabolite production by the fecal microbiota from normal weight and obese individuals	In-vitro Digestion
16	10.1089/jmf.2013.0092	prebiotic effect of fructooligosaccharide in the simulator of the human intestinal microbial ecosystem shime model	Reactor
2	10.1093/bioinformatics/btw107	an algorithm for designing minimal microbial communities with desired metabolic capacities	Bioreactor
15	10.1093/FEMSEC/FIAA105	a prebiotic-enhanced lipid-based nutrient supplement lnsp increases bifidobacterium relative abundance and enhances short-chain fatty acid production in simulated colonic microbiota from undernourished infants	Simulated Colon
4	10.1093/femsec/fiab111	nutrient load acts as a driver of gut microbiota load community composition and metabolic functionality in the simulator of the human intestinal microbial ecosystem	Simulator of Human
7	10.1093/femsec/fiv054	interindividual differences in response to treatment with butyrate-producing butyricicoccus pullicaecorum 25-3t studied in an in vitro gut model	Gut Model

3	10.1093/femsec/fiw233	in vitro fermentation of b-gos impact on faecal bacterial populations and metabolic activity in autistic and non-autistic children	Gut Model
2	10.1093/femsec/fix088	effect of dietary nucleosides and yeast extracts on composition and metabolic activity of infant gut microbiota in polyferms colonic fermentation models	Continous Fermentation
2	10.1093/femsec/fiy140	simulation and modeling of dietary changes in the infant gut microbiome	Continous Culture
8	10.1093/femsec/fiy186	mucin degradation niche as a driver of microbiome composition and akkermansia muciniphila abundance in a dynamic gut model is donor independent	Simulated Colon
4	10.1093/femsec/fiz096	microbial communities in a dynamic in vitro model for the human ileum resemble the human ileal microbiota	In-vitro Model
10	10.1093/femsle/fnaa135	a toddler shime model to study microbiota of young children	In-vitro Gastrointestinal
2	10.1093/femsle/fnab042	acidic ph enhances butyrate production from pectin by faecal microbiota	Continous Culture
5	10.1093/JAC/DKAA386	eravacycline a novel tetracycline derivative does not induce clostridioides difficile infection in an in vitro human gut model	Gut Model
3	10.1093/jac/dkg267	effects of cefotaxime and desacetylcefotaxime upon clostridium difficile proliferation and toxin production in a triple-stage chemostat model of the human gut	Gut Model
3	10.1093/jac/dkl364	tigecycline does not induce proliferation or cytotoxin production by epidemic clostridium difficile strains in a human gut model	Gut Model
2	10.1093/jac/dkq345	rifaximin modulates the colonic microbiota of patients with crohns disease an in vitro approach using a continuous culture colonic model system	Continous Culture
2	10.1093/jac/dks529	mixed infection by clostridium difficile in an in vitro model of the human gut	In-vitro Model
3	10.1093/jac/dkt107	evaluation of antimicrobial activity of ceftaroline against clostridium difficile and propensity to induce c difficile infection in an in vitro human gut model	Gut Model
2	10.1093/jac/dkt411	in vitro activity of cadazolid against clinically relevant clostridium difficile isolates and in an in vitro gut model of c difficile infection	Gut Model
4	10.1093/jac/dku116	comparison of planktonic and biofilm-associated communities of clostridium difficile and indigenous gut microbiota in a triple-stage chemostat gut model	Gut Model
3	10.1093/jac/dkv108	recurrence of dual-strain clostridium difficile infection in an in vitro human gut model	Gut Model
3	10.1093/jac/dkv156	efficacy of alternative fidaxomicin dosing regimens for treatment of simulated clostridium difficile infection in an in vitro human gut model	In-vitro Model
3	10.1093/jac/dkv453	efficacy of vancomycin extended-dosing regimens for treatment of simulated clostridium difficile infection within an in vitro human gut model	Gut Model
5	10.1093/jas/skz357	development and validation of the simulator of the canine intestinal microbial ecosystem scime1	In-vitro Model
8	10.1093/jn/130.10.2599	fermentation by gut microbiota cultured in a simulator of the human intestinal microbial ecosystem is improved by supplementing a soygerm powder	In-vitro Model
12	10.1093/jn/136.4.946	administration of equol-producing bacteria alters the equol production status in the simulator of the gastrointestinal microbial ecosystem shime	Shime
2	10.1093/jn/136.7.1862	the prenylflavonoid isoxanthohumol from hops humulus lupulus l is activated into the potent phytoestrogen 8-prenylnaringenin in vitro and in the human intestine	Shime

2	10.1093/mmy/myx156	antagonistic effect of saccharomyces cerevisiae ktp and issatchenkia occidentalis apc on hyphal development and adhesion of candida albicans	Simulated Gastroin-testinal
2	10.1094/CCHEM-05-13-0095-FI	importance of location of digestion and colonic fermentation of starch related to its quality	In-vitro Digestion
2	10.1094/CCHEM-07-10-0098	in vitro digestion of rs4-type resistant wheat and potato starches and fermentation of indigestible fractions	In-vitro Digestion
2	10.1095/biolreprod.112.103366	microbial products alter the expression of membrane-associated mucin and antimicrobial peptides in a three-dimensional human endocervical epithelial cell model	In-vitro Model
2	10.1096/fj.201801414R	development of a host-microbiome model of the small intestine	In-vitro Model
6	10.1097/00008469-199202000-00004	faecal steroids and colorectal cancer the effect of lactulose on faecal bacterial metabolism in a continuous culture model of the large intestine	Chemostat
2	10.1097/00024382-199607000-00007	intracellular survival of enteric bacteria in cultured human enterocytes	In-vitro Model
4	10.1097/01.mat.0000191345.45735.00	in vitro and in vivo assessment of intraintestinal bacteriotherapy in chronic kidney disease	Shime
2	10.1097/MCG.0000000000000711	an advanced in vitro technology platform to study the mechanism of action of prebiotics and probiotics in the gastrointestinal tract	Simulator of Human
2	10.1099/0022-1317-50-9-833	modulation of genotoxic enzyme activities by non-digestible oligosaccharide metabolism in in-vitro human gut bacterial ecosystems	Continous Culture
2	10.1099/00222615-47-5-407	ecological and physiological studies on large intestinal bacteria in relation to production of hydrolytic and reductive enzymes involved in formation of genotoxic metabolites	Continous Culture
4	10.1099/mic.0.042036-0	unexpected consequences of administering bacteriocinogenic probiotic strains for salmonella populations revealed by an in vitro colonic model of the child gut	Reactor
2	10.1099/mic.0.042044-0	evaluating the microbial diversity of an in vitro model of the human large intestine by phylogenetic microarray analysis	In-vitro Model
2	10.1108/NFS-08-2015-0093	antioxidant potential of phenolic-rich two varieties of nigerian local rice and their anti-cholinesterase activities after in vitro digestion	Simulated Gastroin-testinal
2	10.1109/TBME.2006.883698	biocompatibility of a lab-on-a-pill sensor in artificial gastrointestinal environments	In-vitro Gastroin-testinal
2	10.1111/1462-2920.12399	antibiotic treatments and microbes in the gut	In-vitro Model
2	10.1111/1462-2920.12552	rapid succession of uncultured marine bacterial and archaeal populations in a denitrifying continuous culture	Chemostat
2	10.1111/1462-2920.12824	disturbance opens recruitment sites for bacterial colonization in activated sludge	Bioreactor
12	10.1111/1462-2920.13622	mucosa-associated biohydrogenating microbes protect the simulated colon microbiome from stress associated with high concentrations of poly-unsaturated fat	Shime
11	10.1111/1462-2920.14381	introducing insoluble wheat bran as a gut microbiota niche in an in vitro dynamic gut model stimulates propionate and butyrate production and induces colon region specific shifts in the luminal and mucosal microbial community	In-vitro Model
2	10.1111/1462-2920.14437	neutral mechanisms and niche differentiation in steady-state insular microbial communities revealed by single cell analysis	Reactor
3	10.1111/1462-2920.14705	bifidobacterium bifidum and the infant gut microbiota an intriguing case of microbe-host co-evolution	Bioreactor

2	10.1111/1471-0307.12215	effect of different matrices on probiotic resistance to invitro simulated gastrointestinal conditions	Simulated Gastrointestinal
2	10.1111/1471-0307.12630	microbial physico-chemical and sensory characteristics of mango juice-enriched probiotic dairy drinks	In-vitro Gastrointestinal
2	10.1111/1541-4337.12410	dietary exposures to common emulsifiers and their impact on the gut microbiota is there a cause for concern	In-vitro Model
2	10.1111/1541-4337.12473	technological regulatory and ethical aspects of in vitro meat a future slaughter-free harvest	Artificial Gut
2	10.1111/1574-6941.12041	effect of galactooligosaccharides and bifidobacterium animalis bb-12 on growth of lactobacillus amylovorus dsm 16698 microbial community structure and metabolite production in an in vitro colonic model set up with human or pig microbiota	In-vitro Model
2	10.1111/1574-6941.12435	integrated omics analysis for studying the microbial community response to a ph perturbation of a cellulose-degrading bioreactor culture	Reactor
3	10.1111/1574-6968.12515	bifidobacterium animalis ssp lactis bi07 modulates the tumor necrosis factor alpha-dependent imbalances of the enterocyte-associated intestinal microbiota fraction	In-vitro Model
2	10.1111/1750-3841.12326	viability and resistance of lactobacilli isolated from cocoa fermentation to simulated gastrointestinal digestive steps in soy yogurt	Simulated Gastrointestinal
2	10.1111/1750-3841.13725	in vitro digestion and fermentation of microencapsulated tributyrin for the delivery of butyrate	In-vitro Digestion
2	10.1111/1750-3841.13820	effect of type of protein-based microcapsules and storage at various ambient temperatures on the survival and heat tolerance of spray dried lactobacillus acidophilus	Simulated Gastrointestinal
4	10.1111/1750-3841.14086	whole tibetan hull-less barley exhibit stronger effect on promoting growth of genus bifidobacterium than refined barley in vitro	In-vitro Digestion
6	10.1111/1750-3841.14377	bioaccessibility of some essential minerals in three selected australian pulse varieties using an in vitro gastrointestinal digestion model	In-vitro Digestion
2	10.1111/1750-3841.15013	optimal conditions for the encapsulation of weissella cibaria jw15 using alginate and chicory root and evaluation of capsule stability in a simulated gastrointestinal system	Simulated Gastrointestinal
2	10.1111/1750-3841.15399	physicochemical properties of soy protein hydrolysate and its formulation and stability with encapsulated probiotic underinvitrogastrointestinal environment	In-vitro Gastrointestinal
2	10.1111/1750-3841.15695	impact of encapsulating probiotics with cocoa powder on the viability of probiotics during chocolate processing storage and in vitro gastrointestinal digestion	In-vitro Gastrointestinal
4	10.1111/1750-3841.15852	effects of digested flours from four different sweet potato ipomoea batatas l root varieties on the composition and metabolic activity of human colonic microbiota in vitro	Simulated Gastrointestinal
6	10.1111/1758-2229.12056	arabinogalactan and fructo-oligosaccharides have a different fermentation profile in the simulator of the human intestinal microbial ecosystem shime	Shime
2	10.1111/asj.13047	selection and characterization of broad-spectrum antibacterial substance-producing lactobacillus curvatus pa40 as a potential probiotic for feed additives	Gastrointestinal Model

2	10.1111/ijfs.13536	stability of antioxidant peptides from duck meat after post-mortem ageing	Simulated Gastroin-testinal
2	10.1111/ijfs.13697	evaluation of the prebiotic potential of five kiwifruit cultivars after simulated gastrointestinal digestion and fermentation with human faecal bacteria	Simulated Gastroin-testinal
2	10.1111/ijfs.14142	characteristic of polysaccharides from flammulina velutipes invitro digestion under salivary simulated gastric and small intestinal conditions and fermentation by human gut microbiota	In-vitro Digestion
2	10.1111/ijfs.14224	in vitro gastrointestinal digestion of mango by-product snacks potential absorption of polyphenols and antioxidant capacity	In-vitro Gastroin-testinal
2	10.1111/ijfs.14337	effects of simulated gastric and intestinal digestion on chitooligosaccharides in two in vitro models	In-vitro Digestion
2	10.1111/ijfs.15083	metabolic and microbial modulation of phenolic compounds from raspberry leaf extract under in vitro digestion and fermentation	In-vitro Digestion
2	10.1111/ijfs.15278	digestion of pectic polysaccharide from brassica rapa l invitro and its effect on the intestinal microbiota in cyclophosphamide-treated mice	Simulated Gastroin-testinal
2	10.1111/j.1365-2621.2001.tb08220.x	continuous production of pectic oligosaccharides in an enzyme membrane reactor	Reactor
2	10.1111/j.1365-2621.2002.tb08720.x	continuous production of oligodextrans via controlled hydrolysis of dextran in an enzyme membrane reactor	Reactor
2	10.1111/j.1365-2672.1996.tb04331.x	enumeration of human colonic bacteria producing phenolic and indolic compounds effects of ph carbohydrate availability and retention time on dissimilatory aromatic amino acid metabolism	Continous Culture
2	10.1111/j.1365-2672.2004.02182.x	microbiological monitoring in the biodegradation of sewage sludge and food waste	Reactor
2	10.1111/j.1365-2672.2006.03084.x	inulin-type fructans of longer degree of polymerization exert more pronounced in vitro prebiotic effects	Shime
2	10.1111/j.1365-2672.2007.03342.x	induction of acid resistance in bifidobacterium a mechanism for improving desirable traits of potentially probiotic strains	Simulated Gastroin-testinal
6	10.1111/j.1365-2672.2008.04015.x	chemopreventive effects from prebiotic inulin towards microbial 2-amino-1-methyl-6-phenylimidazo45-bpyridine bioactivation	Shime
3	10.1111/j.1462-2920.2008.01815.x	linking phylogenetic identities of bacteria to starch fermentation in an in vitro model of the large intestine by rna-based stable isotope probing	In-vitro Model
2	10.1111/j.1462-5822.2011.01706.x	enteroaggregative escherichia coli promotes transepithelial migration of neutrophils through a conserved 12-lipoxygenase pathway	In-vitro Model
4	10.1111/j.1472-765X.2009.02698.x	panose a new prebiotic candidate	Simulated Colon
2	10.1111/j.1574-6941.2005.00016.x	studies on the effect of system retention time on bacterial populations colonizing a three-stage continuous culture model of the human large gut using fish techniques	Continous Culture
10	10.1111/j.1574-6941.2006.00117.x	new three-stage in vitro model for infant colonic fermentation with immobilized fecal microbiota	In-vitro Model
2	10.1111/j.1574-6941.2006.00118.x	comparative effects of exopolysaccharides from lactic acid bacteria and fructo-oligosaccharides on infant gut microbiota tested in an in vitro colonic model with immobilized cells	Chemostat
2	10.1111/j.1574-6941.2007.00291.x	molecular assessment of complex microbial communities degrading long chain fatty acids in methanogenic bioreactors	Reactor

4	10.1111/j.1574-6941.2007.00327.x	lactobacillus gasseri gasser am63t degrades oxalate in a multistage continuous culture simulator of the human colonic microbiota	Continous Culture
6	10.1111/j.1574-6941.2007.00412.x	glycerol induces reuterin production and decreases escherichia coli population in an in vitro model of colonic fermentation with immobilized human feces	In-vitro Model
4	10.1111/j.1574-6941.2008.00625.x	new in vitro colonic fermentation model for salmonella infection in the child gut	In-vitro Model
2	10.1111/j.1574-6941.2011.01069.x	fate and efficacy of lacticin 3147-producing lactococcus lactis in the mammalian gastrointestinal tract	Simulated Gastrointestinal
7	10.1111/j.1574-6941.2011.01252.x	decreased colonization of fecal clostridium coccoideseubacterium rectale species from ulcerative colitis patients in an in vitro dynamic gut model with mucin environment	Gut Model
5	10.1111/j.1574-6941.2011.01279.x	effect of a synbiotic on microbial community structure in a continuous culture model of the gastric microbiota in enteral nutrition patients	Fermenter
8	10.1111/j.1574-6941.2012.01330.x	the composition and metabolic activity of child gut microbiota demonstrate differential adaptation to varied nutrient loads in an in vitro model of colonic fermentation	In-vitro Model
8	10.1111/j.1574-6968.1994.tb06813.x	enrichment of bifidobacteria from human gut contents by oligofructose using continuous culture	Chemostat
4	10.1111/j.1574-6968.2000.tb08867.x	chromosomal integration of the green fluorescent protein gene in lactic acid bacteria and the survival of marked strains in human gut simulations	Fermenter
2	10.1111/j.1600-0463.2012.02902.x	characterization of rhodopseudomonas palustris strain 2c as a potential probiotic	Simulated Gastrointestinal
2	10.1111/j.1740-0929.2006.00372.x	stimulation of butyrate production through the metabolic interaction among lactic acid bacteria lactobacillus acidophilus and lactic acid-utilizing bacteria megasphaera elsdenii in porcine cecal digesta	In-vitro Model
2	10.1111/j.1745-4514.2009.00222.x	optimization of enzymatic synthesis of isomalto-oligosaccharides production	Reactor
2	10.1111/j.1750-3841.2011.02602.x	effect of mannoproteins on the growth gastrointestinal viability and adherence to caco-2 cells of lactic acid bacteria	Simulated Gastrointestinal
3	10.1111/j.1751-7915.2008.00064.x	arabinoxylan-oligosaccharides axos affect the proteincarbohydrate fermentation balance and microbial population dynamics of the simulator of human intestinal microbial ecosystem	Reactor
2	10.1111/j.1751-7915.2011.00308.x	incorporating a mucosal environment in a dynamic gut model results in a more representative colonization by lactobacilli	Shime
2	10.1111/jam.15329	in vitro and in vivo activity of new strains of bacillus subtilis against esbl-producing escherichia coli an experimental study	In-vitro Model
3	10.1111/jfbc.12903	effect of pereskia aculeata mill in vitro and in overweight humans a randomized controlled trial	In-vitro Gastrointestinal
2	10.1111/jfpp.12704	the monitoring via an in vitro digestion system of the bioactive content of vegetable juice fermented with saccharomyces cerevisiae and saccharomyces boulardii	In-vitro Digestion
2	10.1111/jfpp.14922	antioxidant activity of pickled sauced meat before and after cooking and in vitro gastrointestinal digestion	Simulated Gastrointestinal
2	10.1111/jphp.12978	in vitro gastrointestinal biotransformation and characterization of a desmodium adscendens decoction the first step in unravelling its behaviour in the human body	Simulated Gastrointestinal

3	10.1111/lam.13080	the survival of irradiated lactobacilli in the simulated gastrointestinal conditions with antibiotic ceftazidime	Simulated Gastroin-testinal
2	10.1111/mec.15033	antibiotic disturbance affects aquatic microbial community composition and food web interactions but not community resilience	Continous Culture
2	10.1111/mec.15747	bacterial dispersal and drift drive microbiome diversity patterns within a population of feral hindgut fermenters	Fermenter
3	10.1128/AAC.00306-08	effects of exposure of clostridium difficile pcr ribotypes 027 and 001 to fluoroquinolones in a human gut model	Gut Model
3	10.1128/AAC.01581-18	omadacycline gut microbiome exposure does not induce clostridium difficile proliferation or toxin production in a model that simulates the proximal medial and distal human colon	Gut Model
2	10.1128/AEM.00327-12	higher-level production of volatile fatty acids in vitro by chicken gut microbiotas than by human gut microbiotas as determined by functional analyses	Chemostat
2	10.1128/AEM.00376-17	selected probiotic lactobacilli have the capacity to hydrolyze gluten peptides during simulated gastrointestinal digestion	Simulated Gastroin-testinal
21	10.1128/AEM.00759-10	microbial community development in a dynamic gut model is reproducible colon region specific and selective for bacteroidetes and clostridium cluster ix	In-vitro Colon
2	10.1128/AEM.01073-19	an in vitro enrichment strategy for formulating synergistic synbiotics	Fermenter
2	10.1128/AEM.01705-20	processing has differential effects on microbiota-accessible carbohydrates in whole grains during in vitro fermentation	In-vitro Digestion
4	10.1128/AEM.02244-10	adherence and cytokine induction in caco-2 cells by bacterial populations from a three-stage continuous-culture model of the large intestine	Continous Culture
4	10.1128/AEM.02647-20	mucin as a functional niche is a more important driver of in vitro gut microbiota composition and functionality than akkermansia muciniphila supplementation	In-vitro Model
3	10.1128/AEM.02730-19	prebiotics inhibit proteolysis by gut bacteria in a host diet-dependent manner a three-stage continuous in vitro gut model experiment	Gut Model
3	10.1128/AEM.02783-18	bifidobacterium bifidum atcc 15696 and bifidobacterium breve 24b metabolic interaction based on 2-o-fucosyl-lactose studied in steady-state cultures in a freter-style chemostat	Chemostat
8	10.1128/AEM.02893-17	complementary mechanisms for degradation of inulin-type fructans and arabinoxylan oligosaccharides among bifidobacterial strains suggest bacterial cooperation	Shime
2	10.1128/AEM.03001-13	impact of substratum surface on microbial community structure and treatment performance in biological aerated filters	Reactor
6	10.1128/AEM.03303-12	enterohemorrhagic escherichia coli o157 h7 survival in an in vitro model of the human large intestine and interactions with probiotic yeasts and resident microbiota	In-vitro Model
2	10.1128/AEM.04050-14	akkermansia muciniphila adheres to enterocytes and strengthens the integrity of the epithelial cell layer	In-vitro Model
3	10.1128/AEM.06385-11	potential probiotic kluyveromyces marxianus b0399 modulates the immune response in caco-2 cells and peripheral blood mononuclear cells and impacts the human gut microbiota in an in vitro colonic model system	Continous Culture
2	10.1128/AEM.06394-11	unexpected stability of bacteroidetes and firmicutes communities in laboratory biogas reactors fed with different defined substrates	Reactor
2	10.1128/AEM.67.7.2895-2902.2001	antacid increases survival of vibrio vulnificus and vibrio vulnificus phage in a gastrointestinal model	Simulated Gastroin-testinal
2	10.1128/AEM.69.4.1920-1927.2003	nondigestible oligosaccharides enhance bacterial colonization resistance against clostridium difficile in vitro	Chemostat

2	10.1128/AEM.70.8.4505-4511.2004	polydextrose lactitol and fructo-oligosaccharide fermentation by colonic bacteria in a three-stage continuous culture system	Continous Culture
2	10.1128/AEM.72.4.3042-3045.2006	in vitro and in vivo survival and transit tolerance of potentially probiotic strains carried by artichokes in the gastrointestinal tract	Simulated Gastrointestinal
2	10.1128/JB.00985-13	type 1 fimbriae contribute to catheter-associated urinary tract infections caused by escherichia coli	In-vitro Model
3	10.1128/mSphere.00564-17	ecological stability properties of microbial communities assessed by flow cytometry	Reactor
2	10.1128/mSystems.00185-19	species deletions from microbiome consortia reveal key metabolic interactions between gut microbes	Bioreactor
2	10.1128/mSystems.00404-19	effects of antibiotic pretreatment of an ulcerative colitis-derived fecal microbial community on the integration of therapeutic bacteria in vitro	Bioreactor
2	10.1136/gut.2003.037580	prebiotic carbohydrates modify the mucosa associated microflora of the human large bowel	Chemostat
2	10.1136/gutjnl-2016-313099	dietary emulsifiers directly alter human microbiota composition and gene expression ex vivo potentiating intestinal inflammation	Shime
2	10.1137/S0036139901390416	bacterial wall attachment in a flow reactor	Reactor
2	10.1139/cjm-2015-0446	characterization of functional properties of enterococcus faecium strains isolated from human gut	In-vitro Digestion
2	10.1155/2011/587470	higher molecular weight polyethylene glycol increases cell proliferation while improving barrier function in an in vitro colon cancer model	In-vitro Colon
12	10.1155/2014/365738	matrix effects on the stability and antioxidant activity of red cabbage anthocyanins under simulated gastrointestinal digestion	Simulated Gastrointestinal
2	10.1155/2014/602832	microencapsulated bifidobacterium longum subsp infantis atcc 15697 favorably modulates gut microbiota and reduces circulating endotoxins in f344 rats	Bioreactor
3	10.1155/2017/5176384	survival of five strains of shiga toxigenic escherichia coli in a sausage fermentation model and subsequent sensitivity to stress from gastric acid and intestinal fluid	In-vitro Digestion
2	10.1158/0008-5472.CAN-20-2983	diet alters entero-mammary signaling to regulate the breast microbiome and tumorigenesis	In-vitro Model
2	10.1177/00220345910700070401	synergistic degradation of mucin by streptococcus oralis and streptococcus sanguis in mixed chemostat cultures	Chemostat
2	10.1177/0148607105029005388	do ph and temperature play a role in gastrostomy tube deterioration	In-vitro Model
4	10.1186/1471-2180-12-247	biogenic amine production by the wine lactobacillus brevis ioeb 9809 in systems that partially mimic the gastrointestinal tract stress	Simulated Gastrointestinal
2	10.1186/1471-2180-12-47	effects of probiotics and antibiotics on the intestinal homeostasis in a computer controlled model of the large intestine	In-vitro Model
4	10.1186/1471-230X-13-100	lactobacillus acidophilus crl 1014 improved gut health in the shime reactor	Reactor
2	10.1186/s12866-018-1210-z	lactic acid bacteria isolated from european badgers meles meles reduce the viability and survival of bacillus calmette-guerin bcg vaccine and influence the immune response to bcg in a human macrophage model	In-vitro Model
3	10.1186/s12866-019-1669-2	method comparison for the direct enumeration of bacterial species using a chemostat model of the human colon	Gut Model
2	10.1186/s12866-021-02220-3	human microbiota modulation via qsec sensor kinase mediated in the escherichia coli o104h4 outbreak strain infection in microbiome model	Shime

6	10.1186/s12876-021-01687-8	onset of acid-neutralizing action of a calciummagnesium carbonate-based antacid using an artificial stomach model an in vitro evaluation	Reactor
16	10.1186/s12915-020-00860-x	spatial and temporal modulation of enterotoxigenic e coli h10407 pathogenesis and interplay with microbiota in human gut models	In-vitro Model
2	10.1186/s13068-016-0634-7	piezo-tolerant natural gas-producing microbes under accumulating pco2	Reactor
2	10.1186/s13068-018-1193-x	increasing the economic value of lignocellulosic stillage through medium-chain fatty acid production	Reactor
6	10.1186/s13099-014-0044-y	synergistic effects of bifidobacterium thermophilum rbl67 and selected prebiotics on inhibition of salmonella colonization in the swine proximal colon polyferms model	In-vitro Model
2	10.1186/s13099-016-0144-y	clostridium difficile colonization and antibiotics response in polyferms continuous model mimicking elderly intestinal fermentation	Reactor
2	10.1186/s13104-015-1407-2	effects of freezing storage on the dna extraction and microbial evaluation from anaerobic digested sludges	Reactor
2	10.1186/s13104-020-05338-1	enhancement of intestinal epithelial barrier function by weissella confusa f213 and lactobacillus rhamnosus fbb81 probiotic candidates in an in vitro model of hydrogen peroxide-induced inflammatory bowel disease	In-vitro Model
2	10.1186/s13568-019-0911-5	bifidogenic and butyrogenic effects of young barely leaf extract in an in vitro human colonic microbiota model	In-vitro Model
2	10.1186/s40104-021-00584-0	weaning-associated feed deprivation stress causes microbiota disruptions in a novel mucin-containing in vitro model of the piglet colon mpigut-ivm	In-vitro Model
2	10.1186/s40168-015-0106-5	cultivation of stable reproducible microbial communities from different fecal donors using minibioreactor arrays mbras	Reactor
4	10.1186/s40168-018-0584-3	dynamic linear models guide design and analysis of microbiota studies within artificial human guts	Artificial Gut
2	10.1186/s40168-019-0682-x	coupling growth kinetics modeling with machine learning reveals microbial immigration impacts and identifies key environmental parameters in a biological wastewater treatment process	Reactor
2	10.1186/s40168-020-00940-8	perinatal environment shapes microbiota colonization and infant growth impact on host response and intestinal function	In-vitro Model
4	10.1186/s40168-021-01036-7	isolation and characterisation of crass002 a crass-like phage from the human gut that infects bacteroides xylanisolvens	Chemostat
2	10.11949/j.issn.0438-1157.20181488	parameter estimation and cold model experiments of gastrointestinal simulation reactor	In-vitro Digestion
2	10.11975/j.issn.1002-6819.2019.02.036	ultra-high pressure treatment improving physicochemical properties and probiotic community of tartary buckwheat starch	In-vitro Model
6	10.1205/fbp.04396	an engineering model of the human colon	Gut Model
2	10.1264/jsme2.ME21045	ecogenomics reveals microbial metabolic networks in a psychrophilic methanogenic bioreactor treating soy sauce production wastewater	Reactor
2	10.12688/f1000research.17870.2	a study protocol for a randomised crossover study evaluating the effect of diets differing in carbohydrate quality on ileal content and appetite regulation in healthy humans	Continous Fermentation
2	10.12688/wellcomeopenres.15628.3	improving causality in microbiome research can human genetic epidemiology help	In-vitro Model
2	10.1271/bbb.60022	effects of lactose on colon microbial community structure and function in a four-stage semi-continuous culture system	Continous Culture
2	10.1289/ehp.0901794	arsenic metabolism by human gut microbiota upon in vitro digestion of contaminated soils	In-vitro Digestion

4	10.13227/j.hjkx.2016.06.045	effects of human gut microbiota on bioaccessibility of soil cd cr and ni using shime model	Shime
2	10.13671/j.hjkxxb.2019.0041	study on the characteristics of microbial community in anaerobic fluidized bed membrane bioreactor for domestic wastewater treatment afmbr	Reactor
5	10.1371/journal.pone.0023227	the influence of staphylococcus aureus on gut microbial ecology in an in vitro continuous culture human colonic model system	In-vitro Model
4	10.1371/journal.pone.0049740	persistence of anticancer activity in berry extracts after simulated gastrointestinal digestion and colonic fermentation	Simulated Gastroin-testinal
2	10.1371/journal.pone.0072620	fate of ingested clostridium difficile spores in mice	Simulated Gastroin-testinal
5	10.1371/journal.pone.0077128	in vitro fermentation of nutriose fb06 a wheat dextrin soluble fibre in a continuous culture human colonic model system	Gut Model
4	10.1371/journal.pone.0077772	novel polyfermentor intestinal model polyferms for controlled ecological studies validation and effect of ph	Reactor
3	10.1371/journal.pone.0088396	development and validation of a chemostat gut model to study both planktonic and biofilm modes of growth of clostridium difficile and human microbiota	Gut Model
2	10.1371/journal.pone.0113864	in vitro characterization of the impact of different substrates on metabolite production energy extraction and composition of gut microbiota from lean and obese subjects	In-vitro Model
2	10.1371/journal.pone.0123498	the intestinal barrier in irritable bowel syndrome subtype-specific effects of the systemic compartment in an in vitro model	In-vitro Model
2	10.1371/journal.pone.0133427	impact of bioreactor environment and recovery method on the profile of bacterial populations from water distribution systems	Reactor
4	10.1371/journal.pone.0142793	design and investigation of polyferms in vitro continuous fermentation models inoculated with immobilized fecal microbiota mimicking the elderly colon	Reactor
2	10.1371/journal.pone.0145309	a spatially continuous model of carbohydrate digestion and transport processes in the colon	Reactor
2	10.1371/journal.pone.0157958	effects of the food manufacturing chain on the viability and functionality of bifidobacterium animalis through simulated gastrointestinal conditions	Simulated Gastroin-testinal
2	10.1371/journal.pone.0159159	new insights into the enterococcus faecium and streptococcus gallolyticus subsp gallolyticus host interaction mechanisms	In-vitro Model
2	10.1371/journal.pone.0162604	an in vitro approach to study effects of prebiotics and probiotics on the faecal microbiota and selected immune parameters relevant to the elderly	Fermenter
2	10.1371/journal.pone.0167785	acute effects of sugars and artificial sweeteners on small intestinal sugar transport a study using caco-2 cells as an in vitro model of the human enterocyte	In-vitro Model
2	10.1371/journal.pone.0197692	establishing a mucosal gut microbial community in vitro using an artificial simulator	Simulator of Human
3	10.1371/journal.pone.0208906	use of lactobacillus crispatus to produce a probiotic cheese as potential gender food for preventing gynaecological infections	Reactor
2	10.1371/journal.pone.0256548	apparent diet digestibility of captive colobines in relation to stomach types with special reference to fibre digestion	Fermenter
2	10.13982/j.mfst.1673-9078.2016.6.048	analysis of probiotic properties and safety of lactobacillus reuteri	Simulated Gastroin-testinal
2	10.13982/j.mfst.1673-9078.2017.1.007	screening of intestinal lactobacillus from bama centenarians and their probiotic characteristics	Simulated Gastroin-testinal

4	10.13982/j.mfst.1673-9078.2021.4.0782	study on the effect of jupi decoction on the structure of intestinal flora based on shime shime	Shime
2	10.1501/vetfak_0000002212	effects of chicory inulin on ruminal fermentation in vitro hindiba inulininin rumen fermantasyonu zerine in vitro etkileri	Fermenter
2	10.1515/ijfe-2016-0008	impact of encapsulated lactobacillus casei 01 along with pasteurized purple-rice drinks on modulating colon microbiome using a digestive model	Simulator of Human
4	10.1515/pjfns-2017-0010	antioxidative effects of phenolic compounds of mushroom mycelia in simulated regions of the human colon in vitro study	In-vitro Gastroin-testinal
2	10.1530/JME-20-0094	short-chain fatty acids as novel therapeutics for gestational diabetes	In-vitro Model
2	10.15414/JMBFS.2018-19.8.3.940-946	effect of processing procedures on in vitro digestibility and colonic fermentation of riceberry rice	In-vitro Digestion
2	10.15414/JMBFS.2020.9.6.1126-1133	evaluation of lactobacillus paracasei lp11 and lactobacillus rhamnosus 64 potential as candidates for use as probiotics in functional foods	Simulated Gastroin-testinal
2	10.16429/j.1009-7848.2020.07.006	modulation of oligosaccharides with different monosaccharide composition on the human gut microbiota	Simulator of Human
2	10.17113/ftb.58.04.20.6424	influence of diclofenac on activated sludge bacterial communities in fed-batch reactors	Reactor
2	10.1902/jop.2013.130003	high concentration but low activity of hepatocyte growth factor in periodontitis	In-vitro Model
2	10.19675/j.cnki.1006-687x.2017.07017	effect of iron addition on the performance of anoxic-oxic membrane process and biological phosphorus removal	Reactor
8	10.2134/jeq2004.1343	polycyclic aromatic hydrocarbon release from a soil matrix in the in vitro gastrointestinal tract	In-vitro Gastroin-testinal
2	10.2147/IJN.S97177	a novel dissolution media for testing drug release from a nanostructured polysaccharide-based colon specific drug delivery system an approach to alternative colon media	Simulated Colon
2	10.2166/wst.1998.0537	biofilms in drinking water systems - a possible reservoir for helicobacter pylori	Chemostat
2	10.2174/187152008783497037	plant polyphenolics as anti-invasive cancer agents	Shime
2	10.2298/JSC181105015Z	probiotic potential of lactobacillus fermentum g-4 originating from the meconium of newborns	Simulated Gastroin-testinal
2	10.2306/scienceasia1513-1874.2007.33.197	dynamic processes permitting stable coexistence of antimicrobial resistant and non-resistant organisms in a gastrointestinal tract model	Chemostat
2	10.2306/scienceasia1513-1874.2019.45.538	characterization of prebiotics and their synergistic activities with lactobacillus probiotics for -glucuronidase reduction	Simulated Gastroin-testinal
2	10.24275/rmiq/Alim2319	germinated soybean protein hydrolysate ionic gelation encapsulation and release under colonic conditions hidrolizado proteico de soya germinada encapsulacin por gelacin inica y su liberacin en condiciones colnicas	In-vitro Digestion
2	10.2527/2004.8292615x	in vitro fermentation of various fiber and starch sources by pig fecal inocula	Fermenter
2	10.2527/jas.2014-8425	evaluation of soluble corn fiber on chemical composition and nitrogen-corrected true metabolizable energy and its effects on in vitro fermentation and in vivo responses in dogs	In-vitro Digestion
3	10.3109/02652048.2013.834990	enrichment of bifidobacterium longum subsp infantis atcc 15697 within the human gut microbiota using alginate-poly-l-lysine-alginate microencapsulation oral delivery system an in vitro analysis using a computer-controlled dynamic human gastrointestinal model	Gastrointestinal Model

3	10.3109/08910609409141354	validation of the simulator of the human intestinal microbial ecosystem shime reactor using microorganism-associated activities	Reactor
4	10.3109/08910609609166446	effect of lactobacilli on the ecology of the gastro-intestinal microbiota cultured in the shime reactor	Reactor
4	10.3109/09637486.2015.1095865	the degradation of curcuminoids in a human faecal fermentation model	In-vitro Model
2	10.3109/09637486.2015.1134444	antimicrobial activity of selected synbiotics targeted for the elderly against pathogenic escherichia coli strains	Gut Model
2	10.3136/fstr.18.713	selection of lactic acid bacteria as starter cultures for fermented meat products	Simulated Gastroin-testinal
2	10.3168/jds.2008-1437	investigating unsaturated fat monensin or bromoethanesulfonate in continuous cultures retaining ruminal protozoa ii interaction of treatment and presence of protozoa on prokaryotic communities	Fermenter
2	10.3168/jds.2013-6582	preliminary selection for potential probiotic bifidobacterium isolated from subjects of different chinese ethnic groups and evaluation of their fermentation and storage characteristics in bovine milk	Simulated Gastroin-testinal
2	10.3168/jds.2013-7514	lactobacillus casei myl01 modulates the proinflammatory state induced by ethanol in an in vitro model	In-vitro Model
4	10.3168/jds.2015-9356	a novel method for screening of potential probiotics for high adhesion capability	Chemostat
2	10.3168/jds.2018-14581	comparison of antioxidant activities of bovine whey proteins before and after simulated gastrointestinal digestion	Simulated Gastroin-testinal
2	10.3168/jds.2019-17264	microbial transglutaminase alters the immunogenic potential and cross-reactivity of horse and cow milk proteins	Simulated Gastroin-testinal
2	10.3168/jds.2019-18078	buffalo milk increases viability and resistance of probiotic bacteria in dairy beverages under in vitro simulated gastrointestinal conditions	Simulated Gastroin-testinal
2	10.3168/jds.S0022-0302(03)73621-2	the impact of fermentation and in vitro digestion on the formation of angiotensin-i-converting enzyme inhibitory activity from pea and whey protein	In-vitro Digestion
6	10.31989/ffhd.v1i10.119	fermentation by gut microbiota cultured in a simulator of the human intestinal microbial ecosystem is improved by probiotic enterococcus faecium crl 183	Reactor
3	10.31989/ffhd.v5i11.214	recent researches on prebiotics for gut health in thailand	Artificial Gut
2	10.3389/fbioe.2015.00027	the microbial community of a passive biochemical reactor treating arsenic zinc and sulfate-rich seepage	Reactor
2	10.3389/fbioe.2020.00022	bioreactor with electrically deformable curved membranes for mechanical stimulation of cell cultures	In-vitro Model
4	10.3389/fbioe.2020.00024	use of changestat for growth rate studies of gut microbiota	Chemostat
2	10.3389/fbioe.2020.585815	integrated continuous bioprocess development for ace-inhibitory peptide production by lactobacillus helveticus strains in membrane bioreactor	Simulated Gastroin-testinal
2	10.3389/fbioe.2021.695306	diverse profile of fermentation byproducts from thin stillage	Reactor
3	10.3389/fbioe.2021.725443	effect of oxygen contamination on propionate and caproate formation in anaerobic fermentation	Reactor
2	10.3389/fimmu.2019.00094	sialyllactose and galactooligosaccharides promote epithelial barrier functioning and distinctly modulate microbiota composition and short chain fatty acid production in vitro	In-vitro Model

2	10.3389/fmicb.2016.00477	effect of tulathromycin on colonization resistance antimicrobial resistance and virulence of human gut microbiota in chemostats	Chemostat
2	10.3389/fmicb.2017.02623	uncovering the potential of termite gut microbiome for lignocellulose bioconversion in anaerobic batch bioreactors	Artificial Gut
2	10.3389/fmicb.2019.01954	a comprehensive antimicrobial activity evaluation of the recombinant microcin j25 against the foodborne pathogens salmonella and e coli o157h7 by using a matrix of conditions	Simulated Gastroin-testinal
2	10.3389/fmicb.2021.699858	production storage stability and susceptibility testing of reuterin and its impact on the murine fecal microbiome and volatile organic compound profile	Bioreactor
2	10.3389/fmicb.2021.703421	pathogen challenge and dietary shift alter microbiota composition and activity in a mucin-associated in vitro model of the piglet colon mpigut-ivm simulating weaning transition	In-vitro Model
2	10.3389/fnut.2019.00184	in vitro study of lactobacillus paracasei cncm i-1518 in healthy and clostridioides difficile colonized elderly gut microbiota	Continous Fermenta-tion
2	10.3389/fnut.2020.00091	in vitro fermentation of digested milk fat globule membrane from ruminant milk modulates piglet ileal and caecal microbiota	In-vitro Digestion
10	10.3389/fnut.2020.608495	comparison of the bifidogenic effects of goat and cow milk-based infant formulas to human breast milk in an in vitro gut model for 3-month-old infants	In-vitro Digestion
2	10.3389/fnut.2021.707763	in vitro colon fermentation of soluble arabinoxylan is modified through milling and extrusion	In-vitro Colon
2	10.3389/fped.2020.572633	enterococcus faecalis isolated from infant feces inhibits toxigenic clostridioides clostridium difficile	Simulated Gastroin-testinal
3	10.3389/fvets.2021.753527	screening and identification of latilactobacillus curvatus z12 from rumen fluid of an adult female sika deer as a potential probiotic for feed additives	Gastrointestinal Model
2	10.3390/ani11082212	associative effects between forages and concentrates on in vitro fermentation of working equine diets	Fermenter
4	10.3390/antiox10111668	potential of persimmon dietary fiber obtained from byproducts as antioxidant prebiotic and modulating agent of the intestinal epithelial barrier function	In-vitro Digestion
6	10.3390/antiox7030043	microbial biotransformation of a polyphenol-rich potato extract affects antioxidant capacity in a simulated gastrointestinal model	Simulated Gastroin-testinal
2	10.3390/antiox8080307	caffeoylquinic acids and flavonoids of fringed sagewort artemisia frigidawilld hplc-dad-esi-qqq-ms profile hplc-dad quantification in vitro digestion stability and antioxidant capacity	Simulated Gastroin-testinal
4	10.3390/antiox9040302	metabolomic study to evaluate the transformations of extra-virgin olive oils antioxidant phytochemicals during in vitro gastrointestinal digestion	In-vitro Digestion
4	10.3390/antiox9080762	dietary lipids influence bioaccessibility of polyphenols from black carrots and affect microbial diversity under simulated gastrointestinal digestion	Simulated Gastroin-testinal
2	10.3390/app11135765	probiotic potential of a novel vitamin b2-overproducing lactobacillus plantarum strain hy7715 isolated from kimchi	Simulated Gastroin-testinal
4	10.3390/cells10040823	a new strain of christensenella minuta as a potential biotherapy for obesity and associated metabolic diseases	Shime

3	10.3390/cimb43030113	assessment of bioavailability after in vitro digestion and first pass metabolism of bioactive peptides from collagen hydrolysates	Simulated Gastrointestinal
2	10.3390/en11010124	evolving microbial communities in cellulose-fed microbial fuel cell	Reactor
2	10.3390/foods10010179	colon bioaccessibility under in vitro gastrointestinal digestion of different coffee brews chemically profiled through uhplc-q-orbitrap hrms	Simulated Gastrointestinal
2	10.3390/foods10020337	changes in the organosulfur and polyphenol compound profiles of black and fresh onion during simulated gastrointestinal digestion	Simulated Gastrointestinal
3	10.3390/foods10020457	antioxidant activity and bio-accessibility of polyphenols in black carrot daucus carota l ssp sativus var atrorubens alef and two derived products during simulated gastrointestinal digestion and colonic fermentation	Simulated Gastrointestinal
2	10.3390/foods10050921	traditional and non-conventional pasta-making processes effect on in vitro starch digestibility	Simulated Gastrointestinal
2	10.3390/foods10050962	influence of fermentation of pasteurised papaya puree with different lactic acid bacterial strains on quality and bioaccessibility of phenolic compounds during in vitro digestion	In-vitro Digestion
2	10.3390/foods10061198	effect of adding resistant maltodextrin to pasteurized orange juice on bioactive compounds and their bioaccessibility	In-vitro Digestion
2	10.3390/foods10061297	co-encapsulated synbiotics and immobilized probiotics in human health and gut microbiota modulation	Simulated Gastrointestinal
4	10.3390/foods10061420	effect of freeze drying and simulated gastrointestinal digestion on phenolic metabolites and antioxidant property of the natal plum carissa macrocarpa	Simulated Gastrointestinal
4	10.3390/foods10081748	formation of lipid and protein oxidation products during in vitro gastrointestinal digestion of dry-cured loins with different contents of nitratenitrite added	In-vitro Digestion
2	10.3390/foods10081812	delivery of phenolic compounds peptides and -glucan to the gastrointestinal tract by incorporating dietary fibre-rich mushrooms into sorghum biscuits	In-vitro Digestion
2	10.3390/foods10092123	metabolism of phenolics of tetrastigma hemsleyanum roots under in vitro digestion and colonic fermentation as well as their in vivo antioxidant activity in rats	In-vitro Digestion
2	10.3390/foods10092150	a review on chromatography-mass spectrometry applications on anthocyanin and ellagitannin metabolites of blackberries and raspberries	In-vitro Model
2	10.3390/foods10102322	changes in physicochemical and biological properties of polyphenolic-protein-polysaccharide ternary complexes from hovenia dulcis after in vitro simulated saliva-gastrointestinal digestion	In-vitro Digestion
2	10.3390/foods10102435	effect of high hydrostatic pressure on the extractability and bioaccessibility of carotenoids and their esters from papaya carica papaya l and its impact on tissue microstructure	Simulated Gastrointestinal
4	10.3390/foods7010008	absorption and metabolism of phenolics from digests of polyphenol-rich potato extracts using the caco-2hepg2 co-culture system	Reactor
2	10.3390/foods9060756	masking the perceived astringency of proanthocyanidins in beverages using oxidized starch hydrogel microencapsulation	In-vitro Digestion
2	10.3390/foods9081121	the human microbial metabolism of quercetin in different formulations an in vitro evaluation	In-vitro Model

4	10.3390/foods9121816	the composition and antioxidant activity of bound phenolics in three legumes and their metabolism and bioaccessibility of gastrointestinal tract	Simulated Gastrointestinal
4	10.3390/foods9121836	in vitro gastrointestinal digestion and colonic catabolism of mango mangifera indica l pulp polyphenols	In-vitro Digestion
2	10.3390/ijerph13111088	changes in composition and function of human intestinal microbiota exposed to chlorpyrifos in oil as assessed by the shime model	Shime
2	10.3390/ijerph15050993	study of factors influencing the bioaccessibility of triazolone in cherry tomatoes using a static shime model	Shime
2	10.3390/ijms20081925	a small in vitro fermentation model for screening the gut microbiota effects of different fiber preparations	In-vitro Colon
2	10.3390/ijms21030906	in vitro evaluation of different prebiotics on the modulation of gut microbiota composition and function in morbid obese and normal-weight subjects	In-vitro Model
6	10.3390/life11030192	transfer of antibiotic resistance plasmid from commensal e coli towards human intestinal microbiota in the m-shime effect of e coli dosis human individual and antibiotic use	Shime
4	10.3390/metabo10030111	compound characterization and metabolic profile elucidation after in vitro gastrointestinal and hepatic biotransformation of an herniaria hirsuta extract using unbiased dynamic metabolomic data analysis	In-vitro Gastrointestinal
4	10.3390/microorganisms3040725	dynamic in vitro models of the human gastrointestinal tract as relevant tools to assess the survival of probiotic strains and their interactions with gut microbiota	In-vitro Model
2	10.3390/microorganisms7120641	the simplified human intestinal microbiota sihumix shows high structural and functional resistance against changing transit times in in vitro bioreactors	Bioreactor
2	10.3390/microorganisms8010057	the effect of encapsulated powder of goji berry lycium barbarum on growth and survival of probiotic bacteria	Simulated Gastrointestinal
2	10.3390/microorganisms8010060	probiotic supplementation in a clostridium difficile-infected gastrointestinal model is associated with restoring metabolic function of microbiota	Gastrointestinal Model
3	10.3390/microorganisms8071028	bacillus subtilis hu58 and bacillus coagulans sc208 probiotics reduced the effects of antibiotic-induced gut microbiome dysbiosis in an m-shime model	Gut Model
2	10.3390/microorganisms8071053	evaluation of the probiotic properties and the capacity to form biofilms of various lactobacillus strains	In-vitro Model
2	10.3390/microorganisms8101513	bacteroides thetaiotaomicron fosters the growth of butyrate-producing anaerostipes caccae in the presence of lactose and total human milk carbohydrates	Bioreactor
2	10.3390/microorganisms8101582	bioaminergic responses in an in vitro system studying human gut microbiotakiwifruit interactions	In-vitro Gastrointestinal
2	10.3390/microorganisms8111650	streptococcus thermophiles dmst-h2 promotes recovery in mice with antibiotic-associated diarrhea	Simulated Gastrointestinal
2	10.3390/microorganisms9010075	on-site blackwater treatment fosters microbial groups and functions to efficiently and robustly recover carbon and nutrients	Reactor
4	10.3390/microorganisms9020358	an oral fmt capsule as efficient as an enema for microbiota reconstruction following disruption by antibiotics as assessed in an in vitro human gut model	In-vitro Model
2	10.3390/microorganisms9051049	establishment of an in vitro system of the human intestinal microbiota effect of cultivation conditions and influence of three donor stool samples	Bioreactor

2	10.3390/microorganisms9071378	effects of wine and its microbialderived metabolites on intestinal permeability using simulated gastrointestinal digestioncolonic fermentation and caco2 intestinal cell models	Simulated Gastrointestinal
2	10.3390/min9090563	recovery of metals from waste lithium ion battery leachates using biogenic hydrogen sulfide	Reactor
2	10.3390/molecules23071804	effects of in vitro digestion on the content and biological activity of polyphenols from acacia mearnsii bark	In-vitro Digestion
2	10.3390/molecules24050828	in vitro infant faecal fermentation of low viscosity barley -glucan and its acid hydrolyzed derivatives evaluation of their potential as novel prebiotics	Bioreactor
4	10.3390/molecules24193605	in vitro fermentation of polysaccharides from aloe vera and the evaluation of antioxidant activity and production of short chain fatty acids	In-vitro Colon
3	10.3390/molecules24203791	effects of olive and pomegranate by-products on human microbiota a study using the shime in vitro simulator	Shime
3	10.3390/molecules25010184	mainly dimers and trimers of chinese bayberry leaves proanthocyanidins blps are utilized by gut microbiota in vitro digestion and fermentation coupled with caco-2 transportation	In-vitro Digestion
2	10.3390/molecules25030722	microencapsulation of anthocyanin extracted from purple flesh cultivated potatoes by spray drying and its effects on in vitro gastrointestinal digestion	In-vitro Digestion
4	10.3390/molecules25040940	the effect of formulation of curcuminoids on their metabolism by human colonic microbiota	In-vitro Model
4	10.3390/molecules25194354	in vitro digestion and fermentation by human fecal microbiota of polysaccharides from flaxseed	In-vitro Digestion
4	10.3390/molecules25215201	in vitro prebiotic effects of malto-oligosaccharides containing water-soluble dietary fiber	In-vitro Digestion
2	10.3390/molecules26071968	chemical composition in vitro bioaccessibility and antioxidant activity of polyphenolic compounds from nutraceutical fennel waste extract	Simulated Gastrointestinal
2	10.3390/molecules26082330	an in vitro pilot fermentation study on the impact of chlorella pyrenoidosa on gut microbiome composition and metabolites in healthy and coeliac subjects	Gut Model
2	10.3390/molecules26185468	elucidation of interaction between whey proteins and proanthocyanidins and its protective effects on proanthocyanidins during in-vitro digestion and storage	In-vitro Digestion
2	10.3390/nu10070912	bioaccessibility and bioavailability of a marine-derived multimineral aquamin-magnesium	In-vitro Digestion
2	10.3390/nu10111711	colon bioaccessibility and antioxidant activity of white green and black tea polyphenols extract after in vitro simulated gastrointestinal digestion	Simulated Gastrointestinal
2	10.3390/nu11030674	the safety and tolerability of a potential alginate-based iron chelator results of a healthy participant study	Shime
2	10.3390/nu11061424	in vitro interactions of dietary fibre enriched food ingredients with primary and secondary bile acids	In-vitro Digestion
2	10.3390/nu11071663	predicting and testing bioavailability of magnesium supplements	Shime
8	10.3390/nu11092007	probiotic supplementation is associated with increased antioxidant capacity and copper chelation in c difficile-infected fecal water	Simulated Gastrointestinal
2	10.3390/nu12061552	in vitro evaluation of prebiotic properties of a commercial artichoke inflorescence extract revealed bifidogenic effects	Shime
2	10.3390/nu12061802	in vitro fermentation of sheep and cow milk using infant fecal bacteria	In-vitro Digestion

5	10.3390/nu12092800	whole blueberry and isolated polyphenol-rich fractions modulate specific gut microbes in an in vitro colon model and in a pilot study in human consumers	In-vitro Colon
4	10.3390/nu12092808	effects of human milk oligosaccharides on the adult gut microbiota and barrier function	Shime
3	10.3390/nu12103067	wolffia globosamankai plant-based protein contains bioactive vitamin b12 and is well absorbed in humans	Bioreactor
2	10.3390/nu13010031	basolateral secretion from caco-2 cells pretreated with fecal waters from breast cancer patients affects mcf7 cell viability	In-vitro Model
10	10.3390/nu13030726	a comparison of the in vitro effects of 2fucosyllactose and lactose on the composition and activity of gut microbiota from infants and toddlers	In-vitro Model
7	10.3390/nu13030787	colonic in vitro model assessment of the prebiotic potential of bread fortified with polyphenols rich olive fiber	In-vitro Colon
4	10.3390/nu13041125	antioxidant vitamins and prebiotic fos and xos differentially shift microbiota composition and function and improve intestinal epithelial barrier in vitro	Shime
3	10.3390/nu13041332	pork liver pt enriched with persimmon coproducts effect of in vitro gastrointestinal digestion on its fatty acid and polyphenol profile stability	In-vitro Gastrointestinal
5	10.3390/nu13061866	the metabolomic-gut-clinical axis of mankai plant-derived dietary polyphenols	Artificial Gut
4	10.3390/nu13082720	gastrointestinal digestion model assessment of peptide diversity and microbial fermentation products of collagen hydrolysates	In-vitro Digestion
2	10.3390/nu13082746	saccharin and sucralose protect the glomerular microvasculature in vitro against vegf-induced permeability	In-vitro Model
2	10.3390/nu13093190	human milk oligosaccharide 3-gl improves influenza-specific vaccination responsiveness and immunity after deoxynivalenol exposure in preclinical models	In-vitro Model
2	10.3390/nu13113915	a citrus fruit extract high in polyphenols beneficially modulates the gut microbiota of healthy human volunteers in a validated in vitro model of the colon	In-vitro Model
3	10.3390/nu6031115	effect of the novel polysaccharide polyglycoplex on short-chain fatty acid production in a computer-controlled in vitro model of the human large intestine	In-vitro Model
2	10.3390/nu9070767	dose-dependent prebiotic effect of lactulose in a computer-controlled in vitro model of the human large intestine	In-vitro Model
2	10.3390/nu9090953	effects of simulated human gastrointestinal digestion of two purple-fleshed potato cultivars on anthocyanin composition and cytotoxicity in colonic cancer and non-tumorigenic cells	Gastrointestinal Model
4	10.3390/pathogens10020235	in vitroin vivo validation of stimulatory effect of oat ingredients on lactobacilli	Gut Model
2	10.3390/POLYM12061287	formulation and characterization of gelatin-based hydrogels for the encapsulation of kluyveromyces lactis-applications in packed-bed reactors and probiotics delivery in humans	Reactor
2	10.3390/PR8050593	applying differential neural networks to characterize microbial interactions in an ex vivo gastrointestinal gut simulator	Simulator of Human
2	10.3390/su13169302	removal and survival of fecal indicators in a constructed wetland after uasb pre-treatment	Reactor
2	10.3724/SP.J.1145.2015.11061	process construction and fermentation optimization for butyrate production from lactate	Reactor
2	10.37290/ijpp2641-7197.16:22–30	metabolism of wheat dextrin partially hydrolysed guar gum and inulin by bifidobacterium lactis or lactobacillus acidophilus in an in vitro gut model fermentation system	Gut Model
2	10.3748/wjg.v20.i40.14805	intestinal microbiota pathogenesis and fecal microbiota transplantation for inflammatory bowel disease	Chemostat
2	10.3750/AIEP/02251	potential gut adherent probiotic bacteria isolated from rohu labeo rohita actinopterygii cypriniformes cyprinidae characterisation exo-enzyme production pathogen inhibition cell surface hydrophobicity and bio-film formation	In-vitro Model

2	10.3791/57699	assessing the viability of a synthetic bacterial consortium on the in vitro gut host-microbe interface	Simulated Gastrointestinal
4	10.3791/59054	applying advanced in vitro culturing technology to study the human gut microbiota	In-vitro Model
2	10.3844/ajabssp.2010.37.42	viability of lactobacillus delbrueckii under human gastrointestinal conditions simulated in vitro	In-vitro Gastrointestinal
2	10.3892/ol.2021.13003	gold nanoparticles enhance microrna 31 detection in colon cancer cells after inhibition with chlorogenic acid	In-vitro Colon
2	10.3906/biy-1503-9	production of recombinant human dipeptidyl peptidase iv from sf9 cells in microbial fermenters	Bioreactor
3	10.3920/BM2009.0009	survival and metabolic activity of the ganedenbc30 strain of bacillus coagulans in a dynamic in vitro model of the stomach and small intestine	In-vitro Model
3	10.3920/BM2009.0025	xylo-oligosaccharides enhance the growth of bifidobacteria and bifidobacterium lactis in a simulated colon model	Simulated Colon
3	10.3920/BM2010.0038	measuring non-steady-state metabolic fluxes in starch-converting faecal microbiota in vitro	In-vitro Model
2	10.3920/BM2014.0111	production of immune response mediators by ht-29 intestinal cell-lines in the presence of bifidobacterium-treated infant microbiota	Continous Culture
2	10.3920/BM2015.0006	in vitro characterisation of the fermentation profile and prebiotic capacity of gold-fleshed kiwifruit	Simulated Gastrointestinal
2	10.3920/BM2015.0023	bifidobacterium longum d2 enhances microbial degradation of long-chain arabinoxylans in an in vitro model of the proximal colon	In-vitro Model
2	10.3920/BM2016.0013	in vitro colonisation of the distal colon by akkermansia muciniphila is largely mucin and ph dependent	Shime
2	10.3920/BM2018.0037	spores of bacillus coagulans gbi-30 6086 show high germination survival and enzyme activity in a dynamic computer-controlled in vitro model of the gastrointestinal tract	In-vitro Model
2	10.3920/BM2018.0088	effects of functional pasta ingredients on different gut microbiota as revealed by tim-2 in vitro model of the proximal colon	In-vitro Model
2	10.3920/BM2020.0004	bacteroides fragilis prevents salmonella heidelberg translocation in co-culture model mimicking intestinal epithelium	In-vitro Model
2	10.3920/BM2020.0151	lean and obese microbiota differences in in vitro fermentation of food-by-products	In-vitro Model
2	10.3969/j.issn.2095-6002.2019.04.007	study on production of short chain fatty acids from yam oligosaccharides by intestinal probiotics fermentation in vitro	Simulated Colon
2	10.4014/jmb.1705.05055	isolation of probiotic piliated lactobacillus rhamnosus strains from human fecal microbiota using spaa antiserum-based colony immunoblotting	In-vitro Gastrointestinal
4	10.4067/S0717-97072021000305268	effect of in vitro digestion gastrointestinal of the extract aqueou of leaves of ugni molinae on the viability of colorectal cancer cells	In-vitro Digestion
2	10.4103/ijehe.ijehe_56_20	microbial and composition changes during vermicomposting process resulting from decomposable domestic waste cow manure and dewatered sludge	Reactor

2	10.4141/CJAS-2014-051	effect of propionibacterium freudenreichii on ruminal fermentation patterns methane production and lipid biohydrogenation of beef finishing diets containing flaxseed oil in a rumen simulation technique les effets de propionibacterium freudenreichii sur les profils de fermentation ruminale la production de mthane et la biohydrognation des lipides des dites de finition bovine contenant de lhuile de lin dans une technique de simulation du rumen	Artificial Gut
2	10.4251/wjgo.v6.i1.1	approaches that ascertain the role of dietary compounds in colonic cancer cells	In-vitro Model
2	10.4315/0362-028X-71.10.2014	survival of listeria monocytogenes in a simulated dynamic gastrointestinal model during storage of inoculated bologna and salami slices in vacuum packages	Gastrointestinal Model
3	10.4315/0362-028X-72.5.959	characterization of planktonic and biofilm communities of day-of-hatch chicks cecal microflora and their resistance to salmonella colonization	Chemostat
2	10.4315/0362-028X.JFP-18-213	changes of antibiotic resistance phenotype in outbreak-linked salmonella enterica strains after exposure to human simulated gastrointestinal conditions in chicken meat	Simulated Gastrointestinal
4	10.4315/0362-028X.JFP-18-587	a bacteriophage cocktail eliminates salmonella typhimurium from the human colonic microbiome while preserving cytokine signaling and preventing attachment to and invasion of human cells by salmonella in vitro	Shime
2	10.5004/dwt.2018.22797	decarbonization and denitrification characteristics of a coupling abr-mfc-mec process treating black water	Reactor
2	10.5056/jnm16042	colonic transit time is a driven force of the gut microbiota composition and metabolism in vitro evidence	Chemostat
2	10.7506/spkx1002-6630-20191009-053	degradation of sialoglycoprotein from carassius auratus eggs by simulated gastrointestinal tract in vitro	Simulated Gastrointestinal
2	10.7506/spkx1002-6630-20191027-300	effects of in vitro gastrointestinal digestion and colonic fermentation on bioavailability and antioxidant activity of phenolic compounds in changhei hulless barley	In-vitro Gastrointestinal
2	10.7506/spkx1002-6630-20200110-125	gastrointestinal digestion and fermentation characteristics in vitro of breads incorporated with three different polysaccharides 3	Simulated Gastrointestinal
2	10.7537/marslsj120215.03	altered chicken cecal microbial communities affect salmonella colonization	Chemostat
2	10.7717/peerj.2701	soymilk residue okara as a natural immobilization carrier for lactobacillus plantarum cells enhances soymilk fermentation glucosidic isoflavone bioconversion and cell survival under simulated gastric and intestinal conditions	Simulated Gastrointestinal
4	10.7717/peerj.4017	5-fluorouracil and irinotecan sn-38 have limited impact on colon microbial functionality and composition in vitro	Shime
2	10.7845/kjm.2013.232	effects of prebiotics and probiotics on swine intestinal microflora and fermentation products in vitro fermentation	In-vitro Model
2	10.7845/kjm.2015.5066	eco-friendly remediation and reuse for coastal dredged materials using a bioaugmentation technology	Bioreactor
2	NA	azo polymers for colon targeted drug delivery	Simulated Gastrointestinal
2	NA	effects of different prebiotics on viability under in vitro gastrointestinal conditions and sensory properties of fermented milk	Simulated Gastrointestinal

2	NA	isolation identification and evaluation of novel probiotic strains isolated from feces of breast-fed infants	Simulated Gastrointestinal
4	NA	resistance of listeria monocytogenes in simulated gastrointestinal systems	Simulated Gastrointestinal
2	NA	survivability and metabolic activity of lactobacillus casei 01 incorporating lychee juice plus inulin under simulated gastrointestinal environment	Simulated Gastrointestinal
2	NA	acacia gum improves the gut barrier functionality in vitro	Shime
2	NA	construction of the process of butyrate production from lactate and the optimization of fermentation parameters	Reactor
2	NA	pathogens reduction in vermicompost process resulted from the mixed sludge treatments-household wastes	Reactor
2	NA	production of galacto-oligosaccharides from lactose by immobilized -galactosidase	Reactor
2	NA	reduction of pathogens from mixture of cow manure domestic waste and wastewater treatment plant sludge by vermicomposting process	Reactor
3	NA	chocolate an ideal carrier for probiotics	In-vitro Model
2	NA	development validation and implementation of an in vitro model for the study of metabolic and immune function in normal and inflamed human colonic epithelium	In-vitro Model
4	NA	opening ways for soy isoflavone benefits to a larger public	In-vitro Model
2	NA	the survival of and cytokine induction by lactic acid bacteria after passage through a gastrointestinal model	In-vitro Gastrointestinal
2	NA	effect of process on phytochemicals and the function of improving fecal microflora of seabuckthorn leaf tea	In-vitro Digestion
2	NA	effects of different sea buckthorn leaf tea processing technologies on nutrient level and fecal microflora in vitro	In-vitro Digestion
4	NA	influence of enzyme-resistant fraction of sorghum sorghum bicolor l flour on gut microflora composition short chain fatty acid production and toxic substance metabolism	In-vitro Digestion
2	NA	prebiotic potential of some exopolysaccharides produced by lactic acid bacteria	In-vitro Digestion
4	NA	in vitro digestion models for dietary phenolic compounds	In-vitro Colon
2	NA	comparative analysis of digestive ecosystems in the rumen of the cow and in the caecum of the rabbit analyse compare des cosystmes digestifs du rumen de la vache et du caecum du lapin	Fermenter
2	NA	impact of ganedenbc30 bacillus coagulans gbi-30 6086 on population dynamics of the human gut microbiota in a continuous culture fermentation system	Continous Culture
4	NA	biodiversity of human faecal bacteria isolated from phytic acid enriched chemostat fermenters	Chemostat
2	NA	intelligent neural network for bacteria classification an innovation in artificial neural network	Artificial Gut

Table S8: Source journals of publications (e.g. journal names) (n = 401).

Journal	Number of Publications
FOOD RESEARCH INTERNATIONAL	60
FOOD AND FUNCTION	59
JOURNAL OF AGRICULTURAL AND FOOD CHEMISTRY	58
JOURNAL OF FUNCTIONAL FOODS	58
FOOD CHEMISTRY	50
LWT	37
FEMS MICROBIOLOGY ECOLOGY	32
APPLIED AND ENVIRONMENTAL MICROBIOLOGY	31
NUTRIENTS	31
APPLIED MICROBIOLOGY AND BIOTECHNOLOGY	22
JOURNAL OF ANTIMICROBIAL CHEMOTHERAPY	22
FOODS	21
PLOS ONE	21
FOOD HYDROCOLLOIDS	19
BENEFICIAL MICROBES	18
FRONTIERS IN MICROBIOLOGY	16
JOURNAL OF FOOD SCIENCE	16
SCIENTIFIC REPORTS	16
ENVIRONMENTAL SCIENCE AND TECHNOLOGY	15
JOURNAL OF THE SCIENCE OF FOOD AND AGRICULTURE	15
MICROORGANISMS	14
CARBOHYDRATE POLYMERS	13
FOOD BIOSCIENCE	13
INTERNATIONAL JOURNAL OF FOOD SCIENCE AND TECHNOLOGY	13
INTERNATIONAL JOURNAL OF FOOD SCIENCES AND NUTRITION	13
MOLECULAR NUTRITION AND FOOD RESEARCH	13
MOLECULES	12
ANAEROBE	11
BRITISH JOURNAL OF NUTRITION	11
JOURNAL OF APPLIED MICROBIOLOGY	11
JOURNAL OF MICROBIOLOGICAL METHODS	11
BMC MICROBIOLOGY	10
FOOD MICROBIOLOGY	10
ANIMAL FEED SCIENCE AND TECHNOLOGY	9
ENVIRONMENTAL MICROBIOLOGY	9
INTERNATIONAL JOURNAL OF FOOD MICROBIOLOGY	9
INTERNATIONAL JOURNAL OF PHARMACEUTICS	9
JOURNAL OF DAIRY SCIENCE	9
MICROBIAL ECOLOGY IN HEALTH AND DISEASE	9
SCIENCE OF THE TOTAL ENVIRONMENT	9
ANNALS OF MICROBIOLOGY	8
INTERNATIONAL JOURNAL OF BIOLOGICAL MACROMOLECULES	8
LWT - FOOD SCIENCE AND TECHNOLOGY	8
CHEMOSPHERE	7
EUROPEAN JOURNAL OF NUTRITION	7
FOOD AND CHEMICAL TOXICOLOGY	7
FRONTIERS IN BIOENGINEERING AND BIOTECHNOLOGY	7
JOURNAL OF NUTRITION	7
PROBIOTICS AND ANTIMICROBIAL PROTEINS	7
ANTIOXIDANTS	6
ENVIRONMENTAL POLLUTION	6
INTERNATIONAL DAIRY JOURNAL	6
MICROBIAL ECOLOGY	6
MICROBIOME	6
BIOTECHNOLOGY AND BIOENGINEERING	5
FEMS MICROBIOLOGY LETTERS	5
FRONTIERS IN NUTRITION	5
REGULATORY TOXICOLOGY AND PHARMACOLOGY	5
ANTIMICROBIAL AGENTS AND CHEMOTHERAPY	4
FOOD AND BIOPRODUCTS PROCESSING	4
GUT MICROBES	4

INNOVATIVE FOOD SCIENCE AND EMERGING TECHNOLOGIES	4
JOURNAL OF ANIMAL SCIENCE	4
JOURNAL OF FOOD PROTECTION	4
JOURNAL OF FOOD SCIENCE AND TECHNOLOGY	4
JOURNAL OF HAZARDOUS MATERIALS	4
JOURNAL OF MEDICAL MICROBIOLOGY	4
NATURE COMMUNICATIONS	4
PLANT FOODS FOR HUMAN NUTRITION	4
SHIPIN KEXUE/FOOD SCIENCE	4
TOXICOLOGY IN VITRO	4
AGRO FOOD INDUSTRY HI-TECH	3
APPLIED SCIENCES (SWITZERLAND)	3
BIOACTIVE CARBOHYDRATES AND DIETARY FIBRE	3
CEREAL CHEMISTRY	3
CURRENT MICROBIOLOGY	3
EUROPEAN JOURNAL OF PHARMACEUTICS AND BIOPHARMACEUTICS	3
GASTROENTEROLOGY	3
GUT PATHOGENS	3
INTERNATIONAL JOURNAL OF ENVIRONMENTAL RESEARCH AND PUBLIC HEALTH	3
INTERNATIONAL JOURNAL OF MOLECULAR SCIENCES	3
ISME JOURNAL	3
JOURNAL OF CHROMATOGRAPHY B: ANALYTICAL TECHNOLOGIES IN THE BIOMEDICAL AND LIFE SCIENCES	3
JOURNAL OF FOOD ENGINEERING	3
JOURNAL OF PERIODONTOLOGY	3
JOURNAL OF PROTEOME RESEARCH	3
LETTERS IN APPLIED MICROBIOLOGY	3
MODERN FOOD SCIENCE AND TECHNOLOGY	3
MSYSTEMS	3
PEERJ	3
PROCEEDINGS OF THE NATIONAL ACADEMY OF SCIENCES OF THE UNITED STATES OF AMERICA	3
RSC ADVANCES	3
WORLD JOURNAL OF MICROBIOLOGY AND BIOTECHNOLOGY	3
ACS OMEGA	2
ADVANCED HEALTHCARE MATERIALS	2
ADVANCED MATERIALS TECHNOLOGIES	2
ADVANCED SCIENCE	2
ALGAL RESEARCH	2
ANIMAL SCIENCE JOURNAL	2
APPLIED BIOCHEMISTRY AND BIOTECHNOLOGY	2
ARCHIVES OF ORAL BIOLOGY	2
ARTIFICIAL CELLS BLOOD SUBSTITUTES AND BIOTECHNOLOGY	2
BIOMED RESEARCH INTERNATIONAL	2
BIOSENSORS AND BIOELECTRONICS	2
BIOTECHNOLOGY FOR BIOFUELS	2
BIOTECHNOLOGY JOURNAL	2
BIOTECHNOLOGY LETTERS	2
BMC BIOLOGY	2
BMC GASTROENTEROLOGY	2
BMC RESEARCH NOTES	2
CHEMICAL ENGINEERING JOURNAL	2
CHINESE JOURNAL OF APPLIED AND ENVIRONMENTAL BIOLOGY	2
COMPREHENSIVE REVIEWS IN FOOD SCIENCE AND FOOD SAFETY	2
CURRENT ISSUES IN INTESTINAL MICROBIOLOGY	2
DISSOLUTION TECHNOLOGIES	2
ECOTOXICOLOGY AND ENVIRONMENTAL SAFETY	2
ENVIRONMENTAL HEALTH PERSPECTIVES	2
ENVIRONMENTAL SCIENCE AND POLLUTION RESEARCH	2
ENVIRONMENTAL SCIENCE: NANO	2
EUROPEAN JOURNAL OF CLINICAL NUTRITION	2
EUROPEAN JOURNAL OF PHARMACEUTICAL SCIENCES	2
FERMENTATION	2
FOOD CONTROL	2

FOOD DIGESTION	2
FOOD SCIENCE AND BIOTECHNOLOGY	2
FOOD SCIENCE AND TECHNOLOGY RESEARCH	2
FOODBORNE PATHOGENS AND DISEASE	2
FUNCTIONAL FOODS IN HEALTH AND DISEASE	2
GUT	2
HELIYON	2
INDUSTRIAL CROPS AND PRODUCTS	2
INFECTION AND IMMUNITY	2
INTERNATIONAL BIODETERIORATION AND BIODEGRADATION	2
INTERNATIONAL FOOD RESEARCH JOURNAL	2
INTERNATIONAL JOURNAL OF DAIRY TECHNOLOGY	2
INTERNATIONAL JOURNAL OF MEDICAL MICROBIOLOGY	2
INTERNATIONAL JOURNAL OF PROBIOTICS AND PREBIOTICS	2
JAMA PEDIATRICS	2
JOURNAL OF BIOSCIENCE AND BIOENGINEERING	2
JOURNAL OF CLINICAL GASTROENTEROLOGY	2
JOURNAL OF ENVIRONMENTAL ENGINEERING (UNITED STATES)	2
JOURNAL OF ENVIRONMENTAL QUALITY	2
JOURNAL OF ENVIRONMENTAL SCIENCE AND HEALTH - PART A TOXIC/HAZARDOUS SUBSTANCES AND ENVIRONMENTAL ENGINEERING	2
JOURNAL OF ETHNOPHARMACOLOGY	2
JOURNAL OF FOOD AND NUTRITION RESEARCH	2
JOURNAL OF FOOD BIOCHEMISTRY	2
JOURNAL OF FOOD COMPOSITION AND ANALYSIS	2
JOURNAL OF FOOD MEASUREMENT AND CHARACTERIZATION	2
JOURNAL OF FOOD PROCESSING AND PRESERVATION	2
JOURNAL OF MEDICINAL FOOD	2
JOURNAL OF MICROBIOLOGY	2
JOURNAL OF MICROBIOLOGY BIOTECHNOLOGY AND FOOD SCIENCES	2
JOURNAL OF MICROENCAPSULATION	2
KOREAN JOURNAL OF MICROBIOLOGY	2
METHODS IN MOLECULAR BIOLOGY	2
MICROBIAL BIOTECHNOLOGY	2
MICROBIOLOGY	2
MOLECULAR ECOLOGY	2
MOLECULES (BASEL SWITZERLAND)	2
NANOIMPACT	2
NPJ BIOFILMS AND MICROBIOMES	2
NUTRITION RESEARCH	2
PATHOGENS	2
PEDIATRIC RESEARCH	2
RESEARCH IN MICROBIOLOGY	2
SCIENCEASIA	2
STARCH/STAERKE	2
AAPS PHARMSCITECH	1
ACS APPLIED MATERIALS AND INTERFACES	1
ACS INFECTIOUS DISEASES	1
ACS SYMPOSIUM SERIES	1
ACTA BIOCHIMICA POLONICA	1
ACTA BIOTECHNOLOGICA	1
ACTA ICHTHYOLOGICA ET PISCATORIA	1
ADVANCED BIOSYSTEMS	1
ADVANCED MATERIALS	1
ADVANCES IN DENTAL RESEARCH	1
AGRICULTURE AND NATURAL RESOURCES	1
AICHE JOURNAL	1
AMB EXPRESS	1
AMERICAN JOURNAL OF AGRICULTURAL AND BIOLOGICAL SCIENCE	1
AMERICAN JOURNAL OF CLINICAL NUTRITION	1
AMERICAN JOURNAL OF PHYSICAL ANTHROPOLOGY	1
AMERICAN JOURNAL OF PHYSIOLOGY - ENDOCRINOLOGY AND METABOLISM	1
AMERICAN JOURNAL OF PRIMATOLOGY	1
ANALYTICAL CHEMISTRY	1

ANGEWANDTE CHEMIE - INTERNATIONAL EDITION	1
ANIMALS	1
ANKARA UNIVERSITESI VETERINER FAKULTESI DERGISI	1
ANNALES PHARMACEUTIQUES FRANCAISES	1
ANNALS OF NEUROLOGY	1
ANNUAL REVIEW OF FOOD SCIENCE AND TECHNOLOGY	1
ANTIBIOTICS	1
ANTONIE VAN LEEUWENHOEK INTERNATIONAL JOURNAL OF GENERAL AND MOLECULAR MICROBIOLOGY	1
APMIS	1
AQUACULTURAL ENGINEERING	1
ARCHIVES OF ENVIRONMENTAL CONTAMINATION AND TOXICOLOGY	1
ARCHIVES OF SURGERY	1
ARTIFICIAL CELLS NANOMEDICINE AND BIOTECHNOLOGY	1
ASAIO JOURNAL	1
AUTISM RESEARCH	1
BEVERAGES	1
BIOCHIP JOURNAL	1
BIOFACTORS	1
BIOINFORMATICS	1
BIOINSPIRATION AND BIOMIMETICS	1
BIOLOGY OF REPRODUCTION	1
BIOMATERIALS	1
BIOMATERIALS SCIENCE	1
BIOMEDICAL CHROMATOGRAPHY	1
BIOMEDICAL MICRODEVICES	1
BIOMOLECULES	1
BIOORGANIC AND MEDICINAL CHEMISTRY	1
BIOPROCESS AND BIOSYSTEMS ENGINEERING	1
BIOSCIENCE BIOTECHNOLOGY AND BIOCHEMISTRY	1
BIOTECHNOLOGY	1
BOTANICAL REVIEW	1
BRAIN RESEARCH	1
BRAZILIAN JOURNAL OF MICROBIOLOGY	1
CANADIAN JOURNAL OF ANIMAL SCIENCE	1
CANADIAN JOURNAL OF MICROBIOLOGY	1
CANCER RESEARCH	1
CELL BIOLOGY INTERNATIONAL	1
CELL HOST AND MICROBE	1
CELLULAR MICROBIOLOGY	1
CHEMICAL AND BIOLOGICAL TECHNOLOGIES IN AGRICULTURE	1
CHEMICAL ENGINEERING RESEARCH AND DESIGN	1
CHEMICAL ENGINEERING SCIENCE	1
CLINICAL INFECTIOUS DISEASES	1
COMMUNICATIONS BIOLOGY	1
COMPREHENSIVE PHYSIOLOGY	1
CONTEMPORARY CLINICAL TRIALS COMMUNICATIONS	1
CRITICAL REVIEWS IN FOOD SCIENCE AND NUTRITION	1
CRITICAL REVIEWS IN MICROBIOLOGY	1
CURRENT ISSUES IN MOLECULAR BIOLOGY	1
CURRENT MEDICAL SCIENCE	1
CYTOMETRY PART A	1
CYTOTECHNOLOGY	1
DANISH MEDICAL JOURNAL	1
DESALINATION AND WATER TREATMENT	1
DIGESTIVE DISEASES AND SCIENCES	1
DMM DISEASE MODELS AND MECHANISMS	1
DRUG DELIVERY	1
EBIOMEDICINE	1
ECOLOGY AND EVOLUTION	1
ENERGIES	1
ENERGY AND ENVIRONMENTAL SCIENCE	1
ENVIRONMENT INTERNATIONAL	1
ENVIRONMENTAL CHEMISTRY	1
ENVIRONMENTAL ENGINEERING SCIENCE	1

ENVIRONMENTAL MICROBIOLOGY REPORTS	1
ENVIRONMENTAL PROCESSES	1
ENVIRONMENTAL TECHNOLOGY AND INNOVATION	1
EUROPEAN JOURNAL OF CANCER PREVENTION	1
EUROPEAN JOURNAL OF IMMUNOLOGY	1
EXPERT OPINION ON DRUG DISCOVERY	1
FASEB JOURNAL	1
FOLIA MICROBIOLOGICA	1
FOOD AND BIOPROCESS TECHNOLOGY	1
FOOD BIOTECHNOLOGY	1
FOOD QUALITY AND SAFETY	1
FOOD SCIENCE AND TECHNOLOGY	1
FOOD SCIENCE OF ANIMAL RESOURCES	1
FOOD TECHNOLOGY AND BIOTECHNOLOGY	1
FRONTIERS IN CELLULAR AND INFECTION MICROBIOLOGY	1
FRONTIERS IN IMMUNOLOGY	1
FRONTIERS IN PEDIATRICS	1
FRONTIERS IN VETERINARY SCIENCE	1
GRAEFE'S ARCHIVE FOR CLINICAL AND EXPERIMENTAL OPHTHALMOLOGY	1
HEPATOLOGY	1
HUAGONG XUEBAO/CIESC JOURNAL	1
HUANJING KEXUE XUEBAO/ACTA SCIENTIAE CIRCUMSTANTIAE	1
HUANJING KEXUE/ENVIRONMENTAL SCIENCE	1
IEEE TRANSACTIONS ON BIOMEDICAL ENGINEERING	1
IMMUNOBIOLOGY	1
INDIAN JOURNAL OF PHARMACEUTICAL SCIENCES	1
INTERNATIONAL JOURNAL OF BIOCHEMISTRY AND CELL BIOLOGY	1
INTERNATIONAL JOURNAL OF ENVIRONMENTAL HEALTH ENGINEERING	1
INTERNATIONAL JOURNAL OF FOOD ENGINEERING	1
INTERNATIONAL JOURNAL OF HYDROGEN ENERGY	1
INTERNATIONAL JOURNAL OF MICROBIOLOGY	1
INTERNATIONAL JOURNAL OF NANOMEDICINE	1
INTERNATIONAL JOURNAL OF PHARMACEUTICS: X	1
INTERNATIONAL JOURNAL OF POLYMER SCIENCE	1
INTERNATIONAL JOURNAL OF RECYCLING OF ORGANIC WASTE IN AGRICULTURE	1
ITALIAN JOURNAL OF FOOD SCIENCE	1
JOURNAL OF ANIMAL AND VETERINARY ADVANCES	1
JOURNAL OF ANIMAL SCIENCE AND BIOTECHNOLOGY	1
JOURNAL OF APPLIED BACTERIOLOGY	1
JOURNAL OF BACTERIOLOGY	1
JOURNAL OF BIOMEDICINE AND BIOTECHNOLOGY	1
JOURNAL OF CEREAL SCIENCE	1
JOURNAL OF CHINESE INSTITUTE OF FOOD SCIENCE AND TECHNOLOGY	1
JOURNAL OF CLEANER PRODUCTION	1
JOURNAL OF CLINICAL PERIODONTOLOGY	1
JOURNAL OF DENTAL RESEARCH	1
JOURNAL OF DIETARY SUPPLEMENTS	1
JOURNAL OF ENVIRONMENTAL HEALTH SCIENCE AND ENGINEERING	1
JOURNAL OF ENVIRONMENTAL MONITORING	1
JOURNAL OF ENVIRONMENTAL SCIENCE AND HEALTH - PART B PESTICIDES FOOD CONTAMINANTS AND AGRICULTURAL WASTES	1
JOURNAL OF ENVIRONMENTAL SCIENCE AND HEALTH. PART A TOXIC/HAZARDOUS SUBSTANCES & ENVIRONMENTAL ENGINEERING	1
JOURNAL OF FOOD SCIENCE AND TECHNOLOGY (CHINA)	1
JOURNAL OF GENERAL MICROBIOLOGY	1
JOURNAL OF GLOBAL ANTIMICROBIAL RESISTANCE	1
JOURNAL OF HOSPITAL INFECTION	1
JOURNAL OF INHERITED METABOLIC DISEASE	1
JOURNAL OF MATERIALS CHEMISTRY B	1
JOURNAL OF MICROBIOLOGY AND BIOTECHNOLOGY	1
JOURNAL OF MOLECULAR ENDOCRINOLOGY	1
JOURNAL OF MOLECULAR MICROBIOLOGY AND BIOTECHNOLOGY	1
JOURNAL OF NEUROGASTROENTEROLOGY AND MOTILITY	1

JOURNAL OF NUTRITIONAL BIOCHEMISTRY	1
JOURNAL OF PARENTERAL AND ENTERAL NUTRITION	1
JOURNAL OF PHARMACEUTICAL AND BIOMEDICAL ANALYSIS	1
JOURNAL OF PHARMACEUTICAL SCIENCES	1
JOURNAL OF PHARMACY AND PHARMACOLOGY	1
JOURNAL OF PROTEOMICS	1
JOURNAL OF PURE AND APPLIED MICROBIOLOGY	1
JOURNAL OF RADIOLOGICAL PROTECTION	1
JOURNAL OF THE AMERICAN COLLEGE OF NUTRITION	1
JOURNAL OF THE CHILEAN CHEMICAL SOCIETY	1
JOURNAL OF THE MEDICAL ASSOCIATION OF THAILAND	1
JOURNAL OF THE SERBIAN CHEMICAL SOCIETY	1
JOURNAL OF THEORETICAL BIOLOGY	1
JOURNAL OF VISUALIZED EXPERIMENTS	1
JOURNAL OF VISUALIZED EXPERIMENTS : JOVE	1
JOURNAL OF WATER PROCESS ENGINEERING	1
KLINIKARZT	1
LAB ON A CHIP	1
LIFE	1
LIFE SCIENCE JOURNAL	1
MATERIALS TODAY BIO	1
MBIO	1
MEAT SCIENCE	1
MED	1
MEDICAL MYCOLOGY	1
METABOLITES	1
METABOLOMICS	1
MICROBES AND ENVIRONMENTS	1
MINERAL PROCESSING AND EXTRACTIVE METALLURGY REVIEW	1
MINERALS	1
MOLECULAR AND CELLULAR BIOCHEMISTRY	1
MOLECULAR BIOSYSTEMS	1
MOLECULAR THERAPY - NUCLEIC ACIDS	1
MSPHERE	1
MYCOTOXIN RESEARCH	1
NANOMATERIALS	1
NATURE BIOMEDICAL ENGINEERING	1
NATURE PROTOCOLS	1
NMR IN BIOMEDICINE	1
NONGYE GONGCHENG XUEBAO/TRANSACTIONS OF THE CHINESE SOCIETY OF AGRICULTURAL ENGINEERING	1
NUTRITION AND CANCER	1
NUTRITION AND FOOD SCIENCE	1
NUTRITION METABOLISM AND CARDIOVASCULAR DISEASES	1
ONCOLOGY LETTERS	1
ONCOTARGET	1
OXIDATION COMMUNICATIONS	1
PATHOLOGY RESEARCH AND PRACTICE	1
PHYSIOLOGICAL GENOMICS	1
PHYTOMEDICINE	1
PLANTA MEDICA	1
POLISH JOURNAL OF FOOD AND NUTRITION SCIENCES	1
POLYMER BULLETIN	1
POLYMERS	1
POLYMERS FOR ADVANCED TECHNOLOGIES	1
PREPARATIVE BIOCHEMISTRY AND BIOTECHNOLOGY	1
PROCEEDINGS OF THE NUTRITION SOCIETY	1
PROCESS BIOCHEMISTRY	1
PROCESS SAFETY AND ENVIRONMENTAL PROTECTION	1
PROCESSES	1
PROTEIN EXPRESSION AND PURIFICATION	1
RAPID COMMUNICATIONS IN MASS SPECTROMETRY	1
RESEARCH IN VETERINARY SCIENCE	1
REVISTA MEXICANA DE INGENIERA QUIMICA	1
ROMANIAN BIOTECHNOLOGICAL LETTERS	1

SAUDI JOURNAL OF BIOLOGICAL SCIENCES	1
SENSORS AND ACTUATORS B: CHEMICAL	1
SHOCK	1
SIAM JOURNAL ON APPLIED MATHEMATICS	1
SMALL	1
SOFTWAREX	1
SUSTAINABILITY (SWITZERLAND)	1
SYSTEMATIC AND APPLIED MICROBIOLOGY	1
TAIWANESE JOURNAL OF AGRICULTURAL CHEMISTRY AND FOOD SCIENCE	1
TALANTA	1
THE BRITISH JOURNAL OF NUTRITION	1
TISSUE ENGINEERING - PART C: METHODS	1
TOXICOLOGY LETTERS	1
TURKISH JOURNAL OF BIOLOGY	1
UROLOGICAL RESEARCH	1
VETERINARY QUARTERLY	1
VETERINARY SCIENCE COMMUNICATIONS	1
VIRUSES	1
VTT PUBLICATIONS	1
WASTE AND BIOMASS VALORIZATION	1
WATER RESEARCH	1
WATER SCIENCE AND TECHNOLOGY	1
WELLCOME OPEN RESEARCH	1
WORLD JOURNAL OF GASTROINTESTINAL ONCOLOGY	1
ZEITSCHRIFT FUR GASTROENTEROLOGIE	1
ZENTRALBLATT FUR BAKTERIOLOGIE MIKROBIOLOGIE UND HYGIENE - ABT. 1 ORIG. A	1

Table S9: New authors introduced per year (1978 - 2022).

Year	New authors/year
1978	2
1982	3
1985	4
1987	9
1988	3
1989	4
1990	5
1991	2
1992	6
1993	10
1994	3
1995	19
1996	18
1997	9
1998	23
1999	13
2000	28
2001	22
2002	16
2003	34
2004	62
2005	56
2006	77
2007	76
2008	48
2009	74
2010	113
2011	162
2012	199
2013	275
2014	251
2015	337
2016	508
2017	402
2018	446
2019	737
2020	743
2021	876
2022	43

Table S10: Authors affiliation country. List was retrieved with interllixir program using the same list of documents and manually verified.

Author	Country	Count
Aalvink S	France	1
Abadía-garcía L	Mexico	1
Abadias M	Spain	1
Abbasi S	Canada	1
Abbaszadeh S	Iran	1
Abdennebi-najar L	France	1
Abdullah A	Malaysia	2
Abdur-rashid K	United Kingdom	1
Abel-santos E	United States of America	1
Abou-elwafa abdallah M	United Kingdom	1
Abou-samra E	Canada	1
Abraham A-l	France	1
Abraham Ag	Spain	2
Abrahamsson B	Switzerland	2
Abratt Vr	United Kingdom	1
Abrunhosa L	Colombia	1
Abud-archila M	United States of America	1
Acar Et	Turkey	1
Acevedo Nc	United States of America	1
Acevedo-fani A	New Zealand	1
Adamberg K	Estonia	3
Adamberg S	Estonia	2
Adarkwah-yiadom M	South Africa	1
Addeo F	Italy	1
Adel-patient K	France	1
Adiels M	Sweden	1
Adisakwattana S	Thailand	1
Adorno Mat	Brazil	11
Adouard N	France	2
Afkhami F	Canada	2
Afzaal M	Pakistan	1
Agellon Lb	Canada	1
Agudelo Cd	Colombia	1
Aguilera-velázquez Jr	Spain	1
Aguirre M	Netherlands	4
Aguirre-calvo Tr	Spain	2
Ah Uv	Switzerland	1
Ahammad Sz	China	1
Aharoni A	United States of America	1
Ahire Jj	India	1
Ahlborn N	New Zealand	1
Ahles S	Netherlands	1
Ahluwalia A	Italy	4
Ahmad W	China	1
Ahn Y	United States of America	1
Ahonen I	United States of America	1
Ahrenkiel Dw	Denmark	1
Ahring Bk	United States of America	1
Ai C	China	1
Ai L	China	1
Ai Y	China	1
Ai Z	China	1
Aidy ei S	France	1
Aizenberg-gershtein Y	Israel	1
Ajlouni S	Australia	5
Akan E	Turkey	1
Akindahunsi Aa	Brazil	1
Akinola Sa	France	1
Akiyama T	France	1
Akman Pk	Turkey	1

Akyüz S	Turkey	1
Al Kf	United Kingdom	1
Al-otaibi Mm	Saudi Arabia	1
Al-salami H	Canada	1
Alamilla-beltrán L	Mexico	1
Alander M	Netherlands	2
Alao Of	Brazil	1
Alard J	France	1
Alarifi S	United Kingdom	1
Alarifi Sn	United Kingdom	1
Alarjani Km	Kuwait	1
Alava P	China	5
Alazzeh Ay	Brazil	1
Albertengo L	Brazil	1
Alberto S-bj	Sweden	1
Albrecht S	Netherlands	2
Alegria A	Spain	4
Alessandri G	France	1
Alexander R	Brazil	1
Alfred rider J	United States of America	1
Alge Dl	United States of America	1
Ali Hm	Egypt	1
Ali M	United States of America	1
Allard M	United States of America	1
Alldrick Aj	United Kingdom	1
Allegrini P	Italy	2
Allen R	United States of America	1
Allen-vercoe E	Canada	10
Allert S	Germany	1
Allison C	United Kingdom	1
Allison J	United States of America	1
Allsopp P	United Kingdom	3
Almada Cn	Brazil	1
Almada-érix Cn	Brazil	1
Almeida A	Belgium	1
Almeida da costa Wk	Brazil	1
Almeida G	Portugal	1
Almeida M	France	2
Alminger M	Sweden	3
Alminger Ml	Sweden	1
Almutairi Am	Kuwait	1
Alonso Jl	Spain	2
Alpsten M	Sweden	1
Alqurashi Rm	United Kingdom	1
Alric M	France	15
Altringham J	United Kingdom	2
Alvarado-jasso Gm	Mexico	1
Alvarado-moreno Ja	Mexico	1
Alvarez Md	Spain	2
Alvarez-puebla Ra	Spain	1
Alvarez-rivera G	Spain	1
Alvarez-silva C	Denmark	1
Alves Â	France	1
Alves Ji	Portugal	2
Alves Mm	Portugal	1
Alvito P	France	4
Amadò R	United States of America	1
Amaretti A	Italy	2
Amaro A	France	1
Amaya-llano Sl	Mexico	1
America Ahp	United States of America	1
Ames Jm	United Kingdom	1
Amin I	Malaysia	1
Ammar Rm	Germany	1
Amorim C	Portugal	2

Amorim M	France	2
Ampuero D	Chile	1
An J-x	China	1
Anand S	United States of America	1
Anandharamakrishnan C	India	1
Anantharaman K	United States of America	1
Anderson A	United Kingdom	1
Andlid T	Sweden	1
Andrade Rmsd	France	1
Andremont A	France	1
Andrés C	Brazil	1
Andrews K	United Kingdom	1
Andriamihaja M	France	1
Andrioli Jl	Brazil	1
Ángel rufián-henares J	Spain	1
Angeli A	Israel	1
Angenent Lt	United States of America	3
Anglenius H	United States of America	1
Anguera M	Spain	1
Anishaparvin A	India	1
Anjum M	Denmark	1
Ann augustin M	Australia	1
Annapure UNITED-STATES	India	1
Annunziata G	Italy	1
Annuzzi G	Sweden	1
Ansell J	New Zealand	3
Anson Nm	Netherlands	2
Ansorena D	Spain	1
Antoine T	France	1
Antoniassi R	Brazil	1
Antonissen G	Austria	1
Antranikian G	Germany	1
Anu-appaiah Ka	India	1
Anuyahong T	Thailand	1
Aotsuka Y	Japan	1
Apajalahti Jha	United States of America	1
Apers S	Belgium	1
Apichartsrangkoon A	Thailand	6
Aponte M	Italy	1
Apostolou A	United States of America	1
Aragon Dc	Brazil	1
Aragon-alegro Lc	Brazil	1
Aragón-rojas S	United Kingdom	1
Arai N	United Kingdom	1
Arapitsas P	United States of America	1
Araújo Ls	Brazil	1
Arbizu S	United States of America	1
Arboleya S	Spain	2
Arcand Y	United Kingdom	4
Arcoraci T	Italy	1
Ardila Ms	Germany	1
Arellano K	South Korea	1
Arena E	Italy	1
Arendt Ek	France	1
Argyri Aa	United Kingdom	1
Ariëns Rmc	United States of America	2
Arike L	Estonia	1
Arilla E	Spain	1
Arinos S	Germany	1
Arioglu-tuncil S	United States of America	1
Arisoy S	Turkey	1
Arlorio M	Italy	1
Arning E	Canada	1
Arnone Aa	United States of America	1
Arojju Sk	New Zealand	1

Aroonkesorn A	Thailand	1
Arqués Jl	Spain	1
Arranz E	Ireland	1
Arriaga-pizano L	Mexico	1
Arrieta-ortiz Ml	United States of America	1
Arrigoni E	United States of America	1
Arroyo Mc	Belgium	1
Arruda Hs	Brazil	1
Arshad Mu	Pakistan	1
Artacho A	Brazil	1
Arumugam M	Denmark	1
Arvízu Sm	Mexico	1
Asavarut P	Thailand	1
Ashwin H	United Kingdom	1
Aspholm M	Norway	1
Assunção R	France	3
Astiasarán I	Spain	1
Astiazarán-garcía H	Mexico	1
Atamer Z	France	1
Atanasov J	Germany	1
Athanasiou A	United Kingdom	1
Athayde Ml	Brazil	1
Atraki R	Iran	1
Attri S	India	1
Aubry M	France	1
Auchtung J	United States of America	1
Auchtung Jm	United States of America	2
Aucoin Mg	Canada	1
Auer L	France	1
Augustin Ma	Australia	1
Aura A-m	Finland	14
Austad Sn	United States of America	1
Avalakki Uk	India	1
Avello M	Chile	1
Avendaño-pérez G	United Kingdom	1
Ávila-reyes Sv	Mexico	1
Avram I	Romania	2
Awika J	United States of America	1
Awika Jm	United States of America	1
Awussi Aa	France	1
Axelsson L	Norway	1
Ay M	Turkey	1
Ayala-zavala Jf	Mexico	2
Aydin O	Turkey	1
Ayimbila F	Thailand	1
Aymard P	France	1
Aynaou A-e	Netherlands	1
Aytekin Ö	Japan	1
Ayub Ka	China	1
Ayub Maz	United States of America	1
Azad Mb	United States of America	1
Azadbakht O	Iran	1
Azadi B	Canada	2
Azarpazhooh E	China	2
Azeredo J	Portugal	1
Azevedo L	Brazil	1
Aziz-kalbhenn H	Germany	1
Azizkhani M	Iran	1
Baarlen Pv	Netherlands	1
Babaahmadifooladi M	Brazil	1
Babji As	Malaysia	1
Baccolo G	Italy	1
Bach V	France	3
Badieenejad A	Iran	1
Bae H	Singapore	1

Bae Hj	South Korea	1
Bagci U	Turkey	1
Bagkar P	India	1
Bahrami B	United Kingdom	4
Bai J	China	1
Bai S	China	1
Bai W	China	1
Bai Y	China	1
Baines Sd	United Kingdom	13
Bajic D	Netherlands	1
Bajka B	Italy	1
Bajury Dm	Malaysia	1
Bakalis S	United Kingdom	3
Bake B	Sweden	1
Balakrishnan G	United States of America	1
Balance S	France	1
Balasooriya H	Australia	1
Balasuriya Gk	Sweden	1
Baldelli bombelli F	Italy	1
Baldwin Sa	Denmark	1
Baleeiro Fcf	Germany	1
Baliga Ns	United States of America	1
Ballance S	France	2
Ballet N	France	2
Ballyk Mm	United States of America	1
Balthazar Cf	Brazil	1
Baltzer S	Switzerland	1
Ban L	China	1
Bandara N	Australia	1
Bandsholm O	United States of America	1
Banerjee S	India	1
Banfield Jf	United States of America	1
Bannour M	Italy	1
Banz Y	Switzerland	1
Bao Q	China	1
Bao T	China	1
Bao X	China	1
Bao Y	China	1
Baran M	Belgium	1
Baranyi J	United Kingdom	1
Barber X	France	1
Barberá R	Spain	3
Barbieri E	United States of America	1
Bard J-m	France	1
Bardi Mj	Iran	1
Barer Mr	United Kingdom	1
Barker Sa	United Kingdom	2
Barmpalia-davis Im	United States of America	2
Barone M	Italy	1
Barra A	France	1
Barrera C	Brazil	1
Barretta C	Brazil	1
Barros L	Portugal	2
Barroso E	Belgium	4
Barry J-l	France	5
Barry Jl	France	1
Bartholomew M	United States of America	1
Bartholomew Mj	United States of America	1
Bartolomé B	Spain	10
Barton W	France	1
Bas-bellver C	Brazil	1
Basit Aw	United Kingdom	3
Bassères E	United States of America	1
Basson Nj	South Africa	1
Bast A	Netherlands	3

Basta N	United States of America	1
Basta Nt	United States of America	3
Batard E	France	1
Batista Clfm	Brazil	1
Batra Mr	United States of America	1
Battistini C	Brazil	2
Bauer L	United States of America	1
Bauer Ll	United States of America	2
Bauer R	Germany	1
Baumann S	Germany	1
Baumgartner S	Germany	1
Bautista J	Spain	1
Bayne T	United States of America	2
Bayne Tf	Belgium	1
Bazan Gc	United States of America	1
Bazzocco S	France	1
Beak Dg	United States of America	3
Beards E	United Kingdom	1
Beards Ej	United Kingdom	1
Bearne Ca	United Kingdom	1
Beaumont M	France	3
Becker Ab	United States of America	1
Beckman C	Netherlands	1
Bedani R	Brazil	4
Bednarcik Ja	United States of America	1
Beeck R	Germany	1
Beekmann K	Netherlands	1
Befus Ad	United States of America	1
Beggs Ad	United Kingdom	1
Begovic Jm	Serbia	1
Begum- haque S	United States of America	1
Behsnilian D	Germany	1
Bein A	United States of America	1
Beirnaert C	Belgium	1
Bekhit Ae-da	New Zealand	2
Belaïnoussi Y	France	1
Bélair V	Canada	1
Beldarrain-iznaga T	Cuba	1
Belik J	Canada	1
Bell A	United Kingdom	1
Bell Ca	Sweden	1
Bell N	New Zealand	1
Bellamine A	United States of America	1
Bellanger A	France	1
Belleville C	France	1
Bellmann S	Germany	2
Bello-pérez La	United States of America	1
Bellumori M	Italy	1
Beloshapka An	United States of America	1
Belzer C	Netherlands	6
Bemer P	France	1
Benamara M	United States of America	1
Bendali F	Canada	1
Benedé S	Brazil	1
Bengoa Aa	Spain	1
Bengtsson T	Sweden	1
Benítez-páez A	Spain	1
Bentley K	United Kingdom	2
Bentley We	United States of America	1
Bentley-hewitt Kl	New Zealand	3
Bently K	United Kingdom	1
Benton K	United States of America	1
Berchtold L	Netherlands	1
Berckmans P	Belgium	1
Berean Kj	United Kingdom	1

Beresford Saa	United States of America	1
Berezhnaya Y	Belgium	1
Bergenståhl B	Sweden	1
Berger A	United States of America	1
Berger D	Austria	1
Bergillos-meca T	Italy	1
Bergström Jh	Sweden	1
Berlinghof F	Germany	1
Bermúdez-humarán Lg	France	1
Bernaerts K	United States of America	1
Bernal-martínez Lr	Mexico	1
Bernalier A	France	1
Bernasconi Oj	Italy	1
Bernasconi S	France	1
Berner Az	Switzerland	1
Berni canani R	Italy	1
Berressem D	Germany	1
Bertelsen K	United States of America	1
Bertha C-t	Sweden	1
Bertkova I	United States of America	1
Bertoldo pacheco Mt	Brazil	1
Berton-carabin Cc	France	1
Bertram Hc	United States of America	2
Bertrand D	France	1
Bertrand E	France	1
Bes M	France	1
Betley J	United States of America	1
Betoret N	Brazil	1
Bettencourt A	Portugal	1
Bevilacqua A	Brazil	2
Beyssac E	France	1
Bezabih Y	France	1
Bhandari B	France	1
Bharadwaj S	United States of America	1
Bhat Hf	United Kingdom	1
Bhat Zf	United Kingdom	2
Bhattacharya K	United States of America	1
Bhushan B	United States of America	1
Bhushette Pr	India	1
Biagi E	Italy	1
Biagini F	Italy	1
Bianchi F	Brazil	6
Bianchi M	United Kingdom	1
Biasini B	Italy	1
Biavasco F	Italy	1
Bidabadi M	United Kingdom	1
Bijttebier S	Belgium	2
Bindelle J	Belgium	1
Binoti Ml	Brazil	1
Binsl TAIWAN	Netherlands	1
Birchenough Gmh	Sweden	1
Bircher L	Switzerland	2
Birringer M	Germany	1
Bisharat L	United Kingdom	1
Bisignano C	Italy	2
Biswas A	New Zealand	1
Bitounis D	United States of America	1
Bittinger K	United States of America	4
Biyani A	India	1
Bjornson Hs	United States of America	1
Blachier F	France	1
Blake Dp	United Kingdom	1
Blancas-benitez Fj	Mexico	2
Blanchfield Jt	Australia	1
Blanco-morales V	Spain	2

Blancquaert L	Belgium	1
Blanquet-diot S	France	15
Blasi F	Italy	1
Blatchford P	New Zealand	2
Blatchford Pa	United States of America	2
Blennow A	Denmark	2
Blijenberg B	Netherlands	2
Blondeel Ejm	Canada	1
Bloom Rj	United States of America	2
Blum Je	France	1
Boaro Aa	United States of America	1
Bobin-dubigeon C	France	2
Bobokalonov J	United States of America	1
Bocher Btw	United States of America	1
Bochi Vc	Brazil	1
Bock C	United States of America	1
Bodin O	Sweden	1
Boeckaert C	Belgium	1
Boedeker Ec	United States of America	1
Boekhorst J	United States of America	1
Boeri L	Italy	1
Bøgh Kl	France	1
Bogsan Csb	Italy	1
Bohn T	France	3
Bohnen Jma	United States of America	1
Boileau T	Belgium	1
Boileau TAIWAN	United States of America	1
Bokaei S	Iran	1
Bolca S	France	2
Boligon Aa	Brazil	1
Boll Ej	United States of America	1
Bollom Ma	United States of America	1
Bolten Cj	Switzerland	1
Bomhof E	Netherlands	1
Bondue P	Belgium	2
Bongaerts Rj	United Kingdom	1
Bongers Rs	Netherlands	2
Bonifácio - lopes T	France	1
Bonk F	Germany	1
Bonnarme P	France	1
Bonnet C	France	1
Boon N	Belgium	12
Boone Mn	Belgium	1
Boonrungsiman S	Thailand	1
Borde A	Switzerland	1
Bordenave N	Egypt	1
Bordonaro M	Netherlands	1
Bordoni A	Italy	1
Borén J	Sweden	1
Borenstein E	United States of America	1
Borges arcucio L	Brazil	1
Borges Cwp	Brazil	1
Borges F	France	1
Borgonovi Tf	Brazil	1
Borin S	Italy	1
Bornet F	France	1
Bornhorst Gm	United States of America	3
Bornstein Jc	Sweden	1
Borrel G	France	1
Borruso L	Italy	1
Borst W	Netherlands	1
Bortolaia V	Denmark	1
Bosscher D	United States of America	1
Botelho Rba	Brazil	1
Bothe Mk	Germany	1

Bottani M	Italy	1
Bottari B	Italy	1
Bottiglieri T	Canada	1
Boucinha L	France	1
Boudry C	Belgium	1
Bounsaythip C	Finland	1
Bourlieu C	France	5
Bourlieu-lacanal C	France	1
Bourlioux P	United States of America	1
Bourriaud C	France	1
Bours P	Netherlands	1
Bousarghin L	France	1
Boutrou R	France	2
Bouwmeester H	Netherlands	1
Bouzerzour K	France	1
Boxall Nj	Australia	1
Braber S	United States of America	1
Bracht A	Brazil	2
Bracke M	Belgium	1
Bradham K	United States of America	1
Braegger Cp	Switzerland	1
Brahma S	United States of America	1
Bralatei E	United Kingdom	1
Bramley Pm	United Kingdom	1
Brandão Lr	Brazil	1
Brandão Trs	France	2
Brandis A	Germany	1
Brasca M	Italy	1
Brauer M	United States of America	1
Bravo D	France	2
Bravo L	Spain	2
Bravo Ll	Spain	1
Brazeilles R	France	1
Breault Dt	United States of America	2
Brennan Cs	China	1
Brennan Ma	China	1
Brennan-craddock We	United Kingdom	1
Bresciani L	Italy	7
Breynaert A	Belgium	2
Briandet R	France	1
Briard-bion V	France	1
Brighenti F	Italy	2
Brighina S	Italy	1
Brígida Ais	Brazil	1
Brigidi P	Italy	5
Brignardello J	United Kingdom	1
Brindani N	United States of America	1
Brink Ev	Netherlands	1
Brinques Gb	United States of America	1
Britton Ra	United States of America	2
Briviba K	Germany	2
Brkljaca R	United Kingdom	1
Brochot C	France	1
Brochu M	Canada	1
Brodkorb A	France	5
Broekaert Wf	Belgium	2
Bronsoms S	Spain	1
Bronson Sm	United States of America	1
Brook Jr	United States of America	1
Brooks Spj	Canada	1
Brose F	Belgium	1
Broudiscou Af	France	2
Broudiscou L-p	France	2
Brouns F	United States of America	1
Brown Cj	United States of America	1

Brown Ct	United States of America	1
Brown Em	United Kingdom	1
Brück Wm	Denmark	1
Brugère J-f	France	4
Brugman S	Netherlands	1
Bruil Ma	Netherlands	1
Bruins Me	China	1
Bruins Mj	Netherlands	1
Bruneau A	France	3
Bruno C	Italy	1
Bruntha devi P	India	1
Bruyneel B	Netherlands	1
Bry L	United States of America	1
Bryant Ja	United Kingdom	1
Brynskov J	Denmark	1
Bubeck S	United States of America	1
Bubeck Ss	United States of America	1
Buchert J	Finland	3
Buchheim-schmidt S	Germany	1
Buck R	United States of America	1
Buckin V	Ireland	1
Buckley Am	United Kingdom	7
Budd K	United States of America	1
Budding Ae	Netherlands	2
Buettner A	Germany	1
Buniowska M	Spain	1
Bunzel M	Germany	1
Buoncompagni L	Italy	1
Burel A	France	1
Buret Ag	Canada	1
Burgain J	France	1
Burgos G	Canada	1
Burgos-edwards A	Chile	2
Burley J	United States of America	1
Burns P	Brazil	1
Burton Jp	United Kingdom	1
Busfield Jjc	Italy	1
Bussche Jv	Belgium	1
Bussolo de souza C	Netherlands	3
Butcher J	Canada	2
Butler Jm	United States of America	1
Butler M	United Kingdom	1
Butler Pj	United States of America	1
Cabellos J	Spain	1
Cabral Jms	United States of America	1
Cabral Lmc	France	1
Cacabelos N	Spain	1
Cacabelos R	Spain	1
Cacciola F	Italy	1
Cáceres-jiménez S	Spain	1
Cacopardo L	Italy	1
Caggia C	Italy	1
Cahú Tb	Denmark	1
Cahyanto Mn	Indonesia	1
Cai C	United Kingdom	1
Cai D	China	2
Cai M	China	1
Cai S	China	2
Cai W	China	1
Cai X	China	11
Cai X-l	China	1
Cai Y	United States of America	2
Cakmak I	Turkey	1
Calamari El	United States of America	1
Calani L	Italy	5

Calanni F	Italy	1
Calatayud arroyo M	Spain	3
Calatayud M	Belgium	7
Calero-diaz G	Spain	1
Calhelha Rc	Brazil	1
Callewaert C	Belgium	1
Callister Sj	United States of America	1
Calvert G	Australia	1
Calvigioni M	Italy	1
Calvo Mi	Spain	1
Camacho Dm	United States of America	1
Camacho-díaz Bh	Mexico	1
Camara Ma	Spain	1
Camel V	France	1
Camp Pjm	Netherlands	1
Campagnol Pcb	Brazil	1
Campanella Oh	United States of America	1
Campbell D	Belgium	2
Campos Da	France	1
Campos-madueno Ei	Italy	1
Campos-vega R	Mexico	6
Camprini L	Italy	1
Canadian healthy infant longitudinal development s	United States of America	1
Candela M	Italy	1
Canelli G	Switzerland	1
Cannie I	France	1
Cano Mp	Spain	1
Cano-sampedro E	Mexico	1
Cantón R	Spain	1
Cao H-q	China	2
Cao J	China	3
Cao M	China	1
Cao W	China	1
Cao Y	China	1
Capanoglu E	Italy	1
Capocaccia L	Italy	1
Capozzi F	France	1
Cappitelli F	Italy	1
Capuano E	China	3
Carafa I	Spain	1
Carasi P	Spain	2
Carattoli A	Italy	1
Carbonell-capella Jm	Spain	1
Carbonero F	United States of America	1
Carbonero-aguilar P	Spain	1
Cardador A	Mexico	1
Cardarelli Hr	Netherlands	1
Cardemil L	Chile	1
Cárdenas-castro Ap	Mexico	3
Cardone G	Italy	1
Cardoso Bb	Portugal	2
Cardoso Pg	Brazil	1
Cardot J-m	France	3
Carlos da silva júnior E	Brazil	1
Carlsson N-g	Sweden	1
Carlucci C	United Kingdom	1
Carman Rj	United States of America	4
Carmona M	Spain	1
Carnero Ea	United States of America	1
Carpenter D	United States of America	1
Carpi F	Italy	2
Carpine R	Switzerland	1
Carracci F	United States of America	1
Carrasco-pozo C	France	1
Carrascosa Av	Spain	1

Carrera I	Spain	1
Carrier Rl	United States of America	1
Carrière F	France	5
Carriero C	United Kingdom	1
Carrillo W	Spain	1
Carvajal Ri	Chile	1
Carvalho M	France	1
Casal S	Portugal	1
Casarotti Sn	Brazil	2
Casciano F	Italy	2
Casey Pg	Ireland	1
Cash H	United States of America	2
Casiraghi Mc	Italy	2
Castaldo L	Italy	3
Castaño-tostado E	Mexico	1
Castellani C	Germany	1
Castellano Lrc	Brazil	1
Castellano P	Argentina	1
Castillo Mj	United States of America	1
Catalán U	Spain	1
Catharino Rr	Brazil	1
Cattaneo S	Italy	1
Cattenoz T	France	3
Cattò C	Italy	1
Cava R	Spain	1
Cavalcanti Mt	Brazil	1
Cavallo N	Italy	1
Cave M	Netherlands	1
Cavero Ry	Spain	1
Cavina P	Italy	1
Çavus O	China	1
Cebrián R	Spain	1
Cecchi L	Italy	1
Cedraro N	Italy	1
Ceglarek U	United States of America	2
Cei D	Italy	1
Cela D	United Kingdom	1
Celandroni F	Italy	1
Celep E	Turkey	1
Cen S	China	1
Centanni M	Italy	2
Centler F	Germany	1
Cérbulo-vázquez A	Mexico	1
Ceri H	Canada	1
Cermeño S	Spain	1
Cerniglia Ce	United States of America	1
Ceuppens S	Belgium	1
Cha Jw	South Korea	1
Cha Kh	South Korea	2
Chaboub L	United States of America	1
Chadwick R	United States of America	1
Chae S-j	South Korea	1
Chagas barros Rg	Brazil	2
Chai B	United States of America	1
Chai J	China	1
Chai Y	China	1
Chaikham P	Thailand	10
Chaiongkarn A	Thailand	1
Chait Ya	Canada	1
Chaiwong T	Thailand	1
Chalancon S	France	7
Chambers M	United Kingdom	1
Chamignon C	France	1
Champ M	France	1
Champagne C	United States of America	1

Champagne Cp	Canada	1
Chan Hm	United States of America	1
Chandran matheyambath A	Canada	2
Chang B	Australia	1
Chang D	Israel	2
Chang Mw	United States of America	1
Chang Sw	Kuwait	1
Chang X	China	1
Chang Y-s	United States of America	1
Chang Yb	South Korea	1
Chang Yh	South Korea	2
Chang-graham Al	United States of America	1
Changrue V	Thailand	1
Chanket W	Thailand	1
Chanyi Rm	United Kingdom	1
Charalampopoulos D	United Kingdom	2
Charbonneau Mr	United States of America	1
Chardon K	France	1
Charehsaz M	Turkey	1
Charleer L	Belgium	1
Charnchai P	Thailand	1
Chassaing B	United States of America	1
Chassard C	Switzerland	13
Chatanon L	Thailand	1
Chatterjee R	United States of America	1
Chaucheyras-durand F	France	2
Chauhan C	United States of America	1
Chaurasia Lk	India	1
Chaves Acsd	Brazil	1
Chaves Av	Brazil	1
Cheah E	Australia	1
Chen B	China	1
Chen C	China	5
Chen D	China	2
Chen D-w	United States of America	1
Chen E	United States of America	1
Chen F	China	6
Chen G	China	1
Chen H	China	10
Chen H-c	Taiwan	1
Chen J	China	7
Chen L	China	7
Chen M	China	1
Chen M-h	United States of America	1
Chen M-j	Taiwan	1
Chen P	China	2
Chen Q	China	1
Chen S	China	4
Chen T	China	3
Chen V	United States of America	1
Chen W	China	6
Chen X	China	11
Chen Xd	China	3
Chen Y	China	7
Chen Ya	United Kingdom	1
Chen Yf	China	1
Chen Z	China	3
Chen Z-j	China	1
Cheng D	China	1
Cheng G	China	2
Cheng K	China	2
Cheng K-c	Taiwan	1
Cheng K-w	China	2
Cheng Ky	Australia	1
Cheng L	China	2

Cheng M	China	1
Cheng P	China	2
Cheng S	China	1
Cheng W-y	China	1
Cheng X	China	1
Cherbut C	France	4
Cherbuy C	France	1
Chernetskyy M	France	1
Cherr Gn	United States of America	1
Chesnel L	United Kingdom	1
Chessa S	United Kingdom	2
Chew B	United States of America	1
Cheynier V	France	1
Chi H	United Kingdom	1
Chi J	China	1
Chi L-l	Taiwan	1
Chi-keung cheung P	China	1
Chia Lw	Netherlands	2
Chiang C-k	Canada	1
Chiang M-l	Taiwan	1
Chiarello E	Italy	1
Chiba A	United States of America	1
Chicault C	France	1
Chichger H	United Kingdom	2
Child Mw	United Kingdom	2
Chilton Ch	United Kingdom	18
Chin J	United Kingdom	1
Chiodelli G	Italy	2
Chirikova Nk	Russian Federation	1
Chiu C-a	United States of America	1
Chiu Pc	United States of America	1
Chiu Y-h	Taiwan	1
Chmiel Ja	United Kingdom	1
Chmiela M	Poland	1
Cho D	South Korea	1
Cho K	Singapore	1
Cho K-h	South Korea	1
Cho M	United States of America	1
Cho Mg	South Korea	1
Cho S-j	South Korea	1
Cho Sk	South Korea	1
Choe J-s	South Korea	1
Choi A-j	South Korea	1
Choi E-j	South Korea	1
Choi Hs	South Korea	2
Choi I	South Korea	1
Choi S-h	France	1
Choi Sh	United States of America	1
Choi Y-s	South Korea	2
Chojnacka K	Poland	1
Chokiatirote E	Thailand	1
Chonchúir Fn	France	1
Chotiko A	United States of America	1
Chourasia Mk	Japan	1
Chow J	United States of America	1
Chrisp Md	Australia	1
Christakopoulos P	United Kingdom	1
Christgen B	China	1
Christides T	United Kingdom	1
Chroszcz A	Poland	1
Chu Y	China	2
Chung Ks	South Korea	1
Chung Wsf	United States of America	1
Chung Y	Belgium	1
Churilov L	Sweden	1

Chusak C	Thailand	1
Ciampaglia R	Italy	1
Cianci R	Italy	1
Cichocki N	United Kingdom	2
Cichoski Aj	Brazil	1
Cichy K	United States of America	1
Cid C	Spain	4
Cieplak T	Denmark	3
Cieslinska A	Poland	1
Cifuentes A	Spain	1
Cilla A	Spain	2
Cinquin C	Canada	4
Cioffi S	Italy	1
Cires Mj	France	1
Citterio B	Italy	1
Claerbout A-s	Spain	1
Claeys B	Belgium	1
Claeys M	Belgium	1
Clark E	United Kingdom	6
Clark Ev	United Kingdom	1
Clark S	United States of America	1
Clarke Sr	United Kingdom	2
Clarke Tb	United Kingdom	1
Claus G	Austria	1
Clauss M	Japan	1
Clear Kyj	United States of America	1
Clemente A	France	2
Cleusix V	Switzerland	1
Cloarec D	France	1
Clooney Ag	France	1
Cocero Mj	Spain	1
Cochrane K	Canada	2
Codoñer-franch P	Spain	1
Coia Hg	United States of America	1
Colantuono A	United States of America	1
Colás-medà P	Spain	1
Cole J	United States of America	1
Coleman N	United Kingdom	1
Coles Lt	New Zealand	1
Collado Mc	Spain	2
Collins C	United Kingdom	1
Collins M	United Kingdom	1
Colque-navarro P	Spain	1
Colt M	United States of America	1
Coman Mm	Italy	1
Comas Jc	France	1
Combet E	United Kingdom	2
Comi I	Norway	3
Commane Dm	Italy	2
Comtet-marre S	France	1
Conchillo J	Netherlands	1
Conde-moreno E	Spain	1
Condette Cj	France	1
Connolly Ml	United Kingdom	1
Connolly Pc	United States of America	1
Conroy Bf	United States of America	1
Consolandi C	Italy	1
Conte A	Italy	1
Conterno L	United Kingdom	1
Conti F	Italy	1
Converti A	Italy	1
Conway POLAND	Sweden	1
Cook Kl	United States of America	1
Cooney Jm	New Zealand	1
Cooper Jm	United Kingdom	2

Coppola R	Italy	1
Corbin Kd	United States of America	1
Corbo Mr	Italy	1
Cordonnier C	France	2
Corich V	Italy	1
Cornelis C	Netherlands	1
Corno G	Italy	1
Corollaro Ml	Italy	1
Corona G	Italy	1
Corpe Cp	United Kingdom	1
Corpet De	France	1
Corradi M	Italy	1
Corrêa Rcg	Brazil	2
Correa Vg	Brazil	3
Correa-betanzo J	Canada	1
Corredig M	Norway	4
Corriveau Mc	United Kingdom	1
Corrochano Ar	Ireland	1
Corstens Mn	France	1
Cortés C	Spain	1
Cortes-gallardo Jp	Mexico	1
Cortes-limon Am	Mexico	1
Corti A	Italy	1
Coscueta Er	France	1
Cossu M	Italy	1
Costa antunes Ae	Brazil	1
Costa Cm	France	1
Costa Cmdsf	France	1
Costa E	France	1
Costa J	Italy	3
Costa Jr	France	1
Costa Mgm	Brazil	1
Costa Mm	Brazil	1
Costabile A	United Kingdom	12
Costabile Af	United Kingdom	1
Costamagna G	Italy	1
Costello Cm	United States of America	1
Cotta Ma	United States of America	1
Cotter Pd	Ireland	3
Coulier L	Netherlands	1
Courau S	Germany	1
Courtin Cm	Belgium	4
Coussa-charley M	Canada	2
Couto Pt	Brazil	1
Coutte F	France	1
Coutts Tm	United Kingdom	1
Cozzolino A	Italy	1
Craig Dqm	United Kingdom	2
Crawford Se	United States of America	1
Creason J	United States of America	1
Creed Jt	United States of America	1
Cresci A	Italy	1
Crespo J	Spain	1
Crèvecoeur S	Belgium	2
Crippen Tl	United Kingdom	1
Crispie F	Ireland	2
Cristina de almeida bianchini campos R	Brazil	1
Cristofori F	Germany	1
Crittenden An	Germany	1
Cronce Mj	United States of America	1
Crook Dw	United Kingdom	2
Crooke Ps	United States of America	1
Croubels S	Austria	1
Crowther Gs	United Kingdom	15
Crozier A	United Kingdom	3

Cruz Ag	Brazil	2
Cruz Jc	United States of America	1
Cruz R	Portugal	1
Cruz rubio Jm	Netherlands	1
Cruz-diaz N	United States of America	1
Cruz-ortiz R	Mexico	1
Cseresnyes Z	Germany	1
Cudennec B	France	1
Cueva C	Spain	9
Cuevas-rodríguez Eo	United Kingdom	1
Cuevas-tena M	Spain	2
Cui J	China	2
Cui K	China	2
Cui S	China	2
Cui W	China	1
Cui Y	China	14
Cui Y-s	China	1
Cumming Drs	United Kingdom	1
Cummings Jh	United Kingdom	3
Cummins Ck	United States of America	1
Cunha Sc	Portugal	1
Cyr M	United States of America	1
Czaczyk K	Poland	1
Czarnocki Z	Poland	1
Czerski A	Poland	1
D'alessandro M	Italy	1
D'amico S	Austria	1
D'arcy Br	Australia	2
D'auria G	Spain	1
D'egidio Mg	Italy	1
da conceição Ml	Brazil	2
da cruz almeida Ét	Brazil	1
da silva Bp	Brazil	1
da silva campelo borges G	Brazil	1
da silva Dc	Brazil	1
da silva duarte V	Italy	1
da silva Ghr	Brazil	1
da silva Ma	Brazil	2
da silva vieira Ar	Brazil	1
Dabour N	Egypt	1
Daep C	United States of America	1
Daghio M	Italy	1
Daguet D	Belgium	3
Dai H	China	1
Dai M	China	3
Dai R	China	1
Daigneault M	United Kingdom	1
Daigneault Mc	Canada	2
Dainty Jr	Italy	1
Daisley Ba	United Kingdom	1
Dal bello F	Italy	1
Daley D	United States of America	1
Daliu P	Italy	1
Dall'asta M	Italy	4
Dall'asta M	Italy	2
Dalley D	United Kingdom	1
Dalsgaard A	Denmark	1
Daly Km	France	1
Damiano C	Italy	1
Dan T	China	1
Dang Z	China	1
Danhof Ha	United States of America	1
Daniel R	Germany	1
Daniel S	United States of America	1
Dantas duarte menezes Fn	Brazil	1

Darby A	United Kingdom	1
Darragh Aj	New Zealand	1
Dary A	France	1
Das R	China	2
Dasanayake K	Australia	1
Dastych J	Canada	1
Dathong J	Thailand	1
Daube G	Belgium	3
Daud Na	Malaysia	1
Dave Jm	India	1
David La	United States of America	2
David-birman T	Israel	1
Davies K	United Kingdom	2
Davis G	United Kingdom	3
Davis S	United States of America	1
Davis T	United States of America	1
Dawczynski C	Germany	2
Dayton Ea	United States of America	1
de albuquerque Tmr	Brazil	3
de alencar Er	Brazil	1
de ancos B	Spain	1
de andrade arruda fernandes I	Brazil	1
De angelis E	Italy	1
De angelis M	Italy	2
De antoni Gl	France	1
de araújo Ff	Brazil	1
De baere S	Austria	1
de barcelos Sc	Brazil	1
de barros ranke Ff	Brazil	1
De beer T	Belgium	2
De bellis P	Italy	1
De blaiser A	Belgium	1
De bodt J	Belgium	4
De boever P	Belgium	4
de bona da silva C	Brazil	1
De bruijn Wjc	China	1
de carla bassetto M	Brazil	1
de carvalho fino L	Brazil	1
De carvalho Kg	Brazil	1
De carvalho Nm	France	2
de castilhos J	Italy	1
De chavez P	Belgium	1
De chavez Pj	France	1
de cosío-barrón Acg	Mexico	1
de figueiredo Fc	Brazil	1
de figueiredo P	United States of America	1
De gobba C	Denmark	1
de godoy Mrc	United States of America	1
De graaf Aa	Netherlands	4
de gunzburg J	France	1
De haan Bj	Netherlands	1
De haan P	Netherlands	1
De jong A	Netherlands	2
De keukeleire D	Belgium	2
de la fuente E	United Kingdom	1
de las heras A	Mexico	1
de lima brito I	Brazil	1
de lima Lc	Brazil	1
de lima zollner R	Brazil	1
de llano Dg	Spain	1
de los reyes-gavilán Cg	Spain	5
De marchi L	Italy	1
De maria C	Italy	1
De martinis Ecp	Netherlands	3
de matos Fe	Brazil	1

de matta Vm	Brazil	1
de medeiros Ll	Brazil	1
de mello tieghi T	Denmark	1
De melo Anf	United States of America	1
de melo franco Bdg	Netherlands	1
de menezes Cr	Brazil	1
de moraes flores Em	Brazil	1
De mulder T	Belgium	1
De NORWAYni I	Italy	1
de oliva-neto P	Brazil	1
de oliveira Cs	Brazil	1
de oliveira Meg	Brazil	1
De oliveira Sc	France	2
de oliveira Spa	Brazil	1
De paepe E	Belgium	1
De paepe K	Belgium	9
De pascale S	Italy	1
De pascual-teresa S	Spain	1
de paulo farias D	Brazil	1
de peña M-p	Spain	4
De prisco A	Italy	1
De ryck T	Belgium	2
de sá-nakanishi Ab	Brazil	1
De sales Cv	United States of America	1
de santana Ehw	Brazil	1
De santiago E	Spain	2
De smet I	Belgium	3
De smet S	Belgium	1
de sousa galvão M	Brazil	1
de souza aquino J	Brazil	1
de souza Cb	Brazil	1
de souza Cfm	United States of America	1
de souza Cgm	Brazil	1
De souza Chb	Brazil	2
de souza El	Brazil	7
de souza lago H	Italy	1
de souza leite M	United States of America	1
de souza oliveira Rp	Italy	1
De souza Rj	United States of America	1
de valdez Gf	Brazil	1
de vos M	Belgium	2
De vos P	Netherlands	1
De vos R	Netherlands	1
De vos Rc	Netherlands	1
De vos Wm	Netherlands	10
de vrese M	Germany	1
de vries S	Netherlands	1
De vuyst L	Belgium	3
De waard P	Netherlands	3
De weirdt R	Belgium	4
de wiele Tv	Belgium	4
De wouters T	Netherlands	1
De zwart Ll	Netherlands	1
Deane Sm	South Africa	1
Decker Ea	United States of America	1
Decroos K	Belgium	5
Degirmencioglu N	Turkey	1
Deglaire A	France	3
Degnan Ba	United Kingdom	1
Dehghani F	United States of America	1
Dekel A	Israel	1
del campo R	Spain	1
Del juncal-guzmán D	Mexico	1
Del rio D	Italy	8
del toro Gv	Mexico	1

Delafiori J	Brazil	1
Delanaud S	France	3
Delaney Ml	United States of America	1
Delcenserie V	Belgium	5
Delcour Ja	Belgium	2
Delgado-andrade C	France	1
Delgenès J-p	France	1
Deliloglu gürhan Si	Japan	1
Dell S	United States of America	1
Dellarosa N	France	1
Dellinger Ep	United States of America	1
Delmas D	France	1
Delon Lc	Australia	1
Delort-laval J	France	1
Delpivo C	Spain	1
Demarqui Fm	United States of America	1
Demirbas F	Turkey	1
Demirci M	Turkey	1
Demma Z	United States of America	1
Demokritou P	United States of America	1
Demuth T	Switzerland	1
Denburg Ja	United States of America	1
Deng B	China	1
Deng H	China	1
Deng J	China	1
Deng K	China	1
Deng Q	China	1
Deng R	China	2
Deng Y	China	1
Deng Z	China	3
Dengre R	Japan	1
Denis S	France	16
Denkov Nd	Netherlands	1
Depaola A	United States of America	1
Depeint F	France	2
Deplancke B	United States of America	1
Deracinois B	France	1
Derave W	Belgium	1
Derde M	Belgium	2
Derks Tgj	United States of America	1
Derrien M	France	4
Dertli E	Turkey	2
Derycke L	Belgium	1
Desai Ms	United States of America	1
Deschamps C	France	1
Deschamps J	France	1
Desideri D	Italy	1
Desmet I	France	1
Desobry S	France	1
Desouza A	United States of America	1
Deutschmann O	Germany	1
Deutz Nep	Netherlands	2
Devaux M-f	France	1
Devesa V	United Kingdom	1
Devriese S	Belgium	1
Dewar M	United Kingdom	1
Dezutter O	Netherlands	1
Dheer R	United States of America	1
Dhivya S	India	1
Dhooge W	France	1
Dhulster P	France	1
Di cagno R	Germany	1
Di cesare A	Italy	1
Di natale C	France	1
Di nunzio M	Italy	1

Di patria V	Italy	1
Di pierro F	France	1
Di silvio D	Italy	1
Di T	China	1
Diana C-r	Mexico	1
Dianawati D	China	1
Dias Mi	Portugal	2
Dias-audibert Fl	Brazil	1
Diaz-bone Ra	Germany	1
Dibenedetto N	United States of America	1
Dicks Lmt	South Africa	1
Dickson M	United States of America	1
Didelez M	Belgium	1
Didelot X	United Kingdom	1
Dien Bs	United States of America	1
Díez-municio M	United Kingdom	1
Diez-sánchez E	Spain	1
Dijkema C	Netherlands	2
Dijkstra Jw	United Kingdom	1
Dilger Rn	United States of America	1
Dimaano L	United States of America	1
Dimitrov Mr	Brazil	1
Dinan Tg	France	1
Ding J	China	3
Ding S	China	2
Ding T	China	1
Ding W-p	China	1
Ding X	China	1
Dingle Ke	United Kingdom	1
Diniz-silva Ht	Brazil	1
Dion M	France	1
Dionisi F	Switzerland	1
Diotallevi C	Israel	2
Dirks B	United States of America	3
Dirson E	France	1
Dixit Y	India	1
do carmo Mav	Brazil	1
do egito As	Brazil	1
Dobbe Cjg	Netherlands	1
Dobrindt U	Germany	1
Dobson A	Ireland	2
Dodd M	Canada	1
Dodi R	Italy	1
Dodoo C	United States of America	1
Dogan K	Turkey	1
Dolinsky Vw	United States of America	1
Dolly P	India	1
Dolores álvarez M	Spain	1
Domenici C	Italy	2
Domínguez-avila Ja	Mexico	1
Domínguez-fernández M	Spain	1
Dominguez-lopez A	Mexico	1
Donà V	Italy	1
Dong H	China	1
Dong J	China	1
Dong K	China	1
Dong L	China	4
Dong M	China	4
Dong R	China	2
Dong W	China	2
Dong Y	China	1
Donnelly Dj	Canada	2
Donohue Tj	United States of America	2
Donovan Jd	United States of America	1
Doo E-h	France	1

Doré J	France	3
Dos santos Cs	Brazil	1
dos santos Kmo	Brazil	2
dos santos lima M	Brazil	2
dos santos Tf	Brazil	1
Dostal A	Switzerland	3
Dou Z	China	1
Douny C	Belgium	2
Dowd Se	United States of America	1
Drago Sr	Argentina	1
Draijer R	Netherlands	1
Draper La	France	1
Drewes Je	United States of America	1
Dreyer L	South Africa	1
Drider D	France	1
Drozdzynska A	Poland	1
Du F	China	1
Du H	China	13
Du H-l	China	1
Du J	China	1
Du L	China	2
Du laing G	China	6
Du M	China	1
Du P	China	1
Du X	China	1
Du Y	China	1
Duan H	China	1
Duan M	China	1
Duan S	China	1
Duan X	China	1
Duan Y	China	1
Duarte martino Hs	Brazil	1
Duarte Wf	Brazil	1
Dubey Ak	India	1
Duda-chodak A	Poland	1
Dudefoi W	France	1
Dudeja Pk	United States of America	1
Dueñas M	Spain	1
Duerden Bi	United Kingdom	1
Dufour C	United States of America	2
Dufourny S	Belgium	2
Dugan Mer	Brazil	1
Dugo P	Italy	1
Duijsens D	Belgium	1
Duncan He	United States of America	1
Duncan Sh	United States of America	3
Dunn Sr	United States of America	1
Dunngalvin G	France	1
Dunshea FRANCE	United Kingdom	1
Duodu Kg	South Africa	3
Dupont D	France	10
Dupuy B	United States of America	1
Duque Alrf	Brazil	2
Durán-páramo E	Mexico	1
Durand Hk	United States of America	2
Duranti S	France	1
Durkee S	United States of America	1
Dutilh Be	United States of America	1
Dutta D	India	1
Duysburgh C	Belgium	10
Dykes L	United States of America	2
Dziedzic K	Poland	2
Eberl Hj	Canada	3
Ebrahimi A	Iran	1
Ebrahimi Aa	Iran	1

Eck A	Netherlands	2
Eckert Em	Italy	1
Edberg Sc	United States of America	1
Ede Jd	United States of America	1
Ede-cintesun E	Turkey	1
Edwards C	France	1
Edwards Ca	United Kingdom	5
Edwards Js	United States of America	1
Edwards V	Switzerland	1
Eeckhaut E	Belgium	2
Eeckhaut V	Belgium	2
Egert M	Netherlands	2
Egger L	France	6
Eggert Ls	United States of America	1
Eglmeier J	Germany	1
Ehrampoush Mh	Iran	1
Eijkel Jct	Netherlands	1
Einerhand Aw	United Kingdom	1
Eisner P	Germany	1
Ekbatan Ss	Canada	2
El hage R	Belgium	2
El hage Ra	Belgium	1
El oufir L	France	1
El Sn	Turkey	1
Elamin E	Netherlands	1
Elie A-m	France	1
Elliott S	United States of America	1
Ellis M	Sweden	1
Emanuelli T	Brazil	1
Emerson R	United States of America	1
Emily M	France	1
Emin Ma	Germany	1
Emmanuel A	South Korea	1
Endimiani A	Italy	1
Endo A	Spain	1
Endres Bt	United States of America	1
Eng A	United States of America	1
Eng F	Brazil	1
Eng G	United States of America	1
Engel J	Germany	1
Engelke Uf	United States of America	1
Engelmann B	Germany	4
Engevik Ac	United States of America	2
Engevik Ka	United States of America	1
Engevik Ma	United States of America	2
Ennahar S	France	1
Enuwosa E	United Kingdom	2
Epstein Mm	France	1
Erba D	Italy	1
Erbakan M	United States of America	1
Ergin F	Turkey	1
Erlandsen Sl	United States of America	1
Ermund A	Sweden	1
Escuriet R	Spain	1
Espín Jc	Belgium	1
Espinal-ruiz M	United States of America	1
Espinosa L	Germany	1
Espinoza-serrano D	Mexico	1
Esteban-fernández A	Spain	3
Esteban-muñoz A	Spain	1
Estes M	United States of America	1
Estes Mk	United States of America	1
Esteve Mj	Spain	1
Esteves pintado Mm	Brazil	1
Estévez Am	Chile	1

Estévez-santiago R	Spain	1
Estill M	United States of America	1
Estrada-parra S	Mexico	1
Etienne-mesmin L	France	4
Evans A	Netherlands	1
Evans B	United States of America	1
Everaert N	Belgium	5
Ewin D	United Kingdom	5
Ewin Dj	United Kingdom	1
Exarchou V	Belgium	1
Eyre Dw	United Kingdom	1
Faccini A	Australia	1
Fachi Mm	Brazil	1
Fadden K	United States of America	1
Fadel Cw	United States of America	1
Fadhilah J	Malaysia	1
Fahey Gc	United States of America	3
Fahey jr Gc	United States of America	1
Failla Ml	United States of America	1
Fakhoury M	Canada	2
Fallico B	Italy	1
Falony G	Belgium	2
Faloye Of	Brazil	1
Fan B	China	1
Fan C	China	1
Fan L	China	1
Fan M	China	1
Fan T	China	1
Fan X	China	1
Fan Y	China	1
Fang D	China	1
Fang L	China	1
Fang Lc	China	1
Fang W	China	1
Fang Y	China	1
Fang Z	Australia	2
Fardet A	France	1
Farmer S	Netherlands	5
Farnworth Er	Canada	1
Farràs M	Spain	1
Faruk mizrak Ö	Turkey	1
Fässler C	United States of America	1
Fatmawati Nnd	Japan	1
Faulds C	Finland	1
Faulds Cb	France	1
Faulks Rm	United Kingdom	6
Fava F	Israel	2
Favari C	Italy	2
Fawley Wn	United Kingdom	1
Faye T	Norway	1
Feduzi L	Italy	1
Fehlbaum S	Switzerland	5
Feldmann J	United Kingdom	1
Felice Vd	France	1
Felis Ge	Italy	1
Fellah B	Italy	1
Feng Q	China	1
Feng R-p	China	1
Feng X	China	4
Feng X-w	China	1
Fenlon Dr	United Kingdom	1
Ferat-osorio E	Mexico	1
Ferchichi A	Italy	1
Fergelot P	France	1
Ferguson Sa	New Zealand	1

Feria-gervasio D	France	3
Fernandes garcia E	Brazil	1
Fernandes lemos júnior Wj	Italy	1
Fernandes Tv	Brazil	1
Fernández Ah	United States of America	1
Fernandez B	Canada	3
Fernández de palencia P	France	1
Fernández H	United States of America	1
Fernández M	France	1
Fernández N	Spain	1
Fernández-de córdova Ml	Spain	1
Fernandez-gutierrez Mm	Netherlands	1
Fernández-jalao I	Spain	2
Fernández-lópez J	Spain	5
Fernández-miyakawa M	Spain	1
Fernández-poyatos Mp	Spain	1
Fernández-romero A	Spain	1
Fernández-rosas E	Spain	1
Fernández-tomé S	France	1
Feron G	France	1
Feroz H	United States of America	1
Ferracane R	Italy	2
Ferranti P	Italy	2
Ferraretto A	Italy	1
Ferrari V	Italy	1
Ferreira dutra corrêa M	Italy	1
Ferreira Icfr	Portugal	2
Ferreira madureira Arm	Brazil	1
Ferreira Msl	France	1
Ferreira V	France	1
Ferreira-lazarte A	Spain	2
Ferrer Md	Spain	2
Ferrizzi D	United States of America	1
Ferruzzi Mg	United States of America	1
Fidaleo M	Italy	1
Fiedorowicz E	Poland	1
Fievez V	Belgium	1
Figeys D	Canada	3
Figge Mt	Germany	1
Figueroa Lm	Mexico	1
Figuerola F	Chile	1
Finch T	United States of America	1
Fioravante guerra A	Italy	2
Firkins Jl	United States of America	1
Firmino L	Italy	1
Firrman J	United States of America	4
Fishbain S	United Kingdom	1
Flahaut C	France	1
Flanagan A	United States of America	1
Flanagan Bm	Australia	1
Flanet raj Sr	Kuwait	1
Fledderus J	Netherlands	1
Fleige L	Belgium	2
Flint Hj	United States of America	3
Fliss I	Canada	8
Flores G	United Kingdom	1
Flórez Ab	Netherlands	1
Florez-rojas Js	United States of America	1
Flourié B	France	1
Flügel M	Germany	1
Flynn C	France	1
Fogliano V	Italy	6
Fois Cam	United States of America	1
Foligné B	France	1
Folkerts G	United States of America	1

Follador R	Switzerland	1
Fölster-holst R	Germany	1
Foltz M	Belgium	1
Fonseca Bds	Brazil	1
Fonseca Vr	Brazil	1
Fontaneto D	Italy	1
Fonteles Tv	Brazil	1
Fonty G	France	1
Forano E	France	2
Ford Cb	United Kingdom	1
Forney Lj	United States of America	1
Forssten S	United States of America	1
Forssten Sd	United States of America	3
Fortney Nw	Brazil	1
Fortunato G	Italy	1
Fotschki B	Poland	1
Fotschki J	Poland	1
Fouassier E	United States of America	1
Foubert K	Belgium	2
Fouhy F	Ireland	1
Fourmestraux C	France	3
Fournier E	France	1
Fowler K	United States of America	1
Frachet A	United States of America	1
Francavilla R	Italy	2
Francinelli V	Italy	1
Francino Mp	Spain	3
François O	France	1
Frandsen Hl	Denmark	1
Franks Ae	Sweden	1
Franz C	France	1
Franzetti A	Italy	1
Frediani G	Italy	1
Freeman J	United Kingdom	18
Freilich S	Israel	1
Freire Fc	Brazil	1
Freitas Mq	Brazil	1
Freitas Sp	Brazil	1
Frémont M	France	1
Friedler E	Israel	1
Friedman Ea	United States of America	1
Friedman SPAIN	United States of America	1
Frígola A	Spain	1
Fritz Jv	United States of America	1
Fritz-wallace K	Germany	3
Froidmont E	Belgium	1
Frontela-saseta C	Spain	1
Frutos Mj	Spain	2
Fryer Pj	United Kingdom	3
Fu D	China	1
Fu J	China	1
Fu M-x	China	1
Fu P	China	1
Fu X	China	3
Fu Y	China	7
Fu Y-y	China	1
Fuchs P	Germany	1
Fuentealba C	Chile	1
Fuentes S	Canada	3
Fuentes-aguilar Rq	Mexico	1
Fujimoto J	France	1
Fukuda I	Japan	1
Fukuma N	Japan	1
Fukushima M	Japan	2
Funahashi T	Japan	1

Furtado Dn	Brazil	1
Furtado martins Em	Brazil	1
Gaber M	United States of America	1
Gaboriau F	France	1
Gaci N	France	3
Gadermaier G	France	1
Gadimli Ai	Russian Federation	1
Gadonna-widehem P	France	1
Gagnon M	Switzerland	2
Gahler Rj	Netherlands	1
Gaiani C	France	1
Gaigg B	Germany	1
Gaikwad V	United Kingdom	1
Gaillez J	Belgium	1
Gaisawat Mb	Canada	3
Gaisford S	United States of America	2
Gajewski A	Poland	1
Galia W	France	5
Galibert M-d	France	1
Gall Gl	China	1
Gall Sd-l	France	1
Gallawa Cm	United States of America	1
Gallier S	New Zealand	1
Gallo A	Italy	1
Gallo V	Italy	1
Galmiche Jp	France	1
Gamlath S	Australia	1
Gan R-y	China	4
Ganan M	Spain	1
Gandarillas M	Chile	1
Gandomi H	Iran	1
Ganis Jc	United Kingdom	1
Gantner N	United States of America	1
Gao B	China	1
Gao J	China	5
Gao L	United States of America	2
Gao M	China	1
Gao M-j	China	2
Gao S	China	1
Gao Y	China	1
Gao Z	China	1
Gapp Em	Chile	1
Garcia Al	United Kingdom	1
Garcia C	France	1
Garcia Ef	Brazil	1
Garcia Hs	Mexico	1
Garcia S	United Kingdom	1
García-almendarez B	Mexico	1
García-bermejo L	Spain	1
García-gamboa R	Mexico	1
García-gasca Mt	Mexico	1
García-gonzález A	Mexico	1
Garcia-llatas G	Spain	3
García-mantrana I	Spain	1
García-rodríguez A	Spain	3
García-romero Mt	United States of America	1
García-segovia P	Spain	1
García-villalba R	Belgium	1
Gardiner Ge	Ireland	1
Garey Kw	United States of America	1
Gargano G	France	1
Garrait G	France	1
Garrett Da	United States of America	1
Garrido D	Chile	2
Garrido Jj	France	1

Garridoa D	Chile	1
Garrote Gl	Spain	1
Garssen J	United States of America	1
Garuglieri E	Italy	1
Gasc C	France	1
Gasperotti M	United Kingdom	1
Gasson M	United Kingdom	1
Gathercole J	New Zealand	1
Gathumbi J	Austria	1
Gati Ns	Germany	1
Gatti M	Italy	1
Gaudichon C	France	1
Gaudioso G	Israel	1
Gaudreau H	Canada	1
Gautam L	United Kingdom	1
Gavara R	Spain	1
Gavriliuc S	United Kingdom	1
Gay-quéheillard J	France	1
Gayoso L	Spain	1
Gaytán-martínez M	Mexico	2
Gazzaniga Fs	United States of America	1
Gbassi Gk	France	1
Ge X	China	1
Ge Y	China	1
Gearry R	United Kingdom	1
Gee Jm	United Kingdom	2
Gee Jn	United Kingdom	1
Geelhoed Js	Germany	1
Geirnaert A	Switzerland	8
Gemmi C	Austria	1
Geng F	China	1
Geng Q	China	1
Genisheva Za	Brazil	1
Genovese Mi	Brazil	1
George S	United States of America	1
Georgé S	France	1
George Sm	United Kingdom	1
George T	United Kingdom	1
Geornaras I	United States of America	2
Geraldo Ja	Denmark	1
Gérard P	France	1
Gérard-champod M	France	1
Gerber Gk	United States of America	1
Gerlach Jc	United States of America	1
Gerowitt B	Germany	1
Gerrity D	United States of America	1
Gevrenova R	France	1
Gewirtz AUSTRIA	United States of America	1
Ghadimi D	Germany	1
Ghelardi E	Italy	1
Gheller Me	France	1
Ghilardi M	Italy	1
Ghisoni S	Italy	2
Ghorbani M	United Kingdom	1
Ghosh Ar	India	1
Ghosh K	India	1
Ghosh S	United Kingdom	1
Ghosh Ts	France	1
Ghyselinck J	Belgium	6
Giacomini A	Italy	1
Gianotti A	Italy	1
Giavasis I	Greece	1
Gibb M	United States of America	1
Gibbons S	United Kingdom	1
Giblin L	Ireland	1

Gibson G	United Kingdom	5
Gibson Gr	United Kingdom	37
Gibson Pr	Australia	2
Gidley Mj	Australia	2
Giesy Jp	China	1
Gijs M	Switzerland	1
Gil-izquierdo A	France	1
Gil-martinez J	France	1
Gil-ramírez A	Spain	1
Gil-sánchez I	Spain	4
Gilboa Y	Israel	1
Gill Cir	United Kingdom	5
Gill Pa	Australia	1
Gillberg C	Sweden	1
Gilli G	Italy	1
Gilmour Sg	United Kingdom	1
Giménez B	Spain	1
Ginestra G	Italy	1
Gioielli La	Brazil	1
Giordano C	Italy	1
Giordano Rc	Brazil	1
Gioria S	United Kingdom	1
Giovando S	Spain	1
Girinathan Bp	United States of America	1
Gisbert-quilis P	Spain	1
Giuberti G	Italy	5
Giuffrida F	Switzerland	1
Giuliani C	Italy	2
Giusti S	Italy	2
Glaab E	United States of America	1
Glahn Rp	United States of America	1
Glei M	Germany	3
Glibetic M	Serbia	2
Gliniewicz K	United States of America	1
Glöckl G	Germany	1
Glover Sc	United States of America	1
Gmeiner M	Austria	1
Gnanasekaran T	Denmark	1
Gobbetti M	Italy	3
Gocer Emc	Turkey	1
Goderska K	Poland	1
Godnzález-aguilar Ga	Mexico	1
Godon J-j	France	1
Goel G	India	1
Goicoechea E	Spain	1
Gokulan K	United States of America	2
Golbini mofrad M	Iran	1
Golding M	France	3
Goldstein N	Switzerland	1
Goltz S	Belgium	1
Gomaa A	Canada	1
Gombossy de melo franco Bd	Brazil	1
Gomes A	France	1
Gomes Am	France	1
Gomes Bc	Brazil	1
Gomes de oliveira Me	Brazil	1
Gómez del pulgar Em	Spain	1
Gómez E	Spain	1
Gómez Ja	Colombia	1
Gómez-caravaca Am	Spain	1
Gómez-nieto Má	Spain	1
Gomez-roldan V	Netherlands	2
Gómez-sala B	France	1
Gonçalves Ecbda	France	1
Gonçalves Ga	Brazil	1

Gonçalves Lm	Portugal	1
Gonçalves Lrb	Brazil	1
Gonçalves S	United States of America	1
Gong L	China	2
Gong T	United States of America	1
Goñi I	Spain	2
Gonzàlez alvarez Do	France	1
González de llano D	Spain	2
González E	Spain	1
González-aguilar Ga	Mexico	4
González-avila M	Mexico	1
González-barrio R	United Kingdom	1
González-bermúdez Ca	Spain	1
González-córdova Af	Mexico	1
Gonzalez-escalona N	United States of America	1
Gonzalez-lopez J	Spain	2
Gonzalez-martinez A	Germany	1
Gonzalez-salvador I	Italy	1
Gonzalez-sanjosé Ml	Spain	1
Goossens M	Australia	1
Gopalacharyulu P	Finland	2
Gordon Ji	United States of America	2
Górecka D	Poland	2
Gori G	Italy	1
Gosalbes Mj	Spain	3
Goto A	Japan	1
Gotoh K	Japan	1
Gotteland M	France	3
Gough R	France	1
Goulas Ak	United Kingdom	2
Goulette Tr	United States of America	1
Gouseti O	United Kingdom	2
Govers C	United States of America	2
Gowd V	China	2
Grabherr R	Germany	1
Grace We	United States of America	1
Gradilla-hernández Ms	Mexico	1
Graf K	Germany	2
Graham Dw	China	1
Gramacho Ac	Portugal	1
Gramenzi A	Italy	1
Grandison As	United Kingdom	1
Grangette C	France	1
Granica S	Poland	1
Gras Sl	Australia	1
Grasemann H	United States of America	1
Grattepanche F	Switzerland	2
Gratz R	Germany	1
Grauwet T	Belgium	3
Graverholt G	Denmark	1
Green R	Germany	1
Greenhalgh K	United States of America	1
Greenwood SPAIN	United States of America	1
Gregory Ke	United States of America	1
Greiner R	Germany	1
Greppi A	Netherlands	1
Gresnigt Ms	Germany	2
Gresse R	France	2
Greuel Rj	United Kingdom	1
Greve Lj	United Kingdom	1
Gribbon Lt	United Kingdom	1
Griffith Lg	United States of America	1
Grimaldi R	United Kingdom	1
Grimm V	United Kingdom	1
Gröger M	Germany	2

Grootaert C	Belgium	11
Gross G	Netherlands	2
Grosso M	Italy	1
Grosu-tudor S-s	Belgium	1
Grün C	Germany	1
Grundy Mm-l	France	1
Gruppen H	Netherlands	1
Grzelakowski M	United States of America	1
Gschaedler A	Mexico	1
Gu Az	United States of America	1
Gu C	United Kingdom	1
Gu L	China	1
Gu M	United States of America	2
Gu Q	China	1
Gu X	China	1
Gualberto Nc	Brazil	2
Guan Y	China	2
Guardabassi L	Denmark	1
Gueimonde M	Spain	4
Guéneau V	France	1
Guergoletto Kb	United Kingdom	1
Guerin E	France	1
Guerin J	France	1
Guérin-deremaux L	France	2
Guerra A	France	1
Guerrini L	Spain	1
Guidone A	Italy	1
Guillemin H	France	1
Guillén Md	Spain	1
Guillon F	France	1
Guimarães Jt	Brazil	1
Gulati M	India	1
Gullón B	France	3
Gullón P	France	2
Gunalan V	Denmark	1
Gunenc A	Canada	1
Gunn Jc	United States of America	1
Günther S	Germany	1
Guo B	China	2
Guo C	United States of America	1
Guo F	China	2
Guo H	China	3
Guo J	China	2
Guo L	China	1
Guo M	China	2
Guo R	China	1
Guo T	China	1
Guo W	China	1
Guo Y	United States of America	4
Guo Z	United States of America	1
Gupta Ak	India	1
Gupta P	United States of America	1
Gurbuz O	Turkey	1
Guri A	Canada	1
Gurudutt Ps	India	1
Gutiérrez N	Chile	1
Gutiérrez Op	Netherlands	1
Gutierrez-merino J	United Kingdom	1
Gutiérrez-miceli Fa	United States of America	1
Gutiérrez-sarmiento W	United States of America	1
Gutiérrez-uribe Ja	Mexico	1
Guyot S	France	2
Guzman Jjl	United States of America	1
Gwala S	Belgium	1
Ha N	Australia	1

Ha S-y	South Korea	1
Ha Sk	South Korea	1
Haange S-b	United States of America	1
Haange Sb	Germany	1
Haas A	Germany	1
Haas R	Germany	1
Habe H	Japan	1
Habib S	Canada	1
Hachibamba T	United States of America	1
Hack A	Netherlands	1
Hackam D	United States of America	1
Haenen Grmm	Netherlands	2
Haffner Fb	France	1
Haid A	Austria	1
Haindl R	Germany	2
Hajitou A	Thailand	1
Hakala Tk	Finland	1
Halimi C	France	1
Hall Jf	United States of America	1
Hall Lj	United Kingdom	1
Hall Ms	Australia	1
Hallam S	Denmark	1
Haller D	Germany	1
Halpern M	Israel	1
Haltrich D	Thailand	1
Ham J-s	South Korea	1
Hamaker Br	United States of America	4
Hamamoto T	Japan	1
Hamdi M	Spain	2
Hamelink S	Belgium	1
Haminiuk Cwi	Brazil	1
Hammami R	Canada	2
Han H	China	1
Han J	China	5
Han K	Japan	1
Han K-h	Japan	1
Han L	China	1
Han Ns	South Korea	3
Han S	China	1
Han Y	China	1
Han Z	China	1
Han Z-y	China	1
Hanajima D	Japan	1
Hanhineva K	Denmark	1
Hansen L	Belgium	1
Hanson Kj	United Kingdom	1
Hanson Nj	Brazil	1
Hansson Gc	Sweden	1
Hanteer O	Denmark	1
Hao H	China	3
Hao L	China	1
Hao Y	China	2
Hara Y	United States of America	1
Haraldsson A-k	Sweden	1
Haratifar S	Canada	1
Hari Ar	United States of America	1
Harms H	Germany	1
Harmsen Hj	Belgium	1
Harmsen Hjm	United Kingdom	1
Harrad S	United Kingdom	1
Harris Hc	United Kingdom	3
Harrison Stl	United States of America	1
Harro Jm	China	1
Harrysson H	Netherlands	1
Harstad Om	Brazil	1

Hartmanis Lm	United States of America	1
Hartzell Al	Switzerland	2
Haruta S	Japan	1
Hashemi M	Iran	1
Hashikura N	Japan	1
Hashizume K	Japan	1
Hassan Yi	Canada	1
Hasselwander O	United States of America	1
Hatanaka M	Netherlands	1
Haug Mc	France	1
Havenaar R	Netherlands	8
Hayder M	Poland	1
Hayglass Kt	United States of America	1
He H	China	1
He J	China	1
He L	China	1
He Ml	Brazil	1
He Y	China	2
He Z	China	2
Headley J	Belgium	1
Headley Jv	Belgium	1
Hebeiss I	Germany	1
Hedderley Di	United States of America	2
Hegele R	United States of America	1
Hegland A	Switzerland	1
Heikamp-dejong I	Canada	2
Heilig Hghj	Netherlands	1
Heinig U	United States of America	1
Heinl S	Germany	1
Heinlein A	Germany	1
Heinonen M	Serbia	1
Heinsen F-a	Netherlands	1
Heir E	Norway	1
Heller Kj	Germany	1
Hellmig S	Netherlands	1
Helou C	France	1
Hemeryck Ly	Belgium	2
Hendra R	Indonesia	1
Hendrick C	Belgium	1
Hendricks G	United States of America	1
Hendrickx M	Belgium	1
Hendrickx Me	Belgium	2
Henn Mr	United Kingdom	1
Hennebel T	Belgium	1
Henry A	United Kingdom	1
Henry Ag	Germany	1
Henry G	France	1
Henry M	France	1
Heo K	South Korea	1
Herath Td	United States of America	1
Herberth G	Germany	3
Herbst-kralovetz Mm	United States of America	1
Heredia Fj	United States of America	1
Heringa J	Netherlands	1
Herkenhoff Me	United States of America	1
Hermans N	Belgium	3
Hernández A	Spain	1
Hernández muñoz P	Spain	1
Hernández-álvarez Aj	Canada	2
Hernández-arriaga Am	Mexico	1
Hernandez-gordillo V	United States of America	1
Hernandez-hernandez O	Spain	1
Hernández-ledesma B	Spain	3
Hernández-maldonado Lm	Mexico	1
Hernandez-mendoza A	Mexico	1

Hernandez-raquet G	France	1
Hernandez-sanabria E	Belgium	7
Hernandez-tapia G	Mexico	1
Hernando I	Spain	2
Hernanz D	United States of America	1
Hernot Dc	United States of America	1
Herranz B	Spain	3
Herrera-cazares La	Mexico	1
Herrera-lópez Ej	Mexico	1
Herrero Er	United States of America	1
Herrero M	United Kingdom	2
Hesta M	Belgium	2
Hettel M	Germany	1
Hettiarachchy Ns	United States of America	1
Hettich Rl	United States of America	1
Heyerick A	France	1
Heyndrickx M	Belgium	3
Hibberd Aa	United States of America	2
Hidalgo-martinez S	Netherlands	1
Higashiyama T	United Kingdom	1
Higuero N	Spain	1
Hildebrand F	Switzerland	1
Hileuskaya K	China	1
Hill C	Ireland	4
Hill-yardin El	Sweden	1
Hillewaere Xkd	Belgium	1
Hillman K	United Kingdom	1
Hinc K	Poland	1
Hinojosa Mg	Spain	1
Hinojosa-nogueira D	Spain	2
Hinrichs J	France	1
Hirano K	Spain	1
Hirvonen J	United States of America	1
Hoag Sw	China	1
Hoang H-h	South Korea	1
Hobbs Jk	United Kingdom	1
Hobden Mr	United Kingdom	1
Hodgson Jm	Australia	1
Hodson Mp	Australia	1
Hoebler C	France	3
Hoeflinger Jl	United States of America	1
Hoffman A	Netherlands	1
Hoffman M	United States of America	1
Hofmann A	United Kingdom	1
Hogenkamp A	United States of America	1
Höke H	Germany	1
Holck Al	Norway	1
Holcomb Sj	United States of America	1
Holguin Fo	United States of America	1
Holmes E	United Kingdom	1
Holmes M	United Kingdom	1
Holness Dl	United States of America	1
Holo H	Brazil	1
Holopainen-mantila U	France	1
Holt C	United States of America	1
Holwerda Ht	Turkey	1
Holzapfel Wh	South Korea	1
Homthawornchoo W	Austria	1
Honda H	United States of America	2
Hong K-b	South Korea	1
Hong Sw	South Korea	1
Hongpattarakere T	Italy	2
Honorato Tl	Brazil	1
Hood Ga	United Kingdom	1
Hope Ck	United States of America	1

Hopkins Mj	United Kingdom	3
Hopkins W	United States of America	1
Hora K	Turkey	1
Hori T	Japan	2
Horniblow Rd	United Kingdom	1
Horvath A	Germany	1
Horvath Pj	United States of America	1
Hoshi N	Japan	1
Hoshino S	Japan	1
Hoshino T	Japan	1
Hoshizawa M	Japan	1
Hosie S	Sweden	1
Hospattankar A	Switzerland	1
Hossain Mn	Australia	1
Hosseinian F	Canada	1
Hotchkiss AUSTRIA	United States of America	1
Hotchkiss Jr	United States of America	1
Hotchkiss S	Italy	1
Hou M	United Kingdom	1
Hou Y	United Kingdom	1
How E	Canada	1
Howell A	France	1
Howell K	United Kingdom	1
Howerton A	United States of America	1
Hoyles L	United States of America	1
Hsieh H-y	Taiwan	1
Hu B	China	2
Hu C	China	1
Hu G	China	1
Hu G-a	China	1
Hu J	China	2
Hu J-l	China	1
Hu J-s	China	1
Hu P	China	1
Hu Q	China	3
Hu S	United States of America	1
Hu T	China	1
Hu X	China	3
Hu Y	China	1
Hu Y-c	China	1
Hua R-m	China	1
Huang A	China	1
Huang F	China	3
Huang G-h	China	2
Huang H	China	1
Huang H-y	Taiwan	1
Huang J	China	5
Huang L	China	2
Huang M	China	1
Huang Q	United States of America	6
Huang S	China	2
Huang W	China	2
Huang X	China	2
Huang Y	China	4
Huang Y-j	United States of America	1
Huang Y-y	China	1
Huatan H	United Kingdom	2
Hubbard AUSTRIA m	United Kingdom	1
Hube B	Germany	2
Huber O	Germany	1
Huber P	Brazil	1
Hübschmann T	United Kingdom	1
Huddy Rj	United States of America	1
Hughes Rm	Italy	1
Hughes Sa	United Kingdom	1

Huhtinen H	Finland	1
Huis in’t veld Jhj	Netherlands	2
Hullar Maj	United States of America	1
Humberto H-s	Mexico	1
Hur Sj	South Korea	3
Hurtado-martinez M	Germany	1
Huscroft Gs	United Kingdom	1
Hussain R	Canada	1
Hutchings Sc	Australia	1
Hutkins R	United States of America	1
Hutkins Rw	United States of America	3
Huuskonen L	Finland	1
Huynh K	United States of America	1
Hwang N	South Korea	1
Hyde Ja	United States of America	1
Hyland Np	France	1
Hyötyläinen T	Finland	2
Hyser Jm	United States of America	1
Ianniello Rg	Italy	1
Ianovska Ma	Netherlands	1
Ibánez E	Spain	1
Ichikawa S	Japan	1
Ichim Te	United States of America	1
Iddir M	France	1
Igarashi Y	Japan	1
Igual M	Spain	1
Ilestam N	Sweden	1
Ilhan Ze	United States of America	1
Im P	South Korea	1
Imai T	Japan	1
Immanuel Src	United States of America	1
Immerseel Fv	Belgium	1
Indrati R	Indonesia	1
Infantes-garcia Mr	Belgium	1
Ingber De	United States of America	1
Ingram Jr	United States of America	2
Ingviya N	Thailand	1
Innocenti M	Italy	3
Inoue J	Japan	1
Intaratrakul K	Thailand	1
Iori A	France	1
Iqbal Th	United Kingdom	1
Iqbal Z	China	2
Iraporda C	Argentina	1
Isaev Ji	Russian Federation	1
Isay saad Sm	Brazil	1
Isermann B	Germany	1
Ishii M	Japan	1
Ishimi Y	Japan	1
Ishnaiwer M	France	1
Isibasi A	Mexico	1
Isidoro haminiuk Cw	Brazil	1
Iskandar Km	Canada	1
Iskandar Mm	Canada	6
Islas-espinoza M	Mexico	1
Ismail A	Malaysia	1
Ismail Bb	China	1
Ispirli H	Turkey	1
Iturrospe E	Belgium	1
Ivannikov D	Germany	1
Ivanov V	Singapore	1
Ivanov Vn	Singapore	1
Ivey Kl	Australia	1
Ivusic polic I	Canada	2
Iyer R	United States of America	1

Izquierdo E	France	1
Izzo L	Italy	4
Jackson G	United States of America	1
Jacob-lopes E	Brazil	1
Jacobo-velázquez Da	Mexico	1
Jacobs Dm	Netherlands	2
Jacobs G	Belgium	2
Jacobs H	Belgium	2
Jacobsen Id	Germany	1
Jacquot C	France	1
Jacxsens L	Brazil	1
Jadhav D	India	1
Jäger C	United States of America	1
Jäger Ps	Germany	1
Jaime-fonseca Mr	United Kingdom	2
Jain M	India	1
Jain Sk	Japan	1
Jalil Amm	United Kingdom	1
Jalili-firoozinezhad S	United States of America	1
James K	France	1
Jamieson He	United Kingdom	1
Jana B	Denmark	1
Janeczek M	Poland	1
Janer G	Spain	1
Jang D	South Korea	1
Jang Ey	South Korea	1
Jang Hw	South Korea	1
Jannin V	United States of America	1
Janssen Am	Netherlands	1
Janssenduijghuijsen L	France	1
Jantama K	Thailand	1
Jantama Ss	Thailand	1
Jaramillo-flores Me	Mexico	1
Jardin J	France	2
Jarrige J-f	France	1
Jasinskyte D	Denmark	1
Jaskari J	Belgium	1
Jauregi P	France	1
Jauregui R	Germany	4
Javaudin F	France	1
Jayemanne A	China	1
Jechorek Rp	United States of America	1
Jeevarathinam As	United States of America	1
Jehmlich N	Germany	3
Jellison Kl	United States of America	1
Jensen B-b	France	1
Jensen Bb	China	2
Jensen Hm	United States of America	2
Jensen Sl	United States of America	1
Jeon Jy	South Korea	1
Jeong Hw	South Korea	1
Jeong J-c	South Korea	1
Jeong S	Singapore	1
Jeong Y	South Korea	1
Jeraci Jl	United States of America	1
Jespersen L	Denmark	4
Jha A	United States of America	1
Jha R	United States of America	2
Ji Y	China	2
Jia B	China	1
Jia S	China	1
Jia X	China	4
Jialengbieke B	China	1
Jiang L	China	3
Jiang Ll	China	1

Jiang M	China	1
Jiang Q	China	1
Jiang T	China	2
Jiang Ta	Belgium	1
Jiang X	China	1
Jiang Y	China	2
Jiang Z	China	2
Jianzhong Z	China	1
Jiao X-a	United States of America	1
Jilani H	Spain	2
Jiménez-aspee F	Chile	1
Jiménez-girón A	Belgium	3
Jiménez-hernández N	Spain	3
Jiménez-marín A	France	1
Jin H	China	1
Jin Jb	South Korea	2
Jin L	China	1
Jin Y	United States of America	2
Jin Y-i	South Korea	1
Jing B	China	1
Jirarattanarangsri W	Thailand	2
Jo C	South Korea	1
Jo K	South Korea	3
Joan V	Belgium	1
Jobsis Cmh	New Zealand	1
Johannessen Ea	United Kingdom	1
Johansson Cs	Sweden	1
Johansson Mev	Sweden	1
John wickham Ms	Italy	1
Johnson C	France	1
Johnson It	United Kingdom	2
Jolivet-gougeon A	France	1
Joly C	France	1
Jonathan M	Brazil	1
Jones B	France	1
Jones Cs	United Kingdom	1
Jones D	United States of America	1
Jones Da	United States of America	1
Jonkers Dmae	Netherlands	3
Joo W	South Korea	2
Jorens Pg	Belgium	1
Jorge Yf	Mexico	1
Jørgensen H	China	1
Jörger M	Austria	1
José jara-palacios M	United States of America	1
Joseph Gs	India	1
Jovanovic-lješkovic Nm	Serbia	1
Joyce Sa	France	1
Juániz I	Spain	2
Juge N	United States of America	1
Juhr N-c	Germany	1
Jung Ey	South Korea	1
Jung H-j	South Korea	1
Jung Jy	United States of America	1
Jung S	South Korea	2
Jung Ys	South Korea	1
Junjua M	France	1
Kachenpukdee N	United States of America	1
Kadowaki Y	Japan	1
Kahouli I	Canada	1
Kainulainen V	Finland	1
Kaksonen Ah	Australia	2
Kalantar-zadeh K	Australia	2
Kalazich J	Chile	1
Kalicki B	Poland	1

Kalmokoff M	Canada	1
Kamalian N	Malaysia	1
Kamil A	Belgium	2
Kamiloglu S	Serbia	2
Kämpfer Aam	United Kingdom	1
Kampmann K	Germany	1
Kanase N	United Kingdom	1
Kanchanatawee S	Thailand	1
Kang H-j	South Korea	1
Kang Hj	South Korea	1
Kang J-h	South Korea	1
Kang Jh	South Korea	1
Kang K	South Korea	1
Kang Q	China	1
Kang S-s	South Korea	1
Kang X	China	1
Kang X-c	China	1
Kantor Rs	United States of America	1
Kao D	United Kingdom	1
Kapel N	France	1
Kaplan A	Germany	2
Kappings V	Germany	1
Karakas Cy	Saudi Arabia	1
Karakaya S	Spain	4
Karalis K	United States of America	1
Karas A	United Kingdom	2
Karatzas Kag	United Kingdom	1
Karboune S	Canada	1
Karbstein Hp	Germany	1
Kardum N	Serbia	1
Karim N	China	1
Karimi E	Indonesia	1
Karimi H	Iran	2
Karnati Skr	United States of America	1
Kartinee K	Malaysia	1
Kasapis S	United Kingdom	1
Kashchenko Ni	Russian Federation	1
Kashi Y	Israel	1
Kasipandi M	India	1
Kasper Dl	United States of America	1
Kasper Lh	United States of America	1
Katina K	Denmark	1
Kato Cg	Brazil	1
Katona G	Sweden	1
Kattge S	Germany	1
Kaur P	India	1
Ke X	China	1
Keawsompong S	Thailand	2
Kedzia B	France	1
Keegan Kp	United States of America	1
Kehl A	Germany	1
Keim Jp	Chile	1
Keleszade E	Italy	1
Keller D	Netherlands	7
Keller T	Germany	1
Kellingray L	China	1
Kelly Pm	Ireland	1
Kemmitt J	United States of America	1
Kemperman Ra	France	1
Kemsawasd V	Thailand	3
Kendall Pa	United States of America	2
Kennedy A	United Kingdom	1
Kennedy Ob	United Kingdom	1
Keppler S	United Kingdom	1
Kerckhof F-m	Belgium	4

Kerckhof Fm	Belgium	1
Kerr Da	Australia	1
KerrSOUTH KOREA	United States of America	1
Kersten E	Germany	1
Kesari S	United States of America	1
Keshavarzian A	United States of America	1
Keshvardoust P	Singapore	1
Kester Jc	United States of America	1
Kettle Mj	United Kingdom	1
Khademhosseini A	United States of America	1
Khairallah J	Canada	3
Khakimov B	Denmark	1
Khan A	Denmark	2
Khare S	United States of America	2
Khaskheli Gb	China	1
Kheadr E	Egypt	1
Khiralla G	Egypt	1
Khodaei N	Canada	1
Khokhlova Ev	France	1
Khongkow M	Thailand	1
Khoo C	Spain	1
Khorsi-cauet H	France	3
Khoshnoodi M	Denmark	1
Khursigara Cm	Canada	2
Kiely B	Ireland	1
Kienesberger B	Germany	1
Kilua A	Japan	1
Kim Bk	South Korea	1
Kim Cy	United States of America	1
Kim D-o	South Korea	1
Kim D-w	South Korea	1
Kim H-j	South Korea	1
Kim Hj	United States of America	1
Kim I-c	South Korea	1
Kim I-h	South Korea	1
Kim I-s	South Korea	2
Kim J	United States of America	1
Kim J-h	South Korea	1
Kim J-y	South Korea	1
Kim Jy	South Korea	1
Kim L	United States of America	1
Kim M	South Korea	2
Kim M-s	United States of America	2
Kim Sh	South Korea	1
Kim Sw	United States of America	1
Kim T-j	South Korea	1
Kim Wj	South Korea	1
Kim Y-h	South Korea	1
Kim Y-m	United States of America	1
King L	United Kingdom	1
King Vae	Taiwan	1
Kingsbury Z	United States of America	1
Kingwatee N	Thailand	1
Kinsner-ovaskainen A	United Kingdom	1
Kirkhus B	France	2
Kiss Ak	Poland	1
Kitazono E	Japan	1
Kleerebezem M	Netherlands	4
Kleerebezem R	Netherlands	1
Klein B	Brazil	1
Kleinsteuber S	Germany	2
Klement P	Germany	1
Klewicka E	Poland	1
Klinck B	Netherlands	1
Klinder A	United Kingdom	4

Klinkenberg M	Netherlands	1
Kloch M	United States of America	1
Klosterbuer A	Switzerland	1
Kluijtmans Laj	United States of America	1
Klymiuk I	Germany	1
Klymus K	United States of America	1
Knecht H	Netherlands	1
Kneifel W	Austria	1
Knøchel S	Denmark	1
Knol J	Netherlands	2
Knudsen A	United States of America	1
Knudsen Keb	China	1
Ko G	South Korea	2
Ko K-c	China	1
Koaze H	Japan	1
Kobayashi I	Japan	1
Kobor Ms	United States of America	1
Koc F	France	1
Kocer E	Turkey	1
Koda Y	Australia	1
Koehler A	Germany	1
Koehnlein Ea	Brazil	2
Koehnlein Ém	Brazil	1
Koelsch R	Germany	1
Koenen Me	Netherlands	7
Koh J	United States of America	1
Koh S-c	South Korea	2
Kohler C	Switzerland	1
Koike H	Japan	1
Koistinen Vm	Denmark	1
Kojouharov Hv	United States of America	1
Kok Cr	United States of America	1
Kolba N	United States of America	1
Kolida S	United Kingdom	6
Kollmann Tr	United States of America	1
Kolodziej B	France	1
Kolsteren P	Belgium	1
Komalasari Nlgy	Japan	1
Komolka P	Poland	1
Kondo A	Japan	6
Kong C	Netherlands	1
Kong F	United States of America	1
Kong J	China	1
Kong L	China	1
Konopka Ae	United States of America	1
Konstantinidis Kt	United States of America	1
Kontula P	Belgium	2
Koo J	United States of America	1
Koper Jeb	Italy	1
Kopf-bolanz Ka	Switzerland	1
Korber Dr	Canada	1
Kortman Gam	United States of America	1
Koskinen Pep	Finland	1
Kössö T	France	1
Kosters Mh	United Kingdom	1
Kostyra E	Poland	1
Kot W	Denmark	1
Kotla Ng	United States of America	1
Kotlo Ku	United States of America	1
Koubala Bb	Cameroon	1
Koutsos A	United Kingdom	1
Kovatcheva-datchary P	Germany	1
Koyama H	Japan	1
Koziolkiewicz M	Poland	1
Kozlowski F	France	1

Kozlowski P	Poland	1
Kozu H	Japan	1
Kozyrskyj Al	United States of America	1
Kraft B	Germany	1
Kraigher B	Slovenia	1
Krajmalnik-brown R	United States of America	4
Kramer I	Germany	1
Kraneveld Ad	United States of America	1
Krause J	Germany	1
Krause Jl	Germany	3
Krause R	Austria	1
Krenning G	Netherlands	1
Kretschmer K	Germany	1
Krishnan K	Belgium	3
Kroell F	France	1
Krogfelt Ka	United States of America	1
Król P	United States of America	1
Kruger Mf	Brazil	1
Kruimel J	Netherlands	1
Krych L	Netherlands	1
Kuancha C	Thailand	1
Kuang J-h	China	1
Kubachka Km	United States of America	1
Kubow S	Canada	9
Kucera Gl	United States of America	1
Kuchinka-koch A	Germany	1
Kücükcetin A	Turkey	1
Kücükcetin Io	Turkey	1
Kuerman M	China	1
Kuipers Ej	Netherlands	1
Kulbe Kd	Austria	1
Kulikouskaya V	China	1
Kulkarni J	India	1
Kulozik U	Germany	2
Kumar M	United States of America	1
Kumar Tks	United States of America	1
Kuramae Ee	Brazil	1
Kuroda K	Japan	1
Kurt E	Turkey	1
Kvakova M	United States of America	1
Kwak Y-k	Spain	1
Kwan H-s	China	1
Kwasnica Ma	United States of America	1
Kweon O	United States of America	1
Kwok Ly	China	1
La marca M	Italy	1
Labij E	Netherlands	1
Lacerda massa Nm	Brazil	1
Lacroix C	Switzerland	22
Lade S	India	1
Laerke Hn	China	1
Lafay S	France	1
Lafrance C-p	Canada	1
Laganà M	Italy	1
Lagarda Mj	Spain	4
Laghi L	Italy	2
Laguna L	Spain	4
Lahaye M	France	1
Lahiani Mh	United States of America	1
Lahti L	Netherlands	1
Lahtinen Sj	United States of America	1
Laillet B	France	1
Laing Gd	Belgium	2
Laird Bd	Canada	6
Lajoie F	France	1

Lam K-l	China	1
Lamacchia C	Italy	1
Lambrecht E	Belgium	2
Lamichhane S	United States of America	2
Lammens C	Belgium	1
Lammers Pj	United States of America	1
Lampakis D	Greece	1
Lampe Jw	United States of America	1
Lanciotti R	Italy	3
Landau K	Egypt	1
Landete Jm	Spain	1
Landriscina L	Italy	1
Landuyt A	France	1
Langella P	France	1
Langlands Sj	United Kingdom	1
Lápez P	France	1
Lapidot M	United States of America	2
Lapointe G	Canada	4
Lapomarda A	Italy	1
Lappas M	Australia	1
Laprise C	United States of America	1
Lara-abia S	Spain	1
Larché M	United States of America	1
Larder Ce	Canada	2
Laroche B	France	1
Larondelle Y	France	1
Larré C	France	1
Larrosa M	Spain	1
Larsen N	Denmark	2
Larsson A	Sweden	1
Larsson K	Sweden	2
Larsson M	Netherlands	1
Lasch J	Germany	1
Last A	Germany	2
Laukens D	Belgium	5
Laukens K	Belgium	1
Lavado G	Spain	1
Laven Ra	United Kingdom	1
Lavermicocca P	Italy	1
Lavin R	United States of America	1
Lawlor Pg	Ireland	1
Lawuyi B	Canada	2
Laya A	Cameroon	1
Layden Bt	United States of America	1
Lazarini T	Switzerland	1
Lazarova D	Netherlands	1
Lazuka A	France	1
Le blay G	Switzerland	8
Le bourvellec C	France	1
Le camus C	France	1
Le D	United States of America	1
Le feunteun S	France	5
le goff O	France	2
Le lay C	Canada	1
Le marc Y	Italy	1
Le roux Y	France	1
Le treut A	France	1
Le Tyl	United States of America	1
Lean Mej	United States of America	1
Leandro SPAIN	Brazil	1
Lebrun S	Belgium	5
Lecannu G	France	1
Lechler R	United Kingdom	1
Leclerc M	France	1
Lee B-h	United States of America	1

Lee Dy	South Korea	2
Lee Eh	South Korea	1
Lee Hj	South Korea	1
Lee J	United States of America	3
Lee J-c	United States of America	1
Lee J-h	South Korea	1
Lee J-l	South Korea	1
Lee Jh	South Korea	1
Lee S	South Korea	4
Lee S-d	South Korea	1
Lee Sj	New Zealand	2
Lee Sy	South Korea	3
Lee T	South Korea	1
Lee Y	United States of America	2
Lefebvre Dl	United States of America	1
Leferink Am	Netherlands	1
Legrain-raspaud S	France	1
Lehrter V	France	1
Lehtinen P	Netherlands	1
Lei F	United States of America	2
Lei Q	China	1
Leiknes T	Singapore	1
Leite Akf	Brazil	1
Leite de souza E	Brazil	1
Leiva-vega J	Cuba	1
Léké A	France	1
Lelievre Sa	United States of America	1
Lemos junior Wjf	Italy	1
Lenbury Y	United States of America	1
Leng J	United Kingdom	1
León C	Spain	1
León-camacho M	Spain	1
Leonardi A	Italy	1
Léonil J	France	1
Leriche F	France	4
Lerma-aguilera A	Spain	2
Leroy F	Slovenia	1
Lesellier S	United Kingdom	1
Lesic-arsic B	France	1
Lesmes U	Israel	8
Leung K	Canada	1
Leusink-muis T	United States of America	1
Leusmann Db	Germany	1
Levi Cs	Israel	1
Levina M	United States of America	1
Levine Ea	United States of America	1
Levy O	United States of America	1
Lewanika Tr	United Kingdom	1
Lewis Jr	Australia	1
Li A	United States of America	1
Li B	China	3
Li C	China	10
Li D	United States of America	4
Li Df	China	1
Li E	China	1
Li F	China	3
Li G	China	1
Li Gy	United States of America	1
Li H	China	4
Li J	China	5
Li Jv	United Kingdom	1
Li L	China	11
Li L-q	China	1
Li M	China	2
Li Mh	China	1

Li N	China	3
Li Q	China	2
Li Q-y	China	2
Li R-q	China	1
Li S	China	3
Li T	China	1
Li W	China	5
Li X	China	19
Li Y	China	11
Li Z	China	11
Li Z-h	China	1
Li Z-j	China	1
Li Z-t	China	1
Liang A-h	China	2
Liang S	United States of America	1
Liang X-l	China	1
Liang Y	China	1
Liang Z	China	1
Liao M	China	2
Liao N	China	1
Liao S	China	1
Liao X	China	1
Liao Z	China	1
Licht Tr	Denmark	1
Lidén G	Sweden	1
Lightburn B	Canada	1
Likotrafiti E	United Kingdom	2
Lim J	South Korea	1
Lim Sf	China	1
Lim Sj	Malaysia	1
Lim T	Canada	2
Lima Fv	Brazil	1
Lima Mds	Brazil	5
Limage R	United States of America	1
Lin Ah-m	United States of America	1
Lin GERMANY S	Germany	1
Lin H	China	2
Lin J-t	Taiwan	1
Lin L	China	1
Lin M-y	Taiwan	1
Lin Q	China	1
Lin S	United Kingdom	1
Lin S-l	Taiwan	1
Lin Y	China	1
Lin Y-f	United States of America	1
Lindbäck T	Norway	1
Lindeboom Ref	South Korea	1
Lindemann Sr	United States of America	2
Ling L	South Korea	1
Lips A	Netherlands	1
Liu A	China	1
Liu B	China	2
Liu C	United Kingdom	2
Liu D	China	4
Liu D-b	China	1
Liu D-m	China	1
Liu F	China	3
Liu G	China	2
Liu H	China	8
Liu J	China	6
Liu L	United States of America	12
Liu Ls	United States of America	1
Liu M	China	2
Liu P	China	1
Liu Q	United States of America	3

Liu Q-h	China	1
Liu R	Canada	1
Liu S	China	7
Liu S-f	China	1
Liu W	China	9
Liu W-h	Taiwan	1
Liu W-t	United States of America	1
Liu Wj	China	1
Liu X	China	6
Liu Y	China	10
Liu Y-y	China	2
Liu Z	China	11
Livney Yd	Israel	1
Livrelli V	France	4
Lizunkova P	United Kingdom	1
Llorent-martínez Ej	Spain	2
Lo curto A	Italy	2
Lo curto R	Italy	1
Loarca-pina G	Mexico	4
Loarca-piña Mgf	Mexico	2
Locher Hh	United Kingdom	1
Loening-baucke V	Germany	1
Logan Be	United States of America	1
Loh Sp	Malaysia	1
Lohith K	India	1
Lok S	China	1
Lollo Pc	Brazil	1
Lombardi G	Italy	2
Lomillo Jg	Spain	1
Long C	Netherlands	1
Longo borges L	Argentina	1
Longshaw C	United Kingdom	1
Longshaw Cm	United Kingdom	2
Lonigro Sl	Italy	1
Lönn J	Sweden	1
Lönnerdal B	United States of America	1
Loonen Lmp	Italy	1
Lopert R	United States of America	1
Lopes Aca	Brazil	1
Lopes Np	Brazil	1
López-barrera D	Canada	1
López-barrera Dm	Mexico	1
López-carballo G	Spain	1
López-de-dicastillo C	Spain	1
López-giraldo Lj	Spain	1
López-lópez A	Spain	1
Lopez-lopez C	Spain	1
López-macías C	Mexico	1
López-nicolás R	Spain	1
Lopez-pena Cl	United States of America	1
Loréal O	France	1
Lorenzi E	Italy	1
Lorkowski S	Germany	2
Lorson E	France	1
Lotti C	Israel	1
Louis P	United States of America	1
Louro H	Portugal	1
Loveday Sm	New Zealand	1
Lovegrove Ja	United Kingdom	2
Lovering Am	United Kingdom	1
Low Dy	Australia	2
Lu G	China	2
Lu M	China	1
Lu X	United States of America	4
Lu Y	China	2

Lubkowicz D	United States of America	1
Lucas P	France	1
Lucas-gonzalez R	Spain	4
Luchese Rh	Italy	1
Lucheta Ar	Brazil	1
Luciano Wa	Brazil	1
Lucini L	Italy	5
Ludidi S	Netherlands	1
Ludwig Ia	Spain	4
Lugli Ga	France	1
Lujan H	United States of America	1
Lulitanond V	Thailand	1
Lumeau S	France	1
Luna Gm	Italy	1
Luna Ra	United States of America	2
Luo B	China	1
Luo R	China	1
Luo X	United States of America	1
Luo Y	China	7
Luo Y-s	China	1
Luo Z-g	China	1
Luque-badillo Ac	Mexico	1
Luta G	Belgium	2
Luu T-h	France	1
Lux R	United States of America	1
Lux S	Germany	1
Luz Rl	Brazil	1
Luzardo-ocampo I	Colombia	2
Lv Z	China	1
Lvova L	France	1
Lynch Bs	Canada	1
Lynch G	United States of America	1
Lynch Gl	United States of America	1
Lynch Km	France	1
Lyon Mr	Netherlands	1
Lyte M	United States of America	1
Lyu F	Australia	1
Lyu R	China	1
Lyu S	China	1
Ma C	China	2
Ma F	China	1
Ma G	China	2
Ma H	China	1
Ma N	China	1
Ma P	China	1
Ma Q	China	1
Ma T	China	1
Ma X	China	2
Ma Y	China	4
Ma Z	China	1
Maathuis Ajh	Netherlands	14
Maccaferri S	Italy	2
Macedo dantas A	Brazil	1
Macfarlane Gt	United Kingdom	23
Macfarlane S	United Kingdom	15
Machado ribeiro Tr	Brazil	1
Macià A	Spain	3
Maciel Gm	Brazil	1
Maciel Jf	Brazil	2
Macierzanka A	France	3
Mack D	Canada	1
Mackay Wg	United Kingdom	1
Mackenzie Da	Belgium	1
Mackie A	United Kingdom	8
Mackie Ar	France	4

Macpherson Cw	Canada	2
Macri J	United States of America	1
Maddiboyina B	United States of America	1
Maddox I	New Zealand	1
Maddox Is	New Zealand	1
Madempudi Rs	India	1
Madhwani T	United Kingdom	1
Madruga Ms	Brazil	2
Madsen Js	Denmark	1
Madureira Ar	France	2
Maffeis C	Italy	1
Magalhães R	France	1
Måge I	Norway	1
Magee P	United Kingdom	1
Magliaro C	Italy	1
Magnani M	Brazil	10
Magne L	France	1
Maguin E	France	1
Mahalak K	United States of America	1
Maheswaran P	India	1
Maheux M	Canada	1
Mahler Gj	United States of America	4
Maierl M	Austria	1
Maignien L	Belgium	2
Maillard M	France	1
Maillot M	France	1
Mainente F	Italy	1
Mainville I	United Kingdom	3
Maisto M	Italy	1
Maity C	India	1
Majdoub Yoe	Italy	1
Mäkelä N	Finland	1
Mäkeläinen H	United States of America	2
Mäkeläinen Hs	United States of America	1
Mäkivuokko H	United States of America	1
Mäkivuokko Ha	United States of America	2
Maldonado-celis Me	Colombia	1
Maldonado-gómez Mx	United States of America	3
Maldonado-mateus Ly	Spain	1
Malhotra M	Canada	1
Malhotra-kumar S	Belgium	1
Malik M	United States of America	1
Mallett Ak	United Kingdom	1
Maluquer de motes C	United Kingdom	1
Mamone G	Italy	1
Mamone V	Italy	1
Man Y-b	China	1
Manap Mya	Malaysia	1
Mancabelli L	Italy	3
Mancilla-herrera I	Mexico	1
Mancinelli D	United States of America	1
Mancino W	France	1
Mandalari G	Italy	7
Manderson K	United States of America	1
Mandhane Pj	United States of America	1
Mandic-mulec I	Slovenia	1
Manfredini G	Italy	1
Mang L	China	1
Mangiaterra G	Italy	1
Mangifesta M	France	1
Mangin I	France	1
Manhevi Ve	France	1
Manhivi Ve	South Africa	1
Manikandan A	India	1
Maningat Cc	United States of America	1

Mank M	Netherlands	2
Mann J	United Kingdom	1
Mann Jc	United Kingdom	1
Manowsky J	Germany	1
Manrique Gd	Argentina	1
Mantovani Fd	Brazil	1
Manuel lópez-romero J	Spain	1
Manzanos Mj	Spain	1
Manzi Ap	France	1
Mao D	China	3
Mao H	China	1
Mapelli F	Italy	1
Maqueda M	Spain	1
Maquet V	Belgium	2
Maran Bm	Brazil	1
March Jc	United States of America	1
Marchesi Jr	United Kingdom	1
Marchettini N	Italy	1
Marchioni E	France	2
Márcia canaan Jm	Brazil	1
Marcial-coba Ms	Denmark	1
Marcos R	Spain	2
Marcus Ak	United States of America	1
Marcussen Jø	United States of America	1
Marczely J	United States of America	1
Marefati A	Sweden	1
Margier M	France	1
Mariadassou M	France	1
Marier D	France	1
Marín FRANCE	Spain	1
Marina Z	Malaysia	1
Marinescu D	Canada	2
Marison Iw	Switzerland	1
Mariutti Lrb	Brazil	1
Markiewicz Lh	Poland	1
Marlowe Fw	Germany	1
Marol-bonnin S	France	1
Maróstica junior Mr	Brazil	1
Marques A	Portugal	1
Marques Cnh	United States of America	3
Marsaux B	Switzerland	1
Marshall Dl	United States of America	1
Marsono Y	Indonesia	1
Marteau P	Netherlands	2
Martens Ec	United States of America	1
Martí N	Spain	1
Martin D	Germany	1
Martín del campo St	Mexico	1
Martin Em	United States of America	1
Martin J-f	France	2
Martin L	France	1
Martín-alvarez Pj	Belgium	4
Martín-carrasco I	Spain	1
Martin-morales A	United Kingdom	1
Martin-pascual J	Spain	1
Martín-peláez S	Spain	2
Martina A	Italy	1
Martínez FRANCE	Mexico	1
Martínez G	Spain	1
Martinez Mf	Canada	1
Martinez Rcr	Netherlands	3
Martínez-costa C	Spain	1
Martínez-cuesta Mc	Belgium	5
Martínez-faedo C	Spain	2
Martínez-férez A	Spain	2

Martínez-fernández Ja	Spain	1
Martinez-fierro Ml	Mexico	1
Martínez-iglesias O	Spain	1
Martínez-madrid Mc	Spain	1
Martínez-monzó J	Spain	1
Martinez-rodriguez Aj	Spain	1
Martínez-villaluenga C	Spain	1
Martini D	Italy	1
Martini S	Italy	1
Martino M	Brazil	1
Martins C	France	3
Martins Ml	Brazil	1
Martinucci V	Italy	1
Martoni C	Canada	1
Martorana M	Italy	1
Marze S	Spain	3
Marzorati M	Belgium	44
Masclee A	Netherlands	1
Mashitoa Fm	France	1
Masi P	Italy	1
Masoero F	Italy	1
Mason Lm	United States of America	1
Mason Sl	United Kingdom	2
Massounga bora Af	China	1
Mat Djl	France	1
Matallana Lg	Colombia	1
Mateos R	Spain	3
Mathers Jc	United Kingdom	2
Mathwig K	Netherlands	1
Mathys A	Switzerland	1
Matrella S	Italy	1
Matsuda I	Japan	1
Matsumoto T	Japan	1
Matsunaga Yt	Japan	1
Matsushita O	Japan	1
Mattarelli P	Italy	1
Matte Tc	Brazil	1
Mattei G	Italy	1
Mattei L	United States of America	1
Mattes A	Denmark	1
Mattila I	Finland	9
Mattila O	United States of America	1
Mattila-sandholm T	Finland	4
Mattioli-belmonte M	Italy	1
Matysiewicz M	Poland	1
Maufoux L	France	1
Maukonen J	United States of America	3
Maulvault Al	Portugal	1
Maurer Lh	Brazil	1
Maurer M	Germany	1
Maurício Lds	Brazil	1
Mauriello G	Italy	1
Mauzy Ca	United States of America	1
Mayar M	Netherlands	1
Mayer Mj	China	1
Mayer-miebach E	Germany	1
Mayeur C	France	1
Mayne J	Canada	3
Mayo B	Netherlands	1
Mayrhofer S	Germany	1
Mayura Ipb	Japan	1
Mazzantini D	Italy	1
Mc conville Ml	United Kingdom	1
Mcallister Ta	Brazil	1
Mcbain Aj	United Kingdom	4

Mccartney Al	United States of America	2
Mccleary Bv	United Kingdom	1
Mcclements Dj	United States of America	9
Mcclure Dd	United States of America	1
Mcconnell B	Belgium	2
Mccormick Ba	United States of America	1
Mccuskey S	United States of America	1
Mcdonald Jak	Canada	5
Mcdougall Gj	United Kingdom	1
Mcfarlan C	United Kingdom	1
Mcgee M	United States of America	1
Mcghie T	United Kingdom	1
Mcghie Tk	United States of America	1
Mckay Dm	Canada	1
Mckeown Sj	Sweden	1
Mcleod A	Norway	1
Mcloughlin Pd	United Kingdom	1
Mcnabb Wc	New Zealand	1
Mcneill Mr	New Zealand	1
Mcnulty Np	United States of America	1
Mcquaid A	France	1
Mcshane Mj	United States of America	1
Mcwilliam leitch Ec	United Kingdom	1
Meale Sj	Brazil	1
Meddah AUSTRIA t	France	1
Medina Da	Chile	3
Medina M	France	1
Medina S	France	1
Medyukhina A	Germany	1
Mees E	Belgium	1
Megerlin F	United States of America	1
Mehta D	United States of America	1
Mehta R	United States of America	1
Mehta Ry	United States of America	1
Mehta T	Spain	1
Mei R	United States of America	1
Meier D	Germany	1
Meier S	United States of America	1
Meir Ay	Germany	1
Meireles Maa	Brazil	1
Melgar-bermudez E	Canada	1
Meli Ma	Italy	2
Melia Cd	United States of America	1
Mellmann A	Germany	1
Mello Rdo	Brazil	1
Mena P	Italy	5
Ménard O	France	9
Mendes E	Portugal	1
Mendes Lw	Brazil	1
Mendonça Cmn	Italy	1
Mendoza S	Mexico	1
Mendoza-díaz S	Canada	1
Menezes Fndd	Brazil	1
Meng H	China	1
Meng X	China	1
Menghebilige	China	1
Mennah-govela Ya	United States of America	1
Menzies Is	United Kingdom	1
Mercadante Az	Brazil	2
Mercado-mercado G	Mexico	1
Mercadod-mercadod G	Mexico	1
Merchant Ha	United States of America	1
Mercuri A	United Kingdom	2
Merrill Ea	United States of America	1
Mertens-talcott Su	United States of America	1

Mes Jj	United States of America	1
Mesa V	United States of America	1
Mesquita Mc	Brazil	1
Messias oliveira M	Brazil	1
Messier S	Canada	1
Meucci S	Italy	1
Meulen Rvd	Belgium	1
Meyer Pd	Netherlands	1
Meynier A	France	2
Meysman Fjr	Netherlands	1
Miambi E	France	1
Miao M	China	1
Miao S	France	1
Micard V	France	1
Michel C	France	1
Michelini S	Italy	1
Michels D	Belgium	1
Michniewicz J	Poland	1
Michon C	France	2
Miclotte L	Belgium	1
Middendorf-bauchart B	Germany	1
Miekisch W	Germany	1
Mielcarz Dw	United States of America	1
Mielle P	France	1
Miettinen M	Netherlands	1
Miguel ochando-pulido J	Spain	1
Miguez B	Spain	2
Mikkelsen Ll	Denmark	1
Milani C	France	1
Milenkovic D	United States of America	1
Miller Ge	United States of America	1
Miller Ma	United States of America	2
Miller Mj	United States of America	1
Mills C	United Kingdom	1
Mills Enc	France	1
Minekus M	Netherlands	9
Minnaar A	United States of America	2
Minnebo Y	Belgium	1
Miossec C	France	1
Miotto M	Brazil	1
Mira A	Spain	2
Miralles B	Spain	6
Mirhosseini H	Malaysia	1
Misaghi A	Iran	1
Mishra Ak	India	1
Mistry P	United Kingdom	1
Mitra S	United Kingdom	1
Mitsagga C	Greece	1
Miyata K	Japan	1
Mnich E	Poland	1
Modesto M	Italy	1
Modica S	Belgium	1
Moens F	Belgium	3
Moens Lg	Belgium	1
Mohamad yusop S	Malaysia	1
Moissl-eichinger C	Germany	1
Mokhtari M	Iran	2
Molavi F	Iran	1
Molina E	Brazil	1
Molinero N	Spain	1
Molino S	Spain	4
Möllby R	Spain	1
Molle D	France	1
Molly K	Belgium	3
Monaci L	Italy	1

Mondello L	Italy	1
Mondor M	United Kingdom	1
Mondot S	France	1
Monforte Ar	France	1
Moniz K	France	1
Monnier A	France	1
Montalvo-gonzález E	Mexico	4
Montanari Sr	Brazil	1
Monteiro Mjp	France	2
Montemurro F	Italy	1
Montero M	Brazil	1
Montes Cy	Mexico	1
Montesano D	Italy	2
Montilla A	United Kingdom	1
Montoya Ca	New Zealand	1
Moodley A	Denmark	1
Moon Js	South Korea	2
Moonmungmee S	Thailand	1
Moore Ji	Canada	1
Moorthy As	Canada	3
Moqbel R	United States of America	1
Mora D	Italy	1
Mora-escobedo R	Mexico	2
Moraes Tj	United States of America	1
Morales Mlv	Brazil	2
Moran Ap	Poland	1
Moreira Cg	Brazil	2
Moreira R	Spain	1
Moreira simabuco F	Brazil	1
Morel Fb	Belgium	1
Morelli L	Italy	1
Moreno Fj	Spain	3
Moreno I	Spain	1
Moreno T	Spain	1
Moreno-arribas Mv	Spain	12
Moreno-chamba B	Spain	1
Moreno-olivas F	United States of America	2
Moreno-ortega A	Spain	3
Moreno-rojas Jm	Spain	4
Moreno-rojas R	Spain	1
Morera M	France	1
Mori T	Japan	1
Moriez R	France	1
Moritz T	Denmark	1
Morla-folch J	Spain	1
Morovic W	United States of America	1
Mortelé O	Belgium	1
Mortensen Pb	United Kingdom	1
Morton Jd	New Zealand	2
Mosele Ji	Spain	4
Moser D	United States of America	1
Moshe H	Israel	1
Mosig As	Germany	2
Mosquera Emb	Switzerland	1
Moss R	Canada	1
Mosser J	France	1
Motelica-wagenaar Am	Netherlands	1
Motilva M-j	Spain	4
Motta O	Italy	1
Mottawea W	Egypt	1
Moughan Pj	New Zealand	1
Mountzouris Kc	United Kingdom	3
Moura Ib	United Kingdom	7
Mousavi K	Iran	1
Mousset P-y	France	1

Moye Zd	Belgium	1
Mtui D	United States of America	1
Mu H	China	1
Mueller M	Netherlands	1
Mueller S	Germany	1
Muenster U	Germany	1
Muinde kimatu B	China	1
Muir Jg	Australia	2
Mukherjee A	India	1
Mukherjee S	United States of America	2
Mulenos Mr	United States of America	1
Mulero M	United Kingdom	1
Mulet-cabero A-i	United Kingdom	2
Mulinacci N	Italy	3
Mullaney J	New Zealand	1
Muller Ei	Brazil	1
Müller S	Germany	4
Mullish Bh	United Kingdom	1
Munakata-marr J	United States of America	1
Muñiz P	Spain	1
Muñoz La	Spain	2
Muñoz O	Chile	1
Muñoz-gonzález I	Spain	2
Muñoz-palazon B	Spain	2
Muñoz-tamayo R	France	1
Murakami R	Japan	1
Murota I	Netherlands	1
Murphy M	France	1
Murray B	United Kingdom	1
Murray Bs	France	1
Mustafa S	Malaysia	1
Mutukumira A	New Zealand	2
Nabi-meibodi M	Iran	1
Nacke H	Germany	1
Nadeem Mt	Pakistan	1
Naessens W	Brazil	1
Naeye T	United Kingdom	1
Naficy S	United States of America	1
Nagata R	Japan	1
Nagengast F	France	1
Naidoo V	Spain	1
Naim F	Canada	1
Nair Sm	United Kingdom	1
Nakajima M	Japan	1
Nakajima T	Japan	1
Nakamura Y	Netherlands	1
Nakashima A	Japan	1
Nakata Y	Japan	1
Nakayama Y	Japan	1
Nakka S	Sweden	1
Nakkarach A	Thailand	1
Nalin T	United States of America	1
Nam D-g	South Korea	1
Nam Tg	South Korea	1
Namdee K	Thailand	1
Napolitano A	Italy	2
Narain N	Brazil	2
Narayanan S	India	1
Narbad A	United Kingdom	5
Narciso V	Italy	1
Narihiro T	Japan	1
Narvaes da rocha campos A	Brazil	1
Narváez A	Italy	3
Narváez-cuenca C-e	United States of America	1
Nassar A	Canada	1

Nataro Jp	United States of America	1
Naumann S	Germany	1
Naumovski N	Australia	1
Nauta A	Netherlands	1
Navarrete P	France	1
Navarro Rr	Japan	1
Nayeri F	Sweden	1
Naylor Ta	United States of America	1
Nazih H	France	1
Ndagijimana M	Italy	1
Nderitu Am	United States of America	1
Neckermann K	Austria	1
Nedelcu I	Romania	1
Neelamraju J	India	1
Neerven Rjjv	Netherlands	1
Nelson Gm	United States of America	1
Nelson Kg	United States of America	1
Nelson Mt	United States of America	1
Neri J	Turkey	1
Neri-numa Ia	Brazil	1
Nesme J	Denmark	1
Nestor B	United States of America	1
Neto Mc	France	1
Nettleship I	United States of America	1
Netto Fm	Brazil	2
Neutsch L	Switzerland	1
Neve H	France	1
Neves bezerra Rm	Brazil	1
Neves Ma	Japan	1
Newton Df	United Kingdom	2
Nezer C	Belgium	1
Ng C-y	United Kingdom	1
Ngampanya B	Thailand	1
Nguyen T-h	Thailand	1
Nguyen-ngo C	Australia	1
Ni D	China	1
Ni F	China	1
Ni Y	China	1
Nichelle Sm	Brazil	1
Nicholson M	United States of America	1
Nicholson S	United Kingdom	6
Nickerson Mt	Canada	1
Nicolella C	United Kingdom	1
Nicoli Jr	Brazil	2
Nie C	China	1
Nie Q	China	1
Nie S	China	2
Nie S-p	China	1
Nie X-r	China	2
Nie Y	China	1
Nièa S	Romania	1
Niedzwiedzka Km	United States of America	1
Niegowska M	United States of America	1
Nielsen Ds	Denmark	5
Nielsen Fgg	United States of America	1
Nielsen S	Denmark	2
Nielsen Sd	United Kingdom	1
Niemelä K	Finland	2
Niemi P	Finland	3
Nietzsche S	Germany	1
Nieva-echevarría B	Spain	1
Nilghaz A	Australia	1
Ning Z	Canada	3
Ningegowda Ma	India	1
Niño arias Fc	Argentina	1

Nisbet Dj	United Kingdom	1
Nishida Vs	Brazil	1
Nishida Y	United Kingdom	1
Nishidono Y	Japan	1
Nissen L	Italy	1
Nitisinprasert S	Thailand	1
Nitride C	Italy	1
Nittayasut N	Thailand	1
Niyirora C	United States of America	1
Noack J	Switzerland	1
Nobre C	Colombia	1
Nobu Mk	Japan	1
Nocetti M	Italy	1
Nocianitri Ka	Japan	1
Nock D	Germany	1
Noel Ar	United Kingdom	1
Nogacka Am	Spain	3
Nogueira Da	Brazil	1
Noguera Dr	United States of America	2
Nollet L	Belgium	6
Noori N	Iran	1
Nopens I	Brazil	1
Noratto G	United States of America	1
Nordgaard I	United Kingdom	1
Nordlund E	Finland	5
Normington C	United Kingdom	2
Noten B	Netherlands	1
Nouws Jfm	United Kingdom	1
Novak R	United States of America	1
Novellino E	Italy	1
Nowicki M	France	1
Ntemiri A	France	2
Nueno palop C	United Kingdom	1
Nueno-palop C	United Kingdom	2
Nuncio-jáuregui N	Spain	1
Nunes J	France	1
Nychas Gje	United Kingdom	1
Nyström L	Switzerland	1
O’brien Nm	Brazil	2
O’brien P	United Kingdom	1
O’callagahan Y	Brazil	1
O’callaghan Tf	France	1
O’connor Pm	France	1
O’connor R	United Kingdom	4
O’donnell Mm	France	2
O’donohue M	France	1
O’flaherty Eaa	Ireland	1
O’may Ga	United Kingdom	2
O’meara S	United Kingdom	1
O’neill Ee	Ireland	1
O’sullivan O	Ireland	2
O’sullivan Ó	France	1
O’toole Pw	France	2
O’brien Nm	France	1
O’gorman Dm	France	1
O’riordan P	France	1
O’sullivan O	France	1
O’toole Pw	France	1
Obermüller B	Germany	1
Obuchowski M	Poland	1
Ochoa-repáraz J	United States of America	1
Odamaki T	Japan	1
Oddi S	Brazil	1
Ofiteru Id	United Kingdom	1
Ogata A	Japan	2

Oh M-h	South Korea	1
Ohra-aho T	Italy	1
Ohura K	Japan	1
Oishi K	France	1
Oki K	France	1
Oksman-caldentey K-m	Finland	1
Olano A	United Kingdom	1
Olano-martin E	United Kingdom	2
Olennikov Dn	Russian Federation	1
Oliaee Ma	Iran	1
Oliphant K	Canada	5
Oliva J	Spain	1
Oliveira Dr	Brazil	1
Oliveira M	Spain	1
Oliveira Sd	France	1
Oliviero T	China	1
Olmedilla-alonso B	Spain	1
Omedi Jo	China	1
Onderdonk Ab	United States of America	1
Öngen G	Japan	1
Ooi Ybh	China	1
Ooki Gn	Brazil	1
Oomah Bd	Canada	1
Oomen Ag	Netherlands	2
Oonsivilai R	United States of America	1
Oosterveld A	Netherlands	1
Opalinski S	Poland	1
Opperhuizen A	Netherlands	1
Ordóñez Jl	Spain	1
Ordóñez-díaz Jl	Spain	2
Orešic M	Finland	3
Orlandi M	Italy	1
Orsat V	Canada	1
Ortega-barrales P	Spain	1
Ortega-heras M	Spain	1
Ortega-vidal J	Spain	1
Ortíz-somovilla V	Spain	2
Ortúzar V	Chile	2
Oruna-concha Mj	Italy	1
Osawa R	Japan	1
Oscarsson E	Sweden	1
Oskoueian E	Indonesia	1
Osorio-díaz P	Mexico	2
Ossieur Wp	Belgium	1
Ossiprandi Mc	France	1
Østman B	United States of America	1
Oswald Bp	United States of America	1
Ott Sj	Netherlands	1
Otte J	Denmark	1
Ottman N	Finland	1
Ou Jz	Australia	1
Ou S	China	2
Ouattara Da	France	1
Ouedraogo J-b	Belgium	1
Ouellette M	Canada	1
Ouethrani M	France	1
Ouwehand Ac	United States of America	7
Ouyang W	Canada	2
Oyarzábal Is	United Kingdom	1
Ozel B	Turkey	1
Ozmen togay S	Turkey	1
Oztop Mh	Turkey	1
Öztürk H	Turkey	1
Paalme T	Estonia	1
Pacheco A	Mexico	1

Pacheco Kc	Mexico	1
Pacifico S	Italy	1
Packull-mccormick Sr	Canada	1
Padilha M	Brazil	2
Pagaling E	United States of America	1
Page Cp	United States of America	1
Pakroo S	Italy	1
Palanisamy M	Spain	1
Palanivel R	India	1
Pälchen K	Belgium	1
Paliy O	Spain	1
Paliyath G	Canada	1
Pallares pallares A	Belgium	1
Palm E	Sweden	1
Pan C	United States of America	1
Pan C-h	South Korea	2
Pan J	Canada	1
Pan Y	United States of America	3
Panagou Ez	United Kingdom	1
Panasevich Mr	United States of America	1
Pandolfi F	Italy	1
Pang B	China	1
Pang M	China	1
Pankasemsuk T	Thailand	1
Pannella G	Italy	1
Panozzo J	Australia	1
Panya M	Thailand	1
Paolesse R	France	1
Paparo L	Italy	1
Papon Y	France	2
Papp K	United States of America	1
Parada J	Spain	2
Parada-alfonso F	United States of America	1
Parajó Jc	Spain	2
Paré Pd	United States of America	1
Parente E	Italy	1
Parham Nj	United Kingdom	1
Parimelazhagan T	India	1
Park H	United States of America	2
Park J	United States of America	1
Park J-c	South Korea	1
Park J-s	South Korea	1
Park S	South Korea	1
Parkar Sg	United States of America	4
Parker Ml	Italy	1
Parkhill J	United States of America	1
Parolin C	Italy	2
Parra-llorca A	Spain	1
Parreira Vr	Canada	1
Parseh I	Iran	1
Parsons Mb	United Kingdom	1
Pasc A	France	1
Pasinetti Gm	United States of America	1
Pastene E	Chile	2
Pastore Gm	Brazil	2
Pastore L	Italy	1
Pastoriza de la cueva S	Spain	1
Pastoriza S	Spain	4
Pastrana L	France	1
Pastrana Lm	France	1
Pasu M	United States of America	1
Patangia D	France	1
Patarroyo Jl	United States of America	1
Patel Bg	United States of America	1
Patra M	United States of America	1

Patrignani F	Italy	3
Paul A	Canada	4
Paul ross R	Ireland	1
Pauletto R	Brazil	1
Pavlidis D	Italy	1
Payne An	Switzerland	2
Payne P	New Zealand	1
Pazos-perez N	Spain	1
Peak D	Canada	4
Pearson Nm	United Kingdom	1
Pechenyak B	United States of America	1
Pechlivanis A	United Kingdom	1
Pecka E	Poland	1
Pede Gd	Italy	1
Pedersen G	Denmark	1
Pedro alberto V-l	Mexico	1
Pedro Sg-d	Spain	1
Peeters L	Belgium	1
Pei F	China	1
Peirotén Á	Spain	1
Pekor C	United States of America	1
Peláez C	Belgium	5
Pelinescu D	Romania	3
Pellanda P	France	1
Pellegrini M	Italy	2
Pelpolage S	Japan	1
Pelpolage Sw	Japan	1
Peltier J	United States of America	1
Pelzer S	Germany	1
Peña-ocaña Ba	United States of America	1
Peng C	China	1
Peng D	China	1
Peng X	China	2
Peng X-c	China	1
Peng Y	China	2
Penna Alb	Brazil	2
Peralta Rm	Brazil	3
Perdijk O	Netherlands	1
Pereboom Dpkh	Netherlands	1
Pereira de paula B	Italy	1
Pereira Epr	Brazil	1
Pereira gomes Am	Brazil	1
Pereira Jo	France	1
Pereira Ma	Portugal	3
Pereira Ra	Brazil	1
Pereira Uc	Brazil	1
Pereira-caro G	Spain	5
Pérez álvarez Ja	Spain	1
Pérez ibarreche M	Argentina	1
Pérez R	Chile	1
Pérez-álvarez Já	Spain	4
Perez-burillo S	Spain	5
Pérez-jiménez J	Spain	1
Pérez-pérez V	Mexico	1
Perina Np	Switzerland	1
Perkins Sd	United States of America	1
Pernthaner A	New Zealand	1
Perregaux C	Switzerland	1
Perreten V	Italy	1
Perrin C	France	1
Perry Ids	United States of America	1
Peru K	Belgium	1
Peru Km	Belgium	1
Perullini M	Spain	2
Péry Arr	France	1

Peterbauer C	Germany	1
Peters S	Netherlands	1
Peters U	Germany	1
Peto Tea	United Kingdom	1
Petrangolini G	Italy	2
Petricevic Sm	Serbia	1
Petrof Eo	United Kingdom	1
Petrotos K	Greece	1
Petruzzi L	Italy	1
Petry Fc	Brazil	1
Pettersen Ks	Norway	1
Pettersson D	Denmark	1
Pettersson H	Vietnam	1
Petzold iii He	United States of America	1
Peucelle V	France	1
Peyret P	France	1
Pferschy-wenzig E-m	Germany	1
Pfetzing P	Germany	1
Pham Vt	Netherlands	3
Phatvej W	Thailand	1
Phillipsen Mb	United States of America	1
Phipps Je	United States of America	1
Phongthai S	Austria	1
Pi X	United States of America	1
Piano A	Canada	1
Piazentin Acm	Italy	1
Piazza Rmf	Brazil	2
Picariello G	France	3
Picque D	France	1
Pielech-przybylska K	United States of America	1
Pieper Dh	Belgium	6
Pieters H-j	Netherlands	1
Pieters J	Belgium	1
Pieters L	Belgium	3
Piette G	Canada	1
Pignataro G	Italy	1
Pihlanto A	France	1
Pillai N	Australia	2
Pimentel L	France	1
Pimentel Tc	Brazil	1
Pin C	United Kingdom	1
Pinart M	United States of America	1
Piñeiro Sa	United States of America	1
Pinheiro Acb	Brazil	1
Pinheiro I	Belgium	5
Pino Mt	Chile	1
Pino-garcía Rd	Spain	1
Pino-hernández E	Colombia	1
Pintado M	France	3
Pintado Me	France	4
Pintado Mm	France	1
Pinto F	Chile	1
Pinto Gas	Brazil	1
Pirovani Me	Argentina	1
Pisapia L	Italy	1
Piskorz-ogórek K	Poland	1
Pitart J	Belgium	4
Pithva Sp	India	1
Pitino I	Italy	2
Pitsiladis A	Sweden	1
Piwowarski Jp	Poland	1
Planes D	Spain	1
Playford Rj	United Kingdom	1
Plos S	United Kingdom	1
Plugge Cm	Netherlands	2

Podsdek A	Poland	1
Poeker Sa	Switzerland	2
Poirson C	France	1
Poissant J	United Kingdom	1
Pontarolo R	Brazil	1
Pontoppidan K	Denmark	1
Popineau Y	France	1
Pöppe J	Germany	1
Porras yaruro Jf	France	1
Portela Ia	Brazil	1
Portetelle D	Belgium	1
Portmann R	Switzerland	6
Pospich R	Germany	1
Possemiers S	Belgium	35
Pot B	France	1
Poutanen K	Finland	9
Poveda Cg	France	1
Poveda turrado C	Italy	1
Povey M	United Kingdom	1
Poyatos Jm	Spain	1
Pozo-bayón Ma	Spain	1
Pradhan Sh	United States of America	1
Pradilla D	United States of America	1
Prakash S	Canada	10
Prandi B	Italy	1
Prasertsan P	United Kingdom	1
Prasitpuriprecha C	Thailand	1
Pratten J	United States of America	1
Preciado-ortiz R	Mexico	1
Prestidge C	Australia	1
Prieto-chávez Jl	Mexico	1
Prince Rl	Australia	1
Privat M	France	1
Priyadarisini Vb	India	1
Priyadarshini M	United States of America	1
Probert Hm	United States of America	1
Pronk M	United States of America	1
Props R	Belgium	1
Prosser C	New Zealand	1
Proto A	Italy	1
Proudmana C	United Kingdom	1
Prudencio SPAIN	Brazil	1
Prudhviraj G	India	1
Prueksasri S	Thailand	1
Pu Y	China	1
Puente L	Brazil	1
Puhakka Ja	Finland	1
Pujos E	France	2
Puspitojati E	Indonesia	1
Putaala H	United States of America	1
Putri Ak	Netherlands	1
Puttasontiphot T	United States of America	1
Puttipipatkhachorn S	Thailand	1
Pygall Sr	United Kingdom	1
Pyle Dl	United Kingdom	1
Pyle S	United Kingdom	1
Qi B	China	1
Qi H	China	2
Qi X	China	1
Qi Y	China	1
Qian H	China	1
Qiao S	China	2
Qiao X	China	1
Qin B	Switzerland	1
Qin J	China	1

Qin W	China	1
Qing S	China	1
Qiu Y	China	1
Qu F	China	1
Quartieri A	Italy	1
Quatrin A	Brazil	1
Quayle Aj	United States of America	1
Quehl A	France	1
Queiroga Rdcrde	Brazil	1
Quercia S	Italy	1
Quero Gm	Italy	1
Quezada Mp	Chile	1
Quigley Me	United Kingdom	1
Quiles A	Spain	2
Quilter K	France	1
Quintanilla-carvajal Mx	United Kingdom	1
Quintela Jc	United Kingdom	1
Quinten T	Belgium	1
Quintero Dfg	United States of America	1
Quirós-sauceda A	Mexico	1
Quirós-sauceda Ae	Mexico	1
Quirynen M	United States of America	1
Quraishi Mn	United Kingdom	1
Qvirist L	Italy	1
Raas T	United States of America	1
Raasch M	Germany	1
Raba G	Estonia	2
Rabelo Mc	Brazil	1
Rabini S	Germany	1
Raboni M	Italy	1
Rabot S	France	3
Ract Jnr	Brazil	1
Rada Ec	Italy	1
Raddatz Gc	Brazil	1
Radtke Al	United States of America	1
Raes J	Belgium	5
Raes K	Belgium	3
Ragagnin de menezes C	Brazil	1
Ragione Rl	United Kingdom	1
Rahimi kalateh shah mohammad G	Indonesia	1
Rahman Mh	China	1
Rai DENMARK	Ireland	1
Raimondi Mt	Italy	1
Raimondi S	Italy	2
Rajabi-siahboomi Ar	United States of America	1
Rajanna das C	Belgium	1
Rajaselvam J	Kuwait	1
Rajilic-stojanovic M	Netherlands	2
Rajkovic A	Belgium	2
Ramakrishna S	United States of America	1
Ramalheira R	Portugal	1
Ramalhosa F	Sweden	1
Ramasamy UNITED-STATES	Netherlands	1
Rame V	France	1
Ramirez Mu	United States of America	1
Ramirez-castillo Da	Mexico	1
Ramírez-jiménez Ak	Mexico	2
Ramiro-garcia J	Netherlands	1
Ramona Y	Japan	1
Ramos Am	Brazil	1
Ramos do egyptoqueiroga Rdc	Brazil	1
Rampelli S	Italy	1
Rampelotto C	Brazil	1
Ramsay Ag	United Kingdom	1
Ramsey C	United States of America	1

Ranadheera Cs	Australia	4
Randazzo Cl	Italy	1
Ranganathan N	United States of America	1
Ranganathan P	United States of America	1
Ranjanoro T	Belgium	1
Ranjith kumar M	India	1
Rao S-q	United States of America	1
Rapp Be	Germany	1
Rasmussen S	United States of America	1
Rastall R	United Kingdom	1
Rastall Ra	United Kingdom	16
Råstam M	Sweden	1
Ratering S	Germany	1
Ratjen F	United States of America	1
Rattanachaikunsopon P	Thailand	1
Rattanakul C	United States of America	1
Rattanamongkonkul S	United States of America	1
Rattanasena P	Thailand	2
Rausch Kd	United States of America	1
Rautonen N	United States of America	4
Rautonen Ne	United States of America	2
Ravald N	Sweden	1
Raveschot C	France	1
Ravindran B	Kuwait	1
Rawdkuen S	Austria	1
Rawi Mh	Malaysia	2
Rea Mc	France	5
Read Mn	United States of America	1
Read Nw	United Kingdom	1
Rebaza M	Belgium	1
Reboul E	France	1
Recio I	Spain	7
Redruello B	Netherlands	1
Redzynia M	Poland	1
Reed Ka	United Kingdom	1
Reeves Sg	United States of America	1
Regalado-gonzález C	Mexico	1
Reglero G	Spain	1
Rehman A	Netherlands	2
Reid Dc	United Kingdom	1
Reid G	United Kingdom	1
Reid Sj	United Kingdom	1
Reimer Ra	Netherlands	1
Reinheimer J	Brazil	1
Reisner A	Austria	1
Rejón-orantes Jc	United States of America	1
Remize F	France	1
Remon Jp	Belgium	2
Remondetto Ge	Canada	1
Ren G	China	1
Ren H	United States of America	1
Ren H-q	China	1
Ren J	China	1
Ren P	China	2
Ren Q	China	1
Ren R	China	1
Ren T	United States of America	1
Renard Cmgc	France	1
Renaud J	Canada	1
Rengarajan S	India	1
Reppas C	Germany	1
Requena T	Belgium	5
Réquilé M	France	1
Restrepo-sánchez L-p	United States of America	1
Restuccia C	Italy	1

Rettedal E	New Zealand	1
Reunanen J	Finland	1
Reyes A	United States of America	1
Reyes Lh	United States of America	1
Reyes-vega Ml	Mexico	1
Reygner J	France	1
Reynolds N	United Kingdom	2
Rezadehbashi M	Denmark	1
Rha C-s	South Korea	1
Rhazi L	France	2
Rhimi M	France	1
Ribecco C	Italy	1
Ribeiro Go	Brazil	1
Ribeiro Lo	Brazil	1
Ribeiro Mpa	Brazil	1
Ribeiro R	Brazil	1
Ribeiro Tb	France	1
Ribeiro Trm	Brazil	1
Ribeiro Ts	Brazil	1
Ribeiro Vr	Brazil	1
Ricardo de castro leite júnior B	Brazil	1
Ricci A	Italy	1
Ricciardi A	Italy	1
Rice Sa	Singapore	1
Rich Gt	Italy	1
Richard N	Netherlands	2
Richardson Aj	United Kingdom	1
Rider Dl	United States of America	1
Rietjens Imcm	Netherlands	1
Rigby N	United Kingdom	4
Riggio O	Italy	1
Righetto ziegler D	Italy	1
Rigozzi E	Switzerland	1
Rikalovic Mg	Serbia	1
Rincón-rosales R	United States of America	1
Rinott E	Israel	3
Rios-ibarra Cp	Mexico	1
Rioux Kp	Canada	1
Rioux L-e	France	1
Risbourg B	France	1
Risco E	Spain	1
Ristic Sm	Serbia	1
Ritieni A	Italy	3
Ritter P	Switzerland	1
Rittmann Be	United States of America	1
Riva A	Italy	2
Rivas-montoya E	Spain	1
Rivellese Aa	Sweden	1
Rivero-pérez Md	Spain	1
Rivière A	Belgium	3
Rizzello Cg	Italy	1
Rizzo D	United States of America	1
Robert P	Spain	1
Roberts A	Canada	1
Robins Rj	France	1
Robinson A	United Kingdom	1
Robinson Cd	United States of America	1
Robinson Le	United States of America	1
Robinson Pj	United States of America	1
Rocchetti G	Italy	5
Rocchi M	Italy	1
Rocha faria duque Al	Brazil	1
Rochat F	Switzerland	2
Rodbles-sánchez Rm	Mexico	1
Rode Tm	Norway	1

Rodehutscord M	Germany	1
Rodes L	Canada	6
Rodrigues Db	Brazil	1
Rodrigues E	Brazil	1
Rodrigues Lr	Portugal	2
Rodrigues Rf	Brazil	1
Rodrigues S	Brazil	2
Rodríguez Ms	Brazil	1
Rodríguez-alcalá Lm	France	1
Rodríguez-calvo A	Spain	1
Rodríguez-carrasco Y	Italy	3
Rodríguez-martínez S	Israel	1
Rodríguez-rojo S	Spain	1
Rodriguez-sanchez A	Spain	2
Rodriquez Dc	China	1
Roeselers G	Netherlands	2
Roessle C	Switzerland	1
Rogelj I	Slovenia	1
Roger L	Netherlands	1
Rohatgi G	United States of America	1
Rohfritsch Z	Switzerland	1
Rohwer Fl	United States of America	1
Roldán-guerra Fj	Spain	2
Rolim Frl	Brazil	1
Rolle-kampczyk U	Germany	5
Rolo D	Portugal	1
Romanazzi V	Italy	1
Romanin De	France	1
Romano A	Italy	3
Romano C	Brazil	1
Rombouts C	Belgium	1
Romero M-p	Spain	2
Romero-hernández B	Spain	1
Romo-vaquero M	Belgium	1
Romond Mb	France	1
Rompelberg Cjm	Netherlands	2
Romyasamit C	Thailand	1
Rondia P	Belgium	2
Rooney Cm	United Kingdom	1
Roorda Ak	United States of America	1
Roos S	Belgium	1
Ropers M-h	France	1
Ros-berruezo G	Spain	1
Rosa Nn	France	1
Rosa Prf	Brazil	2
Rosa-sibakov N	United States of America	3
Rose D	United States of America	1
Rose Dj	United States of America	6
Roselino Mn	Brazil	1
Roselli C	Italy	2
Rosenbaum Ma	United States of America	2
Röske I	Germany	1
Rösler U	Germany	1
Ross Bp	Australia	1
Ross Cn	United States of America	1
Ross Gr	Argentina	1
Ross P	Ireland	1
Ross Rp	France	5
Rossi Ea	Brazil	6
Rossi F	Italy	1
Rossi M	Italy	2
Rossi Rc	Italy	1
Rossoni serao Mc	United States of America	1
Rota C	France	1
Rotbart A	Australia	1

Rotsaert C	Belgium	2
Rouau X	France	1
Roughead Z	Switzerland	1
Roume H	France	1
Rousseau F	France	1
Roussel C	Belgium	4
Roussel Y	France	1
Rousselon N	France	1
Rovalino-córdova Am	Netherlands	1
Rowland I	United Kingdom	5
Rowland Ir	United Kingdom	5
Roy Nc	New Zealand	1
Roy R	Australia	1
Ruan W	United States of America	2
Ruas-madiedo P	Spain	4
Rubel Ia	Argentina	1
Rubió L	Spain	1
Rufián-henares Ja	Spain	8
Rui M	China	1
Rui X	China	5
Ruiz Lm	Spain	1
Ruiz-garbajosa P	Spain	1
Ruiz-medina A	Spain	1
Ruiz-perez F	United States of America	1
Ruíz-pérez S	Spain	1
Ruiz-riaguas A	Spain	1
Ruiz-rodríguez A	Spain	1
Ruíz-valdiviezo Vm	Mexico	2
Rumney C	France	1
Rumney Cj	United Kingdom	1
Russmayer H	Germany	1
Russo P	France	1
Ruxrungtham K	Thailand	1
Ruzik L	Poland	1
Ryan J	Australia	1
Rymenans L	Belgium	1
Rytka J	Switzerland	1
Sa S-j	South Korea	1
Sa Z	China	1
Saad Smi	Brazil	14
Saadi Mr	Belgium	1
Saarela M	United States of America	2
Saarinen M	United States of America	2
Saarinen Mt	United States of America	4
Sabally K	Canada	4
Sabinski F	Germany	1
Saboe P	United States of America	1
Sabour Mr	United Kingdom	1
Sabra W	Germany	1
Sadaghian sadabad M	Belgium	1
Sadeghi ekbatan S	Canada	1
Saeed F	Pakistan	1
Saeed M	Pakistan	1
Saengkrit N	Thailand	1
Saengsuwan P	Thailand	1
Saenz C	Denmark	1
Saha S	Canada	2
Sahan Y	Turkey	1
Sahin Aw	France	1
Sahm K	Germany	1
Sahota Ss	United Kingdom	1
Saija A	Italy	1
Saikaly Pe	United States of America	1
Saikumar S	India	1
Sailer M	United Kingdom	1

Saito Vst	Brazil	1
Sakaguchi M	Japan	1
Sakai Y	France	1
Sakamoto Ik	Brazil	7
Sakurai K	Japan	1
Salam Kw	United States of America	1
Salawu So	Brazil	1
Salazar N	Spain	4
Salazar-bermeo J	Spain	1
Salazar-lópez Nj	Mexico	1
Salden B	Netherlands	1
Salden Bn	Belgium	1
Salehi F	Iran	1
Salem Azm	Mexico	1
Salgaço Mk	Brazil	3
Salinas C	Chile	1
Salles C	France	1
Salli K	United States of America	1
Salmerón-ruiz Ml	Mexico	1
Salminen Sj	United States of America	1
Salt L	Italy	1
Salvador V	France	1
Salvia-trujillo L	Belgium	1
Saman P	Thailand	1
Samtlebe M	France	1
Samuelsson Lm	New Zealand	1
Sanatkar R	Indonesia	1
Sanchez Ji	Belgium	1
Sánchez Oj	Colombia	1
Sánchez-burgos Ja	Mexico	1
Sánchez-díaz Am	Spain	1
Sánchez-moreno C	Spain	1
Sánchez-moya T	Spain	1
Sánchez-parra M	Spain	1
Sánchez-patán F	Belgium	2
Sánchez-rivera L	Turkey	1
Sánchez-velázquez Oa	United Kingdom	1
Sanchón J	France	1
Sandberg A-s	Sweden	2
Sander A	United States of America	1
Sanders L	Netherlands	1
Sandford A	United States of America	1
Sanei H	Denmark	1
Sanguansri L	Australia	1
Sannasiddappa Th	United Kingdom	2
Sansosti Mc	Italy	1
Sant’ana As	Brazil	2
Santacruz A	Mexico	1
Santagapita Pr	Spain	2
Santana andrade Jk	Brazil	2
Santerre Cr	United States of America	1
Santiago-lópez L	Mexico	1
Santoni Mm	United States of America	1
Santos Bn	Brazil	1
Santos Cn	Spain	3
Santos de oliveira C	Brazil	1
Santos ornellas Rm	Brazil	1
Santos Tt	Brazil	1
Santos-hernández M	Italy	1
Sanz Ml	Spain	1
Sanz Y	France	3
Sarama Rj	United States of America	1
Saravanan R	India	1
Sarbini Sr	Malaysia	4
Sarbu I	Romania	1

Sariçay Y	Ireland	1
Sarkar A	France	1
Saroj Db	India	1
Sarriá B	Spain	3
Sartoratto A	Switzerland	1
Sasaki D	Japan	7
Sasaki K	Japan	7
Sassi M	France	1
Sathivel S	United States of America	1
Sathyabama S	India	1
Sato S	Japan	1
Sato Y	Japan	1
Satokaria R	Finland	1
Saucier L	Canada	1
Saulnier L	France	1
Saura D	Spain	1
Saura-calixto F	Spain	1
Savard P	Canada	1
Savas Bs	Turkey	1
Savasli E	Turkey	1
Savelkoul Hfj	Netherlands	1
Savelkoul Phm	Netherlands	2
Savidge T	United States of America	2
Savidge Tc	United States of America	1
Savva Gm	United States of America	1
Saxton K	United Kingdom	5
Sáyago-ayerdi Sg	Mexico	12
Sayagod-ayerdi S	Mexico	1
Sayas-barberá E	Italy	1
Sayes Cm	United States of America	1
Sazali Ih	Malaysia	1
Sbrana T	Italy	1
Scarborough Mj	United States of America	1
Scazzina F	Italy	1
Schaepe Ss	Germany	1
Schaepkens E	Netherlands	1
Schaffner Dw	United States of America	1
Schantz Ab	United States of America	1
Schäpe Ss	Germany	2
Scharlau D	Germany	1
Schattenberg F	Germany	3
Schatzmayr D	Austria	1
Scheckel Kg	United States of America	3
Scheers N	Sweden	1
Schepers U	Germany	1
Scher J	France	1
Schick P	Germany	1
Schick S	Germany	1
Schilderink R	Netherlands	1
Schindeler A	United States of America	1
Schisano C	Italy	1
Schlörmann W	Germany	3
Schmeda-hirschmann G	Chile	2
Schmelzer E	United States of America	1
Schmid V	Germany	1
Schmidt H	France	1
Schmidt M	Germany	1
Schneider K	United States of America	1
Schneider Rg	United States of America	1
Schneider Y-j	United States of America	1
Schnell S	Germany	1
Schnorr Sl	Germany	1
Schoenlechner R	Austria	1
Schoeters G	Belgium	1
Schollenberger M	Germany	1

Schols H	Netherlands	1
Schols Ha	Netherlands	3
Scholz D	France	1
Schoterman Mhc	Netherlands	1
Schreiber S	Netherlands	1
Schrezenmeir J	Germany	1
Schroeder Bo	Sweden	1
Schroën K	France	1
Schroeter K	Canada	5
Schultz G	Brazil	1
Schultz-darken N	United States of America	1
Schulz S	Germany	1
Schuren Fhj	Netherlands	1
Schütte A	Sweden	1
Schwab C	France	2
Schwander F	Switzerland	1
Schwarm M	Germany	1
Schwartz Ivd	United States of America	1
Schweiggert-weisz U	Germany	1
Schwejda-guettes S	Germany	1
Schwintner C	France	1
Scippo M-l	Belgium	2
Scott J	United States of America	1
Scott Ja	United States of America	1
Scott Kp	United Kingdom	1
Sears Mr	United States of America	1
Sebedio J-l	France	2
Sebra R	United Kingdom	1
Seczyk L	France	1
Seesuriyachan P	Thailand	1
Seger Go	Sweden	1
Seger M	United States of America	1
Segerink Li	Netherlands	1
Seguí L	Brazil	1
Seguin-devaux C	United States of America	1
Segura-carretero A	Spain	1
Seib Pa	United States of America	1
Seidler K	United Kingdom	1
Seifert A	Israel	1
Seifert N	Netherlands	2
Seino S	Japan	1
Seke F	South Africa	1
Seker A	Turkey	1
Sela Da	United States of America	1
Sela I	Germany	1
Selak M	Belgium	2
Selinheimo E	Netherlands	2
Selma Mv	Belgium	1
Selma-royo M	Spain	1
Selomulya C	China	1
Senés-guerrero C	Mexico	1
Senizza B	Italy	2
Sentandreu V	Spain	2
Seong H	South Korea	1
Seppänen-laakso T	United States of America	1
Serradell Mdla	Spain	2
Serrano Jce	Spain	2
Serrazanetti Di	Italy	2
Severgnini M	Italy	1
Sevillano-armesto E	Cuba	1
Sforza S	Italy	1
Shade C	United States of America	1
Shafer K	United States of America	1
Shah Np	China	3
Shah P	United States of America	1

Shahidi F	China	1
Shai I	Israel	3
Shan S	United States of America	1
Shanahan F	France	3
Shang H	China	1
Shang K	China	1
Shang X	China	1
Shani levi C	Switzerland	1
Shanmugam S	Brazil	1
Shao M-f	China	1
Shao R	China	1
Shao W	Canada	1
Sharaby Y	Israel	1
Sharma Ak	United States of America	1
Sharp Pa	United Kingdom	1
Sharp R	United Kingdom	1
Shatkin Ja	United States of America	1
Shearman S	United Kingdom	1
Sheffield Cl	United Kingdom	1
Sheikh J	United States of America	1
Shen J	China	1
Shen L	United States of America	1
Shen N	China	1
Shen Q	Italy	4
Shen S	China	1
Shen X	China	1
Shen Y	United States of America	1
Sheng G	China	1
Sheng G-p	China	1
Sheng Y	China	1
Sheppard Ae	United Kingdom	1
Shewry Pr	United Kingdom	1
Shi J	China	3
Shi L	China	2
Shi M	China	1
Shi X	China	1
Shi Y	China	2
Shi Y-c	United States of America	1
Shi Y-h	China	2
Shifrin Y	Canada	1
Shim J-j	South Korea	1
Shim Ky	South Korea	1
Shimoni E	United Kingdom	1
Shin D	United States of America	1
Shin I-s	South Korea	1
Shin J	South Korea	1
Shin Sg	South Korea	1
Shin Sy	South Korea	1
Shinkura R	Japan	1
Shirtliff Me	China	1
Shishir Mri	China	3
Shkoporov An	France	1
Shoko T	South Africa	1
Shortt Ct	Italy	1
Shou Y	China	1
Show POLAND	China	1
Shu Q	New Zealand	2
Shubethar S	India	1
Si G	China	1
Sicardi V	France	1
Siciliano S	Belgium	1
Siciliano Sd	Canada	8
Sidor K	Poland	1
Sienkiewicz-szlapka E	Poland	1
Signori pereira K	Italy	1

Sillam-dussès D	France	1
Silva Ek	Brazil	1
Silva El	Brazil	1
Silva Fgd	Brazil	1
Silva J	Portugal	1
Silva Jc	Spain	1
Silva júnior Jcd	Brazil	1
Silva M	Spain	1
Silva Mj	Portugal	1
Silva Rr	Brazil	1
Silva S	France	4
Silva Sn	France	1
Silvério Sc	Portugal	2
Silverman F	United States of America	1
Silverman Jd	United States of America	2
Silvetti T	Italy	1
Silvi S	Italy	1
Simmons L	United States of America	1
Simões da silva Tm	Italy	1
Simon J	United States of America	1
Simon Ma	United States of America	2
Simons E	United States of America	1
Simons R	Netherlands	1
Simpson Bk	Iran	2
Sims Im	New Zealand	1
Simsek S	Spain	2
Singer G	Germany	1
Singh H	New Zealand	1
Singh Rp	United States of America	4
Singh S	United States of America	1
Singh Sk	India	1
Singh V	United States of America	2
Singkhamanan K	Thailand	1
Singu Bd	India	1
Sinigaglia M	Italy	1
Sinnott R	Belgium	2
Sion B	France	1
Sips Ajam	Netherlands	2
Siragusa S	Germany	1
Siriaco A	France	1
Sirinupong N	Denmark	1
Siroli L	Italy	3
Sisconeto bisinotto M	Brazil	1
Sitati N	United States of America	1
Sivakumar D	South Africa	2
Sivieri K	Brazil	16
Siwczak F	Germany	1
Sjögren J	Sweden	1
Skeie S	Norway	1
Skenderidis P	Greece	1
Skjerdal T	Norway	1
Slabbert Rm	South Africa	2
Slavin J	Switzerland	1
Sleno L	Canada	4
Sloan Wt	United Kingdom	1
Slomka V	United States of America	1
Smagghe G	Belgium	5
Smeds A	Finland	1
Smeds Ai	France	1
Smeets Pam	Netherlands	1
Smeets-peeters M	France	1
Smet Id	Belgium	1
Smid Ej	Netherlands	1
Smidt Cr	Spain	1
Smidt H	Netherlands	8

Smit Gpa	United States of America	1
Smit J	France	1
Smith Ar	United Kingdom	1
Smith B	United States of America	1
Smith C	United States of America	2
Smith Ea	United Kingdom	1
Smith H	United States of America	1
Smith Hl	United States of America	1
Smith Sr	United States of America	1
Smoczynska P	Poland	1
Smolen Ja	United States of America	1
So D	Australia	1
Soares J	France	1
Soares Mb	Brazil	1
Soffer N	Denmark	1
Sofos Jn	United States of America	2
Soghomonyan D	Armenia	1
Sojoudi S	Iran	1
Sokolenko S	Canada	1
Soler-rivas C	Spain	1
Solís G	Spain	1
Solomkin Js	United States of America	1
Sonenshein Al	United States of America	1
Song H	China	2
Song M	United States of America	1
Song Q	China	1
Song R	China	1
Song S	China	1
Song Y	China	1
Song Z	China	1
Sontag-strohm T	Finland	1
Sørensen H	Denmark	1
Sørensen Sj	Denmark	1
Sorrentino E	Italy	1
Sost Mm	Netherlands	1
Soto-pantoja Dr	United States of America	1
Souchon I	France	2
Souquet J-m	France	1
Sousa Dz	Portugal	1
Sousa T	Switzerland	1
Souza olegário L	Brazil	1
Souza pedrosa Gt	Brazil	1
Sözer N	Finland	1
Spano G	France	1
Spatz M	France	1
Spear Jr	United States of America	1
Speckmann B	Germany	1
Spencer Jpe	Italy	1
Sphabmixay P	United States of America	1
Spinler Jk	United States of America	1
Spittal W	United Kingdom	4
Spratt P	United Kingdom	1
Sprenger N	Switzerland	1
Sproule-willoughby Km	Canada	1
Sreeja Ps	India	1
Srivoramas T	Thailand	1
Stabnikova Ov	Singapore	1
Stadlbauer V	Germany	1
Staffolo Md	Brazil	1
Stafussa Ap	Brazil	1
Stahl B	France	3
Stalmach A	United Kingdom	1
Stamm Jm	United Kingdom	1
Stams Ajm	Portugal	1
Stanislawska Ij	Poland	1

Stanley Kn	United Kingdom	1
Stanton C	France	2
Stanton Mm	Canada	1
Starkenburg Sr	United States of America	1
Steck J	Germany	1
Stedman A	United Kingdom	1
Steenholdt C	Denmark	1
Steer Te	United Kingdom	2
Steinert Re	Netherlands	3
Stephens N	France	1
Sterzo Cl	Italy	1
Stevens Mja	Switzerland	1
Stevens Y	Netherlands	1
Stewart Cs	United Kingdom	2
Stewart D	United Kingdom	1
Steyaert A	Belgium	1
Stintzi A	Canada	3
Stockdale Sr	France	1
Stoffers H	France	1
Stofilova J	United States of America	1
Stoklosinski H	United Kingdom	1
Stolaki M	Netherlands	1
Stoll T	Switzerland	1
Stone V	United Kingdom	1
Storey De	United States of America	1
Stothart Mr	United Kingdom	1
Stover Jf	Germany	1
Stowell J	United States of America	2
Stoyanov S	Netherlands	1
Strahinic Id	Serbia	1
Strain Cr	France	1
Sträuber H	Germany	1
Stringer A	Belgium	1
Strous M	Germany	1
Struijs K	Belgium	1
Struve C	United States of America	1
Su A	China	2
Su K	China	1
Suárez A	Spain	3
Suárez M	Spain	1
Subbarao P	United States of America	1
Subedi S	United States of America	1
Subirade M	Canada	1
Succi M	Italy	1
Sugier D	France	1
Suh Hj	South Korea	1
Sui X	China	1
Sujaya In	Japan	1
Sulakvelidze A	United States of America	2
Sulek K	Denmark	1
Suleria Har	United Kingdom	1
Šuligoj T	United States of America	1
Sultan S	Egypt	1
Sultana Ms	China	3
Sultanbawa Y	South Africa	1
Suman T	India	1
Sumeri I	Estonia	1
Sun B	China	3
Sun Cl	China	1
Sun G	China	13
Sun G-x	China	4
Sun J-m	China	1
Sun L	United States of America	1
Sun P	China	1
Sun Q	United States of America	1

Sun S	China	1
Sun T	China	2
Sun Ts	China	1
Sun X	China	3
Sun Y	China	6
Sun Z	China	1
Sung Jh	South Korea	1
Sunnapu O	United States of America	1
Suo H	China	2
Supraditareporn W	Thailand	1
Süring C	United Kingdom	1
Sutton Kh	United States of America	2
Suwardana Gnr	Japan	1
Suzuki K	Japan	1
Svensson B	United States of America	1
Swaby Am	Canada	1
Swaminathan M	Sweden	1
Swann J	United Kingdom	1
Swann Jr	United Kingdom	2
Swanson Ks	United States of America	3
Swenor B	United States of America	1
Swiatecka D	Poland	1
Swiatecki A	Poland	1
Swidsinski A	Germany	1
Swidsinski S	Germany	1
Swinkels Dw	United States of America	1
Sybesma W	Netherlands	1
Szawkalo J	Poland	1
Szwengiel A	Poland	2
Szymanski K	Poland	1
Szymona K	United States of America	1
Tabernero M	Spain	2
Tack Fmg	Belgium	5
Taddei F	Italy	1
Tagliazucchi D	Italy	1
Takagi R	Japan	1
Takaro Tk	United States of America	1
Taketani M	United States of America	1
Tako E	United States of America	4
Taladrid D	Spain	2
Tallarico adorno Ma	Brazil	1
Tallarico Ma	Brazil	1
Tallarico-adorno Ma	Brazil	1
Tamanai-shacoori Z	France	1
Tamang B	India	1
Tamargo A	Spain	7
Tamburello A	Norway	1
Taminiau B	Belgium	4
Tamminen T	Finland	3
Tan B	China	1
Tan L	China	2
Tan S	Australia	1
Tanaka K	Japan	2
Tanes C	United States of America	4
Tang C	China	1
Tang P	United States of America	1
Tang W	China	1
Tang X	China	1
Tanner Sa	Switzerland	2
Tannocka Gw	New Zealand	1
Tao J	China	1
Tao S	China	2
Tao T	China	1
Tao W	China	2
Tao Y	China	3

Tao Z	China	1
Tapia-maruri D	Mexico	1
Tardif Sd	United States of America	1
Tarko T	Poland	1
Tarrah A	Italy	1
Tassou Cc	United Kingdom	1
Tatsuya U	South Korea	1
Taupp M	Denmark	1
Tavares A	France	1
Tavares L	France	1
Tavaria F	France	1
Tavaria Fk	France	1
Tavilli E	Italy	1
Tay J-h	Singapore	2
Tay St-l	Singapore	2
Taylan O	Saudi Arabia	1
Tcholakova S	Netherlands	1
Tebbutt S	United States of America	1
Tegetmeyer He	Germany	1
Teixeira F	France	1
Teixeira Ja	France	2
Teixeira Jac	Brazil	1
Teixeira P	France	2
Teles santos T	Brazil	1
Temisak S	Thailand	1
Temiz A	Turkey	1
Temme Ij	Germany	1
Temple-boyer P	France	1
Tenailleau E	France	1
Tenore Gc	Italy	1
Teodorowicz M	Poland	1
Teravest Ma	United States of America	1
Terpend K	Belgium	1
Tesic D	Austria	1
Tessier Fj	France	1
Teughels W	United States of America	1
Tevere S	Switzerland	1
Thakkar Rd	United States of America	2
Thalacker-mercer A	France	1
Thas O	Australia	1
Thatrimontrichai A	Thailand	1
Theis S	United Kingdom	1
Theoduloz C	Chile	1
Theunis M	Belgium	1
Thévenot J	France	3
Thevenot-sergentet D	France	1
Théwis A	Belgium	1
Thierry B	Australia	1
Thieu Nq	Vietnam	1
Thomas Bc	United States of America	1
Thomas M	France	1
Thomas-gahring A	United States of America	1
Thompson Lu	United States of America	1
Thompson POLAND	Australia	1
Thomson P	Chile	1
Thuanthong M	Denmark	1
Thuissard-vasallo I	Spain	1
Thum C	New Zealand	1
Thumann Ta	Germany	1
Thursz Mr	United Kingdom	1
Tian B	China	2
Tian F	China	2
Tian G	China	1
Tian L	China	1
Tian S	China	1

Tian Y	China	1
Tibbe Mp	Netherlands	1
Tiedje Jm	United States of America	1
Tieghi Tdm	Denmark	1
Tiihonen K	United States of America	2
Till H	Germany	1
Tilston E	United Kingdom	1
Timm D	Switzerland	1
Tims S	United States of America	2
Tinck M	Belgium	1
Tipaldi L	Italy	1
Tirella A	Italy	1
Tirwa Rk	India	1
Tison C	France	1
Tiwari Up	United States of America	1
Tjalsma H	United States of America	1
To T	United States of America	1
Tobo A	Japan	1
Tochio T	Spain	1
Toczylowska-maminska R	United States of America	1
Todhunter S	United Kingdom	1
Todhunter Sl	United Kingdom	13
Todorov Sd	Brazil	2
Toe Lc	Belgium	1
Toker Ny	Poland	1
Tomaino A	Italy	1
Tomaro-duchesneau C	Canada	4
Tomas M	Italy	1
Tomas-barberan Fa	Belgium	1
Tomassen Mm	Netherlands	1
Tomasula P	United States of America	1
Tomasula Pm	United States of America	1
Tomé D	France	1
Tomé Tm	Switzerland	1
Tompkins Ta	Canada	3
Tong Ahy	China	1
Tong P	China	1
Tonon Rv	France	1
Topp E	Canada	1
Tornero-martínez A	Mexico	1
Tornuk F	Turkey	1
Toro-uribe S	Spain	1
Torres Ci	United States of America	1
Torres E	Chile	1
Torres Jc	Spain	1
Torres-gregorio M	Mexico	1
Torriani S	Italy	1
Toth I	Australia	1
Tottey W	France	3
Toutain B	France	1
Toutounchi Ns	United States of America	1
Tovaglieri A	United States of America	1
Tovar J	Sweden	3
Toyohara K	Japan	1
Traina Sj	United States of America	3
Tran Tht	Belgium	1
Tran Ttt	France	1
Travaglia F	Italy	1
Traversi D	Italy	1
Trchounian A	Armenia	1
Trejo Sa	Spain	1
Tremblay J	Canada	1
Tremonte P	Italy	1
Trevisan M	Italy	3
Triantis V	Netherlands	1

Tribuzy de magalhães cordeiro Am	Brazil	1
Trindade Csf	Brazil	1
Tringe S	United States of America	1
Tripodi L	Italy	1
Trivedi Hm	United States of America	1
Trivedi Mk	United States of America	1
Troise Ad	Italy	1
Troost F	Netherlands	1
Troost Fj	Netherlands	1
Trost K	Denmark	1
Trovatti uetanabaro Ap	Brazil	1
Trower Tm	United States of America	2
Truchado P	Germany	3
Trumper Dl	United States of America	1
Trzaskowski M	Poland	1
Tsaban G	Germany	2
Tsai J-j	Taiwan	1
Tsakalidou E	Italy	2
Tsao R	Canada	1
Tselepis C	United Kingdom	1
Tsen J-h	Taiwan	1
Tseng Y-t	Taiwan	1
Tsermoula P	Ireland	1
Tsilia V	Belgium	1
Tsitko I	United States of America	1
Tsuda H	Japan	1
Tsukahara T	Japan	1
Tu J	China	1
Tu P	China	1
Tufail T	Pakistan	1
Tullberg C	Sweden	1
Tumbleson Me	United States of America	1
Tuncil Ye	United States of America	2
Tunesi M	Italy	1
Tuohy K	Israel	3
Tuohy Km	United Kingdom	10
Turgeon S	France	1
Turner P	France	1
Turroni F	France	1
Turroni S	Italy	2
Turvey Se	United States of America	1
Tzima K	Ireland	1
Tzortzis G	United Kingdom	3
Uemura K	Japan	1
Ueno Y	Japan	1
Uetanabaro Apt	Brazil	1
Ugolini L	Italy	1
Uhlig S	Austria	1
Ulleberg Ek	France	1
Ulrici A	Italy	1
Umbelino cavallini Dc	Brazil	1
Unal G	Turkey	1
Unc A	United States of America	1
Undeland I	Sweden	2
Untersmayr E	France	1
Uraipan S	Italy	1
Urbán P	United Kingdom	1
Urdaci Mc	France	1
Uribe M	Chile	1
Uriot O	France	3
Urvil P	Sweden	1
Ushida K	Japan	1
Üstün-aytekin Ö	Turkey	1
Uyttendaele M	Belgium	2
Uyttendaele S	Belgium	1

Vacca M	Germany	1
Vacon Fl	France	1
Vaes W	Netherlands	1
Vaidya Y	India	1
Valderrama-rincón Jd	United States of America	1
Valenzuela R	France	1
Valerio F	Italy	1
Valero M	Spain	1
Valero-cases E	Spain	2
Vallejo-cordoba B	Mexico	1
Vallender Ej	United States of America	1
Valls R-m	Spain	1
Valtchev P	United States of America	1
Vamanu A	Romania	1
Vamanu E	Romania	4
Van aken Ga	Netherlands	1
van ark I	United States of America	1
Van beek Jhgm	Netherlands	1
Van bilsen J	Netherlands	1
Van camp J	Belgium	7
Van coillie E	Belgium	2
Van craeyveld V	Belgium	1
Van de velde F	Argentina	1
Van de westerlo Ema	United States of America	1
Van de wiele T	Belgium	68
Van de wiele Tr	Canada	5
Van de wielea T	Belgium	1
Van den abbeele P	Belgium	45
van den berg A	Netherlands	1
van den berg F	Denmark	2
van den heuvel Eg	Netherlands	2
van den heuvel Eghm	Netherlands	1
Van der auwera A	Belgium	1
Van der hoeven Js	Netherlands	1
Van der hooft Jjj	Netherlands	1
van der linde C	Italy	1
Van der lugt T	Netherlands	1
van der mey D	Germany	1
van der vossen Jmbm	Germany	1
Van der wiele T	Thailand	1
Van der woude Jcj	Netherlands	1
van der zande M	Netherlands	1
van dinter R	United States of America	1
van dongen Kcw	Netherlands	1
van dooren I	Belgium	1
Van dorsten F	Netherlands	1
Van dorsten Fa	Netherlands	1
Van duynhoven J	Netherlands	1
Van duynhoven Jp	Netherlands	1
Van eijnatten Ejm	Netherlands	1
Van haute Mj	United States of America	1
Van hecke T	Belgium	1
van heel Da	United Kingdom	1
Van herreweghen F	Germany	4
Van hoorebeke L	Belgium	1
Van immerseel F	Belgium	1
Van leeuwen S	Netherlands	1
Van leuven Jt	United States of America	1
Van lier Jb	South Korea	1
Van lieshout Gaa	Netherlands	1
Van limpt K	Netherlands	1
Van loey A	Belgium	1
Van loey Am	Belgium	1
Van loosdrecht Mcm	United States of America	1
Van meervenne E	Belgium	1

Van mele M	United States of America	1
Van nuenen Mhmc	Netherlands	2
van nuijs Aln	Belgium	1
van rijn M	United States of America	1
Van rymenant E	Netherlands	1
Van saene Jjm	United Kingdom	1
van sinderen D	France	1
van staden Ad	South Africa	1
van valenberg Hjf	Italy	1
van wandelen Mtr	United States of America	1
Van wijmelbeke L	Belgium	1
Van wijnen J	Netherlands	1
Van yken J	Australia	1
Van zanten Gc	United States of America	1
Van't land B	United States of America	1
Vandamme T	France	1
Vande velde I	Belgium	1
Vande woestyne M	Belgium	2
Vandekerkove P	France	1
Vanden bussche J	Belgium	2
Vaneechoutte M	Belgium	1
Vanhaecke L	Belgium	6
Vanhoecke B	Belgium	3
Vanlancker E	Belgium	1
Varankovich N	Canada	1
Varasteh S	United States of America	1
Vardakou M	United Kingdom	3
Varesche Mba	Brazil	1
Vasconcelos de oliveira Ce	Brazil	1
Vasconcelos M	France	1
Vasil'eva Ag	Russian Federation	1
Vaughan Ee	Netherlands	3
Vazquez E	United States of America	1
Vazquez gutierrez P	Switzerland	1
Vazquez J	South Korea	1
Vázquez L	Netherlands	1
Vázquez-campos S	Spain	1
Vázquez-chagoyán Jc	Mexico	1
Vázquez-landaverde Pa	Mexico	1
Vázquez-sánchez K	Mexico	2
Veach Bt	United States of America	1
Vecchione A	Italy	1
Vegarud G	Sweden	2
Vegarud Ge	Norway	4
Veide J	Sweden	1
Veiga M	France	2
Velderrain-roddríguez G	Mexico	1
Veldkamp T	United States of America	1
Vélez D	United Kingdom	2
Venema K	Netherlands	50
Venkataraman A	United States of America	1
Vennos C	Russian Federation	1
Ventura M	Italy	3
Vera c N	Brazil	1
Verachtert P	Belgium	1
Verbeke E	France	1
Verbeke K	Belgium	1
Verbeken K	Belgium	1
Verbruggen S	Netherlands	5
Verdickt E	Belgium	1
Verdier C	France	2
Verdu Ef	Germany	2
Vergara C	Chile	1
Vergéres G	Switzerland	2
Vergoignan C	France	1

Verhelst A	Belgium	5
Verhoeckx K	France	1
Verhoeven J	Netherlands	5
Verkempinck She	Belgium	2
Vermeiren J	United States of America	3
Vermeirssen V	Belgium	2
Vernay T	France	1
Vernon J	United Kingdom	1
Verpoorte E	Netherlands	1
Verruck S	Brazil	1
Versalovic J	United States of America	2
Verschaeve L	Belgium	1
Verspreet J	Belgium	2
Verstraelen H	Germany	1
Verstraete W	Belgium	36
Verstrepen L	Belgium	3
Verthé K	Belgium	2
Vertzoni M	Germany	1
Vervaeck A	Belgium	1
Vervaet C	Belgium	3
Vervoort Jjm	Netherlands	2
Verwei M	Netherlands	1
Vet Lem	Brazil	1
Vetrani C	Sweden	1
Viau M	France	1
Vicente Aa	France	1
Vicente Aamos	Brazil	1
Vichez-vargas R	Belgium	1
Vickers R	United Kingdom	1
Victoria moreno-arribas M	Spain	1
Vidi P-a	United States of America	1
Vidovic Nk	China	1
Vieira Ads	Brazil	5
Vieira Cr	Brazil	1
Vieira Crw	Brazil	1
Vignaroli C	Italy	1
Vigneswaran S	Singapore	1
Vigsnaes Lk	Belgium	4
Vijayabharathi R	India	1
Vila C	Spain	2
Vila L	Spain	1
Vilas-boas A	France	1
Vilas-boas Aa	France	1
Vilcacundo R	Spain	2
Vilchez-vargas R	Germany	6
Villa F	Italy	1
Villageliú Dn	United States of America	1
Villalobos-carvajal R	Cuba	1
Villamiel M	Spain	2
Villanueva-carvajal A	Mexico	1
Villar A	Spain	1
Villas-boas Mb	Brazil	1
Villegas-ochoa Ma	Mexico	1
Villela dias C	Brazil	1
Villemejane C	France	1
Vilpponen-salmela T	Finland	1
Vinarov Z	Netherlands	1
Vinarova L	Netherlands	1
Viñas I	Spain	1
Vincken J-p	China	2
Vinderola Cg	Brazil	1
Vinderola G	Argentina	3
Viotti P	Italy	1
Visconti A	Italy	2
Vishnubhotla R	United States of America	1

Vissenaekens H	Belgium	1
Vitaglione P	Italy	3
Vital M	Belgium	3
Vitali B	Italy	3
Vittori N	United States of America	1
Viuda-martos M	Spain	5
Vlaeminck B	Belgium	1
Vlckova K	France	1
Vo Td	Canada	1
Voigt Ca	United States of America	1
Vollenweider S	Switzerland	1
Volpi N	Italy	1
von bergen M	Germany	5
von stockar U	Switzerland	1
Von wright A	Finland	4
Voolaid M	France	1
Voorspoels S	Belgium	2
Voropaiev M	Germany	1
Vos Md	Belgium	1
Vossen E	Belgium	1
Vozzi G	Italy	1
Vrhovsek U	Israel	3
Vrolijk Mf	Netherlands	1
Vulevic J	United Kingdom	2
Vuono Dc	United States of America	1
Vuopio-varkila J	Netherlands	1
Vuyst Lc	Belgium	1
Vyas Brm	India	1
Wada Y	United States of America	1
Wade Kh	United Kingdom	1
Wagner N	France	1
Wagner R	Brazil	1
Wahl R	France	1
Waldron Kw	Italy	1
Waligora-dupriet A-j	France	1
Walker As	United Kingdom	2
Walker Aw	United Kingdom	3
Walker K	Canada	2
Walker Vk	France	1
Walkowiak J	Poland	1
Wall-medrano A	Mexico	1
Wall-medranod A	Mexico	1
Walter T	France	1
Walton G	United Kingdom	3
Walton Ge	United Kingdom	7
Walvoort Mtc	Netherlands	1
Wan L	China	1
Wang B	China	1
Wang C	China	2
Wang D	China	3
Wang F	China	6
Wang G	China	3
Wang H	China	5
Wang H-s	China	1
Wang J	China	11
Wang J-y	Singapore	1
Wang Jf	China	1
Wang K	China	3
Wang L	China	11
Wang M	China	7
Wang P	China	8
Wang P-p	China	1
Wang Q	China	4
Wang R	China	2
Wang S	China	8

Wang S-p	China	2
Wang T	China	5
Wang W	China	4
Wang X	China	15
Wang Xn	China	1
Wang Y	China	17
Wang Z	China	6
Wang Za	China	1
Wasteson Y	Norway	1
Watanabe T	Japan	1
Watharkar Rb	China	1
Wavreille J	Belgium	1
Webster Tj	United States of America	1
Weese Js	United Kingdom	1
Wegrzyn Tf	New Zealand	1
Wehkamp T	United States of America	1
Wei A	China	1
Wei C	China	1
Wei H	China	3
Wei J	China	1
Wei Q	China	1
Wei W	China	1
Wei X	China	1
Wei Y	China	1
Wei Z	China	2
Weier Sa	United States of America	1
Weijma J	South Korea	1
Weinstein Da	United States of America	1
Weir Jc	United States of America	1
Weiseth B	Canada	1
Weiss G	Denmark	1
Weitschies W	Germany	3
Welling Gw	United Kingdom	1
Wells Cl	United States of America	1
Wells Jm	Italy	1
Welti-chanes J	Spain	1
Wen C	China	1
Wendland I	Germany	1
Wendy lou Wy	United States of America	1
Werner Jj	United States of America	1
Wessling St	Australia	1
Westerbeek Hjm	Netherlands	1
Westerhoff P	United States of America	1
Westerhuis Ja	United States of America	1
Westerman Pr	Germany	1
Westermann M	Germany	1
Westfall S	United States of America	1
Whitacre S	United States of America	1
White Jc	China	1
White Pj	United States of America	1
White T	United States of America	1
Wiazecki K	Poland	1
Wichers Hj	United States of America	2
Wichienchot S	United Kingdom	2
Wicker L	United States of America	1
Wickham Mjs	United Kingdom	1
Wickham Msj	United Kingdom	9
Widmer G	United States of America	1
Widmer W	United States of America	1
Wiebe L	United States of America	1
Wiele Tvd	France	1
Wiertsema S	United States of America	1
Wiese M	Denmark	3
Wiesinger J	United States of America	1
Wightman Jd	United Kingdom	1

Wiik-miettinen F	United States of America	1
Wilcox H	United Kingdom	1
Wilcox Mh	United Kingdom	29
Wilkinson V	United Kingdom	1
Williams Ba	Australia	2
Williams K	United States of America	1
Williams Pn	United Kingdom	2
Williams T	United States of America	1
Wilmes P	United States of America	1
Wils D	France	2
Wilson Aj	United Kingdom	1
Wilson As	United States of America	1
Wilson Fp	United States of America	1
Wilson M	United States of America	2
Wimmer Rf	United States of America	1
Winkler P	Germany	1
Wissenbach DENMARK	Germany	1
Withayagiat U	Thailand	1
Witkowska Z	Poland	1
Woestyne Mv	Belgium	1
Wójcik E	Canada	1
Wollny T	Germany	1
Wolowczuk I	France	1
Wong Ckc	China	1
Wong M-h	China	1
Woo K	United States of America	1
Woo Mw	China	1
Wood J	Sweden	1
Wood S	Netherlands	1
Woodburn Ma	United States of America	2
Woodford N	United Kingdom	1
Woolston J	Belgium	1
Wopereis H	Netherlands	1
Worametrachanon S	Thailand	2
Worley Jn	United States of America	1
Wouters R	Belgium	3
Wragg J	Netherlands	1
Wright C	United States of America	1
Wróblewska B	Poland	1
Wu B	China	2
Wu C	Taiwan	1
Wu D-t	China	4
Wu G	China	2
Wu Gd	United States of America	1
Wu H	China	1
Wu J	China	5
Wu J-y	Taiwan	3
Wu L	Australia	1
Wu M	United States of America	2
Wu P	China	2
Wu Q	China	5
Wu Q-l	United States of America	1
Wu R	China	1
Wu S	China	1
Wu T	Serbia	2
Wu W	China	1
Wu X	Taiwan	7
Wu X-w	China	1
Wu Y	China	3
Wu Z	China	2
Wynne A	United Kingdom	1
Wyse C	United Kingdom	1
Xavier Bb	Belgium	1
Xia S	China	1
Xia Y	China	1

Xian Q	United States of America	1
Xiang C	China	2
Xiang S	China	2
Xiang X	China	1
Xiao C	China	1
Xiao H	United States of America	5
Xiao J	China	2
Xiao J-j	China	2
Xiao J-z	Japan	1
Xiao L	China	1
Xiao P	China	1
Xiao X	China	1
Xiao Y	China	1
Xiaohong H	China	1
Xiaoli L	China	1
Xiaoyu Z	China	1
Xie B	China	1
Xie C	China	1
Xie G	China	1
Xie H	China	1
Xie H-n	Spain	1
Xie J	China	4
Xie M	China	2
Xie M-y	China	1
Xie T-q	China	1
Xie X	China	3
Xing B	United States of America	2
Xing G	China	1
Xing H	China	1
Xing J	China	1
Xing S	China	1
Xiong J	China	1
Xiong L-y	China	1
Xiong Y	China	1
Xiudong X	China	1
Xu F	China	1
Xu H	China	3
Xu J	China	4
Xu L	China	2
Xu M	United States of America	2
Xu N	China	1
Xu Q	China	2
Xu R	China	1
Xu S	China	1
Xu W	China	1
Xu X	China	2
Xu Y	China	5
Xu Z	China	4
Xue B	China	1
Xue Y	China	3
Xue Z	China	1
Yacyshyn B	United States of America	1
Yacyshyn Mb	United States of America	1
Yadav Ak	India	1
Yadav H	United States of America	1
Yadav V	United Kingdom	2
Yadhav Mp	United States of America	1
Yalcinkaya N	Sweden	1
Yamada M	Japan	1
Yamamori A	Japan	2
Yamamoto N	Netherlands	1
Yaman M	Turkey	1
Yamauchi M	Japan	1
Yan H	United States of America	1
Yan J	China	1

Yan L	China	1
Yan Q	China	1
Yan R	China	1
Yan S	China	1
Yan T	United States of America	1
Yan W	China	1
Yan X	China	3
Yáñez R	France	1
Yang A	China	1
Yang C	China	3
Yang D	China	1
Yang H	China	1
Yang J	United States of America	10
Yang K	United States of America	2
Yang L	China	3
Yang M	China	2
Yang Q	China	1
Yang S	China	5
Yang S-t	United States of America	1
Yang W	China	2
Yang X	United States of America	3
Yang Y	China	9
Yang Z	China	2
Yang Z-q	United States of America	1
Yanyan Z	China	1
Yao A-f	France	1
Yao Ck	Australia	2
Yao J	China	1
Yao S	China	1
Yao T	China	1
Yao Y	China	1
Yaqoob P	Italy	1
Yaskolka meir A	United States of America	1
Yasuda K	Japan	1
Yata T	Thailand	1
Yayota M	Japan	1
Yazici Ma	Turkey	1
Yazourh A	France	1
Yde Cc	United States of America	2
Ye H	China	2
Ye K	China	1
Ye L	China	2
Ye X	China	1
Ye Z	China	1
Yebra Mj	Spain	2
Yen S	Canada	4
Yesilada E	Turkey	1
Yeung J	Canada	1
Yi B	South Korea	1
Yi S	Singapore	1
Yilmaz Mt	Saudi Arabia	1
Yilmaz O	United States of America	1
Yin H	China	1
Yin J	United States of America	2
Yin L	China	1
Yin M	China	1
Yin N	China	13
Yin N-y	China	1
Yin X	China	1
Yin Y	China	5
Yin Y-q	United States of America	1
Ying L	China	1
Ying W	China	1
Yolou Fs	France	1
Yong Hi	South Korea	1

Yong Q	China	1
Yong T	China	1
Yoo Jg	South Korea	1
Yoo M-s	South Korea	1
Yoon Hs	South Korea	1
Yoon Jy	United States of America	1
Yoshida K	Japan	2
You L	China	2
You X	China	2
You Y	United States of America	1
Young A	United States of America	2
Young Jf	United States of America	2
Young W	New Zealand	3
Youravong W	Denmark	2
Yu C	China	1
Yu H	China	2
Yu Hd	United States of America	3
Yu J	China	1
Yu J-j	China	1
Yu L	China	1
Yu Q	China	2
Yu R	China	1
Yu T	China	1
Yu X	China	1
Yu Y	China	5
Yu Z	United States of America	2
Yuan L	China	1
Yuan Q	China	3
Yuan Y	China	1
Yuan Z	China	3
Yuanying X	China	1
Yun jiang	China	1
Zafrilla P	Spain	1
Zahradnik Ac	Belgium	1
Zaiat M	Brazil	1
Zamfir M	Belgium	1
Zamora J	Spain	1
Zamora O	Chile	1
Zamora-gasga Vm	Sweden	4
Zanchi R	Italy	1
Zanelli Cf	United States of America	1
Zangara A	Spain	1
Zann V	Switzerland	1
Zanoni F	Italy	1
Zarrella I	Italy	1
Zarur coelho Ma	Italy	1
Zavala L	Spain	1
Zavišic Gn	Serbia	1
Zawadzki W	Poland	1
Zecchin S	Italy	1
Zechner El	Austria	1
Zeibich L	Germany	2
Zeijdner E	Netherlands	1
Zelicha H	United States of America	2
Zeng A-p	Germany	1
Zeng X	China	3
Zeng Z	China	2
Zenhausern F	United States of America	1
Zepka Lq	Brazil	1
Zerr W	Germany	1
Zetzmann S	Germany	1
Zhai Q	China	1
Zhan X	China	3
Zhan X-b	China	1
Zhang B	China	5

Zhang C	China	4
Zhang D	China	3
Zhang G	China	2
Zhang H	China	13
Zhang Hp	China	1
Zhang J	China	4
Zhang K	China	1
Zhang L	China	1
Zhang M	China	5
Zhang Q	China	5
Zhang R	United States of America	5
Zhang S	China	3
Zhang T	China	5
Zhang W	China	3
Zhang X	China	11
Zhang X-x	China	2
Zhang X-z	China	1
Zhang Y	China	15
Zhang Y-n	United Kingdom	1
Zhang Yy	Australia	1
Zhang Z	China	10
Zhang Z-n	China	1
Zhang Z-x	China	1
Zhao C	China	1
Zhao D	United States of America	2
Zhao E	China	1
Zhao G	China	1
Zhao J	China	1
Zhao L	China	6
Zhao M	China	1
Zhao Md	United States of America	1
Zhao Q	New Zealand	2
Zhao R	China	1
Zhao S	China	1
Zhao X	China	2
Zhao X-h	China	3
Zhao Y	China	3
Zhao Z	China	1
Zhao Z-c	China	1
Zheng D-w	China	1
Zheng J	China	2
Zheng Js	China	1
Zheng Q	China	1
Zheng T	United States of America	1
Zheng X	China	2
Zheng Y	China	5
Zheng Z	China	1
Zhong J	China	1
Zhong Z	China	1
Zhou D	China	1
Zhou F	China	1
Zhou G	China	1
Zhou J	China	2
Zhou M	China	1
Zhou Q-y	China	1
Zhou S	China	1
Zhou W	China	1
Zhou X	China	3
Zhou X-h	United States of America	1
Zhou Y	China	1
Zhu B	United States of America	2
Zhu C	China	1
Zhu C-l	China	1
Zhu D	China	1
Zhu J	United States of America	6

Zhu K	China	3
Zhu L	China	10
Zhu Q	China	1
Zhu W	Australia	1
Zhu X	China	3
Zhu Y	China	3
Zhu Yh	China	1
Zhu yong-guan Y-g	United Kingdom	1
Zhu Z-j	China	2
Zhuang W-q	Singapore	1
Zihler A	Switzerland	4
Zihler berner A	Switzerland	2
Zijlstra Rt	United States of America	1
Zimmermann Mb	Switzerland	2
Zoccatelli G	Italy	1
Zoet Fd	Netherlands	1
Zoetendal E	France	1
Zoetendal Eg	Netherlands	4
Zorraquín-peña I	Spain	2
Zorraquín-penìa I	Spain	1
Zotta T	Italy	1
Zou H	China	1
Zou L	China	1
Zou Q	China	1
Zou Y	China	1
Zoumpopoulou G	United Kingdom	1
Zum felde T	Canada	1
Zuo F	China	1
Zuo Yy	United States of America	1
Zura L	Brazil	1

[*] Access to Interllixir program https://carlac.intellixir.fr/cenm provided by The Universidad del Valle

Table S11: Authors affiliation institution. List was retrieved with interllixir program using the same list of documents and manually verified.

Author: publications count	Short affiliations
Vazquez E (1)	ABBOTT US (1)
Chow J (1)	ABBOTT US (1)
Buck R (1)	ABBOTT US (1)
Lohith K (1)	ACADEMY SCIENTIFIC INNOVATIVE RES ACSIR CSIR CENTRAL FOOD TECHNOLOGICAL RES INST CFTRI MYSURU INDIA (1)
Anu-appaiah Ka (1)	ACADEMY SCIENTIFIC INNOVATIVE RES ACSIR CSIR CENTRAL FOOD TECHNOLOGICAL RES INST CFTRI MYSURU INDIA (1)
Simons R (1)	ACATRIS SPECIALITIES HOLDING BV PO BOX ZG GIESSEN NETHERLANDS (1)
Langlands Sj (1)	ADDENBROOKES HOSP CAMBRIDGE UNITED KINGDOM (1)
Coleman N (1)	ADDENBROOKES HOSP CAMBRIDGE UNITED KINGDOM (1)
Van der auwera A (1)	ADREM DATA LAB MATHEMATICSCOMPUTER UNIV ANTWERP MIDDELHEIMLAAN ANTWERP BELGIUM (1)
Peeters L (1)	ADREM DATA LAB MATHEMATICSCOMPUTER UNIV ANTWERP MIDDELHEIMLAAN ANTWERP BELGIUM (1)
Laukens K (1)	ADREM DATA LAB MATHEMATICSCOMPUTER UNIV ANTWERP MIDDELHEIMLAAN ANTWERP BELGIUM (1)
Beirnaert C (1)	ADREM DATA LAB MATHEMATICSCOMPUTER UNIV ANTWERP MIDDELHEIMLAAN ANTWERP BELGIUM (1)
Puspitojati E (1)	AGENCY COUNSELING RESOURCE DEVELOPMENT MINISTRY REPUBLIC INDONESIA JL HARSONO R M JAKARTA SELATAN JAKARTA INDONESIA (1)
Marsono Y (1)	AGENCY COUNSELING RESOURCE DEVELOPMENT MINISTRY REPUBLIC INDONESIA JL HARSONO R M JAKARTA SELATAN JAKARTA INDONESIA (1)
Indrati R (1)	AGENCY COUNSELING RESOURCE DEVELOPMENT MINISTRY REPUBLIC INDONESIA JL HARSONO R M JAKARTA SELATAN JAKARTA INDONESIA (1)
Cahyanto Mn (1)	AGENCY COUNSELING RESOURCE DEVELOPMENT MINISTRY REPUBLIC INDONESIA JL HARSONO R M JAKARTA SELATAN JAKARTA INDONESIA (1)
Samuelsson Lm (1)	AGRESEARCH LTD GRASSLANDS RES CTR PALMERSTON NORTH NEW ZEALAND (1)
Mullaney J (1)	AGRESEARCH LTD GRASSLANDS RES CTR PALMERSTON NORTH NEW ZEALAND (1)
Ahlborn N (1)	AGRESEARCH LTD GRASSLANDS RES CTR PALMERSTON NORTH NEW ZEALAND (1)
Wegrzyn Tf (1)	AGRESEARCH LTD TENNENT DRIVE PRIVATE BAG PALMERSTON NORTH NEW ZEALAND (1)
Singh H (1)	AGRESEARCH LTD TENNENT DRIVE PRIVATE BAG PALMERSTON NORTH NEW ZEALAND (1)
Loveday Sm (1)	AGRESEARCH LTD TENNENT DRIVE PRIVATE BAG PALMERSTON NORTH NEW ZEALAND (1)
Acevedo-fani A (1)	AGRESEARCH LTD TENNENT DRIVE PRIVATE BAG PALMERSTON NORTH NEW ZEALAND (1)
Saucier L (1)	AGRI FOOD CANADA FOOD RES DEVELOPMENT CTR SAINT HYACINTHE QUE CANADA (1)
Piette G (1)	AGRI FOOD CANADA FOOD RES DEVELOPMENT CTR SAINT HYACINTHE QUE CANADA (1)
Naim F (1)	AGRI FOOD CANADA FOOD RES DEVELOPMENT CTR SAINT HYACINTHE QUE CANADA (1)
Messier S (1)	AGRI FOOD CANADA FOOD RES DEVELOPMENT CTR SAINT HYACINTHE QUE CANADA (1)
Topp E (1)	AGRI FOOD CANADA RES BRANCH SANDFORD STREET LONDON ONT N5V 4T3 CANADA (1)
Leung K (1)	AGRI FOOD CANADA RES BRANCH SANDFORD STREET LONDON ONT N5V 4T3 CANADA (1)
Yadhav Mp (1)	AGRICULTURAL RESEARCH SERVICE US (1)
Wu Gd (1)	AGRICULTURAL RESEARCH SERVICE US (1)
Widmer W (1)	AGRICULTURAL RESEARCH SERVICE US (1)
Tumbleson Me (1)	AGRICULTURAL RESEARCH SERVICE US (1)
Tomasula Pm (1)	AGRICULTURAL RESEARCH SERVICE US (1)
Tomasula P (1)	AGRICULTURAL RESEARCH SERVICE US (1)
Thomas-gahring A (1)	AGRICULTURAL RESEARCH SERVICE US (1)
Singh V (2)	AGRICULTURAL RESEARCH SERVICE US (1)
Rausch Kd (1)	AGRICULTURAL RESEARCH SERVICE US (1)
Pinart M (1)	AGRICULTURAL RESEARCH SERVICE US (1)
Mattei L (1)	AGRICULTURAL RESEARCH SERVICE US (1)

Manderson K (1)	AGRICULTURAL RESEARCH SERVICE US (1)
Mahalak K (1)	AGRICULTURAL RESEARCH SERVICE US (1)
Liu Ls (1)	AGRICULTURAL RESEARCH SERVICE US (1)
Hotchkiss At (1)	AGRICULTURAL RESEARCH SERVICE US (1)
Grace We (1)	AGRICULTURAL RESEARCH SERVICE US (1)
Friedman Es (1)	AGRICULTURAL RESEARCH SERVICE US (1)
Evans B (1)	AGRICULTURAL RESEARCH SERVICE US (1)
Dien Bs (1)	AGRICULTURAL RESEARCH SERVICE US (1)
Daniel S (1)	AGRICULTURAL RESEARCH SERVICE US (1)
Chen M-h (1)	AGRICULTURAL RESEARCH SERVICE US (1)
Bobokalonov J (1)	AGRICULTURAL RESEARCH SERVICE US (1)
Beloshapka An (1)	AGRICULTURAL RESEARCH SERVICE US (1)
Liu L (12)	AGRICULTURAL RESEARCH SERVICE US (3)
Tanes C (4)	AGRICULTURAL RESEARCH SERVICE US (4)
Firrman J (4)	AGRICULTURAL RESEARCH SERVICE US (4)
Bittinger K (4)	AGRICULTURAL RESEARCH SERVICE US (4)
Nuncio-jáuregui N (1)	AGRO FOOD MIGUEL HERNANDEZ UNIV CTRA BENIEL KM ORIHUELA ALICANTE SPAIN (1)
Valero-cases E (2)	AGRO FOOD MIGUEL HERNANDEZ UNIV CTRA BENIEL KM ORIHUELA ALICANTE SPAIN (2)
Frutos Mj (2)	AGRO FOOD MIGUEL HERNANDEZ UNIV CTRA BENIEL KM ORIHUELA ALICANTE SPAIN (2)
Shin Sy (1)	AGRO FOOD RESOURCE NAT ACADEMY RDA JEONJU SOUTH KOREA (1)
Ling L (1)	AGRO FOOD RESOURCE NAT ACADEMY RDA JEONJU SOUTH KOREA (1)
Kim T-j (1)	AGRO FOOD RESOURCE NAT ACADEMY RDA JEONJU SOUTH KOREA (1)
Kang J-h (1)	AGRO FOOD RESOURCE NAT ACADEMY RDA JEONJU SOUTH KOREA (1)
Cho Sk (1)	AGRO FOOD RESOURCE NAT ACADEMY RDA JEONJU SOUTH KOREA (1)
Moon Js (2)	AGRO FOOD RESOURCE NAT ACADEMY RDA JEONJU SOUTH KOREA (2)
Joo W (2)	AGRO FOOD RESOURCE NAT ACADEMY RDA JEONJU SOUTH KOREA (2)
Choi Hs (2)	AGRO FOOD RESOURCE NAT ACADEMY RDA JEONJU SOUTH KOREA (2)
Van der wiele T (1)	AGRO INDUSTRY CHIANG MAI UNIV CHIANG MAI THAILAND (1)
Supraditareporn W (1)	AGRO INDUSTRY CHIANG MAI UNIV CHIANG MAI THAILAND (1)
Chokiatirote E (1)	AGRO INDUSTRY CHIANG MAI UNIV CHIANG MAI THAILAND (1)
Jirarattanarangsri W (2)	AGRO INDUSTRY CHIANG MAI UNIV CHIANG MAI THAILAND (2)
Muñoz O (1)	AGRO NEGOCIOS ENOLOGIA FACULTAD CIENCIAS AGRONOMICAS UNIV CHILE AVENIDA SANTA ROSA PINTANA SANTIAGO CHILE (1)
Fuentealba C (1)	AGRO NEGOCIOS ENOLOGIA FACULTAD CIENCIAS AGRONOMICAS UNIV CHILE AVENIDA SANTA ROSA PINTANA SANTIAGO CHILE (1)
Figuerola F (1)	AGRO NEGOCIOS ENOLOGIA FACULTAD CIENCIAS AGRONOMICAS UNIV CHILE AVENIDA SANTA ROSA PINTANA SANTIAGO CHILE (1)
Estévez Am (1)	AGRO NEGOCIOS ENOLOGIA FACULTAD CIENCIAS AGRONOMICAS UNIV CHILE AVENIDA SANTA ROSA PINTANA SANTIAGO CHILE (1)
Ampuero D (1)	AGRO NEGOCIOS ENOLOGIA FACULTAD CIENCIAS AGRONOMICAS UNIV CHILE AVENIDA SANTA ROSA PINTANA SANTIAGO CHILE (1)
Verachtert P (1)	AGROBIOCHEM PRECISION LIVESTOCK TERRA UNIV LIEGE GEMBLOUX BELGIUM (1)
Scippo M-l (2)	AGROBIOCHEM PRECISION LIVESTOCK TERRA UNIV LIEGE GEMBLOUX BELGIUM (1)
Douny C (2)	AGROBIOCHEM PRECISION LIVESTOCK TERRA UNIV LIEGE GEMBLOUX BELGIUM (1)
Brose F (1)	AGROBIOCHEM PRECISION LIVESTOCK TERRA UNIV LIEGE GEMBLOUX BELGIUM (1)
Tomé D (1)	AGROPARISTECHUMR0914 PHYSIOLOGIE DU COMPORTEMENT ALIMENTAIRE RUE CLAUDE BERNARD PARIS FRANCE (1)
Sanchón J (1)	AGROPARISTECHUMR0914 PHYSIOLOGIE DU COMPORTEMENT ALIMENTAIRE RUE CLAUDE BERNARD PARIS FRANCE (1)
Gaudichon C (1)	AGROPARISTECHUMR0914 PHYSIOLOGIE DU COMPORTEMENT ALIMENTAIRE RUE CLAUDE BERNARD PARIS FRANCE (1)
Fernández-tomé S (1)	AGROPARISTECHUMR0914 PHYSIOLOGIE DU COMPORTEMENT ALIMENTAIRE RUE CLAUDE BERNARD PARIS FRANCE (1)
Shani levi C (1)	AGROSCOPE BERN SWITZERLAND (1)
Schwander F (1)	AGROSCOPE BERN SWITZERLAND (1)
Kopf-bolanz Ka (1)	AGROSCOPE BERN SWITZERLAND (1)
Goldstein N (1)	AGROSCOPE BERN SWITZERLAND (1)
Gijs M (1)	AGROSCOPE BERN SWITZERLAND (1)

Vergéres G (2)	AGROSCOPE BERN SWITZERLAND (2)
Portmann R (6)	AGROSCOPE BERN SWITZERLAND (6)
Ritter P (1)	AGROSCOPE LIEBEFELD POSIEUX RES STATION ALP SCHWARZENBURGSTRASSE CH BERNE SWITZERLAND (1)
Kohler C (1)	AGROSCOPE LIEBEFELD POSIEUX RES STATION ALP SCHWARZENBURGSTRASSE CH BERNE SWITZERLAND (1)
Ah Uv (1)	AGROSCOPE LIEBEFELD POSIEUX RES STATION ALP SCHWARZENBURGSTRASSE CH BERNE SWITZERLAND (1)
Yamauchi M (1)	AIST JP (1)
Yamada M (1)	AIST JP (1)
Ueno Y (1)	AIST JP (1)
Tobo A (1)	AIST JP (1)
Sato Y (1)	AIST JP (1)
Nobu Mk (1)	AIST JP (1)
Navarro Rr (1)	AIST JP (1)
Narihiro T (1)	AIST JP (1)
Kuroda K (1)	AIST JP (1)
Koike H (1)	AIST JP (1)
Ishii M (1)	AIST JP (1)
Igarashi Y (1)	AIST JP (1)
Haruta S (1)	AIST JP (1)
Hanajima D (1)	AIST JP (1)
Habe H (1)	AIST JP (1)
Ogata A (2)	AIST JP (2)
Hori T (2)	AIST JP (2)
Paul ross R (1)	ALIMENTARY HEALTH LTD CORK IRELAND (1)
Kiely B (1)	ALIMENTARY HEALTH LTD CORK IRELAND (1)
Fouhy F (1)	ALIMENTARY HEALTH LTD CORK IRELAND (1)
Ross P (1)	ALIMENTARY PHARMABIOTIC CTR UNIV CORK IRELAND (1)
Lawlor Pg (1)	ALIMENTARY PHARMABIOTIC CTR UNIV CORK IRELAND (1)
Gardiner Ge (1)	ALIMENTARY PHARMABIOTIC CTR UNIV CORK IRELAND (1)
Casey Pg (1)	ALIMENTARY PHARMABIOTIC CTR UNIV CORK IRELAND (1)
O'sullivan O (2)	ALIMENTARY PHARMABIOTIC CTR UNIV CORK IRELAND (2)
Dobson A (2)	ALIMENTARY PHARMABIOTIC CTR UNIV CORK IRELAND (2)
Crispie F (2)	ALIMENTARY PHARMABIOTIC CTR UNIV CORK IRELAND (2)
Cotter Pd (3)	ALIMENTARY PHARMABIOTIC CTR UNIV CORK IRELAND (2)
Zhang J (4)	ALL BANKS (1)
Li Q (2)	ALL BANKS (1)
Huang A (1)	ALL BANKS (1)
Chu Y (2)	ALL BANKS (1)
Chen B (1)	ALL BANKS (1)
Marina Z (1)	ALLIANCE RES INNOVATION FOOD ARIF FAC UNIVERSITI TEKNOLOGI MARA NEGERI SEMBILAN KUALA PILAH CAMPUS KUALA PILAH NEGERI SEMBILAN MALAYSIA (1)
Loh Sp (1)	ALLIANCE RES INNOVATION FOOD ARIF FAC UNIVERSITI TEKNOLOGI MARA NEGERI SEMBILAN KUALA PILAH CAMPUS KUALA PILAH NEGERI SEMBILAN MALAYSIA (1)
Kartinee K (1)	ALLIANCE RES INNOVATION FOOD ARIF FAC UNIVERSITI TEKNOLOGI MARA NEGERI SEMBILAN KUALA PILAH CAMPUS KUALA PILAH NEGERI SEMBILAN MALAYSIA (1)
Fadhilah J (1)	ALLIANCE RES INNOVATION FOOD ARIF FAC UNIVERSITI TEKNOLOGI MARA NEGERI SEMBILAN KUALA PILAH CAMPUS KUALA PILAH NEGERI SEMBILAN MALAYSIA (1)
Amin I (1)	ALLIANCE RES INNOVATION FOOD ARIF FAC UNIVERSITI TEKNOLOGI MARA NEGERI SEMBILAN KUALA PILAH CAMPUS KUALA PILAH NEGERI SEMBILAN MALAYSIA (1)
Vanden bussche J (2)	ANALYSIS GHENT UNIV MERELBEKE BELGIUM (1)
El hage Ra (1)	ANALYSIS GHENT UNIV MERELBEKE BELGIUM (1)
Bussche Jv (1)	ANALYSIS GHENT UNIV MERELBEKE BELGIUM (1)
Yang X (3)	APPLE US (1)
Sun L (1)	APPLE US (1)
Li D (4)	APPLE US (1)
Guo Y (4)	APPLE US (1)
Guo C (1)	APPLE US (1)
Emmanuel A (1)	AQUACULTURE NAT INST FISHERIES BUSAN SOUTH KOREA (1)

Mukherjee A (1)	AQUACULTURE UNIV BURDWAN GOLAPBAG BURDWAN WEST BENGAL INDIA (1)
Ghosh K (1)	AQUACULTURE UNIV BURDWAN GOLAPBAG BURDWAN WEST BENGAL INDIA (1)
Dutta D (1)	AQUACULTURE UNIV BURDWAN GOLAPBAG BURDWAN WEST BENGAL INDIA (1)
Banerjee S (1)	AQUACULTURE UNIV BURDWAN GOLAPBAG BURDWAN WEST BENGAL INDIA (1)
Thierry B (1)	ARC CTR EXCELLENCE CONVERGENT NANO UNIV SOUTH AUSTRALIA CITY WEST CAMPUS ADELAIDE SA AUSTRALIA (1)
Prestidge C (1)	ARC CTR EXCELLENCE CONVERGENT NANO UNIV SOUTH AUSTRALIA CITY WEST CAMPUS ADELAIDE SA AUSTRALIA (1)
Nilghaz A (1)	ARC CTR EXCELLENCE CONVERGENT NANO UNIV SOUTH AUSTRALIA CITY WEST CAMPUS ADELAIDE SA AUSTRALIA (1)
Delon Lc (1)	ARC CTR EXCELLENCE CONVERGENT NANO UNIV SOUTH AUSTRALIA CITY WEST CAMPUS ADELAIDE SA AUSTRALIA (1)
Cheah E (1)	ARC CTR EXCELLENCE CONVERGENT NANO UNIV SOUTH AUSTRALIA CITY WEST CAMPUS ADELAIDE SA AUSTRALIA (1)
Rubel Ia (1)	AREA BIOQUIMICA ALIMENTOS FACULTAD CIENCIAS EXACTAS UNIV NACIONAL PLATA CALLE PLATA ARGENTINA (1)
Manrique Gd (1)	AREA BIOQUIMICA ALIMENTOS FACULTAD CIENCIAS EXACTAS UNIV NACIONAL PLATA CALLE PLATA ARGENTINA (1)
Iraporda C (1)	AREA BIOQUIMICA ALIMENTOS FACULTAD CIENCIAS EXACTAS UNIV NACIONAL PLATA CALLE PLATA ARGENTINA (1)
Graverholt G (1)	ARLA FOODS DK (1)
Brück Wm (1)	ARLA FOODS DK (1)
Moorthy As (3)	ATLANTIC FOOD HORTICULTURE RES STATION AGRI FOOD CANADA KENTVILLE NS CANADA (1)
Kalmokoff M (1)	ATLANTIC FOOD HORTICULTURE RES STATION AGRI FOOD CANADA KENTVILLE NS CANADA (1)
Eberl Hj (3)	ATLANTIC FOOD HORTICULTURE RES STATION AGRI FOOD CANADA KENTVILLE NS CANADA (1)
Brooks Spj (1)	ATLANTIC FOOD HORTICULTURE RES STATION AGRI FOOD CANADA KENTVILLE NS CANADA (1)
Savas Bs (1)	AYDIN ADNAN MENDERES UNIV FAC DAIRY KOCARLI AYDIN TURKEY (1)
Akan E (1)	AYDIN ADNAN MENDERES UNIV FAC DAIRY KOCARLI AYDIN TURKEY (1)
Yazourh A (1)	BACTERIOLOGIE FACULTE DES PHARMACEUTIQUES RUE DU PR LAGUESSE BP LILLE FRANCE (1)
Romond Mb (1)	BACTERIOLOGIE FACULTE DES PHARMACEUTIQUES RUE DU PR LAGUESSE BP LILLE FRANCE (1)
Risbourg B (1)	BACTERIOLOGIE FACULTE DES PHARMACEUTIQUES RUE DU PR LAGUESSE BP LILLE FRANCE (1)
Meddah Att (1)	BACTERIOLOGIE FACULTE DES PHARMACEUTIQUES RUE DU PR LAGUESSE BP LILLE FRANCE (1)
Desmet I (1)	BACTERIOLOGIE FACULTE DES PHARMACEUTIQUES RUE DU PR LAGUESSE BP LILLE FRANCE (1)
Maillard M (1)	BARRY CALLEBAUT (1)
Landuyt A (1)	BARRY CALLEBAUT (1)
Ravindran B (1)	BASIC PUBLIC AUTHORITY TRAINING PAAET ALARDYIA KUWAIT (1)
Rajaselvam J (1)	BASIC PUBLIC AUTHORITY TRAINING PAAET ALARDYIA KUWAIT (1)
Flanet raj Sr (1)	BASIC PUBLIC AUTHORITY TRAINING PAAET ALARDYIA KUWAIT (1)
Chang Sw (1)	BASIC PUBLIC AUTHORITY TRAINING PAAET ALARDYIA KUWAIT (1)
Almutairi Am (1)	BASIC PUBLIC AUTHORITY TRAINING PAAET ALARDYIA KUWAIT (1)
Alarjani Km (1)	BASIC PUBLIC AUTHORITY TRAINING PAAET ALARDYIA KUWAIT (1)
Voropaiev M (1)	BAYER DE (1)
Vertzoni M (1)	BAYER DE (1)
van der mey D (1)	BAYER DE (1)
Thumann Ta (1)	BAYER DE (1)
Reppas C (1)	BAYER DE (1)
Rabini S (1)	BAYER DE (1)
Pferschy-wenzig E-m (1)	BAYER DE (1)
Nock D (1)	BAYER DE (1)
Muenster U (1)	BAYER DE (1)
Moissl-eichinger C (1)	BAYER DE (1)
Kersten E (1)	BAYER DE (1)
Bauer R (1)	BAYER DE (1)
Aziz-kalbhenn H (1)	BAYER DE (1)
Ammar Rm (1)	BAYER DE (1)
Parseh I (1)	BEHBAHAN FAC BEHBAHAN IRAN (1)

Mousavi K (1)	BEHBAHAN FAC BEHBAHAN IRAN (1)
Karimi H (2)	BEHBAHAN FAC BEHBAHAN IRAN (1)
Hashemi M (1)	BEHBAHAN FAC BEHBAHAN IRAN (1)
Golbini mofrad M (1)	BEHBAHAN FAC BEHBAHAN IRAN (1)
Badieenejad A (1)	BEHBAHAN FAC BEHBAHAN IRAN (1)
Azadbakht O (1)	BEHBAHAN FAC BEHBAHAN IRAN (1)
Qiao X (1)	BEIJING KEY MEAT PROCESSING BEIJING CHINA (1)
Cheng X (1)	BEIJING KEY MEAT PROCESSING BEIJING CHINA (1)
Subedi S (1)	BINGHAMTON UNIV BINGHAMTON NY UNITED STATES (1)
Malik M (1)	BINGHAMTON UNIV BINGHAMTON NY UNITED STATES (1)
Limage R (1)	BINGHAMTON UNIV BINGHAMTON NY UNITED STATES (1)
Kolba N (1)	BINGHAMTON UNIV BINGHAMTON NY UNITED STATES (1)
Guo Z (1)	BINGHAMTON UNIV BINGHAMTON NY UNITED STATES (1)
Moreno-olivas F (2)	BINGHAMTON UNIV BINGHAMTON NY UNITED STATES (2)
Tako E (4)	BINGHAMTON UNIV BINGHAMTON NY UNITED STATES (3)
Marques Cnh (3)	BINGHAMTON UNIV BINGHAMTON NY UNITED STATES (3)
Mahler Gj (4)	BINGHAMTON UNIV BINGHAMTON NY UNITED STATES (4)
Maddox I (1)	BIOACTIVE RES NEW ZEALAND MT ALBERT AUCKLAND NEW ZEALAND (1)
Maddox Is (1)	BIOACTIVES RES NEW ZEALAND AUCKLAND NEW ZEALAND (1)
Stevens Y (1)	BIOACTOR B V MAASTRICHT GS NETHERLANDS (1)
Sost Mm (1)	BIOACTOR B V MAASTRICHT GS NETHERLANDS (1)
Ahles S (1)	BIOACTOR B V MAASTRICHT GS NETHERLANDS (1)
Van rymenant E (1)	BIOACTOR BV MAASTRICHT NETHERLANDS (1)
Salden B (1)	BIOACTOR BV MAASTRICHT NETHERLANDS (1)
Noten B (1)	BIOACTOR BV MAASTRICHT NETHERLANDS (1)
Saman P (1)	BIODIVERSITY RES CTR THAILAND INST SCIENTIFIC TECHNOLOGICAL RES PATHUM THANI THAILAND (1)
Phatvej W (1)	BIODIVERSITY RES CTR THAILAND INST SCIENTIFIC TECHNOLOGICAL RES PATHUM THANI THAILAND (1)
Moonmungmee S (1)	BIODIVERSITY RES CTR THAILAND INST SCIENTIFIC TECHNOLOGICAL RES PATHUM THANI THAILAND (1)
Kuancha C (1)	BIODIVERSITY RES CTR THAILAND INST SCIENTIFIC TECHNOLOGICAL RES PATHUM THANI THAILAND (1)
Dathong J (1)	BIODIVERSITY RES CTR THAILAND INST SCIENTIFIC TECHNOLOGICAL RES PATHUM THANI THAILAND (1)
Chatanon L (1)	BIODIVERSITY RES CTR THAILAND INST SCIENTIFIC TECHNOLOGICAL RES PATHUM THANI THAILAND (1)
Chaiongkarn A (1)	BIODIVERSITY RES CTR THAILAND INST SCIENTIFIC TECHNOLOGICAL RES PATHUM THANI THAILAND (1)
Pitsiladis A (1)	BIOGAIA AB MOBILVAGEN LUND SWEDEN (1)
Oscarsson E (1)	BIOGAIA AB MOBILVAGEN LUND SWEDEN (1)
Marefati A (1)	BIOGAIA AB MOBILVAGEN LUND SWEDEN (1)
Ilestam N (1)	BIOGAIA AB MOBILVAGEN LUND SWEDEN (1)
Bergenståhl B (1)	BIOGAIA AB MOBILVAGEN LUND SWEDEN (1)
Goel G (1)	BIOINFORMATICS JAYPEE UNIV INFORMATION WAKNAGHAT SOLAN INDIA (1)
Attri S (1)	BIOINFORMATICS JAYPEE UNIV INFORMATION WAKNAGHAT SOLAN INDIA (1)
Rattanamongkonkul S (1)	BIOMATHEMATICS GROUP VANDERBITT UNIV NASHVILLE TN UNITED STATES (1)
Rattanakul C (1)	BIOMATHEMATICS GROUP VANDERBITT UNIV NASHVILLE TN UNITED STATES (1)
Puttasontiphot T (1)	BIOMATHEMATICS GROUP VANDERBITT UNIV NASHVILLE TN UNITED STATES (1)
Lenbury Y (1)	BIOMATHEMATICS GROUP VANDERBITT UNIV NASHVILLE TN UNITED STATES (1)
Hotchkiss Jr (1)	BIOMATHEMATICS GROUP VANDERBITT UNIV NASHVILLE TN UNITED STATES (1)
Crooke Ps (1)	BIOMATHEMATICS GROUP VANDERBITT UNIV NASHVILLE TN UNITED STATES (1)
Fakhoury M (2)	BIOMEDICAL ARTIFICIAL CELLS ORGANS RES CTR MCGILL UNIV UNIV STREET MONTREAL QC H3A 2B4 CANADA (1)
Coussa-charley M (2)	BIOMEDICAL ARTIFICIAL CELLS ORGANS RES CTR MCGILL UNIV UNIV STREET MONTREAL QC H3A 2B4 CANADA (1)
Al-salami H (1)	BIOMEDICAL ARTIFICIAL CELLS ORGANS RES CTR MCGILL UNIV UNIV STREET MONTREAL QC H3A 2B4 CANADA (1)
Ouyang W (2)	BIOMEDICAL ARTIFICIAL ORGAN RES CTR MCGILL UNIV MONTREAL QUE CANADA (1)
Lim T (2)	BIOMEDICAL ARTIFICIAL ORGAN RES CTR MCGILL UNIV MONTREAL QUE CANADA (1)
Lawuyi B (2)	BIOMEDICAL ARTIFICIAL ORGAN RES CTR MCGILL UNIV MONTREAL QUE CANADA (1)

Afkhami F (2)	BIOMEDICAL ARTIFICIAL ORGAN RES CTR MCGILL UNIV MONTREAL QUE CANADA (1)
Sadeghi ekbatan S (1)	BIOMEDICAL DUFF BUILDING RUE UNIV MONTREAL QC H3A2B4 CANADA (1)
Ekbatan Ss (2)	BIOMEDICAL DUFF BUILDING RUE UNIV MONTREAL QC H3A2B4 CANADA (1)
Khairallah J (3)	BIOMEDICAL DUFF BUILDING RUE UNIV MONTREAL QC H3A2B4 CANADA (2)
Zum felde T (1)	BIOMEDICAL MCGILL UNIV UNIV STREET ROOM MONTREAL QC H3A 2B4 CANADA (1)
Melgar-bermudez E (1)	BIOMEDICAL MCGILL UNIV UNIV STREET ROOM MONTREAL QC H3A 2B4 CANADA (1)
Iskandar Km (1)	BIOMEDICAL MCGILL UNIV UNIV STREET ROOM MONTREAL QC H3A 2B4 CANADA (1)
How E (1)	BIOMEDICAL MCGILL UNIV UNIV STREET ROOM MONTREAL QC H3A 2B4 CANADA (1)
Burgos G (1)	BIOMEDICAL MCGILL UNIV UNIV STREET ROOM MONTREAL QC H3A 2B4 CANADA (1)
Shao W (1)	BIOMEDICAL THERAPY RES BIOMEDICAL ARTIFICIAL CELLS ORGANS RES CTR UNIV STREET MONTREAL QC H3A 2B4 CANADA (1)
Malhotra M (1)	BIOMEDICAL THERAPY RES BIOMEDICAL ARTIFICIAL CELLS ORGANS RES CTR UNIV STREET MONTREAL QC H3A 2B4 CANADA (1)
Kahouli I (1)	BIOMEDICAL THERAPY RES BIOMEDICAL ARTIFICIAL CELLS ORGANS RES CTR UNIV STREET MONTREAL QC H3A 2B4 CANADA (1)
Valderrama-rincón Jd (1)	BIOMEDICAL UNIV LOS ANDES BOGOTA DC UNITED STATES (1)
Reyes Lh (1)	BIOMEDICAL UNIV LOS ANDES BOGOTA DC UNITED STATES (1)
Pradilla D (1)	BIOMEDICAL UNIV LOS ANDES BOGOTA DC UNITED STATES (1)
Patarroyo Jl (1)	BIOMEDICAL UNIV LOS ANDES BOGOTA DC UNITED STATES (1)
Florez-rojas Js (1)	BIOMEDICAL UNIV LOS ANDES BOGOTA DC UNITED STATES (1)
Cruz Jc (1)	BIOMEDICAL UNIV LOS ANDES BOGOTA DC UNITED STATES (1)
Uhlig S (1)	BIOMIN HOLDING GMBH BIOMIN RES CTR TECHNOPARK TULLN AUSTRIA (1)
Schatzmayr D (1)	BIOMIN HOLDING GMBH BIOMIN RES CTR TECHNOPARK TULLN AUSTRIA (1)
Neckermann K (1)	BIOMIN HOLDING GMBH BIOMIN RES CTR TECHNOPARK TULLN AUSTRIA (1)
Gemmi C (1)	BIOMIN HOLDING GMBH BIOMIN RES CTR TECHNOPARK TULLN AUSTRIA (1)
Gathumbi J (1)	BIOMIN HOLDING GMBH BIOMIN RES CTR TECHNOPARK TULLN AUSTRIA (1)
De baere S (1)	BIOMIN HOLDING GMBH BIOMIN RES CTR TECHNOPARK TULLN AUSTRIA (1)
Croubels S (1)	BIOMIN HOLDING GMBH BIOMIN RES CTR TECHNOPARK TULLN AUSTRIA (1)
Claus G (1)	BIOMIN HOLDING GMBH BIOMIN RES CTR TECHNOPARK TULLN AUSTRIA (1)
Antonissen G (1)	BIOMIN HOLDING GMBH BIOMIN RES CTR TECHNOPARK TULLN AUSTRIA (1)
Prasitpuriprecha C (1)	BIOPHARMACY FAC UBON RATCHATHANI UNIV WARINCHAMRAP UBON RATCHATHANI THAILAND (1)
Kanchanatawee S (1)	BIOPHARMACY FAC UBON RATCHATHANI UNIV WARINCHAMRAP UBON RATCHATHANI THAILAND (1)
Jantama Ss (1)	BIOPHARMACY FAC UBON RATCHATHANI UNIV WARINCHAMRAP UBON RATCHATHANI THAILAND (1)
Jantama K (1)	BIOPHARMACY FAC UBON RATCHATHANI UNIV WARINCHAMRAP UBON RATCHATHANI THAILAND (1)
Charnchai P (1)	BIOPHARMACY FAC UBON RATCHATHANI UNIV WARINCHAMRAP UBON RATCHATHANI THAILAND (1)
Jorge Yf (1)	BIOPROCESOS UNIDAD PROFESIONAL INTERDISCIPLINARIA BIOTECNOLOGIA UPIBI INST POLITECNICO NACIONAL IPN AV ACUEDUCTO S N BARRIO LAGUNA TICOMAN MEXICO DF CP07340 MEXICO (1)
Humberto H-s (1)	BIOPROCESOS UNIDAD PROFESIONAL INTERDISCIPLINARIA BIOTECNOLOGIA UPIBI INST POLITECNICO NACIONAL IPN AV ACUEDUCTO S N BARRIO LAGUNA TICOMAN MEXICO DF CP07340 MEXICO (1)
Diana C-r (1)	BIOPROCESOS UNIDAD PROFESIONAL INTERDISCIPLINARIA BIOTECNOLOGIA UPIBI INST POLITECNICO NACIONAL IPN AV ACUEDUCTO S N BARRIO LAGUNA TICOMAN MEXICO DF CP07340 MEXICO (1)
Thomson P (1)	BIOPROCESS PONTIFICIA UNIV CATOLICA CHILE CHILE (1)
Pinto F (1)	BIOPROCESS PONTIFICIA UNIV CATOLICA CHILE CHILE (1)
Gutiérrez N (1)	BIOPROCESS PONTIFICIA UNIV CATOLICA CHILE CHILE (1)
Garridoa D (1)	BIOPROCESS PONTIFICIA UNIV CATOLICA CHILE CHILE (1)
Ortúzar V (2)	BIOPROCESS PONTIFICIA UNIV CATOLICA CHILE CHILE (2)
Medina Da (3)	BIOPROCESS PONTIFICIA UNIV CATOLICA CHILE CHILE (2)
Garrido D (2)	BIOPROCESS PONTIFICIA UNIV CATOLICA CHILE CHILE (2)
Pacheco Kc (1)	BIOPROCESS UPIBI NAT POLYTECHNIC INST IPN AVE ACUEDUCTO S N COL BARRIO LAGUNA TICOMAN DISTRITO FEDERAL MEXICO (1)

Martínez Fr (1)	BIOPROCESS UPIBI NAT POLYTECHNIC INST IPN AVE ACUEDUCTO S N COL BARRIO LAGUNA TICOMAN DISTRITO FEDERAL MEXICO (1)
Durán-páramo E (1)	BIOPROCESS UPIBI NAT POLYTECHNIC INST IPN AVE ACUEDUCTO S N COL BARRIO LAGUNA TICOMAN DISTRITO FEDERAL MEXICO (1)
del toro Gv (1)	BIOPROCESS UPIBI NAT POLYTECHNIC INST IPN AVE ACUEDUCTO S N COL BARRIO LAGUNA TICOMAN DISTRITO FEDERAL MEXICO (1)
Suman T (1)	BIOPROSPECTING BHARATHIAR UNIV COIMBATORE TAMIL NADU INDIA (1)
Sreeja Ps (1)	BIOPROSPECTING BHARATHIAR UNIV COIMBATORE TAMIL NADU INDIA (1)
Saikumar S (1)	BIOPROSPECTING BHARATHIAR UNIV COIMBATORE TAMIL NADU INDIA (1)
Parimelazhagan T (1)	BIOPROSPECTING BHARATHIAR UNIV COIMBATORE TAMIL NADU INDIA (1)
Manikandan A (1)	BIOPROSPECTING BHARATHIAR UNIV COIMBATORE TAMIL NADU INDIA (1)
Kasipandi M (1)	BIOPROSPECTING BHARATHIAR UNIV COIMBATORE TAMIL NADU INDIA (1)
Dhivya S (1)	BIOPROSPECTING BHARATHIAR UNIV COIMBATORE TAMIL NADU INDIA (1)
Vyas Brm (1)	BIOSCIENCES SAURASHTRA UNIV RAJKOT GUJARAT INDIA (1)
Pithva Sp (1)	BIOSCIENCES SAURASHTRA UNIV RAJKOT GUJARAT INDIA (1)
Dave Jm (1)	BIOSCIENCES SAURASHTRA UNIV RAJKOT GUJARAT INDIA (1)
Zawadzki W (1)	BIOSTRUCTURE FAC MEDICINE WROCLAW UNIV LIFE SMOLUCHOWSKIEGO WROCLAW POLAND (1)
Witkowska Z (1)	BIOSTRUCTURE FAC MEDICINE WROCLAW UNIV LIFE SMOLUCHOWSKIEGO WROCLAW POLAND (1)
Toker Ny (1)	BIOSTRUCTURE FAC MEDICINE WROCLAW UNIV LIFE SMOLUCHOWSKIEGO WROCLAW POLAND (1)
Pecka E (1)	BIOSTRUCTURE FAC MEDICINE WROCLAW UNIV LIFE SMOLUCHOWSKIEGO WROCLAW POLAND (1)
Opalinski S (1)	BIOSTRUCTURE FAC MEDICINE WROCLAW UNIV LIFE SMOLUCHOWSKIEGO WROCLAW POLAND (1)
Janeczek M (1)	BIOSTRUCTURE FAC MEDICINE WROCLAW UNIV LIFE SMOLUCHOWSKIEGO WROCLAW POLAND (1)
Czerski A (1)	BIOSTRUCTURE FAC MEDICINE WROCLAW UNIV LIFE SMOLUCHOWSKIEGO WROCLAW POLAND (1)
Chroszcz A (1)	BIOSTRUCTURE FAC MEDICINE WROCLAW UNIV LIFE SMOLUCHOWSKIEGO WROCLAW POLAND (1)
Chojnacka K (1)	BIOSTRUCTURE FAC MEDICINE WROCLAW UNIV LIFE SMOLUCHOWSKIEGO WROCLAW POLAND (1)
Sadaghian sadabad M (1)	BIOSYSTEMS GHENT UNIV COUPURE LINKS B GENT BELGIUM (1)
Pieters J (1)	BIOSYSTEMS GHENT UNIV COUPURE LINKS B GENT BELGIUM (1)
Hennebel T (1)	BIOSYSTEMS GHENT UNIV COUPURE LINKS B GENT BELGIUM (1)
Harmsen Hj (1)	BIOSYSTEMS GHENT UNIV COUPURE LINKS B GENT BELGIUM (1)
Derycke L (1)	BIOSYSTEMS GHENT UNIV COUPURE LINKS B GENT BELGIUM (1)
De ryck T (2)	BIOSYSTEMS GHENT UNIV COUPURE LINKS B GENT BELGIUM (1)
Bracke M (1)	BIOSYSTEMS GHENT UNIV COUPURE LINKS B GENT BELGIUM (1)
Skenderidis P (1)	BIOSYSTEMS UNIV THESSALY LARISSA GREECE (1)
Petrotos K (1)	BIOSYSTEMS UNIV THESSALY LARISSA GREECE (1)
Mitsagga C (1)	BIOSYSTEMS UNIV THESSALY LARISSA GREECE (1)
Lampakis D (1)	BIOSYSTEMS UNIV THESSALY LARISSA GREECE (1)
Giavasis I (1)	BIOSYSTEMS UNIV THESSALY LARISSA GREECE (1)
Wavreille J (1)	BREEDING QUALITY PRODUCTION WELFARE PRODUCTION SECTORS WALLOON RES CTR RUE LIROUX GEMBLOUX B BELGIUM (1)
Froidmont E (1)	BREEDING QUALITY PRODUCTION WELFARE PRODUCTION SECTORS WALLOON RES CTR RUE LIROUX GEMBLOUX B BELGIUM (1)
Didelez M (1)	BREEDING QUALITY PRODUCTION WELFARE PRODUCTION SECTORS WALLOON RES CTR RUE LIROUX GEMBLOUX B BELGIUM (1)
Yaskolka meir A (1)	BRIGHAM WOMENS HOSPITAL US (1)
Worley Jn (1)	BRIGHAM WOMENS HOSPITAL US (1)
Trivedi Mk (1)	BRIGHAM WOMENS HOSPITAL US (1)
Tovaglieri A (1)	BRIGHAM WOMENS HOSPITAL US (1)
Swenor B (1)	BRIGHAM WOMENS HOSPITAL US (1)
Sun Q (1)	BRIGHAM WOMENS HOSPITAL US (1)
Sonenshein Al (1)	BRIGHAM WOMENS HOSPITAL US (1)
Peltier J (1)	BRIGHAM WOMENS HOSPITAL US (1)
Onderdonk Ab (1)	BRIGHAM WOMENS HOSPITAL US (1)
Novak R (1)	BRIGHAM WOMENS HOSPITAL US (1)
Nestor B (1)	BRIGHAM WOMENS HOSPITAL US (1)
Lin Y-f (1)	BRIGHAM WOMENS HOSPITAL US (1)
Levy O (1)	BRIGHAM WOMENS HOSPITAL US (1)

Lavin R (1)	BRIGHAM WOMENS HOSPITAL US (1)
Kasper Dl (1)	BRIGHAM WOMENS HOSPITAL US (1)
Jha A (1)	BRIGHAM WOMENS HOSPITAL US (1)
Jalili-firoozinezhad S (1)	BRIGHAM WOMENS HOSPITAL US (1)
Ingber De (1)	BRIGHAM WOMENS HOSPITAL US (1)
Immanuel Src (1)	BRIGHAM WOMENS HOSPITAL US (1)
Hoffman M (1)	BRIGHAM WOMENS HOSPITAL US (1)
Heinig U (1)	BRIGHAM WOMENS HOSPITAL US (1)
Haange S-b (1)	BRIGHAM WOMENS HOSPITAL US (1)
Gregory Ke (1)	BRIGHAM WOMENS HOSPITAL US (1)
Gonzalez-escalona N (1)	BRIGHAM WOMENS HOSPITAL US (1)
Girinathan Bp (1)	BRIGHAM WOMENS HOSPITAL US (1)
Gerber Gk (1)	BRIGHAM WOMENS HOSPITAL US (1)
Gazzaniga Fs (1)	BRIGHAM WOMENS HOSPITAL US (1)
García-romero Mt (1)	BRIGHAM WOMENS HOSPITAL US (1)
Fadel Cw (1)	BRIGHAM WOMENS HOSPITAL US (1)
Dupuy B (1)	BRIGHAM WOMENS HOSPITAL US (1)
Dimaano L (1)	BRIGHAM WOMENS HOSPITAL US (1)
Dibenedetto N (1)	BRIGHAM WOMENS HOSPITAL US (1)
Delaney Ml (1)	BRIGHAM WOMENS HOSPITAL US (1)
Cummins Ck (1)	BRIGHAM WOMENS HOSPITAL US (1)
Cronce Mj (1)	BRIGHAM WOMENS HOSPITAL US (1)
Colt M (1)	BRIGHAM WOMENS HOSPITAL US (1)
Chang Y-s (1)	BRIGHAM WOMENS HOSPITAL US (1)
Camacho Dm (1)	BRIGHAM WOMENS HOSPITAL US (1)
Calamari El (1)	BRIGHAM WOMENS HOSPITAL US (1)
Cabral Jms (1)	BRIGHAM WOMENS HOSPITAL US (1)
Bry L (1)	BRIGHAM WOMENS HOSPITAL US (1)
Bein A (1)	BRIGHAM WOMENS HOSPITAL US (1)
Baliga Ns (1)	BRIGHAM WOMENS HOSPITAL US (1)
Arrieta-ortiz Ml (1)	BRIGHAM WOMENS HOSPITAL US (1)
Arapitsas P (1)	BRIGHAM WOMENS HOSPITAL US (1)
Allard M (1)	BRIGHAM WOMENS HOSPITAL US (1)
Aharoni A (1)	BRIGHAM WOMENS HOSPITAL US (1)
Mallett Ak (1)	BRITISH INDUSTRIAL RES ASSOCIATION WOODMANSTERNE ROAD CARSHALTON SURREY SM5 4DS UNITED KINGDOM (1)
Coutts Tm (1)	BRITISH INDUSTRIAL RES ASSOCIATION WOODMANSTERNE ROAD CARSHALTON SURREY SM5 4DS UNITED KINGDOM (1)
Brennan-craddock We (1)	BRITISH INDUSTRIAL RES ASSOCIATION WOODMANSTERNE ROAD CARSHALTON SURREY SM5 4DS UNITED KINGDOM (1)
Bearne Ca (1)	BRITISH INDUSTRIAL RES ASSOCIATION WOODMANSTERNE ROAD CARSHALTON SURREY SM5 4DS UNITED KINGDOM (1)
Alldrick Aj (1)	BRITISH INDUSTRIAL RES ASSOCIATION WOODMANSTERNE ROAD CARSHALTON SURREY SM5 4DS UNITED KINGDOM (1)
Rowland Ir (5)	BRITISH INDUSTRIAL RES ASSOCIATION WOODMANSTERNE ROAD CARSHALTON SURREY SM5 4DS UNITED KINGDOM (2)
Zheng T (1)	BRUNSWICK US (1)
Zhao D (2)	BRUNSWICK US (1)
Wu Q-l (1)	BRUNSWICK US (1)
Westfall S (1)	BRUNSWICK US (1)
Smith B (1)	BRUNSWICK US (1)
Simon J (1)	BRUNSWICK US (1)
Shen L (1)	BRUNSWICK US (1)
Schaffner Dw (1)	BRUNSWICK US (1)
Pasinetti Gm (1)	BRUNSWICK US (1)
Pan Y (3)	BRUNSWICK US (1)
Niedzwiedzka Km (1)	BRUNSWICK US (1)
Merchant Ha (1)	BRUNSWICK US (1)
Liu Q (3)	BRUNSWICK US (1)
Estill M (1)	BRUNSWICK US (1)
Dodoo C (1)	BRUNSWICK US (1)
De sales Cv (1)	BRUNSWICK US (1)
De melo Anf (1)	BRUNSWICK US (1)
Chatterjee R (1)	BRUNSWICK US (1)
Carracci F (1)	BRUNSWICK US (1)

Gaisford S (2)	BRUNSWICK US (2)
Mehta D (1)	BUBECK SCIENTIFIC RAINBOW DRIVE #9418 LIVINGSTON TX UNITED STATES (1)
Desouza A (1)	BUBECK SCIENTIFIC RAINBOW DRIVE #9418 LIVINGSTON TX UNITED STATES (1)
Bubeck Ss (1)	BUBECK SCIENTIFIC RAINBOW DRIVE #9418 LIVINGSTON TX UNITED STATES (1)
Bubeck S (1)	BUBECK SCIENTIFIC RAINBOW DRIVE #9418 LIVINGSTON TX UNITED STATES (1)
Young A (2)	BUBECK SCIENTIFIC RAINBOW DRIVE #9418 LIVINGSTON TX UNITED STATES (2)
Bayne T (2)	BUBECK SCIENTIFIC RAINBOW DRIVE #9418 LIVINGSTON TX UNITED STATES (2)
Rengarajan S (1)	CAMPUS ALAGAPPA UNIV KARAIKUDI TAMIL NADU INDIA (1)
Palanivel R (1)	CAMPUS ALAGAPPA UNIV KARAIKUDI TAMIL NADU INDIA (1)
Vrolijk Mf (1)	CAMPUS VENLO MAASTRICHT UNIV VENLO NETHERLANDS (1)
Van leeuwen S (1)	CAMPUS VENLO MAASTRICHT UNIV VENLO NETHERLANDS (1)
Van der lugt T (1)	CAMPUS VENLO MAASTRICHT UNIV VENLO NETHERLANDS (1)
Opperhuizen A (1)	CAMPUS VENLO MAASTRICHT UNIV VENLO NETHERLANDS (1)
Moore Ji (1)	CANADIAN INST RES TORONTO CANADA (1)
Mack D (1)	CANADIAN INST RES TORONTO CANADA (1)
Chiang C-k (1)	CANADIAN INST RES TORONTO CANADA (1)
Stintzi A (3)	CANADIAN INST RES TORONTO CANADA (2)
Mayne J (3)	CANADIAN INST RES TORONTO CANADA (2)
Van immerseel F (1)	CANCER RES GHENT UNIV PINTELAAN B GENT BELGIUM (1)
Roos S (1)	CANCER RES GHENT UNIV PINTELAAN B GENT BELGIUM (1)
Mackenzie Da (1)	CANCER RES GHENT UNIV PINTELAAN B GENT BELGIUM (1)
Derde M (2)	CANCER RES GHENT UNIV PINTELAAN B GENT BELGIUM (1)
Vanhoecke B (3)	CANCER RES GHENT UNIV PINTELAAN B GENT BELGIUM (2)
White T (1)	CAPSULES INGREDIENTS LONZA INC MORRISTOWN NJ UNITED STATES (1)
Van mele M (1)	CAPSULES INGREDIENTS LONZA INC MORRISTOWN NJ UNITED STATES (1)
Jannin V (1)	CAPSULES INGREDIENTS LONZA INC MORRISTOWN NJ UNITED STATES (1)
Fowler K (1)	CAPSULES INGREDIENTS LONZA INC MORRISTOWN NJ UNITED STATES (1)
Durkee S (1)	CAPSULES INGREDIENTS LONZA INC MORRISTOWN NJ UNITED STATES (1)
Bellamine A (1)	CAPSULES INGREDIENTS LONZA INC MORRISTOWN NJ UNITED STATES (1)
Vermeiren J (3)	CARGILL US (1)
Parkhill J (1)	CARGILL US (1)
Louis P (1)	CARGILL US (1)
Flint Hj (3)	CARGILL US (1)
Fässler C (1)	CARGILL US (1)
Duncan Sh (3)	CARGILL US (1)
Chung Wsf (1)	CARGILL US (1)
Brouns F (1)	CARGILL US (1)
Bosscher D (1)	CARGILL US (1)
Arrigoni E (1)	CARGILL US (1)
Amadò R (1)	CARGILL US (1)
Silva J (1)	CATHOLIC UNIV PORTO PORTO PORTUGAL (1)
Ramalheira R (1)	CATHOLIC UNIV PORTO PORTO PORTUGAL (1)
Azeredo J (1)	CATHOLIC UNIV PORTO PORTO PORTUGAL (1)
Almeida G (1)	CATHOLIC UNIV PORTO PORTO PORTUGAL (1)
Verhoeckx K (1)	CEA FR (1)
Untersmayr E (1)	CEA FR (1)
Turner P (1)	CEA FR (1)
Smit J (1)	CEA FR (1)
Larré C (1)	CEA FR (1)
Gadermaier G (1)	CEA FR (1)
Epstein Mm (1)	CEA FR (1)
Bøgh Kl (1)	CEA FR (1)
Adel-patient K (1)	CEA FR (1)
Silvério Sc (2)	CEB CTR UNIVERSIDADE DO MINHO CAMPUS GUALTAR BRAGA PORTUGAL (2)
Rodrigues Lr (2)	CEB CTR UNIVERSIDADE DO MINHO CAMPUS GUALTAR BRAGA PORTUGAL (2)
Pereira Ma (3)	CEB CTR UNIVERSIDADE DO MINHO CAMPUS GUALTAR BRAGA PORTUGAL (2)
Cardoso Bb (2)	CEB CTR UNIVERSIDADE DO MINHO CAMPUS GUALTAR BRAGA PORTUGAL (2)
Amorim C (2)	CEB CTR UNIVERSIDADE DO MINHO CAMPUS GUALTAR BRAGA PORTUGAL (2)
Alves Ji (2)	CEB CTR UNIVERSIDADE DO MINHO CAMPUS GUALTAR BRAGA PORTUGAL (2)
Kim H-j (1)	CENTRAL AREA CROP NAT INST CROP RURAL DEVELOPMENT ADMINISTRATION SUWON SOUTH KOREA (1)
Silva Mj (1)	CESAM CTR MARINE STUDIES UNIV AVEIRO CAMPUS UNIVERSITARIO SANTIAGO AVEIRO PORTUGAL (1)
Rolo D (1)	CESAM CTR MARINE STUDIES UNIV AVEIRO CAMPUS UNIVERSITARIO SANTIAGO AVEIRO PORTUGAL (1)

Louro H (1)	CESAM CTR MARINE STUDIES UNIV AVEIRO CAMPUS UNIVERSITARIO SANTIAGO AVEIRO PORTUGAL (1)
Gramacho Ac (1)	CESAM CTR MARINE STUDIES UNIV AVEIRO CAMPUS UNIVERSITARIO SANTIAGO AVEIRO PORTUGAL (1)
Gonçalves Lm (1)	CESAM CTR MARINE STUDIES UNIV AVEIRO CAMPUS UNIVERSITARIO SANTIAGO AVEIRO PORTUGAL (1)
Bettencourt A (1)	CESAM CTR MARINE STUDIES UNIV AVEIRO CAMPUS UNIVERSITARIO SANTIAGO AVEIRO PORTUGAL (1)
León-camacho M (1)	CHARACTERIZATION QUALITY INST GRASA SPANISH NAT RES SEVILLE SPAIN (1)
Lavado G (1)	CHARACTERIZATION QUALITY INST GRASA SPANISH NAT RES SEVILLE SPAIN (1)
Higuero N (1)	CHARACTERIZATION QUALITY INST GRASA SPANISH NAT RES SEVILLE SPAIN (1)
Cava R (1)	CHARACTERIZATION QUALITY INST GRASA SPANISH NAT RES SEVILLE SPAIN (1)
Wiertsema S (1)	CHILDREN MEDICAL CENTER US (1)
Weinstein Da (1)	CHILDREN MEDICAL CENTER US (1)
Wehkamp T (1)	CHILDREN MEDICAL CENTER US (1)
Varasteh S (1)	CHILDREN MEDICAL CENTER US (1)
Van't land B (1)	CHILDREN MEDICAL CENTER US (1)
van wandelen Mtr (1)	CHILDREN MEDICAL CENTER US (1)
van rijn M (1)	CHILDREN MEDICAL CENTER US (1)
van ark I (1)	CHILDREN MEDICAL CENTER US (1)
Toutounchi Ns (1)	CHILDREN MEDICAL CENTER US (1)
Tims S (2)	CHILDREN MEDICAL CENTER US (1)
Smit Gpa (1)	CHILDREN MEDICAL CENTER US (1)
Schwartz Ivd (1)	CHILDREN MEDICAL CENTER US (1)
Perry Ids (1)	CHILDREN MEDICAL CENTER US (1)
Nalin T (1)	CHILDREN MEDICAL CENTER US (1)
Leusink-muis T (1)	CHILDREN MEDICAL CENTER US (1)
Kraneveld Ad (1)	CHILDREN MEDICAL CENTER US (1)
Hogenkamp A (1)	CHILDREN MEDICAL CENTER US (1)
Garssen J (1)	CHILDREN MEDICAL CENTER US (1)
Folkerts G (1)	CHILDREN MEDICAL CENTER US (1)
Derks Tgj (1)	CHILDREN MEDICAL CENTER US (1)
de souza Cfm (1)	CHILDREN MEDICAL CENTER US (1)
Cai Y (2)	CHILDREN MEDICAL CENTER US (1)
Braber S (1)	CHILDREN MEDICAL CENTER US (1)
Si G (1)	CHINESE PLA BEIJING CHINA (1)
Ren R (1)	CHINESE PLA BEIJING CHINA (1)
Liang Z (1)	CHINESE PLA BEIJING CHINA (1)
Fan B (1)	CHINESE PLA BEIJING CHINA (1)
Bai S (1)	CHINESE PLA BEIJING CHINA (1)
Kim Bk (1)	CHUNG ANG UNIV ANSEONG SOUTH KOREA (1)
Kang Jh (1)	CHUNG ANG UNIV ANSEONG SOUTH KOREA (1)
Kang Hj (1)	CHUNG ANG UNIV ANSEONG SOUTH KOREA (1)
Jang Hw (1)	CHUNG ANG UNIV ANSEONG SOUTH KOREA (1)
Cho Mg (1)	CHUNG ANG UNIV ANSEONG SOUTH KOREA (1)
Lee Sy (3)	CHUNG ANG UNIV ANSEONG SOUTH KOREA (2)
Lee Dy (2)	CHUNG ANG UNIV ANSEONG SOUTH KOREA (2)
Hur Sj (3)	CHUNG ANG UNIV ANSEONG SOUTH KOREA (3)
Han Ns (3)	CHUNGBUK NAT UNIV CHEONGJU SOUTH KOREA (3)
Zorraquín-penìa I (1)	CIBER PUBLIC MADRID SPAIN (1)
Victoria moreno-arribas M (1)	CIBER PUBLIC MADRID SPAIN (1)
González de llano D (2)	CIBER PUBLIC MADRID SPAIN (1)
Theoduloz C (1)	CIENCIAS BASICAS BIOMEDICAS FACULTAD CIENCIAS SALUD UNIV TALCA TALCA CHILE (1)
Jiménez-aspee F (1)	CIENCIAS BASICAS BIOMEDICAS FACULTAD CIENCIAS SALUD UNIV TALCA TALCA CHILE (1)
Mendes E (1)	CIIMAR UNIVERSIDADE DO PORTO RUA DOS BRAGAS PORTO PORTUGAL (1)
Maulvault Al (1)	CIIMAR UNIVERSIDADE DO PORTO RUA DOS BRAGAS PORTO PORTUGAL (1)
Marques A (1)	CIIMAR UNIVERSIDADE DO PORTO RUA DOS BRAGAS PORTO PORTUGAL (1)
Cunha Sc (1)	CIIMAR UNIVERSIDADE DO PORTO RUA DOS BRAGAS PORTO PORTUGAL (1)
Cruz R (1)	CIIMAR UNIVERSIDADE DO PORTO RUA DOS BRAGAS PORTO PORTUGAL (1)
Casal S (1)	CIIMAR UNIVERSIDADE DO PORTO RUA DOS BRAGAS PORTO PORTUGAL (1)
Verthé K (2)	CLIN I GHENT UNIV COUPURE LINKS GHENT BELGIUM (1)
Vaneechoutte M (1)	CLIN I GHENT UNIV COUPURE LINKS GHENT BELGIUM (1)
Yolou Fs (1)	CNRS FR (1)

Wolowczuk I (1)	CNRS FR (1)
Walter T (1)	CNRS FR (1)
Waligora-dupriet A-j (1)	CNRS FR (1)
Vergoignan C (1)	CNRS FR (1)
Vandekerkove P (1)	CNRS FR (1)
Vandamme T (1)	CNRS FR (1)
Vacon Fl (1)	CNRS FR (1)
Toutain B (1)	CNRS FR (1)
Tenailleau E (1)	CNRS FR (1)
Temple-boyer P (1)	CNRS FR (1)
Sugier D (1)	CNRS FR (1)
Sillam-dussès D (1)	CNRS FR (1)
Seczyk L (1)	CNRS FR (1)
Sarkar A (1)	CNRS FR (1)
Sanz Y (3)	CNRS FR (1)
Salles C (1)	CNRS FR (1)
Sakai Y (1)	CNRS FR (1)
Rousseau F (1)	CNRS FR (1)
Robins Rj (1)	CNRS FR (1)
Rhimi M (1)	CNRS FR (1)
Pot B (1)	CNRS FR (1)
Peucelle V (1)	CNRS FR (1)
Péry Arr (1)	CNRS FR (1)
Pasc A (1)	CNRS FR (1)
Paolesse R (1)	CNRS FR (1)
Ouattara Da (1)	CNRS FR (1)
Oliveira Sd (1)	CNRS FR (1)
O'donohue M (1)	CNRS FR (1)
Nazih H (1)	CNRS FR (1)
Muñoz-tamayo R (1)	CNRS FR (1)
Mosser J (1)	CNRS FR (1)
Monnier A (1)	CNRS FR (1)
Mielle P (1)	CNRS FR (1)
Michel C (1)	CNRS FR (1)
Miambi E (1)	CNRS FR (1)
Medina M (1)	CNRS FR (1)
Martin L (1)	CNRS FR (1)
Mariadassou M (1)	CNRS FR (1)
Mangin I (1)	CNRS FR (1)
Maguin E (1)	CNRS FR (1)
Lvova L (1)	CNRS FR (1)
Luu T-h (1)	CNRS FR (1)
Lehrter V (1)	CNRS FR (1)
Leclerc M (1)	CNRS FR (1)
Le treut A (1)	CNRS FR (1)
Lazuka A (1)	CNRS FR (1)
Laroche B (1)	CNRS FR (1)
Kroell F (1)	CNRS FR (1)
Kozlowski F (1)	CNRS FR (1)
Kolodziej B (1)	CNRS FR (1)
Kedzia B (1)	CNRS FR (1)
Izquierdo E (1)	CNRS FR (1)
Hernandez-raquet G (1)	CNRS FR (1)
Henry M (1)	CNRS FR (1)
Henry G (1)	CNRS FR (1)
Haffner Fb (1)	CNRS FR (1)
Grundy Mm-l (1)	CNRS FR (1)
Grangette C (1)	CNRS FR (1)
Gevrenova R (1)	CNRS FR (1)
Gbassi Gk (1)	CNRS FR (1)
Galibert M-d (1)	CNRS FR (1)
Feron G (1)	CNRS FR (1)
Fergelot P (1)	CNRS FR (1)
Ennahar S (1)	CNRS FR (1)
Emily M (1)	CNRS FR (1)

Edwards C (1)	CNRS FR (1)
Dirson E (1)	CNRS FR (1)
Di natale C (1)	CNRS FR (1)
Dellarosa N (1)	CNRS FR (1)
Choi S-h (1)	CNRS FR (1)
Chicault C (1)	CNRS FR (1)
Chernetskyy M (1)	CNRS FR (1)
Capozzi F (1)	CNRS FR (1)
Brochot C (1)	CNRS FR (1)
Briard-bion V (1)	CNRS FR (1)
Bourriaud C (1)	CNRS FR (1)
Bourlieu-lacanal C (1)	CNRS FR (1)
Bobin-dubigeon C (2)	CNRS FR (1)
Bellanger A (1)	CNRS FR (1)
Barra A (1)	CNRS FR (1)
Bard J-m (1)	CNRS FR (1)
Auer L (1)	CNRS FR (1)
Aubry M (1)	CNRS FR (1)
Alard J (1)	CNRS FR (1)
Abraham A-l (1)	CNRS FR (1)
Marchioni E (2)	CNRS FR (2)
Kirkhus B (2)	CNRS FR (2)
De oliveira Sc (2)	CNRS FR (2)
Boutrou R (2)	CNRS FR (2)
Bohn T (3)	CNRS FR (2)
Ballet N (2)	CNRS FR (2)
Ballance S (2)	CNRS FR (2)
Golding M (3)	CNRS FR (3)
Deglaire A (3)	CNRS FR (3)
Carrière F (5)	CNRS FR (5)
Trivedi Hm (1)	COLGATE PALMOLIVE US (1)
Teughels W (1)	COLGATE PALMOLIVE US (1)
Slomka V (1)	COLGATE PALMOLIVE US (1)
Quirynen M (1)	COLGATE PALMOLIVE US (1)
Herrero Er (1)	COLGATE PALMOLIVE US (1)
Daep C (1)	COLGATE PALMOLIVE US (1)
Bernaerts K (1)	COLGATE PALMOLIVE US (1)
Zhu W (1)	COLLABORATIVE RES BIOACTIVE BIOMARKERS CRIBB GROUP UNIV CANBERRA CANBERRA ACT AUSTRALIA (1)
Naumovski N (1)	COLLABORATIVE RES BIOACTIVE BIOMARKERS CRIBB GROUP UNIV CANBERRA CANBERRA ACT AUSTRALIA (1)
Lyu F (1)	COLLABORATIVE RES BIOACTIVE BIOMARKERS CRIBB GROUP UNIV CANBERRA CANBERRA ACT AUSTRALIA (1)
Rajabi-siahboomi Ar (1)	COLORCON INC GLOBAL HEADQUARTERS RUTH ROAD HARLEYSVILLE PA UNITED STATES (1)
Mehta Ry (1)	COLORCON INC GLOBAL HEADQUARTERS RUTH ROAD HARLEYSVILLE PA UNITED STATES (1)
Levina M (1)	COLORCON INC GLOBAL HEADQUARTERS RUTH ROAD HARLEYSVILLE PA UNITED STATES (1)
Ferrizzi D (1)	COLORCON INC GLOBAL HEADQUARTERS RUTH ROAD HARLEYSVILLE PA UNITED STATES (1)
Van de velde F (1)	CONSEJO NACIONAL INVESTIGACIONES CIENTIFICAS TECNICAS CONICET SANTIAGO DEL ESTERO SANTA FE ARGENTINA (1)
Pirovani Me (1)	CONSEJO NACIONAL INVESTIGACIONES CIENTIFICAS TECNICAS CONICET SANTIAGO DEL ESTERO SANTA FE ARGENTINA (1)
Drago Sr (1)	CONSEJO NACIONAL INVESTIGACIONES CIENTIFICAS TECNICAS CONICET SANTIAGO DEL ESTERO SANTA FE ARGENTINA (1)
López-de-dicastillo C (1)	CONTAMINATION LAB INST AGROCHEMISTRY FOOD IATA CSIC AV AGUSTIN ESCARDINO PATERNA SPAIN (1)
López-carballo G (1)	CONTAMINATION LAB INST AGROCHEMISTRY FOOD IATA CSIC AV AGUSTIN ESCARDINO PATERNA SPAIN (1)
Hernández muñoz P (1)	CONTAMINATION LAB INST AGROCHEMISTRY FOOD IATA CSIC AV AGUSTIN ESCARDINO PATERNA SPAIN (1)
Gavara R (1)	CONTAMINATION LAB INST AGROCHEMISTRY FOOD IATA CSIC AV AGUSTIN ESCARDINO PATERNA SPAIN (1)

Subirade M (1)	COOPERATIVE AGROPUR RUE ARMAND FRAPPIER ST HUBERT QUEBEC J3Z 1G5 CANADA (1)
Remondetto Ge (1)	COOPERATIVE AGROPUR RUE ARMAND FRAPPIER ST HUBERT QUEBEC J3Z 1G5 CANADA (1)
Gomaa A (1)	COOPERATIVE AGROPUR RUE ARMAND FRAPPIER ST HUBERT QUEBEC J3Z 1G5 CANADA (1)
Gaudreau H (1)	COOPERATIVE AGROPUR RUE ARMAND FRAPPIER ST HUBERT QUEBEC J3Z 1G5 CANADA (1)
Champagne Cp (1)	COOPERATIVE AGROPUR RUE ARMAND FRAPPIER ST HUBERT QUEBEC J3Z 1G5 CANADA (1)
Sánchez-burgos Ja (1)	COORDINACION TECNOLOGIA ALIMENTOS ORIGEN VEGETAL CTR INVESTIGACION EN ALIMENTACION DESARROLLO CIAD HERMOSILLO SONORA MEXICO (1)
Hernández-maldonado Lm (1)	COORDINACION TECNOLOGIA ALIMENTOS ORIGEN VEGETAL CTR INVESTIGACION EN ALIMENTACION DESARROLLO CIAD HERMOSILLO SONORA MEXICO (1)
Del juncal-guzmán D (1)	COORDINACION TECNOLOGIA ALIMENTOS ORIGEN VEGETAL CTR INVESTIGACION EN ALIMENTACION DESARROLLO CIAD HERMOSILLO SONORA MEXICO (1)
Jacobs H (2)	COSUCRA WARCOING BELGIUM (2)
Xavier Bb (1)	CRITICAL CARE MEDICINE ANTWERP UNIV HOSP PHARMACOTHERAPY UNIV ANTWERP EDEGEM BELGIUM (1)
Verdickt E (1)	CRITICAL CARE MEDICINE ANTWERP UNIV HOSP PHARMACOTHERAPY UNIV ANTWERP EDEGEM BELGIUM (1)
van nuijs Aln (1)	CRITICAL CARE MEDICINE ANTWERP UNIV HOSP PHARMACOTHERAPY UNIV ANTWERP EDEGEM BELGIUM (1)
Mortelé O (1)	CRITICAL CARE MEDICINE ANTWERP UNIV HOSP PHARMACOTHERAPY UNIV ANTWERP EDEGEM BELGIUM (1)
Malhotra-kumar S (1)	CRITICAL CARE MEDICINE ANTWERP UNIV HOSP PHARMACOTHERAPY UNIV ANTWERP EDEGEM BELGIUM (1)
Lammens C (1)	CRITICAL CARE MEDICINE ANTWERP UNIV HOSP PHARMACOTHERAPY UNIV ANTWERP EDEGEM BELGIUM (1)
Jorens Pg (1)	CRITICAL CARE MEDICINE ANTWERP UNIV HOSP PHARMACOTHERAPY UNIV ANTWERP EDEGEM BELGIUM (1)
Iturrospe E (1)	CRITICAL CARE MEDICINE ANTWERP UNIV HOSP PHARMACOTHERAPY UNIV ANTWERP EDEGEM BELGIUM (1)
Sazali Ih (1)	CROP FAC FOOD UNIVERSITI PUTRA MALAYSIA BINTULU CAMPUS SARAWAK MALAYSIA (1)
Rawi Mh (2)	CROP FAC FOOD UNIVERSITI PUTRA MALAYSIA BINTULU CAMPUS SARAWAK MALAYSIA (1)
Bajury Dm (1)	CROP FAC FOOD UNIVERSITI PUTRA MALAYSIA BINTULU CAMPUS SARAWAK MALAYSIA (1)
Abdullah A (2)	CROP FAC FOOD UNIVERSITI PUTRA MALAYSIA BINTULU CAMPUS SARAWAK MALAYSIA (1)
Mohamad yusop S (1)	CROP FAC UNIVERSITI PUTRA MALAYSIA BINTULU CAMPUS BINTULU SARAWAK MALAYSIA (1)
Lim Sj (1)	CROP FAC UNIVERSITI PUTRA MALAYSIA BINTULU CAMPUS BINTULU SARAWAK MALAYSIA (1)
Daud Na (1)	CROP FAC UNIVERSITI PUTRA MALAYSIA BINTULU CAMPUS BINTULU SARAWAK MALAYSIA (1)
Babji As (1)	CROP FAC UNIVERSITI PUTRA MALAYSIA BINTULU CAMPUS BINTULU SARAWAK MALAYSIA (1)
Zahradnik Ac (1)	CRYPTOBIOTIX GHENT BELGIUM (1)
Foltz M (1)	CRYPTOBIOTIX GHENT BELGIUM (1)
Wu L (1)	CSIRO AU (1)
Van yken J (1)	CSIRO AU (1)
Tan S (1)	CSIRO AU (1)
Sanguansri L (1)	CSIRO AU (1)
Kaksonen Ah (2)	CSIRO AU (1)
Gras Sl (1)	CSIRO AU (1)
Faccini A (1)	CSIRO AU (1)
Cheng Ky (1)	CSIRO AU (1)
Chang B (1)	CSIRO AU (1)
Calvert G (1)	CSIRO AU (1)
Boxall Nj (1)	CSIRO AU (1)

Augustin Ma (1)	CSIRO AU (1)
Ann augustin M (1)	CSIRO AU (1)
Sumeri I (1)	CTR FOOD FERMENTATION TECHNOLOGIES TALLINN ESTONIA (1)
Paalme T (1)	CTR FOOD FERMENTATION TECHNOLOGIES TALLINN ESTONIA (1)
Arike L (1)	CTR FOOD FERMENTATION TECHNOLOGIES TALLINN ESTONIA (1)
Raba G (2)	CTR FOOD FERMENTATION TECHNOLOGIES TALLINN ESTONIA (2)
Adamberg S (2)	CTR FOOD FERMENTATION TECHNOLOGIES TALLINN ESTONIA (2)
Adamberg K (3)	CTR FOOD FERMENTATION TECHNOLOGIES TALLINN ESTONIA (3)
Beckman C (1)	CTR HEALTHY EATING FOOD INNOVATION HEFI MAASTRICHT UNIV CAMPUS VENLO VENLO NETHERLANDS (1)
Verhoeven J (5)	CTR HEALTHY EATING FOOD INNOVATION HEFI MAASTRICHT UNIV CAMPUS VENLO VENLO NETHERLANDS (2)
Vázquez L (1)	CTR HEALTHY EATING FOOD INNOVATION MAASTRICHT UNIV CAMPUS VENLO ST JANSWEG RC VENLO NETHERLANDS (1)
Redruello B (1)	CTR HEALTHY EATING FOOD INNOVATION MAASTRICHT UNIV CAMPUS VENLO ST JANSWEG RC VENLO NETHERLANDS (1)
Mayo B (1)	CTR HEALTHY EATING FOOD INNOVATION MAASTRICHT UNIV CAMPUS VENLO ST JANSWEG RC VENLO NETHERLANDS (1)
Flórez Ab (1)	CTR HEALTHY EATING FOOD INNOVATION MAASTRICHT UNIV CAMPUS VENLO ST JANSWEG RC VENLO NETHERLANDS (1)
Montes Cy (1)	CTR INVESTIGACION ASISTENCIA EN TECNOLOGIA DISENO DEL ESTADO JALISCO A C CAMINO ARENERO EL BAJIO ZAPOPAN JALISCO CP MEXICO (1)
Herrera-lópez Ej (1)	CTR INVESTIGACION ASISTENCIA EN TECNOLOGIA DISENO DEL ESTADO JALISCO A C CAMINO ARENERO EL BAJIO ZAPOPAN JALISCO CP MEXICO (1)
Gschaedler A (1)	CTR INVESTIGACION ASISTENCIA EN TECNOLOGIA DISENO DEL ESTADO JALISCO A C CAMINO ARENERO EL BAJIO ZAPOPAN JALISCO CP MEXICO (1)
Gradilla-hernández Ms (1)	CTR INVESTIGACION ASISTENCIA EN TECNOLOGIA DISENO DEL ESTADO JALISCO A C CAMINO ARENERO EL BAJIO ZAPOPAN JALISCO CP MEXICO (1)
González-avila M (1)	CTR INVESTIGACION ASISTENCIA EN TECNOLOGIA DISENO DEL ESTADO JALISCO A C CAMINO ARENERO EL BAJIO ZAPOPAN JALISCO CP MEXICO (1)
García-gonzález A (1)	CTR INVESTIGACION ASISTENCIA EN TECNOLOGIA DISENO DEL ESTADO JALISCO A C CAMINO ARENERO EL BAJIO ZAPOPAN JALISCO CP MEXICO (1)
García-gamboa R (1)	CTR INVESTIGACION ASISTENCIA EN TECNOLOGIA DISENO DEL ESTADO JALISCO A C CAMINO ARENERO EL BAJIO ZAPOPAN JALISCO CP MEXICO (1)
Fuentes-aguilar Rq (1)	CTR INVESTIGACION ASISTENCIA EN TECNOLOGIA DISENO DEL ESTADO JALISCO A C CAMINO ARENERO EL BAJIO ZAPOPAN JALISCO CP MEXICO (1)
Villegas-ochoa Ma (1)	CTR INVESTIGACION EN ALIMENTACION DESARROLLO A C CARRETERA GUSTAVO ENRIQUE ASTIAZARAN ROSAS COL VICTORIA HERMOSILLO SONORA MEXICO (1)
Salmerón-ruiz Ml (1)	CTR INVESTIGACION EN ALIMENTACION DESARROLLO A C CARRETERA GUSTAVO ENRIQUE ASTIAZARAN ROSAS COL VICTORIA HERMOSILLO SONORA MEXICO (1)
Salazar-lópez Nj (1)	CTR INVESTIGACION EN ALIMENTACION DESARROLLO A C CARRETERA GUSTAVO ENRIQUE ASTIAZARAN ROSAS COL VICTORIA HERMOSILLO SONORA MEXICO (1)
Domínguez-avila Ja (1)	CTR INVESTIGACION EN ALIMENTACION DESARROLLO A C CARRETERA GUSTAVO ENRIQUE ASTIAZARAN ROSAS COL VICTORIA HERMOSILLO SONORA MEXICO (1)
Pedro alberto V-l (1)	CTR INVESTIGACION EN CIENCIA APLICADA TECNOLOGIA AVANZADA DEL INST POLITECNICO NACIONAL INST POLITECNICO NACIONAL SANTIAGO QUERETARO MEXICO (1)
Loarca-piña Mgf (2)	CTR INVESTIGACION EN CIENCIA APLICADA TECNOLOGIA AVANZADA DEL INST POLITECNICO NACIONAL INST POLITECNICO NACIONAL SANTIAGO QUERETARO MEXICO (1)
Cárdenas-castro Ap (3)	CTR INVESTIGACION EN CIENCIA APLICADA TECNOLOGIA AVANZADA DEL INST POLITECNICO NACIONAL INST POLITECNICO NACIONAL SANTIAGO QUERETARO MEXICO (1)
Seidler K (1)	CTR LIFESTYLE CNELM CHAPEL GARDEN RECTORY ROAD WOKINGHAM BERKSHIRE RG40 1DH UNITED KINGDOM (1)
Pearson Nm (1)	CTR LIFESTYLE CNELM CHAPEL GARDEN RECTORY ROAD WOKINGHAM BERKSHIRE RG40 1DH UNITED KINGDOM (1)
Nielsen Sd (1)	CTR LIFESTYLE CNELM CHAPEL GARDEN RECTORY ROAD WOKINGHAM BERKSHIRE RG40 1DH UNITED KINGDOM (1)
Rotsaert C (2)	CTR MICROBIAL CMET FAC BIOSCIENCE GHENT UNIV COUPURE LINKS GHENT BELGIUM (2)
Vlaeminck B (1)	CTR MICROBIAL CMET GHENT UNIV BELGIUM (1)
Vichez-vargas R (1)	CTR MICROBIAL CMET GHENT UNIV BELGIUM (1)
Salden Bn (1)	CTR MICROBIAL CMET GHENT UNIV BELGIUM (1)
Rebaza M (1)	CTR MICROBIAL CMET GHENT UNIV BELGIUM (1)

Ranjanoro T (1)	CTR MICROBIAL CMET GHENT UNIV BELGIUM (1)
Ossieur Wp (1)	CTR MICROBIAL CMET GHENT UNIV BELGIUM (1)
Modica S (1)	CTR MICROBIAL CMET GHENT UNIV BELGIUM (1)
Mees E (1)	CTR MICROBIAL CMET GHENT UNIV BELGIUM (1)
Jiang Ta (1)	CTR MICROBIAL CMET GHENT UNIV BELGIUM (1)
Goltz S (1)	CTR MICROBIAL CMET GHENT UNIV BELGIUM (1)
Fievez V (1)	CTR MICROBIAL CMET GHENT UNIV BELGIUM (1)
Devriese S (1)	CTR MICROBIAL CMET GHENT UNIV BELGIUM (1)
de vos M (2)	CTR MICROBIAL CMET GHENT UNIV BELGIUM (1)
De chavez P (1)	CTR MICROBIAL CMET GHENT UNIV BELGIUM (1)
De blaiser A (1)	CTR MICROBIAL CMET GHENT UNIV BELGIUM (1)
Chung Y (1)	CTR MICROBIAL CMET GHENT UNIV BELGIUM (1)
Boileau T (1)	CTR MICROBIAL CMET GHENT UNIV BELGIUM (1)
Berezhnaya Y (1)	CTR MICROBIAL CMET GHENT UNIV BELGIUM (1)
Bayne Tf (1)	CTR MICROBIAL CMET GHENT UNIV BELGIUM (1)
Vital M (3)	CTR MICROBIAL CMET GHENT UNIV BELGIUM (2)
Maquet V (2)	CTR MICROBIAL CMET GHENT UNIV BELGIUM (2)
Hernandez-sanabria E (7)	CTR MICROBIAL CMET GHENT UNIV BELGIUM (3)
Pieper Dh (6)	CTR MICROBIAL CMET GHENT UNIV BELGIUM (5)
Hillman K (1)	CTR MICROBIOLOGICAL RES SCOTTISH CRAIBSTONE ESTATE ABERDEEN AB21 9YA UNITED KINGDOM (1)
Fenlon Dr (1)	CTR MICROBIOLOGICAL RES SCOTTISH CRAIBSTONE ESTATE ABERDEEN AB21 9YA UNITED KINGDOM (1)
Blake Dp (1)	CTR MICROBIOLOGICAL RES SCOTTISH CRAIBSTONE ESTATE ABERDEEN AB21 9YA UNITED KINGDOM (1)
Ravald N (1)	CTR PUBLIC CARE COUNTY OSTERGOTLAND LINKOPING SWEDEN (1)
Palm E (1)	CTR PUBLIC CARE COUNTY OSTERGOTLAND LINKOPING SWEDEN (1)
Nayeri F (1)	CTR PUBLIC CARE COUNTY OSTERGOTLAND LINKOPING SWEDEN (1)
Nakka S (1)	CTR PUBLIC CARE COUNTY OSTERGOTLAND LINKOPING SWEDEN (1)
Lönn J (1)	CTR PUBLIC CARE COUNTY OSTERGOTLAND LINKOPING SWEDEN (1)
Johansson Cs (1)	CTR PUBLIC CARE COUNTY OSTERGOTLAND LINKOPING SWEDEN (1)
Bengtsson T (1)	CTR PUBLIC CARE COUNTY OSTERGOTLAND LINKOPING SWEDEN (1)
Ouellette M (1)	CTR RECHERCHE EN INFECTIOLOGIE L UNIV LAVAL AXE MALADIES INFECTIEUSES IMMUNITAIRES CTR RECHERCHE DU CHU QUEBEC QUEBEC QC CANADA (1)
Le lay C (1)	CTR RECHERCHE EN INFECTIOLOGIE L UNIV LAVAL AXE MALADIES INFECTIEUSES IMMUNITAIRES CTR RECHERCHE DU CHU QUEBEC QUEBEC QC CANADA (1)
Hammami R (2)	CTR RECHERCHE EN INFECTIOLOGIE L UNIV LAVAL AXE MALADIES INFECTIEUSES IMMUNITAIRES CTR RECHERCHE DU CHU QUEBEC QUEBEC QC CANADA (1)
Ross Gr (1)	CTR REFERENCIA PARA LACTOBACILOS CERELA CONICET CHACABUCO TUCUMAN ARGENTINA (1)
Pérez ibarreche M (1)	CTR REFERENCIA PARA LACTOBACILOS CERELA CONICET CHACABUCO TUCUMAN ARGENTINA (1)
Niño arias Fc (1)	CTR REFERENCIA PARA LACTOBACILOS CERELA CONICET CHACABUCO TUCUMAN ARGENTINA (1)
Longo borges L (1)	CTR REFERENCIA PARA LACTOBACILOS CERELA CONICET CHACABUCO TUCUMAN ARGENTINA (1)
Castellano P (1)	CTR REFERENCIA PARA LACTOBACILOS CERELA CONICET CHACABUCO TUCUMAN ARGENTINA (1)
Neelamraju J (1)	CTR RES DEVELOPMENT UNIQUE BIOTECH LTD PLOT PHASE ALEXANDRIA KNOWLEDGE PARK HYDERABAD TELANGANA INDIA (1)
Madempudi Rs (1)	CTR RES DEVELOPMENT UNIQUE BIOTECH LTD PLOT PHASE ALEXANDRIA KNOWLEDGE PARK HYDERABAD TELANGANA INDIA (1)
Ahire Jj (1)	CTR RES DEVELOPMENT UNIQUE BIOTECH LTD PLOT PHASE ALEXANDRIA KNOWLEDGE PARK HYDERABAD TELANGANA INDIA (1)
Kardum N (1)	CTR RES EXCELLENCE INST RES UNIV BELGRADE SERBIA (1)
Heinonen M (1)	CTR RES EXCELLENCE INST RES UNIV BELGRADE SERBIA (1)
Wu T (2)	CTR RES EXCELLENCE INST RES UNIV BELGRADE SERBIA (2)
Kamiloglu S (2)	CTR RES EXCELLENCE INST RES UNIV BELGRADE SERBIA (2)
Glibetic M (2)	CTR RES EXCELLENCE INST RES UNIV BELGRADE SERBIA (2)
Thas O (1)	CTR STATISTICAL SURVEY METHODOLOGY UNIV WOLLONGONG WOLLONGONG NSW AUSTRALIA (1)

Goossens M (1)	CTR STATISTICAL SURVEY METHODOLOGY UNIV WOLLONGONG WOLLONGONG NSW AUSTRALIA (1)
Stams Ajm (1)	CTR UNIV MINHO CAMPUS GUALTAR PORTUGAL (1)
Sousa Dz (1)	CTR UNIV MINHO CAMPUS GUALTAR PORTUGAL (1)
Alves Mm (1)	CTR UNIV MINHO CAMPUS GUALTAR PORTUGAL (1)
Minnebo Y (1)	CTR VIB HERESTRAAT LEUVEN BELGIUM (1)
Shin I-s (1)	DAESANG KR (1)
Pan C-h (2)	DAESANG KR (1)
Jin Jb (2)	DAESANG KR (1)
Jeon Jy (1)	DAESANG KR (1)
Cha Kh (2)	DAESANG KR (1)
Cha Jw (1)	DAESANG KR (1)
Shafer K (1)	DAILY RESTORE LLC WIXOM MI UNITED STATES (1)
Kesari S (1)	DAILY RESTORE LLC WIXOM MI UNITED STATES (1)
Ichim Te (1)	DAILY RESTORE LLC WIXOM MI UNITED STATES (1)
Jung S (2)	DAIRY CHUNGNAM NAT UNIV DAEJEON SOUTH KOREA (2)
Choi Y-s (2)	DAIRY CHUNGNAM NAT UNIV DAEJEON SOUTH KOREA (2)
Kheadr E (1)	DAIRY FAC UNIV ALEXANDRIA ALEXANDRIA EGYPT (1)
Dabour N (1)	DAIRY FAC UNIV ALEXANDRIA ALEXANDRIA EGYPT (1)
Prosser C (1)	DAIRY GOAT CO OPERATIVE NZ LTD HAMILTON NEW ZEALAND (1)
Gallier S (1)	DAIRY GOAT CO OPERATIVE NZ LTD HAMILTON NEW ZEALAND (1)
Martínez-faedo C (2)	DAIRY PRODUCTS INST PRODUCTOS LACTEOS ASTURIAS IPLA CSIC VILLAVICIOSA ASTURIAS SPAIN (2)
Arboleya S (2)	DAIRY PRODUCTS INST PRODUCTOS LACTEOS ASTURIAS IPLA CSIC VILLAVICIOSA ASTURIAS SPAIN (2)
Ruas-madiedo P (4)	DAIRY PRODUCTS INST PRODUCTOS LACTEOS ASTURIAS IPLA CSIC VILLAVICIOSA ASTURIAS SPAIN (3)
Nogacka Am (3)	DAIRY PRODUCTS INST PRODUCTOS LACTEOS ASTURIAS IPLA CSIC VILLAVICIOSA ASTURIAS SPAIN (3)
Gueimonde M (4)	DAIRY PRODUCTS INST PRODUCTOS LACTEOS ASTURIAS IPLA CSIC VILLAVICIOSA ASTURIAS SPAIN (4)
de los reyes-gavilán Cg (5)	DAIRY PRODUCTS INST PRODUCTOS LACTEOS ASTURIAS IPLA CSIC VILLAVICIOSA ASTURIAS SPAIN (4)
Fliss I (8)	DAIRY RES CTR STELA UNIV LAVAL QUEBEC QUE CANADA (4)
Cinquin C (4)	DAIRY RES CTR STELA UNIV LAVAL QUEBEC QUE CANADA (4)
Zou H (1)	DALIAN OCEAN UNIV DALIAN CHINA (1)
Yan J (1)	DALIAN OCEAN UNIV DALIAN CHINA (1)
Qi Y (1)	DALIAN OCEAN UNIV DALIAN CHINA (1)
Mikkelsen Ll (1)	DANISH INST RES CTR FOULUM P O BOX TJELE DENMARK DENMARK (1)
Voolaid M (1)	DANONE FR (1)
Tison C (1)	DANONE FR (1)
Schwab C (2)	DANONE FR (1)
Oki K (1)	DANONE FR (1)
Oishi K (1)	DANONE FR (1)
Moriez R (1)	DANONE FR (1)
Maufoux L (1)	DANONE FR (1)
Lesic-arsic B (1)	DANONE FR (1)
Legrain-raspaud S (1)	DANONE FR (1)
Haug Mc (1)	DANONE FR (1)
Fujimoto J (1)	DANONE FR (1)
Brazeilles R (1)	DANONE FR (1)
Belaïnoussi Y (1)	DANONE FR (1)
Akiyama T (1)	DANONE FR (1)
Adouard N (2)	DANONE FR (1)
Aalvink S (1)	DANONE FR (1)
Stahl B (3)	DANONE FR (2)
Fourmestraux C (3)	DANONE FR (3)
Derrien M (4)	DANONE FR (3)
Ochoa-repáraz J (1)	DARTMOUTH LEBANON NH UNITED STATES (1)
Mielcarz Dw (1)	DARTMOUTH LEBANON NH UNITED STATES (1)
Kasper Lh (1)	DARTMOUTH LEBANON NH UNITED STATES (1)
Begum- haque S (1)	DARTMOUTH LEBANON NH UNITED STATES (1)
van den heuvel Eghm (1)	DELFT UNIV DELFT NETHERLANDS (1)
Nauta A (1)	DELFT UNIV DELFT NETHERLANDS (1)
Motelica-wagenaar Am (1)	DELFT UNIV DELFT NETHERLANDS (1)

Meysman Fjr (1)	DELFT UNIV DELFT NETHERLANDS (1)
Kleerebezem R (1)	DELFT UNIV DELFT NETHERLANDS (1)
Hidalgo-martinez S (1)	DELFT UNIV DELFT NETHERLANDS (1)
Dezutter O (1)	DELFT UNIV DELFT NETHERLANDS (1)
Jäger Ps (1)	DIREKTOR KLINIK KLINISCHE UNIVERSITATSKLINIKUM DUSSELDORF MOORENSTR DUSSELDORF GERMANY (1)
Haas R (1)	DIREKTOR KLINIK KLINISCHE UNIVERSITATSKLINIKUM DUSSELDORF MOORENSTR DUSSELDORF GERMANY (1)
Wopereis H (1)	DRUG DISCOVERY UTRECHT INST UTRECHT UNIV UNIVERSITEITSWEG UTRECHT CG NETHERLANDS (1)
Van limpt K (1)	DRUG DISCOVERY UTRECHT INST UTRECHT UNIV UNIVERSITEITSWEG UTRECHT CG NETHERLANDS (1)
Sybesma W (1)	DSM NL (1)
Rehman A (2)	DSM NL (1)
Krych L (1)	DSM NL (1)
Greppi A (1)	DSM NL (1)
De wouters T (1)	DSM NL (1)
Bruins Mj (1)	DSM NL (1)
Berchtold L (1)	DSM NL (1)
Bajic D (1)	DSM NL (1)
Seifert N (2)	DSM NL (2)
Richard N (2)	DSM NL (2)
Pham Vt (3)	DSM NL (2)
Steinert Re (3)	DSM NL (3)
Smith Ea (1)	DUNN CTR HILLS ROAD CAMBRIDGE CB2 2DH UNITED KINGDOM (1)
Quigley Me (1)	DUNN CTR HILLS ROAD CAMBRIDGE CB2 2DH UNITED KINGDOM (1)
Degnan Ba (1)	DUNN CTR HILLS ROAD CAMBRIDGE CB2 2DH UNITED KINGDOM (1)
Wiik-miettinen F (1)	DUPONT US (1)
Wiebe L (1)	DUPONT US (1)
Westerhuis Ja (1)	DUPONT US (1)
Van zanten Gc (1)	DUPONT US (1)
Tsitko I (1)	DUPONT US (1)
Svensson B (1)	DUPONT US (1)
Seppänen-laakso T (1)	DUPONT US (1)
Salminen Sj (1)	DUPONT US (1)
Salli K (1)	DUPONT US (1)
Saarela M (2)	DUPONT US (1)
Rosa-sibakov N (3)	DUPONT US (1)
Putaala H (1)	DUPONT US (1)
Probert Hm (1)	DUPONT US (1)
Morovic W (1)	DUPONT US (1)
Meier S (1)	DUPONT US (1)
Mattila O (1)	DUPONT US (1)
Marcussen Jø (1)	DUPONT US (1)
Mäkivuokko H (1)	DUPONT US (1)
Mäkeläinen Hs (1)	DUPONT US (1)
Lahtinen Sj (1)	DUPONT US (1)
Knudsen A (1)	DUPONT US (1)
Jensen Sl (1)	DUPONT US (1)
Hirvonen J (1)	DUPONT US (1)
Hasselwander O (1)	DUPONT US (1)
Forssten S (1)	DUPONT US (1)
Bertelsen K (1)	DUPONT US (1)
Bandsholm O (1)	DUPONT US (1)
Apajalahti Jha (1)	DUPONT US (1)
Anglenius H (1)	DUPONT US (1)
Ahonen I (1)	DUPONT US (1)
Young Jf (2)	DUPONT US (2)
Yde Cc (2)	DUPONT US (2)
Tiihonen K (2)	DUPONT US (2)
Stowell J (2)	DUPONT US (2)
Saarinen M (2)	DUPONT US (2)
Rautonen Ne (2)	DUPONT US (2)
Maukonen J (3)	DUPONT US (2)
Mäkivuokko Ha (2)	DUPONT US (2)

Mäkeläinen H (2)	DUPONT US (2)
Lamichhane S (2)	DUPONT US (2)
Jensen Hm (2)	DUPONT US (2)
Hibberd Aa (2)	DUPONT US (2)
Bertram Hc (2)	DUPONT US (2)
Forssten Sd (3)	DUPONT US (3)
Saarinen Mt (4)	DUPONT US (4)
Rautonen N (4)	DUPONT US (4)
Ouwehand Ac (7)	DUPONT US (7)
Pratten J (1)	EASTMAN US (1)
Pasu M (1)	EASTMAN US (1)
Jackson G (1)	EASTMAN US (1)
Hope Ck (1)	EASTMAN US (1)
Flanagan A (1)	EASTMAN US (1)
Wilson M (2)	EASTMAN US (2)
Tack Fmg (5)	ECOCHEMISTRY FAC BIOSCIENCE GHENT UNIV COUPURE LINKS GHENT BELGIUM (2)
Porras yaruro Jf (1)	ECOLE NATIONALE SUPERIEURE DES MINES SAINT ETIENNE FRANCE (1)
Larondelle Y (1)	ECOLE NATIONALE SUPERIEURE DES MINES SAINT ETIENNE FRANCE (1)
Iddir M (1)	ECOLE NATIONALE SUPERIEURE DES MINES SAINT ETIENNE FRANCE (1)
Unal G (1)	EGE UNIV FAC DAIRY IZMIR TURKEY (1)
Kocer E (1)	EGE UNIV FAC DAIRY IZMIR TURKEY (1)
Vicente Aamos (1)	EMBRAPA AGROINDUSTRIA ALIMENTOS RIO JANEIRO BRAZIL (1)
Teixeira Jac (1)	EMBRAPA AGROINDUSTRIA ALIMENTOS RIO JANEIRO BRAZIL (1)
Ribeiro Lo (1)	EMBRAPA AGROINDUSTRIA ALIMENTOS RIO JANEIRO BRAZIL (1)
Pinheiro Acb (1)	EMBRAPA AGROINDUSTRIA ALIMENTOS RIO JANEIRO BRAZIL (1)
Genisheva Za (1)	EMBRAPA AGROINDUSTRIA ALIMENTOS RIO JANEIRO BRAZIL (1)
Freitas Sp (1)	EMBRAPA AGROINDUSTRIA ALIMENTOS RIO JANEIRO BRAZIL (1)
de matta Vm (1)	EMBRAPA AGROINDUSTRIA ALIMENTOS RIO JANEIRO BRAZIL (1)
Brígida Ais (1)	EMBRAPA AGROINDUSTRIA ALIMENTOS RIO JANEIRO BRAZIL (1)
Schneider Y-j (1)	EMBRIA SE CREELVIEW DRIVE ANKENY IA UNITED STATES (1)
Robinson Le (1)	EMBRIA SE CREELVIEW DRIVE ANKENY IA UNITED STATES (1)
Reeves Sg (1)	EMBRIA SE CREELVIEW DRIVE ANKENY IA UNITED STATES (1)
Raas T (1)	EMBRIA SE CREELVIEW DRIVE ANKENY IA UNITED STATES (1)
Šuligoj T (1)	EMULATE INC DRYDOCK AVE BOSTON MA UNITED STATES (1)
Savva Gm (1)	EMULATE INC DRYDOCK AVE BOSTON MA UNITED STATES (1)
Karalis K (1)	EMULATE INC DRYDOCK AVE BOSTON MA UNITED STATES (1)
Juge N (1)	EMULATE INC DRYDOCK AVE BOSTON MA UNITED STATES (1)
Apostolou A (1)	EMULATE INC DRYDOCK AVE BOSTON MA UNITED STATES (1)
Prieto-chávez Jl (1)	ENCB IPN MEXICO CITY MEXICO (1)
Mancilla-herrera I (1)	ENCB IPN MEXICO CITY MEXICO (1)
López-macías C (1)	ENCB IPN MEXICO CITY MEXICO (1)
Isibasi A (1)	ENCB IPN MEXICO CITY MEXICO (1)
Ferat-osorio E (1)	ENCB IPN MEXICO CITY MEXICO (1)
Estrada-parra S (1)	ENCB IPN MEXICO CITY MEXICO (1)
Cérbulo-vázquez A (1)	ENCB IPN MEXICO CITY MEXICO (1)
Arriaga-pizano L (1)	ENCB IPN MEXICO CITY MEXICO (1)
Alvarado-moreno Ja (1)	ENCB IPN MEXICO CITY MEXICO (1)
Keim Jp (1)	ESCUELA GRADUADOS FACULTAD CIENCIAS AGRARIAS ALIMENTARIAS UNIV AUSTRAL CHILE INDEPENDENCIA VALDIVIA CHILE (1)
Gapp Em (1)	ESCUELA GRADUADOS FACULTAD CIENCIAS AGRARIAS ALIMENTARIAS UNIV AUSTRAL CHILE INDEPENDENCIA VALDIVIA CHILE (1)
Gandarillas M (1)	ESCUELA GRADUADOS FACULTAD CIENCIAS AGRARIAS ALIMENTARIAS UNIV AUSTRAL CHILE INDEPENDENCIA VALDIVIA CHILE (1)
Maldonado-celis Me (1)	ESCUELA NUTRICION DIETETICA UNIV ANTIOQUIA CIUDADELA ROBLEDO CARRERA MEDELLIN AA COLOMBIA (1)
Luzardo-ocampo I (2)	ESCUELA NUTRICION DIETETICA UNIV ANTIOQUIA CIUDADELA ROBLEDO CARRERA MEDELLIN AA COLOMBIA (1)
Agudelo Cd (1)	ESCUELA NUTRICION DIETETICA UNIV ANTIOQUIA CIUDADELA ROBLEDO CARRERA MEDELLIN AA COLOMBIA (1)
Vazquez gutierrez P (1)	ETH ZURICH ZURICH SWITZERLAND (1)
Rytka J (1)	ETH ZURICH ZURICH SWITZERLAND (1)
Rigozzi E (1)	ETH ZURICH ZURICH SWITZERLAND (1)
Nyström L (1)	ETH ZURICH ZURICH SWITZERLAND (1)
Follador R (1)	ETH ZURICH ZURICH SWITZERLAND (1)

Edwards V (1)	ETH ZURICH ZURICH SWITZERLAND (1)
Demuth T (1)	ETH ZURICH ZURICH SWITZERLAND (1)
Berner Az (1)	ETH ZURICH ZURICH SWITZERLAND (1)
Banz Y (1)	ETH ZURICH ZURICH SWITZERLAND (1)
Baltzer S (1)	ETH ZURICH ZURICH SWITZERLAND (1)
Chassard C (13)	ETH ZURICH ZURICH SWITZERLAND (12)
Lacroix C (22)	ETH ZURICH ZURICH SWITZERLAND (18)
Zimmermann Mb (2)	ETH ZURICH ZURICH SWITZERLAND (2)
Zihler berner A (2)	ETH ZURICH ZURICH SWITZERLAND (2)
Tanner Sa (2)	ETH ZURICH ZURICH SWITZERLAND (2)
Poeker Sa (2)	ETH ZURICH ZURICH SWITZERLAND (2)
Payne An (2)	ETH ZURICH ZURICH SWITZERLAND (2)
Grattepanche F (2)	ETH ZURICH ZURICH SWITZERLAND (2)
Gagnon M (2)	ETH ZURICH ZURICH SWITZERLAND (2)
Bircher L (2)	ETH ZURICH ZURICH SWITZERLAND (2)
Dostal A (3)	ETH ZURICH ZURICH SWITZERLAND (3)
Zihler A (4)	ETH ZURICH ZURICH SWITZERLAND (4)
Geirnaert A (8)	ETH ZURICH ZURICH SWITZERLAND (4)
Fehlbaum S (5)	ETH ZURICH ZURICH SWITZERLAND (4)
Le blay G (8)	ETH ZURICH ZURICH SWITZERLAND (5)
Sharaby Y (1)	EVOLUTIONARY FAC NATURAL UNIV HAIFA HAIFA ISRAEL (1)
Rodríguez-martínez S (1)	EVOLUTIONARY FAC NATURAL UNIV HAIFA HAIFA ISRAEL (1)
Halpern M (1)	EVOLUTIONARY FAC NATURAL UNIV HAIFA HAIFA ISRAEL (1)
Gilboa Y (1)	EVOLUTIONARY FAC NATURAL UNIV HAIFA HAIFA ISRAEL (1)
Friedler E (1)	EVOLUTIONARY FAC NATURAL UNIV HAIFA HAIFA ISRAEL (1)
Dekel A (1)	EVOLUTIONARY FAC NATURAL UNIV HAIFA HAIFA ISRAEL (1)
Aizenberg-gershtein Y (1)	EVOLUTIONARY FAC NATURAL UNIV HAIFA HAIFA ISRAEL (1)
Vacca M (1)	EVONIK DE (1)
Speckmann B (1)	EVONIK DE (1)
Siragusa S (1)	EVONIK DE (1)
Schwarm M (1)	EVONIK DE (1)
Pelzer S (1)	EVONIK DE (1)
Flügel M (1)	EVONIK DE (1)
Di cagno R (1)	EVONIK DE (1)
Cristofori F (1)	EVONIK DE (1)
Pereboom Dpkh (1)	EXPOSURE ASSESSMENT NATL INST PUB HLTH P O BOX NL BA BILTHOVEN NETHERLANDS (1)
Dobbe Cjg (1)	EXPOSURE ASSESSMENT NATL INST PUB HLTH P O BOX NL BA BILTHOVEN NETHERLANDS (1)
Bruil Ma (1)	EXPOSURE ASSESSMENT NATL INST PUB HLTH P O BOX NL BA BILTHOVEN NETHERLANDS (1)
Khiralla G (1)	FAC AIN SHAMS UNIV SHUBRA EL KHEIMA CAIRO EGYPT (1)
Ali Hm (1)	FAC AIN SHAMS UNIV SHUBRA EL KHEIMA CAIRO EGYPT (1)
Kücükcetin Io (1)	FAC AKDENIZ UNIV ANTALYA TURKEY (1)
Kücükcetin A (1)	FAC AKDENIZ UNIV ANTALYA TURKEY (1)
Gocer Emc (1)	FAC AKDENIZ UNIV ANTALYA TURKEY (1)
Ergin F (1)	FAC AKDENIZ UNIV ANTALYA TURKEY (1)
Zamfir M (1)	FAC BIOENGINEERING VRIJE UNIVERSITEIT BRUSSEL BRUSSELS BELGIUM (1)
Vuyst Lc (1)	FAC BIOENGINEERING VRIJE UNIVERSITEIT BRUSSEL BRUSSELS BELGIUM (1)
Vos Md (1)	FAC BIOENGINEERING VRIJE UNIVERSITEIT BRUSSEL BRUSSELS BELGIUM (1)
Verbeke K (1)	FAC BIOENGINEERING VRIJE UNIVERSITEIT BRUSSEL BRUSSELS BELGIUM (1)
Tinck M (1)	FAC BIOENGINEERING VRIJE UNIVERSITEIT BRUSSEL BRUSSELS BELGIUM (1)
Steyaert A (1)	FAC BIOENGINEERING VRIJE UNIVERSITEIT BRUSSEL BRUSSELS BELGIUM (1)
Selak M (2)	FAC BIOENGINEERING VRIJE UNIVERSITEIT BRUSSEL BRUSSELS BELGIUM (1)
Meulen Rvd (1)	FAC BIOENGINEERING VRIJE UNIVERSITEIT BRUSSEL BRUSSELS BELGIUM (1)
Immerseel Fv (1)	FAC BIOENGINEERING VRIJE UNIVERSITEIT BRUSSEL BRUSSELS BELGIUM (1)
Grosu-tudor S-s (1)	FAC BIOENGINEERING VRIJE UNIVERSITEIT BRUSSEL BRUSSELS BELGIUM (1)
Falony G (2)	FAC BIOENGINEERING VRIJE UNIVERSITEIT BRUSSEL BRUSSELS BELGIUM (2)
Villas-boas Mb (1)	FAC CIENCIAS MEDICAS UNICAMP CAMPINAS SAO PAULO BRAZIL (1)
Molina E (1)	FAC CIENCIAS MEDICAS UNICAMP CAMPINAS SAO PAULO BRAZIL (1)
de lima zollner R (1)	FAC CIENCIAS MEDICAS UNICAMP CAMPINAS SAO PAULO BRAZIL (1)
Benedé S (1)	FAC CIENCIAS MEDICAS UNICAMP CAMPINAS SAO PAULO BRAZIL (1)
Oliaee Ma (1)	FAC CIVIL BABOL NOSHIRVANI UNIV BABOL IRAN (1)
Bardi Mj (1)	FAC CIVIL BABOL NOSHIRVANI UNIV BABOL IRAN (1)
Ryan J (1)	FAC DEAKIN UNIV BURWOOD VIC AUSTRALIA (1)

Hutchings Sc (1)	FAC DEAKIN UNIV BURWOOD VIC AUSTRALIA (1)
Gamlath S (1)	FAC DEAKIN UNIV BURWOOD VIC AUSTRALIA (1)
Fang Z (2)	FAC DEAKIN UNIV BURWOOD VIC AUSTRALIA (1)
Bandara N (1)	FAC DEAKIN UNIV BURWOOD VIC AUSTRALIA (1)
Ismail A (1)	FAC FORESTRY UNIVERSITI PUTRA MALAYSIA KAMPUS BINTULU SARAWAK SARAWAK BINTULU MALAYSIA (1)
Zavišic Gn (1)	FAC FUTURA UNIV SINGIDUNUM POZESKA 83A BELGRADE SERBIA (1)
Strahinic Id (1)	FAC FUTURA UNIV SINGIDUNUM POZESKA 83A BELGRADE SERBIA (1)
Ristic Sm (1)	FAC FUTURA UNIV SINGIDUNUM POZESKA 83A BELGRADE SERBIA (1)
Rikalovic Mg (1)	FAC FUTURA UNIV SINGIDUNUM POZESKA 83A BELGRADE SERBIA (1)
Petricevic Sm (1)	FAC FUTURA UNIV SINGIDUNUM POZESKA 83A BELGRADE SERBIA (1)
Jovanovic-lješkovic Nm (1)	FAC FUTURA UNIV SINGIDUNUM POZESKA 83A BELGRADE SERBIA (1)
Begovic Jm (1)	FAC FUTURA UNIV SINGIDUNUM POZESKA 83A BELGRADE SERBIA (1)
Prueksasri S (1)	FAC INDUSTRIAL SILPAKORN UNIV NAKORN PATHOM THAILAND (1)
Ngampanya B (1)	FAC INDUSTRIAL SILPAKORN UNIV NAKORN PATHOM THAILAND (1)
Yaman M (1)	FAC ISTANBUL SABAHATTIN ZAIM UNIV TURKEY (1)
Kurt E (1)	FAC ISTANBUL SABAHATTIN ZAIM UNIV TURKEY (1)
Faruk mizrak Ö (1)	FAC ISTANBUL SABAHATTIN ZAIM UNIV TURKEY (1)
Ede-cintesun E (1)	FAC ISTANBUL SABAHATTIN ZAIM UNIV TURKEY (1)
Demirci M (1)	FAC ISTANBUL SABAHATTIN ZAIM UNIV TURKEY (1)
Tatsuya U (1)	FAC LIFE SARI JEJU NAT UNIV JEJU SOUTH KOREA (1)
Hwang N (1)	FAC LIFE SARI JEJU NAT UNIV JEJU SOUTH KOREA (1)
Sultan S (1)	FAC MANSOURA UNIV MANSOURA EGYPT (1)
Mottawea W (1)	FAC MANSOURA UNIV MANSOURA EGYPT (1)
Landau K (1)	FAC MANSOURA UNIV MANSOURA EGYPT (1)
Bordenave N (1)	FAC MANSOURA UNIV MANSOURA EGYPT (1)
Öztürk H (1)	FAC MEDICINE UNIV ANKARA ANKARA TURKEY (1)
Tran Tht (1)	FAC MEDICINE UNIV LIEGE LIEGE BELGIUM (1)
Théwis A (1)	FAC MEDICINE UNIV LIEGE LIEGE BELGIUM (1)
Portetelle D (1)	FAC MEDICINE UNIV LIEGE LIEGE BELGIUM (1)
Nezer C (1)	FAC MEDICINE UNIV LIEGE LIEGE BELGIUM (1)
Hendrick C (1)	FAC MEDICINE UNIV LIEGE LIEGE BELGIUM (1)
Gaillez J (1)	FAC MEDICINE UNIV LIEGE LIEGE BELGIUM (1)
Boudry C (1)	FAC MEDICINE UNIV LIEGE LIEGE BELGIUM (1)
Bindelle J (1)	FAC MEDICINE UNIV LIEGE LIEGE BELGIUM (1)
Crèvecoeur S (2)	FAC MEDICINE UNIV LIEGE LIEGE BELGIUM (2)
Bondue P (2)	FAC MEDICINE UNIV LIEGE LIEGE BELGIUM (2)
Lebrun S (5)	FAC MEDICINE UNIV LIEGE LIEGE BELGIUM (3)
Everaert N (5)	FAC MEDICINE UNIV LIEGE LIEGE BELGIUM (3)
Delcenserie V (5)	FAC MEDICINE UNIV LIEGE LIEGE BELGIUM (3)
Daube G (3)	FAC MEDICINE UNIV LIEGE LIEGE BELGIUM (3)
Taminiau B (4)	FAC MEDICINE UNIV LIEGE LIEGE BELGIUM (4)
Wójcik E (1)	FAC MEDICINE UNIV OTTAWA OTTAWA K1H 8M5 CANADA (1)
Dastych J (1)	FAC MEDICINE UNIV OTTAWA OTTAWA K1H 8M5 CANADA (1)
Ning Z (3)	FAC MEDICINE UNIV OTTAWA OTTAWA K1H 8M5 CANADA (2)
Figeys D (3)	FAC MEDICINE UNIV OTTAWA OTTAWA K1H 8M5 CANADA (2)
Butcher J (2)	FAC MEDICINE UNIV OTTAWA OTTAWA K1H 8M5 CANADA (2)
Sanatkar R (1)	FAC NATURAL UNIV RIAU PEKANBARU INDONESIA (1)
Rahimi kalateh shah mohammad G (1)	FAC NATURAL UNIV RIAU PEKANBARU INDONESIA (1)
Oskoueian E (1)	FAC NATURAL UNIV RIAU PEKANBARU INDONESIA (1)
Karimi E (1)	FAC NATURAL UNIV RIAU PEKANBARU INDONESIA (1)
Hendra R (1)	FAC NATURAL UNIV RIAU PEKANBARU INDONESIA (1)
Pankasemsuk T (1)	FAC PHRANAKHON SI AYUTTHAYA RAJABHAT UNIV PHRANAKHON SI AYUTTHAYA THAILAND (1)
Kingwatee N (1)	FAC PHRANAKHON SI AYUTTHAYA RAJABHAT UNIV PHRANAKHON SI AYUTTHAYA THAILAND (1)
Changrue V (1)	FAC PHRANAKHON SI AYUTTHAYA RAJABHAT UNIV PHRANAKHON SI AYUTTHAYA THAILAND (1)
Rattanasena P (2)	FAC PHRANAKHON SI AYUTTHAYA RAJABHAT UNIV PHRANAKHON SI AYUTTHAYA THAILAND (2)
Chaikham P (10)	FAC PHRANAKHON SI AYUTTHAYA RAJABHAT UNIV PHRANAKHON SI AYUTTHAYA THAILAND (6)
Thatrimontrichai A (1)	FAC PRINCE SONGKLA UNIV SONGKHLA THAILAND (1)
Singkhamanan K (1)	FAC PRINCE SONGKLA UNIV SONGKHLA THAILAND (1)

Saengsuwan P (1)	FAC PRINCE SONGKLA UNIV SONGKHLA THAILAND (1)
Romyasamit C (1)	FAC PRINCE SONGKLA UNIV SONGKHLA THAILAND (1)
Ingviya N (1)	FAC PRINCE SONGKLA UNIV SONGKHLA THAILAND (1)
Chanket W (1)	FAC PRINCE SONGKLA UNIV SONGKHLA THAILAND (1)
Aroonkesorn A (1)	FAC PRINCE SONGKLA UNIV SONGKHLA THAILAND (1)
Nabi-meibodi M (1)	FAC SHAHID SADOUGHI UNIV YAZD IRAN (1)
Molavi F (1)	FAC SHAHID SADOUGHI UNIV YAZD IRAN (1)
Ehrampoush Mh (1)	FAC SHAHID SADOUGHI UNIV YAZD IRAN (1)
Ebrahimi Aa (1)	FAC SHAHID SADOUGHI UNIV YAZD IRAN (1)
Vamanu A (1)	FAC UNIV AGRONOMIC MEDICINE MARASTI BLVD DISTRICT BUCHAREST ROMANIA (1)
Sarbu I (1)	FAC UNIV AGRONOMIC MEDICINE MARASTI BLVD DISTRICT BUCHAREST ROMANIA (1)
Nièa S (1)	FAC UNIV AGRONOMIC MEDICINE MARASTI BLVD DISTRICT BUCHAREST ROMANIA (1)
Nedelcu I (1)	FAC UNIV AGRONOMIC MEDICINE MARASTI BLVD DISTRICT BUCHAREST ROMANIA (1)
Avram I (2)	FAC UNIV AGRONOMIC MEDICINE MARASTI BLVD DISTRICT BUCHAREST ROMANIA (2)
Vamanu E (4)	FAC UNIV AGRONOMIC MEDICINE MARASTI BLVD DISTRICT BUCHAREST ROMANIA (3)
Pelinescu D (3)	FAC UNIV AGRONOMIC MEDICINE MARASTI BLVD DISTRICT BUCHAREST ROMANIA (3)
Yazici Ma (1)	FAC UNIV ISTANBUL TURKEY (1)
Üstün-aytekin Ö (1)	FAC UNIV ISTANBUL TURKEY (1)
Tornuk F (1)	FAC UNIV ISTANBUL TURKEY (1)
Seker A (1)	FAC UNIV ISTANBUL TURKEY (1)
Savasli E (1)	FAC UNIV ISTANBUL TURKEY (1)
Neri J (1)	FAC UNIV ISTANBUL TURKEY (1)
Hora K (1)	FAC UNIV ISTANBUL TURKEY (1)
Holwerda Ht (1)	FAC UNIV ISTANBUL TURKEY (1)
Dogan K (1)	FAC UNIV ISTANBUL TURKEY (1)
Cakmak I (1)	FAC UNIV ISTANBUL TURKEY (1)
Arisoy S (1)	FAC UNIV ISTANBUL TURKEY (1)
Akman Pk (1)	FAC UNIV ISTANBUL TURKEY (1)
Laya A (1)	FAC UNIV MAROUA P O BOX MAROUA CAMEROON (1)
Koubala Bb (1)	FAC UNIV MAROUA P O BOX MAROUA CAMEROON (1)
Teodorowicz M (1)	FAC UNIV WARMIA MAZURY OLSZTYN POLAND (1)
Swiatecki A (1)	FAC UNIV WARMIA MAZURY OLSZTYN POLAND (1)
Swiatecka D (1)	FAC UNIV WARMIA MAZURY OLSZTYN POLAND (1)
Sienkiewicz-szlapka E (1)	FAC UNIV WARMIA MAZURY OLSZTYN POLAND (1)
Sidor K (1)	FAC UNIV WARMIA MAZURY OLSZTYN POLAND (1)
Piskorz-ogórek K (1)	FAC UNIV WARMIA MAZURY OLSZTYN POLAND (1)
Matysiewicz M (1)	FAC UNIV WARMIA MAZURY OLSZTYN POLAND (1)
Markiewicz Lh (1)	FAC UNIV WARMIA MAZURY OLSZTYN POLAND (1)
Kostyra E (1)	FAC UNIV WARMIA MAZURY OLSZTYN POLAND (1)
Fiedorowicz E (1)	FAC UNIV WARMIA MAZURY OLSZTYN POLAND (1)
Cieslinska A (1)	FAC UNIV WARMIA MAZURY OLSZTYN POLAND (1)
Mustafa S (1)	FAC UNIVERSITI PUTRA MALAYSIA UPM UPM SERDANG MALAYSIA (1)
Mirhosseini H (1)	FAC UNIVERSITI PUTRA MALAYSIA UPM UPM SERDANG MALAYSIA (1)
Manap Mya (1)	FAC UNIVERSITI PUTRA MALAYSIA UPM UPM SERDANG MALAYSIA (1)
Kamalian N (1)	FAC UNIVERSITI PUTRA MALAYSIA UPM UPM SERDANG MALAYSIA (1)
Yesilada E (1)	FAC YEDITEPE UNIV ATASEHIR ISTANBUL TURKEY (1)
Charehsaz M (1)	FAC YEDITEPE UNIV ATASEHIR ISTANBUL TURKEY (1)
Celep E (1)	FAC YEDITEPE UNIV ATASEHIR ISTANBUL TURKEY (1)
Akyüz S (1)	FAC YEDITEPE UNIV ATASEHIR ISTANBUL TURKEY (1)
Acar Et (1)	FAC YEDITEPE UNIV ATASEHIR ISTANBUL TURKEY (1)
Villanueva-carvajal A (1)	FACULTAD CIENCIAS AGRICOLAS UNIV AUTONOMA DEL ESTADO MEXICO CAMPUS UNIVERSITARIO EL CERRILLCARR TOLUCA IXTLAHUACA KM ENTRONQUE EL CERRILLO APDO POSTAL TOLUCA ESTADO MEXICO MEXICO (1)
García-gasca Mt (1)	FACULTAD CIENCIAS AGRICOLAS UNIV AUTONOMA DEL ESTADO MEXICO CAMPUS UNIVERSITARIO EL CERRILLCARR TOLUCA IXTLAHUACA KM ENTRONQUE EL CERRILLO APDO POSTAL TOLUCA ESTADO MEXICO MEXICO (1)

Dominguez-lopez A (1)	FACULTAD CIENCIAS AGRICOLAS UNIV AUTONOMA DEL ESTADO MEXICO CAMPUS UNIVERSITARIO EL CERRILLCARR TOLUCA IXTLAHUACA KM ENTRONQUE EL CERRILLO APDO POSTAL TOLUCA ESTADO MEXICO MEXICO (1)
Bernal-martínez Lr (1)	FACULTAD CIENCIAS AGRICOLAS UNIV AUTONOMA DEL ESTADO MEXICO CAMPUS UNIVERSITARIO EL CERRILLCARR TOLUCA IXTLAHUACA KM ENTRONQUE EL CERRILLO APDO POSTAL TOLUCA ESTADO MEXICO MEXICO (1)
Salinas C (1)	FACULTAD CIENCIAS UNIV CHILE LAS PALMERAS SANTIAGO CHILE (1)
Quezada Mp (1)	FACULTAD CIENCIAS UNIV CHILE LAS PALMERAS SANTIAGO CHILE (1)
Cardemil L (1)	FACULTAD CIENCIAS UNIV CHILE LAS PALMERAS SANTIAGO CHILE (1)
Vázquez-chagoyán Jc (1)	FACULTAD MEDICINA ZOOTECNIA UNIV AUTONOMA DEL ESTADO MEXICO MEXICO (1)
Salem Azm (1)	FACULTAD MEDICINA ZOOTECNIA UNIV AUTONOMA DEL ESTADO MEXICO MEXICO (1)
Islas-espinoza M (1)	FACULTAD MEDICINA ZOOTECNIA UNIV AUTONOMA DEL ESTADO MEXICO MEXICO (1)
de las heras A (1)	FACULTAD MEDICINA ZOOTECNIA UNIV AUTONOMA DEL ESTADO MEXICO MEXICO (1)
José jara-palacios M (1)	FARO TECHNOLOGIES US (1)
Hernanz D (1)	FARO TECHNOLOGIES US (1)
Heredia Fj (1)	FARO TECHNOLOGIES US (1)
Gonçalves S (1)	FARO TECHNOLOGIES US (1)
Zepka Lq (1)	FEDERAL UNIV SANTA MARIARIO GRANDE DO SUL BRAZIL (1)
Wagner R (1)	FEDERAL UNIV SANTA MARIARIO GRANDE DO SUL BRAZIL (1)
Ragagnin de menezes C (1)	FEDERAL UNIV SANTA MARIARIO GRANDE DO SUL BRAZIL (1)
Raddatz Gc (1)	FEDERAL UNIV SANTA MARIARIO GRANDE DO SUL BRAZIL (1)
Muller Ei (1)	FEDERAL UNIV SANTA MARIARIO GRANDE DO SUL BRAZIL (1)
Jacob-lopes E (1)	FEDERAL UNIV SANTA MARIARIO GRANDE DO SUL BRAZIL (1)
Fonseca Vr (1)	FEDERAL UNIV SANTA MARIARIO GRANDE DO SUL BRAZIL (1)
de moraes flores Em (1)	FEDERAL UNIV SANTA MARIARIO GRANDE DO SUL BRAZIL (1)
de bona da silva C (1)	FEDERAL UNIV SANTA MARIARIO GRANDE DO SUL BRAZIL (1)
Cichoski Aj (1)	FEDERAL UNIV SANTA MARIARIO GRANDE DO SUL BRAZIL (1)
Campagnol Pcb (1)	FEDERAL UNIV SANTA MARIARIO GRANDE DO SUL BRAZIL (1)
Gonçalves Ga (1)	FEDERAL UNIV SOUTH BORDER REALEZAPARANA BRAZIL (1)
de souza Cgm (1)	FEDERAL UNIV SOUTH BORDER REALEZAPARANA BRAZIL (1)
de sá-nakanishi Ab (1)	FEDERAL UNIV SOUTH BORDER REALEZAPARANA BRAZIL (1)
Ningegowda Ma (1)	FERMENTATION BIOENGINEERING CENTRAL FOOD TECHNOLOGICAL RES INST MYSORE INDIA (1)
Gurudutt Ps (1)	FERMENTATION BIOENGINEERING CENTRAL FOOD TECHNOLOGICAL RES INST MYSORE INDIA (1)
Szwengiel A (2)	FERMENTATION BIOSYNTHESIS FOOD POZNAN UNIV LIFE POLAND (1)
Smoczynska P (1)	FERMENTATION BIOSYNTHESIS FOOD POZNAN UNIV LIFE POLAND (1)
Komolka P (1)	FERMENTATION BIOSYNTHESIS FOOD POZNAN UNIV LIFE POLAND (1)
Górecka D (2)	FERMENTATION BIOSYNTHESIS FOOD POZNAN UNIV LIFE POLAND (1)
Dziedzic K (2)	FERMENTATION BIOSYNTHESIS FOOD POZNAN UNIV LIFE POLAND (1)
Czaczyk K (1)	FERMENTATION BIOSYNTHESIS FOOD POZNAN UNIV LIFE POLAND (1)
Tarko T (1)	FERMENTATION UNIV KRAKOW UL BALICKA CRACOW POLAND (1)
Duda-chodak A (1)	FERMENTATION UNIV KRAKOW UL BALICKA CRACOW POLAND (1)
Tannocka Gw (1)	FERRIER RES INST VICTORIA UNIV WELLINGTON PETONE NEW ZEALAND (1)
Sims Im (1)	FERRIER RES INST VICTORIA UNIV WELLINGTON PETONE NEW ZEALAND (1)
Ferguson Sa (1)	FERRIER RES INST VICTORIA UNIV WELLINGTON PETONE NEW ZEALAND (1)
Biswas A (1)	FERRIER RES INST VICTORIA UNIV WELLINGTON PETONE NEW ZEALAND (1)
Verschaeve L (1)	FLEMISH INST VITO BOERETANG MOL BELGIUM (1)
Schoeters G (1)	FLEMISH INST VITO BOERETANG MOL BELGIUM (1)
Berckmans P (1)	FLEMISH INST VITO BOERETANG MOL BELGIUM (1)
Oztop Mh (1)	FOOD AHI EVRAN UNIV KIRSEHIR TURKEY (1)
Ozel B (1)	FOOD AHI EVRAN UNIV KIRSEHIR TURKEY (1)
Aydin O (1)	FOOD AHI EVRAN UNIV KIRSEHIR TURKEY (1)
Moreno-rojas Jm (4)	FOOD ANDALUSIAN INST FISHERIES RES TRAINING IFAPA ALAMEDA DEL OBISPO AVDA MENENDEZ PIDAL S N CORDOBA SPAIN (3)
Ispirli H (1)	FOOD BAYBURT UNIV BAYBURT TURKEY (1)
Dertli E (2)	FOOD BAYBURT UNIV BAYBURT TURKEY (1)
Demirbas F (1)	FOOD BAYBURT UNIV BAYBURT TURKEY (1)
Tzima K (1)	FOOD BIOSCIENCES TEAGASC FOOD RES CTR ASHTOWN DUBLIN D15 DN3K IRELAND (1)

Rai Dk (1)	FOOD BIOSCIENCES TEAGASC FOOD RES CTR ASHTOWN DUBLIN D15 DN3K IRELAND (1)
Zhu D (1)	FOOD BOHAI UNIV JINZHOULIAONING CHINA (1)
Ma T (1)	FOOD BOHAI UNIV JINZHOULIAONING CHINA (1)
Cai W (1)	FOOD BOHAI UNIV JINZHOULIAONING CHINA (1)
Ban L (1)	FOOD BOHAI UNIV JINZHOULIAONING CHINA (1)
Yang L (3)	FOOD BOHAI UNIV JINZHOULIAONING CHINA (2)
Song H (2)	FOOD BOHAI UNIV JINZHOULIAONING CHINA (2)
Temiz A (1)	FOOD CANAKKALE ONSEKIZ MART UNIV CANAKKALE TURKEY (1)
Ozmen togay S (1)	FOOD CANAKKALE ONSEKIZ MART UNIV CANAKKALE TURKEY (1)
Bagci U (1)	FOOD CANAKKALE ONSEKIZ MART UNIV CANAKKALE TURKEY (1)
Ay M (1)	FOOD CANAKKALE ONSEKIZ MART UNIV CANAKKALE TURKEY (1)
Hosseinian F (1)	FOOD CARLETON UNIV COLONEL BY DRIVE OTTAWA ONTARIO K1S 5B6 CANADA (1)
Gunenc A (1)	FOOD CARLETON UNIV COLONEL BY DRIVE OTTAWA ONTARIO K1S 5B6 CANADA (1)
Chait Ya (1)	FOOD CARLETON UNIV COLONEL BY DRIVE OTTAWA ONTARIO K1S 5B6 CANADA (1)
Bendali F (1)	FOOD CARLETON UNIV COLONEL BY DRIVE OTTAWA ONTARIO K1S 5B6 CANADA (1)
Wu S (1)	FOOD DALIAN POLYTECHNIC UNIV DALIAN CHINA (1)
Wen C (1)	FOOD DALIAN POLYTECHNIC UNIV DALIAN CHINA (1)
Song S (1)	FOOD DALIAN POLYTECHNIC UNIV DALIAN CHINA (1)
Ma N (1)	FOOD DALIAN POLYTECHNIC UNIV DALIAN CHINA (1)
Duan M (1)	FOOD DALIAN POLYTECHNIC UNIV DALIAN CHINA (1)
Ai C (1)	FOOD DALIAN POLYTECHNIC UNIV DALIAN CHINA (1)
Suh Hj (1)	FOOD DONGGUK UNIV SOUTH KOREA (1)
Shin J (1)	FOOD DONGGUK UNIV SOUTH KOREA (1)
Jung Ey (1)	FOOD DONGGUK UNIV SOUTH KOREA (1)
Jang Ey (1)	FOOD DONGGUK UNIV SOUTH KOREA (1)
Hong K-b (1)	FOOD DONGGUK UNIV SOUTH KOREA (1)
Chang Yb (1)	FOOD DONGGUK UNIV SOUTH KOREA (1)
Sánchez-rivera L (1)	FOOD FAC EGE UNIV IZMIR TURKEY (1)
El Sn (1)	FOOD FAC EGE UNIV IZMIR TURKEY (1)
Nieva-echevarría B (1)	FOOD FAC LASCARAY RES CTR UNIV BASQUE COUNTRY UPV EHU PASEO UNIV N VITORIA SPAIN (1)
Manzanos Mj (1)	FOOD FAC LASCARAY RES CTR UNIV BASQUE COUNTRY UPV EHU PASEO UNIV N VITORIA SPAIN (1)
Guillén Md (1)	FOOD FAC LASCARAY RES CTR UNIV BASQUE COUNTRY UPV EHU PASEO UNIV N VITORIA SPAIN (1)
Goicoechea E (1)	FOOD FAC LASCARAY RES CTR UNIV BASQUE COUNTRY UPV EHU PASEO UNIV N VITORIA SPAIN (1)
Azizkhani M (1)	FOOD FAC MEDICINE AMOL UNIV SPECIAL MODERN TECHNOLOGIES AFTAB ST HARAZ AV AMOL IRAN (1)
Atraki R (1)	FOOD FAC MEDICINE AMOL UNIV SPECIAL MODERN TECHNOLOGIES AFTAB ST HARAZ AV AMOL IRAN (1)
Hossain Mn (1)	FOOD FAC UNIV MELBOURNE MELBOURNE VIC AUSTRALIA (1)
Ranadheera Cs (4)	FOOD FAC UNIV MELBOURNE MELBOURNE VIC AUSTRALIA (2)
Sarbini Sr (4)	FOOD FAC UNIVERSITI KEBANGSAAN MALAYSIA BANGI SELANGOR MALAYSIA (2)
Wróblewska B (1)	FOOD FOOD INST REPRODUCTION FOOD RES POLISH ACADEMY OLSZTYN POLAND (1)
Kalicki B (1)	FOOD FOOD INST REPRODUCTION FOOD RES POLISH ACADEMY OLSZTYN POLAND (1)
Fotschki J (1)	FOOD FOOD INST REPRODUCTION FOOD RES POLISH ACADEMY OLSZTYN POLAND (1)
Fotschki B (1)	FOOD FOOD INST REPRODUCTION FOOD RES POLISH ACADEMY OLSZTYN POLAND (1)
Al-otaibi Mm (1)	FOOD FOOD KING FAISAL UNIV HOFUF P O BOX AL AHSA SAUDI ARABIA (1)
Toe Lc (1)	FOOD GHENT UNIV COUPURE LINKS GHENT BELGIUM (1)
Ouedraogo J-b (1)	FOOD GHENT UNIV COUPURE LINKS GHENT BELGIUM (1)
Morel Fb (1)	FOOD GHENT UNIV COUPURE LINKS GHENT BELGIUM (1)
Kolsteren P (1)	FOOD GHENT UNIV COUPURE LINKS GHENT BELGIUM (1)
Villalobos-carvajal R (1)	FOOD INDUSTRY RES INST GUATAO ROAD KM ½ HAVANA CUBA (1)
Sevillano-armesto E (1)	FOOD INDUSTRY RES INST GUATAO ROAD KM ½ HAVANA CUBA (1)
Leiva-vega J (1)	FOOD INDUSTRY RES INST GUATAO ROAD KM ½ HAVANA CUBA (1)

Beldarrain-iznaga T (1)	FOOD INDUSTRY RES INST GUATAO ROAD KM ½ HAVANA CUBA (1)
Shubethar S (1)	FOOD INNOVATION CTR TATA CHEMICALS LTDPUNE INDIA (1)
Saravanan R (1)	FOOD INNOVATION CTR TATA CHEMICALS LTDPUNE INDIA (1)
Narayanan S (1)	FOOD INNOVATION CTR TATA CHEMICALS LTDPUNE INDIA (1)
Maheswaran P (1)	FOOD INNOVATION CTR TATA CHEMICALS LTDPUNE INDIA (1)
Lade S (1)	FOOD INNOVATION CTR TATA CHEMICALS LTDPUNE INDIA (1)
Jain M (1)	FOOD INNOVATION CTR TATA CHEMICALS LTDPUNE INDIA (1)
Jadhav D (1)	FOOD INNOVATION CTR TATA CHEMICALS LTDPUNE INDIA (1)
Dubey Ak (1)	FOOD INNOVATION CTR TATA CHEMICALS LTDPUNE INDIA (1)
Avalakki Uk (1)	FOOD INNOVATION CTR TATA CHEMICALS LTDPUNE INDIA (1)
Tsilia V (1)	FOOD INST FISHERIES RES ILVO MELLE BELGIUM (1)
Welti-chanes J (1)	FOOD INST FOOD RES CIAL CSIC UAM MADRID SPAIN (1)
Taladrid D (2)	FOOD INST FOOD RES CIAL CSIC UAM MADRID SPAIN (1)
Lara-abia S (1)	FOOD INST FOOD RES CIAL CSIC UAM MADRID SPAIN (1)
Khoo C (1)	FOOD INST FOOD RES CIAL CSIC UAM MADRID SPAIN (1)
Cano Mp (1)	FOOD INST FOOD RES CIAL CSIC UAM MADRID SPAIN (1)
Adarkwah-yiadom M (1)	FOOD INST FOOD WELL BEING UNIV PRETORIA PRIVATE BAG X20 HATFIELD SOUTH AFRICA (1)
Duodu Kg (3)	FOOD INST FOOD WELL BEING UNIV PRETORIA PRIVATE BAG X20 HATFIELD SOUTH AFRICA (3)
Vollenweider S (1)	FOOD INST FOOD ZURICH SWITZERLAND (1)
Cleusix V (1)	FOOD INST FOOD ZURICH SWITZERLAND (1)
Singu Bd (1)	FOOD INST MATUNGA E MUMBAI INDIA (1)
Bhushette Pr (1)	FOOD INST MATUNGA E MUMBAI INDIA (1)
Annapure Us (1)	FOOD INST MATUNGA E MUMBAI INDIA (1)
Van craeyveld V (1)	FOOD KATHOLIEKE UNIVERSITEIT LEUVEN LEUVEN BELGIUM (1)
Sanchez Ji (1)	FOOD KATHOLIEKE UNIVERSITEIT LEUVEN LEUVEN BELGIUM (1)
Baran M (1)	FOOD KATHOLIEKE UNIVERSITEIT LEUVEN LEUVEN BELGIUM (1)
Delcour Ja (2)	FOOD KATHOLIEKE UNIVERSITEIT LEUVEN LEUVEN BELGIUM (2)
Courtin Cm (4)	FOOD KATHOLIEKE UNIVERSITEIT LEUVEN LEUVEN BELGIUM (2)
Broekaert Wf (2)	FOOD KATHOLIEKE UNIVERSITEIT LEUVEN LEUVEN BELGIUM (2)
Verspreet J (2)	FOOD LEUVEN FOOD RES CTR LFORCE FAC BIOSCIENCE KU LEUVEN LEUVEN BELGIUM (2)
Van loey Am (1)	FOOD LEUVEN FOOD RES CTR LFORCE MICROBIAL SYSTEMS M2S KU LEUVEN KASTEELPARK ARENBERG PB LEUVEN BELGIUM (1)
Salvia-trujillo L (1)	FOOD LEUVEN FOOD RES CTR LFORCE MICROBIAL SYSTEMS M2S KU LEUVEN KASTEELPARK ARENBERG PB LEUVEN BELGIUM (1)
Saadi Mr (1)	FOOD LEUVEN FOOD RES CTR LFORCE MICROBIAL SYSTEMS M2S KU LEUVEN KASTEELPARK ARENBERG PB LEUVEN BELGIUM (1)
Moens Lg (1)	FOOD LEUVEN FOOD RES CTR LFORCE MICROBIAL SYSTEMS M2S KU LEUVEN KASTEELPARK ARENBERG PB LEUVEN BELGIUM (1)
Infantes-garcia Mr (1)	FOOD LEUVEN FOOD RES CTR LFORCE MICROBIAL SYSTEMS M2S KU LEUVEN KASTEELPARK ARENBERG PB LEUVEN BELGIUM (1)
Charleer L (1)	FOOD LEUVEN FOOD RES CTR LFORCE MICROBIAL SYSTEMS M2S KU LEUVEN KASTEELPARK ARENBERG PB LEUVEN BELGIUM (1)
Verkempinck She (2)	FOOD LEUVEN FOOD RES CTR LFORCE MICROBIAL SYSTEMS M2S KU LEUVEN KASTEELPARK ARENBERG PB LEUVEN BELGIUM (2)
Hendrickx Me (2)	FOOD LEUVEN FOOD RES CTR LFORCE MICROBIAL SYSTEMS M2S KU LEUVEN KASTEELPARK ARENBERG PB LEUVEN BELGIUM (2)
Grauwet T (3)	FOOD LEUVEN FOOD RES CTR LFORCE MICROBIAL SYSTEMS M2S KU LEUVEN KASTEELPARK ARENBERG PB LEUVEN BELGIUM (2)
Cho S-j (1)	FOOD LIFE KANGWON NAT UNIV CHUNCHEON SOUTH KOREA (1)
Bertha C-t (1)	FOOD LUND UNIV LUND SWEDEN (1)
Alberto S-bj (1)	FOOD LUND UNIV LUND SWEDEN (1)
Zamora-gasga Vm (4)	FOOD LUND UNIV LUND SWEDEN (2)
Tovar J (3)	FOOD LUND UNIV LUND SWEDEN (2)
Moughan Pj (1)	FOOD MASSEY UNIV PALMERSTON NORTH NEW ZEALAND (1)
Darragh Aj (1)	FOOD MASSEY UNIV PALMERSTON NORTH NEW ZEALAND (1)
Coles Lt (1)	FOOD MASSEY UNIV PALMERSTON NORTH NEW ZEALAND (1)
Khodaei N (1)	FOOD MCGILL UNIVERSITYQC H9X 3V9 CANADA (1)
Karboune S (1)	FOOD MCGILL UNIVERSITYQC H9X 3V9 CANADA (1)
Pernthaner A (1)	FOOD NEW ZEALAND INST PLANT FOOD RES LTD PALMERSTON NORTH NEW ZEALAND (1)
Jobsis Cmh (1)	FOOD NEW ZEALAND INST PLANT FOOD RES LTD PALMERSTON NORTH NEW ZEALAND (1)

Cooney Jm (1)	FOOD NEW ZEALAND INST PLANT FOOD RES LTD PALMERSTON NORTH NEW ZEALAND (1)
Ansell J (3)	FOOD NEW ZEALAND INST PLANT FOOD RES LTD PALMERSTON NORTH NEW ZEALAND (2)
Bentley-hewitt Kl (3)	FOOD NEW ZEALAND INST PLANT FOOD RES LTD PALMERSTON NORTH NEW ZEALAND (3)
Tamburello A (1)	FOOD NORWEGIAN UNIV LIFE AS NORWAY (1)
Skeie S (1)	FOOD NORWEGIAN UNIV LIFE AS NORWAY (1)
Faye T (1)	FOOD NORWEGIAN UNIV LIFE AS NORWAY (1)
Corredig M (4)	FOOD NORWEGIAN UNIV LIFE AS NORWAY (2)
Comi I (3)	FOOD NORWEGIAN UNIV LIFE AS NORWAY (2)
Vegarud Ge (4)	FOOD NORWEGIAN UNIV LIFE AS NORWAY (4)
Walkowiak J (1)	FOOD POZNAN UNIV LIFE WOJSKA POLSKIEGO POZNAN POLAND (1)
Michniewicz J (1)	FOOD POZNAN UNIV LIFE WOJSKA POLSKIEGO POZNAN POLAND (1)
Kozlowski P (1)	FOOD POZNAN UNIV LIFE WOJSKA POLSKIEGO POZNAN POLAND (1)
Goderska K (1)	FOOD POZNAN UNIV LIFE WOJSKA POLSKIEGO POZNAN POLAND (1)
Drozdzynska A (1)	FOOD POZNAN UNIV LIFE WOJSKA POLSKIEGO POZNAN POLAND (1)
Van eijnatten Ejm (1)	FOOD PROCESS WAGENINGEN UNIV BORNSE WEILANDEN WAGENINGEN WE NETHERLANDS (1)
Smeets Pam (1)	FOOD PROCESS WAGENINGEN UNIV BORNSE WEILANDEN WAGENINGEN WE NETHERLANDS (1)
Mayar M (1)	FOOD PROCESS WAGENINGEN UNIV BORNSE WEILANDEN WAGENINGEN WE NETHERLANDS (1)
Sahan Y (1)	FOOD PROCESSING BANDIRMA VOCATIONAL HIGH BANDIRMA ONYEDI EYLUL UNIV BALIKESIR TURKEY (1)
Gurbuz O (1)	FOOD PROCESSING BANDIRMA VOCATIONAL HIGH BANDIRMA ONYEDI EYLUL UNIV BALIKESIR TURKEY (1)
Degirmencioglu N (1)	FOOD PROCESSING BANDIRMA VOCATIONAL HIGH BANDIRMA ONYEDI EYLUL UNIV BALIKESIR TURKEY (1)
Rovalino-córdova Am (1)	FOOD QUALITY DESIGN WAGENINGEN UNIV RES NETHERLANDS (1)
Shen Q (4)	FOOD QUALITY RES INNOVATION CTR FONDAZIONE EDMUND MACH S MICHELE TN ITALY (2)
Farnworth Er (1)	FOOD RES DEVELOPMENT CTR AGRI FOOD CANADA CASAVANT BLV WEST ST HYACINTHE QUE J2S CANADA (1)
Veach Bt (1)	FOOD SAFETY CTR MEDICINE U S FOOD DRUG ADMINISTRATION ROCKVILLE MD UNITED STATES (1)
Piñeiro Sa (1)	FOOD SAFETY CTR MEDICINE U S FOOD DRUG ADMINISTRATION ROCKVILLE MD UNITED STATES (1)
Kweon O (1)	FOOD SAFETY CTR MEDICINE U S FOOD DRUG ADMINISTRATION ROCKVILLE MD UNITED STATES (1)
Khare S (2)	FOOD SAFETY CTR MEDICINE U S FOOD DRUG ADMINISTRATION ROCKVILLE MD UNITED STATES (1)
Jung Jy (1)	FOOD SAFETY CTR MEDICINE U S FOOD DRUG ADMINISTRATION ROCKVILLE MD UNITED STATES (1)
Gokulan K (2)	FOOD SAFETY CTR MEDICINE U S FOOD DRUG ADMINISTRATION ROCKVILLE MD UNITED STATES (1)
Cerniglia Ce (1)	FOOD SAFETY CTR MEDICINE U S FOOD DRUG ADMINISTRATION ROCKVILLE MD UNITED STATES (1)
Ahn Y (1)	FOOD SAFETY CTR MEDICINE U S FOOD DRUG ADMINISTRATION ROCKVILLE MD UNITED STATES (1)
Thum C (1)	FOOD TEAM AGRESEARCH GRASSLANDS PALMERSTON NORTH NEW ZEALAND (1)
Roy Nc (1)	FOOD TEAM AGRESEARCH GRASSLANDS PALMERSTON NORTH NEW ZEALAND (1)
Montoya Ca (1)	FOOD TEAM AGRESEARCH GRASSLANDS PALMERSTON NORTH NEW ZEALAND (1)
Mcnabb Wc (1)	FOOD TEAM AGRESEARCH GRASSLANDS PALMERSTON NORTH NEW ZEALAND (1)
Seifert A (1)	FOOD TECHNION ISRAEL INST HAIFA ISRAEL (1)
Moshe H (1)	FOOD TECHNION ISRAEL INST HAIFA ISRAEL (1)
Livney Yd (1)	FOOD TECHNION ISRAEL INST HAIFA ISRAEL (1)
Levi Cs (1)	FOOD TECHNION ISRAEL INST HAIFA ISRAEL (1)
Kashi Y (1)	FOOD TECHNION ISRAEL INST HAIFA ISRAEL (1)
Freilich S (1)	FOOD TECHNION ISRAEL INST HAIFA ISRAEL (1)
David-birman T (1)	FOOD TECHNION ISRAEL INST HAIFA ISRAEL (1)
Lesmes U (8)	FOOD TECHNION ISRAEL INST HAIFA ISRAEL (7)
Tsermoula P (1)	FOOD UNIV CORK WESTERN ROAD CORK T12 YN60 IRELAND (1)
O'neill Ee (1)	FOOD UNIV CORK WESTERN ROAD CORK T12 YN60 IRELAND (1)
O'flaherty Eaa (1)	FOOD UNIV CORK WESTERN ROAD CORK T12 YN60 IRELAND (1)

Ivusic polic I (2)	FOOD UNIV GUELPHON N1G 2W1 CANADA (1)
Chandran matheyambath A (2)	FOOD UNIV GUELPHON N1G 2W1 CANADA (1)
Sontag-strohm T (1)	FOOD UNIV HELSINKI P O BOX 66FIN FINLAND (1)
Mäkelä N (1)	FOOD UNIV HELSINKI P O BOX 66FIN FINLAND (1)
Hodson Mp (1)	FOOD UNIV QUEENSLAND4072 AUSTRALIA (1)
Flanagan Bm (1)	FOOD UNIV QUEENSLAND4072 AUSTRALIA (1)
Williams Ba (2)	FOOD UNIV QUEENSLAND4072 AUSTRALIA (2)
Low Dy (2)	FOOD UNIV QUEENSLAND4072 AUSTRALIA (2)
Gidley Mj (2)	FOOD UNIV QUEENSLAND4072 AUSTRALIA (2)
D'arcy Br (2)	FOOD UNIV QUEENSLAND4072 AUSTRALIA (2)
Tomassen Mm (1)	FOOD WAGENINGEN UR BORNSE WEILANDEN WAGENINGEN 6708WG NETHERLANDS (1)
Bai Y (1)	FOSHAN UNIV FOSHAN CHINA (1)
Schweiggert-weisz U (1)	FRAUNHOFER DE (1)
Naumann S (1)	FRAUNHOFER DE (1)
Heinlein A (1)	FRAUNHOFER DE (1)
Haller D (1)	FRAUNHOFER DE (1)
Eisner P (1)	FRAUNHOFER DE (1)
Eglmeier J (1)	FRAUNHOFER DE (1)
Buettner A (1)	FRAUNHOFER DE (1)
van der vossen Jmbm (1)	FRESENIUS DE (1)
Stover Jf (1)	FRESENIUS DE (1)
Schwejda-guettes S (1)	FRESENIUS DE (1)
Kuchinka-koch A (1)	FRESENIUS DE (1)
Koehler A (1)	FRESENIUS DE (1)
Gaigg B (1)	FRESENIUS DE (1)
Bothe Mk (1)	FRESENIUS DE (1)
Berressem D (1)	FRESENIUS DE (1)
Bellmann S (2)	FRESENIUS DE (1)
Verpoorte E (1)	FRIESLANDCAMPINA AMERSFOORT NETHERLANDS (1)
Van lieshout Gaa (1)	FRIESLANDCAMPINA AMERSFOORT NETHERLANDS (1)
Triantis V (1)	FRIESLANDCAMPINA AMERSFOORT NETHERLANDS (1)
Schuren Fhj (1)	FRIESLANDCAMPINA AMERSFOORT NETHERLANDS (1)
Savelkoul Hfj (1)	FRIESLANDCAMPINA AMERSFOORT NETHERLANDS (1)
Perdijk O (1)	FRIESLANDCAMPINA AMERSFOORT NETHERLANDS (1)
Neerven Rjjv (1)	FRIESLANDCAMPINA AMERSFOORT NETHERLANDS (1)
Mathwig K (1)	FRIESLANDCAMPINA AMERSFOORT NETHERLANDS (1)
Ianovska Ma (1)	FRIESLANDCAMPINA AMERSFOORT NETHERLANDS (1)
Fernandez-gutierrez Mm (1)	FRIESLANDCAMPINA AMERSFOORT NETHERLANDS (1)
De haan P (1)	FRIESLANDCAMPINA AMERSFOORT NETHERLANDS (1)
Brugman S (1)	FRIESLANDCAMPINA AMERSFOORT NETHERLANDS (1)
Brink Ev (1)	FRIESLANDCAMPINA AMERSFOORT NETHERLANDS (1)
Bouwmeester H (1)	FRIESLANDCAMPINA AMERSFOORT NETHERLANDS (1)
Baarlen Pv (1)	FRIESLANDCAMPINA AMERSFOORT NETHERLANDS (1)
Nam D-g (1)	FUNCTIONAL FOOD AGROFOOD RESOURCES NATL INST RURAL DEVELOPMENT ADMINISTRATION JEONJU SOUTH KOREA (1)
Im P (1)	FUNCTIONAL FOOD AGROFOOD RESOURCES NATL INST RURAL DEVELOPMENT ADMINISTRATION JEONJU SOUTH KOREA (1)
Choi A-j (1)	FUNCTIONAL FOOD AGROFOOD RESOURCES NATL INST RURAL DEVELOPMENT ADMINISTRATION JEONJU SOUTH KOREA (1)
Choe J-s (1)	FUNCTIONAL FOOD AGROFOOD RESOURCES NATL INST RURAL DEVELOPMENT ADMINISTRATION JEONJU SOUTH KOREA (1)
Satokaria R (1)	FUNCTIONAL FOODS FORUM UNIV TURKU TURKU FINLAND (1)
Reunanen J (1)	FUNCTIONAL FOODS FORUM UNIV TURKU TURKU FINLAND (1)
Ottman N (1)	FUNCTIONAL FOODS FORUM UNIV TURKU TURKU FINLAND (1)
Kainulainen V (1)	FUNCTIONAL FOODS FORUM UNIV TURKU TURKU FINLAND (1)
Huuskonen L (1)	FUNCTIONAL FOODS FORUM UNIV TURKU TURKU FINLAND (1)
Huhtinen H (1)	FUNCTIONAL FOODS FORUM UNIV TURKU TURKU FINLAND (1)
Tunesi M (1)	G NATTA POLITECNICO MILANO PIAZZA LEONARDO DA VINCI MILAN ITALY (1)
Raimondi Mt (1)	G NATTA POLITECNICO MILANO PIAZZA LEONARDO DA VINCI MILAN ITALY (1)
Laganà M (1)	G NATTA POLITECNICO MILANO PIAZZA LEONARDO DA VINCI MILAN ITALY (1)
Giordano C (1)	G NATTA POLITECNICO MILANO PIAZZA LEONARDO DA VINCI MILAN ITALY (1)
Boeri L (1)	G NATTA POLITECNICO MILANO PIAZZA LEONARDO DA VINCI MILAN ITALY (1)
Hoyles L (1)	GANEDEN BIOTECH INC MAYFIELD HEIGHTS OH UNITED STATES (1)
Mccartney Al (2)	GANEDEN BIOTECH INC MAYFIELD HEIGHTS OH UNITED STATES (2)

Honda H (2)	GANEDEN BIOTECH INC MAYFIELD HEIGHTS OH UNITED STATES (2)
van dinter R (1)	GANEDEN INC LANDERBROOK DRIVE MAYFIELD HEIGHTS OH UNITED STATES (1)
Cash H (2)	GANEDEN INC LANDERBROOK DRIVE MAYFIELD HEIGHTS OH UNITED STATES (2)
Szymanski K (1)	GASTROIMMUNOLOGY INST FAC UNIV LODZ LODZ POLAND (1)
Obuchowski M (1)	GASTROIMMUNOLOGY INST FAC UNIV LODZ LODZ POLAND (1)
Moran Ap (1)	GASTROIMMUNOLOGY INST FAC UNIV LODZ LODZ POLAND (1)
Mnich E (1)	GASTROIMMUNOLOGY INST FAC UNIV LODZ LODZ POLAND (1)
Hinc K (1)	GASTROIMMUNOLOGY INST FAC UNIV LODZ LODZ POLAND (1)
Gajewski A (1)	GASTROIMMUNOLOGY INST FAC UNIV LODZ LODZ POLAND (1)
Chmiela M (1)	GASTROIMMUNOLOGY INST FAC UNIV LODZ LODZ POLAND (1)
Hernot Dc (1)	GENERAL MILLS US (1)
Fahey jr Gc (1)	GENERAL MILLS US (1)
Boileau Tw (1)	GENERAL MILLS US (1)
Zorraquín-peña I (2)	GENOMICS CTR RES PUBLIC FISABIO FOUNDATIONVALENCIA SPAIN (1)
López-lópez A (1)	GENOMICS CTR RES PUBLIC FISABIO FOUNDATIONVALENCIA SPAIN (1)
Mira A (2)	GENOMICS CTR RES PUBLIC FISABIO FOUNDATIONVALENCIA SPAIN (2)
Ferrer Md (2)	GENOMICS CTR RES PUBLIC FISABIO FOUNDATIONVALENCIA SPAIN (2)
Woolston J (1)	GHENT UNIV GHENT BELGIUM (1)
Vissenaekens H (1)	GHENT UNIV GHENT BELGIUM (1)
Vervaeck A (1)	GHENT UNIV GHENT BELGIUM (1)
Vermeirssen V (2)	GHENT UNIV GHENT BELGIUM (1)
Verbeken K (1)	GHENT UNIV GHENT BELGIUM (1)
Vanlancker E (1)	GHENT UNIV GHENT BELGIUM (1)
Van wijmelbeke L (1)	GHENT UNIV GHENT BELGIUM (1)
Van hoorebeke L (1)	GHENT UNIV GHENT BELGIUM (1)
Van de wielea T (1)	GHENT UNIV GHENT BELGIUM (1)
Tomas-barberan Fa (1)	GHENT UNIV GHENT BELGIUM (1)
Stringer A (1)	GHENT UNIV GHENT BELGIUM (1)
Selma Mv (1)	GHENT UNIV GHENT BELGIUM (1)
Rymenans L (1)	GHENT UNIV GHENT BELGIUM (1)
Romo-vaquero M (1)	GHENT UNIV GHENT BELGIUM (1)
Rajanna das C (1)	GHENT UNIV GHENT BELGIUM (1)
Quinten T (1)	GHENT UNIV GHENT BELGIUM (1)
Props R (1)	GHENT UNIV GHENT BELGIUM (1)
Peru Km (1)	GHENT UNIV GHENT BELGIUM (1)
Moye Zd (1)	GHENT UNIV GHENT BELGIUM (1)
Miclotte L (1)	GHENT UNIV GHENT BELGIUM (1)
Joan V (1)	GHENT UNIV GHENT BELGIUM (1)
Hillewaere Xkd (1)	GHENT UNIV GHENT BELGIUM (1)
Headley Jv (1)	GHENT UNIV GHENT BELGIUM (1)
Hansen L (1)	GHENT UNIV GHENT BELGIUM (1)
Hamelink S (1)	GHENT UNIV GHENT BELGIUM (1)
García-villalba R (1)	GHENT UNIV GHENT BELGIUM (1)
Espín Jc (1)	GHENT UNIV GHENT BELGIUM (1)
Derave W (1)	GHENT UNIV GHENT BELGIUM (1)
Claeys B (1)	GHENT UNIV GHENT BELGIUM (1)
Ceuppens S (1)	GHENT UNIV GHENT BELGIUM (1)
Callewaert C (1)	GHENT UNIV GHENT BELGIUM (1)
Boone Mn (1)	GHENT UNIV GHENT BELGIUM (1)
Blancquaert L (1)	GHENT UNIV GHENT BELGIUM (1)
Arroyo Mc (1)	GHENT UNIV GHENT BELGIUM (1)
Almeida A (1)	GHENT UNIV GHENT BELGIUM (1)
Uyttendaele M (2)	GHENT UNIV GHENT BELGIUM (2)
Sánchez-patán F (2)	GHENT UNIV GHENT BELGIUM (2)
Remon Jp (2)	GHENT UNIV GHENT BELGIUM (2)
Rajkovic A (2)	GHENT UNIV GHENT BELGIUM (2)
Jiménez-girón A (3)	GHENT UNIV GHENT BELGIUM (2)
El hage R (2)	GHENT UNIV GHENT BELGIUM (2)
De weirdt R (4)	GHENT UNIV GHENT BELGIUM (2)
De beer T (2)	GHENT UNIV GHENT BELGIUM (2)
Van de wiele T (68)	GHENT UNIV GHENT BELGIUM (29)
Vervaet C (3)	GHENT UNIV GHENT BELGIUM (3)
Requena T (5)	GHENT UNIV GHENT BELGIUM (3)
Raes K (3)	GHENT UNIV GHENT BELGIUM (3)
Raes J (5)	GHENT UNIV GHENT BELGIUM (3)

Peláez C (5)	GHENT UNIV GHENT BELGIUM (3)
Martínez-cuesta Mc (5)	GHENT UNIV GHENT BELGIUM (3)
Martín-alvarez Pj (4)	GHENT UNIV GHENT BELGIUM (3)
Kerckhof F-m (4)	GHENT UNIV GHENT BELGIUM (3)
Decroos K (5)	GHENT UNIV GHENT BELGIUM (3)
De bodt J (4)	GHENT UNIV GHENT BELGIUM (3)
Barroso E (4)	GHENT UNIV GHENT BELGIUM (3)
Roussel C (4)	GHENT UNIV GHENT BELGIUM (4)
Laukens D (5)	GHENT UNIV GHENT BELGIUM (4)
Smagghe G (5)	GHENT UNIV GHENT BELGIUM (5)
Van camp J (7)	GHENT UNIV GHENT BELGIUM (6)
Grootaert C (11)	GHENT UNIV GHENT BELGIUM (6)
De paepe K (9)	GHENT UNIV GHENT BELGIUM (6)
Verstraete W (36)	GHENT UNIV GHENT BELGIUM (7)
Van meervenne E (1)	GHENT UNIV SALISBURYLAAN MERELBEKE BELGIUM (1)
Rombouts C (1)	GHENT UNIV SALISBURYLAAN MERELBEKE BELGIUM (1)
Hemeryck Ly (2)	GHENT UNIV SALISBURYLAAN MERELBEKE BELGIUM (1)
De paepe E (1)	GHENT UNIV SALISBURYLAAN MERELBEKE BELGIUM (1)
Van coillie E (2)	GHENT UNIV SALISBURYLAAN MERELBEKE BELGIUM (2)
Lambrecht E (2)	GHENT UNIV SALISBURYLAAN MERELBEKE BELGIUM (2)
Eeckhaut V (2)	GHENT UNIV SALISBURYLAAN MERELBEKE BELGIUM (2)
Di silvio D (1)	GIULIO NATTA POLITECNICO MILANO MILAN ITALY (1)
Baldelli bombelli F (1)	GIULIO NATTA POLITECNICO MILANO MILAN ITALY (1)
Bajka B (1)	GIULIO NATTA POLITECNICO MILANO MILAN ITALY (1)
Rizzo D (1)	GLAXOSMITHKLINE GB US (1)
Naylor Ta (1)	GLAXOSMITHKLINE GB US (1)
Connolly Pc (1)	GLAXOSMITHKLINE GB US (1)
Butler Jm (1)	GLAXOSMITHKLINE GB US (1)
Roy R (1)	GROUP UNIV MELBOURNEVIC AUSTRALIA (1)
Nguyen-ngo C (1)	GROUP UNIV MELBOURNEVIC AUSTRALIA (1)
Lappas M (1)	GROUP UNIV MELBOURNEVIC AUSTRALIA (1)
Tsao R (1)	GUELPH FOOD RES CTR AGRI FOOD CANADA GUELPH CANADA (1)
Renaud J (1)	GUELPH FOOD RES CTR AGRI FOOD CANADA GUELPH CANADA (1)
Liu R (1)	GUELPH FOOD RES CTR AGRI FOOD CANADA GUELPH CANADA (1)
Hassan Yi (1)	GUELPH FOOD RES CTR AGRI FOOD CANADA GUELPH CANADA (1)
Stewart Cs (2)	GUT ROWETT RES INST BUCKBURN ABERDEEN AB21 9SB UNITED KINGDOM (1)
Stanley Kn (1)	GUT ROWETT RES INST BUCKBURN ABERDEEN AB21 9SB UNITED KINGDOM (1)
Richardson Aj (1)	GUT ROWETT RES INST BUCKBURN ABERDEEN AB21 9SB UNITED KINGDOM (1)
Mcwilliam leitch Ec (1)	GUT ROWETT RES INST BUCKBURN ABERDEEN AB21 9SB UNITED KINGDOM (1)
Laven Ra (1)	GUT ROWETT RES INST BUCKBURN ABERDEEN AB21 9SB UNITED KINGDOM (1)
Welling Gw (1)	GUT ROWETT RES INST GREENBURN ROAD BUCKSBURN ABERDEEN AB21 9SB UNITED KINGDOM (1)
Scott Kp (1)	GUT ROWETT RES INST GREENBURN ROAD BUCKSBURN ABERDEEN AB21 9SB UNITED KINGDOM (1)
Ramsay Ag (1)	GUT ROWETT RES INST GREENBURN ROAD BUCKSBURN ABERDEEN AB21 9SB UNITED KINGDOM (1)
Harmsen Hjm (1)	GUT ROWETT RES INST GREENBURN ROAD BUCKSBURN ABERDEEN AB21 9SB UNITED KINGDOM (1)
Zelicha H (2)	HARVARD BOSTON MA UNITED STATES (2)
Lapidot M (2)	HARVARD BOSTON MA UNITED STATES (2)
Ceglarek U (2)	HARVARD BOSTON MA UNITED STATES (2)
Sträuber H (1)	HELMHOLTZ CTR RES UFZ LEIPZIG GERMANY (1)
Baleeiro Fcf (1)	HELMHOLTZ CTR RES UFZ LEIPZIG GERMANY (1)
Ardila Ms (1)	HELMHOLTZ CTR RES UFZ LEIPZIG GERMANY (1)
Müller S (4)	HELMHOLTZ CTR RES UFZ LEIPZIG GERMANY (2)
Kleinsteuber S (2)	HELMHOLTZ CTR RES UFZ LEIPZIG GERMANY (2)
Kovatcheva-datchary P (1)	HENKEL DE (1)
Robinson Pj (1)	HENRY M JACKSON FOUNDATION BETHESDA MD UNITED STATES (1)
Nelson Mt (1)	HENRY M JACKSON FOUNDATION BETHESDA MD UNITED STATES (1)
Merrill Ea (1)	HENRY M JACKSON FOUNDATION BETHESDA MD UNITED STATES (1)
Mauzy Ca (1)	HENRY M JACKSON FOUNDATION BETHESDA MD UNITED STATES (1)
Lubkowicz D (1)	HENRY M JACKSON FOUNDATION BETHESDA MD UNITED STATES (1)
Holt C (1)	HENRY M JACKSON FOUNDATION BETHESDA MD UNITED STATES (1)
Greenwood Es (1)	HENRY M JACKSON FOUNDATION BETHESDA MD UNITED STATES (1)
Coia Hg (1)	HENRY M JACKSON FOUNDATION BETHESDA MD UNITED STATES (1)

Charbonneau Mr (1)	HENRY M JACKSON FOUNDATION BETHESDA MD UNITED STATES (1)
Castillo Mj (1)	HENRY M JACKSON FOUNDATION BETHESDA MD UNITED STATES (1)
Sulek K (1)	HERLEV HOSP HERLEV DENMARK (1)
Steenholdt C (1)	HERLEV HOSP HERLEV DENMARK (1)
Licht Tr (1)	HERLEV HOSP HERLEV DENMARK (1)
Frandsen Hl (1)	HERLEV HOSP HERLEV DENMARK (1)
Brynskov J (1)	HERLEV HOSP HERLEV DENMARK (1)
Sultanbawa Y (1)	HORTICULTURE TSHWANE UNIV PRETORIA WEST SOUTH AFRICA (1)
Shoko T (1)	HORTICULTURE TSHWANE UNIV PRETORIA WEST SOUTH AFRICA (1)
Seke F (1)	HORTICULTURE TSHWANE UNIV PRETORIA WEST SOUTH AFRICA (1)
Manhivi Ve (1)	HORTICULTURE TSHWANE UNIV PRETORIA WEST SOUTH AFRICA (1)
Slabbert Rm (2)	HORTICULTURE TSHWANE UNIV PRETORIA WEST SOUTH AFRICA (2)
Sivakumar D (2)	HORTICULTURE TSHWANE UNIV PRETORIA WEST SOUTH AFRICA (2)
Pedersen G (1)	HVIDOVRE UNIV HOSP HVIDOVRE DENMARK (1)
Weir Jc (1)	ILLUMINA US (1)
Tjalsma H (1)	ILLUMINA US (1)
Swinkels Dw (1)	ILLUMINA US (1)
Nielsen Fgg (1)	ILLUMINA US (1)
Kortman Gam (1)	ILLUMINA US (1)
Kluijtmans Laj (1)	ILLUMINA US (1)
Kingsbury Z (1)	ILLUMINA US (1)
Keegan Kp (1)	ILLUMINA US (1)
Engelke Uf (1)	ILLUMINA US (1)
Dutilh Be (1)	ILLUMINA US (1)
Boekhorst J (1)	ILLUMINA US (1)
Betley J (1)	ILLUMINA US (1)
Bravo Ll (1)	IMDEA FOOD INST CEI UAM CSIC CARRETERA CANTO BLANCO MADRID SPAIN (1)
Tabernero M (2)	IMDEA FOOD INST CEI UAM CSIC CARRETERA CANTO BLANCO MADRID SPAIN (2)
van heel Da (1)	IMPERIAL LONDON LONDON UNITED KINGDOM (1)
Playford Rj (1)	IMPERIAL LONDON LONDON UNITED KINGDOM (1)
Ng C-y (1)	IMPERIAL LONDON LONDON UNITED KINGDOM (1)
Lechler R (1)	IMPERIAL LONDON LONDON UNITED KINGDOM (1)
Ghosh S (1)	IMPERIAL LONDON LONDON UNITED KINGDOM (1)
Butler M (1)	IMPERIAL LONDON LONDON UNITED KINGDOM (1)
Zoetendal E (1)	INRA FR (1)
Wiele Tvd (1)	INRA FR (1)
Walker Vk (1)	INRA FR (1)
Wahl R (1)	INRA FR (1)
Wagner N (1)	INRA FR (1)
Villemejane C (1)	INRA FR (1)
Viau M (1)	INRA FR (1)
Verbeke E (1)	INRA FR (1)
Valenzuela R (1)	INRA FR (1)
Ulleberg Ek (1)	INRA FR (1)
Turgeon S (1)	INRA FR (1)
Tavares L (1)	INRA FR (1)
Tavares A (1)	INRA FR (1)
Stoffers H (1)	INRA FR (1)
Spano G (1)	INRA FR (1)
Souquet J-m (1)	INRA FR (1)
Smeets-peeters M (1)	INRA FR (1)
Smeds Ai (1)	INRA FR (1)
Siriaco A (1)	INRA FR (1)
Sion B (1)	INRA FR (1)
Schroën K (1)	INRA FR (1)
Schmidt H (1)	INRA FR (1)
Saulnier L (1)	INRA FR (1)
Samtlebe M (1)	INRA FR (1)
Salvador V (1)	INRA FR (1)
Russo P (1)	INRA FR (1)
Rumney C (1)	INRA FR (1)
Rousselon N (1)	INRA FR (1)
Roussel Y (1)	INRA FR (1)
Roume H (1)	INRA FR (1)
Rouau X (1)	INRA FR (1)

Rosa Nn (1)	INRA FR (1)
Ropers M-h (1)	INRA FR (1)
Rioux L-e (1)	INRA FR (1)
Rhazi L (2)	INRA FR (1)
Reygner J (1)	INRA FR (1)
Renard Cmgc (1)	INRA FR (1)
Reboul E (1)	INRA FR (1)
Quehl A (1)	INRA FR (1)
Privat M (1)	INRA FR (1)
Popineau Y (1)	INRA FR (1)
Poirson C (1)	INRA FR (1)
Pihlanto A (1)	INRA FR (1)
Picque D (1)	INRA FR (1)
Peyret P (1)	INRA FR (1)
Perrin C (1)	INRA FR (1)
Ouethrani M (1)	INRA FR (1)
Nowicki M (1)	INRA FR (1)
Neve H (1)	INRA FR (1)
Navarrete P (1)	INRA FR (1)
Nagengast F (1)	INRA FR (1)
Murray Bs (1)	INRA FR (1)
Moniz K (1)	INRA FR (1)
Mondot S (1)	INRA FR (1)
Molle D (1)	INRA FR (1)
Mills Enc (1)	INRA FR (1)
Micard V (1)	INRA FR (1)
Mayeur C (1)	INRA FR (1)
Mat Djl (1)	INRA FR (1)
Marol-bonnin S (1)	INRA FR (1)
Margier M (1)	INRA FR (1)
Maillot M (1)	INRA FR (1)
Magne L (1)	INRA FR (1)
Lumeau S (1)	INRA FR (1)
Lucas P (1)	INRA FR (1)
Lorson E (1)	INRA FR (1)
Léonil J (1)	INRA FR (1)
Lecannu G (1)	INRA FR (1)
Le roux Y (1)	INRA FR (1)
Le bourvellec C (1)	INRA FR (1)
Lápez P (1)	INRA FR (1)
Laillet B (1)	INRA FR (1)
Lahaye M (1)	INRA FR (1)
Kössö T (1)	INRA FR (1)
Kemperman Ra (1)	INRA FR (1)
Junjua M (1)	INRA FR (1)
Jensen B-b (1)	INRA FR (1)
Jarrige J-f (1)	INRA FR (1)
Janssenduijghuijsen L (1)	INRA FR (1)
Holopainen-mantila U (1)	INRA FR (1)
Hinrichs J (1)	INRA FR (1)
Heyerick A (1)	INRA FR (1)
Halimi C (1)	INRA FR (1)
Guillon F (1)	INRA FR (1)
Guillemin H (1)	INRA FR (1)
Gotteland M (3)	INRA FR (1)
Godon J-j (1)	INRA FR (1)
Gérard P (1)	INRA FR (1)
Georgé S (1)	INRA FR (1)
Galmiche Jp (1)	INRA FR (1)
Franz C (1)	INRA FR (1)
Fonty G (1)	INRA FR (1)
Foligné B (1)	INRA FR (1)
Flourié B (1)	INRA FR (1)
Fernández M (1)	INRA FR (1)
Fernández de palencia P (1)	INRA FR (1)

Faulds Cb (1)	INRA FR (1)
Fardet A (1)	INRA FR (1)
El oufir L (1)	INRA FR (1)
Dudefoi W (1)	INRA FR (1)
Doo E-h (1)	INRA FR (1)
Dhooge W (1)	INRA FR (1)
Devaux M-f (1)	INRA FR (1)
Depeint F (2)	INRA FR (1)
Delort-laval J (1)	INRA FR (1)
Delgenès J-p (1)	INRA FR (1)
Delgado-andrade C (1)	INRA FR (1)
Dary A (1)	INRA FR (1)
Corstens Mn (1)	INRA FR (1)
Corpet De (1)	INRA FR (1)
Condette Cj (1)	INRA FR (1)
Cloarec D (1)	INRA FR (1)
Cires Mj (1)	INRA FR (1)
Cheynier V (1)	INRA FR (1)
Champ M (1)	INRA FR (1)
Carvalho M (1)	INRA FR (1)
Carrasco-pozo C (1)	INRA FR (1)
Camel V (1)	INRA FR (1)
Bouzerzour K (1)	INRA FR (1)
Bornet F (1)	INRA FR (1)
Bonnet C (1)	INRA FR (1)
Bonnarme P (1)	INRA FR (1)
Bolca S (2)	INRA FR (1)
Blachier F (1)	INRA FR (1)
Bes M (1)	INRA FR (1)
Bertrand D (1)	INRA FR (1)
Berton-carabin Cc (1)	INRA FR (1)
Bernalier A (1)	INRA FR (1)
Belleville C (1)	INRA FR (1)
Bazzocco S (1)	INRA FR (1)
Barry Jl (1)	INRA FR (1)
Balance S (1)	INRA FR (1)
Aymard P (1)	INRA FR (1)
Awussi Aa (1)	INRA FR (1)
Atamer Z (1)	INRA FR (1)
Antoine T (1)	INRA FR (1)
Andriamihaja M (1)	INRA FR (1)
Aidy ei S (1)	INRA FR (1)
Abdennebi-najar L (1)	INRA FR (1)
Dupont D (10)	INRA FR (10)
Souchon I (2)	INRA FR (2)
Sebedio J-l (2)	INRA FR (2)
Pujos E (2)	INRA FR (2)
Picariello G (3)	INRA FR (2)
Papon Y (2)	INRA FR (2)
Michon C (2)	INRA FR (2)
Meynier A (2)	INRA FR (2)
Martins C (3)	INRA FR (2)
Martin J-f (2)	INRA FR (2)
Jardin J (2)	INRA FR (2)
Hoebler C (3)	INRA FR (2)
Guyot S (2)	INRA FR (2)
Clemente A (2)	INRA FR (2)
Broudiscou L-p (2)	INRA FR (2)
Broudiscou Af (2)	INRA FR (2)
Assunção R (3)	INRA FR (2)
Rabot S (3)	INRA FR (3)
Macierzanka A (3)	INRA FR (3)
Cattenoz T (3)	INRA FR (3)
Bruneau A (3)	INRA FR (3)
Alvito P (4)	INRA FR (3)

Mackie Ar (4)	INRA FR (4)
Cherbut C (4)	INRA FR (4)
Brodkorb A (5)	INRA FR (4)
Barry J-l (5)	INRA FR (4)
Le feunteun S (5)	INRA FR (5)
Egger L (6)	INRA FR (5)
Bourlieu C (5)	INRA FR (5)
Ménard O (9)	INRA FR (9)
Vernay T (1)	INSERM FR (1)
Thomas M (1)	INSERM FR (1)
Thevenot-sergentet D (1)	INSERM FR (1)
Tamanai-shacoori Z (1)	INSERM FR (1)
Sassi M (1)	INSERM FR (1)
Miossec C (1)	INSERM FR (1)
Loréal O (1)	INSERM FR (1)
Lajoie F (1)	INSERM FR (1)
Kapel N (1)	INSERM FR (1)
Jolivet-gougeon A (1)	INSERM FR (1)
Javaudin F (1)	INSERM FR (1)
Ishnaiwer M (1)	INSERM FR (1)
Gall Sd-l (1)	INSERM FR (1)
Gaboriau F (1)	INSERM FR (1)
Fournier E (1)	INSERM FR (1)
Dion M (1)	INSERM FR (1)
Deschamps C (1)	INSERM FR (1)
de gunzburg J (1)	INSERM FR (1)
Comtet-marre S (1)	INSERM FR (1)
Cherbuy C (1)	INSERM FR (1)
Cannie I (1)	INSERM FR (1)
Burel A (1)	INSERM FR (1)
Bousarghin L (1)	INSERM FR (1)
Bezabih Y (1)	INSERM FR (1)
Bemer P (1)	INSERM FR (1)
Batard E (1)	INSERM FR (1)
Andremont A (1)	INSERM FR (1)
Almeida M (2)	INSERM FR (1)
Cordonnier C (2)	INSERM FR (2)
Thévenot J (3)	INSERM FR (3)
Ying W (1)	INST AGRO PRODUCT PROCESSING JIANGSU ACADEMY NANJING JIANGSU CHINA (1)
Ying L (1)	INST AGRO PRODUCT PROCESSING JIANGSU ACADEMY NANJING JIANGSU CHINA (1)
Xiudong X (1)	INST AGRO PRODUCT PROCESSING JIANGSU ACADEMY NANJING JIANGSU CHINA (1)
Xiaoli L (1)	INST AGRO PRODUCT PROCESSING JIANGSU ACADEMY NANJING JIANGSU CHINA (1)
Jianzhong Z (1)	INST AGRO PRODUCT PROCESSING JIANGSU ACADEMY NANJING JIANGSU CHINA (1)
Muñoz La (2)	INST AGROCHEMISTRY FOOD IATA C CATEDRATICO AGUSTIN ESCARDINO BENLLOCH PATERNA SPAIN (2)
Steck J (1)	INST BIOSCIENCES FOOD PHYTOCHEMISTRY KARLSRUHE INST KARLSRUHE GERMANY (1)
Schmid V (1)	INST BIOSCIENCES FOOD PHYTOCHEMISTRY KARLSRUHE INST KARLSRUHE GERMANY (1)
Mayer-miebach E (1)	INST BIOSCIENCES FOOD PHYTOCHEMISTRY KARLSRUHE INST KARLSRUHE GERMANY (1)
Karbstein Hp (1)	INST BIOSCIENCES FOOD PHYTOCHEMISTRY KARLSRUHE INST KARLSRUHE GERMANY (1)
Emin Ma (1)	INST BIOSCIENCES FOOD PHYTOCHEMISTRY KARLSRUHE INST KARLSRUHE GERMANY (1)
Bunzel M (1)	INST BIOSCIENCES FOOD PHYTOCHEMISTRY KARLSRUHE INST KARLSRUHE GERMANY (1)
Behsnilian D (1)	INST BIOSCIENCES FOOD PHYTOCHEMISTRY KARLSRUHE INST KARLSRUHE GERMANY (1)

Zamora O (1)	INST CIENCIA TECNOLOGIA ALIMENTOS ICYTAL FACULTAD CIENCIAS AGRARIAS UNIV AUSTRAL CHILE AV JULIO SARRAZIN S N CAMPUS ISLA TEJA VALDIVIA CHILE (1)
Vergara C (1)	INST CIENCIA TECNOLOGIA ALIMENTOS ICYTAL FACULTAD CIENCIAS AGRARIAS UNIV AUSTRAL CHILE AV JULIO SARRAZIN S N CAMPUS ISLA TEJA VALDIVIA CHILE (1)
Uribe M (1)	INST CIENCIA TECNOLOGIA ALIMENTOS ICYTAL FACULTAD CIENCIAS AGRARIAS UNIV AUSTRAL CHILE AV JULIO SARRAZIN S N CAMPUS ISLA TEJA VALDIVIA CHILE (1)
Pino Mt (1)	INST CIENCIA TECNOLOGIA ALIMENTOS ICYTAL FACULTAD CIENCIAS AGRARIAS UNIV AUSTRAL CHILE AV JULIO SARRAZIN S N CAMPUS ISLA TEJA VALDIVIA CHILE (1)
Pérez R (1)	INST CIENCIA TECNOLOGIA ALIMENTOS ICYTAL FACULTAD CIENCIAS AGRARIAS UNIV AUSTRAL CHILE AV JULIO SARRAZIN S N CAMPUS ISLA TEJA VALDIVIA CHILE (1)
Kalazich J (1)	INST CIENCIA TECNOLOGIA ALIMENTOS ICYTAL FACULTAD CIENCIAS AGRARIAS UNIV AUSTRAL CHILE AV JULIO SARRAZIN S N CAMPUS ISLA TEJA VALDIVIA CHILE (1)
Olmedilla-alonso B (1)	INST CIENCIA TECNOLOGIA ALIMENTOS NUTRICION ICTAN CSIC MADRID SPAIN (1)
Martinez-rodriguez Aj (1)	INST CIENCIA TECNOLOGIA ALIMENTOS NUTRICION ICTAN CSIC MADRID SPAIN (1)
Ganan M (1)	INST CIENCIA TECNOLOGIA ALIMENTOS NUTRICION ICTAN CSIC MADRID SPAIN (1)
Estévez-santiago R (1)	INST CIENCIA TECNOLOGIA ALIMENTOS NUTRICION ICTAN CSIC MADRID SPAIN (1)
De pascual-teresa S (1)	INST CIENCIA TECNOLOGIA ALIMENTOS NUTRICION ICTAN CSIC MADRID SPAIN (1)
Carrascosa Av (1)	INST CIENCIA TECNOLOGIA ALIMENTOS NUTRICION ICTAN CSIC MADRID SPAIN (1)
Wall-medrano A (1)	INST CIENCIAS BIOMEDICAS CIENCIAS QUIMICO BIOLOGICAS UNIV AUTONOMA CIUDAD JUAREZ ANILLO ENVOLVENTE DEL PRONAF ESTOCOLMO S N CIUDAD JUAREZ CHIHUAHUA MEXICO (1)
Vázquez-landaverde Pa (1)	INST CIENCIAS BIOMEDICAS CIENCIAS QUIMICO BIOLOGICAS UNIV AUTONOMA CIUDAD JUAREZ ANILLO ENVOLVENTE DEL PRONAF ESTOCOLMO S N CIUDAD JUAREZ CHIHUAHUA MEXICO (1)
Reyes-vega Ml (1)	INST CIENCIAS BIOMEDICAS CIENCIAS QUIMICO BIOLOGICAS UNIV AUTONOMA CIUDAD JUAREZ ANILLO ENVOLVENTE DEL PRONAF ESTOCOLMO S N CIUDAD JUAREZ CHIHUAHUA MEXICO (1)
Herrera-cazares La (1)	INST CIENCIAS BIOMEDICAS CIENCIAS QUIMICO BIOLOGICAS UNIV AUTONOMA CIUDAD JUAREZ ANILLO ENVOLVENTE DEL PRONAF ESTOCOLMO S N CIUDAD JUAREZ CHIHUAHUA MEXICO (1)
Gaytán-martínez M (2)	INST CIENCIAS BIOMEDICAS CIENCIAS QUIMICO BIOLOGICAS UNIV AUTONOMA CIUDAD JUAREZ ANILLO ENVOLVENTE DEL PRONAF ESTOCOLMO S N CIUDAD JUAREZ CHIHUAHUA MEXICO (1)
Rogelj I (1)	INST DAIRY PROBIOTICS BIOTECHNICAL FAC UNIV LJUBLJANA GROBLJE DOMZALE SLOVENIA (1)
Leroy F (1)	INST DAIRY PROBIOTICS BIOTECHNICAL FAC UNIV LJUBLJANA GROBLJE DOMZALE SLOVENIA (1)
Redzynia M (1)	INST FERMENTATION WOLCZANSKA LODZ POLAND (1)
Podsdek A (1)	INST FERMENTATION WOLCZANSKA LODZ POLAND (1)
Koziolkiewicz M (1)	INST FERMENTATION WOLCZANSKA LODZ POLAND (1)
Klewicka E (1)	INST FERMENTATION WOLCZANSKA LODZ POLAND (1)
Zhao S (1)	INST FOOD CHINESE ACADEMY BEIJING CHINA (1)
Zhao C (1)	INST FOOD CHINESE ACADEMY BEIJING CHINA (1)
Tian G (1)	INST FOOD CHINESE ACADEMY BEIJING CHINA (1)
Pérez-jiménez J (1)	INST FOOD ICTAN CSIC MADRID SPAIN (1)
Martínez-villaluenga C (1)	INST FOOD ICTAN CSIC MADRID SPAIN (1)
Bravo L (2)	INST FOOD ICTAN CSIC MADRID SPAIN (2)
Alvarez Md (2)	INST FOOD ICTAN CSIC MADRID SPAIN (2)
Sarriá B (3)	INST FOOD ICTAN CSIC MADRID SPAIN (3)
Mateos R (3)	INST FOOD ICTAN CSIC MADRID SPAIN (3)
Herranz B (3)	INST FOOD ICTAN CSIC MADRID SPAIN (3)
Salt L (1)	INST FOOD PRODUCTION NAT RES BARI ITALY (1)
Monaci L (1)	INST FOOD PRODUCTION NAT RES BARI ITALY (1)

De angelis E (1)	INST FOOD PRODUCTION NAT RES BARI ITALY (1)
Visconti A (2)	INST FOOD PRODUCTION NAT RES BARI ITALY (2)
Silva M (1)	INST FOOD RES CIAL CSIC UAM C NICOLAS CABRERA MADRID SPAIN (1)
Molinero N (1)	INST FOOD RES CIAL CSIC UAM C NICOLAS CABRERA MADRID SPAIN (1)
de llano Dg (1)	INST FOOD RES CIAL CSIC UAM C NICOLAS CABRERA MADRID SPAIN (1)
Gil-sánchez I (4)	INST FOOD RES CIAL CSIC UAM C NICOLAS CABRERA MADRID SPAIN (2)
Laguna L (4)	INST FOOD RES CIAL CSIC UAM C NICOLAS CABRERA MADRID SPAIN (3)
Bartolomé B (10)	INST FOOD RES CIAL CSIC UAM C NICOLAS CABRERA MADRID SPAIN (4)
Cueva C (9)	INST FOOD RES CIAL CSIC UAM C NICOLAS CABRERA MADRID SPAIN (5)
Tamargo A (7)	INST FOOD RES CIAL CSIC UAM C NICOLAS CABRERA MADRID SPAIN (6)
Moreno-arribas Mv (12)	INST FOOD RES CIAL CSIC UAM C NICOLAS CABRERA MADRID SPAIN (6)
Wickham Mjs (1)	INST FOOD RES NORWICH NR4 7UA UNITED KINGDOM (1)
Pin C (1)	INST FOOD RES NORWICH NR4 7UA UNITED KINGDOM (1)
Nueno palop C (1)	INST FOOD RES NORWICH NR4 7UA UNITED KINGDOM (1)
Mann J (1)	INST FOOD RES NORWICH NR4 7UA UNITED KINGDOM (1)
George Sm (1)	INST FOOD RES NORWICH NR4 7UA UNITED KINGDOM (1)
Gasson M (1)	INST FOOD RES NORWICH NR4 7UA UNITED KINGDOM (1)
Christakopoulos P (1)	INST FOOD RES NORWICH NR4 7UA UNITED KINGDOM (1)
Baranyi J (1)	INST FOOD RES NORWICH NR4 7UA UNITED KINGDOM (1)
Avendaño-pérez G (1)	INST FOOD RES NORWICH NR4 7UA UNITED KINGDOM (1)
Nueno-palop C (2)	INST FOOD RES NORWICH NR4 7UA UNITED KINGDOM (2)
Mercuri A (2)	INST FOOD RES NORWICH NR4 7UA UNITED KINGDOM (2)
Huatan H (2)	INST FOOD RES NORWICH NR4 7UA UNITED KINGDOM (2)
Chessa S (2)	INST FOOD RES NORWICH NR4 7UA UNITED KINGDOM (2)
Vardakou M (3)	INST FOOD RES NORWICH NR4 7UA UNITED KINGDOM (3)
Rigby N (4)	INST FOOD RES NORWICH NR4 7UA UNITED KINGDOM (3)
Narbad A (5)	INST FOOD RES NORWICH NR4 7UA UNITED KINGDOM (3)
Wickham Msj (9)	INST FOOD RES NORWICH NR4 7UA UNITED KINGDOM (5)
Mackie A (8)	INST FOOD RES NORWICH NR4 7UA UNITED KINGDOM (5)
Faulks Rm (6)	INST FOOD RES NORWICH NR4 7UA UNITED KINGDOM (5)
Sheffield Cl (1)	INST FOOD RES NORWICH RES PARK NORWICH UNITED KINGDOM (1)
Nisbet Dj (1)	INST FOOD RES NORWICH RES PARK NORWICH UNITED KINGDOM (1)
Crippen Tl (1)	INST FOOD RES NORWICH RES PARK NORWICH UNITED KINGDOM (1)
Bongaerts Rj (1)	INST FOOD RES NORWICH RES PARK NORWICH UNITED KINGDOM (1)
Andrews K (1)	INST FOOD RES NORWICH RES PARK NORWICH UNITED KINGDOM (1)
Zhou W (1)	INST FOOD ZHEJIANG ACADEMY HANGZHOU CHINA (1)
Xing J (1)	INST FOOD ZHEJIANG ACADEMY HANGZHOU CHINA (1)
Lin Y (1)	INST FOOD ZHEJIANG ACADEMY HANGZHOU CHINA (1)
Wendland I (1)	INST FRITZ HABER WEG KARLSRUHE GERMANY (1)
Schepers U (1)	INST FRITZ HABER WEG KARLSRUHE GERMANY (1)
Rapp Be (1)	INST FRITZ HABER WEG KARLSRUHE GERMANY (1)
Kattge S (1)	INST FRITZ HABER WEG KARLSRUHE GERMANY (1)
Kappings V (1)	INST FRITZ HABER WEG KARLSRUHE GERMANY (1)
Ivannikov D (1)	INST FRITZ HABER WEG KARLSRUHE GERMANY (1)
Hettel M (1)	INST FRITZ HABER WEG KARLSRUHE GERMANY (1)
Hebeiss I (1)	INST FRITZ HABER WEG KARLSRUHE GERMANY (1)
Grün C (1)	INST FRITZ HABER WEG KARLSRUHE GERMANY (1)
Deutschmann O (1)	INST FRITZ HABER WEG KARLSRUHE GERMANY (1)
Orsat V (1)	INST FUNCTIONAL FOODS QUEBECQC CANADA (1)
Moss R (1)	INST FUNCTIONAL FOODS QUEBECQC CANADA (1)
Maheux M (1)	INST FUNCTIONAL FOODS QUEBECQC CANADA (1)
Lightburn B (1)	INST FUNCTIONAL FOODS QUEBECQC CANADA (1)
Lafrance C-p (1)	INST FUNCTIONAL FOODS QUEBECQC CANADA (1)
Brochu M (1)	INST FUNCTIONAL FOODS QUEBECQC CANADA (1)
Bélair V (1)	INST FUNCTIONAL FOODS QUEBECQC CANADA (1)
Tufail T (1)	INST HOME FOOD GOVERNMENT UNIV FAISALABAD PAKISTAN (1)
Saeed M (1)	INST HOME FOOD GOVERNMENT UNIV FAISALABAD PAKISTAN (1)
Saeed F (1)	INST HOME FOOD GOVERNMENT UNIV FAISALABAD PAKISTAN (1)
Nadeem Mt (1)	INST HOME FOOD GOVERNMENT UNIV FAISALABAD PAKISTAN (1)
Arshad Mu (1)	INST HOME FOOD GOVERNMENT UNIV FAISALABAD PAKISTAN (1)
Afzaal M (1)	INST HOME FOOD GOVERNMENT UNIV FAISALABAD PAKISTAN (1)
Xiao J (2)	INST INNOVATIVE DEVELOPMENT FOOD INDUSTRY SHENZHEN UNIV SHENZHEN CHINA (2)
Suo H (2)	INST INNOVATIVE DEVELOPMENT FOOD INDUSTRY SHENZHEN UNIV SHENZHEN CHINA (2)

Pozo-bayón Ma (1)	INST INVESTIGACION EN CIENCIAS ALIMENTACION CIAL CSIC UAM SPAIN (1)
Dueñas M (1)	INST INVESTIGACION EN CIENCIAS ALIMENTACION CIAL CSIC UAM SPAIN (1)
Carrillo W (1)	INST INVESTIGACION EN CIENCIAS ALIMENTACION CIAL CSIC UAM SPAIN (1)
Vilcacundo R (2)	INST INVESTIGACION EN CIENCIAS ALIMENTACION CIAL CSIC UAM SPAIN (2)
Simsek S (2)	INST INVESTIGACION EN CIENCIAS ALIMENTACION CIAL CSIC UAM SPAIN (2)
Muñoz-gonzález I (2)	INST INVESTIGACION EN CIENCIAS ALIMENTACION CIAL CSIC UAM SPAIN (2)
Esteban-fernández A (3)	INST INVESTIGACION EN CIENCIAS ALIMENTACION CIAL CSIC UAM SPAIN (2)
Santos Cn (3)	INST INVESTIGACION EN CIENCIAS ALIMENTACION CIAL CSIC UAM SPAIN (3)
Marze S (3)	INST INVESTIGACION EN CIENCIAS ALIMENTACION CIAL CSIC UAM SPAIN (3)
Hernández-ledesma B (3)	INST INVESTIGACION EN CIENCIAS ALIMENTACION CIAL CSIC UAM SPAIN (3)
Miralles B (6)	INST INVESTIGACION EN CIENCIAS ALIMENTACION CIAL CSIC UAM SPAIN (4)
Karakaya S (4)	INST INVESTIGACION EN CIENCIAS ALIMENTACION CIAL CSIC UAM SPAIN (4)
Recio I (7)	INST INVESTIGACION EN CIENCIAS ALIMENTACION CIAL CSIC UAM SPAIN (6)
Vinderola G (3)	INST LACTOLOGIA INDUSTRIAL INLAIN UNL CONICET FACULTAD INGENIERIA QUIMICA UNIV NACIONAL DEL LITORAL SANTA FE ARGENTINA (3)
Tegetmeyer He (1)	INST MAX PLANCK DE (1)
Strous M (1)	INST MAX PLANCK DE (1)
Schnorr Sl (1)	INST MAX PLANCK DE (1)
Meier D (1)	INST MAX PLANCK DE (1)
Marlowe Fw (1)	INST MAX PLANCK DE (1)
Kraft B (1)	INST MAX PLANCK DE (1)
Henry Ag (1)	INST MAX PLANCK DE (1)
Geelhoed Js (1)	INST MAX PLANCK DE (1)
Crittenden An (1)	INST MAX PLANCK DE (1)
Xiao J-z (1)	INST MORINAGA MILK INDUSTRY CO LTD ZAMA JAPAN (1)
Odamaki T (1)	INST MORINAGA MILK INDUSTRY CO LTD ZAMA JAPAN (1)
Murakami R (1)	INST MORINAGA MILK INDUSTRY CO LTD ZAMA JAPAN (1)
Hashikura N (1)	INST MORINAGA MILK INDUSTRY CO LTD ZAMA JAPAN (1)
Preciado-ortiz R (1)	INST NACIONAL INVESTIGACIONES FORESTALES AGRICOLAS PECUARIAS INIFAP CAMPO BAJIO APARTADO POSTAL CELAYA GUANAJUATO MEXICO (1)
Mendoza S (1)	INST NACIONAL INVESTIGACIONES FORESTALES AGRICOLAS PECUARIAS INIFAP CAMPO BAJIO APARTADO POSTAL CELAYA GUANAJUATO MEXICO (1)
Puhakka Ja (1)	INST OT TAMPERE UNIV P O BOX FIN TAMPERE FINLAND (1)
Koskinen Pep (1)	INST OT TAMPERE UNIV P O BOX FIN TAMPERE FINLAND (1)
Tornero-martínez A (1)	INST POLITECNICO NACIONAL CEPROBI CARRETERA YAUTEPEC JOJUTLA KM YAUTEPEC C P MORELOS MEXICO (1)
Osorio-díaz P (2)	INST POLITECNICO NACIONAL CEPROBI CARRETERA YAUTEPEC JOJUTLA KM YAUTEPEC C P MORELOS MEXICO (1)
Mora-escobedo R (2)	INST POLITECNICO NACIONAL CEPROBI CARRETERA YAUTEPEC JOJUTLA KM YAUTEPEC C P MORELOS MEXICO (1)
Jaramillo-flores Me (1)	INST POLITECNICO NACIONAL CEPROBI CARRETERA YAUTEPEC JOJUTLA KM YAUTEPEC C P MORELOS MEXICO (1)
Cruz-ortiz R (1)	INST POLITECNICO NACIONAL CEPROBI CARRETERA YAUTEPEC JOJUTLA KM YAUTEPEC C P MORELOS MEXICO (1)
Ávila-reyes Sv (1)	INST POLITECNICO NACIONAL CEPROBI CARRETERA YAUTEPEC JOJUTLA KM YAUTEPEC C P MORELOS MEXICO (1)
Alvarado-jasso Gm (1)	INST POLITECNICO NACIONAL CEPROBI CARRETERA YAUTEPEC JOJUTLA KM YAUTEPEC C P MORELOS MEXICO (1)
Tapia-maruri D (1)	INST POLITECNICO NACIONAL CEPROBI KM CARRETERA YAUTEPEC JOJUTLA COL SAN ISIDRO YAUTEPEC MORELOS MEXICO (1)
Pérez-pérez V (1)	INST POLITECNICO NACIONAL CEPROBI KM CARRETERA YAUTEPEC JOJUTLA COL SAN ISIDRO YAUTEPEC MORELOS MEXICO (1)
Cano-sampedro E (1)	INST POLITECNICO NACIONAL CEPROBI KM CARRETERA YAUTEPEC JOJUTLA COL SAN ISIDRO YAUTEPEC MORELOS MEXICO (1)
Camacho-díaz Bh (1)	INST POLITECNICO NACIONAL CEPROBI KM CARRETERA YAUTEPEC JOJUTLA COL SAN ISIDRO YAUTEPEC MORELOS MEXICO (1)
Alamilla-beltrán L (1)	INST POLITECNICO NACIONAL CEPROBI KM CARRETERA YAUTEPEC JOJUTLA COL SAN ISIDRO YAUTEPEC MORELOS MEXICO (1)
von stockar U (1)	INST SWISS FEDERAL INST EPFL LAUSANNE CH SWITZERLAND (1)
Stoll T (1)	INST SWISS FEDERAL INST EPFL LAUSANNE CH SWITZERLAND (1)
Perregaux C (1)	INST SWISS FEDERAL INST EPFL LAUSANNE CH SWITZERLAND (1)
Marison Iw (1)	INST SWISS FEDERAL INST EPFL LAUSANNE CH SWITZERLAND (1)
Sabinski F (1)	INST UNIV MUNSTER HUFFERSTRASSE D MUNSTER GERMANY (1)
Leusmann Db (1)	INST UNIV MUNSTER HUFFERSTRASSE D MUNSTER GERMANY (1)
Mishra Ak (1)	INTEGRATIVE BIOSCIENCES VIT UNIV VELLORE TAMIL NADU INDIA (1)

Ghosh Ar (1)	INTEGRATIVE BIOSCIENCES VIT UNIV VELLORE TAMIL NADU INDIA (1)
Naidoo V (1)	INTERNATIONAL CTR GENOMIC MEDICINE EUROESPES BIOMEDICAL RES CTR BERGONDO SPAIN (1)
Martínez-iglesias O (1)	INTERNATIONAL CTR GENOMIC MEDICINE EUROESPES BIOMEDICAL RES CTR BERGONDO SPAIN (1)
Carrera I (1)	INTERNATIONAL CTR GENOMIC MEDICINE EUROESPES BIOMEDICAL RES CTR BERGONDO SPAIN (1)
Cacabelos R (1)	INTERNATIONAL CTR GENOMIC MEDICINE EUROESPES BIOMEDICAL RES CTR BERGONDO SPAIN (1)
Cacabelos N (1)	INTERNATIONAL CTR GENOMIC MEDICINE EUROESPES BIOMEDICAL RES CTR BERGONDO SPAIN (1)
Vo Td (1)	INTERTEK SCIENTIFIC REGULATORY CONSULTANCY ARGENTIA ROAD SUITE MISSISSAUGA L5N 2X7 CANADA (1)
Roberts A (1)	INTERTEK SCIENTIFIC REGULATORY CONSULTANCY ARGENTIA ROAD SUITE MISSISSAUGA L5N 2X7 CANADA (1)
Lynch Bs (1)	INTERTEK SCIENTIFIC REGULATORY CONSULTANCY ARGENTIA ROAD SUITE MISSISSAUGA L5N 2X7 CANADA (1)
Sulakvelidze A (2)	INTRALYTIX INC COLUMBUS CTR E PRATT STREET BALTIMORE MD UNITED STATES (2)
Van saene Jjm (1)	INVERESK INTERNATIONAL LTD TRANENT EH33 2NE UNITED KINGDOM (1)
Stamm Jm (1)	INVERESK INTERNATIONAL LTD TRANENT EH33 2NE UNITED KINGDOM (1)
Nouws Jfm (1)	INVERESK INTERNATIONAL LTD TRANENT EH33 2NE UNITED KINGDOM (1)
Mc conville Ml (1)	INVERESK INTERNATIONAL LTD TRANENT EH33 2NE UNITED KINGDOM (1)
Dijkstra Jw (1)	INVERESK INTERNATIONAL LTD TRANENT EH33 2NE UNITED KINGDOM (1)
Pérez álvarez Ja (1)	IPOA RES GROUP AGRO FOOD ESCUELA POLITECNICA SUPERIOR ORIHUELA MIGUEL HERNANDEZ UNIV ORIHUELA ALICANTE SPAIN (1)
Pérez-álvarez Já (4)	IPOA RES GROUP AGRO FOOD ESCUELA POLITECNICA SUPERIOR ORIHUELA MIGUEL HERNANDEZ UNIV ORIHUELA ALICANTE SPAIN (3)
Lucas-gonzalez R (4)	IPOA RES GROUP AGRO FOOD ESCUELA POLITECNICA SUPERIOR ORIHUELA MIGUEL HERNANDEZ UNIV ORIHUELA ALICANTE SPAIN (3)
Viuda-martos M (5)	IPOA RES GROUP AGRO FOOD ESCUELA POLITECNICA SUPERIOR ORIHUELA MIGUEL HERNANDEZ UNIV ORIHUELA ALICANTE SPAIN (4)
Fernández-lópez J (5)	IPOA RES GROUP AGRO FOOD ESCUELA POLITECNICA SUPERIOR ORIHUELA MIGUEL HERNANDEZ UNIV ORIHUELA ALICANTE SPAIN (4)
Morera M (1)	IPSEN FR (1)
Bussolo de souza C (3)	JOHAN KARSCHSTRAAT WAGENINGEN TN NETHERLANDS (3)
Shinkura R (1)	JST JP (1)
Mori T (1)	JST JP (1)
Inoue J (1)	JST JP (1)
Hoshi N (1)	JST JP (1)
Zerr W (1)	JUSTUS LIEBIG UNIV GIEßEN INST IZF GIEßEN GERMANY (1)
Schnell S (1)	JUSTUS LIEBIG UNIV GIEßEN INST IZF GIEßEN GERMANY (1)
Schmidt M (1)	JUSTUS LIEBIG UNIV GIEßEN INST IZF GIEßEN GERMANY (1)
Ratering S (1)	JUSTUS LIEBIG UNIV GIEßEN INST IZF GIEßEN GERMANY (1)
Kramer I (1)	JUSTUS LIEBIG UNIV GIEßEN INST IZF GIEßEN GERMANY (1)
Kampmann K (1)	JUSTUS LIEBIG UNIV GIEßEN INST IZF GIEßEN GERMANY (1)
Simpson Bk (2)	KHORASAN RAZAVI NATURAL RESOURCES RES CTR AREEO MASHHAD IRAN (2)
Yilmaz Mt (1)	KING ABDULAZIZ UNIV FAC INDUSTRIAL JEDDAH SAUDI ARABIA (1)
Taylan O (1)	KING ABDULAZIZ UNIV FAC INDUSTRIAL JEDDAH SAUDI ARABIA (1)
Karakas Cy (1)	KING ABDULAZIZ UNIV FAC INDUSTRIAL JEDDAH SAUDI ARABIA (1)
Sharp Pa (1)	KINGS COLLEGE LONDON GB (1)
O'brien P (1)	KINGS COLLEGE LONDON GB (1)
Ganis Jc (1)	KINGS COLLEGE LONDON GB (1)
Corpe Cp (1)	KINGS COLLEGE LONDON GB (1)
Christides T (1)	KINGS COLLEGE LONDON GB (1)
Zenhausern F (1)	KOCH US (1)
Wilmes P (1)	KOCH US (1)
Shah P (1)	KOCH US (1)
Seguin-devaux C (1)	KOCH US (1)
Niegowska M (1)	KOCH US (1)
Jäger C (1)	KOCH US (1)
Greenhalgh K (1)	KOCH US (1)
Glaab E (1)	KOCH US (1)
Fritz Jv (1)	KOCH US (1)
Frachet A (1)	KOCH US (1)

Estes M (1)	KOCH US (1)
Desai Ms (1)	KOCH US (1)
Ha S-y (1)	KOREA MARITIME OCEAN UNIV BUSAN SOUTH KOREA (1)
Koh S-c (2)	KOREA MARITIME OCEAN UNIV BUSAN SOUTH KOREA (2)
Kim I-s (2)	KOREA MARITIME OCEAN UNIV BUSAN SOUTH KOREA (2)
Wimmer Rf (1)	KRAFT FOODS US (1)
Petzold iii He (1)	KRAFT FOODS US (1)
Fernández H (1)	KRAFT FOODS US (1)
Fernández Ah (1)	KRAFT FOODS US (1)
Batra Mr (1)	KRAFT FOODS US (1)
Bartholomew Mj (1)	KRAFT FOODS US (1)
Bartholomew M (1)	KRAFT FOODS US (1)
Simon Ma (2)	KRAFT FOODS US (2)
Miller Ma (2)	KRAFT FOODS US (2)
Van loey A (1)	KU LEUVEN MICROBIAL SYSTEMS MSUP2 SUPS LEUVEN FOOD RES CTR LFORCE FOOD KASTEELPARK ARENBERG PB LEUVEN BELGIUM (1)
Pallares pallares A (1)	KU LEUVEN MICROBIAL SYSTEMS MSUP2 SUPS LEUVEN FOOD RES CTR LFORCE FOOD KASTEELPARK ARENBERG PB LEUVEN BELGIUM (1)
Pälchen K (1)	KU LEUVEN MICROBIAL SYSTEMS MSUP2 SUPS LEUVEN FOOD RES CTR LFORCE FOOD KASTEELPARK ARENBERG PB LEUVEN BELGIUM (1)
Michels D (1)	KU LEUVEN MICROBIAL SYSTEMS MSUP2 SUPS LEUVEN FOOD RES CTR LFORCE FOOD KASTEELPARK ARENBERG PB LEUVEN BELGIUM (1)
Hendrickx M (1)	KU LEUVEN MICROBIAL SYSTEMS MSUP2 SUPS LEUVEN FOOD RES CTR LFORCE FOOD KASTEELPARK ARENBERG PB LEUVEN BELGIUM (1)
Gwala S (1)	KU LEUVEN MICROBIAL SYSTEMS MSUP2 SUPS LEUVEN FOOD RES CTR LFORCE FOOD KASTEELPARK ARENBERG PB LEUVEN BELGIUM (1)
Duijsens D (1)	KU LEUVEN MICROBIAL SYSTEMS MSUP2 SUPS LEUVEN FOOD RES CTR LFORCE FOOD KASTEELPARK ARENBERG PB LEUVEN BELGIUM (1)
Terpend K (1)	LABMET GHENT UNIV COUPURE LINKS GHENT BELGIUM (1)
Kerckhof Fm (1)	LABMET GHENT UNIV COUPURE LINKS GHENT BELGIUM (1)
De mulder T (1)	LABMET GHENT UNIV COUPURE LINKS GHENT BELGIUM (1)
Daguet D (3)	LABMET GHENT UNIV COUPURE LINKS GHENT BELGIUM (3)
Boon N (12)	LABMET GHENT UNIV COUPURE LINKS GHENT BELGIUM (4)
Quirós-sauceda Ae (1)	LABORATORIO ANTIOXIDANTES ALIMENTOS FUNCIONALES CTR INVESTIGACION EN ALIMENTACION DESARROLLO A C CARRETERA A VICTORIA KM HERMOSILLO SONORA CP MEXICO (1)
Mercado-mercado G (1)	LABORATORIO ANTIOXIDANTES ALIMENTOS FUNCIONALES CTR INVESTIGACION EN ALIMENTACION DESARROLLO A C CARRETERA A VICTORIA KM HERMOSILLO SONORA CP MEXICO (1)
González-aguilar Ga (4)	LABORATORIO ANTIOXIDANTES ALIMENTOS FUNCIONALES CTR INVESTIGACION EN ALIMENTACION DESARROLLO A C CARRETERA A VICTORIA KM HERMOSILLO SONORA CP MEXICO (2)
Blancas-benitez Fj (2)	LABORATORIO ANTIOXIDANTES ALIMENTOS FUNCIONALES CTR INVESTIGACION EN ALIMENTACION DESARROLLO A C CARRETERA A VICTORIA KM HERMOSILLO SONORA CP MEXICO (2)
Montalvo-gonzález E (4)	LABORATORIO INTEGRAL INVESTIGACION EN ALIMENTOS TECNOLOGICO NACIONAL MEXICO INST TECNOLOGICO TEPIC TEPIC MEXICO (2)
Vallejo-cordoba B (1)	LABORATORIO QUIMICA BIOTECNOLOGIA PRODUCTOS LACTEOS CTR INVESTIGACION EN ALIMENTACION DESARROLLO A C HERMOSILLO MEXICO (1)
Torres-gregorio M (1)	LABORATORIO QUIMICA BIOTECNOLOGIA PRODUCTOS LACTEOS CTR INVESTIGACION EN ALIMENTACION DESARROLLO A C HERMOSILLO MEXICO (1)
Santiago-lópez L (1)	LABORATORIO QUIMICA BIOTECNOLOGIA PRODUCTOS LACTEOS CTR INVESTIGACION EN ALIMENTACION DESARROLLO A C HERMOSILLO MEXICO (1)
Hernandez-mendoza A (1)	LABORATORIO QUIMICA BIOTECNOLOGIA PRODUCTOS LACTEOS CTR INVESTIGACION EN ALIMENTACION DESARROLLO A C HERMOSILLO MEXICO (1)
González-córdova Af (1)	LABORATORIO QUIMICA BIOTECNOLOGIA PRODUCTOS LACTEOS CTR INVESTIGACION EN ALIMENTACION DESARROLLO A C HERMOSILLO MEXICO (1)
Garcia Hs (1)	LABORATORIO QUIMICA BIOTECNOLOGIA PRODUCTOS LACTEOS CTR INVESTIGACION EN ALIMENTACION DESARROLLO A C HERMOSILLO MEXICO (1)
Schmeda-hirschmann G (2)	LABORATORIO QUIMICA PRODUCTOS NATURALES INST QUIMICA RECURSOS NATURALES UNIV TALCA TALCA CHILE (2)
Burgos-edwards A (2)	LABORATORIO QUIMICA PRODUCTOS NATURALES INST QUIMICA RECURSOS NATURALES UNIV TALCA TALCA CHILE (2)
Gresse R (2)	LALLEMAND SAS BLAGNAC FRANCE (2)
Chaucheyras-durand F (2)	LALLEMAND SAS BLAGNAC FRANCE (2)

Davies K (2)	LEEDS TEACHING HOSPITALS NHS TRUST LEEDS UNITED KINGDOM (2)
Vázquez-campos S (1)	LEITAT TECHNOLOGICAL CTR C INNOVACIO TERRASSA SPAIN (1)
Janer G (1)	LEITAT TECHNOLOGICAL CTR C INNOVACIO TERRASSA SPAIN (1)
Fernández-rosas E (1)	LEITAT TECHNOLOGICAL CTR C INNOVACIO TERRASSA SPAIN (1)
Delpivo C (1)	LEITAT TECHNOLOGICAL CTR C INNOVACIO TERRASSA SPAIN (1)
Cabellos J (1)	LEITAT TECHNOLOGICAL CTR C INNOVACIO TERRASSA SPAIN (1)
Yadav Ak (1)	LOVELY PROFESSIONAL UNIV PHAGWARA PUNJAB INDIA (1)
Vaidya Y (1)	LOVELY PROFESSIONAL UNIV PHAGWARA PUNJAB INDIA (1)
Singh Sk (1)	LOVELY PROFESSIONAL UNIV PHAGWARA PUNJAB INDIA (1)
Prudhviraj G (1)	LOVELY PROFESSIONAL UNIV PHAGWARA PUNJAB INDIA (1)
Kaur P (1)	LOVELY PROFESSIONAL UNIV PHAGWARA PUNJAB INDIA (1)
Gulati M (1)	LOVELY PROFESSIONAL UNIV PHAGWARA PUNJAB INDIA (1)
Lidén G (1)	LUND UNIV P O BOX LUND SWEDEN (1)
Keller D (7)	MAASTRICHT UNIV CAMPUS VENLO HEALTHY EATING FOOD INNOVATION ST JANSWEG VENLO RC NETHERLANDS (2)
Farmer S (5)	MAASTRICHT UNIV CAMPUS VENLO HEALTHY EATING FOOD INNOVATION ST JANSWEG VENLO RC NETHERLANDS (2)
Savelkoul Phm (2)	MAASTRICHT UNIV CTR PO BOX MAASTRICHT AZ NETHERLANDS (2)
Budding Ae (2)	MAASTRICHT UNIV CTR PO BOX MAASTRICHT AZ NETHERLANDS (2)
Troost Fj (1)	MAASTRICHT UNIV MAASTRICHT LIMBURG NETHERLANDS (1)
van dooren I (1)	MASS SPECTROMETRY UNIV ANTWERP ANTWERP BELGIUM (1)
Theunis M (1)	MASS SPECTROMETRY UNIV ANTWERP ANTWERP BELGIUM (1)
Exarchou V (1)	MASS SPECTROMETRY UNIV ANTWERP ANTWERP BELGIUM (1)
Claeys M (1)	MASS SPECTROMETRY UNIV ANTWERP ANTWERP BELGIUM (1)
Apers S (1)	MASS SPECTROMETRY UNIV ANTWERP ANTWERP BELGIUM (1)
Zhao Q (2)	MASSEY UNIV AUCKLAND NEW ZEALAND (2)
Shu Q (2)	MASSEY UNIV AUCKLAND NEW ZEALAND (2)
Mutukumira A (2)	MASSEY UNIV AUCKLAND NEW ZEALAND (2)
Lee Sj (2)	MASSEY UNIV AUCKLAND NEW ZEALAND (2)
Larder Ce (2)	MCGILL UNIV LAKESHORE STE ANNE BELLEVUE QC H9X3V9 CANADA (1)
Tompkins Ta (3)	MCGILL UNIV LAKESHORE STE ANNE BELLEVUE QC H9X3V9 CANADA (2)
Macpherson Cw (2)	MCGILL UNIV LAKESHORE STE ANNE BELLEVUE QC H9X3V9 CANADA (2)
Gaisawat Mb (3)	MCGILL UNIV LAKESHORE STE ANNE BELLEVUE QC H9X3V9 CANADA (2)
Kubow S (9)	MCGILL UNIV LAKESHORE STE ANNE BELLEVUE QC H9X3V9 CANADA (4)
Iskandar Mm (6)	MCGILL UNIV LAKESHORE STE ANNE BELLEVUE QC H9X3V9 CANADA (4)
Swaby Am (1)	MCGILL UNIV MONTREAL QC CANADA (1)
Nassar A (1)	MCGILL UNIV MONTREAL QC CANADA (1)
Martoni C (1)	MCGILL UNIV MONTREAL QC CANADA (1)
Hussain R (1)	MCGILL UNIV MONTREAL QC CANADA (1)
Habib S (1)	MCGILL UNIV MONTREAL QC CANADA (1)
Agellon Lb (1)	MCGILL UNIV MONTREAL QC CANADA (1)
Abbasi S (1)	MCGILL UNIV MONTREAL QC CANADA (1)
Saha S (2)	MCGILL UNIV MONTREAL QC CANADA (2)
Paul A (4)	MCGILL UNIV MONTREAL QC CANADA (2)
Marinescu D (2)	MCGILL UNIV MONTREAL QC CANADA (2)
Tomaro-duchesneau C (4)	MCGILL UNIV MONTREAL QC CANADA (3)
Rodes L (6)	MCGILL UNIV MONTREAL QC CANADA (4)
Prakash S (10)	MCGILL UNIV MONTREAL QC CANADA (5)
Donnelly Dj (2)	MCGILL UNIV STE ANNE BELLEVUE QC H9X 3V9 CANADA (2)
Azadi B (2)	MCGILL UNIV STE ANNE BELLEVUE QC H9X 3V9 CANADA (2)
Sleno L (4)	MCGILL UNIV STE ANNE BELLEVUE QC H9X 3V9 CANADA (3)
Sabally K (4)	MCGILL UNIV STE ANNE BELLEVUE QC H9X 3V9 CANADA (3)
Rios-ibarra Cp (1)	MEDICINE ACADEMIC MEDICINE UNIV AUTONOMA ZACATECAS ZACATECAS MEXICO (1)
Ramirez-castillo Da (1)	MEDICINE ACADEMIC MEDICINE UNIV AUTONOMA ZACATECAS ZACATECAS MEXICO (1)
Martinez-fierro Ml (1)	MEDICINE ACADEMIC MEDICINE UNIV AUTONOMA ZACATECAS ZACATECAS MEXICO (1)
Luque-badillo Ac (1)	MEDICINE ACADEMIC MEDICINE UNIV AUTONOMA ZACATECAS ZACATECAS MEXICO (1)
Jacobo-velázquez Da (1)	MEDICINE ACADEMIC MEDICINE UNIV AUTONOMA ZACATECAS ZACATECAS MEXICO (1)
Hernandez-tapia G (1)	MEDICINE ACADEMIC MEDICINE UNIV AUTONOMA ZACATECAS ZACATECAS MEXICO (1)

Espinoza-serrano D (1)	MEDICINE ACADEMIC MEDICINE UNIV AUTONOMA ZACATECAS ZACATECAS MEXICO (1)
Cortes-limon Am (1)	MEDICINE ACADEMIC MEDICINE UNIV AUTONOMA ZACATECAS ZACATECAS MEXICO (1)
Cortes-gallardo Jp (1)	MEDICINE ACADEMIC MEDICINE UNIV AUTONOMA ZACATECAS ZACATECAS MEXICO (1)
Schaepkens E (1)	MEDICINE NUTRIM MAASTRICHT NETHERLANDS (1)
Pieters H-j (1)	MEDICINE NUTRIM MAASTRICHT NETHERLANDS (1)
Masclee A (1)	MEDICINE NUTRIM MAASTRICHT NETHERLANDS (1)
Ludidi S (1)	MEDICINE NUTRIM MAASTRICHT NETHERLANDS (1)
Kruimel J (1)	MEDICINE NUTRIM MAASTRICHT NETHERLANDS (1)
Elamin E (1)	MEDICINE NUTRIM MAASTRICHT NETHERLANDS (1)
Conchillo J (1)	MEDICINE NUTRIM MAASTRICHT NETHERLANDS (1)
Bours P (1)	MEDICINE NUTRIM MAASTRICHT NETHERLANDS (1)
Thompson Pl (1)	MEDICINE ROYAL PERTH HOSP UNIV WESTERN AUSTRALIA PERTH WA AUSTRALIA (1)
Prince Rl (1)	MEDICINE ROYAL PERTH HOSP UNIV WESTERN AUSTRALIA PERTH WA AUSTRALIA (1)
Lewis Jr (1)	MEDICINE ROYAL PERTH HOSP UNIV WESTERN AUSTRALIA PERTH WA AUSTRALIA (1)
Kerr Da (1)	MEDICINE ROYAL PERTH HOSP UNIV WESTERN AUSTRALIA PERTH WA AUSTRALIA (1)
Ivey Kl (1)	MEDICINE ROYAL PERTH HOSP UNIV WESTERN AUSTRALIA PERTH WA AUSTRALIA (1)
Hodgson Jm (1)	MEDICINE ROYAL PERTH HOSP UNIV WESTERN AUSTRALIA PERTH WA AUSTRALIA (1)
Stanton Mm (1)	MEDICINE UNIV CALGARY CTR HOSP DRIVE NW CALGARY AB T2N 4N1 CANADA (1)
Sproule-willoughby Km (1)	MEDICINE UNIV CALGARY CTR HOSP DRIVE NW CALGARY AB T2N 4N1 CANADA (1)
Rioux Kp (1)	MEDICINE UNIV CALGARY CTR HOSP DRIVE NW CALGARY AB T2N 4N1 CANADA (1)
Mckay Dm (1)	MEDICINE UNIV CALGARY CTR HOSP DRIVE NW CALGARY AB T2N 4N1 CANADA (1)
Ceri H (1)	MEDICINE UNIV CALGARY CTR HOSP DRIVE NW CALGARY AB T2N 4N1 CANADA (1)
Buret Ag (1)	MEDICINE UNIV CALGARY CTR HOSP DRIVE NW CALGARY AB T2N 4N1 CANADA (1)
Zechner El (1)	MEDICINE UNIV GRAZ GRAZ AUSTRIA (1)
Tesic D (1)	MEDICINE UNIV GRAZ GRAZ AUSTRIA (1)
Reisner A (1)	MEDICINE UNIV GRAZ GRAZ AUSTRIA (1)
Maierl M (1)	MEDICINE UNIV GRAZ GRAZ AUSTRIA (1)
Krause R (1)	MEDICINE UNIV GRAZ GRAZ AUSTRIA (1)
Jörger M (1)	MEDICINE UNIV GRAZ GRAZ AUSTRIA (1)
Haid A (1)	MEDICINE UNIV GRAZ GRAZ AUSTRIA (1)
Berger D (1)	MEDICINE UNIV GRAZ GRAZ AUSTRIA (1)
Espinosa L (1)	MERCK KGAA DE (1)
Courau S (1)	MERCK KGAA DE (1)
Storey De (1)	MERCK SHARP DOHME US (1)
Melia Cd (1)	MERCK SHARP DOHME US (1)
Mason Lm (1)	MERCK SHARP DOHME US (1)
Gupta P (1)	MERCK SHARP DOHME US (1)
Fadden K (1)	MERCK SHARP DOHME US (1)
Burley J (1)	MERCK SHARP DOHME US (1)
Vijayabharathi R (1)	MICROBIAL BHARATHIAR UNIV COIMBATORE INDIA (1)
Sathyabama S (1)	MICROBIAL BHARATHIAR UNIV COIMBATORE INDIA (1)
Ranjith kumar M (1)	MICROBIAL BHARATHIAR UNIV COIMBATORE INDIA (1)
Priyadarisini Vb (1)	MICROBIAL BHARATHIAR UNIV COIMBATORE INDIA (1)
Bruntha devi P (1)	MICROBIAL BHARATHIAR UNIV COIMBATORE INDIA (1)
Laing Gd (2)	MICROBIAL COUPURE LINKS GENT BELGIUM (2)
Heyndrickx M (3)	MICROBIAL COUPURE LINKS GENT BELGIUM (2)
Struijs K (1)	MICROBIAL FAC BIOSCIENCE GHENT UNIV B GHENT BELGIUM (1)
Eeckhaut E (2)	MICROBIAL FAC BIOSCIENCE GHENT UNIV B GHENT BELGIUM (1)
De keukeleire D (2)	MICROBIAL FAC BIOSCIENCE GHENT UNIV B GHENT BELGIUM (2)
Campbell D (2)	MICROBIAL FAC BIOSCIENCE GHENT UNIV B GHENT BELGIUM (2)
Uyttendaele S (1)	MICROBIAL GHENT UNIV COUPURE LINKS GHENT B BELGIUM (1)
Siciliano S (1)	MICROBIAL GHENT UNIV COUPURE LINKS GHENT B BELGIUM (1)
Peru K (1)	MICROBIAL GHENT UNIV COUPURE LINKS GHENT B BELGIUM (1)
Headley J (1)	MICROBIAL GHENT UNIV COUPURE LINKS GHENT B BELGIUM (1)
Boeckaert C (1)	MICROBIAL GHENT UNIV COUPURE LINKS GHENT B BELGIUM (1)
Vanhaecke L (6)	MICROBIAL GHENT UNIV COUPURE LINKS GHENT B BELGIUM (2)

de wiele Tv (4)	MICROBIAL GHENT UNIV COUPURE LINKS GHENT B BELGIUM (2)
Van herreweghen F (4)	MICROBIAL INTERACTIONS PROCESSES RES GROUP HELMHOLTZ CTR RES BRAUNSCHWEIG GERMANY (2)
Truchado P (3)	MICROBIAL INTERACTIONS PROCESSES RES GROUP HELMHOLTZ CTR RES BRAUNSCHWEIG GERMANY (2)
Jauregui R (4)	MICROBIAL INTERACTIONS PROCESSES RES GROUP HELMHOLTZ CTR RES BRAUNSCHWEIG GERMANY (4)
Vilchez-vargas R (6)	MICROBIAL INTERACTIONS PROCESSES RES GROUP HELMHOLTZ CTR RES BRAUNSCHWEIG GERMANY (5)
Last A (2)	MICROBIAL PATHOGENICITY MECHANISMS LEIBNIZ INST NATURAL PRODUCT RES HANS KNOELL INST JENA GERMANY (2)
Graf K (2)	MICROBIAL PATHOGENICITY MECHANISMS LEIBNIZ INST NATURAL PRODUCT RES HANS KNOELL INST JENA GERMANY (2)
Woestyne Mv (1)	MICROBIAL UNIV GENT BELGIUM (1)
Vande velde I (1)	MICROBIAL UNIV GENT BELGIUM (1)
Smet Id (1)	MICROBIAL UNIV GENT BELGIUM (1)
Kontula P (2)	MICROBIAL UNIV GENT BELGIUM (1)
Jaskari J (1)	MICROBIAL UNIV GENT BELGIUM (1)
Wouters R (3)	MICROBIAL UNIV GENT BELGIUM (2)
Vande woestyne M (2)	MICROBIAL UNIV GENT BELGIUM (2)
De smet I (3)	MICROBIAL UNIV GENT BELGIUM (2)
De boever P (4)	MICROBIAL UNIV GENT BELGIUM (2)
Molly K (3)	MICROBIAL UNIV GENT BELGIUM (3)
Nollet L (6)	MICROBIAL UNIV GENT BELGIUM (4)
So D (1)	MONASH UNIV ALFRED HOSP MELBOURNE AUSTRALIA (1)
Rotbart A (1)	MONASH UNIV ALFRED HOSP MELBOURNE AUSTRALIA (1)
Ou Jz (1)	MONASH UNIV ALFRED HOSP MELBOURNE AUSTRALIA (1)
Ha N (1)	MONASH UNIV ALFRED HOSP MELBOURNE AUSTRALIA (1)
Gill Pa (1)	MONASH UNIV ALFRED HOSP MELBOURNE AUSTRALIA (1)
Chrisp Md (1)	MONASH UNIV ALFRED HOSP MELBOURNE AUSTRALIA (1)
Yao Ck (2)	MONASH UNIV ALFRED HOSP MELBOURNE AUSTRALIA (2)
Muir Jg (2)	MONASH UNIV ALFRED HOSP MELBOURNE AUSTRALIA (2)
Gibson Pr (2)	MONASH UNIV ALFRED HOSP MELBOURNE AUSTRALIA (2)
Ferreira Icfr (2)	MOUNTAIN RES CTR CIMO ESA POLYTECHNIC INST BRAGANCA IPB CAMPUS SANTA APOLONIA BRAGANCA PORTUGAL (2)
Dias Mi (2)	MOUNTAIN RES CTR CIMO ESA POLYTECHNIC INST BRAGANCA IPB CAMPUS SANTA APOLONIA BRAGANCA PORTUGAL (2)
Barros L (2)	MOUNTAIN RES CTR CIMO ESA POLYTECHNIC INST BRAGANCA IPB CAMPUS SANTA APOLONIA BRAGANCA PORTUGAL (2)
Pygall Sr (1)	MSD DEVELOPMENT HERTFORD ROAD HODDESTON HERTFORDSHIRE EN11 9BU UNITED KINGDOM (1)
Mann Jc (1)	MSD DEVELOPMENT HERTFORD ROAD HODDESTON HERTFORDSHIRE EN11 9BU UNITED KINGDOM (1)
Oomah Bd (1)	NAT BIOPRODUCTS BIOPROCESSES PACIFIC AGRI FOOD RES CTR AGRI FOOD CANADA SUMMERLAND BC V0H 1Z0 CANADA (1)
Mendoza-díaz S (1)	NAT BIOPRODUCTS BIOPROCESSES PACIFIC AGRI FOOD RES CTR AGRI FOOD CANADA SUMMERLAND BC V0H 1Z0 CANADA (1)
López-barrera D (1)	NAT BIOPRODUCTS BIOPROCESSES PACIFIC AGRI FOOD RES CTR AGRI FOOD CANADA SUMMERLAND BC V0H 1Z0 CANADA (1)
Williams K (1)	NAT CTR TOXICOLOGICAL RES U S FOOD DRUG ADMINISTRATION NCTR RD JEFFERSON AK UNITED STATES (1)
Lahiani Mh (1)	NAT CTR TOXICOLOGICAL RES U S FOOD DRUG ADMINISTRATION NCTR RD JEFFERSON AK UNITED STATES (1)
Sa S-j (1)	NAT INST R D A SEONGHWAN SOUTH KOREA (1)
Park J-c (1)	NAT INST R D A SEONGHWAN SOUTH KOREA (1)
Lee S-d (1)	NAT INST R D A SEONGHWAN SOUTH KOREA (1)
Kim Y-h (1)	NAT INST R D A SEONGHWAN SOUTH KOREA (1)
Kim I-h (1)	NAT INST R D A SEONGHWAN SOUTH KOREA (1)
Kim I-c (1)	NAT INST R D A SEONGHWAN SOUTH KOREA (1)
Kim D-w (1)	NAT INST R D A SEONGHWAN SOUTH KOREA (1)
Jung H-j (1)	NAT INST R D A SEONGHWAN SOUTH KOREA (1)
Cho K-h (1)	NAT INST R D A SEONGHWAN SOUTH KOREA (1)
Chae S-j (1)	NAT INST R D A SEONGHWAN SOUTH KOREA (1)
Tsuda H (1)	NAT INST TOYAMA SHINJUKU KU TOKYO JAPAN (1)
Matsumoto T (1)	NAT INST TOYAMA SHINJUKU KU TOKYO JAPAN (1)

Ishimi Y (1)	NAT INST TOYAMA SHINJUKU KU TOKYO JAPAN (1)
Foubert K (2)	NATURAL PRODUCTS FOOD RES ANALYSIS NATURA UNIV ANTWERP ANTWERP BELGIUM (2)
Breynaert A (2)	NATURAL PRODUCTS FOOD RES ANALYSIS NATURA UNIV ANTWERP ANTWERP BELGIUM (2)
Bijttebier S (2)	NATURAL PRODUCTS FOOD RES ANALYSIS NATURA UNIV ANTWERP ANTWERP BELGIUM (2)
Pieters L (3)	NATURAL PRODUCTS FOOD RES ANALYSIS NATURA UNIV ANTWERP ANTWERP BELGIUM (3)
Hermans N (3)	NATURAL PRODUCTS FOOD RES ANALYSIS NATURA UNIV ANTWERP ANTWERP BELGIUM (3)
Tomé Tm (1)	NESTLE CH (1)
Tevere S (1)	NESTLE CH (1)
Sprenger N (1)	NESTLE CH (1)
Sartoratto A (1)	NESTLE CH (1)
Roughead Z (1)	NESTLE CH (1)
Rohfritsch Z (1)	NESTLE CH (1)
Roessle C (1)	NESTLE CH (1)
Qin B (1)	NESTLE CH (1)
Perina Np (1)	NESTLE CH (1)
Neutsch L (1)	NESTLE CH (1)
Mosquera Emb (1)	NESTLE CH (1)
Mathys A (1)	NESTLE CH (1)
Marsaux B (1)	NESTLE CH (1)
Lazarini T (1)	NESTLE CH (1)
Klosterbuer A (1)	NESTLE CH (1)
Hildebrand F (1)	NESTLE CH (1)
Giuffrida F (1)	NESTLE CH (1)
Dionisi F (1)	NESTLE CH (1)
Carpine R (1)	NESTLE CH (1)
Canelli G (1)	NESTLE CH (1)
Bolten Cj (1)	NESTLE CH (1)
Rochat F (2)	NESTLE CH (2)
Hartzell Al (2)	NESTLE CH (2)
O'may Ga (2)	NINEWELLS HOSP DUNDEE UNITED KINGDOM (2)
Rode Tm (1)	NOFIMA AS AS NORWAY (1)
Mcleod A (1)	NOFIMA AS AS NORWAY (1)
Måge I (1)	NOFIMA AS AS NORWAY (1)
Holck Al (1)	NOFIMA AS AS NORWAY (1)
Heir E (1)	NOFIMA AS AS NORWAY (1)
Axelsson L (1)	NOFIMA AS AS NORWAY (1)
Thieu Nq (1)	NONG LAM UNIV LINHTRUNG WARD THUDUC DISTRICT HOCHIMINH CITY VIET NAM (1)
Pettersson H (1)	NONG LAM UNIV LINHTRUNG WARD THUDUC DISTRICT HOCHIMINH CITY VIET NAM (1)
Vennos C (1)	NORTH EASTERN FEDERAL UNIV BELINSKY STREET YAKUTSK RUSSIAN FEDERATION (1)
Vasil'eva Ag (1)	NORTH EASTERN FEDERAL UNIV BELINSKY STREET YAKUTSK RUSSIAN FEDERATION (1)
Olennikov Dn (1)	NORTH EASTERN FEDERAL UNIV BELINSKY STREET YAKUTSK RUSSIAN FEDERATION (1)
Kashchenko Ni (1)	NORTH EASTERN FEDERAL UNIV BELINSKY STREET YAKUTSK RUSSIAN FEDERATION (1)
Isaev Ji (1)	NORTH EASTERN FEDERAL UNIV BELINSKY STREET YAKUTSK RUSSIAN FEDERATION (1)
Gadimli Ai (1)	NORTH EASTERN FEDERAL UNIV BELINSKY STREET YAKUTSK RUSSIAN FEDERATION (1)
Chirikova Nk (1)	NORTH EASTERN FEDERAL UNIV BELINSKY STREET YAKUTSK RUSSIAN FEDERATION (1)
Zann V (1)	NOVARTIS CH (1)
Timm D (1)	NOVARTIS CH (1)
Sousa T (1)	NOVARTIS CH (1)
Slavin J (1)	NOVARTIS CH (1)
Noack J (1)	NOVARTIS CH (1)
Hospattankar A (1)	NOVARTIS CH (1)

Borde A (1)	NOVARTIS CH (1)
Abrahamsson B (2)	NOVARTIS CH (1)
Trost K (1)	NOVONORDISK DK (1)
Saenz C (1)	NOVONORDISK DK (1)
Moritz T (1)	NOVONORDISK DK (1)
Khan A (2)	NOVONORDISK DK (1)
Hanteer O (1)	NOVONORDISK DK (1)
Gunalan V (1)	NOVONORDISK DK (1)
Gnanasekaran T (1)	NOVONORDISK DK (1)
Geraldo Ja (1)	NOVONORDISK DK (1)
Arumugam M (1)	NOVONORDISK DK (1)
Alvarez-silva C (1)	NOVONORDISK DK (1)
Ahrenkiel Dw (1)	NOVONORDISK DK (1)
Pontoppidan K (1)	NOVOZYMES DK (1)
Pettersson D (1)	NOVOZYMES DK (1)
Tremblay J (1)	NRC CNRC CA (1)
Piano A (1)	NRC CNRC CA (1)
Wu J-y (3)	NTU TW (1)
Wu C (1)	NTU TW (1)
Tseng Y-t (1)	NTU TW (1)
Liu W-h (1)	NTU TW (1)
Lin J-t (1)	NTU TW (1)
Chiang M-l (1)	NTU TW (1)
Cheng K-c (1)	NTU TW (1)
Chen M-j (1)	NTU TW (1)
Chen H-c (1)	NTU TW (1)
Sojoudi S (1)	OCCUPATIONAL SHAHID SADOUGHI UNIV YAZD IRAN (1)
Salehi F (1)	OCCUPATIONAL SHAHID SADOUGHI UNIV YAZD IRAN (1)
Ebrahimi A (1)	OCCUPATIONAL SHAHID SADOUGHI UNIV YAZD IRAN (1)
Trower Tm (2)	PFR US (1)
Simmons L (1)	PFR US (1)
Phipps Je (1)	PFR US (1)
Mcghie Tk (1)	PFR US (1)
Hopkins W (1)	PFR US (1)
Herath Td (1)	PFR US (1)
Hedderley Di (2)	PFR US (1)
Blatchford Pa (2)	PFR US (1)
Sutton Kh (2)	PFR US (2)
Parkar Sg (4)	PFR US (2)
Ingram Jr (2)	PFR US (2)
Torres E (1)	PHARMACOGNOSY FAC UNIV CONCEPCION CONCEPCION CHILE (1)
Carvajal Ri (1)	PHARMACOGNOSY FAC UNIV CONCEPCION CONCEPCION CHILE (1)
Avello M (1)	PHARMACOGNOSY FAC UNIV CONCEPCION CONCEPCION CHILE (1)
Pastene E (2)	PHARMACOGNOSY FAC UNIV CONCEPCION CONCEPCION CHILE (2)
Zhao Md (1)	PLANT SCIENCES US (1)
Liang S (1)	PLANT SCIENCES US (1)
Li Gy (1)	PLANT SCIENCES US (1)
Choi Sh (1)	PLANT SCIENCES US (1)
Tallarico-adorno Ma (1)	PROCESSES LPB SAO CARLOS EESC USP SAO CARLOS SAO PAULO BRAZIL (1)
Sarama Rj (1)	PROCTER AND GAMBLE US (1)
Garrett Da (1)	PROCTER AND GAMBLE US (1)
Failla Ml (1)	PROCTER AND GAMBLE US (1)
Duysburgh C (10)	PRODIGEST (10)
Possemiers S (35)	PRODIGEST (15)
Voorspoels S (2)	PRODIGEST (2)
Vigsnaes Lk (4)	PRODIGEST (2)
Sinnott R (2)	PRODIGEST (2)
Moens F (3)	PRODIGEST (2)
Mcconnell B (2)	PRODIGEST (2)
Maignien L (2)	PRODIGEST (2)
Luta G (2)	PRODIGEST (2)
Kamil A (2)	PRODIGEST (2)
Jacobs G (2)	PRODIGEST (2)
Hesta M (2)	PRODIGEST (2)
Fleige L (2)	PRODIGEST (2)

Verstrepen L (3)	PRODIGEST (3)
Krishnan K (3)	PRODIGEST (3)
Calatayud M (7)	PRODIGEST (3)
Van den abbeele P (45)	PRODIGEST (31)
Marzorati M (44)	PRODIGEST (33)
Pitart J (4)	PRODIGEST (4)
Verhelst A (5)	PRODIGEST (5)
Pinheiro I (5)	PRODIGEST (5)
Ghyselinck J (6)	PRODIGEST (6)
Vossen E (1)	PRODUCT QUALITY PRODUCTION GHENT UNIV MELLE BELGIUM (1)
Van hecke T (1)	PRODUCT QUALITY PRODUCTION GHENT UNIV MELLE BELGIUM (1)
De smet S (1)	PRODUCT QUALITY PRODUCTION GHENT UNIV MELLE BELGIUM (1)
Rondia P (2)	PRODUCTION SECTORS SUSTAINABILITY WALLOON RES CTR RUE LIROUX GEMBLOUX B BELGIUM (2)
Dufourny S (2)	PRODUCTION SECTORS SUSTAINABILITY WALLOON RES CTR RUE LIROUX GEMBLOUX B BELGIUM (2)
López-barrera Dm (1)	PROGRAMA POSGRADO DEL CTR REPUBLICA PROPAC RES STUDIES FOOD UNIV AUTONOMA QUERETARO SANTIAGO QUERETARO MEXICO (1)
Hernández-arriaga Am (1)	PROGRAMA POSGRADO DEL CTR REPUBLICA PROPAC RES STUDIES FOOD UNIV AUTONOMA QUERETARO SANTIAGO QUERETARO MEXICO (1)
de cosío-barrón Acg (1)	PROGRAMA POSGRADO DEL CTR REPUBLICA PROPAC RES STUDIES FOOD UNIV AUTONOMA QUERETARO SANTIAGO QUERETARO MEXICO (1)
Vázquez-sánchez K (2)	PROGRAMA POSGRADO DEL CTR REPUBLICA PROPAC RES STUDIES FOOD UNIV AUTONOMA QUERETARO SANTIAGO QUERETARO MEXICO (2)
Loarca-pina G (4)	PROGRAMA POSGRADO DEL CTR REPUBLICA PROPAC RES STUDIES FOOD UNIV AUTONOMA QUERETARO SANTIAGO QUERETARO MEXICO (2)
Campos-vega R (6)	PROGRAMA POSGRADO DEL CTR REPUBLICA PROPAC RES STUDIES FOOD UNIV AUTONOMA QUERETARO SANTIAGO QUERETARO MEXICO (4)
Regalado-gonzález C (1)	PROGRAMA POSGRADO EN ALIMENTOS DEL CTR REPUBLICA PROPAC FACULTAD QUIMICA UNIV AUTONOMA QUERETARO CTR UNIVERSITARIO CERRO LAS CAMPANAS S N QUERETARO QRO MEXICO (1)
Martín del campo St (1)	PROGRAMA POSGRADO EN ALIMENTOS DEL CTR REPUBLICA PROPAC FACULTAD QUIMICA UNIV AUTONOMA QUERETARO CTR UNIVERSITARIO CERRO LAS CAMPANAS S N QUERETARO QRO MEXICO (1)
García-almendarez B (1)	PROGRAMA POSGRADO EN ALIMENTOS DEL CTR REPUBLICA PROPAC FACULTAD QUIMICA UNIV AUTONOMA QUERETARO CTR UNIVERSITARIO CERRO LAS CAMPANAS S N QUERETARO QRO MEXICO (1)
Castaño-tostado E (1)	PROGRAMA POSGRADO EN ALIMENTOS DEL CTR REPUBLICA PROPAC FACULTAD QUIMICA UNIV AUTONOMA QUERETARO CTR UNIVERSITARIO CERRO LAS CAMPANAS S N QUERETARO QRO MEXICO (1)
Cardador A (1)	PROGRAMA POSGRADO EN ALIMENTOS DEL CTR REPUBLICA PROPAC FACULTAD QUIMICA UNIV AUTONOMA QUERETARO CTR UNIVERSITARIO CERRO LAS CAMPANAS S N QUERETARO QRO MEXICO (1)
Arvízu Sm (1)	PROGRAMA POSGRADO EN ALIMENTOS DEL CTR REPUBLICA PROPAC FACULTAD QUIMICA UNIV AUTONOMA QUERETARO CTR UNIVERSITARIO CERRO LAS CAMPANAS S N QUERETARO QRO MEXICO (1)
Amaya-llano Sl (1)	PROGRAMA POSGRADO EN ALIMENTOS DEL CTR REPUBLICA PROPAC FACULTAD QUIMICA UNIV AUTONOMA QUERETARO CTR UNIVERSITARIO CERRO LAS CAMPANAS S N QUERETARO QRO MEXICO (1)
Abadía-garcía L (1)	PROGRAMA POSGRADO EN ALIMENTOS DEL CTR REPUBLICA PROPAC FACULTAD QUIMICA UNIV AUTONOMA QUERETARO CTR UNIVERSITARIO CERRO LAS CAMPANAS S N QUERETARO QRO MEXICO (1)
Bisharat L (1)	QUADRAM INST BIOSCIENCE NORWICH UNITED KINGDOM (1)
Shade C (1)	QUICKSILVER SCIENTIFIC DENVER CO UNITED STATES (1)
Gantner N (1)	QUICKSILVER SCIENTIFIC DENVER CO UNITED STATES (1)
Chan Hm (1)	QUICKSILVER SCIENTIFIC DENVER CO UNITED STATES (1)
Yoo M-s (1)	R BD CTR HY CO LTD GIHEUNGDANJI RO 24BEON GIL GIHEUNG GU YONGIN SI SOUTH KOREA (1)
Shim J-j (1)	R BD CTR HY CO LTD GIHEUNGDANJI RO 24BEON GIL GIHEUNG GU YONGIN SI SOUTH KOREA (1)
Lee J-l (1)	R BD CTR HY CO LTD GIHEUNGDANJI RO 24BEON GIL GIHEUNG GU YONGIN SI SOUTH KOREA (1)
Lee J-h (1)	R BD CTR HY CO LTD GIHEUNGDANJI RO 24BEON GIL GIHEUNG GU YONGIN SI SOUTH KOREA (1)

Kim J-y (1)	R BD CTR HY CO LTD GIHEUNGDANJI RO 24BEON GIL GIHEUNG GU YONGIN SI SOUTH KOREA (1)
Heo K (1)	R BD CTR HY CO LTD GIHEUNGDANJI RO 24BEON GIL GIHEUNG GU YONGIN SI SOUTH KOREA (1)
Choi E-j (1)	R BD CTR HY CO LTD GIHEUNGDANJI RO 24BEON GIL GIHEUNG GU YONGIN SI SOUTH KOREA (1)
Nelson Gm (1)	RES AGENCY RES TRIANGLE PARK NC UNITED STATES (1)
George S (1)	RES AGENCY RES TRIANGLE PARK NC UNITED STATES (1)
Creason J (1)	RES AGENCY RES TRIANGLE PARK NC UNITED STATES (1)
Chadwick R (1)	RES AGENCY RES TRIANGLE PARK NC UNITED STATES (1)
Allison J (1)	RES AGENCY RES TRIANGLE PARK NC UNITED STATES (1)
Cai X (11)	RES CTR ECO CHINESE ACADEMY BEIJING CHINA (11)
Du H (13)	RES CTR ECO CHINESE ACADEMY BEIJING CHINA (12)
Du laing G (6)	RES CTR ECO CHINESE ACADEMY BEIJING CHINA (2)
Alava P (5)	RES CTR ECO CHINESE ACADEMY BEIJING CHINA (2)
Sun G-x (4)	RES CTR ECO CHINESE ACADEMY BEIJING CHINA (4)
Srivoramas T (1)	RES CTR EMERGING KHON KAEN UNIV KHON KAEN THAILAND (1)
Rattanachaikunsopon P (1)	RES CTR EMERGING KHON KAEN UNIV KHON KAEN THAILAND (1)
Panya M (1)	RES CTR EMERGING KHON KAEN UNIV KHON KAEN THAILAND (1)
Lulitanond V (1)	RES CTR EMERGING KHON KAEN UNIV KHON KAEN THAILAND (1)
Chaiwong T (1)	RES CTR EMERGING KHON KAEN UNIV KHON KAEN THAILAND (1)
Mokhtari M (2)	RES CTR SHAHID SADOUGHI UNIV YAZD IRAN (2)
Chang D (2)	RES DEVELOPMENT HINOMAN LTD RISHON LEZION ISRAEL (2)
Mcfarlan C (1)	RES DUNN CTR CAMBRIDGE UNITED KINGDOM (1)
Allison C (1)	RES DUNN CTR CAMBRIDGE UNITED KINGDOM (1)
Cummings Jh (3)	RES DUNN CTR CAMBRIDGE UNITED KINGDOM (2)
Sánchez Oj (1)	RES GROUP FOOD AGRO INDUSTRY UNIV CALDAS CALLE MANIZALES COLOMBIA (1)
Pino-hernández E (1)	RES GROUP FOOD AGRO INDUSTRY UNIV CALDAS CALLE MANIZALES COLOMBIA (1)
Nobre C (1)	RES GROUP FOOD AGRO INDUSTRY UNIV CALDAS CALLE MANIZALES COLOMBIA (1)
Matallana Lg (1)	RES GROUP FOOD AGRO INDUSTRY UNIV CALDAS CALLE MANIZALES COLOMBIA (1)
Gómez Ja (1)	RES GROUP FOOD AGRO INDUSTRY UNIV CALDAS CALLE MANIZALES COLOMBIA (1)
Abrunhosa L (1)	RES GROUP FOOD AGRO INDUSTRY UNIV CALDAS CALLE MANIZALES COLOMBIA (1)
Jo K (3)	RES GROUP FOOD PROCESSING KOREA FOOD RES INST WANJU SOUTH KOREA (2)
Rivière A (3)	RES GROUP INDUSTRIAL FOOD BIOENGINEERING VRIJE UNIVERSITEIT BRUSSEL PLEINLAAN BRUSSELS BELGIUM (2)
De vuyst L (3)	RES GROUP INDUSTRIAL FOOD BIOENGINEERING VRIJE UNIVERSITEIT BRUSSEL PLEINLAAN BRUSSELS BELGIUM (2)
Worametrachanon S (2)	RES INST CHIANG MAI UNIV CHIANG MAI THAILAND (2)
Apichartsrangkoon A (6)	RES INST CHIANG MAI UNIV CHIANG MAI THAILAND (6)
Jain Sk (1)	RES PROJECTS DR HARI SINGH GOUR VISHWAVIDYALAYA SAGAR JAPAN (1)
Dengre R (1)	RES PROJECTS DR HARI SINGH GOUR VISHWAVIDYALAYA SAGAR JAPAN (1)
Chourasia Mk (1)	RES PROJECTS DR HARI SINGH GOUR VISHWAVIDYALAYA SAGAR JAPAN (1)
Van der hoeven Js (1)	RES TRIKON UNIV NIJMEGEN P O BOX HB NIJMEGEN NETHERLANDS (1)
Camp Pjm (1)	RES TRIKON UNIV NIJMEGEN P O BOX HB NIJMEGEN NETHERLANDS (1)
Joseph Gs (1)	RESOURCE DEVELOPMENT CENTRAL FOOD TECHNOLOGICAL RES INST SCIENTIFIC INDUSTRIAL RES CSIR MYSORE INDIA (1)
Dolly P (1)	RESOURCE DEVELOPMENT CENTRAL FOOD TECHNOLOGICAL RES INST SCIENTIFIC INDUSTRIAL RES CSIR MYSORE INDIA (1)
Anishaparvin A (1)	RESOURCE DEVELOPMENT CENTRAL FOOD TECHNOLOGICAL RES INST SCIENTIFIC INDUSTRIAL RES CSIR MYSORE INDIA (1)
Anandharamakrishnan C (1)	RESOURCE DEVELOPMENT CENTRAL FOOD TECHNOLOGICAL RES INST SCIENTIFIC INDUSTRIAL RES CSIR MYSORE INDIA (1)
Young W (3)	RIDDET INST PALMERSTON NORTH NEW ZEALAND (3)
Yoshida K (2)	RIKEN JP (1)
Yasuda K (1)	RIKEN JP (1)
Takagi R (1)	RIKEN JP (1)
Suzuki K (1)	RIKEN JP (1)
Osawa R (1)	RIKEN JP (1)
Nishidono Y (1)	RIKEN JP (1)

Nakashima A (1)	RIKEN JP (1)
Kadowaki Y (1)	RIKEN JP (1)
Fukuda I (1)	RIKEN JP (1)
Aotsuka Y (1)	RIKEN JP (1)
Tanaka K (2)	RIKEN JP (2)
Sasaki K (7)	RIKEN JP (6)
Kondo A (6)	RIKEN JP (6)
Pillai N (2)	RMIT UNIV MELBOURNE AUSTRALIA (2)
Kalantar-zadeh K (2)	RMIT UNIV MELBOURNE AUSTRALIA (2)
Wils D (2)	ROQUETTE LESTREM FRANCE (2)
Guérin-deremaux L (2)	ROQUETTE LESTREM FRANCE (2)
Sahota Ss (1)	ROY HOLLOWAY COLL EGHAM SURREY TW20 0EX UNITED KINGDOM (1)
Menzies Is (1)	ROY HOLLOWAY COLL EGHAM SURREY TW20 0EX UNITED KINGDOM (1)
Bramley Pm (1)	ROY HOLLOWAY COLL EGHAM SURREY TW20 0EX UNITED KINGDOM (1)
Weiseth B (1)	ROYAL ROADS UNIV VICTORIA BC CANADA (1)
Packull-mccormick Sr (1)	ROYAL ROADS UNIV VICTORIA BC CANADA (1)
Dodd M (1)	ROYAL ROADS UNIV VICTORIA BC CANADA (1)
Vlckova K (1)	SCHNEIDER ELECTRIC FR (1)
van sinderen D (1)	SCHNEIDER ELECTRIC FR (1)
Turroni F (1)	SCHNEIDER ELECTRIC FR (1)
Tran Ttt (1)	SCHNEIDER ELECTRIC FR (1)
Thalacker-mercer A (1)	SCHNEIDER ELECTRIC FR (1)
Strain Cr (1)	SCHNEIDER ELECTRIC FR (1)
Stockdale Sr (1)	SCHNEIDER ELECTRIC FR (1)
Stephens N (1)	SCHNEIDER ELECTRIC FR (1)
Shkoporov An (1)	SCHNEIDER ELECTRIC FR (1)
Scholz D (1)	SCHNEIDER ELECTRIC FR (1)
Sahin Aw (1)	SCHNEIDER ELECTRIC FR (1)
Rota C (1)	SCHNEIDER ELECTRIC FR (1)
Quilter K (1)	SCHNEIDER ELECTRIC FR (1)
Pellanda P (1)	SCHNEIDER ELECTRIC FR (1)
Patangia D (1)	SCHNEIDER ELECTRIC FR (1)
Ossiprandi Mc (1)	SCHNEIDER ELECTRIC FR (1)
O'toole Pw (1)	SCHNEIDER ELECTRIC FR (1)
O'sullivan O (1)	SCHNEIDER ELECTRIC FR (1)
O'riordan P (1)	SCHNEIDER ELECTRIC FR (1)
O'gorman Dm (1)	SCHNEIDER ELECTRIC FR (1)
O'brien Nm (1)	SCHNEIDER ELECTRIC FR (1)
O'toole Pw (2)	SCHNEIDER ELECTRIC FR (1)
O'sullivan Ó (1)	SCHNEIDER ELECTRIC FR (1)
O'connor Pm (1)	SCHNEIDER ELECTRIC FR (1)
O'callaghan Tf (1)	SCHNEIDER ELECTRIC FR (1)
Neto Mc (1)	SCHNEIDER ELECTRIC FR (1)
Murphy M (1)	SCHNEIDER ELECTRIC FR (1)
Milani C (1)	SCHNEIDER ELECTRIC FR (1)
Miao S (1)	SCHNEIDER ELECTRIC FR (1)
Mcquaid A (1)	SCHNEIDER ELECTRIC FR (1)
Mangifesta M (1)	SCHNEIDER ELECTRIC FR (1)
Mancino W (1)	SCHNEIDER ELECTRIC FR (1)
Lynch Km (1)	SCHNEIDER ELECTRIC FR (1)
Lugli Ga (1)	SCHNEIDER ELECTRIC FR (1)
Koc F (1)	SCHNEIDER ELECTRIC FR (1)
Khokhlova Ev (1)	SCHNEIDER ELECTRIC FR (1)
Joyce Sa (1)	SCHNEIDER ELECTRIC FR (1)
Jones B (1)	SCHNEIDER ELECTRIC FR (1)
Johnson C (1)	SCHNEIDER ELECTRIC FR (1)
James K (1)	SCHNEIDER ELECTRIC FR (1)
Iori A (1)	SCHNEIDER ELECTRIC FR (1)
Hyland Np (1)	SCHNEIDER ELECTRIC FR (1)
Howell A (1)	SCHNEIDER ELECTRIC FR (1)
Guerin E (1)	SCHNEIDER ELECTRIC FR (1)
Gough R (1)	SCHNEIDER ELECTRIC FR (1)
Gómez-sala B (1)	SCHNEIDER ELECTRIC FR (1)
Gil-martinez J (1)	SCHNEIDER ELECTRIC FR (1)
Ghosh Ts (1)	SCHNEIDER ELECTRIC FR (1)

Gheller Me (1)	SCHNEIDER ELECTRIC FR (1)
Gargano G (1)	SCHNEIDER ELECTRIC FR (1)
Flynn C (1)	SCHNEIDER ELECTRIC FR (1)
Felice Vd (1)	SCHNEIDER ELECTRIC FR (1)
Duranti S (1)	SCHNEIDER ELECTRIC FR (1)
Dunngalvin G (1)	SCHNEIDER ELECTRIC FR (1)
Draper La (1)	SCHNEIDER ELECTRIC FR (1)
Dinan Tg (1)	SCHNEIDER ELECTRIC FR (1)
Di pierro F (1)	SCHNEIDER ELECTRIC FR (1)
De chavez Pj (1)	SCHNEIDER ELECTRIC FR (1)
Daly Km (1)	SCHNEIDER ELECTRIC FR (1)
Comas Jc (1)	SCHNEIDER ELECTRIC FR (1)
Clooney Ag (1)	SCHNEIDER ELECTRIC FR (1)
Chonchúir Fn (1)	SCHNEIDER ELECTRIC FR (1)
Blum Je (1)	SCHNEIDER ELECTRIC FR (1)
Bernasconi S (1)	SCHNEIDER ELECTRIC FR (1)
Barton W (1)	SCHNEIDER ELECTRIC FR (1)
Arendt Ek (1)	SCHNEIDER ELECTRIC FR (1)
Alessandri G (1)	SCHNEIDER ELECTRIC FR (1)
Stanton C (2)	SCHNEIDER ELECTRIC FR (2)
Shanahan F (3)	SCHNEIDER ELECTRIC FR (2)
O'donnell Mm (2)	SCHNEIDER ELECTRIC FR (2)
Ntemiri A (2)	SCHNEIDER ELECTRIC FR (2)
Rea Mc (5)	SCHNEIDER ELECTRIC FR (3)
Ross Rp (5)	SCHNEIDER ELECTRIC FR (5)
Tirwa Rk (1)	SIKKIM UNIV 6TH MILE SAMDUR TADONG SIKKIM INDIA (1)
Tamang B (1)	SIKKIM UNIV 6TH MILE SAMDUR TADONG SIKKIM INDIA (1)
Chaurasia Lk (1)	SIKKIM UNIV 6TH MILE SAMDUR TADONG SIKKIM INDIA (1)
Yao T (1)	SINOPEC CN (1)
Wang W (4)	SINOPEC CN (1)
Sun S (1)	SINOPEC CN (1)
Jiang X (1)	SINOPEC CN (1)
Fu P (1)	SINOPEC CN (1)
Fang L (1)	SINOPEC CN (1)
Dong H (1)	SINOPEC CN (1)
Yacyshyn Mb (1)	SKF SE (1)
Yacyshyn B (1)	SKF SE (1)
Wu Q (5)	SKF SE (1)
Wu G (2)	SKF SE (1)
Weier Sa (1)	SKF SE (1)
Van haute Mj (1)	SKF SE (1)
Tu J (1)	SKF SE (1)
Tian B (2)	SKF SE (1)
Spinler Jk (1)	SKF SE (1)
Smith C (2)	SKF SE (1)
Rose D (1)	SKF SE (1)
Rettedal E (1)	SKF SE (1)
Quintero Dfg (1)	SKF SE (1)
Payne P (1)	SKF SE (1)
Niyirora C (1)	SKF SE (1)
Nicholson M (1)	SKF SE (1)
Mcneill Mr (1)	SKF SE (1)
Lux R (1)	SKF SE (1)
Li A (1)	SKF SE (1)
Kok Cr (1)	SKF SE (1)
Keshavarzian A (1)	SKF SE (1)
Hyser Jm (1)	SKF SE (1)
Hutkins R (1)	SKF SE (1)
Gathercole J (1)	SKF SE (1)
Garey Kw (1)	SKF SE (1)
Estes Mk (1)	SKF SE (1)
Engevik Ka (1)	SKF SE (1)
Endres Bt (1)	SKF SE (1)
Danhof Ha (1)	SKF SE (1)
Crawford Se (1)	SKF SE (1)

Cheng P (2)	SKF SE (1)
Chang-graham Al (1)	SKF SE (1)
Brennan Ma (1)	SKF SE (1)
Brennan Cs (1)	SKF SE (1)
Brahma S (1)	SKF SE (1)
Bhat Hf (1)	SKF SE (1)
Bell N (1)	SKF SE (1)
Bassères E (1)	SKF SE (1)
Bai W (1)	SKF SE (1)
Auchtung J (1)	SKF SE (1)
Arojju Sk (1)	SKF SE (1)
Mason Sl (2)	SKF SE (2)
Luna Ra (2)	SKF SE (2)
Engevik Ma (2)	SKF SE (2)
Engevik Ac (2)	SKF SE (2)
Bhat Zf (2)	SKF SE (2)
Maldonado-gómez Mx (3)	SKF SE (3)
Hutkins Rw (3)	SKF SE (3)
Zhu K (3)	SPICE BEVERAGE RES INST CHINESE ACADEMY TROPICAL WANNING HAINAN CHINA (2)
Hernández-álvarez Aj (2)	ST HYACINTHE RES DEVELOPMENT CTR AGRI FOOD CANADA CASAVANT WEST BOULEVARD SAINT HYACINTHE QC J2S CANADA (2)
Roorda Ak (1)	ST MARY S CTR SAN FRANCISCO UNITED STATES (1)
Rider Dl (1)	ST MARY S CTR SAN FRANCISCO UNITED STATES (1)
Conroy Bf (1)	ST MARY S CTR SAN FRANCISCO UNITED STATES (1)
Alfred rider J (1)	ST MARY S CTR SAN FRANCISCO UNITED STATES (1)
Xiang C (2)	STATE KEY BREEDING BASE ZHEJIANG SUSTAINABLE PEST INST PLANT ZHEJIANG ACADEMY HANGZHOU CHINA (2)
Koehnlein Ea (2)	STATE UNIV MARINGAPARANA BRAZIL (2)
Bracht A (2)	STATE UNIV MARINGAPARANA BRAZIL (2)
Peralta Rm (3)	STATE UNIV MARINGAPARANA BRAZIL (3)
Correa Vg (3)	STATE UNIV MARINGAPARANA BRAZIL (3)
Savard P (1)	STELA DAIRY RES CTR FUNCTIONAL FOODS INST UNIV LAVAL QUEBEC QC CANADA (1)
Fernandez B (3)	STELA DAIRY RES CTR FUNCTIONAL FOODS INST UNIV LAVAL QUEBEC QC CANADA (2)
van staden Ad (1)	STELLENBOSCH UNIV STELLENBOSCH SOUTH AFRICA (1)
Dreyer L (1)	STELLENBOSCH UNIV STELLENBOSCH SOUTH AFRICA (1)
Dicks Lmt (1)	STELLENBOSCH UNIV STELLENBOSCH SOUTH AFRICA (1)
Deane Sm (1)	STELLENBOSCH UNIV STELLENBOSCH SOUTH AFRICA (1)
Basson Nj (1)	STELLENBOSCH UNIV STELLENBOSCH SOUTH AFRICA (1)
Koistinen Vm (1)	STENO CTR NIELS STEENSENS VEJ GENTOFTE DENMARK (1)
Katina K (1)	STENO CTR NIELS STEENSENS VEJ GENTOFTE DENMARK (1)
Hanhineva K (1)	STENO CTR NIELS STEENSENS VEJ GENTOFTE DENMARK (1)
Watanabe T (1)	SUMITOMO GROUP JP (1)
Sakurai K (1)	SUMITOMO GROUP JP (1)
Nakajima T (1)	SUMITOMO GROUP JP (1)
Miyata K (1)	SUMITOMO GROUP JP (1)
Matsunaga Yt (1)	SUMITOMO GROUP JP (1)
Yamamori A (2)	SUMITOMO GROUP JP (2)
Sariçay Y (1)	TEAGASC FOOD RES CTR FERMOY CO CORK IRELAND (1)
Kelly Pm (1)	TEAGASC FOOD RES CTR FERMOY CO CORK IRELAND (1)
Giblin L (1)	TEAGASC FOOD RES CTR FERMOY CO CORK IRELAND (1)
Corrochano Ar (1)	TEAGASC FOOD RES CTR FERMOY CO CORK IRELAND (1)
Buckin V (1)	TEAGASC FOOD RES CTR FERMOY CO CORK IRELAND (1)
Arranz E (1)	TEAGASC FOOD RES CTR FERMOY CO CORK IRELAND (1)
Hill C (4)	TEAGASC FOOD RES CTR FERMOY CO CORK IRELAND (3)
Woodburn Ma (2)	TECHLAB INC PRATT DRIVE BLACKSBURG VA UNITED STATES (2)
Carman Rj (4)	TECHLAB INC PRATT DRIVE BLACKSBURG VA UNITED STATES (2)
Saroj Db (1)	TECHNOLOGIES LTD THANE MAHARASHTRA INDIA (1)
Maity C (1)	TECHNOLOGIES LTD THANE MAHARASHTRA INDIA (1)
Kulkarni J (1)	TECHNOLOGIES LTD THANE MAHARASHTRA INDIA (1)
Gupta Ak (1)	TECHNOLOGIES LTD THANE MAHARASHTRA INDIA (1)
Dixit Y (1)	TECHNOLOGIES LTD THANE MAHARASHTRA INDIA (1)
Biyani A (1)	TECHNOLOGIES LTD THANE MAHARASHTRA INDIA (1)

Bagkar P (1)	TECHNOLOGIES LTD THANE MAHARASHTRA INDIA (1)
Peirotén Á (1)	TECNOLOGIA ALIMENTOS INST NACIONAL INVESTIGACION TECNOLOGIA AGRARIA ALIMENTARIA INIA CARRETERA CORUNA KM MADRID SPAIN (1)
Martínez-fernández Ja (1)	TECNOLOGIA ALIMENTOS INST NACIONAL INVESTIGACION TECNOLOGIA AGRARIA ALIMENTARIA INIA CARRETERA CORUNA KM MADRID SPAIN (1)
Landete Jm (1)	TECNOLOGIA ALIMENTOS INST NACIONAL INVESTIGACION TECNOLOGIA AGRARIA ALIMENTARIA INIA CARRETERA CORUNA KM MADRID SPAIN (1)
Arqués Jl (1)	TECNOLOGIA ALIMENTOS INST NACIONAL INVESTIGACION TECNOLOGIA AGRARIA ALIMENTARIA INIA CARRETERA CORUNA KM MADRID SPAIN (1)
Senés-guerrero C (1)	TECNOLOGICO MONTERREY ESCUELA INGENIERIA CIENCIAS MONTERREY MEXICO (1)
Santacruz A (1)	TECNOLOGICO MONTERREY ESCUELA INGENIERIA CIENCIAS MONTERREY MEXICO (1)
Pacheco A (1)	TECNOLOGICO MONTERREY ESCUELA INGENIERIA CIENCIAS MONTERREY MEXICO (1)
Gutiérrez-uribe Ja (1)	TECNOLOGICO MONTERREY ESCUELA INGENIERIA CIENCIAS MONTERREY MEXICO (1)
Figueroa Lm (1)	TECNOLOGICO MONTERREY ESCUELA INGENIERIA CIENCIAS MONTERREY MEXICO (1)
Ramírez-jiménez Ak (2)	TECNOLOGICO MONTERREY ESCUELA INGENIERIA CIENCIAS MONTERREY MEXICO (2)
Ruíz-valdiviezo Vm (2)	TECNOLOGICO NACIONAL MEXICO TEPIC AV TECNOLOGICO TEPIC NAYARIT CP MEXICO (2)
Sáyago-ayerdi Sg (12)	TECNOLOGICO NACIONAL MEXICO TEPIC AV TECNOLOGICO TEPIC NAYARIT CP MEXICO (6)
Ruan W (2)	TEXAS CHILDREN S HOSP HOUSTON TX UNITED STATES (2)
Zoet Fd (1)	TNO NL (1)
Zeijdner E (1)	TNO NL (1)
Yamamoto N (1)	TNO NL (1)
Wragg J (1)	TNO NL (1)
Wood S (1)	TNO NL (1)
Westerbeek Hjm (1)	TNO NL (1)
Vuopio-varkila J (1)	TNO NL (1)
Verwei M (1)	TNO NL (1)
Van wijnen J (1)	TNO NL (1)
Van der woude Jcj (1)	TNO NL (1)
van den heuvel Eg (2)	TNO NL (1)
Van bilsen J (1)	TNO NL (1)
Van beek Jhgm (1)	TNO NL (1)
Van aken Ga (1)	TNO NL (1)
Vaes W (1)	TNO NL (1)
Troost F (1)	TNO NL (1)
Stolaki M (1)	TNO NL (1)
Smid Ej (1)	TNO NL (1)
Sips Ajam (2)	TNO NL (1)
Schreiber S (1)	TNO NL (1)
Schoterman Mhc (1)	TNO NL (1)
Schols H (1)	TNO NL (1)
Schilderink R (1)	TNO NL (1)
Sanders L (1)	TNO NL (1)
Rompelberg Cjm (2)	TNO NL (1)
Roeselers G (2)	TNO NL (1)
Reimer Ra (1)	TNO NL (1)
Ramiro-garcia J (1)	TNO NL (1)
Ramasamy Us (1)	TNO NL (1)
Putri Ak (1)	TNO NL (1)
Ott Sj (1)	TNO NL (1)
Oosterveld A (1)	TNO NL (1)
Oomen Ag (2)	TNO NL (1)
Nakamura Y (1)	TNO NL (1)
Murota I (1)	TNO NL (1)
Mueller M (1)	TNO NL (1)
Miettinen M (1)	TNO NL (1)
Meyer Pd (1)	TNO NL (1)
Lyon Mr (1)	TNO NL (1)

Lehtinen P (1)	TNO NL (1)
Lazarova D (1)	TNO NL (1)
Larsson M (1)	TNO NL (1)
Lahti L (1)	TNO NL (1)
Labij E (1)	TNO NL (1)
Kuipers Ej (1)	TNO NL (1)
Knecht H (1)	TNO NL (1)
Klinck B (1)	TNO NL (1)
Jonkers Dmae (3)	TNO NL (1)
Janssen Am (1)	TNO NL (1)
Hoffman A (1)	TNO NL (1)
Heringa J (1)	TNO NL (1)
Hellmig S (1)	TNO NL (1)
Heinsen F-a (1)	TNO NL (1)
Heilig Hghj (1)	TNO NL (1)
Hatanaka M (1)	TNO NL (1)
Harrysson H (1)	TNO NL (1)
Hack A (1)	TNO NL (1)
Gutiérrez Op (1)	TNO NL (1)
Gruppen H (1)	TNO NL (1)
Gahler Rj (1)	TNO NL (1)
Evans A (1)	TNO NL (1)
De zwart Ll (1)	TNO NL (1)
de melo franco Bdg (1)	TNO NL (1)
De jong A (2)	TNO NL (1)
Cruz rubio Jm (1)	TNO NL (1)
Coulier L (1)	TNO NL (1)
Cornelis C (1)	TNO NL (1)
Cave M (1)	TNO NL (1)
Cardarelli Hr (1)	TNO NL (1)
Borst W (1)	TNO NL (1)
Bordonaro M (1)	TNO NL (1)
Bomhof E (1)	TNO NL (1)
Binsl Tw (1)	TNO NL (1)
Aynaou A-e (1)	TNO NL (1)
Alander M (2)	TNO NL (1)
Maathuis Ajh (14)	TNO NL (13)
Van nuenen Mhmc (2)	TNO NL (2)
Selinheimo E (2)	TNO NL (2)
Schols Ha (3)	TNO NL (2)
Rajilic-stojanovic M (2)	TNO NL (2)
Marteau P (2)	TNO NL (2)
Huis in't veld Jhj (2)	TNO NL (2)
Haenen Grmm (2)	TNO NL (2)
Eck A (2)	TNO NL (2)
De martinis Ecp (3)	TNO NL (2)
Anson Nm (2)	TNO NL (2)
Albrecht S (2)	TNO NL (2)
Venema K (50)	TNO NL (28)
De waard P (3)	TNO NL (3)
Aguirre M (4)	TNO NL (3)
De graaf Aa (4)	TNO NL (4)
Minekus M (9)	TNO NL (7)
Koenen Me (7)	TNO NL (7)
Havenaar R (8)	TNO NL (7)
Yáñez R (1)	TOTAL FR (1)
Vilas-boas Aa (1)	TOTAL FR (1)
Vilas-boas A (1)	TOTAL FR (1)
Vicente Aa (1)	TOTAL FR (1)
Vasconcelos M (1)	TOTAL FR (1)
Tonon Rv (1)	TOTAL FR (1)
Teixeira P (2)	TOTAL FR (1)
Teixeira Ja (2)	TOTAL FR (1)
Teixeira F (1)	TOTAL FR (1)
Tavaria Fk (1)	TOTAL FR (1)

Tavaria F (1)	TOTAL FR (1)
Soares J (1)	TOTAL FR (1)
Silva Sn (1)	TOTAL FR (1)
Rodríguez-alcalá Lm (1)	TOTAL FR (1)
Ribeiro Tb (1)	TOTAL FR (1)
Poveda Cg (1)	TOTAL FR (1)
Pintado Mm (1)	TOTAL FR (1)
Pimentel L (1)	TOTAL FR (1)
Pereira Jo (1)	TOTAL FR (1)
Pastrana Lm (1)	TOTAL FR (1)
Pastrana L (1)	TOTAL FR (1)
Nunes J (1)	TOTAL FR (1)
Monteiro Mjp (2)	TOTAL FR (1)
Monforte Ar (1)	TOTAL FR (1)
Magalhães R (1)	TOTAL FR (1)
Jauregi P (1)	TOTAL FR (1)
Gonçalves Ecbda (1)	TOTAL FR (1)
Gomes Am (1)	TOTAL FR (1)
Gomes A (1)	TOTAL FR (1)
Ferreira V (1)	TOTAL FR (1)
Ferreira Msl (1)	TOTAL FR (1)
Costa Jr (1)	TOTAL FR (1)
Costa E (1)	TOTAL FR (1)
Costa Cmdsf (1)	TOTAL FR (1)
Costa Cm (1)	TOTAL FR (1)
Coscueta Er (1)	TOTAL FR (1)
Campos Da (1)	TOTAL FR (1)
Cabral Lmc (1)	TOTAL FR (1)
Brandão Trs (2)	TOTAL FR (1)
Bonifácio - lopes T (1)	TOTAL FR (1)
Barber X (1)	TOTAL FR (1)
Andrade Rmsd (1)	TOTAL FR (1)
Amaro A (1)	TOTAL FR (1)
Alves Â (1)	TOTAL FR (1)
Veiga M (2)	TOTAL FR (2)
Madureira Ar (2)	TOTAL FR (2)
Gullón P (2)	TOTAL FR (2)
De carvalho Nm (2)	TOTAL FR (2)
Amorim M (2)	TOTAL FR (2)
Pintado M (3)	TOTAL FR (3)
Gullón B (3)	TOTAL FR (3)
Silva S (4)	TOTAL FR (4)
Pintado Me (4)	TOTAL FR (4)
Harms H (1)	UFZ HELMHOLTZ CTR RES LEIPZIG GERMANY (1)
Günther S (1)	UFZ HELMHOLTZ CTR RES LEIPZIG GERMANY (1)
Centler F (1)	UFZ HELMHOLTZ CTR RES LEIPZIG GERMANY (1)
Bonk F (1)	UFZ HELMHOLTZ CTR RES LEIPZIG GERMANY (1)
Raveschot C (1)	UMR TRANSFRONTALIERE BIOECOAGRO N°1158 UNIV LILLE INRAE UNIV LIEGE UPJV YNCREA UNIV DARTOIS UNIV DU LITTORAL COTE DOPALE ICV INST CHARLES VIOLLETTE LILLE FRANCE (1)
Frémont M (1)	UMR TRANSFRONTALIERE BIOECOAGRO N°1158 UNIV LILLE INRAE UNIV LIEGE UPJV YNCREA UNIV DARTOIS UNIV DU LITTORAL COTE DOPALE ICV INST CHARLES VIOLLETTE LILLE FRANCE (1)
Flahaut C (1)	UMR TRANSFRONTALIERE BIOECOAGRO N°1158 UNIV LILLE INRAE UNIV LIEGE UPJV YNCREA UNIV DARTOIS UNIV DU LITTORAL COTE DOPALE ICV INST CHARLES VIOLLETTE LILLE FRANCE (1)
Drider D (1)	UMR TRANSFRONTALIERE BIOECOAGRO N°1158 UNIV LILLE INRAE UNIV LIEGE UPJV YNCREA UNIV DARTOIS UNIV DU LITTORAL COTE DOPALE ICV INST CHARLES VIOLLETTE LILLE FRANCE (1)
Dhulster P (1)	UMR TRANSFRONTALIERE BIOECOAGRO N°1158 UNIV LILLE INRAE UNIV LIEGE UPJV YNCREA UNIV DARTOIS UNIV DU LITTORAL COTE DOPALE ICV INST CHARLES VIOLLETTE LILLE FRANCE (1)
Deracinois B (1)	UMR TRANSFRONTALIERE BIOECOAGRO N°1158 UNIV LILLE INRAE UNIV LIEGE UPJV YNCREA UNIV DARTOIS UNIV DU LITTORAL COTE DOPALE ICV INST CHARLES VIOLLETTE LILLE FRANCE (1)

Cudennec B (1)	UMR TRANSFRONTALIERE BIOECOAGRO N°1158 UNIV LILLE INRAE UNIV LIEGE UPJV YNCREA UNIV DARTOIS UNIV DU LITTORAL COTE DOPALE ICV INST CHARLES VIOLLETTE LILLE FRANCE (1)
Coutte F (1)	UMR TRANSFRONTALIERE BIOECOAGRO N°1158 UNIV LILLE INRAE UNIV LIEGE UPJV YNCREA UNIV DARTOIS UNIV DU LITTORAL COTE DOPALE ICV INST CHARLES VIOLLETTE LILLE FRANCE (1)
Bertrand E (1)	UMR TRANSFRONTALIERE BIOECOAGRO N°1158 UNIV LILLE INRAE UNIV LIEGE UPJV YNCREA UNIV DARTOIS UNIV DU LITTORAL COTE DOPALE ICV INST CHARLES VIOLLETTE LILLE FRANCE (1)
Vinarova L (1)	UNILEVER GB NL (1)
Vinarov Z (1)	UNILEVER GB NL (1)
Van duynhoven Jp (1)	UNILEVER GB NL (1)
Van duynhoven J (1)	UNILEVER GB NL (1)
Van dorsten Fa (1)	UNILEVER GB NL (1)
Van dorsten F (1)	UNILEVER GB NL (1)
Van der hooft Jjj (1)	UNILEVER GB NL (1)
Tcholakova S (1)	UNILEVER GB NL (1)
Stoyanov S (1)	UNILEVER GB NL (1)
Roger L (1)	UNILEVER GB NL (1)
Peters S (1)	UNILEVER GB NL (1)
Lips A (1)	UNILEVER GB NL (1)
Klinkenberg M (1)	UNILEVER GB NL (1)
Draijer R (1)	UNILEVER GB NL (1)
Denkov Nd (1)	UNILEVER GB NL (1)
De vos Rc (1)	UNILEVER GB NL (1)
De vos R (1)	UNILEVER GB NL (1)
Gross G (2)	UNILEVER GB NL (2)
Vaughan Ee (3)	UNILEVER GB NL (3)
Craig Dqm (2)	UNIV EAST ANGLIA NORWICH NR4 7TJ UNITED KINGDOM (2)
Barker Sa (2)	UNIV EAST ANGLIA NORWICH NR4 7TJ UNITED KINGDOM (2)
Smeds A (1)	UNIV EASTERN FINLAND KUOPIO FINLAND (1)
Faulds C (1)	UNIV EASTERN FINLAND KUOPIO FINLAND (1)
Poutanen K (9)	UNIV EASTERN FINLAND KUOPIO FINLAND (4)
Walvoort Mtc (1)	UNIV GRONINGEN GRONINGEN NETHERLANDS (1)
Krenning G (1)	UNIV GRONINGEN GRONINGEN NETHERLANDS (1)
Kong C (1)	UNIV GRONINGEN GRONINGEN NETHERLANDS (1)
Fledderus J (1)	UNIV GRONINGEN GRONINGEN NETHERLANDS (1)
De vos P (1)	UNIV GRONINGEN GRONINGEN NETHERLANDS (1)
De haan Bj (1)	UNIV GRONINGEN GRONINGEN NETHERLANDS (1)
Parreira Vr (1)	UNIV GUELPH GUELPH CANADA (1)
Yen S (4)	UNIV GUELPH GUELPH CANADA (2)
Cochrane K (2)	UNIV GUELPH GUELPH CANADA (2)
Oliphant K (5)	UNIV GUELPH GUELPH CANADA (3)
Sokolenko S (1)	UNIV GUELPH GUELPH N1G 2W1 CANADA (1)
Haratifar S (1)	UNIV GUELPH GUELPH N1G 2W1 CANADA (1)
Guri A (1)	UNIV GUELPH GUELPH N1G 2W1 CANADA (1)
Blondeel Ejm (1)	UNIV GUELPH GUELPH N1G 2W1 CANADA (1)
Aucoin Mg (1)	UNIV GUELPH GUELPH N1G 2W1 CANADA (1)
Khursigara Cm (2)	UNIV GUELPH GUELPH N1G 2W1 CANADA (2)
Heikamp-dejong I (2)	UNIV GUELPH GUELPH N1G 2W1 CANADA (2)
Fuentes S (3)	UNIV GUELPH GUELPH N1G 2W1 CANADA (2)
Schroeter K (5)	UNIV GUELPH GUELPH N1G 2W1 CANADA (3)
Mcdonald Jak (5)	UNIV GUELPH GUELPH N1G 2W1 CANADA (3)
Lapointe G (4)	UNIV GUELPH GUELPH N1G 2W1 CANADA (3)
Allen-vercoe E (10)	UNIV GUELPH GUELPH N1G 2W1 CANADA (5)
Paliyath G (1)	UNIV GUELPH N1G2W1 CANADA (1)
Correa-betanzo J (1)	UNIV GUELPH N1G2W1 CANADA (1)
Oksman-caldentey K-m (1)	UNIV HELSINKI HELSINKI FINLAND (1)
Bounsaythip C (1)	UNIV HELSINKI HELSINKI FINLAND (1)
Temme Ij (1)	UNIV HOSP MUNSTER INST MUNSTER GERMANY (1)
Middendorf-bauchart B (1)	UNIV HOSP MUNSTER INST MUNSTER GERMANY (1)
Mellmann A (1)	UNIV HOSP MUNSTER INST MUNSTER GERMANY (1)
Kehl A (1)	UNIV HOSP MUNSTER INST MUNSTER GERMANY (1)
Gati Ns (1)	UNIV HOSP MUNSTER INST MUNSTER GERMANY (1)
Dobrindt U (1)	UNIV HOSP MUNSTER INST MUNSTER GERMANY (1)

Vilpponen-salmela T (1)	UNIV KUOPIO INST P O BOX FIN KUOPIO FINLAND (1)
Mandic-mulec I (1)	UNIV LJUBLJANA BIOTECHNICAL FAC FOOD CHAIR VECNA POT LJUBLJANA SLOVENIA (1)
Kraigher B (1)	UNIV LJUBLJANA BIOTECHNICAL FAC FOOD CHAIR VECNA POT LJUBLJANA SLOVENIA (1)
Long C (1)	UNIV MAASTRICHT MAASTRICHT NETHERLANDS (1)
de vries S (1)	UNIV MAASTRICHT MAASTRICHT NETHERLANDS (1)
Verbruggen S (5)	UNIV MAASTRICHT MAASTRICHT NETHERLANDS (2)
Dijkema C (2)	UNIV MAASTRICHT MAASTRICHT NETHERLANDS (2)
Bast A (3)	UNIV MAASTRICHT MAASTRICHT NETHERLANDS (3)
Zhang Yy (1)	UNIV MELBOURNE PARKVILLE VIC AUSTRALIA (1)
Panozzo J (1)	UNIV MELBOURNE PARKVILLE VIC AUSTRALIA (1)
Hall Ms (1)	UNIV MELBOURNE PARKVILLE VIC AUSTRALIA (1)
Dasanayake K (1)	UNIV MELBOURNE PARKVILLE VIC AUSTRALIA (1)
Balasooriya H (1)	UNIV MELBOURNE PARKVILLE VIC AUSTRALIA (1)
Ajlouni S (5)	UNIV MELBOURNE PARKVILLE VIC AUSTRALIA (2)
Morton Jd (2)	UNIV OTAGO DUNEDIN NEW ZEALAND (2)
Bekhit Ae-da (2)	UNIV OTAGO DUNEDIN NEW ZEALAND (2)
Wessling St (1)	UNIV QUEENSLAND BRISBANE QLD AUSTRALIA (1)
Toth I (1)	UNIV QUEENSLAND BRISBANE QLD AUSTRALIA (1)
Ross Bp (1)	UNIV QUEENSLAND BRISBANE QLD AUSTRALIA (1)
Koda Y (1)	UNIV QUEENSLAND BRISBANE QLD AUSTRALIA (1)
Blanchfield Jt (1)	UNIV QUEENSLAND BRISBANE QLD AUSTRALIA (1)
Yeung J (1)	UNIV SASKATCHEWAN SASKATOON CANADA (1)
Varankovich N (1)	UNIV SASKATCHEWAN SASKATOON CANADA (1)
Nickerson Mt (1)	UNIV SASKATCHEWAN SASKATOON CANADA (1)
Martinez Mf (1)	UNIV SASKATCHEWAN SASKATOON CANADA (1)
Korber Dr (1)	UNIV SASKATCHEWAN SASKATOON CANADA (1)
Van de wiele Tr (5)	UNIV SASKATCHEWAN SASKATOON CANADA (3)
Peak D (4)	UNIV SASKATCHEWAN SASKATOON CANADA (4)
Laird Bd (6)	UNIV SASKATCHEWAN SASKATOON CANADA (6)
Siciliano Sd (8)	UNIV SASKATCHEWAN SASKATOON CANADA (8)
van den berg A (1)	UNIV TWENTE P O BOX ENSCHEDE AE NETHERLANDS (1)
Tibbe Mp (1)	UNIV TWENTE P O BOX ENSCHEDE AE NETHERLANDS (1)
Segerink Li (1)	UNIV TWENTE P O BOX ENSCHEDE AE NETHERLANDS (1)
Leferink Am (1)	UNIV TWENTE P O BOX ENSCHEDE AE NETHERLANDS (1)
Eijkel Jct (1)	UNIV TWENTE P O BOX ENSCHEDE AE NETHERLANDS (1)
Taupp M (1)	UNIVs AARHUS DK (1)
Sanei H (1)	UNIVs AARHUS DK (1)
Rezadehbashi M (1)	UNIVs AARHUS DK (1)
Mattes A (1)	UNIVs AARHUS DK (1)
Kot W (1)	UNIVs AARHUS DK (1)
Khoshnoodi M (1)	UNIVs AARHUS DK (1)
Hallam S (1)	UNIVs AARHUS DK (1)
de mello tieghi T (1)	UNIVs AARHUS DK (1)
Baldwin Sa (1)	UNIVs AARHUS DK (1)
Vélez D (2)	UNIVs ABERDEEN GB (1)
Quintela Jc (1)	UNIVs ABERDEEN GB (1)
Feldmann J (1)	UNIVs ABERDEEN GB (1)
Devesa V (1)	UNIVs ABERDEEN GB (1)
de la fuente E (1)	UNIVs ABERDEEN GB (1)
Bralatei E (1)	UNIVs ABERDEEN GB (1)
Walker Aw (3)	UNIVs ABERDEEN GB (2)
Yayota M (1)	UNIVs AICHI JP (1)
Seino S (1)	UNIVs AICHI JP (1)
Matsuda I (1)	UNIVs AICHI JP (1)
Hoshino T (1)	UNIVs AICHI JP (1)
Hoshino S (1)	UNIVs AICHI JP (1)
Funahashi T (1)	UNIVs AICHI JP (1)
Clauss M (1)	UNIVs AICHI JP (1)
Vieira Cr (1)	UNIVs ALFENAS BR (1)
Silva Rr (1)	UNIVs ALFENAS BR (1)
Nogueira Da (1)	UNIVs ALFENAS BR (1)
Duarte martino Hs (1)	UNIVs ALFENAS BR (1)
do carmo Mav (1)	UNIVs ALFENAS BR (1)

da silva Bp (1)	UNIVs ALFENAS BR (1)
Azevedo L (1)	UNIVs ALFENAS BR (1)
Réquilé M (1)	UNIVs AMIENS FR (1)
Léké A (1)	UNIVs AMIENS FR (1)
Joly C (1)	UNIVs AMIENS FR (1)
Gonzàlez alvarez Do (1)	UNIVs AMIENS FR (1)
Gay-quéheillard J (1)	UNIVs AMIENS FR (1)
Chardon K (1)	UNIVs AMIENS FR (1)
Khorsi-cauet H (3)	UNIVs AMIENS FR (2)
Delanaud S (3)	UNIVs AMIENS FR (2)
Bach V (3)	UNIVs AMIENS FR (2)
Yuan L (1)	UNIVs ANHUI CN (1)
Wu X-w (1)	UNIVs ANHUI CN (1)
Wei Z (2)	UNIVs ANHUI CN (1)
Su K (1)	UNIVs ANHUI CN (1)
Sheng G-p (1)	UNIVs ANHUI CN (1)
Shen N (1)	UNIVs ANHUI CN (1)
Shahidi F (1)	UNIVs ANHUI CN (1)
Mang L (1)	UNIVs ANHUI CN (1)
Ma Y (4)	UNIVs ANHUI CN (1)
Liu Q-h (1)	UNIVs ANHUI CN (1)
Liu P (1)	UNIVs ANHUI CN (1)
Li Z-h (1)	UNIVs ANHUI CN (1)
Hua R-m (1)	UNIVs ANHUI CN (1)
Fu Y-y (1)	UNIVs ANHUI CN (1)
Feng R-p (1)	UNIVs ANHUI CN (1)
Xiao J-j (2)	UNIVs ANHUI CN (2)
Wang H (5)	UNIVs ANHUI CN (2)
Shi Y-h (2)	UNIVs ANHUI CN (2)
Ren P (2)	UNIVs ANHUI CN (2)
Liu Y-y (2)	UNIVs ANHUI CN (2)
Liao M (2)	UNIVs ANHUI CN (2)
Cao H-q (2)	UNIVs ANHUI CN (2)
Westerhoff P (1)	UNIVs ARIZONA US (1)
Unc A (1)	UNIVs ARIZONA US (1)
Torres Ci (1)	UNIVs ARIZONA US (1)
Starkenburg Sr (1)	UNIVs ARIZONA US (1)
Smith Sr (1)	UNIVs ARIZONA US (1)
Smith Hl (1)	UNIVs ARIZONA US (1)
Smith H (1)	UNIVs ARIZONA US (1)
Shin D (1)	UNIVs ARIZONA US (1)
Seger M (1)	UNIVs ARIZONA US (1)
Rittmann Be (1)	UNIVs ARIZONA US (1)
Ramakrishna S (1)	UNIVs ARIZONA US (1)
Radtke Al (1)	UNIVs ARIZONA US (1)
Quayle Aj (1)	UNIVs ARIZONA US (1)
Park J (1)	UNIVs ARIZONA US (1)
Park H (2)	UNIVs ARIZONA US (1)
Nelson Kg (1)	UNIVs ARIZONA US (1)
Marcus Ak (1)	UNIVs ARIZONA US (1)
Lee J-c (1)	UNIVs ARIZONA US (1)
Lee J (3)	UNIVs ARIZONA US (1)
Le D (1)	UNIVs ARIZONA US (1)
Lammers Pj (1)	UNIVs ARIZONA US (1)
Kojouharov Hv (1)	UNIVs ARIZONA US (1)
Kim M-s (2)	UNIVs ARIZONA US (1)
Jones Da (1)	UNIVs ARIZONA US (1)
Jones D (1)	UNIVs ARIZONA US (1)
Ilhan Ze (1)	UNIVs ARIZONA US (1)
Holguin Fo (1)	UNIVs ARIZONA US (1)
Herbst-kralovetz Mm (1)	UNIVs ARIZONA US (1)
Emerson R (1)	UNIVs ARIZONA US (1)
Davis T (1)	UNIVs ARIZONA US (1)
Corbin Kd (1)	UNIVs ARIZONA US (1)
Chiu C-a (1)	UNIVs ARIZONA US (1)

Champagne C (1)	UNIVs ARIZONA US (1)
Carnero Ea (1)	UNIVs ARIZONA US (1)
Bock C (1)	UNIVs ARIZONA US (1)
Ballyk Mm (1)	UNIVs ARIZONA US (1)
Dirks B (3)	UNIVs ARIZONA US (3)
Krajmalnik-brown R (4)	UNIVs ARIZONA US (4)
Martin Em (1)	UNIVs ARKANSAS US (1)
Kumar Tks (1)	UNIVs ARKANSAS US (1)
Hettiarachchy Ns (1)	UNIVs ARKANSAS US (1)
Edwards Js (1)	UNIVs ARKANSAS US (1)
Carbonero F (1)	UNIVs ARKANSAS US (1)
Benamara M (1)	UNIVs ARKANSAS US (1)
Villela dias C (1)	UNIVs BAHIA BR (1)
Trovatti uetanabaro Ap (1)	UNIVs BAHIA BR (1)
Trindade Csf (1)	UNIVs BAHIA BR (1)
Teles santos T (1)	UNIVs BAHIA BR (1)
Staffolo Md (1)	UNIVs BAHIA BR (1)
Santos Tt (1)	UNIVs BAHIA BR (1)
Santos ornellas Rm (1)	UNIVs BAHIA BR (1)
Rodríguez Ms (1)	UNIVs BAHIA BR (1)
Reinheimer J (1)	UNIVs BAHIA BR (1)
Montero M (1)	UNIVs BAHIA BR (1)
Messias oliveira M (1)	UNIVs BAHIA BR (1)
Martino M (1)	UNIVs BAHIA BR (1)
de matos Fe (1)	UNIVs BAHIA BR (1)
Burns P (1)	UNIVs BAHIA BR (1)
Borges arcucio L (1)	UNIVs BAHIA BR (1)
Bevilacqua A (2)	UNIVs BAHIA BR (1)
Albertengo L (1)	UNIVs BAHIA BR (1)
Yata T (1)	UNIVs BANGKOK TH (1)
Withayagiat U (1)	UNIVs BANGKOK TH (1)
Temisak S (1)	UNIVs BANGKOK TH (1)
Seesuriyachan P (1)	UNIVs BANGKOK TH (1)
Saengkrit N (1)	UNIVs BANGKOK TH (1)
Ruxrungtham K (1)	UNIVs BANGKOK TH (1)
Puttipipatkhachorn S (1)	UNIVs BANGKOK TH (1)
Nittayasut N (1)	UNIVs BANGKOK TH (1)
Nitisinprasert S (1)	UNIVs BANGKOK TH (1)
Nguyen T-h (1)	UNIVs BANGKOK TH (1)
Namdee K (1)	UNIVs BANGKOK TH (1)
Nakkarach A (1)	UNIVs BANGKOK TH (1)
Khongkow M (1)	UNIVs BANGKOK TH (1)
Intaratrakul K (1)	UNIVs BANGKOK TH (1)
Haltrich D (1)	UNIVs BANGKOK TH (1)
Hajitou A (1)	UNIVs BANGKOK TH (1)
Chusak C (1)	UNIVs BANGKOK TH (1)
Boonrungsiman S (1)	UNIVs BANGKOK TH (1)
Ayimbila F (1)	UNIVs BANGKOK TH (1)
Asavarut P (1)	UNIVs BANGKOK TH (1)
Anuyahong T (1)	UNIVs BANGKOK TH (1)
Adisakwattana S (1)	UNIVs BANGKOK TH (1)
Keawsompong S (2)	UNIVs BANGKOK TH (2)
Kemsawasd V (3)	UNIVs BANGKOK TH (3)
Zavala L (1)	UNIVs BARCELONA ES (1)
Zangara A (1)	UNIVs BARCELONA ES (1)
Villar A (1)	UNIVs BARCELONA ES (1)
Vila L (1)	UNIVs BARCELONA ES (1)
Valls R-m (1)	UNIVs BARCELONA ES (1)
Trejo Sa (1)	UNIVs BARCELONA ES (1)
Smidt Cr (1)	UNIVs BARCELONA ES (1)
Serradell Mdla (2)	UNIVs BARCELONA ES (1)
Selma-royo M (1)	UNIVs BARCELONA ES (1)
Risco E (1)	UNIVs BARCELONA ES (1)
Parra-llorca A (1)	UNIVs BARCELONA ES (1)
Martínez-costa C (1)	UNIVs BARCELONA ES (1)

Martín-peláez S (2)	UNIVs BARCELONA ES (1)
Hernández A (1)	UNIVs BARCELONA ES (1)
Garrote Gl (1)	UNIVs BARCELONA ES (1)
García-mantrana I (1)	UNIVs BARCELONA ES (1)
Farràs M (1)	UNIVs BARCELONA ES (1)
Escuriet R (1)	UNIVs BARCELONA ES (1)
Cortés C (1)	UNIVs BARCELONA ES (1)
Collado Mc (2)	UNIVs BARCELONA ES (1)
Catalán U (1)	UNIVs BARCELONA ES (1)
Carasi P (2)	UNIVs BARCELONA ES (1)
Calatayud arroyo M (3)	UNIVs BARCELONA ES (1)
Bronsoms S (1)	UNIVs BARCELONA ES (1)
Bengoa Aa (1)	UNIVs BARCELONA ES (1)
Abraham Ag (2)	UNIVs BARCELONA ES (1)
Marcos R (2)	UNIVs BARCELONA ES (2)
García-rodríguez A (3)	UNIVs BARCELONA ES (2)
Rizzello Cg (1)	UNIVs BARI IT (1)
Dal bello F (1)	UNIVs BARI IT (1)
Cavallo N (1)	UNIVs BARI IT (1)
Francavilla R (2)	UNIVs BARI IT (2)
De angelis M (2)	UNIVs BARI IT (2)
Zuo F (1)	UNIVs BEIJING CN (1)
Zou Y (1)	UNIVs BEIJING CN (1)
Zhu Yh (1)	UNIVs BEIJING CN (1)
Zhu Q (1)	UNIVs BEIJING CN (1)
Zhou F (1)	UNIVs BEIJING CN (1)
Zhong J (1)	UNIVs BEIJING CN (1)
Zhao X (2)	UNIVs BEIJING CN (1)
Zhao M (1)	UNIVs BEIJING CN (1)
Zhao J (1)	UNIVs BEIJING CN (1)
Zhao G (1)	UNIVs BEIJING CN (1)
Zhang Z-n (1)	UNIVs BEIJING CN (1)
Zhang M (5)	UNIVs BEIJING CN (1)
Zhang L (1)	UNIVs BEIJING CN (1)
Zhai Q (1)	UNIVs BEIJING CN (1)
Zeng Z (2)	UNIVs BEIJING CN (1)
Yuanying X (1)	UNIVs BEIJING CN (1)
Yu R (1)	UNIVs BEIJING CN (1)
Yu L (1)	UNIVs BEIJING CN (1)
Yu H (2)	UNIVs BEIJING CN (1)
Yu C (1)	UNIVs BEIJING CN (1)
You X (2)	UNIVs BEIJING CN (1)
Yong T (1)	UNIVs BEIJING CN (1)
Yin N-y (1)	UNIVs BEIJING CN (1)
Yin M (1)	UNIVs BEIJING CN (1)
Yin L (1)	UNIVs BEIJING CN (1)
Ye X (1)	UNIVs BEIJING CN (1)
Yao Y (1)	UNIVs BEIJING CN (1)
Yanyan Z (1)	UNIVs BEIJING CN (1)
Yang M (2)	UNIVs BEIJING CN (1)
Yang H (1)	UNIVs BEIJING CN (1)
Yang D (1)	UNIVs BEIJING CN (1)
Yan W (1)	UNIVs BEIJING CN (1)
Yan Q (1)	UNIVs BEIJING CN (1)
Xu J (4)	UNIVs BEIJING CN (1)
Xiong J (1)	UNIVs BEIJING CN (1)
Xiaoyu Z (1)	UNIVs BEIJING CN (1)
Xiaohong H (1)	UNIVs BEIJING CN (1)
Xiao C (1)	UNIVs BEIJING CN (1)
Xia S (1)	UNIVs BEIJING CN (1)
Wei J (1)	UNIVs BEIJING CN (1)
Wei C (1)	UNIVs BEIJING CN (1)
Wang Za (1)	UNIVs BEIJING CN (1)
Wang R (2)	UNIVs BEIJING CN (1)
Wang Jf (1)	UNIVs BEIJING CN (1)

Wang G (3)	UNIVs BEIJING CN (1)
Tu P (1)	UNIVs BEIJING CN (1)
Tao Z (1)	UNIVs BEIJING CN (1)
Tao W (2)	UNIVs BEIJING CN (1)
Tao T (1)	UNIVs BEIJING CN (1)
Tao J (1)	UNIVs BEIJING CN (1)
Sun T (2)	UNIVs BEIJING CN (1)
Song Y (1)	UNIVs BEIJING CN (1)
Song Q (1)	UNIVs BEIJING CN (1)
Shen S (1)	UNIVs BEIJING CN (1)
Shen J (1)	UNIVs BEIJING CN (1)
Rahman Mh (1)	UNIVs BEIJING CN (1)
Qin J (1)	UNIVs BEIJING CN (1)
Peng C (1)	UNIVs BEIJING CN (1)
Oliviero T (1)	UNIVs BEIJING CN (1)
Miao M (1)	UNIVs BEIJING CN (1)
Mayer Mj (1)	UNIVs BEIJING CN (1)
Ma X (2)	UNIVs BEIJING CN (1)
Ma P (1)	UNIVs BEIJING CN (1)
Ma H (1)	UNIVs BEIJING CN (1)
Luo R (1)	UNIVs BEIJING CN (1)
Liu G (2)	UNIVs BEIJING CN (1)
Liu F (3)	UNIVs BEIJING CN (1)
Lin Q (1)	UNIVs BEIJING CN (1)
Lin L (1)	UNIVs BEIJING CN (1)
Liao X (1)	UNIVs BEIJING CN (1)
Liao S (1)	UNIVs BEIJING CN (1)
Li Z-j (1)	UNIVs BEIJING CN (1)
Li E (1)	UNIVs BEIJING CN (1)
Li Df (1)	UNIVs BEIJING CN (1)
Laerke Hn (1)	UNIVs BEIJING CN (1)
Knudsen Keb (1)	UNIVs BEIJING CN (1)
Khaskheli Gb (1)	UNIVs BEIJING CN (1)
Kellingray L (1)	UNIVs BEIJING CN (1)
Jørgensen H (1)	UNIVs BEIJING CN (1)
Jing B (1)	UNIVs BEIJING CN (1)
Jiang T (2)	UNIVs BEIJING CN (1)
Jia X (4)	UNIVs BEIJING CN (1)
Jia S (1)	UNIVs BEIJING CN (1)
Jia B (1)	UNIVs BEIJING CN (1)
Jensen Bb (2)	UNIVs BEIJING CN (1)
Huang S (2)	UNIVs BEIJING CN (1)
Huang J (5)	UNIVs BEIJING CN (1)
Hu Y (1)	UNIVs BEIJING CN (1)
Hu T (1)	UNIVs BEIJING CN (1)
He L (1)	UNIVs BEIJING CN (1)
Han Z (1)	UNIVs BEIJING CN (1)
Han L (1)	UNIVs BEIJING CN (1)
Guo J (2)	UNIVs BEIJING CN (1)
Gu X (1)	UNIVs BEIJING CN (1)
Gall Gl (1)	UNIVs BEIJING CN (1)
Feng Q (1)	UNIVs BEIJING CN (1)
Fan C (1)	UNIVs BEIJING CN (1)
Duan H (1)	UNIVs BEIJING CN (1)
Du Y (1)	UNIVs BEIJING CN (1)
Du X (1)	UNIVs BEIJING CN (1)
Du M (1)	UNIVs BEIJING CN (1)
Du H-l (1)	UNIVs BEIJING CN (1)
Du F (1)	UNIVs BEIJING CN (1)
Dong W (2)	UNIVs BEIJING CN (1)
Dong K (1)	UNIVs BEIJING CN (1)
Ding X (1)	UNIVs BEIJING CN (1)
Ding S (2)	UNIVs BEIJING CN (1)
Deng Q (1)	UNIVs BEIJING CN (1)
Deng B (1)	UNIVs BEIJING CN (1)

Cui Y-s (1)	UNIVs BEIJING CN (1)
Chi J (1)	UNIVs BEIJING CN (1)
Cheng L (2)	UNIVs BEIJING CN (1)
Chen Z (3)	UNIVs BEIJING CN (1)
Chen P (2)	UNIVs BEIJING CN (1)
Chen M (1)	UNIVs BEIJING CN (1)
Chang X (1)	UNIVs BEIJING CN (1)
Chai Y (1)	UNIVs BEIJING CN (1)
Cen S (1)	UNIVs BEIJING CN (1)
Capuano E (3)	UNIVs BEIJING CN (1)
Cao Y (1)	UNIVs BEIJING CN (1)
Cao W (1)	UNIVs BEIJING CN (1)
Cao M (1)	UNIVs BEIJING CN (1)
Cao J (3)	UNIVs BEIJING CN (1)
Cai X-l (1)	UNIVs BEIJING CN (1)
Cai S (2)	UNIVs BEIJING CN (1)
Ai Y (1)	UNIVs BEIJING CN (1)
Sun G (13)	UNIVs BEIJING CN (12)
Yin N (13)	UNIVs BEIJING CN (13)
Cui Y (14)	UNIVs BEIJING CN (14)
Zhao Y (3)	UNIVs BEIJING CN (2)
Zhang D (3)	UNIVs BEIJING CN (2)
Wang L (11)	UNIVs BEIJING CN (2)
Tao S (2)	UNIVs BEIJING CN (2)
Shi Y (2)	UNIVs BEIJING CN (2)
Qiao S (2)	UNIVs BEIJING CN (2)
Lu Y (2)	UNIVs BEIJING CN (2)
Li M (2)	UNIVs BEIJING CN (2)
Jiang Z (2)	UNIVs BEIJING CN (2)
Guo B (2)	UNIVs BEIJING CN (2)
Gong L (2)	UNIVs BEIJING CN (2)
Gao J (5)	UNIVs BEIJING CN (2)
Ding J (3)	UNIVs BEIJING CN (2)
Cui K (2)	UNIVs BEIJING CN (2)
Chen S (4)	UNIVs BEIJING CN (2)
Chen C (5)	UNIVs BEIJING CN (2)
Zhang H (13)	UNIVs BEIJING CN (3)
Zhang C (4)	UNIVs BEIJING CN (3)
Yang Y (9)	UNIVs BEIJING CN (3)
Yang S (5)	UNIVs BEIJING CN (3)
Wang S (8)	UNIVs BEIJING CN (3)
Sun B (3)	UNIVs BEIJING CN (3)
Sultana Ms (3)	UNIVs BEIJING CN (3)
Liu X (6)	UNIVs BEIJING CN (3)
Liu J (6)	UNIVs BEIJING CN (3)
Liu H (8)	UNIVs BEIJING CN (3)
Chen F (6)	UNIVs BEIJING CN (3)
Zhang Z (10)	UNIVs BEIJING CN (4)
Wang J (11)	UNIVs BEIJING CN (4)
Li L (11)	UNIVs BEIJING CN (4)
Chen W (6)	UNIVs BEIJING CN (4)
Zhang Y (15)	UNIVs BEIJING CN (5)
Li Z (11)	UNIVs BEIJING CN (5)
Chen X (11)	UNIVs BEIJING CN (5)
Li Y (11)	UNIVs BEIJING CN (6)
Wang P (8)	UNIVs BEIJING CN (8)
Williams Pn (2)	UNIVs BELFAST GB (1)
Lin S (1)	UNIVs BELFAST GB (1)
Hou Y (1)	UNIVs BELFAST GB (1)
Chi H (1)	UNIVs BELFAST GB (1)
Cai C (1)	UNIVs BELFAST GB (1)
Lotti C (1)	UNIVs BENGURION IL (1)
Gaudioso G (1)	UNIVs BENGURION IL (1)
Fava F (2)	UNIVs BENGURION IL (1)
Angeli A (1)	UNIVs BENGURION IL (1)

Vrhovsek U (3)	UNIVs BENGURION IL (2)
Tuohy K (3)	UNIVs BENGURION IL (2)
Diotallevi C (2)	UNIVs BENGURION IL (2)
Shai I (3)	UNIVs BENGURION IL (3)
Rinott E (3)	UNIVs BENGURION IL (3)
Verstraelen H (1)	UNIVs BERLIN DE (1)
Swidsinski S (1)	UNIVs BERLIN DE (1)
Swidsinski A (1)	UNIVs BERLIN DE (1)
Schulz S (1)	UNIVs BERLIN DE (1)
Rösler U (1)	UNIVs BERLIN DE (1)
Pöppe J (1)	UNIVs BERLIN DE (1)
Manowsky J (1)	UNIVs BERLIN DE (1)
Loening-baucke V (1)	UNIVs BERLIN DE (1)
Juhr N-c (1)	UNIVs BERLIN DE (1)
Haas A (1)	UNIVs BERLIN DE (1)
Tselepis C (1)	UNIVs BIRMINGHAM GB (1)
Tilston E (1)	UNIVs BIRMINGHAM GB (1)
Quraishi Mn (1)	UNIVs BIRMINGHAM GB (1)
O'meara S (1)	UNIVs BIRMINGHAM GB (1)
Mistry P (1)	UNIVs BIRMINGHAM GB (1)
Mills C (1)	UNIVs BIRMINGHAM GB (1)
Keppler S (1)	UNIVs BIRMINGHAM GB (1)
Iqbal Th (1)	UNIVs BIRMINGHAM GB (1)
Horniblow Rd (1)	UNIVs BIRMINGHAM GB (1)
Hood Ga (1)	UNIVs BIRMINGHAM GB (1)
Harrad S (1)	UNIVs BIRMINGHAM GB (1)
Hanson Kj (1)	UNIVs BIRMINGHAM GB (1)
Collins C (1)	UNIVs BIRMINGHAM GB (1)
Beggs Ad (1)	UNIVs BIRMINGHAM GB (1)
Abou-elwafa abdallah M (1)	UNIVs BIRMINGHAM GB (1)
Jaime-fonseca Mr (2)	UNIVs BIRMINGHAM GB (2)
Gouseti O (2)	UNIVs BIRMINGHAM GB (2)
Fryer Pj (3)	UNIVs BIRMINGHAM GB (3)
Bakalis S (3)	UNIVs BIRMINGHAM GB (3)
van der linde C (1)	UNIVs BOLOGNA IT (1)
Uraipan S (1)	UNIVs BOLOGNA IT (1)
Severgnini M (1)	UNIVs BOLOGNA IT (1)
Sansosti Mc (1)	UNIVs BOLOGNA IT (1)
Rampelli S (1)	UNIVs BOLOGNA IT (1)
Qvirist L (1)	UNIVs BOLOGNA IT (1)
Quercia S (1)	UNIVs BOLOGNA IT (1)
Nissen L (1)	UNIVs BOLOGNA IT (1)
Ndagijimana M (1)	UNIVs BOLOGNA IT (1)
Modesto M (1)	UNIVs BOLOGNA IT (1)
Michelini S (1)	UNIVs BOLOGNA IT (1)
Mattarelli P (1)	UNIVs BOLOGNA IT (1)
Keleszade E (1)	UNIVs BOLOGNA IT (1)
Hongpattarakere T (2)	UNIVs BOLOGNA IT (1)
Gianotti A (1)	UNIVs BOLOGNA IT (1)
Di nunzio M (1)	UNIVs BOLOGNA IT (1)
D'alessandro M (1)	UNIVs BOLOGNA IT (1)
Consolandi C (1)	UNIVs BOLOGNA IT (1)
Chiarello E (1)	UNIVs BOLOGNA IT (1)
Centanni M (2)	UNIVs BOLOGNA IT (1)
Cavina P (1)	UNIVs BOLOGNA IT (1)
Casciano F (2)	UNIVs BOLOGNA IT (1)
Candela M (1)	UNIVs BOLOGNA IT (1)
Camprini L (1)	UNIVs BOLOGNA IT (1)
Calanni F (1)	UNIVs BOLOGNA IT (1)
Bordoni A (1)	UNIVs BOLOGNA IT (1)
Biagi E (1)	UNIVs BOLOGNA IT (1)
Barone M (1)	UNIVs BOLOGNA IT (1)
Turroni S (2)	UNIVs BOLOGNA IT (2)
Serrazanetti Di (2)	UNIVs BOLOGNA IT (2)
Parolin C (2)	UNIVs BOLOGNA IT (2)

Maccaferri S (2)	UNIVs BOLOGNA IT (2)
Laghi L (2)	UNIVs BOLOGNA IT (2)
Vitali B (3)	UNIVs BOLOGNA IT (3)
Siroli L (3)	UNIVs BOLOGNA IT (3)
Patrignani F (3)	UNIVs BOLOGNA IT (3)
Lanciotti R (3)	UNIVs BOLOGNA IT (3)
Brigidi P (5)	UNIVs BOLOGNA IT (5)
Urdaci Mc (1)	UNIVs BORDEAUX FR (1)
Romanin De (1)	UNIVs BORDEAUX FR (1)
Jacquot C (1)	UNIVs BORDEAUX FR (1)
Elie A-m (1)	UNIVs BORDEAUX FR (1)
De antoni Gl (1)	UNIVs BORDEAUX FR (1)
Zecchin S (1)	UNIVs BOZEN BOLZANO IT (1)
Zarur coelho Ma (1)	UNIVs BOZEN BOLZANO IT (1)
Zanchi R (1)	UNIVs BOZEN BOLZANO IT (1)
Villa F (1)	UNIVs BOZEN BOLZANO IT (1)
Signori pereira K (1)	UNIVs BOZEN BOLZANO IT (1)
Pereira de paula B (1)	UNIVs BOZEN BOLZANO IT (1)
Garuglieri E (1)	UNIVs BOZEN BOLZANO IT (1)
Firmino L (1)	UNIVs BOZEN BOLZANO IT (1)
Fioravante guerra A (2)	UNIVs BOZEN BOLZANO IT (1)
Ferreira dutra corrêa M (1)	UNIVs BOZEN BOLZANO IT (1)
Fernandes lemos júnior Wj (1)	UNIVs BOZEN BOLZANO IT (1)
Erba D (1)	UNIVs BOZEN BOLZANO IT (1)
de souza lago H (1)	UNIVs BOZEN BOLZANO IT (1)
Cattò C (1)	UNIVs BOZEN BOLZANO IT (1)
Cappitelli F (1)	UNIVs BOZEN BOLZANO IT (1)
Borruso L (1)	UNIVs BOZEN BOLZANO IT (1)
Gobbetti M (3)	UNIVs BOZEN BOLZANO IT (3)
Mesquita Mc (1)	UNIVs BRASILIA BR (1)
Leandro Es (1)	UNIVs BRASILIA BR (1)
de alencar Er (1)	UNIVs BRASILIA BR (1)
Botelho Rba (1)	UNIVs BRASILIA BR (1)
Wade Kh (1)	UNIVs BRISTOL GB (1)
Hall Lj (1)	UNIVs BRISTOL GB (1)
Rivero-pérez Md (1)	UNIVs BURGOS ES (1)
Pino-garcía Rd (1)	UNIVs BURGOS ES (1)
Ortega-heras M (1)	UNIVs BURGOS ES (1)
Muñiz P (1)	UNIVs BURGOS ES (1)
Lomillo Jg (1)	UNIVs BURGOS ES (1)
Gonzalez-sanjosé Ml (1)	UNIVs BURGOS ES (1)
Zanelli Cf (1)	UNIVs CALIFORNIA US (1)
Yin J (2)	UNIVs CALIFORNIA US (1)
Yan H (1)	UNIVs CALIFORNIA US (1)
Xian Q (1)	UNIVs CALIFORNIA US (1)
Wu M (2)	UNIVs CALIFORNIA US (1)
Wada Y (1)	UNIVs CALIFORNIA US (1)
Valtchev P (1)	UNIVs CALIFORNIA US (1)
Tringe S (1)	UNIVs CALIFORNIA US (1)
Thomas Bc (1)	UNIVs CALIFORNIA US (1)
Shen Y (1)	UNIVs CALIFORNIA US (1)
Schindeler A (1)	UNIVs CALIFORNIA US (1)
Schantz Ab (1)	UNIVs CALIFORNIA US (1)
Santoni Mm (1)	UNIVs CALIFORNIA US (1)
Saboe P (1)	UNIVs CALIFORNIA US (1)
Rohwer Fl (1)	UNIVs CALIFORNIA US (1)
Rincón-rosales R (1)	UNIVs CALIFORNIA US (1)
Reyes A (1)	UNIVs CALIFORNIA US (1)
Ren T (1)	UNIVs CALIFORNIA US (1)
Ren H (1)	UNIVs CALIFORNIA US (1)
Rejón-orantes Jc (1)	UNIVs CALIFORNIA US (1)
Read Mn (1)	UNIVs CALIFORNIA US (1)
Peña-ocaña Ba (1)	UNIVs CALIFORNIA US (1)
Naficy S (1)	UNIVs CALIFORNIA US (1)
Milenkovic D (1)	UNIVs CALIFORNIA US (1)

Mesa V (1)	UNIVs CALIFORNIA US (1)
Mennah-govela Ya (1)	UNIVs CALIFORNIA US (1)
Megerlin F (1)	UNIVs CALIFORNIA US (1)
Mcnulty Np (1)	UNIVs CALIFORNIA US (1)
Mccuskey S (1)	UNIVs CALIFORNIA US (1)
Mcclure Dd (1)	UNIVs CALIFORNIA US (1)
Lopert R (1)	UNIVs CALIFORNIA US (1)
Lönnerdal B (1)	UNIVs CALIFORNIA US (1)
Lean Mej (1)	UNIVs CALIFORNIA US (1)
Le Tyl (1)	UNIVs CALIFORNIA US (1)
Kumar M (1)	UNIVs CALIFORNIA US (1)
Kong F (1)	UNIVs CALIFORNIA US (1)
Khademhosseini A (1)	UNIVs CALIFORNIA US (1)
Kantor Rs (1)	UNIVs CALIFORNIA US (1)
Iyer R (1)	UNIVs CALIFORNIA US (1)
Huddy Rj (1)	UNIVs CALIFORNIA US (1)
Hu S (1)	UNIVs CALIFORNIA US (1)
Hettich Rl (1)	UNIVs CALIFORNIA US (1)
Harrison Stl (1)	UNIVs CALIFORNIA US (1)
Hara Y (1)	UNIVs CALIFORNIA US (1)
Hall Jf (1)	UNIVs CALIFORNIA US (1)
Gutiérrez-sarmiento W (1)	UNIVs CALIFORNIA US (1)
Gutiérrez-miceli Fa (1)	UNIVs CALIFORNIA US (1)
Grzelakowski M (1)	UNIVs CALIFORNIA US (1)
Gong T (1)	UNIVs CALIFORNIA US (1)
Fouassier E (1)	UNIVs CALIFORNIA US (1)
Fois Cam (1)	UNIVs CALIFORNIA US (1)
Feroz H (1)	UNIVs CALIFORNIA US (1)
Erbakan M (1)	UNIVs CALIFORNIA US (1)
Demarqui Fm (1)	UNIVs CALIFORNIA US (1)
Dehghani F (1)	UNIVs CALIFORNIA US (1)
Cherr Gn (1)	UNIVs CALIFORNIA US (1)
Butler Pj (1)	UNIVs CALIFORNIA US (1)
Brown Ct (1)	UNIVs CALIFORNIA US (1)
Brindani N (1)	UNIVs CALIFORNIA US (1)
Bourlioux P (1)	UNIVs CALIFORNIA US (1)
Bazan Gc (1)	UNIVs CALIFORNIA US (1)
Barbieri E (1)	UNIVs CALIFORNIA US (1)
Banfield Jf (1)	UNIVs CALIFORNIA US (1)
Anantharaman K (1)	UNIVs CALIFORNIA US (1)
Abud-archila M (1)	UNIVs CALIFORNIA US (1)
Traina Sj (3)	UNIVs CALIFORNIA US (3)
Scheckel Kg (3)	UNIVs CALIFORNIA US (3)
Bornhorst Gm (3)	UNIVs CALIFORNIA US (3)
Beak Dg (3)	UNIVs CALIFORNIA US (3)
Basta Nt (3)	UNIVs CALIFORNIA US (3)
Singh Rp (4)	UNIVs CALIFORNIA US (4)
Lizunkova P (1)	UNIVs CAMBRIDGE GB (1)
King L (1)	UNIVs CAMBRIDGE GB (1)
Gautam L (1)	UNIVs CAMBRIDGE GB (1)
Enuwosa E (2)	UNIVs CAMBRIDGE GB (2)
Chichger H (2)	UNIVs CAMBRIDGE GB (2)
Yaqoob P (1)	UNIVs CAMERINO IT (1)
Spencer Jpe (1)	UNIVs CAMERINO IT (1)
Silvi S (1)	UNIVs CAMERINO IT (1)
Shortt Ct (1)	UNIVs CAMERINO IT (1)
Hughes Rm (1)	UNIVs CAMERINO IT (1)
Hotchkiss S (1)	UNIVs CAMERINO IT (1)
Cresci A (1)	UNIVs CAMERINO IT (1)
Corona G (1)	UNIVs CAMERINO IT (1)
Commane Dm (2)	UNIVs CAMERINO IT (1)
Coman Mm (1)	UNIVs CAMERINO IT (1)
Rolim Frl (1)	UNIVs CAMPINA GRANDE BR (1)
Ribeiro Ts (1)	UNIVs CAMPINA GRANDE BR (1)
Queiroga Rdcrde (1)	UNIVs CAMPINA GRANDE BR (1)

dos santos Kmo (2)	UNIVs CAMPINA GRANDE BR (1)
do egito As (1)	UNIVs CAMPINA GRANDE BR (1)
de oliveira Meg (1)	UNIVs CAMPINA GRANDE BR (1)
de barcelos Sc (1)	UNIVs CAMPINA GRANDE BR (1)
Cavalcanti Mt (1)	UNIVs CAMPINA GRANDE BR (1)
Borges Cwp (1)	UNIVs CAMPINA GRANDE BR (1)
Souza pedrosa Gt (1)	UNIVs CAMPINAS BR (1)
Soares Mb (1)	UNIVs CAMPINAS BR (1)
Sisconeto bisinotto M (1)	UNIVs CAMPINAS BR (1)
Silva Fgd (1)	UNIVs CAMPINAS BR (1)
Silva Ek (1)	UNIVs CAMPINAS BR (1)
Rodrigues Rf (1)	UNIVs CAMPINAS BR (1)
Rodrigues E (1)	UNIVs CAMPINAS BR (1)
Rodrigues Db (1)	UNIVs CAMPINAS BR (1)
Rampelotto C (1)	UNIVs CAMPINAS BR (1)
Quatrin A (1)	UNIVs CAMPINAS BR (1)
Petry Fc (1)	UNIVs CAMPINAS BR (1)
Pereira Epr (1)	UNIVs CAMPINAS BR (1)
Pauletto R (1)	UNIVs CAMPINAS BR (1)
O'callagahan Y (1)	UNIVs CAMPINAS BR (1)
O'brien Nm (2)	UNIVs CAMPINAS BR (1)
Nichelle Sm (1)	UNIVs CAMPINAS BR (1)
Neves bezerra Rm (1)	UNIVs CAMPINAS BR (1)
Netto Fm (2)	UNIVs CAMPINAS BR (1)
Neri-numa Ia (1)	UNIVs CAMPINAS BR (1)
Moreira simabuco F (1)	UNIVs CAMPINAS BR (1)
Mello Rdo (1)	UNIVs CAMPINAS BR (1)
Meireles Maa (1)	UNIVs CAMPINAS BR (1)
Maurer Lh (1)	UNIVs CAMPINAS BR (1)
Maróstica junior Mr (1)	UNIVs CAMPINAS BR (1)
Mariutti Lrb (1)	UNIVs CAMPINAS BR (1)
Lollo Pc (1)	UNIVs CAMPINAS BR (1)
Klein B (1)	UNIVs CAMPINAS BR (1)
Guimarães Jt (1)	UNIVs CAMPINAS BR (1)
Freitas Mq (1)	UNIVs CAMPINAS BR (1)
Fonseca Bds (1)	UNIVs CAMPINAS BR (1)
Emanuelli T (1)	UNIVs CAMPINAS BR (1)
Dias-audibert Fl (1)	UNIVs CAMPINAS BR (1)
Delafiori J (1)	UNIVs CAMPINAS BR (1)
de paulo farias D (1)	UNIVs CAMPINAS BR (1)
de menezes Cr (1)	UNIVs CAMPINAS BR (1)
de carvalho fino L (1)	UNIVs CAMPINAS BR (1)
de araújo Ff (1)	UNIVs CAMPINAS BR (1)
da silva Dc (1)	UNIVs CAMPINAS BR (1)
Costa antunes Ae (1)	UNIVs CAMPINAS BR (1)
Catharino Rr (1)	UNIVs CAMPINAS BR (1)
Bochi Vc (1)	UNIVs CAMPINAS BR (1)
Bertoldo pacheco Mt (1)	UNIVs CAMPINAS BR (1)
Balthazar Cf (1)	UNIVs CAMPINAS BR (1)
Arruda Hs (1)	UNIVs CAMPINAS BR (1)
Almada-érix Cn (1)	UNIVs CAMPINAS BR (1)
Almada Cn (1)	UNIVs CAMPINAS BR (1)
Sant'ana As (2)	UNIVs CAMPINAS BR (2)
Pastore Gm (2)	UNIVs CAMPINAS BR (2)
Mercadante Az (2)	UNIVs CAMPINAS BR (2)
Cruz Ag (2)	UNIVs CAMPINAS BR (2)
Thursz Mr (1)	UNIVs CARDIFF GB (1)
Pechlivanis A (1)	UNIVs CARDIFF GB (1)
Mullish Bh (1)	UNIVs CARDIFF GB (1)
Marchesi Jr (1)	UNIVs CARDIFF GB (1)
Li Jv (1)	UNIVs CARDIFF GB (1)
Kao D (1)	UNIVs CARDIFF GB (1)
Holmes E (1)	UNIVs CARDIFF GB (1)
Clarke Tb (1)	UNIVs CARDIFF GB (1)
Brignardello J (1)	UNIVs CARDIFF GB (1)

Restuccia C (1)	UNIVs CATANIA IT (1)
Randazzo Cl (1)	UNIVs CATANIA IT (1)
Poveda turrado C (1)	UNIVs CATANIA IT (1)
Oruna-concha Mj (1)	UNIVs CATANIA IT (1)
Le marc Y (1)	UNIVs CATANIA IT (1)
John wickham Ms (1)	UNIVs CATANIA IT (1)
Fallico B (1)	UNIVs CATANIA IT (1)
Dainty Jr (1)	UNIVs CATANIA IT (1)
Caggia C (1)	UNIVs CATANIA IT (1)
Brighina S (1)	UNIVs CATANIA IT (1)
Arena E (1)	UNIVs CATANIA IT (1)
Pitino I (2)	UNIVs CATANIA IT (2)
Lo curto A (2)	UNIVs CATANIA IT (2)
Santos Bn (1)	UNIVs CEARA BR (1)
Rabelo Mc (1)	UNIVs CEARA BR (1)
Pinto Gas (1)	UNIVs CEARA BR (1)
Leite Akf (1)	UNIVs CEARA BR (1)
Honorato Tl (1)	UNIVs CEARA BR (1)
Gonçalves Lrb (1)	UNIVs CEARA BR (1)
Fonteles Tv (1)	UNIVs CEARA BR (1)
Rodrigues S (2)	UNIVs CEARA BR (2)
Zheng Js (1)	UNIVs CHONGQING CN (1)
Jiang Ll (1)	UNIVs CHONGQING CN (1)
Huang H (1)	UNIVs CHONGQING CN (1)
Fang Lc (1)	UNIVs CHONGQING CN (1)
Deng J (1)	UNIVs CHONGQING CN (1)
Tsen J-h (1)	UNIVs CHUNG HSING TW (1)
Tsai J-j (1)	UNIVs CHUNG HSING TW (1)
Lin S-l (1)	UNIVs CHUNG HSING TW (1)
Lin M-y (1)	UNIVs CHUNG HSING TW (1)
King Vae (1)	UNIVs CHUNG HSING TW (1)
Huang H-y (1)	UNIVs CHUNG HSING TW (1)
Hsieh H-y (1)	UNIVs CHUNG HSING TW (1)
Chiu Y-h (1)	UNIVs CHUNG HSING TW (1)
Chi L-l (1)	UNIVs CHUNG HSING TW (1)
Yao A-f (1)	UNIVs CLERMONT FERRAND FR (1)
Tessier Fj (1)	UNIVs CLERMONT FERRAND FR (1)
Spatz M (1)	UNIVs CLERMONT FERRAND FR (1)
Sicardi V (1)	UNIVs CLERMONT FERRAND FR (1)
Schwintner C (1)	UNIVs CLERMONT FERRAND FR (1)
Rame V (1)	UNIVs CLERMONT FERRAND FR (1)
Marier D (1)	UNIVs CLERMONT FERRAND FR (1)
Manzi Ap (1)	UNIVs CLERMONT FERRAND FR (1)
Le camus C (1)	UNIVs CLERMONT FERRAND FR (1)
Jiménez-marín A (1)	UNIVs CLERMONT FERRAND FR (1)
Helou C (1)	UNIVs CLERMONT FERRAND FR (1)
Guerra A (1)	UNIVs CLERMONT FERRAND FR (1)
Gérard-champod M (1)	UNIVs CLERMONT FERRAND FR (1)
Gasc C (1)	UNIVs CLERMONT FERRAND FR (1)
Garrido Jj (1)	UNIVs CLERMONT FERRAND FR (1)
Garrait G (1)	UNIVs CLERMONT FERRAND FR (1)
Gadonna-widehem P (1)	UNIVs CLERMONT FERRAND FR (1)
François O (1)	UNIVs CLERMONT FERRAND FR (1)
Delmas D (1)	UNIVs CLERMONT FERRAND FR (1)
Bravo D (2)	UNIVs CLERMONT FERRAND FR (1)
Boucinha L (1)	UNIVs CLERMONT FERRAND FR (1)
Borrel G (1)	UNIVs CLERMONT FERRAND FR (1)
Beyssac E (1)	UNIVs CLERMONT FERRAND FR (1)
Blanquet-diot S (15)	UNIVs CLERMONT FERRAND FR (15)
Alric M (15)	UNIVs CLERMONT FERRAND FR (15)
Denis S (16)	UNIVs CLERMONT FERRAND FR (16)
Verdier C (2)	UNIVs CLERMONT FERRAND FR (2)
le goff O (2)	UNIVs CLERMONT FERRAND FR (2)
Forano E (2)	UNIVs CLERMONT FERRAND FR (2)
Uriot O (3)	UNIVs CLERMONT FERRAND FR (3)

Tottey W (3)	UNIVs CLERMONT FERRAND FR (3)
Gaci N (3)	UNIVs CLERMONT FERRAND FR (3)
Feria-gervasio D (3)	UNIVs CLERMONT FERRAND FR (3)
Cardot J-m (3)	UNIVs CLERMONT FERRAND FR (3)
Livrelli V (4)	UNIVs CLERMONT FERRAND FR (4)
Leriche F (4)	UNIVs CLERMONT FERRAND FR (4)
Etienne-mesmin L (4)	UNIVs CLERMONT FERRAND FR (4)
Brugère J-f (4)	UNIVs CLERMONT FERRAND FR (4)
Galia W (5)	UNIVs CLERMONT FERRAND FR (5)
Chalancon S (7)	UNIVs CLERMONT FERRAND FR (7)
Vuono Dc (1)	UNIVs COLORADO US (1)
Spear Jr (1)	UNIVs COLORADO US (1)
Munakata-marr J (1)	UNIVs COLORADO US (1)
Drewes Je (1)	UNIVs COLORADO US (1)
Sofos Jn (2)	UNIVs COLORADO US (2)
Kendall Pa (2)	UNIVs COLORADO US (2)
Geornaras I (2)	UNIVs COLORADO US (2)
Barmpalia-davis Im (2)	UNIVs COLORADO US (2)
Zhou X-h (1)	UNIVs CONNECTICUT US (1)
Yin Y-q (1)	UNIVs CONNECTICUT US (1)
Yang Z-q (1)	UNIVs CONNECTICUT US (1)
Rao S-q (1)	UNIVs CONNECTICUT US (1)
Jiao X-a (1)	UNIVs CONNECTICUT US (1)
Gao L (2)	UNIVs CONNECTICUT US (1)
Edberg Sc (1)	UNIVs CONNECTICUT US (1)
Duncan He (1)	UNIVs CONNECTICUT US (1)
Chen D-w (1)	UNIVs CONNECTICUT US (1)
Youravong W (2)	UNIVs COPENHAGEN DK (1)
Weiss G (1)	UNIVs COPENHAGEN DK (1)
Tieghi Tdm (1)	UNIVs COPENHAGEN DK (1)
Thuanthong M (1)	UNIVs COPENHAGEN DK (1)
Sørensen Sj (1)	UNIVs COPENHAGEN DK (1)
Sørensen H (1)	UNIVs COPENHAGEN DK (1)
Soffer N (1)	UNIVs COPENHAGEN DK (1)
Sirinupong N (1)	UNIVs COPENHAGEN DK (1)
Otte J (1)	UNIVs COPENHAGEN DK (1)
Nesme J (1)	UNIVs COPENHAGEN DK (1)
Moodley A (1)	UNIVs COPENHAGEN DK (1)
Marcial-coba Ms (1)	UNIVs COPENHAGEN DK (1)
Madsen Js (1)	UNIVs COPENHAGEN DK (1)
Knøchel S (1)	UNIVs COPENHAGEN DK (1)
Khakimov B (1)	UNIVs COPENHAGEN DK (1)
Jasinskyte D (1)	UNIVs COPENHAGEN DK (1)
Jana B (1)	UNIVs COPENHAGEN DK (1)
Guardabassi L (1)	UNIVs COPENHAGEN DK (1)
De gobba C (1)	UNIVs COPENHAGEN DK (1)
Dalsgaard A (1)	UNIVs COPENHAGEN DK (1)
Cahú Tb (1)	UNIVs COPENHAGEN DK (1)
Bortolaia V (1)	UNIVs COPENHAGEN DK (1)
Anjum M (1)	UNIVs COPENHAGEN DK (1)
van den berg F (2)	UNIVs COPENHAGEN DK (2)
Nielsen S (2)	UNIVs COPENHAGEN DK (2)
Larsen N (2)	UNIVs COPENHAGEN DK (2)
Blennow A (2)	UNIVs COPENHAGEN DK (2)
Wiese M (3)	UNIVs COPENHAGEN DK (3)
Cieplak T (3)	UNIVs COPENHAGEN DK (3)
Jespersen L (4)	UNIVs COPENHAGEN DK (4)
Nielsen Ds (5)	UNIVs COPENHAGEN DK (5)
Sánchez-parra M (1)	UNIVs CORDOBA ES (1)
Ordóñez Jl (1)	UNIVs CORDOBA ES (1)
Moreno-rojas R (1)	UNIVs CORDOBA ES (1)
De santiago E (2)	UNIVs CORDOBA ES (1)
Cáceres-jiménez S (1)	UNIVs CORDOBA ES (1)
Roldán-guerra Fj (2)	UNIVs CORDOBA ES (2)
Ortíz-somovilla V (2)	UNIVs CORDOBA ES (2)

Ordóñez-díaz Jl (2)	UNIVs CORDOBA ES (2)
Pereira-caro G (5)	UNIVs CORDOBA ES (3)
Moreno-ortega A (3)	UNIVs CORDOBA ES (3)
You Y (1)	UNIVs DELAWARE US (1)
Jin Y (2)	UNIVs DELAWARE US (1)
Chiu Pc (1)	UNIVs DELAWARE US (1)
Pfetzing P (1)	UNIVs DUISBURG-ESSEN DE (1)
Hurtado-martinez M (1)	UNIVs DUISBURG-ESSEN DE (1)
Gonzalez-martinez A (1)	UNIVs DUISBURG-ESSEN DE (1)
Diaz-bone Ra (1)	UNIVs DUISBURG-ESSEN DE (1)
Smith Ar (1)	UNIVs DUNDEE GB (1)
Reid Sj (1)	UNIVs DUNDEE GB (1)
Newton Df (2)	UNIVs DUNDEE GB (1)
Lewanika Tr (1)	UNIVs DUNDEE GB (1)
Kennedy A (1)	UNIVs DUNDEE GB (1)
Abratt Vr (1)	UNIVs DUNDEE GB (1)
Macfarlane Gt (23)	UNIVs DUNDEE GB (10)
Reynolds N (2)	UNIVs DUNDEE GB (2)
Hopkins Mj (3)	UNIVs DUNDEE GB (2)
Child Mw (2)	UNIVs DUNDEE GB (2)
Bahrami B (4)	UNIVs DUNDEE GB (4)
Macfarlane S (15)	UNIVs DUNDEE GB (8)
Urbán P (1)	UNIVs EDINBURGH GB (1)
Stone V (1)	UNIVs EDINBURGH GB (1)
Stewart D (1)	UNIVs EDINBURGH GB (1)
Mcdougall Gj (1)	UNIVs EDINBURGH GB (1)
Magee P (1)	UNIVs EDINBURGH GB (1)
Kinsner-ovaskainen A (1)	UNIVs EDINBURGH GB (1)
Kanase N (1)	UNIVs EDINBURGH GB (1)
Kämpfer Aam (1)	UNIVs EDINBURGH GB (1)
González-barrio R (1)	UNIVs EDINBURGH GB (1)
Gioria S (1)	UNIVs EDINBURGH GB (1)
Brown Em (1)	UNIVs EDINBURGH GB (1)
Valero M (1)	UNIVs ELCHE ES (1)
Saura D (1)	UNIVs ELCHE ES (1)
Salazar-bermeo J (1)	UNIVs ELCHE ES (1)
Moreno-chamba B (1)	UNIVs ELCHE ES (1)
Martínez-madrid Mc (1)	UNIVs ELCHE ES (1)
Martí N (1)	UNIVs ELCHE ES (1)
Wilson Aj (1)	UNIVs EXETER GB (1)
Stothart Mr (1)	UNIVs EXETER GB (1)
Poissant J (1)	UNIVs EXETER GB (1)
Mcloughlin Pd (1)	UNIVs EXETER GB (1)
Henry A (1)	UNIVs EXETER GB (1)
Greuel Rj (1)	UNIVs EXETER GB (1)
Gavriliuc S (1)	UNIVs EXETER GB (1)
Zoccatelli G (1)	UNIVs FLORENCE IT (1)
Zanoni F (1)	UNIVs FLORENCE IT (1)
Mamone V (1)	UNIVs FLORENCE IT (1)
Mainente F (1)	UNIVs FLORENCE IT (1)
Gori G (1)	UNIVs FLORENCE IT (1)
Ghilardi M (1)	UNIVs FLORENCE IT (1)
Frediani G (1)	UNIVs FLORENCE IT (1)
Franzetti A (1)	UNIVs FLORENCE IT (1)
Ferrari V (1)	UNIVs FLORENCE IT (1)
De marchi L (1)	UNIVs FLORENCE IT (1)
Daghio M (1)	UNIVs FLORENCE IT (1)
Cei D (1)	UNIVs FLORENCE IT (1)
Cecchi L (1)	UNIVs FLORENCE IT (1)
Busfield Jjc (1)	UNIVs FLORENCE IT (1)
Bellumori M (1)	UNIVs FLORENCE IT (1)
Giuliani C (2)	UNIVs FLORENCE IT (2)
Carpi F (2)	UNIVs FLORENCE IT (2)
Mulinacci N (3)	UNIVs FLORENCE IT (3)
Innocenti M (3)	UNIVs FLORENCE IT (3)

Yang K (2)	UNIVs FLORIDA US (1)
Yadav H (1)	UNIVs FLORIDA US (1)
Xu M (2)	UNIVs FLORIDA US (1)
Wilson As (1)	UNIVs FLORIDA US (1)
Vishnubhotla R (1)	UNIVs FLORIDA US (1)
Vidi P-a (1)	UNIVs FLORIDA US (1)
Soto-pantoja Dr (1)	UNIVs FLORIDA US (1)
Shan S (1)	UNIVs FLORIDA US (1)
Schneider Rg (1)	UNIVs FLORIDA US (1)
Ramirez Mu (1)	UNIVs FLORIDA US (1)
Levine Ea (1)	UNIVs FLORIDA US (1)
Lelievre Sa (1)	UNIVs FLORIDA US (1)
Kucera Gl (1)	UNIVs FLORIDA US (1)
Glover Sc (1)	UNIVs FLORIDA US (1)
Gaber M (1)	UNIVs FLORIDA US (1)
Cruz-diaz N (1)	UNIVs FLORIDA US (1)
Cook Kl (1)	UNIVs FLORIDA US (1)
Clear Kyj (1)	UNIVs FLORIDA US (1)
Cho M (1)	UNIVs FLORIDA US (1)
Chiba A (1)	UNIVs FLORIDA US (1)
Chauhan C (1)	UNIVs FLORIDA US (1)
Chaboub L (1)	UNIVs FLORIDA US (1)
Bronson Sm (1)	UNIVs FLORIDA US (1)
Bharadwaj S (1)	UNIVs FLORIDA US (1)
Balakrishnan G (1)	UNIVs FLORIDA US (1)
Arnone Aa (1)	UNIVs FLORIDA US (1)
Sinigaglia M (1)	UNIVs FOGGIA IT (1)
Petruzzi L (1)	UNIVs FOGGIA IT (1)
Landriscina L (1)	UNIVs FOGGIA IT (1)
Lamacchia C (1)	UNIVs FOGGIA IT (1)
Gonzalez-salvador I (1)	UNIVs FOGGIA IT (1)
Corbo Mr (1)	UNIVs FOGGIA IT (1)
Bergillos-meca T (1)	UNIVs FOGGIA IT (1)
Zhou D (1)	UNIVs FUJIAN CN (1)
Yu Y (5)	UNIVs FUJIAN CN (1)
Xu W (1)	UNIVs FUJIAN CN (1)
Woo Mw (1)	UNIVs FUJIAN CN (1)
Vincken J-p (2)	UNIVs FUJIAN CN (1)
Tian L (1)	UNIVs FUJIAN CN (1)
Shao R (1)	UNIVs FUJIAN CN (1)
Selomulya C (1)	UNIVs FUJIAN CN (1)
Mao H (1)	UNIVs FUJIAN CN (1)
Liao Z (1)	UNIVs FUJIAN CN (1)
Liang Y (1)	UNIVs FUJIAN CN (1)
Li F (3)	UNIVs FUJIAN CN (1)
Jayemanne A (1)	UNIVs FUJIAN CN (1)
Ge X (1)	UNIVs FUJIAN CN (1)
Fan T (1)	UNIVs FUJIAN CN (1)
Deng R (2)	UNIVs FUJIAN CN (1)
De bruijn Wjc (1)	UNIVs FUJIAN CN (1)
Dai H (1)	UNIVs FUJIAN CN (1)
Cheng D (1)	UNIVs FUJIAN CN (1)
Bruins Me (1)	UNIVs FUJIAN CN (1)
Xu Y (5)	UNIVs FUJIAN CN (2)
Wu P (2)	UNIVs FUJIAN CN (2)
Chen Xd (3)	UNIVs FUJIAN CN (3)
Chen L (7)	UNIVs FUJIAN CN (3)
Ye Z (1)	UNIVs GANSU CN (1)
Wang T (5)	UNIVs GANSU CN (1)
Wang K (3)	UNIVs GANSU CN (1)
Liu D (4)	UNIVs GANSU CN (1)
Dong J (1)	UNIVs GANSU CN (1)
Quero Gm (1)	UNIVs GENOA IT (1)
Mapelli F (1)	UNIVs GENOA IT (1)
Manfredini G (1)	UNIVs GENOA IT (1)

Luna Gm (1)	UNIVs GENOA IT (1)
Fontaneto D (1)	UNIVs GENOA IT (1)
Eckert Em (1)	UNIVs GENOA IT (1)
Di cesare A (1)	UNIVs GENOA IT (1)
Corno G (1)	UNIVs GENOA IT (1)
Borin S (1)	UNIVs GENOA IT (1)
Tiedje Jm (1)	UNIVs GEORGIA US (1)
Østman B (1)	UNIVs GEORGIA US (1)
Konstantinidis Kt (1)	UNIVs GEORGIA US (1)
Gewirtz At (1)	UNIVs GEORGIA US (1)
Cole J (1)	UNIVs GEORGIA US (1)
Chassaing B (1)	UNIVs GEORGIA US (1)
Chai B (1)	UNIVs GEORGIA US (1)
Wyse C (1)	UNIVs GLASGOW GB (1)
Wightman Jd (1)	UNIVs GLASGOW GB (1)
Süring C (1)	UNIVs GLASGOW GB (1)
Stalmach A (1)	UNIVs GLASGOW GB (1)
Sloan Wt (1)	UNIVs GLASGOW GB (1)
Ofiteru Id (1)	UNIVs GLASGOW GB (1)
Johannessen Ea (1)	UNIVs GLASGOW GB (1)
Jalil Amm (1)	UNIVs GLASGOW GB (1)
Hübschmann T (1)	UNIVs GLASGOW GB (1)
Hou M (1)	UNIVs GLASGOW GB (1)
Grimm V (1)	UNIVs GLASGOW GB (1)
Garcia Al (1)	UNIVs GLASGOW GB (1)
Cumming Drs (1)	UNIVs GLASGOW GB (1)
Cooper Jm (2)	UNIVs GLASGOW GB (1)
Cichocki N (2)	UNIVs GLASGOW GB (1)
Combet E (2)	UNIVs GLASGOW GB (2)
Crozier A (3)	UNIVs GLASGOW GB (3)
Edwards Ca (5)	UNIVs GLASGOW GB (4)
Yalcinkaya N (1)	UNIVs GOTHENBURG SE (1)
Wood J (1)	UNIVs GOTHENBURG SE (1)
Vetrani C (1)	UNIVs GOTHENBURG SE (1)
Veide J (1)	UNIVs GOTHENBURG SE (1)
Vegarud G (2)	UNIVs GOTHENBURG SE (1)
Urvil P (1)	UNIVs GOTHENBURG SE (1)
Tullberg C (1)	UNIVs GOTHENBURG SE (1)
Swaminathan M (1)	UNIVs GOTHENBURG SE (1)
Sjögren J (1)	UNIVs GOTHENBURG SE (1)
Seger Go (1)	UNIVs GOTHENBURG SE (1)
Schütte A (1)	UNIVs GOTHENBURG SE (1)
Schroeder Bo (1)	UNIVs GOTHENBURG SE (1)
Scheers N (1)	UNIVs GOTHENBURG SE (1)
Rivellese Aa (1)	UNIVs GOTHENBURG SE (1)
Råstam M (1)	UNIVs GOTHENBURG SE (1)
Ramalhosa F (1)	UNIVs GOTHENBURG SE (1)
Mckeown Sj (1)	UNIVs GOTHENBURG SE (1)
Larsson A (1)	UNIVs GOTHENBURG SE (1)
Katona G (1)	UNIVs GOTHENBURG SE (1)
Johansson Mev (1)	UNIVs GOTHENBURG SE (1)
Hosie S (1)	UNIVs GOTHENBURG SE (1)
Hill-yardin El (1)	UNIVs GOTHENBURG SE (1)
Haraldsson A-k (1)	UNIVs GOTHENBURG SE (1)
Hansson Gc (1)	UNIVs GOTHENBURG SE (1)
Gillberg C (1)	UNIVs GOTHENBURG SE (1)
Franks Ae (1)	UNIVs GOTHENBURG SE (1)
Ermund A (1)	UNIVs GOTHENBURG SE (1)
Ellis M (1)	UNIVs GOTHENBURG SE (1)
Conway Pl (1)	UNIVs GOTHENBURG SE (1)
Churilov L (1)	UNIVs GOTHENBURG SE (1)
Carlsson N-g (1)	UNIVs GOTHENBURG SE (1)
Bornstein Jc (1)	UNIVs GOTHENBURG SE (1)
Borén J (1)	UNIVs GOTHENBURG SE (1)
Bodin O (1)	UNIVs GOTHENBURG SE (1)

Birchenough Gmh (1)	UNIVs GOTHENBURG SE (1)
Bergström Jh (1)	UNIVs GOTHENBURG SE (1)
Bell Ca (1)	UNIVs GOTHENBURG SE (1)
Balasuriya Gk (1)	UNIVs GOTHENBURG SE (1)
Bake B (1)	UNIVs GOTHENBURG SE (1)
Annuzzi G (1)	UNIVs GOTHENBURG SE (1)
Andlid T (1)	UNIVs GOTHENBURG SE (1)
Alpsten M (1)	UNIVs GOTHENBURG SE (1)
Alminger Ml (1)	UNIVs GOTHENBURG SE (1)
Adiels M (1)	UNIVs GOTHENBURG SE (1)
Undeland I (2)	UNIVs GOTHENBURG SE (2)
Sandberg A-s (2)	UNIVs GOTHENBURG SE (2)
Larsson K (2)	UNIVs GOTHENBURG SE (2)
Alminger M (3)	UNIVs GOTHENBURG SE (3)
Torres Jc (1)	UNIVs GRANADA ES (1)
Segura-carretero A (1)	UNIVs GRANADA ES (1)
Ruíz-pérez S (1)	UNIVs GRANADA ES (1)
Ruiz Lm (1)	UNIVs GRANADA ES (1)
Rodríguez-calvo A (1)	UNIVs GRANADA ES (1)
Robert P (1)	UNIVs GRANADA ES (1)
Rivas-montoya E (1)	UNIVs GRANADA ES (1)
Poyatos Jm (1)	UNIVs GRANADA ES (1)
Pastoriza de la cueva S (1)	UNIVs GRANADA ES (1)
Parada J (2)	UNIVs GRANADA ES (1)
Paliy O (1)	UNIVs GRANADA ES (1)
Miguel ochando-pulido J (1)	UNIVs GRANADA ES (1)
Mehta T (1)	UNIVs GRANADA ES (1)
Martin-pascual J (1)	UNIVs GRANADA ES (1)
Maqueda M (1)	UNIVs GRANADA ES (1)
Manuel lópez-romero J (1)	UNIVs GRANADA ES (1)
Maldonado-mateus Ly (1)	UNIVs GRANADA ES (1)
Lopez-lopez C (1)	UNIVs GRANADA ES (1)
González E (1)	UNIVs GRANADA ES (1)
Gómez-nieto Má (1)	UNIVs GRANADA ES (1)
Gómez-caravaca Am (1)	UNIVs GRANADA ES (1)
Giovando S (1)	UNIVs GRANADA ES (1)
Giménez B (1)	UNIVs GRANADA ES (1)
Fernández-miyakawa M (1)	UNIVs GRANADA ES (1)
Esteban-muñoz A (1)	UNIVs GRANADA ES (1)
D'auria G (1)	UNIVs GRANADA ES (1)
Cebrián R (1)	UNIVs GRANADA ES (1)
Calero-diaz G (1)	UNIVs GRANADA ES (1)
Ángel rufián-henares J (1)	UNIVs GRANADA ES (1)
Santagapita Pr (2)	UNIVs GRANADA ES (2)
Rodriguez-sanchez A (2)	UNIVs GRANADA ES (2)
Perullini M (2)	UNIVs GRANADA ES (2)
Muñoz-palazon B (2)	UNIVs GRANADA ES (2)
Martínez-férez A (2)	UNIVs GRANADA ES (2)
Lerma-aguilera A (2)	UNIVs GRANADA ES (2)
Jiménez-hernández N (3)	UNIVs GRANADA ES (2)
Hinojosa-nogueira D (2)	UNIVs GRANADA ES (2)
Gosalbes Mj (3)	UNIVs GRANADA ES (2)
Gonzalez-lopez J (2)	UNIVs GRANADA ES (2)
Aguirre-calvo Tr (2)	UNIVs GRANADA ES (2)
Francino Mp (3)	UNIVs GRANADA ES (3)
Pastoriza S (4)	UNIVs GRANADA ES (4)
Molino S (4)	UNIVs GRANADA ES (4)
Perez-burillo S (5)	UNIVs GRANADA ES (5)
Rufián-henares Ja (8)	UNIVs GRANADA ES (8)
Schick P (1)	UNIVs GREIFSWALD DE (1)
Krause J (1)	UNIVs GREIFSWALD DE (1)
Glöckl G (1)	UNIVs GREIFSWALD DE (1)
Beeck R (1)	UNIVs GREIFSWALD DE (1)
Weitschies W (3)	UNIVs GREIFSWALD DE (3)
Zou Q (1)	UNIVs GUANGDONG CN (1)

Zhou Q-y (1)	UNIVs GUANGDONG CN (1)
Zheng J (2)	UNIVs GUANGDONG CN (1)
Yu J-j (1)	UNIVs GUANGDONG CN (1)
Yin H (1)	UNIVs GUANGDONG CN (1)
Yang Z (2)	UNIVs GUANGDONG CN (1)
Yan S (1)	UNIVs GUANGDONG CN (1)
Yan R (1)	UNIVs GUANGDONG CN (1)
Xue B (1)	UNIVs GUANGDONG CN (1)
Xiao X (1)	UNIVs GUANGDONG CN (1)
Wu Z (2)	UNIVs GUANGDONG CN (1)
Wu R (1)	UNIVs GUANGDONG CN (1)
Wong M-h (1)	UNIVs GUANGDONG CN (1)
Wong Ckc (1)	UNIVs GUANGDONG CN (1)
White Jc (1)	UNIVs GUANGDONG CN (1)
Wei W (1)	UNIVs GUANGDONG CN (1)
Wang P-p (1)	UNIVs GUANGDONG CN (1)
Wang H-s (1)	UNIVs GUANGDONG CN (1)
Wan L (1)	UNIVs GUANGDONG CN (1)
Tang X (1)	UNIVs GUANGDONG CN (1)
Tan L (2)	UNIVs GUANGDONG CN (1)
Shirtliff Me (1)	UNIVs GUANGDONG CN (1)
Shang H (1)	UNIVs GUANGDONG CN (1)
Ren Q (1)	UNIVs GUANGDONG CN (1)
Peng X-c (1)	UNIVs GUANGDONG CN (1)
Peng X (2)	UNIVs GUANGDONG CN (1)
Omedi Jo (1)	UNIVs GUANGDONG CN (1)
Man Y-b (1)	UNIVs GUANGDONG CN (1)
Ma F (1)	UNIVs GUANGDONG CN (1)
Ma C (2)	UNIVs GUANGDONG CN (1)
Luo Z-g (1)	UNIVs GUANGDONG CN (1)
Lu G (2)	UNIVs GUANGDONG CN (1)
Liu D-m (1)	UNIVs GUANGDONG CN (1)
Li S (3)	UNIVs GUANGDONG CN (1)
Li L-q (1)	UNIVs GUANGDONG CN (1)
Li B (3)	UNIVs GUANGDONG CN (1)
Lam K-l (1)	UNIVs GUANGDONG CN (1)
Kwan H-s (1)	UNIVs GUANGDONG CN (1)
Kulikouskaya V (1)	UNIVs GUANGDONG CN (1)
Kuang J-h (1)	UNIVs GUANGDONG CN (1)
Ko K-c (1)	UNIVs GUANGDONG CN (1)
Ke X (1)	UNIVs GUANGDONG CN (1)
Kang X (1)	UNIVs GUANGDONG CN (1)
Jin L (1)	UNIVs GUANGDONG CN (1)
Jin H (1)	UNIVs GUANGDONG CN (1)
Jiang Y (2)	UNIVs GUANGDONG CN (1)
Huang Y-y (1)	UNIVs GUANGDONG CN (1)
Hu J-s (1)	UNIVs GUANGDONG CN (1)
Hoag Sw (1)	UNIVs GUANGDONG CN (1)
Hileuskaya K (1)	UNIVs GUANGDONG CN (1)
He Y (2)	UNIVs GUANGDONG CN (1)
Harro Jm (1)	UNIVs GUANGDONG CN (1)
Giesy Jp (1)	UNIVs GUANGDONG CN (1)
Du J (1)	UNIVs GUANGDONG CN (1)
Dou Z (1)	UNIVs GUANGDONG CN (1)
Di T (1)	UNIVs GUANGDONG CN (1)
Dang Z (1)	UNIVs GUANGDONG CN (1)
Chi-keung cheung P (1)	UNIVs GUANGDONG CN (1)
Cheng W-y (1)	UNIVs GUANGDONG CN (1)
Cheng M (1)	UNIVs GUANGDONG CN (1)
Chen Z-j (1)	UNIVs GUANGDONG CN (1)
Chen G (1)	UNIVs GUANGDONG CN (1)
Zhang G (2)	UNIVs GUANGDONG CN (2)
Zhang B (5)	UNIVs GUANGDONG CN (2)
You L (2)	UNIVs GUANGDONG CN (2)
Xie X (3)	UNIVs GUANGDONG CN (2)

Sun Y (6)	UNIVs GUANGDONG CN (2)
Ou S (2)	UNIVs GUANGDONG CN (2)
Li C (10)	UNIVs GUANGDONG CN (2)
Huang W (2)	UNIVs GUANGDONG CN (2)
Fu X (3)	UNIVs GUANGDONG CN (3)
Yu T (1)	UNIVs GUANGXI CN (1)
Liang X-l (1)	UNIVs GUANGXI CN (1)
Zhu Z-j (2)	UNIVs GUANGXI CN (2)
Liang A-h (2)	UNIVs GUANGXI CN (2)
Li Q-y (2)	UNIVs GUANGXI CN (2)
Huang G-h (2)	UNIVs GUANGXI CN (2)
Cai D (2)	UNIVs GUANGXI CN (2)
Zheng Q (1)	UNIVs HAINAN CN (1)
Yuan Y (1)	UNIVs HAINAN CN (1)
Yao S (1)	UNIVs HAINAN CN (1)
Xu F (1)	UNIVs HAINAN CN (1)
Shen X (1)	UNIVs HAINAN CN (1)
Lasch J (1)	UNIVs HALLE-WITTENBERG DE (1)
Kretschmer K (1)	UNIVs HALLE-WITTENBERG DE (1)
Koelsch R (1)	UNIVs HALLE-WITTENBERG DE (1)
Zeng A-p (1)	UNIVs HAMBURG DE (1)
Sahm K (1)	UNIVs HAMBURG DE (1)
Sabra W (1)	UNIVs HAMBURG DE (1)
Röske I (1)	UNIVs HAMBURG DE (1)
Nacke H (1)	UNIVs HAMBURG DE (1)
Daniel R (1)	UNIVs HAMBURG DE (1)
Antranikian G (1)	UNIVs HAMBURG DE (1)
Zuo Yy (1)	UNIVs HAWAII US (1)
Zijlstra Rt (1)	UNIVs HAWAII US (1)
Yan T (1)	UNIVs HAWAII US (1)
Tiwari Up (1)	UNIVs HAWAII US (1)
Pagaling E (1)	UNIVs HAWAII US (1)
Kim Sw (1)	UNIVs HAWAII US (1)
Kim L (1)	UNIVs HAWAII US (1)
Jha R (2)	UNIVs HAWAII US (2)
Shi J (3)	UNIVs HEBEI CN (1)
Pang M (1)	UNIVs HEBEI CN (1)
Gao Y (1)	UNIVs HEBEI CN (1)
Duan Y (1)	UNIVs HEBEI CN (1)
Xue Y (3)	UNIVs HEBEI CN (2)
Qi H (2)	UNIVs HEBEI CN (2)
Lin H (2)	UNIVs HEBEI CN (2)
Das R (2)	UNIVs HEBEI CN (2)
Wang Q (4)	UNIVs HEBEI CN (3)
Mao D (3)	UNIVs HEBEI CN (3)
Luo Y (7)	UNIVs HEBEI CN (3)
Zhu C-l (1)	UNIVs HEILONGJIANG CN (1)
Xu Z (4)	UNIVs HEILONGJIANG CN (1)
Xu H (3)	UNIVs HEILONGJIANG CN (1)
Sui X (1)	UNIVs HEILONGJIANG CN (1)
Qi B (1)	UNIVs HEILONGJIANG CN (1)
Massounga bora Af (1)	UNIVs HEILONGJIANG CN (1)
Kuerman M (1)	UNIVs HEILONGJIANG CN (1)
Kong L (1)	UNIVs HEILONGJIANG CN (1)
Jiang L (3)	UNIVs HEILONGJIANG CN (1)
Hao L (1)	UNIVs HEILONGJIANG CN (1)
Han H (1)	UNIVs HEILONGJIANG CN (1)
Guo M (2)	UNIVs HEILONGJIANG CN (1)
Geng Q (1)	UNIVs HEILONGJIANG CN (1)
Du P (1)	UNIVs HEILONGJIANG CN (1)
Du L (2)	UNIVs HEILONGJIANG CN (1)
Cui S (2)	UNIVs HEILONGJIANG CN (1)
Bao Y (1)	UNIVs HEILONGJIANG CN (1)
Zhao X-h (3)	UNIVs HEILONGJIANG CN (3)
Vickers R (1)	UNIVs HERTFORDSHIRE GB (1)

Todhunter S (1)	UNIVs HERTFORDSHIRE GB (1)
Locher Hh (1)	UNIVs HERTFORDSHIRE GB (1)
Fawley Wn (1)	UNIVs HERTFORDSHIRE GB (1)
Athanasiou A (1)	UNIVs HERTFORDSHIRE GB (1)
Schollenberger M (1)	UNIVs HOHENHEIM DE (1)
Rodehutscord M (1)	UNIVs HOHENHEIM DE (1)
Greiner R (1)	UNIVs HOHENHEIM DE (1)
Briviba K (2)	UNIVs HOHENHEIM DE (1)
Toyohara K (1)	UNIVs HOKKAIDO JP (1)
Pelpolage Sw (1)	UNIVs HOKKAIDO JP (1)
Pelpolage S (1)	UNIVs HOKKAIDO JP (1)
Nakayama Y (1)	UNIVs HOKKAIDO JP (1)
Nagata R (1)	UNIVs HOKKAIDO JP (1)
Koaze H (1)	UNIVs HOKKAIDO JP (1)
Kitazono E (1)	UNIVs HOKKAIDO JP (1)
Kilua A (1)	UNIVs HOKKAIDO JP (1)
Hoshizawa M (1)	UNIVs HOKKAIDO JP (1)
Han K-h (1)	UNIVs HOKKAIDO JP (1)
Han K (1)	UNIVs HOKKAIDO JP (1)
Hamamoto T (1)	UNIVs HOKKAIDO JP (1)
Goto A (1)	UNIVs HOKKAIDO JP (1)
Fukuma N (1)	UNIVs HOKKAIDO JP (1)
Fukushima M (2)	UNIVs HOKKAIDO JP (2)
Ye L (2)	UNIVs HONG KONG CN (1)
Wei Q (1)	UNIVs HONG KONG CN (1)
Tong Ahy (1)	UNIVs HONG KONG CN (1)
Shao M-f (1)	UNIVs HONG KONG CN (1)
Rodriquez Dc (1)	UNIVs HONG KONG CN (1)
Ooi Ybh (1)	UNIVs HONG KONG CN (1)
Lok S (1)	UNIVs HONG KONG CN (1)
Lim Sf (1)	UNIVs HONG KONG CN (1)
Kang Q (1)	UNIVs HONG KONG CN (1)
Hu P (1)	UNIVs HONG KONG CN (1)
He J (1)	UNIVs HONG KONG CN (1)
Graham Dw (1)	UNIVs HONG KONG CN (1)
Dianawati D (1)	UNIVs HONG KONG CN (1)
Deng K (1)	UNIVs HONG KONG CN (1)
Christgen B (1)	UNIVs HONG KONG CN (1)
Ahammad Sz (1)	UNIVs HONG KONG CN (1)
Zhang T (5)	UNIVs HONG KONG CN (2)
Chen T (3)	UNIVs HONG KONG CN (2)
Shah Np (3)	UNIVs HONG KONG CN (3)
Zhou S (1)	UNIVs HUBEI CN (1)
Zheng D-w (1)	UNIVs HUBEI CN (1)
Zhang X-z (1)	UNIVs HUBEI CN (1)
Yao J (1)	UNIVs HUBEI CN (1)
Xu R (1)	UNIVs HUBEI CN (1)
Xiong L-y (1)	UNIVs HUBEI CN (1)
Xie T-q (1)	UNIVs HUBEI CN (1)
Xiao P (1)	UNIVs HUBEI CN (1)
Wei Y (1)	UNIVs HUBEI CN (1)
Vidovic Nk (1)	UNIVs HUBEI CN (1)
Sun J-m (1)	UNIVs HUBEI CN (1)
Qu F (1)	UNIVs HUBEI CN (1)
Qiu Y (1)	UNIVs HUBEI CN (1)
Peng D (1)	UNIVs HUBEI CN (1)
Ni D (1)	UNIVs HUBEI CN (1)
Li R-q (1)	UNIVs HUBEI CN (1)
Huang F (3)	UNIVs HUBEI CN (1)
Han Z-y (1)	UNIVs HUBEI CN (1)
Guo W (1)	UNIVs HUBEI CN (1)
Guo L (1)	UNIVs HUBEI CN (1)
Feng X-w (1)	UNIVs HUBEI CN (1)
Fang Y (1)	UNIVs HUBEI CN (1)
Fang D (1)	UNIVs HUBEI CN (1)

Dong L (4)	UNIVs HUBEI CN (1)
Ding W-p (1)	UNIVs HUBEI CN (1)
Deng Y (1)	UNIVs HUBEI CN (1)
Chen D (2)	UNIVs HUBEI CN (1)
An J-x (1)	UNIVs HUBEI CN (1)
Ai Z (1)	UNIVs HUBEI CN (1)
Iqbal Z (2)	UNIVs HUBEI CN (2)
Huang L (2)	UNIVs HUBEI CN (2)
Cheng G (2)	UNIVs HUBEI CN (2)
Yuan Z (3)	UNIVs HUBEI CN (3)
Liu Z (11)	UNIVs HUBEI CN (3)
Hao H (3)	UNIVs HUBEI CN (3)
Dai M (3)	UNIVs HUBEI CN (3)
Wang Y (17)	UNIVs HUBEI CN (4)
Zhang Z-x (1)	UNIVs HUNAN CN (1)
Lyu R (1)	UNIVs HUNAN CN (1)
Luo Y-s (1)	UNIVs HUNAN CN (1)
Liu S-f (1)	UNIVs HUNAN CN (1)
Liu D-b (1)	UNIVs HUNAN CN (1)
Kang X-c (1)	UNIVs HUNAN CN (1)
Gu Q (1)	UNIVs HUNAN CN (1)
Gao Z (1)	UNIVs HUNAN CN (1)
Sasaki D (7)	UNIVs HYOGO JP (7)
Uemura K (1)	UNIVs IBARAKI JP (1)
Sato S (1)	UNIVs IBARAKI JP (1)
Neves Ma (1)	UNIVs IBARAKI JP (1)
Nakata Y (1)	UNIVs IBARAKI JP (1)
Nakajima M (1)	UNIVs IBARAKI JP (1)
Kozu H (1)	UNIVs IBARAKI JP (1)
Kobayashi I (1)	UNIVs IBARAKI JP (1)
Ichikawa S (1)	UNIVs IBARAKI JP (1)
Van leuven Jt (1)	UNIVs IDAHO US (1)
Vallender Ej (1)	UNIVs IDAHO US (1)
Toczylowska-maminska R (1)	UNIVs IDAHO US (1)
Tardif Sd (1)	UNIVs IDAHO US (1)
Szymona K (1)	UNIVs IDAHO US (1)
Schultz-darken N (1)	UNIVs IDAHO US (1)
Ross Cn (1)	UNIVs IDAHO US (1)
Pielech-przybylska K (1)	UNIVs IDAHO US (1)
Oswald Bp (1)	UNIVs IDAHO US (1)
Mtui D (1)	UNIVs IDAHO US (1)
Logan Be (1)	UNIVs IDAHO US (1)
Król P (1)	UNIVs IDAHO US (1)
Kloch M (1)	UNIVs IDAHO US (1)
Gliniewicz K (1)	UNIVs IDAHO US (1)
Forney Lj (1)	UNIVs IDAHO US (1)
Brown Cj (1)	UNIVs IDAHO US (1)
Austad Sn (1)	UNIVs IDAHO US (1)
Wilson Fp (1)	UNIVs ILLINOIS US (1)
Wendy lou Wy (1)	UNIVs ILLINOIS US (1)
Vittori N (1)	UNIVs ILLINOIS US (1)
Turvey Se (1)	UNIVs ILLINOIS US (1)
To T (1)	UNIVs ILLINOIS US (1)
Tebbutt S (1)	UNIVs ILLINOIS US (1)
Tang P (1)	UNIVs ILLINOIS US (1)
Takaro Tk (1)	UNIVs ILLINOIS US (1)
Subbarao P (1)	UNIVs ILLINOIS US (1)
Simons E (1)	UNIVs ILLINOIS US (1)
Silverman F (1)	UNIVs ILLINOIS US (1)
Shi Y-c (1)	UNIVs ILLINOIS US (1)
Sharma Ak (1)	UNIVs ILLINOIS US (1)
Sears Mr (1)	UNIVs ILLINOIS US (1)
Scott Ja (1)	UNIVs ILLINOIS US (1)
Scott J (1)	UNIVs ILLINOIS US (1)
Sandford A (1)	UNIVs ILLINOIS US (1)

Rossoni serao Mc (1)	UNIVs ILLINOIS US (1)
Ratjen F (1)	UNIVs ILLINOIS US (1)
Ramsey C (1)	UNIVs ILLINOIS US (1)
Priyadarshini M (1)	UNIVs ILLINOIS US (1)
Paré Pd (1)	UNIVs ILLINOIS US (1)
Panasevich Mr (1)	UNIVs ILLINOIS US (1)
Moraes Tj (1)	UNIVs ILLINOIS US (1)
Moqbel R (1)	UNIVs ILLINOIS US (1)
Miller Mj (1)	UNIVs ILLINOIS US (1)
Miller Ge (1)	UNIVs ILLINOIS US (1)
Mei R (1)	UNIVs ILLINOIS US (1)
Mandhane Pj (1)	UNIVs ILLINOIS US (1)
Macri J (1)	UNIVs ILLINOIS US (1)
Lynch Gl (1)	UNIVs ILLINOIS US (1)
Liu W-t (1)	UNIVs ILLINOIS US (1)
Lefebvre Dl (1)	UNIVs ILLINOIS US (1)
Lee Y (2)	UNIVs ILLINOIS US (1)
Layden Bt (1)	UNIVs ILLINOIS US (1)
Larché M (1)	UNIVs ILLINOIS US (1)
Laprise C (1)	UNIVs ILLINOIS US (1)
Kozyrskyj Al (1)	UNIVs ILLINOIS US (1)
Kotlo Ku (1)	UNIVs ILLINOIS US (1)
Kollmann Tr (1)	UNIVs ILLINOIS US (1)
Kobor Ms (1)	UNIVs ILLINOIS US (1)
Kim J (1)	UNIVs ILLINOIS US (1)
Kerr Kr (1)	UNIVs ILLINOIS US (1)
Holness Dl (1)	UNIVs ILLINOIS US (1)
Hoeflinger Jl (1)	UNIVs ILLINOIS US (1)
Herkenhoff Me (1)	UNIVs ILLINOIS US (1)
Hegele R (1)	UNIVs ILLINOIS US (1)
Hayglass Kt (1)	UNIVs ILLINOIS US (1)
Grasemann H (1)	UNIVs ILLINOIS US (1)
Elliott S (1)	UNIVs ILLINOIS US (1)
Dudeja Pk (1)	UNIVs ILLINOIS US (1)
Dowd Se (1)	UNIVs ILLINOIS US (1)
Donovan Jd (1)	UNIVs ILLINOIS US (1)
Dolinsky Vw (1)	UNIVs ILLINOIS US (1)
Dilger Rn (1)	UNIVs ILLINOIS US (1)
Deplancke B (1)	UNIVs ILLINOIS US (1)
Denburg Ja (1)	UNIVs ILLINOIS US (1)
Dell S (1)	UNIVs ILLINOIS US (1)
De souza Rj (1)	UNIVs ILLINOIS US (1)
de souza leite M (1)	UNIVs ILLINOIS US (1)
de godoy Mrc (1)	UNIVs ILLINOIS US (1)
Daley D (1)	UNIVs ILLINOIS US (1)
Cyr M (1)	UNIVs ILLINOIS US (1)
Chen E (1)	UNIVs ILLINOIS US (1)
Canadian healthy infant longitudinal development s (1)	UNIVs ILLINOIS US (1)
Brook Jr (1)	UNIVs ILLINOIS US (1)
Brauer M (1)	UNIVs ILLINOIS US (1)
Bocher Btw (1)	UNIVs ILLINOIS US (1)
Befus Ad (1)	UNIVs ILLINOIS US (1)
Becker Ab (1)	UNIVs ILLINOIS US (1)
Bauer L (1)	UNIVs ILLINOIS US (1)
Azad Mb (1)	UNIVs ILLINOIS US (1)
Anand S (1)	UNIVs ILLINOIS US (1)
Allen R (1)	UNIVs ILLINOIS US (1)
Bauer Ll (2)	UNIVs ILLINOIS US (2)
Swanson Ks (3)	UNIVs ILLINOIS US (3)
Fahey Gc (3)	UNIVs ILLINOIS US (3)
Santerre Cr (1)	UNIVs INDIANA US (1)
Oonsivilai R (1)	UNIVs INDIANA US (1)
Lin Ah-m (1)	UNIVs INDIANA US (1)
Lee B-h (1)	UNIVs INDIANA US (1)

Kim Cy (1)	UNIVs INDIANA US (1)
Kachenpukdee N (1)	UNIVs INDIANA US (1)
Ferruzzi Mg (1)	UNIVs INDIANA US (1)
Colantuono A (1)	UNIVs INDIANA US (1)
Campanella Oh (1)	UNIVs INDIANA US (1)
Bello-pérez La (1)	UNIVs INDIANA US (1)
Arioglu-tuncil S (1)	UNIVs INDIANA US (1)
Tuncil Ye (2)	UNIVs INDIANA US (2)
Thakkar Rd (2)	UNIVs INDIANA US (2)
Lindemann Sr (2)	UNIVs INDIANA US (2)
Hamaker Br (4)	UNIVs INDIANA US (4)
Zhong Z (1)	UNIVs INNER MONGOLIA CN (1)
Zhang Hp (1)	UNIVs INNER MONGOLIA CN (1)
Wei A (1)	UNIVs INNER MONGOLIA CN (1)
Wang Xn (1)	UNIVs INNER MONGOLIA CN (1)
Sun Ts (1)	UNIVs INNER MONGOLIA CN (1)
Sun Cl (1)	UNIVs INNER MONGOLIA CN (1)
Menghebilige (1)	UNIVs INNER MONGOLIA CN (1)
Meng H (1)	UNIVs INNER MONGOLIA CN (1)
Liu Wj (1)	UNIVs INNER MONGOLIA CN (1)
Li Mh (1)	UNIVs INNER MONGOLIA CN (1)
Kwok Ly (1)	UNIVs INNER MONGOLIA CN (1)
Dan T (1)	UNIVs INNER MONGOLIA CN (1)
Chen Yf (1)	UNIVs INNER MONGOLIA CN (1)
Bao Q (1)	UNIVs INNER MONGOLIA CN (1)
Viotti P (1)	UNIVs INSUBRIA IT (1)
Rada Ec (1)	UNIVs INSUBRIA IT (1)
Raboni M (1)	UNIVs INSUBRIA IT (1)
Conti F (1)	UNIVs INSUBRIA IT (1)
White Pj (1)	UNIVs IOWA US (1)
Villageliú Dn (1)	UNIVs IOWA US (1)
Rasmussen S (1)	UNIVs IOWA US (1)
Lyte M (1)	UNIVs IOWA US (1)
Kim Hj (1)	UNIVs IOWA US (1)
Clark S (1)	UNIVs IOWA US (1)
Bollom Ma (1)	UNIVs IOWA US (1)
Acevedo Nc (1)	UNIVs IOWA US (1)
Ruiz-riaguas A (1)	UNIVs JAEN ES (1)
Ruiz-medina A (1)	UNIVs JAEN ES (1)
Ortega-vidal J (1)	UNIVs JAEN ES (1)
Ortega-barrales P (1)	UNIVs JAEN ES (1)
Fernández-poyatos Mp (1)	UNIVs JAEN ES (1)
Fernández-de córdova Ml (1)	UNIVs JAEN ES (1)
Llorent-martínez Ej (2)	UNIVs JAEN ES (2)
Zetzmann S (1)	UNIVs JENA DE (1)
Wollny T (1)	UNIVs JENA DE (1)
Wissenbach Dk (1)	UNIVs JENA DE (1)
Westermann M (1)	UNIVs JENA DE (1)
Verdu Ef (2)	UNIVs JENA DE (1)
Siwczak F (1)	UNIVs JENA DE (1)
Scharlau D (1)	UNIVs JENA DE (1)
Raasch M (1)	UNIVs JENA DE (1)
Pospich R (1)	UNIVs JENA DE (1)
Nietzsche S (1)	UNIVs JENA DE (1)
Medyukhina A (1)	UNIVs JENA DE (1)
Maurer M (1)	UNIVs JENA DE (1)
Lux S (1)	UNIVs JENA DE (1)
Linde S (1)	UNIVs JENA DE (1)
Jacobsen Id (1)	UNIVs JENA DE (1)
Huber O (1)	UNIVs JENA DE (1)
Höke H (1)	UNIVs JENA DE (1)
Haange Sb (1)	UNIVs JENA DE (1)
Gratz R (1)	UNIVs JENA DE (1)
Figge Mt (1)	UNIVs JENA DE (1)
Cseresnyes Z (1)	UNIVs JENA DE (1)

Birringer M (1)	UNIVs JENA DE (1)
Berlinghof F (1)	UNIVs JENA DE (1)
Baumann S (1)	UNIVs JENA DE (1)
Atanasov J (1)	UNIVs JENA DE (1)
Allert S (1)	UNIVs JENA DE (1)
Mosig As (2)	UNIVs JENA DE (2)
Lorkowski S (2)	UNIVs JENA DE (2)
Hube B (2)	UNIVs JENA DE (2)
Gröger M (2)	UNIVs JENA DE (2)
Gresnigt Ms (2)	UNIVs JENA DE (2)
Dawczynski C (2)	UNIVs JENA DE (2)
Schlörmann W (3)	UNIVs JENA DE (3)
Glei M (3)	UNIVs JENA DE (3)
Zhou X (3)	UNIVs JIANGSU CN (1)
Zhou M (1)	UNIVs JIANGSU CN (1)
Zhou G (1)	UNIVs JIANGSU CN (1)
Zheng Z (1)	UNIVs JIANGSU CN (1)
Zhao Z-c (1)	UNIVs JIANGSU CN (1)
Zhao Z (1)	UNIVs JIANGSU CN (1)
Zhao R (1)	UNIVs JIANGSU CN (1)
Zhao E (1)	UNIVs JIANGSU CN (1)
Zhang K (1)	UNIVs JIANGSU CN (1)
Zhan X-b (1)	UNIVs JIANGSU CN (1)
Yun jiang (1)	UNIVs JIANGSU CN (1)
Yu X (1)	UNIVs JIANGSU CN (1)
Yu J (1)	UNIVs JIANGSU CN (1)
Yong Q (1)	UNIVs JIANGSU CN (1)
Yang C (3)	UNIVs JIANGSU CN (1)
Yang A (1)	UNIVs JIANGSU CN (1)
Yan L (1)	UNIVs JIANGSU CN (1)
Xue Z (1)	UNIVs JIANGSU CN (1)
Xu X (2)	UNIVs JIANGSU CN (1)
Xing G (1)	UNIVs JIANGSU CN (1)
Xie M-y (1)	UNIVs JIANGSU CN (1)
Xie M (2)	UNIVs JIANGSU CN (1)
Xie G (1)	UNIVs JIANGSU CN (1)
Xiao Y (1)	UNIVs JIANGSU CN (1)
Xiang X (1)	UNIVs JIANGSU CN (1)
Wu Y (3)	UNIVs JIANGSU CN (1)
Wang D (3)	UNIVs JIANGSU CN (1)
Tong P (1)	UNIVs JIANGSU CN (1)
Tang W (1)	UNIVs JIANGSU CN (1)
Sun Z (1)	UNIVs JIANGSU CN (1)
Show Pl (1)	UNIVs JIANGSU CN (1)
Shang K (1)	UNIVs JIANGSU CN (1)
Rui M (1)	UNIVs JIANGSU CN (1)
Ren H-q (1)	UNIVs JIANGSU CN (1)
Qing S (1)	UNIVs JIANGSU CN (1)
Qian H (1)	UNIVs JIANGSU CN (1)
Qi X (1)	UNIVs JIANGSU CN (1)
Pei F (1)	UNIVs JIANGSU CN (1)
Nie Y (1)	UNIVs JIANGSU CN (1)
Nie S-p (1)	UNIVs JIANGSU CN (1)
Ni Y (1)	UNIVs JIANGSU CN (1)
Muinde kimatu B (1)	UNIVs JIANGSU CN (1)
Luo B (1)	UNIVs JIANGSU CN (1)
Liu M (2)	UNIVs JIANGSU CN (1)
Liao N (1)	UNIVs JIANGSU CN (1)
Li Z-t (1)	UNIVs JIANGSU CN (1)
Li T (1)	UNIVs JIANGSU CN (1)
Li G (1)	UNIVs JIANGSU CN (1)
Jiang M (1)	UNIVs JIANGSU CN (1)
Ji Y (2)	UNIVs JIANGSU CN (1)
Huang M (1)	UNIVs JIANGSU CN (1)
Hu J-l (1)	UNIVs JIANGSU CN (1)

Hu G-a (1)	UNIVs JIANGSU CN (1)
Hu G (1)	UNIVs JIANGSU CN (1)
Hu B (2)	UNIVs JIANGSU CN (1)
He Z (2)	UNIVs JIANGSU CN (1)
Han Y (1)	UNIVs JIANGSU CN (1)
Guan Y (2)	UNIVs JIANGSU CN (1)
Gao M (1)	UNIVs JIANGSU CN (1)
Fan X (1)	UNIVs JIANGSU CN (1)
Fan M (1)	UNIVs JIANGSU CN (1)
Deng H (1)	UNIVs JIANGSU CN (1)
Dai R (1)	UNIVs JIANGSU CN (1)
Cheng S (1)	UNIVs JIANGSU CN (1)
Bai J (1)	UNIVs JIANGSU CN (1)
Zhang X-x (2)	UNIVs JIANGSU CN (2)
Zhang W (3)	UNIVs JIANGSU CN (2)
Zeng X (3)	UNIVs JIANGSU CN (2)
Yu Q (2)	UNIVs JIANGSU CN (2)
Ye H (2)	UNIVs JIANGSU CN (2)
Yang W (2)	UNIVs JIANGSU CN (2)
Xu Q (2)	UNIVs JIANGSU CN (2)
Xu L (2)	UNIVs JIANGSU CN (2)
Xie J (4)	UNIVs JIANGSU CN (2)
Wu J (5)	UNIVs JIANGSU CN (2)
Wu B (2)	UNIVs JIANGSU CN (2)
Wang M (7)	UNIVs JIANGSU CN (2)
Wang F (6)	UNIVs JIANGSU CN (2)
Tian F (2)	UNIVs JIANGSU CN (2)
Tao Y (3)	UNIVs JIANGSU CN (2)
Su A (2)	UNIVs JIANGSU CN (2)
Shishir Mri (3)	UNIVs JIANGSU CN (2)
Ma G (2)	UNIVs JIANGSU CN (2)
Gao M-j (2)	UNIVs JIANGSU CN (2)
Dong R (2)	UNIVs JIANGSU CN (2)
Cheng K-w (2)	UNIVs JIANGSU CN (2)
Azarpazhooh E (2)	UNIVs JIANGSU CN (2)
Zhang Q (5)	UNIVs JIANGSU CN (3)
Zhan X (3)	UNIVs JIANGSU CN (3)
Wang Z (6)	UNIVs JIANGSU CN (3)
Li W (5)	UNIVs JIANGSU CN (3)
Hu Q (3)	UNIVs JIANGSU CN (3)
Zhu L (10)	UNIVs JIANGSU CN (4)
Liu S (7)	UNIVs JIANGSU CN (4)
Dong M (4)	UNIVs JIANGSU CN (4)
Rui X (5)	UNIVs JIANGSU CN (5)
Xiao L (1)	UNIVs JIANGXI CN (1)
Xia Y (1)	UNIVs JIANGXI CN (1)
Shi X (1)	UNIVs JIANGXI CN (1)
Nie Q (1)	UNIVs JIANGXI CN (1)
He H (1)	UNIVs JIANGXI CN (1)
Guo F (2)	UNIVs JIANGXI CN (1)
Geng F (1)	UNIVs JIANGXI CN (1)
Cheng K (2)	UNIVs JIANGXI CN (1)
Zhang S (3)	UNIVs JIANGXI CN (2)
Wei H (3)	UNIVs JIANGXI CN (2)
Nie S (2)	UNIVs JIANGXI CN (2)
Hu J (2)	UNIVs JIANGXI CN (2)
Zheng Y (5)	UNIVs JIANGXI CN (3)
Li H (4)	UNIVs JIANGXI CN (3)
Deng Z (3)	UNIVs JIANGXI CN (3)
Yang Q (1)	UNIVs JILIN CN (1)
Lyu S (1)	UNIVs JILIN CN (1)
Liu B (2)	UNIVs JILIN CN (1)
Woo K (1)	UNIVs KANSAS US (1)
Thompson Lu (1)	UNIVs KANSAS US (1)
Seib Pa (1)	UNIVs KANSAS US (1)

Maningat Cc (1)	UNIVs KANSAS US (1)
Winkler P (1)	UNIVs KIEL DE (1)
Schrezenmeir J (1)	UNIVs KIEL DE (1)
Heller Kj (1)	UNIVs KIEL DE (1)
Ghadimi D (1)	UNIVs KIEL DE (1)
Fölster-holst R (1)	UNIVs KIEL DE (1)
de vrese M (1)	UNIVs KIEL DE (1)
Weese Js (1)	UNIVs KINGSTON GB (1)
Sabour Mr (1)	UNIVs KINGSTON GB (1)
Robinson A (1)	UNIVs KINGSTON GB (1)
Petrof Eo (1)	UNIVs KINGSTON GB (1)
Parsons Mb (1)	UNIVs KINGSTON GB (1)
Jones Cs (1)	UNIVs KINGSTON GB (1)
Jamieson He (1)	UNIVs KINGSTON GB (1)
Ghorbani M (1)	UNIVs KINGSTON GB (1)
Daigneault M (1)	UNIVs KINGSTON GB (1)
Corriveau Mc (1)	UNIVs KINGSTON GB (1)
Carriero C (1)	UNIVs KINGSTON GB (1)
Carlucci C (1)	UNIVs KINGSTON GB (1)
Bidabadi M (1)	UNIVs KINGSTON GB (1)
Öngen G (1)	UNIVs KUMAMOTO JP (1)
Ohura K (1)	UNIVs KUMAMOTO JP (1)
Imai T (1)	UNIVs KUMAMOTO JP (1)
Deliloglu gürhan Si (1)	UNIVs KUMAMOTO JP (1)
Aytekin Ö (1)	UNIVs KUMAMOTO JP (1)
Ushida K (1)	UNIVs KYOTO JP (1)
Tsukahara T (1)	UNIVs KYOTO JP (1)
Koyama H (1)	UNIVs KYOTO JP (1)
Hashizume K (1)	UNIVs KYOTO JP (1)
Zhu yong-guan Y-g (1)	UNIVs LANCASTER GB (1)
Zhang Y-n (1)	UNIVs LANCASTER GB (1)
Pereira Ra (1)	UNIVs LAVRAS BR (1)
Oliveira Dr (1)	UNIVs LAVRAS BR (1)
Lopes Aca (1)	UNIVs LAVRAS BR (1)
Jacxsens L (1)	UNIVs LAVRAS BR (1)
Duarte Wf (1)	UNIVs LAVRAS BR (1)
Carlos da silva júnior E (1)	UNIVs LAVRAS BR (1)
Cardoso Pg (1)	UNIVs LAVRAS BR (1)
Babaahmadifooladi M (1)	UNIVs LAVRAS BR (1)
Woodford N (1)	UNIVs LEEDS GB (1)
Wilkinson V (1)	UNIVs LEEDS GB (1)
Vernon J (1)	UNIVs LEEDS GB (1)
Suleria Har (1)	UNIVs LEEDS GB (1)
Sheppard Ae (1)	UNIVs LEEDS GB (1)
Shearman S (1)	UNIVs LEEDS GB (1)
Sebra R (1)	UNIVs LEEDS GB (1)
Sánchez-velázquez Oa (1)	UNIVs LEEDS GB (1)
Rooney Cm (1)	UNIVs LEEDS GB (1)
Quintanilla-carvajal Mx (1)	UNIVs LEEDS GB (1)
Povey M (1)	UNIVs LEEDS GB (1)
Peto Tea (1)	UNIVs LEEDS GB (1)
Olano A (1)	UNIVs LEEDS GB (1)
Noel Ar (1)	UNIVs LEEDS GB (1)
Nishida Y (1)	UNIVs LEEDS GB (1)
Murray B (1)	UNIVs LEEDS GB (1)
Mulero M (1)	UNIVs LEEDS GB (1)
Montilla A (1)	UNIVs LEEDS GB (1)
Mondor M (1)	UNIVs LEEDS GB (1)
Mitra S (1)	UNIVs LEEDS GB (1)
Mainville I (3)	UNIVs LEEDS GB (1)
Lovering Am (1)	UNIVs LEEDS GB (1)
Longshaw C (1)	UNIVs LEEDS GB (1)
Kettle Mj (1)	UNIVs LEEDS GB (1)
Huscroft Gs (1)	UNIVs LEEDS GB (1)
Hubbard Atm (1)	UNIVs LEEDS GB (1)

Howell K (1)	UNIVs LEEDS GB (1)
Holmes M (1)	UNIVs LEEDS GB (1)
Hobbs Jk (1)	UNIVs LEEDS GB (1)
Higashiyama T (1)	UNIVs LEEDS GB (1)
Henn Mr (1)	UNIVs LEEDS GB (1)
Gu C (1)	UNIVs LEEDS GB (1)
Gaikwad V (1)	UNIVs LEEDS GB (1)
Ford Cb (1)	UNIVs LEEDS GB (1)
Eyre Dw (1)	UNIVs LEEDS GB (1)
Ewin Dj (1)	UNIVs LEEDS GB (1)
Dunshea Fr (1)	UNIVs LEEDS GB (1)
Dingle Ke (1)	UNIVs LEEDS GB (1)
Didelot X (1)	UNIVs LEEDS GB (1)
Cuevas-rodríguez Eo (1)	UNIVs LEEDS GB (1)
Clark Ev (1)	UNIVs LEEDS GB (1)
Chesnel L (1)	UNIVs LEEDS GB (1)
Bryant Ja (1)	UNIVs LEEDS GB (1)
Bently K (1)	UNIVs LEEDS GB (1)
Ashwin H (1)	UNIVs LEEDS GB (1)
Arai N (1)	UNIVs LEEDS GB (1)
Aragón-rojas S (1)	UNIVs LEEDS GB (1)
Todhunter Sl (13)	UNIVs LEEDS GB (13)
Baines Sd (13)	UNIVs LEEDS GB (13)
Crowther Gs (15)	UNIVs LEEDS GB (14)
Chilton Ch (18)	UNIVs LEEDS GB (17)
Freeman J (18)	UNIVs LEEDS GB (18)
Walker As (2)	UNIVs LEEDS GB (2)
Normington C (2)	UNIVs LEEDS GB (2)
Mulet-cabero A-i (2)	UNIVs LEEDS GB (2)
Longshaw Cm (2)	UNIVs LEEDS GB (2)
Karas A (2)	UNIVs LEEDS GB (2)
Crook Dw (2)	UNIVs LEEDS GB (2)
Bentley K (2)	UNIVs LEEDS GB (2)
Arcand Y (4)	UNIVs LEEDS GB (2)
Altringham J (2)	UNIVs LEEDS GB (2)
Wilcox Mh (29)	UNIVs LEEDS GB (28)
Harris Hc (3)	UNIVs LEEDS GB (3)
Davis G (3)	UNIVs LEEDS GB (3)
Spittal W (4)	UNIVs LEEDS GB (4)
O'connor R (4)	UNIVs LEEDS GB (4)
Saxton K (5)	UNIVs LEEDS GB (5)
Ewin D (5)	UNIVs LEEDS GB (5)
Nicholson S (6)	UNIVs LEEDS GB (6)
Clark E (6)	UNIVs LEEDS GB (6)
Moura Ib (7)	UNIVs LEEDS GB (7)
Buckley Am (7)	UNIVs LEEDS GB (7)
Sela I (1)	UNIVs LEIPZIG DE (1)
Schaepe Ss (1)	UNIVs LEIPZIG DE (1)
Mueller S (1)	UNIVs LEIPZIG DE (1)
Meir Ay (1)	UNIVs LEIPZIG DE (1)
Isermann B (1)	UNIVs LEIPZIG DE (1)
Green R (1)	UNIVs LEIPZIG DE (1)
Brandis A (1)	UNIVs LEIPZIG DE (1)
Arinos S (1)	UNIVs LEIPZIG DE (1)
Zeibich L (2)	UNIVs LEIPZIG DE (2)
Tsaban G (2)	UNIVs LEIPZIG DE (2)
Schattenberg F (3)	UNIVs LEIPZIG DE (2)
Schäpe Ss (2)	UNIVs LEIPZIG DE (2)
Kaplan A (2)	UNIVs LEIPZIG DE (2)
Krause Jl (3)	UNIVs LEIPZIG DE (3)
Jehmlich N (3)	UNIVs LEIPZIG DE (3)
Herberth G (3)	UNIVs LEIPZIG DE (3)
Fritz-wallace K (3)	UNIVs LEIPZIG DE (3)
Engelmann B (4)	UNIVs LEIPZIG DE (4)
von bergen M (5)	UNIVs LEIPZIG DE (5)

Rolle-kampczyk U (5)	UNIVs LEIPZIG DE (5)
Sun X (3)	UNIVs LIAONING CN (1)
Meng X (1)	UNIVs LIAONING CN (1)
Gao B (1)	UNIVs LIAONING CN (1)
Swann J (1)	UNIVs LIVERPOOL GB (1)
Ragione Rl (1)	UNIVs LIVERPOOL GB (1)
Proudmana C (1)	UNIVs LIVERPOOL GB (1)
Leng J (1)	UNIVs LIVERPOOL GB (1)
Darby A (1)	UNIVs LIVERPOOL GB (1)
Viñas I (1)	UNIVs LLEIDA ES (1)
Serrano Jce (2)	UNIVs LLEIDA ES (1)
Rubió L (1)	UNIVs LLEIDA ES (1)
Oliveira M (1)	UNIVs LLEIDA ES (1)
Domínguez-fernández M (1)	UNIVs LLEIDA ES (1)
Colás-medà P (1)	UNIVs LLEIDA ES (1)
Anguera M (1)	UNIVs LLEIDA ES (1)
Abadias M (1)	UNIVs LLEIDA ES (1)
Romero M-p (2)	UNIVs LLEIDA ES (2)
Macià A (3)	UNIVs LLEIDA ES (3)
Motilva M-j (4)	UNIVs LLEIDA ES (4)
Mosele Ji (4)	UNIVs LLEIDA ES (4)
Crespo J (1)	UNIVs LOGRONO ES (1)
Wilcox H (1)	UNIVs LONDON GB (1)
Sharp R (1)	UNIVs LONDON GB (1)
Reid G (1)	UNIVs LONDON GB (1)
Nair Sm (1)	UNIVs LONDON GB (1)
Guergoletto Kb (1)	UNIVs LONDON GB (1)
Grimaldi R (1)	UNIVs LONDON GB (1)
Gilmour Sg (1)	UNIVs LONDON GB (1)
Gibbons S (1)	UNIVs LONDON GB (1)
Garcia S (1)	UNIVs LONDON GB (1)
Flores G (1)	UNIVs LONDON GB (1)
Fishbain S (1)	UNIVs LONDON GB (1)
Dewar M (1)	UNIVs LONDON GB (1)
Daisley Ba (1)	UNIVs LONDON GB (1)
Costabile Af (1)	UNIVs LONDON GB (1)
Chmiel Ja (1)	UNIVs LONDON GB (1)
Chin J (1)	UNIVs LONDON GB (1)
Chanyi Rm (1)	UNIVs LONDON GB (1)
Cela D (1)	UNIVs LONDON GB (1)
Burton Jp (1)	UNIVs LONDON GB (1)
Anderson A (1)	UNIVs LONDON GB (1)
Alqurashi Rm (1)	UNIVs LONDON GB (1)
Alarifi Sn (1)	UNIVs LONDON GB (1)
Al Kf (1)	UNIVs LONDON GB (1)
Abdur-rashid K (1)	UNIVs LONDON GB (1)
Yadav V (2)	UNIVs LONDON GB (2)
Swann Jr (2)	UNIVs LONDON GB (2)
Basit Aw (3)	UNIVs LONDON GB (3)
Ract Jnr (1)	UNIVs LONDRINA BR (1)
Pimentel Tc (1)	UNIVs LONDRINA BR (1)
Mantovani Fd (1)	UNIVs LONDRINA BR (1)
Gioielli La (1)	UNIVs LONDRINA BR (1)
Dos santos Cs (1)	UNIVs LONDRINA BR (1)
de santana Ehw (1)	UNIVs LONDRINA BR (1)
de carla bassetto M (1)	UNIVs LONDRINA BR (1)
Aragon-alegro Lc (1)	UNIVs LONDRINA BR (1)
Aragon Dc (1)	UNIVs LONDRINA BR (1)
De souza Chb (2)	UNIVs LONDRINA BR (2)
Wicker L (1)	UNIVs LOUISIANA US (1)
Sathivel S (1)	UNIVs LOUISIANA US (1)
Koh J (1)	UNIVs LOUISIANA US (1)
Chotiko A (1)	UNIVs LOUISIANA US (1)
Zamora J (1)	UNIVs MADRID ES (1)
Thuissard-vasallo I (1)	UNIVs MADRID ES (1)

Soler-rivas C (1)	UNIVs MADRID ES (1)
Saura-calixto F (1)	UNIVs MADRID ES (1)
Sanz Ml (1)	UNIVs MADRID ES (1)
Sánchez-díaz Am (1)	UNIVs MADRID ES (1)
Ruiz-rodríguez A (1)	UNIVs MADRID ES (1)
Ruiz-garbajosa P (1)	UNIVs MADRID ES (1)
Romero-hernández B (1)	UNIVs MADRID ES (1)
Reglero G (1)	UNIVs MADRID ES (1)
Palanisamy M (1)	UNIVs MADRID ES (1)
Möllby R (1)	UNIVs MADRID ES (1)
Marín Fr (1)	UNIVs MADRID ES (1)
León C (1)	UNIVs MADRID ES (1)
Larrosa M (1)	UNIVs MADRID ES (1)
Kwak Y-k (1)	UNIVs MADRID ES (1)
Hernandez-hernandez O (1)	UNIVs MADRID ES (1)
Gil-ramírez A (1)	UNIVs MADRID ES (1)
García-bermejo L (1)	UNIVs MADRID ES (1)
Fernández-romero A (1)	UNIVs MADRID ES (1)
del campo R (1)	UNIVs MADRID ES (1)
Conde-moreno E (1)	UNIVs MADRID ES (1)
Colque-navarro P (1)	UNIVs MADRID ES (1)
Cifuentes A (1)	UNIVs MADRID ES (1)
Carmona M (1)	UNIVs MADRID ES (1)
Cantón R (1)	UNIVs MADRID ES (1)
Villamiel M (2)	UNIVs MADRID ES (2)
Moreno Fj (3)	UNIVs MADRID ES (2)
Goñi I (2)	UNIVs MADRID ES (2)
Ferreira-lazarte A (2)	UNIVs MADRID ES (2)
Spratt P (1)	UNIVs MANCHESTER GB (1)
Pyle Dl (1)	UNIVs MANCHESTER GB (1)
Nicolella C (1)	UNIVs MANCHESTER GB (1)
Madhwani T (1)	UNIVs MANCHESTER GB (1)
Mcbain Aj (4)	UNIVs MANCHESTER GB (2)
Vozzi G (1)	UNIVs MARCHE IT (1)
Vignaroli C (1)	UNIVs MARCHE IT (1)
Vecchione A (1)	UNIVs MARCHE IT (1)
Rocchi M (1)	UNIVs MARCHE IT (1)
Montemurro F (1)	UNIVs MARCHE IT (1)
Mazzantini D (1)	UNIVs MARCHE IT (1)
Mattioli-belmonte M (1)	UNIVs MARCHE IT (1)
Mangiaterra G (1)	UNIVs MARCHE IT (1)
Magliaro C (1)	UNIVs MARCHE IT (1)
Lapomarda A (1)	UNIVs MARCHE IT (1)
Ghelardi E (1)	UNIVs MARCHE IT (1)
De maria C (1)	UNIVs MARCHE IT (1)
Citterio B (1)	UNIVs MARCHE IT (1)
Celandroni F (1)	UNIVs MARCHE IT (1)
Cedraro N (1)	UNIVs MARCHE IT (1)
Calvigioni M (1)	UNIVs MARCHE IT (1)
Biavasco F (1)	UNIVs MARCHE IT (1)
Biagini F (1)	UNIVs MARCHE IT (1)
Nishida Vs (1)	UNIVs MARINGA BR (1)
Koehnlein Ém (1)	UNIVs MARINGA BR (1)
Sheikh J (1)	UNIVs MARYLAND US (1)
Ruiz-perez F (1)	UNIVs MARYLAND US (1)
Phillipsen Mb (1)	UNIVs MARYLAND US (1)
Papp K (1)	UNIVs MARYLAND US (1)
Nataro Jp (1)	UNIVs MARYLAND US (1)
Moser D (1)	UNIVs MARYLAND US (1)
March Jc (1)	UNIVs MARYLAND US (1)
Kwasnica Ma (1)	UNIVs MARYLAND US (1)
Hartmanis Lm (1)	UNIVs MARYLAND US (1)
Hackam D (1)	UNIVs MARYLAND US (1)
Gerrity D (1)	UNIVs MARYLAND US (1)
Davis S (1)	UNIVs MARYLAND US (1)

Costello Cm (1)	UNIVs MARYLAND US (1)
Chen V (1)	UNIVs MARYLAND US (1)
Chang Mw (1)	UNIVs MARYLAND US (1)
Boedeker Ec (1)	UNIVs MARYLAND US (1)
Bentley We (1)	UNIVs MARYLAND US (1)
Yoon Jy (1)	UNIVs MASSACHUSETTS US (1)
Yilmaz O (1)	UNIVs MASSACHUSETTS US (1)
Wright C (1)	UNIVs MASSACHUSETTS US (1)
Widmer G (1)	UNIVs MASSACHUSETTS US (1)
Webster Tj (1)	UNIVs MASSACHUSETTS US (1)
Voigt Ca (1)	UNIVs MASSACHUSETTS US (1)
Trumper Dl (1)	UNIVs MASSACHUSETTS US (1)
Taketani M (1)	UNIVs MASSACHUSETTS US (1)
Sunnapu O (1)	UNIVs MASSACHUSETTS US (1)
Struve C (1)	UNIVs MASSACHUSETTS US (1)
Sphabmixay P (1)	UNIVs MASSACHUSETTS US (1)
Song M (1)	UNIVs MASSACHUSETTS US (1)
Singh S (1)	UNIVs MASSACHUSETTS US (1)
Sela Da (1)	UNIVs MASSACHUSETTS US (1)
Schneider K (1)	UNIVs MASSACHUSETTS US (1)
Sander A (1)	UNIVs MASSACHUSETTS US (1)
Rohatgi G (1)	UNIVs MASSACHUSETTS US (1)
Restrepo-sánchez L-p (1)	UNIVs MASSACHUSETTS US (1)
Parada-alfonso F (1)	UNIVs MASSACHUSETTS US (1)
Pan C (1)	UNIVs MASSACHUSETTS US (1)
Narváez-cuenca C-e (1)	UNIVs MASSACHUSETTS US (1)
Mccormick Ba (1)	UNIVs MASSACHUSETTS US (1)
Maddiboyina B (1)	UNIVs MASSACHUSETTS US (1)
Luo X (1)	UNIVs MASSACHUSETTS US (1)
Lopez-pena Cl (1)	UNIVs MASSACHUSETTS US (1)
Krogfelt Ka (1)	UNIVs MASSACHUSETTS US (1)
Kotla Ng (1)	UNIVs MASSACHUSETTS US (1)
Kester Jc (1)	UNIVs MASSACHUSETTS US (1)
Kemmitt J (1)	UNIVs MASSACHUSETTS US (1)
Jellison Kl (1)	UNIVs MASSACHUSETTS US (1)
Huynh K (1)	UNIVs MASSACHUSETTS US (1)
Huang Y-j (1)	UNIVs MASSACHUSETTS US (1)
Holcomb Sj (1)	UNIVs MASSACHUSETTS US (1)
Hernandez-gordillo V (1)	UNIVs MASSACHUSETTS US (1)
Hendricks G (1)	UNIVs MASSACHUSETTS US (1)
Griffith Lg (1)	UNIVs MASSACHUSETTS US (1)
Goulette Tr (1)	UNIVs MASSACHUSETTS US (1)
Espinal-ruiz M (1)	UNIVs MASSACHUSETTS US (1)
Eng G (1)	UNIVs MASSACHUSETTS US (1)
Demokritou P (1)	UNIVs MASSACHUSETTS US (1)
Demma Z (1)	UNIVs MASSACHUSETTS US (1)
Decker Ea (1)	UNIVs MASSACHUSETTS US (1)
Carrier Rl (1)	UNIVs MASSACHUSETTS US (1)
Carpenter D (1)	UNIVs MASSACHUSETTS US (1)
Boll Ej (1)	UNIVs MASSACHUSETTS US (1)
Bitounis D (1)	UNIVs MASSACHUSETTS US (1)
Bhushan B (1)	UNIVs MASSACHUSETTS US (1)
Bhattacharya K (1)	UNIVs MASSACHUSETTS US (1)
Benton K (1)	UNIVs MASSACHUSETTS US (1)
Zhang R (5)	UNIVs MASSACHUSETTS US (2)
Xing B (2)	UNIVs MASSACHUSETTS US (2)
Gu M (2)	UNIVs MASSACHUSETTS US (2)
Dufour C (2)	UNIVs MASSACHUSETTS US (2)
Breault Dt (2)	UNIVs MASSACHUSETTS US (2)
Xiao H (5)	UNIVs MASSACHUSETTS US (5)
Mcclements Dj (9)	UNIVs MASSACHUSETTS US (9)
Penna Alb (2)	UNIVs MATO GROSSO BR (1)
Casarotti Sn (2)	UNIVs MATO GROSSO BR (1)
Borgonovi Tf (1)	UNIVs MATO GROSSO BR (1)
Batista Clfm (1)	UNIVs MATO GROSSO BR (1)

Waldron Kw (1)	UNIVs MESSINA IT (1)
Tomaino A (1)	UNIVs MESSINA IT (1)
Saija A (1)	UNIVs MESSINA IT (1)
Rich Gt (1)	UNIVs MESSINA IT (1)
Parker Ml (1)	UNIVs MESSINA IT (1)
Mondello L (1)	UNIVs MESSINA IT (1)
Martorana M (1)	UNIVs MESSINA IT (1)
Majdoub Yoe (1)	UNIVs MESSINA IT (1)
Lo curto R (1)	UNIVs MESSINA IT (1)
Ginestra G (1)	UNIVs MESSINA IT (1)
Dugo P (1)	UNIVs MESSINA IT (1)
Cacciola F (1)	UNIVs MESSINA IT (1)
Arcoraci T (1)	UNIVs MESSINA IT (1)
Bisignano C (2)	UNIVs MESSINA IT (2)
Mandalari G (7)	UNIVs MESSINA IT (6)
Wiesinger J (1)	UNIVs MICHIGAN US (1)
Teravest Ma (1)	UNIVs MICHIGAN US (1)
Robinson Cd (1)	UNIVs MICHIGAN US (1)
Martens Ec (1)	UNIVs MICHIGAN US (1)
Glahn Rp (1)	UNIVs MICHIGAN US (1)
Cotta Ma (1)	UNIVs MICHIGAN US (1)
Cichy K (1)	UNIVs MICHIGAN US (1)
Valerio F (1)	UNIVs MILAN IT (1)
Tomas M (1)	UNIVs MILAN IT (1)
Taddei F (1)	UNIVs MILAN IT (1)
Simões da silva Tm (1)	UNIVs MILAN IT (1)
Silvetti T (1)	UNIVs MILAN IT (1)
Scazzina F (1)	UNIVs MILAN IT (1)
Piazentin Acm (1)	UNIVs MILAN IT (1)
Orlandi M (1)	UNIVs MILAN IT (1)
Ohra-aho T (1)	UNIVs MILAN IT (1)
Morelli L (1)	UNIVs MILAN IT (1)
Mora D (1)	UNIVs MILAN IT (1)
Mendonça Cmn (1)	UNIVs MILAN IT (1)
Masoero F (1)	UNIVs MILAN IT (1)
Martini D (1)	UNIVs MILAN IT (1)
Lonigro Sl (1)	UNIVs MILAN IT (1)
Lombardi G (2)	UNIVs MILAN IT (1)
Lavermicocca P (1)	UNIVs MILAN IT (1)
Gallo A (1)	UNIVs MILAN IT (1)
Ferraretto A (1)	UNIVs MILAN IT (1)
Ferchichi A (1)	UNIVs MILAN IT (1)
Fellah B (1)	UNIVs MILAN IT (1)
Dodi R (1)	UNIVs MILAN IT (1)
de souza oliveira Rp (1)	UNIVs MILAN IT (1)
De noni I (1)	UNIVs MILAN IT (1)
De bellis P (1)	UNIVs MILAN IT (1)
D'egidio Mg (1)	UNIVs MILAN IT (1)
Cossu M (1)	UNIVs MILAN IT (1)
Converti A (1)	UNIVs MILAN IT (1)
Cattaneo S (1)	UNIVs MILAN IT (1)
Cardone G (1)	UNIVs MILAN IT (1)
Capanoglu E (1)	UNIVs MILAN IT (1)
Brasca M (1)	UNIVs MILAN IT (1)
Bottani M (1)	UNIVs MILAN IT (1)
Bogsan Csb (1)	UNIVs MILAN IT (1)
Blasi F (1)	UNIVs MILAN IT (1)
Biasini B (1)	UNIVs MILAN IT (1)
Bannour M (1)	UNIVs MILAN IT (1)
Baccolo G (1)	UNIVs MILAN IT (1)
Senizza B (2)	UNIVs MILAN IT (2)
Montesano D (2)	UNIVs MILAN IT (2)
Ghisoni S (2)	UNIVs MILAN IT (2)
Chiodelli G (2)	UNIVs MILAN IT (2)
Casiraghi Mc (2)	UNIVs MILAN IT (2)

Trevisan M (3)	UNIVs MILAN IT (3)
Rocchetti G (5)	UNIVs MILAN IT (5)
Lucini L (5)	UNIVs MILAN IT (5)
Giuberti G (5)	UNIVs MILAN IT (5)
Vinderola Cg (1)	UNIVs MINAS GERAIS BR (1)
Uetanabaro Apt (1)	UNIVs MINAS GERAIS BR (1)
Saito Vst (1)	UNIVs MINAS GERAIS BR (1)
Romano C (1)	UNIVs MINAS GERAIS BR (1)
Ribeiro Go (1)	UNIVs MINAS GERAIS BR (1)
Meale Sj (1)	UNIVs MINAS GERAIS BR (1)
Mcallister Ta (1)	UNIVs MINAS GERAIS BR (1)
Holo H (1)	UNIVs MINAS GERAIS BR (1)
He Ml (1)	UNIVs MINAS GERAIS BR (1)
Harstad Om (1)	UNIVs MINAS GERAIS BR (1)
Dugan Mer (1)	UNIVs MINAS GERAIS BR (1)
dos santos Tf (1)	UNIVs MINAS GERAIS BR (1)
Costa Mm (1)	UNIVs MINAS GERAIS BR (1)
Chaves Av (1)	UNIVs MINAS GERAIS BR (1)
Araújo Ls (1)	UNIVs MINAS GERAIS BR (1)
Andrioli Jl (1)	UNIVs MINAS GERAIS BR (1)
Alazzeh Ay (1)	UNIVs MINAS GERAIS BR (1)
Nicoli Jr (2)	UNIVs MINAS GERAIS BR (2)
Wells Cl (1)	UNIVs MINNESOTA US (1)
Van de westerlo Ema (1)	UNIVs MINNESOTA US (1)
Mancinelli D (1)	UNIVs MINNESOTA US (1)
Jechorek Rp (1)	UNIVs MINNESOTA US (1)
Erlandsen Sl (1)	UNIVs MINNESOTA US (1)
Berger A (1)	UNIVs MINNESOTA US (1)
Marshall Dl (1)	UNIVs MISSISSIPPI US (1)
Koo J (1)	UNIVs MISSISSIPPI US (1)
Depaola A (1)	UNIVs MISSISSIPPI US (1)
Sitati N (1)	UNIVs MISSOURI US (1)
Klymus K (1)	UNIVs MISSOURI US (1)
Gunn Jc (1)	UNIVs MISSOURI US (1)
Finch T (1)	UNIVs MISSOURI US (1)
Eggert Ls (1)	UNIVs MISSOURI US (1)
Budd K (1)	UNIVs MISSOURI US (1)
Gordon Ji (2)	UNIVs MISSOURI US (2)
Volpi N (1)	UNIVs MODENA AND REGGIO EMILIA IT (1)
Ulrici A (1)	UNIVs MODENA AND REGGIO EMILIA IT (1)
Tagliazucchi D (1)	UNIVs MODENA AND REGGIO EMILIA IT (1)
Sforza S (1)	UNIVs MODENA AND REGGIO EMILIA IT (1)
Quartieri A (1)	UNIVs MODENA AND REGGIO EMILIA IT (1)
Prandi B (1)	UNIVs MODENA AND REGGIO EMILIA IT (1)
Nocetti M (1)	UNIVs MODENA AND REGGIO EMILIA IT (1)
Martini S (1)	UNIVs MODENA AND REGGIO EMILIA IT (1)
Leonardi A (1)	UNIVs MODENA AND REGGIO EMILIA IT (1)
Gatti M (1)	UNIVs MODENA AND REGGIO EMILIA IT (1)
Conte A (1)	UNIVs MODENA AND REGGIO EMILIA IT (1)
Bottari B (1)	UNIVs MODENA AND REGGIO EMILIA IT (1)
Rossi M (2)	UNIVs MODENA AND REGGIO EMILIA IT (2)
Raimondi S (2)	UNIVs MODENA AND REGGIO EMILIA IT (2)
Amaretti A (2)	UNIVs MODENA AND REGGIO EMILIA IT (2)
Tremonte P (1)	UNIVs MOLISE IT (1)
Tipaldi L (1)	UNIVs MOLISE IT (1)
Succi M (1)	UNIVs MOLISE IT (1)
Sorrentino E (1)	UNIVs MOLISE IT (1)
Pannella G (1)	UNIVs MOLISE IT (1)
Cozzolino A (1)	UNIVs MOLISE IT (1)
Coppola R (1)	UNIVs MOLISE IT (1)
Remize F (1)	UNIVs MONTPELLIER FR (1)
Mashitoa Fm (1)	UNIVs MONTPELLIER FR (1)
Manhevi Ve (1)	UNIVs MONTPELLIER FR (1)
Garcia C (1)	UNIVs MONTPELLIER FR (1)
Akinola Sa (1)	UNIVs MONTPELLIER FR (1)

Schick S (1)	UNIVs MUNICH DE (1)
Engel J (1)	UNIVs MUNICH DE (1)
Kulozik U (2)	UNIVs MUNICH DE (2)
Haindl R (2)	UNIVs MUNICH DE (2)
Zafrilla P (1)	UNIVs MURCIA ES (1)
Sánchez-moya T (1)	UNIVs MURCIA ES (1)
Ros-berruezo G (1)	UNIVs MURCIA ES (1)
Planes D (1)	UNIVs MURCIA ES (1)
Oliva J (1)	UNIVs MURCIA ES (1)
Martínez G (1)	UNIVs MURCIA ES (1)
López-nicolás R (1)	UNIVs MURCIA ES (1)
González-bermúdez Ca (1)	UNIVs MURCIA ES (1)
Frontela-saseta C (1)	UNIVs MURCIA ES (1)
Cermeño S (1)	UNIVs MURCIA ES (1)
Camara Ma (1)	UNIVs MURCIA ES (1)
Scher J (1)	UNIVs NANCY METZ FR (1)
Guerin J (1)	UNIVs NANCY METZ FR (1)
Gaiani C (1)	UNIVs NANCY METZ FR (1)
Desobry S (1)	UNIVs NANCY METZ FR (1)
Burgain J (1)	UNIVs NANCY METZ FR (1)
Borges F (1)	UNIVs NANCY METZ FR (1)
Bhandari B (1)	UNIVs NANCY METZ FR (1)
Zotta T (1)	UNIVs NAPLES IT (1)
Wells Jm (1)	UNIVs NAPLES IT (1)
van valenberg Hjf (1)	UNIVs NAPLES IT (1)
Tsakalidou E (2)	UNIVs NAPLES IT (1)
Troise Ad (1)	UNIVs NAPLES IT (1)
Tripodi L (1)	UNIVs NAPLES IT (1)
Travaglia F (1)	UNIVs NAPLES IT (1)
Tenore Gc (1)	UNIVs NAPLES IT (1)
Schisano C (1)	UNIVs NAPLES IT (1)
Santos-hernández M (1)	UNIVs NAPLES IT (1)
Ricciardi A (1)	UNIVs NAPLES IT (1)
Pisapia L (1)	UNIVs NAPLES IT (1)
Pavlidis D (1)	UNIVs NAPLES IT (1)
Pastore L (1)	UNIVs NAPLES IT (1)
Parente E (1)	UNIVs NAPLES IT (1)
Paparo L (1)	UNIVs NAPLES IT (1)
Pacifico S (1)	UNIVs NAPLES IT (1)
Novellino E (1)	UNIVs NAPLES IT (1)
Nitride C (1)	UNIVs NAPLES IT (1)
Narciso V (1)	UNIVs NAPLES IT (1)
Mauriello G (1)	UNIVs NAPLES IT (1)
Masi P (1)	UNIVs NAPLES IT (1)
Mamone G (1)	UNIVs NAPLES IT (1)
Maisto M (1)	UNIVs NAPLES IT (1)
Loonen Lmp (1)	UNIVs NAPLES IT (1)
Koper Jeb (1)	UNIVs NAPLES IT (1)
Ianniello Rg (1)	UNIVs NAPLES IT (1)
Guidone A (1)	UNIVs NAPLES IT (1)
Grosso M (1)	UNIVs NAPLES IT (1)
Gallo V (1)	UNIVs NAPLES IT (1)
De prisco A (1)	UNIVs NAPLES IT (1)
De pascale S (1)	UNIVs NAPLES IT (1)
Damiano C (1)	UNIVs NAPLES IT (1)
Daliu P (1)	UNIVs NAPLES IT (1)
Corollaro Ml (1)	UNIVs NAPLES IT (1)
Cioffi S (1)	UNIVs NAPLES IT (1)
Ciampaglia R (1)	UNIVs NAPLES IT (1)
Bruno C (1)	UNIVs NAPLES IT (1)
Berni canani R (1)	UNIVs NAPLES IT (1)
Arlorio M (1)	UNIVs NAPLES IT (1)
Aponte M (1)	UNIVs NAPLES IT (1)
Annunziata G (1)	UNIVs NAPLES IT (1)
Addeo F (1)	UNIVs NAPLES IT (1)

Romano A (3)	UNIVs NAPLES IT (2)
Napolitano A (2)	UNIVs NAPLES IT (2)
Ferranti P (2)	UNIVs NAPLES IT (2)
Ferracane R (2)	UNIVs NAPLES IT (2)
Vitaglione P (3)	UNIVs NAPLES IT (3)
Rodríguez-carrasco Y (3)	UNIVs NAPLES IT (3)
Ritieni A (3)	UNIVs NAPLES IT (3)
Narváez A (3)	UNIVs NAPLES IT (3)
Izzo L (4)	UNIVs NAPLES IT (3)
Castaldo L (3)	UNIVs NAPLES IT (3)
Fogliano V (6)	UNIVs NAPLES IT (4)
Yang J (10)	UNIVs NEBRASKA US (3)
Rose Dj (6)	UNIVs NEBRASKA US (6)
Patra M (1)	UNIVs NEVADA US (1)
Howerton A (1)	UNIVs NEVADA US (1)
Abel-santos E (1)	UNIVs NEVADA US (1)
Zhu J (6)	UNIVs NEW JERSEY US (2)
Lu X (4)	UNIVs NEW JERSEY US (3)
Huang Q (6)	UNIVs NEW JERSEY US (3)
Werner Jj (1)	UNIVs NEW YORK US (1)
Venkataraman A (1)	UNIVs NEW YORK US (1)
Ranganathan P (1)	UNIVs NEW YORK US (1)
Ranganathan N (1)	UNIVs NEW YORK US (1)
Perkins Sd (1)	UNIVs NEW YORK US (1)
Pechenyak B (1)	UNIVs NEW YORK US (1)
Patel Bg (1)	UNIVs NEW YORK US (1)
Mehta R (1)	UNIVs NEW YORK US (1)
Marczely J (1)	UNIVs NEW YORK US (1)
Jeraci Jl (1)	UNIVs NEW YORK US (1)
Horvath Pj (1)	UNIVs NEW YORK US (1)
Guzman Jjl (1)	UNIVs NEW YORK US (1)
Gu Az (1)	UNIVs NEW YORK US (1)
Friedman Ea (1)	UNIVs NEW YORK US (1)
Dunn Sr (1)	UNIVs NEW YORK US (1)
Dheer R (1)	UNIVs NEW YORK US (1)
Rosenbaum Ma (2)	UNIVs NEW YORK US (2)
Angenent Lt (3)	UNIVs NEW YORK US (3)
Rumney Cj (1)	UNIVs NEWCASTLE GB (1)
Reed Ka (1)	UNIVs NEWCASTLE GB (1)
Nordgaard I (1)	UNIVs NEWCASTLE GB (1)
Mortensen Pb (1)	UNIVs NEWCASTLE GB (1)
George T (1)	UNIVs NEWCASTLE GB (1)
Mathers Jc (2)	UNIVs NEWCASTLE GB (2)
Silverman Jd (2)	UNIVs NORTH CAROLINA US (2)
Mukherjee S (2)	UNIVs NORTH CAROLINA US (2)
Durand Hk (2)	UNIVs NORTH CAROLINA US (2)
David La (2)	UNIVs NORTH CAROLINA US (2)
Bloom Rj (2)	UNIVs NORTH CAROLINA US (2)
Yu Z (2)	UNIVs OHIO US (1)
Yang S-t (1)	UNIVs OHIO US (1)
Whitacre S (1)	UNIVs OHIO US (1)
Solomkin Js (1)	UNIVs OHIO US (1)
Page Cp (1)	UNIVs OHIO US (1)
Kubachka Km (1)	UNIVs OHIO US (1)
Karnati Skr (1)	UNIVs OHIO US (1)
Gallawa Cm (1)	UNIVs OHIO US (1)
Firkins Jl (1)	UNIVs OHIO US (1)
Dellinger Ep (1)	UNIVs OHIO US (1)
Dayton Ea (1)	UNIVs OHIO US (1)
Creed Jt (1)	UNIVs OHIO US (1)
Brinques Gb (1)	UNIVs OHIO US (1)
Bradham K (1)	UNIVs OHIO US (1)
Bohnen Jma (1)	UNIVs OHIO US (1)
Bjornson Hs (1)	UNIVs OHIO US (1)
Bednarcik Ja (1)	UNIVs OHIO US (1)

Basta N (1)	UNIVs OHIO US (1)
Ayub Maz (1)	UNIVs OHIO US (1)
Suwardana Gnr (1)	UNIVs OKAYAMA JP (1)
Sujaya In (1)	UNIVs OKAYAMA JP (1)
Sakaguchi M (1)	UNIVs OKAYAMA JP (1)
Ramona Y (1)	UNIVs OKAYAMA JP (1)
Nocianitri Ka (1)	UNIVs OKAYAMA JP (1)
Mayura Ipb (1)	UNIVs OKAYAMA JP (1)
Matsushita O (1)	UNIVs OKAYAMA JP (1)
Komalasari Nlgy (1)	UNIVs OKAYAMA JP (1)
Gotoh K (1)	UNIVs OKAYAMA JP (1)
Fatmawati Nnd (1)	UNIVs OKAYAMA JP (1)
Wasteson Y (1)	UNIVs OSLO NO (1)
Skjerdal T (1)	UNIVs OSLO NO (1)
Pettersen Ks (1)	UNIVs OSLO NO (1)
Lindbäck T (1)	UNIVs OSLO NO (1)
Aspholm M (1)	UNIVs OSLO NO (1)
Tochio T (1)	UNIVs OVIEDO ES (1)
Suárez M (1)	UNIVs OVIEDO ES (1)
Solís G (1)	UNIVs OVIEDO ES (1)
Hirano K (1)	UNIVs OVIEDO ES (1)
Gómez E (1)	UNIVs OVIEDO ES (1)
Fernández N (1)	UNIVs OVIEDO ES (1)
Endo A (1)	UNIVs OVIEDO ES (1)
Suárez A (3)	UNIVs OVIEDO ES (3)
Salazar N (4)	UNIVs OVIEDO ES (3)
Tarrah A (1)	UNIVs PADUA IT (1)
Rossi Rc (1)	UNIVs PADUA IT (1)
Righetto ziegler D (1)	UNIVs PADUA IT (1)
Pakroo S (1)	UNIVs PADUA IT (1)
Luchese Rh (1)	UNIVs PADUA IT (1)
Lemos junior Wjf (1)	UNIVs PADUA IT (1)
Giacomini A (1)	UNIVs PADUA IT (1)
de castilhos J (1)	UNIVs PADUA IT (1)
da silva duarte V (1)	UNIVs PADUA IT (1)
Corich V (1)	UNIVs PADUA IT (1)
Gayoso L (1)	UNIVs PAMPLONA ES (1)
Claerbout A-s (1)	UNIVs PAMPLONA ES (1)
Cavero Ry (1)	UNIVs PAMPLONA ES (1)
Carafa I (1)	UNIVs PAMPLONA ES (1)
Calvo Mi (1)	UNIVs PAMPLONA ES (1)
Astiasarán I (1)	UNIVs PAMPLONA ES (1)
Ansorena D (1)	UNIVs PAMPLONA ES (1)
Juániz I (2)	UNIVs PAMPLONA ES (2)
Ludwig Ia (4)	UNIVs PAMPLONA ES (3)
de peña M-p (4)	UNIVs PAMPLONA ES (4)
Cid C (4)	UNIVs PAMPLONA ES (4)
Seguí L (1)	UNIVs PARA BR (1)
Betoret N (1)	UNIVs PARA BR (1)
Bas-bellver C (1)	UNIVs PARA BR (1)
Barrera C (1)	UNIVs PARA BR (1)
Artacho A (1)	UNIVs PARA BR (1)
Andrés C (1)	UNIVs PARA BR (1)
Vasconcelos de oliveira Ce (1)	UNIVs PARAIBA BR (1)
Tribuzy de magalhães cordeiro Am (1)	UNIVs PARAIBA BR (1)
Souza olegário L (1)	UNIVs PARAIBA BR (1)
Silva júnior Jcd (1)	UNIVs PARAIBA BR (1)
Ramos do egyptoqueiroga Rdc (1)	UNIVs PARAIBA BR (1)
Portela Ia (1)	UNIVs PARAIBA BR (1)
Pereira gomes Am (1)	UNIVs PARAIBA BR (1)
Menezes Fndd (1)	UNIVs PARAIBA BR (1)
Matte Tc (1)	UNIVs PARAIBA BR (1)
Macedo dantas A (1)	UNIVs PARAIBA BR (1)

Luciano Wa (1)	UNIVs PARAIBA BR (1)
Leite de souza E (1)	UNIVs PARAIBA BR (1)
Lacerda massa Nm (1)	UNIVs PARAIBA BR (1)
Gomes de oliveira Me (1)	UNIVs PARAIBA BR (1)
Garcia Ef (1)	UNIVs PARAIBA BR (1)
Ferreira madureira Arm (1)	UNIVs PARAIBA BR (1)
Fernandes garcia E (1)	UNIVs PARAIBA BR (1)
Esteves pintado Mm (1)	UNIVs PARAIBA BR (1)
Diniz-silva Ht (1)	UNIVs PARAIBA BR (1)
de souza aquino J (1)	UNIVs PARAIBA BR (1)
de sousa galvão M (1)	UNIVs PARAIBA BR (1)
de oliveira Spa (1)	UNIVs PARAIBA BR (1)
de medeiros Ll (1)	UNIVs PARAIBA BR (1)
de lima Lc (1)	UNIVs PARAIBA BR (1)
de lima brito I (1)	UNIVs PARAIBA BR (1)
Dantas duarte menezes Fn (1)	UNIVs PARAIBA BR (1)
da silva vieira Ar (1)	UNIVs PARAIBA BR (1)
da silva campelo borges G (1)	UNIVs PARAIBA BR (1)
da cruz almeida Ét (1)	UNIVs PARAIBA BR (1)
Castellano Lrc (1)	UNIVs PARAIBA BR (1)
Brandão Lr (1)	UNIVs PARAIBA BR (1)
Almeida da costa Wk (1)	UNIVs PARAIBA BR (1)
Magnani M (10)	UNIVs PARAIBA BR (10)
Madruga Ms (2)	UNIVs PARAIBA BR (2)
Maciel Jf (2)	UNIVs PARAIBA BR (2)
dos santos lima M (2)	UNIVs PARAIBA BR (2)
da conceição Ml (2)	UNIVs PARAIBA BR (2)
de albuquerque Tmr (3)	UNIVs PARAIBA BR (3)
Lima Mds (5)	UNIVs PARAIBA BR (4)
de souza El (7)	UNIVs PARAIBA BR (7)
Stafussa Ap (1)	UNIVs PARANA BR (1)
Ribeiro Vr (1)	UNIVs PARANA BR (1)
Pontarolo R (1)	UNIVs PARANA BR (1)
Maciel Gm (1)	UNIVs PARANA BR (1)
Kato Cg (1)	UNIVs PARANA BR (1)
Isidoro haminiuk Cw (1)	UNIVs PARANA BR (1)
Haminiuk Cwi (1)	UNIVs PARANA BR (1)
Fachi Mm (1)	UNIVs PARANA BR (1)
de andrade arruda fernandes I (1)	UNIVs PARANA BR (1)
Calhelha Rc (1)	UNIVs PARANA BR (1)
Corrêa Rcg (2)	UNIVs PARANA BR (2)
Mousset P-y (1)	UNIVs PARIS FR (1)
Medina S (1)	UNIVs PARIS FR (1)
Langella P (1)	UNIVs PARIS FR (1)
Lafay S (1)	UNIVs PARIS FR (1)
Guéneau V (1)	UNIVs PARIS FR (1)
Gil-izquierdo A (1)	UNIVs PARIS FR (1)
Deschamps J (1)	UNIVs PARIS FR (1)
Chamignon C (1)	UNIVs PARIS FR (1)
Briandet R (1)	UNIVs PARIS FR (1)
Bermúdez-humarán Lg (1)	UNIVs PARIS FR (1)
Doré J (3)	UNIVs PARIS FR (2)
Pede Gd (1)	UNIVs PARMA IT (1)
Francinelli V (1)	UNIVs PARMA IT (1)
Riva A (2)	UNIVs PARMA IT (2)
Petrangolini G (2)	UNIVs PARMA IT (2)
Favari C (2)	UNIVs PARMA IT (2)
Dall'asta M (2)	UNIVs PARMA IT (2)
Brighenti F (2)	UNIVs PARMA IT (2)
Allegrini P (2)	UNIVs PARMA IT (2)
Ventura M (3)	UNIVs PARMA IT (3)
Mancabelli L (3)	UNIVs PARMA IT (3)
Dall'asta M (4)	UNIVs PARMA IT (4)
Mena P (5)	UNIVs PARMA IT (5)

Calani L (5)	UNIVs PARMA IT (5)
Bresciani L (7)	UNIVs PARMA IT (7)
Del rio D (8)	UNIVs PARMA IT (8)
Todorov Sd (2)	UNIVs PAULISTA BR (1)
Tallarico Ma (1)	UNIVs PAULISTA BR (1)
Schmelzer E (1)	UNIVs PENNSYLVANIA US (1)
Pekor C (1)	UNIVs PENNSYLVANIA US (1)
Nettleship I (1)	UNIVs PENNSYLVANIA US (1)
Gerlach Jc (1)	UNIVs PENNSYLVANIA US (1)
Tirella A (1)	UNIVs PISA IT (1)
Sbrana T (1)	UNIVs PISA IT (1)
Meucci S (1)	UNIVs PISA IT (1)
Mattei G (1)	UNIVs PISA IT (1)
Martinucci V (1)	UNIVs PISA IT (1)
La marca M (1)	UNIVs PISA IT (1)
Di patria V (1)	UNIVs PISA IT (1)
Corti A (1)	UNIVs PISA IT (1)
Cacopardo L (1)	UNIVs PISA IT (1)
Buoncompagni L (1)	UNIVs PISA IT (1)
Giusti S (2)	UNIVs PISA IT (2)
Domenici C (2)	UNIVs PISA IT (2)
Costa J (3)	UNIVs PISA IT (3)
Ahluwalia A (4)	UNIVs PISA IT (4)
Weijma J (1)	UNIVs POHANG KR (1)
Vazquez J (1)	UNIVs POHANG KR (1)
Van lier Jb (1)	UNIVs POHANG KR (1)
Shin Sg (1)	UNIVs POHANG KR (1)
Lindeboom Ref (1)	UNIVs POHANG KR (1)
Lim J (1)	UNIVs POHANG KR (1)
Kang H-j (1)	UNIVs POHANG KR (1)
Jeong Hw (1)	UNIVs POHANG KR (1)
Holzapfel Wh (1)	UNIVs POHANG KR (1)
Cho D (1)	UNIVs POHANG KR (1)
Arellano K (1)	UNIVs POHANG KR (1)
Zoumpopoulou G (1)	UNIVs READING GB (1)
Wynne A (1)	UNIVs READING GB (1)
Wichienchot S (2)	UNIVs READING GB (1)
Theis S (1)	UNIVs READING GB (1)
Tassou Cc (1)	UNIVs READING GB (1)
Stoklosinski H (1)	UNIVs READING GB (1)
Shimoni E (1)	UNIVs READING GB (1)
Shewry Pr (1)	UNIVs READING GB (1)
Sailer M (1)	UNIVs READING GB (1)
Rastall R (1)	UNIVs READING GB (1)
Pyle S (1)	UNIVs READING GB (1)
Prasertsan P (1)	UNIVs READING GB (1)
Plos S (1)	UNIVs READING GB (1)
Parham Nj (1)	UNIVs READING GB (1)
Panagou Ez (1)	UNIVs READING GB (1)
Oyarzábal Is (1)	UNIVs READING GB (1)
Nychas Gje (1)	UNIVs READING GB (1)
Naeye T (1)	UNIVs READING GB (1)
Mcghie T (1)	UNIVs READING GB (1)
Mccleary Bv (1)	UNIVs READING GB (1)
Martin-morales A (1)	UNIVs READING GB (1)
Liu C (2)	UNIVs READING GB (1)
Koutsos A (1)	UNIVs READING GB (1)
Kosters Mh (1)	UNIVs READING GB (1)
Kennedy Ob (1)	UNIVs READING GB (1)
Karatzas Kag (1)	UNIVs READING GB (1)
Hughes Sa (1)	UNIVs READING GB (1)
Hofmann A (1)	UNIVs READING GB (1)
Hobden Mr (1)	UNIVs READING GB (1)
Herrero M (2)	UNIVs READING GB (1)
Grandison As (1)	UNIVs READING GB (1)

Gee Jn (1)	UNIVs READING GB (1)
Gearry R (1)	UNIVs READING GB (1)
Gasperotti M (1)	UNIVs READING GB (1)
Einerhand Aw (1)	UNIVs READING GB (1)
Díez-municio M (1)	UNIVs READING GB (1)
Conterno L (1)	UNIVs READING GB (1)
Connolly Ml (1)	UNIVs READING GB (1)
Collins M (1)	UNIVs READING GB (1)
Chen Ya (1)	UNIVs READING GB (1)
Bianchi M (1)	UNIVs READING GB (1)
Bell A (1)	UNIVs READING GB (1)
Beards Ej (1)	UNIVs READING GB (1)
Beards E (1)	UNIVs READING GB (1)
Argyri Aa (1)	UNIVs READING GB (1)
Ames Jm (1)	UNIVs READING GB (1)
Alarifi S (1)	UNIVs READING GB (1)
Rastall Ra (16)	UNIVs READING GB (16)
Vulevic J (2)	UNIVs READING GB (2)
Steer Te (2)	UNIVs READING GB (2)
Sannasiddappa Th (2)	UNIVs READING GB (2)
Olano-martin E (2)	UNIVs READING GB (2)
Lovegrove Ja (2)	UNIVs READING GB (2)
Likotrafiti E (2)	UNIVs READING GB (2)
Johnson It (2)	UNIVs READING GB (2)
Goulas Ak (2)	UNIVs READING GB (2)
Gee Jm (2)	UNIVs READING GB (2)
Clarke Sr (2)	UNIVs READING GB (2)
Charalampopoulos D (2)	UNIVs READING GB (2)
Walton G (3)	UNIVs READING GB (3)
Tzortzis G (3)	UNIVs READING GB (3)
Mountzouris Kc (3)	UNIVs READING GB (3)
Klinder A (4)	UNIVs READING GB (3)
Allsopp P (3)	UNIVs READING GB (3)
Gibson Gr (37)	UNIVs READING GB (30)
Gibson G (5)	UNIVs READING GB (4)
Rowland I (5)	UNIVs READING GB (5)
Kolida S (6)	UNIVs READING GB (6)
Walton Ge (7)	UNIVs READING GB (7)
Tuohy Km (10)	UNIVs READING GB (9)
Costabile A (12)	UNIVs READING GB (9)
Riggio O (1)	UNIVs ROME IT (1)
Perreten V (1)	UNIVs ROME IT (1)
Pandolfi F (1)	UNIVs ROME IT (1)
Endimiani A (1)	UNIVs ROME IT (1)
Donà V (1)	UNIVs ROME IT (1)
Costamagna G (1)	UNIVs ROME IT (1)
Cianci R (1)	UNIVs ROME IT (1)
Carattoli A (1)	UNIVs ROME IT (1)
Capocaccia L (1)	UNIVs ROME IT (1)
Campos-madueno Ei (1)	UNIVs ROME IT (1)
Bernasconi Oj (1)	UNIVs ROME IT (1)
Westerman Pr (1)	UNIVs ROSTOCK DE (1)
Till H (1)	UNIVs ROSTOCK DE (1)
Stadlbauer V (1)	UNIVs ROSTOCK DE (1)
Singer G (1)	UNIVs ROSTOCK DE (1)
Russmayer H (1)	UNIVs ROSTOCK DE (1)
Peterbauer C (1)	UNIVs ROSTOCK DE (1)
Obermüller B (1)	UNIVs ROSTOCK DE (1)
Miekisch W (1)	UNIVs ROSTOCK DE (1)
Mayrhofer S (1)	UNIVs ROSTOCK DE (1)
Klymiuk I (1)	UNIVs ROSTOCK DE (1)
Kienesberger B (1)	UNIVs ROSTOCK DE (1)
Horvath A (1)	UNIVs ROSTOCK DE (1)
Heinl S (1)	UNIVs ROSTOCK DE (1)
Grabherr R (1)	UNIVs ROSTOCK DE (1)

Gerowitt B (1)	UNIVs ROSTOCK DE (1)
Fuchs P (1)	UNIVs ROSTOCK DE (1)
Castellani C (1)	UNIVs ROSTOCK DE (1)
Zarrella I (1)	UNIVs SALERNO IT (1)
Rossi F (1)	UNIVs SALERNO IT (1)
Proto A (1)	UNIVs SALERNO IT (1)
Motta O (1)	UNIVs SALERNO IT (1)
Matrella S (1)	UNIVs SALERNO IT (1)
Marchettini N (1)	UNIVs SALERNO IT (1)
Fortunato G (1)	UNIVs SALERNO IT (1)
Vieira Crw (1)	UNIVs SANTA CATARINA BR (1)
Verruck S (1)	UNIVs SANTA CATARINA BR (1)
Prudencio Es (1)	UNIVs SANTA CATARINA BR (1)
Miotto M (1)	UNIVs SANTA CATARINA BR (1)
Maurício Lds (1)	UNIVs SANTA CATARINA BR (1)
Maran Bm (1)	UNIVs SANTA CATARINA BR (1)
Barretta C (1)	UNIVs SANTA CATARINA BR (1)
Salawu So (1)	UNIVs SANTA MARIA BR (1)
Faloye Of (1)	UNIVs SANTA MARIA BR (1)
Boligon Aa (1)	UNIVs SANTA MARIA BR (1)
Athayde Ml (1)	UNIVs SANTA MARIA BR (1)
Alao Of (1)	UNIVs SANTA MARIA BR (1)
Akindahunsi Aa (1)	UNIVs SANTA MARIA BR (1)
Toro-uribe S (1)	UNIVs SANTANDER ES (1)
López-giraldo Lj (1)	UNIVs SANTANDER ES (1)
Ibánez E (1)	UNIVs SANTANDER ES (1)
Alvarez-rivera G (1)	UNIVs SANTANDER ES (1)
Silva Jc (1)	UNIVs SANTIAGO DE COMPOSTELA ES (1)
Moreira R (1)	UNIVs SANTIAGO DE COMPOSTELA ES (1)
Zura L (1)	UNIVs SANTOS BR (1)
Vera c N (1)	UNIVs SANTOS BR (1)
Puente L (1)	UNIVs SANTOS BR (1)
Zaiat M (1)	UNIVs SAO CARLOS BR (1)
Varesche Mba (1)	UNIVs SAO CARLOS BR (1)
Umbelino cavallini Dc (1)	UNIVs SAO CARLOS BR (1)
Tallarico adorno Ma (1)	UNIVs SAO CARLOS BR (1)
Silva El (1)	UNIVs SAO CARLOS BR (1)
Schultz G (1)	UNIVs SAO CARLOS BR (1)
Roselino Mn (1)	UNIVs SAO CARLOS BR (1)
Ribeiro Trm (1)	UNIVs SAO CARLOS BR (1)
Ribeiro R (1)	UNIVs SAO CARLOS BR (1)
Ribeiro Mpa (1)	UNIVs SAO CARLOS BR (1)
Nopens I (1)	UNIVs SAO CARLOS BR (1)
Naessens W (1)	UNIVs SAO CARLOS BR (1)
Márcia canaan Jm (1)	UNIVs SAO CARLOS BR (1)
Machado ribeiro Tr (1)	UNIVs SAO CARLOS BR (1)
Lopes Np (1)	UNIVs SAO CARLOS BR (1)
Lima Fv (1)	UNIVs SAO CARLOS BR (1)
Hanson Nj (1)	UNIVs SAO CARLOS BR (1)
Gomes Bc (1)	UNIVs SAO CARLOS BR (1)
Giordano Rc (1)	UNIVs SAO CARLOS BR (1)
Genovese Mi (1)	UNIVs SAO CARLOS BR (1)
Freire Fc (1)	UNIVs SAO CARLOS BR (1)
Fortney Nw (1)	UNIVs SAO CARLOS BR (1)
Eng F (1)	UNIVs SAO CARLOS BR (1)
de valdez Gf (1)	UNIVs SAO CARLOS BR (1)
Couto Pt (1)	UNIVs SAO CARLOS BR (1)
Chaves Acsd (1)	UNIVs SAO CARLOS BR (1)
Antoniassi R (1)	UNIVs SAO CARLOS BR (1)
Alexander R (1)	UNIVs SAO CARLOS BR (1)
Adorno Mat (11)	UNIVs SAO CARLOS BR (10)
Rosa Prf (2)	UNIVs SAO CARLOS BR (2)
Piazza Rmf (2)	UNIVs SAO CARLOS BR (2)
Moreira Cg (2)	UNIVs SAO CARLOS BR (2)
Morales Mlv (2)	UNIVs SAO CARLOS BR (2)

Duque Alrf (2)	UNIVs SAO CARLOS BR (2)
da silva Ma (2)	UNIVs SAO CARLOS BR (2)
Bianchi F (6)	UNIVs SAO CARLOS BR (4)
Sakamoto Ik (7)	UNIVs SAO CARLOS BR (6)
Vet Lem (1)	UNIVs SAO PAULO BR (1)
Rocha faria duque Al (1)	UNIVs SAO PAULO BR (1)
Ooki Gn (1)	UNIVs SAO PAULO BR (1)
Oddi S (1)	UNIVs SAO PAULO BR (1)
Mendes Lw (1)	UNIVs SAO PAULO BR (1)
Luz Rl (1)	UNIVs SAO PAULO BR (1)
Lucheta Ar (1)	UNIVs SAO PAULO BR (1)
Kuramae Ee (1)	UNIVs SAO PAULO BR (1)
Kruger Mf (1)	UNIVs SAO PAULO BR (1)
Jonathan M (1)	UNIVs SAO PAULO BR (1)
Isay saad Sm (1)	UNIVs SAO PAULO BR (1)
Huber P (1)	UNIVs SAO PAULO BR (1)
Gombossy de melo franco Bd (1)	UNIVs SAO PAULO BR (1)
Furtado Dn (1)	UNIVs SAO PAULO BR (1)
Fernandes Tv (1)	UNIVs SAO PAULO BR (1)
Dimitrov Mr (1)	UNIVs SAO PAULO BR (1)
de souza Cb (1)	UNIVs SAO PAULO BR (1)
de oliva-neto P (1)	UNIVs SAO PAULO BR (1)
de figueiredo Fc (1)	UNIVs SAO PAULO BR (1)
De carvalho Kg (1)	UNIVs SAO PAULO BR (1)
de barros ranke Ff (1)	UNIVs SAO PAULO BR (1)
da silva Ghr (1)	UNIVs SAO PAULO BR (1)
Costa Mgm (1)	UNIVs SAO PAULO BR (1)
Sivieri K (16)	UNIVs SAO PAULO BR (14)
Saad Smi (14)	UNIVs SAO PAULO BR (14)
Padilha M (2)	UNIVs SAO PAULO BR (2)
Battistini C (2)	UNIVs SAO PAULO BR (2)
Salgaço Mk (3)	UNIVs SAO PAULO BR (3)
Vieira Ads (5)	UNIVs SAO PAULO BR (4)
Bedani R (4)	UNIVs SAO PAULO BR (4)
Rossi Ea (6)	UNIVs SAO PAULO BR (5)
Yoon Hs (1)	UNIVs SEOUL KR (1)
Yoo Jg (1)	UNIVs SEOUL KR (1)
Yong Hi (1)	UNIVs SEOUL KR (1)
Yi B (1)	UNIVs SEOUL KR (1)
Sung Jh (1)	UNIVs SEOUL KR (1)
Shim Ky (1)	UNIVs SEOUL KR (1)
Seong H (1)	UNIVs SEOUL KR (1)
Rha C-s (1)	UNIVs SEOUL KR (1)
Park S (1)	UNIVs SEOUL KR (1)
Park J-s (1)	UNIVs SEOUL KR (1)
Oh M-h (1)	UNIVs SEOUL KR (1)
Nam Tg (1)	UNIVs SEOUL KR (1)
Lee T (1)	UNIVs SEOUL KR (1)
Lee Jh (1)	UNIVs SEOUL KR (1)
Lee Hj (1)	UNIVs SEOUL KR (1)
Lee Eh (1)	UNIVs SEOUL KR (1)
Ko G (2)	UNIVs SEOUL KR (1)
Kim Wj (1)	UNIVs SEOUL KR (1)
Kim Sh (1)	UNIVs SEOUL KR (1)
Kim M (2)	UNIVs SEOUL KR (1)
Kim Jy (1)	UNIVs SEOUL KR (1)
Kim J-h (1)	UNIVs SEOUL KR (1)
Kim D-o (1)	UNIVs SEOUL KR (1)
Kang S-s (1)	UNIVs SEOUL KR (1)
Kang K (1)	UNIVs SEOUL KR (1)
Jung Ys (1)	UNIVs SEOUL KR (1)
Jo C (1)	UNIVs SEOUL KR (1)
Jin Y-i (1)	UNIVs SEOUL KR (1)
Jeong Y (1)	UNIVs SEOUL KR (1)

Jeong J-c (1)	UNIVs SEOUL KR (1)
Jang D (1)	UNIVs SEOUL KR (1)
Hong Sw (1)	UNIVs SEOUL KR (1)
Hoang H-h (1)	UNIVs SEOUL KR (1)
Ham J-s (1)	UNIVs SEOUL KR (1)
Ha Sk (1)	UNIVs SEOUL KR (1)
Chung Ks (1)	UNIVs SEOUL KR (1)
Choi I (1)	UNIVs SEOUL KR (1)
Bae Hj (1)	UNIVs SEOUL KR (1)
Lee S (4)	UNIVs SEOUL KR (2)
Chang Yh (2)	UNIVs SEOUL KR (2)
Shanmugam S (1)	UNIVs SERGIPE BR (1)
Santos de oliveira C (1)	UNIVs SERGIPE BR (1)
Pereira Uc (1)	UNIVs SERGIPE BR (1)
de oliveira Cs (1)	UNIVs SERGIPE BR (1)
Santana andrade Jk (2)	UNIVs SERGIPE BR (2)
Narain N (2)	UNIVs SERGIPE BR (2)
Gualberto Nc (2)	UNIVs SERGIPE BR (2)
Chagas barros Rg (2)	UNIVs SERGIPE BR (2)
Moreno I (1)	UNIVs SEVILLE ES (1)
Martín-carrasco I (1)	UNIVs SEVILLE ES (1)
Hinojosa Mg (1)	UNIVs SEVILLE ES (1)
Carbonero-aguilar P (1)	UNIVs SEVILLE ES (1)
Bautista J (1)	UNIVs SEVILLE ES (1)
Aguilera-velázquez Jr (1)	UNIVs SEVILLE ES (1)
Xu N (1)	UNIVs SHAANXI CN (1)
Shang X (1)	UNIVs SHAANXI CN (1)
Sa Z (1)	UNIVs SHAANXI CN (1)
Pang B (1)	UNIVs SHAANXI CN (1)
Nie C (1)	UNIVs SHAANXI CN (1)
Ma Z (1)	UNIVs SHAANXI CN (1)
Ma Q (1)	UNIVs SHAANXI CN (1)
Cui W (1)	UNIVs SHAANXI CN (1)
Yan X (3)	UNIVs SHAANXI CN (2)
Li J (5)	UNIVs SHAANXI CN (2)
Hu X (3)	UNIVs SHAANXI CN (2)
Yin X (1)	UNIVs SHANDONG CN (1)
Wei X (1)	UNIVs SHANDONG CN (1)
Wang B (1)	UNIVs SHANDONG CN (1)
Tian Y (1)	UNIVs SHANDONG CN (1)
Shi L (2)	UNIVs SHANDONG CN (1)
Kong J (1)	UNIVs SHANDONG CN (1)
Guo T (1)	UNIVs SHANDONG CN (1)
Gao S (1)	UNIVs SHANDONG CN (1)
Fan Y (1)	UNIVs SHANDONG CN (1)
Dong Y (1)	UNIVs SHANDONG CN (1)
Cui J (2)	UNIVs SHANDONG CN (1)
Wang C (2)	UNIVs SHANDONG CN (2)
Li N (3)	UNIVs SHANDONG CN (2)
Hao Y (2)	UNIVs SHANDONG CN (2)
Liu Y (10)	UNIVs SHANDONG CN (3)
Zhu C (1)	UNIVs SHANGHAI CN (1)
Zhou Y (1)	UNIVs SHANGHAI CN (1)
Xu S (1)	UNIVs SHANGHAI CN (1)
Xiong Y (1)	UNIVs SHANGHAI CN (1)
Xing S (1)	UNIVs SHANGHAI CN (1)
Xie C (1)	UNIVs SHANGHAI CN (1)
Xie B (1)	UNIVs SHANGHAI CN (1)
Tang C (1)	UNIVs SHANGHAI CN (1)
Tan B (1)	UNIVs SHANGHAI CN (1)
Song Z (1)	UNIVs SHANGHAI CN (1)
Sheng Y (1)	UNIVs SHANGHAI CN (1)
Sheng G (1)	UNIVs SHANGHAI CN (1)
Lv Z (1)	UNIVs SHANGHAI CN (1)
Lu M (1)	UNIVs SHANGHAI CN (1)

Jiang Q (1)	UNIVs SHANGHAI CN (1)
Hu C (1)	UNIVs SHANGHAI CN (1)
Han S (1)	UNIVs SHANGHAI CN (1)
Guo R (1)	UNIVs SHANGHAI CN (1)
Fu J (1)	UNIVs SHANGHAI CN (1)
Chen Q (1)	UNIVs SHANGHAI CN (1)
Ai L (1)	UNIVs SHANGHAI CN (1)
Ahmad W (1)	UNIVs SHANGHAI CN (1)
Zhao L (6)	UNIVs SHANGHAI CN (2)
Peng Y (2)	UNIVs SHANGHAI CN (2)
Zhang X (11)	UNIVs SHANGHAI CN (3)
Chen Y (7)	UNIVs SHANGHAI CN (3)
Li X (19)	UNIVs SHANGHAI CN (4)
Read Nw (1)	UNIVs SHEFFIELD GB (1)
Duerden Bi (1)	UNIVs SHEFFIELD GB (1)
Zou L (1)	UNIVs SICHUAN CN (1)
Wu W (1)	UNIVs SICHUAN CN (1)
Wu H (1)	UNIVs SICHUAN CN (1)
Qin W (1)	UNIVs SICHUAN CN (1)
Liu A (1)	UNIVs SICHUAN CN (1)
Hu Y-c (1)	UNIVs SICHUAN CN (1)
Fu M-x (1)	UNIVs SICHUAN CN (1)
Duan X (1)	UNIVs SICHUAN CN (1)
Duan S (1)	UNIVs SICHUAN CN (1)
Wang S-p (2)	UNIVs SICHUAN CN (2)
Nie X-r (2)	UNIVs SICHUAN CN (2)
Huang X (2)	UNIVs SICHUAN CN (2)
Yuan Q (3)	UNIVs SICHUAN CN (3)
Guo H (3)	UNIVs SICHUAN CN (3)
Fu Y (7)	UNIVs SICHUAN CN (3)
Wu D-t (4)	UNIVs SICHUAN CN (4)
Gan R-y (4)	UNIVs SICHUAN CN (4)
Zhuang W-q (1)	UNIVs SINGAPORE SG (1)
Yi S (1)	UNIVs SINGAPORE SG (1)
Wang J-y (1)	UNIVs SINGAPORE SG (1)
Vigneswaran S (1)	UNIVs SINGAPORE SG (1)
Stabnikova Ov (1)	UNIVs SINGAPORE SG (1)
Rice Sa (1)	UNIVs SINGAPORE SG (1)
Leiknes T (1)	UNIVs SINGAPORE SG (1)
Keshvardoust P (1)	UNIVs SINGAPORE SG (1)
Jeong S (1)	UNIVs SINGAPORE SG (1)
Ivanov Vn (1)	UNIVs SINGAPORE SG (1)
Ivanov V (1)	UNIVs SINGAPORE SG (1)
Cho K (1)	UNIVs SINGAPORE SG (1)
Bae H (1)	UNIVs SINGAPORE SG (1)
Tay St-l (2)	UNIVs SINGAPORE SG (2)
Tay J-h (2)	UNIVs SINGAPORE SG (2)
Wall-medranod A (1)	UNIVs SONORA MX (1)
Velderrain-roddríguez G (1)	UNIVs SONORA MX (1)
Sayagod-ayerdi S (1)	UNIVs SONORA MX (1)
Rodbles-sánchez Rm (1)	UNIVs SONORA MX (1)
Quirós-sauceda A (1)	UNIVs SONORA MX (1)
Mercadod-mercadod G (1)	UNIVs SONORA MX (1)
Godnzález-aguilar Ga (1)	UNIVs SONORA MX (1)
Ayala-zavala Jf (2)	UNIVs SONORA MX (1)
Astiazarán-garcía H (1)	UNIVs SONORA MX (1)
Wu X (7)	UNIVs SOOCHOW TW (2)
Kasapis S (1)	UNIVs SOUTH WALES GB (1)
Greve Lj (1)	UNIVs SOUTH WALES GB (1)
Brkljaca R (1)	UNIVs SOUTH WALES GB (1)
Berean Kj (1)	UNIVs SOUTH WALES GB (1)
Reid Dc (1)	UNIVs ST ANDREWS GB (1)
Mackay Wg (1)	UNIVs ST ANDREWS GB (1)
Gribbon Lt (1)	UNIVs ST ANDREWS GB (1)
Barer Mr (1)	UNIVs ST ANDREWS GB (1)

Stedman A (1)	UNIVs SURREY GB (1)
Maluquer de motes C (1)	UNIVs SURREY GB (1)
Lesellier S (1)	UNIVs SURREY GB (1)
Gutierrez-merino J (1)	UNIVs SURREY GB (1)
Dalley D (1)	UNIVs SURREY GB (1)
Chambers M (1)	UNIVs SURREY GB (1)
Xie H-n (1)	UNIVs TARRAGONA ES (1)
Pedro Sg-d (1)	UNIVs TARRAGONA ES (1)
Pazos-perez N (1)	UNIVs TARRAGONA ES (1)
Morla-folch J (1)	UNIVs TARRAGONA ES (1)
Guerrini L (1)	UNIVs TARRAGONA ES (1)
Gisbert-quilis P (1)	UNIVs TARRAGONA ES (1)
Alvarez-puebla Ra (1)	UNIVs TARRAGONA ES (1)
Noori N (1)	UNIVs TEHRAN IR (1)
Misaghi A (1)	UNIVs TEHRAN IR (1)
Gandomi H (1)	UNIVs TEHRAN IR (1)
Bokaei S (1)	UNIVs TEHRAN IR (1)
Abbaszadeh S (1)	UNIVs TEHRAN IR (1)
Versalovic J (2)	UNIVs TENNESSEE US (2)
Sterzo Cl (1)	UNIVs TERAMO IT (1)
Sayas-barberá E (1)	UNIVs TERAMO IT (1)
Ricci A (1)	UNIVs TERAMO IT (1)
Ribecco C (1)	UNIVs TERAMO IT (1)
Pignataro G (1)	UNIVs TERAMO IT (1)
Gramenzi A (1)	UNIVs TERAMO IT (1)
Pellegrini M (2)	UNIVs TERAMO IT (2)
Williams T (1)	UNIVs TEXAS US (1)
Stofilova J (1)	UNIVs TEXAS US (1)
Smolen Ja (1)	UNIVs TEXAS US (1)
Shatkin Ja (1)	UNIVs TEXAS US (1)
Sayes Cm (1)	UNIVs TEXAS US (1)
Savidge Tc (1)	UNIVs TEXAS US (1)
Pradhan Sh (1)	UNIVs TEXAS US (1)
Noratto G (1)	UNIVs TEXAS US (1)
Nderitu Am (1)	UNIVs TEXAS US (1)
Mulenos Mr (1)	UNIVs TEXAS US (1)
Mertens-talcott Su (1)	UNIVs TEXAS US (1)
Mcshane Mj (1)	UNIVs TEXAS US (1)
Lujan H (1)	UNIVs TEXAS US (1)
Kvakova M (1)	UNIVs TEXAS US (1)
Jeevarathinam As (1)	UNIVs TEXAS US (1)
Hyde Ja (1)	UNIVs TEXAS US (1)
Hachibamba T (1)	UNIVs TEXAS US (1)
Gibb M (1)	UNIVs TEXAS US (1)
Ede Jd (1)	UNIVs TEXAS US (1)
de figueiredo P (1)	UNIVs TEXAS US (1)
Chew B (1)	UNIVs TEXAS US (1)
Bertkova I (1)	UNIVs TEXAS US (1)
Awika Jm (1)	UNIVs TEXAS US (1)
Awika J (1)	UNIVs TEXAS US (1)
Arbizu S (1)	UNIVs TEXAS US (1)
Alge Dl (1)	UNIVs TEXAS US (1)
Savidge T (2)	UNIVs TEXAS US (2)
Minnaar A (2)	UNIVs TEXAS US (2)
Dykes L (2)	UNIVs TEXAS US (2)
Britton Ra (2)	UNIVs TEXAS US (2)
Auchtung Jm (2)	UNIVs TEXAS US (2)
Walker K (2)	UNIVs TORONTO CA (1)
Shifrin Y (1)	UNIVs TORONTO CA (1)
Pan J (1)	UNIVs TORONTO CA (1)
Daigneault Mc (2)	UNIVs TORONTO CA (1)
Bottiglieri T (1)	UNIVs TORONTO CA (1)
Belik J (1)	UNIVs TORONTO CA (1)
Arning E (1)	UNIVs TORONTO CA (1)
Abou-samra E (1)	UNIVs TORONTO CA (1)

Beaumont M (3)	UNIVs TOULOUSE FR (3)
Traversi D (1)	UNIVs TURIN IT (1)
Romanazzi V (1)	UNIVs TURIN IT (1)
Lorenzi E (1)	UNIVs TURIN IT (1)
Gilli G (1)	UNIVs TURIN IT (1)
Tavilli E (1)	UNIVs TUSCIA IT (1)
Fidaleo M (1)	UNIVs TUSCIA IT (1)
Gill Cir (5)	UNIVs ULSTER GB (5)
Ugolini L (1)	UNIVs URBINO IT (1)
Feduzi L (1)	UNIVs URBINO IT (1)
Desideri D (1)	UNIVs URBINO IT (1)
Roselli C (2)	UNIVs URBINO IT (2)
Meli Ma (2)	UNIVs URBINO IT (2)
Sánchez-moreno C (1)	UNIVs VALENCIA ES (1)
Martínez-monzó J (1)	UNIVs VALENCIA ES (1)
Igual M (1)	UNIVs VALENCIA ES (1)
Gómez del pulgar Em (1)	UNIVs VALENCIA ES (1)
García-segovia P (1)	UNIVs VALENCIA ES (1)
Frígola A (1)	UNIVs VALENCIA ES (1)
Fernández-jalao I (2)	UNIVs VALENCIA ES (1)
Esteve Mj (1)	UNIVs VALENCIA ES (1)
Dolores álvarez M (1)	UNIVs VALENCIA ES (1)
Diez-sánchez E (1)	UNIVs VALENCIA ES (1)
de ancos B (1)	UNIVs VALENCIA ES (1)
Codoñer-franch P (1)	UNIVs VALENCIA ES (1)
Carbonell-capella Jm (1)	UNIVs VALENCIA ES (1)
Buniowska M (1)	UNIVs VALENCIA ES (1)
Benítez-páez A (1)	UNIVs VALENCIA ES (1)
Arilla E (1)	UNIVs VALENCIA ES (1)
Yebra Mj (2)	UNIVs VALENCIA ES (2)
Sentandreu V (2)	UNIVs VALENCIA ES (2)
Quiles A (2)	UNIVs VALENCIA ES (2)
Jilani H (2)	UNIVs VALENCIA ES (2)
Hernando I (2)	UNIVs VALENCIA ES (2)
Hamdi M (2)	UNIVs VALENCIA ES (2)
Cuevas-tena M (2)	UNIVs VALENCIA ES (2)
Cilla A (2)	UNIVs VALENCIA ES (2)
Blanco-morales V (2)	UNIVs VALENCIA ES (2)
Garcia-llatas G (3)	UNIVs VALENCIA ES (3)
Barberá R (3)	UNIVs VALENCIA ES (3)
Lagarda Mj (4)	UNIVs VALENCIA ES (4)
Alegria A (4)	UNIVs VALENCIA ES (4)
Rodríguez-rojo S (1)	UNIVs VALLADOLID ES (1)
Moreno T (1)	UNIVs VALLADOLID ES (1)
Cocero Mj (1)	UNIVs VALLADOLID ES (1)
Torriani S (1)	UNIVs VERONA IT (1)
Martina A (1)	UNIVs VERONA IT (1)
Maffeis C (1)	UNIVs VERONA IT (1)
Felis Ge (1)	UNIVs VERONA IT (1)
Corradi M (1)	UNIVs VERONA IT (1)
Ricardo de castro leite júnior B (1)	UNIVs VICOSA BR (1)
Ramos Am (1)	UNIVs VICOSA BR (1)
Narvaes da rocha campos A (1)	UNIVs VICOSA BR (1)
Montanari Sr (1)	UNIVs VICOSA BR (1)
Martins Ml (1)	UNIVs VICOSA BR (1)
Furtado martins Em (1)	UNIVs VICOSA BR (1)
Cristina de almeida bianchini campos R (1)	UNIVs VICOSA BR (1)
Binoti Ml (1)	UNIVs VICOSA BR (1)
Schoenlechner R (1)	UNIVs VIENNE AT (1)
Rawdkuen S (1)	UNIVs VIENNE AT (1)
Phongthai S (1)	UNIVs VIENNE AT (1)
Kulbe Kd (1)	UNIVs VIENNE AT (1)
Kneifel W (1)	UNIVs VIENNE AT (1)

Homthawornchoo W (1)	UNIVs VIENNE AT (1)
Gmeiner M (1)	UNIVs VIENNE AT (1)
D’amico S (1)	UNIVs VIENNE AT (1)
Vila C (2)	UNIVs VIGO ES (2)
Parajó Jc (2)	UNIVs VIGO ES (2)
Miguez B (2)	UNIVs VIGO ES (2)
Alonso Jl (2)	UNIVs VIGO ES (2)
Wiazecki K (1)	UNIVs WARSAW PL (1)
Trzaskowski M (1)	UNIVs WARSAW PL (1)
Szawkalo J (1)	UNIVs WARSAW PL (1)
Stanislawska Ij (1)	UNIVs WARSAW PL (1)
Ruzik L (1)	UNIVs WARSAW PL (1)
Piwowarski Jp (1)	UNIVs WARSAW PL (1)
Kiss Ak (1)	UNIVs WARSAW PL (1)
Hayder M (1)	UNIVs WARSAW PL (1)
Granica S (1)	UNIVs WARSAW PL (1)
Czarnocki Z (1)	UNIVs WARSAW PL (1)
Van loosdrecht Mcm (1)	UNIVs WASHINGTON US (1)
Salam Kw (1)	UNIVs WASHINGTON US (1)
Saikaly Pe (1)	UNIVs WASHINGTON US (1)
Pronk M (1)	UNIVs WASHINGTON US (1)
Lampe Jw (1)	UNIVs WASHINGTON US (1)
Konopka Ae (1)	UNIVs WASHINGTON US (1)
Kim Y-m (1)	UNIVs WASHINGTON US (1)
Hullar Maj (1)	UNIVs WASHINGTON US (1)
Hari Ar (1)	UNIVs WASHINGTON US (1)
Eng A (1)	UNIVs WASHINGTON US (1)
Callister Sj (1)	UNIVs WASHINGTON US (1)
Borenstein E (1)	UNIVs WASHINGTON US (1)
Boaro Aa (1)	UNIVs WASHINGTON US (1)
Beresford Saa (1)	UNIVs WASHINGTON US (1)
Ali M (1)	UNIVs WASHINGTON US (1)
Ahring Bk (1)	UNIVs WASHINGTON US (1)
Pi X (1)	UNIVs WEST VIRGINIA US (1)
Zhu B (2)	UNIVs WEST VIRGINIA US (2)
Lei F (2)	UNIVs WEST VIRGINIA US (2)
Yu Hd (3)	UNIVs WEST VIRGINIA US (3)
Scarborough Mj (1)	UNIVs WISCONSIN US (1)
Mcgee M (1)	UNIVs WISCONSIN US (1)
Lynch G (1)	UNIVs WISCONSIN US (1)
Dickson M (1)	UNIVs WISCONSIN US (1)
Noguera Dr (2)	UNIVs WISCONSIN US (2)
Donohue Tj (2)	UNIVs WISCONSIN US (2)
Peters U (1)	UNIVs WITTEN-HERDECKE DE (1)
Martin D (1)	UNIVs WITTEN-HERDECKE DE (1)
Klement P (1)	UNIVs WITTEN-HERDECKE DE (1)
Keller T (1)	UNIVs WITTEN-HERDECKE DE (1)
Buchheim-schmidt S (1)	UNIVs WITTEN-HERDECKE DE (1)
Baumgartner S (1)	UNIVs WITTEN-HERDECKE DE (1)
Ren J (1)	UNIVs XINJIANG CN (1)
Jialengbieke B (1)	UNIVs XINJIANG CN (1)
Fan L (1)	UNIVs XINJIANG CN (1)
Bao T (1)	UNIVs XINJIANG CN (1)
Zhou J (2)	UNIVs XINJIANG CN (2)
Zhu Y (3)	UNIVs YUNNAN CN (2)
Huang Y (4)	UNIVs YUNNAN CN (2)
Ye K (1)	UNIVs ZHEJIANG CN (1)
Xing H (1)	UNIVs ZHEJIANG CN (1)
Xie H (1)	UNIVs ZHEJIANG CN (1)
Watharkar Rb (1)	UNIVs ZHEJIANG CN (1)
Tian S (1)	UNIVs ZHEJIANG CN (1)
Sun P (1)	UNIVs ZHEJIANG CN (1)
Song R (1)	UNIVs ZHEJIANG CN (1)
Shou Y (1)	UNIVs ZHEJIANG CN (1)
Shi M (1)	UNIVs ZHEJIANG CN (1)

Ren G (1)	UNIVs ZHEJIANG CN (1)
Pu Y (1)	UNIVs ZHEJIANG CN (1)
Ni F (1)	UNIVs ZHEJIANG CN (1)
Mu H (1)	UNIVs ZHEJIANG CN (1)
Lei Q (1)	UNIVs ZHEJIANG CN (1)
Karim N (1)	UNIVs ZHEJIANG CN (1)
Ismail Bb (1)	UNIVs ZHEJIANG CN (1)
Gu L (1)	UNIVs ZHEJIANG CN (1)
Ge Y (1)	UNIVs ZHEJIANG CN (1)
Fu D (1)	UNIVs ZHEJIANG CN (1)
Fang W (1)	UNIVs ZHEJIANG CN (1)
Ding T (1)	UNIVs ZHEJIANG CN (1)
Chai J (1)	UNIVs ZHEJIANG CN (1)
Çavus O (1)	UNIVs ZHEJIANG CN (1)
Cai M (1)	UNIVs ZHEJIANG CN (1)
Bao X (1)	UNIVs ZHEJIANG CN (1)
Ayub Ka (1)	UNIVs ZHEJIANG CN (1)
Zheng X (2)	UNIVs ZHEJIANG CN (2)
Xiang S (2)	UNIVs ZHEJIANG CN (2)
Han J (5)	UNIVs ZHEJIANG CN (2)
Gowd V (2)	UNIVs ZHEJIANG CN (2)
Feng X (4)	UNIVs ZHEJIANG CN (2)
Chen H (10)	UNIVs ZHEJIANG CN (2)
Zhu X (3)	UNIVs ZHEJIANG CN (3)
Liu W (9)	UNIVs ZHEJIANG CN (3)
Yin Y (5)	UNIVs ZHEJIANG CN (4)
Wang X (15)	UNIVs ZHEJIANG CN (4)
Chen J (7)	UNIVs ZHEJIANG CN (4)
Stevens Mja (1)	UNIVs ZURICH CH (1)
Hegland A (1)	UNIVs ZURICH CH (1)
Braegger Cp (1)	UNIVs ZURICH CH (1)
Von wright A (4)	VTT FOOD RES P O BOX FIN VTT FINLAND (2)
Mattila-sandholm T (4)	VTT FOOD RES P O BOX FIN VTT FINLAND (2)
Niemelä K (2)	VTT TECH RES CTR FINLAND ESPOO FINLAND (2)
Hyötyläinen T (2)	VTT TECH RES CTR FINLAND ESPOO FINLAND (2)
Gopalacharyulu P (2)	VTT TECH RES CTR FINLAND ESPOO FINLAND (2)
Tamminen T (3)	VTT TECH RES CTR FINLAND ESPOO FINLAND (3)
Orešic M (3)	VTT TECH RES CTR FINLAND ESPOO FINLAND (3)
Niemi P (3)	VTT TECH RES CTR FINLAND ESPOO FINLAND (3)
Buchert J (3)	VTT TECH RES CTR FINLAND ESPOO FINLAND (3)
Mattila I (9)	VTT TECH RES CTR FINLAND ESPOO FINLAND (6)
Aura A-m (14)	VTT TECH RES CTR FINLAND ESPOO FINLAND (7)
Sözer N (1)	VTT TECH RES CTR FINLAND P O BOX FI VTT FINLAND (1)
Hakala Tk (1)	VTT TECH RES CTR FINLAND P O BOX FI VTT FINLAND (1)
Nordlund E (5)	VTT TECH RES CTR FINLAND P O BOX FI VTT FINLAND (2)
van dongen Kcw (1)	WAGENINGEN FOOD SAFETY RES WFSR PART WAGENINGEN UNIV RES P O BOX WAGENINGEN AE NETHERLANDS (1)
van der zande M (1)	WAGENINGEN FOOD SAFETY RES WFSR PART WAGENINGEN UNIV RES P O BOX WAGENINGEN AE NETHERLANDS (1)
Rietjens Imcm (1)	WAGENINGEN FOOD SAFETY RES WFSR PART WAGENINGEN UNIV RES P O BOX WAGENINGEN AE NETHERLANDS (1)
Bruyneel B (1)	WAGENINGEN FOOD SAFETY RES WFSR PART WAGENINGEN UNIV RES P O BOX WAGENINGEN AE NETHERLANDS (1)
Beekmann K (1)	WAGENINGEN FOOD SAFETY RES WFSR PART WAGENINGEN UNIV RES P O BOX WAGENINGEN AE NETHERLANDS (1)
Deutz Nep (2)	WAGENINGEN NMR CTR NETHERLANDS (2)
Mank M (2)	WAGENINGEN UNIV RES WAGENINGEN WE NETHERLANDS (2)
Knol J (2)	WAGENINGEN UNIV RES WAGENINGEN WE NETHERLANDS (2)
Chia Lw (2)	WAGENINGEN UNIV RES WAGENINGEN WE NETHERLANDS (2)
Bongers Rs (2)	WAGENINGEN UNIV RES WAGENINGEN WE NETHERLANDS (2)
Blijenberg B (2)	WAGENINGEN UNIV RES WAGENINGEN WE NETHERLANDS (2)
Vervoort Jjm (2)	WAGENINGEN UNIV WAGENINGEN NETHERLANDS (2)
Plugge Cm (2)	WAGENINGEN UNIV WAGENINGEN NETHERLANDS (2)
Martinez Rcr (3)	WAGENINGEN UNIV WAGENINGEN NETHERLANDS (2)
Jacobs Dm (2)	WAGENINGEN UNIV WAGENINGEN NETHERLANDS (2)

Gomez-roldan V (2)	WAGENINGEN UNIV WAGENINGEN NETHERLANDS (2)
Egert M (2)	WAGENINGEN UNIV WAGENINGEN NETHERLANDS (2)
Zoetendal Eg (4)	WAGENINGEN UNIV WAGENINGEN NETHERLANDS (3)
Belzer C (6)	WAGENINGEN UNIV WAGENINGEN NETHERLANDS (3)
Kleerebezem M (4)	WAGENINGEN UNIV WAGENINGEN NETHERLANDS (4)
Smidt H (8)	WAGENINGEN UNIV WAGENINGEN NETHERLANDS (7)
De vos Wm (10)	WAGENINGEN UNIV WAGENINGEN NETHERLANDS (8)
Wichers Hj (2)	WESTERN DIGITAL US (1)
Veldkamp T (1)	WESTERN DIGITAL US (1)
Mes Jj (1)	WESTERN DIGITAL US (1)
Govers C (2)	WESTERN DIGITAL US (1)
Ariëns Rmc (2)	WESTERN DIGITAL US (1)
America Ahp (1)	WESTERN DIGITAL US (1)
Trchounian A (1)	YEREVAN STATE UNIV YEREVAN ARMENIA (1)
Soghomonyan D (1)	YEREVAN STATE UNIV YEREVAN ARMENIA (1)
Blatchford P (2)	ZESPRI INTERNATIONAL LTD PO BOX MT MAUNGANUI NEW ZEALAND (2)

* Access to Interllixir program https://carlac.intellixir.fr/cenm provided by The Universidad del Valle

Table S12: New terms introduced per year. List was retrieved with interllixir program using the same list of documents.

Concept	Doc.Type	Total occurrence (current study, excluding time settings)
**Year 2021**		
Gl	All	5
Microbiome modulation	All	5
**Year 2020**		
Fermentation characteristic	All	6
Phascolarctobacterium	All	6
Phylum level	All	6
Collinsella	All	5
Decrease ph	All	5
Firmicute bacteroidete ratio	All	5
Fulltext	All	5
Reactive oxygen specy	All	5
Vch gmbh	All	5
**Year 2019**		
Reduce sugar	All	11
Increase abundance	All	9
Antibiotic resistance gene	All	8
Microbiome model	All	8
Gastrointestinal resistance	All	7
Molar ratio	All	7
Uv	All	7
Burden	All	6
Encapsulation efficiency	All	6
Goblet	All	6
Mill	All	6
Bf	All	5
Fl	All	5
Metabolic disease	All	5
Microbial taxa	All	5
Microwave	All	5
Pollution	All	5
Resistant bacteria	All	5
Simulate git condition	All	5
Simulate human adult	All	5
Sugar content	All	5
Total polyphenol content	All	5
Untargete metabolomic	All	5
Vitro microbiota	All	5
**Year 2018**		
Clostridioide	All	14
Clostridioide difficile	All	13
Hmos	All	11
Qtof	All	10
Tpc	All	10
Clostridioide difficile infection	All	9
Modulator	All	9
Fucosyllactose	All	8
Megamonas	All	8
Infrared	All	7
Prevotellaceae	All	7
Beta diversity	All	6
Correlation analysis	All	6
Ferric reduce	All	6
Hmo	All	6
Lachnospira	All	6
Ros	All	6
Sterol	All	6
Succinic	All	6
Bb 46	All	5
Bifidobacterium longum bb 46	All	5

Blackberry	All	5
Chip model	All	5
Content increase	All	5
Data support	All	5
Di	All	5
Dorea	All	5
Dw	All	5
Emphasize	All	5
Enterotoxigenic escherichia coli	All	5
Erysipelotrichaceae	All	5
Etec	All	5
Ferric reduce antioxidant power	All	5
Fibroblast	All	5
Gcm	All	5
Generation probiotic	All	5
Gut microbiota fermentation	All	5
Human individual	All	5
Intestinal stage	All	5
Longum bb 46	All	5
Md	All	5
Microbial consortia	All	5
Occludin	All	5
Potential prebiotic property	All	5
Propionate produce	All	5
Stool sample	All	5
Wpi	All	5
Zo 1	All	5
**Year 2017**		
Generation sequence	All	11
Firmicute bacteroidete	All	10
Urolithin	All	9
Vitro simulate gastrointestinal digestion	All	9
Coprococcus	All	8
Novel prebiotic	All	8
Prebiotic ingredient	All	8
Snack	All	8
Antioxidant power	All	7
Inhabit	All	7
Mrna	All	7
Soy protein	All	7
Tio2	All	7
Trad	All	7
Transporter	All	7
Vitro gastrointestinal condition	All	7
Caco 2 cell model	All	6
Company	All	6
Gut microbiota metabolism	All	6
Healthy food	All	6
Metabolic syndrome	All	6
Sutterella	All	6
Vitro fecal fermentation	All	6
Caffeoylquinic acid	All	5
Com	All	5
Cu	All	5
Food additive	All	5
Http	All	5
Human gut microbiota composition	All	5
Ice cream	All	5
Large intestine fermentation	All	5
Major role	All	5
Microbiota diversity	All	5
Non encapsulate	All	5
Seafood	All	5
Signal pathway	All	5
Simulate gastrointestinal model	All	5

Soy protein isolate	All	5
Stem	All	5
Tandem	All	5
Titanium dioxide	All	5
Variance	All	5
**Year 2016**		
Taxa	All	20
Human milk oligosaccharide	All	16
Nanoparticle	All	16
Lachnospiraceae	All	15
Lipolysis	All	14
Bifidobacteriaceae	All	13
Chlorogenic	All	13
Hydroxycinnamic acid	All	13
Chlorogenic acid	All	11
Microbiome composition	All	11
Multivariate	All	10
Np	All	10
Polyphenol content	All	10
Green tea	All	9
Lactobacillaceae	All	9
Leaf	All	9
Nm	All	9
Shigella	All	9
Untargete	All	9
Caffeic acid	All	8
Ellagitannin	All	8
Hazard	All	8
Human adult	All	8
Microparticle	All	8
Miseq	All	8
Dietary exposure	All	7
Inform	All	7
Microbiota population	All	7
Oxygen radical absorbance capacity	All	7
Positive correlation	All	7
Rutin	All	7
Bacteroidaceae	All	6
Gastric digest	All	6
Genus bifidobacterium	All	6
Genus level	All	6
Insect	All	6
Malondialdehyde	All	6
Microbiota community	All	6
Nano	All	6
Nh4	All	6
Pathogenicity	All	6
Polyunsaturated fatty acid	All	6
Structural characteristic	All	6
Trolox	All	6
Vitro digest	All	6
Whey protein isolate	All	6
Mg	All	6
Bilophila	All	5
Carotenoid bioaccessibility	All	5
Cellular uptake	All	5
Chemistry 2016	All	5
Co encapsulate	All	5
Colon microbiome	All	5
Data analysis	All	5
Dihydrocaffeic	All	5
Expression level	All	5
Fiber content	All	5
Gastrointestinal health	All	5
Gut epithelium	All	5

Intestinal level	All	5
Miseq sequence	All	5
Muscle	All	5
Natural source	All	5
Novel therapeutic	All	5
Pellet	All	5
Pepsin activity	All	5
Persimmon	All	5
Phenylalanine	All	5
Protein hydrolysis	All	5
Restoration	All	5
Room temperature	All	5
Vitro gastrointestinal digestion model	All	5
Xylooligosaccharide	All	5
**Year 2015**		
Oxygen	All	23
Human milk	All	22
Akkermansia muciniphila	All	18
Muciniphila	All	18
Hydroxycinnamic	All	17
Uhplc	All	17
Human gut microbiome	All	16
Alpha amylase	All	15
Gae	All	14
Macrophage	All	14
Mango	All	14
Ruminococcaceae	All	14
Amplicon sequence	All	13
Colon phase	All	13
Resistance gene	All	13
Aggregation	All	12
Gastrointestinal simulation	All	12
Microfluidic	All	12
Phenolic profile	All	12
Ellagic	All	11
Monosaccharide	All	11
Parabacteroide	All	11
Vitro simulate digestion	All	11
Benzoic	All	10
Intestinal health	All	10
Simgi	All	10
Benzoic acid	All	9
Ellagic acid	All	9
Static model	All	9
Control sample	All	8
Gid	All	8
Gluten	All	8
Mother	All	8
Recognition	All	8
Rrna gene amplicon sequence	All	8
Vitro gastrointestinal simulation	All	8
Attribution	All	7
Bioaccessibility index	All	7
Biopolymer	All	7
Cultivar	All	7
Driver	All	7
Elisa	All	7
Food grade	All	7
Habitat	All	7
Phenylacetic acid	All	7
Verrucomicrobia	All	7
Ammonium ion	All	6
Chemokine	All	6
Credit	All	6
Dynamic gastrointestinal simulator	All	6

Endothelial cell	All	6
Fecal water	All	6
Gi digestion	All	6
Gut commensal	All	6
Intestinal permeability	All	6
Linkage	All	6
Mangifera indica	All	6
Microbial dysbiosis	All	6
Nf kappab	All	6
Operational taxonomic unit	All	6
Optimal condition	All	6
Original author	All	6
Pasta	All	6
Peptide release	All	6
Pma	All	6
Pomegranate	All	6
Protect probiotic	All	6
Translate	All	6
Unrestrict	All	6
Acacia gum	All	5
Ammonium level	All	5
Ataulfo	All	5
Basolateral	All	5
Blautia	All	5
Collagen	All	5
Coordinate	All	5
Cultivable	All	5
Dietary strategy	All	5
Digestible fraction	All	5
Expression analysis	All	5
Foundation	All	5
Hemolytic	All	5
Ib	All	5
Il 1beta	All	5
Interferon	All	5
Intestinal inflammation	All	5
Lab isolate	All	5
Lactate dehydrogenase	All	5
Major phenolic	All	5
Mangifera indica l	All	5
Nam	All	5
New therapeutic	All	5
Non digestible fraction	All	5
Orbitrap	All	5
Positive modulation	All	5
Production process	All	5
Promise probiotic	All	5
Proof	All	5
Teer	All	5
Vanillic	All	5
Var	All	5
**Year 2014**		
Antibiotic resistance	All	19
Score	All	17
Hand	All	15
Tno	All	15
Il 8	All	14
Interplay	All	14
Biomarker	All	13
Infogest	All	13
Ac	All	12
Barrier integrity	All	12
Dad	All	12
Diabetes	All	12
Heat treatment	All	12

Alpha diversity	All	11
Caffeic	All	11
Characterisation	All	11
Differentiation	All	11
Fmt	All	11
Metagenomic	All	11
Placebo	All	11
Rrna gene amplicon	All	11
Byproduct	All	10
Fecal microbiota transplantation	All	10
Imbalance	All	10
Immune cell	All	10
Pathogenic bacteria	All	10
Pomace	All	10
Positive control	All	10
Bean	All	9
Gallate	All	9
Human donor	All	9
Illumina	All	9
Phenylacetic	All	9
Protein digestibility	All	9
Shelf life	All	9
Consensus	All	8
Glycan	All	8
Lc ms ms	All	8
Olive oil	All	8
Recurrent cdi	All	8
Transwell	All	8
Antiproliferative	All	7
Emulsifier	All	7
Flavor	All	7
Food formulation	All	7
Gastric lipase	All	7
Gastro intestinal digestion	All	7
Hplc dad	All	7
Increment	All	7
Microbiota metabolism	All	7
Nanomaterial	All	7
Physical property	All	7
Probiotic characteristic	All	7
Raw material	All	7
Tyrosol	All	7
Anthocyanin content	All	6
Banana	All	6
Birth	All	6
Control trial	All	6
Dynamic system	All	6
Epigallocatechin	All	6
Increase acetate	All	6
Inflammatory disease	All	6
Liberate	All	6
Microbial interaction	All	6
Simulate gastric digestion	All	6
Tlr	All	6
Transcriptome	All	6
Tween	All	6
Adverse condition	All	5
Antiproliferative activity	All	5
Bb12	All	5
Blueberry	All	5
Cassava	All	5
Egcg	All	5
Enzyme link immunosorbent	All	5
Gnotobiotic	All	5
Goat	All	5

Hinder	All	5
Ibd patient	All	5
Insulin	All	5
Insulin resistance	All	5
Intestinal enzyme	All	5
Physicochemical parameter	All	5
Quinoa	All	5
Satiety	All	5
Simulate clostridium difficile infection	All	5
Static digestion	All	5
Survey	All	5
Syringic	All	5
Water emulsion	All	5
**Year 2013**		
Antioxidant activity	All	59
Age	All	33
Total phenolic	All	30
Phenolic content	All	23
Frap	All	19
Vitro simulate gastrointestinal	All	18
Abt	All	17
Actinobacteria	All	17
Hydroxybenzoic	All	17
Hydroxybenzoic acid	All	15
Motility	All	15
Mucosal simulator	All	15
Total phenolic content	All	15
Flavonol	All	14
Protein isolate	All	13
Orac	All	12
Bioactive peptide	All	11
Consortia	All	11
Host health	All	11
Intestinal microbiome	All	11
Trigger	All	11
Unravel	All	11
Agave	All	10
Dynamic simulator	All	10
Faecal fermentation	All	10
Niche	All	10
Storage period	All	10
Vitro batch fermentation	All	10
Endpoint	All	9
Favor	All	9
Functional food ingredient	All	9
Hrm	All	9
Metabolite produce	All	9
Microbiota modulation	All	9
Polyferm	All	9
Radical scavenge activity	All	9
Uplc	All	9
Alleviate	All	8
Bacteroidete ratio	All	8
Clostridium cluster xiva	All	8
Cream	All	8
Digest fraction	All	8
Food process	All	8
Guar gum	All	8
Highest antioxidant	All	8
Metabolite profile	All	8
Mw	All	8
Probiotic survival	All	8
Probiotic viability	All	8
Public health	All	8
Suitability	All	8

Vitro simulate gastrointestinal condition	All	8
Anti inflammatory activity	All	7
Bioconversion	All	7
Gut barrier	All	7
New york	All	7
Ol	All	7
Pasteurization	All	7
Soymilk	All	7
Storage stability	All	7
Topic	All	7
Weissella	All	7
Acetylate	All	6
Antioxidant property	All	6
Business media new york	All	6
Ceftriaxone	All	6
Circulation	All	6
Coffee	All	6
Ep	All	6
Glycemic index	All	6
Hp	All	6
Human fecal inocula	All	6
Indigenous gut microbiota	All	6
Korean society	All	6
Metabolic process	All	6
Metabolic response	All	6
Microbial phenolic metabolite	All	6
Model inoculate	All	6
Negative control	All	6
Phenolic composition	All	6
Probiotic lactobacillus acidophilus	All	6
Probiotic microorganism	All	6
Resolution mass spectrometry	All	6
Sh	All	6
Tea polyphenol	All	6
Agave fructan	All	5
Antimicrobial concentration	All	5
Bifidobacterium pseudocatenulatum	All	5
Brew	All	5
Brewer	All	5
Caccae	All	5
Decade	All	5
Fecal microbial community	All	5
Fortify	All	5
Gut microbial composition	All	5
Gut microbial ecosystem	All	5
Improve gut health	All	5
Increase metabolic activity	All	5
International society	All	5
Metabolic fate	All	5
Microbial derive	All	5
Oxidative dna damage	All	5
Polyferm model	All	5
Situ hybridisation	All	5
Spent grain	All	5
Subpopulation	All	5
Substitution	All	5
Vitro tool	All	5
Wine polyphenol	All	5
**Year 2012**		
Oil	All	32
Tea	All	23
Emulsion	All	22
Gallic	All	22
Food science	All	21
Ion	All	21

M shime	All	21
Fecal fermentation	All	20
Vitamin	All	20
Cell model	All	18
Gallic acid	All	18
Lipase	All	17
Lp	All	16
Nutraceutical	All	16
Mechanistic	All	15
Encapsulate probiotic	All	14
Potential health	All	14
Bacterial composition	All	12
Human cell	All	12
Red wine	All	12
Richness	All	12
Clarify	All	11
Complexe	All	11
Enhancement	All	11
Functional property	All	11
Gc ms	All	11
Human fecal sample	All	11
La5	All	11
Mucus layer	All	11
Pasteurize	All	11
Sd page	All	11
Survivability	All	11
Casei 01	All	10
Chronic disease	All	10
Colon fermentation	All	10
Dietary intervention	All	10
Intestinal juice	All	10
Pbet	All	10
Potential health benefit	All	10
Scientist	All	10
Ulcerative colitis	All	10
Urine	All	10
Acidophilus la5	All	9
Biofilm formation	All	9
Black tea	All	9
Control group	All	9
Exploit	All	9
Fermentation metabolite	All	9
Lactobacillus casei 01	All	9
Microbial degradation	All	9
Oral exposure	All	9
Oxidative stress	All	9
Proven	All	9
Streptococcus thermophilus	All	9
Antioxidant potential	All	8
Berry	All	8
Droplet	All	8
Exact	All	8
Immunity	All	8
Lactobacillus acidophilus la5	All	8
Lipid digestion	All	8
Mucosal environment	All	8
Organic acid production	All	8
Ph 5	All	8
Recapitulate	All	8
Uc	All	8
Breve	All	7
Fermentation supernatant	All	7
Inflammatory property	All	7
Key role	All	7
Lipopolysaccharide	All	7

Micronutrient	All	7
Mucosal microbiota	All	7
Probiotic cell	All	7
Signature	All	7
Simulate gastrointestinal environment	All	7
Vitro assessment	All	7
Zn	All	7
Anti inflammatory property	All	6
Arsenic speciation	All	6
Bacillus cereus	All	6
Cereus	All	6
Chelate	All	6
Filter	All	6
Inflammatory response	All	6
Lgg	All	6
Normal weight	All	6
Ssp	All	6
Structural feature	All	6
Threat	All	6
Tract digestion	All	6
Vitro colon fermentation	All	6
Aa	All	5
Biotechnology	All	5
Colon lactobacilli	All	5
Community analysis	All	5
Contributor	All	5
Cook rice	All	5
Culture supernatant	All	5
Destruction	All	5
Differe	All	5
Disturbance	All	5
Ferment food	All	5
Gallic acid equivalent	All	5
Gut microbial metabolism	All	5
Host cell	All	5
Individual phenolic	All	5
Intestinal cell line	All	5
Kiwifruit	All	5
Lactate production	All	5
Media b	All	5
Powerful tool	All	5
Probiotic mixture	All	5
Proinflammatory cytokine	All	5
Tea extract	All	5
Tendency	All	5
Trap	All	5
Zinc	All	5
**Year 2011**		
Antioxidant	All	112
Microbiome	All	110
Chemistry	All	61
Royal society	All	59
Gut microbiome	All	58
Antioxidant capacity	All	49
Inflammation	All	38
Proteobacteria	All	34
Dysbiosis	All	33
Metabolomic	All	22
Cell viability	All	20
Cell culture	All	18
Medicine	All	17
Morphology	All	16
Gut microbe	All	15
Microstructure	All	15
Ms ms	All	15

Ex vivo	All	14
Vitro dynamic	All	14
Wine	All	14
Biology	All	13
Protein content	All	13
Pyrosequence	All	13
Simulate cdi	All	13
Wageningen academic publisher	All	13
Oxidation	All	12
Metabolome	All	11
Refrigerate storage	All	11
Roast	All	11
Starter	All	11
Apoptosis	All	10
Colon cancer cell	All	10
Fecal material	All	10
Ht29 mtx	All	10
Lactis bb 12	All	10
Yogurt	All	10
Bacterial viability	All	9
Gut environment	All	9
Gut fermentation	All	9
Microencapsulate	All	9
Participant	All	9
Warrante	All	9
Arsenite	All	8
Bifidobacterium animalis subsp	All	8
Digestion system	All	8
Enterococcus faecium	All	8
Exchange	All	8
Hypothese	All	8
Opportunity	All	8
Ow	All	8
Preservation	All	8
Simulate gastrointestinal juice	All	8
Bacillus coagulan gbi 30	All	7
Cncm	All	7
Drug release	All	7
Flavanol	All	7
Mediator	All	7
Probiotic bifidobacterium	All	7
Salivary	All	7
Total phenol	All	7
Transepithelial electrical resistance	All	7
Xenobiotic	All	7
Amylose	All	6
Bifidobacterium specy	All	6
Brain	All	6
Compromise	All	6
Digestive fluid	All	6
Flight	All	6
Food digestion	All	6
Food supplement	All	6
Glycation	All	6
Gut microorganism	All	6
Hepg2	All	6
Hydroxyphenylpropionic	All	6
Lipid metabolism	All	6
Log cycle	All	6
Model drug	All	6
Probiotic supplementation	All	6
Pseudomonas	All	6
Release rate	All	6
Rich source	All	6
Scaffold	All	6

Soil sample	All	6
Total antioxidant capacity	All	6
Vitro condition	All	6
Vitro gastric digestion	All	6
Atmosphere	All	5
Bacterial infection	All	5
Boulardii	All	5
Cell cycle	All	5
Dgm	All	5
Energy metabolism	All	5
Enteric pathogen	All	5
Fat content	All	5
Flight mass spectrometry	All	5
Fragmentation	All	5
Indigestible fraction	All	5
Microbe microbe	All	5
Microbial profile	All	5
Purity	All	5
Salivarius	All	5
Salt concentration	All	5
Starter culture	All	5
Storage time	All	5
Strain probiotic	All	5
Tim 1 system	All	5
Total concentration	All	5
Virulence determinant	All	5
Wheat bread	All	5
**Year 2010**		
Firmicute	All	57
Bacteroidete	All	54
Gut microbiota composition	All	40
Vitro simulate	All	39
Beverage	All	38
Akkermansia	All	30
Disorder	All	28
Obesity	All	27
Dpph	All	23
Fermentation process	All	22
Human fecal microbiota	All	22
Phylum	All	20
Scavenge	All	20
Food industry	All	19
Cd	All	18
Gut microbial community	All	18
John	All	18
Phytochemical	All	18
Son	All	18
Metabolic profile	All	17
Probiotic candidate	All	17
Probiotic lactobacillus	All	17
Chip	All	15
Coagulan	All	15
Phyla	All	15
Radical scavenge	All	15
Bacillus coagulan	All	14
Clostridium cluster	All	14
Fermentation profile	All	14
Accessibility	All	13
Microencapsulation	All	13
Xiva	All	13
Colonic condition	All	12
Ethanol	All	12
Healthy adult	All	12
Vitro gastric	All	12
Beta lactoglobulin	All	11

Dna damage	All	11
Duodenal digestion	All	11
Gastrointestinal juice	All	11
Harbor	All	11
Mr	All	11
Valeric	All	11
Vitro fermentation model	All	11
Chitosan	All	10
Butyric acid production	All	9
Digestion product	All	9
Distal region	All	9
Germinate	All	9
Valeric acid	All	9
Xos	All	9
Ag	All	8
Bacteriocin	All	8
Beef	All	8
Colonic model system	All	8
Cow	All	8
Crohn	All	8
Dynamic gastric model	All	8
Foodborne pathogen	All	8
Gastrointestinal tract condition	All	8
Human gastric	All	8
Modulatory	All	8
Pesticide	All	8
Antibiotic resistant	All	7
Body weight	All	7
Boil	All	7
Chicken	All	7
Dietary supplement	All	7
Dynamic gut model	All	7
Gastrointestinal digest	All	7
Git condition	All	7
Pediococcus	All	7
Recovery rate	All	7
Replacement	All	7
Simulate gastrointestinal tract condition	All	7
Simulate human digestion	All	7
Succinate	All	7
Xylo oligosaccharide	All	7
Cow milk	All	6
Cross link	All	6
Intestine condition	All	6
Jejunal	All	6
Silico	All	6
Trypsin	All	6
Vivo condition	All	6
Bacterial enumeration	All	5
Biotic	All	5
Central role	All	5
Dpph radical	All	5
Epitope	All	5
Fibre content	All	5
Ganedenbc30	All	5
Human gastrointestinal condition	All	5
Intestinal mucosa	All	5
Mary ann liebert	All	5
N butyric acid	All	5
Phylogenetic microarray	All	5
Potential probiotic candidate	All	5
Protease	All	5
Resolve	All	5
Strain exhibit	All	5
Young children	All	5

Year 2009

Potential prebiotic	All	33
Anti inflammatory	All	31
Food product	All	27
Children	All	22
Gc	All	22
Plasma	All	18
Prebiotic activity	All	18
Tim 1	All	18
Cheese	All	16
Intestinal fluid	All	16
Antibiotic treatment	All	15
Child	All	15
Colon vessel	All	15
Colonic microbial	All	15
Digestive enzyme	All	15
Hydroxyphenyl	All	15
Colonic metabolism	All	14
Occurrence	All	14
Adherence	All	13
Df	All	13
Hydrophobicity	All	13
Animalis subsp	All	11
Ibd	All	11
Phenylpropionic	All	11
Simulate colonic fermentation	All	11
Microbial group	All	10
Spik	All	10
Chemopreventive	All	9
Cholesterol	All	9
Efflux	All	9
Human health risk	All	9
Intestinal environment	All	9
Skim milk	All	9
Spray	All	9
Spray dry	All	9
Colonic metabolite	All	8
Consistency	All	8
Licensee biom central	All	8
Phenylpropionic acid	All	8
Simulate intestinal fluid	All	8
Vitro bioaccessibility	All	8
American dairy science association	All	7
Amoxicillin	All	7
Bioprocess	All	7
Child gut	All	7
Clostridium coccoide eubacterium rectale	All	7
Coumaric	All	7
Culture media	All	7
Healthier	All	7
Hg	All	7
Physico	All	7
Predigest	All	7
Serovar	All	7
Soluble fiber	All	7
Bifidobacterium lactis	All	6
Chemopreventive potential	All	6
Dialysate	All	6
Disappear	All	6
Emergence	All	6
Fa	All	6
Fecal coliform	All	6
Human health risk assessment	All	6
Human proximal	All	6
Lactobacillus specy	All	6

Prebiotic inulin	All	6
Probiotic culture	All	6
Protein fermentation	All	6
Shime system	All	6
Stage chemostat gut model	All	6
Tailor	All	6
Ammonia concentration	All	5
Antibiotic susceptibility	All	5
Bioreactor model	All	5
Cardiovascular disease	All	5
Coumaric acid	All	5
Dna sequence	All	5
Human git	All	5
Intestinal microbe	All	5
Isomalto	All	5
Isovalerate	All	5
Lactate level	All	5
Ligand	All	5
Milieu	All	5
Moxifloxacin	All	5
New prebiotic	All	5
Pcr dgge analysis	All	5
Physico chemical	All	5
Promise candidate	All	5
Proximal large intestine	All	5
Roseburia spp	All	5
Salmonella infection	All	5
Semi dynamic	All	5
Simulate colon model	All	5
Te	All	5
Time dependent	All	5
Total scfa production	All	5
V v	All	5
**Year 2008**		
Human gut microbiota	All	90
Gastrointestinal condition	All	71
Simulate gastrointestinal condition	All	57
Fruit	All	48
Intestinal digestion	All	41
Vitro gut model	All	39
Prevotella	All	35
Fate	All	33
Simulate gastric	All	33
Encapsulation	All	32
Git	All	27
Polyphenolic	All	24
Grain	All	22
Probiotic property	All	21
Catechin	All	19
Cancer cell	All	17
Rhamnosus gg	All	15
Apple	All	14
Invasion	All	14
Vitro human gut model	All	14
Dosage	All	13
Probiotic potential	All	13
Recurrence	All	13
Instil	All	11
Lactobacillus rhamnosus gg	All	11
Acting	All	10
Corn	All	10
Disintegration	All	10
Dynamic gastrointestinal model	All	10
Acidification	All	9
Bacteroide prevotella	All	9

Discriminate	All	9
Evolution	All	9
Gastrointestinal survival	All	9
Glycerol	All	9
Texture	All	9
Chocolate	All	8
Collection	All	8
Ir	All	8
Lb	All	8
Proanthocyanidin	All	8
Simulate gastric juice	All	8
Vitro evaluation	All	8
Cocoa	All	7
Contamination	All	7
Epicatechin	All	7
Human digestive tract	All	7
Immunomodulatory	All	7
Prevalence	All	7
Reduce c	All	7
Triplicate	All	7
Commercial probiotic	All	6
Difficile pcr ribotype 027	All	6
Feasibility	All	6
Human intervention	All	6
Ng	All	6
Shirota	All	6
Vancomycin instillation	All	6
Allergen	All	5
Bacterial adhesion	All	5
Bioreactor system	All	5
Ifn	All	5
Ifn gamma	All	5
Ige	All	5
Macromolecule	All	5
Mycotoxin	All	5
Population increase	All	5
Probiotic food	All	5
Recrudescence	All	5
Reuterin	All	5
Scfa profile	All	5
Thermal process	All	5
**Year 2007**		
Polyphenol	All	102
Tim	All	53
Candidate	All	47
Industry	All	45
Alginate	All	31
Tim 2	All	31
Dynamic gastrointestinal	All	28
Consumer	All	26
Llc	All	25
Permeability	All	23
Strain isolate	All	21
Fructan	All	20
Phylogenetic	All	20
Ferulic	All	18
Technologist	All	18
Colon region	All	17
Nmr	All	17
Arabinoxylan	All	16
Ferulic acid	All	16
Optimization	All	16
Vegetative	All	16
Metal	All	15
Soybean	All	15

Bcfa	All	14
Food technologist	All	14
Symptom	All	14
Total polyphenol	All	14
Microcapsule	All	13
Propionate production	All	13
Titre	All	13
Calcium	All	12
Complex microbial community	All	12
Chemical industry	All	11
Cytotoxin titre	All	11
Inhibitor	All	11
Rna	All	11
Blend	All	10
Dietary polyphenol	All	10
Perturbation	All	10
Tannin	All	10
Trait	All	10
Fermentum	All	9
Genome	All	9
Lactobacillus fermentum	All	9
Total viable count	All	9
Vegetative cell	All	9
Ax	All	8
Difficile ribotype	All	8
Difficile total viable	All	8
Gasseri	All	8
Healthcare	All	8
Intestinal ecosystem	All	8
Lysine	All	8
Spore count	All	8
Beneficial property	All	7
Caco 2 cell monolayer	All	7
Condense tannin	All	7
Difficile pcr ribotype	All	7
Difficile total viable count	All	7
Gene encode	All	7
Increase production	All	7
Lactobacillus gasseri	All	7
Oral delivery	All	7
Parent	All	7
Prebiotic candidate	All	7
Prim	All	7
Arsenic bioaccessibility	All	6
Biogenic amine	All	6
Gastrointestinal tract model	All	6
Human gastrointestinal model	All	6
Proteolytic activity	All	6
Stress condition	All	6
Bioaccessible fraction	All	5
Candidate prebiotic	All	5
Cc	All	5
Dietary intake	All	5
Difficile ribotype 027	All	5
Fermentation activity	All	5
Formate	All	5
Hormone	All	5
Human intestinal ecosystem	All	5
Intestinal bioaccessibility	All	5
Methanol	All	5
Polymeric	All	5
Shime colon	All	5
Transferase	All	5
**Year 2006**		
Highlight	All	52

Lipid	All	52
Tolerance	All	41
Qpcr	All	40
Spore	All	40
Cook	All	35
Simulate gastrointestinal tract	All	32
Molecule	All	29
Gmbh	All	28
Human gut model	All	28
Standardize	All	26
Chemotherapy	All	23
Germination	All	23
Shime model	All	23
Soy	All	23
Homeostasis	All	22
Kgaa	All	22
Lab	All	22
Vch verlag gmbh	All	22
Viscosity	All	22
Batch fermentation	All	21
Clostridium difficile infection	All	21
Colonic model	All	21
Weinheim	All	21
Antimicrobial chemotherapy	All	20
British society	All	20
Sd	All	20
Arsenic	All	19
Phenotype	All	19
Ribotype	All	19
Immune response	All	18
Biological activity	All	16
Intestinal model	All	16
Ribotype 027	All	16
Cell monolayer	All	15
Difficile spore	All	15
Gastrointestinal environment	All	15
Transplantation	All	15
Ca	All	14
Consortium	All	14
Infant gut	All	14
Inflammatory cytokine	All	14
Pcr ribotype	All	14
Speciation	All	14
Metabolite production	All	13
Mic	All	13
Microbial community structure	All	13
Closer	All	12
Coefficient	All	12
Fem	All	12
Gastric condition	All	12
Oxide	All	12
Adsorption	All	11
Blackwell	All	11
Pcr ribotype 027	All	11
Selectivity	All	11
Spore germination	All	11
Butyrate producer	All	10
Colon simulator	All	10
Difficile strain	All	10
Extrusion	All	10
Lactobacillus paracasei	All	10
Arsenate	All	9
Atopobium	All	9
Deficiency	All	9
Digest sample	All	9

Lactobacillus enterococcus	All	9
Root	All	9
Women	All	9
Cytotoxin production	All	8
Genomic	All	8
Infant gut microbiota	All	8
Starche	All	8
Infant microbiota	All	7
Kidney	All	7
Pb	All	7
Sorption	All	7
Vitro colonic model	All	7
Artichoke	All	6
Behave	All	6
Confound	All	6
Difficile spore germination	All	6
Exopolysaccharide	All	6
Human population	All	6
Lactic acid production	All	6
Pilot	All	6
Semi continuous	All	6
Simulate gastrointestinal system	All	6
Apoptotic	All	5
Batch model	All	5
Caco 2 cell line	All	5
Colon microbial community	All	5
Cytokine production	All	5
Digestible starch	All	5
Digestion profile	All	5
Excretion	All	5
Expression profile	All	5
Extrude	All	5
Hop	All	5
Interindividual difference	All	5
Interindividual variability	All	5
Microbial consortium	All	5
Rd	All	5
Ribotype 001	All	5
Toll	All	5
X ray absorption	All	5
**Year 2005**		
Microbiota composition	All	90
Simulate gastrointestinal digestion	All	72
Ingredient	All	62
Gi	All	53
Bioactivity	All	40
Characterization	All	39
Colon model	All	37
Vitro colon	All	37
Protection	All	36
Anthocyanin	All	34
Intestinal phase	All	34
Gas chromatography	All	31
Pepsin	All	30
Monolayer	All	27
Phenolic acid	All	26
Epithelial barrier	All	24
Coat	All	23
Gastrointestinal fluid	All	23
Incorporation	All	23
Performance liquid chromatography	All	23
Human intestinal microbiota	All	22
Peel	All	22
Animalis	All	21
Food ingredient	All	21

Vitro colon model	All	21
Bb	All	20
Galactooligosaccharide	All	20
Gi tract	All	20
Spectroscopy	All	19
Bifidobacterium animalis	All	18
Disruption	All	18
Quercetin	All	18
Live	All	17
Simulate gastrointestinal fluid	All	17
Bifidum	All	15
Capsule	All	15
Delivery system	All	15
Human faecal microbiota	All	15
Prebiotic property	All	15
Receptor	All	15
Dynamic model	All	14
Gastrointestinal simulator	All	14
Ht29	All	14
Intestinal barrier	All	14
Isolation	All	14
Biosynthesis	All	13
Dissolution	All	13
Junction	All	13
Metabolic pathway	All	13
Physiological condition	All	13
Tetracycline	All	13
Bb 12	All	12
Human intestine	All	12
Oral administration	All	12
Solubility	All	12
Staphylococcus aureus	All	12
Baseline	All	11
Butyrate produce bacteria	All	11
Butyrogenic	All	11
Klebsiella	All	11
Promotion	All	11
Utilise	All	11
Vivo model	All	11
Bifidobacterium bifidum	All	10
Broad spectrum	All	10
Chemostat gut model	All	10
Fermentation property	All	10
Hydrolyse	All	10
Intestinal epithelial barrier	All	10
Potency	All	10
Potential benefit	All	10
Proteolysis	All	10
Protocatechuic acid	All	10
Digestive condition	All	9
Nuclear magnetic resonance	All	9
Orange juice	All	9
Total bacteria	All	9
Clostridium histolyticum	All	8
Dairy product	All	8
Engineering	All	8
Enterica	All	8
Hydroxyphenylacetic	All	8
Ph 3	All	8
Salmonella enterica	All	8
Caco 2 monolayer	All	7
Conjugation	All	7
Cyanidin	All	7
Drug delivery	All	7
Foodstuff	All	7

Gas chromatography mass spectrometry	All	7
Gelatin	All	7
Glass	All	7
Human colon microbiota	All	7
Lignan	All	7
Metabolite concentration	All	7
Ph 1	All	7
Resilience	All	7
Simulate digestive	All	7
Vitro digestion fermentation	All	7
Approx	All	6
Bioassay	All	6
Cm	All	6
Colon digest	All	6
Control condition	All	6
Erythromycin	All	6
Examination	All	6
Gut model system	All	6
Hydroxyphenylacetic acid	All	6
Increase butyrate production	All	6
Kefir	All	6
Leuconostoc mesenteroide	All	6
Mesenteroide	All	6
Rye	All	6
Adenocarcinoma cell	All	5
Cell survival	All	5
Chain fatty acid concentration	All	5
Colon target	All	5
Enzymatic digestion	All	5
Epithelia	All	5
Gastric acid	All	5
Gastric acidity	All	5
Hindgut	All	5
Individual variation	All	5
Insoluble fraction	All	5
Peptone	All	5
Ph level	All	5
Polyethylene glycol	All	5
Prebiotic index	All	5
Simulate stomach	All	5
Skin	All	5
**Year 2004**		
Bioactive	All	88
Fecal microbiota	All	75
Rrna gene	All	64
Ms	All	60
Mucosal	All	51
Mass spectrometry	All	49
Fermentation model	All	43
Liquid chromatography	All	42
Log cfu	All	42
Vitro colonic	All	40
American society	All	37
Extraction	All	36
Prebiotic potential	All	36
Profil	All	33
Duodenal	All	31
Gene expression	All	30
Pool	All	30
Lc	All	29
Real time	All	28
Integrity	All	27
Vitro colonic fermentation	All	27
Colon compartment	All	26
Rrna gene sequence	All	26

Survival rate	All	26
Gum	All	24
Quantitative pcr	All	23
Simulate colon	All	23
Bead	All	22
Contaminate	All	22
Pulp	All	22
Bowel disease	All	21
Inflammatory bowel disease	All	20
Freeze dry	All	19
Rice	All	19
Microbial community composition	All	18
Mucosa	All	18
Ph 2	All	17
Slurry	All	17
Intestinal condition	All	16
Contaminate soil	All	15
Disrupt	All	15
Chromatography mass spectrometry	All	14
Emerge	All	14
Lc ms	All	14
Pcr dgge	All	14
Colonic fermentation model	All	13
Dc	All	13
Fecal inocula	All	13
Healthy individual	All	13
Microbial fermentation	All	13
Persist	All	13
Potato	All	13
Tc	All	13
Cell proliferation	All	12
Cr	All	12
Intestinal fermentation	All	12
Model simulate	All	12
Pc	All	12
H7	All	11
Infect	All	11
Interindividual	All	11
Replicate	All	11
Coculture	All	10
Coli o157	All	10
Commensal bacteria	All	10
Escherichia coli o157	All	10
Intestinal flora	All	10
Bacillus subtilis	All	9
Bacterial diversity	All	9
Dissolve	All	9
H incubation	All	9
Inactivation	All	9
Intestinal absorption	All	9
New insight	All	9
Real time pcr	All	9
Transcription	All	9
Acidity	All	8
Aglycone	All	8
Bifidobacterium infantis	All	8
Butyrate concentration	All	8
Colonic environment	All	8
Equation	All	8
Faecal slurry	All	8
Fecal slurry	All	8
Gastric ph	All	8
Highest concentration	All	8
Hospital	All	8
Human intestinal tract	All	8

Pancreatic juice	All	8
Polydextrose	All	8
Solid phase	All	8
Titer	All	8
Transcriptional	All	8
Virulence gene	All	8
Bifidobacterial specy	All	7
Complex microbiota	All	7
Fecal microflora	All	7
Flux	All	7
Gastric fluid	All	7
Hydrocarbon	All	7
Upregulate	All	7
Human fecal inoculum	All	6
Inulin addition	All	6
Liquid chromatography mass spectrometry	All	6
Microbial production	All	6
Non digest	All	6
Piglet	All	6
Soluble dietary fiber	All	6
Specy composition	All	6
Vitro colonic fermentation model	All	6
Vitro gastrointestinal tract	All	6
Bacterial concentration	All	5
Corroborate	All	5
Ileal effluent	All	5
Imo	All	5
Legume	All	5
Log unit	All	5
Membrane bioreactor	All	5
Metabolic product	All	5
Nacl	All	5
Pah	All	5
Ph condition	All	5
Polycyclic aromatic hydrocarbon	All	5
Porosity	All	5
Prototype	All	5
Pulse	All	5
Vitro culture	All	5
Vitro prebiotic	All	5
Window	All	5
**Year 2003**		
Gastrointestinal digestion	All	200
Bioaccessibility	All	133
Vitro gastrointestinal digestion	All	119
Pathogen	All	87
Barrier	All	71
Colonic microbiota	All	68
Digestion model	All	66
Vitro digestion model	All	46
Hplc	All	45
Ingestion	All	45
Commensal	All	43
Faecalibacterium	All	43
Bioaccessible	All	42
Clostridium difficile	All	39
Difficile infection	All	39
Bifidobacterium spp	All	33
Human colonic microbiota	All	32
Community structure	All	31
Cdi	All	29
Seed	All	29
Antimicrobial activity	All	28
Roseburia	All	26
Human digestion	All	25

Prausnitzii	All	25
Soil	All	25
Matrice	All	24
Oxford university	All	24
Beneficial bacteria	All	23
Faecalibacterium prausnitzii	All	23
Ingest	All	23
Inhibitory activity	All	23
Toxin	All	23
Vancomycin	All	23
Cytotoxin	All	21
Breast	All	19
Pathogenesis	All	18
Rectale	All	18
Eubacterium rectale	All	17
Ruminococcus	All	17
Saccharomyce	All	17
Diarrhea	All	16
Cerevisiae	All	15
Coccoide	All	15
Glycoside	All	15
Instillation	All	15
Saccharomyce cerevisiae	All	15
Toxin production	All	15
Opportunistic	All	14
Clostridium spp	All	13
Colitis	All	13
Polymerization	All	13
Rrna sequence	All	12
Opportunistic pathogen	All	11
Saliva	All	11
Breast milk	All	10
Scan electron microscopy	All	10
Ace	All	9
Colonization resistance	All	9
Energy source	All	9
Monoculture	All	9
Standardise	All	9
Digestive juice	All	8
Exclusion	All	8
Intestinalis	All	8
Milk protein	All	8
Pea	All	8
Propensity	All	8
Roseburia intestinalis	All	8
Angiotensin	All	7
Antibiotic exposure	All	7
Centrifugation	All	7
Counterpart	All	7
Cytotoxic	All	7
Depletion	All	7
Enterococcus spp	All	7
Fecal culture	All	7
Food matrice	All	7
Toxic metabolite	All	7
Bioaccessibility value	All	6
Biological property	All	6
Confocal microscopy	All	6
Film	All	6
Plaque	All	6
Vitro human digestion	All	6
Ace inhibitory activity	All	5
Complex carbohydrate	All	5
Difficile population	All	5
Difficile proliferation	All	5

Ic50	All	5
Nosocomial	All	5
Overcom	All	5
Pea protein	All	5
Phosphate buffer saline	All	5
Rationale	All	5
**Year 2002**		
Gastric digestion	All	44
Food matrix	All	39
Microbial metabolism	All	28
Fish	All	26
Fluorescence	All	26
Organic acid	All	24
Situ hybridization	All	24
Amplicon	All	20
Cultivation	All	20
Bread	All	19
Biomass	All	18
Carbon source	All	17
Cytometry	All	17
Flow cytometry	All	17
Diffusion	All	16
Status	All	16
Gastric empty	All	14
Label	All	14
Digestive system	All	9
Enzymatic hydrolysis	All	9
Dynamic digestion	All	8
Granule	All	7
Washout	All	7
Antimicrobial agent	All	6
Regime	All	6
Total carbohydrate	All	6
Ammonium concentration	All	5
Oral phase	All	5
Solid food	All	5
TRUE	All	5
**Year 2001**		
Simulate gastrointestinal	All	204
Pcr	All	80
Storage	All	58
Survive	All	57
Infant	All	53
Health benefit	All	52
Functional food	All	49
Adhesion	All	47
Microbial composition	All	40
Electrophoresis	All	39
Bacillus	All	36
Gel electrophoresis	All	35
Dna	All	31
Community composition	All	30
Dgge	All	29
Gos	All	29
Viable count	All	25
Denature gradient gel electrophoresis	All	24
Susceptibility	All	24
Kda	All	19
Plate count	All	17
Dominate	All	16
Ferment milk	All	16
Galacto	All	16
Non digestible	All	16
Rdna	All	16
W v	All	16

Chemostat model	All	14
Fingerprint	All	14
Galacto oligosaccharide	All	14
Gastrointestinal system	All	14
Ciprofloxacin	All	13
Gastrointestinal transit	All	13
Methodology	All	13
W w	All	12
Gastric compartment	All	11
Valerate	All	11
Vivo data	All	11
Ci	All	10
Methylation	All	10
Polymerase chain reaction	All	10
Potential beneficial	All	10
Gut flora	All	9
Identity	All	9
Mathematical model	All	9
Segment	All	9
Inactivate	All	8
Megasphaera	All	8
Proliferate	All	8
Rdna sequence	All	8
Residence time	All	8
Band	All	7
Human colonic microflora	All	7
Infant formula	All	7
Log reduction	All	7
Phage	All	7
Phylotype	All	7
Similarity	All	7
Simulate gastrointestinal transit	All	7
Beneficial health	All	6
Bifidobacterial population	All	6
Confirmation	All	6
Dgge analysis	All	6
Enzyme link	All	6
Infant fece	All	6
Overgrowth	All	6
Swine	All	6
Tank reactor	All	6
Ultrafiltration	All	6
Warrant	All	6
Wean	All	6
Anaerobic fermentation	All	5
Antacid	All	5
Clone	All	5
Community dynamic	All	5
Enzyme concentration	All	5
Illness	All	5
Immobilization	All	5
Intestinal compartment	All	5
Methylate	All	5
Microbial specy	All	5
Molecular analysis	All	5
Oryzae	All	5
Primer	All	5
Subunit	All	5
**Year 2000**		
Prebiotic	All	206
Rrna	All	94
Milk	All	93
Scfas	All	67
Salt	All	63
Fece	All	56

Difficile	All	53
Bile salt	All	50
Butyrate production	All	49
Flavonoid	All	40
Casei	All	39
Fos	All	37
Powder	All	36
Mug	All	34
Fructooligosaccharide	All	32
Lactobacillus casei	All	32
Synbiotic	All	27
Ammonium	All	26
Colon microbiota	All	26
Microbiological society	All	26
Damage	All	25
Federation	All	23
Bacterial specy	All	22
European microbiological society	All	22
Toxicity	All	22
Microbial activity	All	21
Colon cancer	All	20
Suspension	All	20
Yeast	All	20
Duodenum	All	19
Staphylococcus	All	19
Human fece	All	18
Conjugate	All	17
Cytotoxicity	All	16
Fecal inoculum	All	16
Fermentative	All	15
Healthy volunteer	All	15
Persistence	All	15
Reuteri	All	15
Competition	All	14
Culture medium	All	14
Intestinal microflora	All	13
Dextran	All	12
Lactobacillus reuteri	All	12
Treatment period	All	12
Cell count	All	11
Maltodextrin	All	11
Microbial ecology	All	11
Isoflavone	All	10
Precipitation	All	10
Probiotic product	All	10
Bacteria isolate	All	9
Counteract	All	9
Fusobacterium	All	9
Glycosidic	All	9
Stabilization period	All	9
Veillonella	All	9
Flavonoid content	All	8
Control diet	All	7
Control period	All	7
Enterobacteria	All	7
Fermentation rate	All	7
Gram positive bacteria	All	7
Increase concentration	All	7
Log10 cfu	All	7
Total scfas	All	7
Viable cell count	All	7
Albican	All	6
Anaerobic batch culture	All	6
Candida albican	All	6
Concentration decrease	All	6

Concentration increase	All	6
Human intestinal microflora	All	6
Jejunum	All	6
Mtt	All	6
Nucleatum	All	6
Rumen	All	6
Bacteroide vulgatus	All	5
Daidzein	All	5
Dimethyl	All	5
Fecal flora	All	5
Fusobacterium nucleatum	All	5
Human fecal flora	All	5
Lactobacillus sp	All	5
Reactor vessel	All	5
Sulfide	All	5
Wk	All	5

* Access to Interllixir program https://carlac.intellixir.fr/cenm provided by The Universidad del Valle

Table S13: Summary of countries associated with each publication.

Country	Number of Publications
CHINA	195
UNITED KINGDOM	131
USA	123
BELGIUM	106
SPAIN	99
BRAZIL	67
ITALY	65
CANADA	63
NETHERLANDS	59
FRANCE	57
GERMANY	35
MEXICO	30
SWITZERLAND	26
FINLAND	22
THAILAND	22
KOREA	21
AUSTRALIA	20
IRELAND	18
JAPAN	17
NEW ZEALAND	17
TURKEY	17
INDIA	15
CHILE	14
DENMARK	14
PORTUGAL	13
POLAND	12
SWEDEN	12
ARGENTINA	9
IRAN	8
ISRAEL	8
SOUTH AFRICA	8
MALAYSIA	7
GREECE	6
ROMANIA	5
HONG KONG	4
AUSTRIA	3
NORWAY	3
SAUDI ARABIA	3
COLOMBIA	2
EGYPT	2
ESTONIA	2
INDONESIA	2
LUXEMBOURG	2
SINGAPORE	2
TUNISIA	2
ARMENIA	1
BULGARIA	1
CAMEROON	1
LEBANON	1
NIGERIA	1
PAKISTAN	1
SERBIA	1
SLOVAKIA	1
SLOVENIA	1

Supplementary Material Section 4

Outcome of a comparison between “refined” and “unrefined” search query datasets.

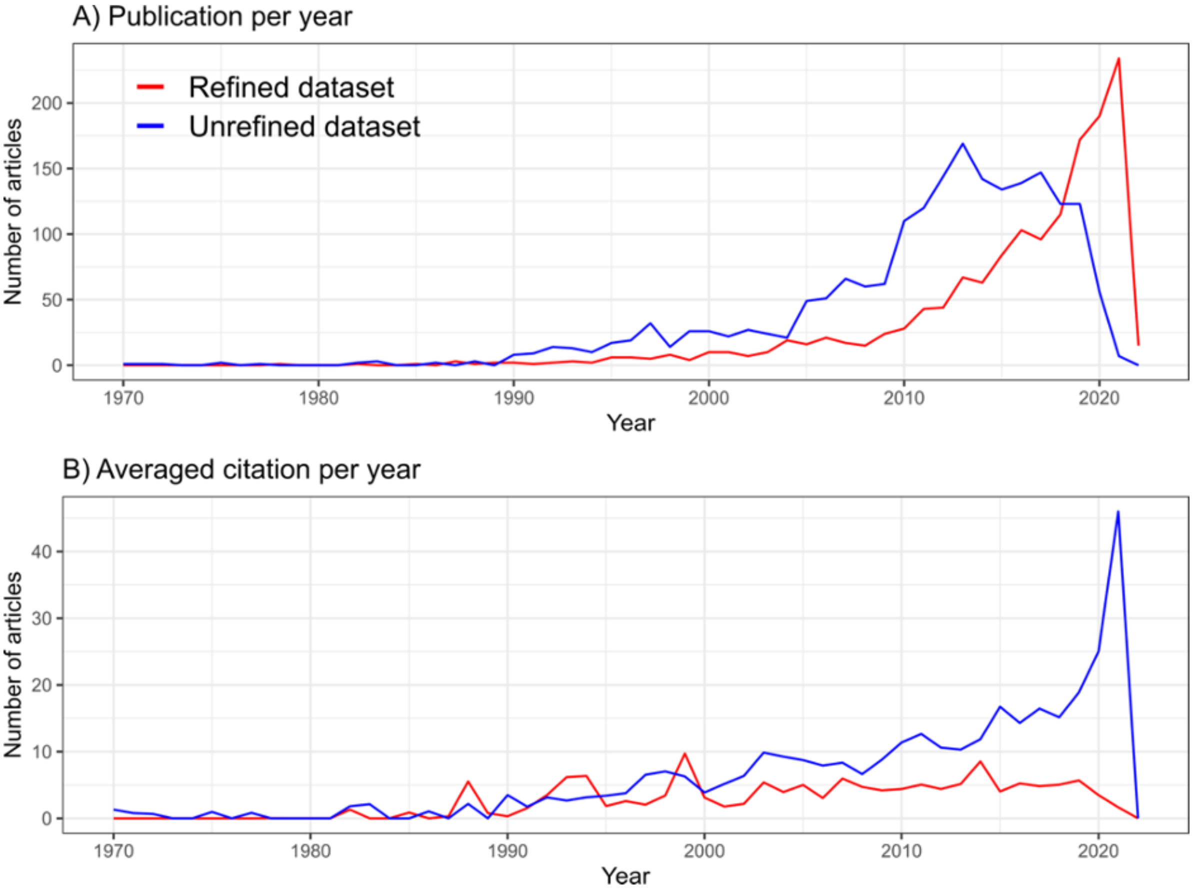

Figure S1: Search outcome comparison of the A) publications and B) citations per year between Refined (red) and Unrefined (blue) search queries.

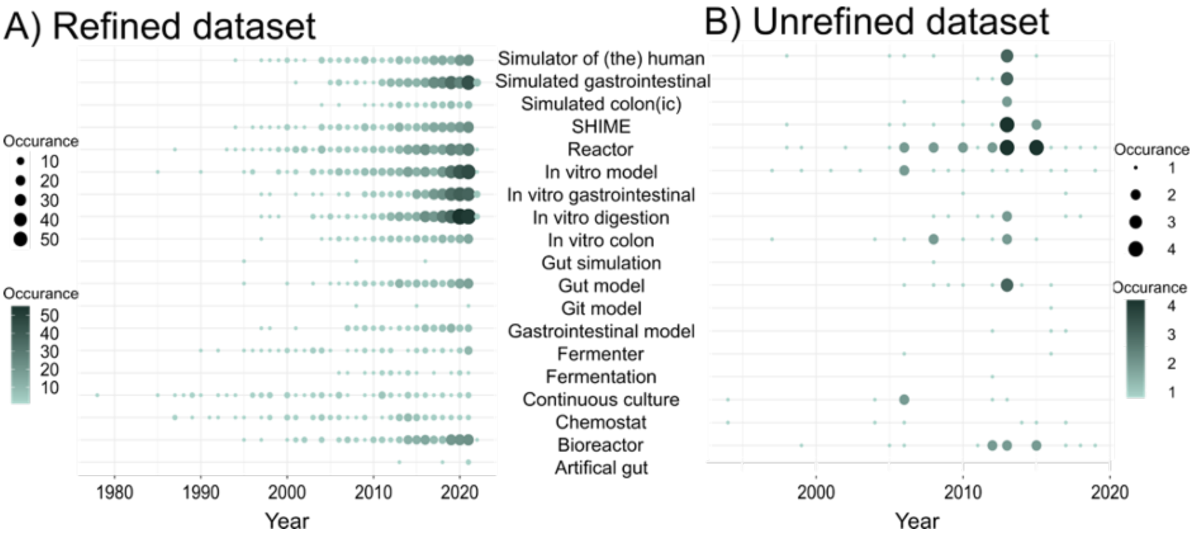

Figure S2: Prevalence of RFST from the (A) Refined dataset containing 1,451 publications (presence of keywords searched from the title, abstract and article keyword fields) compared to (B) Unrefined dataset containing 2,000 publications where no specific keywords were used upon article search.

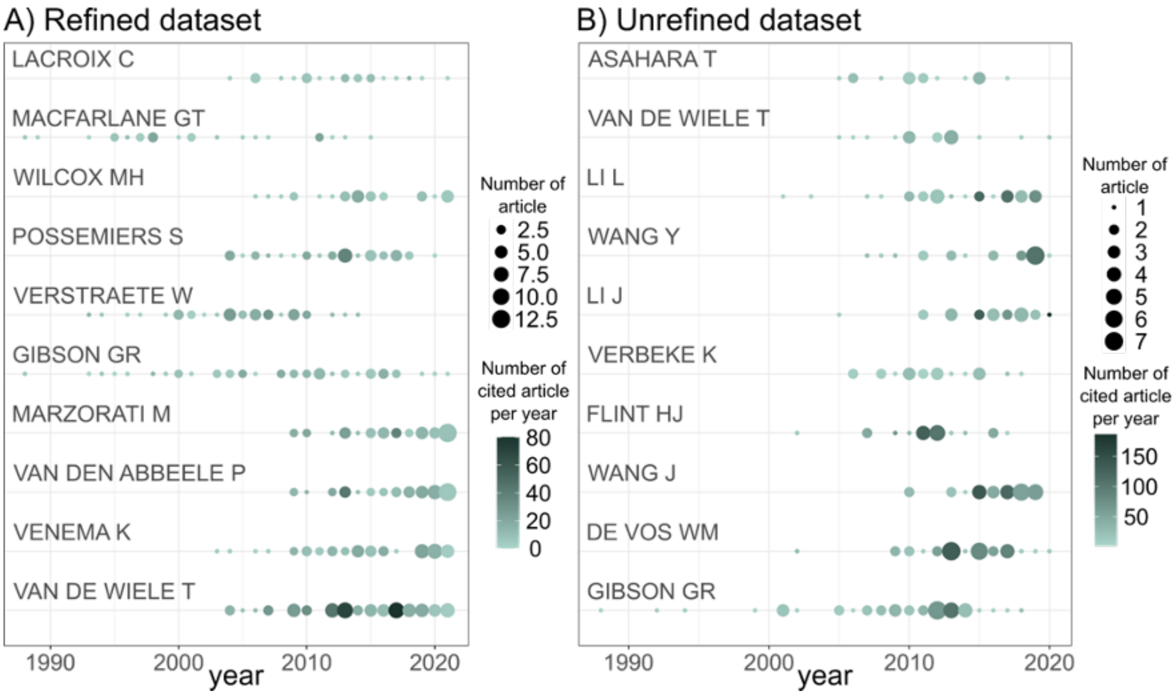

Figure S3: Top 10 authors by publication timeline plot. The number of articles over time is illustrated by the size of the circle and the yearly averaged number of citations is presented by the colour gradient. A) Refined dataset, B) Unrefined dataset.

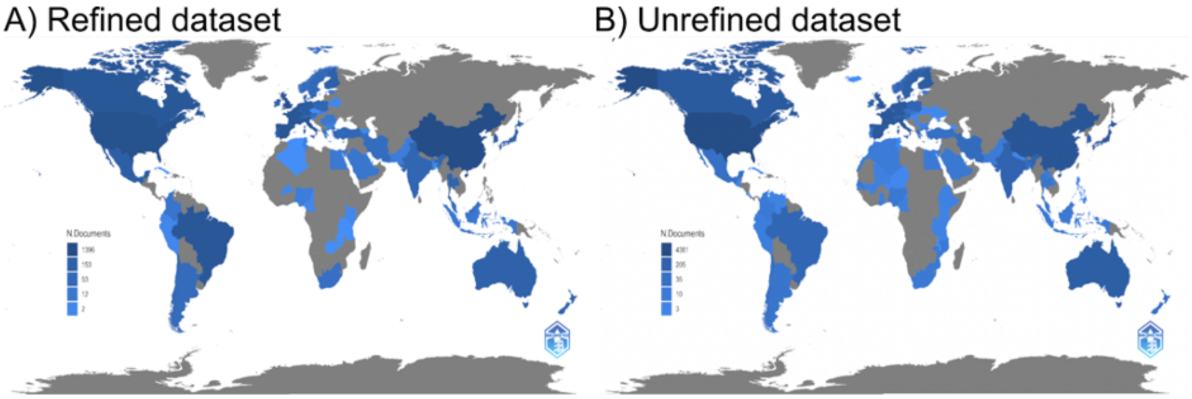

Figure S4: World heatmap illustration of the number of publications per country from two Scopus search query outcomes. A) Refined dataset, B) Unrefined dataset.

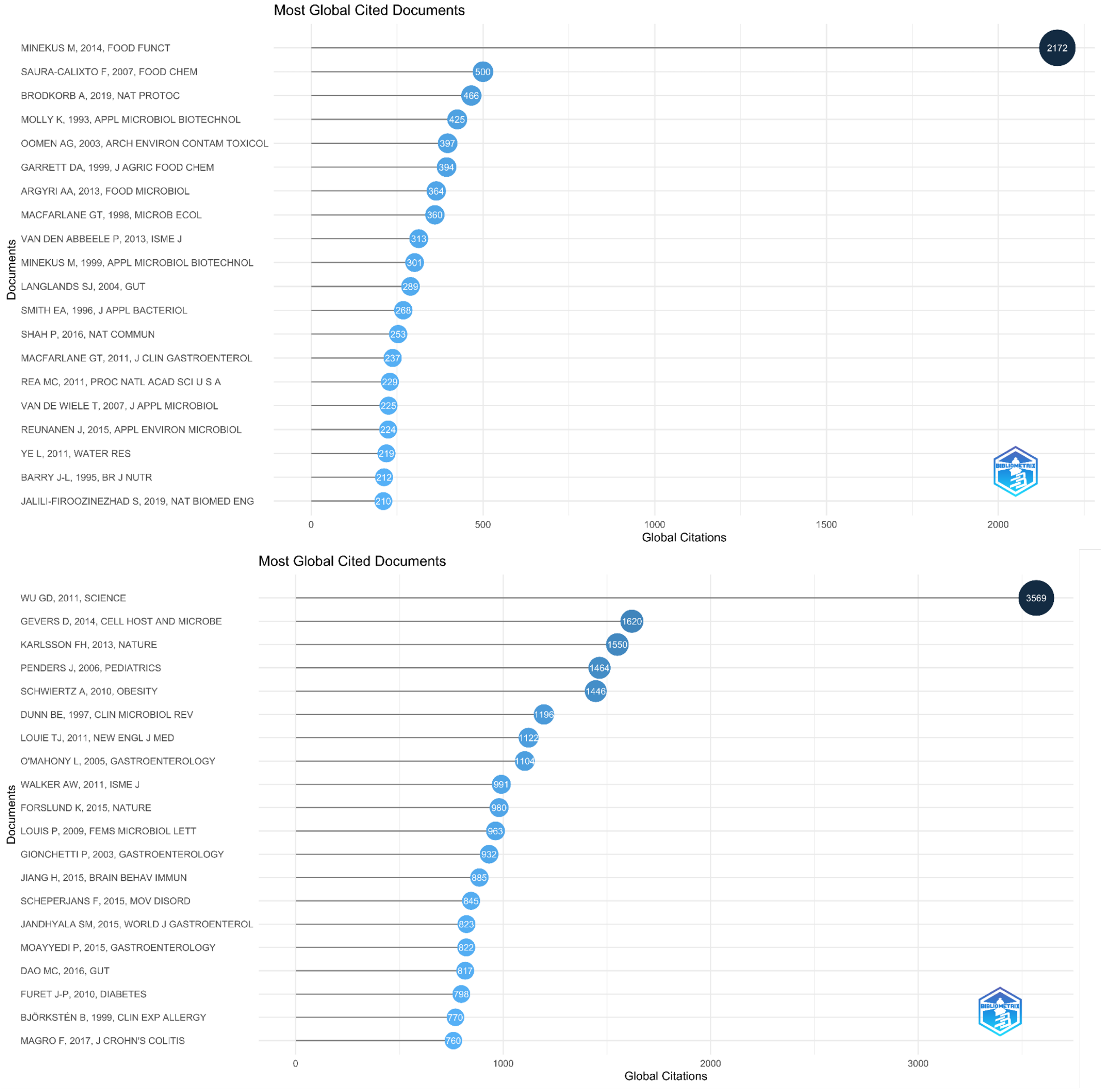

Figure S5: Most cited articles shown as Global Citations (TC) which represents the total citations that an article has received from the documents indexed. A) Refined dataset, B) Unrefined dataset.

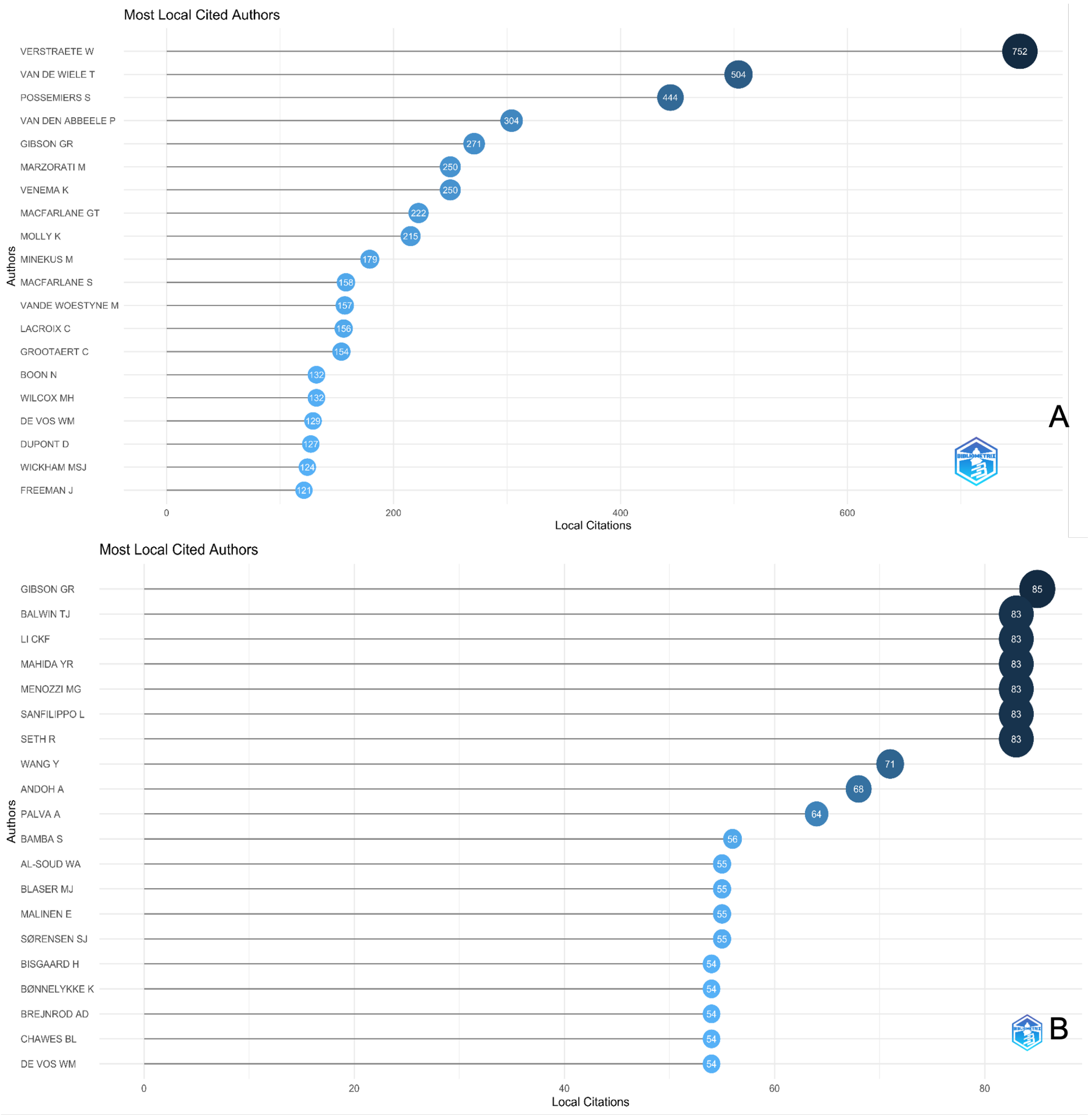

Figure S6: Most cited articles shown as Local Citations (LC) which represents the total citations that an article, included in each collection, received internally. A) Refined dataset, B) Unrefined dataset.

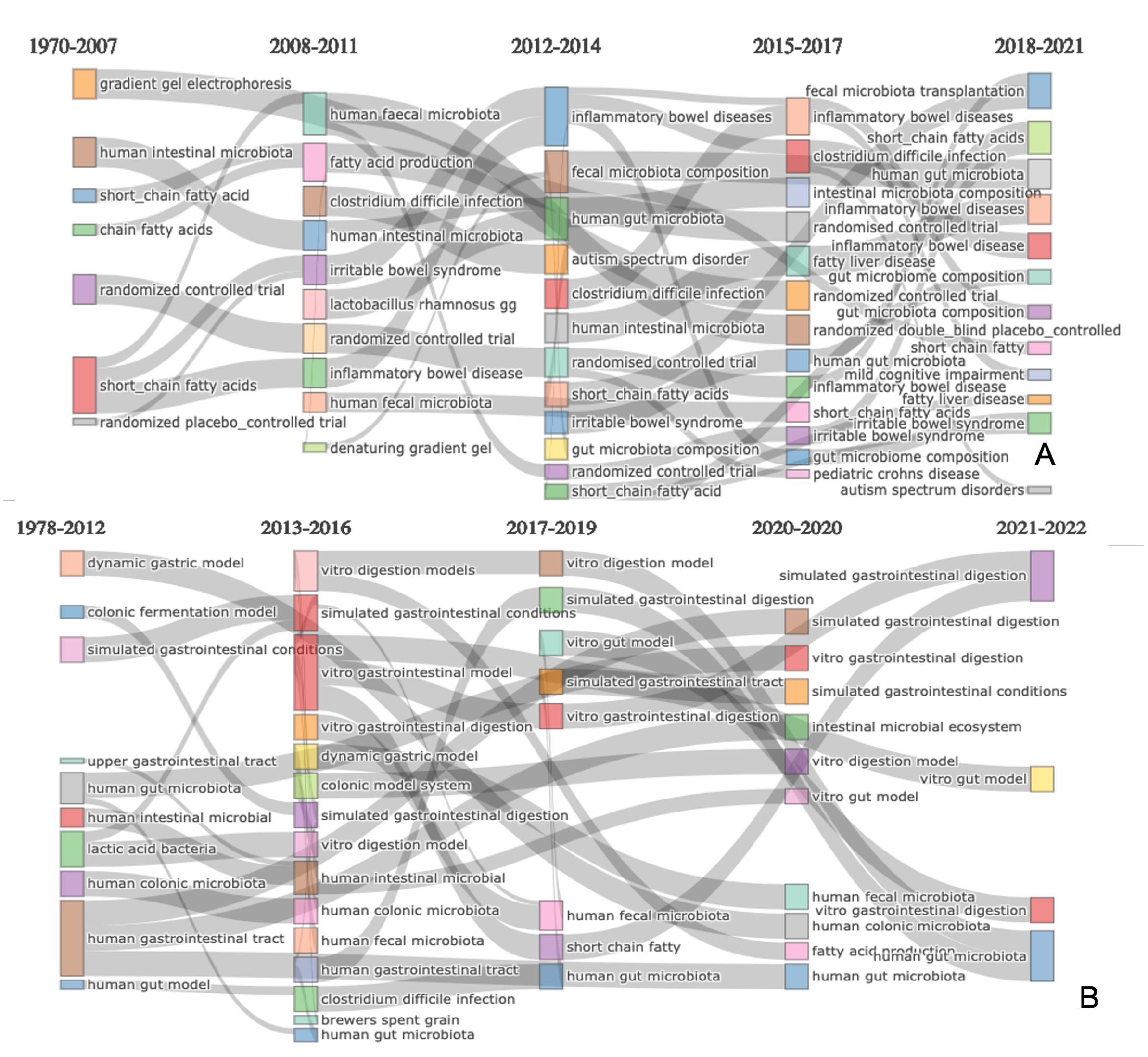

Figure S7: Visualization of the thematic evolution in titles presents the main research areas and their evolution using the Sankey diagram. A) Refined dataset, B) Unrefined dataset.

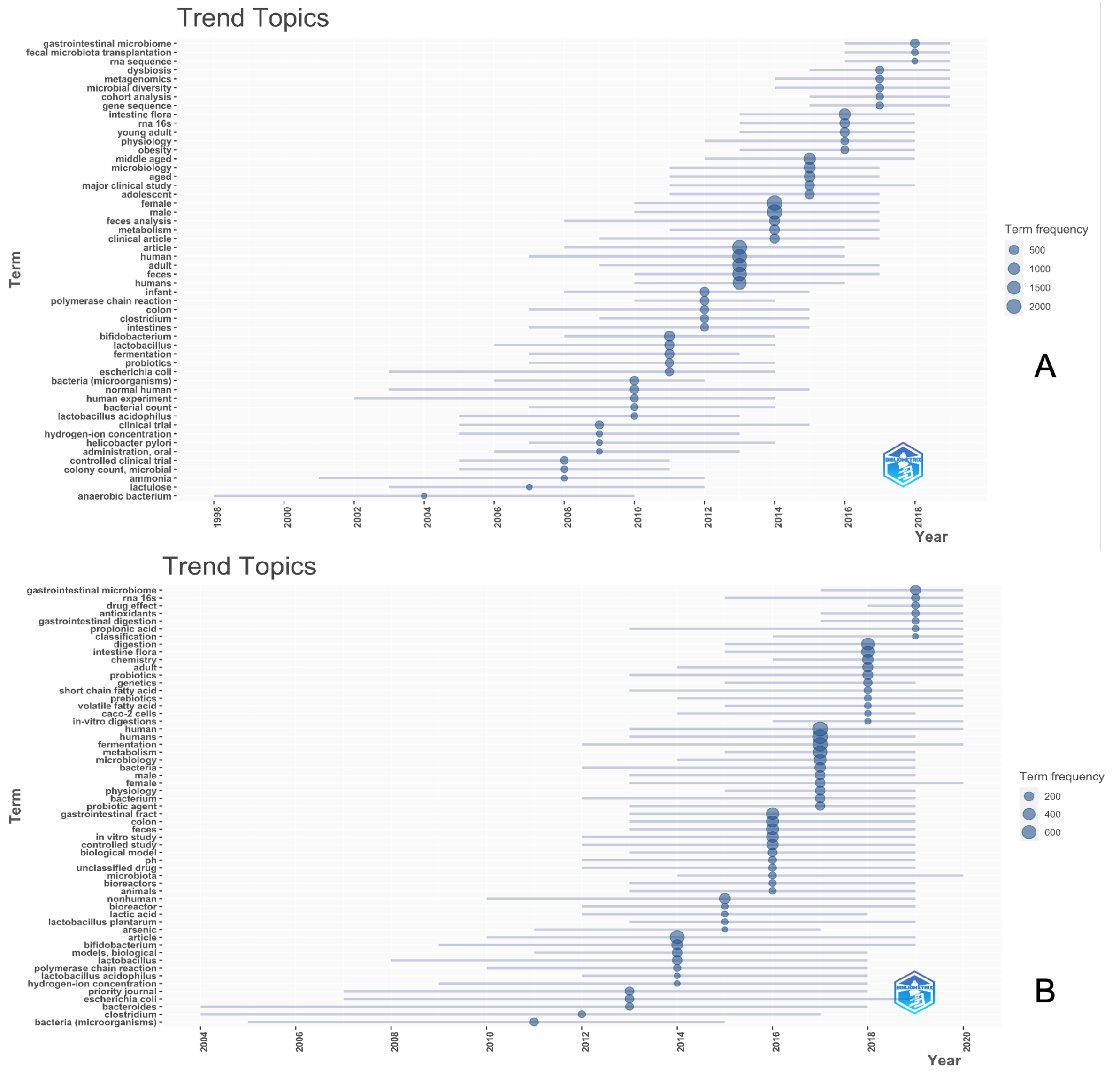

Figure S8: Visualization of the trend topics in key terms. A) Refined dataset, B) Unrefined dataset.

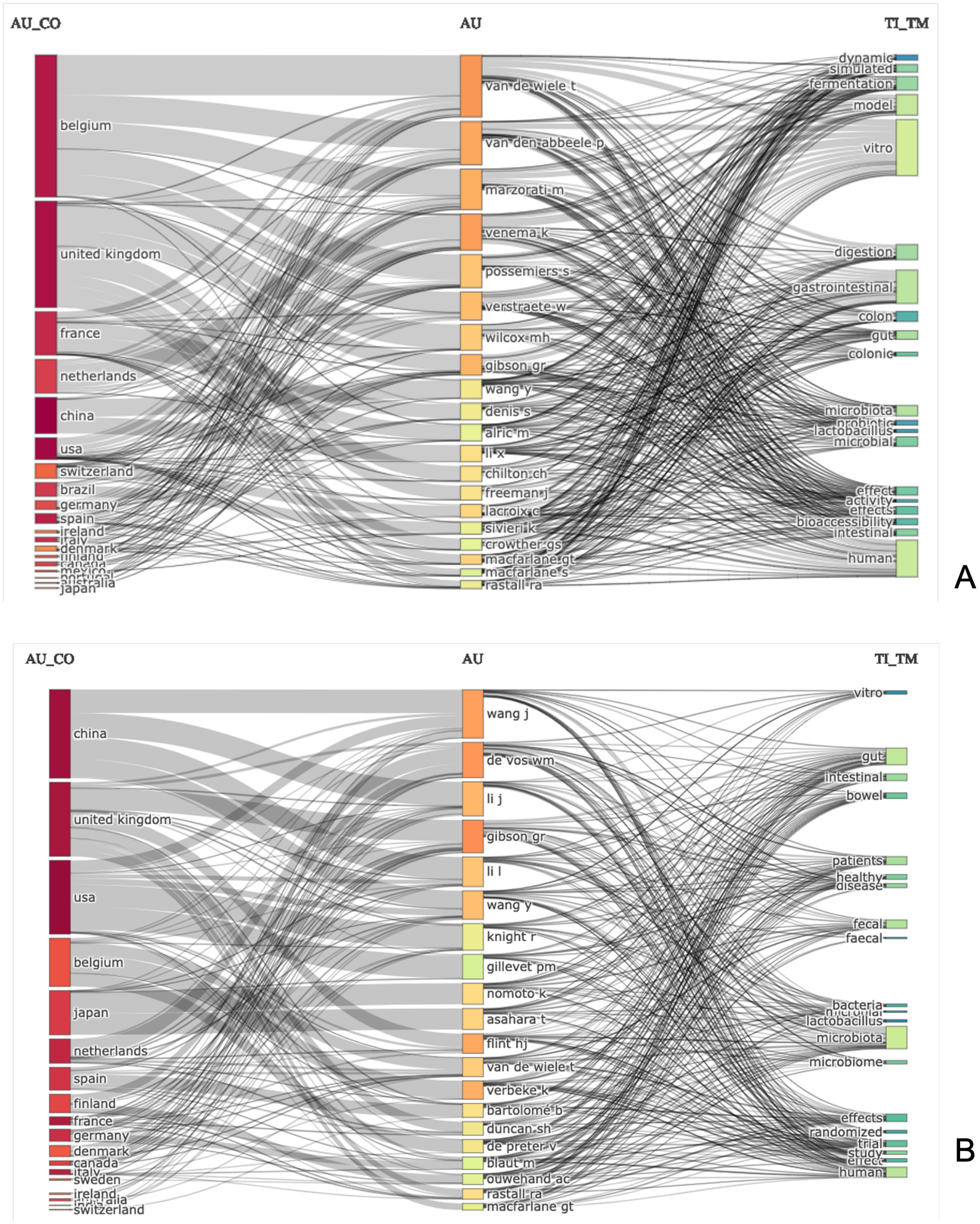

Figure S9: Visualization of the main items of three fields (Country, Authors and word frequency in the title), and their relation through a Sankey diagram. A) Refined dataset, B) Unrefined dataset.
